# Safety, pharmacokinetics and antiviral activity of PGT121, a broadly neutralizing monoclonal antibody against HIV-1: a randomized, placebo-controlled, phase 1 clinical trial

**DOI:** 10.1038/s41591-021-01509-0

**Published:** 2021-10-07

**Authors:** Kathryn E. Stephenson, Boris Julg, C. Sabrina Tan, Rebecca Zash, Stephen R. Walsh, Charlotte-Paige Rolle, Ana N. Monczor, Sofia Lupo, Huub C. Gelderblom, Jessica L. Ansel, Diane G. Kanjilal, Lori F. Maxfield, Joseph Nkolola, Erica N. Borducchi, Peter Abbink, Jinyan Liu, Lauren Peter, Abishek Chandrashekar, Ramya Nityanandam, Zijin Lin, Alessandra Setaro, Joseph Sapiente, Zhilin Chen, Lisa Sunner, Tyler Cassidy, Chelsey Bennett, Alicia Sato, Bryan Mayer, Alan S. Perelson, Allan deCamp, Frances H. Priddy, Kshitij Wagh, Elena E. Giorgi, Nicole L. Yates, Roberto C. Arduino, Edwin DeJesus, Georgia D. Tomaras, Michael S. Seaman, Bette Korber, Dan H. Barouch

**Affiliations:** 1 Center for Virology and Vaccine Research, Beth Israel Deaconess Medical Center, Boston, MA USA; 2 Division of Infectious Diseases, Beth Israel Deaconess Medical Center, Boston, MA USA; 3 Ragon Institute of MGH, MIT and Harvard, Cambridge, MA USA; 4 Infectious Disease Division, Massachusetts General Hospital, Boston, MA USA; 5 Orlando Immunology Center, Orlando, FL USA; 6 McGovern Medical School at The University of Texas Health Science Center, Houston, TX USA; 7 International AIDS Vaccine Initiative, New York, NY USA; 8 Theoretical Biology and Biophysics Group, Los Alamos National Laboratory, Los Alamos, NM USA; 9 Statistical Center for HIV/AIDS Research and Prevention, Fred Hutchinson Cancer Research Center, Seattle, WA USA; 10 Duke Human Vaccine Institute, Duke University, Durham, NC USA; 11 Departments of Surgery, Immunology and Molecular Genetics and Microbiology, Duke University, Durham, NC USA; 12 New Mexico Consortium, Los Alamos, NM USA

**Keywords:** Retrovirus, Immunotherapy

## Abstract

Human immunodeficiency virus (HIV)-1-specific broadly neutralizing monoclonal antibodies are currently under development to treat and prevent HIV-1 infection. We performed a single-center, randomized, double-blind, dose-escalation, placebo-controlled trial of a single administration of the HIV-1 V3-glycan-specific antibody PGT121 at 3, 10 and 30 mg kg^–1^ in HIV-uninfected adults and HIV-infected adults on antiretroviral therapy (ART), as well as a multicenter, open-label trial of one infusion of PGT121 at 30 mg kg^–1^ in viremic HIV-infected adults not on ART (no. NCT02960581). The primary endpoints were safety and tolerability, pharmacokinetics (PK) and antiviral activity in viremic HIV-infected adults not on ART. The secondary endpoints were changes in anti-PGT121 antibody titers and CD4^+^ T-cell count, and development of HIV-1 sequence variations associated with PGT121 resistance. Among 48 participants enrolled, no treatment-related serious adverse events, potential immune-mediated diseases or Grade 3 or higher adverse events were reported. The most common reactions among PGT121 recipients were intravenous/injection site tenderness, pain and headache. Absolute and relative CD4^+^ T-cell counts did not change following PGT121 infusion in HIV-infected participants. Neutralizing anti-drug antibodies were not elicited. PGT121 reduced plasma HIV RNA levels by a median of 1.77 log in viremic participants, with a viral load nadir at a median of 8.5 days. Two individuals with low baseline viral loads experienced ART-free viral suppression for ≥168 days following antibody infusion, and rebound viruses in these individuals demonstrated full or partial PGT121 sensitivity. The trial met the prespecified endpoints. These data suggest that further investigation of the potential of antibody-based therapeutic strategies for long-term suppression of HIV is warranted, including in individuals off ART and with low viral load.

## Abstract (web summary)

A single dose of a broadly neutralizing, HIV-specific antibody transiently reduces viral load in plasma, and in some individuals is associated with durable virus suppression in the absence of antiretroviral therapy.

## Main

Human immunodeficiency virus-1-specific broadly neutralizing monoclonal antibodies (bNAbs) that target the HIV-1 envelope (Env) protein are currently under development as potential tools to treat and prevent HIV-1 infection. Several bNAbs have been tested to date in HIV-1-infected individuals, including the CD4-binding, site-specific antibodies VRC01, 3BNC117, VRC07-523LS and N6-LS, the V3-glycan-specific antibody 10–1074 and the V2-apex-specific antibodies PGDM1400 and CAP256-VRC26.25 (refs. ^1–8^). These bNAbs (alone or in combination) have been shown to decrease plasma HIV RNA levels by 1–2 log in viremic, HIV-1-infected participants not on ART^1–4^. In these studies, bNAb resistance emerged in nearly all treated participants who received monotherapy, but in two notable cases^4^ the repeated administration of a combination of two bNAbs in the setting of viremia maintained viral suppression for 3 months without development of resistance to either antibody. bNAbs have also been shown to delay viral rebound in HIV-1-infected participants on ART who were undergoing analytical treatment interruption^6–8^. However, it has not yet been demonstrated whether a single bNAb given once can maintain viral suppression longer than 3 months without the development of resistance.

PGT121 is a monoclonal antibody isolated in 2011 from an African donor infected with HIV-1 subtype A, whose sera had demonstrated superior neutralization breadth and potency in an observational cohort, and so was considered an ‘elite neutralizer’^9^. PGT121 targets a V3-glycan-dependent site on HIV-1 Env^9^. Structural studies suggest that PGT121 inhibits gp120 binding to the CD4 receptor by interfering with Env receptor engagement and blocking viral entry^10^, and in vitro experiments demonstrated that PGT121 (ref. ^9^) was able to neutralize 64% of 634 diverse tier 2 (for example, more difficult to neutralize) HIV-1 strains tested and recorded in the Los Alamos HIV database tool CATNAP^11^ (data available in Supplementary material), with a geometric mean half-maximal inhibitory concentration (IC_50_) of 0.07 µg ml^–1^ among sensitive isolates. PGT121 decreased viral load (VL) in simian–human immunodeficiency virus (SHIV)-infected rhesus macaques^12^, delayed SHIV rebound following ART interruption when combined with a Toll-like receptor 7 agonist^13^ and protected uninfected monkeys against SHIV challenge at a plasma concentration of <5 µg ml^–1^ (refs. ^14,15^).

Here we report the results of a multicenter phase 1 clinical trial testing the safety, PK, antiviral activity and evolutionary mechanisms of escape of PGT121 in viremic HIV-infected adults not on ART (*n* = 13), as well as safety and PK in HIV-uninfected adults (*n* = 20) and HIV-infected adults on ART (*n* = 15). In the first part of the study we evaluated three doses (3, 10 and 30 mg kg^–1^) and two routes (intravenous (IV) and subcutaneous (SC)) of PGT121 given once in HIV-uninfected adults and HIV-infected adults on ART; participants were randomized 4:1 to receive PGT121 or placebo. In the second part of the study, viremic HIV-infected adults not on ART received a single IV administration of PGT121 at 30 mg kg^–1^.

## Results

### Study population

**Fig. 1:**
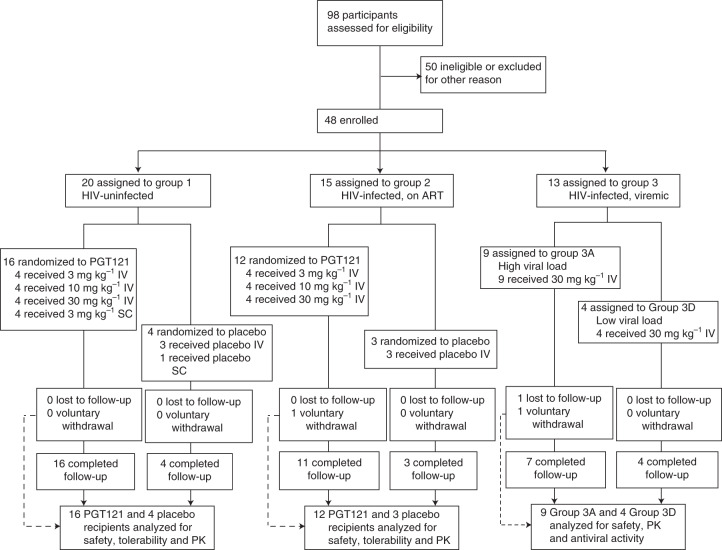
Trial profile. Participant recruitment, randomization, and follow up are depicted. Participants were enrolled concurrently at BIDMC (Beth Israel Deaconess Medical Center), OIC (Orlando Immunology Center), and HART (Houston AIDS Research Team, McGovern Medical School at The University of Texas Health Science Center).

Ninety-eight participants were screened, of whom 50 were found to be ineligible or were excluded for other reasons (Fig. 1). The first participant was enrolled on 17 January 2017, the last on 22 January 2019 and the final study visit was completed on 8 July 2019. The most common reason for screen failure was unwillingness to comply with the protocol requirements (Supplementary Table 1). In Part 1 of the study, HIV-uninfected adults (*n* = 20) were enrolled in Group 1 and HIV-infected adults on ART (*n* = 15) in Group 2. After determination that PGT121 administered at 30 mg kg^–1^ was safe and well tolerated in Groups 1 and 2, Part 2 of the study was initiated with HIV-infected participants not on ART and viremic (*n* = 13) being enrolled in Group 3. As prespecified in the protocol, participants in Group 3 were assigned to one of two groups based on their screening plasma HIV RNA levels: nine participants were assigned to the high-viral-load group (2,000–100,000 copies ml^–1^) and four to the low-viral-load group (100–2,000 copies ml^–1^). Demographics of the study population are shown in Table 1 and Supplementary Table 2.

**Table 1:** Study population

Parameter	HIV-uninfected			HIV-infected on ART		HIV-infected viremic
	Active	Placebo		Active	Placebo	Active
	*n* = 16	*n* = 4		*n* = 12	*n* = 3	*n* = 13
Age in years, mean (range)	28 (19–48)	25 (19–28)		44 (27–62)	44 (29–56)	31 (20–51)
Male, *n* (%)	6 (38)	3 (75)		10 (83)	3 (100)	12 (92)
Years since HIV diagnosis, mean (range)				14 (1–27)	15 (6–27)	3 (0–20)
Absolute CD4^+^ T-cell count, median (IQR)				706 (530–838)	738 (708–757)	568 (494–920)
HIV-1 RNA (copies ml^–1^) on day 0, median (IQR)			High VL, *n* = 9			21,040 (9,660–28,990)
			Low VL, *n* = 4			270 (185–550)

### Safety and tolerability

No related serious adverse events (SAEs), potential immune-mediated diseases or Grade 3 or higher solicited or unsolicited adverse events (AEs) were reported during the study (Supplementary Tables 3–5). One Group 3 PGT121 recipient reported a pregnancy 17 weeks after study product infusion (day 119); pregnancy was confirmed. The participant initiated ART immediately and delivered a healthy baby, who will be followed for safety until the end of the first year of life. Local solicited symptoms were reported by 12/41 (29%) PGT121 recipients and 4/7 (57%) placebo recipients. Systemic solicited symptoms were reported by 12/41 (29%) PGT121 recipients and 2/7 (29%) placebo recipients. The most common reactions among active PGT121 participants were IV/injection site tenderness (*n* = 11/41 (27%)), pain (*n* = 7/41 (17%)) and headache (*n* = 8/41 (20%)). Absolute and relative CD4^+^ T-cell counts did not change following PGT121 administration in HIV-infected participants in Groups 2 or 3 (Supplementary Table 6).

### Pharmacokinetics

**Fig. 2:**
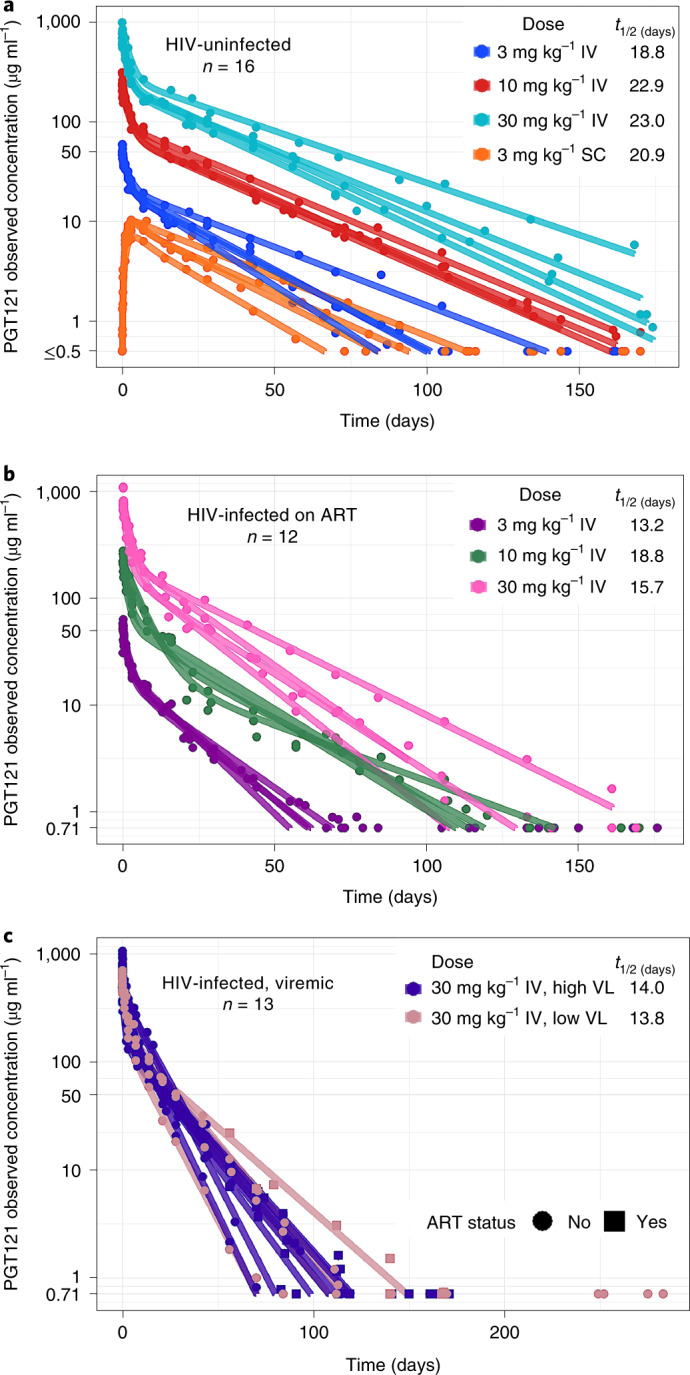
PGT121 PK. **a**–**c**, The PK profile of serum PGT121 is shown for HIV-uninfected participants (**a**), HIV-infected participants on antiretroviral therapy (**b**) and HIV-infected viremic participants (**c**).

**Extended Data Fig. 1:**
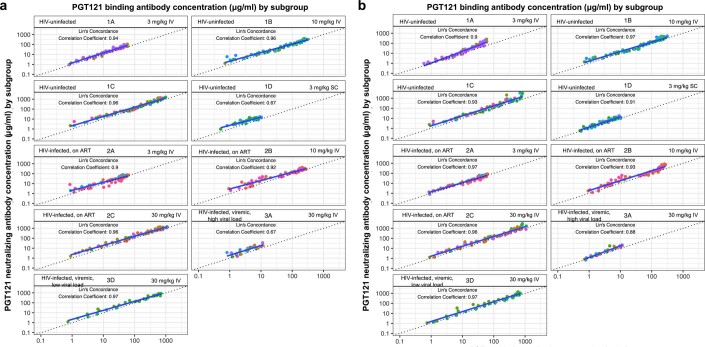
Correlation between PGT121 neutralizing and binding antibody concentrations. Concordance between PGT121 concentrations measured by binding and neutralizing antibody assays using pseudovirus strains CNE30 (**a**) and X2088_c9 (**b**). The dotted line is the identity line. Solid line is the trend line. Data are colored by participant. Lin’s concordance correlation coefficient is a measurement of accuracy and precision which is used to assess agreement between two sets of measurements.

The median (minimum, maximum) PGT121 elimination half-life was 22 (16, 29) days for HIV-uninfected participants, 16 (11, 30) days for HIV-infected participants on ART and 14 (8, 19) days for HIV-infected participants off ART and viremic (Fig. 2a–c and Supplementary Tables 7–9). HIV-uninfected participants had greater PGT121 exposure over time compared to HIV-infected participants who received the same doses (Supplementary Table 9). The more rapid clearance rate among HIV-infected participants was probably due to the so-called sink effect, in which PGT121–virus complexes are removed and taken out of circulation. Interestingly, PGT121 concentrations measured by binding (direct readout) and neutralizing (indirect functional readout) antibody assays using the viruses CNE30 and X2088_c9 displayed substantial concordance (Extended Data Fig. 1a,b).

### Anti-drug antibodies

**Extended Data Fig. 2:**
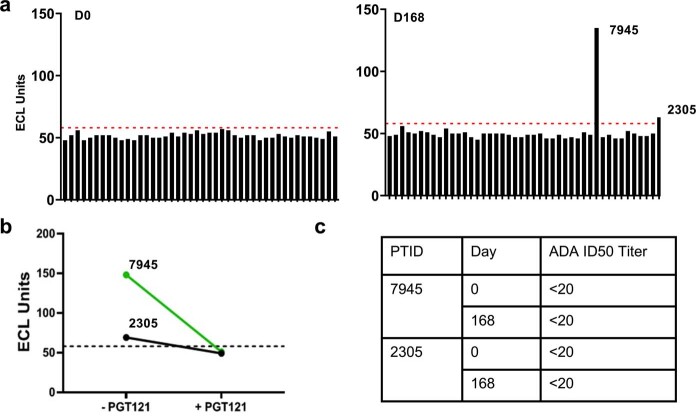
Anti-drug antibody responses. (**a**) Binding anti-drug antibodies at day 0 and day 168 by participant (each black bar represents a separate participant). The red dotted line indicates the run-specific floating cut-point. (**b**) Binding anti-drug antibodies in the setting of PGT121 or no PGT121 competition in participants 7945 and 2305. (**c**) Anti-PGT121 neutralizing activity (ID50 titers) at days 0 and 168 for participants 7945 and 2305.

At baseline, samples from all participants were below the threshold for positivity and were therefore determined to be PGT121 anti-drug antibody (ADA) negative (Extended Data Fig. 2a). At the last available time point for each participant, all samples remained ADA negative with the exception of samples from two participants (PTID 7945 from Group 2, at 30 mg kg^–1^ dose, and 2305 from Group 3) that gave positive signals on day 168 and were confirmed by PGT121 competition assay (Extended Data Fig. 2a,b). For both individuals, positive ADA serum samples were analyzed (using their day 0 baseline samples as reference) in a functional ADA assay in which the participant’s serum was incubated with titrated amounts of PGT121 in TZM.bl cells infected with a PGT121-sensitive HIV-1 pseudovirus to assess anti-PGT121 neutralizing activity (Extended Data Fig. 2c). ADA ID_50_ neutralization titers against PGT121 were below the assay limit of detection (<20) for both samples, indicating that detected ADA were non-neutralizing.

### Antiviral activity

**Extended Data Fig. 3:**
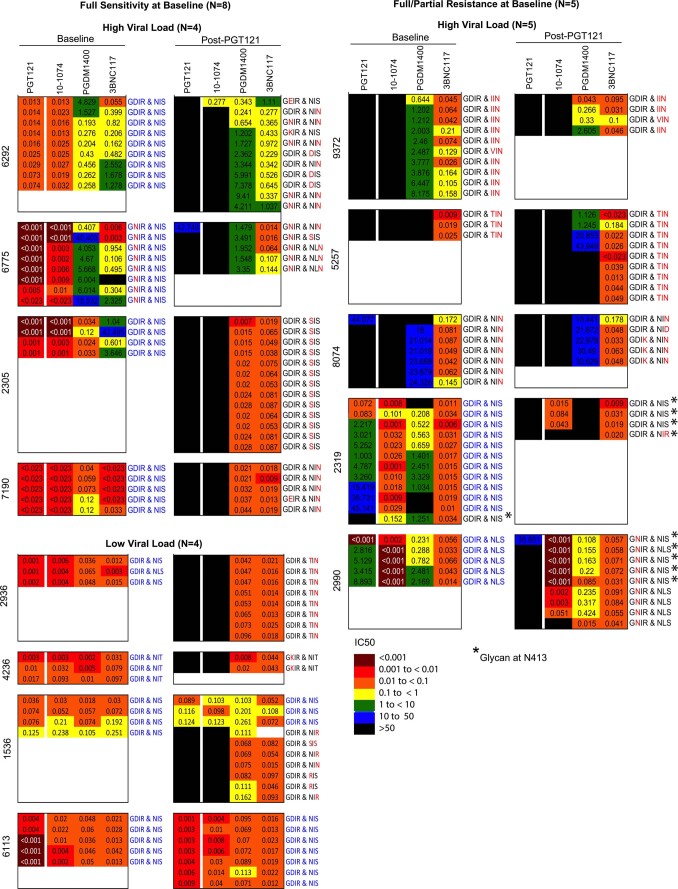
Viral sensitivity to PGT121 and other bNAbs before and after PGT121 infusion. IC_50_ neutralization scores (in µg/ml) are shown for each participant: PGT121 is shown on the left, followed by 10–1074 that also targets the V3 glycan, then a V2 apex bNAb (PGDM1400), then a CD4bs bNAb (3BNC117). Baseline and post PGT121 treatment values are shown, followed by the amino acid sequence of the GDIR motif (HXB2 positions 324–327) and the N-linked glycosylation motif (positions 332–334). Blue amino acids indicate sensitivity signatures for PGT121, while black and red amino acids indicate resistance signatures.

Thirteen HIV-infected participants not on ART were enrolled, all with a HIV-1 viral load at screening of between 100 and <2,000 copies ml^–1^ (low-viral-load group, *n* = 4) or between 2,000 and 100,000 copies ml^–1^ (high-viral-load group, *n* = 9; Table 1 and Supplementary Table 10). HIV-1 pseudoviruses constructed from plasma single-genome amplification of circulating viruses were tested for bNAb sensitivity by TZM-bl assay at baseline, with 8 of 13 participants (61.5%) demonstrating full sensitivity to PGT121 at baseline (Extended Data Fig. 3), consistent with in vitro predictions of 59% sensitivity against 159 clade B viruses (CATNAP data; Supplementary material). Of note, 6 of 13 participants demonstrated either complete or partial levels of resistance (IC_50_ > 1 µg ml^–1^) to at least two bNAbs of different epitope specificity at baseline, demonstrating the prevalence of multi-bNAb resistance during chronic infection (Extended Data Fig. 3).

**Fig. 3:**
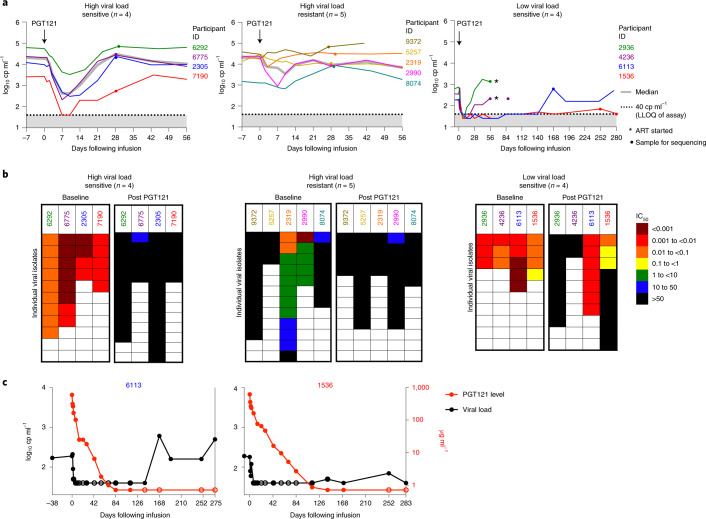
HIV-1 RNA viral load and sensitivity to PGT121 before and after PGT121 infusion. **a**, Plasma HIV-1 RNA levels are shown following PGT121 infusion in HIV-infected viremic participants not on ART. The arrow indicates the day of PGT121 infusion, the gray line equals median value and the dotted line indicates the lower limit of quantification for HIV-1 RNA levels (40 copies (cp) ml^–1^). Asterisks indicate the time point at which ART was started, and dots indicate when samples were collected for sequencing. **b**, IC_50_ neutralization scores (µg ml^–1^), indicating sensitivity or resistance to PGT121 among isolated viruses, are shown for each participant, before and after PGT121 administration. **c**, HIV-1 viral load and PGT121 serum levels are shown for participants 6113 and 1536 following PGT121 infusion. Open circles indicate that either HIV-1 RNA (black) or PGT121 level (red) was below the limit of detection.

**Extended Data Fig. 4:**
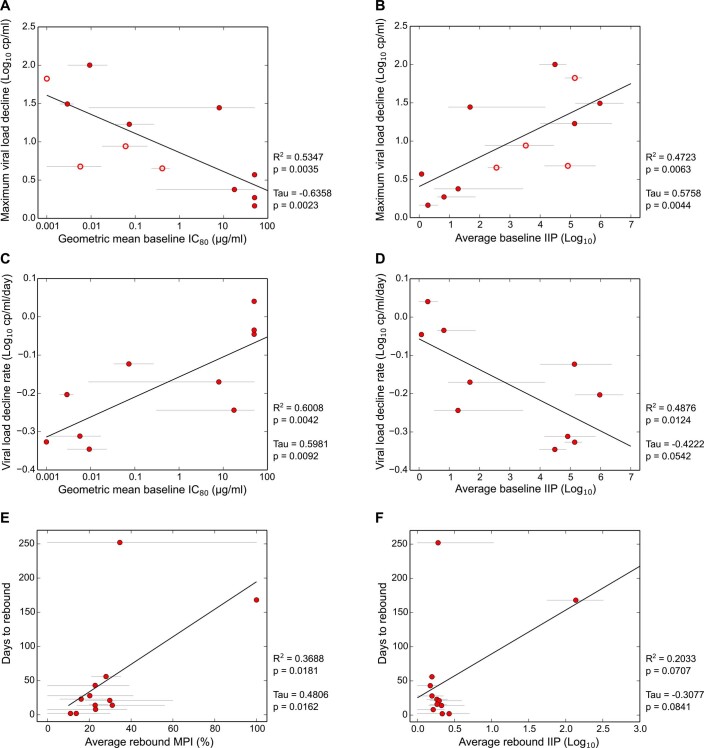
Correlation between PGT121 neutralization and viral load kinetics. (**a**) Correlation between maximum viral load drop and PGT121 geometric mean IC80 titers for baseline viruses. Open symbols indicate participants whose minimum viral load was below the lower limit of quantitation (40 copies/ml). Grey lines indicate range of individual baseline virus IC_80_ titers. Black line is the linear regression trend line. (**b**) Same as (A), but using predicted average instantaneous inhibitory potential (IIP) for baseline viruses (see Methods). (**c**) Correlation between initial viral load decline rate and geometric mean PGT121 IC_80_ titers for baseline viruses. Grey lines indicate range of IC_80_ titers, black line the trend. See methods for initial viral load decline rate calculations. (**d**) Same as (C) but using average IIP as in (B). (**e**) Correlation between days to rebound and average maximum percent inhibition (MPI) of rebound viruses by PGT121. Because most rebound viruses had IC80 above threshold (50 µg/ml), we used MPI defined as the observed percent neutralization of each virus at 50 µg/ml. Time to rebound was calculated as the first timepoint when the viral load post infusion returns to 0.5 Log10 copies/ml below the baseline or higher. (**f**) Same as (E), but using average IIP for rebound viruses. In each panel, statistics using Pearson moment correlation and Kendall Tau rank test are shown. One sided p-values are reported.

In the high-viral-load group, the median viral load at day 0 was 21,040 (interquartile range (IQR) = 9,660–28,990). Following a single IV infusion of PGT121 at 30 mg kg^–1^ (Fig. 3a), four participants with PGT121 sensitivity and high viral load at baseline showed a decrease in viral load of 1.28–2.14 log (median 1.77 log), with a viral load nadir at 7–10 days (median 8.5 days), before viral load increased back to baseline by day 28. Rebound viruses isolated at this time point demonstrated PGT121 resistance (Fig. 3b). The three participants with complete baseline PGT121 resistance had no change in viral load compared to baseline following PGT121 infusion, while participants with a mix of sensitive and relatively resistant viral variants had brief dips in viral load (Fig. 3a and Supplementary Table 10). Notably, baseline virus sensitivity to PGT121 significantly correlated with the maximum drop in viral load observed following infusion (Kendall’s *τ* = −0.64, *P* = 0.0023), as well as with faster decline in viral load (Kendall’s *τ* = 0.60, *P* = 0.0092) (Extended Data Fig. 4a,c). These correlations remained significant even when factoring in changing levels of PGT121 over time (Extended Data Fig. 4b,d)^16,17^. On the other hand, time to viral rebound could not be fully explained by the degree of PGT121 resistance present in rebounding viruses (Extended Data Fig. 4e,f). This suggests that other, unmeasured, factors, such as the presence of minority resistant variants among baseline circulating or latent viruses, might drive the time to rebound.

In the low-viral-load group, the median viral load at day 0 was 270 copies ml^–1^ (IQ = 185–550) and circulating viruses were all PGT121 sensitive (Fig. 3). Following a single IV infusion of PGT121 at 30 mg kg^–1^ (Fig. 3a), all four participants had a decrease in viral load to <40 copies ml^–1^ (lower limit of quantification (LLoQ) of assay) by day 7. Two participants (PTIDs 2936 and 4236) had viral rebound by day 28, and rebound viruses were PGT121 resistant (Fig. 3b). However, in two participants (PTIDs 6113 and 1536) viral load stayed suppressed at <40 copies ml^–1^ until rebound was detected at ≥168 days (Fig. 3c).

For both participants who exhibited long-term virologic control (PTIDs 6113 and 1536), viral rebound did not occur until PGT121 serum levels were below the limit of detection, suggesting substantial viral control even at very low antibody levels. For participant 6113, viral rebound was observed on day 168 and no PGT121 resistance was detected among rebound viruses (Fig. 3b). For participant 1536, viral rebound was not detected until the participant was brought back for a long-term extension of the study, at day 252 (Fig. 3b). Rebound viruses isolated from participant 1536 at that time point had a mixture of sensitivities: 7/10 were resistant while 3/10 remained sensitive (Fig. 3b). Emtricitabine and lamivudine drug levels of participants 6113 and 1536 were obtained at visit 11 (day 84), and both antiretroviral drug levels were below the limit of quantitation (note, these antiretrovirals were chosen for testing because either emtricitabine or lamivudine was present in nearly all available combination ART regimens at that time).

### Pathways to PGT121 resistance

**Extended Data Fig. 5:**
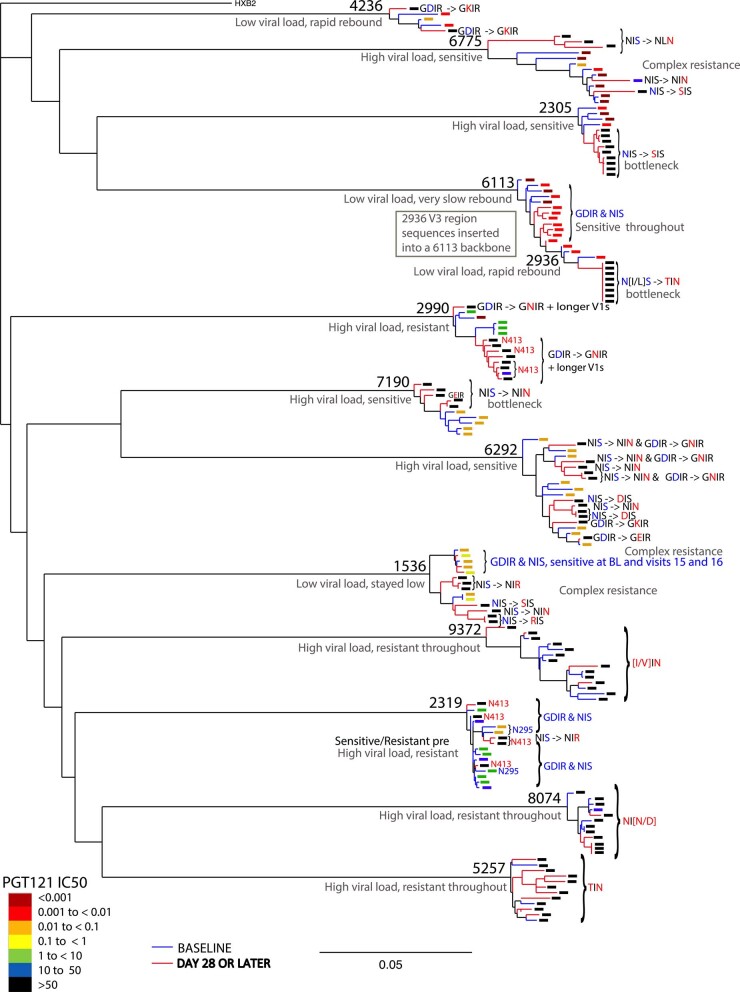
Maximum likelihood phylogenetic tree of HIV-1 Env sequences isolated from participants pre- and post-PGT121 treatment. The branch colors indicate sequences sampled at baseline (blue) versus post-PGT121 treatment (red). The colored box at every leaf indicates PGT121 sensitivity as an IC_50_ score in a TZM.bl assay; black is undetectable, blue and green are relatively resistant, and yellow-through-red increasingly sensitive. The likely amino acids that confer PGT121 resistance mutation are indicated to the right of the branches. If a four-letter motif is indicated, it represents positions 324–327 (HXB2 numbering), a conserved part PGT121 epitope; GDIR is the common consensus form and is associated with antibody sensitivity. If a three-letter motif is indicated, it refers to positions 332–334; most commonly this is a glycosylation site (NIS), and an N-linked glycan is generally critical for V3 bNAb sensitivity. Blue letters indicate PGT121 sensitive signature amino acids, red indicate resistant. The glycosylation motif can be lost by either the loss of the Asn (N) at N332, or the loss of a Thr (T) or Ser (S) at S334. D325 is the most variable position in the highly conserved GDIR motif. D325N does not always confer resistances (for example participant 6775), but is generally associated with resistance (for example participants 6292 and 2990).

**Extended Data Fig. 6:**
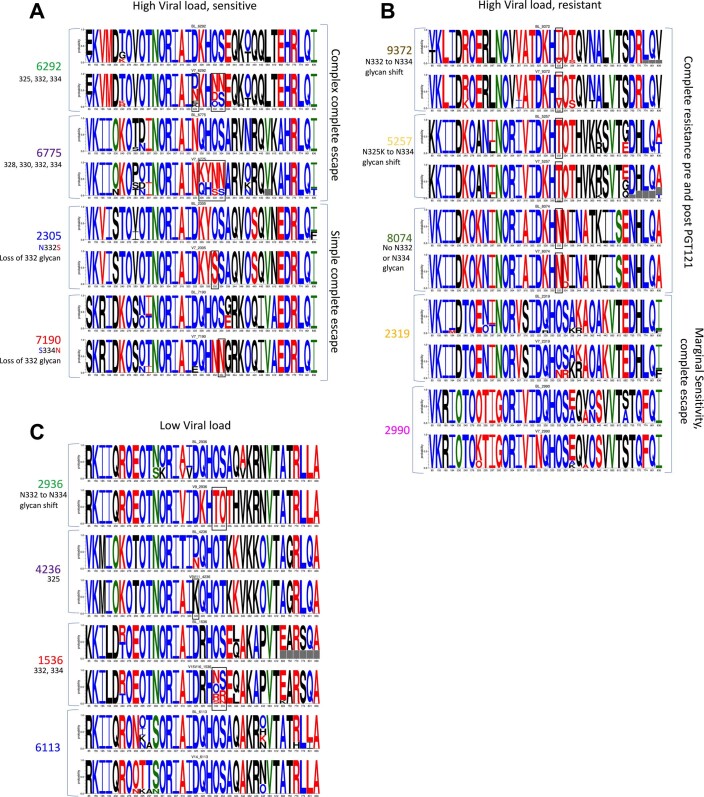
V3 glycan signatures. Glycan signatures are displayed for participants with (**a**) high viral load and sensitive viruses at baseline, (**b**) high viral load and resistant viruses at baseline, and (**c**) low viral load at baseline. LOGO plots are shown representing the sensitivity and resistance mutations for all V3 glycan signatures. The height of the letter indicates the frequences the sample, for the baseline on the top, and the post PGT121 treatment directly underneath. The putative resistance conferring mutations are boxed in black. Red letters indicate resistance, blue sensitivity, green depended on the antibody, and black indicate amino acids not supported statistically as either a sensistivity or resistance signature. The signatures outside of the binding region are more likely to modulate levels of sensitivity, and are seldom perturbed.

**Extended Data Fig. 7:**
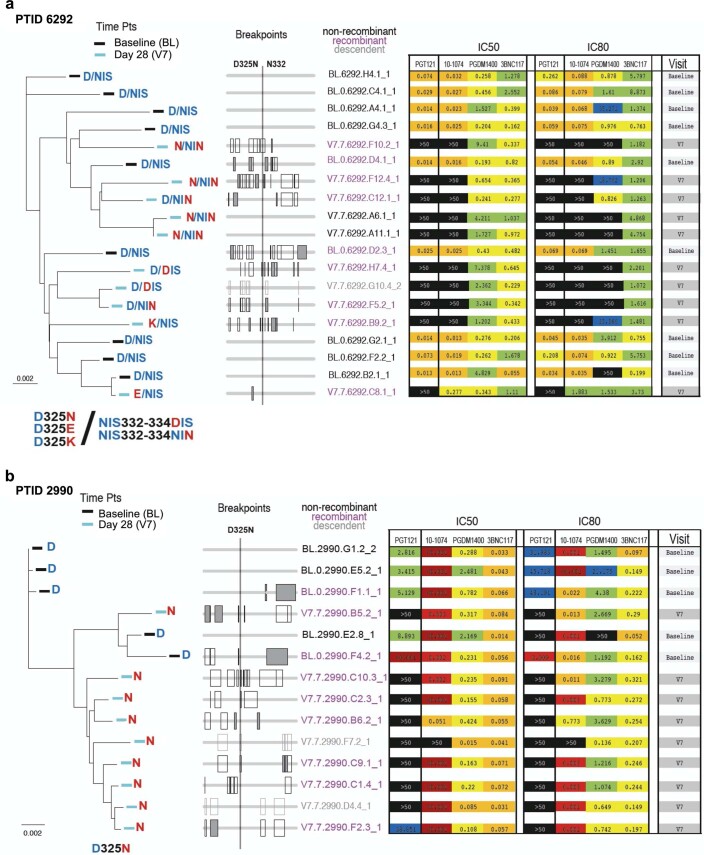
Complex recombination patterns among two participants. The role of recombination in carrying forward resistant form is shown for study participants 6292 (top) and 2990 (bottom). Left panels show the phylogenetic trees with leaves labeled according to the sensitive/resistant form they are carrying; in the middle panels the corresponding sequences (in the same order as the trees on the left) are each represented by a line, and recombinants display regions where recombination breakpoints likely occurred as rectangles (solid rectangles indicate statistically significant recombination breakpoints); corresponding neutralization data (IC50 and IC80) is shown on the right. In both study participants recombination has no impact at baseline as all forms are PGT121 sensitive. However, complex forms of resistance emerge at visit 7. In 6292, these involve D325 alone, N332 alone, and a combination of both. At visit 7, in 2 out of 2 recombination events between a sensitive and a resistant parental form, the resistant form is found in the recombinant, yet all visit 7 viruses are resistant. Either these mutations are occurring de novo on recombinant backbones, or they are resulting from recombination of parental forms that carry the mutation but have not been sampled. In 2990, recombination carries forward PGT121 resistant forms in 3 out of 3 events where the parental strains carry a sensitive and resistant form each. Of note, D325N does not confer 10–1074 resistance in 2990, neither does D325E in 6292. However, D325K, found in 6292, gives rise to resistance to both 10–1074 and PGT121.

**Extended Data Fig. 8:**
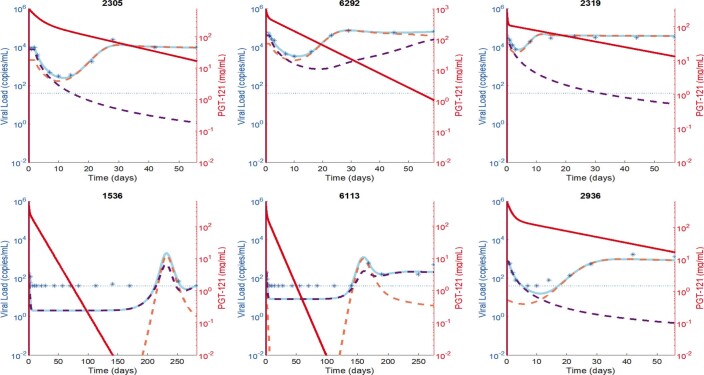
Modeling the effects of PGT121 on plasma viral load. Best fit of viral dynamic model (light blue line) to measured viral load data (blue asterisks) for 6 participants. PGT121 serum concentration is shown in solid red. Horizontal dotted line indicates the viral load limit of detection. Participants 2990,6292 and 2305 shown in the top row belong to the high viral load group, with the remaining patients belonging to the low viral load group. The model follows two populations of virus, PGT121 sensitive and PGT121 resistant, denoted by the purple and orange dashed lines, respectively, while the total viral load is a solid light blue line. The level of resistance is allowed to vary among participants. For 2305, 2936, 2990, 6292 and 6113 the resistant population is predicted to pre-exist at the indicated levels. For the long-term controller 6113 the pre-existing resistant virus is predicted to be partially sensitive to the antibody and after PGT121 administration its level quickly decreases below the x-axis and does not reappear until after day 100. For the other long-term controller 1536, the resistant population may pre-exist at a baseline level of 10^–3^ copies/ml or lower or may arise by mutation. Loss of control in both 1536 and 6113 is predicted to occur due to a resurgence of resistant virus. For the periods of observation indicated, the resistant population is predicted to persist (2305, 2936 and 2990) or to be replaced by drug sensitive virus as the antibody concentration wanes in 6292 and the long-term controllers 1536 and 6113.

There were several distinct viral evolutionary patterns associated with the emergence of PGT121 resistance following treatment. Three participants (2305, 7190 and 2936) demonstrated selection of a single resistant sequence variant, which emerged in a single lineage from more diverse baseline sequences (Extended Data Figs. 5 and 6). In these participants, loss of glycan N332 was associated with complete resistance to PGT121, consistent with previous studies^18^. For all other participants, PGT121 resistance was associated with more complex patterns of viral escape involving multiple resistance mutations (Extended Data Figs. 3 and 5–8) and multiple phylogenetic lineages (Extended Data Fig. 5). Resistance was mainly conferred by removal of the glycan at 332 (mutations at 332 and/or 334) but, in a few cases, also by mutations at site 325 in the GDIR motif located within Env V3 loop positions 324–327, a region critical for binding to the coreceptor CCR5 (refs. ^19,20^) as well as V3 glycan bNAbs, and a well-established PGT121 resistance site^18^. For example in participants 2990 and 4236, PGT121-resistant viruses had Asn at 325 (D325N) and Lys at 325 (D325K), respectively, while retaining the glycan at N332. Rebound viruses from participants 6292 and 7190 showed resistance mutations at both 332/334 and 325. For participant 2319, a resistance pattern was observed where one amino acid change introduced a glycan at position N413 (Extended Data Figs. 5 and 6). The close structural proximity to glycan N33220 suggests that this modification is a candidate for driving of PGT121 resistance.

Eleven (11) of the 13 viremic participants had evidence of recombination in rebound viruses and, in two of these, recombination was shown to impact the acquisition of resistance to PGT121. Participant 6292 had highly diverse resistance signatures following PGT121 treatment, with multiple escape variants in both the D325 site (D to N, E or K) and the glycosylation sequon at N332 (NIS to either DIS or NIN), and different combinations of resistance mutations at the two sites were found. Most of the complex resistance forms in this participant were recombinants (Extended Data Fig. 7a). Participant 2990 had a single resistance mutation that emerged after PGT121 treatment, D325N. All of the viruses sampled following treatment carried D325N and were identified as recombinants. These viruses were relatively diverse, suggesting that the resistance mutation was moving through the quasispecies via recombination (Extended Data Fig. 7b).

We sequenced viruses from the two patients with long-term, antibiotic-mediated suppression at very late time points after virus rebound, at day 252 and beyond (Fig. 3a and Extended Data Fig. 6c). For participant 1536, some rebound viruses retained sensitivity to PGT121 while others were completely resistant; the phylogenetic tree suggests that the re-emerging sensitive forms were very closely related to a baseline lineage (Extended Data Fig. 5). Viruses from participant 6113 were sensitive both before and after treatment (Fig. 3b).

While for most participants PGT121 resistance was accompanied by 10–1074 resistance, for participants 2990 and 2319, PGT121-resistant viruses remained highly sensitive to 10–1074 (Extended Data Fig. 3). Only one participant acquired resistance to a neutralizing antibody with an unrelated target, either PGDM1400 (a V2 antibody) or 3BNC117 (a CD4bs antibody), following administration of PGT121 (a V3 antibody) (Extended Data Fig. 3). Participant 2319 developed full resistance to PGDM1400, which was probably carried along when the virus selected for PGT121 resistance during treatment.

Building on previously established methods^21,22^, we developed a viral dynamic model for bNAb-mediated control of HIV-1 infection and fit it to viral load data from viremic patients in this study. Our model accurately fits the viral load data and makes predictions about the temporal dynamics of sensitive and resistant populations (Extended Data Fig. 8). For example, in some participants, such as 2305, 2936 and 2990, the model predicted that a resistant population pre-exists, drives viral load rebound back to baseline and persists as the dominant population until the end of the observation period at day 56 (Extended Data Fig. 8). For both long-term controllers, 1536 and 6113, the model predicts that both sensitive and resistant populations are exquisitely controlled by PGT121 initially, and that the loss of control is due to an increase in the resistant population as antibody pressure wanes. However, ultimately the resistant population is replaced by a PGT121-sensitive population, possibly due to the lower replicative fitness of the resistant population (note, replicative fitness of isolated viruses was not tested in this study). The replacement by PGT121-sensitive virus after decay of PGT121 in serum was also seen for patient 6292.

### Immune responses

**Fig. 4:**
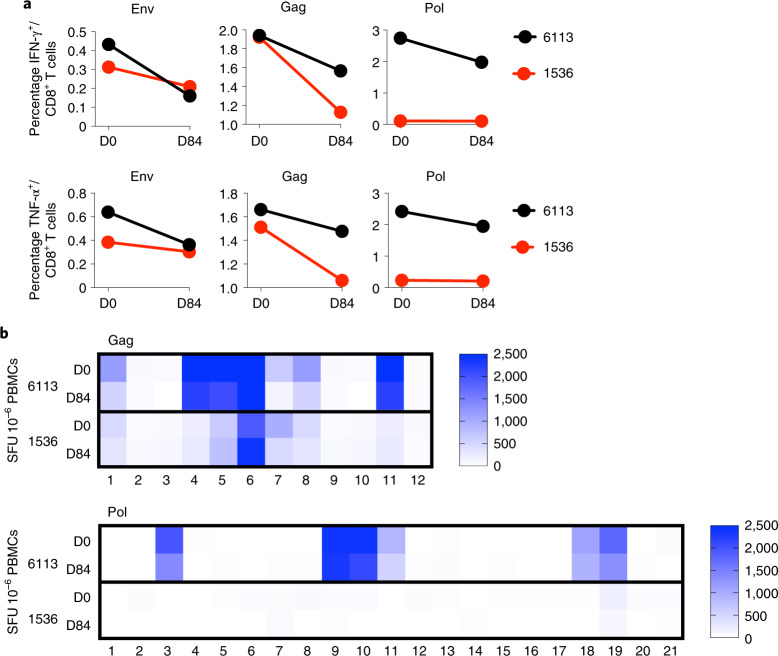
Immunologic responses following PGT121 infusion in two long-term suppressors. **a**, The percentages of Env-, Gag- and Pol-specific CD8^+^ T cells secreting IFN-γ and TNF-α Env are shown for participants 6113 and 1536 at day 0 (D0) and day 84 (D84). **b**, The magnitude of Gag- and Pol-specific IFN-γ-secreting cellular immune responses (spot-forming units (SFU) 10^–6^ PBMCs) is plotted by peptide subpool for participants 6113 and 1536 at D0 and D84.

**Extended Data Fig. 9:**
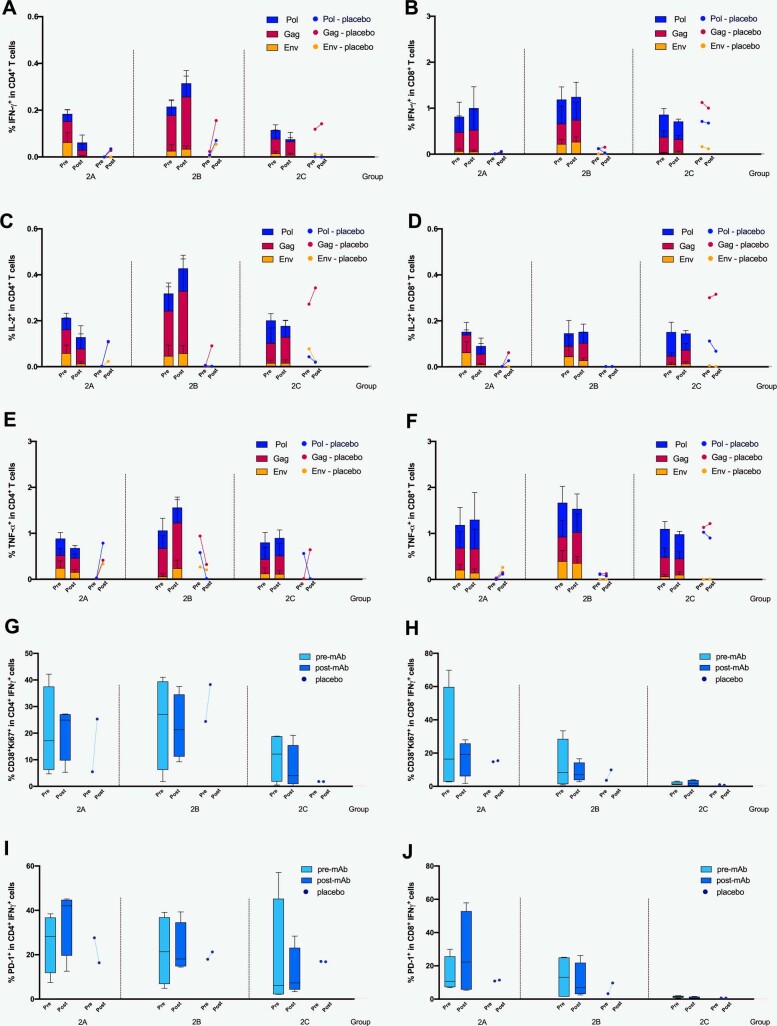
Immunologic responses following PGT121 infusion in HIV-infected participants on ART. The number of IFN-γ secreting CD4 + and CD8 + T cells (A and B, respectively), IL-2 secreting CD4 + and CD8 + T cells (C and D, respectively) and TNF-a secreting CD4 + and CD8 + T cells (E and F, respectively) recognizing peptide subpools across the Gag, Pol and Env protein were measured by flow cytometry in all group 2 A, B and C participants (PGT121 and placebo recipients, N = 15) at baseline (pre-mAb) and at day 28 (post-mAb). For panels A-F, data are presented as median values + /− 95% CI; n = 4 (active) and n = 1 (placebo) for each subgroup. Frequencies of activated CD38 and Ki67 expressing IFN-γ secreting CD4 + and CD8 + T cells (G and H, respectively), and frequencies of PD-1 expressing IFN-γ secreting CD4 + and CD8 + T cells (I and J, respectively) at baseline (pre-mAb) and at day 28 (post-mAb) were assessed by flow cytometry. Boxplots display minimum, maximum, median and interquartile range; n = 4 (active) and n = 1 (placebo) for each subgroup.

Recent data in rhesus macaque and human studies suggest that bNAb administration can enhance the autologous HIV-specific T-cell response^23,24^. To explore whether this potential vaccinal effect may have contributed to the long-term, antibiotic-mediated suppression observed for participants 6113 and 1536, we evaluated HIV-1-specific T-cell magnitude, breadth and functionality at baseline and at day 84. We found no increase in the total number of Env-, Gag- or Pol-specific interferon-gamma (IFN-γ)- or tumor necrosis factor-alpha (TNF-α-secreting T cells following infusion (Fig. 4a). Furthermore, no expansion of T-cell breadth (Fig. 4b)—as determined by the number of IFN-γ-secreting T cells recognizing peptide subpools across Gag and Pol proteins—was observed. We also investigated whether PGT121 had any immunomodulatory effect in the group of HIV-infected participants on ART with stably suppressed viral load (Group 2). However, we were unable to detect any major changes in Gag-, Pol- and Env-specific IFN-γ-, IL-2- or TNF-α-secreting CD4 or CD8 T cells, or frequencies of activated CD38^+^ Ki67^+^ or exhausted PD-1-expressing CD4 and CD8 T cells specifically when compared to placebo control individuals (Extended Data Fig. 9). These data therefore support the conclusion that the viral control observed was mediated by bNAb alone and was not likely to have been supported by improved T-cell immunity following treatment.

## Discussion

In this study, we demonstrate that the bNAb PGT121 potently and transiently inhibited HIV-1 RNA replication in viremic, HIV-infected individuals who had PGT121-sensitive viruses at baseline. Following a single IV infusion of PGT121 at 30 mg kg^–1^, participants with PGT121 sensitivity and high viral load at baseline had a brisk response, with a median 1.77 log decrease in viral load within a median 8.5 days. Notably, two out of four individuals with a low viral load at baseline demonstrated drug-free viral suppression for ≥168 days following a single infusion of PGT121. For these two participants, viral rebound occurred only when PGT121 was no longer detectable in serum, and their rebound viruses still demonstrated full or partial PGT121 sensitivity. ART-free viral load suppression ≥168 days has not previously been reported in the literature following one dose of an individual or combined bNAb treatment for HIV.

Consistent with previous studies, PGT121 monotherapy in 11 out of 13 HIV-infected viremic participants led to the emergence of PGT121 resistance and rebound back to baseline viral loads by day 28. Among the 12 individuals that displayed at least partial resistance to PGT121 following treatment, resistance could be readily explained in 11 by loss of the glycan at N332 (in seven participants), the loss of the D325 (two participants) or the loss of both N332 and D325 (two participants). In two participants (6292 and 2990), the loss of the D325 form was probably propagated due to recombination^25^ (Extended Data Fig. 5). One participant (6113) had viruses that were sensitive to PGT121 both before and after treatment; this individual maintained drug-free viral suppression through their day 168 visit, and rebound was detected at their next visit on day 252. For this individual it is possible that resistance was never elicited, though it is also possible that resistant viruses might have emerged after day 168 but had already been replaced by a more fit form by the time sampling was performed at day 252.

The simultaneous emergence of many distinct forms of resistance suggests that these variants may have been present at baseline, either at a low frequency in circulating strains or in the latent reservoir, and selection imposed by the PGT121 antibody rapidly revealed the pre-existing complexity among posttreatment samples.^26^. A hypothesis consistent with these data is that participants may have had a previous antibody response that targeted the V3 epitope region, the virus escaped, the antibody response waned and a more fit PGT121-sensitive form came to dominate the circulating virus quasispecies. These data, together with our observation that PGT121 resistance caused resistance to other bNAbs in only one out of 12 participants, underscore the importance of combining bNAbs that target distinct and nonoverlapping epitopes in future studies for HIV treatment or prevention.

While both PGT121 and 10–1074 bind to carbohydrate-dependent epitopes on V3, previous data suggested that PGT121 might have a higher threshold to resistance than 10–1074 due to more promiscuous binding to heterogeneous N-glycan compositions on HIV-1 envelope spikes^14,27,28^. We did not observe such a higher threshold for PGT121 in our study. Three participants (2305, 7190 and 2936) developed complete resistance to both PGT121 and 10–1074 associated with loss of glycan N332, and two participants (2990 and 2319) developed complete resistance to PGT121 but remained highly sensitive to 10–1074 (Extended Data Figs. 5 and 6). These data suggest that there may be additional differences in the binding properties of these two antibodies that could account for varying resistance patterns in rebound viruses.

Of note, our analysis of pathways to resistance following PGT121 administration is limited by the fact that we were able to measure only those viruses that were found in the circulation at the specific time point of the blood-draw, and this sample probably does not represent the entirety of viral quasispecies present in the participant. Nevertheless, these are the viruses that dominate in peripheral blood pre- and post-bNAb treatment, driving the infection.

Previous studies have explored whether bNAb treatment for HIV may induce a so-called vaccinal effect, whereby the administration of the bNAb enhances host immune responses and leads to better viral control^23,24^. To investigate this hypothesis, we investigated whether our two participants with long-term suppression had any change in their cellular immune responses. We found that there was no increase in magnitude, phenotype or activation and exhaustion status in either participant following bNAb administration. Our conclusion is that the long-term virologic suppression in these two participants is probably due to the exquisite potency of PGT121, even at levels below the limit of quantitation, coupled with the fact that both participants had low viral loads at baseline. PGT121 may have a higher barrier to resistance than other single bNAbs tested thus far, accounting for this long-term suppression, and/or participants with low viral load at baseline may have a limited number of pre-existing viral quasispecies at the time of PGT121 administration. It is also possible that other, unmeasured, immune responses, such as natural killer cell or complex humoral immune responses, may have exerted some immune-mediated control on viral replication that we were not able to detect.

Finally, our data demonstrate that PGT121 monoclonal antibody was safe and well tolerated in both HIV-uninfected and HIV-infected individuals up to 30 mg kg^–1^ IV, and at 3 mg kg^–1^ SC in HIV-uninfected adults. The half-life of PGT121 was 22 and 14 days in HIV-uninfected and -infected participants, respectively. PGT121 did not appear to generate any functional anti-drug antibodies after a single dose, because no neutralizing anti-PGT121 antibodies were detected at the end of follow-up in any participant.

In summary, PGT121 was safe, well tolerated and had a brisk antiviral effect in viremic, HIV-1-infected participants whose viruses were sensitive to PGT121 at baseline. In two participants with low viral loads at baseline, a single administration of PGT121 was associated with long-term, Ab-mediated viral suppression for up to, and greater than, 6 months. Although the generalizability of our data is limited by our small sample size, they suggest that PGT121 should be tested further for its ability to maintain viral suppression in the setting of ART interruption, or to block HIV acquisition, particularly when used in combination with other bNAbs.

## Methods

### Study design

The study evaluated the safety, PK and antiviral activity of PGT121 monoclonal antibody (mAb). Part 1 of the study was a single-center, randomized, double-blind, dose-escalation, placebo-controlled trial of PGT121 in HIV-uninfected adults and HIV-infected adults on antiretroviral therapy (ART) at Beth Israel Deaconess Medical Center (BIDMC), Boston, MA, USA. Part 2 of the study was a multicenter, open-label trial of PGT121 in viremic HIV-infected adults not on ART at three sites: BIDMC, Boston, MA; Orlando Immunology Center (OIC), Orlando, FL; and Houston AIDS Research Team (HART), McGovern Medical School at The University of Texas Health Science Center at Houston, TX (all USA). The protocol was approved by the BIDMC Institutional Review Board (IRB), the OIC IRB and the HART Committee for the Protection of Human Subjects. The study was registered at ClinicalTrials.gov (no. NCT02960581). In Part 1 we evaluated three doses (3, 10 and 30 mg kg^–1^) and two routes (IV and SC) of PGT121 given once (Fig. 1a). Each participant in Part 1 received either PGT121 or placebo at a ratio of 4:1 within each subgroup. In Part 2, all participants received a single IV administration of PGT121 at 30 mg kg^–1^. There was no placebo or blinding for Part 2.

### Study participants

Participants were eligible for the trial across groups if they had a body mass index >18 and <35 and if they did not have any clinically important acute or chronic medical condition (besides HIV). To enroll, sexually active participants had to be willing to use contraception for three months following investigational product (IP) administration, and could not be pregnant or breastfeeding. Participants were eligible for Group 1 if they were also 18–50 years of age, at low risk for HIV infection and willing to maintain low-risk behavior. HIV-infected participants were eligible for either Group 2 or 3 if they were 18–65 years of age, had ≥300 CD4 cells μl^–1^ and no history of acquired immune deficiency syndrome (AIDS)-defining illness within the previous 5 years. HIV-infected participants were eligible for Group 2 if they were currently on ART with suppression of plasma HIV-1 viral load <50 copies ml^–1^ for >6 months. HIV-infected participants were eligible for Group 3 if they were not receiving ART, willing to defer ART treatment for at least 56 days after administration of IP (after appropriate counseling) and had an HIV-1 viral load of either 2,000–100,000 or 100–2,000 copies ml^–1^. All participants gave written informed consent and successfully completed an assessment of understanding before the initiation of study procedures.

### Randomization and masking

In Part 1, eligible participants were enrolled into the lowest dose subgroup (Group 1A or 2A) depending on their HIV serostatus. Dose subgroups in Groups 1 and 2 were enrolled in parallel. Participants in each subgroup were identified by a unique study identification number and randomized according to the schedule prepared by the Data Coordinating Center (DCC, Emmes Company, LLC) before the start of the study. Participants were automatically assigned a specific allocation number as they were enrolled into the data entry system. An unblinding list (pharmacy list) was provided to the unblinded site pharmacist by the DCC. Study staff and participants were blinded to the allocation of IP. A site pharmacist was unblinded for preparation of the study product. Blinded participants were informed about their assignment (product/placebo) at study completion, once the data were locked. Groups 1 and 2 were unblinded separately after the last participant in the respective groups had completed study participation. Because PGT121 and placebo (saline) appeared identical in the infusion bag, no masking was required. The five participants in each subgroup were randomized at a ratio of four PGT121 recipients to one placebo recipient. At each dose level in Part 1, IP administration was separated by at least 24 h for each of the first three participants. Randomization ensured that at least two participants received active product and were observed for at least 24 h before administration to additional participants.

### Investigational products

PGT121 is a recombinant, fully human mAb of the IgG1 isotype that binds to the HIV envelope. PGT121 was formulated in a 20 mM acetate, 9% sucrose, 0.008% polysorbate 80, pH 5.2 formulation buffer at a concentration of 50 mg ml^–1^. Each 10-ml vial contained 6 ml of PGT121. Placebo was 0.9% sodium chloride. Participants received either PGT121 or placebo via IV infusion or SC injection, the former over approximately 60 min. No local or topical anesthetic was used before SC injections. IP was injected over a period of 1–5 min, preferably in the abdomen. The maximum volume injected SC per site was approximately 2.5 ml. If multiple SC injections were needed, the distance between injection sites was at least 1 inch.

### Safety assessments

Local and systemic reactogenicity safety data were collected for 3 days after IP administration (see Supplementary Information for protocol and schedule of procedures). Data on unsolicited AEs were collected until 56 days following IP administration. Potential immune-mediated diseases (pIMDs) were considered AEs of special interest since these could potentially be caused by immune responses to the IP. Data on pIMDs and SAEs were collected through to study Day 168. Blood samples for serum chemistry and hematology were collected at 12 time points throughout the study, as were urine samples for pregnancy testing and urinalysis. Blood samples for HIV-1 viral load and CD4^+^ T-cell count were collected throughout the study from HIV-infected participants. Medical monitoring was provided by a protocol safety review team and an independent safety monitoring committee. Local and systemic AEs were graded by the NIAID Division of AIDS Table for Grading the Severity of Adult and Pediatric Adverse Events v.2.1, July 2017. For the first 24 h after IP infusion or injection, any infusion-related reactions, including cytokine release syndrome, were graded by the National Cancer Institute Common Terminology Criteria for Adverse Events v.5.0, 27 November 2017. Peripheral blood was collected to determine PGT121 serum levels, anti-drug antibodies, HIV sequencing and immunogenicity, among other research assessments outlined in the study protocol (Supplementary material).

### Antiretroviral therapy counseling

HIV-infected participants who were not on ART received ART counseling upon entering the study and 8 weeks after administration of IP. Participants who had not initiated, or made plans to initiate, ART by the final study visit received ART counseling again at their final study visit. HIV-infected participants who were on ART (Group 2) were counseled on the importance of continuing ART throughout the study, and were not required to interrupt ART after administration of IP.

### HIV-1 viral load and CD4^+^ T-cell measurement

Plasma HIV-1 viral load was measured at BIDMC by real-time PCR assay using the Roche COBAS AmpliPrep/COBAS TaqMan HIV-1 Test, v.2.0 (LLoQ = 23 copies ml^–1^), until it was replaced with a transcription-mediated amplification assay using the Hologic Aptima HIV-1 Quant Assay (LLoQ = 32 copies ml^–1^). HIV-1 viral load was measured at OIC and HART by real-time PCR using the Abbott Real Time HIV-1 assay (LLoQ = 40 copies ml^–1^); assays were performed at LabCorp. CD4^+^ T-cell counts were measured using a clinical flow cytometry assay performed at either LabCorp or BIDMC.

### Determination of PGT121 serum levels

(1) Binding antibody multiplex assay: PGT121 levels were determined on a Bio-Plex instrument (Bio-Rad) using standardized HIV-1 Luminex assays as previously described^29–32^. PGT121 concentrations were representative of three separate assays, where each sample was run in duplicate. The standard curve was PGT121 IgG mAb, titrated in assay diluent. The negative controls were CH58 (an irrelevant mAb) and blank beads. Samples with PGT121 concentrations of LLoQ < 0.5 µg ml^–1^ for HIV-uninfected participants, and 0.71 μg ml^–1^ for HIV-infected participants, at a dilution of 1:50 were designated as 0.5 and 0.71 for plotting purposes for HIV-uninfected and -infected participants, respectively. Samples with PGT121 concentrations above the LLoQ at 1:50 dilution were further tested at various dilution factors to obtain median fluorescent intensity (MFI) in the linear range of the standard curve, and the resulting concentrations from the standard curve were averaged. Samples that were positive at or greater than the limit of detection with an MFI threefold over the preinfusion MFI with a PGT121 concentration lower than LLoQ were designated positive; samples with observed concentrations lower than the LLoQ were designated negative. (2) TZM-bl neutralization assay: the neutralizing actively of passively infused PGT121 was measured as a function of reduction in Tat-regulated luciferase (Luc) reporter gene expression in TZM-bl cells^33–36^. Neutralization titers were measured in pre- and postinfusion immune sera against indicator viruses X2088_c9 and CNE30 (viruses sensitive to neutralization by PGT121) and MuLV (negative control virus). Titers ranged from a minimum of 1:20 to a maximum of 1:1,562,500, with values outside of this range considered censored. Serum concentrations of PGT121 were calculated for each indicator virus by multiplying the serum ID_50_ titer at each time point with the median IC_50_ titer of the PGT121 drug product for each respective virus. The median IC_50_ titer of PGT121 against viruses X2088_c9 and CNE30 was determined by neutralization assays utilizing the clinical PGT121 drug product (Catalent, no. 1-FIN-2442), tested at a primary concentration of 10 μg ml^–1^ with fivefold dilution series. Replicate assays were performed on multiple days by independent operators (*n* = 12 for each virus). All preinfusion serum samples from HIV-infected or -uninfected participants were shown to have no neutralizing activity against PGT121-specific indicator viruses X2088_c9 and CNE30. For Group 2 participants (HIV-infected on ART), serum samples were tested using viruses CNE30, X2088_c9 and MuLV pseudotyped using an SG3Env backbone vector engineered with three site-directed mutations to confer resistance to inhibition by antiretroviral drugs present in the serum samples (triple mutant no. Q148H/K101P/Y181C).

### Measurement of ADA levels

Anti-drug antibodies against PGT121 were measured using a qualified electrochemiluminescence (ECL)-based bridging immunoassay. In brief, test serum samples were first subjected to an acid-dissociation step in glycine-HCl acid buffer on a plate shaker for 1 h. Acid-treated samples were mixed (1:1) with a mastermix containing equimolar amounts (0.5 µg ml^–1^) of PGT121 covalently conjugated to biotin or ruthenium labels and incubation on a plate shaker overnight. After overnight incubation, samples were transferred to a 96-well functionalized streptavidin plate (Meso Scale Discovery) and shaken for 1 h on a plate shaker. The streptavidin plate was washed several times, Tris-based read buffer containing tripropylamine (TPA) was added and the presence of ADA bridging complexes detected on a MESO QuickPlex SQ-120 plate reader (Meso Scale Discovery). The ECL signal generated was proportional to the level of ADA bridging complexes captured in each sample. Any samples that were above the assay floating cutoff point for positivity were considered putative ADA positive and were reanalyzed in a competitive inhibition confirmatory assay using the same assay format in the presence and absence of a molar excess (200 µg ml^–1^) of unlabeled PGT121 in the assay mastermix. Inhibition of >20% in the presence of excess PGT121 was used to confirm ADA positivity. Functional anti-PGT121 ADA activity was measured in TZM.bl cells as previously described^37^. Briefly, patient serum samples were serially diluted in the presence of a partial inhibitory dose of PGT121, and neutralizing activity against the PGT121-sensitive virus X2088_c9 was measured. ADA titers are expressed as the reciprocal sample dilution required to decrease the neutralizing activity of the bNAb by 50%. A murine anti-PGT121 antibody was utilized as a positive control.

### Sequencing of participant HIV-1 *env* genes and production of pseudoviruses

Single-genome amplification (SGA) assays were performed essentially as previously described^38^. Briefly, HIV-1 RNA was isolated and reverse transcribed to viral complementary DNA. First-round PCR was carried out with Q5 High-Fidelity 2X Master Mix (NEB) together with HIV B primers. Amplicons from cDNA dilutions showing <30% positive were considered to have resulted from a single cDNA amplification and were processed for sequencing. For each sample, 15–30 sequences were analyzed. Selected viral sequences isolated from the plasma of each participant by SGA were used to generate pseudoviruses as previously described^34^.

### Assessment of ART drug levels in plasma

Plasma samples from 6113 and 1536 from visit 11 were quantitatively analyzed for emtricitabine and lamivudine at the University of Nebraska Medical Center. These two antiretroviral drugs were chosen for testing because at least one of them is present in commonly used combination antiretroviral regimens.

### Immunogenicity assessments

HIV-specific T-cell responses were measured by IFN-γ enzyme-linked immunospot (ELISPOT) assay using potential T-cell epitopes (PTE) Env, Pol and Gag peptide libraries^39,40^. ELISPOT assay was performed as follows. White membrane plates (Millipore) were coated at 4 ˚C overnight with 10 µg ml^–1^ anti-human IFN-γ (Mabtech). Rested peripheral blood mononuclear cells (PBMCs) were plated at 2 × 10^6^ with PTE Env, Pol or Gag pools at 2 µg ml^–1^ for 18 h at 37 ˚C. Development was achieved by the addition of biotin (Mabtech), antibiotin (VectorLabs) and chromagen (Pierce). Background subtraction was noted by matched DMSO peptide concentration of 0.4%. Multiparameter intracellular cytokine staining (ICS) assays were performed essentially as described^41^. ICS assays were performed with 10^6^ PBMCs incubated for 6 h at 37 °C with medium, 10 pg ml^–1^ phorbol myristate acetate and 1 µg ml^–1^ ionomycin (Sigma-Aldrich), or 1 µg ml^–1^ HIV-1 Env, Gag or Pol peptide pools. Cultures contained monensin (GolgiStop, BD Biosciences), brefeldin A (GolgiPlug, BD Biosciences) and 1 µg ml^–1^ mAb against human CD49d (clone 9F10). Cells were then stained with predetermined titers of mAbs against CD3 (clone SP34.2, Alexa 700), CD4 (clone L200, BV786), CD8 (clone SK1, APC H7), CD38 (clone HIT2, BUV805) and PD-1 (clone EH12.2H7, Pacific Blue), and stained intracellularly with IFN-γ (clone B27, BUV395), IL-2 (clone MQ1–17H12, BUV737), TNF-α (clone Mab11, BV650) and Ki67 (clone B56, FITC). IFN-γ backgrounds were <0.05% in PBMCs.

### Endpoints

The primary endpoints were, for safety and tolerability: (1) proportion of participants with moderate or greater reactogenicity (for example, solicited AEs) for 3 days following administration of PGT121 mAb; (2) proportion of participants with moderate or greater and/or PGT121 mAb-related unsolicited AEs, including safety laboratory parameters, following administration of PGT121 mAb for the first 56 days following administration of IP; and (3) proportion of participants with PGT121 mAb-related SAEs throughout the study period. The primary endpoints for PK were elimination half-life (*t*_1/2_), clearance (CL/F), volume of distribution (Vz/F), area under the concentration decay curve (AUC) and impact of viral load and/or ART on PGT121 disposition (elimination *t*_1/2_), clearance (CL/F), volume of distribution (Vz/F) and total exposure. The primary endpoint for antiviral activity among viremic HIV-infected adults not on ART was a change in plasma HIV-1 RNA levels from baseline (mean of pre-entry and entry values). The secondary endpoints were change in serum anti-PGT121 antibody titers from baseline, change in CD4^+^ T-cell count and frequency compared to baseline as measured by single-platform flow cytometry, and development of HIV-1 sequence variations in epitopes known to result in reduced PGT121 mAb neutralization susceptibility. Additional exploratory assessments could include, but were not limited to, the following: HIV-specific IgG/IgA binding responses by ELISA; HIV-specific cellular immune responses by ELISPOT; HIV-specific antibody function by ADCC, ADCP and ADCVI assays; resistance mutations to current ARVs; PGT121 mAb levels in mucosal secretions; changes in total HIV-1 DNA and two-long terminal repeat (LTR) circular HIV-1 DNA in resting or total CD4^+^ T cells; and in vitro neutralization of HIV isolates with participant’s serum following administration of PGT121 mAb. Available samples from time points during the optional long-term extension (LTE) phase were also used for determination of long-term durability of immune responses. The primary endpoints for safety, tolerability and PK were changed in protocol v.6.0, to include evaluation of both IV and SC routes, after the addition of subgroup 1D to the trial design. All primary endpoints were also changed in protocol v.8.0, to include evaluation of data from participants enrolled in the optional LTE phase that allowed additional follow-up of participants beyond day 168 (see Supplementary material for all protocol versions).

### Sample size and statistical analyses

Because this was an exploratory proof-of-concept trial, analyses are descriptive and sample sizes were selected based on collection of key information necessary to plan future trials. (1) The sample size for safety and tolerability analysis was 35–56 participants, according to the dose-escalation design. For life-threatening AEs related to IP: if none of the 12 (maximum 18) participants receiving IP experienced such reactions, the 95% upper confidence bound for the rate of these AEs in the population was calculated to be 26.5% (or 18.5% when *n* = 18). The frequency of moderate or greater reactogenicity events was determined and compared between groups. The frequency of SAEs adjudged possibly, probably or related to the IP was determined. All AEs were analyzed and grouped by seriousness, severity and relationship to the IP (as judged by the investigator). An interim analysis of group data was carried out according to the study schema without unblinding the study to investigators or participants. At the end of the study, a full analysis was prepared. Unused and spurious data were listed separately and excluded from the statistical analysis. Missing data were excluded from the statistical analysis. (2) The sample size for PK analysis was four PGT121 and one placebo recipient, sufficient to provide information about the PK of PGT121. The data were fit to standard two-compartment population models using the stochastic approximation expectation-maximization estimation method in Monolix (v.2019R1, Lixoft SAS, 2019). Residual variability was estimated using an additive plus proportional error model, and models were performed separately by HIV infection status. Correlations between confidence interval (Cl) and volume of the central compartment (Vc) were significant (Pearson’s correlation coefficient *P* ≤ 0.05) and were included in the models. Median-adjusted, log_10_-transformed weight was significantly correlated (Pearson’s correlation coefficient *P* ≤ 0.05) with the volume of the peripheral compartment (Vp) in the HIV-uninfected model and with Vc and Cl in the HIV-infected model, and those relationships were also included in the models. Distribution and elimination half-lives were computed using the resulting model. AUC was estimated by calculating the integral of the predicted concentration time curve from infusion to infinity. Peak concentration (*C*_max_) was computed as the maximum observed concentration. Summary descriptive results of individual estimates of PK parameters, including AUC, *C*_max_, *t*_1/2_ and clearance results, are reported by dose cohort. Correlation between PK and reported safety and pharmacodynamic outcomes also explored parameters to examine exposure–effect relationships. (3) The frequency and levels of anti-PGT121 antibodies were calculated and tabulated. (4) The sample size for virologic analysis in Group 3A was nine participants. The primary outcome for this analysis was defined as change in log_10_ viral load between Day 0 (day of infusion) and Day 7. No placebo participants were enrolled as part of this design. For the analysis of sample size and power, log_10_ viral load differences from baseline for each participant were simulated from a normal distribution, with a standard deviation of 0.5. This value was chosen by examining a study of the antiretroviral drug raltegravir, which demonstrated a mean estimated standard deviation of the change in baseline of 0.47 (ref. ^42^). This is a conservative estimate, as the variability of viral loads near the lower range might be expected also to be lower. The statistical test performed was the signed-rank test, which incorporated the ‘shift’ parameter of −0.9 log_10_. An evaluation of potential harm (increased viral load) was also performed with the signed-rank test, which examined the null hypothesis of no change in viral load (a shift of 0 log_10_ following IP administration) against the one-sided alternative hypothesis that viral load is increased following IP administration. Each efficacy test was performed at the level *α* = 0.05, and each test for harm was performed at the level 2*α* = 0.10, to provide additional sensitivity in detection of potential harm. (5) The sample size for antiviral activity in Group 3D was up to six participants. No efficacy endpoints were tested in Group 3D because participants were HIV infected with low viral loads at baseline (102–2 × 103 copies ml^–1^). Immunologic and virologic endpoints were determined as described in protocol v.8.0 section 4.1 (Supplementary material).

### Viral neutralization sensitivity modeling

As shown previously, in vitro neutralization curves can be accurately modeled as Hill curves, using IC_50_ and IC_80_ titers to fix the half-maximum and slope parameters^17^. When both IC_50_ and IC_80_ were within the concentrations tested, this procedure was used to calculate the percentage neutralization of a virus at a given PGT121 concentration. However, when IC_50_ or IC_80_ titers were outside the concentration ranges tested, we fit a Hill curve to the raw percentage neutralization versus concentration data. Curve fitting was done using absolute error minimization, utilizing the L-BFGS-B algorithm as implemented in the Python Scipy Optimize package: https://docs.scipy.org/doc/scipy/reference/optimize.minimize-lbfgsb.html. The instantaneous inhibitory potential (IIP) was calculated as –log_10_(1.0 – *f*), where *f* is the fraction neutralized at a given bNAb concentration^16^. Average IIP for baseline viruses in Extended Data Fig. 4 was calculated as follows. First, for each participant, bNAb concentrations were extracted for 1 day following infusion up to the time point of minimum viral load. The former was to account for distribution of bNAb to tissues. Next, for each of these concentrations, IIP was calculated as above for each baseline virus for that participant, and the time-averaged IIP found by numerically integrating the IIP curve as a function of time, divided by integral of unity for the same time points. Simpson’s rule, as implemented in Scipy, was used for calculations (https://docs.scipy.org/doc/scipy/reference/generated/scipy.integrate.simpson.html). These time-averaged IIP values for each baseline virus from each participant were averaged to determine the average IIP for each participant. The same procedure was followed to determine the average rebound virus IIP, as shown in Extended Data Fig. 3, with the exception of using PGT121 concentrations from the time of minimum viral load to that to rebound.

### Viral load decline and rebound characteristics

We used three characteristics of viral load trajectories in each participant to capture the antiviral efficacy of PGT121: (1) maximum viral load drop from baseline, (2) initial decline rate of viral load following infusion and (3) time to viral rebound. The calculation of the first characteristic was straightforward for all participants, except for four participants for whom the minimum viral load was below the lower limit of quantitation (LLoQ = 40 copies ml^–1^); for such participants the minimum viral load was assumed to be 40 copies ml^–1^. For the second characteristic, we calculated the average slope of log_10_ viral load over time using the following strategy. For each participant, we first identified the time window of consistent viral load decline as that between minimum viral load and the earliest time point following infusion such that viral load consistently fell over time in this window. This strategy ensured that the early nonmonotonic behavior of viral load (as seen for participants 1536, 4236 and so on) would not impact calculation of decline rate. In case of multiple time points with minimum viral load, the earliest was chosen. For all time points in the window of viral load decline, the rate was calculated as the slope of log_10_ viral load versus time curve. Slopes were calculated using a robust linear regression algorithm, the Theil-Sen estimator as implemented in Scipy (https://docs.scipy.org/doc/scipy-0.15.1/reference/generated/scipy.stats.mstats.theilslopes.html), and time points with viral load below LLoQ were ignored. For two participants, 1536 and 4236, decline rates could not be determined due to the combination of highly nonlinear early viral load kinetics and the minimum viral load dropping below LLoQ; these participants are excluded from the data in Extended Data Fig. 3c,d. For two participants, 9372 and 5257, because viral loads did not decline sufficiently due to PGT121 resistance at baseline, the average slope of viral load for the first 10 days was used. The third characteristic was the time to rebound, for which we assumed that rebound or restoration of viremia is characterized by viral loads 0.5 log_10_ below baseline preinfusion viremia, or higher. Thus, the time to rebound was calculated as that between infusion and the earliest time point that met the rebound viral load criterion.

### Sequence analysis

Phylogenetic trees were constructed using IQ-tree^43^, and recombination analysis was performed with RAPR^25^, both via interfaces at the Los Alamos Database. Sequences are available at GenBank (see Supplementary Table 12 for accession numbers).

### Important changes to methods after trial commencement

Eligibility criteria were changed in protocol v.5.0 to remove the inclusion criteria of CD4 nadir >200, and to expand the age range for Group 2 and 3 participants, from 18–50 to 18–65 years of age. These changes were made to facilitate recruitment and to meet the approval of the safety monitoring committee (SMC), protocol safety review team (PSRT) and the BIDMC IRB. Protocol v.6.0 added a subcutaneous administration arm to Part 1. The study design was changed in protocol v.7.0 to eliminate subgroups 3B, 3C, 3E and 3F, which would have evaluated lower doses of PGT121 in viremic individuals but were not feasible to enroll. A LTE was added in protocol v.8.0 to allow additional follow-up of participants beyond day 168. Protocol v.6.0, 7.0 and 8.0 were also approved by the SMC, PSRT and BIDMC IRB.

### Reporting Summary

Further information on research design is available in the Nature Research Reporting Summary linked to this article.

## Online content

Any methods, additional references, Nature Research reporting summaries, source data, extended data, supplementary information, acknowledgements, peer review information; details of author contributions and competing interests; and statements of data and code availability are available at 10.1038/s41591-021-01509-0.

## Supplementary information

Supplementary Information Supplementary Tables 1–12, methods, CONSORT 2010 checklist, protocol and amendments.

Reporting Summary

Supplementary Table 1 Spreadsheet of HIV-1 strains tested and recorded in CATNAP, the Los Alamos HIV database tool.

## Data Availability

All viral sequences identified in this study are publicly available via GenBank (see Supplementary Table 12 for GenBank accession numbers). Comprehensive data on HIV genetic sequences and immunological epitopes used for analysis in this study are publicly available via Los Alamos National Laboratory (hiv.lanl.gov/content/index, with most relevant sequences included in Supplementary material). Additional requests for access to the study data can be submitted to D.H.B. (dbarouch@bidmc.harvard.edu). Data containing protected health information or that may identify a participant are restricted, and therefore additional data requests must be reviewed before release.
