# Supplementary material for: Safety, pharmacokinetics and antiviral activity of PGT121, a broadly neutralizing monoclonal antibody against HIV-1: a randomized, placebo-controlled, phase 1 clinical trial
Source: Nat Med. 2021 Oct 7;27(10):1718–24. doi: 10.1038/s41591-021-01509-0 (PMC8516645; doi:10.1038/s41591-021-01509-0)
Supplement: Supplementary file 1 — Supplementary Tables 1–12, methods, CONSORT 2010 checklist, protocol and amendments. [file 41591_2021_1509_MOESM1_ESM.pdf]

nature portfolio

https://doi.org/10.1038/s41591-021-01509-0

Supplementary information

Safety, pharmacokinetics and antiviral activity of PGT121, a broadly neutralizing monoclonal antibody against HIV-1: a randomized, placebo-controlled, phase 1 clinical trial

In the format provided by the authors and unedited

Supplemental Table 1. Summary of Screen Failures

Inclusion/Exclusion/Other Category	Inclusion/Exclusion/Other Criterion	Number of Times Item Marked Ineligible
Inclusion, Exclusion and Other	Number of participants failing any eligibility criterion	50
Inclusion	Willing to comply with protocol	18
	Informed consent obtained	2
	Female willing to undergo pregnancy testing	1
	CD4 cells	1
	Currently on ART	3
Exclusion	Any exclusion any criterion	47
	Clinically significant medical condition	3
	Alcohol or substance use	2
	Bleeding disorder	1
	Not in the best interest of volunteer	4
	Body mass index	4
	Infectious disease	1
	Any abnormality Group 1	1
	Hemoglobin	2
	Absolute neutrophil count	1
	Absolute lymphocyte count	1
	Platelets	2
	aPTT	1
	INR	1
	Sodium	1
	Potassium	1
	Creatinine	3

Supplemental Table 1. Summary of Screen Failures - *continued*

Inclusion/Exclusion/Other Category	Inclusion/Exclusion/Other Criterion	Number of Times Item Marked Ineligible
	AST	2
	ALT	1
	Total bilirubin	3
	Alkaline phosphatase	1
	Albumin	1
	Creatine kinase	2
	C-reactive protein	2
	C3 complement	1
	C4 complement	2
	Protein	1
	Blood	1
	Any abnormal. Groups 2&3	1
Other Reasons	Any other reason	14
	IP side effects	1
	Enrollment limit reached	2
	Withdrawal of consent	4
	Partner/family influence	1
	Other/Not further specified	6

Supplemental Table 2: Demographic and Other Baseline Characteristics

	Group 1 (HIV-uninfected)		Group 2 (HIV-infected)		Group 3 (HIV-infected)
	**Placebo (N=4)**	**Active (N=16)**	**Placebo (N=3)**	**Active (N=12)**	**Active (N=13)**
Sex at Birth					
Female	1 (25.0%)	10 (62.5%)	0	2 (16.7%)	1 (7.7%)
Male	3 (75.0%)	6 (37.5%)	3 (100.0%)	10 (83.3%)	12 (92.3%)
Race					
Asian	2 (50.0%)	2 (12.5%)	0	0	0
Black	0	1 (6.3%)	0	3 (25.0%)	5 (38.5%)
Unknown (Did not specify)	1 (25.0%)	0	0	0	0
Unknown (Mixed race)	0	0	1 (33.3%)	0	0
Unknown (Other)	0	1 (6.3%)	0	0	0
White	1 (25.0%)	11 (68.8%)	2 (66.7%)	9 (75.0%)	8 (61.5%)
White, American Indian	0	1 (6.3%)	0	0	0
Ethnicity					
Hispanic or Latino	2 (50.0%)	2 (12.5%)	1 (33.3%)	1 (8.3%)	5 (38.5%)
Not Hispanic and Not Latino	2 (50.0%)	14 (87.5%)	2 (66.7%)	11 (91.7%)	8 (61.5%)
Age (yrs)					
Mean	24.5	27.7	44.3	44.2	31.2
Range	19-28	19-48	29-56	27-62	20-51
Height (cm)					
Mean	170.6	168.1	177.3	176.0	176.6
Range	152.9-185	153.7-183.9	168.9-186.1	162.9-186.8	157.5-193.1
Weight (kg)					
Mean	79.6	71.3	82.6	81.9	78.7
Range	70.6-94.1	58.4-92.7	65.7-102.1	68.3-107.8	51.5-103.1
BMI (kg/m^2)					
Mean	27.6	25.1	26.0	26.4	25.1
Range	24.0-34.2	21.4-30.4	23.0-29.5	21.3-31.5	19.6-31.9
Years Since HIV-1 Diagnosis					
Mean	N/A	N/A	15.3	13.9	2.5
Range	-	-	6.0-27.0	1.0-27.0	0.0-20.0

Supplemental Table 3. Grade 1 or Greater Solicited Symptoms, by Group, Dose Level, and Severity

		Groups 1A-1D (HIV-uninfected)					Groups 2A-2C (HIV-infected)				Group 3A (HIV-infected)	Group 3D (HIV-infected)
		**Placebo (N=4)**	**3 mg/kg IV (N=4)**	**10 mg/kg IV (N=4)**	**30 mg/kg IV (N=4)**	**3 mg/kg SC (N=4)**	**Placebo (N=3)**	**3 mg/kg IV (N=4)**	**10 mg/kg IV (N=4)**	**30 mg/kg IV (N=4)**	**30 mg/kg IV (N=9)**	**30 mg/kg IV (N=4)**
**Reaction**	**Severity**	**n (%)**	**n (%)**	**n (%)**	**n (%)**	**n (%)**	**n (%)**	**n (%)**	**n (%)**	**n (%)**	**n (%)**	**n (%)**
**Any Symptom (Local or Systemic)**	Grade 1	1 (25.0)	1 (25.0)	1 (25.0)	2 (50.0)	1 (25.0)	2 (66.7)	0	2 (50.0)	1 (25.0)	2 (22.2)	1 (25.0)
	Grade 2	1 (25.0)	1 (25.0)	1 (25.0)	1 (25.0)	0	0	0	1 (25.0)	1 (25.0)	0	1 (25.0)
**Any Local Symptom**	Grade 1	2 (50.0)	1 (25.0)	1 (25.0)	3 (75.0)	1 (25.0)	2 (66.7)	0	2 (50.0)	0	1 (11.1)	0
	Grade 2	0	1 (25.0)	1 (25.0)	0	0	0	0	1 (25.0)	0	0	0
Pain	Grade 1	0	0	2 (50.0)	0	0	1 (33.3)	0	2 (50.0)	0	1 (11.1)	0
	Grade 2	0	1 (25.0)	0	0	0	0	0	0	0	0	0
Tenderness	Grade 1	2 (50.0)	1 (25.0)	2 (50.0)	3 (75.0)	1 (25.0)	1 (33.3)	0	2 (50.0)	0	1 (11.1)	0
	Grade 2	0	0	0	0	0	0	0	1 (25.0)	0	0	0
Pruritus	Grade 1	1 (25.0)	1 (25.0)	0	1 (25.0)	0	0	0	0	0	0	0
	Grade 2	0	0	0	0	0	0	0	0	0	0	0
Erythema/Skin Discoloration	Grade 1	0	0	0	1 (25.0)	0	0	0	0	0	0	0
	Grade 2	0	0	1 (25.0)	0	0	0	0	0	0	0	0
Swelling/Hardening	Grade 1	0	0	0	0	1 (25.0)	0	0	1 (25.0)	0	0	0
	Grade 2	0	0	0	0	0	0	0	0	0	0	0
**Any Systemic Symptom**	Grade 1	1 (25.0)	1 (25.0)	1 (25.0)	1 (25.0)	0	0	0	1 (25.0)	1 (25.0)	2 (22.2)	1 (25.0)
	Grade 2	1 (25.0)	0	0	1 (25.0)	0	0	0	1 (25.0)	1 (25.0)	0	1 (25.0)
Chills	Grade 1	0	1 (25.0)	0	1 (25.0)	0	0	0	0	0	0	0
	Grade 2	0	0	0	0	0	0	0	0	0	0	0
Malaise	Grade 1	0	1 (25.0)	0	0	0	0	0	0	1 (25.0)	0	1 (25.0)
	Grade 2	1 (25.0)	0	0	1 (25.0)	0	0	0	0	0	0	0

Supplemental Table 3. Grade 1 or Greater Solicited Symptoms, By Group, Dose Level, and Severity - *continued*

		Groups 1A-1D (HIV-uninfected)					Groups 2A-2C (HIV-infected)				Group 3A (HIV-infected)	Group 3D (HIV-infected)
		Placebo (N=4)	3 mg/kg IV (N=4)	10 mg/kg IV (N=4)	30 mg/kg IV (N=4)	3 mg/kg SC (N=4)	Placebo (N=3)	3 mg/kg IV (N=4)	10 mg/kg IV (N=4)	30 mg/kg IV (N=4)	30 mg/kg IV (N=9)	30 mg/kg IV (N=4)
Reaction	Severity	n (%)	n (%)	n (%)	n (%)	n (%)	n (%)	n (%)	n (%)	n (%)	n (%)	n (%)
Myalgia	Grade 1	2 (50.0)	0	1 (25.0)	1 (25.0)	0	0	0	0	0	0	0
	Grade 2	0	0	0	0	0	0	0	0	0	0	0
Arthralgia	Grade 1	0	0	0	1 (25.0)	0	0	0	0	0	0	0
	Grade 2	0	0	0	0	0	0	0	0	0	0	0
Headache	Grade 1	1 (25.0)	1 (25.0)	0	1 (25.0)	0	0	0	1 (25.0)	0	2 (22.2)	0
	Grade 2	1 (25.0)	0	0	1 (25.0)	0	0	0	1 (25.0)	1 (25.0)	0	0
Nausea	Grade 1	0	0	1 (25.0)	0	0	0	0	0	0	0	0
	Grade 2	0	0	0	1 (25.0)	0	0	0	0	0	0	0
Vomiting	Grade 1	0	0	0	0	0	0	0	0	0	0	0
	Grade 2	0	0	0	0	0	0	0	0	0	0	0
Maximum Temperature (Grade 1: ≥38.0 and <38.6, Grade 2: ≥38.6 and <39.3)	Grade 1	0	0	0	0	0	0	0	0	0	0	0
	Grade 2	0	0	0	0	0	0	0	0	0	0	1 (25.0)

Supplemental Table 4. Unsolicited AEs Through Study Day 56, By Group

	Group 1 (HIV-uninfected)										Group 2 (HIV-infected)										Group 3 (HIV-infected)				
	Placebo (N=4)					Active (N=16)					Placebo (N=3)					Active (N=12)					Active (N=13)				
	AEs				Vols with AE	AEs				Vols with AE	AEs				Vols with AE	AEs				Vols with AE	AEs				Vols with AE
	All	Severity Grade				All	Severity Grade				All	Severity Grade				All	Severity Grade				All	Severity Grade			
**MedDRA PT**	#	Gr1 (#)	Gr2 (#)	Gr3 (#)	n (%)	#	Gr1 (#)	Gr2 (#)	Gr3 (#)	n (%)	#	Gr1 (#)	Gr2 (#)	Gr3 (#)	n (%)	#	Gr1 (#)	Gr2 (#)	Gr3 (#)	n (%)	#	Gr1 (#)	Gr2 (#)	Gr3 (#)	n (%)
Fatigue						1	0	1	0	1 (6.3)						2	1	1	0	2 (16.7)	1	1	0	0	1 (7.7)
Headache						1	1	0	0	1 (6.3)											1	1	0	0	1 (7.7)
Nasal congestion	1	1	0	0	1 (25.0)	1	1	0	0	1 (6.3)															
Upper respiratory tract infection						2	1	1	0	2 (12.5)															
Urinary tract infection						2	0	2	0	2 (12.5)															
Vessel puncture site bruise						1	1	0	0	1 (6.3)						1	1	0	0	1 (8.3)					
Viral infection	1	1	0	0	1 (25.0)	1	1	0	0	1 (6.3)															
Abdominal pain																1	0	1	0	1 (8.3)					
Alanine aminotransferase increased																					1	0	1	0	1 (7.7)
Arthropod bite																					1	1	0	0	1 (7.7)
Bursitis											1	0	0	1	1 (33.3)										
CD4 lymphocytes decreased																					1	0	1	0	1 (7.7)
Cough	1	1	0	0	1 (25.0)																				
Dizziness																					1	1	0	0	1 (7.7)
Dry skin																1	1	0	0	1 (8.3)					
Dyspepsia						1	1	0	0	1 (6.3)															
Exposure to communicable disease																1	0	1	0	1 (8.3)					
Folliculitis																1	0	1	0	1 (8.3)					
Fungal infection						1	0	1	0	1 (6.3)															
Gastroenteritis						1	0	1	0	1 (6.3)															

Supplemental Table 4. Unsolicited AEs Through Study Day 56, By Group and MedDRA PT - *continued*

	Group 1 (HIV-uninfected)										Group 2 (HIV-infected)										Group 3 (HIV-infected)				
	Placebo (N=4)					Active (N=16)					Placebo (N=3)					Active (N=12)					Active (N=13)				
	AEs				Vols with AE	AEs				Vols with AE	AEs				Vols with AE	AEs				Vols with AE	AEs				Vols with AE
	All	Severity Grade				All	Severity Grade				All	Severity Grade				All	Severity Grade				All	Severity Grade			
**MedDRA PT**	**#**	**Gr1 (#)**	**Gr2 (#)**	**Gr3 (#)**	**n (%)**	**#**	**Gr1 (#)**	**Gr2 (#)**	**Gr3 (#)**	**n (%)**	**#**	**Gr1 (#)**	**Gr2 (#)**	**Gr3 (#)**	**n (%)**	**#**	**Gr1 (#)**	**Gr2 (#)**	**Gr3 (#)**	**n (%)**	**#**	**Gr1 (#)**	**Gr2 (#)**	**Gr3 (#)**	**n (%)**
Haematuria																1	1	0	0	1 (8.3)					
Joint injury																					1	0	1	0	1 (7.7)
Nasopharyngitis																1	1	0	0	1 (8.3)					
Rectal haemorrhage																1	1	0	0	1 (8.3)					
Syphilis																1	0	1	0	1 (8.3)					
Throat irritation						1	1	0	0	1 (6.3)															
Tonsillitis						1	0	0	1	1 (6.3)															
Vessel puncture site haematoma											1	1	0	0	1 (33.3)										
Viral upper respiratory tract infection						1	1	0	0	1 (6.3)															
Vision blurred																					1	1	0	0	1 (7.7)
**Total**	**3**	**3**	**0**	**0**	**3 (75.0)**	**15**	**8**	**6**	**1**	**8 (50.0)**	**2**	**1**	**0**	**1**	**1 (33.3)**	**11**	**6**	**5**	**0**	**7 (58.3)**	**8**	**5**	**3**	**0**	**6 (46.2)**

Supplemental Table 5. Related, Unsolicited Adverse Events Through Study Day 56, By Group

	Group 1 (HIV-uninfected)										Group 2 (HIV-infected)										Group 3 (HIV-infected)				
	Placebo (N=4)					Active (N=16)					Placebo (N=3)					Active (N=12)					Active (N=13)				
	AEs				Vols with AE	AEs				Vols with AE	AEs				Vols with AE	AEs				Vols with AE	AEs				Vols with AE
	All	Severity Grade				All	Severity Grade				All	Severity Grade				All	Severity Grade				All	Severity Grade			
**MedDRA PT**	**#**	**Gr1 (#)**	**Gr2 (#)**	**Gr3 (#)**	**n (%)**	**#**	**Gr1 (#)**	**Gr2 (#)**	**Gr3 (#)**	**n (%)**	**#**	**Gr1 (#)**	**Gr2 (#)**	**Gr3 (#)**	**n (%)**	**#**	**Gr1 (#)**	**Gr2 (#)**	**Gr3 (#)**	**n (%)**	**#**	**Gr1 (#)**	**Gr2 (#)**	**Gr3 (#)**	**n (%)**
Fatigue						1	0	1	0	1 (6.3)											1	1	0	0	1 (7.7)
Gastroenteritis						1	0	1	0	1 (6.3)															
Headache						1	1	0	0	1 (6.3)															
**Total**						**3**	**1**	**2**	**0**	**3 (18.8)**											**1**	**1**	**0**	**0**	**1 (7.7)**

* Relationship to investigational product was assessed as possible, probable or definite by the site investigator.

Supplemental Table 6. CD4 (cells/uL and %) By Group and Timepoint

			Visits											
			01 Scr	02 Day 0	02A Day 1	02C Day 3	03 Day 7	05 Day 14	07 Day 28	09 Day 56	11 Day 84	12 Day 112	13 Day 140	14 Day 168
**CD4 (cells/uL)**														
Group 2 (HIV-infected)	Placebo (N=3)	Mean, Median	687.7, 766.0	734.3, 738.0	NA	NA	630.0, 706.0	632.5, 632.5	592.0, 619.0	694.7, 718.0	NA	NA	NA	849.0, 886.0
		Min, Max	470, 827	708, 757	NA	NA	432, 752	541, 724	453, 704	471, 895	NA	NA	NA	608, 1053
	Active (N=12)	Mean, Median	763.3, 805.0	721.7, 705.5	NA	NA	766.0, 783.0	764.0, 763.5	723.6, 680.0	742.4, 685.5	975.0, 975.0	1023.0, 1023.0	849.0, 849.0	828.3, 795.0
		Min, Max	331, 1391	333, 1231	NA	NA	377, 1100	371, 1207	389, 1347	378, 1200	975, 975	853, 1193	849, 849	386, 1586
Group 3 (HIV-infected)	Active (N=13)	Mean, Median	649.5, 614.0	694.4, 567.5	NA	NA	690.0, 701.0	666.8, 589.5	751.5, 571.0	729.4, 629.0	NA	NA	NA	750.8, 673.0
		Min, Max	364, 972	252, 1349	NA	NA	285, 1058	268, 1030	290, 1445	300, 1374	NA	NA	NA	280, 1846
**CD4 (%)**														
Group 2 (HIV-infected)	Placebo (N=3)	Mean, Median	35.73, 34.80	39.67, 40.00	NA	NA	38.47, 40.00	37.05, 37.05	36.83, 36.40	38.73, 37.30	NA	NA	NA	38.77, 39.00
		Min, Max	33, 39.4	32.9, 46.1	NA	NA	33.6, 41.8	36, 38.1	35, 39.1	34, 44.9	NA	NA	NA	33, 44.3
	Active (N=12)	Mean, Median	37.92, 38.25	37.65, 37.95	NA	NA	36.16, 35.60	36.87, 34.90	36.07, 35.50	34.94, 35.95	44.30, 44.30	47.90, 47.90	46.00, 46.00	37.50, 36.70
		Min, Max	19, 53.5	20, 58.6	NA	NA	20, 46.5	20, 56.2	21, 49.9	19, 46	44.3, 44.3	39, 56.8	46, 46	20, 60
Group 3 (HIV-infected)	Active (N=13)	Mean, Median	32.66, 34.60	34.81, 34.70	NA	NA	33.33, 32.55	35.44, 37.80	34.34, 34.00	34.53, 34.70	NA	NA	NA	37.57, 36.00
		Min, Max	14, 45.1	14, 51.9	NA	NA	15, 49.3	14.1, 51.8	11.6, 51.6	12.1, 52.4	NA	NA	NA	14, 56.6

Supplemental Table 7. HIV-uninfected PK Parameters: Estimate, 95 % Confidence Interval (CI) and % Relative Standard Error (RSE)

Parameter	Estimate	95% CI	%RSE
**Fixed Effects**			
F	0.366	(0.283, 0.449)	11.552
ka	0.371	(0.303, 0.44)	9.415
CL	0.272	(0.236, 0.307)	6.682
V_c	3.351	(2.93, 3.773)	6.418
Q	1.037	(0.821, 1.253)	10.616
V_p	4.526	(4.126, 4.926)	4.507
$\beta_{Vp_{wt}}$	0.519	(0.105, 0.933)	40.671
**Standard Deviation of the Random Effects**			
ω_{CL}	0.227	(0.141, 0.312)	19.300
ω_{Vc}	0.221	(0.138, 0.304)	19.234
ω_{Q}	0.302	(0.135, 0.47)	28.238
ω_{Vp}	0.064	(0.007, 0.121)	45.469
**Correlations**			
ρ_{VcCL}	0.795	(0.584, 1.007)	13.572
**Error Model Parameters**			
σ (constant)	0.245	(0.188, 0.301)	11.752
σ(proportional)	0.086	(0.075, 0.096)	6.312

Supplemental Table 8. HIV-uninfected: Half-life Median (Min, Max) Estimates

Parameter	Estimate (days)	(Minimum, Maximum)
Distribution Half-life	1.1	(0.8, 1.5)
Elimination Half-life	22.0	(15.5, 28.8)

Supplemental Table 9. Pharmacokinetic parameters after PGT121 administration.

	N	Cmax (µg/mL)	AUC (µg*d/mL)	Distribution Half-life (d)	Elimination Half-life (d)	CL/F (L/d)	Vz/F (L)
3 mg/kg IV HIV-	4	52 (6)	642 (144)	1.1 (0.3)	19.7 (4.3)	0.34 (0.08)	9.36 (1.08)
10 mg/kg IV HIV-	4	254 (46)	2899 (365)	1.3 (0.2)	22.8 (0.9)	0.24 (0.04)	7.84 (1.05)
30 mg/kg IV HIV-	4	759 (168)	9003 (2468)	1.2 (0.2)	23.9 (3.6)	0.24 (0.02)	8.20 (0.70)
3 mg/kg SC HIV-	4	10 (1)	326 (62)	1.0 (0.2)	22.3 (2.9)	0.73 (0.17)	23.06 (11.1)
3 mg/kg IV HIV+	4	54 (9)	405 (12)	1.1 (0.4)	13.3 (1.8)	0.58 (0.09)	11.10 (2.03)
10 mg/kg IV HIV+	4	241 (32)	1920 (193)	2.4 (1.5)	21.2 (5.9)	0.42 (0.08)	12.48 (2.96)
30 mg/kg IV HIV+	4	929 (174)	5799 (1450)	1.4 (0.4)	16.6 (3.5)	0.48 (0.17)	11.04 (2.67)
30 mg/kg IV HIV+ off ART high VL	9	731 (203)	4460 (902)	1.2 (0.6)	12.5 (3)	0.56 (0.10)	10.11 (3.11)
30 mg/kg IV HIV+ off ART low VL	4	648 (49)	4206 (1045)	1.3 (0.2)	14 (4.3)	0.57 (0.32)	10.29 (3.08)

Data are mean (SD). Pharmacokinetic parameters were calculated for all participants who received PGT121 from the 2 compartment model. Half-lives was calculated from all doses. Cmax = maximum serum concentration. AUC=area under the concentration versus time curve. CL/F=apparent clearance. Vz/F = apparent volume of distribution. IV= intravenous. SC= subcutaneous.

Supplemental Table 10. Summary of HIV Viral Loads, By Timepoint (Groups 2 and 3 only)

	Groups 2A-2C (HIV-Infected)											
	Placebo			3 IV			10 IV			30 IV		
Study Day	Detectable (n/N)	Median (IQR)	Range	Detectable (n/N)	Median (IQR)	Range	Detectable (n/N)	Median (IQR)	Range	Detectable (n/N)	Median (IQR)	Range
Screening	2/3	20 (17 - 20)	<20 - 20	1/4	17 (17 - 18)	<20 - <20	3/4	20 (18 - 20)	<20 - 20	1/4	17 (17 - 40)	<20 - 63
0	1/3	17 (17 - 20)	<20 - 20	0/4	17 (17 - 17)	<20 - <20	0/4	17 (17 - 17)	<20 - <20	3/4	20 (18 - 30)	<20 - 40
1	0/3	17 (17 - 17)	<20 - <20	0/4	17 (17 - 17)	<20 - <20	2/4	18 (17 - 20)	<20 - 20	1/4	17 (17 - 18)	<20 - 20
2	1/2	18 (17 - 20)	<20 - <20	0/4	17 (17 - 17)	<20 - <20	1/2	87 (17 - 158)	<20 - 158	1/4	17 (17 - 18)	<20 - 20
3	1/3	17 (17 - 20)	<20 - 20	1/4	17 (17 - 18)	<20 - <20	0/4	17 (17 - 17)	<20 - <20	1/4	17 (17 - 40)	<20 - 63
7	0/3	17 (17 - 17)	<20 - <20	0/4	17 (17 - 17)	<20 - <20	2/3	20 (17 - 20)	<20 - 20	1/3	17 (17 - 63)	<20 - 63
14	1/3	17 (17 - 20)	<20 - 20	1/4	17 (17 - 18)	<20 - <20	0/4	17 (17 - 17)	<20 - <20	2/4	18 (17 - 89)	<20 - 158
21	1/3	17 (17 - 20)	<20 - 20	0/4	17 (17 - 17)	<20 - <20	1/3	17 (17 - 20)	<20 - 20	1/4	17 (17 - 18)	<20 - 20
28	1/3	17 (17 - 63)	<20 - 63	1/4	17 (17 - 18)	<20 - <20	0/3	17 (17 - 17)	<20 - <20	3/4	20 (18 - 20)	<20 - 20
42	0/3	17 (17 - 17)	<20 - <20	0/4	17 (17 - 17)	<20 - <20	0/3	17 (17 - 17)	<20 - <20	3/4	26 (18 - 215)	<20 - 398
56	0/3	17 (17 - 17)	<20 - <20	1/3	17 (17 - 200)	<20 - 200	2/3	20 (17 - 20)	<20 - 20	2/4	24 (17 - 41)	<20 - 50
70	1/3	17 (17 - 20)	<20 - 20	1/4	17 (17 - 18)	<20 - <20	0/4	17 (17 - 17)	<20 - <20	2/4	18 (17 - 26)	<20 - 32
84	2/3	20 (17 - 20)	<20 - 20	1/3	17 (17 - 50)	<20 - 50	1/4	17 (17 - 18)	<20 - 20	1/3	17 (12 - 20)	<30 - 20
112	2/3	20 (17 - 40)	<20 - 40	1/3	17 (17 - 20)	<20 - 20	3/4	20 (18 - 20)	<20 - 20	1/3	17 (12 - 32)	<30 - 32
140	1/3	17 (17 - 50)	<20 - 50	1/4	17 (17 - 18)	<20 - 20	2/4	18 (17 - 20)	<20 - 20	1/3	12 (12 - 63)	<30 - 63
168	3/3	20 (20 - 32)	20 - 32	0/4	17 (17 - 17)	<20 - <20	1/4	17 (17 - 18)	<20 - 20	1/3	12 (12 - 32)	<30 - 32

Detectable values <LLOQ (lower limit of quantitation) were imputed by the LLOQ corresponding to the RNAPCR KIT used.
Where LLOQ=40 (Real Time), 20 (COBAS) and 30 (Aptima) copies/mL.
Not Detectable values were imputed as follows: 25 (Real Time), 16.5 (COBAS) and 12 (Aptima) copies/mL.

Supplemental Table 10. Summary of HIV Viral Loads, By Timepoint (Groups 2 and 3 only) *- continued*

	Groups 3A and 3D (HIV-Infected)					
	30 IV			30 IV		
Study Day	Detectable (n/N)	Median (IQR)	Range	Detectable (n/N)	Median (IQR)	Range
Screening	9/9	28940 (8760 - 44800)	2450 - 72100	4/4	220 (180 - 310)	170 - 370
0	9/9	21040 (9660 - 28990)	1250 - 55700	4/4	270 (185 - 550)	180 - 750
1	9/9	20200 (7850 - 22680)	1250 - 39800	4/4	385 (145 - 575)	80 - 590
2	9/9	9450 (5100 - 21380)	1270 - 27500	4/4	140 (75 - 445)	60 - 700
3	8/8	5565 (3165 - 20360)	800 - 36000	4/4	100 (65 - 125)	50 - 130
7	9/9	860 (490 - 12300)	40 - 34000	3/4	40 (33 - 40)	<40 - <40
10	8/8	1980 (360 - 8755)	<40 - 48500	2/4	33 (25 - 40)	<40 - <40
14	9/9	5600 (1350 - 10200)	200 - 41200	2/4	33 (25 - 60)	<40 - 80
21	8/8	14670 (6330 - 30225)	200 - 40000	3/4	40 (33 - 90)	<40 - 140
28	9/9	24090 (8790 - 31100)	540 - 69700	3/4	75 (33 - 330)	<40 - 550
42	9/9	14460 (9470 - 30160)	3150 - 98600	3/4	75 (33 - 925)	<40 - 1740
56	8/8	7905 (4340 - 21205)	1860 - 65900	2/4	123 (25 - 770)	<40 - 1320
70	6/7	7900 (40 - 15860)	<40 - 60200	1/4	25 (25 - 33)	<40 - <40
84	4/8	58 (25 - 5925)	<40 - 29170	3/4	40 (33 - 370)	<40 - 700
112	3/7	25 (25 - 8280)	<40 - 11730	3/4	40 (33 - 195)	<40 - 350
140	3/6	26 (25 - 15640)	<40 - 22310	3/4	40 (33 - 45)	<40 - 50
168	3/7	25 (25 - 40)	23 - 16070	2/4	33 (25 - 325)	<40 - 610

Detectable values <LLOQ (lower limit of quantitation) were imputed by the LLOQ corresponding to the RNAPCR KIT used.
Where LLOQ=40 (Real Time), 20 (COBAS) and 30 (Aptima) copies/mL.
Not Detectable values were imputed as follows: 25 (Real Time), 16.5 (COBAS) and 12 (Aptima) copies/mL.

Supplemental Table 11. Correlation of HIV Pseudovirus Sensitivity to 10-1074 and PGT121

N	10-1074	PGT121	%
308	Sensitive	Sensitive	58%
21	Sensitive	Resistant	3.9%
50	Resistant	Sensitive	9.4%
151	Resistant	Resistant	28%

Supplemental Table 12. GenBank Accession Numbers

BankIt2470553	6292_BL_C4_1	MZ396980
BankIt2470553	6292_BL_B2_1	MZ396981
BankIt2470553	6292_BL_D2_3	MZ396982
BankIt2470553	6292_BL_G2_1	MZ396983
BankIt2470553	6292_BL_F2_2	MZ396984
BankIt2470553	6292_BL_H4_2	MZ396985
BankIt2470553	6292_BL_D4_1	MZ396986
BankIt2470553	6292_BL_G4_3	MZ396987
BankIt2470553	6292_BL_A4_1	MZ396988
BankIt2470553	6292_V7_H7_4	MZ396989
BankIt2470553	6292_V7_G10_4	MZ396990
BankIt2470553	6292_V7_G8_4	MZ396991
BankIt2470553	6292_V7_F5_2	MZ396992
BankIt2470553	6292_V7_B9_2	MZ396993
BankIt2470553	6292_V7_C8_1	MZ396994
BankIt2470553	6292_V7_A6_1	MZ396995
BankIt2470553	6292_V7_A11_1	MZ396996
BankIt2470553	6292_V7_F12_4	MZ396997
BankIt2470553	6292_V7_F10_2	MZ396998
BankIt2470553	6292_V7_C12_1	MZ396999
BankIt2470553	9372_BL_E2_1	MZ397000
BankIt2470553	9372_BL_C3_3	MZ397001
BankIt2470553	9372_BL_H4_1	MZ397002
BankIt2470553	9372_BL_E11_1	MZ397003
BankIt2470553	9372_BL_A10_1	MZ397004
BankIt2470553	9372_BL_C5_2	MZ397005
BankIt2470553	9372_BL_F5_4	MZ397006
BankIt2470553	9372_BL_F7_1	MZ397007
BankIt2470553	9372_BL_E6_1	MZ397008
BankIt2470553	9372_BL_E7_1	MZ397009
BankIt2470553	9372_V7_G2_2	MZ397010
BankIt2470553	9372_V7_E10_3	MZ397011
BankIt2470553	9372_V7_F1_2	MZ397012
BankIt2470553	9372_V7_G4_2	MZ397013
BankIt2470553	6775_V7_D3_3	MZ397014
BankIt2470553	6775_V7_D2_4	MZ397015
BankIt2470553	6775_V7_E5_1	MZ397016
BankIt2470553	6775_V7_D6_3	MZ397017
BankIt2470553	6775_V7_D7_3	MZ397018
BankIt2470553	6775_BL_G3_4	MZ397019
BankIt2470553	6775_BL_G10_2	MZ397020
BankIt2470553	6775_BL_H10_4	MZ397021
BankIt2470553	6775_BL_H7_3	MZ397022
BankIt2470553	6775_BL_F5_2	MZ397023
BankIt2470553	6775_BL_B10_3	MZ397024
BankIt2470553	6775_BL_E5_2	MZ397025
BankIt2470553	6775_BL_E3_3	MZ397026
BankIt2470553	6113_V14_A10_1	MZ397027
BankIt2470553	6113_V14_A11_1	MZ397028
BankIt2470553	6113_V14_C7_3	MZ397029

Supplemental Table 12. GenBank Accession Numbers - *continued*

BankIt2470553	6113_V14_B5_2	MZ397030
BankIt2470553	6113_V14_D8_2	MZ397031
BankIt2470553	6113_V14_E1_2	MZ397032
BankIt2470553	6113_V14_G11_1	MZ397033
BankIt2470553	6113_BL_A5_4	MZ397034
BankIt2470553	6113_BL_C10_4	MZ397035
BankIt2470553	6113_BL_A3_4	MZ397036
BankIt2470553	6113_BL_D6_4	MZ397037
BankIt2470553	6113_BL_B4_3	MZ397038
BankIt2470553	2936_V9_B3_2	MZ397039
BankIt2470553	2936_V9_C3_2	MZ397040
BankIt2470553	2936_V9_B4_3	MZ397041
BankIt2470553	2936_V9_A3_3	MZ397042
BankIt2470553	2936_V9_C5_2	MZ397043
BankIt2470553	2936_V9_D3_3	MZ397044
BankIt2470553	2936_V9_E3_3	MZ397045
BankIt2470553	2936_V9_F2_3	MZ397046
BankIt2470553	2936_BL_E10_2	MZ397047
BankIt2470553	2936_BL_B10_3	MZ397048
BankIt2470553	2936_BL_C1_3	MZ397049
BankIt2470553	2990_V7_C2_3	MZ397050
BankIt2470553	2990_BL_E2_8	MZ397051
BankIt2470553	2990_V7_B6_2	MZ397052
BankIt2470553	2990_V7_F7_2	MZ397053
BankIt2470553	2990_V7_B5_2	MZ397054
BankIt2470553	2990_V7_C1_4	MZ397055
BankIt2470553	2990_V7_C9_1	MZ397056
BankIt2470553	2990_V7_C10_3	MZ397057
BankIt2470553	2990_V7_D4_4	MZ397058
BankIt2470553	2990_V7_F2_3	MZ397059
BankIt2470553	2990_BL_F4_2	MZ397060
BankIt2470553	2990_BL_F1_1	MZ397061
BankIt2470553	2990_BL_G1_2	MZ397062
BankIt2470553	2990_BL_E5_2	MZ397063
BankIt2470553	1536_V15_C7_1	MZ397064
BankIt2470553	1536_V15_D12_1	MZ397065
BankIt2470553	1536_V15_A12_4	MZ397066
BankIt2470553	1536_V15_C11_2	MZ397067
BankIt2470553	1536_V15_D6_1	MZ397068
BankIt2470553	1536_V15_D11_4	MZ397069
BankIt2470553	1536_BL_E5_3	MZ397070
BankIt2470553	1536_BL_E6_1	MZ397071
BankIt2470553	1536_BL_E11_4	MZ397072
BankIt2470553	1536_BL_G9_4	MZ397073
BankIt2470553	1536_V16_B7_3	MZ397074
BankIt2470553	1536_V16_B1_3	MZ397075
BankIt2470553	1536_V16_A9_2	MZ397076
BankIt2470553	1536_V16_B6_1	MZ397077
BankIt2470553	2305_V7_E5_2	MZ397078
BankIt2470553	2305_V7_F1_4	MZ397079

Supplemental Table 12. GenBank Accession Numbers - *continued*

BankIt2470553	2305_V7_G2_2	MZ397080
BankIt2470553	2305_V7_G6_1	MZ397081
BankIt2470553	2305_V7_G5_4	MZ397082
BankIt2470553	2305_V7_G7_1	MZ397083
BankIt2470553	2305_V7_G9_2	MZ397084
BankIt2470553	2305_V7_H5_1	MZ397085
BankIt2470553	2305_V7_H11_2	MZ397086
BankIt2470553	2305_BL_H4_3	MZ397087
BankIt2470553	2305_BL_G1_1	MZ397088
BankIt2470553	2305_BL_G2_1	MZ397089
BankIt2470553	2305_BL_H1_3	MZ397090
BankIt2470553	4236_V11_D8_4	MZ397091
BankIt2470553	4236_V9_G10_3	MZ397092
BankIt2470553	4236_BL_C5_3	MZ397093
BankIt2470553	4236_BL_A9_1	MZ397094
BankIt2470553	4236_BL_C1_1	MZ397095
BankIt2470553	5257_BL_F4_4_A3_v2.0	MZ397096
BankIt2470553	5257_BL_B12_4_A4_v2.0	MZ397097
BankIt2470553	5257_BL_A3_2_A8_v2.0	MZ397098
BankIt2470553	5257_V7_E2_1	MZ397099
BankIt2470553	5257_V7_H3_1	MZ397100
BankIt2470553	5257_V7_G10_1	MZ397101
BankIt2470553	5257_V7_B92_A4_v2.0	MZ397102
BankIt2470553	5257_V7_C11_A5__v2.0	MZ397103
BankIt2470553	5257_V7_B123_A6__v2.0	MZ397104
BankIt2470553	5257_V7_D21_A7_v2.0	MZ397105
BankIt2470553	5257_V7_D61_A8_v2.0	MZ397106
BankIt2470553	5257_V7_D71_A9_v2.0	MZ397107
BankIt2470553	7190_V7_A9_8	MZ397108
BankIt2470553	7190_V7_F3_5	MZ397109
BankIt2470553	7190_V7_G1_8	MZ397110
BankIt2470553	7190_V7_A3_5	MZ397111
BankIt2470553	7190_V7_B11_4	MZ397112
BankIt2470553	7190_BL_C12_2	MZ397113
BankIt2470553	7190_BL_B1_1	MZ397114
BankIt2470553	7190_BL_A9_3	MZ397115
BankIt2470553	7190_BL_A3_2	MZ397116
BankIt2470553	7190_BL_D10_4	MZ397117
BankIt2470553	8074_V7_E2_2	MZ397118
BankIt2470553	8074_V7_E4_4	MZ397119
BankIt2470553	8074_V7_E10_4	MZ397120
BankIt2470553	8074_V7_G10_1	MZ397121
BankIt2470553	8074_V7_E11_1	MZ397122
BankIt2470553	8074_BL_F3_3	MZ397123
BankIt2470553	8074_BL_E12_4	MZ397124
BankIt2470553	8074_BL_E8_1	MZ397125
BankIt2470553	8074_BL_F2_4	MZ397126
BankIt2470553	8074_BL_D10_2	MZ397127
BankIt2470553	8074_BL_F11_2	MZ397128
BankIt2470553	8074_BL_F6_2	MZ397129

Supplemental Table 12. GenBank Accession Numbers - *continued*

BankIt2470553	2319_BL_H7_1	MZ397130
BankIt2470553	2319_BL_F1_1	MZ397131
BankIt2470553	2319_BL_H1_1	MZ397132
BankIt2470553	2319_BL_E12_2	MZ397133
BankIt2470553	2319_BL_H4_4	MZ397134
BankIt2470553	2319_BL_E10_2	MZ397135
BankIt2470553	2319_BL_E3_1	MZ397136
BankIt2470553	2319_BL_A3_2_A1_v2.0	MZ397137
BankIt2470553	2319_BL_B1_2_A3_v2.0	MZ397138
BankIt2470553	2319_BL_B2_4_A3_v2.0	MZ397139
BankIt2470553	2319_BL_B3_2_A4_v2.0	MZ397140
BankIt2470553	2319_BL_C1_4_A5_v2.0	MZ397141
BankIt2470553	2319_V7_B1_3_A1_v2.0	MZ397142
BankIt2470553	2319_V7_C1_4	MZ397143
BankIt2470553	2319_V7_H3_2_A3_v2.0	MZ397144
BankIt2470553	2319_V7_D2_4_A1_v2.0	MZ397145

Supplemental Methods

Viral Kinetic Model

We used a mathematical model to further characterize the evolution of treatment resistance during therapy with PGT-121. Viral dynamic models track the concentration of target cells (T), viral particles (V) and productively infected cells (I) by using a system of ordinary differential equations (ODEs) (Borducchi et al., 2016; Conway & Perelson, 2015; Hill et al., 2018; Perelson, 2002). As we were interested in the development of resistance to PGT-121, we included in the model viral populations that are either sensitive or resistant to the effects of PGT-121, although we do not assume that treatment resistance is absolute. This is an approached previously used to describe the emergence of antiretroviral drug resistance (Rong et al., 2007) . We denoted the total viral concentration by $V(t) = V_S(t) + V_r(t)$, where $V_S(t)$ and $V_r(t)$ are the concentrations of sensitive and resistant viral populations, respectively. Here due to limited information we do not model the individual viral strains that were identified by sequencing. We also split the infected cell population into cells infected by sensitive virus $I_S(t)$ and cells infected by resistant virus $I_r(t)$. Using this dichotomic classification simplifies the viral diversity in HIV, but it allows us to study the role of treatment resistance in driving viral rebound. The mathematical model is

$$\frac{d}{dt}T(t) = \lambda_T - \beta_s(t)T(t)V_s(t) - \beta_r(t)T(t)V_r(t) - d_T T(t)$$

$$\frac{d}{dt}I_s(t) = f(1-\mu)\beta_s(t)T(t)V_s(t) - \delta[I_s(t)]^{\omega}$$

$$\frac{d}{dt}V_s(t) = pI_s(t) - cV_s(t)$$

$$\frac{d}{dt}I_r(t) = f\mu\beta_s(t)T(t)V_s(t) + f\beta_r(t)T(t)V_r(t) - \delta[I_r(t)]^{\omega}$$

$$\frac{d}{dt}V_r(t) = pI_r(t) - c\,V_r(t),$$

$$\beta_s(t) = \begin{cases} \beta_s \text{ if } t < \tau \\ \dfrac{\beta_s}{1+\alpha_s A(t)} \text{ if } t \geq \tau, \end{cases} \quad \beta_r(t) = \begin{cases} \beta_r \text{ if } t < \tau \\ \dfrac{\beta_r}{1+\alpha_r A(t)} \text{ if } t \geq \tau. \end{cases}$$

Target cells are produced at a constant rate λ_T, are cleared linearly at rate d_T or are infected after contacting a viral particle with strain specific infection rate β_i. To account for abortive infection, a percentage f of these contacts lead to productively infected cells. Following Holte et al. (Holte et al., 2006), we model clearance of infected cells using the nonlinear term $\delta[I(t)]^{\omega}$ to account for immune mediated clearance of infected cells. Productively infected cells produce viral particles with the same sensitivity to PGT-121 as the particle which infected the cell at rate p. We assume that the dynamics $V_S(t)$ and $V_r(t)$ are similar, with identical production and clearance rates, but with infectivity rates β_S and β_r and neutralizing effects of PGT-121 given by α_S and α_r, respectively. The parameter τ in the infectivity rates β_i represents the time lag between administration of PGT-121 and the resulting decay in the plasma viral load.

We also include explicit mutation from sensitive virus into resistant virus. We assume that mutation occurs during the infection of target cells, and use the constant mutation probability

$\mu = 2.16 \times 10^{-5}$ estimated for base substitutions (Lee et al., 2009). Recombination may also occur and in this simple model is assumed to occur with the same mutation rate.

For participants 6113 and 1536, who both exhibit long term viral control with sustained viral loads below the limit of quantification, we included in the model the reactivation of latently infected cells. Reactivation of latently infected cells in the other participants, who all exhibited higher loads, was assumed to make a negligible contribution to the viral load and was neglected. Given the long half-life of latently infected cells, we assumed that the concentration of latently infected cells was constant for the period of observation, so $L(t) = L_0$. We also assumed that the latently infected cells in these two subjects, who exhibited no baseline resistance, contain proviruses that are sensitive to PGT-121, and that these latently infected cells reactivate and become productively infected cells at a constant per capita rate a. Consequently, for these two participants, the equation for the concentration of sensitive infected cells is

$$\frac{d}{dt} I_s(t) = aL_0 + f(1-\mu)\beta_s(t)T(t)V_s(t) - \delta[I_s(t)]^{\omega}$$

We fixed the infection independent death rate of target cells to be $d_T = 0.01$ /day (Mohri et al., 2001), the clearance of viral particles as c = 23 /day (Ramratnam et al., 1999). Moreover, we set the percentage of abortive infection to be 95%, so f = 0.05 (Doitsh et al., 2010). We tested the sensitivity of our results to these parameter values by refitting the model to the participant data for fixed values of $c = 100$/day and $d_T = 0.2, 0.003, 0.005$/day (De Boer et al., 2010; Ahmed et al., 2015; Luo et al., 2012; Reeves et al., 2020) and noted no qualitative difference in our results. We use the mean concentration of CD4+ cells for patients in the trial at baseline as the initial target cell concentration, so T(0) = 649.5 cells/mL. We assume that a proportion ρ of the virus is resistant to PGT-121 at baseline, so the baseline sensitive viral load satisfies $V_s(0) = (1 - \rho)\, V(0)$. Further, we assume that at baseline the viral load is at a set-point and we thus calculate the initial density of infected cells via

$$I_s(0) = \frac{cV_s(0)}{p}, I_r(0) = \frac{cV_r(0)}{p} \text{ and } I(0) = I_s(0) + I_r(0).$$

During ART, the maximum death rate of infected cells in chronically infected patients is approximately 1.5 /day (Markowitz et al., 2003) , and we therefore set $\delta = \frac{1.5}{I(0)^{\omega-1}}$. Having determined $I_s(0)$ and $I_r(0)$, we calculate the strain specific infection rates and production rate of target cells by enforcing that the system is in equilibrium before treatment, which gives

$$\beta_s = \frac{\delta I_s(0)^{\omega}}{fT(0)V_s(0)}, \beta_r = \frac{\delta I_r(0)^{\omega}}{fT(0)V_r(0)}, \text{ and } \lambda_T = T(0)(\beta_S V_s(0) + \beta_r V_r(0) + d_T).$$

We fit the remaining parameters, $\rho, p, \alpha_s, \alpha_r, \tau$ and ω and the product aL_0 for participants 6113 and 1536 by minimizing the sum of squared error between the observed viral load and the simulated viral load. Finally, we used a two-compartment pharmacokinetic model for the dynamics of PGT-121. The results for participants 6113 and 1536 should be considered

speculative given the small number of viral load measurements above the limit of detection in these two participants.

Ahmed, R., Westera, L., Drylewicz, J., Elemans, M., Zhang, Y., Kelly, E., Reljic, R., Tesselaar, K., de Boer, R. J., Macallan, D. C., Borghans, J. A. M., & Asquith, B. (2015). Reconciling Estimates of Cell Proliferation from Stable Isotope Labeling Experiments. *PLOS Computational Biology*, *11*(10), e1004355. https://doi.org/10.1371/journal.pcbi.1004355

Borducchi, E. N., Cabral, C., Stephenson, K. E., Liu, J., Abbink, P., Ng'ang'a, D., Nkolola, J. P., Brinkman, A. L., Peter, L., Lee, B. C., Jimenez, J., Jetton, D., Mondesir, J., Mojta, S., Chandrashekar, A., Molloy, K., Alter, G., Gerold, J. M., Hill, A. L., *et al*. Barouch, D. H. (2016). Ad26/MVA therapeutic vaccination with TLR7 stimulation in SIV-infected rhesus monkeys. *Nature*, *540*(7632), 284–287. https://doi.org/10.1038/nature20583

Conway, J. M., & Perelson, A. S. (2015). Post-treatment control of HIV infection. *Proc. Natl Acad. Sci.*, *112*(17), 5467–5472. https://doi.org/10.1073/pnas.1419162112

De Boer, R. J. Ribeiro, R. M. & Perelson, A. S. (2010). Current estimates for HIV-1 production imply rapid viral clearance in lymphoid tissue. *PLoS Comput. Biol.* https://doi.org/10.1371/journal.pcbi.1000906

Doitsh, G., Cavrois, M., Lassen, K. G., Zepeda, O., Yang, Z., Santiago, M. L., Hebbeler, A. M., & Greene, W. C. (2010). Abortive HIV Infection Mediates CD4 T Cell Depletion and Inflammation in Human Lymphoid Tissue. *Cell*, *143*(5), 789–801. https://doi.org/10.1016/j.cell.2010.11.001

Hill, A. L., Rosenbloom, D. I. S., Nowak, M. A., & Siliciano, R. F. (2018). Insight into treatment of HIV infection from viral dynamics models. *Immunol. Rev.*, *285*(1), 9–25. https://doi.org/10.1111/imr.12698

Holte, S. E., Melvin, A. J., Mullins, J. I., Tobin, N. H., & Frenkel, L. M. (2006). Density-Dependent Decay in HIV-1 Dynamics. *JAIDS*, *41*(3), 266–276. https://doi.org/10.1097/01.qai.0000199233.69457.e4

Lee, H. Y., Giorgi, E. E., Keele, B. F., Gaschen, B., Athreya, G. S., Salazar-Gonzalez, J. F., Pham, K. T., Goepfert, P. A., Michael Kilby, J., Saag, M. S., Delwart, E. L., Busch, M. P., Hahn, B. H., Shaw, G. M., Korber, B. T., Bhattacharya, T., & Perelson, A. S. (2009). Modeling sequence evolution in acute HIV-1 infection. *J. Theoret. Biol.*, *261*(2), 341–360. https://doi.org/10.1016/j.jtbi.2009.07.038

Luo, R., Piovoso, M. J., Martinez-Picado, J., & Zurakowski, R. (2012). HIV Model Parameter Estimates from Interruption Trial Data including Drug Efficacy and Reservoir Dynamics. *PLoS ONE*, *7*(7), e40198. https://doi.org/10.1371/journal.pone.0040198

Markowitz, M., Louie, M., Hurley, A., Sun, E., Di Mascio, M., Perelson, A. S., & Ho, D. D. (2003). A Novel Antiviral Intervention Results in More Accurate Assessment of Human Immunodeficiency Virus Type 1 Replication Dynamics and T-Cell Decay In Vivo. *J. Virol.* *77*(8), 5037–5038. https://doi.org/10.1128/JVI.77.8.5037-5038.2003

Mohri, H., Perelson, A. S., Tung, K., Ribeiro, R. M., Ramratnam, B., Markowitz, M., Kost, R., Hurley, Weinberger, L., Cesar, D., Hellerstein, M. K., & Ho, D. D. (2001). Increased

Turnover of T Lymphocytes in HIV-1 Infection and Its Reduction by Antiretroviral Therapy. *J. Exp.l Med.*, *194*(9), 1277–1288. https://doi.org/10.1084/jem.194.9.1277

Perelson, A. S. (2002). Modelling viral and immune system dynamics. *Nature Rev. Immunol.*, *2*(1), 28–36. https://doi.org/10.1038/nri700

Ramratnam, B., Bonhoeffer, S., Binley, J., Hurley, A., Zhang, L., Mittler, J. E., Markowitz, M., Moore, J. P., Perelson, A. S., & Ho, D. D. (1999). Rapid production and clearance of HIV-1 and hepatitis C virus assessed by large volume plasma apheresis. *The Lancet*, *354*(9192), 1782–1785. https://doi.org/10.1016/S0140-6736(99)02035-8

Reeves, D. B., Huang, Y., Duke, E. R., Mayer, B. T., Fabian Cardozo-Ojeda, E., Boshier, F. A., Swan, D. A., Rolland, M., Robb, M. L., Mascola, J. R., Cohen, M. S., Corey, L., Gilbert, P. B., & Schiffer, J. T. (2020). Mathematical modeling to reveal breakthrough mechanisms in the HIV Antibody Mediated Prevention (AMP) trials. *PLoS Comput. Biol. 16*(2), 1–27. https://doi.org/10.1371/journal.pcbi.1007626

Rong, L., Feng, Z., & Perelson, A. S. (2007). Emergence of HIV-1 Drug Resistance During Antiretroviral Treatment. *Bull. Math. Biol.*, *69*(6), 2027–2060. https://doi.org/10.1007/s11538-007-9203-3

CONSORT 2010 checklist of information to include when reporting a randomised trial*

Section/Topic	Item No	Checklist item	Reported on page No
**Title and abstract**			
	1a	Identification as a randomised trial in the title	N/A
	1b	Structured summary of trial design, methods, results, and conclusions (for specific guidance see CONSORT for abstracts)	3
**Introduction**			
Background and objectives	2a	Scientific background and explanation of rationale	5-6
	2b	Specific objectives or hypotheses	5-6
**Methods**			
Trial design	3a	Description of trial design (such as parallel, factorial) including allocation ratio	17
	3b	Important changes to methods after trial commencement (such as eligibility criteria), with reasons	31
Participants	4a	Eligibility criteria for participants	17-18
	4b	Settings and locations where the data were collected	17
Interventions	5	The interventions for each group with sufficient details to allow replication, including how and when they were actually administered	19
Outcomes	6a	Completely defined pre-specified primary and secondary outcome measures, including how and when they were assessed	25-26
	6b	Any changes to trial outcomes after the trial commenced, with reasons	31
Sample size	7a	How sample size was determined	26-27
	7b	When applicable, explanation of any interim analyses and stopping guidelines	N/A
Randomisation:			
Sequence generation	8a	Method used to generate the random allocation sequence	18-19
	8b	Type of randomisation; details of any restriction (such as blocking and block size)	18-19
Allocation concealment mechanism	9	Mechanism used to implement the random allocation sequence (such as sequentially numbered containers), describing any steps taken to conceal the sequence until interventions were assigned	18-19
Implementation	10	Who generated the random allocation sequence, who enrolled participants, and who assigned participants to interventions	18-19
Blinding	11a	If done, who was blinded after assignment to interventions (for example, participants, care providers, those	18-19

		assessing outcomes) and how	
	11b	If relevant, description of the similarity of interventions	18-19
Statistical methods	12a	Statistical methods used to compare groups for primary and secondary outcomes	26-28
	12b	Methods for additional analyses, such as subgroup analyses and adjusted analyses	26-28
**Results**			
Participant flow (a diagram is strongly recommended)	13a	For each group, the numbers of participants who were randomly assigned, received intended treatment, and were analysed for the primary outcome	6-7
	13b	For each group, losses and exclusions after randomisation, together with reasons	6-7
Recruitment	14a	Dates defining the periods of recruitment and follow-up	6-7
	14b	Why the trial ended or was stopped	N/A
Baseline data	15	A table showing baseline demographic and clinical characteristics for each group	Fig 2a
Numbers analysed	16	For each group, number of participants (denominator) included in each analysis and whether the analysis was by original assigned groups	6-13
Outcomes and estimation	17a	For each primary and secondary outcome, results for each group, and the estimated effect size and its precision (such as 95% confidence interval)	6-13
	17b	For binary outcomes, presentation of both absolute and relative effect sizes is recommended	6-13
Ancillary analyses	18	Results of any other analyses performed, including subgroup analyses and adjusted analyses, distinguishing pre-specified from exploratory	6-13
Harms	19	All important harms or unintended effects in each group (for specific guidance see CONSORT for harms)	N/A
**Discussion**			
Limitations	20	Trial limitations, addressing sources of potential bias, imprecision, and, if relevant, multiplicity of analyses	14-16
Generalisability	21	Generalisability (external validity, applicability) of the trial findings	14-16
Interpretation	22	Interpretation consistent with results, balancing benefits and harms, and considering other relevant evidence	14-16
**Other information**			
Registration	23	Registration number and name of trial registry	17
Protocol	24	Where the full trial protocol can be accessed, if available	26
Funding	25	Sources of funding and other support (such as supply of drugs), role of funders	31

*We strongly recommend reading this statement in conjunction with the CONSORT 2010 Explanation and Elaboration for important clarifications on all the items. If relevant, we also recommend reading CONSORT extensions for cluster randomised trials, non-inferiority and equivalence trials, non-pharmacological treatments, herbal interventions, and pragmatic trials. Additional extensions are forthcoming: for those and for up to date references relevant to this checklist, see www.consort-statement.org.

**Protocol Title:**	A Phase 1 Randomized Placebo-controlled Clinical Trial of the Safety, Pharmacokinetics and Antiviral Activity of PGT121 Monoclonal Antibody (mAb) in HIV-uninfected and HIV-infected Adults
**Protocol Number:**	IAVI T001
**Phase:**	Phase 1
**Regulatory Investigational Product Number:**	New IND submission
**Sponsor:**	International AIDS Vaccine Initiative (IAVI)<br>125 Broad Street, 9th Floor<br>New York, New York 10004<br>USA
**Sponsor Status**	Not for-Profit Organization
**Date of Protocol Version:**	05 August 2016<br>01.0<br>IRB Submission

THE CONFIDENTIAL INFORMATION IN THIS DOCUMENT IS PROVIDED TO YOU AS AN INVESTIGATOR, POTENTIAL INVESTIGATOR, OR CONSULTANT, FOR REVIEW BY YOU, YOUR STAFF, AND APPLICABLE INSTITUTIONAL REVIEW BOARDS (IRBS) AND/OR INDEPENDENT ETHICS COMMITTEES (IECS). IT IS UNDERSTOOD THAT THE INFORMATION WILL NOT BE DISCLOSED TO OTHERS, EXCEPT TO THE EXTENT NECESSARY TO OBTAIN ETHICAL AND REGULATORY APPROVAL FROM THE RESPECTIVE COMMITTEE'S AGENCIES AND INFORMED CONSENT FROM THOSE PERSONS TO WHOM THE INVESTIGATIONAL PRODUCT MAY BE ADMINISTERED.

SYNOPSIS

**TITLE**	A Phase 1 Randomized Placebo-controlled Clinical Trial of the Safety, Pharmacokinetics and Antiviral Activity of PGT121 Monoclonal Antibody (mAb) in HIV-uninfected and HIV-infected Adults
**PROTOCOL NUMBER**	IAVI T001
**PHASE**	Phase 1
**SPONSOR**	International AIDS Vaccine Initiative (IAVI)<br>125 Broad Street, 9th Floor<br>New York, New York 10004,<br>USA
**SPONSOR STATUS**	Not for Profit Organization
**STUDY PRODUCTS**	PGT121 monoclonal antibody (mAb)
**OBJECTIVES**	**Primary Objectives**<br>• To evaluate the safety and tolerability of a single intravenous (IV) infusion of PGT121 mAb at 3, 10, and 30 mg/kg in HIV-uninfected adults and HIV-infected adults<br>• To evaluate the pharmacokinetic (PK) profile of IV infusion of PGT121 mAb in HIV-uninfected and HIV-infected adults<br>• To evaluate the antiviral activity of IV infusion of PGT121 mAb in HIV-infected adults not on ART<br><br>**Secondary Objective**<br>• To determine if PGT121 induces anti-PGT121 antibodies<br>• To determine the effect of PGT121 mAb on CD4+ T cell counts in HIV-infected adults<br>• To determine if PGT121 mAb induces viral escape mutants in viremic HIV-infected adults not on ART<br><br>**Exploratory Objectives**<br>• To determine if PGT121 mAb has any impact on the host immune responses (i.e., HIV-specific cellular and humoral immune response)<br>• To determine the effect of PGT121 mAb on the size of the latent HIV reservoir in HIV-infected adults<br>• To determine PGT121 mAb levels in mucosal secretions in HIV-uninfected and HIV-infected adults<br>• To measure in vitro neutralization of HIV isolates with participant's serum post PGT121 mAb IV infusion<br>• To determine if PGT121 mAb has any impact on resistance mutations to ARVs

ENDPOINTS

Primary:

Safety and Tolerability:
We will measure the following endpoints to evaluate the safety and tolerability of PGT121 mAb in HIV-uninfected and HIV-infected adults:

1. Proportion of participants with moderate or greater reactogenicity (e.g., solicited adverse events) for 3 days following IV infusion of PGT121 mAb.
2. Proportion of participants with moderate or greater and/or PGT121 mAb-related unsolicited adverse events (AEs), including safety laboratory (biochemical, hematological) parameters, following IV infusion of PGT121 mAb for the first 56 days post administration of Investigational Product.
3. Proportion of participants with PGT121 mAb-related serious adverse events (SAEs) throughout the study period.

Pharmacokinetics:
We will measure the following endpoints to determine the pharmacokinetics following IV infusion of PGT121 mAb in HIV-uninfected and HIV-infected adults:

1. Elimination half-life (t1/2)
2. Clearance (CL/F)
3. Volume of distribution (Vz/F)
4. Area under the concentration decay curve (AUC)
5. Impact of viral load and/or ART on PGT121 disposition (elimination half-life (t1/2), clearance (CL/F), volume of distribution (Vz/F), total exposure

Antiviral activity:
We will calculate the following endpoints to determine the antiviral activity following IV infusion of PGT121 mAb in viremic HIV-infected adults not on ART:

1. Change in plasma HIV-1 RNA levels from baseline (mean of pre-entry and entry values)

Secondary:

Anti-PGT121 antibodies:
We will calculate the following endpoint to determine if anti-PGT121 antibodies are elicited following IV infusion of PGT121 mAb in HIV-uninfected and HIV-infected adults:

1. Change in serum anti-PGT121 antibody titers from baseline

CD4+ T cell count:
We will calculate the following endpoint to determine if IV infusion of PGT121 mAb has any impact on CD4+ T cell counts in HIV-infected

adults:

1. Change in CD4+ T cell count and frequency compared to baseline as measured by single platform flow cytometry

HIV genotyping/phenotyping of circulating virus for evaluation of PGT121 mAb -induced escape mutations:
We will compare plasma virus sequences before and after IV infusion of PGT121 mAb to determine if PGT121 mAb induces viral escape mutations in viremic HIV-infected adults not on ART.

1. Development of sequence variations in epitopes known to result in reduced PGT121 mAb neutralization susceptibility.

Exploratory:

Additional assessments may include, but are not limited to, the following: HIV-specific IgG/IgA binding responses by ELISA, HIV-specific cellular immune responses by ELISPOT, HIV-specific antibody function by ADCC, ADCP, and ADCVI assays, resistance mutations to current ARVs, PGT121 mAb levels in mucosal secretions, changes in total HIV-1 DNA and 2-long terminal repeat (LTR) circular HIV-1 DNA in resting or total CD4 T cells and in vitro neutralization of HIV isolates with participant's serum post IV infusion of PGT121 mAb.

STUDY DESIGN TABLE

	Group	Participants	Sub-Group	Regimen	N	Dose (mg/kg)
Part 1[1]	1[3]	HIV-uninfected participants	1A	PGT121/Placebo	4/1 (6/2 if DLT[2])	3
			1B	PGT121/Placebo	4/1 (6/2 if DLT)	10
			1C	PGT121/Placebo	4/1 (6/2 if DLT)	30
	2[3]	HIV-infected on ART, (<50 cp/ml)	2A	PGT121/Placebo	4/1 (6/2 if DLT[2])	3
			2B	PGT121/Placebo	4/1 (6/2 if DLT)	10
			2C	PGT121/Placebo	4/1 (6/2 if DLT)	30
Safety Monitoring Committee Review[4]						
Part 2	3[5]	HIV-Infected off ART (VL $2x10^3$ – $1x10^5$cp/ml)	3A[6]	PGT121	6 (max 9)	30
			3B	PGT121	8 (max 12)	10
			3C	PGT121	10 (max 15)	3
		HIV-Infected off ART (VL $1x10^2$ – $2x10^3$ cp/ml)	3D[7]	PGT121	3	30
			3E	PGT121	3	10
			3F	PGT121	3	3

DLT, dose limiting toxicity; ART, antiretroviral therapy; cp, copies; ml, milliliter
Administration of PGT 121 will be by intravenous infusion (IV)

1. Eligible participants for Groups 1 and 2 will be enrolled according to their HIV-serostatus and will occur in parallel. At each dose level in Part 1, investigational product (IP) administration will be separated by at least 4 days for each of the first 3 participants, to ensure at least 1 participant receives active product and is observed for at least 4 days before administration to additional participants.
2. A dose limiting toxicity (DLT) will be defined as 1) any Grade 3 or greater reactogenicity or adverse event judged by the study investigators as at least possibly related to investigational product or 2) any SAE considered at least possibly related to investigational product.
3. Within each group, the PSRT will review data. If no DLT occurs within 2 weeks from infusion of the 5 participants in a dose group, dose escalation to the next dose group will proceed. If 1 DLT occurs, 3 additional participants will be enrolled; randomized between PGT121 mAb and placebo at a 2:1 ratio. If no additional DLTs occur within 2 weeks of infusion in the 8 total participants (3:1 ratio of PGT121 mAb to placebo), study can proceed with enrolment of the next dose group. If 2 or more DLTs accumulate that are the same, similar, or in the same System Organ Class, dosing will be halted and the next lower dose level will be declared the maximum tolerated dose (MTD). If no DLT occurs in the final dose group, MTD will be the highest dose given (subgroups 1C and 2C 30mg/kg) after 14 days of follow-up.
4. Following IP administration of the last participant in both Groups 1 and 2, an independent Safety Monitoring Committee (SMC) will review at least the first 14 days of safety data to confirm MTD in each group, and determine whether, and at what dose, Group 3 can initiate enrollment.
5. Group 3 will start with the MTD as determined in Part 1. Group 3 will start with subgroups 3A

	and 3D if the MTD is 30mg/kg, subgroups 3B and 3E if the MTD is 10mg/kg and subgroups 3C and 3F if the MTD is 3mg/kg.<br>6. If subgroup 3A achieves a mean decline in HIV RNA of $\geq$0.9 log compared to baseline, enrolment into subgroup 3A will be stopped and the next lower dose subgroup 3B will be initiated. The same rule will apply for progression from subgroup 3B to 3C. At least 6, 8 and 10 but up to 9, 12 and 15 participants, will be enrolled in subgroups 3A, 3B, and 3C respectively, until the minimum effective dose is determined. If a mean decline $\geq$0.9 log in HIV RNA is not achieved, enrollment will be stopped at the completion of enrolment at that dose level.<br>7. As soon as subgroup 3D has enrolled 3 participants, enrolment into subgroup 3E will be initiated. The same rule will apply for progression from subgroup 3E to 3F. In the event that dose de-escalation in subgroups 3A-C is stopped due to failure to meet virologic endpoints, dose de-escalation in subgroups 3D-F will continue.
**METHODS**	See Schedule of Procedures, Appendix A
**STUDY POPULATION**	The study population will include three different groups:<br>Group 1 will include HIV-uninfected males or females aged 18-50 years old who are willing to maintain low risk behavior for HIV infection; principal exclusion criteria include confirmed HIV-infection, pregnancy or lactation, significant acute or chronic disease and clinically significant laboratory abnormalities. Group 2 will include HIV-infected males or females aged 18-50 years old on a stable antiretroviral regimen with HIV-1 RNA plasma level <50 copies/ml, CD4 cell count > 300 cells/uL and CD4 nadir > 200 cell/uL; principal exclusion criteria include history of AIDS-defining illness, significant acute or chronic medical condition other than HIV infection, and clinically significant laboratory abnormalities. Group 3 will include HIV-infected males or females aged 18-50 years old, not on antiretroviral therapy for > 6 month with detectable HIV-1 RNA plasma level between 100 and 100,000 copies/ml, CD4 cell count > 300 cells/uL and CD4 nadir > 200 cell/uL; principal exclusion criteria include history of AIDS-defining illness, significant acute or chronic medical condition other than HIV infection, and clinically significant laboratory abnormalities.
**NUMBER OF PARTICIPANTS**	63-93 participants will be included.
**DOSE ESCALATION and PAUSE RULES**	The first part of this study is a dose-escalation trial in HIV-uninfected adults and HIV-infected adults on ART with suppressed viral load, as indicated in the study design table.<br><br>If 2 or more DLTs occur that are the same, similar, or in the same System Organ Class, dosing will be halted and the next lower dose level will be declared the maximum tolerated dose (MTD) within this group. If no DLT occurs in the final dose group, MTD will be the highest dose given (subgroups 1C and 2C 30mg/kg) after 14 days of follow-up.<br><br>The Protocol Safety Review Team (PSRT) will review safety data through at least day 14 post-IP administration for all participants in the 1st dose group (subgroups 1A and 2A) prior to allowing enrolment of participants into the 2nd dose group (subgroups 1B and 2B). The PSRT will review safety data through at least day 14 post-IP administration for all participants in the 2nd dose group (subgroups 1B and 2B) prior to allowing enrolment of participants into the 3rd dose group (subgroups 1C and 2C).

	Following IP administration of the last participant in both Groups 1 and 2, an independent Safety Monitoring Committee (SMC) will review safety data through at least day 14 post-IP administration for all participants to confirm MTD, and determine whether, and at what dose, Group 3 can initiate enrollment.<br><br>The second part of this study is a dose-de-escalation trial in HIV-infected adults not on ART, as indicated in the study design table.<br><br>The study will be paused for a safety review by the investigators and the independent SMC if 1) 1 or more participants experiences a Serious Adverse Event that is judged possibly, probably or definitely related to the IP, 2) There is a participant death assessed as possibly, probably or definitely related to the IP, 3) if 2 or more participants experience grade 3 adverse events in the same System Organ Class that are considered to be at least possibly related to IP or 4) any grade 4 adverse event. See protocol section 17.3.
**FORMULATIONS, VOLUMES AND ROUTES OF INJECTIONS**	**PGT121 mAb**: PGT121 mAb is a recombinant, fully human monoclonal antibody (mAb) of the IgG1 isotype that binds to the HIV envelope. The concentration and volume of product in each vial is 50 mg/mL, 6mL in each vial. PGT121 mAb will be given intravenously in this study.
**DURATION OF STUDY PARTICIPATION**	Participants will be screened up to 42 days before IP administration and will be followed for 24 weeks. The anticipated study duration for each participant is approximately 6 months from screening through last study visit. It is anticipated that it will take approximately 4.5 months to enroll Groups 1 and 2. It is anticipated that it will take approximately 8 months to enroll the entire study. The exact duration depends on the recruitment rate and how many participants will be required per dose group.
**RANDOMIZATION and BLINDING**	This is a randomized, placebo-controlled study for groups 1 and 2, and open label for group 3 who will not receive placebo.
**EVALUATION FOR INTERCURRENT HIV INFECTION:**	Participants in Group 1 (HIV-uninfected) will be tested for HIV according to the Schedule of Procedures. Test results will be interpreted according to a pre-determined diagnostic algorithm. HIV testing at additional time points may be performed upon the request of the participant and Principal Investigator or designee as medical or social circumstances warrant.
**SAFETY MONITORING AND STATISTICAL CONSIDE-RATIONS:**	All clinical trial data collected, identified only by a study identification number, will be entered into the clinical trial database.<br><br>Safety will continually be monitored by the Investigators, the Sponsor's Medical Monitor and a Protocol Safety Review Team (PSRT); detailed pause criteria are pre-defined.<br><br>Safety data will be reviewed by an independent Safety Monitoring Committee (SMC). *Ad hoc* safety review may be specifically requested by the Sponsor, the Principal Investigators, Ethics Committees, Regulatory Authorities, or by the SMC. All clinical and routine laboratory data will be included in the safety analysis. At the end of the study, a full analysis will be prepared.

BLE OF CONTENTS

ABBREVIATIONS 11
CONTACT INFORMATION 12
1.0 SIGNATURE PAGE 13
2.0 INTRODUCTION AND BACKGROUND INFORMATION 14
2.1 Study Rationale..14
2.2 Experience with PGT121..15
3.0 STUDY OBJECTIVES...15
3.1 Primary Objectives 15
3.2 Secondary Objectives 15
3.3 Exploratory Objectives: 15
4.0 STUDY ENDPOINTS 15
4.1 Study Endpoints...15
4.1.1 Primary Endpoints...15
4.1.2 Secondary Endpoints 16
4.1.3 Exploratory Endpoints 17
5.0 STUDY DESIGN 17
5.1 Definition of Dose Limiting Toxicity and Maximum Tolerated Dose..............................17
5.2 Dose Escalation – Groups 1 and 2: Determination of Maximum Tolerated Dose..........17
5.3 Dose De-Escalation- Group 3: Determination of Minimum Effective Dose....................18
5.4 Duration of the Study 20
5.5 Study Population..20
5.6 Inclusion Criteria 20
5.7 Exclusion Criteria 22
5.8 Recruitment of Participants 26
6.0 STUDY VISITS..26
6.1 Screening Period...26
6.2 IV infusion of PGT121 mAb Visit 27
6.3 Post-IV infusion of PGT121 mAb Visits 28
6.4 Additional Follow-up Visits 28
6.5 Unscheduled Visits..28
6.6 Final Study Visit or Early Termination Visit 28
7.0 STUDY PROCEDURES 28
7.1 Informed Consent Process...28
7.2 Medical History and Physical Examination 29
7.3 HIV Testing and HIV-test Counselling (Group 1) 29
7.4 HIV Risk Reduction Counselling...30
7.5 Family Planning Counselling 30
7.6 ART Counselling (Group 3) 30
7.7 Specimens 30
7.8 Reimbursement...30
7.9 Randomization and Blinding...30
7.10 Un-blinding Procedure for Individual Participants 31
7.11 Assessment of IP related HIV sero-positivity 31

8.0 INVESTIGATIONAL PRODUCT 31
8.1 Description 31
8.2 Shipment and Storage 32
8.3 Preparation of Investigational Product (IP) 32
8.4 Administration of Investigational Product 33
8.5 Accountability and Disposal of Investigational Product 33
9.0 ASSESSMENTS 33
9.1 Safety Assessments 33
9.1.1 Local reactogenicity 33
9.1.2 Systemic reactogenicity 33
9.1.3 Vital Signs 34
9.1.4 Other Adverse Events 34
9.1.5 Concomitant Medications 34
9.1.6 Routine laboratory parameters 34
9.1.7 Specific screening tests: 35
9.2 Virologic Assessments 35
9.3 Exploratory Immunogenicity Assessments 36
9.3.1 Antibody Responses 36
9.3.2 Cellular Responses 36
9.3.3 PBMC, Serum and Plasma Storage 36
9.4 Other Assessments 36
9.4.1 HIV Antibody Testing 36
9.4.2 Pharmacokinetics 36
9.4.3 HLA Typing 37
9.4.5 Pregnancy Test 37
9.4.6 HIV Risk Assessment (Group 1) 37
9.4.7 Social Impact Assessment 37
10.0 ADVERSE EVENTS 37
10.1 Definition 37
10.2 Assessment of Severity of Adverse Events 38
10.3 Relationship to Investigational Product 38
10.4 Serious Adverse Events 39
10.5 Reporting Potential Immune-Mediated Diseases 40
10.6 Clinical Management of Adverse Events 41
10.7 Pregnancy 41
10.8 Intercurrent HIV Infection (Group 1) 41
11.0 MANAGEMENT OF HIV ISSUES DURING AND FOLLOWING STUDY 42
11.1 HIV Testing – Group 1 42
11.2 Social Discrimination as a Result of IP-related antibodies 42
11.3 HIV infection – Group 1 42
11.3.1 Counselling 42
11.3.2 Referral for Support/Care 43
12.0 WITHDRAWAL FROM STUDY 43
12.1 Deferral of IV infusion of IP 43
12.2 Withdrawal from the Study (Early Termination) 43
13.0 DATA HANDLING 44
13.1 Data Collection and Record Keeping at the Study Site 44

13.3 Data Entry at the Study Site 44
13.4 Data Analysis 44

14.0 STATISTICAL CONSIDERATIONS 45

14.1 Safety and Tolerability Analysis....................45
14.2 Pharmacokinetic Analysis 46
14.3 Virologic Analysis for Dose De-escalation in Groups 3A-C....................46
14.4 Analysis of Antiviral Activity and Dose De-escalation in Groups 3D-F 49
14.5 Secondary and Exploratory Immunologic and Virologic Analyses 49

15.0 QUALITY CONTROL AND QUALITY ASSURANCE....................50

16.0 DATA AND BIOLOGICAL MATERIAL 50

17.0 ADMINISTRATIVE STRUCTURE 50

17.1 Protocol Safety Review Team 50
17.2 Safety Monitoring Committee (SMC) 51
17.2.1 Content of Interim Safety Review 51
17.2.2 SMC Review of Group 1 and 2 data prior to starting Group 3....................51
17.3 Criteria for Pausing the Study....................51
17.4 Study Supervision 52
17.5 Study Monitoring 53
17.6 Investigator's Records....................53

18.0 INDEMNITY 53

19.0 PUBLICATION....................53

20.0 ETHICAL CONSIDERATIONS....................54

APPENDIX A: SCHEDULE OF PROCEDURES....................55

APPENDIX B: LOW RISK CRITERIA....................58

APPENDIX C: ADVERSE EVENT SEVERITY ASSESSMENT TABLE....................60

Major Clinical Conditions....................62
Cardiovascular 62
Dermatologic 64
Endocrine and Metabolic 64
Gastro-intestinal 66
Musculoskeletal....................68
Neurologic....................69
Pregnancy, Puerperium, and Perinatal....................71
Psychiatric....................71
Respiratory....................71
Sensory 72
Systemic....................74
Urinary77
Site Reactions to Injections and Infusions....................77
Laboratory Values....................79
Chemistries 79
Hematology 82
Urinalysis....................84

ABBREVIATIONS

Abbreviation	Term
**AE**	Adverse Event
**AIDS**	Acquired Immunodeficiency Syndrome
**ALT**	Alanine-Aminotransferase
**ART**	Antiretroviral Therapy
**AST**	Aspartate-Aminotransferase
**CFC**	Cytokine Flow Cytometry
**CMI**	Cell Mediated Immunity
**CRF**	Case Report Form
**CTL**	Cytotoxic T Lymphocyte
**DCC**	Data Coordinating Center
**DLT**	Dose Limiting Toxicity
**DNA**	Deoxyribonucleic Acid
**ELISA**	Enzyme Linked Immunosorbent Assay
**GCP**	Good Clinical Practice
**GFR**	Glomerular Filtration Rate
**HIV**	Human Immunodeficiency Virus
**HLA**	Human Leukocyte Antigen
**HSV**	Herpes Simplex Virus
**IAVI**	International AIDS Vaccine Initiative
**IDES**	Internet Data Entry System
**ICH**	International Council for Harmonization of Technical Requirements for Pharmaceuticals for Human Use
**IP**	Investigational Product
**IND**	Investigational New Drug Application
**IV**	Intravenous
**Kg**	Kilogram
**mAb**	Monoclonal Antibody
**mg**	Milligram
**MED**	Minimum Effective Dose
**MTD**	Maximum Tolerated Dose
**PCR**	Polymerase Chain Reaction
**PBMC**	Peripheral Blood Mononuclear Cells
**PK**	Pharmacokinetic
**RPR**	Rapid Plasma Reagin
**SAE**	Serious Adverse Event
**SIV**	Simian Immunodeficiency Virus
**SOP**	Standard Operating Procedure
**SOM**	Study Operations Manual
**SMC**	Safety Monitoring Committee
**STD**	Sexually Transmitted Disease
**TPHA**	Treponema Pallidum Hemagglutination

CONTACT INFORMATION

Detailed contact information provided in the Study Operation Manual (SOM)

**Sponsor Contact:**	
Frances Priddy MD MPH<br>Executive Director and Chief Medical Officer<br>International AIDS Vaccine Initiative<br>125 Broad Street, 9th Floor<br>New York, New York 10004	Phone: +1-212-328-7461<br>Mobile: +1-646-287-8943<br>Fax: +1-608-203-5501<br>E-mail: fpriddy@iavi.org
**Clinical Research Center Contacts:**	
Kathryn Stephenson MD MPH<br>Center for Virology and Vaccine Research<br>Clinical Trials Unit<br>Beth Israel Deaconess Medical Center<br>E / CLS – 1036<br>330 Brookline Avenue<br>Boston, Massachusetts 02215	Phone: +1-617-735-4556<br>Mobile: +1-917-836-9150<br>Fax: +1-617-735-4566<br>E-mail: kstephen@bidmc.harvard.edu

.

1.0 SIGNATURE PAGE

The signatures below constitute the approval of this protocol and the appendices and provide the necessary assurances that this study will be conducted in compliance with the protocol, Good Clinical Practices (GCP) and the applicable regulatory requirement(s).

Sponsor:

Signed: See electronic signature manifest Date: August 5, 2016

Frances Priddy MD MPH
Executive Director and Chief Medical Officer, Medical Affairs, IAVI

Principal Investigator:

Signed: Date:

Name (please print):

Name of institution (please print):

2.0 INTRODUCTION AND BACKGROUND INFORMATION

More than 78 million people have been infected with HIV and 39 million people have died since the beginning of the AIDS epidemic[1]. In 2014, there were 1.2 million deaths attributable to HIV infection and 2 million newly infected with HIV[2]. One reason that such high rates of AIDS-related deaths continue to occur globally – despite the advent of drugs that are highly effective at suppressing HIV replication – is that only two in five people living with HIV have access to antiretroviral therapy[1]. The other reason for continued AIDS-related mortality is that ART does not cure HIV infection and must be maintained for a lifetime. Even in the United States (US), only 30% of the 1.2 million people living with HIV have suppressed HIV to undetectable levels, despite the fact that most HIV-infected people in the US have access to ART[3] It is clear that antiretroviral therapy is necessary but not sufficient to end the AIDS epidemic, both in the US and globally, and that novel efforts to prevent and treat HIV are critically needed.

2.1 Study Rationale

This is a Phase 1 study to evaluate the safety, tolerability, pharmacokinetics and anti-viral efficacy of the PGT121 monoclonal antibody for HIV prevention and therapy. PGT121 mAb is a recombinant human IgG1 monoclonal antibody that targets a V3 glycan-dependent epitope region of the HIV envelope protein[4,5]. PGT121 mAb was chosen for this study because it is potent, neutralizes a wide array of HIV viruses, and can prevent and treat simian-human immunodeficiency virus (SHIV) in rhesus monkeys.

The recent discovery of multiple potent and broadly neutralizing antibodies (bNAbs) against HIV has led to the re-emergence of the concept that antibodies may be useful for both prevention and therapy. HIV-specific antibodies that target the HIV envelope (Env) can prevent SHIV infection in rhesus monkeys and have shown to reduce HIV RNA levels in humans temporarily[6-10]. Until recently, these antibodies were few in number, targeted a narrow spectrum of HIV strains and Env epitopes, and were not potent enough for practical use. In the last five years, the field has changed dramatically: new developments in high throughput single-cell PCR-amplification and novel soluble Env baits have led to the isolation of new monoclonal antibodies with extraordinary potency and breadth[11]. These bNAbs may be effective for prevention of HIV infection when administered passively[12,13].

PGT121 mAb was selected for development because of the following critical attributes:

- PGT121 mAb is 10 to 100-fold more potent than the previous best-in-class CD4bs antibodies VRC01, VRC07, and 3BNC117 [11,14,15].
- PGT121 mAb affords superior protective efficacy against SHIV acquisition in monkeys compared to VRC01, 3BNC117, and 10-1074 [16] (and unpublished data).
- PGT121 mAb has superior therapeutic efficacy in SHIV-infected monkeys compared to VRC01, 3BNC117, and 10-1074 [7] (and unpublished data).
- PGT121 mAb may have a higher bar to escape in vivo as compared with other V3 glycan and CD4bs antibodies as a result of making multiple glycan contacts [14].
- PGT121 mAb combined with PGDM1400 (a novel bNab targeting the envelope trimer apex) neutralizes 98-99% of global HIV-1 viruses tested and has unparalleled potency with a median IC50 of 0.007 µg/ml[14].

The potency and breadth of PGT121 mAb, both alone and in combination with other bNAbs, raise the possibility that combinations may be effective for HIV prophylaxis at

low doses and against global viruses. An antibody that is effective at low doses may eventually be given subcutaneously, which would reduce the cost. It is these features that make PGT121 mAb particularly well-suited for preventing and/or treating HIV in the developing world, where it is critical that a public health intervention be low cost, easy to deliver, and effective in diverse settings.

2.2 Experience with PGT121

There is no previous clinical experience with PGT121 mAb. Several other HIV monoclonal antibodies are currently in clinical development as passive HIV immunoprophylaxis, or as potential therapeutics. Data from phase 1 studies shows acceptable preliminary safety and tolerability profiles for these products, but varying levels of anti-viral effects [6,17]. A comprehensive summary of phase 1 studies of HIV monoclonal antibodies can be found in the Investigator's Brochure.

3.0 STUDY OBJECTIVES

3.1 Primary Objectives

- To evaluate the safety and tolerability of a single intravenous (IV) infusion of PGT121 mAb at 3, 10, and 30 mg/kg in HIV-uninfected adults and HIV-infected adults.
- To evaluate the pharmacokinetic (PK) profile of IV infusion of PGT121 mAb in HIV-uninfected and HIV-infected adults.
- To evaluate the antiviral activity of IV infusion of PGT121 mAb in HIV-infected adults not on ART.

3.2 Secondary Objectives

- To determine if PGT121 mAb induces anti-PGT121 antibodies.
- To determine the effect of PGT121 mAb on CD4 T-cell counts in HIV-infected adults.
- To determine if PGT121 mAb induces viral escape mutants in viremic HIV-infected adults not on ART.

3.3 Exploratory Objectives:

- To determine if PGT121 mAb has any impact on the host immune responses (i.e., HIV-specific cellular and humoral immune response).
- To determine the effect of PGT121 mAb on the size of the latent HIV reservoir in HIV-infected adults.
- To determine PGT121 mAb levels in mucosal secretions in HIV-uninfected and HIV-infected adults.
- To measure in vitro neutralization of HIV isolates with participant's serum post PGT121 mAb IV infusion.
- To determine if PGT121 mAb has any impact on resistance mutations to ARVs.

4.0 STUDY ENDPOINTS

4.1 Study Endpoints

4.1.1 Primary Endpoints

Safety and Tolerability:

We will measure the following endpoints to evaluate the safety and tolerability of PGT121 mAb in HIV-uninfected and HIV-infected adults:

1. Proportion of participants with moderate or greater reactogenicity (e.g., solicited adverse events) for 3 days following IV infusion of PGT121 mAb.
2. Proportion of participants with moderate or greater and/or PGT121 mAb-related unsolicited adverse events (AEs), including safety laboratory (biochemical, hematological) parameters, following IV infusion of PGT121 mAb for the first 56 days post administration of Investigational Product.
3. Proportion of participants with PGT121 mAb-related serious adverse events (SAEs) throughout the study period.

Pharmacokinetics:

We will measure the following endpoints to determine the pharmacokinetics following IV infusion of PGT121 mAb in HIV-uninfected and HIV-infected adults:

1. Elimination half-life (t1/2)
2. Clearance (CL/F)
3. Volume of distribution (Vz/F)
4. Area under the concentration decay curve (AUC)
5. Impact of viral load and/or ART on PGT121 disposition (elimination half-life (t1/2), clearance (CL/F), volume of distribution (Vz/F), total exposure

Antiviral activity:

We will calculate the following endpoints to determine the antiviral activity following IV infusion of PGT121 mAb in viremic HIV-infected adults not on ART.

1. Change in plasma HIV-1 RNA levels from baseline (mean of pre-entry and entry values)

4.1.2 Secondary Endpoints

Anti-PGT121 antibodies:

We will calculate the following endpoint to determine if anti-PGT121 antibodies are elicited following IV infusion of PGT121 mAb in HIV-uninfected and HIV-infected adults:

1. Change in serum anti-PGT121 antibody titers from baseline

CD4+ T cell count:

We will calculate the following endpoint to determine if IV infusion of PGT121 mAb has any impact on CD4+ T cell counts in HIV-infected adults:

1. Change in CD4+ T cell count and frequency compared to baseline as measured by single platform flow cytometry

HIV genotyping/phenotyping of circulating virus for evaluation of PGT121-induced escape mutations:

We will compare plasma virus sequences before and after IV infusion of PGT121 mAb to determine if PGT121 mAb induces viral escape mutations in viremic HIV-infected adults not on ART.

1. Development of sequence variations in epitopes known to result in reduced PGT121 neutralization susceptibility

4.1.3 Exploratory Endpoints

Additional assessments may include but are not limited to the following: HIV-specific IgG/IgA binding responses by ELISA, HIV-specific cellular immune responses by ELISPOT, HIV-specific antibody function by ADCC, ADCP, and ADCVI assays, resistance mutations to current ARVs, PGT121 mAb levels in mucosal secretions, changes in total HIV-1 DNA and 2-long terminal repeat (LTR) circular HIV-1 DNA in resting or total CD4 T cells and in vitro neutralization of HIV isolates with participant's serum post IV infusion of PGT121 mAb.

5.0 STUDY DESIGN

The study is a randomized, placebo-controlled study for groups 1 and 2, and open label for group 3 who will not receive placebo.

5.1 Definition of Dose Limiting Toxicity and Maximum Tolerated Dose

A dose limiting toxicity (DLT) will be defined as 1) any Grade 3 or greater reactogenicity or adverse event judged by the study investigators as at least possibly related to investigational product or 2) any SAE considered at least possibly related investigational product.
Maximum Tolerated Dose (MTD) will be declared when 2 or more DLTs occur that are the same, similar, or in the same System Organ Class or if no DLT occurs in the final dose subgroup, MTD will be the highest dose given (groups 1C and 2C 30mg/kg) after 14 days of follow-up.

5.2 Dose Escalation – Groups 1 and 2: Determination of Maximum Tolerated Dose

In Groups 1 and 2, (Part 1), the administrations of PGT121 mAb escalate by dose as shown below in Table 5.3.1, Study Design (5 participants per dose subgroup, 4:1 ratio of IP to placebo for each dose subgroup).

Sentinel Recipients

Within each dose group (subgroups 1A and 2A, subgroups 1B and 2B, subgroups 1C and 2C), the first 3 participant infusions will be separated by at least 4 days, to allow for observation of Investigational product (IP)-related adverse events. Dose subgroups will be enrolled in parallel, meaning that the 1st participant may be from subgroup 1A, the 2nd from subgroup 2A, the 3rd from subgroup 2A, all with 4 days in between dosing.

Because there is 1 placebo in each dose subgroup and the subgroups are dosed in parallel, the first 3 recipients will be treated as sentinel recipients (at least 1 will receive the IP). If no reactogenicity and adverse events that are considered to be related to IP (possibly, probably or definitely related) and are graded as severe or worse (Grade 3 or 4 on the DAIDS Toxicity Table) occur within 4 days after injection, the second participant may be injected. If no events meeting the criteria described above occur within 4 days after the 3rd participant is infused, then the remainder of participants in that dose group will be infused. If events meeting the criteria described above do occur for the first 3 participants in a dose group, they will be reviewed by the Safety Monitoring Committee (SMC) to determine whether further infusions may proceed.

Dose Escalation and Determination of Maximum Tolerated Dose

Safety data through day 14 post-IP administration visit for all participants in the first dose group (1A and 2A) will be reviewed by the Protocol Safety Review Team (PSRT) prior to allowing enrollment of participants into the second dose group (1B and 2B). The review process will be repeated between the second and third (1C and 2C) dose groups. Following administration of IP for the last participant in both Groups 1 and 2, an independent Safety Monitoring Committee (SMC) will review safety data to confirm Maximum Tolerated Dose (MTD) and determine whether, and at what dose, Group 3 can initiate enrollment.

Within each group, if no DLT occurs within 2 weeks from infusion of the first 5 participants in a dose group, dose escalation to the next dose group will proceed. If 1 DLT occurs, 3 additional participants will be enrolled; randomized between PGT121 mAb and placebo at a 2:1 ratio. If no additional DLTs occur within 2 weeks of infusion in the 8 mAb total participants (3:1 ratio of PGT121 mAb to placebo), the study can proceed with enrollment of the next dose group. If 2 or more DLTs accumulate in a subgroup that are the same, similar, or in the same organ class, dosing will be halted and the next lower dose level will be declared the maximum tolerated dose (MTD). If no DLT occurs in the final dose group, MTD will be the highest dose given (subgroups 1C and 2C 30mg/kg) after 14 days of follow-up.

5.3 Dose De-Escalation- Group 3: Determination of Minimum Effective Dose

Upon approval by the SMC (see section 17.2.2), group 3 (Part 2), PGT121 mAb administrations will de-escalate by dose as shown below in Table 5.3.1.

Group 3 will start with the MTD (i.e. subgroups 3A and 3D if the MTD is 30 mg/kg, subgroups 3B and 3C if the MTD is 10 mg/kg, or subgroups 3C and 3F if the MTD is 3 mg/kg) as determined by the SMC from data in Part 1.

If subgroup 3A (n = 6) achieves a mean decline in HIV RNA of $\geq$0.9 log compared to baseline, enrollment into subgroup 3A will be stopped, and the next lower dose subgroup 3B will be initiated. The same rule will apply for progression from subgroup 3B to 3C. At least 6, 8 and 10 but up to 9, 12 and 15 participants will be enrolled in subgroups 3A, 3B and 3C respectively, until the minimum effective dose is determined. In each subgroup, if a mean decline >0.9 log in HIV RNA is not achieved, enrollment will be stopped at the completion of enrollment at that dose level.

Three participants will be enrolled in each group 3D, 3E and 3F. As soon as subgroup 3D has enrolled 3 participants, enrollment into subgroup 3E will be initiated. The same rule will apply for progression from subgroup 3E to 3F. In the event that dose de-escalation in subgroups 3A-C is stopped due to failure to meet virologic endpoints, dose de-escalation in subgroups 3D-F will continue.

Table 5.3.1 Study Design Table

	Group	Participants	Sub-Group	Regimen	N	Dose (mg/kg)
Part 1	1	HIV-uninfected participants	1A	PGT121/Placebo	4/1 (6/2 if DLT)	3
			1B	PGT121/Placebo	4/1 (6/2 if DLT)	10
			1C	PGT121/Placebo	4/1 (6/2 if DLT)	30
	2	HIV-infected on ART, (<50 cp/ml)	2A	PGT121/Placebo	4/1 (6/2 if DLT)	3
			2B	PGT121/Placebo	4/1 (6/2 if DLT)	10
			2C	PGT121/Placebo	4/1 (6/2 if DLT)	30
Safety Monitoring Committee Review						
Part 2	3	HIV-infected off ART (VL 2x10^3 – 1x10^5cp/ml)	3A	PGT121	6 (max 9)	30
			3B	PGT121	8 (max 12)	10
			3C	PGT121	10 (max 15)	3
		HIV-infected off ART (VL 1x10^2 – 2x10^3 cp/ml)	3D	PGT121	3	30
			3E	PGT121	3	10
			3F	PGT121	3	3

Table 5.3.2 Decision Tree, Groups 3A, 3B, 3C

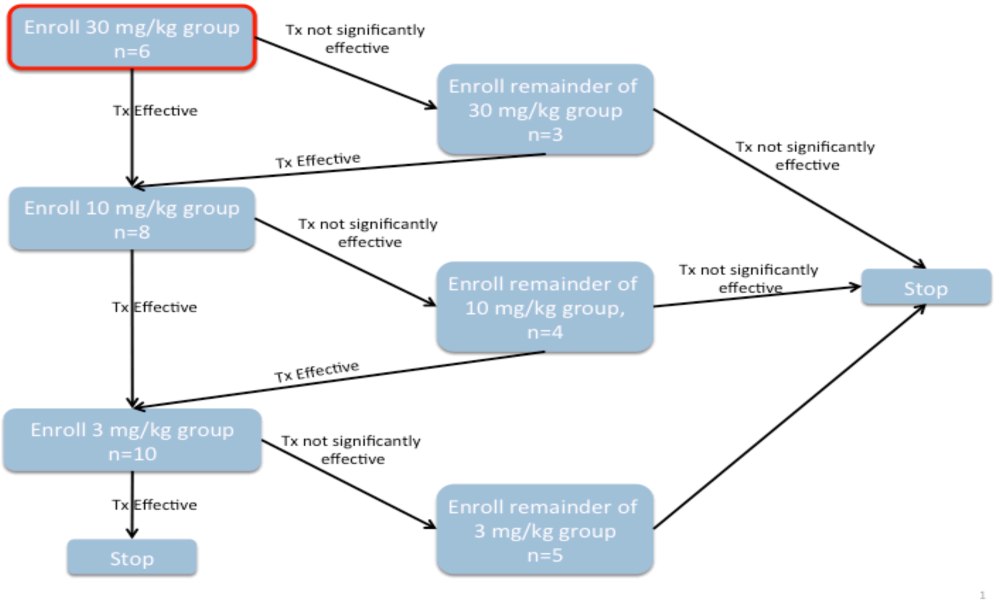

"not significantly effective" = does not achieve mean decrease of ≥0.9 log HIV RNA

5.4 Duration of the Study

Participants will be screened up to 42 days before IP administration of PGT121 mAb and will be followed for 24 weeks.

It will take approximately 8 months to enroll the entire study. The exact duration depends on the recruitment rate and how many participants will be required per dose group as specified in sections 5.2 and 5.3.

5.5 Study Population

The study population consists of HIV-uninfected male or female adults (Group 1), HIV-infected male or female adults on ART (Group 2), and HIV-infected males and female adults not on ART (group 3) who meet the detailed inclusion and exclusion criteria listed below, and who in the opinion of the investigator or designee, understand the study and provide written informed consent.

Approximately 63-93 participants (81 investigational product recipients, 12 placebo recipients) who meet all eligibility criteria will be included in the study. An over-enrollment of up to 5% (up to 5 participants total) will be permitted in the study to facilitate rapid enrollment.

5.6 Inclusion Criteria

Inclusion criteria for all participants:

1. At least 18 years of age on the day of screening and has not reached his or her 51st birthday on the day of signing the Informed Consent Document.

2. Willing to comply with the requirements of the protocol and available for follow-up for the planned duration of the study;

3. In the opinion of the Principal Investigator or designee and based on Assessment of Informed Consent Understanding results, has understood the information provided and potential impact and/or risks linked to IV infusion and participation in the trial; written informed consent will be obtained from the participant before any study-related procedures are performed;

4. All heterosexually active female participants must commit to use an effective method of contraception for 3 months following IP administration, including:
    a. Condoms (male or female) with or without spermicide
    b. Diaphragm or cervical cap with spermicide
    c. Intrauterine device, or contraceptive implant
    d. Hormonal contraception
    e. Successful vasectomy in the male partner (considered successful if a woman reports that a male partner has [1] documentation of azoospermia by microscopy (< 1 year ago), or [2] a vasectomy more than 2 years ago with no resultant pregnancy despite sexual activity post-vasectomy)
    f. Not be of reproductive potential, such as having undergone hysterectomy, bilateral oophorectomy, or tubal ligation, postmenopausal (>45 years of age with amenorrhea for at least 2 years, or any age with amenorrhea for at least 6 months and a serum follicle stimulating hormone [FSH] level >40 IU/L); surgically sterile: no additional contraception required.

    Women, who are not heterosexually active at screening, must agree to utilize an effective method of contraception if they become hetero-sexually active, as outlined above.

5. All sexually active males, regardless of reproductive potential, must be willing to consistently use an effective method of contraception (such as consistent male condoms with male and/or female partners from the day of IP administration until at least 3 months following IP administration to avoid exposure of partners to IP in ejaculate, and to prevent conception with female partners.

6. All female participants must be willing to undergo urine pregnancy tests at time points indicated in the Schedule of Procedures and must test negative prior to IP administration;

7. A woman must agree not to donate eggs (ova, oocytes) for the purpose of assisted reproduction until 3 months after receiving IP administration. A man must agree not to donate sperm until 3 months after IP administration;

8. Willing to forgo donations of blood and/or any other tissues, including bone marrow, during the study and, for those HIV-uninfected participants who test HIV-positive due to IP administration, until the anti-HIV antibody titers become undetectable.

Specific inclusion criteria for HIV-uninfected participants (Group 1):

9. Willing to undergo HIV testing, risk reduction counselling and receive HIV test results;

10. Low risk for HIV infection (see section (9.4.6) and willing to maintain low-risk behaviour for the duration of the trial (Appendix B);

11. Healthy male or female, as assessed by a medical history, physical exam, and laboratory tests;

Specific inclusion criteria for HIV-infected participants (Groups 2 and 3):

12. Confirmed HIV-1 infection (HIV Ab+ or HIV RNA+) by documentation in the medical records or in-clinic HIV testing;

13. CD4 ≥ 300 cells/µl;

14. No history of AIDS-defining illness or CD4 < 200 cells/µl.

Group 2:

15. Currently on ART, and documentation of continuous combination ART (cART) treatment with suppression of plasma HIV-1 viral load < 50 copies / ml for greater than 6 months, measured on at least 2 independent occasions, and with a viral load < 50 copies / ml at time of screening (within 42 days prior to IP administration). cART is defined as a regimen including > 2 compounds, e.g. 2x nucleoside reverse transcriptase inhibitors plus either non-nucleoside reverse transcriptase inhibitor or protease inhibitor or integrase inhibitor.

Group 3:

16. Not receiving cART, and (after appropriate counselling) willing to defer cART treatment for at least 56 days after administration of IP;

17. HIV-1 viral load either between 2000-100,000 copies / ml (Group 3A, 3B, 3C) or between 100-2000 copies / ml (Group 3D, 3E and 3F) at 2 independent occasions within 12 months prior to study enrollment, with confirmation during the screening period (3 viral loads on independent occasions).

5.7 Exclusion Criteria

Exclusion criteria for all participants:

1. Any clinically significant acute or chronic medical condition, other than HIV infection, that is considered progressive or in the opinion of the investigator makes the participant unsuitable for participation in the study;

2. If female, pregnant, lactating or planning a pregnancy during the period of screening through completion of the study;

3. In the past 6 months a history of alcohol or substance use, including marijuana, judged by the Investigator to potentially interfere with participant study compliance;

4. Bleeding disorder that was diagnosed by a physician (e.g., factor deficiency, coagulopathy or platelet disorder that requires special precautions). Note: A participant who states that he or she has easy bruising or bleeding, but does not have a formal diagnosis and has intramuscular injections and blood draws without any adverse experience, is eligible;

5. History of a splenectomy;

6. Receipt of live attenuated vaccine within the previous 60 days or planned receipt within 60 days after administration of IP; or receipt of other vaccine within the previous 14 days or planned receipt within 14 days after infusion with IP (exception is live attenuated influenza vaccine within 14 days);

7. Receipt of blood transfusion or blood-derived products within the previous 3 months;

8. Participation in another clinical trial of an investigational product currently, within the previous 3 months or expected participation during this study;

9. Prior receipt of an investigational HIV vaccine candidate, monoclonal antibody or polyclonal immunoglobulin (note: receipt of placebo in a previous HIV vaccine or monoclonal antibody trial will not exclude a participant from participation if documentation is available and the Medical Monitor gives approval);

10. History of severe local or systemic reactogenicity to injections or IV infusion (e.g., anaphylaxis, respiratory difficulties, angioedema);

11. HIV-specific antibodies that significantly cross-react with PGT121 mAb pharmacokinetic assays;

12. Psychiatric condition that compromises safety of the participant and precludes compliance with the protocol. Specifically excluded are persons with psychoses within the past 3 years, ongoing risk for suicide, or history of suicide attempt or gesture within the past 3 years;

13. If, in the opinion of the Principal Investigator, it is not in the best interest of the participant to participate in the trial;

14. Seizure disorder: a participant who has had a seizure in the last 3 years is excluded. (Not excluded: a participant with a history of seizures who has neither required medications nor had a seizure for 3 years.)

15. Body mass index ≥ 30 or ≤ 18.0.

16. Infectious disease: chronic hepatitis B infection (HbsAg), current hepatitis C infection (HCV Ab positive and HCV RNA positive) or interferon-alfa treatment for chronic hepatitis C infection in the past year, or active syphilis (RPR confirmed by TPHA).

17. A history of malignancy within the past 5 years (prior to screening) or ongoing malignancy;

18. Active, serious infections (other than HIV-1 infection) requiring parenteral antibiotic, antiviral or antifungal therapy within 30 days prior to enrollment;

Specific exclusion criteria for HIV-uninfected participants (Group 1):

19. Confirmed HIV-1 or HIV-2 infection;

20. Any clinically relevant abnormality on history or examination including history of immunodeficiency or autoimmune disease; use of systemic corticosteroids, immunosuppressive, anticancer, or other medications considered significant by the investigator within the previous 6 months.

    The following exceptions are permitted and will not exclude study participation: use of corticosteroid nasal spray for rhinitis, topical corticosteroids for an acute uncomplicated dermatitis; or a short course (duration of 10 days or less, or a single injection) of corticosteroid for a non-chronic condition (based on investigator clinical judgment) at least 6 weeks prior to enrollment in this study.

Specific exclusion criteria for HIV-uninfected participants (Group 1) and HIV-infected participants who are on ART (Group 2):

21. Any of the following abnormal laboratory parameters listed below:

    Hematology

    – Hemoglobin <10.5 g/dL in females; hemoglobin <11.0 g/dL in males
    – Absolute Neutrophil Count (ANC): ≤1000/mm^3
    – Absolute Lymphocyte Count (ALC): < 650/mm^3
    – Platelets: < 125,000 mm^3 or ≥ 550,000/mm^3

    Coagulation

    - aPTT: >1.25x ULN
    - INR: ≥1.1 x ULN

    Chemistry

    - Sodium ≤135 mEq/L or ≥ 146 mEq/L
    - Potassium ≤3.4 mEq/L or ≥ 5.6 mEq/L
    - Creatinine ≥1.1 x ULN
    - AST ≥1.25 x ULN
    - ALT ≥1.25 x ULN
    - Total bilirubin ≥1.25 x ULN
    - Alkaline phosphatase ≥1.25 x ULN
    - Albumin ≤ 3.0 g/dL or ≤ 30 g/L
    - Creatine kinase ≥3.0 x ULN
    - C-reactive protein >10 mg/L
    - C3 complement ≤ 0.9g/L
    - C4 complement ≤ 0.1g/L

Urinalysis

Clinically significant abnormal dipstick confirmed by microscopy:
– Protein = 1+ or more
– Blood = 1+ or more (not due to menses)

Specific exclusion criteria for HIV-infected participants who are on ART (Group 2) and for HIV-infected participants who are not on ART (Group 3):

22. Any clinically relevant abnormality on history or examination including history of immunodeficiency or autoimmune disease, other than HIV; use of systemic corticosteroids, immunosuppressive, anticancer, or other medications considered significant by the investigator within the previous 6 months.

    The following exceptions are permitted and will not exclude study participation: use of corticosteroid nasal spray for rhinitis, topical corticosteroids for an acute uncomplicated dermatitis; or a short course (duration of 10 days or less, or a single injection) of corticosteroid for a non-chronic condition (based on investigator clinical judgment) at least 6 weeks prior to enrollment in this study.

Specific exclusion criteria for HIV-infected participants who are not on ART (Group 3)

23. Resistance of autologous HIV to PGT121 neutralization in vitro;

24. Any of the following abnormal laboratory parameters listed below:

Hematology

– Hemoglobin <8.5 g/dL
– Absolute Neutrophil Count (ANC): <1000/mm^3
– Platelets: < 50,000 mm^3 or ≥ 550,000/mm^3

Coagulation

- aPTT: >1.25x ULN
- INR: ≥1.1 x ULN

Chemistry

- Estimated Glomerular filtration rate (GFR) ≥ <u><</u> 50 mL/min according to the Cockcroft-Gault formula for creatinine clearance:
  - Male: $\frac{(140 - \text{age in years}) \times (\text{wt in kg})}{72 \times (\text{serum creatinine in mg/dL})} = CLcr\ (mL/min)$
  - Female: $\frac{(140 - \text{age in years}) \times (\text{wt in kg}) \times 0.85}{72 \times (\text{serum creatinine in mg/dL})} = CLcr\ (mL/min)$

- AST >5 x ULN
- ALT >5 x ULN
- Total bilirubin >2.5 x ULN
- Alkaline phosphatase >5 x ULN

Urinalysis

- Any RBC, protein or leukocytes greater than 1+, confirmed by microscopy and consistent with clinically significant disease.

5.8 Recruitment of Participants

Adult male and female participants may be recruited through in-clinic referrals, information presented to community organizations, hospitals, colleges, other institutions and/or advertisements to the general public or from existing cohorts. The information distributed will contain contact details of the trial site.

6.0 STUDY VISITS

6.1 Screening Period

During Screening, study staff will perform the following procedures:

- Provide and/or review the Informed Consent Document and answer any questions about the study prior to obtaining written informed consent.
- Complete Assessment of Informed Consent Understanding (AOU). Please refer to the Study Operations Manual (SOM)

If the participant agrees to participate, passes the AOU and provides written informed consent, study staff will:

- Conduct HIV test counselling, HIV testing, and HIV risk reduction counselling, as applicable
- Conduct family planning counselling, refer for pregnancy prevention counselling if necessary
- Conduct ART counselling, if applicable
- Perform a comprehensive medical history
- Collect concomitant medication information
- Perform a general physical examination (Refer to Section 7.2)
- Collect specimens for all tests as indicated in the Schedule of Procedures in Appendix A (for details see Analytical Plan (AP).

When available, the screening laboratory tests will be reviewed by the trial physician. Screening laboratory test(s) may be repeated once at the discretion of the principal investigator or designee to investigate any isolated abnormalities.

If the screening visit occurs more than 42 days prior to the date of administration of IP, all screening procedures must be repeated except the comprehensive medical history may be replaced by an interim medical history and the Participant Information Sheet of the Informed Consent Document should be reviewed.

If a participant has signed the Consent Form but does not meet the eligibility criteria, the records must be kept at the site.

6.2 IV infusion of PGT121 mAb Visit

Prior to the administration of IP, study staff will:

- Answer any questions the participant may have about the study
- Review the Informed Consent Document with the participant
- Review screening safety laboratory data
- Administer HIV risk assessment (Group 1)
- Conduct HIV test counselling, and HIV risk reduction counselling
- Conduct family planning counselling as per site specific procedures and ensure compliance with respective pregnancy prevention method, and discuss male condom use with all male participants
- Review interim medical history
- Collect concomitant medication information
- Weigh participant and record vital signs
- Perform a symptom-directed physical examination (Refer to Section 7.2)
- Assess at baseline local and systemic signs and symptoms (this includes an examination of IV infusion site)
- Collect specimens for all tests as indicated in the Schedule of Procedures see Appendix A (for details see AP).
- Obtain pregnancy test results prior to administration of IP.

Assign an allocation number to the participant according to the instructions specified in the Study Operations Manual.

At the time of administration of IP and after IV infusion of IP, study staff will:

- Administer the IP as specified in Section 8.4, Administration of Investigational Product and according to the instructions specified in the SOM.
- Observe participant closely during the infusion of IP and for at least 30 minutes after IV infusion of IP has ended for any acute reactogenicity. At the end of the observation period study staff will:
    - Record vital signs (pulse, respiratory rate, blood pressure and temperature)
    - Assess any local and systemic reactogenicity
    - Assess any other adverse events
- Every hour after IV infusion of IP, starting hour 1 through 12, the study staff will:
    - Record vital signs (pulse, respiratory rate, blood pressure and temperature)
    - Assess any local and systemic reactogenicity
    - Assess any other adverse events
    - Collect PK samples according to the Schedule of Procedures

If a volunteer has an abnormal laboratory value that is known, at the time of infusion, follow the specified guidelines (Section 12.0).

6.3 Post-IV infusion of PGT121 mAb Visits

The participant will be asked to return to the clinic for post-IP administration visits as indicated in the Schedule of Procedures (see Appendix A) for an assessment by clinic staff. The participant will be asked to maintain a Memory Aid for local and systemic reactogenicity from the day of IP administration for the next 3 days (for a total of 4 days including day of IP administration). Study staff will review the Memory Aid with the participant and determine the severity of the reactions through discussion with the participant.

The following procedures will be conducted at these visits:

- Review interim medical history
- Collect concomitant medication information
- Perform a symptom-directed physical examination if any signs or symptoms are present
- Assess vital signs (pulse, respiratory rate, blood pressure and temperature)
- Assess any adverse events and local and systemic reactogenicity (Days 1, 2, 3) including reviewing the Memory Aid.
- Collect specimens for all tests as indicated in the Schedule of Procedures (Appendix A) and AP).

6.4 Additional Follow-up Visits

Assessments and procedures will be performed according to the Schedule of Procedures (Appendix A).

6.5 Unscheduled Visits

Unscheduled Visits/Contacts are visits/contacts that are not described in the Schedule of Procedures (Appendix A). Unscheduled visits may occur any time during the study:

- For administrative reasons, e.g., the participant may have questions for study staff or may need to re-schedule a follow-up visit.
- To obtain laboratory test results from a previous visit.
- For other reasons as requested by the participant or site investigator.

All unscheduled visits will be documented in the participants' study records on applicable source documents and entered into the Case Report Form (CRF).

6.6 Final Study Visit or Early Termination Visit

Assessments and procedures will be performed according to the Schedule of Procedures (Appendix A).

7.0 STUDY PROCEDURES

7.1 Informed Consent Process

A Master Informed Consent Document consisting of a Participant Information Sheet and a Consent Form is provided by the Sponsor to the trial site. This document is made site-specific and translated (if necessary), submitted and approved by the Institutional

Review Board (IRB). The Master and site specific Informed Consent Documents are separate documents and should not be part of the protocol.

Participant Information Sheet

A qualified member of the study staff will conduct the informed consent process by reviewing the Participant Information Sheet and document it in the clinic notes.

Consent Form

The participant's consent to participate must be obtained by him/her signing and dating the Consent Form. The person obtaining consent will also sign.

The signed and dated Informed Consent Document must remain at the study site. A copy of the signed/marked and dated Informed Consent Document will be offered to the participant to take home. Those participants who do not wish to take a copy will be required to document that they declined to do so.

7.2 Medical History and Physical Examination

Medical History

At screening, a comprehensive medical history will be collected including previous IV infusions and reaction to IV infusion, history of sexually transmitted infection (STI) and pregnancy prevention practices. At subsequent visits, an interim medical history will be performed.

Physical Examination

General Physical Examination

A general physical examination includes examination of head/ears/eyes/nose and throat, skin, respiratory, cardiovascular, abdominal, limited neurological and musculoskeletal and external ano-genital systems (for HIV-infected participants only) at the time points indicated in the Schedule of Procedures (see Appendix A).

Symptom-Directed Physical Examination

A symptom-directed physical examination is a targeted examination based on the volunteer's history or observation. If deemed necessary, this examination should be done at the time points indicated in the schedule of procedures (see Appendix A).

Measuring Height and Weight

Includes measuring the height and weight at the time points indicated in the Schedule of Procedures (see Appendix A).

Vital Signs

Vital signs including pulse, respiratory rate, blood pressure and temperature are measured and recorded at the time points indicated in the Schedule of Procedures (see Appendix A)

7.3 HIV Testing and HIV-test Counselling (Group 1)

Study staff will perform pre-HIV test counselling prior to collecting blood for an HIV test, and post-HIV test counselling when HIV test results are available. This is referred to as

HIV-test counselling, and done according to the CDC guidelines. For more information on HIV testing and HIV-test counselling, see Section 11.0. A screening questionnaire and other tools may be used.

7.4 HIV Risk Reduction Counselling

HIV risk reduction counselling will be provided to all participants as outlined by site-specific SOPs.

Study staff will provide HIV risk reduction counselling based on reported individual risk and provide free condoms, as appropriate, at every visit. Group 1 will receive HIV risk reduction counselling and for Groups 2 and 3, HIV risk reduction counselling will be conducted as secondary prevention to reduce onward transmission.

7.5 Family Planning Counselling

Study staff will counsel participants about the importance of preventing pregnancies and of using condoms, as well as other effective family planning methods, as appropriate. Participants may be referred for family planning services as necessary according to site-specific SOPs as detailed in the SOM. Pregnancy prevention methods chosen and compliance will be documented.

7.6 ART Counselling (Group 3)

HIV-infected participants who are not on ART will receive ART counselling upon entering the study and 8 weeks after administration of IP. Participants who have not initiated or made plans to initiate ART by the final study visit will receive ART counselling again at their final study visit.

7.7 Specimens

Approximately 32 ml of blood will be collected at the Screening Visit and approximately 16-24 or 110-142 ml of blood will be collected at later visit, depending on study procedures (see Appendix A), usually from the antecubital fossa.

Optional collection of rectal and/or cervical mucosal secretions will be obtained using a rectal sponge or cervical Softcup for those participants that consent.

All specimens will be handled according to the procedures specified in the AP.

In the event of an abnormal laboratory value, participants may be asked to have an additional sample collected at the discretion of the Principal Investigator or designee.

7.8 Reimbursement

Participants will be reimbursed for their time, effort and for costs to cover their travel expenses to the study site and any inconvenience caused due to study participation. Site specific-reimbursement amounts will be documented in the site-specific Participant Information Sheet, and approved by the Institutional Review Board.

7.9 Randomization and Blinding

Participants will be identified by a unique study identification number.

Participants will be randomized according to the randomization schedule prepared by the statisticians at the Data Coordinating Center (DCC) prior to the start of the study. Participants will be automatically assigned a specific allocation number as they are enrolled into the data entry system. An unblinding list (Pharmacy List) will be provided to the unblinded site pharmacist by the DCC.

This is a randomized, double-blind placebo-controlled study for groups 1 and 2, and an open label study for group 3. For Groups 1 and 2, study staff (investigator and clinical personnel monitoring the safety and laboratory assay results) and participants will be blinded with respect to the allocation of Investigational Product (PGT121 mAb or placebo). A site pharmacist will be unblinded for the purposes of preparing study product.

A participant will be considered enrolled once he/she has been assigned an allocation number.

Blinded participants will be informed about their assignment (product/placebo) at study completion, once the database is locked. Should a study participant be unblinded during the study, the study participant will be followed up until the end of the study according to the Schedule of Procedures (Appendix A).

7.10 Un-blinding Procedure for Individual Participants

Un-blinding of an individual participant may be indicated in the event of a medical emergency if the clinical management of the participant would be altered by knowledge of the treatment assignment.

The un-blinded information should be restricted to a small group of individuals involved in clinical management/medical treatment of the participant (e.g. treating physician) and the blind must be maintained for those responsible for the study assessments.

The reasons for un-blinding should be documented and the IAVI Chief Medical Officer, the Medical Monitor and the DCC should be notified as soon as possible. The procedures and contact numbers for un-blinding are outlined in the SOM.

7.11 Assessment of IP related HIV sero-positivity

It is possible that PGT121 mAb or an immune response to PGT121 mAb could cause a positive result on a diagnostic HIV antibody test. A Group 1 volunteer who tests HIV antibody positive at the end of the study will have additional testing to distinguish actual HIV infection from IP-related responses. The volunteer will be informed of his/her positive HIV antibody test result and offered continuing follow-up until the HIV antibody test becomes negative.

8.0 INVESTIGATIONAL PRODUCT

8.1 Description

A summary of the Investigational Products is shown in Table 8.1-1.

Table 8.1-1 Investigational Products

Product / Placebo	Dosage level	Total volume in IP container	Total IP or placebo volume to be injected into a 100 mL saline IV bag (for an 88 kg body weight**)	Total volume to be Infused (for an 88 kg body weight**)
IP: PGT121 (50 mg/mL)	3 mg/kg	6 mL per vial	5.3 mL	105.3 mL
	10 mg/kg		17.6 mL	117.6 mL
	30 mg/kg		52.8 mL	152.8 mL
Placebo: 0.9% Sodium Chloride Injection USP (Saline)*	3 mg/kg matching***	NA	5.3 mL***	105.3 mL***
	10 mg/kg matching***		17.6 mL***	117.6 mL***
	30 mg/kg matching***		52.8 mL***	152.8 mL***

* The Placebo provided will be a commercially-available saline partial addition IV bag.

** The actual volume to be injected will be based on the dose group and the weight of the participant at the time of IP administration. The example included here is the average weight of an adult male in the US (88kg) (http://www.cdc.gov/nchs/data/series/sr_11/sr11_252.pdf)

*** For placebo IV infusions: saline from an additional IV bag will be injected into the saline IV bag intended for administration, to match the volume used for a PGT121 mAb injection in the same dose group, to prevent unblinding.

8.2 Shipment and Storage

Authorization to ship the Investigational Product to the site will be provided in writing by the Sponsor, upon confirmation that all required critical documents for shipment authorization are completed. The Investigational Product will be shipped maintaining the required storage conditions and stored in a secure location in the clinical site's pharmacy.

The Investigational Product is formulated in a 20 mM Acetate, 9% Sucrose, 0.008% polysorbate 80, pH 5.2 formulation buffer at a concentration of 50 mg/mL. Each 10 ml vial will contain 6 ml of IP stored at <- 20°C. Each vial will be labelled with the name of the product, Lot number, concentration, storage temperature, date of manufacturing, contact information of the Sponsor and a US cautionary statement. Several such vials will be packaged in a box. Each box will also be labelled with similar information as the vial label.

8.3 Preparation of Investigational Product (IP)

Detailed instruction will be provided to the site pharmacist in the SOM for preparing each of the investigational products. The site pharmacist will not be blinded, but the study physician/designee administering the IP will be blinded. Product should be administered within 6 hours of preparation. Example calculations for final volume for IV infusion are illustrated in Table 8.1-1. Instructions for storing used vials for reconciliation until the end of the study and subsequent disposal will be provided in the SOM. Syringes or other components in direct contact with investigational products will be disposed of in a biohazard container and incinerated or autoclaved.

8.4 Administration of Investigational Product

Investigational Product will be administered at the enrollment visit.

The IP will be injected into a 0.9% Saline bag. The participant will receive the IP via IV infusion. Participants will receive infusion over approximately 60 minutes, allowing for clinician discretion. Further information on the IV infusion of the IP is supplied in the SOM and study documents.

8.5 Accountability and Disposal of Investigational Product

All used vials will be retained at the pharmacy at the end of each IP administration visit. The date, allocation number and location of storage of the returned vials will be recorded.

During the study, the IP accountability forms including receipt and dispensing of vials will be kept and monitored.

At the end of the study, the used and unused IP vials will be handled according to instructions of Sponsor.

Further information on accountability and disposal of IP is supplied in the SOM.

9.0 ASSESSMENTS

9.1 Safety Assessments

Data on local and systemic reactogenicity (i.e., solicited AEs) will be collected by structured interview and medical examination. Data on other adverse events will be collected with open-ended questions. All data will be recorded on the appropriate source documents and entered into the study database. Participants will be given a Memory Aid, which is a tool to assist with collecting reactogenicity data.

Local and systemic reactogenicity events will be assessed by study staff prior to IV infusion of IP, at approximately 30 minutes after IP administration start, at 1 hour after IP administration start, and subsequently every hour for the first 12 hours post-IP administration. Study staff will review the Memory Aid with the participant, and determine the severity of the reactions on days 1-3 through discussion with the participant.

9.1.1 Local reactogenicity

The presence of local reactogenicity will be assessed at the time points specified in the Schedule of Procedures (Appendix A).
Pain, tenderness, erythema/skin discoloration, swelling/hardening or pruritus will be assessed and graded using Appendix C, Adverse Event Severity Assessment Table, as a guideline.

9.1.2 Systemic reactogenicity

The presence of systemic reactogenicity will be assessed at the time points specified in the Schedule of Procedures (Appendix A).

Fever, chills, headache, nausea, vomiting, malaise, myalgia and arthralgia will be assessed and graded using the Appendix C, Adverse Event Severity Assessment Table as a guideline.

9.1.3 Vital Signs

At the administration of IP visit, vital signs (pulse, respiratory rate, blood pressure and temperature) will be measured by study staff prior to IP administration, at approximately 30 minutes post IP administration and hourly until 12 hours after IV infusion start. For the other study visits vital signs will be assessed at the time points specified in the Schedule of Procedures (Appendix A).

9.1.4 Other Adverse Events

Other adverse events (AEs) will be collected through 56 days after IP administration in all participants. Serious Adverse Events (SAEs) will be collected throughout the entire study period. Potential Immune Mediated Diseases (pIMDs), as defined in Section 10.5, will be collected throughout the study period, using the SAE reporting process. Open-ended questions will be asked at time points according to the Schedule of Procedures (Appendix A). All adverse events will be graded using Appendix C, Adverse Event Severity Assessment Table, as a guideline and will be assessed for causality to the IP. For more information regarding adverse events refer to Section 10.0, Adverse Events.

9.1.5 Concomitant Medications

Concomitant receipt of Investigational Products is prohibited during the study.

Contraceptive use and use of medication at study entry will be documented. (See DCF instructions)

During the study, information regarding concomitant medications and reasons for their use will be solicited from the study participants for 56 days. Ongoing concomitant medications will be recorded until end of study.

9.1.6 Routine laboratory parameters

Table 9.1.6-1 shows the laboratory parameters that will be measured routinely. The samples for these tests will be collected at the time points indicated in the Schedule of Procedures (Appendix A).

Table 9.1.6-1: Laboratory Parameters

Laboratory Parameter	Test
Hematology and Coagulation	Hemoglobin, hematocrit, leukocytes, platelets, absolute neutrophil count (ANC), absolute lymphocyte count (ALC), activate partial thromboplastin time (aPTT), international normalized ratio (INR)
Clinical Chemistry	Sodium, potassium, creatinine, aspartate aminotransferase (AST), alanine aminotransferase (ALT), total bilirubin, alkaline phosphatase, albumin, creatine kinase, C-reactive protein, C3 complement, C4 complement

Urinalysis	Dipstick test for protein, blood glucose, ketones, esterase (leukocytes) and nitrite. If clinically significant abnormalities (e.g., blood, protein, leukocytes) are found on dipstick test, then further test(s) will be performed (e.g., microscopy, culture)
T cell panel (Groups 2 and 3)	CD4 T cell count and frequency by single platform flow cytometry

9.1.7 Specific screening tests:

Participants will be screened to exclude the following diseases:

- Hepatitis B: positive for hepatitis B surface antigen (HBsAg)
- Hepatitis C: positive for hepatitis C RNA (HCV antibody test, followed by HCV RNA test if HCV antibody positive)
- Active syphilis: confirmed diagnosis (e.g.; positive RPR confirmed by TPHA)

A negative Hepatitis B and Hepatitis C result can be documented from the medical record only if the result is from a test administered less than 6 months ago.

Participants will also be screened to exclude the following laboratory parameters:

- Autologous PGT121-like antibody ELISA level above the cut-off;
- Resistance of autologous HIV to PGT121 neutralization *in vitro* (HIV viremic participants only, Group 3)
- Estimated Glomerular filtration rate or GFR (group 3 only)

9.1.8 Monitoring for anti-PGT121 antibodies:

Participants will be evaluated for the development of antibodies to PGT121 mAb by ELISA according to the Schedule of Procedures (Appendix A).

9.2 Virologic Assessments

Table 9.2-1 shows the virologic parameters that will be measured routinely. The samples for these tests will be collected at the time points indicated in the Schedule of Procedures (Appendix A).

Table 9.2-1: Virologic Assessment Table

**Virologic Parameter**	**Test**
Antiviral Activity	Plasma HIV RNA levels
Anti-reservoir activity	Cell-associated HIV-1 RNA levels in resting CD4 T cells; total HIV-1 DNA and 2-long terminal repeat (LTR) HIV-1 DNA circles in resting or total CD4 T cells; quantitative viral outgrowth assay (qVOA)
Other	Genotyping of plasma HIV RNA for evaluation of PGT121-induced escape mutations

9.3 Exploratory Immunogenicity Assessments

Humoral immune response assays will include, but are not limited to Env-specific Ab-binding assays, virus neutralization assay, and assays for Ab functionality. Cellular immune response assays will include, but are not limited to IFNγ ELISPOT assay, ICS, and multiparameter flow cytometry. Exploratory assessments on mucosal samples will include, but are not limited to characterization of Env-specific binding Abs. Priority assays are listed below.

9.3.1 Antibody Responses

- Env-specific binding Abs (titers and breadth).
- Env-specific nAbs (titers and breadth).
- Env-specific functional Abs (phagocytosis score and breadth).
- Env-specific binding Ab isotypes (IgA, IgG1-4) (titers and breadth).

9.3.2 Cellular Responses

- IFNγ peripheral blood mononuclear cell (PBMC) responders to peptide pools and subpools of Potential T-cell epitopes, PTE Env/Gag/Pol peptides.
- $CD4^+$ and $CD8^+$ T-cell functionality (% cells producing e.g. IFNγ, IL-2, IL-4, TNFα).
- T-cell development with emphasis on follicular helper T-cells and memory differentiation.

9.3.3 PBMC, Serum and Plasma Storage

Samples of cryopreserved PBMC, plasma and serum will be stored as indicated in the Analytical Plan (AP) and, if the participant consents, may be used for the purposes of standardization, quality control and for future assays related to HIV prevention or treatment research and development. These samples will be archived and the testing laboratories will be blinded to the participant's identity.

9.4 Other Assessments

9.4.1 HIV Antibody Testing

All HIV-uninfected participants (Group 1) will be tested for HIV antibodies as indicated in the Schedule of Procedures (Appendix A) or as needed, if medical or social circumstances arise. All participants will receive HIV risk reduction counselling and pre- and post-HIV-test counselling, as specified in Section 7.3 Counselling.

9.4.2 Pharmacokinetics

Blood draws for pharmacokinetics will be done on the day of IP administration immediately before starting IV infusion of IP, at the end of the IP administration, and 30 minutes and 3 hours after the end of the IP administration. Additional draws will be done at 6, 9, 12 and 24 hours after the start of the IP administration. Thereafter, pharmacokinetic draws will be done as indicated in

the Schedule of Procedures (Appendix A). PGT121 mAb serum or plasma levels will be determined using two methods: a sandwich ELISA using a murine anti-idiotype antibody to PGT121 mAb, and a neutralization assay.

PGT121 mAb pharmacokinetic analysis will be performed using standard non-compartmental analysis methods to estimate elimination half-life (t1/2), clearance (CL/F), volume of distribution (Vz/F), Area under the concentration decay curve (AUC), impact of viral load and/or ART on PGT121 mAb disposition (elimination half-life (t1/2), clearance (CL/F), volume of distribution (Vz/F) and total exposure. PGT 121 accumulation will also be examined in rectal and cervical mucosal secretions collected with rectal sponges or cervical Softcups in study participants who specifically consented for these procedures. Descriptive results will be reported for the pharmacokinetic parameters by dose subgroup.

Exploratory analysis using population analysis methods simultaneously combining all pharmacokinetic data across all doses and treatment groups will be performed for quantitative characterization of differences in PGT121 mAb disposition by dose, participant group or disease state.

9.4.3 HLA Typing

Samples for HLA typing will be collected as specified in the AP and may be analyzed as warranted.

9.4.5 Pregnancy Test

A urine pregnancy test for all female participants will be performed by measurement of human chorionic gonadotrophin (βhCG) at time points indicated in the Schedule of Procedures (Appendix A). The results of the pregnancy test must be negative prior to IV infusion of PGT121 mAb. See section 10.7 for description of pregnancy after administration of IP.

9.4.6 HIV Risk Assessment (Group 1)

Study staff will assess participants for their past and current risk of acquiring HIV at time points indicated in Schedule of Procedures (Appendix A).

9.4.7 Social Impact Assessment

A brief assessment of the impact of participation in the study will be administered to participants at their final study visit.

10.0 ADVERSE EVENTS

10.1 Definition

An adverse event (AE) is any untoward medical occurrence in a participant administered an Investigational Product and which does not necessarily have a causal relationship with the Investigational Product. An AE can therefore be any unfavourable or unintended sign (including an abnormal laboratory finding), symptom, or disease, temporally associated with the use of Investigational Product whether or not related to the Investigational Product.

Assessment of severity of all AEs, including and seriousness of AEs, is ultimately the responsibility of the Principal Investigator of each site. Refer to the DIVISION OF AIDS (DAIDS) TABLE FOR GRADING THE SEVERITY OF ADULT AND PEDIATRIC ADVERSE EVENTS Version 2.0, November 2014 for additional guidance.

10.2 Assessment of Severity of Adverse Events

The following general criteria should be used in assessing adverse events as mild, moderate, severe or very severe at the time of evaluation:

Grade 1 (Mild): Symptoms causing no or minimal interference with usual social & functional activities

Grade 2 (Moderate): Symptoms causing greater than minimal interference with usual social & functional activities

Grade 3 (Severe): Symptoms causing inability to perform usual social & functional activities

Grade 4 (Very Severe): Symptoms causing inability to perform basic self-care functions OR Medical or operative intervention indicated to prevent permanent impairment, persistent disability, or death

Guidelines for assessing the severity of specific adverse events and laboratory abnormalities are listed in Appendix C, Adverse Event Severity Assessment Table.

10.3 Relationship to Investigational Product

Assessment of relationship of an AE or SAE to Investigational Product (IP) is the responsibility of the Principal Investigator or designee. All medically indicated and available diagnostic methods (e.g., laboratory, blood smear, culture, X-ray, etc.) should be used to assess the nature and cause of the AE/SAE. Best clinical and scientific judgment should be used to assess relationship of AE/SAEs to the IP and/or other cause.

The following should be considered:

- Presence/absence of a clear temporal (time) sequence between administration of the IP and the onset of AE/SAE
- Presence/absence of another cause that could more likely explain the AE/SAE (concurrent disease, concomitant medication, environmental or toxic factors)
- Whether or not the AE/SAE follows a known response pattern associated with the IP

The relationship assessment should be reported as one of the following:

Not Related: clearly explained by another cause (concurrent disease, concomitant medication, environmental or toxic factors, etc.).

Unlikely: more likely explained by another cause (concurrent disease, concomitant medication, environmental or toxic factors, etc.).

Possibly: equally likely explained by another cause but the possibility of the IP relationship cannot be ruled out (e.g., reasonably well temporally related and/or follows a known IP response pattern but equally well explained by another cause).

Probably: more likely explained by the IP (e.g., reasonably well temporally related and/or follows a known IP response pattern and less likely explained by another cause).

Definitely: clearly related and most likely explained by the IP.

For the purpose of expedited safety reporting, all possibly, probably or definitely related SAEs are considered IP-related SAEs.

10.4 Serious Adverse Events

An adverse event is reported as a "Serious Adverse Event" if it meets any of the following criteria (as per International Conference on Harmonisation [ICH] Good Clinical Practice [GCP] Guidelines):

- Results in death
- Is life threatening
- Results in persistent or significant disability/incapacity
- Requires in-participant hospitalization or prolongs existing hospitalization
- Is a congenital anomaly/birth defect or spontaneous abortion
- Any other important medical condition that requires medical or surgical intervention to prevent permanent impairment of a body function or structure

Elective surgery for pre-existing condition that did not increase in severity or frequency is not considered an SAE.

Serious Adverse Events (SAEs) should be reported within 24 hours of the site becoming aware of the event, and sent to the Sponsor as described in the SOM.

To discuss IP-related SAEs or any urgent medical questions related to the SAE, the site investigator should contact one of the IAVI Medical Monitors directly (see Contact List in the SOM).

The IAVI SAE Report Form should be completed with all the available information at the time of reporting and sent to the Sponsor as described in the SOM. The minimum data required in reporting an SAE are the study identification number, date of birth, gender, event description (in as much detail as is known at the time), onset date of event (if available), reason event is classified as serious, reporting source (name of Principal Investigator or designee), and relationship to the IP as assessed by the investigator.

The Principal Investigator or designee is required to prepare a detailed written report with follow up until resolution or until it is judged by the Principal Investigator or designee to have stabilized.

The Principal Investigator or designee must notify the local IRB/IEC of all SAEs as appropriate. In case of IP-related SAEs, the Sponsor will notify responsible regulatory authorities, Safety Monitoring Committee (SMC), and other study sites where the same IP is being tested.

More details on SAE definitions and reporting requirements are provided in the SOM.

Serious Event Prior to Investigational Product Administration

If a serious event occurs in the period between the participant signing the Informed Consent Form and receiving the IV infusion of IP, the event will be reported using the SAE form and following the same procedures for SAE reporting, as indicated in Section 10.4. The timing of the event will be indicated by using the relevant checkbox on the SAE form.

10.5 Reporting Potential Immune-Mediated Diseases

Potential immune-mediated diseases (pIMDs) are a subset of AEs that include both clearly autoimmune diseases and also other inflammatory and/or neurologic disorders that may or may not have an autoimmune etiology. These events are of special interest since they could potentially be caused by immune responses to the IP. The investigator/designee should report such adverse events within the same time limits (following confirmation of an AE as a pIMD; see last paragraph of this section below), and using the same CRF pages, as utilized for SAEs. The investigator or his/her designee will evaluate the occurrence of pIMDs at every visit/contact during the study. IAVI will also expect investigators/designee to provide additional information about pIMD events. AEs to be reported and documented as pIMDs include:

Neuroinflammatory disorders: optic neuritis, cranial nerve disorders (including Bell's palsy), multiple sclerosis, demyelinating disease, transverse myelitis, Guillain-Barré syndrome, myasthenia gravis, encephalitis, neuritis.

Musculoskeletal disorders: systemic lupus erythematosus, cutaneous lupus, Sjögren's syndrome, scleroderma, dermatomyositis, polymyositis, myopathy, rheumatoid arthritis and juvenile rheumatoid arthritis, polymyalgia rheumatica or temporal arteritis, reactive arthritis, psoriatic arthropathy, ankylosing spondylitis, undifferentiated spondyloarthropathy.

Gastrointestinal disorders: Crohn's disease, ulcerative colitis or proctitis, celiac disease.

Metabolic diseases: autoimmune thyroiditis, Grave's or Basedow's disease, Hashimoto thyroiditis, insulin-dependent diabetes mellitus (IDDM), Addison's disease.

Skin disorders: psoriasis, vitiligo, Raynaud's phenomenon, erythema nodosum, autoimmune bullous skin diseases.

Others: autoimmune hemolytic anemia, thrombocytopenia, antiphospholipid syndrome, *vasculitis, pernicious anemia, autoimmune hepatitis, primary biliary cirrhosis, primary sclerosing cholangitis, autoimmune glomerulonephritis, autoimmune uveitis, autoimmune myocarditis/cardiomyopathy, sarcoidosis, Stevens-Johnson syndrome, Behçet's syndrome.

Infusion site reactions: Grade 3 or 4 injection site reactions lasting more than 2 days.

*Vasculitis: Vasculitis, Diffuse vasculitis, leucocytoclastic vasculitis, polyarteritis nodosa, microscopic polyangiitis, Wegener's granulomatosis, anti-neutrophil cytoplasmic antibody positive vasculitis, Henoch-schönlein purpura, allergic granulomatous angiitis (Churg-Strauss disease), Kawasaki disease, Takayasu's arteritis, temporal arteritis (giant cell arteritis), renal vasculitis.

Medical judgement should be exercised in deciding whether other disorders/diseases have an autoimmune origin and should also be reported as described above, and this judgement is the investigator's prerogative. Whenever sufficient data exist to substantiate any of the diagnoses in the above list, the event must be reported as a pIMD. While the intent of pIMD reporting is to be inclusive, isolated nonspecific symptoms, which might (or might not) represent the above diagnoses, should be captured as AEs but not reported as pIMDs until the diagnosis can be defended.

10.6 Clinical Management of Adverse Events

Adverse events (AEs) will be managed by the clinical study team who will assess, provide first line of care as appropriate and refer to health care and treatment facilities as warranted. If any treatment/medical care is required as a result of the harm caused by the IP or study procedures, this will be provided free of charge.

If a participant has an AE and/or abnormal laboratory value that is known at the time of IV infusion of IP, the specifications of Section 12.0 will be followed.

Participants will be followed until the AE resolves or stabilizes or up to the end of the study, whichever comes last. If at the end of the study, an AE (including clinically significant laboratory abnormality) that is considered possibly, probably or definitely related to the IP is unresolved, follow-up will continue until resolution if possible and/or the participant will be referred.

10.7 Pregnancy

Although not considered an AE, if a female participant becomes pregnant during the study, it is the responsibility of the Principal Investigator or designee to report the pregnancy promptly to IAVI using the designated forms. The participant will be followed for safety until the end of pregnancy or study completion, whichever occurs last. If possible, approximately 2–4 weeks after delivery, the baby will be examined by a physician to assess its health status and the results will be reported to the Sponsor. The baby will be examined again by a Physician around age 1, and the results will be reported to the Sponsor.

Complications of pregnancy that meet criteria for SAEs, specified in Section 10.4 of this Protocol (e.g., hospitalization for eclampsia, spontaneous abortion, etc.) should be reported as SAEs.

10.8 Intercurrent HIV Infection (Group 1)

HIV infection cannot be directly caused by the IP. If a participant acquires HIV through exposure in the community, at any time after the IV infusion of IP, the participant should be offered referral to appropriate care and treatment facilities. The participant will continue to be followed in the study for safety assessments.

Intercurrent HIV infection in study participants, although not considered an SAE, must be reported promptly to IAVI using the designated forms. However, medical conditions associated with the HIV infection that meet criteria for being serious specified in the Section 10.4 of this Protocol (e.g., sepsis, *Pneumocystis jiroveci [carinii] pneumonia*, etc.) should be reported as SAEs using the SAE Report Form.

11.0 MANAGEMENT OF HIV ISSUES DURING AND FOLLOWING STUDY

11.1 HIV Testing – Group 1

Group 1 participants will be tested for HIV antibodies as indicated in the Schedule of Procedures (Appendix A) or as needed, if medical or social circumstances arise. All participants will receive HIV risk reduction counselling and pre- and post-HIV-test counselling, as specified in Section 11.2.1, Counselling (Group 1).

It is possible that PGT121 or an immune response to PGT121 mAb could cause a positive result on a diagnostic HIV antibody test. An IP recipient who falsely tests HIV positive with a diagnostic HIV antibody test at the end of the study will be informed of his/her positive test result and offered continuing follow-up until the test becomes negative.

If a participant acquires HIV through exposure in the community, at any time after the administration of IP, the participant will be offered referral to appropriate care and treatment facilities. The participant will continue to be followed in the study for safety assessments.

Should a participant require HIV testing outside of the study for personal reasons, it is recommended that the participant contact the study staff first. HIV testing can be done at the study site and then processed at an independent laboratory as above. Written evidence of HIV status (HIV-infected or HIV-uninfected) will be provided upon request.

11.2 Social Discrimination as a Result of IP-related antibodies

In order to minimize the possibility of social discrimination in participants (if any) who test positive on a diagnostic HIV antibody test due to IP-related antibodies, appropriate diagnostic HIV testing and certification will be provided both during and after the study as needed.

11.3 HIV infection – Group 1

Group 1 participants who are diagnosed with HIV infection at screening or during the study (intercurrent HIV-infection) will be provided the following:

11.3.1 Counselling

The participant will be counselled by the study investigators or designated counsellors. The counselling process will assist the participant with the following issues:

- Psychological and social implications of HIV infection

- Who to inform and what to say
- Implications for sexual partners
- Implications for child-bearing
- Avoidance of transmission to others in future
- Mandatory reporting to the state, in some instances

11.3.2 Referral for Support/Care

Participants will be referred to a participant support center or institution of his/her choice for a full discussion of the clinical aspects of HIV infection. Referral will be made to a designated physician or center

12.0 WITHDRAWAL FROM STUDY

12.1 Deferral of IV infusion of IP

An IV infusion of IP may be temporarily deferred if the participant is clinically ill at the time of the administration of IP visit and/or presents with fever (≥ 100.4 F) at the time of the administration of IP visit. A participant must be clinically well and afebrile for a minimum of a 24-hour consecutive period prior to administration of IP.

Any planned or unplanned deferral of infusion of IP will be discussed with the Sponsor. Participants will be deferred from infusion of IP for any of the following reasons:

1. Pregnancy
2. A disease or condition or adverse event that may develop, regardless of relationship to Investigational Product, if the Principal Investigator or designee is of the opinion that administration of IP will jeopardize the safety of the participant
3. Participant's request to defer infusion

The following events require resolution and/or review of clinical history by the Principal Investigator or designee and consultation with the Medical Monitor, prior to administration of IP:

- Any abnormal laboratory value, as outlined in section 5.7, Exclusion Criteria, Hematology, Chemistry, Urinalysis that is known at the time of infusion and have not resolved. Abnormal results should be confirmed on the original sample and/or repeated at least once to confirm abnormal values.
- Receipt of inactivated/killed/subunit vaccines (non-HIV) or immunoglobulin within the previous 14 days. Receipt of live attenuated vaccines within the previous 60 days.
- Participating in another clinical study of an Investigational Product

12.2 Withdrawal from the Study (Early Termination)

Participants may be withdrawn from the study permanently for the following reasons:

1. Participants may withdraw from the study at any time if they wish, for any reason
2. The Principal Investigator or designee has reason to believe that the participant is not complying with the protocol
3. If the Sponsor decides to terminate or suspend the study

If a participant withdraws or is withdrawn from the study, all termination visit procedures will be performed according to the Schedule of Procedures (Appendix A) where possible. Every effort will be made to determine and document the reason for withdrawal.

13.0 DATA HANDLING

13.1 Data Collection and Record Keeping at the Study Site

Data Collection: All study data will be collected by the clinical study staff using designated source documents and entered onto the appropriate electronic CRFs (eCRFs). Access to eCRFs will be provided via an electronic data entry system hosted by the Data Coordination Center. All study data must be verifiable to the source documentation. A file will be held for each participant at the clinic(s) containing all the source documents. Source documentation will be available for review to ensure that the collected data are consistent with the CRFs.

All CRFs and laboratory reports will be reviewed by the clinical team, who will ensure that they are accurate and complete.

Source documents and other supporting documents will be kept in a secure location. Standard GCP practices will be followed to ensure accurate, reliable and consistent data collection.

Source documents include but are not limited to:

- Signed Informed Consent Documents
- Progress notes
- Data collection forms
- Documentation of any existing conditions or past conditions relevant to eligibility
- Printed laboratory results
- Print out of the IDES generated enrollment confirmation
- All Adverse Events
- Concomitant medications
- Local and systemic reactogenicity events

13.3 Data Entry at the Study Site

The data collected at the site will be recorded onto the eCRFs by the study staff and entered into a database. To provide for real time assessment of safety, data should be entered as soon as reasonably feasible after a visit occurs.

13.4 Data Analysis

The Sponsor, PIs and Product Developers will agree on how data will be analyzed and presented prior to unblinding of the study.

The DCC will conduct the data analysis and will provide interim safety and final study reports for the Sponsor, Principal Investigators, the PSRT and SMC and the regulatory authorities, as appropriate.

14.0 STATISTICAL CONSIDERATIONS

14.1 Safety and Tolerability Analysis

14.1.1 Sample Size

The sample size for safety and tolerability analysis will be 30-48 participants according to the dose escalation design used to characterize the safety profile of one IV infusion of PGT121 mAb, at one of three dose levels, to HIV-uninfected and HIV-infected individuals (groups 1 and 2).

14.1.2 Null Hypothesis

As this is an exploratory proof of concept trial and analysis will be descriptive, no formal null hypothesis will be tested.

14.1.3 Statistical Power and Analysis and Dose Escalation Rules

The frequency of moderate or greater local and systemic reactogenicity events will be determined and compared between groups.

The frequency of SAEs judged possibly, probably or related to the IP will be determined.

All AEs will be analyzed and, grouped by seriousness, severity and relationship to the Investigational Product (as judged by the investigator).

For life-threatening adverse events related to Investigational Product: if none of the 12 (max 18) participants receiving Investigational Products experience such reactions, then the 95 % upper confidence bound for the rate of these adverse events in the population is 26.5% (or 18.5% if n=18).

All AEs will be analysed and grouped by seriousness, severity and relationship to the IP (as judged by the investigator).

For life-threatening adverse events related to IP: if none of the 12 (max 18) participants in either Group 1 or Group 2 who receive the IP experience such reactions then the 95% upper confidence bound for the rate of these adverse events in the population is 26.5% (or 18.5% if n=18).

An interim analysis of group data will be carried out according to the study schema (Table 5.3.1) without unblinding the study to investigators or participants. At the end of the study, a full analysis will be prepared.

Based on previous experience with IAVI Phase 1 IP studies, it is expected that the amount of missing, unused or spurious data will be insignificant. Unused and spurious data will be listed separately and excluded from the statistical analysis. Missing data will be excluded from the statistical analysis.

14.2 Pharmacokinetic Analysis

14.2.1 Sample Size

The sample size for pharmacokinetic analysis will be 4 per dose subgroup, sufficient to provide sufficient information for the planned analyses.

14.2.2 Null Hypothesis

As this is an exploratory proof of concept trial and analysis will be descriptive, no formal null hypothesis will be tested.

14.2.3 Statistical Power and Analysis

Disposition of PGT121 mAb will be evaluated in this study. Based on the PK profile of other human monoclonal antibodies, it is expected that the half-life of PGT121 mAb will be 14 to 21 days. Previously published data indicates that the pharmacokinetics of PGT121 and 3BNC117 are fairly similar across a non-human primate cohort and within the same non-human primate (clearance of 3BNC117 appears to be marginally faster than that for PGT121).

Commonly reported PK parameters will be calculated using standard non-compartmental slope/height/area/moment (SHAM) analysis methods. Summary descriptive results of PK parameters, including AUC, Cmax, T1/2, and clearance results will be reported by dose cohort. Dose normalized plots of PK parameters will be presented. Correlation between PK and reported safety and pharmacodynamic outcomes will also be explored parameters in order to examine exposure-effect relationships.

A more powerful exploratory analysis to quantitatively determine the dose, participant and disease impact on PGT121 mAb pharmacokinetics, and correlate exposure with response, while correctly accounting for variance based on population intrinsic factors such as weight and gender will be performed. Using the proposed population analysis approach we will be able to simultaneously examine the magnitude and the rate of change to PGT121 disposition driven by HIV-1 RNA levels and/or ART, and also examine the magnitude and the rate of decline in log copies/ml of HIV-1 RNA plasma levels from baseline.

The frequency and levels of anti-PGT121 antibodies will be calculated and tabulated.

14.3 Virologic Analysis for Dose De-escalation in Groups 3A-C

14.3.1 Sample Size

The sample size for virologic analysis in Groups 3A-C will be 24-36 participants according to the dose de-escalation design described below.

14.3.2 Null Hypothesis

The null hypothesis is that there is no difference in antiviral activity between PGT121 mAb and placebo.

14.3.3 Statistical Power and Analysis

The virologic analysis described in this section relates to Groups 3A-C of the study design, in which dose de-escalation is performed in an adaptive study design in HIV-infected participants off ART with plasma HIV RNA levels of 2 x 10^3 – 10^5 copies/ml. This section assumes that Part 1 of the study has successfully demonstrated that there is a safe dose level of the IP such that the study is carried forward into Part 2.

The primary efficacy outcome for this analysis is defined as change in log10 viral load between Day 0 (day of infusion) and Day 7. The minimum clinically significant value for this outcome is defined as a difference of -0.9 log10.

The study plan for Groups 3A-C is designed so that the IP dose level may be de-escalated in a stepwise manner from the highest dose to the lowest dose, until a given dose level cannot be concluded to be efficacious. If any given dose level is proven to be efficacious at an interim analysis, enrolment for that dose level may cease, and the next lowest dose group may be enrolled. In the unlikely event that IP administration leads to increased viral load, this may be detected by this design. No placebo participants are enrolled as part of this design.

This design represents a dose de-escalation beginning at 30 mg/kg. The actual starting dose will be the MTD as determined by the SMC based on data from Part 1, therefore the starting dose may be 30mg/kg, 10 mg/kg or 3 mg/kg. If the starting dose is 30 mg/kg, then de-escalation will begin with Group 3A. If the starting dose is 10 mg/kg, then de-escalation will begin with Group 3B. If the starting dose is 3 mg/kg, then only Group 3C will be enrolled.

Assuming the starting dose is 30 mg/kg, an interim analysis of Group 3A will be performed after all 6 participants have reached 7 days following IP administration. The following scenarios may then occur, depending on the data:

- If the mean response in the first 6 participants is a decrease greater than or equal to -0.9 log10 HIV RNA, the IP will be determined to be effective at 30 mg/kg, enrollment into Group 3A will cease, and enrollment into Group 3B will begin.
- If the mean response in the first 6 participants is a decrease smaller than -0.9 log10 HIV RNA, then an additional 3 participants will be enrolled into Group 3A. After the additional 3 participants have reached 7 days following IP administration, an analysis of Group 3A (N=9) will be performed:
    - If the mean response in all Group 3A participants is a decrease greater than or equal to -0.9 log10 HIV RNA, the IP will be determined to be effective at 30 mg/kg, and enrollment into Group 3B will begin.
    - If the mean response in all 9 participants is a decrease smaller than -0.9 log10 HIV RNA, then the IP will be determined to be ineffective at 30 mg/kg and Groups 3B and 3C will not be enrolled. In this scenario, no dose of IP will be determined to be effective.

If 30 mg/kg is determined to be an effective dose, then Group 3B will be enrolled at 10 mg/kg. An interim analysis of Group 3B will be performed after 8 participants have

reached 7 days following IP administration. The following scenarios may then occur, depending on the data:

- If the mean response in the first 8 participants is a decrease greater than or equal to -0.9 log10 HIV RNA, the IP will be determined to be effective at 10 mg/kg, enrollment into Group 3B will cease, and enrollment into Group 3C will begin.
- If the mean response in the first 8 participants is a decrease smaller than -0.9 log10 HIV RNA, then an additional 4 participants will be enrolled into Group 3B. After the additional 4 participants have reached 7 days following IP administration, an analysis of Group 3B (N=12) will be performed:
  - If the mean response in all Group 3B participants is a decrease greater than or equal to -0.9 log10 HIV RNA, the IP will be determined to be effective at 10 mg/kg, and enrollment into Group 3C will begin.
  - If the mean response in all 12 participants is a decrease smaller than -0.9 log10 HIV RNA, then the IP will be determined to be ineffective at 10 mg/kg, and Group 3C will not be enrolled. In this scenario, the minimum effective dose will be determined to be 30 mg/kg.

If 10 mg/kg is determined to be an effective dose, then Group 3C will be enrolled at 3 mg/kg. An interim analysis of Group 3C will be performed after 10 participants have reached 7 days following IP administration. The following scenarios may then occur, depending on the data:

- If the mean response in the first 10 participants is a decrease greater than or equal to -0.9 log10 HIV RNA, the IP will be determined to be effective at 3 mg/kg and enrollment into Group 3C will cease. In this scenario, the minimum effective dose of the IP will be determined to be 3 mg/kg.
- If the mean response in the first 10 participants is a decrease smaller than -0.9 log10 HIV RNA, then an additional 5 participants will be enrolled into Group 3C. After the additional 5 participants have reached 7 days following IP administration, an analysis of Group 3C (N=15) will be performed:
  - If the mean response in all Group 3C participants is a decrease greater than or equal to -0.9 log10 HIV RNA, the minimum effective dose will be determined to be 3 mg/kg.
  - If the mean response in all 15 participants is a decrease smaller than -0.9 log10 HIV RNA, then the IP will be determined to be ineffective at 3 mg/kg. In this scenario, the minimum effective dose will be determined to be 10 mg/kg.

For the analysis of sample size and power, log10 viral load differences from baseline for each participant were simulated from a normal distribution, with a standard deviation of 0.5. This value was chosen by examining a study of the antiretroviral drug raltegravir, which demonstrated a mean estimated standard deviation of the change of baseline of 0.47[18]. This is a conservative estimate, as the variability of viral loads near the lower range might be expected to also be lower.

The statistical test performed will be the Signed-ranktest, which will incorporate the "shift" parameter of -0.9 log10 (the minimum clinically significant difference selected for this study). An evaluation of potential harm (increased viral load) will also be performed with the Signed ranktest; this test will examine the null hypothesis of no change in viral load (a shift of 0.0 log10 following IP administration) against the one-sided alternative

hypothesis that the viral load is increased following IP administration. Each efficacy test will be performed at the level α = 0.05. Each test for harm will be performed at level 2α = 0.10, in order to provide additional sensitivity to detect potential harm.

14.4 Analysis of Antiviral Activity and Dose De-escalation in Groups 3D-F

14.4.1 Sample Size

The sample size for antiviral activity will be 3-9 participants, depending on the MTD.

14.4.2 Null Hypothesis

As this is an exploratory proof of concept trial and analysis will be descriptive in this population, no formal null hypothesis will be tested.

14.4.3 Statistical Power and Analysis

No efficacy endpoints will be tested in Groups 3D-F as participants are HIV-infected with low viral loads at baseline (10^2 – $2x10^3$ copies/ml). Immunologic and virologic endpoints will be determined as described in Section 4.1. As soon as subgroup 3D has enrolled 3 participants, enrollment into subgroup 3E will be initiated. The same rule will apply for progression from subgroup 3E to 3F. In the event that dose de-escalation in subgroups 3A-C is stopped due to failure to meet virologic endpoints, dose de-escalation in subgroups 3D-F will continue.

14.5 Secondary and Exploratory Immunologic and Virologic Analyses

14.5.1 Sample Size

The sample size for secondary and exploratory immunologic and virologic analysis will be 63-93 participants.

14.5.2 Null Hypothesis

No formal hypothesis on immunologic or virologic responses will be tested, with the exception of the change in viral load described in Section 14.3.

14.5.3 Statistical Power and Analysis

Descriptive statistics (actual values and changes from reference) will be calculated for continuous immunologic and virologic parameters at all time points. Graphical representations of changes in parameters will be made as applicable. Differences between groups at a specific time point will be tested for exploratory purposes by a 2-sample t-test if the data appear to be normally distributed. If not, the non-parametric Wilcoxon rank sum test will be used. If portions of the measurements are censored below the assay quantification limit, the Gehan-Wilcoxon test will be employed. All statistical tests will be two-sided and will be considered statistically significant if $p < 0.05$.

Frequency tabulations will be calculated for discrete (qualitative) immunologic and virologic parameters at all time points. Significant differences between groups will be determined by a 2-sided Fisher's exact test.

Interim immunologic and virologic analyses of grouped data may be performed without unblinding the study to investigators or participants.

15.0 QUALITY CONTROL AND QUALITY ASSURANCE

To ensure the quality and reliability of the data collected and generated and the ethical conduct of this study, a Study Operations Manual (SOM) will be developed. All deviations will be reported and investigated. The SOM describes reporting and deviation documentation requirements and procedures.

Regular monitoring will be performed according to ICH-GCP as indicated in Section 17.5.

An independent audit of the study and study sites may be performed by the Sponsor or designee to establish the status of applicable quality systems. Inspection by regulatory authorities may also occur.

By signing the protocol, the Principal Investigators agree to facilitate study related monitoring, audits, IRB/IEC review and regulatory inspection(s) and direct access to source documents. Such information will be treated as strictly confidential and under no circumstances be made publicly available.

16.0 DATA AND BIOLOGICAL MATERIAL

All data and biological material collected through the study shall be managed in accordance with the Clinical Trial Agreement (CTA). Distribution and use of these data will be conducted by agreement of all parties.

The computerized raw data generated will be held by the DCC on behalf of the Sponsor. The study sites will also hold the final data files and tables generated for the purpose of analysis.

17.0 ADMINISTRATIVE STRUCTURE

The Principal Investigator will be responsible for all aspects of the study at the study site.

17.1 Protocol Safety Review Team

A PSRT will be formed to monitor the clinical safety data. During the administration of IP phase of the trial, the PSRT will review the clinical safety data on a weekly basis via electronic distribution of reports. An ad hoc PSRT review meeting will occur if any of the members of the PSRT requests a special review to discuss a specific safety issue or as specified in the Study Operations Manual. After the administration of IP phase the PSRT will review the clinical safety data at least monthly.

The PSRT will consist of the IAVI Medical Monitor(s), and the PI or designee from each clinical team. The study chair or an IAVI Medical Monitor may be the PSRT chair. *Ex officio* members will include the IAVI Chief Medical Officer and an unblinded IAVI Medical Monitor.

Additional PSRT participants may include the following, as needed:

- Co-investigators and trial site senior clinical research nursing staff
- Laboratory directors
- Data management, study statistician and regulatory staff

The PSRT membership and procedures are detailed in the PSRT charter.

17.2 Safety Monitoring Committee (SMC)

The SMC will consist of independent clinicians/scientists/statisticians/ethicists who are not involved in the study. Investigators responsible for the clinical care of participants or representative of the Sponsor may not be a member of the SMC. Details of membership, chair and co-chair and responsibilities are outlined in the SMC charter.

Principal Investigator(s) or designee and/or a Sponsor representative may be asked to join an open session of the SMC meeting to provide information on study conduct, present data or to respond to questions.

Safety data will be reviewed by the SMC at pre-specified time points and at an ad-hoc basis.

17.2.1 Content of Interim Safety Review

The SMC will be asked to review the following blinded data:

- Summary of reactogenicity (i.e., solicited adverse events)
- All adverse events judged by the Principal Investigator or designee to be possibly, probably or definitely related to IP
- All laboratory results confirmed on retest and judged by the Principal Investigator or designee to be clinically significant
- All SAEs

An unblinded presentation of all above noted events may also be made available for the SMC for their review if required by any member of the SMC.

17.2.2 SMC Review of Group 1 and 2 data prior to starting Group 3

Following IV infusion of IP of the last participant in Groups 1 and 2, the Safety Monitoring Committee (SMC) will review safety data through the day 14 post-IV infusion visit for all participants to confirm MTD in each group, and determine whether, and at what dose level, Group 3 can initiate enrollment.

17.3 Criteria for Pausing the Study

Enrollment and administration of IP will be stopped and a safety review conducted by the SMC for any of the following criteria:

1. One or more participants experience an SAE that is judged possibly, probably or definitely related to IP.
2. There is a participant death assessed as possibly, probably or definitely related to the IP.
3. Two or more participants experience Grade 3 adverse events in the same category System Organ Class that are considered to be at least possibly related to IP or

4. Any grade 4 adverse event that is considered to be at least possibly related to IP.

Table 2: AE notification and safety pause/AE review rules

Event and relationship to study product	Severity	Occurrence	Site PI action	PSRT or SMC action
SAE, related[1]	Any	Any	Phone, email or fax forms to sponsor within 24 hours	Study pause within 24 hours, refer to SMC for review
SAE, not related[2]	Grade 5	Any	Phone, email or fax forms to sponsor within 24 hours	PSRT review within 2 business days to consider pause
AE[3], related	Grade 3 or 4[4]	Second[5]	Phone, email or fax notification to sponsor within 24 hours	Study pause within 24 hours, refer to SMC for review
AE[3], related	Grade 3 or 4[4]	First	Phone, email or fax notification to sponsor within 24 hours	PSRT review within 2 business days to consider pause

[1] Related SAE refers to SAE deemed to be definitely, probably, or possibly related to study vaccine.

[2] Not related SAE refers to SAE deemed to be probably not related or not related to the study vaccine.

[3] Does not include the following reactogenicity symptoms (fever, fatigue/malaise, myalgia, arthralgia, chills, headache, nausea, vomiting).

[4] If no evidence of disease is present other than an abnormal laboratory value, the test must be repeated (entailing blood re-draw) at least one time. The verification period will be a maximum of 72 hours after initial awareness of the abnormal laboratory value. When signs and symptoms are present, repeat test will not be needed.

[5] PSRT will determine whether the reported related AE (Grade 3 or 4) is a second occurrence of a previously reported AE (Grade 3 or 4).

The Sponsor will request a review by the SMC, (or the SMC chair if other SMC members cannot be convened), to be held within 2 business days of the Sponsor learning of the event. The individual participant(s)/or study may be unblinded at the discretion of the SMC.

Following this review, the SMC will make a recommendation regarding the continuation or suspension of the administration of the IP or the trial and communicate this decision immediately to the Sponsor. The Sponsor then will inform the Principal Investigators without delay.

Additional *ad hoc* review may be specifically requested by the Sponsor, the Principal Investigator(s) or by the SMC.

17.4 Study Supervision

The SMC, the IAVI Chief Medical Officer (CMO) and the IAVI Medical Monitor(s) have access to progress report(s) of this study. Close cooperation will be necessary to track study progress, respond to queries about proper study implementation and management, address issues in a timely manner, and assure consistent documentation,

and share information effectively. Rates of accrual, retention, and other parameters relevant to the site's performance will be regularly and closely monitored by the study team.

17.5 Study Monitoring

On-and/or off-site monitoring will ensure that the study is conducted in compliance with human subjects' protection and other research regulations and guidelines, recorded and reported in accordance with the protocol, is consistent with SOPs, GCP, applicable regulatory requirements and locally accepted practices. The monitor will confirm the quality and accuracy of data at the site by validation of CRFs against the source documents, such as clinical records. The investigators, as well as participants through consenting to the study, agree that the monitor may inspect study facilities and source records (e.g., informed consent forms, clinic and laboratory records, other source documents), as well as observe the performance of study procedures (in accordance with site IRB requirements). Such information will be treated as strictly confidential and will under no circumstances be made publicly available.

The monitoring will adhere to GCP guidelines. The Principal Investigator will permit inspection of the facilities and all study-related documentation by authorized representatives of IAVI, and Government and Regulatory Authorities responsible for this study.

17.6 Investigator's Records

Study records include administrative documentation—e.g., reports and correspondence relating to the study—as well as documentation related to each participant screened and/or enrolled in the study—including informed consent forms, case report forms, and all other source documents. The investigator will maintain and store, in a secure manner, complete, accurate, and current study records for a minimum of 2 years after marketing application approval or the study is discontinued and applicable national and local health authorities are notified. IAVI will notify the Principal Investigator of these events.

18.0 INDEMNITY

The Sponsor and Institution are responsible to have appropriate liability insurance. For research-related injuries and/or medical problems determined to result from receiving the IP, treatment including necessary emergency treatment and proper follow-up care will be made available to the participant free of charge at the expense of the Sponsor.

19.0 PUBLICATION

A primary manuscript describing safety, anti-viral effect and immune responses in this trial will be prepared promptly after the data analysis is available.

Authors will be representatives of each trial site, the data management and statistical analysis center, the laboratories, the product developer and the sponsor, participant to the generally accepted criteria of contributions to the design and conduct of the study, the analysis of data and writing of the manuscript. Precedence will be given to authors from the site enrolling the

greatest number of participants. Manuscripts will be reviewed by representatives of each participating group as specified in the CTA**.**

20.0 ETHICAL CONSIDERATIONS

The Principal Investigator will ensure that the study is conducted in compliance with the protocol, SOPs in accordance with guidelines formulated by the ICH for GCP in clinical studies, the ethical principles that have their origins in the Declaration of Helsinki and applicable local standards and regulatory requirements.

APPENDIX A: SCHEDULE OF PROCEDURES

Study Month		0								1		2		3	4	5	6
**Study Week**		**0**				**1**		**2**	**3**	**4**	**6**	**8**	**10**	**12**	**16**	**20**	**24**
**Study Day**	**Scr**	**0**	**1**	**2**	**3**	**7**	**10[5]**	**14**	**21**	**28**	**42**	**56**	**70**	**84**	**112**	**140**	**168/ET[8]**
**Visit Windows (Days)**	**-42**	**0**	**0**	**0**	**0**	**± 1**	**0**	**± 2**	**± 2**	**± 2**	**± 3**	**± 3**	**±3**	**±7**	**±7**	**±7**	**±7**
**INVESTIGATIONAL PRODUCT**																	
Investigational Product /Placebo		X															
**CONSENT/ASSESSMENTS/COUNSELLING**																	
Informed Consent	X																
Assessment of Understanding	X																
HIV Risk Assessment[3]		X															X
HIV Risk Reduction Counselling[2]	X	X								X		X		X	X	X	X
HIV-test Counselling[3]	X	X															X
ART counseling[5]	X	X										X					X
Family Planning Counselling	X	X															
Social Impact Assessment																	X
**CLINICAL SAFETY ASSESSMENTS**																	
Comprehensive Medical History	X																
Interim Medical History		X	X	X	X	X	X	X	X	X	X	X					
Concomitant Medications	X	X	X	X	X	X	X	X	X	X	X	X					
General Physical Exam	X																X
Directed Physical Exam		X	X	X	X	X	X	X	X	X	X	X	X	X	X	X	
Weight	X	X															X
Height	X																
Vital Signs	X	X[4]	X	X	X	X	X	X	X	X	X	X	X	X	X	X	X
Local & Systemic Reactogenicity		X[4]	X	X	X												
Adverse Events		X	X	X	X	X	X	X	X	X	X	X					
Serious Adverse Events and pIMD	X	X	X	X	X	X	X	X	X	X	X	X	X	X	X	X	X

Study Month		0								1		2		3	4	5	6
**Study Week**		**0**				**1**		**2**	**3**	**4**	**6**	**8**	**10**	**12**	**16**	**20**	**24**
**Study Day**	**Scr**	**0**	**1**	**2**	**3**	**7**	**10[5]**	**14**	**21**	**28**	**42**	**56**	**70**	**84**	**112**	**140**	**168/ET[8]**
**Visit Windows (Days)**	**-42**	**0**	**0**	**0**	**0**	**± 1**	**0**	**± 2**	**± 2**	**± 2**	**± 3**	**± 3**	**±3**	**±7**	**±7**	**±7**	**±7**
**CLINICAL LABORATORY TESTS**																	
Hematology and Coagulation	X	X			X	X		X		X		X					X
CD4[1]	X	X				X		X		X		X					X
Clinical Chemistry	X	X			X	X		X		X		X					X
Urine Dipstick[10]	X	X			X	X		X		X		X					X
Urine Pregnancy test	X	X												X			X
Active Syphilis	X																
Hepatitis B	X																
Hepatitis C	X																
HIV diagnostic (4th generation Ag/Ab test)[3]	X	X															X
HIV Viral Load[1]	X	X	X	X	X	X	X	X	X	X	X	X	X	X	X	X	X
**RESEARCH LABORATORY TESTS**	-	-	-	-	-	-	-	-	-	-	-	-	-	-	-	-	-
HIV testing for PGT121 susceptibility[11,5]	X																
HIV SGA sequencing[12]		X								X							X
HIV genotypic testing for ART resistance[12]		X								X							X
HIV reservoir size assessment[1]	X					X				X							
Humoral Assays[6]		X			X	X		X		X		X		X			X
Cellular Assays[6]		X				X		X		X		X		X			X
HLA typing		X															
**PHARMACOKINETICS PGT121 ELISA**	X[7]	X[7]	X	X	X	X		X	X	X	X	X	X	X	X	X	X
**MUCOSAL SAMPLING[9]**		X[9]	X			X		X									
**PLASMA/SERUM STORAGE**	X	X	X	X	X	X	X	X	X	X		X		X			X
**PBMCs STORAGE**		X				X		X		X		X		X			X

1. For groups 2 and 3 only
2. Group 1: will receive HIV risk reduction counselling; Groups 2 and 3: HIV risk reduction counselling as secondary prevention to reduce onward transmission
3. Group 1 only
4. At baseline, approximately 30 minutes after IP administration start, and at hours 1 through 12 after IV infusion start. Local and systemic reactogenicity will be assessed by clinic staff at visits on study days 1, 2 and 3. Local and Systemic reactogenicity will also be assessed by the participant using the Memory Aid on study days 1, 2, and 3
5. Group 3 only
6. See Laboratory Analytical Plan for details
7. Day 0 PK draws done immediately before IP administration, at the end of the IV infusion of IP, and 30 minutes and 3 hours post end of the IP administration. Additional PK draws on day 0 are done 6, 9 and 12 hours after the start of the IV infusion of IP. The screening sample is not a PK assessment per se, the PGT121 ELISA will be done to exclude autologous PGT121-like antibody levels above the cut-off. See SOM for details
8. Early Termination (ET): Procedures to be performed at ET are the same as last visit procedures
9. Cervico-vaginal and/or rectal mucosal sampling (optional) on Day 0 must be done prior to IV infusion of IP. See SOM for details
10. Urinalysis for group 3 will only be conducted at visits after screening if clinically indicated.
11. Baseline assessment of participants autologous HIV for neutralization susceptibility to PGT121 in-vitro (group 3 only).
12. HIV envelope single genome amplification (SGA) sequencing and ART resistance genotyping will be performed in all subjects of group 3 and in subjects of group 2 only if they develop detectable plasma viremia >1000 HIV cp/ml during the study.

APPENDIX B: LOW RISK CRITERIA

Low risk will be defined as:

1. **SEXUAL BEHAVIORS**

   In the **last 12 months** did not:

   - Have oral, vaginal or anal intercourse with an HIV-infected partner, or partner who uses injection drugs.
   - Gave or receive money, drugs, gifts, or services in exchange for oral, vaginal or anal sex

   AND

   In the **last 6 months** has abstained from penile/anal or penile/vaginal intercourse OR

   In the **last 6 months**:

   - Had 4 or fewer partners of the opposite birth sex for vaginal and/or anal intercourse, OR

   Is MSM (person born male with partner(s) born male) who, in the **last 12 months**:

   - Had 2 or fewer MSM partners for anal intercourse and had no unprotected anal sex with MSM, OR
   - Had unprotected anal intercourse with only 1 MSM partner, within a monogamous relationship lasting at least 12 months (during which neither partner had any other partners). If the monogamous relationship ended, the participant may then have had protected anal intercourse with 1 other MSM partner (total 2 or fewer partners in the last 12 months).

   Is a transgender person, regardless of the point on the transition spectrum, having sex with men (born male) and/or other transgender persons, who in the **last 12 months**:

   - Had 2 or fewer partners for anal or vaginal intercourse, and had no unprotected anal or vaginal sex, OR
   - Had unprotected anal or vaginal intercourse sex with 1 partner only within a monogamous relationship lasting at least 12 months (during which neither partner had any other partners). If the monogamous relationship ended, may then have had protected anal or vaginal sex with one other partner (total 2 or fewer partners in the last 12 months).

   AND

   Uses or intends to use condoms in situations which may include penile/anal or penile/vaginal intercourse with new partners of unknown HIV status, occasional partners, partners outside a primary relationship, and/or partners known to have other partners.

2. **NON-SEXUAL BEHAVIORS**

   In the **last 12 months** did not:

   - Inject drugs or other substances without a prescription

- Use cocaine, methamphetamine, or excessive alcohol, which in the investigator's judgement, rendered the participant at greater than low risk for acquiring HIV infection

The investigator's judgement should consider local epidemiologic information about HIV prevalence in the area and community networks.

A participant is NOT appropriate for inclusion if he/she:

Acquired an STI (ie, new infection) in the last 12 months:

- Syphilis
- Gonorrhea
- Non-gonococcal urethritis
- HSV-2
- Chlamydia
- Pelvic inflammatory disease (PID)
- Trichomonas
- Mucopurulent cervicitis
- Epididymitis
- Proctitis
- Lymphogranuloma venereum
- Chancroid
- Hepatitis B

APPENDIX C: ADVERSE EVENT SEVERITY ASSESSMENT TABLE

Adapted from: DIVISION OF AIDS (DAIDS) TABLE FOR GRADING THE SEVERITY OF ADULT AND PEDIATRIC ADVERSE EVENTS Version 2.0, November 2014

The *Division of AIDS* (*DAIDS) Table for Grading the Severity of Adult and Pediatric Adverse Events, Version 2.0* consists of parameters, or AEs, with severity grading guidance that are to be used in DAIDS clinical trials for safety data reporting to maintain accuracy and consistency in the evaluation of AEs.

I. Instructions and Clarifications

Grading Adult and Pediatric AEs

When a single parameter is not appropriate for grading an AE in both adult and pediatric populations, separate parameters with specified age ranges are provided. If no distinction between adult and pediatric populations has been made, the listed parameter should be used for grading an AE in both populations.

Note: The term "severe" is not the same as "serious" in classifying AEs. The severity of a specific event describes its intensity, and it is the intensity which is graded. Seriousness, which is not graded, relates to an outcome of an AE and is a regulatory definition.

Estimating Severity Grade for Parameters Not Identified in the Table

The functional table below should be used to grade the severity of an AE that is not specifically identified in the grading table. In addition, all deaths related to an AE are to be classified as grade 5.

PARAMETER	GRADE 1 MILD	GRADE 2 MODERATE	GRADE 3 SEVERE	GRADE 4 POTENTIALLY LIFE-THREATENING
**Clinical** adverse event **NOT** identified elsewhere in the grading table	Mild symptoms causing no or minimal interference with usual social & functional activities with intervention not indicated	Moderate symptoms causing greater than minimal interference with usual social & functional activities with intervention indicated	Severe symptoms causing inability to perform usual social & functional activities with intervention or hospitalization indicated	Potentially life-threatening symptoms causing inability to perform basic self-care functions with intervention indicated to prevent permanent impairment, persistent disability, or death

Determining Severity Grade for Parameters "Between Grades"

If the severity of an AE could fall in either one of two grades (e.g., the severity of an AE could be either Grade 2 or Grade 3), sites should select the higher of the two grades.

Laboratory Values

General. An asymptomatic, abnormal laboratory finding without an accompanying AE should not be reported to DAIDS in an expedited timeframe unless it meets protocol-specific reporting requirements. Sites should refer to the applicable network standards for reporting abnormal laboratory findings on the CRFs.

Values Below Grade 1

Any laboratory value that is between the ULN and grade 1 (for high values) or the LLN and grade 1 (for low values) should not be graded or reported as an AE. Sites should consult the Manual for Expedited Reporting of Adverse Events to DAIDS, Version 2.0 and their protocol when making an assessment of the need to report an AE.

Overlap of Local Laboratory Normal Values with Grading Table Ranges.

When local laboratory normal values fall within grading table laboratory ranges, the severity grading is based on the ranges in the grading table unless there is a protocol-specific grading criterion for the laboratory value. For example, “Magnesium, Low" has a grade 1 range of 1.2 to < 1.4 mEq/L, while a particular laboratory’s normal range for magnesium may be 1.3 to 2.8 mEq/L. If a study participant’s magnesium laboratory value is 1.3 mEq/L, the laboratory value should be graded as grade 1.

II. Definitions of terms used in the Table:

Basic Self-care Functions	Adult<br>Activities such as bathing, dressing, toileting, transfer or movement, continence, and feeding.<br><br>Young Children<br>Activities that are age and culturally appropriate, such as feeding one’s self with culturally appropriate eating implements.
LLN	Lower limit of normal
Intervention	Medical, surgical, or other procedures recommended or provided by a healthcare professional for the treatment of an adverse event.
NA	Not Applicable
ULN	Upper limit of normal
Usual Social & Functional Activities	Activities which adults and children perform on a routine basis and those which are part of regular activities of daily living, for example:<br><br>Adults<br>Adaptive tasks and desirable activities, such as going to work, shopping, cooking, use of transportation, or pursuing a hobby.<br><br>Young Children<br>Activities that are age and culturally appropriate, such as social interactions, play activities, or learning tasks.

Major Clinical Conditions

Cardiovascular

PARAMETER	GRADE 1 MILD	GRADE 2 MODERATE	GRADE 3 SEVERE	GRADE 4 POTENTIALLY LIFE-THREATENING
**Arrhythmia** (by ECG or physical examination) *Specify type, if applicable*	No symptoms AND No intervention indicated	No symptoms AND Non-urgent intervention indicated	Non-life-threatening symptoms AND Non-urgent intervention indicated	Life-threatening arrhythmia OR Urgent intervention indicated
**Blood Pressure Abnormalities**[1] ***Hypertension*** *(with the lowest reading taken after repeat testing during a visit)* *≥ 18 years of age*	140 to < 160 mmHg systolic OR 90 to < 100 mmHg diastolic	≥ 160 to < 180 mmHg systolic OR ≥ 100 to < 110 mmHg diastolic	≥ 180 mmHg systolic OR ≥ 110 mmHg diastolic	Life-threatening consequences in a participant not previously diagnosed with hypertension (e.g., malignant hypertension) OR Hospitalization indicated
< 18 years of age	> 120/80 mmHg	≥ 95th to < 99th percentile + 5 mmHg adjusted for age, height, and gender (systolic and/or diastolic)	≥ 99th percentile + 5 mmHg adjusted for age, height, and gender (systolic and/or diastolic)	Life-threatening consequences in a participant not previously diagnosed with hypertension (e.g., malignant hypertension) OR Hospitalization indicated
Hypotension	No symptoms	Symptoms corrected with oral fluid replacement	Symptoms AND IV fluids indicated	Shock requiring use of vasopressors or mechanical assistance to maintain blood pressure
**Cardiac Ischemia or Infarction** *Report only one*	NA	NA	New symptoms with ischemia (stable angina) OR New testing consistent with ischemia	Unstable angina OR Acute myocardial infarction
**Heart Failure**	No symptoms AND Laboratory or cardiac imaging abnormalities	Symptoms with mild to moderate activity or exertion	Symptoms at rest or with minimal activity or exertion (e.g., hypoxemia) OR Intervention indicated (e.g., oxygen)	Life-threatening consequences OR Urgent intervention indicated (e.g., vasoactive medications, ventricular assist device, heart transplant)
**Hemorrhage** (with significant acute blood loss)	NA	Symptoms AND No transfusion indicated	Symptoms AND Transfusion of ≤ 2 units packed RBCs indicated	Life-threatening hypotension OR Transfusion of > 2 units packed RBCs (for children, packed RBCs > 10 cc/kg) indicated

PARAMETER	GRADE 1 MILD	GRADE 2 MODERATE	GRADE 3 SEVERE	GRADE 4 POTENTIALLY LIFE-THREATENING
**Prolonged PR Interval or AV Block** *Report only one* *> 16 years of age*	PR interval 0.21 to < 0.25 seconds	PR interval ≥ 0.25 seconds OR Type I 2nd degree AV block	Type II 2nd degree AV block OR Ventricular pause ≥ 3.0 seconds	Complete AV block
≤ 16 years of age	1st degree AV block (PR interval > normal for age and rate)	Type I 2nd degree AV block	Type II 2nd degree AV block OR Ventricular pause ≥ 3.0 seconds	Complete AV block
**Prolonged QTc Interval[2]**	0.45 to 0.47 seconds	> 0.47 to 0.50 seconds	> 0.50 seconds OR ≥ 0.06 seconds above baseline	Life-threatening consequences (e.g., Torsade de pointes, other associated serious ventricular dysrhythmia)
**Thrombosis or Embolism** *Report only one*	NA	Symptoms AND No intervention indicated	Symptoms AND Intervention indicated	Life-threatening embolic event (e.g., pulmonary embolism, thrombus)

[1] Blood pressure norms for children < 18 years of age can be found in: Expert Panel on Integrated Guidelines for Cardiovascular Health and Risk Reduction in Children and Adolescents. *Pediatrics* 2011;128;S213; originally published online November 14, 2011; DOI: 10.1542/peds.2009-2107C

[2] As per Bazett's form

Dermatologic

PARAMETER	GRADE 1 MILD	GRADE 2 MODERATE	GRADE 3 SEVERE	GRADE 4 POTENTIALLY LIFE-THREATENING
**Alopecia** (scalp only)	Detectable by study participant, caregiver, or physician AND<br>Causing no or minimal interference with usual social & functional activities	Obvious on visual inspection AND<br>Causing greater than minimal interference with usual social & functional activities	NA	NA
**Bruising**	Localized to one area	Localized to more than one area	Generalized	NA
**Cellulitis**	NA	Non-parenteral treatment indicated (e.g., oral antibiotics, antifungals, antivirals)	IV treatment indicated (e.g., IV antibiotics, antifungals, antivirals)	Life-threatening consequences (e.g., sepsis, tissue necrosis)
**Hyperpigmentation**	Slight or localized causing no or minimal interference with usual social & functional activities	Marked or generalized causing greater than minimal interference with usual social & functional activities	NA	NA
**Hypopigmentation**	Slight or localized causing no or minimal interference with usual social & functional activities	Marked or generalized causing greater than minimal interference with usual social & functional activities	NA	NA
**Petechiae**	Localized to one area	Localized to more than one area	Generalized	NA
**Pruritus**[3] (without skin lesions)	Itching causing no or minimal interference with usual social & functional activities	Itching causing greater than minimal interference with usual social & functional activities	Itching causing inability to perform usual social & functional activities	NA
**Rash**<br>*Specify type, if applicable*	Localized rash	Diffuse rash OR<br>Target lesions	Diffuse rash AND<br>Vesicles or limited number of bullae or superficial ulcerations of mucous membrane limited to one site	Extensive or generalized bullous lesions OR<br>Ulceration of mucous membrane involving two or more distinct mucosal sites OR Stevens-<br>Johnson syndrome OR<br>Toxic epidermal necrolysis

[3] For pruritus associated with injections or infusions, see the *Site Reactions to Injections and Infusions* section (page 23).

Endocrine and Metabolic

PARAMETER	GRADE 1 MILD	GRADE 2 MODERATE	GRADE 3 SEVERE	GRADE 4 POTENTIALLY LIFE-THREATENING
**Diabetes Mellitus**	Controlled without medication	Controlled with medication OR Modification of current medication regimen	Uncontrolled despite treatment modification OR Hospitalization for immediate glucose control indicated	Life-threatening consequences (e.g., ketoacidosis, hyperosmolar non-ketotic coma, end organ failure)
**Gynecomastia**	Detectable by study participant, caregiver, or physician AND Causing no or minimal interference with usual social & functional activities	Obvious on visual inspection AND Causing pain with greater than minimal interference with usual social & functional activities	Disfiguring changes AND Symptoms requiring intervention or causing inability to perform usual social & functional activities	NA
**Hyperthyroidism**	No symptoms AND Abnormal laboratory value	Symptoms causing greater than minimal interference with usual social & functional activities OR Thyroid suppression therapy indicated	Symptoms causing inability to perform usual social & functional activities OR Uncontrolled despite treatment modification	Life-threatening consequences (e.g., thyroid storm)
**Hypothyroidism**	No symptoms AND Abnormal laboratory value	Symptoms causing greater than minimal interference with usual social & functional activities OR Thyroid replacement therapy indicated	Symptoms causing inability to perform usual social & functional activities OR Uncontrolled despite treatment modification	Life-threatening consequences (e.g., myxedema coma)
**Lipoatrophy**[4]	Detectable by study participant, caregiver, or physician AND Causing no or minimal interference with usual social & functional activities	Obvious on visual inspection AND Causing greater than minimal interference with usual social & functional activities	Disfiguring changes	NA
**Lipohypertrophy**[5]	Detectable by study participant, caregiver, or physician AND Causing no or minimal interference with usual social & functional activities	Obvious on visual inspection AND Causing greater than minimal interference with usual social & functional activities	Disfiguring changes	NA

[4] Definition: A disorder characterized by fat loss in the face, extremities, and buttocks.
[5] Definition: A disorder characterized by abnormal fat accumulation on the back of the neck, breasts, and abdomen.

Gastro-intestinal

PARAMETER	GRADE 1 MILD	GRADE 2 MODERATE	GRADE 3 SEVERE	GRADE 4 POTENTIALLY LIFE-THREATENING
**Anorexia**	Loss of appetite without decreased oral intake	Loss of appetite associated with decreased oral intake without significant weight loss	Loss of appetite associated with significant weight loss	Life-threatening consequences OR Aggressive intervention indicated (e.g., tube feeding, total parenteral nutrition)
**Ascites**	No symptoms	Symptoms AND Intervention indicated (e.g., diuretics, therapeutic paracentesis)	Symptoms recur or persist despite intervention	Life-threatening consequences
**Bloating or Distension**<br>*Report only one*	Symptoms causing no or minimal interference with usual social & functional activities	Symptoms causing greater than minimal interference with usual social & functional activities	Symptoms causing inability to perform usual social & functional activities	NA
**Cholecystitis**	NA	Symptoms AND Medical intervention indicated	Radiologic, endoscopic, or operative intervention indicated	Life-threatening consequences (e.g., sepsis, perforation)
**Constipation**	NA	Persistent constipation requiring regular use of dietary modifications, laxatives, or enemas	Obstipation with manual evacuation indicated	Life-threatening consequences (e.g., obstruction)
**Diarrhea**<br>*≥ 1 year of age*	Transient or intermittent episodes of unformed stools OR Increase of ≤ 3 stools over baseline per 24-hour period	Persistent episodes of unformed to watery stools OR Increase of 4 to 6 stools over baseline per 24-hour period	Increase of ≥ 7 stools per 24-hour period OR IV fluid replacement indicated	Life-threatening consequences (e.g., hypotensive shock)
< 1 year of age	Liquid stools (more unformed than usual) but usual number of stools	Liquid stools with increased number of stools OR Mild dehydration	Liquid stools with moderate dehydration	Life-threatening consequences (e.g., liquid stools resulting in severe dehydration, hypotensive shock)
**Dysphagia or Odynophagia**<br>*Report only one and specify location*	Symptoms but able to eat usual diet	Symptoms causing altered dietary intake with no intervention indicated	Symptoms causing severely altered dietary intake with intervention indicated	Life-threatening reduction in oral intake
**Gastrointestinal Bleeding**	Not requiring intervention other than iron supplement	Endoscopic intervention indicated	Transfusion indicated	Life-threatening consequences (e.g., hypotensive shock)

PARAMETER	GRADE 1 MILD	GRADE 2 MODERATE	GRADE 3 SEVERE	GRADE 4 POTENTIALLY LIFE-THREATENING
**Mucositis or Stomatitis** *Report only one and specify location*	Mucosal erythema	Patchy pseudomembranes or ulcerations	Confluent pseudomembranes or ulcerations OR Mucosal bleeding with minor trauma	Life-threatening consequences (e.g., aspiration, choking) OR Tissue necrosis OR Diffuse spontaneous mucosal bleeding
**Nausea**	Transient (< 24 hours) or intermittent AND No or minimal interference with oral intake	Persistent nausea resulting in decreased oral intake for 24 to 48 hours	Persistent nausea resulting in minimal oral intake for > 48 hours OR Rehydration indicated (e.g., IV fluids)	Life-threatening consequences (e.g., hypotensive shock)
**Pancreatitis**	NA	Symptoms with hospitalization not indicated	Symptoms with hospitalization indicated	Life-threatening consequences (e.g., circulatory failure, hemorrhage, sepsis)
**Perforation** (colon or rectum)	NA	NA	Intervention indicated	Life-threatening consequences
**Proctitis**	Rectal discomfort with no intervention indicated	Symptoms causing greater than minimal interference with usual social & functional activities OR Medical intervention indicated	Symptoms causing inability to perform usual social & functional activities OR Operative intervention indicated	Life-threatening consequences (e.g., perforation)
**Rectal Discharge**	Visible discharge	Discharge requiring the use of pads	NA	NA
**Vomiting**	Transient or intermittent AND No or minimal interference with oral intake	Frequent episodes with no or mild dehydration	Persistent vomiting resulting in orthostatic hypotension OR Aggressive rehydration indicated (e.g., IV fluids)	Life-threatening consequences (e.g., hypotensive shock)

Musculoskeletal

PARAMETER	GRADE 1 MILD	GRADE 2 MODERATE	GRADE 3 SEVERE	GRADE 4 POTENTIALLY LIFE-THREATENING
**Arthralgia**	Joint pain causing no or minimal interference with usual social & functional activities	Joint pain causing greater than minimal interference with usual social & functional activities	Joint pain causing inability to perform usual social & functional activities	Disabling joint pain causing inability to perform basic self-care functions
**Arthritis**	Stiffness or joint swelling causing no or minimal interference with usual social & functional activities	Stiffness or joint swelling causing greater than minimal interference with usual social & functional activities	Stiffness or joint swelling causing inability to perform usual social & functional activities	Disabling joint stiffness or swelling causing inability to perform basic self-care functions
**Myalgia** (generalized)	Muscle pain causing no or minimal interference with usual social & functional activities	Muscle pain causing greater than minimal interference with usual social & functional activities	Muscle pain causing inability to perform usual social & functional activities	Disabling muscle pain causing inability to perform basic self-care functions
**Osteonecrosis**	NA	No symptoms but with radiographic findings AND No operative intervention indicated	Bone pain with radiographic findings OR Operative intervention indicated	Disabling bone pain with radiographic findings causing inability to perform basic self-care functions
**Osteopenia**[6] *> 30 years of age*	BMD t-score -2.5 to -1	NA	NA	NA
< 30 years of age	BMD z-score -2 to -1	NA	NA	NA
**Osteoporosis**[6] *> 30 years of age*	NA	BMD t-score < -2.5	Pathologic fracture (e.g., compression fracture causing loss of vertebral height)	Pathologic fracture causing life-threatening consequences
< 30 years of age	NA	BMD z-score < -2	Pathologic fracture (e.g., compression fracture causing loss of vertebral height)	Pathologic fracture causing life-threatening consequences

6 BMD t and z scores can be found in: Kanis JA on behalf of the World Health Organization Scientific Group (2007). Assessment of osteoporosis at the primary health-care level. Technical Report. World Health Organization Collaborating Centre for Metabolic Bone Diseases, University of Sheffield, UK. 2007: Printed by the University of Sheffield.

Neurologic

PARAMETER	GRADE 1 MILD	GRADE 2 MODERATE	GRADE 3 SEVERE	GRADE 4 POTENTIALLY LIFE-THREATENING
**Acute CNS Ischemia**	NA	NA	Transient ischemic attack	Cerebral vascular accident (e.g., stroke with neurological deficit)
**Altered Mental Status** (for Dementia, see *Cognitive, Behavioral, or Attentional Disturbance* below)	Changes causing no or minimal interference with usual social & functional activities	Mild lethargy or somnolence causing greater than minimal interference with usual social & functional activities	Confusion, memory impairment, lethargy, or somnolence causing inability to perform usual social & functional activities	Delirium OR Obtundation OR Coma
**Ataxia**	Symptoms causing no or minimal interference with usual social & functional activities OR No symptoms with ataxia detected on examination	Symptoms causing greater than minimal interference with usual social & functional activities	Symptoms causing inability to perform usual social & functional activities	Disabling symptoms causing inability to perform basic self-care functions
**Cognitive, Behavioral, or Attentional Disturbance** (includes dementia and attention deficit disorder) *Specify type, if applicable*	Disability causing no or minimal interference with usual social & functional activities OR Specialized resources not indicated	Disability causing greater than minimal interference with usual social & functional activities OR Specialized resources on part-time basis indicated	Disability causing inability to perform usual social & functional activities OR Specialized resources on a full-time basis indicated	Disability causing inability to perform basic self-care functions OR Institutionalization indicated
**Developmental Delay** *< 18 years of age* *Specify type, if applicable*	Mild developmental delay, either motor or cognitive, as determined by comparison with a developmental screening tool appropriate for the setting	Moderate developmental delay, either motor or cognitive, as determined by comparison with a developmental screening tool appropriate for the setting	Severe developmental delay, either motor or cognitive, as determined by comparison with a developmental screening tool appropriate for the setting	Developmental regression, either motor or cognitive, as determined by comparison with a developmental screening tool appropriate for the setting
**Headache**	Symptoms causing no or minimal interference with usual social & functional activities	Symptoms causing greater than minimal interference with usual social & functional activities	Symptoms causing inability to perform usual social & functional activities	Symptoms causing inability to perform basic self-care functions OR Hospitalization indicated OR Headache with significant impairment of alertness or other neurologic function

PARAMETER	GRADE 1 MILD	GRADE 2 MODERATE	GRADE 3 SEVERE	GRADE 4 POTENTIALLY LIFE-THREATENING
**Neuromuscular Weakness** (includes myopathy and neuropathy) *Specify type, if applicable*	Minimal muscle weakness causing no or minimal interference with usual social & functional activities OR No symptoms with decreased strength on examination	Muscle weakness causing greater than minimal interference with usual social & functional activities	Muscle weakness causing inability to perform usual social & functional activities	Disabling muscle weakness causing inability to perform basic self-care functions OR Respiratory muscle weakness impairing ventilation
**Neurosensory Alteration** (includes paresthesia and painful neuropathy) *Specify type, if applicable*	Minimal paresthesia causing no or minimal interference with usual social & functional activities OR No symptoms with sensory alteration on examination	Sensory alteration or paresthesia causing greater than minimal interference with usual social & functional activities	Sensory alteration or paresthesia causing inability to perform usual social & functional activities	Disabling sensory alteration or paresthesia causing inability to perform basic self-care functions
**Seizures** ***New Onset Seizure*** *≥ 18 years of age*	NA	NA	1 to 3 seizures	Prolonged and repetitive seizures (e.g., status epilepticus) OR Difficult to control (e.g., refractory epilepsy)
< 18 years of age (includes new or pre-existing febrile seizures)	Seizure lasting < 5 minutes with < 24 hours postictal state	Seizure lasting 5 to < 20 minutes with < 24 hours postictal state	Seizure lasting ≥ 20 minutes OR > 24 hours postictal state	Prolonged and repetitive seizures (e.g., status epilepticus) OR Difficult to control (e.g., refractory epilepsy)
Pre-existing Seizure	NA	Increased frequency from previous level of control without change in seizure character	Change in seizure character either in duration or quality (e.g., severity or focality)	Prolonged and repetitive seizures (e.g., status epilepticus) OR Difficult to control (e.g., refractory epilepsy)
**Syncope**	Near syncope without loss of consciousness (e.g., pre-syncope)	Loss of consciousness with no intervention indicated	Loss of consciousness AND Hospitalization or intervention required	NA

Pregnancy, Puerperium, and Perinatal

PARAMETER	GRADE 1 MILD	GRADE 2 MODERATE	GRADE 3 SEVERE	GRADE 4 POTENTIALLY LIFE-THREATENING
**Fetal Death or Stillbirth** (report using mother's participant ID) *Report only one*	NA	NA	Fetal loss occurring at > 20 weeks gestation	NA
**Preterm Delivery**[7] (report using mother's participant ID)	Delivery at 34 to < 37 weeks gestational age	Delivery at 28 to < 34 weeks gestational age	Delivery at 24 to < 28 weeks gestational age	Delivery at < 24 weeks gestational age
**Spontaneous Abortion or Miscarriage**[8] (report using mother's participant ID) *Report only one*	Chemical pregnancy	Uncomplicated spontaneous abortion or miscarriage	Complicated spontaneous abortion or miscarriage	NA

[7] Definition: A delivery of a live-born neonate occurring at > 20 to < 37 weeks gestational age.
[8] Definition: A clinically recognized pregnancy occurring at < 20 weeks gestational age.

Psychiatric

PARAMETER	GRADE 1 MILD	GRADE 2 MODERATE	GRADE 3 SEVERE	GRADE 4 POTENTIALLY LIFE-THREATENING
**Insomnia**	Mild difficulty falling asleep, staying asleep, or waking up early	Moderate difficulty falling asleep, staying asleep, or waking up early	Severe difficulty falling asleep, staying asleep, or waking up early	NA
**Psychiatric Disorders** (includes anxiety, depression, mania, and psychosis) *Specify disorder*	Symptoms with intervention not indicated OR Behavior causing no or minimal interference with usual social & functional activities	Symptoms with intervention indicated OR Behavior causing greater than minimal interference with usual social & functional activities	Symptoms with hospitalization indicated OR Behavior causing inability to perform usual social & functional activities	Threatens harm to self or others OR Acute psychosis OR Behavior causing inability to perform basic self-care functions
**Suicidal Ideation or Attempt** *Report only one*	Preoccupied with thoughts of death AND No wish to kill oneself	Preoccupied with thoughts of death AND Wish to kill oneself with no specific plan or intent	Thoughts of killing oneself with partial or complete plans but no attempt to do so OR Hospitalization indicated	Suicide attempted

Respiratory

PARAMETER	GRADE 1 MILD	GRADE 2 MODERATE	GRADE 3 SEVERE	GRADE 4 POTENTIALLY LIFE-THREATENING
**Acute Bronchospasm**	Forced expiratory volume in 1 second or peak flow reduced to ≥ 70 to < 80% OR Mild symptoms with intervention not indicated	Forced expiratory volume in 1 second or peak flow 50 to < 70% OR Symptoms with intervention indicated OR Symptoms causing greater than minimal interference with usual social & functional activities	Forced expiratory volume in 1 second or peak flow 25 to < 50% OR Symptoms causing inability to perform usual social & functional activities	Forced expiratory volume in 1 second or peak flow < 25% OR Life-threatening respiratory or hemodynamic compromise OR Intubation
**Dyspnea or Respiratory Distress** *Report only one*	Dyspnea on exertion with no or minimal interference with usual social functional activities OR Wheezing OR Minimal increase in respiratory rate for age	Dyspnea on exertion causing greater than minimal interference with usual social & functional activities OR Nasal flaring OR Intercostal retractions OR Pulse oximetry 90 to < 95%	Dyspnea at rest causing inability to perform usual social & functional activities OR Pulse oximetry < 90%	Respiratory failure with ventilator support indicated (e.g., CPAP, BPAP, intubation)

Sensory

PARAMETER	GRADE 1 MILD	GRADE 2 MODERATE	GRADE 3 SEVERE	GRADE 4 POTENTIALLY LIFE-THREATENING
**Hearing Loss** *≥ 12 years of age*	NA	Hearing aid or intervention not indicated	Hearing aid or intervention indicated	Profound bilateral hearing loss (> 80 dB at 2 kHz and above) OR Non-serviceable hearing (i.e., >50 dB audiogram and <50% speech discrimination)
< 12 years of age (based on a 1, 2, 3, 4, 6 and 8 kHz audiogram)	> 20 dB hearing loss at ≤ 4 kHz	> 20 dB hearing loss at > 4 kHz	> 20 dB hearing loss at ≥ 3 kHz in one ear with additional speech language related services indicated (where available) OR Hearing loss sufficient to indicate therapeutic intervention, including hearing aids	Audiologic indication for cochlear implant and additional speech-language related services indicated (where available)

**Tinnitus**	Symptoms causing no or minimal interference with usual social & functional activities with intervention not indicated	Symptoms causing greater than minimal interference with usual social & functional activities with intervention indicated	Symptoms causing inability to perform usual social & functional activities	NA
**Uveitis**	No symptoms AND Detectable on examination	Anterior uveitis with symptoms OR Medicamylasal intervention indicated	Posterior or pan-uveitis OR Operative intervention indicated	Disabling visual loss in affected eye(s)
**Vertigo**	Vertigo causing no or minimal interference with usual social & functional activities	Vertigo causing greater than minimal interference with usual social & functional activities	Vertigo causing inability to perform usual social & functional activities	Disabling vertigo causing inability to perform basic self-care functions
**Visual Changes** (assessed from baseline)	Visual changes causing no or minimal interference with usual social & functional activities	Visual changes causing greater than minimal interference with usual social & functional activities	Visual changes causing inability to perform usual social & functional activities	Disabling visual loss in affected eye(s)

Systemic

PARAMETER	GRADE 1 MILD	GRADE 2 MODERATE	GRADE 3 SEVERE	GRADE 4 POTENTIALLY LIFE-THREATENING
**Acute Allergic Reaction**	Localized urticaria (wheals) with no medical intervention indicated	Localized urticaria with intervention indicated OR Mild angioedema with no intervention indicated	Generalized urticaria OR Angioedema with intervention indicated OR Symptoms of mild bronchospasm	Acute anaphylaxis OR Life-threatening bronchospasm OR Laryngeal edema
**Chills**	Symptoms causing no or minimal interference with usual social & functional activities	Symptoms causing greater than minimal interference with usual social & functional activities	Symptoms causing inability to perform usual social & functional activities	NA
**Cytokine Release Syndrome**[9]	Mild signs and symptoms AND Therapy (i.e., antibody infusion) interruption not indicated	Therapy (i.e., antibody infusion) interruption indicated AND Responds promptly to symptomatic treatment OR Prophylactic medications indicated for ≤ 24 hours	Prolonged severe signs and symptoms OR Recurrence of symptoms following initial improvement	Life-threatening consequences (e.g., requiring pressor or ventilator support)
**Fatigue or Malaise** *Report only one*	Symptoms causing no or minimal interference with usual social & functional activities	Symptoms causing greater than minimal interference with usual social & functional activities	Symptoms causing inability to perform usual social & functional activities	Incapacitating symptoms of fatigue or malaise causing inability to perform basic self-care functions
**Fever** (non-axillary temperatures only)	38.0 to < 38.6°C or 100.4 to < 101.5°F	≥ 38.6 to < 39.3°C or > 101.5 to < 102.7°F	≥ 39.3 to < 40.0°C or > 102.7 to < 104.0°F	≥ 40.0°C or > 104.0°F
**Pain**[10] (not associated with study agent	Pain causing no or minimal interference	Pain causing greater than minimal	Pain causing inability to perform usual social	Disabling pain causing inability to perform basic self-care functions OR

injections and not specified elsewhere)<br>*Specify location*	with usual social & functional activities	interference with usual social & functional activities	& functional activities	Hospitalization indicated
**Serum Sickness**[11]	Mild signs and symptoms	Moderate signs and symptoms AND<br>Intervention indicated (e.g., antihistamines)	Severe signs and symptoms AND<br>Higher level intervention indicated (e.g., steroids or IV fluids)	Life-threatening consequences (e.g.,

[9] Definition: A disorder characterized by nausea, headache, tachycardia, hypotension, rash, and/or shortness of breath.
[10] For pain associated with injections or infusions, see the *Site Reactions to Injections and Infusions* section (page 23).
[11] Definition: A disorder characterized by fever, arthralgia, myalgia, skin eruptions, lymphadenopathy, marked discomfort, and/or dyspnea.

PARAMETER	GRADE 1 MILD	GRADE 2 MODERATE	GRADE 3 SEVERE	GRADE 4 POTENTIALLY LIFE-THREATENING
**Underweight**[12]<br>*> 5 to 19 years of age*	NA	WHO BMI z-score < -2 to < -3	WHO BMI z-score < -3	WHO BMI z-score < -3 with life-threatening consequences
2 to 5 years of age	NA	WHO Weight-for-height z-score < -2 to < -3	WHO Weight-for-height z-score < -3	WHO Weight-for-height z-score < -3 with life-threatening consequences
< 2 years of age	NA	WHO Weight-for-length z-score < -2 to < -3	WHO Weight-for-length z-score < -3	WHO Weight-for-length z-score < -3 with life-threatening consequences
**Weight Loss** (excludes postpartum weight loss)	NA	5 to < 9% loss in body weight from baseline	≥ 9 to < 20% loss in body weight from baseline	≥ 20% loss in body weight from baseline OR<br>Aggressive intervention indicated (e.g., tube feeding, total parenteral nutrition)

Weight Loss (excludes postpartum weight loss) NA 5 to < 9% loss in body weight from baseline ≥ 9 to < 20% loss in body weight from baseline

[12] WHO reference tables may be accesed by clicking the desired age range or by accessing the following URLs: http://www.who.int/growthref/who2007 bmi for age/en/ for participants > 5 to 19 years of age and http://www.who.int/childgrowth/standards/chart catalogue/en/ for those < 5 years of age.

Urinary

PARAMETER	GRADE 1 MILD	GRADE 2 MODERATE	GRADE 3 SEVERE	GRADE 4 POTENTIALLY LIFE-THREATENING
**Urinary Tract Obstruction**	NA	Signs or symptoms of urinary tract obstruction without hydronephrosis or renal dysfunction	Signs or symptoms of urinary tract obstruction with hydronephrosis or renal dysfunction	Obstruction causing life-threatening consequences

Site Reactions to Injections and Infusions

PARAMETER	GRADE 1 MILD	GRADE 2 MODERATE	GRADE 3 SEVERE	GRADE 4 POTENTIALLY LIFE-THREATENING
**Injection Site Pain or Tenderness** *Report only one*	Pain or tenderness causing no or minimal limitation of use of limb	Pain or tenderness causing greater than minimal limitation of use of limb	Pain or tenderness causing inability to perform usual social & functional activities	Pain or tenderness causing inability to perform basic self-care function OR Hospitalization indicated
**Injection Site Erythema or Redness**[13] *Report only one* *> 15 years of age*	2.5 to < 5 cm in diameter OR 6.25 to < 25 cm² surface area AND Symptoms causing no or minimal interference with usual social & functional activities	> 5 to < 10 cm in diameter OR > 25 to < 100 cm² surface area OR Symptoms causing greater than minimal interference with usual social & functional activities	> 10 cm in diameter OR > 100 cm² surface area OR Ulceration OR Secondary infection OR Phlebitis OR Sterile abscess OR Drainage OR Symptoms causing inability to perform usual social & functional activities	Potentially life-threatening consequences (e.g., abscess, exfoliative dermatitis, necrosis involving dermis or deeper tissue)
< 15 years of age	≤ 2.5 cm in diameter	> 2.5 cm in diameter with < 50% surface area of the extremity segment involved (e.g., upper arm or thigh)	≥ 50% surface area of the extremity segment involved (e.g., upper arm or thigh) OR Ulceration OR Secondary infection OR Phlebitis OR Sterile abscess OR Drainage	Potentially life-threatening consequences (e.g., abscess, exfoliative dermatitis, necrosis involving dermis or deeper tissue)
**Injection Site Induration or Swelling** *Report only one* *> 15 years of age*	Same as for **Injection Site Erythema or Redness,** *> 15 years of age*	Same as for **Injection Site Erythema or Redness,** *> 15 years of age*	Same as for **Injection Site Erythema or Redness,** *> 15 years of age*	Same as for **Injection Site Erythema or Redness,** *> 15 years of age*

≤ 15 years of age	Same as for **Injection Site Erythema or Redness,** *< 15 years of age*	Same as for **Injection Site Erythema or Redness,** *< 15 years of age*	Same as for **Injection Site Erythema or Redness,** *< 15 years of age*	Same as for **Injection Site Erythema or Redness,** *< 15 years of age*
**Injection Site Pruritus**	Itching localized to the injection site that is relieved spontaneously or in < 48 hours of treatment	Itching beyond the injection site that is not generalized OR<br>Itching localized to the injection site requiring > 48 hours treatment	Generalized itching causing inability to perform usual social & functional activities	NA

[13] Injection Site Erythema or Redness should be evaluated and graded using the greatest single diameter or measured surface area.

Laboratory Values

Chemistries

PARAMETER	GRADE 1 MILD	GRADE 2 MODERATE	GRADE 3 SEVERE	GRADE 4 POTENTIALLY LIFE-THREATENING
**Acidosis**	NA	pH ≥ 7.3 to < LLN	pH < 7.3 without life-threatening consequences	pH < 7.3 with life-threatening consequences
**Albumin, Low** (g/dL; *g/L*)	3.0 to < LLN<br>*30 to < LLN*	≥ 2.0 to < 3.0<br>*≥ 20 to < 30*	< 2.0<br>*< 20*	NA
**Alkaline Phosphatase, High**	1.25 to < 2.5 x ULN	2.5 to < 5.0 x ULN	5.0 to < 10.0 x ULN	≥ 10.0 x ULN
**Alkalosis**	NA	pH > ULN to ≤ 7.5	pH > 7.5 without life-threatening consequences	pH > 7.5 with life-threatening consequences
**ALT or SGPT, High**<br>*Report only one*	1.25 to < 2.5 x ULN	2.5 to < 5.0 x ULN	5.0 to < 10.0 x ULN	≥ 10.0 x ULN
**Amylase (Pancreatic) or Amylase (Total), High**<br>*Report only one*	1.1 to < 1.5 x ULN	1.5 to < 3.0 x ULN	3.0 to < 5.0 x ULN	≥ 5.0 x ULN
**AST or SGOT, High**<br>*Report only one*	1.25 to < 2.5 x ULN	2.5 to < 5.0 x ULN	5.0 to < 10.0 x ULN	≥ 10.0 x ULN
**Bicarbonate, Low** (mEq/L; *mmol/L*)	16.0 to < LLN<br>*16.0 to < LLN*	11.0 to < 16.0<br>*11.0 to < 16.0*	8.0 to < 11.0<br>*8.0 to < 11.0*	< 8.0<br>*< 8.0*
**Bilirubin**<br>***Direct Bilirubin[14], High***<br>*> 28 days of age*	NA	NA	> ULN	> ULN with life-threatening consequences (e.g., signs and symptoms of liver failure)
≤ 28 days of age	ULN to ≤ 1 mg/dL	> 1 to ≤ 1.5 mg/dL	> 1.5 to ≤ 2 mg/dL	> 2 mg/dL
Total Bilirubin, High<br>*> 28 days of age*	1.1 to < 1.6 x ULN	1.6 to < 2.6 x ULN	2.6 to < 5.0 x ULN	≥ 5.0 x ULN
≤ 28 days of age	See Appendix A. Total Bilirubin for Term and Preterm Neonates	See Appendix A. Total Bilirubin for Term and Preterm Neonates	See Appendix A. Total Bilirubin for Term and Preterm Neonates	See Appendix A. Total Bilirubin for Term and Preterm Neonates
**Calcium, High** (mg/dL; *mmol/L*)<br>*> 7 days of age*	10.6 to < 11.5<br>*2.65* to *< 2.88*	11.5 to < 12.5<br>*2.88* to *< 3.13*	12.5 to < 13.5<br>*3.13* to *< 3.38*	≥ 13.5<br>*≥ 3.38*
< 7 days of age	11.5 to < 12.4<br>*2.88* to *< 3.10*	12.4 to < 12.9<br>*3.10* to *< 3.23*	12.9 to < 13.5<br>*3.23* to *< 3.38*	≥ 13.5<br>*≥ 3.38*

[14] Direct bilirubin > 1.5 mg/dL in a participant < 28 days of age should be graded as grade 2, if < 10% of the total bilirubin.

PARAMETER	GRADE 1<br>MILD	GRADE 2<br>MODERATE	GRADE 3<br>SEVERE	GRADE 4<br>POTENTIALLY<br>LIFE-<br>THREATENING
**Calcium (Ionized), High**<br>(mg/dL; *mmol/L*)	> ULN to < 6.0<br>*> ULN to < 1.5*	6.0 to < 6.4<br>*1.5 to < 1.6*	6.4 to < 7.2<br>*1.6 to < 1.8*	≥ 7.2<br>*2 1.8*
**Calcium, Low**<br>(mg/dL;<br>*mmol/L*)<br>*2 7 days of age*	7.8 to < 8.4<br>*1.95* to *< 2.10*	7.0 to < 7.8<br>*1.75* to *< 1.95*	6.1 to < 7.0<br>*1.53* to *< 1.75*	< 6.1<br>*< 1.53*
< 7 days of age	6.5 to < 7.5<br>*1.63* to *< 1.88*	6.0 to < 6.5<br>*1.50* to *< 1.63*	5.50 to < 6.0<br>*1.38* to *< 1.50*	< 5.50<br>*< 1.38*
**Calcium (Ionized), Low**<br>(mg/dL; *mmol/L*)	< LLN to 4.0<br>*< LLN to 1.0*	3.6 to < 4.0<br>*0.9 to < 1.0*	3.2 to < 3.6<br>*0.8 to < 0.9*	< 3.2<br>*< 0.8*
**Cardiac Troponin I, High**	NA	NA	NA	Levels consistent with myocardial infarction or unstable angina as defined by the local laboratory
**Creatine Kinase, High**	3 to < 6 x ULN	6 to < 10 x ULN	10 to < 20 x ULN	≥ 20 x ULN
**Creatinine, High**	1.1 to 1.3 x ULN	> 1.3 to 1.8 x ULN OR Increase of > 0.3 mg/dL above baseline	> 1.8 to < 3.5 x ULN OR Increase of 1.5 to < 2.0 x above baseline	≥ 3.5 x ULN OR Increase of ≥ 2.0 x above baseline
**Creatinine Clearance[15] or eGFR, Low**<br>*Report only one*	NA	< 90 to 60 ml/min or ml/min/1.73 m^2 OR 10 to < 30% decrease from baseline	< 60 to 30 ml/min or ml/min/1.73 m^2 OR ≥ 30 to < 50% decrease from baseline	< 30 ml/min or ml/min/1.73 m^2 OR ≥ 50% decrease from baseline or dialysis needed
**Glucose**<br>(mg/dL; *mmol/L*)<br>***Fasting, High***	110 to 125<br>*6.11 to < 6.95*	> 125 to 250<br>*6.95 to < 13.89*	> 250 to 500<br>*13.89 to < 27.75*	> 500<br>*≥ 27.75*
Nonfasting, High	116 to 160<br>*6.44 to < 8.89*	> 160 to 250<br>*8.89 to < 13.89*	> 250 to 500<br>*13.89 to < 27.75*	> 500<br>*≥ 27.75*
**Glucose, Low**<br>(mg/dL; *mmol/L*)<br>*2 1 month of age*	55 to 64<br>*3.05 to 3.55*	40 to < 55<br>*2.22 to < 3.05*	30 to < 40<br>*1.67 to < 2.22*	< 30<br>*< 1.67*
< 1 month of age	50 to 54<br>*2.78 to 3.00*	40 to < 50<br>*2.22 to < 2.78*	30 to < 40<br>*1.67 to < 2.22*	< 30<br>*< 1.67*
**Lactate, High**	ULN to < 2.0 x ULN without acidosis	≥ 2.0 x ULN without acidosis	Increased lactate with pH < 7.3 without life-threatening consequences	Increased lactate with pH < 7.3 with life-threatening consequences

15 Use the applicable formula (i.e., Cockroft-Gault in mL/min or Schwatrz in mL/min/1.73m^2).

PARAMETER	GRADE 1 MILD	GRADE 2 MODERATE	GRADE 3 SEVERE	GRADE 4 POTENTIALLY LIFE-THREATENING
**Lipase, High**	1.1 to < 1.5 x ULN	1.5 to < 3.0 x ULN	3.0 to < 5.0 x ULN	≥ 5.0 x ULN
**Lipid Disorders** (mg/dL; *mmol/L*) *Cholesterol, Fasting, High* > 18 years of age	200 to < 240 *5.18 to < 6.19*	240 to < 300 *6.19 to < 7.77*	≥ 300 *≥ 7.77*	NA
< 18 years of age	170 to < 200 *4.40 to < 5.15*	200 to < 300 *5.15 to < 7.77*	≥ 300 *≥ 7.77*	NA
LDL, Fasting, High *> 18 years of age*	130 to < 160 *3.37 to < 4.12*	160 to < 190 *4.12 to < 4.90*	≥ 190 *≥ 4.90*	NA
> 2 to < 18 years of age	110 to < 130 *2.85 to < 3.34*	130 to < 190 *3.34 to < 4.90*	≥ 190 *> 4.90*	NA
Triglycerides, Fasting, High	150 to 300 *1.71 to 3.42*	>300 to 500 *>3.42 to 5.7*	>500 to < 1,000 *>5.7 to 11.4*	> 1,000 *> 11.4*
**Magnesium[16], Low** (mEq/L; *mmol/L*)	1.2 to < 1.4 *0.60 to < 0.70*	0.9 to < 1.2 *0.45 to < 0.60*	0.6 to < 0.9 *0.30 to < 0.45*	< 0.6 *< 0.30*
**Phosphate, Low** (mg/dL; *> 14 years of age*	2.0 to < LLN *0.81 to < LLN*	1.4 to < 2.0 *0.65 to < 0.81*	1.0 to < 1.4 *0.32 to < 0.65*	< 1.0 *< 0.32*
1 to 14 years of age	3.0 to < 3.5 *0.97 to < 1.13*	2.5 to < 3.0 *0.81 to < 0.97*	1.5 to < 2.5 *0.48 to < 0.81*	< 1.5 *< 0.48*
< 1 year of age	3.5 to < 4.5 *1.13 to < 1.45*	2.5 to < 3.5 *0.81 to < 1.13*	1.5 to < 2.5 *0.48 to < 0.81*	< 1.5 *< 0.48*
**Potassium, High** (mEq/L; *mmol/L*)	5.6 to < 6.0 *5.6 to < 6.0*	6.0 to < 6.5 *6.0 to < 6.5*	6.5 to < 7.0 *6.5 to < 7.0*	≥ 7.0 *≥ 7.0*
**Potassium, Low** (mEq/L; *mmol/L*)	3.0 to < 3.4 *3.0 to < 3.4*	2.5 to < 3.0 *2.5 to < 3.0*	2.0 to < 2.5 *2.0 to < 2.5*	< 2.0 *< 2.0*
**Sodium, High** (mEq/L; *mmol/L*)	146 to < 150 *146 to < 150*	150 to < 154 *150 to < 154*	154 to < 160 *154 to < 160*	≥ 160 *≥ 160*
**Sodium, Low** (mEq/L; *mmol/L*)	130 to < 135 *130 to < 135*	125 to < 130 *125 to < 135*	121 to < 125 *121 to < 125*	≤ 120 *≤ 120*
**Uric Acid, High** (mg/dL; *mmol/L*)	7.5 to < 10.0 *0.45 to < 0.59*	10.0 to < 12.0 *0.59 to < 0.71*	12.0 to < 15.0 *0.71 to < 0.89*	≥ 15.0 *≥ 0.89*

16 To convert a magnesium value from mg/dL to mmol/L, laboratories should multiply by 0.4114.

Hematology

PARAMETER	GRADE 1 MILD	GRADE 2 MODERATE	GRADE 3 SEVERE	GRADE 4 POTENTIALLY LIFE-THREATENING
**Absolute CD4+ Count, Low** (cell/mm^3; *cells/L*)<br>*> 5 years of age (not HIV infected)*	300 to < 400<br>*300 to < 400*	200 to < 300<br>*200 to < 300*	100 to < 200<br>*100 to < 200*	< 100<br>*< 100*
**Absolute Lymphocyte Count, Low** (cell/mm^3; *cells/L*)<br>*> 5 years of age (not HIV infected)*	600 to < 650<br>*0.600 x 10^9 to < 0.650 x 10^9*	500 to < 600<br>*0.500 x 10^9 to < 0.600 x 10^9*	350 to < 500<br>*0.350 x 10^9 to < 0.500 x 10^9*	< 350<br>*< 0.350 x 10^9*
**Absolute Neutrophil Count (ANC), Low** (cells/mm^3; *cells/L*)<br>*> 7 days of age*	800 to 1,000<br>*0.800 x 10^9 to 1.000 x 10^9*	600 to 799<br>*0.600 x 10^9 to 0.799 x 10^9*	400 to 599<br>*0.400 x 10^9 to 0.599 x 10^9*	< 400<br>*< 0.400 x 10^9*
2 to 7 days of age	1,250 to 1,500<br>*1.250 x 10^9 to 1.500 x 10^9*	1,000 to 1,249<br>*1.000 x 10^9 to 1.249 x 10^9*	750 to 999<br>*0.750 x 10^9 to 0.999 x 10^9*	< 750<br>*< 0.750 x 10^9*
≤ 1 day of age	4,000 to 5,000<br>*4.000 x 10^9 to 5.000 x 10^9*	3,000 to 3,999<br>*3.000 x 10^9 to 3.999 x 10^9*	1,500 to 2,999<br>*1.500 x 10^9 to 2.999 x 10^9*	< 1,500<br>*< 1.500 x 10^9*
**Fibrinogen, Decreased** (mg/dL; *g/L*)	100 to < 200<br>*1.00 to < 2.00*<br>OR<br>0.75 to < 1.00 x LLN	75 to < 100<br>*0.75 to < 1.00*<br>OR<br>≥ 0.50 to < 0.75 x LLN	50 to < 75<br>*0.50 to < 0.75*<br>OR<br>0.25 to < 0.50 x LLN	< 50<br>*< 0.50*<br>OR<br>< 0.25 x LLN<br>OR Associated with gross bleeding
**Hemoglobin[17], Low** (g/dL; *mmol/L*)[18]<br>*≥ 13 years of age (male only)*	10.0 to 10.9<br>*6.19 to 6.76*	9.0 to < 10.0<br>*5.57 to < 6.19*	7.0 to < 9.0<br>*4.34 to < 5.57*	< 7.0<br>*< 4.34*
≥ 13 years of age (female only)	9.5 to 10.4<br>*5.88 to 6.48*	8.5 to < 9.5<br>*5.25 to < 5.88*	6.5 to < 8.5<br>*4.03 to < 5.25*	< 6.5<br>*< 4.03*

[17] Male and female sex are defined as sex at birth.
[18] The conversion factor used to convert g/dL to mmol/L is 0.6206 and is the most commonly used conversion factor. For grading hemoglobin results obtained by an analytic method with a conversion factor other than 0.6206, the result must be converted to g/dL using the appropriate conversion factor for the particular laboratory.

PARAMETER	GRADE 1 MILD	GRADE 2 MODERATE	GRADE 3 SEVERE	GRADE 4 POTENTIALLY LIFE-THREATENING
57 days of age to < 13 years of age (male and female)	9.5 to 10.4<br>*5.88 to 6.48*	8.5 to < 9.5<br>*5.25 to < 5.88*	6.5 to < 8.5<br>*4.03 to < 5.25*	< 6.5<br>*< 4.03*
36 to 56 days of age (male and female)	8.5 to 9.6<br>*5.26 to 5.99*	7.0 to < 8.5<br>*4.32 to < 5.26*	6.0 to < 7.0<br>*3.72 to < 4.32*	< 6.0<br>*< 3.72*
22 to 35 days of age (male and female)	9.5 to 11.0<br>*5.88 to 6.86*	8.0 to < 9.5<br>*4.94 to < 5.88*	6.7 to < 8.0<br>*4.15 to < 4.94*	< 6.7<br>*< 4.15*
8 to < 21 days of age (male and female)	11.0 to 13.0<br>*6.81 to 8.10*	9.0 to < 11.0<br>*5.57 to < 6.81*	8.0 to < 9.0<br>*4.96 to < 5.57*	< 8.0<br>*< 4.96*
< 7 days of age (male and female)	13.0 to 14.0<br>*8.05 to 8.72*	10.0 to < 13.0<br>*6.19 to < 8.05*	9.0 to < 10.0<br>*5.59 to < 6.19*	< 9.0<br>*< 5.59*
**INR, High** (not on anticoagulation therapy)	1.1 to < 1.5 x ULN	1.5 to < 2.0 x ULN	2.0 to < 3.0 x ULN	≥ 3.0 x ULN
**Methemoglobin** (% hemoglobin)	5.0 to < 10.0%	10.0 to < 15.0%	15.0 to < 20.0%	≥ 20.0%
**PTT, High** (not on anticoagulation therapy)	1.1 to < 1.66 x ULN	1.66 to < 2.33 x ULN	2.33 to < 3.00 x ULN	≥ 3.00 x ULN
**Platelets, Decreased** (cells/mm^3; *cells/L*)	100,000 to < 124,999<br>*100.000 x 10^9 to < 124.999 x 10^9*	50,000 to < 100,000<br>*50.000 x 10^9 to < 100.000 x 10^9*	25,000 to < 50,000<br>*25.000 x 10^9 to < 50.000 x 10^9*	< 25,000<br>*< 25.000 x 10^9*
**PT, High** (not on anticoagulation therapy	1.1 to < 1.25 x ULN	1.25 to < 1.50 x ULN	1.50 to < 3.00 x ULN	≥ 3.00 x ULN
**WBC, Decreased** (cells/mm^3; *cells/L*)<br>*> 7 days of age*	2,000 to 2,499<br>*2.000 x 10^9 to 2.499 x 10^9*	1,500 to 1,999<br>*1.500 x 10^9 to 1.999 x 10^9*	1,000 to 1,499<br>*1.000 x 10^9 to 1.499 x 10^9*	< 1,000<br>*< 1.000 x 10^9*
< 7 days of age	5,500 to 6,999<br>*5.500 x 10^9 to 6.999 x 10^9*	4,000 to 5,499<br>*4.000 x 10^9 to 5.499 x 10^9*	2,500 to 3,999<br>*2.500 x 10^9 to 3.999 x 10^9*	< 2,500<br>*< 2.500 x 10^9*

Urinalysis

PARAMETER	GRADE 1 MILD	GRADE 2 MODERATE	GRADE 3 SEVERE	GRADE 4 POTENTIALLY LIFE-THREATENING
**Glycosuria** (random collection tested by dipstick)	Trace to 1+ or < 250 mg	2+ or > 250 to < 500 mg	> 2+ or > 500 mg	NA
**Hematuria** (not to be reported based on dipstick findings or on blood believed to be of menstrual origin)	6 to < 10 RBCs per high power field	≥ 10 RBCs per high power field	Gross, with or without clots OR<br>With RBC casts OR<br>Intervention indicated	Life-threatening consequences
**Proteinuria** (random collection tested by dipstick)	1+	2+	3+ or higher	NA

References

1 (UNAIDS)., J. U. N. P. o. H. A. The Gap Report., (UNAIDS, 2014).
2 UNAIDS. AIDS by the numbers 2015. (2015).
3 CDC. CDC. Vital Signs: HIV Diagnosis, Care, and Treatment Among Persons Living with HIV- United States 2011. *MMWR* **4**, 1-6 (2014).
4 Jardine, J. *et al.* Rational HIV immunogen design to target specific germline B cell receptors. *Science* **340**, 711-716, doi:10.1126/science.1234150 (2013).
5 Sok, D. *et al.* Promiscuous glycan site recognition by antibodies to the high-mannose patch of gp120 broadens neutralization of HIV. *Sci Transl Med* **6**, 236ra263, doi:10.1126/scitranslmed.3008104 (2014).
6 Caskey, M. *et al.* Viraemia suppressed in HIV-1-infected humans by broadly neutralizing antibody 3BNC117. *Nature* **522**, 487-491, doi:10.1038/nature14411 (2015).
7 Barouch, D. H. *et al.* Therapeutic efficacy of potent neutralizing HIV-1-specific monoclonal antibodies in SHIV-infected rhesus monkeys. *Nature* **503**, 224-228, doi:10.1038/nature12744 (2013).
8 Hessell, A. J. *et al.* Broadly neutralizing human anti-HIV antibody 2G12 is effective in protection against mucosal SHIV challenge even at low serum neutralizing titers. *PLoS Pathog* **5**, e1000433, doi:10.1371/journal.ppat.1000433 (2009).
9 Hessell, A. J. *et al.* Effective, low-titer antibody protection against low-dose repeated mucosal SHIV challenge in macaques. *Nat Med* **15**, 951-954, doi:10.1038/nm.1974 (2009).
10 Moldt, B. *et al.* Highly potent HIV-specific antibody neutralization in vitro translates into effective protection against mucosal SHIV challenge in vivo. *Proceedings of the National Academy of Sciences of the United States of America* **109**, 18921-18925, doi:10.1073/pnas.1214785109 (2012).
11 Walker, L. M. *et al.* Broad neutralization coverage of HIV by multiple highly potent antibodies. *Nature* **477**, 466-470, doi:10.1038/nature10373 (2011).
12 Haynes, B. F. & McElrath, M. J. Progress in HIV-1 vaccine development. *Curr Opin HIV AIDS* **8**, 326-332, doi:10.1097/COH.0b013e328361d178 (2013).
13 Burton, D. R. & Mascola, J. R. Antibody responses to envelope glycoproteins in HIV-1 infection. *Nat Immunol* **16**, 571-576, doi:10.1038/ni.3158 (2015).
14 Sok, D. *et al.* Recombinant HIV envelope trimer selects for quaternary-dependent antibodies targeting the trimer apex. *Proc Natl Acad Sci U S A* **111**, 17624-17629, doi:10.1073/pnas.1415789111 (2014).
15 Scheid, J. F. *et al.* Sequence and structural convergence of broad and potent HIV antibodies that mimic CD4 binding. *Science* **333**, 1633-1637, doi:10.1126/science.1207227 (2011).
16 Shingai, M. *et al.* Passive transfer of modest titers of potent and broadly neutralizing anti-HIV monoclonal antibodies block SHIV infection in macaques. *J Exp Med* **211**, 2061-2074, doi:10.1084/jem.20132494 (2014).
17 Lynch, R. M. *et al.* Virologic effects of broadly neutralizing antibody VRC01 administration during chronic HIV-1 infection. *Sci Transl Med* **7**, 319ra206, doi:10.1126/scitranslmed.aad5752 (2015).
18 Andrade, A. *et al.* Three distinct phases of HIV-1 RNA decay in treatment-naive patients receiving raltegravir-based antiretroviral therapy: ACTG A5248. *J Infect Dis* **208**, 884-891, doi:10.1093/infdis/jit272 (2013).

International AIDS Vaccine Initiative

DOCUMENT NUMBER:
DOCUMENT TITLE:
DOCUMENT NOTES:

Document Information

Revision:	Vault:
Status:	Document Type:

Date Information

Effective Date:	Expiration Date:
Release Date:	Next Review Date:

Control Information

Author:	Owner:
Previous Number:	Change Number:

Signature Manifest

Document Number: TMF-02-0166 **Revision:** 0

Title: Protocol PGT121

All dates and times are in Eastern Time Zone.

Protocol PGT121

Change Request Approval

Name/Signature	Title	Date	Meaning/Reason
Dani Vooijs (DVOOIJS)			
Katherine Crisafi (KCRISAFI)			
Michele Fong Lim (MFONGLIM)	Director Quality Systems	21 Nov 2015, 09:45:00 AM	Approved

CMO Approval

Name/Signature	Title	Date	Meaning/Reason
Frances Priddy (FPRIDDY)	Chief Medical Officer	05 Aug 2016, 01:02:25 PM	Approved

QA Final Release

Name/Signature	Title	Date	Meaning/Reason
Michele Fong Lim (MFONGLIM)	Director Quality Systems	05 Aug 2016, 02:30:33 PM	Approved

Notify

Name/Signature	Title	Date	Meaning/Reason
Hubrecht Gelderblom (HGELDERBLOM)		05 Aug 2016, 02:30:34 PM	Email Sent
Lisa Sunner (LSUNNER)		05 Aug 2016, 02:30:34 PM	Email Sent
Melissa Schroeter (MSCHROETER)		05 Aug 2016, 02:30:34 PM	Email Sent

**Protocol Title:**	A Phase 1 Randomized Placebo-controlled Clinical Trial of the Safety, Pharmacokinetics and Antiviral Activity of PGT121 Monoclonal Antibody (mAb) in HIV-uninfected and HIV-infected Adults
**Protocol Number:**	IAVI T001
**Phase:**	Phase 1
**Regulatory Investigational Product Number:**	New IND submission
**Sponsor:**	International AIDS Vaccine Initiative (IAVI)<br>125 Broad Street, 9th Floor<br>New York, New York 10004<br>USA
**Sponsor Status**	Not for-Profit Organization
**Date of Protocol Version:**	09 September 2016<br>02.0<br>Revision to IRB Submission<br><br>05 August 2016<br>01.0<br>IRB Submission

THE CONFIDENTIAL INFORMATION IN THIS DOCUMENT IS PROVIDED TO YOU AS AN INVESTIGATOR, POTENTIAL INVESTIGATOR, OR CONSULTANT, FOR REVIEW BY YOU, YOUR STAFF, AND APPLICABLE INSTITUTIONAL REVIEW BOARDS (IRBS) AND/OR INDEPENDENT ETHICS COMMITTEES (IECS). IT IS UNDERSTOOD THAT THE INFORMATION WILL NOT BE DISCLOSED TO OTHERS, EXCEPT TO THE EXTENT NECESSARY TO OBTAIN ETHICAL AND REGULATORY APPROVAL FROM THE RESPECTIVE COMMITTEE'S AGENCIES AND INFORMED CONSENT FROM THOSE PERSONS TO WHOM THE INVESTIGATIONAL PRODUCT MAY BE ADMINISTERED.

SYNOPSIS

**TITLE**	A Phase 1 Randomized Placebo-controlled Clinical Trial of the Safety, Pharmacokinetics and Antiviral Activity of PGT121 Monoclonal Antibody (mAb) in HIV-uninfected and HIV-infected Adults
**PROTOCOL NUMBER**	IAVI T001
**PHASE**	Phase 1
**SPONSOR**	International AIDS Vaccine Initiative (IAVI)<br>125 Broad Street, 9th Floor<br>New York, New York 10004,<br>USA
**SPONSOR STATUS**	Not for Profit Organization
**STUDY PRODUCTS**	PGT121 monoclonal antibody (mAb)
**OBJECTIVES**	**Primary Objectives**<br>• To evaluate the safety and tolerability of a single intravenous (IV) infusion of PGT121 mAb at 3, 10, and 30 mg/kg in HIV-uninfected adults and HIV-infected adults<br>• To evaluate the pharmacokinetic (PK) profile of IV infusion of PGT121 mAb in HIV-uninfected and HIV-infected adults<br>• To evaluate the antiviral activity of IV infusion of PGT121 mAb in HIV-infected adults not on ART<br><br>**Secondary Objective**<br>• To determine if PGT121 induces anti-PGT121 antibodies<br>• To determine the effect of PGT121 mAb on CD4+ T cell counts in HIV-infected adults<br>• To determine if PGT121 mAb induces viral escape mutants in viremic HIV-infected adults not on ART<br><br>**Exploratory Objectives**<br>• To determine if PGT121 mAb has any impact on the host immune responses (i.e., HIV-specific cellular and humoral immune response)<br>• To determine the effect of PGT121 mAb on the size of the latent HIV reservoir in HIV-infected adults<br>• To determine PGT121 mAb levels in mucosal secretions in HIV-uninfected and HIV-infected adults<br>• To measure in vitro neutralization of HIV isolates with participant's serum post PGT121 mAb IV infusion<br>• To determine if PGT121 mAb has any impact on resistance mutations to ARVs

**ENDPOINTS**	***Primary***:<br><br>*Safety and Tolerability:*<br>We will measure the following endpoints to evaluate the safety and tolerability of PGT121 mAb in HIV-uninfected and HIV-infected adults:<br><br>1. Proportion of participants with moderate or greater reactogenicity (e.g., solicited adverse events) for 3 days following IV infusion of PGT121 mAb.<br>2. Proportion of participants with moderate or greater and/or PGT121 mAb-related unsolicited adverse events (AEs), including safety laboratory (biochemical, hematological) parameters, following IV infusion of PGT121 mAb for the first 56 days post administration of Investigational Product.<br>3. Proportion of participants with PGT121 mAb-related serious adverse events (SAEs) throughout the study period.<br><br>*Pharmacokinetics:*<br>We will measure the following endpoints to determine the pharmacokinetics following IV infusion of PGT121 mAb in HIV-uninfected and HIV-infected adults:<br><br>1. Elimination half-life (t1/2)<br>2. Clearance (CL/F)<br>3. Volume of distribution (Vz/F)<br>4. Area under the concentration decay curve (AUC)<br>5. Impact of viral load and/or ART on PGT121 disposition (elimination half-life (t1/2), clearance (CL/F), volume of distribution (Vz/F), total exposure<br><br>*Antiviral activity:*<br>We will calculate the following endpoints to determine the antiviral activity following IV infusion of PGT121 mAb in viremic HIV-infected adults not on ART:<br><br>1. Change in plasma HIV-1 RNA levels from baseline (mean of pre-entry and entry values)<br><br>***Secondary***:<br><br>*Anti-PGT121 antibodies:*<br>We will calculate the following endpoint to determine if anti-PGT121 antibodies are elicited following IV infusion of PGT121 mAb in HIV-uninfected and HIV-infected adults:<br><br>1. Change in serum anti-PGT121 antibody titers from baseline<br><br>*CD4+ T cell count:*<br>We will calculate the following endpoint to determine if IV infusion of PGT121 mAb has any impact on CD4+ T cell counts in HIV-infected

adults:

1. Change in CD4+ T cell count and frequency compared to baseline as measured by single platform flow cytometry

HIV genotyping/phenotyping of circulating virus for evaluation of PGT121 mAb -induced escape mutations:
We will compare plasma virus sequences before and after IV infusion of PGT121 mAb to determine if PGT121 mAb induces viral escape mutations in viremic HIV-infected adults not on ART.

1. Development of sequence variations in epitopes known to result in reduced PGT121 mAb neutralization susceptibility.

Exploratory:

Additional assessments may include, but are not limited to, the following: HIV-specific IgG/IgA binding responses by ELISA, HIV-specific cellular immune responses by ELISPOT, HIV-specific antibody function by ADCC, ADCP, and ADCVI assays, resistance mutations to current ARVs, PGT121 mAb levels in mucosal secretions, changes in total HIV-1 DNA and 2-long terminal repeat (LTR) circular HIV-1 DNA in resting or total CD4 T cells and in vitro neutralization of HIV isolates with participant's serum post IV infusion of PGT121 mAb.

STUDY DESIGN TABLE

	Group	Participants	Sub-Group	Regimen	N	Dose (mg/kg)
Part 1[1]	1[3]	HIV-uninfected participants	1A	PGT121/Placebo	4/1 (6/2 if DLT[2])	3
			1B	PGT121/Placebo	4/1 (6/2 if DLT)	10
			1C	PGT121/Placebo	4/1 (6/2 if DLT)	30
	2[3]	HIV-infected on ART, (<50 cp/ml)	2A	PGT121/Placebo	4/1 (6/2 if DLT[2])	3
			2B	PGT121/Placebo	4/1 (6/2 if DLT)	10
			2C	PGT121/Placebo	4/1 (6/2 if DLT)	30
Safety Monitoring Committee Review[4]						
Part 2	3[5]	HIV-Infected off ART (VL $2x10^3$ – $1x10^5$cp/ml)	3A[6]	PGT121	6 (max 9)	30
			3B	PGT121	8 (max 12)	10
			3C	PGT121	10 (max 15)	3
		HIV-Infected off ART (VL $1x10^2$ – $2x10^3$ cp/ml)	3D[7]	PGT121	3	30
			3E	PGT121	3	10
			3F	PGT121	3	3

DLT, dose limiting toxicity; ART, antiretroviral therapy; cp, copies; ml, milliliter
Administration of PGT 121 will be by intravenous infusion (IV)

1. Eligible participants for Groups 1 and 2 will be enrolled according to their HIV-serostatus and will occur in parallel. At each dose level in Part 1, investigational product (IP) administration will be separated by at least 4 days for each of the first 3 participants, to ensure at least 1 participant receives active product and is observed for at least 4 days before administration to additional participants.
2. A dose limiting toxicity (DLT) will be defined as 1) any Grade 3 or greater reactogenicity or adverse event judged by the study investigators as at least possibly related to investigational product or 2) any SAE considered at least possibly related to investigational product.
3. Within each group, the PSRT will review data. If no DLT occurs within 2 weeks from infusion of the 5 participants in a dose group, dose escalation to the next dose group will proceed. If 1 DLT occurs, 3 additional participants will be enrolled; randomized between PGT121 mAb and placebo at a 2:1 ratio. If no additional DLTs occur within 2 weeks of infusion in the 8 total participants (3:1 ratio of PGT121 mAb to placebo), study can proceed with enrolment of the next dose group. If 2 or more DLTs accumulate that are the same, similar, or in the same System Organ Class, dosing will be halted and the next lower dose level will be declared the maximum tolerated dose (MTD). If no DLT occurs in the final dose group, MTD will be the highest dose given (subgroups 1C and 2C 30mg/kg) after 14 days of follow-up.
4. Following IP administration of the last participant in both Groups 1 and 2, an independent Safety Monitoring Committee (SMC) will review at least the first 14 days of safety data to confirm MTD in each group, and determine whether, and at what dose, Group 3 can initiate enrollment.
5. Group 3 will start with the MTD as determined in Part 1. Group 3 will start with subgroups 3A and

	3D if the MTD is 30mg/kg, subgroups 3B and 3E if the MTD is 10mg/kg and subgroups 3C and 3F if the MTD is 3mg/kg.<br>6. If subgroup 3A achieves a mean decline in HIV RNA of ≥0.9 log compared to baseline, enrolment into subgroup 3A will be stopped and the next lower dose subgroup 3B will be initiated. The same rule will apply for progression from subgroup 3B to 3C. At least 6, 8 and 10 but up to 9, 12 and 15 participants, will be enrolled in subgroups 3A, 3B, and 3C respectively, until the minimum effective dose is determined. If a mean decline ≥0.9 log in HIV RNA is not achieved, enrollment will be stopped at the completion of enrolment at that dose level.<br>7. As soon as subgroup 3D has enrolled 3 participants, enrolment into subgroup 3E will be initiated. The same rule will apply for progression from subgroup 3E to 3F. In the event that dose de-escalation in subgroups 3A-C is stopped due to failure to meet virologic endpoints, dose de-escalation in subgroups 3D-F will continue.
**METHODS**	See Schedule of Procedures, Appendix A
**STUDY POPULATION**	The study population will include three different groups:<br>Group 1 will include HIV-uninfected males or females aged 18-50 years old who are willing to maintain low risk behavior for HIV infection; principal exclusion criteria include confirmed HIV-infection, pregnancy or lactation, significant acute or chronic disease and clinically significant laboratory abnormalities. Group 2 will include HIV-infected males or females aged 18-50 years old on a stable antiretroviral regimen with HIV-1 RNA plasma level <50 copies/ml, CD4 cell count > 300 cells/uL and CD4 nadir > 200 cell/uL; principal exclusion criteria include history of AIDS-defining illness, significant acute or chronic medical condition other than HIV infection, and clinically significant laboratory abnormalities. Group 3 will include HIV-infected males or females aged 18-50 years old, not on antiretroviral therapy for > 6 month with detectable HIV-1 RNA plasma level between 100 and 100,000 copies/ml, CD4 cell count > 300 cells/uL and CD4 nadir > 200 cell/uL; principal exclusion criteria include history of AIDS-defining illness, significant acute or chronic medical condition other than HIV infection, and clinically significant laboratory abnormalities.
**NUMBER OF PARTICIPANTS**	63-93 participants will be included.
**DOSE ESCALATION and PAUSE RULES**	The first part of this study is a dose-escalation trial in HIV-uninfected adults and HIV-infected adults on ART with suppressed viral load, as indicated in the study design table.<br><br>If 2 or more DLTs occur that are the same, similar, or in the same System Organ Class, dosing will be halted and the next lower dose level will be declared the maximum tolerated dose (MTD) within this group. If no DLT occurs in the final dose group, MTD will be the highest dose given (subgroups 1C and 2C 30mg/kg) after 14 days of follow-up.<br><br>The Protocol Safety Review Team (PSRT) will review safety data through at least day 14 post-IP administration for all participants in the 1st dose group (subgroups 1A and 2A) prior to allowing enrolment of participants into the 2nd dose group (subgroups 1B and 2B). The PSRT will review safety data through at least day 14 post-IP administration for all participants in the 2nd dose group (subgroups 1B and 2B) prior to allowing enrolment of participants into the 3rd dose group (subgroups 1C and 2C).

	Following IP administration of the last participant in both Groups 1 and 2, an independent Safety Monitoring Committee (SMC) will review safety data through at least day 14 post-IP administration for all participants to confirm MTD, and determine whether, and at what dose, Group 3 can initiate enrollment.<br><br>The second part of this study is a dose-de-escalation trial in HIV-infected adults not on ART, as indicated in the study design table.<br><br>The study will be paused for a safety review by the investigators and the independent SMC if 1) 1 or more participants experiences a Serious Adverse Event that is judged possibly, probably or definitely related to the IP, 2) There is a participant death assessed as possibly, probably or definitely related to the IP, 3) if 2 or more participants experience grade 3 adverse events in the same System Organ Class that are considered to be at least possibly related to IP or 4) any grade 4 adverse event. See protocol section 17.3.
**FORMULATIONS, VOLUMES AND ROUTES OF ADMINISTRATION**	**PGT121 mAb**: PGT121 mAb is a recombinant, fully human monoclonal antibody (mAb) of the IgG1 isotype that binds to the HIV envelope. The concentration and volume of product in each vial is 50 mg/mL, 6mL in each vial. PGT121 mAb will be given intravenously in this study.
**DURATION OF STUDY PARTICIPATION**	Participants will be screened up to 42 days before IP administration and will be followed for 24 weeks. The anticipated study duration for each participant is approximately 6 months from screening through last study visit. It is anticipated that it will take approximately 4.5 months to enroll Groups 1 and 2. It is anticipated that it will take approximately 8 months to enroll the entire study. The exact duration depends on the recruitment rate and how many participants will be required per dose group.
**RANDOMIZATION and BLINDING**	This is a randomized, placebo-controlled study for groups 1 and 2, and open label for group 3 who will not receive placebo.
**EVALUATION FOR INTERCURRENT HIV INFECTION:**	Participants in Group 1 (HIV-uninfected) will be tested for HIV according to the Schedule of Procedures. Test results will be interpreted according to a pre-determined diagnostic algorithm. HIV testing at additional time points may be performed upon the request of the participant and Principal Investigator or designee as medical or social circumstances warrant.
**SAFETY MONITORING AND STATISTICAL CONSIDE-RATIONS:**	All clinical trial data collected, identified only by a study identification number, will be entered into the clinical trial database.<br><br>Safety will continually be monitored by the Investigators, the Sponsor's Medical Monitor and a Protocol Safety Review Team (PSRT); detailed pause criteria are pre-defined.<br><br>Safety data will be reviewed by an independent Safety Monitoring Committee (SMC). *Ad hoc* safety review may be specifically requested by the Sponsor, the Principal Investigators, Ethics Committees, Regulatory Authorities, or by the SMC. All clinical and routine laboratory data will be included in the safety analysis. At the end of the study, a full analysis will be prepared.

TABLE OF CONTENTS

ABBREVIATIONS **11**

CONTACT INFORMATION **12**

1.0 SIGNATURE PAGE **13**

2.0 INTRODUCTION AND BACKGROUND INFORMATION **14**

2.1 Study Rationale 14
2.2 Experience with PGT121 15

3.0 STUDY OBJECTIVES **15**

3.1 Primary Objectives 15
3.2 Secondary Objectives 15
3.3 Exploratory Objectives: 15

4.0 STUDY ENDPOINTS **15**

4.1 Study Endpoints 15
4.1.1 Primary Endpoints 15
4.1.2 Secondary Endpoints 16
4.1.3 Exploratory Endpoints 17

5.0 STUDY DESIGN **17**

5.1 Definition of Dose Limiting Toxicity and Maximum Tolerated Dose 17
5.2 Dose Escalation – Groups 1 and 2: Determination of Maximum Tolerated Dose 17
5.3 Dose De-Escalation- Group 3: Determination of Minimum Effective Dose 18
5.4 Duration of the Study 20
5.5 Study Population 20
5.6 Inclusion Criteria 20
5.7 Exclusion Criteria 22
5.8 Recruitment of Participants 26

6.0 STUDY VISITS **26**

6.1 Screening Period 26
6.2 IV infusion of PGT121 mAb Visit 27
6.3 Post-IV infusion of PGT121 mAb Visits 28
6.4 Additional Follow-up Visits 28
6.5 Unscheduled Visits 28
6.6 Final Study Visit or Early Termination Visit 28

7.0 STUDY PROCEDURES **28**

7.1 Informed Consent Process 28
7.2 Medical History and Physical Examination 29
7.3 HIV Testing and HIV-test Counselling (Group 1) 29
7.4 HIV Risk Reduction Counselling 30
7.5 Family Planning Counselling 30
7.6 ART Counselling (Group 3) 30
7.7 Specimens 30
7.8 Reimbursement 30
7.9 Randomization and Blinding 31
7.10 Un-blinding Procedure for Individual Participants 31
7.11 Assessment of IP related HIV sero-positivity 31

8.0 INVESTIGATIONAL PRODUCT 32
8.1 Description 32
8.2 Shipment and Storage 32
8.3 Preparation of Investigational Product (IP) 33
8.4 Administration of Investigational Product 33
8.5 Accountability and Disposal of Investigational Product 33
9.0 ASSESSMENTS 33
9.1 Safety Assessments 33
9.1.1 Local reactogenicity 34
9.1.2 Systemic reactogenicity 34
9.1.3 Vital Signs 34
9.1.4 Other Adverse Events 34
9.1.5 Concomitant Medications 34
9.1.6 Routine laboratory parameters 34
9.1.7 Specific screening tests: 35
9.2 Virologic Assessments 35
9.3 Exploratory Immunogenicity Assessments 36
9.3.1 Antibody Responses 36
9.3.2 Cellular Responses 36
9.3.3 PBMC, Serum and Plasma Storage 36
9.4 Other Assessments 36
9.4.1 HIV Antibody Testing 36
9.4.2 Pharmacokinetics 37
9.4.3 HLA Typing 37
9.4.5 Pregnancy Test 37
9.4.6 HIV Risk Assessment (Group 1) 37
9.4.7 Social Impact Assessment 37
10.0 ADVERSE EVENTS 38
10.1 Definition 38
10.2 Assessment of Severity of Adverse Events 38
10.3 Relationship to Investigational Product 38
10.4 Serious Adverse Events 39
10.5 Reporting Potential Immune-Mediated Diseases 40
10.6 Clinical Management of Adverse Events 41
10.7 Pregnancy 41
10.8 Intercurrent HIV Infection (Group 1) 42
11.0 MANAGEMENT OF HIV ISSUES DURING AND FOLLOWING STUDY 42
11.1 HIV Testing – Group 1 42
11.2 Social Discrimination as a Result of IP-related antibodies 42
11.3 HIV infection – Group 1 43
11.3.1 Counselling 43
11.3.2 Referral for Support/Care 43
12.0 WITHDRAWAL FROM STUDY 43
12.1 Deferral of IV infusion of IP 43
12.2 Withdrawal from the Study (Early Termination) 44
13.0 DATA HANDLING 44
13.1 Data Collection and Record Keeping at the Study Site 44

13.3 Data Entry at the Study Site 44
13.4 Data Analysis 45
14.0 STATISTICAL CONSIDERATIONS 45
14.1 Safety and Tolerability Analysis 45
14.2 Pharmacokinetic Analysis 46
14.3 Virologic Analysis for Dose De-escalation in Groups 3A-C 47
14.4 Analysis of Antiviral Activity and Dose De-escalation in Subgroups 3D-F 49
14.5 Secondary and Exploratory Immunologic and Virologic Analyses 49
15.0 QUALITY CONTROL AND QUALITY ASSURANCE 50
16.0 DATA AND BIOLOGICAL MATERIAL 50
17.0 ADMINISTRATIVE STRUCTURE 50
17.1 Protocol Safety Review Team 50
17.2 Safety Monitoring Committee (SMC) 51
17.2.1 Content of Interim Safety Review 51
17.2.2 SMC Review of Group 1 and 2 data prior to starting Group 3 51
17.3 Criteria for Pausing the Study 52
17.4 Study Supervision 53
17.5 Study Monitoring 53
17.6 Investigator's Records 53
18.0 INDEMNITY 53
19.0 PUBLICATION 54
20.0 ETHICAL CONSIDERATIONS 54
APPENDIX A: SCHEDULE OF PROCEDURES 55
APPENDIX B: LOW RISK CRITERIA 58
Laboratory Values 77
Chemistries 77
Hematology 80
Urinalysis 82

ABBREVIATIONS

Abbreviation	Term
**AE**	Adverse Event
**AIDS**	Acquired Immunodeficiency Syndrome
**ALT**	Alanine-Aminotransferase
**ART**	Antiretroviral Therapy
**AST**	Aspartate-Aminotransferase
**CFC**	Cytokine Flow Cytometry
**CMI**	Cell Mediated Immunity
**CRF**	Case Report Form
**CTL**	Cytotoxic T Lymphocyte
**DCC**	Data Coordinating Center
**DLT**	Dose Limiting Toxicity
**DNA**	Deoxyribonucleic Acid
**ELISA**	Enzyme Linked Immunosorbent Assay
**GCP**	Good Clinical Practice
**GFR**	Glomerular Filtration Rate
**HIV**	Human Immunodeficiency Virus
**HLA**	Human Leukocyte Antigen
**HSV**	Herpes Simplex Virus
**IAVI**	International AIDS Vaccine Initiative
**IDES**	Internet Data Entry System
**ICH**	International Council for Harmonization of Technical Requirements for Pharmaceuticals for Human Use
**IP**	Investigational Product
**IND**	Investigational New Drug Application
**IV**	Intravenous
**Kg**	Kilogram
**mAb**	Monoclonal Antibody
**mg**	Milligram
**MED**	Minimum Effective Dose
**MTD**	Maximum Tolerated Dose
**PCR**	Polymerase Chain Reaction
**PBMC**	Peripheral Blood Mononuclear Cells
**PK**	Pharmacokinetic
**RPR**	Rapid Plasma Reagin
**SAE**	Serious Adverse Event
**SIV**	Simian Immunodeficiency Virus
**SOP**	Standard Operating Procedure
**SOM**	Study Operations Manual
**SMC**	Safety Monitoring Committee
**STD**	Sexually Transmitted Disease
**TPHA**	Treponema Pallidum Hemagglutination

CONTACT INFORMATION

Detailed contact information provided in the Study Operation Manual (SOM)

**Sponsor Contact:**	
Frances Priddy MD MPH<br>Executive Director and Chief Medical Officer<br>International AIDS Vaccine Initiative<br>125 Broad Street, 9th Floor<br>New York, New York 10004	Phone: +1-212-328-7461<br>Mobile: +1-646-287-8943<br>Fax: +1-608-203-5501<br>E-mail: fpriddy@iavi.org
**Clinical Research Center Contacts:**	
Kathryn Stephenson MD MPH<br>Center for Virology and Vaccine Research<br>Clinical Trials Unit<br>Beth Israel Deaconess Medical Center<br>E / CLS – 1036<br>330 Brookline Avenue<br>Boston, Massachusetts 02215	Phone: +1-617-735-4556<br>Mobile: +1-917-836-9150<br>Fax: +1-617-735-4566<br>E-mail: kstephen@bidmc.harvard.edu

.

1.0 SIGNATURE PAGE

The signatures below constitute the approval of this protocol and the appendices and provide the necessary assurances that this study will be conducted in compliance with the protocol, Good Clinical Practices (GCP) and the applicable regulatory requirement(s).

Sponsor:

Signed: See electronic signature manifest Date: August 5, 2016

Frances Priddy MD MPH
Executive Director and Chief Medical Officer, Medical Affairs, IAVI

Principal Investigator:

Signed: Date:

Name (please print):

Name of institution (please print):

2.0 INTRODUCTION AND BACKGROUND INFORMATION

More than 78 million people have been infected with HIV and 39 million people have died since the beginning of the AIDS epidemic[1]. In 2014, there were 1.2 million deaths attributable to HIV infection and 2 million newly infected with HIV[2]. One reason that such high rates of AIDS-related deaths continue to occur globally – despite the advent of drugs that are highly effective at suppressing HIV replication – is that only two in five people living with HIV have access to antiretroviral therapy[1]. The other reason for continued AIDS-related mortality is that ART does not cure HIV infection and must be maintained for a lifetime. Even in the United States (US), only 30% of the 1.2 million people living with HIV have suppressed HIV to undetectable levels, despite the fact that most HIV-infected people in the US have access to ART[3] It is clear that antiretroviral therapy is necessary but not sufficient to end the AIDS epidemic, both in the US and globally, and that novel efforts to prevent and treat HIV are critically needed.

2.1 Study Rationale

This is a Phase 1 study to evaluate the safety, tolerability, pharmacokinetics and anti-viral efficacy of the PGT121 monoclonal antibody for HIV prevention and therapy. PGT121 mAb is a recombinant human IgG1 monoclonal antibody that targets a V3 glycan-dependent epitope region of the HIV envelope protein[4,5]. PGT121 mAb was chosen for this study because it is potent, neutralizes a wide array of HIV viruses, and can prevent and treat simian-human immunodeficiency virus (SHIV) in rhesus monkeys.

The recent discovery of multiple potent and broadly neutralizing antibodies (bNAbs) against HIV has led to the re-emergence of the concept that antibodies may be useful for both prevention and therapy. HIV-specific antibodies that target the HIV envelope (Env) can prevent SHIV infection in rhesus monkeys and have shown to reduce HIV RNA levels in humans temporarily[6-10]. Until recently, these antibodies were few in number, targeted a narrow spectrum of HIV strains and Env epitopes, and were not potent enough for practical use. In the last five years, the field has changed dramatically: new developments in high throughput single-cell PCR-amplification and novel soluble Env baits have led to the isolation of new monoclonal antibodies with extraordinary potency and breadth[11]. These bNAbs may be effective for prevention of HIV infection when administered passively[12,13].

PGT121 mAb was selected for development because of the following critical attributes:

- PGT121 mAb is 10 to 100-fold more potent than the previous best-in-class CD4bs antibodies VRC01, VRC07, and 3BNC117 [11,14,15].
- PGT121 mAb affords superior protective efficacy against SHIV acquisition in monkeys compared to VRC01, 3BNC117, and 10-1074 [16] (and unpublished data).
- PGT121 mAb has superior therapeutic efficacy in SHIV-infected monkeys compared to VRC01, 3BNC117, and 10-1074 [7] (and unpublished data).
- PGT121 mAb may have a higher bar to escape in vivo as compared with other V3 glycan and CD4bs antibodies as a result of making multiple glycan contacts [14].
- PGT121 mAb combined with PGDM1400 (a novel bNab targeting the envelope trimer apex) neutralizes 98-99% of global HIV-1 viruses tested and has unparalleled potency with a median IC50 of 0.007 µg/ml[14].

The potency and breadth of PGT121 mAb, both alone and in combination with other bNAbs, raise the possibility that combinations may be effective for HIV prophylaxis at

low doses and against global viruses. An antibody that is effective at low doses may eventually be given subcutaneously, which would reduce the cost. It is these features that make PGT121 mAb particularly well-suited for preventing and/or treating HIV in the developing world, where it is critical that a public health intervention be low cost, easy to deliver, and effective in diverse settings.

2.2 Experience with PGT121

There is no previous clinical experience with PGT121 mAb. Several other HIV monoclonal antibodies are currently in clinical development as passive HIV immunoprophylaxis, or as potential therapeutics. Data from phase 1 studies shows acceptable preliminary safety and tolerability profiles for these products, but varying levels of anti-viral effects [6,17]. A comprehensive summary of phase 1 studies of HIV monoclonal antibodies can be found in the Investigator's Brochure.

3.0 STUDY OBJECTIVES

3.1 Primary Objectives

- To evaluate the safety and tolerability of a single intravenous (IV) infusion of PGT121 mAb at 3, 10, and 30 mg/kg in HIV-uninfected adults and HIV-infected adults.
- To evaluate the pharmacokinetic (PK) profile of IV infusion of PGT121 mAb in HIV-uninfected and HIV-infected adults.
- To evaluate the antiviral activity of IV infusion of PGT121 mAb in HIV-infected adults not on ART.

3.2 Secondary Objectives

- To determine if PGT121 mAb induces anti-PGT121 antibodies.
- To determine the effect of PGT121 mAb on CD4 T-cell counts in HIV-infected adults.
- To determine if PGT121 mAb induces viral escape mutants in viremic HIV-infected adults not on ART.

3.3 Exploratory Objectives:

- To determine if PGT121 mAb has any impact on the host immune responses (i.e., HIV-specific cellular and humoral immune response).
- To determine the effect of PGT121 mAb on the size of the latent HIV reservoir in HIV-infected adults.
- To determine PGT121 mAb levels in mucosal secretions in HIV-uninfected and HIV-infected adults.
- To measure in vitro neutralization of HIV isolates with participant's serum post PGT121 mAb IV infusion.
- To determine if PGT121 mAb has any impact on resistance mutations to ARVs.

4.0 STUDY ENDPOINTS

4.1 Study Endpoints

4.1.1 Primary Endpoints

Safety and Tolerability:

We will measure the following endpoints to evaluate the safety and tolerability of PGT121 mAb in HIV-uninfected and HIV-infected adults:

1. Proportion of participants with moderate or greater reactogenicity (e.g., solicited adverse events) for 3 days following IV infusion of PGT121 mAb.
2. Proportion of participants with moderate or greater and/or PGT121 mAb-related unsolicited adverse events (AEs), including safety laboratory (biochemical, hematological) parameters, following IV infusion of PGT121 mAb for the first 56 days post administration of Investigational Product.
3. Proportion of participants with PGT121 mAb-related serious adverse events (SAEs) throughout the study period.

Pharmacokinetics:

We will measure the following endpoints to determine the pharmacokinetics following IV infusion of PGT121 mAb in HIV-uninfected and HIV-infected adults:

1. Elimination half-life (t1/2)
2. Clearance (CL/F)
3. Volume of distribution (Vz/F)
4. Area under the concentration decay curve (AUC)
5. Impact of viral load and/or ART on PGT121 disposition (elimination half-life (t1/2), clearance (CL/F), volume of distribution (Vz/F), total exposure

Antiviral activity:

We will calculate the following endpoints to determine the antiviral activity following IV infusion of PGT121 mAb in viremic HIV-infected adults not on ART.

1. Change in plasma HIV-1 RNA levels from baseline (mean of pre-entry and entry values)

4.1.2 Secondary Endpoints

Anti-PGT121 antibodies:

We will calculate the following endpoint to determine if anti-PGT121 antibodies are elicited following IV infusion of PGT121 mAb in HIV-uninfected and HIV-infected adults:

1. Change in serum anti-PGT121 antibody titers from baseline

CD4+ T cell count:

We will calculate the following endpoint to determine if IV infusion of PGT121 mAb has any impact on CD4+ T cell counts in HIV-infected adults:

1. Change in CD4+ T cell count and frequency compared to baseline as measured by single platform flow cytometry

HIV genotyping/phenotyping of circulating virus for evaluation of PGT121-induced escape mutations:

We will compare plasma virus sequences before and after IV infusion of PGT121 mAb to determine if PGT121 mAb induces viral escape mutations in viremic HIV-infected adults not on ART.

1. Development of sequence variations in epitopes known to result in reduced PGT121 neutralization susceptibility

4.1.3 Exploratory Endpoints

Additional assessments may include but are not limited to the following: HIV-specific IgG/IgA binding responses by ELISA, HIV-specific cellular immune responses by ELISPOT, HIV-specific antibody function by ADCC, ADCP, and ADCVI assays, resistance mutations to current ARVs, PGT121 mAb levels in mucosal secretions, changes in total HIV-1 DNA and 2-long terminal repeat (LTR) circular HIV-1 DNA in resting or total CD4 T cells and in vitro neutralization of HIV isolates with participant's serum post IV infusion of PGT121 mAb.

5.0 STUDY DESIGN

The study is a randomized, placebo-controlled study for groups 1 and 2, and open label for group 3 who will not receive placebo.

5.1 Definition of Dose Limiting Toxicity and Maximum Tolerated Dose

A dose limiting toxicity (DLT) will be defined as 1) any Grade 3 or greater reactogenicity or adverse event judged by the study investigators as at least possibly related to investigational product or 2) any SAE considered at least possibly related investigational product.
Maximum Tolerated Dose (MTD) will be declared when 2 or more DLTs occur that are the same, similar, or in the same System Organ Class or if no DLT occurs in the final dose subgroup, MTD will be the highest dose given (groups 1C and 2C 30mg/kg) after 14 days of follow-up.

5.2 Dose Escalation – Groups 1 and 2: Determination of Maximum Tolerated Dose

In Groups 1 and 2, (Part 1), the administrations of PGT121 mAb escalate by dose as shown below in Table 5.3.1, Study Design (5 participants per dose subgroup, 4:1 ratio of IP to placebo for each dose subgroup).

Sentinel Recipients

Within each dose group (subgroups 1A and 2A, subgroups 1B and 2B, subgroups 1C and 2C), the first 3 participant infusions will be separated by at least 4 days, to allow for observation of Investigational product (IP)-related adverse events. Dose subgroups will be enrolled in parallel, meaning that the 1st participant may be from subgroup 1A, the 2nd from subgroup 2A, the 3rd from subgroup 2A, all with 4 days in between dosing.

Because there is 1 placebo in each dose subgroup and the subgroups are dosed in parallel, the first 3 recipients will be treated as sentinel recipients (at least 1 will receive the IP). If no reactogenicity and adverse events that are considered to be related to IP (possibly, probably or definitely related) and are graded as severe or worse (Grade 3 or 4 on the DAIDS Toxicity Table) occur within 4 days after infusion, the second participant may be injected. If no events meeting the criteria described above occur within 4 days after the 3rd participant is infused, then the remainder of participants in that dose group will be infused. If events meeting the criteria described above do occur for the first 3 participants in a dose group, they will be reviewed by the Safety Monitoring Committee (SMC) to determine whether further infusions may proceed.

Dose Escalation and Determination of Maximum Tolerated Dose

Safety data through day 14 post-IP administration visit for all participants in the first dose group (1A and 2A) will be reviewed by the Protocol Safety Review Team (PSRT) prior to allowing enrollment of participants into the second dose group (1B and 2B). The review process will be repeated between the second and third (1C and 2C) dose groups. Following administration of IP for the last participant in both Groups 1 and 2, an independent Safety Monitoring Committee (SMC) will review safety data to confirm Maximum Tolerated Dose (MTD) and determine whether, and at what dose, Group 3 can initiate enrollment.

Within each group, if no DLT occurs within 2 weeks from infusion of the first 5 participants in a dose group, dose escalation to the next dose group will proceed. If 1 DLT occurs, 3 additional participants will be enrolled; randomized between PGT121 mAb and placebo at a 2:1 ratio. If no additional DLTs occur within 2 weeks of infusion in the 8 mAb total participants (3:1 ratio of PGT121 mAb to placebo), the study can proceed with enrollment of the next dose group. If 2 or more DLTs accumulate in a subgroup that are the same, similar, or in the same organ class, dosing will be halted and the next lower dose level will be declared the maximum tolerated dose (MTD). If no DLT occurs in the final dose group, MTD will be the highest dose given (subgroups 1C and 2C 30mg/kg) after 14 days of follow-up.

5.3 Dose De-Escalation- Group 3: Determination of Minimum Effective Dose

Upon approval by the SMC (see section 17.2.2), group 3 (Part 2), PGT121 mAb administrations will de-escalate by dose as shown below in Table 5.3.1.

Group 3 will start with the MTD (i.e. subgroups 3A and 3D if the MTD is 30 mg/kg, subgroups 3B and 3C if the MTD is 10 mg/kg, or subgroups 3C and 3F if the MTD is 3 mg/kg) as determined by the SMC from data in Part 1.

If subgroup 3A (n = 6) achieves a mean decline in HIV RNA of $\geq$0.9 log compared to baseline, enrollment into subgroup 3A will be stopped, and the next lower dose subgroup 3B will be initiated. The same rule will apply for progression from subgroup 3B to 3C. At least 6, 8 and 10 but up to 9, 12 and 15 participants will be enrolled in subgroups 3A, 3B and 3C respectively, until the minimum effective dose is determined. In each subgroup, if a mean decline >0.9 log in HIV RNA is not achieved, enrollment will be stopped at the completion of enrollment at that dose level.

Three participants will be enrolled in each group 3D, 3E and 3F. As soon as subgroup 3D has enrolled 3 participants, enrollment into subgroup 3E will be initiated. The same rule will apply for progression from subgroup 3E to 3F. In the event that dose de-escalation in subgroups 3A-C is stopped due to failure to meet virologic endpoints, dose de-escalation in subgroups 3D-F will continue.

Table 5.3.1 Study Design Table

	Group	Participants	Sub-Group	Regimen	N	Dose (mg/kg)
Part 1	1	HIV-uninfected participants	1A	PGT121/Placebo	4/1 (6/2 if DLT)	3
			1B	PGT121/Placebo	4/1 (6/2 if DLT)	10
			1C	PGT121/Placebo	4/1 (6/2 if DLT)	30
	2	HIV-infected on ART, (<50 cp/ml)	2A	PGT121/Placebo	4/1 (6/2 if DLT)	3
			2B	PGT121/Placebo	4/1 (6/2 if DLT)	10
			2C	PGT121/Placebo	4/1 (6/2 if DLT)	30
Safety Monitoring Committee Review						
Part 2	3	HIV-infected off ART (VL $2x10^3$ – $1x10^5$cp/ml)	3A	PGT121	6 (max 9)	30
			3B	PGT121	8 (max 12)	10
			3C	PGT121	10 (max 15)	3
		HIV-infected off ART (VL $1x10^2$ – $2x10^3$ cp/ml)	3D	PGT121	3	30
			3E	PGT121	3	10
			3F	PGT121	3	3

Table 5.3.2 Decision Tree, Groups 3A, 3B, 3C

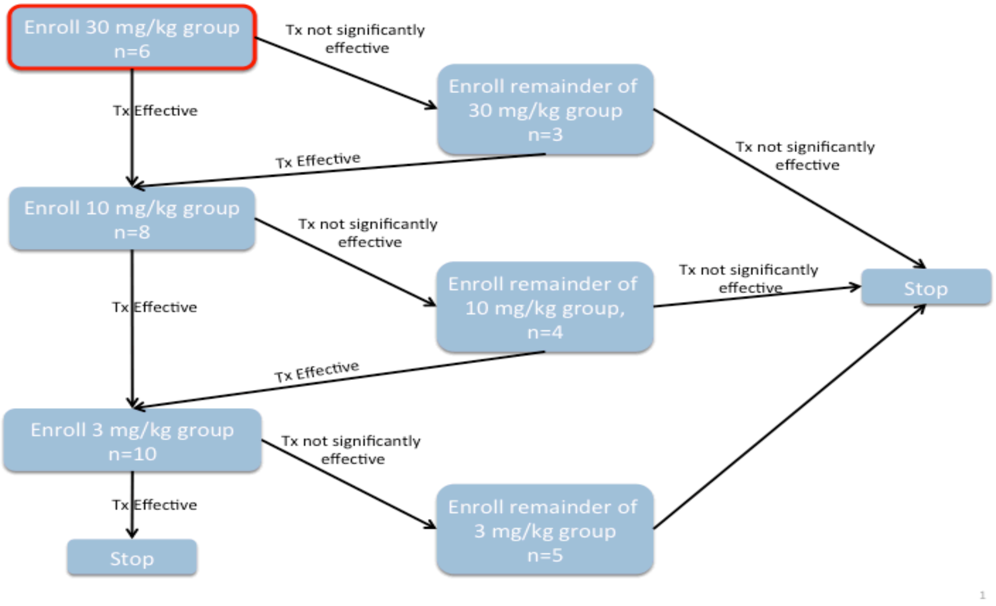

"not significantly effective" = does not achieve mean decrease of ≥0.9 log HIV RNA

5.4 Duration of the Study

Participants will be screened up to 42 days before IP administration of PGT121 mAb and will be followed for 24 weeks.

It will take approximately 8 months to enroll the entire study. The exact duration depends on the recruitment rate and how many participants will be required per dose group as specified in sections 5.2 and 5.3.

5.5 Study Population

The study population consists of HIV-uninfected male or female adults (Group 1), HIV-infected male or female adults on ART (Group 2), and HIV-infected males and female adults not on ART (group 3) who meet the detailed inclusion and exclusion criteria listed below, and who in the opinion of the investigator or designee, understand the study and provide written informed consent.

Approximately 63-93 participants (81 investigational product recipients, 12 placebo recipients) who meet all eligibility criteria will be included in the study. An over-enrollment of up to 5% (up to 5 participants total) will be permitted in the study to facilitate rapid enrollment.

5.6 Inclusion Criteria

Inclusion criteria for all participants:

1. At least 18 years of age on the day of screening and has not reached his or her 51st birthday on the day of signing the Informed Consent Document.

2. Willing to comply with the requirements of the protocol and available for follow-up for the planned duration of the study;

3. In the opinion of the Principal Investigator or designee and based on Assessment of Informed Consent Understanding results, has understood the information provided and potential impact and/or risks linked to IV infusion and participation in the trial; written informed consent will be obtained from the participant before any study-related procedures are performed;

4. All heterosexually active female participants must commit to use an effective method of contraception for 3 months following IP administration, including:
    a. Condoms (male or female) with or without spermicide
    b. Diaphragm or cervical cap with spermicide
    c. Intrauterine device, or contraceptive implant
    d. Hormonal contraception
    e. Successful vasectomy in the male partner (considered successful if a woman reports that a male partner has [1] documentation of azoospermia by microscopy (< 1 year ago), or [2] a vasectomy more than 2 years ago with no resultant pregnancy despite sexual activity post-vasectomy)
    f. Not be of reproductive potential, such as having undergone hysterectomy, bilateral oophorectomy, or tubal ligation, postmenopausal (>45 years of age with amenorrhea for at least 2 years, or any age with amenorrhea for at least 6 months and a serum follicle stimulating hormone [FSH] level >40 IU/L); surgically sterile: no additional contraception required.

   Women, who are not heterosexually active at screening, must agree to utilize an effective method of contraception if they become hetero-sexually active, as outlined above.

5. All sexually active males, regardless of reproductive potential, must be willing to consistently use an effective method of contraception (such as consistent male condoms with male and/or female partners from the day of IP administration until at least 3 months following IP administration to avoid exposure of partners to IP in ejaculate, and to prevent conception with female partners.

6. All female participants must be willing to undergo urine pregnancy tests at time points indicated in the Schedule of Procedures and must test negative prior to IP administration;

7. A woman must agree not to donate eggs (ova, oocytes) for the purpose of assisted reproduction until 3 months after receiving IP administration. A man must agree not to donate sperm until 3 months after IP administration;

8. Willing to forgo donations of blood and/or any other tissues, including bone marrow, during the study and, for those HIV-uninfected participants who test HIV-positive due to IP administration, until the anti-HIV antibody titers become undetectable.

Specific inclusion criteria for HIV-uninfected participants (Group 1):

9. Willing to undergo HIV testing, risk reduction counselling and receive HIV test results;

10. Low risk for HIV infection (see section (9.4.6) and willing to maintain low-risk behaviour for the duration of the trial (Appendix B);

11. Healthy male or female, as assessed by a medical history, physical exam, and laboratory tests;

Specific inclusion criteria for HIV-infected participants (Groups 2 and 3):

12. Confirmed HIV-1 infection (HIV Ab+ or HIV RNA+) by documentation in the medical records or in-clinic HIV testing;

13. CD4 ≥ 300 cells/µl;

14. No history of AIDS-defining illness or CD4 < 200 cells/µl.

Group 2:

15. Currently on ART, and documentation of continuous combination ART (cART) treatment with suppression of plasma HIV-1 viral load < 50 copies / ml for greater than 6 months, measured on at least 2 independent occasions, and with a viral load < 50 copies / ml at time of screening (within 42 days prior to IP administration). cART is defined as a regimen including > 2 compounds, e.g. 2x nucleoside reverse transcriptase inhibitors plus either non-nucleoside reverse transcriptase inhibitor or protease inhibitor or integrase inhibitor.

Group 3:

16. Not receiving cART, and (after appropriate counselling) willing to defer cART treatment for at least 56 days after administration of IP;

17. HIV-1 viral load either between 2000-100,000 copies / ml (Group 3A, 3B, 3C) or between 100-2000 copies / ml (Group 3D, 3E and 3F) at 2 independent occasions within 12 months prior to study enrollment, with confirmation during the screening period (3 viral loads on independent occasions).

5.7 Exclusion Criteria

Exclusion criteria for all participants:

1. Any clinically significant acute or chronic medical condition, other than HIV infection, that is considered progressive or in the opinion of the investigator makes the participant unsuitable for participation in the study;

2. If female, pregnant, lactating or planning a pregnancy during the period of screening through completion of the study;

3. In the past 6 months a history of alcohol or substance use, including marijuana, judged by the Investigator to potentially interfere with participant study compliance;

4. Bleeding disorder that was diagnosed by a physician (e.g., factor deficiency, coagulopathy or platelet disorder that requires special precautions). Note: A participant who states that he or she has easy bruising or bleeding, but does not have a formal diagnosis and has intramuscular injections and blood draws without any adverse experience, is eligible;

5. History of a splenectomy;

6. Receipt of live attenuated vaccine within the previous 60 days or planned receipt within 60 days after administration of IP; or receipt of other vaccine within the previous 14 days or planned receipt within 14 days after infusion with IP (exception is live attenuated influenza vaccine within 14 days);

7. Receipt of blood transfusion or blood-derived products within the previous 3 months;

8. Participation in another clinical trial of an investigational product currently, within the previous 3 months or expected participation during this study;

9. Prior receipt of an investigational HIV vaccine candidate, monoclonal antibody or polyclonal immunoglobulin (note: receipt of placebo in a previous HIV vaccine or monoclonal antibody trial will not exclude a participant from participation if documentation is available and the Medical Monitor gives approval);

10. History of severe local or systemic reactogenicity to injections or IV infusion (e.g., anaphylaxis, respiratory difficulties, angioedema);

11. HIV-specific antibodies that significantly cross-react with PGT121 mAb pharmacokinetic assays;

12. Psychiatric condition that compromises safety of the participant and precludes compliance with the protocol. Specifically excluded are persons with psychoses within the past 3 years, ongoing risk for suicide, or history of suicide attempt or gesture within the past 3 years;

13. If, in the opinion of the Principal Investigator, it is not in the best interest of the participant to participate in the trial;

14. Seizure disorder: a participant who has had a seizure in the last 3 years is excluded. (Not excluded: a participant with a history of seizures who has neither required medications nor had a seizure for 3 years.)

15. Body mass index ≥ 30 or ≤ 18.0.

16. Infectious disease: chronic hepatitis B infection (HbsAg), current hepatitis C infection (HCV Ab positive and HCV RNA positive) or interferon-alfa treatment for chronic hepatitis C infection in the past year, or active syphilis (RPR confirmed by TPHA).

17. A history of malignancy within the past 5 years (prior to screening) or ongoing malignancy;

18. Active, serious infections (other than HIV-1 infection) requiring parenteral antibiotic, antiviral or antifungal therapy within 30 days prior to enrollment;

Specific exclusion criteria for HIV-uninfected participants (Group 1):

19. Confirmed HIV-1 or HIV-2 infection;

20. Any clinically relevant abnormality on history or examination including history of immunodeficiency or autoimmune disease; use of systemic corticosteroids, immunosuppressive, anticancer, or other medications considered significant by the investigator within the previous 6 months.

    The following exceptions are permitted and will not exclude study participation: use of corticosteroid nasal spray for rhinitis, topical corticosteroids for an acute uncomplicated dermatitis; or a short course (duration of 10 days or less, or a single injection) of corticosteroid for a non-chronic condition (based on investigator clinical judgment) at least 6 weeks prior to enrollment in this study.

Specific exclusion criteria for HIV-uninfected participants (Group 1) and HIV-infected participants who are on ART (Group 2):

21. Any of the following abnormal laboratory parameters listed below:

<u>Hematology</u>

– Hemoglobin <10.5 g/dL in females; hemoglobin <11.0 g/dL in males
– Absolute Neutrophil Count (ANC): ≤1000/mm^3
– Absolute Lymphocyte Count (ALC): < 650/mm^3
– Platelets: < 125,000 mm^3 or ≥ 550,000/mm^3

<u>Coagulation</u>

- aPTT: >1.25x ULN
- INR: ≥1.1 x ULN

<u>Chemistry</u>

- Sodium ≤135 mEq/L or ≥ 146 mEq/L
- Potassium ≤3.4 mEq/L or ≥ 5.6 mEq/L
- Creatinine ≥1.1 x ULN
- AST ≥1.25 x ULN
- ALT ≥1.25 x ULN
- Total bilirubin ≥1.25 x ULN
- Alkaline phosphatase ≥1.25 x ULN
- Albumin ≤ 3.0 g/dL or ≤ 30 g/L
- Creatine kinase ≥3.0 x ULN
- C-reactive protein >10 mg/L
- C3 complement ≤ 0.9g/L
- C4 complement ≤ 0.1g/L

Urinalysis

Clinically significant abnormal dipstick confirmed by microscopy:
– Protein = 1+ or more
– Blood = 1+ or more (not due to menses)

Specific exclusion criteria for HIV-infected participants who are on ART (Group 2) and for HIV-infected participants who are not on ART (Group 3):

22. Any clinically relevant abnormality on history or examination including history of immunodeficiency or autoimmune disease, other than HIV; use of systemic corticosteroids, immunosuppressive, anticancer, or other medications considered significant by the investigator within the previous 6 months.

    The following exceptions are permitted and will not exclude study participation: use of corticosteroid nasal spray for rhinitis, topical corticosteroids for an acute uncomplicated dermatitis; or a short course (duration of 10 days or less, or a single injection) of corticosteroid for a non-chronic condition (based on investigator clinical judgment) at least 6 weeks prior to enrollment in this study.

Specific exclusion criteria for HIV-infected participants who are not on ART (Group 3)

23. Resistance of autologous HIV to PGT121 neutralization in vitro;

24. Any of the following abnormal laboratory parameters listed below:

Hematology

– Hemoglobin < 10.0 g/dL
– Absolute Neutrophil Count (ANC): <800 cells/mm^3
– Platelets: < 100,000 cells/mm3

Coagulation

- aPTT: >1.25x ULN
- INR: ≥1.1 x ULN

Chemistry

- Estimated Glomerular filtration rate (GFR) ≤ 80 mL/min according to the Cockcroft-Gault formula for creatinine clearance:
    - Male: $\frac{(140 - \text{age in years}) \times (\text{wt in kg})}{72 \times (\text{serum creatinine in mg/dL})} = \text{CLcr (mL/min)}$
    - Female: $\frac{(140 - \text{age in years}) \times (\text{wt in kg}) \times 0.85}{72 \times (\text{serum creatinine in mg/dL})} = \text{CLcr (mL/min)}$
- AST ≥ 2.5 x ULN

- ALT ≥ 2.5 x ULN
- Total bilirubin ≥ 1.6 x ULN
- Alkaline phosphatase ≥ 5 x ULN

Urinalysis

- Any RBC, protein or leukocytes greater than 1+, confirmed by microscopy and consistent with clinically significant disease.

5.8 Recruitment of Participants

Adult male and female participants may be recruited through in-clinic referrals, information presented to community organizations, hospitals, colleges, other institutions and/or advertisements to the general public or from existing cohorts. The information distributed will contain contact details of the trial site.

6.0 STUDY VISITS

6.1 Screening Period

During Screening, study staff will perform the following procedures:

- Provide and/or review the Informed Consent Document and answer any questions about the study prior to obtaining written informed consent.
- Complete Assessment of Informed Consent Understanding (AOU). Please refer to the Study Operations Manual (SOM)

If the participant agrees to participate, passes the AOU and provides written informed consent, study staff will:

- Conduct HIV test counselling, HIV testing, and HIV risk reduction counselling, as applicable
- Conduct family planning counselling, refer for pregnancy prevention counselling if necessary
- Conduct ART counselling, if applicable
- Perform a comprehensive medical history
- Collect concomitant medication information
- Perform a general physical examination (Refer to Section 7.2)
- Collect specimens for all tests as indicated in the Schedule of Procedures in Appendix A (for details see Analytical Plan (AP).

When available, the screening laboratory tests will be reviewed by the trial physician. Screening laboratory test(s) may be repeated once at the discretion of the principal investigator or designee to investigate any isolated abnormalities.

If the screening visit occurs more than 42 days prior to the date of administration of IP, all screening procedures must be repeated except the comprehensive medical history may be replaced by an interim medical history and the Participant Information Sheet of the Informed Consent Document should be reviewed.

If a participant has signed the Consent Form but does not meet the eligibility criteria, the records must be kept at the site.

6.2 IV infusion of PGT121 mAb Visit

Prior to the administration of IP, study staff will:

- Answer any questions the participant may have about the study
- Review the Informed Consent Document with the participant
- Review screening safety laboratory data
- Administer HIV risk assessment (Group 1)
- Conduct HIV test counselling, and HIV risk reduction counselling
- Conduct family planning counselling as per site specific procedures and ensure compliance with respective pregnancy prevention method, and discuss male condom use with all male participants
- Review interim medical history
- Collect concomitant medication information
- Weigh participant and record vital signs
- Perform a symptom-directed physical examination (Refer to Section 7.2)
- Assess at baseline local and systemic signs and symptoms (this includes an examination of IV infusion site)
- Collect specimens for all tests as indicated in the Schedule of Procedures see Appendix A (for details see AP).
- Obtain pregnancy test results prior to administration of IP.

Assign an allocation number to the participant according to the instructions specified in the Study Operations Manual.

At the time of administration of IP and after IV infusion of IP, study staff will:

- Administer the IP as specified in Section 8.4, Administration of Investigational Product and according to the instructions specified in the SOM.
- Observe participant closely during the infusion of IP and for at least 30 minutes after IV infusion of IP has ended for any acute reactogenicity. At the end of the observation period study staff will:
  - Record vital signs (pulse, respiratory rate, blood pressure and temperature)
  - Assess any local and systemic reactogenicity
  - Assess any other adverse events
- Every hour after IV infusion of IP, starting hour 1 through 12, the study staff will:
  - Record vital signs (pulse, respiratory rate, blood pressure and temperature)
  - Assess any local and systemic reactogenicity
  - Assess any other adverse events
  - Collect PK samples according to the Schedule of Procedures

If a participant has an abnormal laboratory value that is known, at the time of infusion, follow the specified guidelines (Section 12.0).

6.3 Post-IV infusion of PGT121 mAb Visits

The participant will be asked to return to the clinic for post-IP administration visits as indicated in the Schedule of Procedures (see Appendix A) for an assessment by clinic staff. The participant will be asked to maintain a Memory Aid for local and systemic reactogenicity from the day of IP administration for the next 3 days (for a total of 4 days including day of IP administration). Study staff will review the Memory Aid with the participant and determine the severity of the reactions through discussion with the participant.

The following procedures will be conducted at these visits:

- Review interim medical history
- Collect concomitant medication information
- Perform a symptom-directed physical examination if any signs or symptoms are present
- Assess vital signs (pulse, respiratory rate, blood pressure and temperature)
- Assess any adverse events and local and systemic reactogenicity (Days 1, 2, 3) including reviewing the Memory Aid.
- Collect specimens for all tests as indicated in the Schedule of Procedures (Appendix A) and AP).

6.4 Additional Follow-up Visits

Assessments and procedures will be performed according to the Schedule of Procedures (Appendix A).

6.5 Unscheduled Visits

Unscheduled Visits/Contacts are visits/contacts that are not described in the Schedule of Procedures (Appendix A). Unscheduled visits may occur any time during the study:

- For administrative reasons, e.g., the participant may have questions for study staff or may need to re-schedule a follow-up visit.
- To obtain laboratory test results from a previous visit.
- For other reasons as requested by the participant or site investigator.

All unscheduled visits will be documented in the participants' study records on applicable source documents and entered into the Case Report Form (CRF).

6.6 Final Study Visit or Early Termination Visit

Assessments and procedures will be performed according to the Schedule of Procedures (Appendix A).

7.0 STUDY PROCEDURES

7.1 Informed Consent Process

A Master Informed Consent Document consisting of a Participant Information Sheet and a Consent Form is provided by the Sponsor to the trial site. This document is made site-specific and translated (if necessary), submitted and approved by the Institutional

Review Board (IRB). The Master and site specific Informed Consent Documents are separate documents and should not be part of the protocol.

Participant Information Sheet

A qualified member of the study staff will conduct the informed consent process by reviewing the Participant Information Sheet and document it in the clinic notes.

Consent Form

The participant's consent to participate must be obtained by him/her signing and dating the Consent Form. The person obtaining consent will also sign.

The signed and dated Informed Consent Document must remain at the study site. A copy of the signed/marked and dated Informed Consent Document will be offered to the participant to take home. Those participants who do not wish to take a copy will be required to document that they declined to do so.

7.2 Medical History and Physical Examination

Medical History

At screening, a comprehensive medical history will be collected including previous IV infusions and reaction to IV infusion, history of sexually transmitted infection (STI) and pregnancy prevention practices. At subsequent visits, an interim medical history will be performed.

Physical Examination

General Physical Examination

A general physical examination includes examination of head/ears/eyes/nose and throat, skin, respiratory, cardiovascular, abdominal, limited neurological and musculoskeletal and external ano-genital systems (for HIV-infected participants only) at the time points indicated in the Schedule of Procedures (see Appendix A).

Symptom-Directed Physical Examination

A symptom-directed physical examination is a targeted examination based on the participant’s history or observation. If deemed necessary, this examination should be done at the time points indicated in the schedule of procedures (see Appendix A).

Measuring Height and Weight

Includes measuring the height and weight at the time points indicated in the Schedule of Procedures (see Appendix A).

Vital Signs

Vital signs including pulse, respiratory rate, blood pressure and temperature are measured and recorded at the time points indicated in the Schedule of Procedures (see Appendix A)

7.3 HIV Testing and HIV-test Counselling (Group 1)

Study staff will perform pre-HIV test counselling prior to collecting blood for an HIV test, and post-HIV test counselling when HIV test results are available. This is referred to as

HIV-test counselling, and done according to the CDC guidelines. For more information on HIV testing and HIV-test counselling, see Section 11.0. A screening questionnaire and other tools may be used.

7.4 HIV Risk Reduction Counselling

HIV risk reduction counselling will be provided to all participants as outlined by site-specific SOPs.

Study staff will provide HIV risk reduction counselling based on reported individual risk and provide free condoms, as appropriate, at every visit. Group 1 will receive HIV risk reduction counselling and for Groups 2 and 3, HIV risk reduction counselling will be conducted as secondary prevention to reduce onward transmission.

7.5 Family Planning Counselling

Study staff will counsel participants about the importance of preventing pregnancies and of using condoms, as well as other effective family planning methods, as appropriate. Participants may be referred for family planning services as necessary according to site-specific SOPs as detailed in the SOM. Pregnancy prevention methods chosen and compliance will be documented.

7.6 ART Counselling (Group 3)

HIV-infected participants who are not on ART will receive ART counselling upon entering the study and 8 weeks after administration of IP. Participants who have not initiated or made plans to initiate ART by the final study visit will receive ART counselling again at their final study visit.

7.7 Specimens

Approximately 150 ml of blood will be collected from participants in Groups 1 and 2, and approximately 205 ml of blood will be collected from participants in Group 3 at the screening visit. At later visits, approximately 8.5 ml to 214 ml of blood will be collected, depending on study procedures and group assignment (see Appendix A), usually from the antecubital fossa.

Optional collection of rectal and/or cervical mucosal secretions will be obtained using a rectal sponge or cervical Softcup for those participants that consent.

All specimens will be handled according to the procedures specified in the AP.

In the event of an abnormal laboratory value, participants may be asked to have an additional sample collected at the discretion of the Principal Investigator or designee.

7.8 Reimbursement

Participants will be reimbursed for their time, effort and for costs to cover their travel expenses to the study site and any inconvenience caused due to study participation. Site specific-reimbursement amounts will be documented in the site-specific Participant Information Sheet, and approved by the Institutional Review Board.

7.9 Randomization and Blinding

Participants will be identified by a unique study identification number.

Participants will be randomized according to the randomization schedule prepared by the statisticians at the Data Coordinating Center (DCC) prior to the start of the study. Participants will be automatically assigned a specific allocation number as they are enrolled into the data entry system. An unblinding list (Pharmacy List) will be provided to the unblinded site pharmacist by the DCC.

This is a randomized, double-blind placebo-controlled study for groups 1 and 2, and an open label study for group 3. For Groups 1 and 2, study staff (investigator and clinical personnel monitoring the safety and laboratory assay results) and participants will be blinded with respect to the allocation of Investigational Product (PGT121 mAb or placebo). A site pharmacist will be unblinded for the purposes of preparing study product.

A participant will be considered enrolled once he/she has been assigned an allocation number.

Blinded participants will be informed about their assignment (product/placebo) at study completion, once the database is locked. Should a study participant be unblinded during the study, the study participant will be followed up until the end of the study according to the Schedule of Procedures (Appendix A).

7.10 Un-blinding Procedure for Individual Participants

Un-blinding of an individual participant may be indicated in the event of a medical emergency if the clinical management of the participant would be altered by knowledge of the treatment assignment.

The un-blinded information should be restricted to a small group of individuals involved in clinical management/medical treatment of the participant (e.g. treating physician) and the blind must be maintained for those responsible for the study assessments.

The reasons for un-blinding should be documented and the IAVI Chief Medical Officer, the Medical Monitor and the DCC should be notified as soon as possible. The procedures and contact numbers for un-blinding are outlined in the SOM.

7.11 Assessment of IP related HIV sero-positivity

It is possible that PGT121 mAb or an immune response to PGT121 mAb could cause a positive result on a diagnostic HIV antibody test. A Group 1 participant who tests HIV antibody positive at the end of the study will have additional testing to distinguish actual HIV infection from IP-related responses. The participant will be informed of his/her positive HIV antibody test result and offered continuing follow-up until the HIV antibody test becomes negative.

8.0 INVESTIGATIONAL PRODUCT

8.1 Description

A summary of the Investigational Products is shown in Table 8.1-1.

Table 8.1-1 Investigational Products

**Product / Placebo**	**Dosage level**	**Total volume in IP container**	**Total IP or placebo volume to be injected into a 100 mL saline IV bag** (for an 88 kg body weight**)	**Total volume to be Infused** (for an 88 kg body weight**)
IP: PGT121 (50 mg/mL)	3 mg/kg	6 mL per vial	5.3 mL	105.3 mL
	10 mg/kg		17.6 mL	117.6 mL
	30 mg/kg		52.8 mL	152.8 mL
Placebo: 0.9% Sodium Chloride Injection USP (Saline)*	3 mg/kg matching***	NA	5.3 mL***	105.3 mL***
	10 mg/kg matching***		17.6 mL***	117.6 mL***
	30 mg/kg matching***		52.8 mL***	152.8 mL***

* The Placebo provided will be a commercially-available saline partial addition IV bag.

** The actual volume to be injected will be based on the dose group and the weight of the participant at the time of IP administration. The example included here is the average weight of an adult male in the US (88kg) (http://www.cdc.gov/nchs/data/series/sr_11/sr11_252.pdf)

*** For placebo IV infusions: saline from an additional IV bag will be injected into the saline IV bag intended for administration, to match the volume used for a PGT121 mAb injection in the same dose group, to prevent unblinding.

8.2 Shipment and Storage

Authorization to ship the Investigational Product to the site will be provided in writing by the Sponsor, upon confirmation that all required critical documents for shipment authorization are completed. The Investigational Product will be shipped maintaining the required storage conditions and stored in a secure location in the clinical site's pharmacy.

The Investigational Product is formulated in a 20 mM Acetate, 9% Sucrose, 0.008% polysorbate 80, pH 5.2 formulation buffer at a concentration of 50 mg/mL. Each 10 ml vial will contain 6 ml of IP stored at <- 20°C. Each vial will be labelled with the name of the product, Lot number, concentration, storage temperature, date of manufacturing, contact information of the Sponsor and a US cautionary statement. Several such vials will be packaged in a box. Each box will also be labelled with similar information as the vial label.

8.3 Preparation of Investigational Product (IP)

Detailed instruction will be provided to the site pharmacist in the SOM for preparing each of the investigational products. The site pharmacist will not be blinded, but the study physician/designee administering the IP will be blinded. Product should be administered within 6 hours of preparation. Example calculations for final volume for IV infusion are illustrated in Table 8.1-1. Instructions for storing used vials for reconciliation until the end of the study and subsequent disposal will be provided in the SOM. Syringes or other components in direct contact with investigational products will be disposed of in a biohazard container and incinerated or autoclaved.

8.4 Administration of Investigational Product

Investigational Product will be administered at the enrollment visit.

The IP will be injected into a 0.9% Saline bag. The participant will receive the IP via IV infusion. Participants will receive infusion over approximately 60 minutes, allowing for clinician discretion. Further information on the IV infusion of the IP is supplied in the SOM and study documents.

8.5 Accountability and Disposal of Investigational Product

All used vials will be retained at the pharmacy at the end of each IP administration visit. The date, allocation number and location of storage of the returned vials will be recorded.

During the study, the IP accountability forms including receipt and dispensing of vials will be kept and monitored.

At the end of the study, the used and unused IP vials will be handled according to instructions of Sponsor.

Further information on accountability and disposal of IP is supplied in the SOM.

9.0 ASSESSMENTS

9.1 Safety Assessments

Data on local and systemic reactogenicity (i.e., solicited AEs) will be collected by structured interview and medical examination. Data on other adverse events will be collected with open-ended questions. All data will be recorded on the appropriate source documents and entered into the study database. Participants will be given a Memory Aid, which is a tool to assist with collecting reactogenicity data.

Local and systemic reactogenicity events will be assessed by study staff prior to IV infusion of IP, at approximately 30 minutes after IP administration start, at 1 hour after IP administration start, and subsequently every hour for the first 12 hours post-IP administration. Study staff will review the Memory Aid with the participant, and determine the severity of the reactions on days 1-3 through discussion with the participant.

9.1.1 Local reactogenicity

The presence of local reactogenicity will be assessed at the time points specified in the Schedule of Procedures (Appendix A).
Pain, tenderness, erythema/skin discoloration, swelling/hardening or pruritus will be assessed and graded using Appendix C, Adverse Event Severity Assessment Table, as a guideline.

9.1.2 Systemic reactogenicity

The presence of systemic reactogenicity will be assessed at the time points specified in the Schedule of Procedures (Appendix A).
Fever, chills, headache, nausea, vomiting, malaise, myalgia and arthralgia will be assessed and graded using the Appendix C, Adverse Event Severity Assessment Table as a guideline.

9.1.3 Vital Signs

At the administration of IP visit, vital signs (pulse, respiratory rate, blood pressure and temperature) will be measured by study staff prior to IP administration, at approximately 30 minutes post IP administration and hourly until 12 hours after IV infusion start. For the other study visits vital signs will be assessed at the time points specified in the Schedule of Procedures (Appendix A).

9.1.4 Other Adverse Events

Other adverse events (AEs) will be collected through 56 days after IP administration in all participants. Serious Adverse Events (SAEs) will be collected throughout the entire study period. Potential Immune Mediated Diseases (pIMDs), as defined in Section 10.5, will be collected throughout the study period, using the SAE reporting process. Open-ended questions will be asked at time points according to the Schedule of Procedures (Appendix A). All adverse events will be graded using Appendix C, Adverse Event Severity Assessment Table, as a guideline and will be assessed for causality to the IP. For more information regarding adverse events refer to Section 10.0, Adverse Events.

9.1.5 Concomitant Medications

Concomitant receipt of Investigational Products is prohibited during the study.

Contraceptive use and use of medication at study entry will be documented. (See DCF instructions)

During the study, information regarding concomitant medications and reasons for their use will be solicited from the study participants for 56 days. Ongoing concomitant medications will be recorded until end of study.

9.1.6 Routine laboratory parameters

Table 9.1.6-1 shows the laboratory parameters that will be measured routinely. The samples for these tests will be collected at the time points indicated in the Schedule of Procedures (Appendix A).

Table 9.1.6-1: Laboratory Parameters

Laboratory Parameter	Test
Hematology and Coagulation	Hemoglobin, hematocrit, leukocytes, platelets, absolute neutrophil count (ANC), absolute lymphocyte count (ALC), activate partial thromboplastin time (aPTT), international normalized ratio (INR)
Clinical Chemistry	Sodium, potassium, creatinine, aspartate aminotransferase (AST), alanine aminotransferase (ALT), total bilirubin, alkaline phosphatase<br>Groups 1 and 2 only: albumin, creatine kinase, C-reactive protein, C3 complement, C4 complement
Urinalysis	Dipstick test for protein, blood glucose, ketones, esterase (leukocytes) and nitrite. If clinically significant abnormalities (e.g., blood, protein, leukocytes) are found on dipstick test, then further test(s) will be performed (e.g., microscopy, culture)
T cell panel (Groups 2 and 3)	CD4 T cell count and frequency by single platform flow cytometry

9.1.7 Specific screening tests:

Participants will be screened to exclude the following diseases:

- Hepatitis B: positive for hepatitis B surface antigen (HBsAg)
- Hepatitis C: positive for hepatitis C RNA (HCV antibody test, followed by HCV RNA test if HCV antibody positive)
- Active syphilis: confirmed diagnosis (e.g.; positive RPR confirmed by TPHA)

A negative Hepatitis B and Hepatitis C result can be documented from the medical record only if the result is from a test administered less than 6 months ago.

Participants will also be screened to exclude the following laboratory parameters:

- Autologous PGT121-like antibody ELISA level above the cut-off;
- Resistance of autologous HIV to PGT121 neutralization *in vitro* (HIV viremic participants only, Group 3)

9.1.8 Monitoring for anti-PGT121 antibodies:

Participants will be evaluated for the development of antibodies to PGT121 mAb (anti-drug antibodies, ADA) by ELISA according to the Schedule of Procedures (Appendix A).

9.2 Virologic Assessments

Table 9.2-1 shows the virologic parameters that will be measured routinely. The samples for these tests will be collected at the time points indicated in the Schedule of Procedures (Appendix A).

Table 9.2-1: Virologic Assessment Table

Virologic	Test

Parameter	
Antiviral Activity	Plasma HIV RNA levels
Anti-reservoir activity	Cell-associated HIV-1 RNA levels in resting CD4 T cells; total HIV-1 DNA and 2-long terminal repeat (LTR) HIV-1 DNA circles in resting or total CD4 T cells; quantitative viral outgrowth assay (qVOA)
Other	Genotyping of plasma HIV RNA for evaluation of PGT121-induced escape mutations; phenotyping of plasma HIV RNA for neutralization susceptibility to PGT121 in-vitro

9.3 Exploratory Immunogenicity Assessments

Humoral immune response assays will include, but are not limited to Env-specific Ab-binding assays, virus neutralization assay, and assays for Ab functionality. Cellular immune response assays will include, but are not limited to IFNγ ELISPOT assay, ICS, and multiparameter flow cytometry. Exploratory assessments on mucosal samples will include, but are not limited to characterization of Env-specific binding Abs. Priority assays are listed below.

9.3.1 Antibody Responses

- Env-specific binding Abs (titers and breadth).
- Env-specific nAbs (titers and breadth).
- Env-specific functional Abs (phagocytosis score and breadth).
- Env-specific binding Ab isotypes (IgA, IgG1-4) (titers and breadth).

9.3.2 Cellular Responses

- IFNγ peripheral blood mononuclear cell (PBMC) responders to peptide pools and subpools of Potential T-cell epitopes, PTE Env/Gag/Pol peptides.
- $CD4^+$ and $CD8^+$ T-cell functionality (% cells producing e.g. IFNγ, IL-2, IL-4, TNFα).
- T-cell development with emphasis on follicular helper T-cells and memory differentiation.

9.3.3 PBMC, Serum and Plasma Storage

Samples of cryopreserved PBMC, plasma and serum will be stored as indicated in the Analytical Plan (AP) and, if the participant consents, may be used for the purposes of standardization, quality control and for future assays related to HIV prevention or treatment research and development. These samples will be archived and the testing laboratories will be blinded to the participant’s identity.

9.4 Other Assessments

9.4.1 HIV Antibody Testing

All HIV-uninfected participants (Group 1) will be tested for HIV antibodies as indicated in the Schedule of Procedures (Appendix A) or as needed, if medical or

social circumstances arise. All participants will receive HIV risk reduction counselling and pre- and post-HIV-test counselling, as specified in Section 7.3 Counselling.

9.4.2 Pharmacokinetics

Blood draws for pharmacokinetics will be done on the day of IP administration immediately before starting IV infusion of IP, at the end of the IP administration, and 30 minutes and 3 hours after the end of the IP administration. Additional draws will be done at 6, 9, 12 and 24 hours after the start of the IP administration. Thereafter, pharmacokinetic draws will be done as indicated in the Schedule of Procedures (Appendix A). PGT121 mAb serum or plasma levels will be determined using two methods: a sandwich ELISA using a murine anti-idiotype antibody to PGT121 mAb, and a neutralization assay.

PGT121 mAb pharmacokinetic analysis will be performed using standard non-compartmental analysis methods to estimate elimination half-life (t1/2), clearance (CL/F), volume of distribution (Vz/F), Area under the concentration decay curve (AUC), impact of viral load and/or ART on PGT121 mAb disposition (elimination half-life (t1/2), clearance (CL/F), volume of distribution (Vz/F) and total exposure. PGT 121 accumulation will also be examined in rectal and cervical mucosal secretions collected with rectal sponges or cervical Softcups in study participants who specifically consented for these procedures. Descriptive results will be reported for the pharmacokinetic parameters by dose subgroup.

Exploratory analysis using population analysis methods simultaneously combining all pharmacokinetic data across all doses and treatment groups will be performed for quantitative characterization of differences in PGT121 mAb disposition by dose, participant group or disease state.

9.4.3 HLA Typing

Samples for HLA typing will be collected as specified in the AP and may be analyzed as warranted.

9.4.5 Pregnancy Test

A urine pregnancy test for all female participants will be performed by measurement of human chorionic gonadotrophin (βhCG) at time points indicated in the Schedule of Procedures (Appendix A). The results of the pregnancy test must be negative prior to IV infusion of PGT121 mAb. See section 10.7 for description of pregnancy after administration of IP.

9.4.6 HIV Risk Assessment (Group 1)

Study staff will assess participants for their past and current risk of acquiring HIV at time points indicated in Schedule of Procedures (Appendix A).

9.4.7 Social Impact Assessment

A brief assessment of the impact of participation in the study will be administered to participants at their final study visit.

10.0 ADVERSE EVENTS

10.1 Definition

An adverse event (AE) is any untoward medical occurrence in a participant administered an Investigational Product and which does not necessarily have a causal relationship with the Investigational Product. An AE can therefore be any unfavourable or unintended sign (including an abnormal laboratory finding), symptom, or disease, temporally associated with the use of Investigational Product whether or not related to the Investigational Product.

Assessment of severity of all AEs, including and seriousness of AEs, is ultimately the responsibility of the Principal Investigator of each site. Refer to the DIVISION OF AIDS (DAIDS) TABLE FOR GRADING THE SEVERITY OF ADULT AND PEDIATRIC ADVERSE EVENTS Version 2.0, November 2014 for additional guidance.

10.2 Assessment of Severity of Adverse Events

The following general criteria should be used in assessing adverse events as mild, moderate, severe or very severe at the time of evaluation:

Grade 1 (Mild): Symptoms causing no or minimal interference with usual social & functional activities

Grade 2 (Moderate): Symptoms causing greater than minimal interference with usual social & functional activities

Grade 3 (Severe): Symptoms causing inability to perform usual social & functional activities

Grade 4 (Very Severe): Symptoms causing inability to perform basic self-care functions OR Medical or operative intervention indicated to prevent permanent impairment, persistent disability, or death

Guidelines for assessing the severity of specific adverse events and laboratory abnormalities are listed in Appendix C, Adverse Event Severity Assessment Table.

10.3 Relationship to Investigational Product

Assessment of relationship of an AE or SAE to Investigational Product (IP) is the responsibility of the Principal Investigator or designee. All medically indicated and available diagnostic methods (e.g., laboratory, blood smear, culture, X-ray, etc.) should be used to assess the nature and cause of the AE/SAE. Best clinical and scientific judgment should be used to assess relationship of AE/SAEs to the IP and/or other cause.

The following should be considered:

- Presence/absence of a clear temporal (time) sequence between administration of the IP and the onset of AE/SAE
- Presence/absence of another cause that could more likely explain the AE/SAE (concurrent disease, concomitant medication, environmental or toxic factors)

- Whether or not the AE/SAE follows a known response pattern associated with the IP

The relationship assessment should be reported as one of the following:

Not Related: clearly explained by another cause (concurrent disease, concomitant medication, environmental or toxic factors, etc.).

Unlikely: more likely explained by another cause (concurrent disease, concomitant medication, environmental or toxic factors, etc.).

Possibly: equally likely explained by another cause but the possibility of the IP relationship cannot be ruled out (e.g., reasonably well temporally related and/or follows a known IP response pattern but equally well explained by another cause).

Probably: more likely explained by the IP (e.g., reasonably well temporally related and/or follows a known IP response pattern and less likely explained by another cause).

Definitely: clearly related and most likely explained by the IP.

For the purpose of expedited safety reporting, all possibly, probably or definitely related SAEs are considered IP-related SAEs.

10.4 Serious Adverse Events

An adverse event is reported as a "Serious Adverse Event" if it meets any of the following criteria (as per International Conference on Harmonisation [ICH] Good Clinical Practice [GCP] Guidelines):

- Results in death
- Is life threatening
- Results in persistent or significant disability/incapacity
- Requires in-participant hospitalization or prolongs existing hospitalization
- Is a congenital anomaly/birth defect or spontaneous abortion
- Any other important medical condition that requires medical or surgical intervention to prevent permanent impairment of a body function or structure

Elective surgery for pre-existing condition that did not increase in severity or frequency is not considered an SAE.

Serious Adverse Events (SAEs) should be reported within 24 hours of the site becoming aware of the event, and sent to the Sponsor as described in the SOM.

To discuss IP-related SAEs or any urgent medical questions related to the SAE, the site investigator should contact one of the IAVI Medical Monitors directly (see Contact List in the SOM).

The IAVI SAE Report Form should be completed with all the available information at the time of reporting and sent to the Sponsor as described in the SOM. The minimum data required in reporting an SAE are the study identification number, date of birth, gender, event description (in as much detail as is known at the time), onset date of event (if available), reason event is classified as serious, reporting source (name of Principal Investigator or designee), and relationship to the IP as assessed by the investigator.

The Principal Investigator or designee is required to prepare a detailed written report with follow up until resolution or until it is judged by the Principal Investigator or designee to have stabilized.

The Principal Investigator or designee must notify the local IRB/IEC of all SAEs as appropriate. In case of IP-related SAEs, the Sponsor will notify responsible regulatory authorities, Safety Monitoring Committee (SMC), and other study sites where the same IP is being tested.

More details on SAE definitions and reporting requirements are provided in the SOM.

Serious Event Prior to Investigational Product Administration

If a serious event occurs in the period between the participant signing the Informed Consent Form and receiving the IV infusion of IP, the event will be reported using the SAE form and following the same procedures for SAE reporting, as indicated in Section 10.4. The timing of the event will be indicated by using the relevant checkbox on the SAE form.

10.5 Reporting Potential Immune-Mediated Diseases

Potential immune-mediated diseases (pIMDs) are a subset of AEs that include both clearly autoimmune diseases and also other inflammatory and/or neurologic disorders that may or may not have an autoimmune etiology. These events are of special interest since they could potentially be caused by immune responses to the IP. The investigator/designee should report such adverse events within the same time limits (following confirmation of an AE as a pIMD; see last paragraph of this section below), and using the same CRF pages, as utilized for SAEs. The investigator or his/her designee will evaluate the occurrence of pIMDs at every visit/contact during the study. IAVI will also expect investigators/designee to provide additional information about pIMD events. AEs to be reported and documented as pIMDs include:

Neuroinflammatory disorders: optic neuritis, cranial nerve disorders (including Bell’s palsy), multiple sclerosis, demyelinating disease, transverse myelitis, Guillain-Barré syndrome, myasthenia gravis, encephalitis, neuritis.

Musculoskeletal disorders: systemic lupus erythematosus, cutaneous lupus, Sjögren’s syndrome, scleroderma, dermatomyositis, polymyositis, myopathy, rheumatoid arthritis and juvenile rheumatoid arthritis, polymyalgia rheumatica or temporal arteritis, reactive arthritis, psoriatic arthropathy, ankylosing spondylitis, undifferentiated spondyloarthropathy.

Gastrointestinal disorders: Crohn’s disease, ulcerative colitis or proctitis, celiac disease.

Metabolic diseases: autoimmune thyroiditis, Grave's or Basedow’s disease, Hashimoto thyroiditis, insulin-dependent diabetes mellitus (IDDM), Addison’s disease.

Skin disorders: psoriasis, vitiligo, Raynaud’s phenomenon, erythema nodosum, autoimmune bullous skin diseases.

Others: autoimmune hemolytic anemia, thrombocytopenia, antiphospholipid syndrome, *vasculitis, pernicious anemia, autoimmune hepatitis, primary biliary cirrhosis, primary sclerosing cholangitis, autoimmune glomerulonephritis, autoimmune uveitis, autoimmune myocarditis/cardiomyopathy, sarcoidosis, Stevens-Johnson syndrome, Behçet's syndrome.

Infusion site reactions: Grade 3 or 4 infusion site reactions lasting more than 2 days.

*Vasculitis: Vasculitis, Diffuse vasculitis, leucocytoclastic vasculitis, polyarteritis nodosa, microscopic polyangiitis, Wegener's granulomatosis, anti-neutrophil cytoplasmic antibody positive vasculitis, Henoch-schönlein purpura, allergic granulomatous angiitis (Churg-Strauss disease), Kawasaki disease, Takayasu's arteritis, temporal arteritis (giant cell arteritis), renal vasculitis.

Medical judgement should be exercised in deciding whether other disorders/diseases have an autoimmune origin and should also be reported as described above, and this judgement is the investigator's prerogative. Whenever sufficient data exist to substantiate any of the diagnoses in the above list, the event must be reported as a pIMD. While the intent of pIMD reporting is to be inclusive, isolated nonspecific symptoms, which might (or might not) represent the above diagnoses, should be captured as AEs but not reported as pIMDs until the diagnosis can be defended.

10.6 Clinical Management of Adverse Events

Adverse events (AEs) will be managed by the clinical study team who will assess, provide first line of care as appropriate and refer to health care and treatment facilities as warranted. If any treatment/medical care is required as a result of the harm caused by the IP or study procedures, this will be provided free of charge.

If a participant has an AE and/or abnormal laboratory value that is known at the time of IV infusion of IP, the specifications of Section 12.0 will be followed.

Participants will be followed until the AE resolves or stabilizes or up to the end of the study, whichever comes last. If at the end of the study, an AE (including clinically significant laboratory abnormality) that is considered possibly, probably or definitely related to the IP is unresolved, follow-up will continue until resolution if possible and/or the participant will be referred.

10.7 Pregnancy

Although not considered an AE, if a female participant becomes pregnant during the study, it is the responsibility of the Principal Investigator or designee to report the pregnancy promptly to IAVI using the designated forms. The participant will be followed for safety until the end of pregnancy or study completion, whichever occurs last. If possible, approximately 2–4 weeks after delivery, the baby will be examined by a physician to assess its health status and the results will be reported to the Sponsor. The baby will be examined again by a Physician around age 1, and the results will be reported to the Sponsor.

Complications of pregnancy that meet criteria for SAEs, specified in Section 10.4 of this Protocol (e.g., hospitalization for eclampsia, spontaneous abortion, etc.) should be reported as SAEs.

10.8 Intercurrent HIV Infection (Group 1)

HIV infection cannot be directly caused by the IP. If a participant acquires HIV through exposure in the community, at any time after the IV infusion of IP, the participant should be offered referral to appropriate care and treatment facilities. The participant will continue to be followed in the study for safety assessments.

Intercurrent HIV infection in study participants, although not considered an SAE, must be reported promptly to IAVI using the designated forms. However, medical conditions associated with the HIV infection that meet criteria for being serious specified in the Section 10.4 of this Protocol (e.g., sepsis, *Pneumocystis jiroveci [carinii] pneumonia*, etc.) should be reported as SAEs using the SAE Report Form.

11.0 MANAGEMENT OF HIV ISSUES DURING AND FOLLOWING STUDY

11.1 HIV Testing – Group 1

Group 1 participants will be tested for HIV antibodies as indicated in the Schedule of Procedures (Appendix A) or as needed, if medical or social circumstances arise. All participants will receive HIV risk reduction counselling and pre- and post-HIV-test counselling, as specified in Section 11.2.1, Counselling (Group 1).

It is possible that PGT121 or an immune response to PGT121 mAb could cause a positive result on a diagnostic HIV antibody test. An IP recipient who falsely tests HIV positive with a diagnostic HIV antibody test at the end of the study will be informed of his/her positive test result and offered continuing follow-up until the test becomes negative.

If a participant acquires HIV through exposure in the community, at any time after the administration of IP, the participant will be offered referral to appropriate care and treatment facilities. The participant will continue to be followed in the study for safety assessments.

Should a participant require HIV testing outside of the study for personal reasons, it is recommended that the participant contact the study staff first. HIV testing can be done at the study site and then processed at an independent laboratory as above. Written evidence of HIV status (HIV-infected or HIV-uninfected) will be provided upon request.

11.2 Social Discrimination as a Result of IP-related antibodies

In order to minimize the possibility of social discrimination in participants (if any) who test positive on a diagnostic HIV antibody test due to IP-related antibodies, appropriate diagnostic HIV testing and certification will be provided both during and after the study as needed.

11.3 HIV infection – Group 1

Group 1 participants who are diagnosed with HIV infection at screening or during the study (intercurrent HIV-infection) will be provided the following:

11.3.1 Counselling

The participant will be counselled by the study investigators or designated counsellors. The counselling process will assist the participant with the following issues:

- Psychological and social implications of HIV infection
- Who to inform and what to say
- Implications for sexual partners
- Implications for child-bearing
- Avoidance of transmission to others in future
- Mandatory reporting to the state, in some instances

11.3.2 Referral for Support/Care

Participants will be referred to a participant support center or institution of his/her choice for a full discussion of the clinical aspects of HIV infection. Referral will be made to a designated physician or center

12.0 WITHDRAWAL FROM STUDY

12.1 Deferral of IV infusion of IP

An IV infusion of IP may be temporarily deferred if the participant is clinically ill at the time of the administration of IP visit and/or presents with fever (≥ 100.4 F) at the time of the administration of IP visit. A participant must be clinically well and afebrile for a minimum of a 24-hour consecutive period prior to administration of IP.

Any planned or unplanned deferral of infusion of IP will be discussed with the Sponsor. Participants will be deferred from infusion of IP for any of the following reasons:

1. Pregnancy
2. A disease or condition or adverse event that may develop, regardless of relationship to Investigational Product, if the Principal Investigator or designee is of the opinion that administration of IP will jeopardize the safety of the participant
3. Participant's request to defer infusion

The following events require resolution and/or review of clinical history by the Principal Investigator or designee and consultation with the Medical Monitor, prior to administration of IP:

- Any abnormal laboratory value, as outlined in section 5.7, Exclusion Criteria, Hematology, Chemistry, Urinalysis that is known at the time of infusion and have not resolved. Abnormal results should be confirmed on the original sample and/or repeated at least once to confirm abnormal values.
- Receipt of inactivated/killed/subunit vaccines (non-HIV) or immunoglobulin within the previous 14 days. Receipt of live attenuated vaccines within the previous 60 days.

- Participating in another clinical study of an Investigational Product

12.2 Withdrawal from the Study (Early Termination)

Participants may be withdrawn from the study permanently for the following reasons:

1. Participants may withdraw from the study at any time if they wish, for any reason
2. The Principal Investigator or designee has reason to believe that the participant is not complying with the protocol
3. If the Sponsor decides to terminate or suspend the study

If a participant withdraws or is withdrawn from the study, all termination visit procedures will be performed according to the Schedule of Procedures (Appendix A) where possible. Every effort will be made to determine and document the reason for withdrawal.

13.0 DATA HANDLING

13.1 Data Collection and Record Keeping at the Study Site

Data Collection: All study data will be collected by the clinical study staff using designated source documents and entered onto the appropriate electronic CRFs (eCRFs). Access to eCRFs will be provided via an electronic data entry system hosted by the Data Coordination Center. All study data must be verifiable to the source documentation. A file will be held for each participant at the clinic(s) containing all the source documents. Source documentation will be available for review to ensure that the collected data are consistent with the CRFs.

All CRFs and laboratory reports will be reviewed by the clinical team, who will ensure that they are accurate and complete.

Source documents and other supporting documents will be kept in a secure location. Standard GCP practices will be followed to ensure accurate, reliable and consistent data collection.

Source documents include but are not limited to:

- Signed Informed Consent Documents
- Progress notes
- Data collection forms
- Documentation of any existing conditions or past conditions relevant to eligibility
- Printed laboratory results
- Print out of the IDES generated enrollment confirmation
- All Adverse Events
- Concomitant medications
- Local and systemic reactogenicity events

13.3 Data Entry at the Study Site

The data collected at the site will be recorded onto the eCRFs by the study staff and entered into a database. To provide for real time assessment of safety, data should be entered as soon as reasonably feasible after a visit occurs.

13.4 Data Analysis

The Sponsor, PIs and Product Developers will agree on how data will be analyzed and presented prior to unblinding of the study.

The DCC will conduct the data analysis and will provide interim safety and final study reports for the Sponsor, Principal Investigators, the PSRT and SMC and the regulatory authorities, as appropriate.

14.0 STATISTICAL CONSIDERATIONS

14.1 Safety and Tolerability Analysis

14.1.1 Sample Size

The sample size for safety and tolerability analysis will be 30-48 participants according to the dose escalation design used to characterize the safety profile of one IV infusion of PGT121 mAb, at one of three dose levels, to HIV-uninfected and HIV-infected individuals (groups 1 and 2).

14.1.2 Null Hypothesis

As this is an exploratory proof of concept trial and analysis will be descriptive, no formal null hypothesis will be tested.

14.1.3 Statistical Power and Analysis and Dose Escalation Rules

The frequency of moderate or greater local and systemic reactogenicity events will be determined and compared between groups.

The frequency of SAEs judged possibly, probably or related to the IP will be determined.

All AEs will be analyzed and, grouped by seriousness, severity and relationship to the Investigational Product (as judged by the investigator).

For life-threatening adverse events related to Investigational Product: if none of the 12 (max 18) participants receiving Investigational Products experience such reactions, then the 95 % upper confidence bound for the rate of these adverse events in the population is 26.5% (or 18.5% if n=18).

All AEs will be analysed and grouped by seriousness, severity and relationship to the IP (as judged by the investigator).

For life-threatening adverse events related to IP: if none of the 12 (max 18) participants in either Group 1 or Group 2 who receive the IP experience such reactions then the 95% upper confidence bound for the rate of these adverse events in the population is 26.5% (or 18.5% if n=18).

An interim analysis of group data will be carried out according to the study schema (Table 5.3.1) without unblinding the study to investigators or participants. At the end of the study, a full analysis will be prepared.

Based on previous experience with IAVI Phase 1 IP studies, it is expected that the amount of missing, unused or spurious data will be insignificant. Unused and spurious data will be listed separately and excluded from the statistical analysis. Missing data will be excluded from the statistical analysis.

14.2 Pharmacokinetic Analysis

14.2.1 Sample Size

The sample size for pharmacokinetic analysis will be 4 per dose subgroup, sufficient to provide sufficient information for the planned analyses.

14.2.2 Null Hypothesis

As this is an exploratory proof of concept trial and analysis will be descriptive, no formal null hypothesis will be tested.

14.2.3 Statistical Power and Analysis

Disposition of PGT121 mAb will be evaluated in this study. Based on the PK profile of other human monoclonal antibodies, it is expected that the half-life of PGT121 mAb will be 14 to 21 days. Previously published data indicates that the pharmacokinetics of PGT121 and 3BNC117 are fairly similar across a non-human primate cohort and within the same non-human primate (clearance of 3BNC117 appears to be marginally faster than that for PGT121).

Commonly reported PK parameters will be calculated using standard non-compartmental slope/height/area/moment (SHAM) analysis methods. Summary descriptive results of PK parameters, including AUC, Cmax, T1/2, and clearance results will be reported by dose cohort. Dose normalized plots of PK parameters will be presented. Correlation between PK and reported safety and pharmacodynamic outcomes will also be explored parameters in order to examine exposure-effect relationships.

A more powerful exploratory analysis to quantitatively determine the dose, participant and disease impact on PGT121 mAb pharmacokinetics, and correlate exposure with response, while correctly accounting for variance based on population intrinsic factors such as weight and gender will be performed. Using the proposed population analysis approach we will be able to simultaneously examine the magnitude and the rate of change to PGT121 disposition driven by HIV-1 RNA levels and/or ART, and also examine the magnitude and the rate of decline in log copies/ml of HIV-1 RNA plasma levels from baseline.

The frequency and levels of anti-PGT121 antibodies will be calculated and tabulated.

14.3 Virologic Analysis for Dose De-escalation in Groups 3A-C

14.3.1 Sample Size

The sample size for virologic analysis in Groups 3A-C will be 24-36 participants according to the dose de-escalation design described below.

14.3.2 Null Hypothesis

The null hypothesis is that there is no difference in antiviral activity between PGT121 mAb and placebo.

14.3.3 Statistical Power and Analysis

The virologic analysis described in this section relates to Groups 3A-C of the study design, in which dose de-escalation is performed in an adaptive study design in HIV-infected participants off ART with plasma HIV RNA levels of 2 x 10^3 – 10^5 copies/ml. This section assumes that Part 1 of the study has successfully demonstrated that there is a safe dose level of the IP such that the study is carried forward into Part 2.

The primary efficacy outcome for this analysis is defined as change in log10 viral load between Day 0 (day of infusion) and Day 7. The minimum clinically significant value for this outcome is defined as a difference of -0.9 log10.

The study plan for Groups 3A-C is designed so that the IP dose level may be de-escalated in a stepwise manner from the highest dose to the lowest dose, until a given dose level cannot be concluded to be efficacious. If any given dose level is proven to be efficacious at an interim analysis, enrolment for that dose level may cease, and the next lowest dose group may be enrolled. In the unlikely event that IP administration leads to increased viral load, this may be detected by this design. No placebo participants are enrolled as part of this design.

This design represents a dose de-escalation beginning at 30 mg/kg. The actual starting dose will be the MTD as determined by the SMC based on data from Part 1, therefore the starting dose may be 30mg/kg, 10 mg/kg or 3 mg/kg. If the starting dose is 30 mg/kg, then de-escalation will begin with Group 3A. If the starting dose is 10 mg/kg, then de-escalation will begin with Group 3B. If the starting dose is 3 mg/kg, then only Group 3C will be enrolled.

Assuming the starting dose is 30 mg/kg, an interim analysis of Group 3A will be performed after all 6 participants have reached 7 days following IP administration. The following scenarios may then occur, depending on the data:

- If the mean response in the first 6 participants is a decrease greater than or equal to -0.9 log10 HIV RNA, the IP will be determined to be effective at 30 mg/kg, enrollment into Group 3A will cease, and enrollment into Group 3B will begin.
- If the mean response in the first 6 participants is a decrease smaller than -0.9 log10 HIV RNA, then an additional 3 participants will be enrolled into Group 3A. After the additional 3 participants have reached 7 days following IP administration, an analysis of Group 3A (N=9) will be performed:

    - If the mean response in all Group 3A participants is a decrease greater than or equal to -0.9 log10 HIV RNA, the IP will be determined to be effective at 30 mg/kg, and enrollment into Group 3B will begin.
    - If the mean response in all 9 participants is a decrease smaller than -0.9 log10 HIV RNA, then the IP will be determined to be ineffective at 30 mg/kg and Groups 3B and 3C will not be enrolled. In this scenario, no dose of IP will be determined to be effective.

If 30 mg/kg is determined to be an effective dose, then Group 3B will be enrolled at 10 mg/kg. An interim analysis of Group 3B will be performed after 8 participants have reached 7 days following IP administration. The following scenarios may then occur, depending on the data:

- If the mean response in the first 8 participants is a decrease greater than or equal to -0.9 log10 HIV RNA, the IP will be determined to be effective at 10 mg/kg, enrollment into Group 3B will cease, and enrollment into Group 3C will begin.
- If the mean response in the first 8 participants is a decrease smaller than -0.9 log10 HIV RNA, then an additional 4 participants will be enrolled into Group 3B. After the additional 4 participants have reached 7 days following IP administration, an analysis of Group 3B (N=12) will be performed:
    - If the mean response in all Group 3B participants is a decrease greater than or equal to -0.9 log10 HIV RNA, the IP will be determined to be effective at 10 mg/kg, and enrollment into Group 3C will begin.
    - If the mean response in all 12 participants is a decrease smaller than -0.9 log10 HIV RNA, then the IP will be determined to be ineffective at 10 mg/kg, and Group 3C will not be enrolled. In this scenario, the minimum effective dose will be determined to be 30 mg/kg.

If 10 mg/kg is determined to be an effective dose, then Group 3C will be enrolled at 3 mg/kg. An interim analysis of Group 3C will be performed after 10 participants have reached 7 days following IP administration. The following scenarios may then occur, depending on the data:

- If the mean response in the first 10 participants is a decrease greater than or equal to -0.9 log10 HIV RNA, the IP will be determined to be effective at 3 mg/kg and enrollment into Group 3C will cease. In this scenario, the minimum effective dose of the IP will be determined to be 3 mg/kg.
- If the mean response in the first 10 participants is a decrease smaller than -0.9 log10 HIV RNA, then an additional 5 participants will be enrolled into Group 3C. After the additional 5 participants have reached 7 days following IP administration, an analysis of Group 3C (N=15) will be performed:
    - If the mean response in all Group 3C participants is a decrease greater than or equal to -0.9 log10 HIV RNA, the minimum effective dose will be determined to be 3 mg/kg.
    - If the mean response in all 15 participants is a decrease smaller than -0.9 log10 HIV RNA, then the IP will be determined to be ineffective at 3 mg/kg. In this scenario, the minimum effective dose will be determined to be 10 mg/kg.

For the analysis of sample size and power, log10 viral load differences from baseline for each participant were simulated from a normal distribution, with a standard deviation of

0.5. This value was chosen by examining a study of the antiretroviral drug raltegravir, which demonstrated a mean estimated standard deviation of the change of baseline of 0.47[18]. This is a conservative estimate, as the variability of viral loads near the lower range might be expected to also be lower.

The statistical test performed will be the Signed-ranktest, which will incorporate the "shift" parameter of -0.9 log10 (the minimum clinically significant difference selected for this study). An evaluation of potential harm (increased viral load) will also be performed with the Signed ranktest; this test will examine the null hypothesis of no change in viral load (a shift of 0.0 log10 following IP administration) against the one-sided alternative hypothesis that the viral load is increased following IP administration. Each efficacy test will be performed at the level $\alpha = 0.05$. Each test for harm will be performed at level $2\alpha = 0.10$, in order to provide additional sensitivity to detect potential harm.

14.4 Analysis of Antiviral Activity and Dose De-escalation in Subgroups 3D-F

14.4.1 Sample Size

The sample size for antiviral activity will be 3-9 participants, depending on the MTD.

14.4.2 Null Hypothesis

As this is an exploratory proof of concept trial and analysis will be descriptive in this population, no formal null hypothesis will be tested.

14.4.3 Statistical Power and Analysis

No efficacy endpoints will be tested in Groups 3D-F as participants are HIV-infected with low viral loads at baseline (10^2 – $2x10^3$ copies/ml). Immunologic and virologic endpoints will be determined as described in Section 4.1. As soon as subgroup 3D has enrolled 3 participants, enrollment into subgroup 3E will be initiated. The same rule will apply for progression from subgroup 3E to 3F. In the event that dose de-escalation in subgroups 3A-C is stopped due to failure to meet virologic endpoints, dose de-escalation in subgroups 3D-F will continue.

14.5 Secondary and Exploratory Immunologic and Virologic Analyses

14.5.1 Sample Size

The sample size for secondary and exploratory immunologic and virologic analysis will be 63-93 participants.

14.5.2 Null Hypothesis

No formal hypothesis on immunologic or virologic responses will be tested, with the exception of the change in viral load described in Section 14.3.

14.5.3 Statistical Power and Analysis

Descriptive statistics (actual values and changes from reference) will be calculated for continuous immunologic and virologic parameters at all time points. Graphical representations of changes in parameters will be made as applicable. Differences between groups at a specific time point will be tested for exploratory purposes by a 2-sample t-test if the data appear to be normally distributed. If not, the non-parametric Wilcoxon rank sum test will be used. If portions of the measurements are censored below the assay quantification limit, the Gehan-Wilcoxon test will be employed. All statistical tests will be two-sided and will be considered statistically significant if $p < 0.05$.

Frequency tabulations will be calculated for discrete (qualitative) immunologic and virologic parameters at all time points. Significant differences between groups will be determined by a 2-sided Fisher's exact test.

Interim immunologic and virologic analyses of grouped data may be performed without unblinding the study to investigators or participants.

15.0 QUALITY CONTROL AND QUALITY ASSURANCE

To ensure the quality and reliability of the data collected and generated and the ethical conduct of this study, a Study Operations Manual (SOM) will be developed. All deviations will be reported and investigated. The SOM describes reporting and deviation documentation requirements and procedures.

Regular monitoring will be performed according to ICH-GCP as indicated in Section 17.5.

An independent audit of the study and study sites may be performed by the Sponsor or designee to establish the status of applicable quality systems. Inspection by regulatory authorities may also occur.

By signing the protocol, the Principal Investigators agree to facilitate study related monitoring, audits, IRB/IEC review and regulatory inspection(s) and direct access to source documents. Such information will be treated as strictly confidential and under no circumstances be made publicly available.

16.0 DATA AND BIOLOGICAL MATERIAL

All data and biological material collected through the study shall be managed in accordance with the Clinical Trial Agreement (CTA). Distribution and use of these data will be conducted by agreement of all parties.

The computerized raw data generated will be held by the DCC on behalf of the Sponsor. The study sites will also hold the final data files and tables generated for the purpose of analysis.

17.0 ADMINISTRATIVE STRUCTURE

The Principal Investigator will be responsible for all aspects of the study at the study site.

17.1 Protocol Safety Review Team

A PSRT will be formed to monitor the clinical safety data. During the administration of IP

phase of the trial, the PSRT will review the clinical safety data on a weekly basis via electronic distribution of reports. An ad hoc PSRT review meeting will occur if any of the members of the PSRT requests a special review to discuss a specific safety issue or as specified in the Study Operations Manual. After the administration of IP phase the PSRT will review the clinical safety data at least monthly.

The PSRT will consist of the IAVI Medical Monitor(s), and the PI or designee from each clinical team. The study chair or an IAVI Medical Monitor may be the PSRT chair. *Ex officio* members will include the IAVI Chief Medical Officer and an unblinded IAVI Medical Monitor.

Additional PSRT participants may include the following, as needed:

- Co-investigators and trial site senior clinical research nursing staff
- Laboratory directors
- Data management, study statistician and regulatory staff

The PSRT membership and procedures are detailed in the PSRT charter.

17.2 Safety Monitoring Committee (SMC)

The SMC will consist of independent clinicians/scientists/statisticians/ethicists who are not involved in the study. Investigators responsible for the clinical care of participants or representative of the Sponsor may not be a member of the SMC. Details of membership, chair and co-chair and responsibilities are outlined in the SMC charter.

Principal Investigator(s) or designee and/or a Sponsor representative may be asked to join an open session of the SMC meeting to provide information on study conduct, present data or to respond to questions.

Safety data will be reviewed by the SMC at pre-specified time points and at an ad-hoc basis.

17.2.1 Content of Interim Safety Review

The SMC will be asked to review the following blinded data:

- Summary of reactogenicity (i.e., solicited adverse events)
- All adverse events judged by the Principal Investigator or designee to be possibly, probably or definitely related to IP
- All laboratory results confirmed on retest and judged by the Principal Investigator or designee to be clinically significant
- All SAEs

An unblinded presentation of all above noted events may also be made available for the SMC for their review if required by any member of the SMC.

17.2.2 SMC Review of Group 1 and 2 data prior to starting Group 3

Following IV infusion of IP of the last participant in Groups 1 and 2, the Safety Monitoring Committee (SMC) will review safety data through the day 14 post-IV infusion visit for all participants to confirm MTD in each group, and determine whether, and at what dose level, Group 3 can initiate enrollment.

17.3 Criteria for Pausing the Study

Enrollment and administration of IP will be stopped and a safety review conducted by the SMC for any of the following criteria:

1. One or more participants experience an SAE that is judged possibly, probably or definitely related to IP.
2. There is a participant death assessed as possibly, probably or definitely related to the IP.
3. Two or more participants experience Grade 3 adverse events in the same category System Organ Class that are considered to be at least possibly related to IP or
4. Any grade 4 adverse event that is considered to be at least possibly related to IP.

Table 2: AE notification and safety pause/AE review rules

Event and relationship to study product	Severity	Occurrence	Site PI action	PSRT or SMC action
SAE, related[1]	Any	Any	Phone, email or fax forms to sponsor within 24 hours	Study pause within 24 hours, refer to SMC for review
SAE, not related[2]	Grade 5	Any	Phone, email or fax forms to sponsor within 24 hours	PSRT review within 2 business days to consider pause
AE[3], related	Grade 3 or 4[4]	Second[5]	Phone, email or fax notification to sponsor within 24 hours	Study pause within 24 hours, refer to SMC for review
AE[3], related	Grade 3 or 4[4]	First	Phone, email or fax notification to sponsor within 24 hours	PSRT review within 2 business days to consider pause

[1] Related SAE refers to SAE deemed to be definitely, probably, or possibly related to study vaccine.

[2] Not related SAE refers to SAE deemed to be probably not related or not related to the study vaccine.

[3] Does not include the following reactogenicity symptoms (fever, fatigue/malaise, myalgia, arthralgia, chills, headache, nausea, vomiting).

[4] If no evidence of disease is present other than an abnormal laboratory value, the test must be repeated (entailing blood re-draw) at least one time. The verification period will be a maximum of 72 hours after initial awareness of the abnormal laboratory value. When signs and symptoms are present, repeat test will not be needed.

[5] PSRT will determine whether the reported related AE (Grade 3 or 4) is a second occurrence of a previously reported AE (Grade 3 or 4).

The Sponsor will request a review by the SMC, (or the SMC chair if other SMC members cannot be convened), to be held within 2 business days of the Sponsor learning of the event. The individual participant(s)/or study may be unblinded at the discretion of the SMC.

Following this review, the SMC will make a recommendation regarding the continuation or suspension of the administration of the IP or the trial and communicate this decision

immediately to the Sponsor. The Sponsor then will inform the Principal Investigators without delay.

Additional *ad hoc* review may be specifically requested by the Sponsor, the Principal Investigator(s) or by the SMC.

17.4 Study Supervision

The SMC, the IAVI Chief Medical Officer (CMO) and the IAVI Medical Monitor(s) have access to progress report(s) of this study. Close cooperation will be necessary to track study progress, respond to queries about proper study implementation and management, address issues in a timely manner, and assure consistent documentation, and share information effectively. Rates of accrual, retention, and other parameters relevant to the site's performance will be regularly and closely monitored by the study team.

17.5 Study Monitoring

On-and/or off-site monitoring will ensure that the study is conducted in compliance with human subjects' protection and other research regulations and guidelines, recorded and reported in accordance with the protocol, is consistent with SOPs, GCP, applicable regulatory requirements and locally accepted practices. The monitor will confirm the quality and accuracy of data at the site by validation of CRFs against the source documents, such as clinical records. The investigators, as well as participants through consenting to the study, agree that the monitor may inspect study facilities and source records (e.g., informed consent forms, clinic and laboratory records, other source documents), as well as observe the performance of study procedures (in accordance with site IRB requirements). Such information will be treated as strictly confidential and will under no circumstances be made publicly available.

The monitoring will adhere to GCP guidelines. The Principal Investigator will permit inspection of the facilities and all study-related documentation by authorized representatives of IAVI, and Government and Regulatory Authorities responsible for this study.

17.6 Investigator's Records

Study records include administrative documentation—e.g., reports and correspondence relating to the study—as well as documentation related to each participant screened and/or enrolled in the study—including informed consent forms, case report forms, and all other source documents. The investigator will maintain and store, in a secure manner, complete, accurate, and current study records for a minimum of 2 years after marketing application approval or the study is discontinued and applicable national and local health authorities are notified. IAVI will notify the Principal Investigator of these events.

18.0 INDEMNITY

The Sponsor and Institution are responsible to have appropriate liability insurance. For research-related injuries and/or medical problems determined to result from receiving the IP,

treatment including necessary emergency treatment and proper follow-up care will be made available to the participant free of charge at the expense of the Sponsor.

19.0 PUBLICATION

A primary manuscript describing safety, anti-viral effect and immune responses in this trial will be prepared promptly after the data analysis is available.

Authors will be representatives of each trial site, the data management and statistical analysis center, the laboratories, the product developer and the sponsor, participant to the generally accepted criteria of contributions to the design and conduct of the study, the analysis of data and writing of the manuscript. Precedence will be given to authors from the site enrolling the greatest number of participants. Manuscripts will be reviewed by representatives of each participating group as specified in the CTA.

20.0 ETHICAL CONSIDERATIONS

The Principal Investigator will ensure that the study is conducted in compliance with the protocol, SOPs in accordance with guidelines formulated by the ICH for GCP in clinical studies, the ethical principles that have their origins in the Declaration of Helsinki and applicable local standards and regulatory requirements.

APPENDIX A: SCHEDULE OF PROCEDURES

Study Month		0								1		2		3	4	5	6
**Study Week**		**0**				**1**		**2**	**3**	**4**	**6**	**8**	**10**	**12**	**16**	**20**	**24**
**Study Day**	**Scr**	**0**	**1**	**2**	**3**	**7**	**10[5]**	**14**	**21**	**28**	**42**	**56**	**70**	**84**	**112**	**140**	**168/ET[9]**
**Visit Windows (Days)**	**-42**	**0**	**0**	**0**	**0**	**± 1**	**0**	**± 2**	**± 2**	**± 2**	**± 3**	**± 3**	**±3**	**±7**	**±7**	**±7**	**±7**
**INVESTIGATIONAL PRODUCT**																	
Investigational Product /Placebo		X															
**CONSENT/ASSESSMENTS/COUNSELLING**																	
Informed Consent	X																
Assessment of Understanding	X																
HIV Risk Assessment[3]		X															X
HIV Risk Reduction Counselling[2]	X	X								X		X		X	X	X	X
HIV-test Counselling[3]	X	X															X
ART counseling[5]	X	X										X					X
Family Planning Counselling	X	X															
Social Impact Assessment																	X
**CLINICAL SAFETY ASSESSMENTS**																	
Comprehensive Medical History	X																
Interim Medical History		X	X	X	X	X	X	X	X	X	X	X					
Concomitant Medications	X	X	X	X	X	X	X	X	X	X	X	X					
General Physical Exam	X																X
Directed Physical Exam		X	X	X	X	X	X	X	X	X	X	X	X	X	X	X	
Weight	X	X															X
Height	X																
Vital Signs	X	X[4]	X	X	X	X	X	X	X	X	X	X	X	X	X	X	X
Local & Systemic Reactogenicity		X[4]	X	X	X												
Adverse Events		X	X	X	X	X	X	X	X	X	X	X					
Serious Adverse Events and pIMD	X	X	X	X	X	X	X	X	X	X	X	X	X	X	X	X	X

Study Month		0								1		2		3	4	5	6
**Study Week**		0				1		2	3	4	6	8	10	12	16	20	24
**Study Day**	Scr	0	1	2	3	7	10[5]	14	21	28	42	56	70	84	112	140	168/ET[9]
**Visit Windows (Days)**	-42	0	0	0	0	± 1	0	± 2	± 2	± 2	± 3	± 3	±3	±7	±7	±7	±7
**CLINICAL LABORATORY TESTS**																	
Hematology and Coagulation	X	X			X	X		X		X		X		X	X	X	X
CD4[1]	X	X				X		X		X		X					X
Clinical Chemistry	X	X			X	X		X		X		X		X	X	X	X
Urine Dipstick[11]	X	X			X	X		X		X		X		X	X	X	X
Urine Pregnancy test	X	X												X			X
Active Syphilis	X																
Hepatitis B	X																
Hepatitis C	X																
HIV diagnostic (4th generation Ag/Ab test)[3]	X	X															X
HIV Viral Load[1]	X	X	X	X	X	X	X	X	X	X	X	X	X	X	X	X	X
**RESEARCH LABORATORY TESTS**	-	-	-	-	-	-	-	-	-	-	-	-	-	-	-	-	-
Anti PGT121 Antibodies (ADA)		X								X		X		X			X
HIV testing for PGT121 susceptibility[6]	X									X							X
HIV SGA sequencing[12]		X								X							X
HIV genotypic testing for ART resistance[12]		X								X							X
HIV reservoir size assessment[1]	X					X				X							
Humoral Assays[7]		X			X	X		X		X		X		X			X
Cellular Assays[7]		X				X		X		X		X		X			X
HLA typing		X															
**PHARMACOKINETICS PGT121 ELISA**	X[7]	X[8]	X	X	X	X		X	X	X	X	X	X	X	X	X	X
**MUCOSAL SAMPLING**		X[10]	X			X		X									
**PLASMA/SERUM STORAGE**	X	X	X	X	X	X	X	X	X	X		X		X			X

Study Month		0								1		2		3	4	5	6
Study Week		0				1		2	3	4	6	8	10	12	16	20	24
Study Day	Scr	0	1	2	3	7	10[5]	14	21	28	42	56	70	84	112	140	168/ET[9]
Visit Windows (Days)	-42	0	0	0	0	± 1	0	± 2	± 2	± 2	± 3	± 3	±3	±7	±7	±7	±7
PBMCs STORAGE	X	X				X		X		X		X		X			X

1. For groups 2 and 3 only
2. Group 1: will receive HIV risk reduction counselling; Groups 2 and 3: HIV risk reduction counselling as secondary prevention to reduce onward transmission
3. Group 1 only
4. At baseline, approximately 30 minutes after IP administration start, and at hours 1 through 12 after IV infusion start. Local and systemic reactogenicity will be assessed by clinic staff at visits on study days 1, 2 and 3. Local and Systemic reactogenicity will also be assessed by the participant using the Memory Aid on study days 1, 2, and 3
5. Group 3 only
6. Baseline assessment of participants autologous HIV for neutralization susceptibility to PGT121 in-vitro (group 3 only).
7. See Laboratory Analytical Plan for details
8. Day 0 PK draws done immediately before IP administration, at the end of the IV infusion of IP, and 30 minutes and 3 hours post end of the IP administration. Additional PK draws on day 0 are done 6, 9 and 12 hours after the start of the IV infusion of IP. The screening sample is not a PK assessment per se, the PGT121 ELISA will be done to exclude autologous PGT121-like antibody levels above the cut-off. See SOM for details
9. Early Termination (ET): Procedures to be performed at ET are the same as last visit procedures
10. Cervico-vaginal and/or rectal mucosal sampling (optional) on Day 0 must be done prior to IV infusion of IP. See SOM for details
11. Urinalysis for group 3 will only be conducted at visits after screening if clinically indicated.
12. HIV envelope single genome amplification (SGA) sequencing and ART resistance genotyping will be performed in all subjects of group 3 and in subjects of group 2 only if they develop detectable plasma viremia >1000 HIV cp/ml during the study.

APPENDIX B: LOW RISK CRITERIA

Low risk will be defined as:

1. **SEXUAL BEHAVIORS**

   In the **last 12 months** did not:

   - Have oral, vaginal or anal intercourse with an HIV-infected partner, or partner who uses injection drugs.
   - Gave or receive money, drugs, gifts, or services in exchange for oral, vaginal or anal sex

   AND

   In the **last 6 months** has abstained from penile/anal or penile/vaginal intercourse

   OR

   In the **last 6 months**:

   - Had 4 or fewer partners of the opposite birth sex for vaginal and/or anal intercourse, OR

   Is MSM (person born male with partner(s) born male) who, in the **last 12 months**:

   - Had 2 or fewer MSM partners for anal intercourse and had no unprotected anal sex with MSM, OR
   - Had unprotected anal intercourse with only 1 MSM partner, within a monogamous relationship lasting at least 12 months (during which neither partner had any other partners). If the monogamous relationship ended, the participant may then have had protected anal intercourse with 1 other MSM partner (total 2 or fewer partners in the last 12 months).

   Is a transgender person, regardless of the point on the transition spectrum, having sex with men (born male) and/or other transgender persons, who in the last 12 months:

   - Had 2 or fewer partners for anal or vaginal intercourse, and had no unprotected anal or vaginal sex, OR
   - Had unprotected anal or vaginal intercourse sex with 1 partner only within a monogamous relationship lasting at least 12 months (during which neither partner had any other partners). If the monogamous relationship ended, may then have had protected anal or vaginal sex with one other partner (total 2 or fewer partners in the last 12 months).

   AND

   Uses or intends to use condoms in situations which may include penile/anal or penile/vaginal intercourse with new partners of unknown HIV status, occasional partners, partners outside a primary relationship, and/or partners known to have other partners.

2. **NON-SEXUAL BEHAVIORS**

   In the **last 12 months** did not:

   - Inject drugs or other substances without a prescription
   - Use cocaine, methamphetamine, or excessive alcohol, which in the investigator's judgement, rendered the participant at greater than low risk for acquiring HIV infection

The investigator's judgement should consider local epidemiologic information about HIV prevalence in the area and community networks.

A participant is NOT appropriate for inclusion if he/she:

Acquired an STI (ie, new infection) in the last 12 months:

- Syphilis
- Gonorrhea
- Non-gonococcal urethritis
- HSV-2
- Chlamydia
- Pelvic inflammatory disease (PID)
- Trichomonas
- Mucopurulent cervicitis
- Epididymitis
- Proctitis
- Lymphogranuloma venereum
- Chancroid
- Hepatitis B

APPENDIX C: ADVERSE EVENT SEVERITY ASSESSMENT TABLE

Adapted from: DIVISION OF AIDS (DAIDS) TABLE FOR GRADING THE SEVERITY OF ADULT AND PEDIATRIC ADVERSE EVENTS Version 2.0, November 2014

The *Division of AIDS* (*DAIDS) Table for Grading the Severity of Adult and Pediatric Adverse Events, Version 2.0* consists of parameters, or AEs, with severity grading guidance that are to be used in DAIDS clinical trials for safety data reporting to maintain accuracy and consistency in the evaluation of AEs.

I. Instructions and Clarifications

Grading Adult and Pediatric AEs
When a single parameter is not appropriate for grading an AE in both adult and pediatric populations, separate parameters with specified age ranges are provided. If no distinction between adult and pediatric populations has been made, the listed parameter should be used for grading an AE in both populations.

Note: The term "severe" is not the same as "serious" in classifying AEs. The severity of a specific event describes its intensity, and it is the intensity which is graded. Seriousness, which is not graded, relates to an outcome of an AE and is a regulatory definition.

Estimating Severity Grade for Parameters Not Identified in the Table
The functional table below should be used to grade the severity of an AE that is not specifically identified in the grading table. In addition, all deaths related to an AE are to be classified as grade 5.

PARAMETER	GRADE 1 MILD	GRADE 2 MODERATE	GRADE 3 SEVERE	GRADE 4 POTENTIALLY LIFE-THREATENING
**Clinical** adverse event **NOT** identified elsewhere in the grading table	Mild symptoms causing no or minimal interference with usual social & functional activities with intervention not indicated	Moderate symptoms causing greater than minimal interference with usual social & functional activities with intervention indicated	Severe symptoms causing inability to perform usual social & functional activities with intervention or hospitalization indicated	Potentially life-threatening symptoms causing inability to perform basic self-care functions with intervention indicated to prevent permanent impairment, persistent disability, or death

Determining Severity Grade for Parameters "Between Grades"
If the severity of an AE could fall in either one of two grades (e.g., the severity of an AE could be either Grade 2 or Grade 3), sites should select the higher of the two grades.

Laboratory Values
General. An asymptomatic, abnormal laboratory finding without an accompanying AE should not be reported to DAIDS in an expedited timeframe unless it meets protocol-specific reporting requirements. Sites should refer to the applicable network standards for reporting abnormal laboratory findings on the CRFs.

Values Below Grade 1
Any laboratory value that is between the ULN and grade 1 (for high values) or the LLN and grade 1 (for low values) should not be graded or reported as an AE. Sites should consult the Manual for Expedited Reporting of Adverse Events to DAIDS, Version 2.0 and their protocol when making an assessment of the need to report an AE.

Overlap of Local Laboratory Normal Values with Grading Table Ranges.
When local laboratory normal values fall within grading table laboratory ranges, the severity grading is based on the ranges in the grading table unless there is a protocol-specific grading criterion for the laboratory value. For example, "Magnesium, Low" has a grade 1 range of 1.2 to < 1.4 mEq/L, while a

particular laboratory's normal range for magnesium may be 1.3 to 2.8 mEq/L. If a study participant's magnesium laboratory value is 1.3 mEq/L, the laboratory value should be graded as grade 1.

II. **Definitions of terms used in the Table:**

Basic Self-care Functions	Adult<br>Activities such as bathing, dressing, toileting, transfer or movement, continence, and feeding.<br><br>Young Children<br>Activities that are age and culturally appropriate, such as feeding one's self with culturally appropriate eating implements.
LLN	Lower limit of normal
Intervention	Medical, surgical, or other procedures recommended or provided by a<br>healthcare professional for the treatment of an adverse event.
NA	Not Applicable
ULN	Upper limit of normal
Usual Social & Functional Activities	Activities which adults and children perform on a routine basis and<br>those which are part of regular activities of daily living, for example:<br><br>Adults<br>Adaptive tasks and desirable activities, such as going to work, shopping, cooking, use of transportation, or pursuing a hobby.<br><br>Young Children<br>Activities that are age and culturally appropriate, such as social interactions, play activities, or learning tasks.

Major Clinical Conditions

Cardiovascular

PARAMETER	GRADE 1 MILD	GRADE 2 MODERATE	GRADE 3 SEVERE	GRADE 4 POTENTIALLY LIFE-THREATENING
**Arrhythmia** (by ECG or physical examination) *Specify type, if applicable*	No symptoms AND No intervention indicated	No symptoms AND Non-urgent intervention indicated	Non-life-threatening symptoms AND Non-urgent intervention indicated	Life-threatening arrhythmia OR Urgent intervention indicated
**Blood Pressure Abnormalities**[1] ***Hypertension*** *(with the lowest reading taken after repeat testing during a visit)* *≥ 18 years of age*	140 to < 160 mmHg systolic OR 90 to < 100 mmHg diastolic	≥ 160 to < 180 mmHg systolic OR ≥ 100 to < 110 mmHg diastolic	≥ 180 mmHg systolic OR ≥ 110 mmHg diastolic	Life-threatening consequences in a participant not previously diagnosed with hypertension (e.g., malignant hypertension) OR Hospitalization indicated
< 18 years of age	> 120/80 mmHg	≥ 95th to < 99th percentile + 5 mmHg adjusted for age, height, and gender (systolic and/or diastolic)	≥ 99th percentile + 5 mmHg adjusted for age, height, and gender (systolic and/or diastolic)	Life-threatening consequences in a participant not previously diagnosed with hypertension (e.g., malignant hypertension) OR Hospitalization indicated
Hypotension	No symptoms	Symptoms corrected with oral fluid replacement	Symptoms AND IV fluids indicated	Shock requiring use of vasopressors or mechanical assistance to maintain blood pressure
**Cardiac Ischemia or Infarction** *Report only one*	NA	NA	New symptoms with ischemia (stable angina) OR New testing consistent with ischemia	Unstable angina OR Acute myocardial infarction
**Heart Failure**	No symptoms AND Laboratory or cardiac imaging abnormalities	Symptoms with mild to moderate activity or exertion	Symptoms at rest or with minimal activity or exertion (e.g., hypoxemia) OR Intervention indicated (e.g., oxygen)	Life-threatening consequences OR Urgent intervention indicated (e.g., vasoactive medications, ventricular assist device, heart transplant)
**Hemorrhage** (with significant acute blood loss)	NA	Symptoms AND No transfusion indicated	Symptoms AND Transfusion of ≤ 2 units packed RBCs indicated	Life-threatening hypotension OR Transfusion of > 2 units packed RBCs (for children, packed RBCs > 10 cc/kg) indicated

[1] Blood pressure norms for children < 18 years of age can be found in: Expert Panel on Integrated Guidelines for Cardiovascular Health and Risk Reduction in Children and Adolescents. *Pediatrics* 2011;128;S213; originally published online November 14, 2011; DOI: 10.1542/peds.2009- 2107C.

PARAMETER	GRADE 1 MILD	GRADE 2 MODERATE	GRADE 3 SEVERE	GRADE 4 POTENTIALLY LIFE-THREATENING
**Prolonged PR Interval or AV Block** *Report only one* *> 16 years of age*	PR interval 0.21 to < 0.25 seconds	PR interval ≥ 0.25 seconds OR Type I 2nd degree AV block	Type II 2nd degree AV block OR Ventricular pause ≥ 3.0 seconds	Complete AV block
≤ 16 years of age	1st degree AV block (PR interval > normal for age and rate)	Type I 2nd degree AV block	Type II 2nd degree AV block OR Ventricular pause ≥ 3.0 seconds	Complete AV block
**Prolonged QTc Interval**[2]	0.45 to 0.47 seconds	> 0.47 to 0.50 seconds	> 0.50 seconds OR ≥ 0.06 seconds above baseline	Life-threatening consequences (e.g., Torsade de pointes, other associated serious ventricular dysrhythmia)
**Thrombosis or Embolism** *Report only one*	NA	Symptoms AND No intervention indicated	Symptoms AND Intervention indicated	Life-threatening embolic event (e.g., pulmonary embolism, thrombus)

[2] As per Bazett's formula.

Dermatologic

PARAMETER	GRADE 1 MILD	GRADE 2 MODERATE	GRADE 3 SEVERE	GRADE 4 POTENTIALLY LIFE-THREATENING
**Alopecia** (scalp only)	Detectable by study participant, caregiver, or physician AND Causing no or minimal interference with usual social & functional activities	Obvious on visual inspection AND Causing greater than minimal interference with usual social & functional activities	NA	NA
**Bruising**	Localized to one area	Localized to more than one area	Generalized	NA
**Cellulitis**	NA	Non-parenteral treatment indicated (e.g., oral antibiotics, antifungals, antivirals)	IV treatment indicated (e.g., IV antibiotics, antifungals, antivirals)	Life-threatening consequences (e.g., sepsis, tissue necrosis)
**Hyperpigmentation**	Slight or localized causing no or minimal interference with usual social & functional activities	Marked or generalized causing greater than minimal interference with usual social & functional activities	NA	NA

**Hypopigmentation**	Slight or localized causing no or minimal interference with usual social & functional activities	Marked or generalized causing greater than minimal interference with usual social & functional activities	NA	NA
**Petechiae**	Localized to one area	Localized to more than one area	Generalized	NA
**Pruritus**[3]<br>(without skin lesions)	Itching causing no or minimal interference with usual social & functional activities	Itching causing greater than minimal interference with usual social & functional activities	Itching causing inability to perform usual social & functional activities	NA
**Rash**<br>*Specify type, if applicable*	Localized rash	Diffuse rash OR Target lesions	Diffuse rash AND Vesicles or limited number of bullae or superficial ulcerations of mucous membrane limited to one site	Extensive or generalized bullous lesions OR Ulceration of mucous membrane involving two or more distinct mucosal sites OR Stevens-Johnson syndrome OR Toxic epidermal necrolysis

[3] For pruritus associated with injections or infusions, see the *Site Reactions to Injections and Infusions* section (page 23).

Endocrine and Metabolic

PARAMETER	GRADE 1<br>MILD	GRADE 2<br>MODERATE	GRADE 3<br>SEVERE	GRADE 4<br>POTENTIALLY LIFE-THREATENING
**Diabetes Mellitus**	Controlled without medication	Controlled with medication OR Modification of current medication regimen	Uncontrolled despite treatment modification OR Hospitalization for immediate glucose control indicated	Life-threatening consequences (e.g., ketoacidosis, hyperosmolar non-ketotic coma, end organ failure)
**Gynecomastia**	Detectable by study participant, caregiver, or physician AND Causing no or minimal interference with usual social & functional activities	Obvious on visual inspection AND Causing pain with greater than minimal interference with usual social & functional activities	Disfiguring changes AND Symptoms requiring intervention or causing inability to perform usual social & functional activities	NA

**Hyperthyroidism**	No symptoms AND Abnormal laboratory value	Symptoms causing greater than minimal interference with usual social & functional activities OR Thyroid suppression therapy indicated	Symptoms causing inability to perform usual social & functional activities OR Uncontrolled despite treatment modification	Life-threatening consequences (e.g., thyroid storm)
**Hypothyroidism**	No symptoms AND Abnormal laboratory value	Symptoms causing greater than minimal interference with usual social & functional activities OR Thyroid replacement therapy indicated	Symptoms causing inability to perform usual social & functional activities OR Uncontrolled despite treatment modification	Life-threatening consequences (e.g., myxedema coma)
**Lipoatrophy**[4]	Detectable by study participant, caregiver, or physician AND Causing no or minimal interference with usual social & functional activities	Obvious on visual inspection AND Causing greater than minimal interference with usual social & functional activities	Disfiguring changes	NA
**Lipohypertrophy**[5]	Detectable by study participant, caregiver, or physician AND Causing no or minimal interference with usual social & functional activities	Obvious on visual inspection AND Causing greater than minimal interference with usual social & functional activities	Disfiguring changes	NA

4 Definition: A disorder characterized by fat loss in the face, extremities, and buttocks.
5 Definition: A disorder characterized by abnormal fat accumulation on the back of the neck, breasts, and abdomen.

Gastro-intestinal

PARAMETER	GRADE 1 MILD	GRADE 2 MODERATE	GRADE 3 SEVERE	GRADE 4 POTENTIALLY LIFE-THREATENING
**Anorexia**	Loss of appetite without decreased oral intake	Loss of appetite associated with decreased oral intake without significant weight loss	Loss of appetite associated with significant weight loss	Life-threatening consequences OR Aggressive intervention indicated (e.g., tube feeding, total parenteral nutrition)
**Ascites**	No symptoms	Symptoms AND Intervention indicated (e.g., diuretics, therapeutic paracentesis)	Symptoms recur or persist despite intervention	Life-threatening consequences

**Bloating or Distension**<br>*Report only one*	Symptoms causing no or minimal interference with usual social & functional activities	Symptoms causing greater than minimal interference with usual social & functional activities	Symptoms causing inability to perform usual social & functional activities	NA
**Cholecystitis**	NA	Symptoms AND Medical intervention indicated	Radiologic, endoscopic, or operative intervention indicated	Life-threatening consequences (e.g., sepsis, perforation)
**Constipation**	NA	Persistent constipation requiring regular use of dietary modifications, laxatives, or enemas	Obstipation with manual evacuation indicated	Life-threatening consequences (e.g., obstruction)
**Diarrhea**<br>*≥ 1 year of age*	Transient or intermittent episodes of unformed stools OR Increase of ≤ 3 stools over baseline per 24-hour period	Persistent episodes of unformed to watery stools OR Increase of 4 to 6 stools over baseline per 24-hour period	Increase of ≥ 7 stools per 24-hour period OR IV fluid replacement indicated	Life-threatening consequences (e.g., hypotensive shock)
< 1 year of age	Liquid stools (more unformed than usual) but usual number of stools	Liquid stools with increased number of stools OR Mild dehydration	Liquid stools with moderate dehydration	Life-threatening consequences (e.g., liquid stools resulting in severe dehydration, hypotensive shock)
**Dysphagia or Odynophagia**<br>*Report only one and specify location*	Symptoms but able to eat usual diet	Symptoms causing altered dietary intake with no intervention indicated	Symptoms causing severely altered dietary intake with intervention indicated	Life-threatening reduction in oral intake
**Gastrointestinal Bleeding**	Not requiring intervention other than iron supplement	Endoscopic intervention indicated	Transfusion indicated	Life-threatening consequences (e.g., hypotensive shock)

PARAMETER	GRADE 1<br>MILD	GRADE 2<br>MODERATE	GRADE 3<br>SEVERE	GRADE 4<br>POTENTIALLY LIFE-THREATENING
**Mucositis or Stomatitis**<br>*Report only one and specify location*	Mucosal erythema	Patchy pseudomembranes or ulcerations	Confluent pseudomembranes or ulcerations OR Mucosal bleeding with minor trauma	Life-threatening consequences (e.g., aspiration, choking) OR Tissue necrosis OR Diffuse spontaneous mucosal bleeding
**Nausea**	Transient (< 24 hours) or intermittent AND No or minimal interference with oral intake	Persistent nausea resulting in decreased oral intake for 24 to 48 hours	Persistent nausea resulting in minimal oral intake for > 48 hours OR Rehydration indicated (e.g., IV fluids)	Life-threatening consequences (e.g., hypotensive shock)

**Pancreatitis**	NA	Symptoms with hospitalization not indicated	Symptoms with hospitalization indicated	Life-threatening consequences (e.g., circulatory failure, hemorrhage, sepsis)
**Perforation** (colon or rectum)	NA	NA	Intervention indicated	Life-threatening consequences
**Proctitis**	Rectal discomfort with no intervention indicated	Symptoms causing greater than minimal interference with usual social & functional activities OR Medical intervention indicated	Symptoms causing inability to perform usual social & functional activities OR Operative intervention indicated	Life-threatening consequences (e.g., perforation)
**Rectal Discharge**	Visible discharge	Discharge requiring the use of pads	NA	NA
**Vomiting**	Transient or intermittent AND No or minimal interference with oral intake	Frequent episodes with no or mild dehydration	Persistent vomiting resulting in orthostatic hypotension OR Aggressive rehydration indicated (e.g., IV fluids)	Life-threatening consequences (e.g., hypotensive shock)

Musculoskeletal

PARAMETER	GRADE 1 MILD	GRADE 2 MODERATE	GRADE 3 SEVERE	GRADE 4 POTENTIALLY LIFE-THREATENING
**Arthralgia**	Joint pain causing no or minimal interference with usual social & functional activities	Joint pain causing greater than minimal interference with usual social & functional activities	Joint pain causing inability to perform usual social & functional activities	Disabling joint pain causing inability to perform basic self-care functions
**Arthritis**	Stiffness or joint swelling causing no or minimal interference with usual social & functional activities	Stiffness or joint swelling causing greater than minimal interference with usual social & functional activities	Stiffness or joint swelling causing inability to perform usual social & functional activities	Disabling joint stiffness or swelling causing inability to perform basic self-care functions
**Myalgia** (generalized)	Muscle pain causing no or minimal interference with usual social & functional activities	Muscle pain causing greater than minimal interference with usual social & functional activities	Muscle pain causing inability to perform usual social & functional activities	Disabling muscle pain causing inability to perform basic self-care functions
**Osteonecrosis**	NA	No symptoms but with radiographic findings AND No operative intervention indicated	Bone pain with radiographic findings OR Operative intervention indicated	Disabling bone pain with radiographic findings causing inability to perform basic self-care functions
**Osteopenia**[6]<br>*≥ 30 years of age*	BMD t-score -2.5 to -1	NA	NA	NA
< 30 years of age	BMD z-score -2 to -1	NA	NA	NA
**Osteoporosis**[6]<br>*≥ 30 years of age*	NA	BMD t-score < -2.5	Pathologic fracture (e.g., compression fracture causing loss of vertebral height)	Pathologic fracture causing life-threatening consequences
< 30 years of age	NA	BMD z-score < -2	Pathologic fracture (e.g., compression fracture causing loss of vertebral height)	Pathologic fracture causing life-threatening consequences

[6] BMD t and z scores can be found in: Kanis JA on behalf of the World Health Organization Scientific Group (2007). Assessment of osteoporosis at the primary health-care level. Technical Report. World Health Organization Collaborating Centre for Metabolic Bone Diseases, University of Sheffield, UK. 2007: Printed by the University of Sheffield.

Neurologic

PARAMETER	GRADE 1<br>MILD	GRADE 2<br>MODERATE	GRADE 3<br>SEVERE	GRADE 4<br>POTENTIALLY LIFE-THREATENING
**Acute CNS Ischemia**	NA	NA	Transient ischemic attack	Cerebral vascular accident (e.g., stroke with neurological deficit)
**Altered Mental Status** (for Dementia, see *Cognitive, Behavioral, or Attentional Disturbance* below)	Changes causing no or minimal interference with usual social & functional activities	Mild lethargy or somnolence causing greater than minimal interference with usual social & functional activities	Confusion, memory impairment, lethargy, or somnolence causing inability to perform usual social & functional activities	Delirium OR Obtundation OR Coma
**Ataxia**	Symptoms causing no or minimal interference with usual social & functional activities OR No symptoms with ataxia detected on examination	Symptoms causing greater than minimal interference with usual social & functional activities	Symptoms causing inability to perform usual social & functional activities	Disabling symptoms causing inability to perform basic self-care functions
**Cognitive, Behavioral, or Attentional Disturbance** (includes dementia and attention deficit disorder) *Specify type, if applicable*	Disability causing no or minimal interference with usual social & functional activities OR Specialized resources not indicated	Disability causing greater than minimal interference with usual social & functional activities OR Specialized resources on part- time basis indicated	Disability causing inability to perform usual social & functional activities OR Specialized resources on a full-time basis indicated	Disability causing inability to perform basic self-care functions OR Institutionalization indicated
**Developmental Delay** *< 18 years of age* *Specify type, if applicable*	Mild developmental delay, either motor or cognitive, as determined by comparison with a developmental screening tool appropriate for the setting	Moderate developmental delay, either motor or cognitive, as determined by comparison with a developmental screening tool appropriate for the setting	Severe developmental delay, either motor or cognitive, as determined by comparison with a developmental screening tool appropriate for the setting	Developmental regression, either motor or cognitive, as determined by comparison with a developmental screening tool appropriate for the setting
**Headache**	Symptoms causing no or minimal interference with usual social & functional activities	Symptoms causing greater than minimal interference with usual social & functional activities	Symptoms causing inability to perform usual social & functional activities	Symptoms causing inability to perform basic self-care functions OR Hospitalization indicated OR Headache with significant impairment of alertness or other neurologic function

PARAMETER	GRADE 1<br>MILD	GRADE 2<br>MODERATE	GRADE 3<br>SEVERE	GRADE 4<br>POTENTIALLY LIFE-THREATENING
**Neuromuscular Weakness** (includes myopathy and neuropathy)<br>*Specify type, if applicable*	Minimal muscle weakness causing no or minimal interference with usual social & functional activities OR No symptoms with decreased strength on examination	Muscle weakness causing greater than minimal interference with usual social & functional activities	Muscle weakness causing inability to perform usual social & functional activities	Disabling muscle weakness causing inability to perform basic self-care functions OR Respiratory muscle weakness impairing ventilation
**Neurosensory Alteration** (includes paresthesia and painful neuropathy)<br>*Specify type, if applicable*	Minimal paresthesia causing no or minimal interference with usual social & functional activities OR No symptoms with sensory alteration on examination	Sensory alteration or paresthesia causing greater than minimal interference with usual social & functional activities	Sensory alteration or paresthesia causing inability to perform usual social & functional activities	Disabling sensory alteration or paresthesia causing inability to perform basic self-care functions
**Seizures**<br>***New Onset Seizure*** *≥ 18 years of age*	NA	NA	1 to 3 seizures	Prolonged and repetitive seizures (e.g., status epilepticus) OR Difficult to control (e.g., refractory epilepsy)
< 18 years of age (includes new or pre- existing febrile seizures)	Seizure lasting < 5 minutes with < 24 hours postictal state	Seizure lasting 5 to < 20 minutes with < 24 hours postictal state	Seizure lasting ≥ 20 minutes OR > 24 hours postictal state	Prolonged and repetitive seizures (e.g., status epilepticus) OR Difficult to control (e.g., refractory epilepsy)
Pre-existing Seizure	NA	Increased frequency from previous level of control without change in seizure character	Change in seizure character either in duration or quality (e.g., severity or focality)	Prolonged and repetitive seizures (e.g., status epilepticus) OR Difficult to control (e.g., refractory epilepsy)
**Syncope**	Near syncope without loss of consciousness (e.g., pre-syncope)	Loss of consciousness with no intervention indicated	Loss of consciousness AND Hospitalization or intervention required	NA

Pregnancy, Puerperium, and Perinatal

PARAMETER	GRADE 1 MILD	GRADE 2 MODERATE	GRADE 3 SEVERE	GRADE 4 POTENTIALLY LIFE-THREATENING
**Fetal Death or Stillbirth** (report using mother's participant ID) *Report only one*	NA	NA	Fetal loss occurring at ≥ 20 weeks gestation	NA
**Preterm Delivery**[7] (report using mother's participant ID)	Delivery at 34 to < 37 weeks gestational age	Delivery at 28 to < 34 weeks gestational age	Delivery at 24 to < 28 weeks gestational age	Delivery at < 24 weeks gestational age
**Spontaneous Abortion or Miscarriage**[8] (report using mother's participant ID) *Report only one*	Chemical pregnancy	Uncomplicated spontaneous abortion or miscarriage	Complicated spontaneous abortion or miscarriage	NA

[7] Definition: A delivery of a live-born neonate occurring at ≥ 20 to < 37 weeks gestational age.
[8] Definition: A clinically recognized pregnancy occurring at < 20 weeks gestational age.

Psychiatric

PARAMETER	GRADE 1 MILD	GRADE 2 MODERATE	GRADE 3 SEVERE	GRADE 4 POTENTIALLY LIFE-THREATENING
**Insomnia**	Mild difficulty falling asleep, staying asleep, or waking up early	Moderate difficulty falling asleep, staying asleep, or waking up early	Severe difficulty falling asleep, staying asleep, or waking up early	NA
**Psychiatric Disorders** (includes anxiety, depression, mania, and psychosis) *Specify disorder*	Symptoms with intervention not indicated OR Behavior causing no or minimal interference with usual social & functional activities	Symptoms with intervention indicated OR Behavior causing greater than minimal interference with usual social & functional activities	Symptoms with hospitalization indicated OR Behavior causing inability to perform usual social & functional activities	Threatens harm to self or others OR Acute psychosis OR Behavior causing inability to perform basic self-care functions
**Suicidal Ideation or Attempt** *Report only one*	Preoccupied with thoughts of death AND No wish to kill oneself	Preoccupied with thoughts of death AND Wish to kill oneself with no specific plan or intent	Thoughts of killing oneself with partial or complete plans but no attempt to do so OR Hospitalization indicated	Suicide attempted

Respiratory

PARAMETER	GRADE 1 MILD	GRADE 2 MODERATE	GRADE 3 SEVERE	GRADE 4 POTENTIALLY LIFE-THREATENING
**Acute Bronchospasm**	Forced expiratory volume in 1 second or peak flow reduced to ≥ 70 to < 80% OR Mild symptoms with intervention not indicated	Forced expiratory volume in 1 second or peak flow 50 to < 70% OR Symptoms with intervention indicated OR Symptoms causing greater than minimal interference with usual social & functional activities	Forced expiratory volume in 1 second or peak flow 25 to < 50% OR Symptoms causing inability to perform usual social & functional activities	Forced expiratory volume in 1 second or peak flow < 25% OR Life-threatening respiratory or Hemodynamic compromise OR Intubation
**Dyspnea or Respiratory Distress** *Report only one*	Dyspnea on exertion with no or minimal interference with usual social & functional activities OR Wheezing OR Minimal increase in respiratory rate for age	Dyspnea on exertion causing greater than minimal interference with usual social & functional activities OR Nasal flaring OR Intercostal retractions OR Pulse oximetry 90 to < 95%	Dyspnea at rest causing inability to perform usual social & functional activities OR Pulse oximetry < 90%	Respiratory failure with ventilator support indicated (e.g., CPAP, BPAP, intubation)

Sensory

PARAMETER	GRADE 1 MILD	GRADE 2 MODERATE	GRADE 3 SEVERE	GRADE 4 POTENTIALLY LIFE-THREATENING
**Hearing Loss** *≥ 12 years of age*	NA	Hearing aid or intervention not indicated	Hearing aid or intervention indicated	Profound bilateral hearing loss (> 80 dB at 2 kHz and above) OR Non-serviceable hearing (i.e., >50 dB audiogram and <50% speech discrimination)
< 12 years of age (based on a 1, 2, 3, 4, 6 and 8 kHz audiogram)	> 20 dB hearing loss at ≤ 4 kHz	> 20 dB hearing loss at > 4 kHz	> 20 dB hearing loss at ≥ 3 kHz in one ear with additional speech language related services indicated (where available) OR Hearing loss sufficient to indicate therapeutic intervention, including hearing aids	Audiologic indication for cochlear implant and additional speech-language related services indicated (where available)

**Tinnitus**	Symptoms causing no or minimal interference with usual social & functional activities with intervention not indicated	Symptoms causing greater than minimal interference with usual social & functional activities with intervention indicated	Symptoms causing inability to perform usual social & functional activities	NA
**Uveitis**	No symptoms AND Detectable on examination	Anterior uveitis with symptoms OR Medicamylasal intervention indicated	Posterior or pan- uveitis OR Operative intervention indicated	Disabling visual loss in affected eye(s)
**Vertigo**	Vertigo causing no or minimal interference with usual social & functional activities	Vertigo causing greater than minimal interference with usual social & functional activities	Vertigo causing inability to perform usual social & functional activities	Disabling vertigo causing inability to perform basic self-care functions
**Visual Changes** (assessed from baseline)	Visual changes causing no or minimal interference with usual social & functional activities	Visual changes causing greater than minimal interference with usual social & functional activities	Visual changes causing inability to perform usual social & functional activities	Disabling visual loss in affected eye(s)

Systemic

PARAMETER	GRADE 1 MILD	GRADE 2 MODERATE	GRADE 3 SEVERE	GRADE 4 POTENTIALLY LIFE-THREATENING
**Acute Allergic Reaction**	Localized urticaria (wheals) with no medical intervention indicated	Localized urticaria with intervention indicated OR Mild angioedema with no intervention indicated	Generalized urticaria OR Angioedema with intervention indicated OR Symptoms of mild bronchospasm	Acute anaphylaxis OR Life-threatening bronchospasm OR Laryngeal edema
**Chills**	Symptoms causing no or minimal interference with usual social & functional activities	Symptoms causing greater than minimal interference with usual social & functional activities	Symptoms causing inability to perform usual social & functional activities	NA
**Cytokine Release Syndrome**[9]	Mild signs and symptoms AND Therapy (i.e., antibody infusion) interruption not indicated	Therapy (i.e., antibody infusion) interruption indicated AND Responds promptly to symptomatic treatment OR Prophylactic medications indicated for ≤ 24 hours	Prolonged severe signs and symptoms OR Recurrence of symptoms following initial improvement	Life-threatening consequences (e.g., requiring pressor or ventilator support)
**Fatigue or Malaise** *Report only one*	Symptoms causing no or minimal interference with usual social & functional activities	Symptoms causing greater than minimal interference with usual social & functional activities	Symptoms causing inability to perform usual social & functional activities	Incapacitating symptoms of fatigue or malaise causing inability to perform basic self-care functions
**Fever** (non-axillary temperatures only)	38.0 to < 38.6°C or 100.4 to < 101.5°F	≥ 38.6 to < 39.3°C or ≥101.5 to < 102.7°F	≥ 39.3 to < 40.0°C or ≥ 102.7 to < 104.0°F	≥ 40.0°C or ≥ 104.0°F
**Pain**[10] (not associated with study agent injections and not specified elsewhere) *Specify location*	Pain causing no or minimal interference with usual social & functional activities	Pain causing greater than minimal interference with usual social & functional activities	Pain causing inability to perform usual social & functional activities	Disabling pain causing inability to perform basic self-care functions OR Hospitalization indicated
**Serum Sickness**[11]	Mild signs and symptoms	Moderate signs and symptoms AND Intervention indicated (e.g., antihistamines)	Severe signs and symptoms AND Higher level intervention indicated (e.g., steroids or IV fluids)	Life-threatening consequences (e.g., requiring pressor or ventilator support)

[9] ~~Definition: A disorder characterized by nausea~~, headache, tachycardia, hypotension, rash, and/or shortness of breath.
[10] For pain associated with injections or infusions, see the *Site Reactions to Injections and Infusions* section (page 23).
[11] Definition: A disorder characterized by fever, arthralgia, myalgia, skin eruptions, lymphadenopathy, marked discomfort, and/or dyspnea.

PARAMETER	GRADE 1 MILD	GRADE 2 MODERATE	GRADE 3 SEVERE	GRADE 4 POTENTIALLY LIFE-THREATENING
**Underweight**[12]<br>*> 5 to 19 years of age*	NA	WHO BMI z-score < -2 to ≤ -3	WHO BMI z-score < -3	WHO BMI z-score < -3 with life-threatening consequences
2 to 5 years of age	NA	WHO Weight-for-height z-score < -2 to ≤-3	WHO Weight-for- height z-score < -3	WHO Weight-for-height z-score < -3 with life-threatening consequences
< 2 years of age	NA	WHO Weight-for-length z-score < -2 to ≤-3	WHO Weight-for- length z-score < -3	WHO Weight-for-length z-score < -3 with life-threatening consequences
**Weight Loss** (excludes postpartum weight loss)	NA	5 to < 9% loss in body weight from baseline	≥ 9 to < 20% loss in body weight from baseline	≥ 20% loss in body weight from baseline OR Aggressive intervention indicated (e.g., tube feeding, total parenteral nutrition)

[12] WHO reference tables may be accesed by clicking the desired age range or by accessing the following URLs: http://www.who.int/growthref/who2007 bmi for age/en/ for participants > 5 to 19 years of age and http://www.who.int/childgrowth/standards/chart catalogue/en/ for those < 5 years of age.

Urinary

PARAMETER	GRADE 1 MILD	GRADE 2 MODERATE	GRADE 3 SEVERE	GRADE 4 POTENTIALLY LIFE-THREATENING
**Urinary Tract Obstruction**	NA	Signs or symptoms of urinary tract obstruction without hydronephrosis or renal dysfunction	Signs or symptoms of urinary tract obstruction with hydronephrosis or renal dysfunction	Obstruction causing life-threatening consequences

Site Reactions to Injections and Infusions

PARAMETER	GRADE 1 MILD	GRADE 2 MODERATE	GRADE 3 SEVERE	GRADE 4 POTENTIALLY LIFE-THREATENING
**Injection Site Pain or Tenderness**<br>*Report only one*	Pain or tenderness causing no or minimal limitation of use of limb	Pain or tenderness causing greater than minimal limitation of use of limb	Pain or tenderness causing inability to perform usual social & functional activities	Pain or tenderness causing inability to perform basic self-care function OR Hospitalization indicated

**Injection Site Erythema or Redness**[13] *Report only one* *> 15 years of age*	2.5 to < 5 cm in diameter OR 6.25 to < 25 cm^2 surface area AND Symptoms causing no or minimal interference with usual social & functional activities	≥ 5 to < 10 cm in diameter OR ≥ 25 to < 100 cm^2 surface area OR Symptoms causing greater than minimal interference with usual social & functional activities	≥ 10 cm in diameter OR ≥ 100 cm^2 surface area OR Ulceration OR Secondary infection OR Phlebitis OR Sterile abscess OR Drainage OR Symptoms causing inability to perform usual social & functional activities	Potentially lifethreatening consequences (e.g., abscess, exfoliative dermatitis, necrosis involving dermis or deeper tissue)
≤15 years of age	≤ 2.5 cm in diameter	> 2.5 cm in diameter with < 50% surface area of the extremity segment involved (e.g., upper arm or thigh)	≥ 50% surface area of the extremity segment involved (e.g., upper arm or thigh) OR Ulceration OR Secondary infection OR Phlebitis OR Sterile abscess OR Drainage	Potentially life-threatening consequences (e.g., abscess, exfoliative dermatitis, necrosis involving dermis or deeper tissue)
**Injection Site Induration or Swelling** *Report only one* *> 15 years of age*	Same as for **Injection Site Erythema or Redness,** *> 15 years of age*	Same as for **Injection Site Erythema or Redness,** *> 15 years of age*	Same as for **Injection Site Erythema or Redness,** *> 15 years of age*	Same as for **Injection Site Erythema or Redness,** *> 15 years of age*
≤ 15 years of age	Same as for **Injection Site Erythema or Redness,** *≤ 15 years of age*	Same as for **Injection Site Erythema or Redness,** *≤ 15 years of age*	Same as for **Injection Site Erythema or Redness,** *≤ 15 years of age*	Same as for **Injection Site Erythema or Redness,** *≤ 15 years of age*
**Injection Site Pruritus**	Itching localized to the injection site that is relieved spontaneously or in < 48 hours of treatment	Itching beyond the injection site that is not generalized OR Itching localized to the injection site requiring ≥ 48 hours treatment	Generalized itching causing inability to perform usual social & functional activities	NA

[13] Injection Site Erythema or Redness should be evaluated and graded using the greatest single diameter or measured surface area.

Laboratory Values

Chemistries

PARAMETER	GRADE 1 MILD	GRADE 2 MODERATE	GRADE 3 SEVERE	GRADE 4 POTENTIALLY LIFE-THREATENING
**Acidosis**	NA	pH ≥ 7.3 to < LLN	pH < 7.3 without life-threatening consequences	pH < 7.3 with life-threatening consequences
**Albumin, Low** (g/dL; *g/L*)	3.0 to < LLN *30 to < LLN*	≥ 2.0 to < 3.0 ≥ *20 to < 30*	< 2.0 *< 20*	NA
**Alkaline Phosphatase, High**	1.25 to < 2.5 x ULN	2.5 to < 5.0 x ULN	5.0 to < 10.0 x ULN	≥ 10.0 x ULN
**Alkalosis**	NA	pH > ULN to ≤ 7.5	pH > 7.5 without life-threatening consequences	pH > 7.5 with life-threatening consequences
**ALT or SGPT, High** *Report only one*	1.25 to < 2.5 x ULN	2.5 to < 5.0 x ULN	5.0 to < 10.0 x ULN	≥ 10.0 x ULN
**Amylase (Pancreatic) or Amylase (Total), High** *Report only one*	1.1 to < 1.5 x ULN	1.5 to < 3.0 x ULN	3.0 to < 5.0 x ULN	≥ 5.0 x ULN
**AST or SGOT, High** *Report only one*	1.25 to < 2.5 x ULN	2.5 to < 5.0 x ULN	5.0 to < 10.0 x ULN	≥ 10.0 x ULN
**Bicarbonate, Low** (mEq/L; *mmol/L*)	16.0 to < LLN *16.0 to < LLN*	11.0 to < 16.0 *11.0 to < 16.0*	8.0 to < 11.0 *8.0 to < 11.0*	< 8.0 *< 8.0*
**Bilirubin** ***Direct Bilirubin[14], High*** *> 28 days of age*	NA	NA	> ULN	> ULN with lifethreatening consequences (e.g., signs and symptoms of liver failure)
≤ 28 days of age	ULN to ≤ 1 mg/dL	> 1 to ≤ 1.5 mg/dL	> 1.5 to ≤ 2 mg/dL	> 2 mg/dL
Total Bilirubin, High *> 28 days of age*	1.1 to < 1.6 x ULN	1.6 to < 2.6 x ULN	2.6 to < 5.0 x ULN	≥ 5.0 x ULN
≤ 28 days of age	See Appendix A. Total Bilirubin for Term and Preterm Neonates	See Appendix A. Total Bilirubin for Term and Preterm Neonates	See Appendix A. Total Bilirubin for Term and Preterm Neonates	See Appendix A. Total Bilirubin for Term and Preterm Neonates
**Calcium, High** (mg/dL; *mmol/L*) *≥ 7 days of age*	10.6 to < 11.5 *2.65* to < *2.88*	11.5 to < 12.5 *2.88* to < *3.13*	12.5 to < 13.5 *3.13* to < *3.38*	≥ 13.5 ≥ *3.38*
< 7 days of age	11.5 to < 12.4 *2.88* to < *3.10*	12.4 to < 12.9 *3.10* to < *3.23*	12.9 to < 13.5 *3.23* to < *3.38*	≥ 13.5 ≥ *3.38*

PARAMETER	GRADE 1 MILD	GRADE 2 MODERATE	GRADE 3 SEVERE	GRADE 4 POTENTIALLY LIFE-THREATENING
**Calcium (Ionized), High** (mg/dL; *mmol/L*)	> ULN to < 6.0 *> ULN to < 1.5*	6.0 to < 6.4 *1.5 to < 1.6*	6.4 to < 7.2 *1.6 to < 1.8*	≥ 7.2<br>*≥ 1.8*
**Calcium, Low** (mg/dL; *mmol/L*)<br>*≥ 7 days of age*	7.8 to < 8.4 *1.95* to < *2.10*	7.0 to < 7.8<br>*1.75* to < *1.95*	6.1 to < 7.0 *1.53* to < *1.75*	< 6.1<br>*< 1.53*
< 7 days of age	6.5 to < 7.5 *1.63* to < *1.88*	6.0 to < 6.5 *1.50* to < *1.63*	5.50 to < 6.0 *1.38* to < *1.50*	< 5.50<br>*< 1.38*
**Calcium (Ionized), Low** (mg/dL; *mmol/L*)	< LLN to 4.0 *< LLN to 1.0*	3.6 to < 4.0 *0.9 to < 1.0*	3.2 to < 3.6 *0.8 to < 0.9*	< 3.2<br>*< 0.8*
**Cardiac Troponin I, High**	NA	NA	NA	Levels consistent with myocardial infarction or unstable angina as defined by the local laboratory
**Creatine Kinase, High**	3 to < 6 x ULN	6 to < 10 x ULN	10 to < 20 x ULN	≥ 20 x ULN
**Creatinine, High**	1.1 to 1.3 x ULN	> 1.3 to 1.8 x ULN OR Increase of<br>> 0.3 mg/dL above baseline	> 1.8 to < 3.5 x ULN OR Increase<br>of 1.5 to < 2.0 x above baseline	≥ 3.5 x ULN OR<br>Increase of ≥ 2.0 x above baseline
**Creatinine Clearance[15] or eGFR, Low**<br>*Report only one*	NA	< 90 to 60 ml/min or ml/min/1.73 m^2 OR<br>10 to < 30% decrease from baseline	< 60 to 30 ml/min or ml/min/1.73 m^2 OR<br>≥ 30 to < 50% decrease from baseline	< 30 ml/min or ml/min/1.73 m^2 OR<br>≥ 50% decrease from baseline or dialysis needed
**Glucose** (mg/dL; *mmol/L*)<br>***Fasting, High***	110 to 125 *6.11 to < 6.95*	> 125 to 250 *6.95 to < 13.89*	> 250 to 500 *13.89 to < 27.75*	> 500<br>*≥ 27.75*
Nonfasting, High	116 to 160 *6.44 to < 8.89*	> 160 to 250 *8.89 to < 13.89*	> 250 to 500 *13.89 to < 27.75*	> 500<br>*≥ 27.75*
**Glucose, Low** (mg/dL; *mmol/L*)<br>*≥ 1 month of age*	55 to 64 *3.05 to 3.55*	40 to < 55 *2.22 to < 3.05*	30 to < 40 *1.67 to < 2.22*	< 30<br>*< 1.67*
< 1 month of age	50 to 54 *2.78 to 3.00*	40 to < 50 *2.22 to < 2.78*	30 to < 40 *1.67 to < 2.22*	< 30<br>*< 1.67*
**Lactate, High**	ULN to < 2.0 x ULN without acidosis	≥ 2.0 x ULN without acidosis	Increased lactate with pH < 7.3 without life-threatening consequences	Increased lactate with pH < 7.3 with life-threatening consequences

[15] Use the applicable formula (i.e., Cockroft-Gault in mL/min or Schwatrz in mL/min/1.73m^2).

PARAMETER	GRADE 1 MILD	GRADE 2 MODERATE	GRADE 3 SEVERE	GRADE 4 POTENTIALLY LIFE-THREATENING
**Lipase, High**	1.1 to < 1.5 x ULN	1.5 to < 3.0 x ULN	3.0 to < 5.0 x ULN	≥ 5.0 x ULN
**Lipid Disorders** (mg/dL; *mmol/L*) ***Cholesterol, Fasting, High***				
≥18 years of age	200 to < 240 *5.18 to < 6.19*	240 to < 300 *6.19 to < 7.77*	≥ 300 *≥ 7.77*	NA
< 18 years of age	170 to < 200 *4.40 to < 5.15*	200 to < 300 *5.15 to < 7.77*	≥ 300 *≥ 7.77*	NA
LDL, Fasting, High *≥18 years of age*	130 to < 160 *3.37 to < 4.12*	160 to < 190 *4.12 to < 4.90*	≥ 190 *≥ 4.90*	NA
> 2 to < 18 years of age	110 to < 130 *2.85 to < 3.34*	130 to < 190 *3.34 to < 4.90*	≥ 190 *≥ 4.90*	NA
Triglycerides, Fasting, High	150 to 300 *1.71 to 3.42*	>300 to 500 *>3.42 to 5.7*	>500 to < 1,000 *>5.7 to 11.4*	> 1,000 *> 11.4*
**Magnesium**[16]**, Low** (mEq/L; *mmol/L*)	1.2 to < 1.4 *0.60 to < 0.70*	0.9 to < 1.2 *0.45 to < 0.60*	0.6 to < 0.9 *0.30 to < 0.45*	< 0.6 *< 0.30*
**Phosphate, Low** (mg/dL; *mmol/L*) *> 14 years of age*	2.0 to < LLN *0.81 to < LLN*	1.4 to < 2.0 *0.65 to < 0.81*	1.0 to < 1.4 *0.32 to < 0.65*	< 1.0 *< 0.32*
1 to 14 years of age	3.0 to < 3.5 *0.97 to < 1.13*	2.5 to < 3.0 *0.81 to < 0.97*	1.5 to < 2.5 *0.48 to < 0.81*	< 1.5 *< 0.48*
< 1 year of age	3.5 to < 4.5 *1.13 to < 1.45*	2.5 to < 3.5 *0.81 to < 1.13*	1.5 to < 2.5 *0.48 to < 0.81*	< 1.5 *< 0.48*
**Potassium, High** (mEq/L; *mmol/L*)	5.6 to < 6.0 *5.6 to < 6.0*	6.0 to < 6.5 *6.0 to < 6.5*	6.5 to < 7.0 *6.5 to < 7.0*	≥ 7.0 *≥ 7.0*
**Potassium, Low** (mEq/L; *mmol/L*)	3.0 to < 3.4 *3.0 to < 3.4*	2.5 to < 3.0 *2.5 to < 3.0*	2.0 to < 2.5 *2.0 to < 2.5*	< 2.0 *< 2.0*
**Sodium, High** (mEq/L; *mmol/L*)	146 to < 150 *146 to < 150*	150 to < 154 *150 to < 154*	154 to < 160 *154 to < 160*	≥ 160 *≥ 160*
**Sodium, Low** (mEq/L; *mmol/L*)	130 to < 135 *130 to < 135*	125 to < 130 *125 to < 135*	121 to < 125 *121 to < 125*	≤ 120 *≤ 120*
**Uric Acid, High** (mg/dL; *mmol/L*)	7.5 to < 10.0 *0.45 to < 0.59*	10.0 to < 12.0 *0.59 to < 0.71*	12.0 to < 15.0 *0.71 to < 0.89*	≥ 15.0 *≥ 0.89*

16 To convert a magnesium value from mg/dL to mmol/L, laboratories should multiply by 0.4114.

Hematology

PARAMETER	GRADE 1 MILD	GRADE 2 MODERATE	GRADE 3 SEVERE	GRADE 4 POTENTIALLY LIFE-THREATENING
**Absolute CD4+ Count, Low**<br>(cell/mm^3; *cells/L*)<br>*> 5 years of age*<br>*(not HIV infected)*	300 to < 400<br>*300 to < 400*	200 to < 300<br>*200 to < 300*	100 to < 200<br>*100 to < 200*	< 100<br>*< 100*
**Absolute Lymphocyte Count, Low**<br>(cell/mm^3; *cells/L*)<br>*> 5 years of age*<br>*(not HIV infected)*	600 to < 650<br>*0.600 x 10^9 to < 0.650 x 10^9*	500 to < 600<br>*0.500 x 10^9 to < 0.600 x 10^9*	350 to < 500<br>*0.350 x 10^9 to < 0.500 x 10^9*	< 350<br>*< 0.350 x 10^9*
**Absolute Neutrophil Count (ANC), Low**<br>(cells/mm^3; *cells/L*)<br>*> 7 days of age*	800 to 1,000<br>*0.800 x 10^9 to 1.000 x 10^9*	600 to 799<br>*0.600 x 10^9 to 0.799 x 10^9*	400 to 599<br>*0.400 x 10^9 to 0.599 x 10^9*	< 400<br>*< 0.400 x 10^9*
2 to 7 days of age	1,250 to 1,500<br>*1.250 x 10^9 to 1.500 x 10^9*	1,000 to 1,249<br>*1.000 x 10^9 to 1.249 x 10^9*	750 to 999<br>*0.750 x 10^9 to 0.999 x 10^9*	< 750<br>*< 0.750 x 10^9*
≤ 1 day of age	4,000 to 5,000<br>*4.000 x 10^9 to 5.000 x 10^9*	3,000 to 3,999<br>*3.000 x 10^9 to 3.999 x 10^9*	1,500 to 2,999<br>*1.500 x 10^9 to 2.999 x 10^9*	< 1,500<br>*< 1.500 x 10^9*
**Fibrinogen, Decreased**<br>(mg/dL; *g/L*)	100 to < 200<br>*1.00 to < 2.00*<br>OR<br>0.75 to < 1.00 x LLN	75 to < 100<br>*0.75 to < 1.00*<br>OR<br>≥ 0.50 to < 0.75 x LLN	50 to < 75<br>*0.50 to < 0.75*<br>OR<br>0.25 to < 0.50 x LLN	< 50<br>*< 0.50*<br>OR<br>< 0.25 x LLN<br>OR Associated with gross bleeding
**Hemoglobin[17], Low** (g/dL; *mmol/L*)[18]<br>*≥ 13 years of age*<br>*(male only)*	10.0 to 10.9<br>*6.19 to 6.76*	9.0 to < 10.0<br>*5.57 to < 6.19*	7.0 to < 9.0<br>*4.34 to < 5.57*	< 7.0<br>*< 4.34*
≥ 13 years of age<br>*(female only)*	9.5 to 10.4<br>*5.88 to 6.48*	8.5 to < 9.5<br>*5.25 to < 5.88*	6.5 to < 8.5<br>*4.03 to < 5.25*	< 6.5<br>*< 4.03*

[17] Male and female sex are defined as sex at birth.
[18] The conversion factor used to convert g/dL to mmol/L is 0.6206 and is the most commonly used conversion factor. For grading hemoglobin results obtained by an analytic method with a conversion factor other than 0.6206, the result must be converted to g/dL using the appropriate conversion factor for the particular laboratory.

PARAMETER	GRADE 1 MILD	GRADE 2 MODERATE	GRADE 3 SEVERE	GRADE 4 POTENTIALLY LIFE-THREATENING

57 days of age to < 13 years of age (male and female)	9.5 to 10.4<br>*5.88 to 6.48*	8.5 to < 9.5<br>*5.25 to < 5.88*	6.5 to < 8.5<br>*4.03 to < 5.25*	< 6.5<br>*< 4.03*
36 to 56 days of age (male and female)	8.5 to 9.6<br>*5.26 to 5.99*	7.0 to < 8.5<br>*4.32 to < 5.26*	6.0 to < 7.0<br>*3.72 to < 4.32*	< 6.0<br>*< 3.72*
22 to 35 days of age (male and female)	9.5 to 11.0<br>*5.88 to 6.86*	8.0 to < 9.5<br>*4.94 to < 5.88*	6.7 to < 8.0<br>*4.15 to < 4.94*	< 6.7<br>*< 4.15*
8 to < 21 days of age (male and female)	11.0 to 13.0<br>*6.81 to 8.10*	9.0 to < 11.0<br>*5.57 to < 6.81*	8.0 to < 9.0<br>*4.96 to < 5.57*	< 8.0<br>*< 4.96*
< 7 days of age (male and female)	13.0 to 14.0<br>*8.05 to 8.72*	10.0 to < 13.0<br>*6.19 to < 8.05*	9.0 to < 10.0<br>*5.59 to < 6.19*	< 9.0<br>*< 5.59*
**INR, High** (not on anticoagulation therapy)	1.1 to < 1.5 x ULN	1.5 to < 2.0 x ULN	2.0 to < 3.0 x ULN	≥ 3.0 x ULN
**Methemoglobin** (% hemoglobin)	5.0 to < 10.0%	10.0 to < 15.0%	15.0 to < 20.0%	≥ 20.0%
**PTT, High** (not on anticoagulation therapy)	1.1 to < 1.66 x ULN	1.66 to < 2.33 x ULN	2.33 to < 3.00 x ULN	≥ 3.00 x ULN
**Platelets, Decreased** (cells/mm^3; *cells/L*)	100,000 to < 124,999<br>*100.000 x 10^9 to < 124.999 x 10^9*	50,000 to < 100,000<br>*50.000 x 10^9 to < 100.000 x 10^9*	25,000 to < 50,000<br>*25.000 x 10^9 to < 50.000 x 10^9*	< 25,000<br>*< 25.000 x 10^9*
**PT, High** (not on anticoagulation therapy	1.1 to < 1.25 x ULN	1.25 to < 1.50 x ULN	1.50 to < 3.00 x ULN	≥ 3.00 x ULN
**WBC, Decreased** (cells/mm^3; *cells/L*)<br>*> 7 days of age*	2,000 to 2,499<br>*2.000 x 10^9 to 2.499 x 10^9*	1,500 to 1,999<br>*1.500 x 10^9 to 1.999 x 10^9*	1,000 to 1,499<br>*1.000 x 10^9 to 1.499 x 10^9*	< 1,000<br>*< 1.000 x 10^9*
< 7 days of age	5,500 to 6,999<br>*5.500 x 10^9 to 6.999 x 10^9*	4,000 to 5,499<br>*4.000 x 10^9 to 5.499 x 10^9*	2,500 to 3,999<br>*2.500 x 10^9 to 3.999 x 10^9*	< 2,500<br>*< 2.500 x 10^9*

Urinalysis

PARAMETER	GRADE 1	GRADE 2	GRADE 3	GRADE 4
**Glycosuria (**random collection tested by dipstick)	Trace to 1+ or ≤ 250 mg	2+ or > 250 to < 500 mg	> 2+ or > 500 mg	NA
**Hematuria** (not to be reported based on dipstick findings or on blood believed to be of menstrual origin)	6 to < 10 RBCs per high power field	≥ 10 RBCs per high power field	Gross, with or without clots OR<br>With RBC casts OR<br>Intervention indicated	Life-threatening consequences
**Proteinuria** (random collection tested by dipstick)	1+	2+	3+ or higher	NA

Page **82** of **83**

References

1 (UNAIDS)., J. U. N. P. o. H. A. The Gap Report., (UNAIDS, 2014).
2 UNAIDS. AIDS by the numbers 2015. (2015).
3 CDC. CDC. Vital Signs: HIV Diagnosis, Care, and Treatment Among Persons Living with HIV- United States 2011. *MMWR* **4**, 1-6 (2014).
4 Jardine, J. *et al.* Rational HIV immunogen design to target specific germline B cell receptors. *Science* **340**, 711-716, doi:10.1126/science.1234150 (2013).
5 Sok, D. *et al.* Promiscuous glycan site recognition by antibodies to the high-mannose patch of gp120 broadens neutralization of HIV. *Sci Transl Med* **6**, 236ra263, doi:10.1126/scitranslmed.3008104 (2014).
6 Caskey, M. *et al.* Viraemia suppressed in HIV-1-infected humans by broadly neutralizing antibody 3BNC117. *Nature* **522**, 487-491, doi:10.1038/nature14411 (2015).
7 Barouch, D. H. *et al.* Therapeutic efficacy of potent neutralizing HIV-1-specific monoclonal antibodies in SHIV-infected rhesus monkeys. *Nature* **503**, 224-228, doi:10.1038/nature12744 (2013).
8 Hessell, A. J. *et al.* Broadly neutralizing human anti-HIV antibody 2G12 is effective in protection against mucosal SHIV challenge even at low serum neutralizing titers. *PLoS Pathog* **5**, e1000433, doi:10.1371/journal.ppat.1000433 (2009).
9 Hessell, A. J. *et al.* Effective, low-titer antibody protection against low-dose repeated mucosal SHIV challenge in macaques. *Nat Med* **15**, 951-954, doi:10.1038/nm.1974 (2009).
10 Moldt, B. *et al.* Highly potent HIV-specific antibody neutralization in vitro translates into effective protection against mucosal SHIV challenge in vivo. *Proceedings of the National Academy of Sciences of the United States of America* **109**, 18921-18925, doi:10.1073/pnas.1214785109 (2012).
11 Walker, L. M. *et al.* Broad neutralization coverage of HIV by multiple highly potent antibodies. *Nature* **477**, 466-470, doi:10.1038/nature10373 (2011).
12 Haynes, B. F. & McElrath, M. J. Progress in HIV-1 vaccine development. *Curr Opin HIV AIDS* **8**, 326-332, doi:10.1097/COH.0b013e328361d178 (2013).
13 Burton, D. R. & Mascola, J. R. Antibody responses to envelope glycoproteins in HIV-1 infection. *Nat Immunol* **16**, 571-576, doi:10.1038/ni.3158 (2015).
14 Sok, D. *et al.* Recombinant HIV envelope trimer selects for quaternary-dependent antibodies targeting the trimer apex. *Proc Natl Acad Sci U S A* **111**, 17624-17629, doi:10.1073/pnas.1415789111 (2014).
15 Scheid, J. F. *et al.* Sequence and structural convergence of broad and potent HIV antibodies that mimic CD4 binding. *Science* **333**, 1633-1637, doi:10.1126/science.1207227 (2011).
16 Shingai, M. *et al.* Passive transfer of modest titers of potent and broadly neutralizing anti-HIV monoclonal antibodies block SHIV infection in macaques. *J Exp Med* **211**, 2061-2074, doi:10.1084/jem.20132494 (2014).
17 Lynch, R. M. *et al.* Virologic effects of broadly neutralizing antibody VRC01 administration during chronic HIV-1 infection. *Sci Transl Med* **7**, 319ra206, doi:10.1126/scitranslmed.aad5752 (2015).
18 Andrade, A. *et al.* Three distinct phases of HIV-1 RNA decay in treatment-naive patients receiving raltegravir-based antiretroviral therapy: ACTG A5248. *J Infect Dis* **208**, 884-891, doi:10.1093/infdis/jit272 (2013).

International AIDS Vaccine Initiative

DOCUMENT NUMBER:
DOCUMENT TITLE:
DOCUMENT NOTES:

Document Information

Revision:	Vault:
Status:	Document Type:

Date Information

Effective Date:	Expiration Date:
Release Date:	Next Review Date:

Control Information

Author:	Owner:
Previous Number:	Change Number:

Signature Manifest

Document Number: TMF-02-0166 **Revision:** 1
Title: Protocol PGT121

All dates and times are in Eastern Time Zone.

T001 Protocol v 2.0

Change Request Approval

Name/Signature	Title	Date	Meaning/Reason
Dani Vooijs (DVOOIJS)			
Michele Fong Lim (MFONGLIM)			
Katherine Crisafi (KCRISAFI)	Director Laboratory QA	12 Sep 2016, 04:04:00 PM	Approved

CMO Approval

Name/Signature	Title	Date	Meaning/Reason
Frances Priddy (FPRIDDY)	Chief Medical Officer	12 Sep 2016, 05:09:49 PM	Approved

QA Final Release

Name/Signature	Title	Date	Meaning/Reason
Dani Vooijs (DVOOIJS)			
Michele Fong Lim (MFONGLIM)			
Katherine Crisafi (KCRISAFI)	Director Laboratory QA	12 Sep 2016, 07:43:01 PM	Approved

Notify

Name/Signature	Title	Date	Meaning/Reason
Lisa Sunner (LSUNNER)		12 Sep 2016, 07:43:01 PM	Email Sent

**Protocol Title:**	A Phase 1 Randomized Placebo-controlled Clinical Trial of the Safety, Pharmacokinetics and Antiviral Activity of PGT121 Monoclonal Antibody (mAb) in HIV-uninfected and HIV-infected Adults
**Protocol Number:**	IAVI T001
**Phase:**	Phase 1
**Regulatory Investigational Product Number:**	New IND submission
**Sponsor:**	International AIDS Vaccine Initiative (IAVI)<br>125 Broad Street, 9th Floor<br>New York, New York 10004<br>USA
**Sponsor Status**	Not for-Profit Organization
**Date of Protocol Version:**	17 October 2016<br>03.0<br>Edits from IRB comments<br><br>09 September 2016<br>02.0<br>Revision to IRB Submission<br><br>05 August 2016<br>01.0<br>IRB Submission

THE CONFIDENTIAL INFORMATION IN THIS DOCUMENT IS PROVIDED TO YOU AS AN INVESTIGATOR, POTENTIAL INVESTIGATOR, OR CONSULTANT, FOR REVIEW BY YOU, YOUR STAFF, AND APPLICABLE INSTITUTIONAL REVIEW BOARDS (IRBS) AND/OR INDEPENDENT ETHICS COMMITTEES (IECS). IT IS UNDERSTOOD THAT THE INFORMATION WILL NOT BE DISCLOSED TO OTHERS, EXCEPT TO THE EXTENT NECESSARY TO OBTAIN ETHICAL AND REGULATORY APPROVAL FROM THE RESPECTIVE COMMITTEE'S AGENCIES AND INFORMED CONSENT FROM THOSE PERSONS TO WHOM THE INVESTIGATIONAL PRODUCT MAY BE ADMINISTERED.

SYNOPSIS

**TITLE**	A Phase 1 Randomized Placebo-controlled Clinical Trial of the Safety, Pharmacokinetics and Antiviral Activity of PGT121 Monoclonal Antibody (mAb) in HIV-uninfected and HIV-infected Adults
**PROTOCOL NUMBER**	IAVI T001
**PHASE**	Phase 1
**SPONSOR**	International AIDS Vaccine Initiative (IAVI)<br>125 Broad Street, 9th Floor<br>New York, New York 10004,<br>USA
**SPONSOR STATUS**	Not for Profit Organization
**STUDY PRODUCTS**	PGT121 monoclonal antibody (mAb)
**OBJECTIVES**	**Primary Objectives**<br>• To evaluate the safety and tolerability of a single intravenous (IV) infusion of PGT121 mAb at 3, 10, and 30 mg/kg in HIV-uninfected adults and HIV-infected adults<br>• To evaluate the pharmacokinetic (PK) profile of IV infusion of PGT121 mAb in HIV-uninfected and HIV-infected adults<br>• To evaluate the antiviral activity of IV infusion of PGT121 mAb in HIV-infected adults not on ART<br><br>**Secondary Objective**<br>• To determine if PGT121 induces anti-PGT121 antibodies<br>• To determine the effect of PGT121 mAb on CD4+ T cell counts in HIV-infected adults<br>• To determine if PGT121 mAb induces viral escape mutants in viremic HIV-infected adults not on ART<br><br>**Exploratory Objectives**<br>• To determine if PGT121 mAb has any impact on the host immune responses (i.e., HIV-specific cellular and humoral immune response)<br>• To determine the effect of PGT121 mAb on the size of the latent HIV reservoir in HIV-infected adults<br>• To determine PGT121 mAb levels in mucosal secretions in HIV-uninfected and HIV-infected adults<br>• To measure in vitro neutralization of HIV isolates with participant's serum post PGT121 mAb IV infusion<br>• To determine if PGT121 mAb has any impact on resistance mutations to ARVs

**ENDPOINTS**	***Primary**:*<br><br>*Safety and Tolerability:*<br>We will measure the following endpoints to evaluate the safety and tolerability of PGT121 mAb in HIV-uninfected and HIV-infected adults:<br><br>1. Proportion of participants with moderate or greater reactogenicity (e.g., solicited adverse events) for 3 days following IV infusion of PGT121 mAb.<br>2. Proportion of participants with moderate or greater and/or PGT121 mAb-related unsolicited adverse events (AEs), including safety laboratory (biochemical, hematological) parameters, following IV infusion of PGT121 mAb for the first 56 days post administration of Investigational Product.<br>3. Proportion of participants with PGT121 mAb-related serious adverse events (SAEs) throughout the study period.<br><br>*Pharmacokinetics:*<br>We will measure the following endpoints to determine the pharmacokinetics following IV infusion of PGT121 mAb in HIV-uninfected and HIV-infected adults:<br><br>1. Elimination half-life (t1/2)<br>2. Clearance (CL/F)<br>3. Volume of distribution (Vz/F)<br>4. Area under the concentration decay curve (AUC)<br>5. Impact of viral load and/or ART on PGT121 disposition (elimination half-life (t1/2), clearance (CL/F), volume of distribution (Vz/F), total exposure<br><br>*Antiviral activity:*<br>We will calculate the following endpoints to determine the antiviral activity following IV infusion of PGT121 mAb in viremic HIV-infected adults not on ART:<br><br>1. Change in plasma HIV-1 RNA levels from baseline (mean of pre-entry and entry values)<br><br>***Secondary**:*<br><br>*Anti-PGT121 antibodies:*<br>We will calculate the following endpoint to determine if anti-PGT121 antibodies are elicited following IV infusion of PGT121 mAb in HIV-uninfected and HIV-infected adults:<br><br>1. Change in serum anti-PGT121 antibody titers from baseline<br><br>*CD4+ T cell count:*<br>We will calculate the following endpoint to determine if IV infusion of PGT121 mAb has any impact on CD4+ T cell counts in HIV-infected

adults:

1. Change in CD4+ T cell count and frequency compared to baseline as measured by single platform flow cytometry

HIV genotyping/phenotyping of circulating virus for evaluation of PGT121 mAb -induced escape mutations:
We will compare plasma virus sequences before and after IV infusion of PGT121 mAb to determine if PGT121 mAb induces viral escape mutations in viremic HIV-infected adults not on ART.

1. Development of sequence variations in epitopes known to result in reduced PGT121 mAb neutralization susceptibility.

Exploratory:

Additional assessments may include, but are not limited to, the following: HIV-specific IgG/IgA binding responses by ELISA, HIV-specific cellular immune responses by ELISPOT, HIV-specific antibody function by ADCC, ADCP, and ADCVI assays, resistance mutations to current ARVs, PGT121 mAb levels in mucosal secretions, changes in total HIV-1 DNA and 2-long terminal repeat (LTR) circular HIV-1 DNA in resting or total CD4 T cells and in vitro neutralization of HIV isolates with participant's serum post IV infusion of PGT121 mAb.

STUDY DESIGN TABLE

	Group	Participants	Sub-Group	Regimen	N	Dose (mg/kg)
Part 1[1]	1[3]	HIV-uninfected participants	1A	PGT121/Placebo	4/1 (6/2 if DLT[2])	3
			1B	PGT121/Placebo	4/1 (6/2 if DLT)	10
			1C	PGT121/Placebo	4/1 (6/2 if DLT)	30
	2[3]	HIV-infected on ART, (<50 cp/ml)	2A	PGT121/Placebo	4/1 (6/2 if DLT[2])	3
			2B	PGT121/Placebo	4/1 (6/2 if DLT)	10
			2C	PGT121/Placebo	4/1 (6/2 if DLT)	30
Safety Monitoring Committee Review[4]						
Part 2	3[5]	HIV-Infected off ART (VL $2x10^3$ – $1x10^5$cp/ml)	3A[6]	PGT121	6 (max 9)	30
			3B	PGT121	8 (max 12)	10
			3C	PGT121	10 (max 15)	3
		HIV-Infected off ART (VL $1x10^2$ – $2x10^3$ cp/ml)	3D[7]	PGT121	3	30
			3E	PGT121	3	10
			3F	PGT121	3	3

DLT, dose limiting toxicity; ART, antiretroviral therapy; cp, copies; ml, milliliter
Administration of PGT 121 will be by intravenous infusion (IV)

1. Eligible participants for Groups 1 and 2 will be enrolled according to their HIV-serostatus and will occur in parallel. At each dose level in Part 1, investigational product (IP) administration will be separated by at least 4 days for each of the first 3 participants. Randomization will ensure at least 2 participants receive active product and are observed for at least 4 days before administration to additional participants.
2. A dose limiting toxicity (DLT) will be defined as 1) any Grade 3 or greater reactogenicity or adverse event judged by the study investigators as at least possibly related to investigational product or 2) any SAE considered at least possibly related to investigational product.
3. Within each group, the PSRT will review data. If no DLT occurs within 2 weeks from infusion of the 5 participants in a dose group, dose escalation to the next dose group will proceed. If 1 DLT occurs, 3 additional participants will be enrolled; randomized between PGT121 mAb and placebo at a 2:1 ratio. If no additional DLTs occur within 2 weeks of infusion in the 8 total participants (3:1 ratio of PGT121 mAb to placebo), study can proceed with enrolment of the next dose group. If 2 or more DLTs accumulate that are the same, similar, or in the same System Organ Class, dosing will be halted and the next lower dose level will be declared the maximum tolerated dose (MTD). If no DLT occurs in the final dose group, MTD will be the highest dose given (subgroups 1C and 2C 30mg/kg) after 14 days of follow-up.
4. Following IP administration of the last participant in both Groups 1 and 2, an independent Safety Monitoring Committee (SMC) will review at least the first 14 days of safety data to confirm MTD in each group, and determine whether, and at what dose, Group 3 can initiate enrollment.
5. Group 3 will start with the MTD as determined in Part 1. Group 3 will start with subgroups 3A and

	3D if the MTD is 30mg/kg, subgroups 3B and 3E if the MTD is 10mg/kg and subgroups 3C and 3F if the MTD is 3mg/kg.<br>6. If subgroup 3A achieves a mean decline in HIV RNA of ≥0.9 log compared to baseline, enrolment into subgroup 3A will be stopped and the next lower dose subgroup 3B will be initiated. The same rule will apply for progression from subgroup 3B to 3C. At least 6, 8 and 10 but up to 9, 12 and 15 participants, will be enrolled in subgroups 3A, 3B, and 3C respectively, until the minimum effective dose is determined. If a mean decline ≥0.9 log in HIV RNA is not achieved, enrollment will be stopped at the completion of enrolment at that dose level.<br>7. As soon as subgroup 3D has enrolled 3 participants, enrolment into subgroup 3E will be initiated. The same rule will apply for progression from subgroup 3E to 3F. In the event that dose de-escalation in subgroups 3A-C is stopped due to failure to meet virologic endpoints, dose de-escalation in subgroups 3D-F will continue.
**METHODS**	See Schedule of Procedures, Appendix A
**STUDY POPULATION**	The study population will include three different groups:<br>Group 1 will include HIV-uninfected males or females aged 18-50 years old who are willing to maintain low risk behavior for HIV infection; principal exclusion criteria include confirmed HIV-infection, pregnancy or lactation, significant acute or chronic disease and clinically significant laboratory abnormalities. Group 2 will include HIV-infected males or females aged 18-50 years old on a stable antiretroviral regimen with HIV-1 RNA plasma level <50 copies/ml, CD4 cell count > 300 cells/uL and CD4 nadir > 200 cell/uL; principal exclusion criteria include history of AIDS-defining illness, significant acute or chronic medical condition other than HIV infection, and clinically significant laboratory abnormalities. Group 3 will include HIV-infected males or females aged 18-50 years old, not on antiretroviral therapy for > 6 month with detectable HIV-1 RNA plasma level between 100 and 100,000 copies/ml, CD4 cell count > 300 cells/uL and CD4 nadir > 200 cell/uL; principal exclusion criteria include history of AIDS-defining illness, significant acute or chronic medical condition other than HIV infection, and clinically significant laboratory abnormalities.
**NUMBER OF PARTICIPANTS**	63-93 participants will be included.
**DOSE ESCALATION and PAUSE RULES**	The first part of this study is a dose-escalation trial in HIV-uninfected adults and HIV-infected adults on ART with suppressed viral load, as indicated in the study design table.<br><br>If 2 or more DLTs occur that are the same, similar, or in the same System Organ Class, dosing will be halted and the next lower dose level will be declared the maximum tolerated dose (MTD) within this group. If no DLT occurs in the final dose group, MTD will be the highest dose given (subgroups 1C and 2C 30mg/kg) after 14 days of follow-up.<br><br>The Protocol Safety Review Team (PSRT) will review safety data through at least day 14 post-IP administration for all participants in the 1st dose group (subgroups 1A and 2A) prior to allowing enrolment of participants into the 2nd dose group (subgroups 1B and 2B). The PSRT will review safety data through at least day 14 post-IP administration for all participants in the 2nd dose group (subgroups 1B and 2B) prior to allowing enrolment of participants into the 3rd dose group (subgroups 1C and 2C).

	Following IP administration of the last participant in both Groups 1 and 2, an independent Safety Monitoring Committee (SMC) will review safety data through at least day 14 post-IP administration for all participants to confirm MTD, and determine whether, and at what dose, Group 3 can initiate enrollment.<br><br>The second part of this study is a dose-de-escalation trial in HIV-infected adults not on ART, as indicated in the study design table.<br><br>The study will be paused for a safety review by the investigators and the independent SMC if 1) 1 or more participants experiences a Serious Adverse Event that is judged possibly, probably or definitely related to the IP, 2) There is a participant death assessed as possibly, probably or definitely related to the IP, 3) if 2 or more participants experience grade 3 adverse events in the same System Organ Class that are considered to be at least possibly related to IP or 4) any grade 4 adverse event. See protocol section 17.3.
**FORMULATIONS, VOLUMES AND ROUTES OF ADMINISTRATION**	**PGT121 mAb**: PGT121 mAb is a recombinant, fully human monoclonal antibody (mAb) of the IgG1 isotype that binds to the HIV envelope. The concentration and volume of product in each vial is 50 mg/mL, 6mL in each vial. PGT121 mAb will be given intravenously in this study.
**DURATION OF STUDY PARTICIPATION**	Participants will be screened up to 42 days before IP administration and will be followed for 24 weeks. The anticipated study duration for each participant is approximately 6 months from screening through last study visit. It is anticipated that it will take approximately 4.5 months to enroll Groups 1 and 2. It is anticipated that it will take approximately 8 months to enroll the entire study. The exact duration depends on the recruitment rate and how many participants will be required per dose group.
**RANDOMIZATION and BLINDING**	This is a randomized, placebo-controlled study for groups 1 and 2, and open label for group 3 who will not receive placebo.
**EVALUATION FOR INTERCURRENT HIV INFECTION:**	Participants in Group 1 (HIV-uninfected) will be tested for HIV according to the Schedule of Procedures. Test results will be interpreted according to a pre-determined diagnostic algorithm. HIV testing at additional time points may be performed upon the request of the participant and Principal Investigator or designee as medical or social circumstances warrant.
**SAFETY MONITORING AND STATISTICAL CONSIDE-RATIONS:**	All clinical trial data collected, identified only by a study identification number, will be entered into the clinical trial database.<br><br>Safety will continually be monitored by the Investigators, the Sponsor’s Medical Monitor and a Protocol Safety Review Team (PSRT); detailed pause criteria are pre-defined.<br><br>Safety data will be reviewed by an independent Safety Monitoring Committee (SMC). *Ad hoc* safety review may be specifically requested by the Sponsor, the Principal Investigators, Ethics Committees, Regulatory Authorities, or by the SMC. All clinical and routine laboratory data will be included in the safety analysis. At the end of the study, a full analysis will be prepared.

TABLE OF CONTENTS

ABBREVIATIONS **11**

CONTACT INFORMATION **12**

1.0 SIGNATURE PAGE **13**

2.0 INTRODUCTION AND BACKGROUND INFORMATION **14**

2.1 Study Rationale 14
2.2 Experience with PGT121 15

3.0 STUDY OBJECTIVES **15**

3.1 Primary Objectives 15
3.2 Secondary Objectives 15
3.3 Exploratory Objectives: 15

4.0 STUDY ENDPOINTS **15**

4.1 Study Endpoints 15
4.1.1 Primary Endpoints 15
4.1.2 Secondary Endpoints 16
4.1.3 Exploratory Endpoints 17

5.0 STUDY DESIGN **17**

5.1 Definition of Dose Limiting Toxicity and Maximum Tolerated Dose 17
5.2 Dose Escalation – Groups 1 and 2: Determination of Maximum Tolerated Dose 17
5.3 Dose De-Escalation- Group 3: Determination of Minimum Effective Dose 18
5.4 Duration of the Study 20
5.5 Study Population 20
5.6 Inclusion Criteria 20
5.7 Exclusion Criteria 22
5.8 Recruitment of Participants 26

6.0 STUDY VISITS **26**

6.1 Screening Period 26
6.2 IV infusion of PGT121 mAb Visit 27
6.3 Post-IV infusion of PGT121 mAb Visits 28
6.4 Additional Follow-up Visits 28
6.5 Unscheduled Visits 28
6.6 Final Study Visit or Early Termination Visit 28

7.0 STUDY PROCEDURES **28**

7.1 Informed Consent Process 28
7.2 Medical History and Physical Examination 29
7.3 HIV Testing and HIV-test Counselling (Group 1) 29
7.4 HIV Risk Reduction Counselling 30
7.5 Family Planning Counselling 30
7.6 ART Counselling (Group 3) 30
7.7 Specimens 30
7.8 Reimbursement 30
7.9 Randomization and Blinding 31
7.10 Un-blinding Procedure for Individual Participants 31
7.11 Assessment of IP related HIV sero-positivity 31

8.0 INVESTIGATIONAL PRODUCT 32
8.1 Description 32
8.2 Shipment and Storage 32
8.3 Preparation of Investigational Product (IP) 33
8.4 Administration of Investigational Product 33
8.5 Accountability and Disposal of Investigational Product 33
9.0 ASSESSMENTS 33
9.1 Safety Assessments 33
9.1.1 Local reactogenicity 34
9.1.2 Systemic reactogenicity 34
9.1.3 Vital Signs 34
9.1.4 Other Adverse Events 34
9.1.5 Concomitant Medications 34
9.1.6 Routine laboratory parameters 34
9.1.7 Specific screening tests: 35
9.2 Virologic Assessments 35
9.3 Exploratory Immunogenicity Assessments 36
9.3.1 Antibody Responses 36
9.3.2 Cellular Responses 36
9.3.3 PBMC, Serum and Plasma Storage 36
9.4 Other Assessments 37
9.4.1 HIV Antibody Testing 37
9.4.2 Pharmacokinetics 37
9.4.3 HLA Typing 37
9.4.5 Pregnancy Test 37
9.4.6 HIV Risk Assessment (Group 1) 37
9.4.7 Social Impact Assessment 38
10.0 ADVERSE EVENTS 38
10.1 Definition 38
10.2 Assessment of Severity of Adverse Events 38
10.3 Relationship to Investigational Product 38
10.4 Serious Adverse Events 39
10.5 Reporting Potential Immune-Mediated Diseases 40
10.6 Clinical Management of Adverse Events 41
10.7 Pregnancy 41
10.8 Intercurrent HIV Infection (Group 1) 42
11.0 MANAGEMENT OF HIV ISSUES DURING AND FOLLOWING STUDY 42
11.1 HIV Testing – Group 1 42
11.2 Social Discrimination as a Result of IP-related antibodies 43
11.3 HIV infection – Group 1 43
11.3.1 Counselling 43
11.3.2 Referral for Support/Care 43
12.0 WITHDRAWAL FROM STUDY 43
12.1 Deferral of IV infusion of IP 43
12.2 Withdrawal from the Study (Early Termination) 44
13.0 DATA HANDLING 44
13.1 Data Collection and Record Keeping at the Study Site 44

13.3 Data Entry at the Study Site 45
13.4 Data Analysis 45

14.0 STATISTICAL CONSIDERATIONS 45

14.1 Safety and Tolerability Analysis 45
14.2 Pharmacokinetic Analysis 46
14.3 Virologic Analysis for Dose De-escalation in Groups 3A-C 47
14.4 Analysis of Antiviral Activity and Dose De-escalation in Subgroups 3D-F 49
14.5 Secondary and Exploratory Immunologic and Virologic Analyses 49

15.0 QUALITY CONTROL AND QUALITY ASSURANCE 50

16.0 DATA AND BIOLOGICAL MATERIAL 50

17.0 ADMINISTRATIVE STRUCTURE 51

17.1 Protocol Safety Review Team 51
17.2 Safety Monitoring Committee (SMC) 51
17.2.1 Content of Interim Safety Review 51
17.2.2 SMC Review of Group 1 and 2 data prior to starting Group 3 52
17.3 Criteria for Pausing the Study 52
17.4 Study Supervision 53
17.5 Study Monitoring 53
17.6 Investigator's Records 53

18.0 INDEMNITY 54

19.0 PUBLICATION 54

20.0 ETHICAL CONSIDERATIONS 54

APPENDIX A: SCHEDULE OF PROCEDURES 55

APPENDIX B: LOW RISK CRITERIA 58

Laboratory Values 77
Chemistries 77
Hematology 80
Urinalysis 82

ABBREVIATIONS

Abbreviation	Term
**AE**	Adverse Event
**AIDS**	Acquired Immunodeficiency Syndrome
**ALT**	Alanine-Aminotransferase
**ART**	Antiretroviral Therapy
**AST**	Aspartate-Aminotransferase
**CFC**	Cytokine Flow Cytometry
**CMI**	Cell Mediated Immunity
**CRF**	Case Report Form
**CTL**	Cytotoxic T Lymphocyte
**DCC**	Data Coordinating Center
**DLT**	Dose Limiting Toxicity
**DNA**	Deoxyribonucleic Acid
**ELISA**	Enzyme Linked Immunosorbent Assay
**GCP**	Good Clinical Practice
**GFR**	Glomerular Filtration Rate
**HIV**	Human Immunodeficiency Virus
**HLA**	Human Leukocyte Antigen
**HSV**	Herpes Simplex Virus
**IAVI**	International AIDS Vaccine Initiative
**IDES**	Internet Data Entry System
**ICH**	International Council for Harmonization of Technical Requirements for Pharmaceuticals for Human Use
**IP**	Investigational Product
**IND**	Investigational New Drug Application
**IV**	Intravenous
**Kg**	Kilogram
**mAb**	Monoclonal Antibody
**mg**	Milligram
**MED**	Minimum Effective Dose
**MTD**	Maximum Tolerated Dose
**PCR**	Polymerase Chain Reaction
**PBMC**	Peripheral Blood Mononuclear Cells
**PK**	Pharmacokinetic
**RPR**	Rapid Plasma Reagin
**SAE**	Serious Adverse Event
**SIV**	Simian Immunodeficiency Virus
**SOP**	Standard Operating Procedure
**SOM**	Study Operations Manual
**SMC**	Safety Monitoring Committee
**STD**	Sexually Transmitted Disease
**TPHA**	Treponema Pallidum Hemagglutination

CONTACT INFORMATION

Detailed contact information provided in the Study Operation Manual (SOM)

**Sponsor Contact:**	
Frances Priddy MD MPH<br>Executive Director and Chief Medical Officer<br>International AIDS Vaccine Initiative<br>125 Broad Street, 9th Floor<br>New York, New York 10004	Phone: +1-212-328-7461<br>Mobile: +1-646-287-8943<br>Fax: +1-608-203-5501<br>E-mail: fpriddy@iavi.org
**Clinical Research Center Contacts:**	
Kathryn Stephenson MD MPH<br>Center for Virology and Vaccine Research<br>Clinical Trials Unit<br>Beth Israel Deaconess Medical Center<br>E / CLS – 1036<br>330 Brookline Avenue<br>Boston, Massachusetts 02215	Phone: +1-617-735-4556<br>Mobile: +1-917-836-9150<br>Fax: +1-617-735-4566<br>E-mail: kstephen@bidmc.harvard.edu

.

1.0 SIGNATURE PAGE

The signatures below constitute the approval of this protocol and the appendices and provide the necessary assurances that this study will be conducted in compliance with the protocol, Good Clinical Practices (GCP) and the applicable regulatory requirement(s).

Sponsor:

Signed: See electronic signature manifest Date:

Frances Priddy MD MPH
Executive Director and Chief Medical Officer, Medical Affairs, IAVI

Principal Investigator:

Signed: Date:

Name (please print):

Name of institution (please print):

2.0 INTRODUCTION AND BACKGROUND INFORMATION

More than 78 million people have been infected with HIV and 39 million people have died since the beginning of the AIDS epidemic[1]. In 2014, there were 1.2 million deaths attributable to HIV infection and 2 million newly infected with HIV[2]. One reason that such high rates of AIDS-related deaths continue to occur globally – despite the advent of drugs that are highly effective at suppressing HIV replication – is that only two in five people living with HIV have access to antiretroviral therapy[1]. The other reason for continued AIDS-related mortality is that ART does not cure HIV infection and must be maintained for a lifetime. Even in the United States (US), only 30% of the 1.2 million people living with HIV have suppressed HIV to undetectable levels, despite the fact that most HIV-infected people in the US have access to ART[3] It is clear that antiretroviral therapy is necessary but not sufficient to end the AIDS epidemic, both in the US and globally, and that novel efforts to prevent and treat HIV are critically needed.

2.1 Study Rationale

This is a Phase 1 study to evaluate the safety, tolerability, pharmacokinetics and anti-viral efficacy of the PGT121 monoclonal antibody for HIV prevention and therapy. PGT121 mAb is a recombinant human IgG1 monoclonal antibody that targets a V3 glycan-dependent epitope region of the HIV envelope protein[4,5]. PGT121 mAb was chosen for this study because it is potent, neutralizes a wide array of HIV viruses, and can prevent and treat simian-human immunodeficiency virus (SHIV) in rhesus monkeys.

The recent discovery of multiple potent and broadly neutralizing antibodies (bNAbs) against HIV has led to the re-emergence of the concept that antibodies may be useful for both prevention and therapy. HIV-specific antibodies that target the HIV envelope (Env) can prevent SHIV infection in rhesus monkeys and have shown to reduce HIV RNA levels in humans temporarily[6-10]. Until recently, these antibodies were few in number, targeted a narrow spectrum of HIV strains and Env epitopes, and were not potent enough for practical use. In the last five years, the field has changed dramatically: new developments in high throughput single-cell PCR-amplification and novel soluble Env baits have led to the isolation of new monoclonal antibodies with extraordinary potency and breadth[11]. These bNAbs may be effective for prevention of HIV infection when administered passively[12,13].

PGT121 mAb was selected for development because of the following critical attributes:

- PGT121 mAb is 10 to 100-fold more potent than the previous best-in-class CD4bs antibodies VRC01, VRC07, and 3BNC117 [11,14,15].
- PGT121 mAb affords superior protective efficacy against SHIV acquisition in monkeys compared to VRC01, 3BNC117, and 10-1074 [16] (and unpublished data).
- PGT121 mAb has superior therapeutic efficacy in SHIV-infected monkeys compared to VRC01, 3BNC117, and 10-1074 [7] (and unpublished data).
- PGT121 mAb may have a higher bar to escape in vivo as compared with other V3 glycan and CD4bs antibodies as a result of making multiple glycan contacts [14].
- PGT121 mAb combined with PGDM1400 (a novel bNab targeting the envelope trimer apex) neutralizes 98-99% of global HIV-1 viruses tested and has unparalleled potency with a median IC50 of 0.007 µg/ml[14].

The potency and breadth of PGT121 mAb, both alone and in combination with other bNAbs, raise the possibility that combinations may be effective for HIV prophylaxis at

low doses and against global viruses. An antibody that is effective at low doses may eventually be given subcutaneously, which would reduce the cost. It is these features that make PGT121 mAb particularly well-suited for preventing and/or treating HIV in the developing world, where it is critical that a public health intervention be low cost, easy to deliver, and effective in diverse settings.

2.2 Experience with PGT121

There is no previous clinical experience with PGT121 mAb. Several other HIV monoclonal antibodies are currently in clinical development as passive HIV immunoprophylaxis, or as potential therapeutics. Data from phase 1 studies shows acceptable preliminary safety and tolerability profiles for these products, but varying levels of anti-viral effects [6,17]. A comprehensive summary of phase 1 studies of HIV monoclonal antibodies can be found in the Investigator's Brochure.

3.0 STUDY OBJECTIVES

3.1 Primary Objectives

- To evaluate the safety and tolerability of a single intravenous (IV) infusion of PGT121 mAb at 3, 10, and 30 mg/kg in HIV-uninfected adults and HIV-infected adults.
- To evaluate the pharmacokinetic (PK) profile of IV infusion of PGT121 mAb in HIV-uninfected and HIV-infected adults.
- To evaluate the antiviral activity of IV infusion of PGT121 mAb in HIV-infected adults not on ART.

3.2 Secondary Objectives

- To determine if PGT121 mAb induces anti-PGT121 antibodies.
- To determine the effect of PGT121 mAb on CD4 T-cell counts in HIV-infected adults.
- To determine if PGT121 mAb induces viral escape mutants in viremic HIV-infected adults not on ART.

3.3 Exploratory Objectives:

- To determine if PGT121 mAb has any impact on the host immune responses (i.e., HIV-specific cellular and humoral immune response).
- To determine the effect of PGT121 mAb on the size of the latent HIV reservoir in HIV-infected adults.
- To determine PGT121 mAb levels in mucosal secretions in HIV-uninfected and HIV-infected adults.
- To measure in vitro neutralization of HIV isolates with participant's serum post PGT121 mAb IV infusion.
- To determine if PGT121 mAb has any impact on resistance mutations to ARVs.

4.0 STUDY ENDPOINTS

4.1 Study Endpoints

4.1.1 Primary Endpoints

Safety and Tolerability:

We will measure the following endpoints to evaluate the safety and tolerability of PGT121 mAb in HIV-uninfected and HIV-infected adults:

1. Proportion of participants with moderate or greater reactogenicity (e.g., solicited adverse events) for 3 days following IV infusion of PGT121 mAb.
2. Proportion of participants with moderate or greater and/or PGT121 mAb-related unsolicited adverse events (AEs), including safety laboratory (biochemical, hematological) parameters, following IV infusion of PGT121 mAb for the first 56 days post administration of Investigational Product.
3. Proportion of participants with PGT121 mAb-related serious adverse events (SAEs) throughout the study period.

Pharmacokinetics:

We will measure the following endpoints to determine the pharmacokinetics following IV infusion of PGT121 mAb in HIV-uninfected and HIV-infected adults:

1. Elimination half-life (t1/2)
2. Clearance (CL/F)
3. Volume of distribution (Vz/F)
4. Area under the concentration decay curve (AUC)
5. Impact of viral load and/or ART on PGT121 disposition (elimination half-life (t1/2), clearance (CL/F), volume of distribution (Vz/F), total exposure

Antiviral activity:

We will calculate the following endpoints to determine the antiviral activity following IV infusion of PGT121 mAb in viremic HIV-infected adults not on ART.

1. Change in plasma HIV-1 RNA levels from baseline (mean of pre-entry and entry values)

4.1.2 Secondary Endpoints

Anti-PGT121 antibodies:

We will calculate the following endpoint to determine if anti-PGT121 antibodies are elicited following IV infusion of PGT121 mAb in HIV-uninfected and HIV-infected adults:

1. Change in serum anti-PGT121 antibody titers from baseline

CD4+ T cell count:

We will calculate the following endpoint to determine if IV infusion of PGT121 mAb has any impact on CD4+ T cell counts in HIV-infected adults:

1. Change in CD4+ T cell count and frequency compared to baseline as measured by single platform flow cytometry

HIV genotyping/phenotyping of circulating virus for evaluation of PGT121-induced escape mutations:

We will compare plasma virus sequences before and after IV infusion of PGT121 mAb to determine if PGT121 mAb induces viral escape mutations in viremic HIV-infected adults not on ART.

1. Development of sequence variations in epitopes known to result in reduced PGT121 neutralization susceptibility

4.1.3 Exploratory Endpoints

Additional assessments may include but are not limited to the following: HIV-specific IgG/IgA binding responses by ELISA, HIV-specific cellular immune responses by ELISPOT, HIV-specific antibody function by ADCC, ADCP, and ADCVI assays, resistance mutations to current ARVs, PGT121 mAb levels in mucosal secretions, changes in total HIV-1 DNA and 2-long terminal repeat (LTR) circular HIV-1 DNA in resting or total CD4 T cells and in vitro neutralization of HIV isolates with participant's serum post IV infusion of PGT121 mAb.

5.0 STUDY DESIGN

The study is a randomized, placebo-controlled study for groups 1 and 2, and open label for group 3 who will not receive placebo.

5.1 Definition of Dose Limiting Toxicity and Maximum Tolerated Dose

A dose limiting toxicity (DLT) will be defined as 1) any Grade 3 or greater reactogenicity or adverse event judged by the study investigators as at least possibly related to investigational product or 2) any SAE considered at least possibly related investigational product.
Maximum Tolerated Dose (MTD) will be declared when 2 or more DLTs occur that are the same, similar, or in the same System Organ Class or if no DLT occurs in the final dose subgroup, MTD will be the highest dose given (groups 1C and 2C 30mg/kg) after 14 days of follow-up.

5.2 Dose Escalation – Groups 1 and 2: Determination of Maximum Tolerated Dose

In Groups 1 and 2, (Part 1), the administrations of PGT121 mAb escalate by dose as shown below in Table 5.3.1, Study Design (5 participants per dose subgroup, 4:1 ratio of IP to placebo for each dose subgroup).

Sentinel Recipients

Within each dose group (subgroups 1A and 2A, subgroups 1B and 2B, subgroups 1C and 2C), the first 3 participant infusions will be separated by at least 4 days, to allow for observation of Investigational product (IP)-related adverse events. Dose subgroups will be enrolled in parallel, meaning that the 1st participant may be from subgroup 1A, the 2nd from subgroup 2A, the 3rd from subgroup 2A, all with 4 days in between dosing.

Because there is 1 placebo in each dose subgroup and the subgroups are dosed in parallel, the first 3 recipients will be treated as sentinel recipients (randomization will ensure that at least 2 will receive the IP). If no reactogenicity and adverse events that are considered to be related to IP (possibly, probably or definitely related) and are graded as severe or worse (Grade 3 or 4 on the DAIDS Toxicity Table) occur within 4 days after infusion of the first participant, the second participant may be injected. If no events meeting the criteria described above occur within 4 days after the 3rd participant is infused, then the remainder of participants in that dose group will be infused. If events meeting the criteria described above do occur for the first 3 participants in a dose group, they will be reviewed by the Safety Monitoring Committee (SMC) to determine whether further infusions may proceed.

Dose Escalation and Determination of Maximum Tolerated Dose

Safety data through day 14 post-IP administration visit for all participants in the first dose group (1A and 2A) will be reviewed by the Protocol Safety Review Team (PSRT) prior to allowing enrollment of participants into the second dose group (1B and 2B). The review process will be repeated between the second and third (1C and 2C) dose groups. Following administration of IP for the last participant in both Groups 1 and 2, an independent Safety Monitoring Committee (SMC) will review safety data to confirm Maximum Tolerated Dose (MTD) and determine whether, and at what dose, Group 3 can initiate enrollment.

Within each group, if no DLT occurs within 2 weeks from infusion of the first 5 participants in a dose group, dose escalation to the next dose group will proceed. If 1 DLT occurs, 3 additional participants will be enrolled; randomized between PGT121 mAb and placebo at a 2:1 ratio. If no additional DLTs occur within 2 weeks of infusion in the 8 mAb total participants (3:1 ratio of PGT121 mAb to placebo), the study can proceed with enrollment of the next dose group. If 2 or more DLTs accumulate in a subgroup that are the same, similar, or in the same organ class, dosing will be halted and the next lower dose level will be declared the maximum tolerated dose (MTD). If no DLT occurs in the final dose group, MTD will be the highest dose given (subgroups 1C and 2C 30mg/kg) after 14 days of follow-up.

5.3 Dose De-Escalation- Group 3: Determination of Minimum Effective Dose

Upon approval by the SMC (see section 17.2.2), group 3 (Part 2), PGT121 mAb administrations will de-escalate by dose as shown below in Table 5.3.1.

Group 3 will start with the MTD (i.e. subgroups 3A and 3D if the MTD is 30 mg/kg, subgroups 3B and 3C if the MTD is 10 mg/kg, or subgroups 3C and 3F if the MTD is 3 mg/kg) as determined by the SMC from data in Part 1.

If subgroup 3A (n = 6) achieves a mean decline in HIV RNA of $\geq$0.9 log compared to baseline, enrollment into subgroup 3A will be stopped, and the next lower dose subgroup 3B will be initiated. The same rule will apply for progression from subgroup 3B to 3C. At least 6, 8 and 10 but up to 9, 12 and 15 participants will be enrolled in subgroups 3A, 3B and 3C respectively, until the minimum effective dose is determined. In each subgroup, if a mean decline >0.9 log in HIV RNA is not achieved, enrollment will be stopped at the completion of enrollment at that dose level.

Three participants will be enrolled in each group 3D, 3E and 3F. As soon as subgroup 3D has enrolled 3 participants, enrollment into subgroup 3E will be initiated. The same rule will apply for progression from subgroup 3E to 3F. In the event that dose de-escalation in subgroups 3A-C is stopped due to failure to meet virologic endpoints, dose de-escalation in subgroups 3D-F will continue.

Table 5.3.1 Study Design Table

	Group	Participants	Sub-Group	Regimen	N	Dose (mg/kg)
Part 1	1	HIV-uninfected participants	1A	PGT121/Placebo	4/1 (6/2 if DLT)	3
			1B	PGT121/Placebo	4/1 (6/2 if DLT)	10
			1C	PGT121/Placebo	4/1 (6/2 if DLT)	30
	2	HIV-infected on ART, (<50 cp/ml)	2A	PGT121/Placebo	4/1 (6/2 if DLT)	3
			2B	PGT121/Placebo	4/1 (6/2 if DLT)	10
			2C	PGT121/Placebo	4/1 (6/2 if DLT)	30
Safety Monitoring Committee Review						
Part 2	3	HIV-infected off ART (VL $2x10^3$ – $1x10^5$cp/ml)	3A	PGT121	6 (max 9)	30
			3B	PGT121	8 (max 12)	10
			3C	PGT121	10 (max 15)	3
		HIV-infected off ART (VL $1x10^2$ – $2x10^3$ cp/ml)	3D	PGT121	3	30
			3E	PGT121	3	10
			3F	PGT121	3	3

Table 5.3.2 Decision Tree, Groups 3A, 3B, 3C

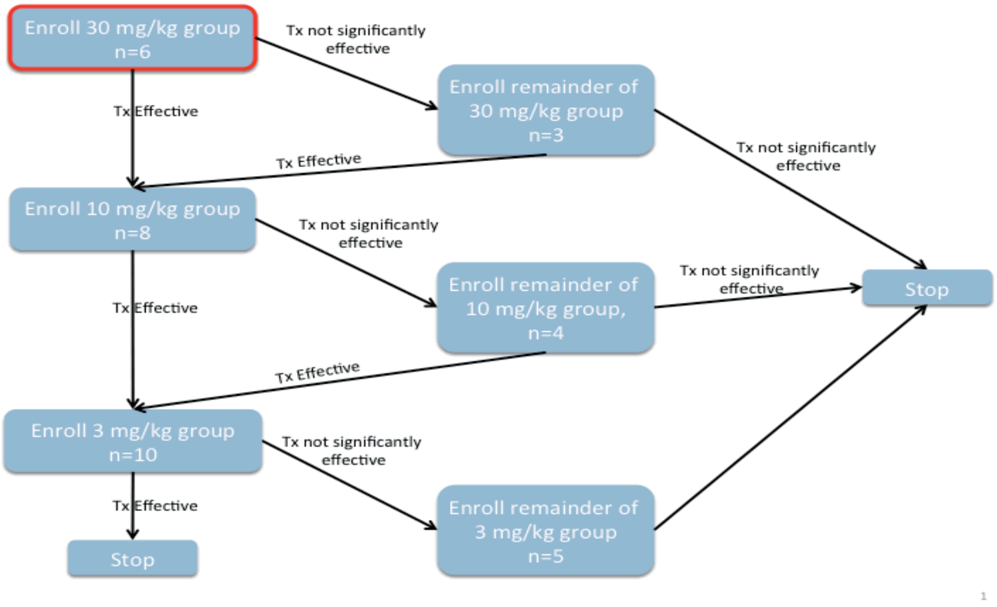

“not significantly effective” = does not achieve mean decrease of >0.9 log HIV RNA

5.4 Duration of the Study

Participants will be screened up to 42 days before IP administration of PGT121 mAb and will be followed for 24 weeks.

It will take approximately 8 months to enroll the entire study. The exact duration depends on the recruitment rate and how many participants will be required per dose group as specified in sections 5.2 and 5.3.

5.5 Study Population

The study population consists of HIV-uninfected male or female adults (Group 1), HIV-infected male or female adults on ART (Group 2), and HIV-infected males and female adults not on ART (group 3) who meet the detailed inclusion and exclusion criteria listed below, and who in the opinion of the investigator or designee, understand the study and provide written informed consent.

Approximately 63-93 participants (81 investigational product recipients, 12 placebo recipients) who meet all eligibility criteria will be included in the study. An over-enrollment of up to 5% (up to 5 participants total) will be permitted in the study to facilitate rapid enrollment.

5.6 Inclusion Criteria

Inclusion criteria for all participants:

1. At least 18 years of age on the day of screening and has not reached his or her 51st birthday on the day of signing the Informed Consent Document.

2. Willing to comply with the requirements of the protocol and available for follow-up for the planned duration of the study;

3. In the opinion of the Principal Investigator or designee and based on Assessment of Informed Consent Understanding results, has understood the information provided and potential impact and/or risks linked to IV infusion and participation in the trial; written informed consent will be obtained from the participant before any study-related procedures are performed;

4. All heterosexually active female participants must commit to use an effective method of contraception for 3 months following IP administration, including:
    a. Condoms (male or female) with or without spermicide
    b. Diaphragm or cervical cap with spermicide
    c. Intrauterine device, or contraceptive implant
    d. Hormonal contraception
    e. Successful vasectomy in the male partner (considered successful if a woman reports that a male partner has [1] documentation of azoospermia by microscopy (< 1 year ago), or [2] a vasectomy more than 2 years ago with no resultant pregnancy despite sexual activity post-vasectomy)
    f. Not be of reproductive potential, such as having undergone hysterectomy, bilateral oophorectomy, or tubal ligation, postmenopausal (>45 years of age with amenorrhea for at least 2 years, or any age with amenorrhea for at least 6 months and a serum follicle stimulating hormone [FSH] level >40 IU/L); surgically sterile: no additional contraception required.

   Women, who are not heterosexually active at screening, must agree to utilize an effective method of contraception if they become hetero-sexually active, as outlined above.

5. All sexually active males, regardless of reproductive potential, must be willing to consistently use an effective method of contraception (such as consistent male condoms with male and/or female partners from the day of IP administration until at least 3 months following IP administration to avoid exposure of partners to IP in ejaculate, and to prevent conception with female partners.

6. All female participants must be willing to undergo urine pregnancy tests at time points indicated in the Schedule of Procedures and must test negative prior to IP administration;

7. A woman must agree not to donate eggs (ova, oocytes) for the purpose of assisted reproduction until 3 months after receiving IP administration. A man must agree not to donate sperm until 3 months after IP administration;

8. Willing to forgo donations of blood and/or any other tissues, including bone marrow, during the study and, for those HIV-uninfected participants who test HIV-positive due to IP administration, until the anti-HIV antibody titers become undetectable.

Specific inclusion criteria for HIV-uninfected participants (Group 1):

9. Willing to undergo HIV testing, risk reduction counselling and receive HIV test results;

10. Low risk for HIV infection (see section (9.4.6) and willing to maintain low-risk behaviour for the duration of the trial (Appendix B);

11. Healthy male or female, as assessed by a medical history, physical exam, and laboratory tests;

Specific inclusion criteria for HIV-infected participants (Groups 2 and 3):

12. Confirmed HIV-1 infection (HIV Ab+ or HIV RNA+) by documentation in the medical records or in-clinic HIV testing;

13. CD4 ≥ 300 cells/µl;

14. No history of AIDS-defining illness or CD4 < 200 cells/µl.

<u>Group 2:</u>

15. Currently on ART, and documentation of continuous combination ART (cART) treatment with suppression of plasma HIV-1 viral load < 50 copies / ml for greater than 6 months, measured on at least 2 independent occasions, and with a viral load < 50 copies / ml at time of screening (within 42 days prior to IP administration). cART is defined as a regimen including > 2 compounds, e.g. 2x nucleoside reverse transcriptase inhibitors plus either non-nucleoside reverse transcriptase inhibitor or protease inhibitor or integrase inhibitor.

<u>Group 3:</u>

16. Not receiving cART, and (after appropriate counselling) willing to defer cART treatment for at least 56 days after administration of IP;

17. HIV-1 viral load either between 2000-100,000 copies / ml (Group 3A, 3B, 3C) or between 100-2000 copies / ml (Group 3D, 3E and 3F) at 2 independent occasions within 12 months prior to study enrollment, with confirmation during the screening period (3 viral loads on independent occasions).

5.7 Exclusion Criteria

Exclusion criteria for all participants:

1. Any clinically significant acute or chronic medical condition, other than HIV infection, that is considered progressive or in the opinion of the investigator makes the participant unsuitable for participation in the study;

2. If female, pregnant, lactating or planning a pregnancy during the period of screening through completion of the study;

3. In the past 6 months a history of alcohol or substance use, including marijuana, judged by the Investigator to potentially interfere with participant study compliance;

4. Bleeding disorder that was diagnosed by a physician (e.g., factor deficiency, coagulopathy or platelet disorder that requires special precautions). Note: A participant who states that he or she has easy bruising or bleeding, but does not have a formal diagnosis and has intramuscular injections and blood draws without any adverse experience, is eligible;

5. History of a splenectomy;

6. Receipt of live attenuated vaccine within the previous 60 days or planned receipt within 60 days after administration of IP; or receipt of other vaccine within the previous 14 days or planned receipt within 14 days after infusion with IP (exception is live attenuated influenza vaccine within 14 days);

7. Receipt of blood transfusion or blood-derived products within the previous 3 months;

8. Participation in another clinical trial of an investigational product currently, within the previous 3 months or expected participation during this study;

9. Prior receipt of an investigational HIV vaccine candidate, monoclonal antibody or polyclonal immunoglobulin (note: receipt of placebo in a previous HIV vaccine or monoclonal antibody trial will not exclude a participant from participation if documentation is available and the Medical Monitor gives approval);

10. History of severe local or systemic reactogenicity to injections or IV infusion (e.g., anaphylaxis, respiratory difficulties, angioedema);

11. HIV-specific antibodies that significantly cross-react with PGT121 mAb pharmacokinetic assays;

12. Psychiatric condition that compromises safety of the participant and precludes compliance with the protocol. Specifically excluded are persons with psychoses within the past 3 years, ongoing risk for suicide, or history of suicide attempt or gesture within the past 3 years;

13. If, in the opinion of the Principal Investigator, it is not in the best interest of the participant to participate in the trial;

14. Seizure disorder: a participant who has had a seizure in the last 3 years is excluded. (Not excluded: a participant with a history of seizures who has neither required medications nor had a seizure for 3 years.)

15. Body mass index ≥ 30 or ≤ 18.0.

16. Infectious disease: chronic hepatitis B infection (HbsAg), current hepatitis C infection (HCV Ab positive and HCV RNA positive) or interferon-alfa treatment for chronic hepatitis C infection in the past year, or active syphilis (RPR confirmed by TPHA).

17. A history of malignancy within the past 5 years (prior to screening) or ongoing malignancy;

18. Active, serious infections (other than HIV-1 infection) requiring parenteral antibiotic, antiviral or antifungal therapy within 30 days prior to enrollment;

Specific exclusion criteria for HIV-uninfected participants (Group 1):

19. Confirmed HIV-1 or HIV-2 infection;

20. Any clinically relevant abnormality on history or examination including history of immunodeficiency or autoimmune disease; use of systemic corticosteroids, immunosuppressive, anticancer, or other medications considered significant by the investigator within the previous 6 months.

    The following exceptions are permitted and will not exclude study participation: use of corticosteroid nasal spray for rhinitis, topical corticosteroids for an acute uncomplicated dermatitis; or a short course (duration of 10 days or less, or a single injection) of corticosteroid for a non-chronic condition (based on investigator clinical judgment) at least 6 weeks prior to enrollment in this study.

Specific exclusion criteria for HIV-uninfected participants (Group 1) and HIV-infected participants who are on ART (Group 2):

21. Any of the following abnormal laboratory parameters listed below:

    Hematology

    – Hemoglobin <10.5 g/dL in females; hemoglobin <11.0 g/dL in males
    – Absolute Neutrophil Count (ANC): ≤1000/mm^3
    – Absolute Lymphocyte Count (ALC): < 650/mm^3
    – Platelets: < 125,000 mm^3 or ≥ 550,000/mm^3

    Coagulation

    - aPTT: >1.25x ULN
    - INR: ≥1.1 x ULN

    Chemistry

    - Sodium ≤135 mEq/L or ≥ 146 mEq/L
    - Potassium ≤3.4 mEq/L or ≥ 5.6 mEq/L
    - Creatinine ≥1.1 x ULN
    - AST ≥1.25 x ULN
    - ALT ≥1.25 x ULN
    - Total bilirubin ≥1.25 x ULN
    - Alkaline phosphatase ≥1.25 x ULN
    - Albumin ≤ 3.0 g/dL or ≤ 30 g/L
    - Creatine kinase ≥3.0 x ULN
    - C-reactive protein >10 mg/L
    - C3 complement ≤ 0.9g/L
    - C4 complement ≤ 0.1g/L

Urinalysis

Clinically significant abnormal dipstick confirmed by microscopy:
– Protein = 1+ or more
– Blood = 1+ or more (not due to menses)

Specific exclusion criteria for HIV-infected participants who are on ART (Group 2) and for HIV-infected participants who are not on ART (Group 3):

22. Any clinically relevant abnormality on history or examination including history of immunodeficiency or autoimmune disease, other than HIV; use of systemic corticosteroids, immunosuppressive, anticancer, or other medications considered significant by the investigator within the previous 6 months.

    The following exceptions are permitted and will not exclude study participation: use of corticosteroid nasal spray for rhinitis, topical corticosteroids for an acute uncomplicated dermatitis; or a short course (duration of 10 days or less, or a single injection) of corticosteroid for a non-chronic condition (based on investigator clinical judgment) at least 6 weeks prior to enrollment in this study.

Specific exclusion criteria for HIV-infected participants who are not on ART (Group 3)

23. Resistance of autologous HIV to PGT121 neutralization in vitro;

24. Any of the following abnormal laboratory parameters listed below:

Hematology

– Hemoglobin < 10.0 g/dL
– Absolute Neutrophil Count (ANC): <800 cells/mm_3
– Platelets: < 100,000 cells/mm3

Coagulation

- aPTT: >1.25x ULN
- INR: ≥1.1 x ULN

Chemistry

- Estimated Glomerular filtration rate (GFR) ≤ 80 mL/min according to the Cockcroft-Gault formula for creatinine clearance:
  - Male: $\frac{(140 - \text{age in years}) \times (\text{wt in kg}) = \text{CLcr (mL/min)}}{72 \times (\text{serum creatinine in mg/dL})}$
  - Female: $\frac{(140 - \text{age in years}) \times (\text{wt in kg}) \times 0.85 = \text{CLcr (mL/min)}}{72 \times (\text{serum creatinine in mg/dL})}$
- AST ≥ 2.5 x ULN

- ALT ≥ 2.5 x ULN
- Total bilirubin ≥ 1.6 x ULN
- Alkaline phosphatase ≥ 5 x ULN

Urinalysis

- Any RBC, protein or leukocytes greater than 1+, confirmed by microscopy and consistent with clinically significant disease.

5.8 Recruitment of Participants

Adult male and female participants may be recruited through in-clinic referrals, information presented to community organizations, hospitals, colleges, other institutions and/or advertisements to the general public or from existing cohorts. The information distributed will contain contact details of the trial site.

6.0 STUDY VISITS

6.1 Screening Period

During Screening, study staff will perform the following procedures:

- Provide and/or review the Informed Consent Document and answer any questions about the study prior to obtaining written informed consent.
- Complete Assessment of Informed Consent Understanding (AOU). Please refer to the Study Operations Manual (SOM)

If the participant agrees to participate, passes the AOU and provides written informed consent, study staff will:

- Conduct HIV test counselling, HIV testing, and HIV risk reduction counselling, as applicable
- Conduct family planning counselling, refer for pregnancy prevention counselling if necessary
- Conduct ART counselling, if applicable
- Perform a comprehensive medical history
- Collect concomitant medication information
- Perform a general physical examination (Refer to Section 7.2)
- Collect specimens for all tests as indicated in the Schedule of Procedures in Appendix A (for details see Analytical Plan (AP).

When available, the screening laboratory tests will be reviewed by the trial physician. Screening laboratory test(s) may be repeated once at the discretion of the principal investigator or designee to investigate any isolated abnormalities.

If the screening visit occurs more than 42 days prior to the date of administration of IP, all screening procedures must be repeated except the comprehensive medical history may be replaced by an interim medical history and the Participant Information Sheet of the Informed Consent Document should be reviewed.

If a participant has signed the Consent Form but does not meet the eligibility criteria, the records must be kept at the site.

6.2 IV infusion of PGT121 mAb Visit

Prior to the administration of IP, study staff will:

- Answer any questions the participant may have about the study
- Review the Informed Consent Document with the participant
- Review screening safety laboratory data
- Administer HIV risk assessment (Group 1)
- Conduct HIV test counselling, and HIV risk reduction counselling
- Conduct family planning counselling as per site specific procedures and ensure compliance with respective pregnancy prevention method, and discuss male condom use with all male participants
- Review interim medical history
- Collect concomitant medication information
- Weigh participant and record vital signs
- Perform a symptom-directed physical examination (Refer to Section 7.2)
- Assess at baseline local and systemic signs and symptoms (this includes an examination of IV infusion site)
- Collect specimens for all tests as indicated in the Schedule of Procedures see Appendix A (for details see AP).
- Obtain pregnancy test results prior to administration of IP.

Assign an allocation number to the participant according to the instructions specified in the Study Operations Manual.

At the time of administration of IP and after IV infusion of IP, study staff will:

- Administer the IP as specified in Section 8.4, Administration of Investigational Product and according to the instructions specified in the SOM.
- Observe participant closely during the infusion of IP and for at least 30 minutes after IV infusion of IP has ended for any acute reactogenicity. At the end of the observation period study staff will:
    - Record vital signs (pulse, respiratory rate, blood pressure and temperature)
    - Assess any local and systemic reactogenicity
    - Assess any other adverse events
- Every hour after IV infusion of IP, starting hour 1 through 12, the study staff will:
    - Record vital signs (pulse, respiratory rate, blood pressure and temperature)
    - Assess any local and systemic reactogenicity
    - Assess any other adverse events
    - Collect PK samples according to the Schedule of Procedures

If a participant has an abnormal laboratory value that is known, at the time of infusion, follow the specified guidelines (Section 12.0).

6.3 Post-IV infusion of PGT121 mAb Visits

The participant will be asked to return to the clinic for post-IP administration visits as indicated in the Schedule of Procedures (see Appendix A) for an assessment by clinic staff. The participant will be asked to maintain a Memory Aid for local and systemic reactogenicity from the day of IP administration for the next 3 days (for a total of 4 days including day of IP administration). Study staff will review the Memory Aid with the participant and determine the severity of the reactions through discussion with the participant.

The following procedures will be conducted at these visits:

- Review interim medical history
- Collect concomitant medication information
- Perform a symptom-directed physical examination if any signs or symptoms are present
- Assess vital signs (pulse, respiratory rate, blood pressure and temperature)
- Assess any adverse events and local and systemic reactogenicity (Days 1, 2, 3) including reviewing the Memory Aid.
- Collect specimens for all tests as indicated in the Schedule of Procedures (Appendix A) and AP).

6.4 Additional Follow-up Visits

Assessments and procedures will be performed according to the Schedule of Procedures (Appendix A).

6.5 Unscheduled Visits

Unscheduled Visits/Contacts are visits/contacts that are not described in the Schedule of Procedures (Appendix A). Unscheduled visits may occur any time during the study:

- For administrative reasons, e.g., the participant may have questions for study staff or may need to re-schedule a follow-up visit.
- To obtain laboratory test results from a previous visit.
- For other reasons as requested by the participant or site investigator.

All unscheduled visits will be documented in the participants' study records on applicable source documents and entered into the Case Report Form (CRF).

6.6 Final Study Visit or Early Termination Visit

Assessments and procedures will be performed according to the Schedule of Procedures (Appendix A).

7.0 STUDY PROCEDURES

7.1 Informed Consent Process

A Master Informed Consent Document consisting of a Participant Information Sheet and a Consent Form is provided by the Sponsor to the trial site. This document is made site-specific and translated (if necessary), submitted and approved by the Institutional

Review Board (IRB). The Master and site specific Informed Consent Documents are separate documents and should not be part of the protocol.

Participant Information Sheet

A qualified member of the study staff will conduct the informed consent process by reviewing the Participant Information Sheet and document it in the clinic notes.

Consent Form

The participant's consent to participate must be obtained by him/her signing and dating the Consent Form. The person obtaining consent will also sign.

The signed and dated Informed Consent Document must remain at the study site. A copy of the signed/marked and dated Informed Consent Document will be offered to the participant to take home. Those participants who do not wish to take a copy will be required to document that they declined to do so.

7.2 Medical History and Physical Examination

Medical History
At screening, a comprehensive medical history will be collected including previous IV infusions and reaction to IV infusion, history of sexually transmitted infection (STI) and pregnancy prevention practices. At subsequent visits, an interim medical history will be performed.

Physical Examination

General Physical Examination
A general physical examination includes examination of head/ears/eyes/nose and throat, skin, respiratory, cardiovascular, abdominal, limited neurological and musculoskeletal and external ano-genital systems (for HIV-infected participants only) at the time points indicated in the Schedule of Procedures (see Appendix A).

Symptom-Directed Physical Examination
A symptom-directed physical examination is a targeted examination based on the participant's history or observation. If deemed necessary, this examination should be done at the time points indicated in the schedule of procedures (see Appendix A).

Measuring Height and Weight
Includes measuring the height and weight at the time points indicated in the Schedule of Procedures (see Appendix A).

Vital Signs
Vital signs including pulse, respiratory rate, blood pressure and temperature are measured and recorded at the time points indicated in the Schedule of Procedures (see Appendix A)

7.3 HIV Testing and HIV-test Counselling (Group 1)

Study staff will perform pre-HIV test counselling prior to collecting blood for an HIV test, and post-HIV test counselling when HIV test results are available. This is referred to as

HIV-test counselling, and done according to the CDC guidelines. For more information on HIV testing and HIV-test counselling, see Section 11.0. A screening questionnaire and other tools may be used.

7.4 HIV Risk Reduction Counselling

HIV risk reduction counselling will be provided to all participants as outlined by site-specific SOPs.

Study staff will provide HIV risk reduction counselling based on reported individual risk and provide free condoms, as appropriate, at every visit. Group 1 will receive HIV risk reduction counselling and for Groups 2 and 3, HIV risk reduction counselling will be conducted as secondary prevention to reduce onward transmission.

7.5 Family Planning Counselling

Study staff will counsel participants about the importance of preventing pregnancies and of using condoms, as well as other effective family planning methods, as appropriate. Participants may be referred for family planning services as necessary according to site-specific SOPs as detailed in the SOM. Pregnancy prevention methods chosen and compliance will be documented.

7.6 ART Counselling (Group 3)

HIV-infected participants who are not on ART will receive ART counselling upon entering the study and 8 weeks after administration of IP. Participants who have not initiated or made plans to initiate ART by the final study visit will receive ART counselling again at their final study visit.

7.7 Specimens

Approximately 150 ml of blood will be collected from participants in Groups 1 and 2, and approximately 205 ml of blood will be collected from participants in Group 3 at the screening visit. At later visits, approximately 8.5 ml to 214 ml of blood will be collected, depending on study procedures and group assignment (see Appendix A), usually from the antecubital fossa.

Optional collection of rectal and/or cervical mucosal secretions will be obtained using a rectal sponge or cervical Softcup for those participants that consent.

All specimens will be handled according to the procedures specified in the AP.

In the event of an abnormal laboratory value, participants may be asked to have an additional sample collected at the discretion of the Principal Investigator or designee.

7.8 Reimbursement

Participants will be reimbursed for their time, effort and for costs to cover their travel expenses to the study site and any inconvenience caused due to study participation. Site specific-reimbursement amounts will be documented in the site-specific Participant Information Sheet, and approved by the Institutional Review Board.

7.9 Randomization and Blinding

Participants will be identified by a unique study identification number.

Participants will be randomized according to the randomization schedule prepared by the statisticians at the Data Coordinating Center (DCC) prior to the start of the study. Participants will be automatically assigned a specific allocation number as they are enrolled into the data entry system. An unblinding list (Pharmacy List) will be provided to the unblinded site pharmacist by the DCC.

This is a randomized, double-blind placebo-controlled study for groups 1 and 2, and an open label study for group 3. For Groups 1 and 2, study staff (investigator and clinical personnel monitoring the safety and laboratory assay results) and participants will be blinded with respect to the allocation of Investigational Product (PGT121 mAb or placebo). A site pharmacist will be unblinded for the purposes of preparing study product.

A participant will be considered enrolled once he/she has been assigned an allocation number.

Blinded participants will be informed about their assignment (product/placebo) at study completion, once the database is locked. Should a study participant be unblinded during the study, the study participant will be followed up until the end of the study according to the Schedule of Procedures (Appendix A).

7.10 Un-blinding Procedure for Individual Participants

Un-blinding of an individual participant may be indicated in the event of a medical emergency if the clinical management of the participant would be altered by knowledge of the treatment assignment.

The un-blinded information should be restricted to a small group of individuals involved in clinical management/medical treatment of the participant (e.g. treating physician) and the blind must be maintained for those responsible for the study assessments.

The reasons for un-blinding should be documented and the IAVI Chief Medical Officer, the Medical Monitor and the DCC should be notified as soon as possible. The procedures and contact numbers for un-blinding are outlined in the SOM.

7.11 Assessment of IP related HIV sero-positivity

It is possible that PGT121 mAb or an immune response to PGT121 mAb could cause a positive result on a diagnostic HIV antibody test. A Group 1 participant who tests HIV antibody positive at the end of the study will have additional testing to distinguish actual HIV infection from IP-related responses. The participant will be informed of his/her positive HIV antibody test result and offered continuing follow-up until the HIV antibody test becomes negative.

8.0 INVESTIGATIONAL PRODUCT

8.1 Description

A summary of the Investigational Products is shown in Table 8.1-1.

Table 8.1-1 Investigational Products

**Product / Placebo**	**Dosage level**	**Total volume in IP container**	**Total IP or placebo volume to be injected into a 100 mL saline IV bag** (for an 88 kg body weight**)	**Total volume to be Infused** (for an 88 kg body weight**)
IP: PGT121 (50 mg/mL)	3 mg/kg	6 mL per vial	5.3 mL	105.3 mL
	10 mg/kg		17.6 mL	117.6 mL
	30 mg/kg		52.8 mL	152.8 mL
Placebo: 0.9% Sodium Chloride Injection USP (Saline)*	3 mg/kg matching***	NA	5.3 mL***	105.3 mL***
	10 mg/kg matching***		17.6 mL***	117.6 mL***
	30 mg/kg matching***		52.8 mL***	152.8 mL***

* The Placebo provided will be a commercially-available saline partial addition IV bag.

** The actual volume to be injected will be based on the dose group and the weight of the participant at the time of IP administration. The example included here is the average weight of an adult male in the US (88kg) (http://www.cdc.gov/nchs/data/series/sr_11/sr11_252.pdf)

*** For placebo IV infusions: saline from an additional IV bag will be injected into the saline IV bag intended for administration, to match the volume used for a PGT121 mAb injection in the same dose group, to prevent unblinding.

8.2 Shipment and Storage

Authorization to ship the Investigational Product to the site will be provided in writing by the Sponsor, upon confirmation that all required critical documents for shipment authorization are completed. The Investigational Product will be shipped maintaining the required storage conditions and stored in a secure location in the clinical site's pharmacy.

The Investigational Product is formulated in a 20 mM Acetate, 9% Sucrose, 0.008% polysorbate 80, pH 5.2 formulation buffer at a concentration of 50 mg/mL. Each 10 ml vial will contain 6 ml of IP stored at <- 20°C. Each vial will be labelled with the name of the product, Lot number, concentration, storage temperature, date of manufacturing, contact information of the Sponsor and a US cautionary statement. Several such vials will be packaged in a box. Each box will also be labelled with similar information as the vial label.

8.3 Preparation of Investigational Product (IP)

Detailed instruction will be provided to the site pharmacist in the SOM for preparing each of the investigational products. The site pharmacist will not be blinded, but the study physician/designee administering the IP will be blinded. Product should be administered within 6 hours of preparation. Example calculations for final volume for IV infusion are illustrated in Table 8.1-1. Procedures for handling used and partially used vials will be provided in the SOM.
Syringes or other components in direct contact with investigational products will be disposed of in a biohazard container and incinerated or autoclaved.

8.4 Administration of Investigational Product

Investigational Product will be administered at the enrollment visit.

The IP will be injected into a 0.9% Saline bag. The participant will receive the IP via IV infusion. Participants will receive infusion over approximately 60 minutes, allowing for clinician discretion. Further information on the IV infusion of the IP is supplied in the SOM and study documents.

8.5 Accountability and Disposal of Investigational Product

All used IP vials will be handled according to instructions in the SOM. The date, allocation number and location of storage of the returned vials will be recorded.

During the study, the IP accountability forms including receipt and dispensing of vials will be kept and monitored.

At the end of the study, the used and unused IP vials will be handled according to instructions of Sponsor.

Further information on accountability and disposal of IP is supplied in the SOM.

9.0 ASSESSMENTS

9.1 Safety Assessments

Data on local and systemic reactogenicity (i.e., solicited AEs) will be collected by structured interview and medical examination. Data on other adverse events will be collected with open-ended questions. All data will be recorded on the appropriate source documents and entered into the study database. Participants will be given a Memory Aid, which is a tool to assist with collecting reactogenicity data.

Local and systemic reactogenicity events will be assessed by study staff prior to IV infusion of IP, at approximately 30 minutes after IP administration start, at 1 hour after IP administration start, and subsequently every hour for the first 12 hours post-IP administration. Study staff will review the Memory Aid with the participant, and determine the severity of the reactions on days 1-3 through discussion with the participant.

9.1.1 Local reactogenicity

The presence of local reactogenicity will be assessed at the time points specified in the Schedule of Procedures (Appendix A).
Pain, tenderness, erythema/skin discoloration, swelling/hardening or pruritus will be assessed and graded using Appendix C, Adverse Event Severity Assessment Table, as a guideline.

9.1.2 Systemic reactogenicity

The presence of systemic reactogenicity will be assessed at the time points specified in the Schedule of Procedures (Appendix A).
Fever, chills, headache, nausea, vomiting, malaise, myalgia and arthralgia will be assessed and graded using the Appendix C, Adverse Event Severity Assessment Table as a guideline.

9.1.3 Vital Signs

At the administration of IP visit, vital signs (pulse, respiratory rate, blood pressure and temperature) will be measured by study staff prior to IP administration, at approximately 30 minutes post IP administration and hourly until 12 hours after IV infusion start. For the other study visits vital signs will be assessed at the time points specified in the Schedule of Procedures (Appendix A).

9.1.4 Other Adverse Events

Other adverse events (AEs) will be collected through 56 days after IP administration in all participants. Serious Adverse Events (SAEs) will be collected throughout the entire study period. Potential Immune Mediated Diseases (pIMDs), as defined in Section 10.5, will be collected throughout the study period, using the SAE reporting process. Open-ended questions will be asked at time points according to the Schedule of Procedures (Appendix A). All adverse events will be graded using Appendix C, Adverse Event Severity Assessment Table, as a guideline and will be assessed for causality to the IP. For more information regarding adverse events refer to Section 10.0, Adverse Events.

9.1.5 Concomitant Medications

Concomitant receipt of Investigational Products is prohibited during the study.

Contraceptive use and use of medication at study entry will be documented. (See DCF instructions)

During the study, information regarding concomitant medications and reasons for their use will be solicited from the study participants for 56 days. Ongoing concomitant medications will be recorded until end of study.

9.1.6 Routine laboratory parameters

Table 9.1.6-1 shows the laboratory parameters that will be measured routinely. The samples for these tests will be collected at the time points indicated in the Schedule of Procedures (Appendix A).

Table 9.1.6-1: Laboratory Parameters

Laboratory Parameter	Test
Hematology and Coagulation	Hemoglobin, hematocrit, leukocytes, platelets, absolute neutrophil count (ANC), absolute lymphocyte count (ALC), activate partial thromboplastin time (aPTT), international normalized ratio (INR)
Clinical Chemistry	Sodium, potassium, creatinine, aspartate aminotransferase (AST), alanine aminotransferase (ALT), total bilirubin, alkaline phosphatase<br>Groups 1 and 2 only: albumin, creatine kinase, C-reactive protein, C3 complement, C4 complement
Urinalysis	Dipstick test for protein, blood glucose, ketones, esterase (leukocytes) and nitrite. If clinically significant abnormalities (e.g., blood, protein, leukocytes) are found on dipstick test, then further test(s) will be performed (e.g., microscopy, culture)
T cell panel (Groups 2 and 3)	CD4 T cell count and frequency by single platform flow cytometry

9.1.7 Specific screening tests:

Participants will be screened to exclude the following diseases:

- Hepatitis B: positive for hepatitis B surface antigen (HBsAg)
- Hepatitis C: positive for hepatitis C RNA (HCV antibody test, followed by HCV RNA test if HCV antibody positive)
- Active syphilis: confirmed diagnosis (e.g.; positive RPR confirmed by TPHA)

A negative Hepatitis B and Hepatitis C result can be documented from the medical record only if the result is from a test administered less than 6 months ago.

Participants will also be screened to exclude the following laboratory parameters:

- Autologous PGT121-like antibody ELISA level above the cut-off;
- Resistance of autologous HIV to PGT121 neutralization *in vitro* (HIV viremic participants only, Group 3)

9.1.8 Monitoring for anti-PGT121 antibodies:

Participants will be evaluated for the development of antibodies to PGT121 mAb (anti-drug antibodies, ADA) by ELISA according to the Schedule of Procedures (Appendix A).

9.2 Virologic Assessments

Table 9.2-1 shows the virologic parameters that will be measured routinely. The samples for these tests will be collected at the time points indicated in the Schedule of Procedures (Appendix A).

Table 9.2-1: Virologic Assessment Table

Virologic Parameter	Test
Antiviral Activity	Plasma HIV RNA levels
Anti-reservoir activity	Cell-associated HIV-1 RNA levels in resting CD4 T cells; total HIV-1 DNA and 2-long terminal repeat (LTR) HIV-1 DNA circles in resting or total CD4 T cells; quantitative viral outgrowth assay (qVOA)
Other	Genotyping of plasma HIV RNA for evaluation of PGT121-induced escape mutations; phenotyping of plasma HIV RNA for neutralization susceptibility to PGT121 in-vitro

9.3 Exploratory Immunogenicity Assessments

Humoral immune response assays will include, but are not limited to Env-specific Ab-binding assays, virus neutralization assay, and assays for Ab functionality. Cellular immune response assays will include, but are not limited to IFNγ ELISPOT assay, ICS, and multiparameter flow cytometry. Exploratory assessments on mucosal samples will include, but are not limited to characterization of Env-specific binding Abs. Priority assays are listed below.

9.3.1 Antibody Responses

- Env-specific binding Abs (titers and breadth).
- Env-specific nAbs (titers and breadth).
- Env-specific functional Abs (phagocytosis score and breadth).
- Env-specific binding Ab isotypes (IgA, IgG1-4) (titers and breadth).

9.3.2 Cellular Responses

- IFNγ peripheral blood mononuclear cell (PBMC) responders to peptide pools and subpools of Potential T-cell epitopes, PTE Env/Gag/Pol peptides.
- $CD4^+$ and $CD8^+$ T-cell functionality (% cells producing e.g. IFNγ, IL-2, IL-4, TNFα).
- T-cell development with emphasis on follicular helper T-cells and memory differentiation.

9.3.3 PBMC, Serum and Plasma Storage

Samples of cryopreserved PBMC, plasma and serum will be stored as indicated in the Analytical Plan (AP) and, if the participant consents, may be used for the purposes of standardization, quality control and for future assays related to HIV prevention or treatment research and development. These samples will be archived and the testing laboratories will be blinded to the participant's identity.

9.4 Other Assessments

9.4.1 HIV Antibody Testing

All HIV-uninfected participants (Group 1) will be tested for HIV antibodies as indicated in the Schedule of Procedures (Appendix A) or as needed, if medical or social circumstances arise. All participants will receive HIV risk reduction counselling and pre- and post-HIV-test counselling, as specified in Section 7.3 Counselling.

9.4.2 Pharmacokinetics

Blood draws for pharmacokinetics will be done on the day of IP administration immediately before starting IV infusion of IP, at the end of the IP administration, and 30 minutes and 3 hours after the end of the IP administration. Additional draws will be done at 6, 9, 12 and 24 hours after the start of the IP administration. Thereafter, pharmacokinetic draws will be done as indicated in the Schedule of Procedures (Appendix A). PGT121 mAb serum or plasma levels will be determined using two methods: a sandwich ELISA using a murine anti-idiotype antibody to PGT121 mAb, and a neutralization assay.

PGT121 mAb pharmacokinetic analysis will be performed using standard non-compartmental analysis methods to estimate elimination half-life (t1/2), clearance (CL/F), volume of distribution (Vz/F), Area under the concentration decay curve (AUC), impact of viral load and/or ART on PGT121 mAb disposition (elimination half-life (t1/2), clearance (CL/F), volume of distribution (Vz/F) and total exposure. PGT 121 accumulation will also be examined in rectal and cervical mucosal secretions collected with rectal sponges or cervical Softcups in study participants who specifically consented for these procedures. Descriptive results will be reported for the pharmacokinetic parameters by dose subgroup.

Exploratory analysis using population analysis methods simultaneously combining all pharmacokinetic data across all doses and treatment groups will be performed for quantitative characterization of differences in PGT121 mAb disposition by dose, participant group or disease state.

9.4.3 HLA Typing

Samples for HLA typing will be collected as specified in the AP and may be analyzed as warranted.

9.4.5 Pregnancy Test

A urine pregnancy test for all female participants will be performed by measurement of human chorionic gonadotrophin (βhCG) at time points indicated in the Schedule of Procedures (Appendix A). The results of the pregnancy test must be negative prior to IV infusion of PGT121 mAb. See section 10.7 for description of pregnancy after administration of IP.

9.4.6 HIV Risk Assessment (Group 1)

Study staff will assess participants for their past and current risk of acquiring HIV at time points indicated in Schedule of Procedures (Appendix A).

9.4.7 Social Impact Assessment

A brief assessment of the impact of participation in the study will be administered to participants at their final study visit.

10.0 ADVERSE EVENTS

10.1 Definition

An adverse event (AE) is any untoward medical occurrence in a participant administered an Investigational Product and which does not necessarily have a causal relationship with the Investigational Product. An AE can therefore be any unfavourable or unintended sign (including an abnormal laboratory finding), symptom, or disease, temporally associated with the use of Investigational Product whether or not related to the Investigational Product.

Assessment of severity of all AEs, including and seriousness of AEs, is ultimately the responsibility of the Principal Investigator of each site. Refer to the DIVISION OF AIDS (DAIDS) TABLE FOR GRADING THE SEVERITY OF ADULT AND PEDIATRIC ADVERSE EVENTS Version 2.0, November 2014 for additional guidance.

10.2 Assessment of Severity of Adverse Events

The following general criteria should be used in assessing adverse events as mild, moderate, severe or very severe at the time of evaluation:

Grade 1 (Mild): Symptoms causing no or minimal interference with usual social & functional activities

Grade 2 (Moderate): Symptoms causing greater than minimal interference with usual social & functional activities

Grade 3 (Severe): Symptoms causing inability to perform usual social & functional activities

Grade 4 (Very Severe): Symptoms causing inability to perform basic self-care functions OR Medical or operative intervention indicated to prevent permanent impairment, persistent disability, or death

Guidelines for assessing the severity of specific adverse events and laboratory abnormalities are listed in Appendix C, Adverse Event Severity Assessment Table.

10.3 Relationship to Investigational Product

Assessment of relationship of an AE or SAE to Investigational Product (IP) is the responsibility of the Principal Investigator or designee. All medically indicated and available diagnostic methods (e.g., laboratory, blood smear, culture, X-ray, etc.) should be used to assess the nature and cause of the AE/SAE. Best clinical and scientific judgment should be used to assess relationship of AE/SAEs to the IP and/or other cause.

The following should be considered:

- Presence/absence of a clear temporal (time) sequence between administration of the IP and the onset of AE/SAE
- Presence/absence of another cause that could more likely explain the AE/SAE (concurrent disease, concomitant medication, environmental or toxic factors)
- Whether or not the AE/SAE follows a known response pattern associated with the IP

The relationship assessment should be reported as one of the following:

Not Related: clearly explained by another cause (concurrent disease, concomitant medication, environmental or toxic factors, etc.).

Unlikely: more likely explained by another cause (concurrent disease, concomitant medication, environmental or toxic factors, etc.).

Possibly: equally likely explained by another cause but the possibility of the IP relationship cannot be ruled out (e.g., reasonably well temporally related and/or follows a known IP response pattern but equally well explained by another cause).

Probably: more likely explained by the IP (e.g., reasonably well temporally related and/or follows a known IP response pattern and less likely explained by another cause).

Definitely: clearly related and most likely explained by the IP.

For the purpose of expedited safety reporting, all possibly, probably or definitely related SAEs are considered IP-related SAEs.

10.4 Serious Adverse Events

An adverse event is reported as a "Serious Adverse Event" if it meets any of the following criteria (as per International Conference on Harmonisation [ICH] Good Clinical Practice [GCP] Guidelines):

- Results in death
- Is life threatening
- Results in persistent or significant disability/incapacity
- Requires in-participant hospitalization or prolongs existing hospitalization
- Is a congenital anomaly/birth defect or spontaneous abortion
- Any other important medical condition that requires medical or surgical intervention to prevent permanent impairment of a body function or structure

Elective surgery for pre-existing condition that did not increase in severity or frequency is not considered an SAE.

Serious Adverse Events (SAEs) should be reported within 24 hours of the site becoming aware of the event, and sent to the Sponsor as described in the SOM.

To discuss IP-related SAEs or any urgent medical questions related to the SAE, the site investigator should contact one of the IAVI Medical Monitors directly (see Contact List in the SOM).

The IAVI SAE Report Form should be completed with all the available information at the time of reporting and sent to the Sponsor as described in the SOM. The minimum data required in reporting an SAE are the study identification number, date of birth, gender, event description (in as much detail as is known at the time), onset date of event (if available), reason event is classified as serious, reporting source (name of Principal Investigator or designee), and relationship to the IP as assessed by the investigator.

The Principal Investigator or designee is required to prepare a detailed written report with follow up until resolution or until it is judged by the Principal Investigator or designee to have stabilized.

The Principal Investigator or designee must notify the local IRB/IEC of all SAEs as appropriate. In case of IP-related SAEs, the Sponsor will notify responsible regulatory authorities, Safety Monitoring Committee (SMC), and other study sites where the same IP is being tested.

More details on SAE definitions and reporting requirements are provided in the SOM.

Serious Event Prior to Investigational Product Administration

If a serious event occurs in the period between the participant signing the Informed Consent Form and receiving the IV infusion of IP, the event will be reported using the SAE form and following the same procedures for SAE reporting, as indicated in Section 10.4. The timing of the event will be indicated by using the relevant checkbox on the SAE form.

10.5 Reporting Potential Immune-Mediated Diseases

Potential immune-mediated diseases (pIMDs) are a subset of AEs that include both clearly autoimmune diseases and also other inflammatory and/or neurologic disorders that may or may not have an autoimmune etiology. These events are of special interest since they could potentially be caused by immune responses to the IP. The investigator/designee should report such adverse events within the same time limits (following confirmation of an AE as a pIMD; see last paragraph of this section below), and using the same CRF pages, as utilized for SAEs. The investigator or his/her designee will evaluate the occurrence of pIMDs at every visit/contact during the study. IAVI will also expect investigators/designee to provide additional information about pIMD events. AEs to be reported and documented as pIMDs include:

Neuroinflammatory disorders: optic neuritis, cranial nerve disorders (including Bell's palsy), multiple sclerosis, demyelinating disease, transverse myelitis, Guillain-Barré syndrome, myasthenia gravis, encephalitis, neuritis.

Musculoskeletal disorders: systemic lupus erythematosus, cutaneous lupus, Sjögren's syndrome, scleroderma, dermatomyositis, polymyositis, myopathy, rheumatoid arthritis and juvenile rheumatoid arthritis, polymyalgia rheumatica or temporal arteritis, reactive arthritis, psoriatic arthropathy, ankylosing spondylitis, undifferentiated spondyloarthropathy.

Gastrointestinal disorders: Crohn's disease, ulcerative colitis or proctitis, celiac disease.

Metabolic diseases: autoimmune thyroiditis, Grave's or Basedow's disease, Hashimoto thyroiditis, insulin-dependent diabetes mellitus (IDDM), Addison's disease.

Skin disorders: psoriasis, vitiligo, Raynaud's phenomenon, erythema nodosum, autoimmune bullous skin diseases.

Others: autoimmune hemolytic anemia, thrombocytopenia, antiphospholipid syndrome, *vasculitis, pernicious anemia, autoimmune hepatitis, primary biliary cirrhosis, primary sclerosing cholangitis, autoimmune glomerulonephritis, autoimmune uveitis, autoimmune myocarditis/cardiomyopathy, sarcoidosis, Stevens-Johnson syndrome, Behçet's syndrome.

Infusion site reactions: Grade 3 or 4 infusion site reactions lasting more than 2 days.

*Vasculitis: Vasculitis, Diffuse vasculitis, leucocytoclastic vasculitis, polyarteritis nodosa, microscopic polyangiitis, Wegener's granulomatosis, anti-neutrophil cytoplasmic antibody positive vasculitis, Henoch-schönlein purpura, allergic granulomatous angiitis (Churg-Strauss disease), Kawasaki disease, Takayasu's arteritis, temporal arteritis (giant cell arteritis), renal vasculitis.

Medical judgement should be exercised in deciding whether other disorders/diseases have an autoimmune origin and should also be reported as described above, and this judgement is the investigator's prerogative. Whenever sufficient data exist to substantiate any of the diagnoses in the above list, the event must be reported as a pIMD. While the intent of pIMD reporting is to be inclusive, isolated nonspecific symptoms, which might (or might not) represent the above diagnoses, should be captured as AEs but not reported as pIMDs until the diagnosis can be defended.

10.6 Clinical Management of Adverse Events

Adverse events (AEs) will be managed by the clinical study team who will assess, provide first line of care as appropriate and refer to health care and treatment facilities as warranted. If any treatment/medical care is required as a result of the harm caused by the IP or study procedures, this will be provided free of charge.

If a participant has an AE and/or abnormal laboratory value that is known at the time of IV infusion of IP, the specifications of Section 12.0 will be followed.

Participants will be followed until the AE resolves or stabilizes or up to the end of the study, whichever comes last. If at the end of the study, an AE (including clinically significant laboratory abnormality) that is considered possibly, probably or definitely related to the IP is unresolved, follow-up will continue until resolution if possible and/or the participant will be referred.

10.7 Pregnancy

Although not considered an AE, if a female participant becomes pregnant during the study, it is the responsibility of the Principal Investigator or designee to report the pregnancy promptly to IAVI using the designated forms. The participant will be followed for safety until the end of pregnancy or study completion, whichever occurs last. If

possible, approximately 2–4 weeks after delivery, the baby will be examined by a physician to assess its health status and the results will be reported to the Sponsor. The baby will be examined again by a Physician around age 1, and the results will be reported to the Sponsor.

Complications of pregnancy that meet criteria for SAEs, specified in Section 10.4 of this Protocol (e.g., hospitalization for eclampsia, spontaneous abortion, etc.) should be reported as SAEs.

10.8 Intercurrent HIV Infection (Group 1)

HIV infection cannot be directly caused by the IP. If a participant acquires HIV through exposure in the community, at any time after the IV infusion of IP, the participant should be offered referral to appropriate care and treatment facilities. The participant will continue to be followed in the study for safety assessments.

Intercurrent HIV infection in study participants, although not considered an SAE, must be reported promptly to IAVI using the designated forms. However, medical conditions associated with the HIV infection that meet criteria for being serious specified in the Section 10.4 of this Protocol (e.g., sepsis, *Pneumocystis jiroveci [carinii] pneumonia*, etc.) should be reported as SAEs using the SAE Report Form.

11.0 MANAGEMENT OF HIV ISSUES DURING AND FOLLOWING STUDY

11.1 HIV Testing – Group 1

Group 1 participants will be tested for HIV antibodies as indicated in the Schedule of Procedures (Appendix A) or as needed, if medical or social circumstances arise. All participants will receive HIV risk reduction counselling and pre- and post-HIV-test counselling, as specified in Section 11.2.1, Counselling (Group 1).

It is possible that PGT121 or an immune response to PGT121 mAb could cause a positive result on a diagnostic HIV antibody test. An IP recipient who falsely tests HIV positive with a diagnostic HIV antibody test at the end of the study will be informed of his/her positive test result and offered continuing follow-up until the test becomes negative.

If a participant acquires HIV through exposure in the community, at any time after the administration of IP, the participant will be offered referral to appropriate care and treatment facilities. The participant will continue to be followed in the study for safety assessments.

Should a participant require HIV testing outside of the study for personal reasons, it is recommended that the participant contact the study staff first. HIV testing can be done at the study site and then processed at an independent laboratory as above. Written evidence of HIV status (HIV-infected or HIV-uninfected) will be provided upon request.

11.2 Social Discrimination as a Result of IP-related antibodies

In order to minimize the possibility of social discrimination in participants (if any) who test positive on a diagnostic HIV antibody test due to IP-related antibodies, appropriate diagnostic HIV testing and certification will be provided both during and after the study as needed.

11.3 HIV infection – Group 1

Group 1 participants who are diagnosed with HIV infection at screening or during the study (intercurrent HIV-infection) will be provided the following:

11.3.1 Counselling

The participant will be counselled by the study investigators or designated counsellors. The counselling process will assist the participant with the following issues:

- Psychological and social implications of HIV infection
- Who to inform and what to say
- Implications for sexual partners
- Implications for child-bearing
- Avoidance of transmission to others in future
- Mandatory reporting to the state, in some instances

11.3.2 Referral for Support/Care

Participants will be referred to a participant support center or institution of his/her choice for a full discussion of the clinical aspects of HIV infection. Referral will be made to a designated physician or center

12.0 WITHDRAWAL FROM STUDY

12.1 Deferral of IV infusion of IP

An IV infusion of IP may be temporarily deferred if the participant is clinically ill at the time of the administration of IP visit and/or presents with fever ($\geq$ 100.4 F) at the time of the administration of IP visit. A participant must be clinically well and afebrile for a minimum of a 24-hour consecutive period prior to administration of IP.

Any planned or unplanned deferral of infusion of IP will be discussed with the Sponsor. Participants will be deferred from infusion of IP for any of the following reasons:

1. Pregnancy
2. A disease or condition or adverse event that may develop, regardless of relationship to Investigational Product, if the Principal Investigator or designee is of the opinion that administration of IP will jeopardize the safety of the participant
3. Participant’s request to defer infusion

The following events require resolution and/or review of clinical history by the Principal Investigator or designee and consultation with the Medical Monitor, prior to administration of IP:

- Any abnormal laboratory value, as outlined in section 5.7, Exclusion Criteria, Hematology, Chemistry, Urinalysis that is known at the time of infusion and have not resolved. Abnormal results should be confirmed on the original sample and/or repeated at least once to confirm abnormal values.
- Receipt of inactivated/killed/subunit vaccines (non-HIV) or immunoglobulin within the previous 14 days. Receipt of live attenuated vaccines within the previous 60 days.
- Participating in another clinical study of an Investigational Product

12.2 Withdrawal from the Study (Early Termination)

Participants may be withdrawn from the study permanently for the following reasons:

1. Participants may withdraw from the study at any time if they wish, for any reason
2. The Principal Investigator or designee has reason to believe that the participant is not complying with the protocol
3. If the Sponsor decides to terminate or suspend the study

If a participant withdraws or is withdrawn from the study, all termination visit procedures will be performed according to the Schedule of Procedures (Appendix A) where possible. Every effort will be made to determine and document the reason for withdrawal.

13.0 DATA HANDLING

13.1 Data Collection and Record Keeping at the Study Site

Data Collection: All study data will be collected by the clinical study staff using designated source documents and entered onto the appropriate electronic CRFs (eCRFs). Access to eCRFs will be provided via an electronic data entry system hosted by the Data Coordination Center. All study data must be verifiable to the source documentation. A file will be held for each participant at the clinic(s) containing all the source documents. Source documentation will be available for review to ensure that the collected data are consistent with the CRFs.

All CRFs and laboratory reports will be reviewed by the clinical team, who will ensure that they are accurate and complete.

Source documents and other supporting documents will be kept in a secure location. Standard GCP practices will be followed to ensure accurate, reliable and consistent data collection.

Source documents include but are not limited to:

- Signed Informed Consent Documents
- Progress notes
- Data collection forms
- Documentation of any existing conditions or past conditions relevant to eligibility
- Printed laboratory results
- Print out of the IDES generated enrollment confirmation
- All Adverse Events
- Concomitant medications
- Local and systemic reactogenicity events

13.3 Data Entry at the Study Site

The data collected at the site will be recorded onto the eCRFs by the study staff and entered into a database. To provide for real time assessment of safety, data should be entered as soon as reasonably feasible after a visit occurs.

13.4 Data Analysis

The Sponsor, PIs and Product Developers will agree on how data will be analyzed and presented prior to unblinding of the study.

The DCC will conduct the data analysis and will provide interim safety and final study reports for the Sponsor, Principal Investigators, the PSRT and SMC and the regulatory authorities, as appropriate.

14.0 STATISTICAL CONSIDERATIONS

14.1 Safety and Tolerability Analysis

14.1.1 Sample Size

The sample size for safety and tolerability analysis will be 30-48 participants according to the dose escalation design used to characterize the safety profile of one IV infusion of PGT121 mAb, at one of three dose levels, to HIV-uninfected and HIV-infected individuals (groups 1 and 2).

14.1.2 Null Hypothesis

As this is an exploratory proof of concept trial and analysis will be descriptive, no formal null hypothesis will be tested.

14.1.3 Statistical Power and Analysis and Dose Escalation Rules

The frequency of moderate or greater local and systemic reactogenicity events will be determined and compared between groups.

The frequency of SAEs judged possibly, probably or related to the IP will be determined.

All AEs will be analyzed and, grouped by seriousness, severity and relationship to the Investigational Product (as judged by the investigator).

For life-threatening adverse events related to Investigational Product: if none of the 12 (max 18) participants receiving Investigational Products experience such reactions, then the 95 % upper confidence bound for the rate of these adverse events in the population is 26.5% (or 18.5% if n=18).

All AEs will be analysed and grouped by seriousness, severity and relationship to the IP (as judged by the investigator).

For life-threatening adverse events related to IP: if none of the 12 (max 18) participants in either Group 1 or Group 2 who receive the IP experience such reactions then the 95% upper confidence bound for the rate of these adverse events in the population is 26.5% (or 18.5% if n=18).

An interim analysis of group data will be carried out according to the study schema (Table 5.3.1) without unblinding the study to investigators or participants. At the end of the study, a full analysis will be prepared.

Based on previous experience with IAVI Phase 1 IP studies, it is expected that the amount of missing, unused or spurious data will be insignificant. Unused and spurious data will be listed separately and excluded from the statistical analysis. Missing data will be excluded from the statistical analysis.

14.2 Pharmacokinetic Analysis

14.2.1 Sample Size

The sample size for pharmacokinetic analysis will be 4 per dose subgroup, sufficient to provide sufficient information for the planned analyses.

14.2.2 Null Hypothesis

As this is an exploratory proof of concept trial and analysis will be descriptive, no formal null hypothesis will be tested.

14.2.3 Statistical Power and Analysis

Disposition of PGT121 mAb will be evaluated in this study. Based on the PK profile of other human monoclonal antibodies, it is expected that the half-life of PGT121 mAb will be 14 to 21 days. Previously published data indicates that the pharmacokinetics of PGT121 and 3BNC117 are fairly similar across a non-human primate cohort and within the same non-human primate (clearance of 3BNC117 appears to be marginally faster than that for PGT121).

Commonly reported PK parameters will be calculated using standard non-compartmental slope/height/area/moment (SHAM) analysis methods. Summary descriptive results of PK parameters, including AUC, Cmax, T1/2, and clearance results will be reported by dose cohort. Dose normalized plots of PK parameters will be presented. Correlation between PK and reported safety and pharmacodynamic outcomes will also be explored parameters in order to examine exposure-effect relationships.

A more powerful exploratory analysis to quantitatively determine the dose, participant and disease impact on PGT121 mAb pharmacokinetics, and correlate exposure with response, while correctly accounting for variance based on population intrinsic factors such as weight and gender will be performed. Using the proposed population analysis approach we will be able to simultaneously examine the magnitude and the rate of change to PGT121 disposition driven by HIV-1 RNA levels and/or ART, and also

examine the magnitude and the rate of decline in log copies/ml of HIV-1 RNA plasma levels from baseline.

The frequency and levels of anti-PGT121 antibodies will be calculated and tabulated.

14.3 Virologic Analysis for Dose De-escalation in Groups 3A-C

14.3.1 Sample Size

The sample size for virologic analysis in Groups 3A-C will be 24-36 participants according to the dose de-escalation design described below.

14.3.2 Null Hypothesis

The null hypothesis is that there is no difference in antiviral activity between PGT121 mAb and placebo.

14.3.3 Statistical Power and Analysis

The virologic analysis described in this section relates to Groups 3A-C of the study design, in which dose de-escalation is performed in an adaptive study design in HIV-infected participants off ART with plasma HIV RNA levels of 2 x 10^3 – 10^5 copies/ml. This section assumes that Part 1 of the study has successfully demonstrated that there is a safe dose level of the IP such that the study is carried forward into Part 2.

The primary efficacy outcome for this analysis is defined as change in log10 viral load between Day 0 (day of infusion) and Day 7. The minimum clinically significant value for this outcome is defined as a difference of -0.9 log10.

The study plan for Groups 3A-C is designed so that the IP dose level may be de-escalated in a stepwise manner from the highest dose to the lowest dose, until a given dose level cannot be concluded to be efficacious. If any given dose level is proven to be efficacious at an interim analysis, enrolment for that dose level may cease, and the next lowest dose group may be enrolled. In the unlikely event that IP administration leads to increased viral load, this may be detected by this design. No placebo participants are enrolled as part of this design.

This design represents a dose de-escalation beginning at 30 mg/kg. The actual starting dose will be the MTD as determined by the SMC based on data from Part 1, therefore the starting dose may be 30mg/kg, 10 mg/kg or 3 mg/kg. If the starting dose is 30 mg/kg, then de-escalation will begin with Group 3A. If the starting dose is 10 mg/kg, then de-escalation will begin with Group 3B. If the starting dose is 3 mg/kg, then only Group 3C will be enrolled.

Assuming the starting dose is 30 mg/kg, an interim analysis of Group 3A will be performed after all 6 participants have reached 7 days following IP administration. The following scenarios may then occur, depending on the data:

- If the mean response in the first 6 participants is a decrease greater than or equal to -0.9 log10 HIV RNA, the IP will be determined to be effective at 30

mg/kg, enrollment into Group 3A will cease, and enrollment into Group 3B will begin.
- If the mean response in the first 6 participants is a decrease smaller than -0.9 log10 HIV RNA, then an additional 3 participants will be enrolled into Group 3A. After the additional 3 participants have reached 7 days following IP administration, an analysis of Group 3A (N=9) will be performed:
    - If the mean response in all Group 3A participants is a decrease greater than or equal to -0.9 log10 HIV RNA, the IP will be determined to be effective at 30 mg/kg, and enrollment into Group 3B will begin.
    - If the mean response in all 9 participants is a decrease smaller than -0.9 log10 HIV RNA, then the IP will be determined to be ineffective at 30 mg/kg and Groups 3B and 3C will not be enrolled. In this scenario, no dose of IP will be determined to be effective.

If 30 mg/kg is determined to be an effective dose, then Group 3B will be enrolled at 10 mg/kg. An interim analysis of Group 3B will be performed after 8 participants have reached 7 days following IP administration. The following scenarios may then occur, depending on the data:
- If the mean response in the first 8 participants is a decrease greater than or equal to -0.9 log10 HIV RNA, the IP will be determined to be effective at 10 mg/kg, enrollment into Group 3B will cease, and enrollment into Group 3C will begin.
- If the mean response in the first 8 participants is a decrease smaller than -0.9 log10 HIV RNA, then an additional 4 participants will be enrolled into Group 3B. After the additional 4 participants have reached 7 days following IP administration, an analysis of Group 3B (N=12) will be performed:
    - If the mean response in all Group 3B participants is a decrease greater than or equal to -0.9 log10 HIV RNA, the IP will be determined to be effective at 10 mg/kg, and enrollment into Group 3C will begin.
    - If the mean response in all 12 participants is a decrease smaller than -0.9 log10 HIV RNA, then the IP will be determined to be ineffective at 10 mg/kg, and Group 3C will not be enrolled. In this scenario, the minimum effective dose will be determined to be 30 mg/kg.

If 10 mg/kg is determined to be an effective dose, then Group 3C will be enrolled at 3 mg/kg. An interim analysis of Group 3C will be performed after 10 participants have reached 7 days following IP administration. The following scenarios may then occur, depending on the data:
- If the mean response in the first 10 participants is a decrease greater than or equal to -0.9 log10 HIV RNA, the IP will be determined to be effective at 3 mg/kg and enrollment into Group 3C will cease. In this scenario, the minimum effective dose of the IP will be determined to be 3 mg/kg.
- If the mean response in the first 10 participants is a decrease smaller than -0.9 log10 HIV RNA, then an additional 5 participants will be enrolled into Group 3C. After the additional 5 participants have reached 7 days following IP administration, an analysis of Group 3C (N=15) will be performed:
    - If the mean response in all Group 3C participants is a decrease greater than or equal to -0.9 log10 HIV RNA, the minimum effective dose will be determined to be 3 mg/kg.

- If the mean response in all 15 participants is a decrease smaller than -0.9 log10 HIV RNA, then the IP will be determined to be ineffective at 3 mg/kg. In this scenario, the minimum effective dose will be determined to be 10 mg/kg.

For the analysis of sample size and power, log10 viral load differences from baseline for each participant were simulated from a normal distribution, with a standard deviation of 0.5. This value was chosen by examining a study of the antiretroviral drug raltegravir, which demonstrated a mean estimated standard deviation of the change of baseline of 0.47[18]. This is a conservative estimate, as the variability of viral loads near the lower range might be expected to also be lower.

The statistical test performed will be the Signed-ranktest, which will incorporate the "shift" parameter of -0.9 log10 (the minimum clinically significant difference selected for this study). An evaluation of potential harm (increased viral load) will also be performed with the Signed ranktest; this test will examine the null hypothesis of no change in viral load (a shift of 0.0 log10 following IP administration) against the one-sided alternative hypothesis that the viral load is increased following IP administration. Each efficacy test will be performed at the level $\alpha = 0.05$. Each test for harm will be performed at level $2\alpha = 0.10$, in order to provide additional sensitivity to detect potential harm.

14.4 Analysis of Antiviral Activity and Dose De-escalation in Subgroups 3D-F

14.4.1 Sample Size

The sample size for antiviral activity will be 3-9 participants, depending on the MTD.

14.4.2 Null Hypothesis

As this is an exploratory proof of concept trial and analysis will be descriptive in this population, no formal null hypothesis will be tested.

14.4.3 Statistical Power and Analysis

No efficacy endpoints will be tested in Groups 3D-F as participants are HIV-infected with low viral loads at baseline (10^2 – $2x10^3$ copies/ml). Immunologic and virologic endpoints will be determined as described in Section 4.1. As soon as subgroup 3D has enrolled 3 participants, enrollment into subgroup 3E will be initiated. The same rule will apply for progression from subgroup 3E to 3F. In the event that dose de-escalation in subgroups 3A-C is stopped due to failure to meet virologic endpoints, dose de-escalation in subgroups 3D-F will continue.

14.5 Secondary and Exploratory Immunologic and Virologic Analyses

14.5.1 Sample Size

The sample size for secondary and exploratory immunologic and virologic analysis will be 63-93 participants.

14.5.2 Null Hypothesis

No formal hypothesis on immunologic or virologic responses will be tested, with the exception of the change in viral load described in Section 14.3.

14.5.3 Statistical Power and Analysis

Descriptive statistics (actual values and changes from reference) will be calculated for continuous immunologic and virologic parameters at all time points. Graphical representations of changes in parameters will be made as applicable. Differences between groups at a specific time point will be tested for exploratory purposes by a 2-sample t-test if the data appear to be normally distributed. If not, the non-parametric Wilcoxon rank sum test will be used. If portions of the measurements are censored below the assay quantification limit, the Gehan-Wilcoxon test will be employed. All statistical tests will be two-sided and will be considered statistically significant if $p < 0.05$.

Frequency tabulations will be calculated for discrete (qualitative) immunologic and virologic parameters at all time points. Significant differences between groups will be determined by a 2-sided Fisher's exact test.

Interim immunologic and virologic analyses of grouped data may be performed without unblinding the study to investigators or participants.

15.0 QUALITY CONTROL AND QUALITY ASSURANCE

To ensure the quality and reliability of the data collected and generated and the ethical conduct of this study, a Study Operations Manual (SOM) will be developed. All deviations will be reported and investigated. The SOM describes reporting and deviation documentation requirements and procedures.

Regular monitoring will be performed according to ICH-GCP as indicated in Section 17.5.

An independent audit of the study and study sites may be performed by the Sponsor or designee to establish the status of applicable quality systems. Inspection by regulatory authorities may also occur.

By signing the protocol, the Principal Investigators agree to facilitate study related monitoring, audits, IRB/IEC review and regulatory inspection(s) and direct access to source documents. Such information will be treated as strictly confidential and under no circumstances be made publicly available.

16.0 DATA AND BIOLOGICAL MATERIAL

All data and biological material collected through the study shall be managed in accordance with the Clinical Trial Agreement (CTA). Distribution and use of these data will be conducted by agreement of all parties.

The computerized raw data generated will be held by the DCC on behalf of the Sponsor. The study sites will also hold the final data files and tables generated for the purpose of analysis.

17.0 ADMINISTRATIVE STRUCTURE

The Principal Investigator will be responsible for all aspects of the study at the study site.

17.1 Protocol Safety Review Team

A PSRT will be formed to monitor the clinical safety data. During the administration of IP phase of the trial, the PSRT will review the clinical safety data on a weekly basis via electronic distribution of reports. An ad hoc PSRT review meeting will occur if any of the members of the PSRT requests a special review to discuss a specific safety issue or as specified in the Study Operations Manual. After the administration of IP phase the PSRT will review the clinical safety data at least monthly.

The PSRT will consist of the IAVI Medical Monitor(s), and the PI or designee from each clinical team. The study chair or an IAVI Medical Monitor may be the PSRT chair. *Ex officio* members will include the IAVI Chief Medical Officer and an unblinded IAVI Medical Monitor.

Additional PSRT participants may include the following, as needed:

- Co-investigators and trial site senior clinical research nursing staff
- Laboratory directors
- Data management, study statistician and regulatory staff

The PSRT membership and procedures are detailed in the PSRT charter.

17.2 Safety Monitoring Committee (SMC)

The SMC will consist of independent clinicians/scientists/statisticians/ethicists who are not involved in the study. Investigators responsible for the clinical care of participants or representative of the Sponsor may not be a member of the SMC. Details of membership, chair and co-chair and responsibilities are outlined in the SMC charter.

Principal Investigator(s) or designee and/or a Sponsor representative may be asked to join an open session of the SMC meeting to provide information on study conduct, present data or to respond to questions.

Safety data will be reviewed by the SMC at pre-specified time points and at an ad-hoc basis.

17.2.1 Content of Interim Safety Review

The SMC will be asked to review the following blinded data:

- Summary of reactogenicity (i.e., solicited adverse events)
- All adverse events judged by the Principal Investigator or designee to be possibly, probably or definitely related to IP
- All laboratory results confirmed on retest and judged by the Principal Investigator or designee to be clinically significant
- All SAEs

An unblinded presentation of all above noted events may also be made available for the SMC for their review if required by any member of the SMC.

17.2.2 SMC Review of Group 1 and 2 data prior to starting Group 3

Following IV infusion of IP of the last participant in Groups 1 and 2, the Safety Monitoring Committee (SMC) will review safety data through the day 14 post-IV infusion visit for all participants to confirm MTD in each group, and determine whether, and at what dose level, Group 3 can initiate enrollment.

17.3 Criteria for Pausing the Study

Enrollment and administration of IP will be stopped and a safety review conducted by the SMC for any of the following criteria:

1. One or more participants experience an SAE that is judged possibly, probably or definitely related to IP.
2. There is a participant death assessed as possibly, probably or definitely related to the IP.
3. Two or more participants experience Grade 3 adverse events in the same category System Organ Class that are considered to be at least possibly related to IP or
4. Any grade 4 adverse event that is considered to be at least possibly related to IP.

Table 2: AE notification and safety pause/AE review rules

Event and relationship to study product	Severity	Occurrence	Site PI action	PSRT or SMC action
SAE, related[1]	Any	Any	Phone, email or fax forms to sponsor within 24 hours	Study pause within 24 hours, refer to SMC for review
SAE, not related[2]	Grade 5	Any	Phone, email or fax forms to sponsor within 24 hours	PSRT review within 2 business days to consider pause
AE[3], related	Grade 3 or 4[4]	Second[5]	Phone, email or fax notification to sponsor within 24 hours	Study pause within 24 hours, refer to SMC for review
AE[3], related	Grade 3 or 4[4]	First	Phone, email or fax notification to sponsor within 24 hours	PSRT review within 2 business days to consider pause

[1] Related SAE refers to SAE deemed to be definitely, probably, or possibly related to study vaccine.

[2] Not related SAE refers to SAE deemed to be probably not related or not related to the study vaccine.

[3] Does not include the following reactogenicity symptoms (fever, fatigue/malaise, myalgia, arthralgia, chills, headache, nausea, vomiting).

[4] If no evidence of disease is present other than an abnormal laboratory value, the test must be repeated (entailing blood re-draw) at least one time. The verification period will be a maximum of 72 hours after initial awareness of the abnormal laboratory value. When signs and symptoms are present, repeat test will not be needed.

[5] PSRT will determine whether the reported related AE (Grade 3 or 4) is a second occurrence of a previously reported AE (Grade 3 or 4).

The Sponsor will request a review by the SMC, (or the SMC chair if other SMC members cannot be convened), to be held within 2 business days of the Sponsor learning of the event. The individual participant(s)/or study may be unblinded at the discretion of the SMC.

Following this review, the SMC will make a recommendation regarding the continuation or suspension of the administration of the IP or the trial and communicate this decision immediately to the Sponsor. The Sponsor then will inform the Principal Investigators without delay.

Additional *ad hoc* review may be specifically requested by the Sponsor, the Principal Investigator(s) or by the SMC.

17.4 Study Supervision

The SMC, the IAVI Chief Medical Officer (CMO) and the IAVI Medical Monitor(s) have access to progress report(s) of this study. Close cooperation will be necessary to track study progress, respond to queries about proper study implementation and management, address issues in a timely manner, and assure consistent documentation, and share information effectively. Rates of accrual, retention, and other parameters relevant to the site's performance will be regularly and closely monitored by the study team.

17.5 Study Monitoring

On-and/or off-site monitoring will ensure that the study is conducted in compliance with human subjects' protection and other research regulations and guidelines, recorded and reported in accordance with the protocol, is consistent with SOPs, GCP, applicable regulatory requirements and locally accepted practices. The monitor will confirm the quality and accuracy of data at the site by validation of CRFs against the source documents, such as clinical records. The investigators, as well as participants through consenting to the study, agree that the monitor may inspect study facilities and source records (e.g., informed consent forms, clinic and laboratory records, other source documents), as well as observe the performance of study procedures (in accordance with site IRB requirements). Such information will be treated as strictly confidential and will under no circumstances be made publicly available.

The monitoring will adhere to GCP guidelines. The Principal Investigator will permit inspection of the facilities and all study-related documentation by authorized representatives of IAVI, and Government and Regulatory Authorities responsible for this study.

17.6 Investigator's Records

Study records include administrative documentation—e.g., reports and correspondence relating to the study—as well as documentation related to each participant screened and/or enrolled in the study—including informed consent forms, case report forms, and all other source documents. The investigator will maintain and store, in a secure manner, complete, accurate, and current study records for a minimum of 2 years after marketing application approval or the study is discontinued and applicable national and local health authorities are notified. IAVI will notify the Principal Investigator of these events.

18.0 INDEMNITY

The Sponsor and Institution are responsible to have appropriate liability insurance. For research-related injuries and/or medical problems determined to result from receiving the IP, treatment including necessary emergency treatment and proper follow-up care will be made available to the participant free of charge at the expense of the Sponsor.

19.0 PUBLICATION

A primary manuscript describing safety, anti-viral effect and immune responses in this trial will be prepared promptly after the data analysis is available.

Authors will be representatives of each trial site, the data management and statistical analysis center, the laboratories, the product developer and the sponsor, participant to the generally accepted criteria of contributions to the design and conduct of the study, the analysis of data and writing of the manuscript. Precedence will be given to authors from the site enrolling the greatest number of participants. Manuscripts will be reviewed by representatives of each participating group as specified in the CTA**.**

20.0 ETHICAL CONSIDERATIONS

The Principal Investigator will ensure that the study is conducted in compliance with the protocol, SOPs in accordance with guidelines formulated by the ICH for GCP in clinical studies, the ethical principles that have their origins in the Declaration of Helsinki and applicable local standards and regulatory requirements.

APPENDIX A: SCHEDULE OF PROCEDURES

Study Month		0								1		2		3	4	5	6
**Study Week**		**0**				**1**		**2**	**3**	**4**	**6**	**8**	**10**	**12**	**16**	**20**	**24**
**Study Day**	**Scr**	**0**	**1**	**2**	**3**	**7**	**10[5]**	**14**	**21**	**28**	**42**	**56**	**70**	**84**	**112**	**140**	**168/ET[9]**
**Visit Windows (Days)**	**-42**	**0**	**0**	**0**	**0**	**± 1**	**0**	**± 2**	**± 2**	**± 2**	**± 3**	**± 3**	**±3**	**±7**	**±7**	**±7**	**±7**
**INVESTIGATIONAL PRODUCT**																	
Investigational Product /Placebo		X															
**CONSENT/ASSESSMENTS/COUNSELLING**																	
Informed Consent	X																
Assessment of Understanding	X																
HIV Risk Assessment[3]		X															X
HIV Risk Reduction Counselling[2]	X	X								X		X		X	X	X	X
HIV-test Counselling[3]	X	X															X
ART counseling[5]	X	X										X					X
Family Planning Counselling	X	X															
Social Impact Assessment																	X
**CLINICAL SAFETY ASSESSMENTS**																	
Comprehensive Medical History	X																
Interim Medical History		X	X	X	X	X	X	X	X	X	X	X					
Concomitant Medications	X	X	X	X	X	X	X	X	X	X	X	X					
General Physical Exam	X																X
Directed Physical Exam		X	X	X	X	X	X	X	X	X	X	X	X	X	X	X	
Weight	X	X															X
Height	X																
Vital Signs	X	X[4]	X	X	X	X	X	X	X	X	X	X	X	X	X	X	X
Local & Systemic Reactogenicity		X[4]	X	X	X												
Adverse Events		X	X	X	X	X	X	X	X	X	X	X					
Serious Adverse Events and pIMD	X	X	X	X	X	X	X	X	X	X	X	X	X	X	X	X	X

Study Month		0								1		2		3	4	5	6
**Study Week**		**0**				**1**		**2**	**3**	**4**	**6**	**8**	**10**	**12**	**16**	**20**	**24**
**Study Day**	**Scr**	**0**	**1**	**2**	**3**	**7**	**10[5]**	**14**	**21**	**28**	**42**	**56**	**70**	**84**	**112**	**140**	**168/ET[9]**
**Visit Windows (Days)**	**-42**	**0**	**0**	**0**	**0**	**± 1**	**0**	**± 2**	**± 2**	**± 2**	**± 3**	**± 3**	**±3**	**±7**	**±7**	**±7**	**±7**
**CLINICAL LABORATORY TESTS**																	
Hematology and Coagulation	X	X			X	X		X		X		X		X	X	X	X
CD4[1]	X	X				X		X		X		X					X
Clinical Chemistry	X	X			X	X		X		X		X		X	X	X	X
Urine Dipstick[11]	X	X			X	X		X		X		X		X	X	X	X
Urine Pregnancy test	X	X												X			X
Active Syphilis	X																
Hepatitis B	X																
Hepatitis C	X																
HIV diagnostic (4th generation Ag/Ab test)[3]	X	X								X							X
HIV Viral Load[1]	X	X	X	X	X	X	X	X	X	X	X	X	X	X	X	X	X
**RESEARCH LABORATORY TESTS**	-	-	-	-	-	-	-	-	-	-	-	-	-	-	-	-	-
Anti PGT121 Antibodies (ADA)		X								X		X		X			X
HIV testing for PGT121 susceptibility[6]	X																X
HIV SGA sequencing[12]	X									X							X
HIV genotypic testing for ART resistance[12]	X									X							X
HIV reservoir size assessment[1]	X							X						X			
Humoral Assays[7]		X			X	X		X		X		X		X			X
Cellular Assays[7]		X				X		X		X		X		X			X
HLA typing		X															
**PHARMACOKINETICS PGT121 ELISA**	X[7]	X[8]	X	X	X	X		X	X	X	X	X	X	X	X	X	X
**MUCOSAL SAMPLING**		X[10]	X			X		X									
**PLASMA/SERUM STORAGE**	X	X	X	X	X	X	X	X	X	X		X		X			X

Study Month		0								1		2		3	4	5	6
**Study Week**		**0**				**1**		**2**	**3**	**4**	**6**	**8**	**10**	**12**	**16**	**20**	**24**
**Study Day**	**Scr**	**0**	**1**	**2**	**3**	**7**	**10[5]**	**14**	**21**	**28**	**42**	**56**	**70**	**84**	**112**	**140**	**168/ET[9]**
**Visit Windows (Days)**	**-42**	**0**	**0**	**0**	**0**	**± 1**	**0**	**± 2**	**± 2**	**± 2**	**± 3**	**± 3**	**±3**	**±7**	**±7**	**±7**	**±7**
**PBMCs STORAGE**		**X**										**X**		**X**			**X**

1. For groups 2 and 3 only
2. Group 1: will receive HIV risk reduction counselling; Groups 2 and 3: HIV risk reduction counselling as secondary prevention to reduce onward transmission
3. Group 1 only
4. At baseline, approximately 30 minutes after IP administration start, and at hours 1 through 12 after IV infusion start. Local and systemic reactogenicity will be assessed by clinic staff at visits on study days 1, 2 and 3. Local and Systemic reactogenicity will also be assessed by the participant using the Memory Aid on study days 1, 2, and 3
5. Group 3 only
6. Baseline assessment of participants autologous HIV for neutralization susceptibility to PGT121 in-vitro (group 3 only).
7. See Laboratory Analytical Plan for details
8. Day 0 PK draws done immediately before IP administration, at the end of the IV infusion of IP, and 30 minutes and 3 hours post end of the IP administration. Additional PK draws on day 0 are done 6, 9 and 12 hours after the start of the IV infusion of IP. The screening sample is not a PK assessment per se, the PGT121 ELISA will be done to exclude autologous PGT121-like antibody levels above the cut-off. See SOM for details
9. Early Termination (ET): Procedures to be performed at ET are the same as last visit procedures
10. Cervico-vaginal and/or rectal mucosal sampling (optional) on Day 0 must be done prior to IV infusion of IP. See SOM for details
11. Urinalysis for group 3 will only be conducted at visits after screening if clinically indicated.
12. HIV envelope single genome amplification (SGA) sequencing and ART resistance genotyping will be performed in all subjects of group 3 and in subjects of group 2 only if they develop detectable plasma viremia >1000 HIV cp/ml during the study.

APPENDIX B: LOW RISK CRITERIA

Low risk will be defined as:

1. **SEXUAL BEHAVIORS**

   In the **last 12 months** did not:

   - Have oral, vaginal or anal intercourse with an HIV-infected partner, or partner who uses injection drugs.
   - Gave or receive money, drugs, gifts, or services in exchange for oral, vaginal or anal sex

   AND

   In the **last 6 months** has abstained from penile/anal or penile/vaginal intercourse

   OR

   In the **last 6 months**:

   - Had 4 or fewer partners of the opposite birth sex for vaginal and/or anal intercourse, OR

Is MSM (person born male with partner(s) born male) who, in the **last 12 months**:

- Had 2 or fewer MSM partners for anal intercourse and had no unprotected anal sex with MSM, OR
- Had unprotected anal intercourse with only 1 MSM partner, within a monogamous relationship lasting at least 12 months (during which neither partner had any other partners). If the monogamous relationship ended, the participant may then have had protected anal intercourse with 1 other MSM partner (total 2 or fewer partners in the last 12 months).

Is a transgender person, regardless of the point on the transition spectrum, having sex with men (born male) and/or other transgender persons, who in the last 12 months:

- Had 2 or fewer partners for anal or vaginal intercourse, and had no unprotected anal or vaginal sex, OR
- Had unprotected anal or vaginal intercourse sex with 1 partner only within a monogamous relationship lasting at least 12 months (during which neither partner had any other partners). If the monogamous relationship ended, may then have had protected anal or vaginal sex with one other partner (total 2 or fewer partners in the last 12 months).

AND

Uses or intends to use condoms in situations which may include penile/anal or penile/vaginal intercourse with new partners of unknown HIV status, occasional partners, partners outside a primary relationship, and/or partners known to have other partners.

2. **NON-SEXUAL BEHAVIORS**

   In the **last 12 months** did not:

   - Inject drugs or other substances without a prescription
   - Use cocaine, methamphetamine, or excessive alcohol, which in the investigator's judgement, rendered the participant at greater than low risk for acquiring HIV infection

The investigator's judgement should consider local epidemiologic information about HIV prevalence in the area and community networks.

A participant is NOT appropriate for inclusion if he/she:

Acquired an STI (ie, new infection) in the last 12 months:

- Syphilis
- Gonorrhea
- Non-gonococcal urethritis
- HSV-2
- Chlamydia
- Pelvic inflammatory disease (PID)
- Trichomonas
- Mucopurulent cervicitis
- Epididymitis
- Proctitis
- Lymphogranuloma venereum
- Chancroid
- Hepatitis B

APPENDIX C: ADVERSE EVENT SEVERITY ASSESSMENT TABLE

Adapted from: DIVISION OF AIDS (DAIDS) TABLE FOR GRADING THE SEVERITY OF ADULT AND PEDIATRIC ADVERSE EVENTS Version 2.0, November 2014

The *Division of AIDS* (*DAIDS) Table for Grading the Severity of Adult and Pediatric Adverse Events, Version 2.0* consists of parameters, or AEs, with severity grading guidance that are to be used in DAIDS clinical trials for safety data reporting to maintain accuracy and consistency in the evaluation of AEs.

I. Instructions and Clarifications

Grading Adult and Pediatric AEs
When a single parameter is not appropriate for grading an AE in both adult and pediatric populations, separate parameters with specified age ranges are provided. If no distinction between adult and pediatric populations has been made, the listed parameter should be used for grading an AE in both populations.

Note: The term "severe" is not the same as "serious" in classifying AEs. The severity of a specific event describes its intensity, and it is the intensity which is graded. Seriousness, which is not graded, relates to an outcome of an AE and is a regulatory definition.

Estimating Severity Grade for Parameters Not Identified in the Table
The functional table below should be used to grade the severity of an AE that is not specifically identified in the grading table. In addition, all deaths related to an AE are to be classified as grade 5.

PARAMETER	GRADE 1 MILD	GRADE 2 MODERATE	GRADE 3 SEVERE	GRADE 4 POTENTIALLY LIFE-THREATENING
**Clinical** adverse event **NOT** identified elsewhere in the grading table	Mild symptoms causing no or minimal interference with usual social & functional activities with intervention not indicated	Moderate symptoms causing greater than minimal interference with usual social & functional activities with intervention indicated	Severe symptoms causing inability to perform usual social & functional activities with intervention or hospitalization indicated	Potentially life-threatening symptoms causing inability to perform basic self-care functions with intervention indicated to prevent permanent impairment, persistent disability, or death

Determining Severity Grade for Parameters "Between Grades"
If the severity of an AE could fall in either one of two grades (e.g., the severity of an AE could be either Grade 2 or Grade 3), sites should select the higher of the two grades.

Laboratory Values
General. An asymptomatic, abnormal laboratory finding without an accompanying AE should not be reported to DAIDS in an expedited timeframe unless it meets protocol-specific reporting requirements. Sites should refer to the applicable network standards for reporting abnormal laboratory findings on the CRFs.

Values Below Grade 1
Any laboratory value that is between the ULN and grade 1 (for high values) or the LLN and grade 1 (for low values) should not be graded or reported as an AE. Sites should consult the Manual for Expedited Reporting of Adverse Events to DAIDS, Version 2.0 and their protocol when making an assessment of the need to report an AE.

Overlap of Local Laboratory Normal Values with Grading Table Ranges.
When local laboratory normal values fall within grading table laboratory ranges, the severity grading is based on the ranges in the grading table unless there is a protocol-specific grading criterion for the laboratory value. For example, "Magnesium, Low" has a grade 1 range of 1.2 to < 1.4 mEq/L, while a

particular laboratory's normal range for magnesium may be 1.3 to 2.8 mEq/L. If a study participant's magnesium laboratory value is 1.3 mEq/L, the laboratory value should be graded as grade 1.

II. Definitions of terms used in the Table:

Basic Self-care Functions	Adult<br>Activities such as bathing, dressing, toileting, transfer or movement, continence, and feeding.<br><br>Young Children<br>Activities that are age and culturally appropriate, such as feeding one's self with culturally appropriate eating implements.
LLN	Lower limit of normal
Intervention	Medical, surgical, or other procedures recommended or provided by a healthcare professional for the treatment of an adverse event.
NA	Not Applicable
ULN	Upper limit of normal
Usual Social & Functional Activities	Activities which adults and children perform on a routine basis and those which are part of regular activities of daily living, for example:<br><br>Adults<br>Adaptive tasks and desirable activities, such as going to work, shopping, cooking, use of transportation, or pursuing a hobby.<br><br>Young Children<br>Activities that are age and culturally appropriate, such as social interactions, play activities, or learning tasks.

Major Clinical Conditions

Cardiovascular

PARAMETER	GRADE 1 MILD	GRADE 2 MODERATE	GRADE 3 SEVERE	GRADE 4 POTENTIALLY LIFE-THREATENING
**Arrhythmia** (by ECG or physical examination) *Specify type, if applicable*	No symptoms AND No intervention indicated	No symptoms AND Non-urgent intervention indicated	Non-life-threatening symptoms AND Non-urgent intervention indicated	Life-threatening arrhythmia OR Urgent intervention indicated
**Blood Pressure Abnormalities[1]** ***Hypertension*** *(with the lowest reading taken after repeat testing during a visit)* *≥ 18 years of age*	140 to < 160 mmHg systolic OR 90 to < 100 mmHg diastolic	≥ 160 to < 180 mmHg systolic OR ≥ 100 to < 110 mmHg diastolic	≥ 180 mmHg systolic OR ≥ 110 mmHg diastolic	Life-threatening consequences in a participant not previously diagnosed with hypertension (e.g., malignant hypertension) OR Hospitalization indicated
< 18 years of age	> 120/80 mmHg	≥ 95th to < 99th percentile + 5 mmHg adjusted for age, height, and gender (systolic and/or diastolic)	≥ 99th percentile + 5 mmHg adjusted for age, height, and gender (systolic and/or diastolic)	Life-threatening consequences in a participant not previously diagnosed with hypertension (e.g., malignant hypertension) OR Hospitalization indicated
Hypotension	No symptoms	Symptoms corrected with oral fluid replacement	Symptoms AND IV fluids indicated	Shock requiring use of vasopressors or mechanical assistance to maintain blood pressure
**Cardiac Ischemia or Infarction** *Report only one*	NA	NA	New symptoms with ischemia (stable angina) OR New testing consistent with ischemia	Unstable angina OR Acute myocardial infarction
**Heart Failure**	No symptoms AND Laboratory or cardiac imaging abnormalities	Symptoms with mild to moderate activity or exertion	Symptoms at rest or with minimal activity or exertion (e.g., hypoxemia) OR Intervention indicated (e.g., oxygen)	Life-threatening consequences OR Urgent intervention indicated (e.g., vasoactive medications, ventricular assist device, heart transplant)
**Hemorrhage** (with significant acute blood loss)	NA	Symptoms AND No transfusion indicated	Symptoms AND Transfusion of ≤ 2 units packed RBCs indicated	Life-threatening hypotension OR Transfusion of > 2 units packed RBCs (for children, packed RBCs > 10 cc/kg) indicated

[1] Blood pressure norms for children < 18 years of age can be found in: Expert Panel on Integrated Guidelines for Cardiovascular Health and Risk Reduction in Children and Adolescents. *Pediatrics* 2011;128;S213; originally published online November 14, 2011; DOI: 10.1542/peds.2009- 2107C.

PARAMETER	GRADE 1 MILD	GRADE 2 MODERATE	GRADE 3 SEVERE	GRADE 4 POTENTIALLY LIFE-THREATENING
**Prolonged PR Interval or AV Block** *Report only one* *> 16 years of age*	PR interval 0.21 to < 0.25 seconds	PR interval ≥ 0.25 seconds OR Type I 2nd degree AV block	Type II 2nd degree AV block OR Ventricular pause ≥ 3.0 seconds	Complete AV block
≤ 16 years of age	1st degree AV block (PR interval > normal for age and rate)	Type I 2nd degree AV block	Type II 2nd degree AV block OR Ventricular pause ≥ 3.0 seconds	Complete AV block
**Prolonged QTc Interval**[2]	0.45 to 0.47 seconds	> 0.47 to 0.50 seconds	> 0.50 seconds OR ≥ 0.06 seconds above baseline	Life-threatening consequences (e.g., Torsade de pointes, other associated serious ventricular dysrhythmia)
**Thrombosis or Embolism** *Report only one*	NA	Symptoms AND No intervention indicated	Symptoms AND Intervention indicated	Life-threatening embolic event (e.g., pulmonary embolism, thrombus)

[2] As per Bazett's formula.

Dermatologic

PARAMETER	GRADE 1 MILD	GRADE 2 MODERATE	GRADE 3 SEVERE	GRADE 4 POTENTIALLY LIFE-THREATENING
**Alopecia** (scalp only)	Detectable by study participant, caregiver, or physician AND Causing no or minimal interference with usual social & functional activities	Obvious on visual inspection AND Causing greater than minimal interference with usual social & functional activities	NA	NA
**Bruising**	Localized to one area	Localized to more than one area	Generalized	NA
**Cellulitis**	NA	Non-parenteral treatment indicated (e.g., oral antibiotics, antifungals, antivirals)	IV treatment indicated (e.g., IV antibiotics, antifungals, antivirals)	Life-threatening consequences (e.g., sepsis, tissue necrosis)
**Hyperpigmentation**	Slight or localized causing no or minimal interference with usual social & functional activities	Marked or generalized causing greater than minimal interference with usual social & functional activities	NA	NA

**Hypopigmentation**	Slight or localized causing no or minimal interference with usual social & functional activities	Marked or generalized causing greater than minimal interference with usual social & functional activities	NA	NA
**Petechiae**	Localized to one area	Localized to more than one area	Generalized	NA
**Pruritus**[3] (without skin lesions)	Itching causing no or minimal interference with usual social & functional activities	Itching causing greater than minimal interference with usual social & functional activities	Itching causing inability to perform usual social & functional activities	NA
**Rash** *Specify type, if applicable*	Localized rash	Diffuse rash OR Target lesions	Diffuse rash AND Vesicles or limited number of bullae or superficial ulcerations of mucous membrane limited to one site	Extensive or generalized bullous lesions OR Ulceration of mucous membrane involving two or more distinct mucosal sites OR Stevens-Johnson syndrome OR Toxic epidermal necrolysis

[3] For pruritus associated with injections or infusions, see the *Site Reactions to Injections and Infusions* section (page 23).

Endocrine and Metabolic

PARAMETER	GRADE 1 MILD	GRADE 2 MODERATE	GRADE 3 SEVERE	GRADE 4 POTENTIALLY LIFE-THREATENING
**Diabetes Mellitus**	Controlled without medication	Controlled with medication OR Modification of current medication regimen	Uncontrolled despite treatment modification OR Hospitalization for immediate glucose control indicated	Life-threatening consequences (e.g., ketoacidosis, hyperosmolar non-ketotic coma, end organ failure)
**Gynecomastia**	Detectable by study participant, caregiver, or physician AND Causing no or minimal interference with usual social & functional activities	Obvious on visual inspection AND Causing pain with greater than minimal interference with usual social & functional activities	Disfiguring changes AND Symptoms requiring intervention or causing inability to perform usual social & functional activities	NA

**Hyperthyroidism**	No symptoms AND Abnormal laboratory value	Symptoms causing greater than minimal interference with usual social & functional activities OR Thyroid suppression therapy indicated	Symptoms causing inability to perform usual social & functional activities OR Uncontrolled despite treatment modification	Life-threatening consequences (e.g., thyroid storm)
**Hypothyroidism**	No symptoms AND Abnormal laboratory value	Symptoms causing greater than minimal interference with usual social & functional activities OR Thyroid replacement therapy indicated	Symptoms causing inability to perform usual social & functional activities OR Uncontrolled despite treatment modification	Life-threatening consequences (e.g., myxedema coma)
**Lipoatrophy**[4]	Detectable by study participant, caregiver, or physician AND Causing no or minimal interference with usual social & functional activities	Obvious on visual inspection AND Causing greater than minimal interference with usual social & functional activities	Disfiguring changes	NA
**Lipohypertrophy**[5]	Detectable by study participant, caregiver, or physician AND Causing no or minimal interference with usual social & functional activities	Obvious on visual inspection AND Causing greater than minimal interference with usual social & functional activities	Disfiguring changes	NA

[4] Definition: A disorder characterized by fat loss in the face, extremities, and buttocks.
[5] Definition: A disorder characterized by abnormal fat accumulation on the back of the neck, breasts, and abdomen.

Gastro-intestinal

PARAMETER	GRADE 1 MILD	GRADE 2 MODERATE	GRADE 3 SEVERE	GRADE 4 POTENTIALLY LIFE-THREATENING
**Anorexia**	Loss of appetite without decreased oral intake	Loss of appetite associated with decreased oral intake without significant weight loss	Loss of appetite associated with significant weight loss	Life-threatening consequences OR Aggressive intervention indicated (e.g., tube feeding, total parenteral nutrition)
**Ascites**	No symptoms	Symptoms AND Intervention indicated (e.g., diuretics, therapeutic paracentesis)	Symptoms recur or persist despite intervention	Life-threatening consequences

**Bloating or Distension**<br>*Report only one*	Symptoms causing no or minimal interference with usual social & functional activities	Symptoms causing greater than minimal interference with usual social & functional activities	Symptoms causing inability to perform usual social & functional activities	NA
**Cholecystitis**	NA	Symptoms AND Medical intervention indicated	Radiologic, endoscopic, or operative intervention indicated	Life-threatening consequences (e.g., sepsis, perforation)
**Constipation**	NA	Persistent constipation requiring regular use of dietary modifications, laxatives, or enemas	Obstipation with manual evacuation indicated	Life-threatening consequences (e.g., obstruction)
**Diarrhea**<br>*≥ 1 year of age*	Transient or intermittent episodes of unformed stools OR Increase of ≤ 3 stools over baseline per 24-hour period	Persistent episodes of unformed to watery stools OR Increase of 4 to 6 stools over baseline per 24-hour period	Increase of ≥ 7 stools per 24-hour period OR IV fluid replacement indicated	Life-threatening consequences (e.g., hypotensive shock)
< 1 year of age	Liquid stools (more unformed than usual) but usual number of stools	Liquid stools with increased number of stools OR Mild dehydration	Liquid stools with moderate dehydration	Life-threatening consequences (e.g., liquid stools resulting in severe dehydration, hypotensive shock)
**Dysphagia or Odynophagia**<br>*Report only one and specify location*	Symptoms but able to eat usual diet	Symptoms causing altered dietary intake with no intervention indicated	Symptoms causing severely altered dietary intake with intervention indicated	Life-threatening reduction in oral intake
**Gastrointestinal Bleeding**	Not requiring intervention other than iron supplement	Endoscopic intervention indicated	Transfusion indicated	Life-threatening consequences (e.g., hypotensive shock)

**PARAMETER**	**GRADE 1 MILD**	**GRADE 2 MODERATE**	**GRADE 3 SEVERE**	**GRADE 4 POTENTIALLY LIFE-THREATENING**
**Mucositis or Stomatitis**<br>*Report only one and specify location*	Mucosal erythema	Patchy pseudomembranes or ulcerations	Confluent pseudomembranes or ulcerations OR Mucosal bleeding with minor trauma	Life-threatening consequences (e.g., aspiration, choking) OR Tissue necrosis OR Diffuse spontaneous mucosal bleeding
**Nausea**	Transient (< 24 hours) or intermittent AND No or minimal interference with oral intake	Persistent nausea resulting in decreased oral intake for 24 to 48 hours	Persistent nausea resulting in minimal oral intake for > 48 hours OR Rehydration indicated (e.g., IV fluids)	Life-threatening consequences (e.g., hypotensive shock)

**Pancreatitis**	NA	Symptoms with hospitalization not indicated	Symptoms with hospitalization indicated	Life-threatening consequences (e.g., circulatory failure, hemorrhage, sepsis)
**Perforation** (colon or rectum)	NA	NA	Intervention indicated	Life-threatening consequences
**Proctitis**	Rectal discomfort with no intervention indicated	Symptoms causing greater than minimal interference with usual social & functional activities OR Medical intervention indicated	Symptoms causing inability to perform usual social & functional activities OR Operative intervention indicated	Life-threatening consequences (e.g., perforation)
**Rectal Discharge**	Visible discharge	Discharge requiring the use of pads	NA	NA
**Vomiting**	Transient or intermittent AND No or minimal interference with oral intake	Frequent episodes with no or mild dehydration	Persistent vomiting resulting in orthostatic hypotension OR Aggressive rehydration indicated (e.g., IV fluids)	Life-threatening consequences (e.g., hypotensive shock)

Musculoskeletal

PARAMETER	GRADE 1 MILD	GRADE 2 MODERATE	GRADE 3 SEVERE	GRADE 4 POTENTIALLY LIFE-THREATENING
**Arthralgia**	Joint pain causing no or minimal interference with usual social & functional activities	Joint pain causing greater than minimal interference with usual social & functional activities	Joint pain causing inability to perform usual social & functional activities	Disabling joint pain causing inability to perform basic self-care functions
**Arthritis**	Stiffness or joint swelling causing no or minimal interference with usual social & functional activities	Stiffness or joint swelling causing greater than minimal interference with usual social & functional activities	Stiffness or joint swelling causing inability to perform usual social & functional activities	Disabling joint stiffness or swelling causing inability to perform basic self-care functions
**Myalgia** (generalized)	Muscle pain causing no or minimal interference with usual social & functional activities	Muscle pain causing greater than minimal interference with usual social & functional activities	Muscle pain causing inability to perform usual social & functional activities	Disabling muscle pain causing inability to perform basic self-care functions
**Osteonecrosis**	NA	No symptoms but with radiographic findings AND No operative intervention indicated	Bone pain with radiographic findings OR Operative intervention indicated	Disabling bone pain with radiographic findings causing inability to perform basic self-care functions
**Osteopenia**[6] *≥ 30 years of age*	BMD t-score -2.5 to -1	NA	NA	NA
< 30 years of age	BMD z-score -2 to -1	NA	NA	NA
**Osteoporosis**[6] *≥ 30 years of age*	NA	BMD t-score < -2.5	Pathologic fracture (e.g., compression fracture causing loss of vertebral height)	Pathologic fracture causing life-threatening consequences
< 30 years of age	NA	BMD z-score < -2	Pathologic fracture (e.g., compression fracture causing loss of vertebral height)	Pathologic fracture causing life-threatening consequences

[6] BMD t and z scores can be found in: Kanis JA on behalf of the World Health Organization Scientific Group (2007). Assessment of osteoporosis at the primary health-care level. Technical Report. World Health Organization Collaborating Centre for Metabolic Bone Diseases, University of Sheffield, UK. 2007: Printed by the University of Sheffield.

Neurologic

PARAMETER	GRADE 1 MILD	GRADE 2 MODERATE	GRADE 3 SEVERE	GRADE 4 POTENTIALLY LIFE-THREATENING
**Acute CNS Ischemia**	NA	NA	Transient ischemic attack	Cerebral vascular accident (e.g., stroke with neurological deficit)
**Altered Mental Status** (for Dementia, see *Cognitive, Behavioral, or Attentional Disturbance* below)	Changes causing no or minimal interference with usual social & functional activities	Mild lethargy or somnolence causing greater than minimal interference with usual social & functional activities	Confusion, memory impairment, lethargy, or somnolence causing inability to perform usual social & functional activities	Delirium OR Obtundation OR Coma
**Ataxia**	Symptoms causing no or minimal interference with usual social & functional activities OR No symptoms with ataxia detected on examination	Symptoms causing greater than minimal interference with usual social & functional activities	Symptoms causing inability to perform usual social & functional activities	Disabling symptoms causing inability to perform basic self-care functions
**Cognitive, Behavioral, or Attentional Disturbance** (includes dementia and attention deficit disorder) *Specify type, if applicable*	Disability causing no or minimal interference with usual social & functional activities OR Specialized resources not indicated	Disability causing greater than minimal interference with usual social & functional activities OR Specialized resources on part- time basis indicated	Disability causing inability to perform usual social & functional activities OR Specialized resources on a full-time basis indicated	Disability causing inability to perform basic self-care functions OR Institutionalization indicated
**Developmental Delay** *< 18 years of age* *Specify type, if applicable*	Mild developmental delay, either motor or cognitive, as determined by comparison with a developmental screening tool appropriate for the setting	Moderate developmental delay, either motor or cognitive, as determined by comparison with a developmental screening tool appropriate for the setting	Severe developmental delay, either motor or cognitive, as determined by comparison with a developmental screening tool appropriate for the setting	Developmental regression, either motor or cognitive, as determined by comparison with a developmental screening tool appropriate for the setting
**Headache**	Symptoms causing no or minimal interference with usual social & functional activities	Symptoms causing greater than minimal interference with usual social & functional activities	Symptoms causing inability to perform usual social & functional activities	Symptoms causing inability to perform basic self-care functions OR Hospitalization indicated OR Headache with significant impairment of alertness or other neurologic function

PARAMETER	GRADE 1<br>MILD	GRADE 2<br>MODERATE	GRADE 3<br>SEVERE	GRADE 4<br>POTENTIALLY LIFE-THREATENING
**Neuromuscular Weakness** (includes myopathy and neuropathy)<br>*Specify type, if applicable*	Minimal muscle weakness causing no or minimal interference with usual social & functional activities OR No symptoms with decreased strength on examination	Muscle weakness causing greater than minimal interference with usual social & functional activities	Muscle weakness causing inability to perform usual social & functional activities	Disabling muscle weakness causing inability to perform basic self-care functions OR Respiratory muscle weakness impairing ventilation
**Neurosensory Alteration** (includes paresthesia and painful neuropathy)<br>*Specify type, if applicable*	Minimal paresthesia causing no or minimal interference with usual social & functional activities OR No symptoms with sensory alteration on examination	Sensory alteration or paresthesia causing greater than minimal interference with usual social & functional activities	Sensory alteration or paresthesia causing inability to perform usual social & functional activities	Disabling sensory alteration or paresthesia causing inability to perform basic self-care functions
**Seizures**<br>***New Onset Seizure*** *≥ 18 years of age*	NA	NA	1 to 3 seizures	Prolonged and repetitive seizures (e.g., status epilepticus) OR Difficult to control (e.g., refractory epilepsy)
< 18 years of age (includes new or pre- existing febrile seizures)	Seizure lasting < 5 minutes with < 24 hours postictal state	Seizure lasting 5 to < 20 minutes with < 24 hours postictal state	Seizure lasting ≥ 20 minutes OR > 24 hours postictal state	Prolonged and repetitive seizures (e.g., status epilepticus) OR Difficult to control (e.g., refractory epilepsy)
Pre-existing Seizure	NA	Increased frequency from previous level of control without change in seizure character	Change in seizure character either in duration or quality (e.g., severity or focality)	Prolonged and repetitive seizures (e.g., status epilepticus) OR Difficult to control (e.g., refractory epilepsy)
**Syncope**	Near syncope without loss of consciousness (e.g., pre-syncope)	Loss of consciousness with no intervention indicated	Loss of consciousness AND Hospitalization or intervention required	NA

Pregnancy, Puerperium, and Perinatal

PARAMETER	GRADE 1<br>MILD	GRADE 2<br>MODERATE	GRADE 3<br>SEVERE	GRADE 4<br>POTENTIALLY LIFE-THREATENING
**Fetal Death or Stillbirth** (report using mother's participant ID)<br>*Report only one*	NA	NA	Fetal loss occurring at ≥ 20 weeks gestation	NA
**Preterm Delivery**[7] (report using mother's participant ID)	Delivery at 34 to < 37 weeks gestational age	Delivery at 28 to < 34 weeks gestational age	Delivery at 24 to < 28 weeks gestational age	Delivery at < 24 weeks gestational age
**Spontaneous Abortion or Miscarriage**[8] (report using mother's participant ID)<br>*Report only one*	Chemical pregnancy	Uncomplicated spontaneous abortion or miscarriage	Complicated spontaneous abortion or miscarriage	NA

7 Definition: A delivery of a live-born neonate occurring at ≥ 20 to < 37 weeks gestational age.
8 Definition: A clinically recognized pregnancy occurring at < 20 weeks gestational age.

Psychiatric

PARAMETER	GRADE 1<br>MILD	GRADE 2<br>MODERATE	GRADE 3<br>SEVERE	GRADE 4<br>POTENTIALLY LIFE-THREATENING
**Insomnia**	Mild difficulty falling asleep, staying asleep, or waking up early	Moderate difficulty falling asleep, staying asleep, or waking up early	Severe difficulty falling asleep, staying asleep, or waking up early	NA
**Psychiatric Disorders** (includes anxiety, depression, mania, and psychosis)<br>*Specify disorder*	Symptoms with intervention not indicated OR Behavior causing no or minimal interference with usual social & functional activities	Symptoms with intervention indicated OR Behavior causing greater than minimal interference with usual social & functional activities	Symptoms with hospitalization indicated OR Behavior causing inability to perform usual social & functional activities	Threatens harm to self or others OR Acute psychosis OR Behavior causing inability to perform basic self-care functions
**Suicidal Ideation or Attempt**<br>*Report only one*	Preoccupied with thoughts of death AND No wish to kill oneself	Preoccupied with thoughts of death AND Wish to kill oneself with no specific plan or intent	Thoughts of killing oneself with partial or complete plans but no attempt to do so OR Hospitalization indicated	Suicide attempted

Respiratory

PARAMETER	GRADE 1 MILD	GRADE 2 MODERATE	GRADE 3 SEVERE	GRADE 4 POTENTIALLY LIFE-THREATENING
**Acute Bronchospasm**	Forced expiratory volume in 1 second or peak flow reduced to ≥ 70 to < 80% OR Mild symptoms with intervention not indicated	Forced expiratory volume in 1 second or peak flow 50 to < 70% OR Symptoms with intervention indicated OR Symptoms causing greater than minimal interference with usual social & functional activities	Forced expiratory volume in 1 second or peak flow 25 to < 50% OR Symptoms causing inability to perform usual social & functional activities	Forced expiratory volume in 1 second or peak flow < 25% OR Life-threatening respiratory or Hemodynamic compromise OR Intubation
**Dyspnea or Respiratory Distress** *Report only one*	Dyspnea on exertion with no or minimal interference with usual social & functional activities OR Wheezing OR Minimal increase in respiratory rate for age	Dyspnea on exertion causing greater than minimal interference with usual social & functional activities OR Nasal flaring OR Intercostal retractions OR Pulse oximetry 90 to < 95%	Dyspnea at rest causing inability to perform usual social & functional activities OR Pulse oximetry < 90%	Respiratory failure with ventilator support indicated (e.g., CPAP, BPAP, intubation)

Sensory

PARAMETER	GRADE 1 MILD	GRADE 2 MODERATE	GRADE 3 SEVERE	GRADE 4 POTENTIALLY LIFE-THREATENING
**Hearing Loss** *≥ 12 years of age*	NA	Hearing aid or intervention not indicated	Hearing aid or intervention indicated	Profound bilateral hearing loss (> 80 dB at 2 kHz and above) OR Non-serviceable hearing (i.e., >50 dB audiogram and <50% speech discrimination)
< 12 years of age (based on a 1, 2, 3, 4, 6 and 8 kHz audiogram)	> 20 dB hearing loss at ≤ 4 kHz	> 20 dB hearing loss at > 4 kHz	> 20 dB hearing loss at ≥ 3 kHz in one ear with additional speech language related services indicated (where available) OR Hearing loss sufficient to indicate therapeutic intervention, including hearing aids	Audiologic indication for cochlear implant and additional speech-language related services indicated (where available)

**Tinnitus**	Symptoms causing no or minimal interference with usual social & functional activities with intervention not indicated	Symptoms causing greater than minimal interference with usual social & functional activities with intervention indicated	Symptoms causing inability to perform usual social & functional activities	NA
**Uveitis**	No symptoms AND Detectable on examination	Anterior uveitis with symptoms OR Medicamylasal intervention indicated	Posterior or pan- uveitis OR Operative intervention indicated	Disabling visual loss in affected eye(s)
**Vertigo**	Vertigo causing no or minimal interference with usual social & functional activities	Vertigo causing greater than minimal interference with usual social & functional activities	Vertigo causing inability to perform usual social & functional activities	Disabling vertigo causing inability to perform basic self-care functions
**Visual Changes** (assessed from baseline)	Visual changes causing no or minimal interference with usual social & functional activities	Visual changes causing greater than minimal interference with usual social & functional activities	Visual changes causing inability to perform usual social & functional activities	Disabling visual loss in affected eye(s)

Systemic

PARAMETER	GRADE 1 MILD	GRADE 2 MODERATE	GRADE 3 SEVERE	GRADE 4 POTENTIALLY LIFE-THREATENING
**Acute Allergic Reaction**	Localized urticaria (wheals) with no medical intervention indicated	Localized urticaria with intervention indicated OR Mild angioedema with no intervention indicated	Generalized urticaria OR Angioedema with intervention indicated OR Symptoms of mild bronchospasm	Acute anaphylaxis OR Life-threatening bronchospasm OR Laryngeal edema
**Chills**	Symptoms causing no or minimal interference with usual social & functional activities	Symptoms causing greater than minimal interference with usual social & functional activities	Symptoms causing inability to perform usual social & functional activities	NA
**Cytokine Release Syndrome**[9]	Mild signs and symptoms AND Therapy (i.e., antibody infusion) interruption not indicated	Therapy (i.e., antibody infusion) interruption indicated AND Responds promptly to symptomatic treatment OR Prophylactic medications indicated for ≤ 24 hours	Prolonged severe signs and symptoms OR Recurrence of symptoms following initial improvement	Life-threatening consequences (e.g., requiring pressor or ventilator support)
**Fatigue or Malaise** *Report only one*	Symptoms causing no or minimal interference with usual social & functional activities	Symptoms causing greater than minimal interference with usual social & functional activities	Symptoms causing inability to perform usual social & functional activities	Incapacitating symptoms of fatigue or malaise causing inability to perform basic self-care functions
**Fever** (non-axillary temperatures only)	38.0 to < 38.6°C or 100.4 to < 101.5°F	≥ 38.6 to < 39.3°C or ≥101.5 to < 102.7°F	≥ 39.3 to < 40.0°C or ≥ 102.7 to < 104.0°F	≥ 40.0°C or ≥ 104.0°F
**Pain**[10] (not associated with study agent injections and not specified elsewhere) *Specify location*	Pain causing no or minimal interference with usual social & functional activities	Pain causing greater than minimal interference with usual social & functional activities	Pain causing inability to perform usual social & functional activities	Disabling pain causing inability to perform basic self-care functions OR Hospitalization indicated
**Serum Sickness**[11]	Mild signs and symptoms	Moderate signs and symptoms AND Intervention indicated (e.g., antihistamines)	Severe signs and symptoms AND Higher level intervention indicated (e.g., steroids or IV fluids)	Life-threatening consequences (e.g., requiring pressor or ventilator support)

[9] ~~Definition: A disorder characterized by nausea~~, headache, tachycardia, hypotension, rash, and/or shortness of breath.
[10] For pain associated with injections or infusions, see the *Site Reactions to Injections and Infusions* section (page 23).
[11] Definition: A disorder characterized by fever, arthralgia, myalgia, skin eruptions, lymphadenopathy, marked discomfort, and/or dyspnea.

PARAMETER	GRADE 1 MILD	GRADE 2 MODERATE	GRADE 3 SEVERE	GRADE 4 POTENTIALLY LIFE-THREATENING
**Underweight**[12] *> 5 to 19 years of age*	NA	WHO BMI z-score < -2 to ≤ -3	WHO BMI z-score < -3	WHO BMI z-score < -3 with life-threatening consequences
2 to 5 years of age	NA	WHO Weight-for-height z-score < -2 to ≤-3	WHO Weight-for- height z-score < -3	WHO Weight-for-height z-score < -3 with life-threatening consequences
< 2 years of age	NA	WHO Weight-for-length z-score < -2 to ≤-3	WHO Weight-for- length z-score < -3	WHO Weight-for-length z-score < -3 with life-threatening consequences
**Weight Loss** (excludes postpartum weight loss)	NA	5 to < 9% loss in body weight from baseline	≥ 9 to < 20% loss in body weight from baseline	≥ 20% loss in body weight from baseline OR Aggressive intervention indicated (e.g., tube feeding, total parenteral nutrition)

[12] WHO reference tables may be accesed by clicking the desired age range or by accessing the following URLs: http://www.who.int/growthref/who2007 bmi for age/en/ for participants > 5 to 19 years of age and http://www.who.int/childgrowth/standards/chart catalogue/en/ for those < 5 years of age.

Urinary

PARAMETER	GRADE 1 MILD	GRADE 2 MODERATE	GRADE 3 SEVERE	GRADE 4 POTENTIALLY LIFE-THREATENING
**Urinary Tract Obstruction**	NA	Signs or symptoms of urinary tract obstruction without hydronephrosis or renal dysfunction	Signs or symptoms of urinary tract obstruction with hydronephrosis or renal dysfunction	Obstruction causing life-threatening consequences

Site Reactions to Injections and Infusions

PARAMETER	GRADE 1 MILD	GRADE 2 MODERATE	GRADE 3 SEVERE	GRADE 4 POTENTIALLY LIFE-THREATENING
**Injection Site Pain or Tenderness** *Report only one*	Pain or tenderness causing no or minimal limitation of use of limb	Pain or tenderness causing greater than minimal limitation of use of limb	Pain or tenderness causing inability to perform usual social & functional activities	Pain or tenderness causing inability to perform basic self-care function OR Hospitalization indicated

**Injection Site Erythema or Redness**[13]<br>*Report only one*<br>*> 15 years of age*	2.5 to < 5 cm in diameter OR 6.25 to < 25 cm^2 surface area AND Symptoms causing no or minimal interference with usual social & functional activities	≥ 5 to < 10 cm in diameter OR ≥ 25 to < 100 cm^2 surface area OR Symptoms causing greater than minimal interference with usual social & functional activities	≥ 10 cm in diameter OR ≥ 100 cm^2 surface area OR Ulceration OR Secondary infection OR Phlebitis OR Sterile abscess OR Drainage OR Symptoms causing inability to perform usual social & functional activities	Potentially lifethreatening consequences (e.g., abscess, exfoliative dermatitis, necrosis involving dermis or deeper tissue)
≤15 years of age	≤ 2.5 cm in diameter	> 2.5 cm in diameter with < 50% surface area of the extremity segment involved (e.g., upper arm or thigh)	≥ 50% surface area of the extremity segment involved (e.g., upper arm or thigh) OR Ulceration OR Secondary infection OR Phlebitis OR Sterile abscess OR Drainage	Potentially life-threatening consequences (e.g., abscess, exfoliative dermatitis, necrosis involving dermis or deeper tissue)
**Injection Site Induration or Swelling**<br>*Report only one*<br>*> 15 years of age*	Same as for **Injection Site Erythema or Redness,** *> 15 years of age*	Same as for **Injection Site Erythema or Redness,** *> 15 years of age*	Same as for **Injection Site Erythema or Redness,** *> 15 years of age*	Same as for **Injection Site Erythema or Redness,** *> 15 years of age*
≤ 15 years of age	Same as for **Injection Site Erythema or Redness,** *≤ 15 years of age*	Same as for **Injection Site Erythema or Redness,** *≤ 15 years of age*	Same as for **Injection Site Erythema or Redness,** *≤ 15 years of age*	Same as for **Injection Site Erythema or Redness,** *≤ 15 years of age*
**Injection Site Pruritus**	Itching localized to the injection site that is relieved spontaneously or in < 48 hours of treatment	Itching beyond the injection site that is not generalized OR Itching localized to the injection site requiring ≥ 48 hours treatment	Generalized itching causing inability to perform usual social & functional activities	NA

[13] Injection Site Erythema or Redness should be evaluated and graded using the greatest single diameter or measured surface area.

Laboratory Values

Chemistries

PARAMETER	GRADE 1 MILD	GRADE 2 MODERATE	GRADE 3 SEVERE	GRADE 4 POTENTIALLY LIFE-THREATENING
**Acidosis**	NA	pH ≥ 7.3 to < LLN	pH < 7.3 without life-threatening consequences	pH < 7.3 with life-threatening consequences
**Albumin, Low** (g/dL; *g/L*)	3.0 to < LLN *30 to < LLN*	≥ 2.0 to < 3.0 ≥ *20 to < 30*	< 2.0<br>< *20*	NA
**Alkaline Phosphatase, High**	1.25 to < 2.5 x ULN	2.5 to < 5.0 x ULN	5.0 to < 10.0 x ULN	≥ 10.0 x ULN
**Alkalosis**	NA	pH > ULN to ≤ 7.5	pH > 7.5 without life-threatening consequences	pH > 7.5 with life-threatening consequences
**ALT or SGPT, High** *Report only one*	1.25 to < 2.5 x ULN	2.5 to < 5.0 x ULN	5.0 to < 10.0 x ULN	≥ 10.0 x ULN
**Amylase (Pancreatic) or Amylase (Total), High** *Report only one*	1.1 to < 1.5 x ULN	1.5 to < 3.0 x ULN	3.0 to < 5.0 x ULN	≥ 5.0 x ULN
**AST or SGOT, High** *Report only one*	1.25 to < 2.5 x ULN	2.5 to < 5.0 x ULN	5.0 to < 10.0 x ULN	≥ 10.0 x ULN
**Bicarbonate, Low** (mEq/L; *mmol/L*)	16.0 to < LLN *16.0 to < LLN*	11.0 to < 16.0 *11.0 to < 16.0*	8.0 to < 11.0 *8.0 to < 11.0*	< 8.0<br>< *8.0*
**Bilirubin**<br>***Direct Bilirubin[14], High***<br>*> 28 days of age*	NA	NA	> ULN	> ULN with lifethreatening consequences (e.g., signs and symptoms of liver failure)
≤ 28 days of age	ULN to ≤ 1 mg/dL	> 1 to ≤ 1.5 mg/dL	> 1.5 to ≤ 2 mg/dL	> 2 mg/dL
Total Bilirubin, High<br>*> 28 days of age*	1.1 to < 1.6 x ULN	1.6 to < 2.6 x ULN	2.6 to < 5.0 x ULN	≥ 5.0 x ULN
≤ 28 days of age	See Appendix A. Total Bilirubin for Term and Preterm Neonates	See Appendix A. Total Bilirubin for Term and Preterm Neonates	See Appendix A. Total Bilirubin for Term and Preterm Neonates	See Appendix A. Total Bilirubin for Term and Preterm Neonates
**Calcium, High** (mg/dL; *mmol/L*)<br>*≥ 7 days of age*	10.6 to < 11.5<br>*2.65* to < *2.88*	11.5 to < 12.5 *2.88* to < *3.13*	12.5 to < 13.5<br>*3.13* to < *3.38*	≥ 13.5<br>≥ *3.38*
< 7 days of age	11.5 to < 12.4 *2.88* to < *3.10*	12.4 to < 12.9 *3.10* to < *3.23*	12.9 to < 13.5 *3.23* to < *3.38*	≥ 13.5<br>≥ *3.38*

PARAMETER	GRADE 1 MILD	GRADE 2 MODERATE	GRADE 3 SEVERE	GRADE 4 POTENTIALLY LIFE-THREATENING
**Calcium (Ionized), High** (mg/dL; *mmol/L*)	> ULN to < 6.0 *> ULN to < 1.5*	6.0 to < 6.4 *1.5 to < 1.6*	6.4 to < 7.2 *1.6 to < 1.8*	≥ 7.2 *≥ 1.8*
**Calcium, Low** (mg/dL; *mmol/L*) *≥ 7 days of age*	7.8 to < 8.4 *1.95* to < *2.10*	7.0 to < 7.8 *1.75* to < *1.95*	6.1 to < 7.0 *1.53* to < *1.75*	< 6.1 *< 1.53*
< 7 days of age	6.5 to < 7.5 *1.63* to < *1.88*	6.0 to < 6.5 *1.50* to < *1.63*	5.50 to < 6.0 *1.38* to < *1.50*	< 5.50 *< 1.38*
**Calcium (Ionized), Low** (mg/dL; *mmol/L*)	< LLN to 4.0 *< LLN to 1.0*	3.6 to < 4.0 *0.9 to < 1.0*	3.2 to < 3.6 *0.8 to < 0.9*	< 3.2 *< 0.8*
**Cardiac Troponin I, High**	NA	NA	NA	Levels consistent with myocardial infarction or unstable angina as defined by the local laboratory
**Creatine Kinase, High**	3 to < 6 x ULN	6 to < 10 x ULN	10 to < 20 x ULN	≥ 20 x ULN
**Creatinine, High**	1.1 to 1.3 x ULN	> 1.3 to 1.8 x ULN OR Increase of > 0.3 mg/dL above baseline	> 1.8 to < 3.5 x ULN OR Increase of 1.5 to < 2.0 x above baseline	≥ 3.5 x ULN OR Increase of ≥ 2.0 x above baseline
**Creatinine Clearance[15] or eGFR, Low** *Report only one*	NA	< 90 to 60 ml/min or ml/min/1.73 m^2 OR 10 to < 30% decrease from baseline	< 60 to 30 ml/min or ml/min/1.73 m^2 OR ≥ 30 to < 50% decrease from baseline	< 30 ml/min or ml/min/1.73 m^2 OR ≥ 50% decrease from baseline or dialysis needed
**Glucose** (mg/dL; *mmol/L*) ***Fasting, High***	110 to 125 *6.11 to < 6.95*	> 125 to 250 *6.95 to < 13.89*	> 250 to 500 *13.89 to < 27.75*	> 500 *≥ 27.75*
Nonfasting, High	116 to 160 *6.44 to < 8.89*	> 160 to 250 *8.89 to < 13.89*	> 250 to 500 *13.89 to < 27.75*	> 500 *≥ 27.75*
**Glucose, Low** (mg/dL; *mmol/L*) *≥ 1 month of age*	55 to 64 *3.05 to 3.55*	40 to < 55 *2.22 to < 3.05*	30 to < 40 *1.67 to < 2.22*	< 30 *< 1.67*
< 1 month of age	50 to 54 *2.78 to 3.00*	40 to < 50 *2.22 to < 2.78*	30 to < 40 *1.67 to < 2.22*	< 30 *< 1.67*
**Lactate, High**	ULN to < 2.0 x ULN without acidosis	≥ 2.0 x ULN without acidosis	Increased lactate with pH < 7.3 without life-threatening consequences	Increased lactate with pH < 7.3 with life-threatening consequences

15 Use the applicable formula (i.e., Cockroft-Gault in mL/min or Schwatrz in mL/min/1.73m^2).

PARAMETER	GRADE 1<br>MILD	GRADE 2<br>MODERATE	GRADE 3<br>SEVERE	GRADE 4<br>POTENTIALLY LIFE-THREATENING
**Lipase, High**	1.1 to < 1.5 x ULN	1.5 to < 3.0 x ULN	3.0 to < 5.0 x ULN	≥ 5.0 x ULN
**Lipid Disorders** (mg/dL; *mmol/L*)<br>***Cholesterol, Fasting, High***<br>*≥18 years of age*	200 to < 240<br>*5.18 to < 6.19*	240 to < 300<br>*6.19 to < 7.77*	≥ 300<br>*≥ 7.77*	NA
< 18 years of age	170 to < 200<br>*4.40 to < 5.15*	200 to < 300<br>*5.15 to < 7.77*	≥ 300<br>*≥ 7.77*	NA
LDL, Fasting, High<br>*≥18 years of age*	130 to < 160<br>*3.37 to < 4.12*	160 to < 190<br>*4.12 to < 4.90*	≥ 190<br>*≥ 4.90*	NA
> 2 to < 18 years of age	110 to < 130<br>*2.85 to < 3.34*	130 to < 190<br>*3.34 to < 4.90*	≥ 190<br>*≥ 4.90*	NA
Triglycerides, Fasting, High	150 to 300<br>*1.71 to 3.42*	>300 to 500<br>*>3.42 to 5.7*	>500 to < 1,000<br>*>5.7 to 11.4*	> 1,000<br>*> 11.4*
**Magnesium[16], Low**<br>(mEq/L; *mmol/L*)	1.2 to < 1.4<br>*0.60 to < 0.70*	0.9 to < 1.2<br>*0.45 to < 0.60*	0.6 to < 0.9<br>*0.30 to < 0.45*	< 0.6<br>*< 0.30*
**Phosphate, Low** (mg/dL; *mmol/L*)<br>*> 14 years of age*	2.0 to < LLN<br>*0.81 to < LLN*	1.4 to < 2.0<br>*0.65 to < 0.81*	1.0 to < 1.4<br>*0.32 to < 0.65*	< 1.0<br>*< 0.32*
1 to 14 years of age	3.0 to < 3.5<br>*0.97 to < 1.13*	2.5 to < 3.0<br>*0.81 to < 0.97*	1.5 to < 2.5<br>*0.48 to < 0.81*	< 1.5<br>*< 0.48*
< 1 year of age	3.5 to < 4.5<br>*1.13 to < 1.45*	2.5 to < 3.5<br>*0.81 to < 1.13*	1.5 to < 2.5<br>*0.48 to < 0.81*	< 1.5<br>*< 0.48*
**Potassium, High**<br>(mEq/L; *mmol/L*)	5.6 to < 6.0<br>*5.6 to < 6.0*	6.0 to < 6.5<br>*6.0 to < 6.5*	6.5 to < 7.0<br>*6.5 to < 7.0*	≥ 7.0<br>*≥ 7.0*
**Potassium, Low**<br>(mEq/L; *mmol/L*)	3.0 to < 3.4<br>*3.0 to < 3.4*	2.5 to < 3.0<br>*2.5 to < 3.0*	2.0 to < 2.5<br>*2.0 to < 2.5*	< 2.0<br>*< 2.0*
**Sodium, High**<br>(mEq/L; *mmol/L*)	146 to < 150<br>*146 to < 150*	150 to < 154<br>*150 to < 154*	154 to < 160<br>*154 to < 160*	≥ 160<br>*≥ 160*
**Sodium, Low**<br>(mEq/L; *mmol/L*)	130 to < 135<br>*130 to < 135*	125 to < 130<br>*125 to < 135*	121 to < 125<br>*121 to < 125*	≤ 120<br>*≤ 120*
**Uric Acid, High**<br>(mg/dL; *mmol/L*)	7.5 to < 10.0<br>*0.45 to < 0.59*	10.0 to < 12.0<br>*0.59 to < 0.71*	12.0 to < 15.0<br>*0.71 to < 0.89*	≥ 15.0<br>*≥ 0.89*

16 To convert a magnesium value from mg/dL to mmol/L, laboratories should multiply by 0.4114.

Hematology

PARAMETER	GRADE 1<br>MILD	GRADE 2<br>MODERATE	GRADE 3<br>SEVERE	GRADE 4<br>POTENTIALLY LIFE-THREATENING
**Absolute CD4+ Count, Low**<br>(cell/mm^3; *cells/L*)<br>*> 5 years of age*<br>*(not HIV infected)*	300 to < 400<br>*300 to < 400*	200 to < 300<br>*200 to < 300*	100 to < 200<br>*100 to < 200*	< 100<br>*< 100*
**Absolute Lymphocyte Count, Low**<br>(cell/mm^3; *cells/L*)<br>*> 5 years of age*<br>*(not HIV infected)*	600 to < 650<br>*0.600 x 10^9 to < 0.650 x 10^9*	500 to < 600<br>*0.500 x 10^9 to < 0.600 x 10^9*	350 to < 500<br>*0.350 x 10^9 to < 0.500 x 10^9*	< 350<br>*< 0.350 x 10^9*
**Absolute Neutrophil Count (ANC), Low**<br>(cells/mm^3; *cells/L*)<br>*> 7 days of age*	800 to 1,000<br>*0.800 x 10^9 to 1.000 x 10^9*	600 to 799<br>*0.600 x 10^9 to 0.799 x 10^9*	400 to 599<br>*0.400 x 10^9 to 0.599 x 10^9*	< 400<br>*< 0.400 x 10^9*
2 to 7 days of age	1,250 to 1,500<br>*1.250 x 10^9 to 1.500 x 10^9*	1,000 to 1,249<br>*1.000 x 10^9 to 1.249 x 10^9*	750 to 999<br>*0.750 x 10^9 to 0.999 x 10^9*	< 750<br>*< 0.750 x 10^9*
≤ 1 day of age	4,000 to 5,000<br>*4.000 x 10^9 to 5.000 x 10^9*	3,000 to 3,999<br>*3.000 x 10^9 to 3.999 x 10^9*	1,500 to 2,999<br>*1.500 x 10^9 to 2.999 x 10^9*	< 1,500<br>*< 1.500 x 10^9*
**Fibrinogen, Decreased**<br>(mg/dL; *g/L*)	100 to < 200<br>*1.00 to < 2.00*<br>OR<br>0.75 to < 1.00 x LLN	75 to < 100<br>*0.75 to < 1.00*<br>OR<br>≥ 0.50 to < 0.75 x LLN	50 to < 75<br>*0.50 to < 0.75*<br>OR<br>0.25 to < 0.50 x LLN	< 50<br>*< 0.50*<br>OR<br>< 0.25 x LLN<br>OR Associated with gross bleeding
**Hemoglobin[17], Low** (g/dL; *mmol/L*)[18]<br>*≥ 13 years of age*<br>*(male only)*	10.0 to 10.9<br>*6.19 to 6.76*	9.0 to < 10.0<br>*5.57 to < 6.19*	7.0 to < 9.0<br>*4.34 to < 5.57*	< 7.0<br>*< 4.34*
≥ 13 years of age<br>*(female only)*	9.5 to 10.4<br>*5.88 to 6.48*	8.5 to < 9.5<br>*5.25 to < 5.88*	6.5 to < 8.5<br>*4.03 to < 5.25*	< 6.5<br>*< 4.03*

[17] Male and female sex are defined as sex at birth.
[18] The conversion factor used to convert g/dL to mmol/L is 0.6206 and is the most commonly used conversion factor. For grading hemoglobin results obtained by an analytic method with a conversion factor other than 0.6206, the result must be converted to g/dL using the appropriate conversion factor for the particular laboratory.

PARAMETER	GRADE 1<br>MILD	GRADE 2<br>MODERATE	GRADE 3<br>SEVERE	GRADE 4<br>POTENTIALLY LIFE-THREATENING

57 days of age to < 13 years of age (male and female)	9.5 to 10.4<br>*5.88 to 6.48*	8.5 to < 9.5<br>*5.25 to < 5.88*	6.5 to < 8.5<br>*4.03 to < 5.25*	< 6.5<br>*< 4.03*
36 to 56 days of age (male and female)	8.5 to 9.6<br>*5.26 to 5.99*	7.0 to < 8.5<br>*4.32 to < 5.26*	6.0 to < 7.0<br>*3.72 to < 4.32*	< 6.0<br>*< 3.72*
22 to 35 days of age (male and female)	9.5 to 11.0<br>*5.88 to 6.86*	8.0 to < 9.5<br>*4.94 to < 5.88*	6.7 to < 8.0<br>*4.15 to < 4.94*	< 6.7<br>*< 4.15*
8 to < 21 days of age (male and female)	11.0 to 13.0<br>*6.81 to 8.10*	9.0 to < 11.0<br>*5.57 to < 6.81*	8.0 to < 9.0<br>*4.96 to < 5.57*	< 8.0<br>*< 4.96*
< 7 days of age (male and female)	13.0 to 14.0<br>*8.05 to 8.72*	10.0 to < 13.0<br>*6.19 to < 8.05*	9.0 to < 10.0<br>*5.59 to < 6.19*	< 9.0<br>*< 5.59*
**INR, High** (not on anticoagulation therapy)	1.1 to < 1.5 x ULN	1.5 to < 2.0 x ULN	2.0 to < 3.0 x ULN	≥ 3.0 x ULN
**Methemoglobin** (% hemoglobin)	5.0 to < 10.0%	10.0 to < 15.0%	15.0 to < 20.0%	≥ 20.0%
**PTT, High** (not on anticoagulation therapy)	1.1 to < 1.66 x ULN	1.66 to < 2.33 x ULN	2.33 to < 3.00 x ULN	≥ 3.00 x ULN
**Platelets, Decreased** (cells/mm^3; *cells/L*)	100,000 to < 124,999<br>*100.000 x 10^9 to < 124.999 x 10^9*	50,000 to < 100,000<br>*50.000 x 10^9 to < 100.000 x 10^9*	25,000 to < 50,000<br>*25.000 x 10^9 to < 50.000 x 10^9*	< 25,000<br>*< 25.000 x 10^9*
**PT, High** (not on anticoagulation therapy	1.1 to < 1.25 x ULN	1.25 to < 1.50 x ULN	1.50 to < 3.00 x ULN	≥ 3.00 x ULN
**WBC, Decreased** (cells/mm^3; *cells/L*)<br>*> 7 days of age*	2,000 to 2,499<br>*2.000 x 10^9 to 2.499 x 10^9*	1,500 to 1,999<br>*1.500 x 10^9 to 1.999 x 10^9*	1,000 to 1,499<br>*1.000 x 10^9 to 1.499 x 10^9*	< 1,000<br>*< 1.000 x 10^9*
< 7 days of age	5,500 to 6,999<br>*5.500 x 10^9 to 6.999 x 10^9*	4,000 to 5,499<br>*4.000 x 10^9 to 5.499 x 10^9*	2,500 to 3,999<br>*2.500 x 10^9 to 3.999 x 10^9*	< 2,500<br>*< 2.500 x 10^9*

Urinalysis

PARAMETER	GRADE 1	GRADE 2	GRADE 3	GRADE 4
**Glycosuria (**random collection tested by dipstick)	Trace to 1+ or ≤ 250 mg	2+ or > 250 to < 500 mg	> 2+ or > 500 mg	NA
**Hematuria** (not to be reported based on dipstick findings or on blood believed to be of menstrual origin)	6 to < 10 RBCs per high power field	≥ 10 RBCs per high power field	Gross, with or without clots OR<br>With RBC casts OR<br>Intervention indicated	Life-threatening consequences
**Proteinuria** (random collection tested by dipstick)	1+	2+	3+ or higher	NA

Page **82** of **83**

References

1 (UNAIDS)., J. U. N. P. o. H. A. The Gap Report., (UNAIDS, 2014).
2 UNAIDS. AIDS by the numbers 2015. (2015).
3 CDC. CDC. Vital Signs: HIV Diagnosis, Care, and Treatment Among Persons Living with HIV- United States 2011. *MMWR* **4**, 1-6 (2014).
4 Jardine, J. *et al.* Rational HIV immunogen design to target specific germline B cell receptors. *Science* **340**, 711-716, doi:10.1126/science.1234150 (2013).
5 Sok, D. *et al.* Promiscuous glycan site recognition by antibodies to the high-mannose patch of gp120 broadens neutralization of HIV. *Sci Transl Med* **6**, 236ra263, doi:10.1126/scitranslmed.3008104 (2014).
6 Caskey, M. *et al.* Viraemia suppressed in HIV-1-infected humans by broadly neutralizing antibody 3BNC117. *Nature* **522**, 487-491, doi:10.1038/nature14411 (2015).
7 Barouch, D. H. *et al.* Therapeutic efficacy of potent neutralizing HIV-1-specific monoclonal antibodies in SHIV-infected rhesus monkeys. *Nature* **503**, 224-228, doi:10.1038/nature12744 (2013).
8 Hessell, A. J. *et al.* Broadly neutralizing human anti-HIV antibody 2G12 is effective in protection against mucosal SHIV challenge even at low serum neutralizing titers. *PLoS Pathog* **5**, e1000433, doi:10.1371/journal.ppat.1000433 (2009).
9 Hessell, A. J. *et al.* Effective, low-titer antibody protection against low-dose repeated mucosal SHIV challenge in macaques. *Nat Med* **15**, 951-954, doi:10.1038/nm.1974 (2009).
10 Moldt, B. *et al.* Highly potent HIV-specific antibody neutralization in vitro translates into effective protection against mucosal SHIV challenge in vivo. *Proceedings of the National Academy of Sciences of the United States of America* **109**, 18921-18925, doi:10.1073/pnas.1214785109 (2012).
11 Walker, L. M. *et al.* Broad neutralization coverage of HIV by multiple highly potent antibodies. *Nature* **477**, 466-470, doi:10.1038/nature10373 (2011).
12 Haynes, B. F. & McElrath, M. J. Progress in HIV-1 vaccine development. *Curr Opin HIV AIDS* **8**, 326-332, doi:10.1097/COH.0b013e328361d178 (2013).
13 Burton, D. R. & Mascola, J. R. Antibody responses to envelope glycoproteins in HIV-1 infection. *Nat Immunol* **16**, 571-576, doi:10.1038/ni.3158 (2015).
14 Sok, D. *et al.* Recombinant HIV envelope trimer selects for quaternary-dependent antibodies targeting the trimer apex. *Proc Natl Acad Sci U S A* **111**, 17624-17629, doi:10.1073/pnas.1415789111 (2014).
15 Scheid, J. F. *et al.* Sequence and structural convergence of broad and potent HIV antibodies that mimic CD4 binding. *Science* **333**, 1633-1637, doi:10.1126/science.1207227 (2011).
16 Shingai, M. *et al.* Passive transfer of modest titers of potent and broadly neutralizing anti-HIV monoclonal antibodies block SHIV infection in macaques. *J Exp Med* **211**, 2061-2074, doi:10.1084/jem.20132494 (2014).
17 Lynch, R. M. *et al.* Virologic effects of broadly neutralizing antibody VRC01 administration during chronic HIV-1 infection. *Sci Transl Med* **7**, 319ra206, doi:10.1126/scitranslmed.aad5752 (2015).
18 Andrade, A. *et al.* Three distinct phases of HIV-1 RNA decay in treatment-naive patients receiving raltegravir-based antiretroviral therapy: ACTG A5248. *J Infect Dis* **208**, 884-891, doi:10.1093/infdis/jit272 (2013).

International AIDS Vaccine Initiative

DOCUMENT NUMBER:

DOCUMENT TITLE:

DOCUMENT NOTES:

Document Information

Revision:	Vault:
Status:	Document Type:

Date Information

Effective Date:	Expiration Date:
Release Date:	Next Review Date:

Control Information

Author:	Owner:
Previous Number:	Change Number:

Signature Manifest

Document Number: TMF-02-0166 **Revision:** 2

Title: Protocol PGT121

All dates and times are in Eastern Time Zone.

T001 Protocol

Change Request Approval

Name/Signature	Title	Date	Meaning/Reason
Katherine Crisafi (KCRISAFI)			
Michele Fong Lim (MFONGLIM)			
Dani Vooijs (DVOOIJS)	Sr. Manager, Clinical Programs	12 Oct 2016, 03:47:40 AM	Approved

CMO Approval

Name/Signature	Title	Date	Meaning/Reason
Frances Priddy (FPRIDDY)	Chief Medical Officer	18 Oct 2016, 12:08:46 AM	Approved

QA Final Release

Name/Signature	Title	Date	Meaning/Reason
Dani Vooijs (DVOOIJS)			
Katherine Crisafi (KCRISAFI)			
Michele Fong Lim (MFONGLIM)	Director Quality Systems	18 Oct 2016, 08:08:16 AM	Approved

Notify

Name/Signature	Title	Date	Meaning/Reason
Lisa Sunner (LSUNNER)		18 Oct 2016, 08:08:16 AM	Email Sent

**Protocol Title:**	A Phase 1 Randomized Placebo-controlled Clinical Trial of the Safety, Pharmacokinetics and Antiviral Activity of PGT121 Monoclonal Antibody (mAb) in HIV-uninfected and HIV-infected Adults
**Protocol Number:**	IAVI T001
**Phase:**	Phase 1
**Regulatory Investigational Product Number:**	New IND submission
**Sponsor:**	International AIDS Vaccine Initiative (IAVI)<br>125 Broad Street, 9th Floor<br>New York, New York 10004<br>USA
**Sponsor Status**	Not for-Profit Organization
**Date of Protocol Version:**	23 November 2016<br>04.0<br>Edits from FDA<br><br>17 October 2016<br>03.0<br>Edits from IRB comments<br><br>09 September 2016<br>02.0<br>Revision to IRB Submission<br><br>05 August 2016<br>01.0<br>IRB Submission

THE CONFIDENTIAL INFORMATION IN THIS DOCUMENT IS PROVIDED TO YOU AS AN INVESTIGATOR, POTENTIAL INVESTIGATOR, OR CONSULTANT, FOR REVIEW BY YOU, YOUR STAFF, AND APPLICABLE INSTITUTIONAL REVIEW BOARDS (IRBS) AND/OR INDEPENDENT ETHICS COMMITTEES (IECS). IT IS UNDERSTOOD THAT THE INFORMATION WILL NOT BE DISCLOSED TO OTHERS, EXCEPT TO THE EXTENT NECESSARY TO OBTAIN ETHICAL AND REGULATORY APPROVAL FROM THE RESPECTIVE COMMITTEE'S AGENCIES AND INFORMED CONSENT FROM THOSE PERSONS TO WHOM THE INVESTIGATIONAL PRODUCT MAY BE ADMINISTERED.

SYNOPSIS

**TITLE**	A Phase 1 Randomized Placebo-controlled Clinical Trial of the Safety, Pharmacokinetics and Antiviral Activity of PGT121 Monoclonal Antibody (mAb) in HIV-uninfected and HIV-infected Adults
**PROTOCOL NUMBER**	IAVI T001
**PHASE**	Phase 1
**SPONSOR**	International AIDS Vaccine Initiative (IAVI)<br>125 Broad Street, 9th Floor<br>New York, New York 10004,<br>USA
**SPONSOR STATUS**	Not for Profit Organization
**STUDY PRODUCTS**	PGT121 monoclonal antibody (mAb)
**OBJECTIVES**	**Primary Objectives**<br>• To evaluate the safety and tolerability of a single intravenous (IV) infusion of PGT121 mAb at 3, 10, and 30 mg/kg in HIV-uninfected adults and HIV-infected adults<br>• To evaluate the pharmacokinetic (PK) profile of IV infusion of PGT121 mAb in HIV-uninfected and HIV-infected adults<br>• To evaluate the antiviral activity of IV infusion of PGT121 mAb in HIV-infected adults not on ART<br><br>**Secondary Objective**<br>• To determine if PGT121 induces anti-PGT121 antibodies<br>• To determine the effect of PGT121 mAb on CD4+ T cell counts in HIV-infected adults<br>• To determine if PGT121 mAb induces viral escape mutants in viremic HIV-infected adults not on ART<br><br>**Exploratory Objectives**<br>• To determine if PGT121 mAb has any impact on the host immune responses (i.e., HIV-specific cellular and humoral immune response)<br>• To determine the effect of PGT121 mAb on the size of the latent HIV reservoir in HIV-infected adults<br>• To determine PGT121 mAb levels in mucosal secretions in HIV-uninfected and HIV-infected adults<br>• To measure in vitro neutralization of HIV isolates with participant's serum post PGT121 mAb IV infusion<br>• To determine if PGT121 mAb has any impact on resistance mutations to ARVs

**ENDPOINTS**	***Primary***:<br><br>*Safety and Tolerability:*<br>We will measure the following endpoints to evaluate the safety and tolerability of PGT121 mAb in HIV-uninfected and HIV-infected adults:<br><br>1. Proportion of participants with moderate or greater reactogenicity (e.g., solicited adverse events) for 3 days following IV infusion of PGT121 mAb.<br>2. Proportion of participants with moderate or greater and/or PGT121 mAb-related unsolicited adverse events (AEs), including safety laboratory (biochemical, hematological) parameters, following IV infusion of PGT121 mAb for the first 56 days post administration of Investigational Product.<br>3. Proportion of participants with PGT121 mAb-related serious adverse events (SAEs) throughout the study period.<br><br>*Pharmacokinetics:*<br>We will measure the following endpoints to determine the pharmacokinetics following IV infusion of PGT121 mAb in HIV-uninfected and HIV-infected adults:<br><br>1. Elimination half-life (t1/2)<br>2. Clearance (CL/F)<br>3. Volume of distribution (Vz/F)<br>4. Area under the concentration decay curve (AUC)<br>5. Impact of viral load and/or ART on PGT121 disposition (elimination half-life (t1/2), clearance (CL/F), volume of distribution (Vz/F), total exposure<br><br>*Antiviral activity:*<br>We will calculate the following endpoints to determine the antiviral activity following IV infusion of PGT121 mAb in viremic HIV-infected adults not on ART:<br><br>1. Change in plasma HIV-1 RNA levels from baseline (mean of pre-entry and entry values)<br><br>***Secondary***:<br><br>*Anti-PGT121 antibodies:*<br>We will calculate the following endpoint to determine if anti-PGT121 antibodies are elicited following IV infusion of PGT121 mAb in HIV-uninfected and HIV-infected adults:<br><br>1. Change in serum anti-PGT121 antibody titers from baseline<br><br>*CD4+ T cell count:*<br>We will calculate the following endpoint to determine if IV infusion of PGT121 mAb has any impact on CD4+ T cell counts in HIV-infected

adults:

1. Change in CD4+ T cell count and frequency compared to baseline as measured by single platform flow cytometry

HIV genotyping/phenotyping of circulating virus for evaluation of PGT121 mAb -induced escape mutations:
We will compare plasma virus sequences before and after IV infusion of PGT121 mAb to determine if PGT121 mAb induces viral escape mutations in viremic HIV-infected adults not on ART.

1. Development of sequence variations in epitopes known to result in reduced PGT121 mAb neutralization susceptibility.

Exploratory:

Additional assessments may include, but are not limited to, the following: HIV-specific IgG/IgA binding responses by ELISA, HIV-specific cellular immune responses by ELISPOT, HIV-specific antibody function by ADCC, ADCP, and ADCVI assays, resistance mutations to current ARVs, PGT121 mAb levels in mucosal secretions, changes in total HIV-1 DNA and 2-long terminal repeat (LTR) circular HIV-1 DNA in resting or total CD4 T cells and in vitro neutralization of HIV isolates with participant's serum post IV infusion of PGT121 mAb.

STUDY DESIGN TABLE

	Group	Participants	Sub-Group	Regimen	N	Dose (mg/kg)
Part 1[1]	1[3]	HIV-uninfected participants	1A	PGT121/Placebo	4/1 (6/2 if DLT[2])	3
			1B	PGT121/Placebo	4/1 (6/2 if DLT)	10
			1C	PGT121/Placebo	4/1 (6/2 if DLT)	30
	2[3]	HIV-infected on ART, (<50 cp/ml)	2A	PGT121/Placebo	4/1 (6/2 if DLT[2])	3
			2B	PGT121/Placebo	4/1 (6/2 if DLT)	10
			2C	PGT121/Placebo	4/1 (6/2 if DLT)	30
Safety Monitoring Committee Review[4]						
Part 2	3[5]	HIV-Infected off ART (VL $2x10^3$ – $1x10^5$cp/ml)	3A[6]	PGT121	6 (max 9)	30
			3B	PGT121	8 (max 12)	10
			3C	PGT121	10 (max 15)	3
		HIV-Infected off ART (VL $1x10^2$ – $2x10^3$ cp/ml)	3D[7]	PGT121	3	30
			3E	PGT121	3	10
			3F	PGT121	3	3

DLT, dose limiting toxicity; ART, antiretroviral therapy; cp, copies; ml, milliliter
Administration of PGT 121 will be by intravenous infusion (IV)

1. Eligible participants for Groups 1 and 2 will be enrolled according to their HIV-serostatus and will occur in parallel. At each dose level in Part 1, investigational product (IP) administration will be separated by at least 4 days for each of the first 3 participants. Randomization will ensure at least 2 participants receive active product and are observed for at least 4 days before administration to additional participants.
2. A dose limiting toxicity (DLT) will be defined as 1) any Grade 3 or greater reactogenicity or adverse event judged by the study investigators as at least possibly related to investigational product or 2) any SAE considered at least possibly related to investigational product.
3. Within each group, the PSRT will review data. If no DLT occurs within 2 weeks from infusion of the 5 participants in a dose group, dose escalation to the next dose group will proceed. If 1 DLT occurs, 3 additional participants will be enrolled; randomized between PGT121 mAb and placebo at a 2:1 ratio. If no additional DLTs occur within 2 weeks of infusion in the 8 total participants (3:1 ratio of PGT121 mAb to placebo), study can proceed with enrolment of the next dose group. If 2 or more DLTs accumulate that are the same, similar, or in the same System Organ Class, dosing will be halted and the next lower dose level will be declared the maximum tolerated dose (MTD). If no DLT occurs in the final dose group, MTD will be the highest dose given (subgroups 1C and 2C 30mg/kg) after 14 days of follow-up.
4. Following IP administration of the last participant in both Groups 1 and 2, an independent Safety Monitoring Committee (SMC) will review at least the first 14 days of safety data to confirm MTD in each group, and determine whether, and at what dose, Group 3 can initiate enrollment.
5. Group 3 will start with the MTD as determined in Part 1. Group 3 will start with subgroups 3A and

	3D if the MTD is 30mg/kg, subgroups 3B and 3E if the MTD is 10mg/kg and subgroups 3C and 3F if the MTD is 3mg/kg.<br>6. If subgroup 3A achieves a mean decline in HIV RNA of ≥0.9 log compared to baseline, enrolment into subgroup 3A will be stopped and the next lower dose subgroup 3B will be initiated. The same rule will apply for progression from subgroup 3B to 3C. At least 6, 8 and 10 but up to 9, 12 and 15 participants, will be enrolled in subgroups 3A, 3B, and 3C respectively, until the minimum effective dose is determined. If a mean decline ≥0.9 log in HIV RNA is not achieved, enrollment will be stopped at the completion of enrolment at that dose level.<br>7. As soon as subgroup 3D has enrolled 3 participants, enrolment into subgroup 3E will be initiated. The same rule will apply for progression from subgroup 3E to 3F. In the event that dose de-escalation in subgroups 3A-C is stopped due to failure to meet virologic endpoints, dose de-escalation in subgroups 3D-F will continue.
**METHODS**	See Schedule of Procedures, Appendix A
**STUDY POPULATION**	The study population will include three different groups:<br>Group 1 will include HIV-uninfected males or females aged 18-50 years old who are willing to maintain low risk behavior for HIV infection; principal exclusion criteria include confirmed HIV-infection, pregnancy or lactation, significant acute or chronic disease and clinically significant laboratory abnormalities. Group 2 will include HIV-infected males or females aged 18-50 years old on a stable antiretroviral regimen with HIV-1 RNA plasma level <50 copies/ml, CD4 cell count > 300 cells/uL and CD4 nadir > 200 cell/uL; principal exclusion criteria include history of AIDS-defining illness, significant acute or chronic medical condition other than HIV infection, and clinically significant laboratory abnormalities. Group 3 will include HIV-infected males or females aged 18-50 years old, not on antiretroviral therapy for > 6 month with detectable HIV-1 RNA plasma level between 100 and 100,000 copies/ml, CD4 cell count > 300 cells/uL and CD4 nadir > 200 cell/uL; principal exclusion criteria include history of AIDS-defining illness, significant acute or chronic medical condition other than HIV infection, and clinically significant laboratory abnormalities.
**NUMBER OF PARTICIPANTS**	63-93 participants will be included.
**DOSE ESCALATION and PAUSE RULES**	The first part of this study is a dose-escalation trial in HIV-uninfected adults and HIV-infected adults on ART with suppressed viral load, as indicated in the study design table.<br><br>If 2 or more DLTs occur that are the same, similar, or in the same System Organ Class, dosing will be halted and the next lower dose level will be declared the maximum tolerated dose (MTD) within this group. If no DLT occurs in the final dose group, MTD will be the highest dose given (subgroups 1C and 2C 30mg/kg) after 14 days of follow-up.<br><br>The Protocol Safety Review Team (PSRT) will review safety data through at least day 14 post-IP administration for all participants in the 1st dose group (subgroups 1A and 2A) prior to allowing enrolment of participants into the 2nd dose group (subgroups 1B and 2B). The PSRT will review safety data through at least day 14 post-IP administration for all participants in the 2nd dose group (subgroups 1B and 2B) prior to allowing enrolment of participants into the 3rd dose group (subgroups 1C and 2C).

	Following IP administration of the last participant in both Groups 1 and 2, an independent Safety Monitoring Committee (SMC) will review safety data through at least day 14 post-IP administration for all participants to confirm MTD, and determine whether, and at what dose, Group 3 can initiate enrollment.<br><br>The second part of this study is a dose-de-escalation trial in HIV-infected adults not on ART, as indicated in the study design table.<br><br>The study will be paused for a safety review by the investigators and the independent SMC if 1) 1 or more participants experiences a Serious Adverse Event that is judged possibly, probably or definitely related to the IP, 2) There is a participant death, regardless of relationship to the IP, 3) if 2 or more participants experience grade 3 adverse events in the same System Organ Class that are considered to be at least possibly related to IP or 4) any grade 4 adverse event. See protocol section 17.3.
**FORMULATIONS, VOLUMES AND ROUTES OF ADMINISTRATION**	**PGT121 mAb**: PGT121 mAb is a recombinant, fully human monoclonal antibody (mAb) of the IgG1 isotype that binds to the HIV envelope. The concentration and volume of product in each vial is 50 mg/mL, 6mL in each vial. PGT121 mAb will be given intravenously in this study.
**DURATION OF STUDY PARTICIPATION**	Participants will be screened up to 42 days before IP administration and will be followed for 24 weeks. The anticipated study duration for each participant is approximately 6 months from screening through last study visit. It is anticipated that it will take approximately 4.5 months to enroll Groups 1 and 2. It is anticipated that it will take approximately 8 months to enroll the entire study. The exact duration depends on the recruitment rate and how many participants will be required per dose group.
**RANDOMIZATION and BLINDING**	This is a randomized, placebo-controlled study for groups 1 and 2, and open label for group 3 who will not receive placebo.
**EVALUATION FOR INTERCURRENT HIV INFECTION:**	Participants in Group 1 (HIV-uninfected) will be tested for HIV according to the Schedule of Procedures. Test results will be interpreted according to a pre-determined diagnostic algorithm. HIV testing at additional time points may be performed upon the request of the participant and Principal Investigator or designee as medical or social circumstances warrant.
**SAFETY MONITORING AND STATISTICAL CONSIDE-RATIONS:**	All clinical trial data collected, identified only by a study identification number, will be entered into the clinical trial database.<br><br>Safety will continually be monitored by the Investigators, the Sponsor's Medical Monitor and a Protocol Safety Review Team (PSRT); detailed pause criteria are pre-defined.<br><br>Safety data will be reviewed by an independent Safety Monitoring Committee (SMC). *Ad hoc* safety review may be specifically requested by the Sponsor, the Principal Investigators, Ethics Committees, Regulatory Authorities, or by the SMC. All clinical and routine laboratory data will be included in the safety analysis. At the end of the study, a full analysis will be prepared.

TABLE OF CONTENTS

ABBREVIATIONS 11

CONTACT INFORMATION 12

1.0 SIGNATURE PAGE 13

2.0 INTRODUCTION AND BACKGROUND INFORMATION 14

2.1 Study Rationale 14
2.2 Experience with PGT121 15

3.0 STUDY OBJECTIVES 15

3.1 Primary Objectives 15
3.2 Secondary Objectives 15
3.3 Exploratory Objectives: 15

4.0 STUDY ENDPOINTS 15

4.1 Study Endpoints 15
4.1.1 Primary Endpoints 15
4.1.2 Secondary Endpoints 16
4.1.3 Exploratory Endpoints 17

5.0 STUDY DESIGN 17

5.1 Definition of Dose Limiting Toxicity and Maximum Tolerated Dose 17
5.2 Dose Escalation – Groups 1 and 2: Determination of Maximum Tolerated Dose 17
5.3 Dose De-Escalation- Group 3: Determination of Minimum Effective Dose 18
5.4 Duration of the Study 20
5.5 Study Population 20
5.6 Inclusion Criteria 20
5.7 Exclusion Criteria 22
5.8 Recruitment of Participants 26

6.0 STUDY VISITS 26

6.1 Screening Period 26
6.2 IV infusion of PGT121 mAb Visit 27
6.3 Post-IV infusion of PGT121 mAb Visits 28
6.4 Additional Follow-up Visits 28
6.5 Unscheduled Visits 28
6.6 Final Study Visit or Early Termination Visit 28

7.0 STUDY PROCEDURES 28

7.1 Informed Consent Process 28
7.2 Medical History and Physical Examination 29
7.3 HIV Testing and HIV-test Counselling (Group 1) 29
7.4 HIV Risk Reduction Counselling 30
7.5 Family Planning Counselling 30
7.6 ART Counselling (Group 3) 30
7.7 Specimens 30
7.8 Reimbursement 30
7.9 Randomization and Blinding 31
7.10 Un-blinding Procedure for Individual Participants 31
7.11 Assessment of IP related HIV sero-positivity 31

8.0 INVESTIGATIONAL PRODUCT 32
8.1 Description 32
8.2 Shipment and Storage 32
8.3 Preparation of Investigational Product (IP) 33
8.4 Administration of Investigational Product 33
8.5 Accountability and Disposal of Investigational Product 33
9.0 ASSESSMENTS 33
9.1 Safety Assessments 33
9.1.1 Local reactogenicity 34
9.1.2 Systemic reactogenicity 34
9.1.3 Vital Signs 34
9.1.4 Other Adverse Events 34
9.1.5 Concomitant Medications 34
9.1.6 Routine laboratory parameters 34
9.1.7 Specific screening tests: 35
9.2 Virologic Assessments 35
9.3 Exploratory Immunogenicity Assessments 36
9.3.1 Antibody Responses 36
9.3.2 Cellular Responses 36
9.3.3 PBMC, Serum and Plasma Storage 36
9.4 Other Assessments 36
9.4.1 HIV Antibody Testing 37
9.4.2 Pharmacokinetics 37
9.4.3 HLA Typing 37
9.4.5 Pregnancy Test 37
9.4.6 HIV Risk Assessment (Group 1) 37
9.4.7 Social Impact Assessment 38
10.0 ADVERSE EVENTS 38
10.1 Definition 38
10.2 Assessment of Severity of Adverse Events 38
10.3 Relationship to Investigational Product 38
10.4 Serious Adverse Events 39
10.5 Reporting Potential Immune-Mediated Diseases 40
10.6 Clinical Management of Adverse Events 41
10.7 Pregnancy 42
10.8 Intercurrent HIV Infection (Group 1) 42
11.0 MANAGEMENT OF HIV ISSUES DURING AND FOLLOWING STUDY 42
11.1 HIV Testing – Group 1 42
11.2 Social Discrimination as a Result of IP-related antibodies 43
11.3 HIV infection – Group 1 43
11.3.1 Counselling 43
11.3.2 Referral for Support/Care 43
12.0 WITHDRAWAL FROM STUDY 43
12.1 Deferral of IV infusion of IP 43
12.2 Withdrawal from the Study (Early Termination) 44
13.0 DATA HANDLING 44
13.1 Data Collection and Record Keeping at the Study Site 44

13.3 Data Entry at the Study Site 45
13.4 Data Analysis 45

14.0 STATISTICAL CONSIDERATIONS 45

14.1 Safety and Tolerability Analysis 45
14.2 Pharmacokinetic Analysis 46
14.3 Virologic Analysis for Dose De-escalation in Groups 3A-C 47
14.4 Analysis of Antiviral Activity and Dose De-escalation in Subgroups 3D-F 49
14.5 Secondary and Exploratory Immunologic and Virologic Analyses 49

15.0 QUALITY CONTROL AND QUALITY ASSURANCE 50

16.0 DATA AND BIOLOGICAL MATERIAL 50

17.0 ADMINISTRATIVE STRUCTURE 51

17.1 Protocol Safety Review Team 51
17.2 Safety Monitoring Committee (SMC) 51
17.2.1 Content of Interim Safety Review 51
17.2.2 SMC Review of Group 1 and 2 data prior to starting Group 3 52
17.3 Criteria for Pausing the Study 52
17.4 Study Supervision 53
17.5 Study Monitoring 54
17.6 Investigator's Records 54

18.0 INDEMNITY 54

19.0 PUBLICATION 54

20.0 ETHICAL CONSIDERATIONS 55

APPENDIX A: SCHEDULE OF PROCEDURES 56

APPENDIX B: LOW RISK CRITERIA 59

Laboratory Values 78
Chemistries 78
Hematology 81
Urinalysis 83

ABBREVIATIONS

Abbreviation	Term
**AE**	Adverse Event
**AIDS**	Acquired Immunodeficiency Syndrome
**ALT**	Alanine-Aminotransferase
**ART**	Antiretroviral Therapy
**AST**	Aspartate-Aminotransferase
**CFC**	Cytokine Flow Cytometry
**CMI**	Cell Mediated Immunity
**CRF**	Case Report Form
**CTL**	Cytotoxic T Lymphocyte
**DCC**	Data Coordinating Center
**DLT**	Dose Limiting Toxicity
**DNA**	Deoxyribonucleic Acid
**ELISA**	Enzyme Linked Immunosorbent Assay
**GCP**	Good Clinical Practice
**GFR**	Glomerular Filtration Rate
**HIV**	Human Immunodeficiency Virus
**HLA**	Human Leukocyte Antigen
**HSV**	Herpes Simplex Virus
**IAVI**	International AIDS Vaccine Initiative
**IDES**	Internet Data Entry System
**ICH**	International Council for Harmonization of Technical Requirements for Pharmaceuticals for Human Use
**IP**	Investigational Product
**IND**	Investigational New Drug Application
**IV**	Intravenous
**Kg**	Kilogram
**mAb**	Monoclonal Antibody
**mg**	Milligram
**MED**	Minimum Effective Dose
**MTD**	Maximum Tolerated Dose
**PCR**	Polymerase Chain Reaction
**PBMC**	Peripheral Blood Mononuclear Cells
**PK**	Pharmacokinetic
**RPR**	Rapid Plasma Reagin
**SAE**	Serious Adverse Event
**SIV**	Simian Immunodeficiency Virus
**SOP**	Standard Operating Procedure
**SOM**	Study Operations Manual
**SMC**	Safety Monitoring Committee
**STD**	Sexually Transmitted Disease
**TPHA**	Treponema Pallidum Hemagglutination

CONTACT INFORMATION

Detailed contact information provided in the Study Operation Manual (SOM)

**Sponsor Contact:**	
Frances Priddy MD MPH<br>Executive Director and Chief Medical Officer<br>International AIDS Vaccine Initiative<br>125 Broad Street, 9th Floor<br>New York, New York 10004	Phone: +1-212-328-7461<br>Mobile: +1-646-287-8943<br>Fax: +1-608-203-5501<br>E-mail: fpriddy@iavi.org
**Clinical Research Center Contacts:**	
Kathryn Stephenson MD MPH<br>Center for Virology and Vaccine Research<br>Clinical Trials Unit<br>Beth Israel Deaconess Medical Center<br>E / CLS – 1036<br>330 Brookline Avenue<br>Boston, Massachusetts 02215	Phone: +1-617-735-4556<br>Mobile: +1-917-836-9150<br>Fax: +1-617-735-4566<br>E-mail: kstephen@bidmc.harvard.edu

.

1.0 SIGNATURE PAGE

The signatures below constitute the approval of this protocol and the appendices and provide the necessary assurances that this study will be conducted in compliance with the protocol, Good Clinical Practices (GCP) and the applicable regulatory requirement(s).

Sponsor:

Signed: See electronic signature manifest Date:

Frances Priddy MD MPH
Executive Director and Chief Medical Officer, Medical Affairs, IAVI

Principal Investigator:

Signed: Date:

Name (please print):

Name of institution (please print):

2.0 INTRODUCTION AND BACKGROUND INFORMATION

More than 78 million people have been infected with HIV and 39 million people have died since the beginning of the AIDS epidemic[1]. In 2014, there were 1.2 million deaths attributable to HIV infection and 2 million newly infected with HIV[2]. One reason that such high rates of AIDS-related deaths continue to occur globally – despite the advent of drugs that are highly effective at suppressing HIV replication – is that only two in five people living with HIV have access to antiretroviral therapy[1]. The other reason for continued AIDS-related mortality is that ART does not cure HIV infection and must be maintained for a lifetime. Even in the United States (US), only 30% of the 1.2 million people living with HIV have suppressed HIV to undetectable levels, despite the fact that most HIV-infected people in the US have access to ART[3] It is clear that antiretroviral therapy is necessary but not sufficient to end the AIDS epidemic, both in the US and globally, and that novel efforts to prevent and treat HIV are critically needed.

2.1 Study Rationale

This is a Phase 1 study to evaluate the safety, tolerability, pharmacokinetics and anti-viral efficacy of the PGT121 monoclonal antibody for HIV prevention and therapy. PGT121 mAb is a recombinant human IgG1 monoclonal antibody that targets a V3 glycan-dependent epitope region of the HIV envelope protein[4,5]. PGT121 mAb was chosen for this study because it is potent, neutralizes a wide array of HIV viruses, and can prevent and treat simian-human immunodeficiency virus (SHIV) in rhesus monkeys.

The recent discovery of multiple potent and broadly neutralizing antibodies (bNAbs) against HIV has led to the re-emergence of the concept that antibodies may be useful for both prevention and therapy. HIV-specific antibodies that target the HIV envelope (Env) can prevent SHIV infection in rhesus monkeys and have shown to reduce HIV RNA levels in humans temporarily[6-10]. Until recently, these antibodies were few in number, targeted a narrow spectrum of HIV strains and Env epitopes, and were not potent enough for practical use. In the last five years, the field has changed dramatically: new developments in high throughput single-cell PCR-amplification and novel soluble Env baits have led to the isolation of new monoclonal antibodies with extraordinary potency and breadth[11]. These bNAbs may be effective for prevention of HIV infection when administered passively[12,13].

PGT121 mAb was selected for development because of the following critical attributes:

- PGT121 mAb is 10 to 100-fold more potent than the previous best-in-class CD4bs antibodies VRC01, VRC07, and 3BNC117 [11,14,15].
- PGT121 mAb affords superior protective efficacy against SHIV acquisition in monkeys compared to VRC01, 3BNC117, and 10-1074 [16] (and unpublished data).
- PGT121 mAb has superior therapeutic efficacy in SHIV-infected monkeys compared to VRC01, 3BNC117, and 10-1074 [7] (and unpublished data).
- PGT121 mAb may have a higher bar to escape in vivo as compared with other V3 glycan and CD4bs antibodies as a result of making multiple glycan contacts [14].
- PGT121 mAb combined with PGDM1400 (a novel bNab targeting the envelope trimer apex) neutralizes 98-99% of global HIV-1 viruses tested and has unparalleled potency with a median IC50 of 0.007 μg/ml[14].

The potency and breadth of PGT121 mAb, both alone and in combination with other bNAbs, raise the possibility that combinations may be effective for HIV prophylaxis at

low doses and against global viruses. An antibody that is effective at low doses may eventually be given subcutaneously, which would reduce the cost. It is these features that make PGT121 mAb particularly well-suited for preventing and/or treating HIV in the developing world, where it is critical that a public health intervention be low cost, easy to deliver, and effective in diverse settings.

2.2 Experience with PGT121

There is no previous clinical experience with PGT121 mAb. Several other HIV monoclonal antibodies are currently in clinical development as passive HIV immunoprophylaxis, or as potential therapeutics. Data from phase 1 studies shows acceptable preliminary safety and tolerability profiles for these products, but varying levels of anti-viral effects [6,17]. A comprehensive summary of phase 1 studies of HIV monoclonal antibodies can be found in the Investigator's Brochure.

3.0 STUDY OBJECTIVES

3.1 Primary Objectives

- To evaluate the safety and tolerability of a single intravenous (IV) infusion of PGT121 mAb at 3, 10, and 30 mg/kg in HIV-uninfected adults and HIV-infected adults.
- To evaluate the pharmacokinetic (PK) profile of IV infusion of PGT121 mAb in HIV-uninfected and HIV-infected adults.
- To evaluate the antiviral activity of IV infusion of PGT121 mAb in HIV-infected adults not on ART.

3.2 Secondary Objectives

- To determine if PGT121 mAb induces anti-PGT121 antibodies.
- To determine the effect of PGT121 mAb on CD4 T-cell counts in HIV-infected adults.
- To determine if PGT121 mAb induces viral escape mutants in viremic HIV-infected adults not on ART.

3.3 Exploratory Objectives:

- To determine if PGT121 mAb has any impact on the host immune responses (i.e., HIV-specific cellular and humoral immune response).
- To determine the effect of PGT121 mAb on the size of the latent HIV reservoir in HIV-infected adults.
- To determine PGT121 mAb levels in mucosal secretions in HIV-uninfected and HIV-infected adults.
- To measure in vitro neutralization of HIV isolates with participant's serum post PGT121 mAb IV infusion.
- To determine if PGT121 mAb has any impact on resistance mutations to ARVs.

4.0 STUDY ENDPOINTS

4.1 Study Endpoints

4.1.1 Primary Endpoints

Safety and Tolerability:

We will measure the following endpoints to evaluate the safety and tolerability of PGT121 mAb in HIV-uninfected and HIV-infected adults:

1. Proportion of participants with moderate or greater reactogenicity (e.g., solicited adverse events) for 3 days following IV infusion of PGT121 mAb.
2. Proportion of participants with moderate or greater and/or PGT121 mAb-related unsolicited adverse events (AEs), including safety laboratory (biochemical, hematological) parameters, following IV infusion of PGT121 mAb for the first 56 days post administration of Investigational Product.
3. Proportion of participants with PGT121 mAb-related serious adverse events (SAEs) throughout the study period.

Pharmacokinetics:

We will measure the following endpoints to determine the pharmacokinetics following IV infusion of PGT121 mAb in HIV-uninfected and HIV-infected adults:

1. Elimination half-life (t1/2)
2. Clearance (CL/F)
3. Volume of distribution (Vz/F)
4. Area under the concentration decay curve (AUC)
5. Impact of viral load and/or ART on PGT121 disposition (elimination half-life (t1/2), clearance (CL/F), volume of distribution (Vz/F), total exposure

Antiviral activity:

We will calculate the following endpoints to determine the antiviral activity following IV infusion of PGT121 mAb in viremic HIV-infected adults not on ART.

1. Change in plasma HIV-1 RNA levels from baseline (mean of pre-entry and entry values)

4.1.2 Secondary Endpoints

Anti-PGT121 antibodies:

We will calculate the following endpoint to determine if anti-PGT121 antibodies are elicited following IV infusion of PGT121 mAb in HIV-uninfected and HIV-infected adults:

1. Change in serum anti-PGT121 antibody titers from baseline

CD4+ T cell count:

We will calculate the following endpoint to determine if IV infusion of PGT121 mAb has any impact on CD4+ T cell counts in HIV-infected adults:

1. Change in CD4+ T cell count and frequency compared to baseline as measured by single platform flow cytometry

HIV genotyping/phenotyping of circulating virus for evaluation of PGT121-induced escape mutations:

We will compare plasma virus sequences before and after IV infusion of PGT121 mAb to determine if PGT121 mAb induces viral escape mutations in viremic HIV-infected adults not on ART.

1. Development of sequence variations in epitopes known to result in reduced PGT121 neutralization susceptibility

4.1.3 Exploratory Endpoints

Additional assessments may include but are not limited to the following: HIV-specific IgG/IgA binding responses by ELISA, HIV-specific cellular immune responses by ELISPOT, HIV-specific antibody function by ADCC, ADCP, and ADCVI assays, resistance mutations to current ARVs, PGT121 mAb levels in mucosal secretions, changes in total HIV-1 DNA and 2-long terminal repeat (LTR) circular HIV-1 DNA in resting or total CD4 T cells and in vitro neutralization of HIV isolates with participant's serum post IV infusion of PGT121 mAb.

5.0 STUDY DESIGN

The study is a randomized, placebo-controlled study for groups 1 and 2, and open label for group 3 who will not receive placebo.

5.1 Definition of Dose Limiting Toxicity and Maximum Tolerated Dose

A dose limiting toxicity (DLT) will be defined as 1) any Grade 3 or greater reactogenicity or adverse event judged by the study investigators as at least possibly related to investigational product or 2) any SAE considered at least possibly related investigational product.
Maximum Tolerated Dose (MTD) will be declared when 2 or more DLTs occur that are the same, similar, or in the same System Organ Class or if no DLT occurs in the final dose subgroup, MTD will be the highest dose given (groups 1C and 2C 30mg/kg) after 14 days of follow-up.

5.2 Dose Escalation – Groups 1 and 2: Determination of Maximum Tolerated Dose

In Groups 1 and 2, (Part 1), the administrations of PGT121 mAb escalate by dose as shown below in Table 5.3.1, Study Design (5 participants per dose subgroup, 4:1 ratio of IP to placebo for each dose subgroup).

Sentinel Recipients

Within each dose group (subgroups 1A and 2A, subgroups 1B and 2B, subgroups 1C and 2C), the first 3 participant infusions will be separated by at least 4 days, to allow for observation of Investigational product (IP)-related adverse events. Dose subgroups will be enrolled in parallel, meaning that the 1st participant may be from subgroup 1A, the 2nd from subgroup 2A, the 3rd from subgroup 2A, all with 4 days in between dosing.

Because there is 1 placebo in each dose subgroup and the subgroups are dosed in parallel, the first 3 recipients will be treated as sentinel recipients (randomization will ensure that at least 2 will receive the IP). If no reactogenicity and adverse events that are considered to be related to IP (possibly, probably or definitely related) and are graded as severe or worse (Grade 3 or 4 on the DAIDS Toxicity Table) occur within 4 days after infusion of the first participant, the second participant may be injected. If no events meeting the criteria described above occur within 4 days after the 3rd participant is infused, then the remainder of participants in that dose group will be infused. If events meeting the criteria described above do occur for the first 3 participants in a dose group, they will be reviewed by the Safety Monitoring Committee (SMC) to determine whether further infusions may proceed.

Dose Escalation and Determination of Maximum Tolerated Dose

Safety data through day 14 post-IP administration visit for all participants in the first dose group (1A and 2A) will be reviewed by the Protocol Safety Review Team (PSRT) prior to allowing enrollment of participants into the second dose group (1B and 2B). The review process will be repeated between the second and third (1C and 2C) dose groups. Following administration of IP for the last participant in both Groups 1 and 2, an independent Safety Monitoring Committee (SMC) will review safety data to confirm Maximum Tolerated Dose (MTD) and determine whether, and at what dose, Group 3 can initiate enrollment.

Within each group, if no DLT occurs within 2 weeks from infusion of the first 5 participants in a dose group, dose escalation to the next dose group will proceed. If 1 DLT occurs, 3 additional participants will be enrolled; randomized between PGT121 mAb and placebo at a 2:1 ratio. If no additional DLTs occur within 2 weeks of infusion in the 8 mAb total participants (3:1 ratio of PGT121 mAb to placebo), the study can proceed with enrollment of the next dose group. If 2 or more DLTs accumulate in a subgroup that are the same, similar, or in the same organ class, dosing will be halted and the next lower dose level will be declared the maximum tolerated dose (MTD). If no DLT occurs in the final dose group, MTD will be the highest dose given (subgroups 1C and 2C 30mg/kg) after 14 days of follow-up.

5.3 Dose De-Escalation- Group 3: Determination of Minimum Effective Dose

Upon approval by the SMC (see section 17.2.2), group 3 (Part 2), PGT121 mAb administrations will de-escalate by dose as shown below in Table 5.3.1.

Group 3 will start with the MTD (i.e. subgroups 3A and 3D if the MTD is 30 mg/kg, subgroups 3B and 3C if the MTD is 10 mg/kg, or subgroups 3C and 3F if the MTD is 3 mg/kg) as determined by the SMC from data in Part 1.

If subgroup 3A (n = 6) achieves a mean decline in HIV RNA of ≥0.9 log compared to baseline, enrollment into subgroup 3A will be stopped, and the next lower dose subgroup 3B will be initiated. The same rule will apply for progression from subgroup 3B to 3C. At least 6, 8 and 10 but up to 9, 12 and 15 participants will be enrolled in subgroups 3A, 3B and 3C respectively, until the minimum effective dose is determined. In each subgroup, if a mean decline >0.9 log in HIV RNA is not achieved, enrollment will be stopped at the completion of enrollment at that dose level.

Three participants will be enrolled in each group 3D, 3E and 3F. As soon as subgroup 3D has enrolled 3 participants, enrollment into subgroup 3E will be initiated. The same rule will apply for progression from subgroup 3E to 3F. In the event that dose de-escalation in subgroups 3A-C is stopped due to failure to meet virologic endpoints, dose de-escalation in subgroups 3D-F will continue.

Table 5.3.1 Study Design Table

	Group	Participants	Sub-Group	Regimen	N	Dose (mg/kg)
Part 1	1	HIV-uninfected participants	1A	PGT121/Placebo	4/1 (6/2 if DLT)	3
			1B	PGT121/Placebo	4/1 (6/2 if DLT)	10
			1C	PGT121/Placebo	4/1 (6/2 if DLT)	30
	2	HIV-infected on ART, (<50 cp/ml)	2A	PGT121/Placebo	4/1 (6/2 if DLT)	3
			2B	PGT121/Placebo	4/1 (6/2 if DLT)	10
			2C	PGT121/Placebo	4/1 (6/2 if DLT)	30
Safety Monitoring Committee Review						
Part 2	3	HIV-infected off ART (VL $2x10^3$ – $1x10^5$cp/ml)	3A	PGT121	6 (max 9)	30
			3B	PGT121	8 (max 12)	10
			3C	PGT121	10 (max 15)	3
		HIV-infected off ART (VL $1x10^2$ – $2x10^3$ cp/ml)	3D	PGT121	3	30
			3E	PGT121	3	10
			3F	PGT121	3	3

Table 5.3.2 Decision Tree, Groups 3A, 3B, 3C

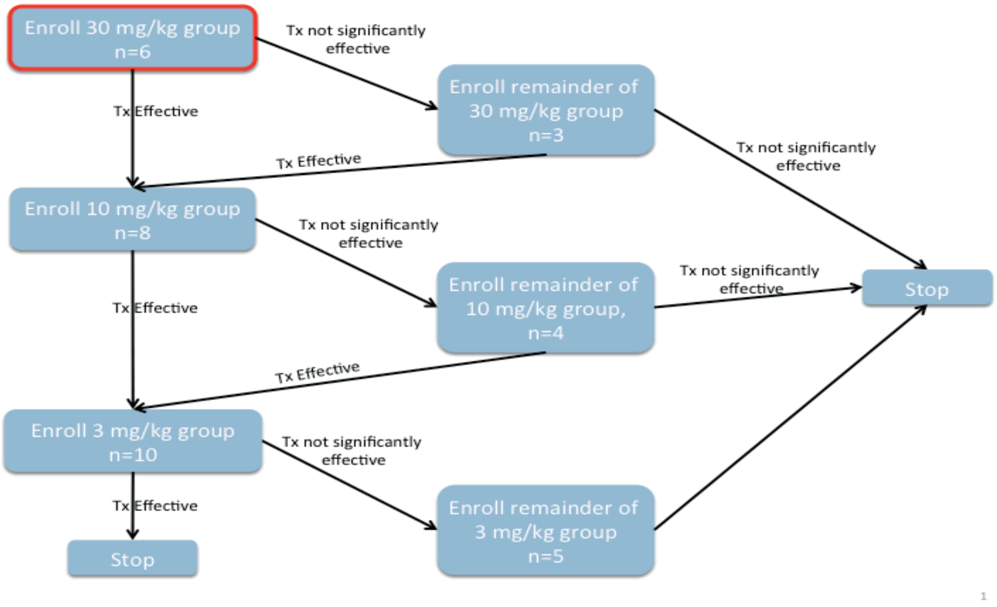

"not significantly effective" = does not achieve mean decrease of ≥0.9 log HIV RNA

5.4 Duration of the Study

Participants will be screened up to 42 days before IP administration of PGT121 mAb and will be followed for 24 weeks.

It will take approximately 8 months to enroll the entire study. The exact duration depends on the recruitment rate and how many participants will be required per dose group as specified in sections 5.2 and 5.3.

5.5 Study Population

The study population consists of HIV-uninfected male or female adults (Group 1), HIV-infected male or female adults on ART (Group 2), and HIV-infected males and female adults not on ART (group 3) who meet the detailed inclusion and exclusion criteria listed below, and who in the opinion of the investigator or designee, understand the study and provide written informed consent.

Approximately 63-93 participants (81 investigational product recipients, 12 placebo recipients) who meet all eligibility criteria will be included in the study. An over-enrollment of up to 5% (up to 5 participants total) will be permitted in the study to facilitate rapid enrollment.

5.6 Inclusion Criteria

Inclusion criteria for all participants:

1. At least 18 years of age on the day of screening and has not reached his or her 51st birthday on the day of signing the Informed Consent Document.

2. Willing to comply with the requirements of the protocol and available for follow-up for the planned duration of the study;

3. In the opinion of the Principal Investigator or designee and based on Assessment of Informed Consent Understanding results, has understood the information provided and potential impact and/or risks linked to IV infusion and participation in the trial; written informed consent will be obtained from the participant before any study-related procedures are performed;

4. All heterosexually active female participants must commit to use an effective method of contraception for 3 months following IP administration, including:
    a. Condoms (male or female) with or without spermicide
    b. Diaphragm or cervical cap with spermicide
    c. Intrauterine device, or contraceptive implant
    d. Hormonal contraception
    e. Successful vasectomy in the male partner (considered successful if a woman reports that a male partner has [1] documentation of azoospermia by microscopy (< 1 year ago), or [2] a vasectomy more than 2 years ago with no resultant pregnancy despite sexual activity post-vasectomy)
    f. Not be of reproductive potential, such as having undergone hysterectomy, bilateral oophorectomy, or tubal ligation, postmenopausal (>45 years of age with amenorrhea for at least 2 years, or any age with amenorrhea for at least 6 months and a serum follicle stimulating hormone [FSH] level >40 IU/L); surgically sterile: no additional contraception required.

   Women, who are not heterosexually active at screening, must agree to utilize an effective method of contraception if they become hetero-sexually active, as outlined above.

5. All sexually active males, regardless of reproductive potential, must be willing to consistently use an effective method of contraception (such as consistent male condoms with male and/or female partners from the day of IP administration until at least 3 months following IP administration to avoid exposure of partners to IP in ejaculate, and to prevent conception with female partners.

6. All female participants must be willing to undergo urine pregnancy tests at time points indicated in the Schedule of Procedures and must test negative prior to IP administration;

7. A woman must agree not to donate eggs (ova, oocytes) for the purpose of assisted reproduction until 3 months after receiving IP administration. A man must agree not to donate sperm until 3 months after IP administration;

8. Willing to forgo donations of blood and/or any other tissues, including bone marrow, during the study and, for those HIV-uninfected participants who test HIV-positive due to IP administration, until the anti-HIV antibody titers become undetectable.

Specific inclusion criteria for HIV-uninfected participants (Group 1):

9. Willing to undergo HIV testing, risk reduction counselling and receive HIV test results;

10. Low risk for HIV infection (see section (9.4.6) and willing to maintain low-risk behaviour for the duration of the trial (Appendix B);

11. Healthy male or female, as assessed by a medical history, physical exam, and laboratory tests;

Specific inclusion criteria for HIV-infected participants (Groups 2 and 3):

12. Confirmed HIV-1 infection (HIV Ab+ or HIV RNA+) by documentation in the medical records or in-clinic HIV testing;

13. CD4 ≥ 300 cells/µl;

14. No history of AIDS-defining illness or CD4 < 200 cells/µl.

Group 2:

15. Currently on ART with no change in ART regimen in the 12 weeks before screening or between screening and enrolment, with suppression of plasma HIV-1 viral load < 50 copies / ml for greater than 6 months, measured on at least 2 independent occasions, and with a viral load < 50 copies / ml at time of screening (within 42 days prior to IP administration). cART is defined as a regimen including > 2 compounds, e.g. 2x nucleoside reverse transcriptase inhibitors plus either non-nucleoside reverse transcriptase inhibitor or protease inhibitor or integrase inhibitor.

Group 3:

16. Not receiving cART, and (after appropriate counselling) willing to defer cART treatment for at least 56 days after administration of IP;

17. HIV-1 viral load either between 2000-100,000 copies / ml (Group 3A, 3B, 3C) or between 100-2000 copies / ml (Group 3D, 3E and 3F) at 2 independent occasions within 12 months prior to study enrollment, with confirmation during the screening period (3 viral loads on independent occasions).

5.7 Exclusion Criteria

Exclusion criteria for all participants:

1. Any clinically significant acute or chronic medical condition, other than HIV infection, that is considered progressive or in the opinion of the investigator makes the participant unsuitable for participation in the study;

2. If female, pregnant, lactating or planning a pregnancy during the period of screening through completion of the study;

3. In the past 6 months a history of alcohol or substance use, including marijuana, judged by the Investigator to potentially interfere with participant study compliance;

4. Bleeding disorder that was diagnosed by a physician (e.g., factor deficiency, coagulopathy or platelet disorder that requires special precautions). Note: A participant who states that he or she has easy bruising or bleeding, but does not have a formal diagnosis and has intramuscular injections and blood draws without any adverse experience, is eligible;

5. History of a splenectomy;

6. Receipt of live attenuated vaccine within the previous 60 days or planned receipt within 60 days after administration of IP; or receipt of other vaccine within the previous 14 days or planned receipt within 14 days after infusion with IP (exception is live attenuated influenza vaccine within 14 days);

7. Receipt of blood transfusion or blood-derived products within the previous 3 months;

8. Participation in another clinical trial of an investigational product currently, within the previous 3 months or expected participation during this study;

9. Prior receipt of an investigational HIV vaccine candidate, monoclonal antibody or polyclonal immunoglobulin (note: receipt of placebo in a previous HIV vaccine or monoclonal antibody trial will not exclude a participant from participation if documentation is available and the Medical Monitor gives approval);

10. History of severe local or systemic reactogenicity to injections or IV infusion (e.g., anaphylaxis, respiratory difficulties, angioedema);

11. Psychiatric condition that compromises safety of the participant and precludes compliance with the protocol. Specifically excluded are persons with psychoses within the past 3 years, ongoing risk for suicide, or history of suicide attempt or gesture within the past 3 years;

12. If, in the opinion of the Principal Investigator, it is not in the best interest of the participant to participate in the trial;

13. Seizure disorder: a participant who has had a seizure in the last 3 years is excluded. (Not excluded: a participant with a history of seizures who has neither required medications nor had a seizure for 3 years.)

14. Body mass index ≥ 30 or ≤ 18.0.

15. Infectious disease: chronic hepatitis B infection (HbsAg), current hepatitis C infection (HCV Ab positive and HCV RNA positive) or interferon-alfa treatment for chronic hepatitis C infection in the past year, or active syphilis (RPR confirmed by TPHA).

16. A history of malignancy within the past 5 years (prior to screening) or ongoing malignancy;

17. Active, serious infections (other than HIV-1 infection) requiring parenteral antibiotic, antiviral or antifungal therapy within 30 days prior to enrollment;

Specific exclusion criteria for HIV-uninfected participants (Group 1):

18. Confirmed HIV-1 or HIV-2 infection;

19. Any clinically relevant abnormality on history or examination including history of immunodeficiency or autoimmune disease; use of systemic corticosteroids, immunosuppressive, anticancer, or other medications considered significant by the investigator within the previous 6 months.

    The following exceptions are permitted and will not exclude study participation: use of corticosteroid nasal spray for rhinitis, topical corticosteroids for an acute uncomplicated dermatitis; or a short course (duration of 10 days or less, or a single injection) of corticosteroid for a non-chronic condition (based on investigator clinical judgment) at least 6 weeks prior to enrollment in this study.

Specific exclusion criteria for HIV-uninfected participants (Group 1) and HIV-infected participants who are on ART (Group 2):

20. Any of the following abnormal laboratory parameters listed below:

    Hematology

    – Hemoglobin <10.5 g/dL in females; hemoglobin <11.0 g/dL in males
    – Absolute Neutrophil Count (ANC): ≤1000/mm^3
    – Absolute Lymphocyte Count (ALC): < 650/mm^3
    – Platelets: < 125,000 mm^3 or ≥ 550,000/mm^3

    Coagulation

    - aPTT: >1.25x ULN
    - INR: ≥1.1 x ULN

    Chemistry

    - Sodium ≤135 mEq/L or ≥ 146 mEq/L
    - Potassium ≤3.4 mEq/L or ≥ 5.6 mEq/L
    - Creatinine ≥1.1 x ULN
    - AST ≥1.25 x ULN
    - ALT ≥1.25 x ULN
    - Total bilirubin ≥1.25 x ULN
    - Alkaline phosphatase ≥1.25 x ULN
    - Albumin ≤ 3.0 g/dL or ≤ 30 g/L
    - Creatine kinase ≥3.0 x ULN
    - C-reactive protein >10 mg/L
    - C3 complement ≤ 0.9g/L
    - C4 complement ≤ 0.1g/L

    Urinalysis

Clinically significant abnormal dipstick confirmed by microscopy:
– Protein = 1+ or more
– Blood = 1+ or more (not due to menses)

Specific exclusion criteria for HIV-infected participants who are on ART (Group 2) and for HIV-infected participants who are not on ART (Group 3):

21. Any clinically relevant abnormality on history or examination including history of immunodeficiency or autoimmune disease, other than HIV; use of systemic corticosteroids, immunosuppressive, anticancer, or other medications considered significant by the investigator within the previous 6 months.

    The following exceptions are permitted and will not exclude study participation: use of corticosteroid nasal spray for rhinitis, topical corticosteroids for an acute uncomplicated dermatitis; or a short course (duration of 10 days or less, or a single injection) of corticosteroid for a non-chronic condition (based on investigator clinical judgment) at least 6 weeks prior to enrollment in this study.

Specific exclusion criteria for HIV-infected participants who are not on ART (Group 3)

22. HIV-specific antibodies that significantly cross-react with PGT121 mAb pharmacokinetic assays

23. Resistance of autologous HIV to PGT121 neutralization in vitro;

24. Any of the following abnormal laboratory parameters listed below:

Hematology

– Hemoglobin < 10.0 g/dL
– Absolute Neutrophil Count (ANC): <800 cells/mm3
– Platelets: < 100,000 cells/mm3

Coagulation

- aPTT: >1.25x ULN
- INR: ≥1.1 x ULN

Chemistry

- Estimated Glomerular filtration rate (GFR) ≤ 80 mL/min according to the Cockcroft-Gault formula for creatinine clearance:
    - Male: $\frac{(140 - \text{age in years}) \times (\text{wt in kg})}{72 \times (\text{serum creatinine in mg/dL})} = \text{CLcr (mL/min)}$
    - Female: $\frac{(140 - \text{age in years}) \times (\text{wt in kg}) \times 0.85}{72 \times (\text{serum creatinine in mg/dL})} = \text{CLcr (mL/min)}$
- AST ≥ 2.5 x ULN

- ALT ≥ 2.5 x ULN
- Total bilirubin ≥ 1.6 x ULN
- Alkaline phosphatase ≥ 5 x ULN

Urinalysis

- Any RBC, protein or leukocytes greater than 1+, confirmed by microscopy and consistent with clinically significant disease.

5.8 Recruitment of Participants

Adult male and female participants may be recruited through in-clinic referrals, information presented to community organizations, hospitals, colleges, other institutions and/or advertisements to the general public or from existing cohorts. The information distributed will contain contact details of the trial site.

6.0 STUDY VISITS

6.1 Screening Period

During Screening, study staff will perform the following procedures:

- Provide and/or review the Informed Consent Document and answer any questions about the study prior to obtaining written informed consent.
- Complete Assessment of Informed Consent Understanding (AOU). Please refer to the Study Operations Manual (SOM)

If the participant agrees to participate, passes the AOU and provides written informed consent, study staff will:

- Conduct HIV test counselling, HIV testing, and HIV risk reduction counselling, as applicable
- Conduct family planning counselling, refer for pregnancy prevention counselling if necessary
- Conduct ART counselling, if applicable
- Perform a comprehensive medical history
- Collect concomitant medication information
- Perform a general physical examination (Refer to Section 7.2)
- Collect specimens for all tests as indicated in the Schedule of Procedures in Appendix A (for details see Analytical Plan (AP).

When available, the screening laboratory tests will be reviewed by the trial physician. Screening laboratory test(s) may be repeated once at the discretion of the principal investigator or designee to investigate any isolated abnormalities.

If the screening visit occurs more than 42 days prior to the date of administration of IP, all screening procedures must be repeated except the comprehensive medical history may be replaced by an interim medical history and the Participant Information Sheet of the Informed Consent Document should be reviewed.

If a participant has signed the Consent Form but does not meet the eligibility criteria, the records must be kept at the site.

6.2 IV infusion of PGT121 mAb Visit

Prior to the administration of IP, study staff will:

- Answer any questions the participant may have about the study
- Review the Informed Consent Document with the participant
- Review screening safety laboratory data
- Administer HIV risk assessment (Group 1)
- Conduct HIV test counselling, and HIV risk reduction counselling
- Conduct family planning counselling as per site specific procedures and ensure compliance with respective pregnancy prevention method, and discuss male condom use with all male participants
- Review interim medical history
- Collect concomitant medication information
- Weigh participant and record vital signs
- Perform a symptom-directed physical examination (Refer to Section 7.2)
- Assess at baseline local and systemic signs and symptoms (this includes an examination of IV infusion site)
- Collect specimens for all tests as indicated in the Schedule of Procedures see Appendix A (for details see AP).
- Obtain pregnancy test results prior to administration of IP.

Assign an allocation number to the participant according to the instructions specified in the Study Operations Manual.

At the time of administration of IP and after IV infusion of IP, study staff will:

- Administer the IP as specified in Section 8.4, Administration of Investigational Product and according to the instructions specified in the SOM.
- Observe participant closely during the infusion of IP and for at least 30 minutes after IV infusion of IP has ended for any acute reactogenicity. At the end of the observation period study staff will:
    - Record vital signs (pulse, respiratory rate, blood pressure and temperature)
    - Assess any local and systemic reactogenicity
    - Assess any other adverse events
- Every hour after IV infusion of IP, starting hour 1 through 12, the study staff will:
    - Record vital signs (pulse, respiratory rate, blood pressure and temperature)
    - Assess any local and systemic reactogenicity
    - Assess any other adverse events
    - Collect PK samples according to the Schedule of Procedures

If a participant has an abnormal laboratory value that is known, at the time of infusion, follow the specified guidelines (Section 12.0).

6.3 Post-IV infusion of PGT121 mAb Visits

The participant will be asked to return to the clinic for post-IP administration visits as indicated in the Schedule of Procedures (see Appendix A) for an assessment by clinic staff. The participant will be asked to maintain a Memory Aid to track any local and systemic reactogenicity the participant experiences, including temperature, from the day of IP administration for the next 3 days (for a total of 4 days including day of IP administration). Study staff will review the Memory Aid with the participant and determine the severity of the reactions through discussion with the participant.

The following procedures will be conducted at these visits:

- Review interim medical history
- Collect concomitant medication information
- Perform a symptom-directed physical examination if any signs or symptoms are present
- Assess vital signs (pulse, respiratory rate, blood pressure and temperature)
- Assess any adverse events and local and systemic reactogenicity (Days 1, 2, 3) including reviewing the Memory Aid.
- Collect specimens for all tests as indicated in the Schedule of Procedures (Appendix A) and AP).

6.4 Additional Follow-up Visits

Assessments and procedures will be performed according to the Schedule of Procedures (Appendix A).

6.5 Unscheduled Visits

Unscheduled Visits/Contacts are visits/contacts that are not described in the Schedule of Procedures (Appendix A). Unscheduled visits may occur any time during the study:

- For administrative reasons, e.g., the participant may have questions for study staff or may need to re-schedule a follow-up visit.
- To obtain laboratory test results from a previous visit.
- For other reasons as requested by the participant or site investigator.

All unscheduled visits will be documented in the participants' study records on applicable source documents and entered into the Case Report Form (CRF).

6.6 Final Study Visit or Early Termination Visit

Assessments and procedures will be performed according to the Schedule of Procedures (Appendix A).

7.0 STUDY PROCEDURES

7.1 Informed Consent Process

A Master Informed Consent Document consisting of a Participant Information Sheet and a Consent Form is provided by the Sponsor to the trial site. This document is made site-specific and translated (if necessary), submitted and approved by the Institutional

Review Board (IRB). The Master and site specific Informed Consent Documents are separate documents and should not be part of the protocol.

Participant Information Sheet

A qualified member of the study staff will conduct the informed consent process by reviewing the Participant Information Sheet and document it in the clinic notes.

Consent Form

The participant's consent to participate must be obtained by him/her signing and dating the Consent Form. The person obtaining consent will also sign.

The signed and dated Informed Consent Document must remain at the study site. A copy of the signed/marked and dated Informed Consent Document will be offered to the participant to take home. Those participants who do not wish to take a copy will be required to document that they declined to do so.

7.2 Medical History and Physical Examination

Medical History

At screening, a comprehensive medical history will be collected including previous IV infusions and reaction to IV infusion, history of sexually transmitted infection (STI) and pregnancy prevention practices. At subsequent visits, an interim medical history will be performed.

Physical Examination

General Physical Examination

A general physical examination includes examination of head/ears/eyes/nose and throat, skin, respiratory, cardiovascular, abdominal, limited neurological and musculoskeletal and external ano-genital systems (for HIV-infected participants only) at the time points indicated in the Schedule of Procedures (see Appendix A).

Symptom-Directed Physical Examination

A symptom-directed physical examination is a targeted examination based on the participant’s history or observation. If deemed necessary, this examination should be done at the time points indicated in the schedule of procedures (see Appendix A).

Measuring Height and Weight

Includes measuring the height and weight at the time points indicated in the Schedule of Procedures (see Appendix A).

Vital Signs

Vital signs including pulse, respiratory rate, blood pressure and temperature are measured and recorded at the time points indicated in the Schedule of Procedures (see Appendix A)

7.3 HIV Testing and HIV-test Counselling (Group 1)

Study staff will perform pre-HIV test counselling prior to collecting blood for an HIV test, and post-HIV test counselling when HIV test results are available. This is referred to as

HIV-test counselling, and done according to the CDC guidelines. For more information on HIV testing and HIV-test counselling, see Section 11.0. A screening questionnaire and other tools may be used.

7.4 HIV Risk Reduction Counselling

HIV risk reduction counselling will be provided to all participants as outlined by site-specific SOPs.

Study staff will provide HIV risk reduction counselling based on reported individual risk and provide free condoms, as appropriate, at every visit. Group 1 will receive HIV risk reduction counselling and for Groups 2 and 3, HIV risk reduction counselling will be conducted as secondary prevention to reduce onward transmission.

7.5 Family Planning Counselling

Study staff will counsel participants about the importance of preventing pregnancies and of using condoms, as well as other effective family planning methods, as appropriate. Participants may be referred for family planning services as necessary according to site-specific SOPs as detailed in the SOM. Pregnancy prevention methods chosen and compliance will be documented.

7.6 ART Counseling (Group 3)

HIV-infected participants who are not on ART will receive ART counselling upon entering the study and 8 weeks after administration of IP. Participants who have not initiated or made plans to initiate ART by the final study visit will receive ART counselling again at their final study visit. HIV-infected participants who are on ART (Group 2) will be counseled on the importance of continuing ART throughout the study, and will not be required to interrupt ART after administration of IP.

7.7 Specimens

Approximately 51 ml of blood will be collected from participants in Group 1, approximately 78 ml from participants in Group 2, and approximately 129 ml of blood will be collected from participants in Group 3 at the screening visit. At later visits, approximately 8.5 ml to 163 ml of blood will be collected, depending on study procedures and group assignment (see Appendix A), usually from the antecubital fossa.

Optional collection of rectal and/or cervical mucosal secretions will be obtained using a rectal sponge or cervical Softcup for those participants that consent.

All specimens will be handled according to the procedures specified in the AP.

In the event of an abnormal laboratory value, participants may be asked to have an additional sample collected at the discretion of the Principal Investigator or designee.

7.8 Reimbursement

Participants will be reimbursed for their time, effort and for costs to cover their travel expenses to the study site and any inconvenience caused due to study participation. Site specific-reimbursement amounts will be documented in the site-specific Participant Information Sheet, and approved by the Institutional Review Board.

7.9 Randomization and Blinding

Participants will be identified by a unique study identification number.

Participants will be randomized according to the randomization schedule prepared by the statisticians at the Data Coordinating Center (DCC) prior to the start of the study. Participants will be automatically assigned a specific allocation number as they are enrolled into the data entry system. An unblinding list (Pharmacy List) will be provided to the unblinded site pharmacist by the DCC.

This is a randomized, double-blind placebo-controlled study for groups 1 and 2, and an open label study for group 3. For Groups 1 and 2, study staff (investigator and clinical personnel monitoring the safety and laboratory assay results) and participants will be blinded with respect to the allocation of Investigational Product (PGT121 mAb or placebo). A site pharmacist will be unblinded for the purposes of preparing study product.

A participant will be considered enrolled once he/she has been assigned an allocation number.

Blinded participants will be informed about their assignment (product/placebo) at study completion, once the database is locked. Should a study participant be unblinded during the study, the study participant will be followed up until the end of the study according to the Schedule of Procedures (Appendix A).

7.10 Un-blinding Procedure for Individual Participants

Un-blinding of an individual participant may be indicated in the event of a medical emergency if the clinical management of the participant would be altered by knowledge of the treatment assignment.

The un-blinded information should be restricted to a small group of individuals involved in clinical management/medical treatment of the participant (e.g. treating physician) and the blind must be maintained for those responsible for the study assessments.

The reasons for un-blinding should be documented and the IAVI Chief Medical Officer, the Medical Monitor and the DCC should be notified as soon as possible. The procedures and contact numbers for un-blinding are outlined in the SOM.

7.11 Assessment of IP related HIV sero-positivity

It is possible that PGT121 mAb or an immune response to PGT121 mAb could cause a positive result on a diagnostic HIV antibody test. A Group 1 participant who tests HIV antibody positive at the end of the study will have additional testing to distinguish actual HIV infection from IP-related responses. The participant will be informed of his/her positive HIV antibody test result and offered continuing follow-up until the HIV antibody test becomes negative.

8.0 INVESTIGATIONAL PRODUCT

8.1 Description

A summary of the Investigational Products is shown in Table 8.1-1.

Table 8.1-1 Investigational Products

**Product / Placebo**	**Dosage level**	**Total volume in IP container**	**Total IP or placebo volume to be injected into a 100 mL saline IV bag** (for an 88 kg body weight**)	**Total volume to be Infused** (for an 88 kg body weight**)
IP: PGT121 (50 mg/mL)	3 mg/kg	6 mL per vial	5.3 mL	105.3 mL
	10 mg/kg		17.6 mL	117.6 mL
	30 mg/kg		52.8 mL	152.8 mL
Placebo: 0.9% Sodium Chloride Injection USP (Saline)*	3 mg/kg matching***	NA	5.3 mL***	105.3 mL***
	10 mg/kg matching***		17.6 mL***	117.6 mL***
	30 mg/kg matching***		52.8 mL***	152.8 mL***

* The Placebo provided will be a commercially-available saline partial addition IV bag.

** The actual volume to be injected will be based on the dose group and the weight of the participant at the time of IP administration. The example included here is the average weight of an adult male in the US (88kg) (http://www.cdc.gov/nchs/data/series/sr_11/sr11_252.pdf)

*** For placebo IV infusions: saline from an additional IV bag will be injected into the saline IV bag intended for administration, to match the volume used for a PGT121 mAb injection in the same dose group, to prevent unblinding.

8.2 Shipment and Storage

Authorization to ship the Investigational Product to the site will be provided in writing by the Sponsor, upon confirmation that all required critical documents for shipment authorization are completed. The Investigational Product will be shipped maintaining the required storage conditions and stored in a secure location in the clinical site's pharmacy.

The Investigational Product is formulated in a 20 mM Acetate, 9% Sucrose, 0.008% polysorbate 80, pH 5.2 formulation buffer at a concentration of 50 mg/mL. Each 10 ml vial will contain 6 ml of IP stored at <- 20°C. Each vial will be labelled with the name of the product, Lot number, concentration, storage temperature, date of manufacturing, contact information of the Sponsor and a US cautionary statement. Several such vials will be packaged in a box. Each box will also be labelled with similar information as the vial label.

8.3 Preparation of Investigational Product (IP)

Detailed instruction will be provided to the site pharmacist in the SOM for preparing each of the investigational products. The site pharmacist will not be blinded, but the study physician/designee administering the IP will be blinded. Product should be administered within 6 hours of preparation. Example calculations for final volume for IV infusion are illustrated in Table 8.1-1. Procedures for handling used and partially used vials will be provided in the SOM.
Syringes or other components in direct contact with investigational products will be disposed of in a biohazard container and incinerated or autoclaved.

8.4 Administration of Investigational Product

Investigational Product will be administered at the enrollment visit.

The IP will be injected into a 0.9% Saline bag. The participant will receive the IP via IV infusion. Participants will receive infusion over approximately 60 minutes, allowing for clinician discretion. Further information on the IV infusion of the IP is supplied in the SOM and study documents.

8.5 Accountability and Disposal of Investigational Product

All used IP vials will be handled according to instructions in the SOM. The date, allocation number and location of storage of the returned vials will be recorded.

During the study, the IP accountability forms including receipt and dispensing of vials will be kept and monitored.

At the end of the study, the used and unused IP vials will be handled according to instructions of Sponsor.

Further information on accountability and disposal of IP is supplied in the SOM.

9.0 ASSESSMENTS

9.1 Safety Assessments

Data on local and systemic reactogenicity (i.e., solicited AEs) will be collected by structured interview and medical examination. Data on other adverse events will be collected with open-ended questions. All data will be recorded on the appropriate source documents and entered into the study database. Participants will be given a Memory Aid, which is a tool to assist with collecting reactogenicity data.

Local and systemic reactogenicity events will be assessed by study staff prior to IV infusion of IP, at approximately 30 minutes after IP administration start, at 1 hour after IP administration start, and subsequently every hour for the first 12 hours post-IP administration. Study staff will review the Memory Aid with the participant, and determine the severity of the reactions on days 1-3 through discussion with the participant.

9.1.1 Local reactogenicity

The presence of local reactogenicity will be assessed at the time points specified in the Schedule of Procedures (Appendix A).
Pain, tenderness, erythema/skin discoloration, swelling/hardening or pruritus will be assessed and graded using Appendix C, Adverse Event Severity Assessment Table, as a guideline.

9.1.2 Systemic reactogenicity

The presence of systemic reactogenicity will be assessed at the time points specified in the Schedule of Procedures (Appendix A).
Fever, chills, headache, nausea, vomiting, malaise, myalgia and arthralgia will be assessed and graded using the Appendix C, Adverse Event Severity Assessment Table as a guideline.

9.1.3 Vital Signs

At the administration of IP visit, vital signs (pulse, respiratory rate, blood pressure and temperature) will be measured by study staff prior to IP administration, at approximately 30 minutes post IP administration and hourly until 12 hours after IV infusion start. For the other study visits vital signs will be assessed at the time points specified in the Schedule of Procedures (Appendix A).

9.1.4 Other Adverse Events

Other adverse events (AEs) will be collected through 56 days after IP administration in all participants. Serious Adverse Events (SAEs) will be collected throughout the entire study period. Potential Immune Mediated Diseases (pIMDs), as defined in Section 10.5, will be collected throughout the study period, using the SAE reporting process. Open-ended questions will be asked at time points according to the Schedule of Procedures (Appendix A). All adverse events will be graded using Appendix C, Adverse Event Severity Assessment Table, as a guideline and will be assessed for causality to the IP. For more information regarding adverse events refer to Section 10.0, Adverse Events.

9.1.5 Concomitant Medications

Concomitant receipt of Investigational Products is prohibited during the study.

Contraceptive use and use of medication at study entry will be documented. (See DCF instructions)

During the study, information regarding concomitant medications and reasons for their use will be solicited from the study participants for 56 days. Ongoing concomitant medications will be recorded until end of study.

9.1.6 Routine laboratory parameters

Table 9.1.6-1 shows the laboratory parameters that will be measured routinely. The samples for these tests will be collected at the time points indicated in the Schedule of Procedures (Appendix A).

Table 9.1.6-1: Laboratory Parameters

Laboratory Parameter	Test
Hematology and Coagulation	Hemoglobin, hematocrit, leukocytes, platelets, absolute neutrophil count (ANC), absolute lymphocyte count (ALC), activate partial thromboplastin time (aPTT), international normalized ratio (INR)
Clinical Chemistry	Sodium, potassium, creatinine, aspartate aminotransferase (AST), alanine aminotransferase (ALT), total bilirubin, alkaline phosphatase<br>Groups 1 and 2 only: albumin, creatine kinase, C-reactive protein, C3 complement, C4 complement
Urinalysis	Dipstick test for protein, blood glucose, ketones, esterase (leukocytes) and nitrite. If clinically significant abnormalities (e.g., blood, protein, leukocytes) are found on dipstick test, then further test(s) will be performed (e.g., microscopy, culture)
T cell panel (Groups 2 and 3)	CD4 T cell count and frequency by single platform flow cytometry

9.1.7 Specific screening tests:

Participants will be screened to exclude the following diseases:

- Hepatitis B: positive for hepatitis B surface antigen (HBsAg)
- Hepatitis C: positive for hepatitis C RNA (HCV antibody test, followed by HCV RNA test if HCV antibody positive)
- Active syphilis: confirmed diagnosis (e.g.; positive RPR confirmed by TPHA)

A negative Hepatitis B and Hepatitis C result can be documented from the medical record only if the result is from a test administered less than 6 months ago.

Participants will also be screened to exclude the following laboratory parameters:

- Resistance of autologous HIV to PGT121 neutralization *in vitro* (HIV viremic participants only, Group 3)

9.1.8 Monitoring for anti-PGT121 antibodies:

Participants will be evaluated for the development of antibodies to PGT121 mAb (anti-drug antibodies, ADA) by ELISA according to the Schedule of Procedures (Appendix A).

9.2 Virologic Assessments

Table 9.2-1 shows the virologic parameters that will be measured routinely. The samples for these tests will be collected at the time points indicated in the Schedule of Procedures (Appendix A).

Table 9.2-1: Virologic Assessment Table

Virologic Parameter	Test
Antiviral Activity	Plasma HIV RNA levels
Anti-reservoir activity	Cell-associated HIV-1 RNA levels in resting CD4 T cells; total HIV-1 DNA and 2-long terminal repeat (LTR) HIV-1 DNA circles in resting or total CD4 T cells; quantitative viral outgrowth assay (qVOA)
Other	Genotyping of plasma HIV RNA for evaluation of PGT121-induced escape mutations; phenotyping of plasma HIV RNA for neutralization susceptibility to PGT121 in-vitro

9.3 Exploratory Immunogenicity Assessments

Humoral immune response assays will include, but are not limited to Env-specific Ab-binding assays, virus neutralization assay, and assays for Ab functionality. Cellular immune response assays will include, but are not limited to IFNγ ELISPOT assay, ICS, and multiparameter flow cytometry. Exploratory assessments on mucosal samples will include, but are not limited to characterization of Env-specific binding Abs. Priority assays are listed below.

9.3.1 Antibody Responses

- Env-specific binding Abs (titers and breadth).
- Env-specific nAbs (titers and breadth).
- Env-specific functional Abs (phagocytosis score and breadth).
- Env-specific binding Ab isotypes (IgA, IgG1-4) (titers and breadth).

9.3.2 Cellular Responses

- IFNγ peripheral blood mononuclear cell (PBMC) responders to peptide pools and subpools of Potential T-cell epitopes, PTE Env/Gag/Pol peptides.
- $CD4^+$ and $CD8^+$ T-cell functionality (% cells producing e.g. IFNγ, IL-2, IL-4, TNFα).
- T-cell development with emphasis on follicular helper T-cells and memory differentiation.

9.3.3 PBMC, Serum and Plasma Storage

Samples of cryopreserved PBMC, plasma and serum will be stored as indicated in the Analytical Plan (AP) and, if the participant consents, may be used for the purposes of standardization, quality control and for future assays related to HIV prevention or treatment research and development. These samples will be archived and the testing laboratories will be blinded to the participant’s identity.

9.4 Other Assessments

9.4.1 HIV Antibody Testing

All HIV-uninfected participants (Group 1) will be tested for HIV antibodies as indicated in the Schedule of Procedures (Appendix A) or as needed, if medical or social circumstances arise. All participants will receive HIV risk reduction counselling and pre- and post-HIV-test counselling, as specified in Section 7.3 Counselling.

9.4.2 Pharmacokinetics

Blood draws for pharmacokinetics will be done on the day of IP administration immediately before starting IV infusion of IP, at the end of the IP administration, and 30 minutes and 3 hours after the end of the IP administration. Additional draws will be done at 6, 9, 12 and 24 hours after the start of the IP administration. Thereafter, pharmacokinetic draws will be done as indicated in the Schedule of Procedures (Appendix A). PGT121 mAb serum or plasma levels will be determined using two methods: a sandwich ELISA using a murine anti-idiotype antibody to PGT121 mAb, and a neutralization assay.

PGT121 mAb pharmacokinetic analysis will be performed using standard non-compartmental analysis methods to estimate elimination half-life (t1/2), clearance (CL/F), volume of distribution (Vz/F), Area under the concentration decay curve (AUC), impact of viral load and/or ART on PGT121 mAb disposition (elimination half-life (t1/2), clearance (CL/F), volume of distribution (Vz/F) and total exposure. PGT 121 accumulation will also be examined in rectal and cervical mucosal secretions collected with rectal sponges or cervical Softcups in study participants who specifically consented for these procedures. Descriptive results will be reported for the pharmacokinetic parameters by dose subgroup.

Exploratory analysis using population analysis methods simultaneously combining all pharmacokinetic data across all doses and treatment groups will be performed for quantitative characterization of differences in PGT121 mAb disposition by dose, participant group or disease state.

9.4.3 HLA Typing

Samples for HLA typing will be collected as specified in the AP and may be analyzed as warranted.

9.4.5 Pregnancy Test

A urine pregnancy test for all female participants will be performed by measurement of human chorionic gonadotrophin (βhCG) at time points indicated in the Schedule of Procedures (Appendix A). The results of the pregnancy test must be negative prior to IV infusion of PGT121 mAb. See section 10.7 for description of pregnancy after administration of IP.

9.4.6 HIV Risk Assessment (Group 1)

Study staff will assess participants for their past and current risk of acquiring HIV at time points indicated in Schedule of Procedures (Appendix A).

9.4.7 Social Impact Assessment

A brief assessment of the impact of participation in the study will be administered to participants at their final study visit.

10.0 ADVERSE EVENTS

10.1 Definition

An adverse event (AE) is any untoward medical occurrence in a participant administered an Investigational Product and which does not necessarily have a causal relationship with the Investigational Product. An AE can therefore be any unfavourable or unintended sign (including an abnormal laboratory finding), symptom, or disease, temporally associated with the use of Investigational Product whether or not related to the Investigational Product.

Assessment of severity of all AEs, including and seriousness of AEs, is ultimately the responsibility of the Principal Investigator of each site. Refer to the DIVISION OF AIDS (DAIDS) TABLE FOR GRADING THE SEVERITY OF ADULT AND PEDIATRIC ADVERSE EVENTS Version 2.0, November 2014 for additional guidance.

10.2 Assessment of Severity of Adverse Events

The following general criteria should be used in assessing adverse events as mild, moderate, severe or very severe at the time of evaluation:

Grade 1 (Mild): Symptoms causing no or minimal interference with usual social & functional activities

Grade 2 (Moderate): Symptoms causing greater than minimal interference with usual social & functional activities

Grade 3 (Severe): Symptoms causing inability to perform usual social & functional activities

Grade 4 (Very Severe): Symptoms causing inability to perform basic self-care functions OR Medical or operative intervention indicated to prevent permanent impairment, persistent disability, or death

Guidelines for assessing the severity of specific adverse events and laboratory abnormalities are listed in Appendix C, Adverse Event Severity Assessment Table.

10.3 Relationship to Investigational Product

Assessment of relationship of an AE or SAE to Investigational Product (IP) is the responsibility of the Principal Investigator or designee. All medically indicated and available diagnostic methods (e.g., laboratory, blood smear, culture, X-ray, etc.) should be used to assess the nature and cause of the AE/SAE. Best clinical and scientific judgment should be used to assess relationship of AE/SAEs to the IP and/or other cause.

The following should be considered:

- Presence/absence of a clear temporal (time) sequence between administration of the IP and the onset of AE/SAE
- Presence/absence of another cause that could more likely explain the AE/SAE (concurrent disease, concomitant medication, environmental or toxic factors)
- Whether or not the AE/SAE follows a known response pattern associated with the IP

The relationship assessment should be reported as one of the following:

Not Related: clearly explained by another cause (concurrent disease, concomitant medication, environmental or toxic factors, etc.).

Unlikely: more likely explained by another cause (concurrent disease, concomitant medication, environmental or toxic factors, etc.).

Possibly: equally likely explained by another cause but the possibility of the IP relationship cannot be ruled out (e.g., reasonably well temporally related and/or follows a known IP response pattern but equally well explained by another cause).

Probably: more likely explained by the IP (e.g., reasonably well temporally related and/or follows a known IP response pattern and less likely explained by another cause).

Definitely: clearly related and most likely explained by the IP.

For the purpose of expedited safety reporting, all possibly, probably or definitely related SAEs are considered IP-related SAEs.

10.4 Serious Adverse Events

An adverse event is reported as a "Serious Adverse Event" if it meets any of the following criteria (as per International Conference on Harmonisation [ICH] Good Clinical Practice [GCP] Guidelines):

- Results in death
- Is life threatening
- Results in persistent or significant disability/incapacity
- Requires in-participant hospitalization or prolongs existing hospitalization
- Is a congenital anomaly/birth defect or spontaneous abortion
- Any other important medical condition that requires medical or surgical intervention to prevent permanent impairment of a body function or structure

Elective surgery for pre-existing condition that did not increase in severity or frequency is not considered an SAE.

Serious Adverse Events (SAEs) should be reported within 24 hours of the site becoming aware of the event, and sent to the Sponsor as described in the SOM.

To discuss IP-related SAEs or any urgent medical questions related to the SAE, the site investigator should contact one of the IAVI Medical Monitors directly (see Contact List in the SOM).

The IAVI SAE Report Form should be completed with all the available information at the time of reporting and sent to the Sponsor as described in the SOM. The minimum data required in reporting an SAE are the study identification number, date of birth, gender, event description (in as much detail as is known at the time), onset date of event (if available), reason event is classified as serious, reporting source (name of Principal Investigator or designee), and relationship to the IP as assessed by the investigator.

The Principal Investigator or designee is required to prepare a detailed written report with follow up until resolution or until it is judged by the Principal Investigator or designee to have stabilized.

The Principal Investigator or designee must notify the local IRB/IEC of all SAEs as appropriate. In case of IP-related SAEs, the Sponsor will notify responsible regulatory authorities, Safety Monitoring Committee (SMC), and other study sites where the same IP is being tested.

More details on SAE definitions and reporting requirements are provided in the SOM.

Serious Event Prior to Investigational Product Administration

If a serious event occurs in the period between the participant signing the Informed Consent Form and receiving the IV infusion of IP, the event will be reported using the SAE form and following the same procedures for SAE reporting, as indicated in Section 10.4. The timing of the event will be indicated by using the relevant checkbox on the SAE form.

10.5 Reporting Potential Immune-Mediated Diseases

Potential immune-mediated diseases (pIMDs) are a subset of AEs that include both clearly autoimmune diseases and also other inflammatory and/or neurologic disorders that may or may not have an autoimmune etiology. These events are of special interest since they could potentially be caused by immune responses to the IP. The investigator/designee should report such adverse events within the same time limits (following confirmation of an AE as a pIMD; see last paragraph of this section below), and using the same CRF pages, as utilized for SAEs. The investigator or his/her designee will evaluate the occurrence of pIMDs at every visit/contact during the study. IAVI will also expect investigators/designee to provide additional information about pIMD events. AEs to be reported and documented as pIMDs include:

Neuroinflammatory disorders: optic neuritis, cranial nerve disorders (including Bell's palsy), multiple sclerosis, demyelinating disease, transverse myelitis, Guillain-Barré syndrome, myasthenia gravis, encephalitis, neuritis.

Musculoskeletal disorders: systemic lupus erythematosus, cutaneous lupus, Sjögren's syndrome, scleroderma, dermatomyositis, polymyositis, myopathy, rheumatoid arthritis and juvenile rheumatoid arthritis, polymyalgia rheumatica or temporal arteritis, reactive arthritis, psoriatic arthropathy, ankylosing spondylitis, undifferentiated spondyloarthropathy.

Gastrointestinal disorders: Crohn's disease, ulcerative colitis or proctitis, celiac disease.

Metabolic diseases: autoimmune thyroiditis, Grave's or Basedow's disease, Hashimoto thyroiditis, insulin-dependent diabetes mellitus (IDDM), Addison's disease.

Skin disorders: psoriasis, vitiligo, Raynaud's phenomenon, erythema nodosum, autoimmune bullous skin diseases.

Others: autoimmune hemolytic anemia, thrombocytopenia, antiphospholipid syndrome, *vasculitis, pernicious anemia, autoimmune hepatitis, primary biliary cirrhosis, primary sclerosing cholangitis, autoimmune glomerulonephritis, autoimmune uveitis, autoimmune myocarditis/cardiomyopathy, sarcoidosis, Stevens-Johnson syndrome, Behçet's syndrome.

Infusion site reactions: Grade 3 or 4 infusion site reactions lasting more than 2 days.

*Vasculitis: Vasculitis, Diffuse vasculitis, leucocytoclastic vasculitis, polyarteritis nodosa, microscopic polyangiitis, Wegener's granulomatosis, anti-neutrophil cytoplasmic antibody positive vasculitis, Henoch-schönlein purpura, allergic granulomatous angiitis (Churg-Strauss disease), Kawasaki disease, Takayasu's arteritis, temporal arteritis (giant cell arteritis), renal vasculitis.

Medical judgement should be exercised in deciding whether other disorders/diseases have an autoimmune origin and should also be reported as described above, and this judgement is the investigator's prerogative. Whenever sufficient data exist to substantiate any of the diagnoses in the above list, the event must be reported as a pIMD. While the intent of pIMD reporting is to be inclusive, isolated nonspecific symptoms, which might (or might not) represent the above diagnoses, should be captured as AEs but not reported as pIMDs until the diagnosis can be defended.

10.6 Clinical Management of Adverse Events

Adverse events (AEs) will be managed by the clinical study team who will assess, provide first line of care as appropriate and refer to health care and treatment facilities as warranted. If any treatment/medical care is required as a result of the harm caused by the IP or study procedures, this will be provided free of charge.

If a participant has an AE and/or abnormal laboratory value that is known at the time of IV infusion of IP, the specifications of Section 12.0 will be followed.

Participants will be followed until the AE resolves or stabilizes or up to the end of the study, whichever comes last. If at the end of the study, an AE (including clinically significant laboratory abnormality) that is considered possibly, probably or definitely related to the IP is unresolved, follow-up will continue until resolution if possible and/or the participant will be referred.

If a participant experiences a significant decrease in CD4 cell count (e.g. – 20% of baseline, or decline to <200 cells/µL) during the course of the trial, CD4+ will be monitored closely until their CD4 count returns to baseline or until the participant initiates ART. Participants whose CD4 cell counts decrease to <200 cells/µL will be promptly informed and will be referred to their primary HIV care provider. Appropriate prophylaxis

against opportunistic infections will be instituted according to accepted U.S. HIV treatment guidelines.

10.7 Pregnancy

Although not considered an AE, if a female participant becomes pregnant during the study, it is the responsibility of the Principal Investigator or designee to report the pregnancy promptly to IAVI using the designated forms. The participant will be followed for safety until the end of pregnancy or study completion, whichever occurs last. If possible, approximately 2–4 weeks after delivery, the baby will be examined by a physician to assess its health status and the results will be reported to the Sponsor. The baby will be examined again by a Physician around age 1, and the results will be reported to the Sponsor.

Complications of pregnancy that meet criteria for SAEs, specified in Section 10.4 of this Protocol (e.g., hospitalization for eclampsia, spontaneous abortion, etc.) should be reported as SAEs.

10.8 Intercurrent HIV Infection (Group 1)

HIV infection cannot be directly caused by the IP. If a participant acquires HIV through exposure in the community, at any time after the IV infusion of IP, the participant should be offered referral to appropriate care and treatment facilities. The participant will continue to be followed in the study for safety assessments.

Intercurrent HIV infection in study participants, although not considered an SAE, must be reported promptly to IAVI using the designated forms. However, medical conditions associated with the HIV infection that meet criteria for being serious specified in the Section 10.4 of this Protocol (e.g., sepsis, *Pneumocystis jiroveci [carinii] pneumonia*, etc.) should be reported as SAEs using the SAE Report Form.

11.0 MANAGEMENT OF HIV ISSUES DURING AND FOLLOWING STUDY

11.1 HIV Testing – Group 1

Group 1 participants will be tested for HIV antibodies as indicated in the Schedule of Procedures (Appendix A) or as needed, if medical or social circumstances arise. All participants will receive HIV risk reduction counselling and pre- and post-HIV-test counselling, as specified in Section 11.2.1, Counselling (Group 1).

It is possible that PGT121 or an immune response to PGT121 mAb could cause a positive result on a diagnostic HIV antibody test. An IP recipient who falsely tests HIV positive with a diagnostic HIV antibody test at the end of the study will be informed of his/her positive test result and offered continuing follow-up until the test becomes negative.

If a participant acquires HIV through exposure in the community, at any time after the administration of IP, the participant will be offered referral to appropriate care and treatment facilities. The participant will continue to be followed in the study for safety assessments.

Should a participant require HIV testing outside of the study for personal reasons, it is recommended that the participant contact the study staff first. HIV testing can be done at the study site and then processed at an independent laboratory as above. Written evidence of HIV status (HIV-infected or HIV-uninfected) will be provided upon request.

11.2 Social Discrimination as a Result of IP-related antibodies

In order to minimize the possibility of social discrimination in participants (if any) who test positive on a diagnostic HIV antibody test due to IP-related antibodies, appropriate diagnostic HIV testing and certification will be provided both during and after the study as needed.

11.3 HIV infection – Group 1

Group 1 participants who are diagnosed with HIV infection at screening or during the study (intercurrent HIV-infection) will be provided the following:

11.3.1 Counselling

The participant will be counselled by the study investigators or designated counsellors. The counselling process will assist the participant with the following issues:

- Psychological and social implications of HIV infection
- Who to inform and what to say
- Implications for sexual partners
- Implications for child-bearing
- Avoidance of transmission to others in future
- Mandatory reporting to the state, in some instances

11.3.2 Referral for Support/Care

Participants will be referred to a participant support center or institution of his/her choice for a full discussion of the clinical aspects of HIV infection. Referral will be made to a designated physician or center

12.0 WITHDRAWAL FROM STUDY

12.1 Deferral of IV infusion of IP

An IV infusion of IP may be temporarily deferred if the participant is clinically ill at the time of the administration of IP visit and/or presents with fever ($\geq$ 100.4 F) at the time of the administration of IP visit. A participant must be clinically well and afebrile for a minimum of a 24-hour consecutive period prior to administration of IP.

Any planned or unplanned deferral of infusion of IP will be discussed with the Sponsor. Participants will be deferred from infusion of IP for any of the following reasons:

1. Pregnancy
2. A disease or condition or adverse event that may develop, regardless of relationship to Investigational Product, if the Principal Investigator or designee is of the opinion that administration of IP will jeopardize the safety of the participant
3. Participant's request to defer infusion

The following events require resolution and/or review of clinical history by the Principal Investigator or designee and consultation with the Medical Monitor, prior to administration of IP:

- Any abnormal laboratory value, as outlined in section 5.7, Exclusion Criteria, Hematology, Chemistry, Urinalysis that is known at the time of infusion and have not resolved. Abnormal results should be confirmed on the original sample and/or repeated at least once to confirm abnormal values.
- Receipt of inactivated/killed/subunit vaccines (non-HIV) or immunoglobulin within the previous 14 days. Receipt of live attenuated vaccines within the previous 60 days.
- Participating in another clinical study of an Investigational Product

12.2 Withdrawal from the Study (Early Termination)

Participants may be withdrawn from the study permanently for the following reasons:

1. Participants may withdraw from the study at any time if they wish, for any reason
2. The Principal Investigator or designee has reason to believe that the participant is not complying with the protocol
3. If the Sponsor decides to terminate or suspend the study

If a participant withdraws or is withdrawn from the study, all termination visit procedures will be performed according to the Schedule of Procedures (Appendix A) where possible. Every effort will be made to determine and document the reason for withdrawal.

13.0 DATA HANDLING

13.1 Data Collection and Record Keeping at the Study Site

<u>Data Collection:</u> All study data will be collected by the clinical study staff using designated source documents and entered onto the appropriate electronic CRFs (eCRFs). Access to eCRFs will be provided via an electronic data entry system hosted by the Data Coordination Center. All study data must be verifiable to the source documentation. A file will be held for each participant at the clinic(s) containing all the source documents. Source documentation will be available for review to ensure that the collected data are consistent with the CRFs.

All CRFs and laboratory reports will be reviewed by the clinical team, who will ensure that they are accurate and complete.

Source documents and other supporting documents will be kept in a secure location. Standard GCP practices will be followed to ensure accurate, reliable and consistent data collection.

Source documents include but are not limited to:

- Signed Informed Consent Documents
- Progress notes
- Data collection forms
- Documentation of any existing conditions or past conditions relevant to eligibility

- Printed laboratory results
- Print out of the IDES generated enrollment confirmation
- All Adverse Events
- Concomitant medications
- Local and systemic reactogenicity events

13.3 Data Entry at the Study Site

The data collected at the site will be recorded onto the eCRFs by the study staff and entered into a database. To provide for real time assessment of safety, data should be entered as soon as reasonably feasible after a visit occurs.

13.4 Data Analysis

The Sponsor, PIs and Product Developers will agree on how data will be analyzed and presented prior to unblinding of the study.

The DCC will conduct the data analysis and will provide interim safety and final study reports for the Sponsor, Principal Investigators, the PSRT and SMC and the regulatory authorities, as appropriate.

14.0 STATISTICAL CONSIDERATIONS

14.1 Safety and Tolerability Analysis

14.1.1 Sample Size

The sample size for safety and tolerability analysis will be 30-48 participants according to the dose escalation design used to characterize the safety profile of one IV infusion of PGT121 mAb, at one of three dose levels, to HIV-uninfected and HIV-infected individuals (groups 1 and 2).

14.1.2 Null Hypothesis

As this is an exploratory proof of concept trial and analysis will be descriptive, no formal null hypothesis will be tested.

14.1.3 Statistical Power and Analysis and Dose Escalation Rules

The frequency of moderate or greater local and systemic reactogenicity events will be determined and compared between groups.

The frequency of SAEs judged possibly, probably or related to the IP will be determined.

All AEs will be analyzed and, grouped by seriousness, severity and relationship to the Investigational Product (as judged by the investigator).

For life-threatening adverse events related to Investigational Product: if none of the 12 (max 18) participants receiving Investigational Products experience such reactions, then

the 95 % upper confidence bound for the rate of these adverse events in the population is 26.5% (or 18.5% if n=18).

All AEs will be analysed and grouped by seriousness, severity and relationship to the IP (as judged by the investigator).

For life-threatening adverse events related to IP: if none of the 12 (max 18) participants in either Group 1 or Group 2 who receive the IP experience such reactions then the 95% upper confidence bound for the rate of these adverse events in the population is 26.5% (or 18.5% if n=18).

An interim analysis of group data will be carried out according to the study schema (Table 5.3.1) without unblinding the study to investigators or participants. At the end of the study, a full analysis will be prepared.

Based on previous experience with IAVI Phase 1 IP studies, it is expected that the amount of missing, unused or spurious data will be insignificant. Unused and spurious data will be listed separately and excluded from the statistical analysis. Missing data will be excluded from the statistical analysis.

14.2 Pharmacokinetic Analysis

14.2.1 Sample Size

The sample size for pharmacokinetic analysis will be 4 per dose subgroup, sufficient to provide sufficient information for the planned analyses.

14.2.2 Null Hypothesis

As this is an exploratory proof of concept trial and analysis will be descriptive, no formal null hypothesis will be tested.

14.2.3 Statistical Power and Analysis

Disposition of PGT121 mAb will be evaluated in this study. Based on the PK profile of other human monoclonal antibodies, it is expected that the half-life of PGT121 mAb will be 14 to 21 days. Previously published data indicates that the pharmacokinetics of PGT121 and 3BNC117 are fairly similar across a non-human primate cohort and within the same non-human primate (clearance of 3BNC117 appears to be marginally faster than that for PGT121).

Commonly reported PK parameters will be calculated using standard non-compartmental slope/height/area/moment (SHAM) analysis methods. Summary descriptive results of PK parameters, including AUC, Cmax, T1/2, and clearance results will be reported by dose cohort. Dose normalized plots of PK parameters will be presented. Correlation between PK and reported safety and pharmacodynamic outcomes will also be explored parameters in order to examine exposure-effect relationships.

A more powerful exploratory analysis to quantitatively determine the dose, participant and disease impact on PGT121 mAb pharmacokinetics, and correlate exposure with response, while correctly accounting for variance based on population intrinsic factors such as weight and gender will be performed. Using the proposed population analysis approach we will be able to simultaneously examine the magnitude and the rate of change to PGT121 disposition driven by HIV-1 RNA levels and/or ART, and also examine the magnitude and the rate of decline in log copies/ml of HIV-1 RNA plasma levels from baseline.

The frequency and levels of anti-PGT121 antibodies will be calculated and tabulated.

14.3 Virologic Analysis for Dose De-escalation in Groups 3A-C

14.3.1 Sample Size

The sample size for virologic analysis in Groups 3A-C will be 24-36 participants according to the dose de-escalation design described below.

14.3.2 Null Hypothesis

The null hypothesis is that there is no difference in antiviral activity between PGT121 mAb and placebo.

14.3.3 Statistical Power and Analysis

The virologic analysis described in this section relates to Groups 3A-C of the study design, in which dose de-escalation is performed in an adaptive study design in HIV-infected participants off ART with plasma HIV RNA levels of 2 x 10^3 – 10^5 copies/ml. This section assumes that Part 1 of the study has successfully demonstrated that there is a safe dose level of the IP such that the study is carried forward into Part 2.

The primary efficacy outcome for this analysis is defined as change in log10 viral load between Day 0 (day of infusion) and Day 7. The minimum clinically significant value for this outcome is defined as a difference of -0.9 log10.

The study plan for Groups 3A-C is designed so that the IP dose level may be de-escalated in a stepwise manner from the highest dose to the lowest dose, until a given dose level cannot be concluded to be efficacious. If any given dose level is proven to be efficacious at an interim analysis, enrolment for that dose level may cease, and the next lowest dose group may be enrolled. In the unlikely event that IP administration leads to increased viral load, this may be detected by this design. No placebo participants are enrolled as part of this design.

This design represents a dose de-escalation beginning at 30 mg/kg. The actual starting dose will be the MTD as determined by the SMC based on data from Part 1, therefore the starting dose may be 30mg/kg, 10 mg/kg or 3 mg/kg. If the starting dose is 30 mg/kg, then de-escalation will begin with Group 3A. If the starting dose is 10 mg/kg, then de-escalation will begin with Group 3B. If the starting dose is 3 mg/kg, then only Group 3C will be enrolled.

Assuming the starting dose is 30 mg/kg, an interim analysis of Group 3A will be performed after all 6 participants have reached 7 days following IP administration. The following scenarios may then occur, depending on the data:

- If the mean response in the first 6 participants is a decrease greater than or equal to -0.9 log10 HIV RNA, the IP will be determined to be effective at 30 mg/kg, enrollment into Group 3A will cease, and enrollment into Group 3B will begin.
- If the mean response in the first 6 participants is a decrease smaller than -0.9 log10 HIV RNA, then an additional 3 participants will be enrolled into Group 3A. After the additional 3 participants have reached 7 days following IP administration, an analysis of Group 3A (N=9) will be performed:
    - If the mean response in all Group 3A participants is a decrease greater than or equal to -0.9 log10 HIV RNA, the IP will be determined to be effective at 30 mg/kg, and enrollment into Group 3B will begin.
    - If the mean response in all 9 participants is a decrease smaller than -0.9 log10 HIV RNA, then the IP will be determined to be ineffective at 30 mg/kg and Groups 3B and 3C will not be enrolled. In this scenario, no dose of IP will be determined to be effective.

If 30 mg/kg is determined to be an effective dose, then Group 3B will be enrolled at 10 mg/kg. An interim analysis of Group 3B will be performed after 8 participants have reached 7 days following IP administration. The following scenarios may then occur, depending on the data:

- If the mean response in the first 8 participants is a decrease greater than or equal to -0.9 log10 HIV RNA, the IP will be determined to be effective at 10 mg/kg, enrollment into Group 3B will cease, and enrollment into Group 3C will begin.
- If the mean response in the first 8 participants is a decrease smaller than -0.9 log10 HIV RNA, then an additional 4 participants will be enrolled into Group 3B. After the additional 4 participants have reached 7 days following IP administration, an analysis of Group 3B (N=12) will be performed:
    - If the mean response in all Group 3B participants is a decrease greater than or equal to -0.9 log10 HIV RNA, the IP will be determined to be effective at 10 mg/kg, and enrollment into Group 3C will begin.
    - If the mean response in all 12 participants is a decrease smaller than -0.9 log10 HIV RNA, then the IP will be determined to be ineffective at 10 mg/kg, and Group 3C will not be enrolled. In this scenario, the minimum effective dose will be determined to be 30 mg/kg.

If 10 mg/kg is determined to be an effective dose, then Group 3C will be enrolled at 3 mg/kg. An interim analysis of Group 3C will be performed after 10 participants have reached 7 days following IP administration. The following scenarios may then occur, depending on the data:

- If the mean response in the first 10 participants is a decrease greater than or equal to -0.9 log10 HIV RNA, the IP will be determined to be effective at 3 mg/kg and enrollment into Group 3C will cease. In this scenario, the minimum effective dose of the IP will be determined to be 3 mg/kg.
- If the mean response in the first 10 participants is a decrease smaller than -0.9 log10 HIV RNA, then an additional 5 participants will be enrolled into Group 3C.

After the additional 5 participants have reached 7 days following IP administration, an analysis of Group 3C (N=15) will be performed:

- If the mean response in all Group 3C participants is a decrease greater than or equal to -0.9 log10 HIV RNA, the minimum effective dose will be determined to be 3 mg/kg.
- If the mean response in all 15 participants is a decrease smaller than -0.9 log10 HIV RNA, then the IP will be determined to be ineffective at 3 mg/kg. In this scenario, the minimum effective dose will be determined to be 10 mg/kg.

For the analysis of sample size and power, log10 viral load differences from baseline for each participant were simulated from a normal distribution, with a standard deviation of 0.5. This value was chosen by examining a study of the antiretroviral drug raltegravir, which demonstrated a mean estimated standard deviation of the change of baseline of 0.47[18]. This is a conservative estimate, as the variability of viral loads near the lower range might be expected to also be lower.

The statistical test performed will be the Signed-ranktest, which will incorporate the "shift" parameter of -0.9 log10 (the minimum clinically significant difference selected for this study). An evaluation of potential harm (increased viral load) will also be performed with the Signed ranktest; this test will examine the null hypothesis of no change in viral load (a shift of 0.0 log10 following IP administration) against the one-sided alternative hypothesis that the viral load is increased following IP administration. Each efficacy test will be performed at the level $\alpha = 0.05$. Each test for harm will be performed at level $2\alpha = 0.10$, in order to provide additional sensitivity to detect potential harm.

14.4 Analysis of Antiviral Activity and Dose De-escalation in Subgroups 3D-F

14.4.1 Sample Size

The sample size for antiviral activity will be 3-9 participants, depending on the MTD.

14.4.2 Null Hypothesis

As this is an exploratory proof of concept trial and analysis will be descriptive in this population, no formal null hypothesis will be tested.

14.4.3 Statistical Power and Analysis

No efficacy endpoints will be tested in Groups 3D-F as participants are HIV-infected with low viral loads at baseline (10^2 – 2×10^3 copies/ml). Immunologic and virologic endpoints will be determined as described in Section 4.1. As soon as subgroup 3D has enrolled 3 participants, enrollment into subgroup 3E will be initiated. The same rule will apply for progression from subgroup 3E to 3F. In the event that dose de-escalation in subgroups 3A-C is stopped due to failure to meet virologic endpoints, dose de-escalation in subgroups 3D-F will continue.

14.5 Secondary and Exploratory Immunologic and Virologic Analyses

14.5.1 Sample Size

The sample size for secondary and exploratory immunologic and virologic analysis will be 63-93 participants.

14.5.2 Null Hypothesis

No formal hypothesis on immunologic or virologic responses will be tested, with the exception of the change in viral load described in Section 14.3.

14.5.3 Statistical Power and Analysis

Descriptive statistics (actual values and changes from reference) will be calculated for continuous immunologic and virologic parameters at all time points. Graphical representations of changes in parameters will be made as applicable. Differences between groups at a specific time point will be tested for exploratory purposes by a 2-sample t-test if the data appear to be normally distributed. If not, the non-parametric Wilcoxon rank sum test will be used. If portions of the measurements are censored below the assay quantification limit, the Gehan-Wilcoxon test will be employed. All statistical tests will be two-sided and will be considered statistically significant if $p < 0.05$.

Frequency tabulations will be calculated for discrete (qualitative) immunologic and virologic parameters at all time points. Significant differences between groups will be determined by a 2-sided Fisher's exact test.

Interim immunologic and virologic analyses of grouped data may be performed without unblinding the study to investigators or participants.

15.0 QUALITY CONTROL AND QUALITY ASSURANCE

To ensure the quality and reliability of the data collected and generated and the ethical conduct of this study, a Study Operations Manual (SOM) will be developed. All deviations will be reported and investigated. The SOM describes reporting and deviation documentation requirements and procedures.

Regular monitoring will be performed according to ICH-GCP as indicated in Section 17.5.

An independent audit of the study and study sites may be performed by the Sponsor or designee to establish the status of applicable quality systems. Inspection by regulatory authorities may also occur.

By signing the protocol, the Principal Investigators agree to facilitate study related monitoring, audits, IRB/IEC review and regulatory inspection(s) and direct access to source documents. Such information will be treated as strictly confidential and under no circumstances be made publicly available.

16.0 DATA AND BIOLOGICAL MATERIAL

All data and biological material collected through the study shall be managed in accordance with the Clinical Trial Agreement (CTA). Distribution and use of these data will be conducted by agreement of all parties.

The computerized raw data generated will be held by the DCC on behalf of the Sponsor. The study sites will also hold the final data files and tables generated for the purpose of analysis.

17.0 ADMINISTRATIVE STRUCTURE

The Principal Investigator will be responsible for all aspects of the study at the study site.

17.1 Protocol Safety Review Team

A PSRT will be formed to monitor the clinical safety data. During the administration of IP phase of the trial, the PSRT will review the clinical safety data on a weekly basis via electronic distribution of reports. An ad hoc PSRT review meeting will occur if any of the members of the PSRT requests a special review to discuss a specific safety issue or as specified in the Study Operations Manual. After the administration of IP phase the PSRT will review the clinical safety data at least monthly.

The PSRT will consist of the IAVI Medical Monitor(s), and the PI or designee from each clinical team. The study chair or an IAVI Medical Monitor may be the PSRT chair. *Ex officio* members will include the IAVI Chief Medical Officer and an unblinded IAVI Medical Monitor.

Additional PSRT participants may include the following, as needed:

- Co-investigators and trial site senior clinical research nursing staff
- Laboratory directors
- Data management, study statistician and regulatory staff

The PSRT membership and procedures are detailed in the PSRT charter.

17.2 Safety Monitoring Committee (SMC)

The SMC will consist of independent clinicians/scientists/statisticians/ethicists who are not involved in the study. Investigators responsible for the clinical care of participants or representative of the Sponsor may not be a member of the SMC. Details of membership, chair and co-chair and responsibilities are outlined in the SMC charter.

Principal Investigator(s) or designee and/or a Sponsor representative may be asked to join an open session of the SMC meeting to provide information on study conduct, present data or to respond to questions.

Safety data will be reviewed by the SMC at pre-specified time points and at an ad-hoc basis.

17.2.1 Content of Interim Safety Review

The SMC will be asked to review the following blinded data:

- Summary of reactogenicity (i.e., solicited adverse events)
- All adverse events judged by the Principal Investigator or designee to be possibly, probably or definitely related to IP
- All laboratory results confirmed on retest and judged by the Principal Investigator or designee to be clinically significant

- All SAEs

An unblinded presentation of all above noted events may also be made available for the SMC for their review if required by any member of the SMC.

17.2.2 SMC Review of Group 1 and 2 data prior to starting Group 3

Following IV infusion of IP of the last participant in Groups 1 and 2, the Safety Monitoring Committee (SMC) will review safety data through the day 14 post-IV infusion visit for all participants to confirm MTD in each group, and determine whether, and at what dose level, Group 3 can initiate enrollment.

17.3 Criteria for Pausing the Study

Enrollment and administration of IP will be stopped and a safety review conducted by the SMC for any of the following criteria:

1. One or more participants experience an SAE that is judged possibly, probably or definitely related to IP.
2. There is a participant death, regardless of relationship to the IP.
3. Two or more participants experience Grade 3 adverse events in the same category System Organ Class that are considered to be at least possibly related to IP or
4. Any grade 4 adverse event that is considered to be at least possibly related to IP.

Table 2: AE notification and safety pause/AE review rules

Event and relationship to study product	Severity	Occurrence	Site PI action	PSRT or SMC action
SAE, related[1]	Any	Any	Phone, email or fax forms to sponsor within 24 hours	Study pause within 24 hours, refer to SMC for review
SAE, not related[2]	Death	Any	Phone, email or fax forms to sponsor within 24 hours	Study pause within 24 hours, refer to SMC for review
AE[3], related	Grade 3 or 4[4]	Second[5]	Phone, email or fax notification to sponsor within 24 hours	Study pause within 24 hours, refer to SMC for review
AE[3], related	Grade 3 or 4[4]	First	Phone, email or fax notification to sponsor within 24 hours	PSRT review within 2 business days to consider pause

[1] Related SAE refers to SAE deemed to be definitely, probably, or possibly related to study vaccine.

[2] Not related SAE refers to SAE deemed to be probably not related or not related to the study vaccine.

[3] Does not include the following reactogenicity symptoms (fever, fatigue/malaise, myalgia, arthralgia, chills, headache, nausea, vomiting).

[4] If no evidence of disease is present other than an abnormal laboratory value, the test must be repeated (entailing blood re-draw) at least one time. The verification period will be a maximum of 72 hours after initial awareness of the abnormal laboratory value. When signs and symptoms are present, repeat test will not be needed.

[5] PSRT will determine whether the reported related AE (Grade 3 or 4) is a second occurrence of a previously reported AE (Grade 3 or 4).

The Sponsor will request a review by the SMC, (or the SMC chair if other SMC members cannot be convened), to be held within 2 business days of the Sponsor learning of the event. The individual participant(s)/or study may be unblinded at the discretion of the SMC.

Following this review, the SMC will make a recommendation regarding the continuation or suspension of the administration of the IP or the trial and communicate this decision immediately to the Sponsor. The Sponsor then will inform the Principal Investigators without delay.

Additional *ad hoc* review may be specifically requested by the Sponsor, the Principal Investigator(s) or by the SMC.

17.4 Study Supervision

The SMC, the IAVI Chief Medical Officer (CMO) and the IAVI Medical Monitor(s) have access to progress report(s) of this study. Close cooperation will be necessary to track study progress, respond to queries about proper study implementation and management, address issues in a timely manner, and assure consistent documentation, and share information effectively. Rates of accrual, retention, and other parameters relevant to the site's performance will be regularly and closely monitored by the study team.

17.5 Study Monitoring

On-and/or off-site monitoring will ensure that the study is conducted in compliance with human subjects' protection and other research regulations and guidelines, recorded and reported in accordance with the protocol, is consistent with SOPs, GCP, applicable regulatory requirements and locally accepted practices. The monitor will confirm the quality and accuracy of data at the site by validation of CRFs against the source documents, such as clinical records. The investigators, as well as participants through consenting to the study, agree that the monitor may inspect study facilities and source records (e.g., informed consent forms, clinic and laboratory records, other source documents), as well as observe the performance of study procedures (in accordance with site IRB requirements). Such information will be treated as strictly confidential and will under no circumstances be made publicly available.

The monitoring will adhere to GCP guidelines. The Principal Investigator will permit inspection of the facilities and all study-related documentation by authorized representatives of IAVI, and Government and Regulatory Authorities responsible for this study.

17.6 Investigator's Records

Study records include administrative documentation—e.g., reports and correspondence relating to the study—as well as documentation related to each participant screened and/or enrolled in the study—including informed consent forms, case report forms, and all other source documents. The investigator will maintain and store, in a secure manner, complete, accurate, and current study records for a minimum of 2 years after marketing application approval or the study is discontinued and applicable national and local health authorities are notified. IAVI will notify the Principal Investigator of these events.

18.0 INDEMNITY

The Sponsor and Institution are responsible to have appropriate liability insurance. For research-related injuries and/or medical problems determined to result from receiving the IP, treatment including necessary emergency treatment and proper follow-up care will be made available to the participant free of charge at the expense of the Sponsor.

19.0 PUBLICATION

A primary manuscript describing safety, anti-viral effect and immune responses in this trial will be prepared promptly after the data analysis is available.

Authors will be representatives of each trial site, the data management and statistical analysis center, the laboratories, the product developer and the sponsor, participant to the generally accepted criteria of contributions to the design and conduct of the study, the analysis of data and writing of the manuscript. Precedence will be given to authors from the site enrolling the greatest number of participants. Manuscripts will be reviewed by representatives of each participating group as specified in the CTA.

20.0 ETHICAL CONSIDERATIONS

The Principal Investigator will ensure that the study is conducted in compliance with the protocol, SOPs in accordance with guidelines formulated by the ICH for GCP in clinical studies, the ethical principles that have their origins in the Declaration of Helsinki and applicable local standards and regulatory requirements.

APPENDIX A: SCHEDULE OF PROCEDURES

**Study Month**		**0**								**1**		**2**		**3**	**4**	**5**	**6**
**Study Week**		**0**				**1**		**2**	**3**	**4**	**6**	**8**	**10**	**12**	**16**	**20**	**24**
**Study Day**	**Scr**	**0**	**1**	**2**	**3**	**7**	**10[5]**	**14**	**21**	**28**	**42**	**56**	**70**	**84**	**112**	**140**	**168/ET[9]**
**Visit Windows (Days)**	**-42**	**0**	**0**	**0**	**0**	**± 1**	**0**	**± 2**	**± 2**	**± 2**	**± 3**	**± 3**	**±3**	**±7**	**±7**	**±7**	**±7**
**INVESTIGATIONAL PRODUCT**																	
Investigational Product /Placebo		**X**															
**CONSENT/ASSESSMENTS/COUNSELLING**																	
Informed Consent	**X**																
Assessment of Understanding	**X**																
HIV Risk Assessment[3]		**X**															**X**
HIV Risk Reduction Counselling[2]	**X**	**X**								**X**		**X**		**X**	**X**	**X**	**X**
HIV-test Counselling[3]	**X**	**X**															**X**
ART counseling[5]	**X**	**X**										**X**					**X**
Family Planning Counselling	**X**	**X**															
Social Impact Assessment																	**X**
**CLINICAL SAFETY ASSESSMENTS**																	
Comprehensive Medical History	**X**																
Interim Medical History		**X**	**X**	**X**	**X**	**X**	**X**	**X**	**X**	**X**	**X**	**X**					
Concomitant Medications	**X**	**X**	**X**	**X**	**X**	**X**	**X**	**X**	**X**	**X**	**X**	**X**					
General Physical Exam	**X**																**X**
Directed Physical Exam		**X**	**X**	**X**	**X**	**X**	**X**	**X**	**X**	**X**	**X**	**X**	**X**	**X**	**X**	**X**	
Weight	**X**	**X**															**X**
Height	**X**																
Vital Signs	**X**	**X[4]**	**X**	**X**	**X**	**X**	**X**	**X**	**X**	**X**	**X**	**X**	**X**	**X**	**X**	**X**	**X**
Local & Systemic Reactogenicity		**X[4]**	**X**	**X**	**X**												
Adverse Events		**X**	**X**	**X**	**X**	**X**	**X**	**X**	**X**	**X**	**X**	**X**					
Serious Adverse Events and pIMD	**X**	**X**	**X**	**X**	**X**	**X**	**X**	**X**	**X**	**X**	**X**	**X**	**X**	**X**	**X**	**X**	**X**

Study Month		0								1		2		3	4	5	6
**Study Week**		**0**				**1**		**2**	**3**	**4**	**6**	**8**	**10**	**12**	**16**	**20**	**24**
**Study Day**	**Scr**	**0**	**1**	**2**	**3**	**7**	**10[5]**	**14**	**21**	**28**	**42**	**56**	**70**	**84**	**112**	**140**	**168/ET[9]**
**Visit Windows (Days)**	**-42**	**0**	**0**	**0**	**0**	**± 1**	**0**	**± 2**	**± 2**	**± 2**	**± 3**	**± 3**	**±3**	**±7**	**±7**	**±7**	**±7**
**CLINICAL LABORATORY TESTS**																	
Hematology and Coagulation	X	X[13]	X		X	X		X		X		X		X	X	X	X
CD4[1]	X	X[13]				X		X		X		X					X
Clinical Chemistry	X	X[13]	X		X	X		X		X		X		X	X	X	X
Urine Dipstick[11]	X	X[13]	X		X	X		X		X		X		X	X	X	X
Urine Pregnancy test	X	X[13]								X		X		X			X
Active Syphilis	X																
Hepatitis B	X																
Hepatitis C	X																
HIV diagnostic (4th generation Ag/Ab test)[3]	X	X[13]								X							X
HIV Viral Load[1]	X	X[13]	X	X	X	X	X	X	X	X	X	X	X	X	X	X	X
**RESEARCH LABORATORY TESTS**	-	-	-	-	-	-	-	-	-	-	-	-	-	-	-	-	-
Anti PGT121 Antibodies (ADA)		X[13]								X		X		X			X
HIV testing for PGT121 susceptibility[6]	X																X
HIV SGA sequencing[12]	X									X							X
HIV genotypic testing for ART resistance[12]	X									X							X
HIV reservoir size assessment[1]	X							X						X			
Humoral Assays[7]		X[13]			X	X		X		X		X		X			X
Cellular Assays[7]		X[13]				X		X		X		X		X			X
HLA typing		X[13]															
**PHARMACOKINETICS PGT121 ELISA**	X[7]	X[8]	X	X	X	X		X	X	X	X	X	X	X	X	X	X
**MUCOSAL SAMPLING**		X[10]	X			X		X									
**PLASMA/SERUM STORAGE**	X	X	X	X	X	X	X	X	X	X		X		X			X

Study Month		0								1		2		3	4	5	6
**Study Week**		**0**				**1**		**2**	**3**	**4**	**6**	**8**	**10**	**12**	**16**	**20**	**24**
**Study Day**	**Scr**	**0**	**1**	**2**	**3**	**7**	**10**[5]	**14**	**21**	**28**	**42**	**56**	**70**	**84**	**112**	**140**	**168/ET**[9]
**Visit Windows (Days)**	**-42**	**0**	**0**	**0**	**0**	**± 1**	**0**	**± 2**	**± 2**	**± 2**	**± 3**	**± 3**	**±3**	**±7**	**±7**	**±7**	**±7**
**PBMCs STORAGE**		**X**										**X**		**X**			**X**

1. For groups 2 and 3 only
2. Group 1: will receive HIV risk reduction counselling; Groups 2 and 3: HIV risk reduction counselling as secondary prevention to reduce onward transmission
3. Group 1 only
4. At baseline, approximately 30 minutes after IP administration start, and at hours 1 through 12 after IV infusion start. Local and systemic reactogenicity will be assessed by clinic staff at visits on study days 1, 2 and 3. Local and Systemic reactogenicity will also be assessed by the participant using the Memory Aid on study days 1, 2, and 3
5. Group 3 only
6. Baseline assessment of participants autologous HIV for neutralization susceptibility to PGT121 in-vitro (group 3 only).
7. See Laboratory Analytical Plan for details
8. Day 0 PK draws done immediately before IP administration, at the end of the IV infusion of IP, and 30 minutes and 3 hours post end of the IP administration. Additional PK draws on day 0 are done 6, 9 and 12 hours after the start of the IV infusion of IP. See SOM for details
9. Early Termination (ET): Procedures to be performed at ET are the same as last visit procedures
10. Cervico-vaginal and/or rectal mucosal sampling (optional) on Day 0 must be done prior to IV infusion of IP. See SOM for details
11. Urinalysis for group 3 will only be conducted at visits after screening if clinically indicated.
12. HIV envelope single genome amplification (SGA) sequencing and ART resistance genotyping will be performed in all subjects of group 3 and in subjects of group 2 only if they develop detectable plasma viremia >1000 HIV cp/ml during the study.
13. Day 0 sample collections for laboratory tests must be done pre-infusion.

APPENDIX B: LOW RISK CRITERIA

Low risk will be defined as:

1. **SEXUAL BEHAVIORS**

   In the **last 12 months** did not:

   - Have oral, vaginal or anal intercourse with an HIV-infected partner, or partner who uses injection drugs.
   - Gave or receive money, drugs, gifts, or services in exchange for oral, vaginal or anal sex

   AND

   In the **last 6 months** has abstained from penile/anal or penile/vaginal intercourse

   OR

   In the **last 6 months**:

   - Had 4 or fewer partners of the opposite birth sex for vaginal and/or anal intercourse, OR

Is MSM (person born male with partner(s) born male) who, in the **last 12 months**:

- Had 2 or fewer MSM partners for anal intercourse and had no unprotected anal sex with MSM, OR
- Had unprotected anal intercourse with only 1 MSM partner, within a monogamous relationship lasting at least 12 months (during which neither partner had any other partners). If the monogamous relationship ended, the participant may then have had protected anal intercourse with 1 other MSM partner (total 2 or fewer partners in the last 12 months).

Is a transgender person, regardless of the point on the transition spectrum, having sex with men (born male) and/or other transgender persons, who in the last 12 months:

- Had 2 or fewer partners for anal or vaginal intercourse, and had no unprotected anal or vaginal sex, OR
- Had unprotected anal or vaginal intercourse sex with 1 partner only within a monogamous relationship lasting at least 12 months (during which neither partner had any other partners). If the monogamous relationship ended, may then have had protected anal or vaginal sex with one other partner (total 2 or fewer partners in the last 12 months).

AND

Uses or intends to use condoms in situations which may include penile/anal or penile/vaginal intercourse with new partners of unknown HIV status, occasional partners, partners outside a primary relationship, and/or partners known to have other partners.

2. **NON-SEXUAL BEHAVIORS**

   In the **last 12 months** did not:

   - Inject drugs or other substances without a prescription
   - Use cocaine, methamphetamine, or excessive alcohol, which in the investigator's judgement, rendered the participant at greater than low risk for acquiring HIV infection

The investigator's judgement should consider local epidemiologic information about HIV prevalence in the area and community networks.

A participant is NOT appropriate for inclusion if he/she:

Acquired an STI (ie, new infection) in the last 12 months:

- Syphilis
- Gonorrhea
- Non-gonococcal urethritis
- HSV-2
- Chlamydia
- Pelvic inflammatory disease (PID)
- Trichomonas
- Mucopurulent cervicitis
- Epididymitis
- Proctitis
- Lymphogranuloma venereum
- Chancroid
- Hepatitis B

APPENDIX C: ADVERSE EVENT SEVERITY ASSESSMENT TABLE

Adapted from: DIVISION OF AIDS (DAIDS) TABLE FOR GRADING THE SEVERITY OF ADULT AND PEDIATRIC ADVERSE EVENTS Version 2.0, November 2014

The *Division of AIDS* (*DAIDS) Table for Grading the Severity of Adult and Pediatric Adverse Events, Version 2.0* consists of parameters, or AEs, with severity grading guidance that are to be used in DAIDS clinical trials for safety data reporting to maintain accuracy and consistency in the evaluation of AEs.

I. Instructions and Clarifications

Grading Adult and Pediatric AEs
When a single parameter is not appropriate for grading an AE in both adult and pediatric populations, separate parameters with specified age ranges are provided. If no distinction between adult and pediatric populations has been made, the listed parameter should be used for grading an AE in both populations.

Note: The term "severe" is not the same as "serious" in classifying AEs. The severity of a specific event describes its intensity, and it is the intensity which is graded. Seriousness, which is not graded, relates to an outcome of an AE and is a regulatory definition.

Estimating Severity Grade for Parameters Not Identified in the Table
The functional table below should be used to grade the severity of an AE that is not specifically identified in the grading table. In addition, all deaths related to an AE are to be classified as grade 5.

PARAMETER	GRADE 1 MILD	GRADE 2 MODERATE	GRADE 3 SEVERE	GRADE 4 POTENTIALLY LIFE-THREATENING
**Clinical** adverse event **NOT** identified elsewhere in the grading table	Mild symptoms causing no or minimal interference with usual social & functional activities with intervention not indicated	Moderate symptoms causing greater than minimal interference with usual social & functional activities with intervention indicated	Severe symptoms causing inability to perform usual social & functional activities with intervention or hospitalization indicated	Potentially life-threatening symptoms causing inability to perform basic self-care functions with intervention indicated to prevent permanent impairment, persistent disability, or death

Determining Severity Grade for Parameters "Between Grades"
If the severity of an AE could fall in either one of two grades (e.g., the severity of an AE could be either Grade 2 or Grade 3), sites should select the higher of the two grades.

Laboratory Values
General. An asymptomatic, abnormal laboratory finding without an accompanying AE should not be reported to DAIDS in an expedited timeframe unless it meets protocol-specific reporting requirements. Sites should refer to the applicable network standards for reporting abnormal laboratory findings on the CRFs.

Values Below Grade 1
Any laboratory value that is between the ULN and grade 1 (for high values) or the LLN and grade 1 (for low values) should not be graded or reported as an AE. Sites should consult the Manual for Expedited Reporting of Adverse Events to DAIDS, Version 2.0 and their protocol when making an assessment of the need to report an AE.

Overlap of Local Laboratory Normal Values with Grading Table Ranges.
When local laboratory normal values fall within grading table laboratory ranges, the severity grading is based on the ranges in the grading table unless there is a protocol-specific grading criterion for the laboratory value. For example, "Magnesium, Low" has a grade 1 range of 1.2 to < 1.4 mEq/L, while a

particular laboratory's normal range for magnesium may be 1.3 to 2.8 mEq/L. If a study participant's magnesium laboratory value is 1.3 mEq/L, the laboratory value should be graded as grade 1.

II. Definitions of terms used in the Table:

Basic Self-care Functions	Adult<br>Activities such as bathing, dressing, toileting, transfer or movement, continence, and feeding.<br><br>Young Children<br>Activities that are age and culturally appropriate, such as feeding one's self with culturally appropriate eating implements.
LLN	Lower limit of normal
Intervention	Medical, surgical, or other procedures recommended or provided by a healthcare professional for the treatment of an adverse event.
NA	Not Applicable
ULN	Upper limit of normal
Usual Social & Functional Activities	Activities which adults and children perform on a routine basis and those which are part of regular activities of daily living, for example:<br><br>Adults<br>Adaptive tasks and desirable activities, such as going to work, shopping, cooking, use of transportation, or pursuing a hobby.<br><br>Young Children<br>Activities that are age and culturally appropriate, such as social interactions, play activities, or learning tasks.

Major Clinical Conditions

Cardiovascular

PARAMETER	GRADE 1 MILD	GRADE 2 MODERATE	GRADE 3 SEVERE	GRADE 4 POTENTIALLY LIFE-THREATENING
**Arrhythmia** (by ECG or physical examination) *Specify type, if applicable*	No symptoms AND No intervention indicated	No symptoms AND Non-urgent intervention indicated	Non-life-threatening symptoms AND Non-urgent intervention indicated	Life-threatening arrhythmia OR Urgent intervention indicated
**Blood Pressure Abnormalities**[1] ***Hypertension*** *(with the lowest reading taken after repeat testing during a visit)* *≥ 18 years of age*	140 to < 160 mmHg systolic OR 90 to < 100 mmHg diastolic	≥ 160 to < 180 mmHg systolic OR ≥ 100 to < 110 mmHg diastolic	≥ 180 mmHg systolic OR ≥ 110 mmHg diastolic	Life-threatening consequences in a participant not previously diagnosed with hypertension (e.g., malignant hypertension) OR Hospitalization indicated
< 18 years of age	> 120/80 mmHg	≥ 95th to < 99th percentile + 5 mmHg adjusted for age, height, and gender (systolic and/or diastolic)	≥ 99th percentile + 5 mmHg adjusted for age, height, and gender (systolic and/or diastolic)	Life-threatening consequences in a participant not previously diagnosed with hypertension (e.g., malignant hypertension) OR Hospitalization indicated
Hypotension	No symptoms	Symptoms corrected with oral fluid replacement	Symptoms AND IV fluids indicated	Shock requiring use of vasopressors or mechanical assistance to maintain blood pressure
**Cardiac Ischemia or Infarction** *Report only one*	NA	NA	New symptoms with ischemia (stable angina) OR New testing consistent with ischemia	Unstable angina OR Acute myocardial infarction
**Heart Failure**	No symptoms AND Laboratory or cardiac imaging abnormalities	Symptoms with mild to moderate activity or exertion	Symptoms at rest or with minimal activity or exertion (e.g., hypoxemia) OR Intervention indicated (e.g., oxygen)	Life-threatening consequences OR Urgent intervention indicated (e.g., vasoactive medications, ventricular assist device, heart transplant)
**Hemorrhage** (with significant acute blood loss)	NA	Symptoms AND No transfusion indicated	Symptoms AND Transfusion of ≤ 2 units packed RBCs indicated	Life-threatening hypotension OR Transfusion of > 2 units packed RBCs (for children, packed RBCs > 10 cc/kg) indicated

[1] Blood pressure norms for children < 18 years of age can be found in: Expert Panel on Integrated Guidelines for Cardiovascular Health and Risk Reduction in Children and Adolescents. *Pediatrics* 2011;128;S213; originally published online November 14, 2011; DOI: 10.1542/peds.2009- 2107C.

PARAMETER	GRADE 1 MILD	GRADE 2 MODERATE	GRADE 3 SEVERE	GRADE 4 POTENTIALLY LIFE-THREATENING
**Prolonged PR Interval or AV Block**<br>*Report only one*<br>*> 16 years of age*	PR interval 0.21 to < 0.25 seconds	PR interval ≥ 0.25 seconds OR Type I 2nd degree AV block	Type II 2nd degree AV block OR Ventricular pause ≥ 3.0 seconds	Complete AV block
≤ 16 years of age	1st degree AV block (PR interval > normal for age and rate)	Type I 2nd degree AV block	Type II 2nd degree AV block OR Ventricular pause ≥ 3.0 seconds	Complete AV block
**Prolonged QTc Interval**[2]	0.45 to 0.47 seconds	> 0.47 to 0.50 seconds	> 0.50 seconds OR ≥ 0.06 seconds above baseline	Life-threatening consequences (e.g., Torsade de pointes, other associated serious ventricular dysrhythmia)
**Thrombosis or Embolism**<br>*Report only one*	NA	Symptoms AND No intervention indicated	Symptoms AND Intervention indicated	Life-threatening embolic event (e.g., pulmonary embolism, thrombus)

[2] As per Bazett's formula.

Dermatologic

PARAMETER	GRADE 1 MILD	GRADE 2 MODERATE	GRADE 3 SEVERE	GRADE 4 POTENTIALLY LIFE-THREATENING
**Alopecia** (scalp only)	Detectable by study participant, caregiver, or physician AND Causing no or minimal interference with usual social & functional activities	Obvious on visual inspection AND Causing greater than minimal interference with usual social & functional activities	NA	NA
**Bruising**	Localized to one area	Localized to more than one area	Generalized	NA
**Cellulitis**	NA	Non-parenteral treatment indicated (e.g., oral antibiotics, antifungals, antivirals)	IV treatment indicated (e.g., IV antibiotics, antifungals, antivirals)	Life-threatening consequences (e.g., sepsis, tissue necrosis)
**Hyperpigmentation**	Slight or localized causing no or minimal interference with usual social & functional activities	Marked or generalized causing greater than minimal interference with usual social & functional activities	NA	NA

**Hypopigmentation**	Slight or localized causing no or minimal interference with usual social & functional activities	Marked or generalized causing greater than minimal interference with usual social & functional activities	NA	NA
**Petechiae**	Localized to one area	Localized to more than one area	Generalized	NA
**Pruritus**[3] (without skin lesions)	Itching causing no or minimal interference with usual social & functional activities	Itching causing greater than minimal interference with usual social & functional activities	Itching causing inability to perform usual social & functional activities	NA
**Rash** *Specify type, if applicable*	Localized rash	Diffuse rash OR Target lesions	Diffuse rash AND Vesicles or limited number of bullae or superficial ulcerations of mucous membrane limited to one site	Extensive or generalized bullous lesions OR Ulceration of mucous membrane involving two or more distinct mucosal sites OR Stevens-Johnson syndrome OR Toxic epidermal necrolysis

[3] For pruritus associated with injections or infusions, see the *Site Reactions to Injections and Infusions* section (page 23).

Endocrine and Metabolic

PARAMETER	GRADE 1 MILD	GRADE 2 MODERATE	GRADE 3 SEVERE	GRADE 4 POTENTIALLY LIFE-THREATENING
**Diabetes Mellitus**	Controlled without medication	Controlled with medication OR Modification of current medication regimen	Uncontrolled despite treatment modification OR Hospitalization for immediate glucose control indicated	Life-threatening consequences (e.g., ketoacidosis, hyperosmolar non-ketotic coma, end organ failure)
**Gynecomastia**	Detectable by study participant, caregiver, or physician AND Causing no or minimal interference with usual social & functional activities	Obvious on visual inspection AND Causing pain with greater than minimal interference with usual social & functional activities	Disfiguring changes AND Symptoms requiring intervention or causing inability to perform usual social & functional activities	NA

**Hyperthyroidism**	No symptoms AND Abnormal laboratory value	Symptoms causing greater than minimal interference with usual social & functional activities OR Thyroid suppression therapy indicated	Symptoms causing inability to perform usual social & functional activities OR Uncontrolled despite treatment modification	Life-threatening consequences (e.g., thyroid storm)
**Hypothyroidism**	No symptoms AND Abnormal laboratory value	Symptoms causing greater than minimal interference with usual social & functional activities OR Thyroid replacement therapy indicated	Symptoms causing inability to perform usual social & functional activities OR Uncontrolled despite treatment modification	Life-threatening consequences (e.g., myxedema coma)
**Lipoatrophy**[4]	Detectable by study participant, caregiver, or physician AND Causing no or minimal interference with usual social & functional activities	Obvious on visual inspection AND Causing greater than minimal interference with usual social & functional activities	Disfiguring changes	NA
**Lipohypertrophy**[5]	Detectable by study participant, caregiver, or physician AND Causing no or minimal interference with usual social & functional activities	Obvious on visual inspection AND Causing greater than minimal interference with usual social & functional activities	Disfiguring changes	NA

[4] Definition: A disorder characterized by fat loss in the face, extremities, and buttocks.
[5] Definition: A disorder characterized by abnormal fat accumulation on the back of the neck, breasts, and abdomen.

Gastro-intestinal

PARAMETER	GRADE 1 MILD	GRADE 2 MODERATE	GRADE 3 SEVERE	GRADE 4 POTENTIALLY LIFE-THREATENING
**Anorexia**	Loss of appetite without decreased oral intake	Loss of appetite associated with decreased oral intake without significant weight loss	Loss of appetite associated with significant weight loss	Life-threatening consequences OR Aggressive intervention indicated (e.g., tube feeding, total parenteral nutrition)
**Ascites**	No symptoms	Symptoms AND Intervention indicated (e.g., diuretics, therapeutic paracentesis)	Symptoms recur or persist despite intervention	Life-threatening consequences

**Bloating or Distension**<br>*Report only one*	Symptoms causing no or minimal interference with usual social & functional activities	Symptoms causing greater than minimal interference with usual social & functional activities	Symptoms causing inability to perform usual social & functional activities	NA
**Cholecystitis**	NA	Symptoms AND Medical intervention indicated	Radiologic, endoscopic, or operative intervention indicated	Life-threatening consequences (e.g., sepsis, perforation)
**Constipation**	NA	Persistent constipation requiring regular use of dietary modifications, laxatives, or enemas	Obstipation with manual evacuation indicated	Life-threatening consequences (e.g., obstruction)
**Diarrhea**<br>*≥ 1 year of age*	Transient or intermittent episodes of unformed stools OR Increase of ≤ 3 stools over baseline per 24-hour period	Persistent episodes of unformed to watery stools OR Increase of 4 to 6 stools over baseline per 24-hour period	Increase of ≥ 7 stools per 24-hour period OR IV fluid replacement indicated	Life-threatening consequences (e.g., hypotensive shock)
< 1 year of age	Liquid stools (more unformed than usual) but usual number of stools	Liquid stools with increased number of stools OR Mild dehydration	Liquid stools with moderate dehydration	Life-threatening consequences (e.g., liquid stools resulting in severe dehydration, hypotensive shock)
**Dysphagia or Odynophagia**<br>*Report only one and specify location*	Symptoms but able to eat usual diet	Symptoms causing altered dietary intake with no intervention indicated	Symptoms causing severely altered dietary intake with intervention indicated	Life-threatening reduction in oral intake
**Gastrointestinal Bleeding**	Not requiring intervention other than iron supplement	Endoscopic intervention indicated	Transfusion indicated	Life-threatening consequences (e.g., hypotensive shock)

PARAMETER	GRADE 1<br>MILD	GRADE 2<br>MODERATE	GRADE 3<br>SEVERE	GRADE 4<br>POTENTIALLY LIFE-THREATENING
**Mucositis or Stomatitis**<br>*Report only one and specify location*	Mucosal erythema	Patchy pseudomembranes or ulcerations	Confluent pseudomembranes or ulcerations OR Mucosal bleeding with minor trauma	Life-threatening consequences (e.g., aspiration, choking) OR Tissue necrosis OR Diffuse spontaneous mucosal bleeding
**Nausea**	Transient (< 24 hours) or intermittent AND No or minimal interference with oral intake	Persistent nausea resulting in decreased oral intake for 24 to 48 hours	Persistent nausea resulting in minimal oral intake for > 48 hours OR Rehydration indicated (e.g., IV fluids)	Life-threatening consequences (e.g., hypotensive shock)

**Pancreatitis**	NA	Symptoms with hospitalization not indicated	Symptoms with hospitalization indicated	Life-threatening consequences (e.g., circulatory failure, hemorrhage, sepsis)
**Perforation** (colon or rectum)	NA	NA	Intervention indicated	Life-threatening consequences
**Proctitis**	Rectal discomfort with no intervention indicated	Symptoms causing greater than minimal interference with usual social & functional activities OR Medical intervention indicated	Symptoms causing inability to perform usual social & functional activities OR Operative intervention indicated	Life-threatening consequences (e.g., perforation)
**Rectal Discharge**	Visible discharge	Discharge requiring the use of pads	NA	NA
**Vomiting**	Transient or intermittent AND No or minimal interference with oral intake	Frequent episodes with no or mild dehydration	Persistent vomiting resulting in orthostatic hypotension OR Aggressive rehydration indicated (e.g., IV fluids)	Life-threatening consequences (e.g., hypotensive shock)

Musculoskeletal

PARAMETER	GRADE 1 MILD	GRADE 2 MODERATE	GRADE 3 SEVERE	GRADE 4 POTENTIALLY LIFE-THREATENING
**Arthralgia**	Joint pain causing no or minimal interference with usual social & functional activities	Joint pain causing greater than minimal interference with usual social & functional activities	Joint pain causing inability to perform usual social & functional activities	Disabling joint pain causing inability to perform basic self-care functions
**Arthritis**	Stiffness or joint swelling causing no or minimal interference with usual social & functional activities	Stiffness or joint swelling causing greater than minimal interference with usual social & functional activities	Stiffness or joint swelling causing inability to perform usual social & functional activities	Disabling joint stiffness or swelling causing inability to perform basic self-care functions
**Myalgia** (generalized)	Muscle pain causing no or minimal interference with usual social & functional activities	Muscle pain causing greater than minimal interference with usual social & functional activities	Muscle pain causing inability to perform usual social & functional activities	Disabling muscle pain causing inability to perform basic self-care functions
**Osteonecrosis**	NA	No symptoms but with radiographic findings AND No operative intervention indicated	Bone pain with radiographic findings OR Operative intervention indicated	Disabling bone pain with radiographic findings causing inability to perform basic self-care functions
**Osteopenia**[6] *≥ 30 years of age*	BMD t-score -2.5 to -1	NA	NA	NA
< 30 years of age	BMD z-score -2 to -1	NA	NA	NA
**Osteoporosis**[6] *≥ 30 years of age*	NA	BMD t-score < -2.5	Pathologic fracture (e.g., compression fracture causing loss of vertebral height)	Pathologic fracture causing life-threatening consequences
< 30 years of age	NA	BMD z-score < -2	Pathologic fracture (e.g., compression fracture causing loss of vertebral height)	Pathologic fracture causing life-threatening consequences

[6] BMD t and z scores can be found in: Kanis JA on behalf of the World Health Organization Scientific Group (2007). Assessment of osteoporosis at the primary health-care level. Technical Report. World Health Organization Collaborating Centre for Metabolic Bone Diseases, University of Sheffield, UK. 2007: Printed by the University of Sheffield.

Neurologic

PARAMETER	GRADE 1<br>MILD	GRADE 2<br>MODERATE	GRADE 3<br>SEVERE	GRADE 4<br>POTENTIALLY LIFE-THREATENING
**Acute CNS Ischemia**	NA	NA	Transient ischemic attack	Cerebral vascular accident (e.g., stroke with neurological deficit)
**Altered Mental Status** (for Dementia, see *Cognitive, Behavioral, or Attentional Disturbance* below)	Changes causing no or minimal interference with usual social & functional activities	Mild lethargy or somnolence causing greater than minimal interference with usual social & functional activities	Confusion, memory impairment, lethargy, or somnolence causing inability to perform usual social & functional activities	Delirium OR Obtundation OR Coma
**Ataxia**	Symptoms causing no or minimal interference with usual social & functional activities OR No symptoms with ataxia detected on examination	Symptoms causing greater than minimal interference with usual social & functional activities	Symptoms causing inability to perform usual social & functional activities	Disabling symptoms causing inability to perform basic self-care functions
**Cognitive, Behavioral, or Attentional Disturbance** (includes dementia and attention deficit disorder) *Specify type, if applicable*	Disability causing no or minimal interference with usual social & functional activities OR Specialized resources not indicated	Disability causing greater than minimal interference with usual social & functional activities OR Specialized resources on part- time basis indicated	Disability causing inability to perform usual social & functional activities OR Specialized resources on a full-time basis indicated	Disability causing inability to perform basic self-care functions OR Institutionalization indicated
**Developmental Delay** *< 18 years of age* *Specify type, if applicable*	Mild developmental delay, either motor or cognitive, as determined by comparison with a developmental screening tool appropriate for the setting	Moderate developmental delay, either motor or cognitive, as determined by comparison with a developmental screening tool appropriate for the setting	Severe developmental delay, either motor or cognitive, as determined by comparison with a developmental screening tool appropriate for the setting	Developmental regression, either motor or cognitive, as determined by comparison with a developmental screening tool appropriate for the setting
**Headache**	Symptoms causing no or minimal interference with usual social & functional activities	Symptoms causing greater than minimal interference with usual social & functional activities	Symptoms causing inability to perform usual social & functional activities	Symptoms causing inability to perform basic self-care functions OR Hospitalization indicated OR Headache with significant impairment of alertness or other neurologic function

PARAMETER	GRADE 1 MILD	GRADE 2 MODERATE	GRADE 3 SEVERE	GRADE 4 POTENTIALLY LIFE-THREATENING
**Neuromuscular Weakness** (includes myopathy and neuropathy) *Specify type, if applicable*	Minimal muscle weakness causing no or minimal interference with usual social & functional activities OR No symptoms with decreased strength on examination	Muscle weakness causing greater than minimal interference with usual social & functional activities	Muscle weakness causing inability to perform usual social & functional activities	Disabling muscle weakness causing inability to perform basic self-care functions OR Respiratory muscle weakness impairing ventilation
**Neurosensory Alteration** (includes paresthesia and painful neuropathy) *Specify type, if applicable*	Minimal paresthesia causing no or minimal interference with usual social & functional activities OR No symptoms with sensory alteration on examination	Sensory alteration or paresthesia causing greater than minimal interference with usual social & functional activities	Sensory alteration or paresthesia causing inability to perform usual social & functional activities	Disabling sensory alteration or paresthesia causing inability to perform basic self-care functions
**Seizures** ***New Onset Seizure*** *≥ 18 years of age*	NA	NA	1 to 3 seizures	Prolonged and repetitive seizures (e.g., status epilepticus) OR Difficult to control (e.g., refractory epilepsy)
< 18 years of age (includes new or pre- existing febrile seizures)	Seizure lasting < 5 minutes with < 24 hours postictal state	Seizure lasting 5 to < 20 minutes with < 24 hours postictal state	Seizure lasting ≥ 20 minutes OR > 24 hours postictal state	Prolonged and repetitive seizures (e.g., status epilepticus) OR Difficult to control (e.g., refractory epilepsy)
Pre-existing Seizure	NA	Increased frequency from previous level of control without change in seizure character	Change in seizure character either in duration or quality (e.g., severity or focality)	Prolonged and repetitive seizures (e.g., status epilepticus) OR Difficult to control (e.g., refractory epilepsy)
**Syncope**	Near syncope without loss of consciousness (e.g., pre-syncope)	Loss of consciousness with no intervention indicated	Loss of consciousness AND Hospitalization or intervention required	NA

Pregnancy, Puerperium, and Perinatal

PARAMETER	GRADE 1<br>MILD	GRADE 2<br>MODERATE	GRADE 3<br>SEVERE	GRADE 4<br>POTENTIALLY LIFE-THREATENING
**Fetal Death or Stillbirth** (report using mother's participant ID)<br>*Report only one*	NA	NA	Fetal loss occurring at ≥ 20 weeks gestation	NA
**Preterm Delivery**[7] (report using mother's participant ID)	Delivery at 34 to < 37 weeks gestational age	Delivery at 28 to < 34 weeks gestational age	Delivery at 24 to < 28 weeks gestational age	Delivery at < 24 weeks gestational age
**Spontaneous Abortion or Miscarriage**[8] (report using mother's participant ID)<br>*Report only one*	Chemical pregnancy	Uncomplicated spontaneous abortion or miscarriage	Complicated spontaneous abortion or miscarriage	NA

[7] Definition: A delivery of a live-born neonate occurring at ≥ 20 to < 37 weeks gestational age.
[8] Definition: A clinically recognized pregnancy occurring at < 20 weeks gestational age.

Psychiatric

PARAMETER	GRADE 1<br>MILD	GRADE 2<br>MODERATE	GRADE 3<br>SEVERE	GRADE 4<br>POTENTIALLY LIFE-THREATENING
**Insomnia**	Mild difficulty falling asleep, staying asleep, or waking up early	Moderate difficulty falling asleep, staying asleep, or waking up early	Severe difficulty falling asleep, staying asleep, or waking up early	NA
**Psychiatric Disorders** (includes anxiety, depression, mania, and psychosis)<br>*Specify disorder*	Symptoms with intervention not indicated OR Behavior causing no or minimal interference with usual social & functional activities	Symptoms with intervention indicated OR Behavior causing greater than minimal interference with usual social & functional activities	Symptoms with hospitalization indicated OR Behavior causing inability to perform usual social & functional activities	Threatens harm to self or others OR Acute psychosis OR Behavior causing inability to perform basic self-care functions
**Suicidal Ideation or Attempt**<br>*Report only one*	Preoccupied with thoughts of death AND No wish to kill oneself	Preoccupied with thoughts of death AND Wish to kill oneself with no specific plan or intent	Thoughts of killing oneself with partial or complete plans but no attempt to do so OR Hospitalization indicated	Suicide attempted

Respiratory

PARAMETER	GRADE 1 MILD	GRADE 2 MODERATE	GRADE 3 SEVERE	GRADE 4 POTENTIALLY LIFE-THREATENING
**Acute Bronchospasm**	Forced expiratory volume in 1 second or peak flow reduced to ≥ 70 to < 80% OR Mild symptoms with intervention not indicated	Forced expiratory volume in 1 second or peak flow 50 to < 70% OR Symptoms with intervention indicated OR Symptoms causing greater than minimal interference with usual social & functional activities	Forced expiratory volume in 1 second or peak flow 25 to < 50% OR Symptoms causing inability to perform usual social & functional activities	Forced expiratory volume in 1 second or peak flow < 25% OR Life-threatening respiratory or Hemodynamic compromise OR Intubation
**Dyspnea or Respiratory Distress** *Report only one*	Dyspnea on exertion with no or minimal interference with usual social & functional activities OR Wheezing OR Minimal increase in respiratory rate for age	Dyspnea on exertion causing greater than minimal interference with usual social & functional activities OR Nasal flaring OR Intercostal retractions OR Pulse oximetry 90 to < 95%	Dyspnea at rest causing inability to perform usual social & functional activities OR Pulse oximetry < 90%	Respiratory failure with ventilator support indicated (e.g., CPAP, BPAP, intubation)

Sensory

PARAMETER	GRADE 1 MILD	GRADE 2 MODERATE	GRADE 3 SEVERE	GRADE 4 POTENTIALLY LIFE-THREATENING
**Hearing Loss** *≥ 12 years of age*	NA	Hearing aid or intervention not indicated	Hearing aid or intervention indicated	Profound bilateral hearing loss (> 80 dB at 2 kHz and above) OR Non-serviceable hearing (i.e., >50 dB audiogram and <50% speech discrimination)
< 12 years of age (based on a 1, 2, 3, 4, 6 and 8 kHz audiogram)	> 20 dB hearing loss at ≤ 4 kHz	> 20 dB hearing loss at > 4 kHz	> 20 dB hearing loss at ≥ 3 kHz in one ear with additional speech language related services indicated (where available) OR Hearing loss sufficient to indicate therapeutic intervention, including hearing aids	Audiologic indication for cochlear implant and additional speech-language related services indicated (where available)

**Tinnitus**	Symptoms causing no or minimal interference with usual social & functional activities with intervention not indicated	Symptoms causing greater than minimal interference with usual social & functional activities with intervention indicated	Symptoms causing inability to perform usual social & functional activities	NA
**Uveitis**	No symptoms AND Detectable on examination	Anterior uveitis with symptoms OR Medicamylasal intervention indicated	Posterior or pan- uveitis OR Operative intervention indicated	Disabling visual loss in affected eye(s)
**Vertigo**	Vertigo causing no or minimal interference with usual social & functional activities	Vertigo causing greater than minimal interference with usual social & functional activities	Vertigo causing inability to perform usual social & functional activities	Disabling vertigo causing inability to perform basic self-care functions
**Visual Changes** (assessed from baseline)	Visual changes causing no or minimal interference with usual social & functional activities	Visual changes causing greater than minimal interference with usual social & functional activities	Visual changes causing inability to perform usual social & functional activities	Disabling visual loss in affected eye(s)

Systemic

PARAMETER	GRADE 1<br>MILD	GRADE 2<br>MODERATE	GRADE 3<br>SEVERE	GRADE 4<br>POTENTIALLY LIFE-THREATENING
**Acute Allergic Reaction**	Localized urticaria (wheals) with no medical intervention indicated	Localized urticaria with intervention indicated OR Mild angioedema with no intervention indicated	Generalized urticaria OR Angioedema with intervention indicated OR Symptoms of mild bronchospasm	Acute anaphylaxis OR Life-threatening bronchospasm OR Laryngeal edema
**Chills**	Symptoms causing no or minimal interference with usual social & functional activities	Symptoms causing greater than minimal interference with usual social & functional activities	Symptoms causing inability to perform usual social & functional activities	NA
**Cytokine Release Syndrome**[9]	Mild signs and symptoms AND<br>Therapy (i.e., antibody infusion) interruption not indicated	Therapy (i.e., antibody infusion) interruption indicated AND Responds promptly to symptomatic treatment<br>OR<br>Prophylactic medications indicated for ≤ 24 hours	Prolonged severe signs and symptoms OR Recurrence of symptoms following initial improvement	Life-threatening consequences (e.g., requiring pressor or ventilator support)
**Fatigue or Malaise**<br>*Report only one*	Symptoms causing no or minimal interference with usual social & functional activities	Symptoms causing greater than minimal interference with usual social & functional activities	Symptoms causing inability to perform usual social & functional activities	Incapacitating symptoms of fatigue or malaise causing inability to perform basic self-care functions
**Fever** (non-axillary temperatures only)	38.0 to < 38.6°C or 100.4 to < 101.5°F	≥ 38.6 to < 39.3°C or ≥101.5 to < 102.7°F	≥ 39.3 to < 40.0°C or ≥ 102.7 to < 104.0°F	≥ 40.0°C or ≥ 104.0°F
**Pain**[10] (not associated with study agent injections and not specified elsewhere)<br>*Specify location*	Pain causing no or minimal interference with usual social & functional activities	Pain causing greater than minimal interference with usual social & functional activities	Pain causing inability to perform usual social & functional activities	Disabling pain causing inability to perform basic self-care functions OR Hospitalization indicated
**Serum Sickness**[11]	Mild signs and symptoms	Moderate signs and symptoms AND Intervention indicated (e.g., antihistamines)	Severe signs and symptoms AND Higher level intervention indicated (e.g., steroids or IV fluids)	Life-threatening consequences (e.g., requiring pressor or ventilator support)

9 ~~Definition: A disorder characterized by nausea~~, headache, tachycardia, hypotension, rash, and/or shortness of breath.
10 For pain associated with injections or infusions, see the *Site Reactions to Injections and Infusions* section (page 23).
11 Definition: A disorder characterized by fever, arthralgia, myalgia, skin eruptions, lymphadenopathy, marked discomfort, and/or dyspnea.

PARAMETER	GRADE 1 MILD	GRADE 2 MODERATE	GRADE 3 SEVERE	GRADE 4 POTENTIALLY LIFE-THREATENING
**Underweight**[12] *> 5 to 19 years of age*	NA	WHO BMI z-score < -2 to ≤ -3	WHO BMI z-score < -3	WHO BMI z-score < -3 with life-threatening consequences
2 to 5 years of age	NA	WHO Weight-for-height z-score < -2 to ≤-3	WHO Weight-for- height z-score < -3	WHO Weight-for-height z-score < -3 with life-threatening consequences
< 2 years of age	NA	WHO Weight-for-length z-score < -2 to ≤-3	WHO Weight-for- length z-score < -3	WHO Weight-for-length z-score < -3 with life-threatening consequences
**Weight Loss** (excludes postpartum weight loss)	NA	5 to < 9% loss in body weight from baseline	≥ 9 to < 20% loss in body weight from baseline	≥ 20% loss in body weight from baseline OR Aggressive intervention indicated (e.g., tube feeding, total parenteral nutrition)

[12] WHO reference tables may be accesed by clicking the desired age range or by accessing the following URLs: http://www.who.int/growthref/who2007 bmi for age/en/ for participants > 5 to 19 years of age and http://www.who.int/childgrowth/standards/chart catalogue/en/ for those < 5 years of age.

Urinary

PARAMETER	GRADE 1 MILD	GRADE 2 MODERATE	GRADE 3 SEVERE	GRADE 4 POTENTIALLY LIFE-THREATENING
**Urinary Tract Obstruction**	NA	Signs or symptoms of urinary tract obstruction without hydronephrosis or renal dysfunction	Signs or symptoms of urinary tract obstruction with hydronephrosis or renal dysfunction	Obstruction causing life-threatening consequences

Site Reactions to Injections and Infusions

PARAMETER	GRADE 1 MILD	GRADE 2 MODERATE	GRADE 3 SEVERE	GRADE 4 POTENTIALLY LIFE-THREATENING
**Injection Site Pain or Tenderness** *Report only one*	Pain or tenderness causing no or minimal limitation of use of limb	Pain or tenderness causing greater than minimal limitation of use of limb	Pain or tenderness causing inability to perform usual social & functional activities	Pain or tenderness causing inability to perform basic self-care function OR Hospitalization indicated

**Injection Site Erythema or Redness**[13]<br>*Report only one*<br>*> 15 years of age*	2.5 to < 5 cm in diameter OR 6.25 to < 25 cm^2 surface area AND Symptoms causing no or minimal interference with usual social & functional activities	≥ 5 to < 10 cm in diameter OR ≥ 25 to < 100 cm^2 surface area OR Symptoms causing greater than minimal interference with usual social & functional activities	≥ 10 cm in diameter OR ≥ 100 cm^2 surface area OR Ulceration OR Secondary infection OR Phlebitis OR Sterile abscess OR Drainage OR Symptoms causing inability to perform usual social & functional activities	Potentially lifethreatening consequences (e.g., abscess, exfoliative dermatitis, necrosis involving dermis or deeper tissue)
≤15 years of age	≤ 2.5 cm in diameter	> 2.5 cm in diameter with < 50% surface area of the extremity segment involved (e.g., upper arm or thigh)	≥ 50% surface area of the extremity segment involved (e.g., upper arm or thigh) OR Ulceration OR Secondary infection OR Phlebitis OR Sterile abscess OR Drainage	Potentially life-threatening consequences (e.g., abscess, exfoliative dermatitis, necrosis involving dermis or deeper tissue)
**Injection Site Induration or Swelling**<br>*Report only one*<br>*> 15 years of age*	Same as for **Injection Site Erythema or Redness,** *> 15 years of age*	Same as for **Injection Site Erythema or Redness,** *> 15 years of age*	Same as for **Injection Site Erythema or Redness,** *> 15 years of age*	Same as for **Injection Site Erythema or Redness,** *> 15 years of age*
≤ 15 years of age	Same as for **Injection Site Erythema or Redness,** *≤ 15 years of age*	Same as for **Injection Site Erythema or Redness,** *≤ 15 years of age*	Same as for **Injection Site Erythema or Redness,** *≤ 15 years of age*	Same as for **Injection Site Erythema or Redness,** *≤ 15 years of age*
**Injection Site Pruritus**	Itching localized to the injection site that is relieved spontaneously or in < 48 hours of treatment	Itching beyond the injection site that is not generalized OR Itching localized to the injection site requiring ≥ 48 hours treatment	Generalized itching causing inability to perform usual social & functional activities	NA

[13] Injection Site Erythema or Redness should be evaluated and graded using the greatest single diameter or measured surface area.

Laboratory Values

Chemistries

PARAMETER	GRADE 1<br>MILD	GRADE 2<br>MODERATE	GRADE 3<br>SEVERE	GRADE 4<br>POTENTIALLY LIFE-THREATENING
**Acidosis**	NA	pH ≥ 7.3 to < LLN	pH < 7.3 without life-threatening consequences	pH < 7.3 with life-threatening consequences
**Albumin, Low** (g/dL; *g/L*)	3.0 to < LLN *30 to < LLN*	≥ 2.0 to < 3.0 ≥ *20 to < 30*	< 2.0<br>*< 20*	NA
**Alkaline Phosphatase, High**	1.25 to < 2.5 x ULN	2.5 to < 5.0 x ULN	5.0 to < 10.0 x ULN	≥ 10.0 x ULN
**Alkalosis**	NA	pH > ULN to ≤ 7.5	pH > 7.5 without life-threatening consequences	pH > 7.5 with life-threatening consequences
**ALT or SGPT, High** *Report only one*	1.25 to < 2.5 x ULN	2.5 to < 5.0 x ULN	5.0 to < 10.0 x ULN	≥ 10.0 x ULN
**Amylase (Pancreatic) or Amylase (Total), High** *Report only one*	1.1 to < 1.5 x ULN	1.5 to < 3.0 x ULN	3.0 to < 5.0 x ULN	≥ 5.0 x ULN
**AST or SGOT, High** *Report only one*	1.25 to < 2.5 x ULN	2.5 to < 5.0 x ULN	5.0 to < 10.0 x ULN	≥ 10.0 x ULN
**Bicarbonate, Low** (mEq/L; *mmol/L*)	16.0 to < LLN *16.0 to < LLN*	11.0 to < 16.0 *11.0 to < 16.0*	8.0 to < 11.0 *8.0 to < 11.0*	< 8.0<br>*< 8.0*
**Bilirubin**<br>***Direct Bilirubin[14], High***<br>*> 28 days of age*	NA	NA	> ULN	> ULN with lifethreatening consequences (e.g., signs and symptoms of liver failure)
≤ 28 days of age	ULN to ≤ 1 mg/dL	> 1 to ≤ 1.5 mg/dL	> 1.5 to ≤ 2 mg/dL	> 2 mg/dL
Total Bilirubin, High<br>*> 28 days of age*	1.1 to < 1.6 x ULN	1.6 to < 2.6 x ULN	2.6 to < 5.0 x ULN	≥ 5.0 x ULN
≤ 28 days of age	See Appendix A. Total Bilirubin for Term and Preterm Neonates	See Appendix A. Total Bilirubin for Term and Preterm Neonates	See Appendix A. Total Bilirubin for Term and Preterm Neonates	See Appendix A. Total Bilirubin for Term and Preterm Neonates
**Calcium, High** (mg/dL; *mmol/L*)<br>*≥ 7 days of age*	10.6 to < 11.5<br>*2.65* to < *2.88*	11.5 to < 12.5 *2.88* to < *3.13*	12.5 to < 13.5<br>*3.13* to < *3.38*	≥ 13.5<br>≥ *3.38*
< 7 days of age	11.5 to < 12.4 *2.88* to < *3.10*	12.4 to < 12.9 *3.10* to < *3.23*	12.9 to < 13.5 *3.23* to < *3.38*	≥ 13.5<br>≥ *3.38*

PARAMETER	GRADE 1<br>MILD	GRADE 2<br>MODERATE	GRADE 3<br>SEVERE	GRADE 4<br>POTENTIALLY LIFE-THREATENING
**Calcium (Ionized), High** (mg/dL; *mmol/L*)	> ULN to < 6.0 *> ULN to < 1.5*	6.0 to < 6.4 *1.5 to < 1.6*	6.4 to < 7.2 *1.6 to < 1.8*	≥ 7.2<br>*≥ 1.8*
**Calcium, Low** (mg/dL; *mmol/L*)<br>*≥ 7 days of age*	7.8 to < 8.4 *1.95* to < *2.10*	7.0 to < 7.8<br>*1.75* to < *1.95*	6.1 to < 7.0 *1.53* to < *1.75*	< 6.1<br>*< 1.53*
< 7 days of age	6.5 to < 7.5 *1.63* to < *1.88*	6.0 to < 6.5 *1.50* to < *1.63*	5.50 to < 6.0 *1.38* to < *1.50*	< 5.50<br>*< 1.38*
**Calcium (Ionized), Low** (mg/dL; *mmol/L*)	< LLN to 4.0 *< LLN to 1.0*	3.6 to < 4.0 *0.9 to < 1.0*	3.2 to < 3.6 *0.8 to < 0.9*	< 3.2<br>*< 0.8*
**Cardiac Troponin I, High**	NA	NA	NA	Levels consistent with myocardial infarction or unstable angina as defined by the local laboratory
**Creatine Kinase, High**	3 to < 6 x ULN	6 to < 10 x ULN	10 to < 20 x ULN	≥ 20 x ULN
**Creatinine, High**	1.1 to 1.3 x ULN	> 1.3 to 1.8 x ULN OR Increase of<br>> 0.3 mg/dL above baseline	> 1.8 to < 3.5 x ULN OR Increase<br>of 1.5 to < 2.0 x above baseline	≥ 3.5 x ULN OR<br>Increase of ≥ 2.0 x above baseline
**Creatinine Clearance[15] or eGFR, Low**<br>*Report only one*	NA	< 90 to 60 ml/min or ml/min/1.73 m^2 OR<br>10 to < 30% decrease from baseline	< 60 to 30 ml/min or ml/min/1.73 m^2 OR<br>≥ 30 to < 50% decrease from baseline	< 30 ml/min or ml/min/1.73 m^2 OR<br>≥ 50% decrease from baseline or dialysis needed
**Glucose** (mg/dL; *mmol/L*)<br>***Fasting, High***	110 to 125 *6.11 to < 6.95*	> 125 to 250 *6.95 to < 13.89*	> 250 to 500 *13.89 to < 27.75*	> 500<br>*≥ 27.75*
Nonfasting, High	116 to 160 *6.44 to < 8.89*	> 160 to 250 *8.89 to < 13.89*	> 250 to 500 *13.89 to < 27.75*	> 500<br>*≥ 27.75*
**Glucose, Low** (mg/dL; *mmol/L*)<br>*≥ 1 month of age*	55 to 64 *3.05 to 3.55*	40 to < 55 *2.22 to < 3.05*	30 to < 40 *1.67 to < 2.22*	< 30<br>*< 1.67*
< 1 month of age	50 to 54 *2.78 to 3.00*	40 to < 50 *2.22 to < 2.78*	30 to < 40 *1.67 to < 2.22*	< 30<br>*< 1.67*
**Lactate, High**	ULN to < 2.0 x ULN without acidosis	≥ 2.0 x ULN without acidosis	Increased lactate with pH < 7.3 without life-threatening consequences	Increased lactate with pH < 7.3 with life-threatening consequences

[15] Use the applicable formula (i.e., Cockroft-Gault in mL/min or Schwatrz in mL/min/1.73m^2).

PARAMETER	GRADE 1 MILD	GRADE 2 MODERATE	GRADE 3 SEVERE	GRADE 4 POTENTIALLY LIFE-THREATENING
**Lipase, High**	1.1 to < 1.5 x ULN	1.5 to < 3.0 x ULN	3.0 to < 5.0 x ULN	≥ 5.0 x ULN
**Lipid Disorders** (mg/dL; *mmol/L*)<br>***Cholesterol, Fasting, High***<br>*≥18 years of age*	200 to < 240<br>*5.18 to < 6.19*	240 to < 300<br>*6.19 to < 7.77*	≥ 300<br>*≥ 7.77*	NA
< 18 years of age	170 to < 200<br>*4.40 to < 5.15*	200 to < 300<br>*5.15 to < 7.77*	≥ 300<br>*≥ 7.77*	NA
LDL, Fasting, High<br>*≥18 years of age*	130 to < 160<br>*3.37 to < 4.12*	160 to < 190<br>*4.12 to < 4.90*	≥ 190<br>*≥ 4.90*	NA
> 2 to < 18 years of age	110 to < 130<br>*2.85 to < 3.34*	130 to < 190<br>*3.34 to < 4.90*	≥ 190<br>*≥ 4.90*	NA
Triglycerides, Fasting, High	150 to 300<br>*1.71 to 3.42*	>300 to 500<br>*>3.42 to 5.7*	>500 to < 1,000<br>*>5.7 to 11.4*	> 1,000<br>*> 11.4*
**Magnesium[16], Low**<br>(mEq/L; *mmol/L*)	1.2 to < 1.4<br>*0.60 to < 0.70*	0.9 to < 1.2<br>*0.45 to < 0.60*	0.6 to < 0.9<br>*0.30 to < 0.45*	< 0.6<br>*< 0.30*
**Phosphate, Low** (mg/dL; *mmol/L*)<br>*> 14 years of age*	2.0 to < LLN<br>*0.81 to < LLN*	1.4 to < 2.0<br>*0.65 to < 0.81*	1.0 to < 1.4<br>*0.32 to < 0.65*	< 1.0<br>*< 0.32*
1 to 14 years of age	3.0 to < 3.5<br>*0.97 to < 1.13*	2.5 to < 3.0<br>*0.81 to < 0.97*	1.5 to < 2.5<br>*0.48 to < 0.81*	< 1.5<br>*< 0.48*
< 1 year of age	3.5 to < 4.5<br>*1.13 to < 1.45*	2.5 to < 3.5<br>*0.81 to < 1.13*	1.5 to < 2.5<br>*0.48 to < 0.81*	< 1.5<br>*< 0.48*
**Potassium, High**<br>(mEq/L; *mmol/L*)	5.6 to < 6.0<br>*5.6 to < 6.0*	6.0 to < 6.5<br>*6.0 to < 6.5*	6.5 to < 7.0<br>*6.5 to < 7.0*	≥ 7.0<br>*≥ 7.0*
**Potassium, Low**<br>(mEq/L; *mmol/L*)	3.0 to < 3.4<br>*3.0 to < 3.4*	2.5 to < 3.0<br>*2.5 to < 3.0*	2.0 to < 2.5<br>*2.0 to < 2.5*	< 2.0<br>*< 2.0*
**Sodium, High**<br>(mEq/L; *mmol/L*)	146 to < 150<br>*146 to < 150*	150 to < 154<br>*150 to < 154*	154 to < 160<br>*154 to < 160*	≥ 160<br>*≥ 160*
**Sodium, Low**<br>(mEq/L; *mmol/L*)	130 to < 135<br>*130 to < 135*	125 to < 130<br>*125 to < 135*	121 to < 125<br>*121 to < 125*	≤ 120<br>*≤ 120*
**Uric Acid, High**<br>(mg/dL; *mmol/L*)	7.5 to < 10.0<br>*0.45 to < 0.59*	10.0 to < 12.0<br>*0.59 to < 0.71*	12.0 to < 15.0<br>*0.71 to < 0.89*	≥ 15.0<br>*≥ 0.89*

[16] To convert a magnesium value from mg/dL to mmol/L, laboratories should multiply by 0.4114.

Hematology

PARAMETER	GRADE 1<br>MILD	GRADE 2<br>MODERATE	GRADE 3<br>SEVERE	GRADE 4<br>POTENTIALLY LIFE-THREATENING
**Absolute CD4+ Count, Low**<br>(cell/mm^3; *cells/L*)<br>*> 5 years of age*<br>*(not HIV infected)*	300 to < 400<br>*300 to < 400*	200 to < 300<br>*200 to < 300*	100 to < 200<br>*100 to < 200*	< 100<br>*< 100*
**Absolute Lymphocyte Count, Low**<br>(cell/mm^3; *cells/L*)<br>*> 5 years of age*<br>*(not HIV infected)*	600 to < 650<br>*0.600 x 10^9 to < 0.650 x 10^9*	500 to < 600<br>*0.500 x 10^9 to < 0.600 x 10^9*	350 to < 500<br>*0.350 x 10^9 to < 0.500 x 10^9*	< 350<br>*< 0.350 x 10^9*
**Absolute Neutrophil Count (ANC), Low**<br>(cells/mm^3; *cells/L*)<br>*> 7 days of age*	800 to 1,000<br>*0.800 x 10^9 to 1.000 x 10^9*	600 to 799<br>*0.600 x 10^9 to 0.799 x 10^9*	400 to 599<br>*0.400 x 10^9 to 0.599 x 10^9*	< 400<br>*< 0.400 x 10^9*
2 to 7 days of age	1,250 to 1,500<br>*1.250 x 10^9 to 1.500 x 10^9*	1,000 to 1,249<br>*1.000 x 10^9 to 1.249 x 10^9*	750 to 999<br>*0.750 x 10^9 to 0.999 x 10^9*	< 750<br>*< 0.750 x 10^9*
≤ 1 day of age	4,000 to 5,000<br>*4.000 x 10^9 to 5.000 x 10^9*	3,000 to 3,999<br>*3.000 x 10^9 to 3.999 x 10^9*	1,500 to 2,999<br>*1.500 x 10^9 to 2.999 x 10^9*	< 1,500<br>*< 1.500 x 10^9*
**Fibrinogen, Decreased**<br>(mg/dL; *g/L*)	100 to < 200<br>*1.00 to < 2.00*<br>OR<br>0.75 to < 1.00 x LLN	75 to < 100<br>*0.75 to < 1.00*<br>OR<br>≥ 0.50 to < 0.75 x LLN	50 to < 75<br>*0.50 to < 0.75*<br>OR<br>0.25 to < 0.50 x LLN	< 50<br>*< 0.50*<br>OR<br>< 0.25 x LLN<br>OR Associated with gross bleeding
**Hemoglobin[17], Low** (g/dL; *mmol/L*)[18]<br>*≥ 13 years of age*<br>*(male only)*	10.0 to 10.9<br>*6.19 to 6.76*	9.0 to < 10.0<br>*5.57 to < 6.19*	7.0 to < 9.0<br>*4.34 to < 5.57*	< 7.0<br>*< 4.34*
≥ 13 years of age<br>*(female only)*	9.5 to 10.4<br>*5.88 to 6.48*	8.5 to < 9.5<br>*5.25 to < 5.88*	6.5 to < 8.5<br>*4.03 to < 5.25*	< 6.5<br>*< 4.03*

[17] Male and female sex are defined as sex at birth.
[18] The conversion factor used to convert g/dL to mmol/L is 0.6206 and is the most commonly used conversion factor. For grading hemoglobin results obtained by an analytic method with a conversion factor other than 0.6206, the result must be converted to g/dL using the appropriate conversion factor for the particular laboratory.

PARAMETER	GRADE 1<br>MILD	GRADE 2<br>MODERATE	GRADE 3<br>SEVERE	GRADE 4<br>POTENTIALLY LIFE-THREATENING

57 days of age to < 13 years of age (male and female)	9.5 to 10.4<br>*5.88 to 6.48*	8.5 to < 9.5<br>*5.25 to < 5.88*	6.5 to < 8.5<br>*4.03 to < 5.25*	< 6.5<br>*< 4.03*
36 to 56 days of age (male and female)	8.5 to 9.6<br>*5.26 to 5.99*	7.0 to < 8.5<br>*4.32 to < 5.26*	6.0 to < 7.0<br>*3.72 to < 4.32*	< 6.0<br>*< 3.72*
22 to 35 days of age (male and female)	9.5 to 11.0<br>*5.88 to 6.86*	8.0 to < 9.5<br>*4.94 to < 5.88*	6.7 to < 8.0<br>*4.15 to < 4.94*	< 6.7<br>*< 4.15*
8 to < 21 days of age (male and female)	11.0 to 13.0<br>*6.81 to 8.10*	9.0 to < 11.0<br>*5.57 to < 6.81*	8.0 to < 9.0<br>*4.96 to < 5.57*	< 8.0<br>*< 4.96*
< 7 days of age (male and female)	13.0 to 14.0<br>*8.05 to 8.72*	10.0 to < 13.0<br>*6.19 to < 8.05*	9.0 to < 10.0<br>*5.59 to < 6.19*	< 9.0<br>*< 5.59*
**INR, High** (not on anticoagulation therapy)	1.1 to < 1.5 x ULN	1.5 to < 2.0 x ULN	2.0 to < 3.0 x ULN	≥ 3.0 x ULN
**Methemoglobin** (% hemoglobin)	5.0 to < 10.0%	10.0 to < 15.0%	15.0 to < 20.0%	≥ 20.0%
**PTT, High** (not on anticoagulation therapy)	1.1 to < 1.66 x ULN	1.66 to < 2.33 x ULN	2.33 to < 3.00 x ULN	≥ 3.00 x ULN
**Platelets, Decreased** (cells/mm^3; *cells/L*)	100,000 to < 124,999<br>*100.000×10^9 to < 124.999×10^9*	50,000 to < 100,000<br>*50.000×10^9 to < 100.000×10^9*	25,000 to < 50,000<br>*25.000×10^9 to < 50.000×10^9*	< 25,000<br>*< 25.000×10^9*
**PT, High** (not on anticoagulation therapy	1.1 to < 1.25 x ULN	1.25 to < 1.50 x ULN	1.50 to < 3.00 x ULN	≥ 3.00 x ULN
**WBC, Decreased** (cells/mm^3; *cells/L*)<br>*> 7 days of age*	2,000 to 2,499<br>*2.000×10^9 to 2.499×10^9*	1,500 to 1,999<br>*1.500×10^9 to 1.999×10^9*	1,000 to 1,499<br>*1.000×10^9 to 1.499×10^9*	< 1,000<br>*< 1.000×10^9*
< 7 days of age	5,500 to 6,999<br>*5.500×10^9 to 6.999×10^9*	4,000 to 5,499<br>*4.000×10^9 to 5.499×10^9*	2,500 to 3,999<br>*2.500×10^9 to 3.999×10^9*	< 2,500<br>*< 2.500×10^9*

Urinalysis

PARAMETER	GRADE 1	GRADE 2	GRADE 3	GRADE 4
**Glycosuria (**random collection tested by dipstick)	Trace to 1+ or ≤ 250 mg	2+ or > 250 to < 500 mg	> 2+ or > 500 mg	NA
**Hematuria** (not to be reported based on dipstick findings or on blood believed to be of menstrual origin)	6 to < 10 RBCs per high power field	≥ 10 RBCs per high power field	Gross, with or without clots OR<br>With RBC casts OR<br>Intervention indicated	Life-threatening consequences
**Proteinuria** (random collection tested by dipstick)	1+	2+	3+ or higher	NA

Page **83** of **84**

References

1 (UNAIDS)., J. U. N. P. o. H. A. The Gap Report., (UNAIDS, 2014).
2 UNAIDS. AIDS by the numbers 2015. (2015).
3 CDC. CDC. Vital Signs: HIV Diagnosis, Care, and Treatment Among Persons Living with HIV- United States 2011. *MMWR* **4**, 1-6 (2014).
4 Jardine, J. *et al.* Rational HIV immunogen design to target specific germline B cell receptors. *Science* **340**, 711-716, doi:10.1126/science.1234150 (2013).
5 Sok, D. *et al.* Promiscuous glycan site recognition by antibodies to the high-mannose patch of gp120 broadens neutralization of HIV. *Sci Transl Med* **6**, 236ra263, doi:10.1126/scitranslmed.3008104 (2014).
6 Caskey, M. *et al.* Viraemia suppressed in HIV-1-infected humans by broadly neutralizing antibody 3BNC117. *Nature* **522**, 487-491, doi:10.1038/nature14411 (2015).
7 Barouch, D. H. *et al.* Therapeutic efficacy of potent neutralizing HIV-1-specific monoclonal antibodies in SHIV-infected rhesus monkeys. *Nature* **503**, 224-228, doi:10.1038/nature12744 (2013).
8 Hessell, A. J. *et al.* Broadly neutralizing human anti-HIV antibody 2G12 is effective in protection against mucosal SHIV challenge even at low serum neutralizing titers. *PLoS Pathog* **5**, e1000433, doi:10.1371/journal.ppat.1000433 (2009).
9 Hessell, A. J. *et al.* Effective, low-titer antibody protection against low-dose repeated mucosal SHIV challenge in macaques. *Nat Med* **15**, 951-954, doi:10.1038/nm.1974 (2009).
10 Moldt, B. *et al.* Highly potent HIV-specific antibody neutralization in vitro translates into effective protection against mucosal SHIV challenge in vivo. *Proceedings of the National Academy of Sciences of the United States of America* **109**, 18921-18925, doi:10.1073/pnas.1214785109 (2012).
11 Walker, L. M. *et al.* Broad neutralization coverage of HIV by multiple highly potent antibodies. *Nature* **477**, 466-470, doi:10.1038/nature10373 (2011).
12 Haynes, B. F. & McElrath, M. J. Progress in HIV-1 vaccine development. *Curr Opin HIV AIDS* **8**, 326-332, doi:10.1097/COH.0b013e328361d178 (2013).
13 Burton, D. R. & Mascola, J. R. Antibody responses to envelope glycoproteins in HIV-1 infection. *Nat Immunol* **16**, 571-576, doi:10.1038/ni.3158 (2015).
14 Sok, D. *et al.* Recombinant HIV envelope trimer selects for quaternary-dependent antibodies targeting the trimer apex. *Proc Natl Acad Sci U S A* **111**, 17624-17629, doi:10.1073/pnas.1415789111 (2014).
15 Scheid, J. F. *et al.* Sequence and structural convergence of broad and potent HIV antibodies that mimic CD4 binding. *Science* **333**, 1633-1637, doi:10.1126/science.1207227 (2011).
16 Shingai, M. *et al.* Passive transfer of modest titers of potent and broadly neutralizing anti-HIV monoclonal antibodies block SHIV infection in macaques. *J Exp Med* **211**, 2061-2074, doi:10.1084/jem.20132494 (2014).
17 Lynch, R. M. *et al.* Virologic effects of broadly neutralizing antibody VRC01 administration during chronic HIV-1 infection. *Sci Transl Med* **7**, 319ra206, doi:10.1126/scitranslmed.aad5752 (2015).
18 Andrade, A. *et al.* Three distinct phases of HIV-1 RNA decay in treatment-naive patients receiving raltegravir-based antiretroviral therapy: ACTG A5248. *J Infect Dis* **208**, 884-891, doi:10.1093/infdis/jit272 (2013).

Page **84** of **84**

International AIDS Vaccine Initiative

DOCUMENT NUMBER:	
DOCUMENT TITLE:	
DOCUMENT NOTES:	

Document Information

Revision:	Vault:
Status:	Document Type:

Date Information

Effective Date:	Expiration Date:
Release Date:	Next Review Date:

Control Information

Author:	Owner:
Previous Number:	Change Number:

Signature Manifest

Document Number: TMF-02-0166 **Revision:** 3

Title: Protocol PGT121

All dates and times are in Eastern Time Zone.

T001 Protocol

Change Request Approval

Name/Signature	Title	Date	Meaning/Reason
Dani Vooijs (DVOOIJS)			
Katherine Crisafi (KCRISAFI)			
Michele Fong Lim (MFONGLIM)	Director Quality Systems	22 Nov 2016, 02:24:05 PM	Approved

CMO Approval

Name/Signature	Title	Date	Meaning/Reason
Frances Priddy (FPRIDDY)	Chief Medical Officer	23 Nov 2016, 02:41:20 PM	Approved

QA Final Release

Name/Signature	Title	Date	Meaning/Reason
Dani Vooijs (DVOOIJS)			
Katherine Crisafi (KCRISAFI)			
Michele Fong Lim (MFONGLIM)	Director Quality Systems	23 Nov 2016, 02:49:07 PM	Approved

Notify

Name/Signature	Title	Date	Meaning/Reason
Lisa Sunner (LSUNNER)		23 Nov 2016, 02:49:07 PM	Email Sent

**Protocol Title:**	A Phase 1 Randomized Placebo-controlled Clinical Trial of the Safety, Pharmacokinetics and Antiviral Activity of PGT121 Monoclonal Antibody (mAb) in HIV-uninfected and HIV-infected Adults
**Protocol Number:**	IAVI T001
**Regulatory Investigational Product Number**	IND 126807
**ClinicalTrials.gov Registry Number**	NCT02960581
**Phase:**	Phase 1
**Sponsor:**	International AIDS Vaccine Initiative (IAVI)<br>125 Broad Street, 9th Floor<br>New York, New York 10004<br>USA
**Sponsor Status**	Not for-Profit Organization
**Date of Protocol Version:**	04 April 2017<br>05.0<br>Protocol Amendment<br><br>23 November 2016<br>04.0<br>Edits from FDA<br><br>17 October 2016<br>03.0<br>Edits from IRB comments<br><br>09 September 2016<br>02.0<br>Revision to IRB Submission<br><br>05 August 2016<br>01.0<br>IRB Submission

THE CONFIDENTIAL INFORMATION IN THIS DOCUMENT IS PROVIDED TO YOU AS AN INVESTIGATOR, POTENTIAL INVESTIGATOR, OR CONSULTANT, FOR REVIEW BY YOU, YOUR STAFF, AND APPLICABLE INSTITUTIONAL REVIEW BOARDS (IRBS) AND/OR INDEPENDENT ETHICS COMMITTEES (IECS). IT IS UNDERSTOOD THAT THE INFORMATION WILL NOT BE DISCLOSED TO OTHERS, EXCEPT TO THE EXTENT NECESSARY TO OBTAIN ETHICAL AND REGULATORY APPROVAL FROM THE RESPECTIVE COMMITTEE'S AGENCIES AND INFORMED CONSENT FROM THOSE PERSONS TO WHOM THE INVESTIGATIONAL PRODUCT MAY BE ADMINISTERED.

SYNOPSIS

**TITLE**	A Phase 1 Randomized Placebo-controlled Clinical Trial of the Safety, Pharmacokinetics and Antiviral Activity of PGT121 Monoclonal Antibody (mAb) in HIV-uninfected and HIV-infected Adults
**PROTOCOL NUMBER**	IAVI T001
**PHASE**	Phase 1
**SPONSOR**	International AIDS Vaccine Initiative (IAVI)<br>125 Broad Street, 9th Floor<br>New York, New York 10004,<br>USA
**SPONSOR STATUS**	Not for Profit Organization
**STUDY PRODUCTS**	PGT121 monoclonal antibody (mAb)
**OBJECTIVES**	**Primary Objectives**<br>• To evaluate the safety and tolerability of a single intravenous (IV) infusion of PGT121 mAb at 3, 10, and 30 mg/kg in HIV-uninfected adults and HIV-infected adults<br>• To evaluate the pharmacokinetic (PK) profile of IV infusion of PGT121 mAb in HIV-uninfected and HIV-infected adults<br>• To evaluate the antiviral activity of IV infusion of PGT121 mAb in HIV-infected adults not on ART<br><br>**Secondary Objective**<br>• To determine if PGT121 induces anti-PGT121 antibodies<br>• To determine the effect of PGT121 mAb on CD4+ T cell counts in HIV-infected adults<br>• To determine if PGT121 mAb induces viral escape mutants in viremic HIV-infected adults not on ART<br><br>**Exploratory Objectives**<br>• To determine if PGT121 mAb has any impact on the host immune responses (i.e., HIV-specific cellular and humoral immune response)<br>• To determine the effect of PGT121 mAb on the size of the latent HIV reservoir in HIV-infected adults<br>• To determine PGT121 mAb levels in mucosal secretions in HIV-uninfected and HIV-infected adults<br>• To measure in vitro neutralization of HIV isolates with participant's serum post PGT121 mAb IV infusion<br>• To determine if PGT121 mAb has any impact on resistance mutations to ARVs

**ENDPOINTS**	***Primary***: <br>*Safety and Tolerability:* We will measure the following endpoints to evaluate the safety and tolerability of PGT121 mAb in HIV-uninfected and HIV-infected adults: <br>1. Proportion of participants with moderate or greater reactogenicity (e.g., solicited adverse events) for 3 days following IV infusion of PGT121 mAb. 2. Proportion of participants with moderate or greater and/or PGT121 mAb-related unsolicited adverse events (AEs), including safety laboratory (biochemical, hematological) parameters, following IV infusion of PGT121 mAb for the first 56 days post administration of Investigational Product. 3. Proportion of participants with PGT121 mAb-related serious adverse events (SAEs) throughout the study period. <br>*Pharmacokinetics:* We will measure the following endpoints to determine the pharmacokinetics following IV infusion of PGT121 mAb in HIV-uninfected and HIV-infected adults: <br>1. Elimination half-life (t1/2) 2. Clearance (CL/F) 3. Volume of distribution (Vz/F) 4. Area under the concentration decay curve (AUC) 5. Impact of viral load and/or ART on PGT121 disposition (elimination half-life (t1/2), clearance (CL/F), volume of distribution (Vz/F), total exposure <br>*Antiviral activity:* We will calculate the following endpoints to determine the antiviral activity following IV infusion of PGT121 mAb in viremic HIV-infected adults not on ART: <br>1. Change in plasma HIV-1 RNA levels from baseline (mean of pre-entry and entry values) <br>***Secondary***: <br>*Anti-PGT121 antibodies:* We will calculate the following endpoint to determine if anti-PGT121 antibodies are elicited following IV infusion of PGT121 mAb in HIV-uninfected and HIV-infected adults: <br>1. Change in serum anti-PGT121 antibody titers from baseline <br>*CD4+ T cell count:* We will calculate the following endpoint to determine if IV infusion of PGT121 mAb has any impact on CD4+ T cell counts in HIV-infected

adults:

1. Change in CD4+ T cell count and frequency compared to baseline as measured by single platform flow cytometry

HIV genotyping/phenotyping of circulating virus for evaluation of PGT121 mAb -induced escape mutations:
We will compare plasma virus sequences before and after IV infusion of PGT121 mAb to determine if PGT121 mAb induces viral escape mutations in viremic HIV-infected adults not on ART.

1. Development of sequence variations in epitopes known to result in reduced PGT121 mAb neutralization susceptibility.

Exploratory:

Additional assessments may include, but are not limited to, the following: HIV-specific IgG/IgA binding responses by ELISA, HIV-specific cellular immune responses by ELISPOT, HIV-specific antibody function by ADCC, ADCP, and ADCVI assays, resistance mutations to current ARVs, PGT121 mAb levels in mucosal secretions, changes in total HIV-1 DNA and 2-long terminal repeat (LTR) circular HIV-1 DNA in resting or total CD4 T cells and in vitro neutralization of HIV isolates with participant's serum post IV infusion of PGT121 mAb.

STUDY DESIGN TABLE

	Group	Participants	Sub-Group	Regimen	N	Dose (mg/kg)
Part 1[1]	1[3]	HIV-uninfected participants	1A	PGT121/Placebo	4/1 (6/2 if DLT[2])	3
			1B	PGT121/Placebo	4/1 (6/2 if DLT)	10
			1C	PGT121/Placebo	4/1 (6/2 if DLT)	30
	2[3]	HIV-infected on ART, (<50 cp/ml)	2A	PGT121/Placebo	4/1 (6/2 if DLT[2])	3
			2B	PGT121/Placebo	4/1 (6/2 if DLT)	10
			2C	PGT121/Placebo	4/1 (6/2 if DLT)	30
Safety Monitoring Committee Review[4]						
Part 2	3[5]	HIV-Infected off ART (VL $2x10^3$ – $1x10^5$cp/ml)	3A[6]	PGT121	6 (max 9)	30
			3B	PGT121	8 (max 12)	10
			3C	PGT121	10 (max 15)	3
		HIV-Infected off ART (VL $1x10^2$ – $2x10^3$ cp/ml)	3D[7]	PGT121	3	30
			3E	PGT121	3	10
			3F	PGT121	3	3

DLT, dose limiting toxicity; ART, antiretroviral therapy; cp, copies; ml, milliliter
Administration of PGT 121 will be by intravenous infusion (IV)

1. Eligible participants for Groups 1 and 2 will be enrolled according to their HIV-serostatus and will occur in parallel. At each dose level in Part 1, investigational product (IP) administration will be separated by at least 4 days for each of the first 3 participants. Randomization will ensure at least 2 participants receive active product and are observed for at least 4 days before administration to additional participants.
2. A dose limiting toxicity (DLT) will be defined as 1) any Grade 3 or greater reactogenicity or adverse event judged by the study investigators as at least possibly related to investigational product or 2) any SAE considered at least possibly related to investigational product.
3. The PSRT will review safety data to determine dose escalation. If no DLT occurs within 2 weeks from infusion of the first 5 participants in a dose subgroup (e.g. 1A), dose escalation to the next dose subgroup will proceed (e.g. 1B). If 1 DLT occurs in a dose subgroup (e.g. 1A), 3 additional participants will be enrolled into that dose subgroup; these 3 participants will be randomized between PGT121 mAb and placebo at a 2:1 ratio. If no additional DLTs occur in that subgroup (e.g. 1A) within 2 weeks of infusion in the 8 total participants (3:1 ratio of PGT121 mAb to placebo), the study can proceed with enrolment of the next dose subgroup (e.g. 1B). If 2 or more DLTs accumulate in a dose subgroup (e.g. 1A) that are the same, similar, or in the same System Organ Class, dosing will be halted in that subgroup (e.g. 1A) and the next lower dose level will be declared the maximum tolerated dose (MTD) for that subgroup (e.g. 1A). When groups are enrolled in parallel, if the MTD is determined in one group (e.g. Group 1) due to the occurrence of 2 or more DLTs in this group, dosing of participants in the parallel group (e.g. Group 2) will be held until the PSRT has reviewed the safety data and determined whether the MTD should be applied

	to both groups. If no DLT occurs in the final dose subgroups, MTD will be the highest dose given (subgroups 1C and/or 2C 30mg/kg) after 14 days of follow-up. Dose escalation in Groups 1 and 2 may occur in parallel, and can occur independent of each other.<br>4. Following IP administration of the last participant in both Groups 1 and 2, an independent Safety Monitoring Committee (SMC) will review at least the first 14 days of safety data to confirm MTD in each group, and determine whether, and at what dose, Group 3 can initiate enrollment.<br>5. Group 3 will start with the MTD as determined in Part 1. Group 3 will start with subgroups 3A and 3D if the MTD is 30mg/kg, subgroups 3B and 3E if the MTD is 10mg/kg and subgroups 3C and 3F if the MTD is 3mg/kg.<br>6. If subgroup 3A achieves a decline in HIV RNA significantly greater than 0.9 logs compared to baseline, enrolment into subgroup 3A will be stopped and the next lower dose subgroup 3B will be initiated. The same rule will apply for progression from subgroup 3B to 3C. At least 6, 8 and 10 but up to 9, 12 and 15 participants, will be enrolled in subgroups 3A, 3B, and 3C respectively, until the minimum effective dose is determined. If a decline significantly greater than 0.9 logs in HIV RNA is not achieved, enrollment will be stopped at the completion of enrolment at that dose level.<br>7. As soon as subgroup 3D has enrolled 3 participants, enrolment into subgroup 3E will be initiated. The same rule will apply for progression from subgroup 3E to 3F. In the event that dose de-escalation in subgroups 3A-C is stopped due to failure to meet virologic endpoints, dose de-escalation in subgroups 3D-F will continue.
**METHODS**	See Schedule of Procedures, Appendices<br>A, B and C
**STUDY POPULATION**	The study population will include three different groups:<br>Group 1 will include HIV-uninfected males or females aged 18-50 years old who are willing to maintain low risk behavior for HIV infection; principal exclusion criteria include confirmed HIV-infection, pregnancy or lactation, significant acute or chronic disease and clinically significant laboratory abnormalities. Group 2 will include HIV-infected males or females aged 18-65 years old on a stable antiretroviral regimen with HIV-1 RNA plasma level <50 copies/ml, CD4 cell count > 300 cells/uL; principal exclusion criteria include history of AIDS-defining illness, significant acute or chronic medical condition other than HIV infection, and clinically significant laboratory abnormalities. Group 3 will include HIV-infected males or females aged 18-65 years old, not on antiretroviral therapy for > 6 month with detectable HIV-1 RNA plasma level between 100 and 100,000 copies/ml, CD4 cell count > 300 cells/uL; principal exclusion criteria include history of AIDS-defining illness, significant acute or chronic medical condition other than HIV infection, and clinically significant laboratory abnormalities.
**NUMBER OF PARTICIPANTS**	63-93 participants will be included.
**DOSE ESCALATION and PAUSE RULES**	The first part of this study is a dose-escalation trial in HIV-uninfected adults and HIV-infected adults on ART with suppressed viral load, as indicated in the study design table.<br><br>If 2 or more DLTs accumulate in a dose subgroup that are the same, similar, or in the same System Organ Class, dosing will be halted and the next lower dose level will be declared the maximum tolerated dose (MTD) within this group. If no DLT occurs in the final dose group, MTD will be the highest dose given (subgroups 1C and 2C 30mg/kg) after 14 days of follow-up.

	The Protocol Safety Review Team (PSRT) will review safety data through at least day 14 post-IP administration for the first 5 participants in each dose subgroup (e.g. 1A) prior to allowing enrolment of participants into the next dose subgroup (e.g. 1B). Dose escalation in Groups 1 and 2 may occur in parallel, and can occur independent of each other.<br><br>Following IP administration of the last participant in both Groups 1 and 2, an independent Safety Monitoring Committee (SMC) will review safety data through at least day 14 post-IP administration for all participants to confirm MTD, and determine whether, and at what dose, Group 3 can initiate enrollment.<br><br>The second part of this study is a dose-de-escalation trial in HIV-infected adults not on ART, as indicated in the study design table.<br><br>The study will be paused for a safety review by the investigators and the independent SMC if 1) 1 or more participants experiences a Serious Adverse Event that is judged possibly, probably or definitely related to the IP, 2) There is a participant death, regardless of relationship to the IP, 3) if 2 or more participants experience grade 3 adverse events in the same System Organ Class that are considered to be at least possibly related to IP or 4) any grade 4 adverse event that is considered to be possibly, probably, or definitely related to IP. See protocol section 17.3.
**FORMULATIONS, VOLUMES AND ROUTES OF ADMINISTRATION**	**PGT121 mAb**: PGT121 mAb is a recombinant, fully human monoclonal antibody (mAb) of the IgG1 isotype that binds to the HIV envelope. The concentration and volume of product in each vial is 50 mg/mL, 6mL in each vial. PGT121 mAb will be given intravenously in this study.
**DURATION OF STUDY PARTICIPATION**	Participants will be screened up to 56 days (Groups 1 and 2) or 42 days (Group 3) before IP administration and will be followed for 24 weeks. The anticipated study duration for each participant is approximately 6 months from screening through last study visit. It is anticipated that it will take approximately 4.5 months to enroll Groups 1 and 2. It is anticipated that it will take approximately 8 months to enroll the entire study. The exact duration depends on the recruitment rate and how many participants will be required per dose group.
**RANDOMIZATION and BLINDING**	This is a randomized, placebo-controlled study for groups 1 and 2, and open label for group 3 who will not receive placebo.
**EVALUATION FOR INTERCURRENT HIV INFECTION:**	Participants in Group 1 (HIV-uninfected) will be tested for HIV according to the Schedule of Procedures. Test results will be interpreted according to a pre-determined diagnostic algorithm. HIV testing at additional time points may be performed upon the request of the participant and Principal Investigator or designee as medical or social circumstances warrant.
**SAFETY MONITORING AND STATISTICAL CONSIDE-RATIONS:**	All clinical trial data collected, identified only by a study identification number, will be entered into the clinical trial database.<br><br>Safety will continually be monitored by the Investigators, the Sponsor's Medical Monitor and a Protocol Safety Review Team (PSRT); detailed pause criteria are pre-defined.<br><br>Safety data will be reviewed by an independent Safety Monitoring

	Committee (SMC). *Ad hoc* safety review may be specifically requested by the Sponsor, the Principal Investigators, Ethics Committees, Regulatory Authorities, or by the SMC. All clinical and routine laboratory data will be included in the safety analysis. At the end of the study, a full analysis will be prepared.

TABLE OF CONTENTS

ABBREVIATIONS **12**

CONTACT INFORMATION **13**

1.0 SIGNATURE PAGE **14**

2.0 INTRODUCTION AND BACKGROUND INFORMATION **15**

2.1 Study Rationale 15
2.2 Experience with PGT121 16

3.0 STUDY OBJECTIVES **16**

3.1 Primary Objectives 16
3.2 Secondary Objectives 16
3.3 Exploratory Objectives: 16

4.0 STUDY ENDPOINTS **16**

4.1 Study Endpoints 16
4.1.1 Primary Endpoints 16
4.1.2 Secondary Endpoints 17
4.1.3 Exploratory Endpoints 18

5.0 STUDY DESIGN **18**

5.1 Definition of Dose Limiting Toxicity and Maximum Tolerated Dose 18
5.2 Dose Escalation – Groups 1 and 2: Determination of Maximum Tolerated Dose 18
5.3 Dose De-Escalation- Group 3: Determination of Minimum Effective Dose 19
5.4 Duration of the Study 21
5.5 Study Population 21
5.6 Inclusion Criteria 22
5.7 Exclusion Criteria 24
5.8 Recruitment of Participants 27

6.0 STUDY VISITS **27**

6.1 Screening Period 27
6.2 IV infusion of PGT121 mAb Visit 28
6.3 Post-IV infusion of PGT121 mAb Visits 29
6.4 Additional Follow-up Visits 29
6.5 Unscheduled Visits 29
6.6 Final Study Visit or Early Termination Visit 30

7.0 STUDY PROCEDURES **30**

7.1 Informed Consent Process 30
7.2 Medical History and Physical Examination 30
7.3 HIV Testing and HIV-test Counselling (Group 1) 31
7.4 HIV Risk Reduction Counselling 31
7.5 Family Planning Counselling 31
7.6 ART Counselling (Group 3) 31
7.7 Specimens 32
7.8 Reimbursement 32
7.9 Randomization and Blinding 32
7.10 Un-blinding Procedure for Individual Participants 33
7.11 Assessment of IP related HIV sero-positivity 33

8.0 INVESTIGATIONAL PRODUCT 33
8.1 Description 33
8.2 Shipment and Storage 34
8.3 Preparation of Investigational Product (IP) 34
8.4 Administration of Investigational Product 34
8.5 Accountability and Disposal of Investigational Product 34
9.0 ASSESSMENTS 35
9.1 Safety Assessments 35
9.1.1 Local reactogenicity 35
9.1.2 Systemic reactogenicity 35
9.1.3 Vital Signs 35
9.1.4 Other Adverse Events 35
9.1.5 Concomitant Medications 36
9.1.6 Routine laboratory parameters 36
9.1.7 Specific screening tests: 36
9.2 Virologic Assessments 37
9.3 Exploratory Immunogenicity Assessments 37
9.3.1 Antibody Responses 37
9.3.2 Cellular Responses 38
9.3.3 PBMC, Serum and Plasma Storage 38
9.4 Other Assessments 38
9.4.1 HIV Antibody Testing (Group 1) 38
9.4.2 Pharmacokinetics 38
9.4.3 HLA Typing 39
9.4.5 Pregnancy Test 39
9.4.6 HIV Risk Assessment (Group 1) 39
9.4.7 Social Impact Assessment 39
10.0 ADVERSE EVENTS 39
10.1 Definition 39
10.2 Assessment of Severity of Adverse Events 39
10.3 Relationship to Investigational Product 40
10.4 Serious Adverse Events 41
10.5 Reporting Potential Immune-Mediated Diseases 42
10.6 Clinical Management of Adverse Events 43
10.7 Pregnancy 43
10.8 Intercurrent HIV Infection (Group 1) 43
11.0 MANAGEMENT OF HIV ISSUES DURING AND FOLLOWING STUDY 44
11.1 HIV Testing – Group 1 44
11.2 Social Discrimination as a Result of IP-related antibodies 44
11.3 HIV infection – Group 1 44
11.3.1 Counselling 44
11.3.2 Referral for Support/Care 45
12.0 WITHDRAWAL FROM STUDY 45
12.1 Deferral of IV infusion of IP 45
12.2 Withdrawal from the Study (Early Termination) 45
13.0 DATA HANDLING 46
13.1 Data Collection and Record Keeping at the Study Site 46

13.3 Data Entry at the Study Site 46
13.4 Data Analysis 46

14.0 STATISTICAL CONSIDERATIONS 47

14.1 Safety and Tolerability Analysis....47
14.2 Pharmacokinetic Analysis 48
14.3 Virologic Analysis for Dose De-escalation in Groups 3A-C 48
14.4 Analysis of Antiviral Activity and Dose De-escalation in Subgroups 3D-F....51
14.5 Secondary and Exploratory Immunologic and Virologic Analyses 51

15.0 QUALITY CONTROL AND QUALITY ASSURANCE....52

16.0 DATA AND BIOLOGICAL MATERIAL 52

17.0 ADMINISTRATIVE STRUCTURE 52

17.1 Protocol Safety Review Team 52
17.2 Safety Monitoring Committee (SMC) 53
17.2.1 Content of Interim Safety Review 53
17.2.2 SMC Review of Group 1 and 2 data prior to starting Group 3....53
17.3 Criteria for Pausing the Study....53
17.4 Study Supervision 55
17.5 Study Monitoring 55
17.6 Investigator's Records....55

18.0 INDEMNITY 55

19.0 PUBLICATION....56

20.0 ETHICAL CONSIDERATIONS....56

APPENDIX A: SCHEDULE OF PROCEDURES – GROUP 1 (A, B, C) 57

APPENDIX B: SCHEDULE OF PROCEDURES – GROUP 2 (A, B, C) 59

APPENDIX C: SCHEDULE OF PROCEDURES – GROUP 3 (A, B, C, D, E, F) 62

APPENDIX D: LOW RISK CRITERIA....65

APPENDIX E: ADVERSE EVENT SEVERITY ASSESSMENT TABLE 67

APPENDIX F CTCAE TABLE 91

APPENDIX G REFERENCES....165

ABBREVIATIONS

Abbreviation	Term
**AE**	Adverse Event
**AIDS**	Acquired Immunodeficiency Syndrome
**ALT**	Alanine-Aminotransferase
**ART**	Antiretroviral Therapy
**AST**	Aspartate-Aminotransferase
**CFC**	Cytokine Flow Cytometry
**CMI**	Cell Mediated Immunity
**CRF**	Case Report Form
**CTL**	Cytotoxic T Lymphocyte
**DCC**	Data Coordinating Center
**DLT**	Dose Limiting Toxicity
**DNA**	Deoxyribonucleic Acid
**ELISA**	Enzyme Linked Immunosorbent Assay
**GCP**	Good Clinical Practice
**GFR**	Glomerular Filtration Rate
**HIV**	Human Immunodeficiency Virus
**HLA**	Human Leukocyte Antigen
**HSV**	Herpes Simplex Virus
**IAVI**	International AIDS Vaccine Initiative
**IDES**	Internet Data Entry System
**ICH**	International Council for Harmonization of Technical Requirements for Pharmaceuticals for Human Use
**IP**	Investigational Product
**IND**	Investigational New Drug Application
**IV**	Intravenous
**Kg**	Kilogram
**mAb**	Monoclonal Antibody
**mg**	Milligram
**MED**	Minimum Effective Dose
**MTD**	Maximum Tolerated Dose
**PCR**	Polymerase Chain Reaction
**PBMC**	Peripheral Blood Mononuclear Cells
**PK**	Pharmacokinetic
**RPR**	Rapid Plasma Reagin
**SAE**	Serious Adverse Event
**SIV**	Simian Immunodeficiency Virus
**SOP**	Standard Operating Procedure
**SOM**	Study Operations Manual
**SMC**	Safety Monitoring Committee
**STD**	Sexually Transmitted Disease
**TPHA**	Treponema Pallidum Hemagglutination

CONTACT INFORMATION

Detailed contact information provided in the Study Operation Manual (SOM)

Sponsor Contact:	
Frances Priddy MD MPH<br>Executive Director and Chief Medical Officer<br>International AIDS Vaccine Initiative<br>125 Broad Street, 9th Floor<br>New York, New York 10004	Phone: +1-212-328-7461<br>Mobile: +1-646-287-8943<br>Fax: +1-608-203-5501<br>E-mail: fpriddy@iavi.org
**Clinical Research Center Contacts:**	
Kathryn Stephenson MD MPH<br>Center for Virology and Vaccine Research<br>Clinical Trials Unit<br>Beth Israel Deaconess Medical Center<br>E / CLS – 1036<br>330 Brookline Avenue<br>Boston, Massachusetts 02215	Phone: +1-617-735-4556<br>Mobile: +1-917-836-9150<br>Fax: +1-617-735-4566<br>E-mail: kstephen@bidmc.harvard.edu

.

1.0 SIGNATURE PAGE

The signatures below constitute the approval of this protocol and the appendices and provide the necessary assurances that this study will be conducted in compliance with the protocol, Good Clinical Practices (GCP) and the applicable regulatory requirement(s).

Sponsor:

Signed: See electronic signature manifest Date:

Frances Priddy MD MPH
Executive Director and Chief Medical Officer, Medical Affairs, IAVI

Principal Investigator:

Signed: Date:

Name (please print):

Name of institution (please print):

2.0 INTRODUCTION AND BACKGROUND INFORMATION

More than 78 million people have been infected with HIV and 39 million people have died since the beginning of the AIDS epidemic[1]. In 2014, there were 1.2 million deaths attributable to HIV infection and 2 million newly infected with HIV[2]. One reason that such high rates of AIDS-related deaths continue to occur globally – despite the advent of drugs that are highly effective at suppressing HIV replication – is that only two in five people living with HIV have access to antiretroviral therapy[1]. The other reason for continued AIDS-related mortality is that ART does not cure HIV infection and must be maintained for a lifetime. Even in the United States (US), only 30% of the 1.2 million people living with HIV have suppressed HIV to undetectable levels, despite the fact that most HIV-infected people in the US have access to ART[3] It is clear that antiretroviral therapy is necessary but not sufficient to end the AIDS epidemic, both in the US and globally, and that novel efforts to prevent and treat HIV are critically needed.

2.1 Study Rationale

This is a Phase 1 study to evaluate the safety, tolerability, pharmacokinetics and anti-viral efficacy of the PGT121 monoclonal antibody for HIV prevention and therapy. PGT121 mAb is a recombinant human IgG1 monoclonal antibody that targets a V3 glycan-dependent epitope region of the HIV envelope protein[4,5]. PGT121 mAb was chosen for this study because it is potent, neutralizes a wide array of HIV viruses, and can prevent and treat simian-human immunodeficiency virus (SHIV) in rhesus monkeys.

The recent discovery of multiple potent and broadly neutralizing antibodies (bNAbs) against HIV has led to the re-emergence of the concept that antibodies may be useful for both prevention and therapy. HIV-specific antibodies that target the HIV envelope (Env) can prevent SHIV infection in rhesus monkeys and have shown to reduce HIV RNA levels in humans temporarily[6-10]. Until recently, these antibodies were few in number, targeted a narrow spectrum of HIV strains and Env epitopes, and were not potent enough for practical use. In the last five years, the field has changed dramatically: new developments in high throughput single-cell PCR-amplification and novel soluble Env baits have led to the isolation of new monoclonal antibodies with extraordinary potency and breadth[11]. These bNAbs may be effective for prevention of HIV infection when administered passively[12,13].

PGT121 mAb was selected for development because of the following critical attributes:

- PGT121 mAb is 10 to 100-fold more potent than the previous best-in-class CD4bs antibodies VRC01, VRC07, and 3BNC117 [11,14,15].
- PGT121 mAb affords superior protective efficacy against SHIV acquisition in monkeys compared to VRC01, 3BNC117, and 10-1074 [16] (and unpublished data).
- PGT121 mAb has superior therapeutic efficacy in SHIV-infected monkeys compared to VRC01, 3BNC117, and 10-1074 [7] (and unpublished data).
- PGT121 mAb may have a higher bar to escape in vivo as compared with other V3 glycan and CD4bs antibodies as a result of making multiple glycan contacts [14].
- PGT121 mAb combined with PGDM1400 (a novel bNab targeting the envelope trimer apex) neutralizes 98-99% of global HIV-1 viruses tested and has unparalleled potency with a median IC50 of 0.007 μg/ml[14].

The potency and breadth of PGT121 mAb, both alone and in combination with other bNAbs, raise the possibility that combinations may be effective for HIV prophylaxis at

low doses and against global viruses. An antibody that is effective at low doses may eventually be given subcutaneously, which would reduce the cost. It is these features that make PGT121 mAb particularly well-suited for preventing and/or treating HIV in the developing world, where it is critical that a public health intervention be low cost, easy to deliver, and effective in diverse settings.

2.2 Experience with PGT121

There is no previous clinical experience with PGT121 mAb. Several other HIV monoclonal antibodies are currently in clinical development as passive HIV immunoprophylaxis, or as potential therapeutics. Data from phase 1 studies shows acceptable preliminary safety and tolerability profiles for these products, but varying levels of anti-viral effects [6,17]. A comprehensive summary of phase 1 studies of HIV monoclonal antibodies can be found in the Investigator's Brochure.

3.0 STUDY OBJECTIVES

3.1 Primary Objectives

- To evaluate the safety and tolerability of a single intravenous (IV) infusion of PGT121 mAb at 3, 10, and 30 mg/kg in HIV-uninfected adults and HIV-infected adults.
- To evaluate the pharmacokinetic (PK) profile of IV infusion of PGT121 mAb in HIV-uninfected and HIV-infected adults.
- To evaluate the antiviral activity of IV infusion of PGT121 mAb in HIV-infected adults not on ART.

3.2 Secondary Objectives

- To determine if PGT121 mAb induces anti-PGT121 antibodies.
- To determine the effect of PGT121 mAb on CD4 T-cell counts in HIV-infected adults.
- To determine if PGT121 mAb induces viral escape mutants in viremic HIV-infected adults not on ART.

3.3 Exploratory Objectives:

- To determine if PGT121 mAb has any impact on the host immune responses (i.e., HIV-specific cellular and humoral immune response).
- To determine the effect of PGT121 mAb on the size of the latent HIV reservoir in HIV-infected adults.
- To determine PGT121 mAb levels in mucosal secretions in HIV-uninfected and HIV-infected adults.
- To measure in vitro neutralization of HIV isolates with participant's serum post PGT121 mAb IV infusion.
- To determine if PGT121 mAb has any impact on resistance mutations to ARVs.

4.0 STUDY ENDPOINTS

4.1 Study Endpoints

4.1.1 Primary Endpoints

Safety and Tolerability:

We will measure the following endpoints to evaluate the safety and tolerability of PGT121 mAb in HIV-uninfected and HIV-infected adults:

1. Proportion of participants with moderate or greater reactogenicity (e.g., solicited adverse events) for 3 days following IV infusion of PGT121 mAb.
2. Proportion of participants with moderate or greater and/or PGT121 mAb-related unsolicited adverse events (AEs), including safety laboratory (biochemical, hematological) parameters, following IV infusion of PGT121 mAb for the first 56 days post administration of Investigational Product.
3. Proportion of participants with PGT121 mAb-related serious adverse events (SAEs) throughout the study period.

Pharmacokinetics:

We will measure the following endpoints to determine the pharmacokinetics following IV infusion of PGT121 mAb in HIV-uninfected and HIV-infected adults:

1. Elimination half-life (t1/2)
2. Clearance (CL/F)
3. Volume of distribution (Vz/F)
4. Area under the concentration decay curve (AUC)
5. Impact of viral load and/or ART on PGT121 disposition (elimination half-life (t1/2), clearance (CL/F), volume of distribution (Vz/F), total exposure

Antiviral activity:

We will calculate the following endpoints to determine the antiviral activity following IV infusion of PGT121 mAb in viremic HIV-infected adults not on ART.

1. Change in plasma HIV-1 RNA levels from baseline (mean of pre-entry and entry values)

4.1.2 Secondary Endpoints

Anti-PGT121 antibodies:

We will calculate the following endpoint to determine if anti-PGT121 antibodies are elicited following IV infusion of PGT121 mAb in HIV-uninfected and HIV-infected adults:

1. Change in serum anti-PGT121 antibody titers from baseline

CD4+ T cell count:

We will calculate the following endpoint to determine if IV infusion of PGT121 mAb has any impact on CD4+ T cell counts in HIV-infected adults:

1. Change in CD4+ T cell count and frequency compared to baseline as measured by single platform flow cytometry

HIV genotyping/phenotyping of circulating virus for evaluation of PGT121-induced escape mutations:

We will compare plasma virus sequences before and after IV infusion of PGT121 mAb to determine if PGT121 mAb induces viral escape mutations in viremic HIV-infected adults not on ART.

1. Development of sequence variations in epitopes known to result in reduced PGT121 neutralization susceptibility

4.1.3 Exploratory Endpoints

Additional assessments may include but are not limited to the following: HIV-specific IgG/IgA binding responses by ELISA, HIV-specific cellular immune responses by ELISPOT, HIV-specific antibody function by ADCC, ADCP, and ADCVI assays, resistance mutations to current ARVs, PGT121 mAb levels in mucosal secretions, changes in total HIV-1 DNA and 2-long terminal repeat (LTR) circular HIV-1 DNA in resting or total CD4 T cells and in vitro neutralization of HIV isolates with participant's serum post IV infusion of PGT121 mAb.

5.0 STUDY DESIGN

The study is a randomized, placebo-controlled study for groups 1 and 2, and open label for group 3 who will not receive placebo.

5.1 Definition of Dose Limiting Toxicity and Maximum Tolerated Dose

A dose limiting toxicity (DLT) will be defined as 1) any Grade 3 or greater reactogenicity or adverse event judged by the study investigators as at least possibly related to investigational product or 2) any SAE considered at least possibly related to investigational product.
Maximum Tolerated Dose (MTD) will be declared when 2 or more DLTs occur that are the same, similar, or in the same System Organ Class or if no DLT occurs in the final dose subgroup, MTD will be the highest dose given (groups 1C and 2C 30mg/kg) after 14 days of follow-up.

5.2 Dose Escalation – Groups 1 and 2: Determination of Maximum Tolerated Dose

In Groups 1 and 2, (Part 1), the administrations of PGT121 mAb escalate by dose as shown below in Table 5.3.1, Study Design (5 participants per dose subgroup, 4:1 ratio of IP to placebo for each dose subgroup).

Sentinel Recipients

Within each dose group (subgroups 1A and 2A, subgroups 1B and 2B, subgroups 1C and 2C), the first 3 participant infusions will be separated by at least 4 days, to allow for observation of Investigational product (IP)-related adverse events. Dose subgroups will be enrolled in parallel, meaning that the 1st participant may be from subgroup 1A, the 2nd

from subgroup 2A, the 3rd from subgroup 2A, all with 4 days in between dosing. Because there is 1 placebo in each dose subgroup and the subgroups are dosed in parallel, the first 3 recipients will be treated as sentinel recipients (randomization will ensure that at least 2 will receive the IP). If no reactogenicity and adverse events that are considered to be related to IP (possibly, probably or definitely related) and are graded as severe or worse (Grade 3 or 4 on the DAIDS Toxicity Table or CTCAE table, see section 9.1.2) occur within 4 days after infusion of the first participant, the second participant may be injected. If no events meeting the criteria described above occur within 4 days after the 3rd participant is infused, then the remainder of participants in that dose group will be infused. If events meeting the criteria described above do occur for the first 3 participants in a dose group, they will be reviewed by the Safety Monitoring Committee (SMC) to determine whether further infusions may proceed.

Dose Escalation and Determination of Maximum Tolerated Dose

Safety data through at least day 14 post-IP administration visit for the first 5 participants in a dose subgroup (e.g. 1A) will be reviewed by the Protocol Safety Review Team (PSRT) prior to allowing enrollment of participants into the next dose subgroup (e.g. 1B). Dose escalation in Groups 1 and 2 may occur in parallel, and can occur independent of each other. Following administration of IP for the last participant in both Groups 1 and 2, an independent Safety Monitoring Committee (SMC) will review safety data to confirm Maximum Tolerated Dose (MTD) and determine whether, and at what dose, Group 3 can initiate enrollment.

If no DLT occurs within 2 weeks from infusion of the first 5 participants in a dose subgroup (e.g. 1A), dose escalation to the next dose subgroup (e.g. 1B) will proceed. If 1 DLT occurs in a dose subgroup (e.g. 1A), 3 additional participants will be enrolled in that dose subgroup; these 3 participants will be randomized between PGT121 mAb and placebo at a 2:1 ratio. If no additional DLTs occur in that dose subgroup (e.g. 1A) within 2 weeks of infusion in the 8 total participants (3:1 ratio of PGT121 mAb to placebo), the study can proceed with enrollment of the next dose subgroup (e.g. 1B). If 2 or more DLTs accumulate in a dose subgroup (e.g. 1A) that are the same, similar, or in the same organ class, dosing will be halted in that subgroups (e.g. 1A) and the next lower dose level will be declared the maximum tolerated dose (MTD) for that subgroup (e.g. 1A). When groups are enrolled in parallel, if the MTD is determined in one group (e.g. Group 1) due to the occurrence of 2 or more DLTs in this group, dosing of participants in the parallel group (e.g. Group 2) will be held until the PSRT has reviewed the safety data and determined whether the MTD should be applied to both groups. If no DLT occurs in the final dose subgroups, MTD will be the highest dose given (subgroups 1C and/or 2C 30mg/kg) after 14 days of follow-up.

5.3 Dose De-Escalation- Group 3: Determination of Minimum Effective Dose

Upon approval by the SMC (see section 17.2.2), group 3 (Part 2), PGT121 mAb administrations will de-escalate by dose as shown below in Table 5.3.1.

Group 3 will start with the MTD (i.e. subgroups 3A and 3D if the MTD is 30 mg/kg, subgroups 3B and 3C if the MTD is 10 mg/kg, or subgroups 3C and 3F if the MTD is 3 mg/kg) as determined by the SMC from data in Part 1.

If subgroup 3A (n = 6) achieves a decline in HIV RNA significantly greater than ≥0.9 log compared to baseline, enrollment into subgroup 3A will be stopped, and the next lower dose subgroup 3B will be initiated. The same rule will apply for progression from subgroup 3B to 3C. At least 6, 8 and 10 but up to 9, 12 and 15 participants will be enrolled in subgroups 3A, 3B and 3C respectively, until the minimum effective dose is determined. In each subgroup, if a decline significantly greater than 0.9 log in HIV RNA is not achieved, enrollment will be stopped at the completion of enrollment at that dose level.

Three participants will be enrolled in each group 3D, 3E and 3F. As soon as subgroup 3D has enrolled 3 participants, enrollment into subgroup 3E will be initiated. The same rule will apply for progression from subgroup 3E to 3F. In the event that dose de-escalation in subgroups 3A-C is stopped due to failure to meet virologic endpoints, dose de-escalation in subgroups 3D-F will continue.

Table 5.3.1 Study Design Table

	Group	Participants	Sub-Group	Regimen	N	Dose (mg/kg)
**Part 1**	1	HIV-uninfected participants	1A	PGT121/Placebo	4/1 (6/2 if DLT)	3
			1B	PGT121/Placebo	4/1 (6/2 if DLT)	10
			1C	PGT121/Placebo	4/1 (6/2 if DLT)	30
	2	HIV-infected on ART, (<50 cp/ml)	2A	PGT121/Placebo	4/1 (6/2 if DLT)	3
			2B	PGT121/Placebo	4/1 (6/2 if DLT)	10
			2C	PGT121/Placebo	4/1 (6/2 if DLT)	30
Safety Monitoring Committee Review						
**Part 2**	3	HIV-infected off ART (VL $2x10^3$ – $1x10^5$cp/ml)	3A	PGT121	6 (max 9)	30
			3B	PGT121	8 (max 12)	10
			3C	PGT121	10 (max 15)	3
		HIV-infected off ART (VL $1x10^2$ – $2x10^3$ cp/ml)	3D	PGT121	3	30
			3E	PGT121	3	10
			3F	PGT121	3	3

Table 5.3.2

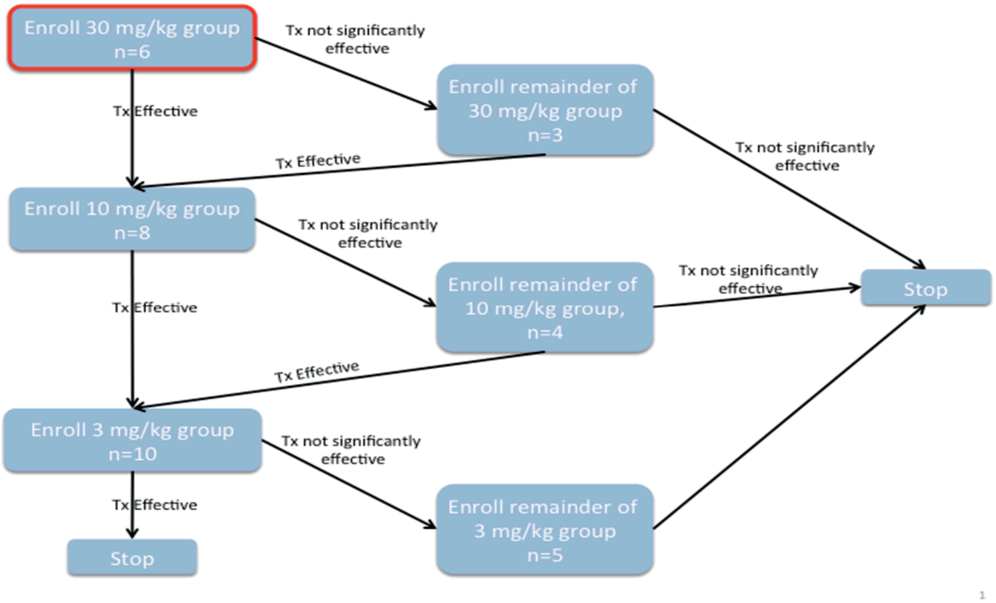

"not significantly effective" = does not achieve a decline in HIV RNA significantly greater than 0.9 logs compared to baseline

5.4 Duration of the Study

Participants will be screened up to 56 days (Groups 1 and 2) or 42 days (Group 3) before IP administration of PGT121 mAb and will be followed for 24 weeks.

It will take approximately 8 months to enroll the entire study. The exact duration depends on the recruitment rate and how many participants will be required per dose group as specified in sections 5.2 and 5.3.

5.5 Study Population

The study population consists of HIV-uninfected male or female adults (Group 1), HIV-infected male or female adults on ART (Group 2), and HIV-infected males and female adults not on ART (group 3) who meet the detailed inclusion and exclusion criteria listed below, and who in the opinion of the investigator or designee, understand the study and provide written informed consent.

Approximately 63-93 participants (81 investigational product recipients, 12 placebo recipients) who meet all eligibility criteria will be included in the study. An over-enrollment of up to 5% (up to 5 participants total) will be permitted in the study to facilitate rapid enrollment.

5.6 Inclusion Criteria

Inclusion criteria for all participants:

1. Willing to comply with the requirements of the protocol and available for follow-up for the planned duration of the study;

2. In the opinion of the Principal Investigator or designee and based on Assessment of Informed Consent Understanding results, has understood the information provided and potential impact and/or risks linked to IV infusion and participation in the trial; written informed consent will be obtained from the participant before any study-related procedures are performed;

3. All heterosexually active female participants must commit to use an effective method of contraception for 3 months following IP administration, including:
    a. Condoms (male or female) with or without spermicide
    b. Diaphragm or cervical cap with spermicide
    c. Intrauterine device, or contraceptive implant
    d. Hormonal contraception
    e. Successful vasectomy in the male partner (considered successful if a woman reports that a male partner has [1] documentation of azoospermia by microscopy (< 1 year ago), or [2] a vasectomy more than 2 years ago with no resultant pregnancy despite sexual activity post-vasectomy)
    f. Not be of reproductive potential, such as having undergone hysterectomy, bilateral oophorectomy, or tubal ligation, postmenopausal (>45 years of age with amenorrhea for at least 2 years, or any age with amenorrhea for at least 6 months and a serum follicle stimulating hormone [FSH] level >40 IU/L); surgically sterile: no additional contraception required.

    Women, who are not heterosexually active at screening, must agree to utilize an effective method of contraception if they become hetero-sexually active, as outlined above.

4. All sexually active males, regardless of reproductive potential, must be willing to consistently use an effective method of contraception (such as consistent male condoms with male and/or female partners from the day of IP administration until at least 3 months following IP administration to avoid exposure of partners to IP in ejaculate, and to prevent conception with female partners.

5. All female participants must be willing to undergo urine pregnancy tests at time points indicated in the Schedule of Procedures and must test negative prior to IP administration;

6. A woman must agree not to donate eggs (ova, oocytes) for the purpose of assisted reproduction until 3 months after receiving IP administration. A man must agree not to donate sperm until 3 months after IP administration;

7. Willing to forgo donations of blood and/or any other tissues, including bone marrow, during the study and, for those HIV-uninfected participants who test HIV-positive due to IP administration, until the anti-HIV antibody titers become undetectable.

Specific inclusion criteria for HIV-uninfected participants (Group 1):

8. At least 18 years of age on the day of screening and has not reached his or her 51st birthday on the day of signing the Informed Consent Document.

9. Willing to undergo HIV testing, risk reduction counselling and receive HIV test results;

10. Low risk for HIV infection (see section (9.4.6) and willing to maintain low-risk behaviour for the duration of the trial (Appendix D);

11. Healthy male or female, as assessed by a medical history, physical exam, and laboratory tests;

Specific inclusion criteria for HIV-infected participants (Groups 2 and 3):

12. At least 18 years of age on the day of screening and has not reached his or her 66th birthday on the day of signing the Informed Consent Document.

13. Confirmed HIV-1 infection (HIV Ab+ or HIV RNA+) by documentation in the medical records or in-clinic HIV testing;

14. CD4 ≥ 300 cells/µl;

15. No history of AIDS-defining illness

<u>Group 2:</u>

16. Currently on ART with no change in ART regimen in the 12 weeks before screening or between screening and enrolment, with suppression of plasma HIV-1 viral load < 50 copies / ml for greater than 6 months, measured on at least 2 independent occasions, and with a viral load < 50 copies / ml at time of screening (within 42 days prior to IP administration). cART is defined as a regimen including > 2 compounds, e.g. 2x nucleoside reverse transcriptase inhibitors plus either non-nucleoside reverse transcriptase inhibitor or protease inhibitor or integrase inhibitor.

<u>Group 3:</u>

17. Not receiving cART, and (after appropriate counselling) willing to defer cART treatment for at least 56 days after administration of IP;

18. HIV-1 viral load either between 2000-100,000 copies / ml (Group 3A, 3B, 3C) or between 100-2000 copies / ml (Group 3D, 3E and 3F) at 2 independent occasions within 12 months prior to study enrollment, with confirmation during the screening period (3 viral loads on independent occasions).

5.7 Exclusion Criteria

Exclusion criteria for all participants:

1. Any clinically significant acute or chronic medical condition, other than HIV infection, that is considered progressive or in the opinion of the investigator makes the participant unsuitable for participation in the study;

2. If female, pregnant, lactating or planning a pregnancy during the period of screening through completion of the study;

3. In the past 6 months a history of alcohol or substance use, including marijuana, judged by the Investigator to potentially interfere with participant study compliance;

4. Bleeding disorder that was diagnosed by a physician (e.g., factor deficiency, coagulopathy or platelet disorder that requires special precautions). Note: A participant who states that he or she has easy bruising or bleeding, but does not have a formal diagnosis and has intramuscular injections and blood draws without any adverse experience, is eligible;

5. History of a splenectomy;

6. Receipt of live attenuated vaccine within the previous 60 days or planned receipt within 60 days after administration of IP; or receipt of other vaccine within the previous 14 days or planned receipt within 14 days after infusion with IP (exception is live attenuated influenza vaccine within 14 days);

7. Receipt of blood transfusion or blood-derived products within the previous 3 months;

8. Participation in another clinical trial of an investigational product currently, within the previous 3 months or expected participation during this study;

9. Prior receipt of an investigational HIV vaccine candidate, monoclonal antibody or polyclonal immunoglobulin (note: receipt of placebo in a previous HIV vaccine or monoclonal antibody trial will not exclude a participant from participation if documentation is available and the Medical Monitor gives approval);

10. History of severe local or systemic reactogenicity to injections or IV infusion (e.g., anaphylaxis, respiratory difficulties, angioedema);

11. Psychiatric condition that compromises safety of the participant and precludes compliance with the protocol. Specifically excluded are persons with psychoses within the past 3 years, ongoing risk for suicide, or history of suicide attempt or gesture within the past 3 years;

12. If, in the opinion of the Principal Investigator, it is not in the best interest of the participant to participate in the trial;

13. Seizure disorder: a participant who has had a seizure in the last 3 years is excluded. (Not excluded: a participant with a history of seizures who has neither required medications nor had a seizure for 3 years.)

14. Body mass index ≥ 30 or ≤ 18.0.

15. Infectious disease: chronic hepatitis B infection (HbsAg), current hepatitis C infection (HCV Ab positive and HCV RNA positive) or interferon-alfa treatment for chronic hepatitis C infection in the past year, or active syphilis.

16. A history of malignancy within the past 5 years (prior to screening) or ongoing malignancy;

17. Active, serious infections (other than HIV-1 infection) requiring parenteral antibiotic, antiviral or antifungal therapy within 30 days prior to enrollment;

Specific exclusion criteria for HIV-uninfected participants (Group 1):

18. Confirmed HIV-1 or HIV-2 infection;

19. Any clinically relevant abnormality on history or examination including history of immunodeficiency or autoimmune disease; use of systemic corticosteroids, immunosuppressive, anticancer, or other medications considered significant by the investigator within the previous 6 months.

    The following exceptions are permitted and will not exclude study participation: use of corticosteroid nasal spray for rhinitis, topical corticosteroids for an acute uncomplicated dermatitis; or a short course (duration of 10 days or less, or a single injection) of corticosteroid for a non-chronic condition (based on investigator clinical judgment) at least 6 weeks prior to enrollment in this study.

Specific exclusion criteria for HIV-uninfected participants (Group 1) and HIV-infected participants who are on ART (Group 2):

20. Any of the following abnormal laboratory parameters listed below:

    <u>Hematology</u>

    – Hemoglobin <10.5 g/dL in females; hemoglobin <11.0 g/dL in males
    – Absolute Neutrophil Count (ANC): ≤1000/mm^3
    – Absolute Lymphocyte Count (ALC): < 650/mm^3
    – Platelets: < 125,000 mm^3 or ≥ 550,000/mm^3

    <u>Coagulation</u>

    - aPTT: >1.25x ULN
    - INR: ≥1.1 x ULN

    <u>Chemistry</u>

- Sodium ≤135 mEq/L or ≥ 146 mEq/L
- Potassium ≤3.4 mEq/L or ≥ 5.6 mEq/L
- Creatinine ≥1.1 x ULN
- AST ≥1.25 x ULN
- ALT ≥1.25 x ULN
- Total bilirubin ≥1.25 x ULN
- Alkaline phosphatase ≥1.25 x ULN
- Albumin ≤ 3.0 g/dL or ≤ 30 g/L
- Creatine kinase ≥3.0 x ULN
- C-reactive protein >10 mg/L
- C3 complement < 0.82 g/L
- C4 complement < 0.14 g/L

Urinalysis

Any of the following abnormal findings if consistent with clinically significant disease:
– Protein = greater than trace on dipstick confirmed by microscopic urinalysis outside institutional range
– Blood = greater than trace on dipstick confirmed by ≥ 3 RBCs/hpf on microscopic urinalysis (not due to menses)

Specific exclusion criteria for HIV-infected participants who are on ART (Group 2) and for HIV-infected participants who are not on ART (Group 3):

21. Any clinically relevant abnormality on history or examination including history of immunodeficiency or autoimmune disease, other than HIV; use of systemic corticosteroids, immunosuppressive, anticancer, or other medications considered significant by the investigator within the previous 6 months.

    The following exceptions are permitted and will not exclude study participation: use of corticosteroid nasal spray for rhinitis, topical corticosteroids for an acute uncomplicated dermatitis; or a short course (duration of 10 days or less, or a single injection) of corticosteroid for a non-chronic condition (based on investigator clinical judgment) at least 6 weeks prior to enrollment in this study.

Specific exclusion criteria for HIV-infected participants who are not on ART (Group 3)

22. Resistance of autologous HIV to PGT121 neutralization in vitro;

23. Any of the following abnormal laboratory parameters listed below:

Hematology

– Hemoglobin < 10.0 g/dL
– Absolute Neutrophil Count (ANC): <800 cells/mm3
– Platelets: < 100,000 cells/mm3

Coagulation

- aPTT: >1.25x ULN
- INR: ≥1.1 x ULN

Chemistry

- Estimated Glomerular filtration rate (GFR) $\leq$ 80 mL/min according to the Cockcroft-Gault formula for creatinine clearance:
  - Male: $\frac{(140 - \text{age in years}) \times (\text{wt in kg}) = \text{CLcr (mL/min)}}{72 \times (\text{serum creatinine in mg/dL})}$
  - Female: $\frac{(140 - \text{age in years}) \times (\text{wt in kg}) \times 0.85 = \text{CLcr (mL/min)}}{72 \times (\text{serum creatinine in mg/dL})}$

- AST ≥ 2.5 x ULN
- ALT ≥ 2.5 x ULN
- Total bilirubin ≥ 1.6 x ULN
- Alkaline phosphatase ≥ 5 x ULN

Urinalysis

Any of the following abnormal findings if consistent with clinically significant disease:
– Protein = greater than 1+ on dipstick confirmed by microscopic urinalysis outside institutional range
– Blood = greater than 1+ on dipstick confirmed by $\geq$ 10 RBCs/hpf on microscopic urinalysis (not due to menses)
- Leukocytes = greater than 1+ on dipstick confirmed by > 10 WBCs/hpf on microscopic urinalysis

5.8 Recruitment of Participants

Adult male and female participants may be recruited through in-clinic referrals, information presented to community organizations, hospitals, colleges, other institutions and/or advertisements to the general public or from existing cohorts. The information distributed will contain contact details of the trial site.

6.0 STUDY VISITS

6.1 Screening Period

During Screening, study staff will perform the following procedures:

- Provide and/or review the Informed Consent Document and answer any questions about the study prior to obtaining written informed consent.
- Complete Assessment of Informed Consent Understanding (AOU). Please refer to the Study Operations Manual (SOM)

If the participant agrees to participate, passes the AOU and provides written informed consent, study staff will:

- Conduct HIV test counselling, HIV testing, and HIV risk reduction counselling, as applicable
- Conduct family planning counselling, refer for pregnancy prevention counselling if necessary
- Administer HIV risk assessment (Group 1)
- Conduct ART counselling (Group 3)
- Perform a comprehensive medical history
- Collect concomitant medication information
- Perform a general physical examination (Refer to Section 7.2)
- Collect specimens for all tests as indicated in the Schedule of Procedures in Appendices A, B and C (for details see Analytical Plan (AP)).

When available, the screening laboratory tests will be reviewed by the trial physician. Screening laboratory test(s) may be repeated once at the discretion of the principal investigator or designee to investigate any isolated abnormalities.

If the screening visit occurs outside the allowable screening window, all screening procedures must be repeated except the comprehensive medical history may be replaced by an interim medical history and the Participant Information Sheet of the Informed Consent Document should be reviewed.

If a participant has signed the Consent Form but does not meet the eligibility criteria, the records must be kept at the site.

6.2 IV infusion of PGT121 mAb Visit

Prior to the administration of IP, study staff will:

- Answer any questions the participant may have about the study
- Review the Informed Consent Document with the participant
- Review screening safety laboratory data
- Conduct HIV test counselling, and HIV risk reduction counselling, as applicable
- Conduct ART counselling (Group 3)
- Conduct family planning counselling as per site specific procedures and ensure compliance with respective pregnancy prevention method, and discuss male condom use with all male participants
- Review interim medical history
- Collect concomitant medication information
- Weigh participant and record vital signs
- Perform a symptom-directed physical examination (Refer to Section 7.2)
- Assess at baseline local and systemic signs and symptoms (this includes an examination of IV infusion site)
- Collect specimens for all tests as indicated in the Schedule of Procedures see Appendices A, B and C (for details see AP).
- Obtain pregnancy test results prior to administration of IP.

Assign an allocation number to the participant according to the instructions specified in the Study Operations Manual.

At the time of administration of IP and after IV infusion of IP, study staff will:

- Administer the IP as specified in Section 8.4, Administration of Investigational Product and according to the instructions specified in the SOM.
- Observe participant closely during the infusion of IP and for at least 30 minutes after IV infusion of IP has ended for any acute reactogenicity. At the end of the observation period study staff will:
    - Record vital signs (pulse, respiratory rate, blood pressure and temperature)
    - Assess any local and systemic reactogenicity
    - Assess any other adverse events
- Every hour after IV infusion of IP, starting hour 1 through 12, the study staff will:
    - Record vital signs (pulse, respiratory rate, blood pressure and temperature)
    - Assess any local and systemic reactogenicity
    - Assess any other adverse events
    - Collect PK samples according to the Schedule of Procedures

If a participant has an abnormal laboratory value that is known, at the time of infusion, follow the specified guidelines (Section 12.0).

6.3 Post-IV infusion of PGT121 mAb Visits

The participant will be asked to return to the clinic for post-IP administration visits as indicated in the Schedule of Procedures (see Appendices A, B and C) for an assessment by clinic staff. The participant will be asked to maintain a Memory Aid to track any local and systemic reactogenicity the participant experiences, including temperature, from the day of IP administration for the next 3 days (for a total of 4 days including day of IP administration). Study staff will review the Memory Aid with the participant and determine the severity of the reactions through discussion with the participant.

The following procedures will be conducted at these visits:

- Review interim medical history
- Collect concomitant medication information
- Perform a symptom-directed physical examination if any signs or symptoms are present
- Assess vital signs (pulse, respiratory rate, blood pressure and temperature)
- Assess any adverse events and local and systemic reactogenicity (Days 1, 2, 3) including reviewing the Memory Aid.
- Collect specimens for all tests as indicated in the Schedule of Procedures (Appendices A, B and C and AP).

6.4 Additional Follow-up Visits

Assessments and procedures will be performed according to the Schedule of Procedures (Appendices A, B and C).

6.5 Unscheduled Visits

Unscheduled Visits/Contacts are visits/contacts that are not described in the Schedule of Procedures (Appendices A, B and C). Unscheduled visits may occur any time during the study:

- For administrative reasons, e.g., the participant may have questions for study staff or may need to re-schedule a follow-up visit.
- To obtain laboratory test results from a previous visit.
- For other reasons as requested by the participant or site investigator.

All unscheduled visits will be documented in the participants' study records on applicable source documents and entered into the Case Report Form (CRF).

6.6 Final Study Visit or Early Termination Visit

Assessments and procedures will be performed according to the Schedule of Procedures (Appendices A, B and C).

7.0 STUDY PROCEDURES

7.1 Informed Consent Process

A Master Informed Consent Document consisting of a Participant Information Sheet and a Consent Form is provided by the Sponsor to the trial site. This document is made site-specific and translated (if necessary), submitted and approved by the Institutional Review Board (IRB). The Master and site specific Informed Consent Documents are separate documents and should not be part of the protocol.

Participant Information Sheet

A qualified member of the study staff will conduct the informed consent process by reviewing the Participant Information Sheet and document it in the clinic notes.

Consent Form

The participant's consent to participate must be obtained by him/her signing and dating the Consent Form. The person obtaining consent will also sign.

The signed and dated Informed Consent Document must remain at the study site. A copy of the signed/marked and dated Informed Consent Document will be offered to the participant to take home. Those participants who do not wish to take a copy will be required to document that they declined to do so.

7.2 Medical History and Physical Examination

Medical History

At screening, a comprehensive medical history will be collected including previous IV infusions and reaction to IV infusion, history of sexually transmitted infection (STI) and pregnancy prevention practices. At subsequent visits, an interim medical history will be performed.

Physical Examination

General Physical Examination

A general physical examination includes examination of head/ears/eyes/nose and throat, skin, respiratory, cardiovascular, abdominal, limited neurological and musculoskeletal

and external ano-genital systems (for HIV-infected participants only) at the time points indicated in the Schedule of Procedures (see Appendices A, B and C).

Symptom-Directed Physical Examination
A symptom-directed physical examination is a targeted examination based on the participant's history or observation. If deemed necessary, this examination should be done at the time points indicated in the schedule of procedures (see Appendices A, B and C).

Measuring Height and Weight
Includes measuring the height and weight at the time points indicated in the Schedule of Procedures (see Appendices A, B and C).

Vital Signs
Vital signs including pulse, respiratory rate, blood pressure and temperature are measured and recorded at the time points indicated in the Schedule of Procedures (see Appendices A, B and C)

7.3 HIV Testing and HIV-test Counselling (Group 1)

Study staff will perform pre-HIV test counselling prior to collecting blood for an HIV test, and post-HIV test counselling when HIV test results are available. This is referred to as HIV-test counselling, and done according to the CDC guidelines. For more information on HIV testing and HIV-test counselling, see Section 11.0. A screening questionnaire and other tools may be used.

7.4 HIV Risk Reduction Counselling

HIV risk reduction counselling will be provided to all participants as outlined by site-specific SOPs.

Study staff will provide HIV risk reduction counselling based on reported individual risk and provide free condoms, as appropriate, at every visit. Group 1 will receive HIV risk reduction counselling and for Groups 2 and 3, HIV risk reduction counselling will be conducted as secondary prevention to reduce onward transmission.

7.5 Family Planning Counselling

Study staff will counsel participants about the importance of preventing pregnancies and of using condoms, as well as other effective family planning methods, as appropriate. Participants may be referred for family planning services as necessary according to site-specific SOPs as detailed in the SOM. Pregnancy prevention methods chosen and compliance will be documented.

7.6 ART Counselling (Group 3)

HIV-infected participants who are not on ART will receive ART counselling upon entering the study and 8 weeks after administration of IP. Participants who have not initiated or made plans to initiate ART by the final study visit will receive ART counselling again at their final study visit. HIV-infected participants who are on ART (Group 2) will be counselled on the importance of continuing ART throughout the study, and will not be required to interrupt ART after administration of IP.

7.7 Specimens

Approximately 50 ml of blood will be collected from participants in Group 1, approximately 78 ml from participants in Group 2, and approximately 150 ml of blood will be collected from participants in Group 3 at the screening visit. At later visits, approximately 8.5 ml to 175 ml of blood will be collected, depending on study procedures and group assignment (see Appendices A, B and C), usually from the antecubital fossa.

Optional collection of rectal and/or cervical mucosal secretions will be obtained using a rectal sponge (or comparable swab) or cervical Softcup (or comparable cervical fluid collection cup) for those participants that consent.

All specimens will be handled according to the procedures specified in the AP or SOPs, if applicable.

In the event of an abnormal laboratory value, participants may be asked to have an additional sample collected at the discretion of the Principal Investigator or designee.

7.8 Reimbursement

Participants will be reimbursed for their time, effort and for costs to cover their travel expenses to the study site and any inconvenience caused due to study participation. Site specific-reimbursement amounts will be documented in the site-specific Participant Information Sheet, and approved by the Institutional Review Board.

7.9 Randomization and Blinding

Participants will be identified by a unique study identification number.

Participants will be randomized according to the randomization schedule prepared by the statisticians at the Data Coordinating Center (DCC) prior to the start of the study. Participants will be automatically assigned a specific allocation number as they are enrolled into the data entry system. An unblinding list (Pharmacy List) will be provided to the unblinded site pharmacist by the DCC.

This is a randomized, double-blind placebo-controlled study for groups 1 and 2, and an open label study for group 3. For Groups 1 and 2, study staff (investigator and clinical personnel monitoring the safety and laboratory assay results) and participants will be blinded with respect to the allocation of Investigational Product (PGT121 mAb or placebo). A site pharmacist will be unblinded for the purposes of preparing study product.

A participant will be considered enrolled once he/she has been assigned an allocation number.

Blinded participants will be informed about their assignment (product/placebo) at study completion, once the database is locked. Should a study participant be unblinded during the study, the study participant will be followed up until the end of the study according to the Schedule of Procedures (see Appendices A and B).

7.10 Un-blinding Procedure for Individual Participants

Un-blinding of an individual participant may be indicated in the event of a medical emergency if the clinical management of the participant would be altered by knowledge of the treatment assignment.

The un-blinded information should be restricted to a small group of individuals involved in clinical management/medical treatment of the participant (e.g. treating physician) and the blind must be maintained for those responsible for the study assessments.

The reasons for un-blinding should be documented and the IAVI Chief Medical Officer, the Medical Monitor and the DCC should be notified as soon as possible. The procedures and contact numbers for un-blinding are outlined in the SOM.

7.11 Assessment of IP related HIV sero-positivity

It is possible that PGT121 mAb or an immune response to PGT121 mAb could cause a positive result on a diagnostic HIV antibody test. A Group 1 participant who tests HIV antibody positive at the end of the study will have additional testing to distinguish actual HIV infection from IP-related responses. The participant will be informed of his/her positive HIV antibody test result and offered continuing follow-up until the HIV antibody test becomes negative.

8.0 INVESTIGATIONAL PRODUCT

8.1 Description

A summary of the Investigational Products is shown in Table 8.1-1.

Table 8.1-1 Investigational Products

**IP (Active Product / Placebo)**	**Dosage level**	**Total volume in IP container**	**Total IP (Active Product or placebo) volume to be injected into a 100 mL saline IV bag** (for an 88 kg body weight**)	**Total volume to be Infused** (for an 88 kg body weight**)
PGT121 (50 mg/mL)	3 mg/kg	6 mL per vial	5.3 mL	105.3 mL
	10 mg/kg		17.6 mL	117.6 mL
	30 mg/kg		52.8 mL	152.8 mL
Placebo: 0.9% Sodium Chloride Injection USP (Saline)*	3 mg/kg matching***	NA	5.3 mL***	105.3 mL***
	10 mg/kg matching***		17.6 mL***	117.6 mL***
	30 mg/kg matching***		52.8 mL***	152.8 mL***

* The Placebo provided will be a commercially-available saline partial addition IV bag.

** The actual volume to be injected will be based on the dose group and the weight of the participant at the time of IP administration. The example included here is the average weight of an adult male in the US (88kg) (http://www.cdc.gov/nchs/data/series/sr_11/sr11_252.pdf)

*** For placebo IV infusions: saline from an additional IV bag will be injected into the saline IV bag intended for administration, to match the volume used for a PGT121 mAb injection in the same dose group, to prevent unblinding.

8.2 Shipment and Storage

Authorization to ship the Investigational Product to the site will be provided in writing by the Sponsor, upon confirmation that all required critical documents for shipment authorization are completed. The Investigational Product will be shipped maintaining the required storage conditions and stored in a secure location in the clinical site's pharmacy.

The Investigational Product is formulated in a 20 mM Acetate, 9% Sucrose, 0.008% polysorbate 80, pH 5.2 formulation buffer at a concentration of 50 mg/mL. Each 10 ml vial will contain 6 ml of IP stored at <- 20°C. Each vial will be labelled with the name of the product, Lot number, concentration, storage temperature, date of manufacturing, contact information of the Sponsor and a US cautionary statement. Several such vials will be packaged in a box. Each box will also be labelled with similar information as the vial label.

8.3 Preparation of Investigational Product (IP)

Detailed instruction will be provided to the site pharmacist in the SOM for preparing each of the investigational products. The site pharmacist will not be blinded, but the study physician/designee administering the IP will be blinded. Product should be administered within 4 hours of preparation. Example calculations for final volume for IV infusion are illustrated in Table 8.1-1. Procedures for handling used and partially used vials will be provided in the SOM.
Syringes or other components in direct contact with investigational products will be disposed of in a biohazard container and incinerated or autoclaved.

8.4 Administration of Investigational Product

Investigational Product will be administered at the enrollment visit.

The IP will be injected into a 0.9% Saline bag. The participant will receive the IP via IV infusion. Participants will receive infusion over approximately 60 minutes, allowing for clinician discretion. Further information on the IV infusion of the IP is supplied in the SOM and study documents.

8.5 Accountability and Disposal of Investigational Product

All used IP vials will be handled according to instructions in the SOM. The date, allocation number and location of storage of the returned vials will be recorded.

During the study, the IP accountability forms including receipt and dispensing of vials will be kept and monitored.

At the end of the study, the used and unused IP vials will be handled according to instructions of Sponsor.

Further information on accountability and disposal of IP is supplied in the SOM.

9.0 ASSESSMENTS

9.1 Safety Assessments

Data on local and systemic reactogenicity (i.e., solicited AEs) will be collected by structured interview and medical examination. Data on other adverse events will be collected with open-ended questions. All data will be recorded on the appropriate source documents and entered into the study database. Participants will be given a Memory Aid, which is a tool to assist with collecting reactogenicity data.

Local and systemic reactogenicity events will be assessed by study staff prior to IV infusion of IP, at approximately 30 minutes after IP administration start, at 1 hour after IP administration start, and subsequently every hour for the first 12 hours post-IP administration. Study staff will review the Memory Aid with the participant, and determine the severity of the reactions on days 1-3 through discussion with the participant.

9.1.1 Local reactogenicity

The presence of local reactogenicity will be assessed at the time points specified in the Schedule of Procedures (Appendices A, B and C).
Pain, tenderness, erythema/skin discoloration, swelling/hardening or pruritus will be assessed and graded using Appendix E, Adverse Event Severity Assessment Table, as a guideline.

9.1.2 Systemic reactogenicity

The presence of systemic reactogenicity will be assessed at the time points specified in the Schedule of Procedures (Appendices A, B and C).
Fever, chills, headache, nausea, vomiting, malaise, myalgia and arthralgia will be assessed and graded using the Appendix E, Adverse Event Severity Assessment Table as a guideline. For the first 24 hours after IP infusion, any infusion related reactions, including cytokine release syndrome, should be graded using the Common Terminology Criteria for Adverse Events (CTCAE) v4.03: June 14, 2010 (Appendix F).

9.1.3 Vital Signs

At the administration of IP visit, vital signs (pulse, respiratory rate, blood pressure and temperature) will be measured by study staff prior to IP administration, at approximately 30 minutes post IP administration and hourly until 12 hours after IV infusion start. For the other study visits vital signs will be assessed at the time points specified in the Schedule of Procedures (Appendices A, B and C).

9.1.4 Other Adverse Events

Other adverse events (AEs) will be collected through 56 days after IP administration in all participants. Serious Adverse Events (SAEs) will be collected throughout the entire study period. Potential Immune Mediated Diseases

(pIMDs), as defined in Section 10.5, will be collected throughout the study period, using the SAE reporting process. Open-ended questions will be asked at time points according to the Schedule of Procedures (Appendices A, B and C). All adverse events will be graded using Appendix E, Adverse Event Severity Assessment Table, as a guideline and will be assessed for causality to the IP. For more information regarding adverse events refer to Section 10.0, Adverse Events.

9.1.5 Concomitant Medications

Concomitant receipt of Investigational Products is prohibited during the study.

Contraceptive use and use of medication at study entry will be documented. (See DCF instructions)

During the study, information regarding concomitant medications and reasons for their use will be solicited from the study participants for 56 days. Ongoing concomitant medications will be recorded until end of study.

9.1.6 Routine laboratory parameters

Table 9.1.6-1 shows the laboratory parameters that will be measured routinely. The samples for these tests will be collected at the time points indicated in the Schedule of Procedures (Appendices A, B and C).

Table 9.1.6-1: Laboratory Parameters

Laboratory Parameter	Test
Hematology and Coagulation	Hemoglobin, hematocrit, leukocytes, platelets, absolute neutrophil count (ANC), absolute lymphocyte count (ALC), activate partial thromboplastin time (aPTT), international normalized ratio (INR)
Clinical Chemistry	Sodium, potassium, creatinine, aspartate aminotransferase (AST), alanine aminotransferase (ALT), total bilirubin, alkaline phosphatase<br>Groups 1 and 2 only: albumin, creatine kinase, C-reactive protein, C3 complement, C4 complement
Urinalysis	Dipstick test for protein, blood glucose, ketones, esterase (leukocytes) and nitrite. If clinically significant abnormalities (e.g., blood, protein, leukocytes) are found on dipstick test, then further test(s) will be performed (e.g., microscopy, culture)
T cell panel (Groups 2 and 3)	CD4 T cell count and frequency by single platform flow cytometry

9.1.7 Specific screening tests:

Participants will be screened to exclude the following diseases:

- Hepatitis B: positive for hepatitis B surface antigen (HBsAg)

- Hepatitis C: positive for hepatitis C RNA (HCV antibody test, followed by HCV RNA test if HCV antibody positive)
  Active syphilis: confirmed diagnosis.

A negative Hepatitis B and Hepatitis C result can be documented from the medical record only if the result is from a test administered less than 6 months ago.

Participants will also be screened to exclude the following laboratory parameters:
- Resistance of autologous HIV to PGT121 neutralization *in vitro*- (HIV viremic participants only, Group 3)

9.1.8 Monitoring for anti-PGT121 antibodies:

Participants will be evaluated for the development of antibodies to PGT121 mAb (anti-drug antibodies, ADA) by ELISA according to the Schedule of Procedures (Appendices A, B and C).

9.2 Virologic Assessments

Table 9.2-1 shows the virologic parameters that will be measured routinely. The samples for these tests will be collected at the time points indicated in the Schedule of Procedures (Appendices B and C).

Table 9.2-1: Virologic Assessment Table

Virologic Parameter	Test
Antiviral Activity	Plasma HIV RNA levels
Anti-reservoir activity	Cell-associated HIV-1 RNA levels in resting CD4 T cells; total HIV-1 DNA and 2-long terminal repeat (LTR) HIV-1 DNA circles in resting or total CD4 T cells; quantitative viral outgrowth assay (qVOA)
Other	Genotyping of plasma HIV RNA for evaluation of PGT121-induced escape mutations; phenotyping of plasma HIV RNA for neutralization susceptibility to PGT121 in-vitro

9.3 Exploratory Immunogenicity Assessments

Humoral immune response assays will include, but are not limited to Env-specific Ab-binding assays, virus neutralization assay, and assays for Ab functionality. Cellular immune response assays will include, but are not limited to IFNγ ELISPOT assay, ICS, and multiparameter flow cytometry. Exploratory assessments on mucosal samples will include, but are not limited to characterization of Env-specific binding Abs. Priority assays are listed below.

9.3.1 Antibody Responses

- Env-specific binding Abs (titers and breadth).
- Env-specific nAbs (titers and breadth).
- Env-specific functional Abs (phagocytosis score and breadth).

- Env-specific binding Ab isotypes (IgA, IgG1-4) (titers and breadth).

9.3.2 Cellular Responses

- IFNγ peripheral blood mononuclear cell (PBMC) responders to peptide pools and subpools of Potential T-cell epitopes, PTE Env/Gag/Pol peptides.
- $CD4^{+}$ and $CD8^{+}$ T-cell functionality (% cells producing e.g. IFNγ, IL-2, IL-4, TNFα).
- T-cell development with emphasis on follicular helper T-cells and memory differentiation.

9.3.3 PBMC, Serum and Plasma Storage

Samples of cryopreserved PBMC, plasma and serum will be stored as indicated in the Analytical Plan (AP) and, if the participant consents, may be used for the purposes of standardization, quality control and for future assays related to HIV prevention or treatment research and development. These samples will be archived and the testing laboratories will be blinded to the participant's identity.

9.4 Other Assessments

9.4.1 HIV Antibody Testing (Group 1)

All HIV-uninfected participants (Group 1) will be tested for HIV antibodies as indicated in the Schedule of Procedures (Appendix A) or as needed, if medical or social circumstances arise. All participants will receive HIV risk reduction counselling and pre- and post-HIV-test counselling, as specified in Section 7.3 Counselling.

9.4.2 Pharmacokinetics

Blood draws for pharmacokinetics will be done on the day of IP administration immediately before starting IV infusion of IP, at the end of the IP administration, and 30 minutes and 3 hours after the end of the IP administration. Additional draws will be done at 6, 9, 12 and 24 hours after the start of the IP administration. Thereafter, pharmacokinetic draws will be done as indicated in the Schedule of Procedures (Appendices A, B and C). PGT121 mAb serum or plasma levels will be determined using two methods: a sandwich ELISA using a murine anti-idiotype antibody to PGT121 mAb, and a neutralization assay.

PGT121 mAb pharmacokinetic analysis will be performed using standard non-compartmental analysis methods to estimate elimination half-life (t1/2), clearance (CL/F), volume of distribution (Vz/F), Area under the concentration decay curve (AUC), impact of viral load and/or ART on PGT121 mAb disposition (elimination half-life (t1/2), clearance (CL/F), volume of distribution (Vz/F) and total exposure. PGT 121 accumulation will also be examined in rectal and cervical mucosal secretions collected with rectal sponges or cervical softcups in study participants who specifically consented for these procedures. Descriptive results will be reported for the pharmacokinetic parameters by dose subgroup.

Exploratory analysis using population analysis methods simultaneously combining all pharmacokinetic data across all doses and treatment groups will be

performed for quantitative characterization of differences in PGT121 mAb disposition by dose, participant group or disease state.

9.4.3 HLA Typing

Samples for HLA typing will be collected as specified in the AP and may be analyzed as warranted.

9.4.5 Pregnancy Test

A urine pregnancy test for all female participants will be performed by measurement of human chorionic gonadotrophin (βhCG) at time points indicated in the Schedule of Procedures (Appendices A, B and C). The results of the pregnancy test must be negative prior to IV infusion of PGT121 mAb. See section 10.7 for description of pregnancy after administration of IP.

9.4.6 HIV Risk Assessment (Group 1)

Study staff will assess participants for their past and current risk of acquiring HIV at time points indicated in Schedule of Procedures (Appendix A).

9.4.7 Social Impact Assessment

A brief assessment of the impact of participation in the study will be administered to participants at their final study visit.

10.0 ADVERSE EVENTS

10.1 Definition

An adverse event (AE) is any untoward medical occurrence in a participant administered an Investigational Product and which does not necessarily have a causal relationship with the Investigational Product. An AE can therefore be any unfavourable or unintended sign (including an abnormal laboratory finding), symptom, or disease, temporally associated with the use of Investigational Product whether or not related to the Investigational Product.

Assessment of severity of all AEs, including and seriousness of AEs, is ultimately the responsibility of the Principal Investigator of each site. Refer to the DIVISION OF AIDS (DAIDS) TABLE FOR GRADING THE SEVERITY OF ADULT AND PEDIATRIC ADVERSE EVENTS Version 2.0, November 2014 and the National Cancer Institute Common Terminology Criteria for Adverse Events (CTCAE) Version 4.03: June 14, 2010 for additional guidance.

10.2 Assessment of Severity of Adverse Events

The following general criteria should be used in assessing adverse events as mild, moderate, severe or very severe at the time of evaluation:

<u>Grade 1 (Mild):</u> Symptoms causing no or minimal interference with usual social & functional activities

<u>Grade 2 (Moderate):</u> Symptoms causing greater than minimal interference with usual social & functional activities

Grade 3 (Severe): Symptoms causing inability to perform usual social & functional activities

Grade 4 (Very Severe): Symptoms causing inability to perform basic self-care functions OR Medical or operative intervention indicated to prevent permanent impairment, persistent disability, or death

Guidelines for assessing the severity of specific adverse events and laboratory abnormalities are listed in Appendix E, Adverse Event Severity Assessment Table.

10.3 Relationship to Investigational Product

Assessment of relationship of an AE or SAE to Investigational Product (IP) is the responsibility of the Principal Investigator or designee. All medically indicated and available diagnostic methods (e.g., laboratory, blood smear, culture, X-ray, etc.) should be used to assess the nature and cause of the AE/SAE. Best clinical and scientific judgment should be used to assess relationship of AE/SAEs to the IP and/or other cause.

The following should be considered:

- Presence/absence of a clear temporal (time) sequence between administration of the IP and the onset of AE/SAE
- Presence/absence of another cause that could more likely explain the AE/SAE (concurrent disease, concomitant medication, environmental or toxic factors)
- Whether or not the AE/SAE follows a known response pattern associated with the IP

The relationship assessment should be reported as one of the following:

Not Related: clearly explained by another cause (concurrent disease, concomitant medication, environmental or toxic factors, etc.).

Unlikely: more likely explained by another cause (concurrent disease, concomitant medication, environmental or toxic factors, etc.).

Possibly: equally likely explained by another cause but the possibility of the IP relationship cannot be ruled out (e.g., reasonably well temporally related and/or follows a known IP response pattern but equally well explained by another cause).

Probably: more likely explained by the IP (e.g., reasonably well temporally related and/or follows a known IP response pattern and less likely explained by another cause).

Definitely: clearly related and most likely explained by the IP.

For the purpose of expedited safety reporting, all possibly, probably or definitely related SAEs are considered IP-related SAEs.

10.4 Serious Adverse Events

An adverse event is reported as a "Serious Adverse Event" if it meets any of the following criteria (as per International Conference on Harmonisation [ICH] Good Clinical Practice [GCP] Guidelines):

- Results in death
- Is life threatening
- Results in persistent or significant disability/incapacity
- Requires in-participant hospitalization or prolongs existing hospitalization
- Is a congenital anomaly/birth defect or spontaneous abortion
- Any other important medical condition that requires medical or surgical intervention to prevent permanent impairment of a body function or structure

Elective surgery for pre-existing condition that did not increase in severity or frequency is not considered an SAE.

Serious Adverse Events (SAEs) should be reported within 24 hours of the site becoming aware of the event, and sent to the Sponsor as described in the SOM.

To discuss IP-related SAEs or any urgent medical questions related to the SAE, the site investigator should contact one of the IAVI Medical Monitors directly (see Contact List in the SOM).

The IAVI SAE Report Form should be completed with all the available information at the time of reporting and sent to the Sponsor as described in the SOM. The minimum data required in reporting an SAE are the study identification number, date of birth, gender, event description (in as much detail as is known at the time), onset date of event (if available), reason event is classified as serious, reporting source (name of Principal Investigator or designee), and relationship to the IP as assessed by the investigator.

The Principal Investigator or designee is required to prepare a detailed written report with follow up until resolution or until it is judged by the Principal Investigator or designee to have stabilized.

The Principal Investigator or designee must notify the local IRB/IEC of all SAEs as appropriate. In case of IP-related SAEs, the Sponsor will notify responsible regulatory authorities, Safety Monitoring Committee (SMC), and other study sites where the same IP is being tested.

More details on SAE definitions and reporting requirements are provided in the SOM.

<u>Serious Event Prior to Investigational Product Administration</u>

If a serious event occurs in the period between the participant signing the Informed Consent Form and receiving the IV infusion of IP, the event will be reported using the SAE form and following the same procedures for SAE reporting, as indicated in Section 10.4. The timing of the event will be indicated by using the relevant checkbox on the SAE form.

10.5 Reporting Potential Immune-Mediated Diseases

Potential immune-mediated diseases (pIMDs) are a subset of AEs that include both clearly autoimmune diseases and also other inflammatory and/or neurologic disorders that may or may not have an autoimmune etiology. These events are of special interest since they could potentially be caused by immune responses to the IP. The investigator/designee should report such adverse events within the same time limits (following confirmation of an AE as a pIMD; see last paragraph of this section below), and using the same CRF pages, as utilized for SAEs. The investigator or his/her designee will evaluate the occurrence of pIMDs at every visit/contact during the study. IAVI will also expect investigators/designee to provide additional information about pIMD events. AEs to be reported and documented as pIMDs include:

Neuroinflammatory disorders: optic neuritis, cranial nerve disorders (including Bell's palsy), multiple sclerosis, demyelinating disease, transverse myelitis, Guillain-Barré syndrome, myasthenia gravis, encephalitis, neuritis.

Musculoskeletal disorders: systemic lupus erythematosus, cutaneous lupus, Sjögren's syndrome, scleroderma, dermatomyositis, polymyositis, myopathy, rheumatoid arthritis and juvenile rheumatoid arthritis, polymyalgia rheumatica or temporal arteritis, reactive arthritis, psoriatic arthropathy, ankylosing spondylitis, undifferentiated spondyloarthropathy.

Gastrointestinal disorders: Crohn's disease, ulcerative colitis or proctitis, celiac disease.

Metabolic diseases: autoimmune thyroiditis, Grave's or Basedow's disease, Hashimoto thyroiditis, insulin-dependent diabetes mellitus (IDDM), Addison's disease.

Skin disorders: psoriasis, vitiligo, Raynaud's phenomenon, erythema nodosum, autoimmune bullous skin diseases.

Others: autoimmune hemolytic anemia, thrombocytopenia, antiphospholipid syndrome, *vasculitis, pernicious anemia, autoimmune hepatitis, primary biliary cirrhosis, primary sclerosing cholangitis, autoimmune glomerulonephritis, autoimmune uveitis, autoimmune myocarditis/cardiomyopathy, sarcoidosis, Stevens-Johnson syndrome, Behçet's syndrome.

Infusion site reactions: Grade 3 or 4 infusion site reactions lasting more than 2 days.

*Vasculitis: Vasculitis, Diffuse vasculitis, leucocytoclastic vasculitis, polyarteritis nodosa, microscopic polyangiitis, Wegener's granulomatosis, anti-neutrophil cytoplasmic antibody positive vasculitis, Henoch-schönlein purpura, allergic granulomatous angiitis (Churg-Strauss disease), Kawasaki disease, Takayasu's arteritis, temporal arteritis (giant cell arteritis), renal vasculitis.

Medical judgement should be exercised in deciding whether other disorders/diseases have an autoimmune origin and should also be reported as described above, and this judgement is the investigator's prerogative. Whenever sufficient data exist to substantiate any of the diagnoses in the above list, the event must be reported as a pIMD. While the intent of pIMD reporting is to be inclusive, isolated nonspecific

symptoms, which might (or might not) represent the above diagnoses, should be captured as AEs but not reported as pIMDs until the diagnosis can be defended.

10.6 Clinical Management of Adverse Events

Adverse events (AEs) will be managed by the clinical study team who will assess, provide first line of care as appropriate and refer to health care and treatment facilities as warranted. If any treatment/medical care is required as a result of the harm caused by the IP or study procedures, this will be provided free of charge.

If a participant has an AE and/or abnormal laboratory value that is known at the time of IV infusion of IP, the specifications of Section 12.0 will be followed.

Participants will be followed until the AE resolves or stabilizes or up to the end of the study, whichever comes last. If at the end of the study, an AE (including clinically significant laboratory abnormality) that is considered possibly, probably or definitely related to the IP is unresolved, follow-up will continue until resolution if possible and/or the participant will be referred.

If a participant experiences a significant decrease in CD4 cell count (e.g. – 20% of baseline, or decline to <200 cells/µL) during the course of the trial, CD4+ will be monitored closely until their CD4 count returns to baseline or until the participant initiates ART. Participants whose CD4 cell counts decrease to <200 cells/µL will be promptly informed and will be referred to their primary HIV care provider. Appropriate prophylaxis against opportunistic infections will be instituted according to accepted U.S. HIV treatment guidelines.

10.7 Pregnancy

Although not considered an AE, if a female participant becomes pregnant during the study, it is the responsibility of the Principal Investigator or designee to report the pregnancy promptly to IAVI using the designated forms. The participant will be followed for safety until the end of pregnancy or study completion, whichever occurs last. If possible, approximately 2–4 weeks after delivery, the baby will be examined by a physician to assess its health status and the results will be reported to the Sponsor. The baby will be examined again by a Physician around age 1, and the results will be reported to the Sponsor.

Complications of pregnancy that meet criteria for SAEs, specified in Section 10.4 of this Protocol (e.g., hospitalization for eclampsia, spontaneous abortion, etc.) should be reported as SAEs.

10.8 Intercurrent HIV Infection (Group 1)

HIV infection cannot be directly caused by the IP. If a participant acquires HIV through exposure in the community, at any time after the IV infusion of IP, the participant should be offered referral to appropriate care and treatment facilities. The participant will continue to be followed in the study for safety assessments.

Intercurrent HIV infection in study participants, although not considered an SAE, must be reported promptly to IAVI using the designated forms. However, medical conditions associated with the HIV infection that meet criteria for being serious specified in the

Section 10.4 of this Protocol (e.g., sepsis, *Pneumocystis jiroveci [carinii] pneumonia*, etc.) should be reported as SAEs using the SAE Report Form.

11.0 MANAGEMENT OF HIV ISSUES DURING AND FOLLOWING STUDY

11.1 HIV Testing – Group 1

Group 1 participants will be tested for HIV antibodies as indicated in the Schedule of Procedures (Appendix A) or as needed, if medical or social circumstances arise. All participants will receive HIV risk reduction counselling and pre- and post-HIV-test counselling, as specified in Section 11.3.1, Counselling (Group 1).

It is possible that PGT121 or an immune response to PGT121 mAb could cause a positive result on a diagnostic HIV antibody test. An IP recipient who falsely tests HIV positive with a diagnostic HIV antibody test at the end of the study will be informed of his/her positive test result and offered continuing follow-up until the test becomes negative.

If a participant acquires HIV through exposure in the community, at any time after the administration of IP, the participant will be offered referral to appropriate care and treatment facilities. The participant will continue to be followed in the study for safety assessments.

Should a participant require HIV testing outside of the study for personal reasons, it is recommended that the participant contact the study staff first. HIV testing can be done at the study site and then processed at an independent laboratory as above. Written evidence of HIV status (HIV-infected or HIV-uninfected) will be provided upon request.

11.2 Social Discrimination as a Result of IP-related antibodies

In order to minimize the possibility of social discrimination in participants (if any) who test positive on a diagnostic HIV antibody test due to IP-related antibodies, appropriate diagnostic HIV testing and certification will be provided both during and after the study as needed.

11.3 HIV infection – Group 1

Group 1 participants who are diagnosed with HIV infection at screening or during the study (intercurrent HIV-infection) will be provided the following:

11.3.1 Counselling

The participant will be counselled by the study investigators or designated counsellors. The counselling process will assist the participant with the following issues:

- Psychological and social implications of HIV infection
- Who to inform and what to say
- Implications for sexual partners
- Implications for child-bearing
- Avoidance of transmission to others in future

- Mandatory reporting to the state, in some instances

11.3.2 Referral for Support/Care

Participants will be referred to a participant support center or institution of his/her choice for a full discussion of the clinical aspects of HIV infection. Referral will be made to a designated physician or center

12.0 WITHDRAWAL FROM STUDY

12.1 Deferral of IV infusion of IP

An IV infusion of IP may be temporarily deferred if the participant is clinically ill at the time of the administration of IP visit and/or presents with fever (> 100.4 F) at the time of the administration of IP. A participant must be clinically well and afebrile for a minimum of a 24-hour consecutive period prior to administration of IP.

Any planned or unplanned deferral of infusion of IP will be discussed with the Sponsor. Participants will be deferred from infusion of IP for any of the following reasons:

1. Pregnancy
2. A disease or condition or adverse event that may develop, regardless of relationship to Investigational Product, if the Principal Investigator or designee is of the opinion that administration of IP will jeopardize the safety of the participant
3. Participant’s request to defer infusion

The following events require resolution and/or review of clinical history by the Principal Investigator or designee and consultation with the Medical Monitor, prior to administration of IP:

- Any abnormal laboratory value, as outlined in section 5.7, Exclusion Criteria, Hematology, Chemistry, Urinalysis that is known at the time of infusion and have not resolved. Abnormal results should be confirmed on the original sample and/or repeated at least once to confirm abnormal values.
- Receipt of inactivated/killed/subunit vaccines (non-HIV) or immunoglobulin within the previous 14 days. Receipt of live attenuated vaccines within the previous 60 days.
- Participating in another clinical study of an Investigational Product

12.2 Withdrawal from the Study (Early Termination)

Participants may be withdrawn from the study permanently for the following reasons:

1. Participants may withdraw from the study at any time if they wish, for any reason
2. The Principal Investigator or designee has reason to believe that the participant is not complying with the protocol
3. If the Sponsor decides to terminate or suspend the study

If a participant withdraws or is withdrawn from the study, all termination visit procedures will be performed according to the Schedule of Procedures (Appendices A, B and C) where possible. Every effort will be made to determine and document the reason for withdrawal.

13.0 DATA HANDLING

13.1 Data Collection and Record Keeping at the Study Site

Data Collection: All study data will be collected by the clinical study staff using designated source documents and entered onto the appropriate electronic CRFs (eCRFs). Access to eCRFs will be provided via an electronic data entry system hosted by the Data Coordination Center. All study data must be verifiable to the source documentation. A file will be held for each participant at the clinic(s) containing all the source documents. Source documentation will be available for review to ensure that the collected data are consistent with the CRFs.

All CRFs and laboratory reports will be reviewed by the clinical team, who will ensure that they are accurate and complete.

Source documents and other supporting documents will be kept in a secure location. Standard GCP practices will be followed to ensure accurate, reliable and consistent data collection.

Source documents include but are not limited to:

- Signed Informed Consent Documents
- Progress notes
- Data collection forms
- Documentation of any existing conditions or past conditions relevant to eligibility
- Printed laboratory results
- Print out of the IDES generated enrollment confirmation
- All Adverse Events
- Concomitant medications
- Local and systemic reactogenicity events

13.3 Data Entry at the Study Site

The data collected at the site will be recorded onto the eCRFs by the study staff and entered into a database. To provide for real time assessment of safety, data should be entered as soon as reasonably feasible after a visit occurs.

13.4 Data Analysis

The Sponsor, PIs and Product Developers will agree on how data will be analyzed and presented prior to unblinding of the study.

The DCC will conduct the data analysis and will provide interim safety and final study reports for the Sponsor, Principal Investigators, the PSRT and SMC and the regulatory authorities, as appropriate.

14.0 STATISTICAL CONSIDERATIONS

14.1 Safety and Tolerability Analysis

14.1.1 Sample Size

The sample size for safety and tolerability analysis will be 30-48 participants according to the dose escalation design used to characterize the safety profile of one IV infusion of PGT121 mAb, at one of three dose levels, to HIV-uninfected and HIV-infected individuals (groups 1 and 2).

14.1.2 Null Hypothesis

As this is an exploratory proof of concept trial and analysis will be descriptive, no formal null hypothesis will be tested.

14.1.3 Statistical Power and Analysis and Dose Escalation Rules

The frequency of moderate or greater local and systemic reactogenicity events will be determined and compared between groups.

The frequency of SAEs judged possibly, probably or related to the IP will be determined.

All AEs will be analyzed and, grouped by seriousness, severity and relationship to the Investigational Product (as judged by the investigator).

For life-threatening adverse events related to Investigational Product: if none of the 12 (max 18) participants receiving Investigational Products experience such reactions, then the 95 % upper confidence bound for the rate of these adverse events in the population is 26.5% (or 18.5% if n=18).

All AEs will be analysed and grouped by seriousness, severity and relationship to the IP (as judged by the investigator).

For life-threatening adverse events related to IP: if none of the 12 (max 18) participants in either Group 1 or Group 2 who receive the IP experience such reactions then the 95% upper confidence bound for the rate of these adverse events in the population is 26.5% (or 18.5% if n=18).

An interim analysis of group data will be carried out according to the study schema (Table 5.3.1) without unblinding the study to investigators or participants. At the end of the study, a full analysis will be prepared.

Based on previous experience with IAVI Phase 1 IP studies, it is expected that the amount of missing, unused or spurious data will be insignificant. Unused and spurious data will be listed separately and excluded from the statistical analysis. Missing data will be excluded from the statistical analysis.

14.2 Pharmacokinetic Analysis

14.2.1 Sample Size

The sample size for pharmacokinetic analysis will be 4 per dose subgroup, sufficient to provide sufficient information for the planned analyses.

14.2.2 Null Hypothesis

As this is an exploratory proof of concept trial and analysis will be descriptive, no formal null hypothesis will be tested.

14.2.3 Statistical Power and Analysis

Disposition of PGT121 mAb will be evaluated in this study. Based on the PK profile of other human monoclonal antibodies, it is expected that the half-life of PGT121 mAb will be 14 to 21 days. Previously published data indicates that the pharmacokinetics of PGT121 and 3BNC117 are fairly similar across a non-human primate cohort and within the same non-human primate (clearance of 3BNC117 appears to be marginally faster than that for PGT121).

Commonly reported PK parameters will be calculated using standard non-compartmental slope/height/area/moment (SHAM) analysis methods. Summary descriptive results of PK parameters, including AUC, Cmax, T1/2, and clearance results will be reported by dose cohort. Dose normalized plots of PK parameters will be presented. Correlation between PK and reported safety and pharmacodynamic outcomes will also be explored parameters in order to examine exposure-effect relationships.

A more powerful exploratory analysis to quantitatively determine the dose, participant and disease impact on PGT121 mAb pharmacokinetics, and correlate exposure with response, while correctly accounting for variance based on population intrinsic factors such as weight and gender will be performed. Using the proposed population analysis approach we will be able to simultaneously examine the magnitude and the rate of change to PGT121 disposition driven by HIV-1 RNA levels and/or ART, and also examine the magnitude and the rate of decline in log copies/ml of HIV-1 RNA plasma levels from baseline.

The frequency and levels of anti-PGT121 antibodies will be calculated and tabulated.

14.3 Virologic Analysis for Dose De-escalation in Groups 3A-C

14.3.1 Sample Size

The sample size for virologic analysis in Groups 3A-C will be 24-36 participants according to the dose de-escalation design described below.

14.3.2 Null Hypothesis

The null hypothesis is that the HIV RNA viral load difference-from-baseline is greater than -0.9 logs.

14.3.3 Statistical Power and Analysis

The virologic analysis described in this section relates to Groups 3A-C of the study design, in which dose de-escalation is performed in an adaptive study design in HIV-infected participants off ART with plasma HIV RNA levels of 2 x 10^3 – 10^5 copies/ml. This section assumes that Part 1 of the study has successfully demonstrated that there is a safe dose level of the IP such that the study is carried forward into Part 2.

The primary efficacy outcome for this analysis is defined as change in log10 viral load between Day 0 (day of infusion) and Day 7. The minimum clinically significant value for this outcome is defined as a difference of -0.9 log10.

The study plan for Groups 3A-C is designed so that the IP dose level may be de-escalated in a stepwise manner from the highest dose to the lowest dose, until a given dose level cannot be concluded to be efficacious. If any given dose level is proven to be efficacious at an interim analysis, enrolment for that dose level may cease, and the next lowest dose group may be enrolled. In the unlikely event that IP administration leads to increased viral load, this may be detected by this design. No placebo participants are enrolled as part of this design.

This design represents a dose de-escalation beginning at 30 mg/kg. The actual starting dose will be the MTD as determined by the SMC based on data from Part 1, therefore the starting dose may be 30mg/kg, 10 mg/kg or 3 mg/kg. If the starting dose is 30 mg/kg, then de-escalation will begin with Group 3A. If the starting dose is 10 mg/kg, then de-escalation will begin with Group 3B. If the starting dose is 3 mg/kg, then only Group 3C will be enrolled.

Assuming the starting dose is 30 mg/kg, an interim analysis of Group 3A will be performed after all 6 participants have reached 7 days following IP administration. The following scenarios may then occur, depending on the data:

- If the response in the first 6 participants results in an HIV RNA viral load difference-from-baseline significantly greater than -0.9 log10, the IP will be determined to be effective at 30 mg/kg, enrollment into Group 3A will cease, and enrollment into Group 3B will begin.
- If the mean response in the first 6 participants is a decrease smaller than -0.9 log10 HIV RNA, then an additional 3 participants will be enrolled into Group 3A. After the additional 3 participants have reached 7 days following IP administration, an analysis of Group 3A (N=9) will be performed:
    - If the response in all Group 3A results in an HIV RNA viral load difference-from-baseline significantly greater than -0.9 log10, the IP will be determined to be effective at 30 mg/kg, and enrollment into Group 3B will begin.
    - If the response in all 9 participants results in an HIV RNA viral load difference-from-baseline not significantly greater than -0.9 log10, then the IP will be determined to be ineffective at 30 mg/kg and Groups 3B and 3C will not be enrolled. In this scenario, no dose of IP will be determined to be effective.

If 30 mg/kg is determined to be an effective dose, then Group 3B will be enrolled at 10 mg/kg. An interim analysis of Group 3B will be performed after 8 participants have reached 7 days following IP administration. The following scenarios may then occur, depending on the data:

- If the response in the first 8 participants results in an HIV RNA viral load difference-from-baseline significantly greater than -0.9 log10, the IP will be determined to be effective at 10 mg/kg, enrollment into Group 3B will cease, and enrollment into Group 3C will begin.
- If the mean response in the first 8 participants is a decrease smaller than -0.9 log10 HIV RNA, then an additional 4 participants will be enrolled into Group 3B. After the additional 4 participants have reached 7 days following IP administration, an analysis of Group 3B (N=12) will be performed:
    - If the response in all Group 3B participants results in an HIV RNA viral load difference-from-baseline significantly greater than -0.9 log10, the IP will be determined to be effective at 10 mg/kg, and enrollment into Group 3C will begin.
    - If the response in all 12 participants results in an HIV RNA viral load difference-from-baseline not significantly greater than -0.9 log10, then the IP will be determined to be ineffective at 10 mg/kg, and Group 3C will not be enrolled. In this scenario, the minimum effective dose will be determined to be 30 mg/kg.

If 10 mg/kg is determined to be an effective dose, then Group 3C will be enrolled at 3 mg/kg. An interim analysis of Group 3C will be performed after 10 participants have reached 7 days following IP administration. The following scenarios may then occur, depending on the data:

- If the response in the first 10 participants results in an HIV RNA viral load difference-from-baseline significantly greater than -0.9 log10, the IP will be determined to be effective at 3 mg/kg and enrollment into Group 3C will cease. In this scenario, the minimum effective dose of the IP will be determined to be 3 mg/kg.
- If the mean response in the first 10 participants is a decrease smaller than -0.9 log10 HIV RNA, then an additional 5 participants will be enrolled into Group 3C. After the additional 5 participants have reached 7 days following IP administration, an analysis of Group 3C (N=15) will be performed:
    - If the response in all Group 3C participants results in an HIV RNA viral load difference-from-baseline not significantly greater than -0.9 log10, the minimum effective dose will be determined to be 3 mg/kg.
    - If the response in all 15 participants results in an HIV RNA viral load difference-from-baseline not significantly greater than -0.9 log10, then the IP will be determined to be ineffective at 3 mg/kg. In this scenario, the minimum effective dose will be determined to be 10 mg/kg.

For the analysis of sample size and power, log10 viral load differences from baseline for each participant were simulated from a normal distribution, with a standard deviation of 0.5. This value was chosen by examining a study of the antiretroviral drug raltegravir, which demonstrated a mean estimated standard deviation of the change of baseline of 0.47[18]. This is a conservative estimate, as the variability of viral loads near the lower range might be expected to also be lower.

The statistical test performed will be the Signed-ranktest, which will incorporate the "shift" parameter of -0.9 log10 (the minimum clinically significant difference selected for this study). An evaluation of potential harm (increased viral load) will also be performed with the Signed ranktest; this test will examine the null hypothesis of no change in viral load (a shift of 0.0 log10 following IP administration) against the one-sided alternative hypothesis that the viral load is increased following IP administration. Each efficacy test will be performed at the level $\alpha = 0.05$. Each test for harm will be performed at level $2\alpha = 0.10$, in order to provide additional sensitivity to detect potential harm.

14.4 Analysis of Antiviral Activity and Dose De-escalation in Subgroups 3D-F

14.4.1 Sample Size

The sample size for antiviral activity will be 3-9 participants, depending on the MTD.

14.4.2 Null Hypothesis

As this is an exploratory proof of concept trial and analysis will be descriptive in this population, no formal null hypothesis will be tested.

14.4.3 Statistical Power and Analysis

No efficacy endpoints will be tested in Groups 3D-F as participants are HIV-infected with low viral loads at baseline (10^2 – $2x10^3$ copies/ml). Immunologic and virologic endpoints will be determined as described in Section 4.1. As soon as subgroup 3D has enrolled 3 participants, enrollment into subgroup 3E will be initiated. The same rule will apply for progression from subgroup 3E to 3F. In the event that dose de-escalation in subgroups 3A-C is stopped due to failure to meet virologic endpoints, dose de-escalation in subgroups 3D-F will continue.

14.5 Secondary and Exploratory Immunologic and Virologic Analyses

14.5.1 Sample Size

The sample size for secondary and exploratory immunologic and virologic analysis will be 63-93 participants.

14.5.2 Null Hypothesis

No formal hypothesis on immunologic or virologic responses will be tested, with the exception of the change in viral load described in Section 14.3.

14.5.3 Statistical Power and Analysis

Descriptive statistics (actual values and changes from reference) will be calculated for continuous immunologic and virologic parameters at all time points. Graphical representations of changes in parameters will be made as applicable. Differences between groups at a specific time point will be tested for exploratory purposes by a 2-sample t-test if the data appear to be normally distributed. If not, the non-parametric Wilcoxon rank sum test will be used. If portions of the measurements are censored

below the assay quantification limit, the Gehan-Wilcoxon test will be employed. All statistical tests will be two-sided and will be considered statistically significant if $p < 0.05$.

Frequency tabulations will be calculated for discrete (qualitative) immunologic and virologic parameters at all time points. Significant differences between groups will be determined by a 2-sided Fisher's exact test.

Interim immunologic and virologic analyses of grouped data may be performed without unblinding the study to investigators or participants.

15.0 QUALITY CONTROL AND QUALITY ASSURANCE

To ensure the quality and reliability of the data collected and generated and the ethical conduct of this study, a Study Operations Manual (SOM) will be developed. All deviations will be reported and investigated. The SOM describes reporting and deviation documentation requirements and procedures.

Regular monitoring will be performed according to ICH-GCP as indicated in Section 17.5.

An independent audit of the study and study sites may be performed by the Sponsor or designee to establish the status of applicable quality systems. Inspection by regulatory authorities may also occur.

By signing the protocol, the Principal Investigators agree to facilitate study related monitoring, audits, IRB/IEC review and regulatory inspection(s) and direct access to source documents. Such information will be treated as strictly confidential and under no circumstances be made publicly available.

16.0 DATA AND BIOLOGICAL MATERIAL

All data and biological material collected through the study shall be managed in accordance with the Clinical Trial Agreement (CTA). Distribution and use of these data will be conducted by agreement of all parties.

The computerized raw data generated will be held by the DCC on behalf of the Sponsor. The study sites will also hold the final data files and tables generated for the purpose of analysis.

17.0 ADMINISTRATIVE STRUCTURE

The Principal Investigator will be responsible for all aspects of the study at the study site.

17.1 Protocol Safety Review Team

A PSRT will be formed to monitor the clinical safety data. During the administration of IP phase of the trial, the PSRT will review the clinical safety data on a weekly basis via electronic distribution of reports. An ad hoc PSRT review meeting will occur if any of the members of the PSRT requests a special review to discuss a specific safety issue or as specified in the Study Operations Manual. After the administration of IP phase the PSRT will review the clinical safety data at least monthly.

The PSRT will consist of the IAVI Medical Monitor(s), and the PI or designee from each clinical team. The study chair or an IAVI Medical Monitor may be the PSRT chair. *Ex officio* members will include the IAVI Chief Medical Officer and an unblinded IAVI Medical Monitor.

Additional PSRT participants may include the following, as needed:

- Co-investigators and trial site senior clinical research nursing staff
- Laboratory directors
- Data management, study statistician and regulatory staff

The PSRT membership and procedures are detailed in the PSRT charter.

17.2 Safety Monitoring Committee (SMC)

The SMC will consist of independent clinicians/scientists/statisticians/ethicists who are not involved in the study. Investigators responsible for the clinical care of participants or representative of the Sponsor may not be a member of the SMC. Details of membership, chair and co-chair and responsibilities are outlined in the SMC charter.

Principal Investigator(s) or designee and/or a Sponsor representative may be asked to join an open session of the SMC meeting to provide information on study conduct, present data or to respond to questions.

Safety data will be reviewed by the SMC at pre-specified time points and at an ad-hoc basis.

17.2.1 Content of Interim Safety Review

The SMC will be asked to review the following blinded data:

- Summary of reactogenicity (i.e., solicited adverse events)
- All adverse events judged by the Principal Investigator or designee to be possibly, probably or definitely related to IP
- All laboratory results confirmed on retest and judged by the Principal Investigator or designee to be clinically significant
- All SAEs

An unblinded presentation of all above noted events may also be made available for the SMC for their review if required by any member of the SMC.

17.2.2 SMC Review of Group 1 and 2 data prior to starting Group 3

Following IV infusion of IP of the last participant in Groups 1 and 2, the Safety Monitoring Committee (SMC) will review safety data through the day 14 post-IV infusion visit for all participants to confirm MTD in each group, and determine whether, and at what dose level, Group 3 can initiate enrollment.

17.3 Criteria for Pausing the Study

Enrollment and administration of IP will be stopped and a safety review conducted by the SMC for any of the following criteria:

1. One or more participants experience an SAE that is judged possibly, probably or definitely related to IP.

2. There is a participant death, regardless of relationship to the IP.
3. Two or more participants experience Grade 3 adverse events in the same category System Organ Class that are considered to be possibly, probably or definitely related to IP or
4. Any grade 4 adverse event that is considered to be possibly, probably or definitely related to IP.

Table 17.3-1: AE notification and safety pause/AE review rules

Event and relationship to study product	Severity	Occurrence	Site PI action	PSRT or SMC action
SAE, possibly, probably or definitely related	Any	Any	Phone, email or fax forms to sponsor within 24 hours	Study pause within 24 hours, refer to SMC for review
SAE, probably not or not related	Death	Any	Phone, email or fax forms to sponsor within 24 hours	Study pause within 24 hours, refer to SMC for review
AE, possibly, probably or definitely related	Grade 4	Any	Phone, email or fax notification to sponsor within 24 hours	Study pause within 24 hours, refer to SMC for review
AE¶, possibly, probably or definitely related	Grade 3*	First	Phone, email or fax notification to sponsor within 24 hours	PSRT review within 2 business days to consider pause
AE¶, possibly, probably or definitely related	Grade 3*	Second‡	Phone, email or fax notification to sponsor within 24 hours	Study pause within 24 hours, refer to SMC for review

¶Does not include the following reactogenicity symptoms (fever, fatigue/malaise, myalgia, arthralgia, chills, headache, nausea, vomiting).

*If no evidence of disease is present other than an abnormal laboratory value, the test must be repeated with a new blood sample at least one time within 72 hours after the investigator becoming aware of the abnormal laboratory value. When signs and symptoms are present, repeat test will not be needed.

‡PSRT will determine whether the reported related AE (Grade 3) is a second occurrence of a previously reported AE (Grade 3).

The Sponsor will request a review by the SMC, (or the SMC chair if other SMC members cannot be convened), to be held within 2 business days of the Sponsor learning of the event. The individual participant(s)/or study may be unblinded at the discretion of the SMC.

Following this review, the SMC will make a recommendation regarding the continuation or suspension of the administration of the IP or the trial and communicate this decision immediately to the Sponsor. The Sponsor then will inform the Principal Investigators without delay.

Additional *ad hoc* review may be specifically requested by the Sponsor, the Principal Investigator(s) or by the SMC.

17.4 Study Supervision

The SMC, the IAVI Chief Medical Officer (CMO) and the IAVI Medical Monitor(s) have access to progress report(s) of this study. Close cooperation will be necessary to track study progress, respond to queries about proper study implementation and management, address issues in a timely manner, and assure consistent documentation, and share information effectively. Rates of accrual, retention, and other parameters relevant to the site's performance will be regularly and closely monitored by the study team.

17.5 Study Monitoring

On-and/or off-site monitoring will ensure that the study is conducted in compliance with human subjects' protection and other research regulations and guidelines, recorded and reported in accordance with the protocol, is consistent with SOPs, GCP, applicable regulatory requirements and locally accepted practices. The monitor will confirm the quality and accuracy of data at the site by validation of CRFs against the source documents, such as clinical records. The investigators, as well as participants through consenting to the study, agree that the monitor may inspect study facilities and source records (e.g., informed consent forms, clinic and laboratory records, other source documents), as well as observe the performance of study procedures (in accordance with site IRB requirements). Such information will be treated as strictly confidential and will under no circumstances be made publicly available.

The monitoring will adhere to GCP guidelines. The Principal Investigator will permit inspection of the facilities and all study-related documentation by authorized representatives of IAVI, and Government and Regulatory Authorities responsible for this study.

17.6 Investigator's Records

Study records include administrative documentation—e.g., reports and correspondence relating to the study—as well as documentation related to each participant screened and/or enrolled in the study—including informed consent forms, case report forms, and all other source documents. The investigator will maintain and store, in a secure manner, complete, accurate, and current study records for a minimum of 2 years after marketing application approval or the study is discontinued and applicable national and local health authorities are notified. IAVI will notify the Principal Investigator of these events.

18.0 INDEMNITY

The Sponsor and Institution are responsible to have appropriate liability insurance. For research-related injuries and/or medical problems determined to result from receiving the IP, treatment including necessary emergency treatment and proper follow-up care will be made available to the participant free of charge at the expense of the Sponsor.

19.0 PUBLICATION

A primary manuscript describing safety, anti-viral effect and immune responses in this trial will be prepared promptly after the data analysis is available.

Authors will be representatives of each trial site, the data management and statistical analysis center, the laboratories, the product developer and the sponsor, participant to the generally accepted criteria of contributions to the design and conduct of the study, the analysis of data and writing of the manuscript. Precedence will be given to authors from the site enrolling the greatest number of participants. Manuscripts will be reviewed by representatives of each participating group as specified in the CTA**.**

20.0 ETHICAL CONSIDERATIONS

The Principal Investigator will ensure that the study is conducted in compliance with the protocol, SOPs in accordance with guidelines formulated by the ICH for GCP in clinical studies, the ethical principles that have their origins in the Declaration of Helsinki and applicable local standards and regulatory requirements.

APPENDIX A: SCHEDULE OF PROCEDURES – GROUP 1 (A, B, C)

**Study Month**		**0**							**1**		**2**		**3**	**4**	**5**	**6**
**Study Week**		**0**				**1**	**2**	**3**	**4**	**6**	**8**	**10**	**12**	**16**	**20**	**24**
**Study Day**	**Scr**	**0**	**1**	**2**	**3**	**7**	**14**	**21**	**28**	**42**	**56**	**70**	**84**	**112**	**140**	**168/ET[4]**
**Visit Windows (Days)**	**-56**	**0**	**0**	**0**	**0**	**± 1**	**± 2**	**± 2**	**± 2**	**± 3**	**± 3**	**±3**	**±7**	**±7**	**±7**	**±7**
**INVESTIGATIONAL PRODUCT**																
Investigational Product /Placebo		X														
**CONSENT/ASSESSMENTS/COUNSELLING**																
Informed Consent	X															
Assessment of Understanding	X															
HIV Risk Assessment	X															X
HIV Risk Reduction Counselling	X	X							X		X		X	X	X	X
HIV-test Counselling	X	X							X							X
Family Planning Counselling	X	X														
Social Impact Assessment																X
**CLINICAL SAFETY ASSESSMENTS**																
Comprehensive Medical History	X															
Interim Medical History		X	X	X	X	X	X	X	X	X	X					
Concomitant Medications	X	X	X	X	X	X	X	X	X	X	X					
General Physical Exam	X															X
Directed Physical Exam		X	X	X	X	X	X	X	X	X	X	X	X	X	X	
Weight	X	X														X
Height	X															
Vital Signs	X	X[1]	X	X	X	X	X	X	X	X	X	X	X	X	X	X
Local & Systemic Reactogenicity		X[1]	X	X	X											
Adverse Events		X	X	X	X	X	X	X	X	X	X					
Serious Adverse Events and pIMD	X	X	X	X	X	X	X	X	X	X	X	X	X	X	X	X

Study Month		0							1		2		3	4	5	6
**Study Week**		**0**				**1**	**2**	**3**	**4**	**6**	**8**	**10**	**12**	**16**	**20**	**24**
**Study Day**	**Scr**	**0**	**1**	**2**	**3**	**7**	**14**	**21**	**28**	**42**	**56**	**70**	**84**	**112**	**140**	**168/ET[4]**
**Visit Windows (Days)**	**-56**	**0**	**0**	**0**	**0**	**± 1**	**± 2**	**± 2**	**± 2**	**± 3**	**± 3**	**±3**	**±7**	**±7**	**±7**	**±7**
**CLINICAL LABORATORY TESTS**																
Hematology and Coagulation	X	X[6]	X		X	X	X		X		X		X	X	X	X
Clinical Chemistry	X	X[6]	X		X	X	X		X		X		X	X	X	X
Urine Dipstick	X	X[6]	X		X	X	X		X		X		X	X	X	X
Urine Pregnancy test	X	X[6]							X		X		X			X
Active Syphilis	X															
Hepatitis B	X															
Hepatitis C	X															
HIV screen (4th generation Ag/Ab test)	X															
Blinded HIV diagnostic testing[2]		X[6]							X							X
**RESEARCH LABORATORY TESTS**	-	-	-	-	-	-	-	-	-	-	-	-	-	-	-	-
Anti PGT121 Antibodies (ADA)		X[6]							X		X		X			X
Humoral Assays[2]		X[6]			X	X	X		X		X		X			X
Cellular Assays[2]		X[6]					X		X		X		X			X
HLA typing		X[6]														
**PHARMACOKINETICS PGT121 ELISA**		X[3]	X	X	X	X	X	X	X	X	X	X	X	X	X	X
**MUCOSAL SAMPLING**		X[5]	X			X	X									
**PLASMA/SERUM STORAGE**		X	X	X	X	X	X	X	X		X		X			X
**PBMCs STORAGE**		X	X	X	X	X		X			X		X			X

1. At baseline, approximately 30 minutes after IP administration start, and at hours 1 through 12 after IV infusion start. Local and systemic reactogenicity will be assessed by clinic staff at visits on study days 1, 2 and 3. Local and Systemic reactogenicity will also be assessed by the participant using the Memory Aid on study days 1, 2, and 3
2. See Laboratory Analytical Plan for details
3. Day 0 PK draws done immediately before IP administration, at the end of the IV infusion of IP, and 30 minutes and 3 hours post end of the IP administration. Additional PK draws on day 0 are done 6, 9 and 12 hours after the start of the IV infusion of IP. See SOM for details
4. Early Termination (ET): Procedures to be performed at ET are the same as last visit procedures
5. Cervico-vaginal and/or rectal mucosal sampling (optional) on Day 0 must be done prior to IV infusion of IP. See SOM for details
6. Day 0 sample collections for laboratory tests must be done pre-infusion.

APPENDIX B: SCHEDULE OF PROCEDURES – GROUP 2 (A, B, C)

**Study Month**		**0**							**1**		**2**		**3**	**4**	**5**	**6**
**Study Week**		**0**				**1**	**2**	**3**	**4**	**6**	**8**	**10**	**12**	**16**	**20**	**24**
**Study Day**	**Scr**	**0**	**1**	**2**	**3**	**7**	**14**	**21**	**28**	**42**	**56**	**70**	**84**	**112**	**140**	**168/ET[5]**
**Visit Windows (Days)**	**-56**	**0**	**0**	**0**	**0**	**± 1**	**± 2**	**± 2**	**± 2**	**± 3**	**± 3**	**±3**	**±7**	**±7**	**±7**	**±7**
**INVESTIGATIONAL PRODUCT**																
Investigational Product /Placebo		**X**														
**CONSENT/ASSESSMENTS/COUNSELLING**																
Informed Consent	**X**															
Assessment of Understanding	**X**															
HIV Risk Reduction Counselling[1]	**X**	**X**							**X**		**X**		**X**	**X**	**X**	**X**
Family Planning Counselling	**X**	**X**														
Social Impact Assessment																**X**
**CLINICAL SAFETY ASSESSMENTS**																
Comprehensive Medical History	**X**															
Interim Medical History		**X**	**X**	**X**	**X**	**X**	**X**	**X**	**X**	**X**	**X**					
Concomitant Medications	**X**	**X**	**X**	**X**	**X**	**X**	**X**	**X**	**X**	**X**	**X**					
General Physical Exam	**X**															**X**
Directed Physical Exam		**X**	**X**	**X**	**X**	**X**	**X**	**X**	**X**	**X**	**X**	**X**	**X**	**X**	**X**	
Weight	**X**	**X**														**X**
Height	**X**															
Vital Signs	**X**	**X[2]**	**X**	**X**	**X**	**X**	**X**	**X**	**X**	**X**	**X**	**X**	**X**	**X**	**X**	**X**
Local & Systemic Reactogenicity		**X[2]**	**X**	**X**	**X**											
Adverse Events		**X**	**X**	**X**	**X**	**X**	**X**	**X**	**X**	**X**	**X**					
Serious Adverse Events and pIMD	**X**	**X**	**X**	**X**	**X**	**X**	**X**	**X**	**X**	**X**	**X**	**X**	**X**	**X**	**X**	**X**
**CLINICAL LABORATORY TESTS**																
Hematology and Coagulation	**X**	**X[8]**	**X**		**X**	**X**	**X**		**X**		**X**		**X**	**X**	**X**	**X**

Study Month		0							1		2		3	4	5	6
**Study Week**		**0**				**1**	**2**	**3**	**4**	**6**	**8**	**10**	**12**	**16**	**20**	**24**
**Study Day**	**Scr**	**0**	**1**	**2**	**3**	**7**	**14**	**21**	**28**	**42**	**56**	**70**	**84**	**112**	**140**	**168/ET[5]**
**Visit Windows (Days)**	**-56**	**0**	**0**	**0**	**0**	**± 1**	**± 2**	**± 2**	**± 2**	**± 3**	**± 3**	**±3**	**±7**	**±7**	**±7**	**±7**
CD4	X	X[8]				X	X		X		X					X
Clinical Chemistry	X	X[8]	X		X	X	X		X		X		X	X	X	X
Urine Dipstick	X	X[8]	X		X	X	X		X		X		X	X	X	X
Urine Pregnancy test	X	X[8]							X		X		X			X
Active Syphilis	X															
Hepatitis B	X															
Hepatitis C	X															
HIV 4th generation Ag/Ab test	X															
HIV Viral Load	X	X[8]	X	X	X	X	X	X	X	X	X	X	X	X	X	X
**RESEARCH LABORATORY TESTS**	-	-	-	-	-	-	-	-	-	-	-	-	-	-	-	-
Anti PGT121 Antibodies (ADA)		X[8]							X		X		X			X
HIV SGA sequencing[7]	X								X							X
HIV genotypic testing for ART resistance[7]	X								X				X			X
HIV reservoir size assessment	X						X						X			
Humoral Assays[3]		X[8]			X	X	X		X		X		X			X
Cellular Assays[3]		X[8]					X		X		X		X			X
HLA typing		X[8]														
**PHARMACOKINETICS PGT121 ELISA**		X[4]	X	X	X	X	X	X	X	X	X	X	X	X	X	X
**MUCOSAL SAMPLING**		X[6]	X			X	X									
**PLASMA/SERUM STORAGE**	X	X	X	X	X	X	X	X	X		X		X			X
**PBMCs STORAGE**		X	X	X	X	X		X			X		X			X

1. HIV risk reduction counselling as secondary prevention to reduce onward transmission
2. At baseline, approximately 30 minutes after IP administration start, and at hours 1 through 12 after IV infusion start. Local and systemic reactogenicity will be assessed by clinic staff at visits on study days 1, 2 and 3. Local and Systemic reactogenicity will also be assessed by the participant using the Memory Aid on study days 1, 2, and 3
3. See Laboratory Analytical Plan for details

4. Day 0 PK draws done immediately before IP administration, at the end of the IV infusion of IP, and 30 minutes and 3 hours post end of the IP administration. Additional PK draws on day 0 are done 6, 9 and 12 hours after the start of the IV infusion of IP. See SOM for details
5. Early Termination (ET): Procedures to be performed at ET are the same as last visit procedures
6. Cervico-vaginal and/or rectal mucosal sampling (optional) on Day 0 must be done prior to IV infusion of IP. See SOM for details
7. HIV envelope single genome amplification (SGA) sequencing and ART resistance genotyping will be only if they develop detectable plasma viremia >1000 HIV cp/ml during the study.
8. Day 0 sample collections for laboratory tests must be done pre-infusion.

APPENDIX C: SCHEDULE OF PROCEDURES – GROUP 3 (A, B, C, D, E, F)

Study Month		0								1		2		3	4	5	6
**Study Week**		**0**				**1**		**2**	**3**	**4**	**6**	**8**	**10**	**12**	**16**	**20**	**24**
**Study Day**	**Scr**	**0**	**1**	**2**	**3**	**7**	**10**	**14**	**21**	**28**	**42**	**56**	**70**	**84**	**112**	**140**	**168/ET[5]**
**Visit Windows (Days)**	**-42**	**0**	**0**	**0**	**0**	**± 1**	**0**	**± 2**	**± 2**	**± 2**	**± 3**	**± 3**	**±3**	**±7**	**±7**	**±7**	**±7**
**INVESTIGATIONAL PRODUCT**																	
Investigational Product /Placebo		X															
**CONSENT/ASSESSMENTS/COUNSELLING**																	
Informed Consent	X																
Assessment of Understanding	X																
HIV Risk Reduction Counselling[1]	X	X								X		X		X	X	X	X
ART counselling	X	X										X					X
Family Planning Counselling	X	X															
Social Impact Assessment																	X
**CLINICAL SAFETY ASSESSMENTS**																	
Comprehensive Medical History	X																
Interim Medical History		X	X	X	X	X	X	X	X	X	X	X					
Concomitant Medications	X	X	X	X	X	X	X	X	X	X	X	X					
General Physical Exam	X																X
Directed Physical Exam		X	X	X	X	X	X	X	X	X	X	X	X	X	X	X	
Weight	X	X															X
Height	X																
Vital Signs	X	X[2]	X	X	X	X	X	X	X	X	X	X	X	X	X	X	X
Local & Systemic Reactogenicity		X[2]	X	X	X												
Adverse Events		X	X	X	X	X	X	X	X	X	X	X					
Serious Adverse Events and pIMD	X	X	X	X	X	X	X	X	X	X	X	X	X	X	X	X	X

Study Month		0								1		2		3	4	5	6
**Study Week**		**0**				**1**		**2**	**3**	**4**	**6**	**8**	**10**	**12**	**16**	**20**	**24**
**Study Day**	**Scr**	**0**	**1**	**2**	**3**	**7**	**10**	**14**	**21**	**28**	**42**	**56**	**70**	**84**	**112**	**140**	**168/ET[5]**
**Visit Windows (Days)**	**-42**	**0**	**0**	**0**	**0**	**± 1**	**0**	**± 2**	**± 2**	**± 2**	**± 3**	**± 3**	**±3**	**±7**	**±7**	**±7**	**±7**
**CLINICAL LABORATORY TESTS**																	
Hematology and Coagulation	X	X[9]	X		X	X		X		X		X		X	X	X	X
CD4	X	X[9]				X		X		X		X					X
Clinical Chemistry	X	X[9]	X		X	X		X		X		X		X	X	X	X
Urine Dipstick[7]	X	X[9]	X		X	X		X		X		X		X	X	X	X
Urine Pregnancy test	X	X[9]								X		X		X			X
Active Syphilis	X																
Hepatitis B	X																
Hepatitis C	X																
HIV 4th generation Ag/Ab test	X																
HIV Viral Load	X	X[9]	X	X	X	X	X	X	X	X	X	X	X	X	X	X	X
**RESEARCH LABORATORY TESTS**	-	-	-	-	-	-	-	-	-	-	-	-	-	-	-	-	-
Anti PGT121 Antibodies (ADA)		X[9]								X		X		X			X
PGT121 susceptibility testing	X									X							X
HIV SGA sequencing[8]	X									X							X
HIV genotypic testing for ART resistance[8]	X									X				X			X
HIV reservoir size assessment[1]	X							X						X			
Humoral Assays[3]		X[9]			X	X		X		X		X		X			X
Cellular Assays[3]		X[9]						X		X		X		X			X
HLA typing		X[9]															
**PHARMACOKINETICS PGT121 ELISA**		X[4]	X	X	X	X		X	X	X	X	X	X	X	X	X	X
**MUCOSAL SAMPLING**		X[6]	X			X		X									
**PLASMA/SERUM STORAGE**		X	X	X	X	X	X	X	X	X		X		X			X
**PBMCs STORAGE**		X	X	X	X	X	X		X			X		X			X

1. HIV risk reduction counselling as secondary prevention to reduce onward transmission
2. At baseline, approximately 30 minutes after IP administration start, and at hours 1 through 12 after IV infusion start. Local and systemic reactogenicity will be assessed by clinic staff at visits on study days 1, 2 and 3. Local and Systemic reactogenicity will also be assessed by the participant using the Memory Aid on study days 1, 2, and 3
3. See Laboratory Analytical Plan for details
4. Day 0 PK draws done immediately before IP administration, at the end of the IV infusion of IP, and 30 minutes and 3 hours post end of the IP administration. Additional PK draws on day 0 are done 6, 9 and 12 hours after the start of the IV infusion of IP. See SOM for details
5. Early Termination (ET): Procedures to be performed at ET are the same as last visit procedures
6. Cervico-vaginal and/or rectal mucosal sampling (optional) on Day 0 must be done prior to IV infusion of IP. See SOM for details
7. Urinalysis will only be conducted at visits after screening if clinically indicated.
8. HIV envelope single genome amplification (SGA) sequencing and ART resistance genotyping will be only if they develop detectable plasma viremia >1000 HIV cp/ml during the study.
9. Day 0 sample collections for laboratory tests must be done pre-infusion.

APPENDIX D: LOW RISK CRITERIA

Low risk will be defined as:

1. **SEXUAL BEHAVIORS**

   In the **last 12 months** did not:

   - Have oral, vaginal or anal intercourse with an HIV-infected partner, or partner who uses injection drugs.
   - Gave or receive money, drugs, gifts, or services in exchange for oral, vaginal or anal sex

   AND

   In the **last 6 months** has abstained from penile/anal or penile/vaginal intercourse

   OR

   In the **last 6 months**:

   - Had 4 or fewer partners of the opposite birth sex for vaginal and/or anal intercourse, OR

   Is MSM (person born male with partner(s) born male) who, in the **last 12 months**:

   - Had 2 or fewer MSM partners for anal intercourse and had no unprotected anal sex with MSM, OR
   - Had unprotected anal intercourse with only 1 MSM partner, within a monogamous relationship lasting at least 12 months (during which neither partner had any other partners). If the monogamous relationship ended, the participant may then have had protected anal intercourse with 1 other MSM partner (total 2 or fewer partners in the last 12 months).

   Is a transgender person, regardless of the point on the transition spectrum, having sex with men (born male) and/or other transgender persons, who in the last 12 months:

   - Had 2 or fewer partners for anal or vaginal intercourse, and had no unprotected anal or vaginal sex, OR
   - Had unprotected anal or vaginal intercourse sex with 1 partner only within a monogamous relationship lasting at least 12 months (during which neither partner had any other partners). If the monogamous relationship ended, may then have had protected anal or vaginal sex with one other partner (total 2 or fewer partners in the last 12 months).

   AND

   Uses or intends to use condoms in situations which may include penile/anal or penile/vaginal intercourse with new partners of unknown HIV status, occasional partners, partners outside a primary relationship, and/or partners known to have other partners.

2. **NON-SEXUAL BEHAVIORS**

   In the **last 12 months** did not:

   - Inject drugs or other substances without a prescription
   - Use cocaine, methamphetamine, or excessive alcohol, which in the investigator's judgement, rendered the participant at greater than low risk for acquiring HIV infection

The investigator's judgement should consider local epidemiologic information about HIV prevalence in the area and community networks.

A participant is NOT appropriate for inclusion if he/she:

Acquired an STI (ie, new infection) in the last 12 months:

- Syphilis
- Gonorrhea
- Non-gonococcal urethritis
- HSV-2
- Chlamydia
- Pelvic inflammatory disease (PID)
- Trichomonas
- Mucopurulent cervicitis
- Epididymitis
- Proctitis
- Lymphogranuloma venereum
- Chancroid
- Hepatitis B

APPENDIX E: ADVERSE EVENT SEVERITY ASSESSMENT TABLE

Adapted from: DIVISION OF AIDS (DAIDS) TABLE FOR GRADING THE SEVERITY OF ADULT AND PEDIATRIC ADVERSE EVENTS Version 2.0, November 2014

The *Division of AIDS* (*DAIDS) Table for Grading the Severity of Adult and Pediatric Adverse Events, Version 2.0* consists of parameters, or AEs, with severity grading guidance that are to be used in DAIDS clinical trials for safety data reporting to maintain accuracy and consistency in the evaluation of AEs.

I. Instructions and Clarifications

Grading Adult and Pediatric AEs

When a single parameter is not appropriate for grading an AE in both adult and pediatric populations, separate parameters with specified age ranges are provided. If no distinction between adult and pediatric populations has been made, the listed parameter should be used for grading an AE in both populations.

Note: The term "severe" is not the same as "serious" in classifying AEs. The severity of a specific event describes its intensity, and it is the intensity which is graded. Seriousness, which is not graded, relates to an outcome of an AE and is a regulatory definition.

Estimating Severity Grade for Parameters Not Identified in the Table

The functional table below should be used to grade the severity of an AE that is not specifically identified in the grading table. In addition, all deaths related to an AE are to be classified as grade 5.

PARAMETER	GRADE 1 MILD	GRADE 2 MODERATE	GRADE 3 SEVERE	GRADE 4 POTENTIALLY LIFE-THREATENING
**Clinical** adverse event **NOT** identified elsewhere in the grading table	Mild symptoms causing no or minimal interference with usual social & functional activities with intervention not indicated	Moderate symptoms causing greater than minimal interference with usual social & functional activities with intervention indicated	Severe symptoms causing inability to perform usual social & functional activities with intervention or hospitalization indicated	Potentially life-threatening symptoms causing inability to perform basic self-care functions with intervention indicated to prevent permanent impairment, persistent disability, or death

Determining Severity Grade for Parameters "Between Grades"

If the severity of an AE could fall in either one of two grades (e.g., the severity of an AE could be either Grade 2 or Grade 3), sites should select the higher of the two grades.

Laboratory Values

General. An asymptomatic, abnormal laboratory finding without an accompanying AE should not be reported to DAIDS in an expedited timeframe unless it meets protocol-specific reporting requirements. Sites should refer to the applicable network standards for reporting abnormal laboratory findings on the CRFs.

Values Below Grade 1

Any laboratory value that is between the ULN and grade 1 (for high values) or the LLN and grade 1 (for low values) should not be graded or reported as an AE. Sites should consult the Manual for Expedited Reporting of Adverse Events to DAIDS, Version 2.0 and their protocol when making an assessment of the need to report an AE.

Overlap of Local Laboratory Normal Values with Grading Table Ranges.

When local laboratory normal values fall within grading table laboratory ranges, the severity grading is based on the ranges in the grading table unless there is a protocol-specific grading criterion for the

laboratory value. For example, “Magnesium, Low" has a grade 1 range of 1.2 to < 1.4 mEq/L, while a particular laboratory’s normal range for magnesium may be 1.3 to 2.8 mEq/L. If a study participant’s magnesium laboratory value is 1.3 mEq/L, the laboratory value should be graded as grade 1.

II. Definitions of terms used in the Table:

Basic Self-care Functions	Adult<br>Activities such as bathing, dressing, toileting, transfer or movement, continence, and feeding.<br><br>Young Children<br>Activities that are age and culturally appropriate, such as feeding one’s self with culturally appropriate eating implements.
LLN	Lower limit of normal
Intervention	Medical, surgical, or other procedures recommended or provided by a healthcare professional for the treatment of an adverse event.
NA	Not Applicable
ULN	Upper limit of normal
Usual Social & Functional Activities	Activities which adults and children perform on a routine basis and those which are part of regular activities of daily living, for example:<br><br>Adults<br>Adaptive tasks and desirable activities, such as going to work, shopping, cooking, use of transportation, or pursuing a hobby.<br><br>Young Children<br>Activities that are age and culturally appropriate, such as social interactions, play activities, or learning tasks.

Major Clinical Conditions

Cardiovascular

PARAMETER	GRADE 1 MILD	GRADE 2 MODERATE	GRADE 3 SEVERE	GRADE 4 POTENTIALLY LIFE-THREATENING
**Arrhythmia** (by ECG or physical examination) *Specify type, if applicable*	No symptoms AND No intervention indicated	No symptoms AND Non-urgent intervention indicated	Non-life-threatening symptoms AND Non-urgent intervention indicated	Life-threatening arrhythmia OR Urgent intervention indicated
**Blood Pressure Abnormalities**[1] ***Hypertension*** *(with the lowest reading taken after repeat testing during a visit)* *≥ 18 years of age*	140 to < 160 mmHg systolic OR 90 to < 100 mmHg diastolic	≥ 160 to < 180 mmHg systolic OR ≥ 100 to < 110 mmHg diastolic	≥ 180 mmHg systolic OR ≥ 110 mmHg diastolic	Life-threatening consequences in a participant not previously diagnosed with hypertension (e.g., malignant hypertension) OR Hospitalization indicated
< 18 years of age	> 120/80 mmHg	≥ 95th to < 99th percentile + 5 mmHg adjusted for age, height, and gender (systolic and/or diastolic)	≥ 99th percentile + 5 mmHg adjusted for age, height, and gender (systolic and/or diastolic)	Life-threatening consequences in a participant not previously diagnosed with hypertension (e.g., malignant hypertension) OR Hospitalization indicated
Hypotension	No symptoms	Symptoms corrected with oral fluid replacement	Symptoms AND IV fluids indicated	Shock requiring use of vasopressors or mechanical assistance to maintain blood pressure
**Cardiac Ischemia or Infarction** *Report only one*	NA	NA	New symptoms with ischemia (stable angina) OR New testing consistent with ischemia	Unstable angina OR Acute myocardial infarction
**Heart Failure**	No symptoms AND Laboratory or cardiac imaging abnormalities	Symptoms with mild to moderate activity or exertion	Symptoms at rest or with minimal activity or exertion (e.g., hypoxemia) OR Intervention indicated (e.g., oxygen)	Life-threatening consequences OR Urgent intervention indicated (e.g., vasoactive medications, ventricular assist device, heart transplant)
**Hemorrhage** (with significant acute blood loss)	NA	Symptoms AND No transfusion indicated	Symptoms AND Transfusion of ≤ 2 units packed RBCs indicated	Life-threatening hypotension OR Transfusion of > 2 units packed RBCs (for children, packed RBCs > 10 cc/kg) indicated

[1] Blood pressure norms for children < 18 years of age can be found in: Expert Panel on Integrated Guidelines for Cardiovascular Health and Risk Reduction in Children and Adolescents. *Pediatrics* 2011;128;S213; originally published online November 14, 2011; DOI: 10.1542/peds.2009- 2107C.

PARAMETER	GRADE 1<br>MILD	GRADE 2<br>MODERATE	GRADE 3<br>SEVERE	GRADE 4<br>POTENTIALLY LIFE-THREATENING
**Prolonged PR Interval or AV Block**<br>*Report only one*<br>*> 16 years of age*	PR interval 0.21 to < 0.25 seconds	PR interval ≥ 0.25 seconds OR Type I 2nd degree AV block	Type II 2nd degree AV block OR Ventricular pause ≥ 3.0 seconds	Complete AV block
≤ 16 years of age	1st degree AV block (PR interval > normal for age and rate)	Type I 2nd degree AV block	Type II 2nd degree AV block OR Ventricular pause ≥ 3.0 seconds	Complete AV block
**Prolonged QTc Interval**[2]	0.45 to 0.47 seconds	> 0.47 to 0.50 seconds	> 0.50 seconds OR ≥ 0.06 seconds above baseline	Life-threatening consequences (e.g., Torsade de pointes, other associated serious ventricular dysrhythmia)
**Thrombosis or Embolism**<br>*Report only one*	NA	Symptoms AND No intervention indicated	Symptoms AND Intervention indicated	Life-threatening embolic event (e.g., pulmonary embolism, thrombus)

[2] As per Bazett's formula.

Dermatologic

PARAMETER	GRADE 1<br>MILD	GRADE 2<br>MODERATE	GRADE 3<br>SEVERE	GRADE 4<br>POTENTIALLY LIFE-THREATENING
**Alopecia** (scalp only)	Detectable by study participant, caregiver, or physician AND Causing no or minimal interference with usual social & functional activities	Obvious on visual inspection AND Causing greater than minimal interference with usual social & functional activities	NA	NA
**Bruising**	Localized to one area	Localized to more than one area	Generalized	NA
**Cellulitis**	NA	Non-parenteral treatment indicated (e.g., oral antibiotics, antifungals, antivirals)	IV treatment indicated (e.g., IV antibiotics, antifungals, antivirals)	Life-threatening consequences (e.g., sepsis, tissue necrosis)
**Hyperpigmentation**	Slight or localized causing no or minimal interference with usual social & functional activities	Marked or generalized causing greater than minimal interference with usual social & functional activities	NA	NA

**Hypopigmentation**	Slight or localized causing no or minimal interference with usual social & functional activities	Marked or generalized causing greater than minimal interference with usual social & functional activities	NA	NA
**Petechiae**	Localized to one area	Localized to more than one area	Generalized	NA
**Pruritus**[3] (without skin lesions)	Itching causing no or minimal interference with usual social & functional activities	Itching causing greater than minimal interference with usual social & functional activities	Itching causing inability to perform usual social & functional activities	NA
**Rash** *Specify type, if applicable*	Localized rash	Diffuse rash OR Target lesions	Diffuse rash AND Vesicles or limited number of bullae or superficial ulcerations of mucous membrane limited to one site	Extensive or generalized bullous lesions OR Ulceration of mucous membrane involving two or more distinct mucosal sites OR Stevens-Johnson syndrome OR Toxic epidermal necrolysis

[3] For pruritus associated with injections or infusions, see the *Site Reactions to Injections and Infusions* section (page 23).

Endocrine and Metabolic

PARAMETER	GRADE 1 MILD	GRADE 2 MODERATE	GRADE 3 SEVERE	GRADE 4 POTENTIALLY LIFE-THREATENING
**Diabetes Mellitus**	Controlled without medication	Controlled with medication OR Modification of current medication regimen	Uncontrolled despite treatment modification OR Hospitalization for immediate glucose control indicated	Life-threatening consequences (e.g., ketoacidosis, hyperosmolar non-ketotic coma, end organ failure)
**Gynecomastia**	Detectable by study participant, caregiver, or physician AND Causing no or minimal interference with usual social & functional activities	Obvious on visual inspection AND Causing pain with greater than minimal interference with usual social & functional activities	Disfiguring changes AND Symptoms requiring intervention or causing inability to perform usual social & functional activities	NA

**Hyperthyroidism**	No symptoms AND Abnormal laboratory value	Symptoms causing greater than minimal interference with usual social & functional activities OR Thyroid suppression therapy indicated	Symptoms causing inability to perform usual social & functional activities OR Uncontrolled despite treatment modification	Life-threatening consequences (e.g., thyroid storm)
**Hypothyroidism**	No symptoms AND Abnormal laboratory value	Symptoms causing greater than minimal interference with usual social & functional activities OR Thyroid replacement therapy indicated	Symptoms causing inability to perform usual social & functional activities OR Uncontrolled despite treatment modification	Life-threatening consequences (e.g., myxedema coma)
**Lipoatrophy**[4]	Detectable by study participant, caregiver, or physician AND Causing no or minimal interference with usual social & functional activities	Obvious on visual inspection AND Causing greater than minimal interference with usual social & functional activities	Disfiguring changes	NA
**Lipohypertrophy**[5]	Detectable by study participant, caregiver, or physician AND Causing no or minimal interference with usual social & functional activities	Obvious on visual inspection AND Causing greater than minimal interference with usual social & functional activities	Disfiguring changes	NA

4 Definition: A disorder characterized by fat loss in the face, extremities, and buttocks.
5 Definition: A disorder characterized by abnormal fat accumulation on the back of the neck, breasts, and abdomen.

Gastro-intestinal

PARAMETER	GRADE 1 MILD	GRADE 2 MODERATE	GRADE 3 SEVERE	GRADE 4 POTENTIALLY LIFE-THREATENING
**Anorexia**	Loss of appetite without decreased oral intake	Loss of appetite associated with decreased oral intake without significant weight loss	Loss of appetite associated with significant weight loss	Life-threatening consequences OR Aggressive intervention indicated (e.g., tube feeding, total parenteral nutrition)
**Ascites**	No symptoms	Symptoms AND Intervention indicated (e.g., diuretics, therapeutic paracentesis)	Symptoms recur or persist despite intervention	Life-threatening consequences

**Bloating or Distension**<br>*Report only one*	Symptoms causing no or minimal interference with usual social & functional activities	Symptoms causing greater than minimal interference with usual social & functional activities	Symptoms causing inability to perform usual social & functional activities	NA
**Cholecystitis**	NA	Symptoms AND Medical intervention indicated	Radiologic, endoscopic, or operative intervention indicated	Life-threatening consequences (e.g., sepsis, perforation)
**Constipation**	NA	Persistent constipation requiring regular use of dietary modifications, laxatives, or enemas	Obstipation with manual evacuation indicated	Life-threatening consequences (e.g., obstruction)
**Diarrhea**<br>*≥ 1 year of age*	Transient or intermittent episodes of unformed stools OR Increase of ≤ 3 stools over baseline per 24-hour period	Persistent episodes of unformed to watery stools OR Increase of 4 to 6 stools over baseline per 24-hour period	Increase of ≥ 7 stools per 24-hour period OR IV fluid replacement indicated	Life-threatening consequences (e.g., hypotensive shock)
< 1 year of age	Liquid stools (more unformed than usual) but usual number of stools	Liquid stools with increased number of stools OR Mild dehydration	Liquid stools with moderate dehydration	Life-threatening consequences (e.g., liquid stools resulting in severe dehydration, hypotensive shock)
**Dysphagia or Odynophagia**<br>*Report only one and specify location*	Symptoms but able to eat usual diet	Symptoms causing altered dietary intake with no intervention indicated	Symptoms causing severely altered dietary intake with intervention indicated	Life-threatening reduction in oral intake
**Gastrointestinal Bleeding**	Not requiring intervention other than iron supplement	Endoscopic intervention indicated	Transfusion indicated	Life-threatening consequences (e.g., hypotensive shock)

**PARAMETER**	**GRADE 1 MILD**	**GRADE 2 MODERATE**	**GRADE 3 SEVERE**	**GRADE 4 POTENTIALLY LIFE-THREATENING**
**Mucositis or Stomatitis**<br>*Report only one and specify location*	Mucosal erythema	Patchy pseudomembranes or ulcerations	Confluent pseudomembranes or ulcerations OR Mucosal bleeding with minor trauma	Life-threatening consequences (e.g., aspiration, choking) OR Tissue necrosis OR Diffuse spontaneous mucosal bleeding
**Nausea**	Transient (< 24 hours) or intermittent AND No or minimal interference with oral intake	Persistent nausea resulting in decreased oral intake for 24 to 48 hours	Persistent nausea resulting in minimal oral intake for > 48 hours OR Rehydration indicated (e.g., IV fluids)	Life-threatening consequences (e.g., hypotensive shock)

**Pancreatitis**	NA	Symptoms with hospitalization not indicated	Symptoms with hospitalization indicated	Life-threatening consequences (e.g., circulatory failure, hemorrhage, sepsis)
**Perforation** (colon or rectum)	NA	NA	Intervention indicated	Life-threatening consequences
**Proctitis**	Rectal discomfort with no intervention indicated	Symptoms causing greater than minimal interference with usual social & functional activities OR Medical intervention indicated	Symptoms causing inability to perform usual social & functional activities OR Operative intervention indicated	Life-threatening consequences (e.g., perforation)
**Rectal Discharge**	Visible discharge	Discharge requiring the use of pads	NA	NA
**Vomiting**	Transient or intermittent AND No or minimal interference with oral intake	Frequent episodes with no or mild dehydration	Persistent vomiting resulting in orthostatic hypotension OR Aggressive rehydration indicated (e.g., IV fluids)	Life-threatening consequences (e.g., hypotensive shock)

Musculoskeletal

PARAMETER	GRADE 1<br>MILD	GRADE 2<br>MODERATE	GRADE 3<br>SEVERE	GRADE 4<br>POTENTIALLY LIFE-THREATENING
**Arthralgia**	Joint pain causing no or minimal interference with usual social & functional activities	Joint pain causing greater than minimal interference with usual social & functional activities	Joint pain causing inability to perform usual social & functional activities	Disabling joint pain causing inability to perform basic self-care functions
**Arthritis**	Stiffness or joint swelling causing no or minimal interference with usual social & functional activities	Stiffness or joint swelling causing greater than minimal interference with usual social & functional activities	Stiffness or joint swelling causing inability to perform usual social & functional activities	Disabling joint stiffness or swelling causing inability to perform basic self-care functions
**Myalgia** (generalized)	Muscle pain causing no or minimal interference with usual social & functional activities	Muscle pain causing greater than minimal interference with usual social & functional activities	Muscle pain causing inability to perform usual social & functional activities	Disabling muscle pain causing inability to perform basic self-care functions
**Osteonecrosis**	NA	No symptoms but with radiographic findings AND No operative intervention indicated	Bone pain with radiographic findings OR Operative intervention indicated	Disabling bone pain with radiographic findings causing inability to perform basic self-care functions
**Osteopenia**[6]<br>*≥ 30 years of age*	BMD t-score -2.5 to -1	NA	NA	NA
< 30 years of age	BMD z-score -2 to -1	NA	NA	NA
**Osteoporosis**[6]<br>*≥ 30 years of age*	NA	BMD t-score < -2.5	Pathologic fracture (e.g., compression fracture causing loss of vertebral height)	Pathologic fracture causing life-threatening consequences
< 30 years of age	NA	BMD z-score < -2	Pathologic fracture (e.g., compression fracture causing loss of vertebral height)	Pathologic fracture causing life-threatening consequences

[6] BMD t and z scores can be found in: Kanis JA on behalf of the World Health Organization Scientific Group (2007). Assessment of osteoporosis at the primary health-care level. Technical Report. World Health Organization Collaborating Centre for Metabolic Bone Diseases, University of Sheffield, UK. 2007: Printed by the University of Sheffield.

Neurologic

PARAMETER	GRADE 1 MILD	GRADE 2 MODERATE	GRADE 3 SEVERE	GRADE 4 POTENTIALLY LIFE-THREATENING
**Acute CNS Ischemia**	NA	NA	Transient ischemic attack	Cerebral vascular accident (e.g., stroke with neurological deficit)
**Altered Mental Status** (for Dementia, see *Cognitive, Behavioral, or Attentional Disturbance* below)	Changes causing no or minimal interference with usual social & functional activities	Mild lethargy or somnolence causing greater than minimal interference with usual social & functional activities	Confusion, memory impairment, lethargy, or somnolence causing inability to perform usual social & functional activities	Delirium OR Obtundation OR Coma
**Ataxia**	Symptoms causing no or minimal interference with usual social & functional activities OR No symptoms with ataxia detected on examination	Symptoms causing greater than minimal interference with usual social & functional activities	Symptoms causing inability to perform usual social & functional activities	Disabling symptoms causing inability to perform basic self-care functions
**Cognitive, Behavioral, or Attentional Disturbance** (includes dementia and attention deficit disorder) *Specify type, if applicable*	Disability causing no or minimal interference with usual social & functional activities OR Specialized resources not indicated	Disability causing greater than minimal interference with usual social & functional activities OR Specialized resources on part- time basis indicated	Disability causing inability to perform usual social & functional activities OR Specialized resources on a full-time basis indicated	Disability causing inability to perform basic self-care functions OR Institutionalization indicated
**Developmental Delay** *< 18 years of age* *Specify type, if applicable*	Mild developmental delay, either motor or cognitive, as determined by comparison with a developmental screening tool appropriate for the setting	Moderate developmental delay, either motor or cognitive, as determined by comparison with a developmental screening tool appropriate for the setting	Severe developmental delay, either motor or cognitive, as determined by comparison with a developmental screening tool appropriate for the setting	Developmental regression, either motor or cognitive, as determined by comparison with a developmental screening tool appropriate for the setting
**Headache**	Symptoms causing no or minimal interference with usual social & functional activities	Symptoms causing greater than minimal interference with usual social & functional activities	Symptoms causing inability to perform usual social & functional activities	Symptoms causing inability to perform basic self-care functions OR Hospitalization indicated OR Headache with significant impairment of alertness or other neurologic function

PARAMETER	GRADE 1<br>MILD	GRADE 2<br>MODERATE	GRADE 3<br>SEVERE	GRADE 4<br>POTENTIALLY LIFE-THREATENING
**Neuromuscular Weakness** (includes myopathy and neuropathy)<br>*Specify type, if applicable*	Minimal muscle weakness causing no or minimal interference with usual social & functional activities OR No symptoms with decreased strength on examination	Muscle weakness causing greater than minimal interference with usual social & functional activities	Muscle weakness causing inability to perform usual social & functional activities	Disabling muscle weakness causing inability to perform basic self-care functions OR Respiratory muscle weakness impairing ventilation
**Neurosensory Alteration** (includes paresthesia and painful neuropathy)<br>*Specify type, if applicable*	Minimal paresthesia causing no or minimal interference with usual social & functional activities OR No symptoms with sensory alteration on examination	Sensory alteration or paresthesia causing greater than minimal interference with usual social & functional activities	Sensory alteration or paresthesia causing inability to perform usual social & functional activities	Disabling sensory alteration or paresthesia causing inability to perform basic self-care functions
**Seizures**<br>***New Onset Seizure*** *≥ 18 years of age*	NA	NA	1 to 3 seizures	Prolonged and repetitive seizures (e.g., status epilepticus) OR Difficult to control (e.g., refractory epilepsy)
< 18 years of age (includes new or pre- existing febrile seizures)	Seizure lasting < 5 minutes with < 24 hours postictal state	Seizure lasting 5 to < 20 minutes with < 24 hours postictal state	Seizure lasting ≥ 20 minutes OR > 24 hours postictal state	Prolonged and repetitive seizures (e.g., status epilepticus) OR Difficult to control (e.g., refractory epilepsy)
Pre-existing Seizure	NA	Increased frequency from previous level of control without change in seizure character	Change in seizure character either in duration or quality (e.g., severity or focality)	Prolonged and repetitive seizures (e.g., status epilepticus) OR Difficult to control (e.g., refractory epilepsy)
**Syncope**	Near syncope without loss of consciousness (e.g., pre-syncope)	Loss of consciousness with no intervention indicated	Loss of consciousness AND Hospitalization or intervention required	NA

Pregnancy, Puerperium, and Perinatal

PARAMETER	GRADE 1 MILD	GRADE 2 MODERATE	GRADE 3 SEVERE	GRADE 4 POTENTIALLY LIFE-THREATENING
**Fetal Death or Stillbirth** (report using mother's participant ID) *Report only one*	NA	NA	Fetal loss occurring at ≥ 20 weeks gestation	NA
**Preterm Delivery**[7] (report using mother's participant ID)	Delivery at 34 to < 37 weeks gestational age	Delivery at 28 to < 34 weeks gestational age	Delivery at 24 to < 28 weeks gestational age	Delivery at < 24 weeks gestational age
**Spontaneous Abortion or Miscarriage**[8] (report using mother's participant ID) *Report only one*	Chemical pregnancy	Uncomplicated spontaneous abortion or miscarriage	Complicated spontaneous abortion or miscarriage	NA

[7] Definition: A delivery of a live-born neonate occurring at ≥ 20 to < 37 weeks gestational age.
[8] Definition: A clinically recognized pregnancy occurring at < 20 weeks gestational age.

Psychiatric

PARAMETER	GRADE 1 MILD	GRADE 2 MODERATE	GRADE 3 SEVERE	GRADE 4 POTENTIALLY LIFE-THREATENING
**Insomnia**	Mild difficulty falling asleep, staying asleep, or waking up early	Moderate difficulty falling asleep, staying asleep, or waking up early	Severe difficulty falling asleep, staying asleep, or waking up early	NA
**Psychiatric Disorders** (includes anxiety, depression, mania, and psychosis) *Specify disorder*	Symptoms with intervention not indicated OR Behavior causing no or minimal interference with usual social & functional activities	Symptoms with intervention indicated OR Behavior causing greater than minimal interference with usual social & functional activities	Symptoms with hospitalization indicated OR Behavior causing inability to perform usual social & functional activities	Threatens harm to self or others OR Acute psychosis OR Behavior causing inability to perform basic self-care functions
**Suicidal Ideation or Attempt** *Report only one*	Preoccupied with thoughts of death AND No wish to kill oneself	Preoccupied with thoughts of death AND Wish to kill oneself with no specific plan or intent	Thoughts of killing oneself with partial or complete plans but no attempt to do so OR Hospitalization indicated	Suicide attempted

Respiratory

PARAMETER	GRADE 1 MILD	GRADE 2 MODERATE	GRADE 3 SEVERE	GRADE 4 POTENTIALLY LIFE-THREATENING
**Acute Bronchospasm**	Forced expiratory volume in 1 second or peak flow reduced to ≥ 70 to < 80% OR Mild symptoms with intervention not indicated	Forced expiratory volume in 1 second or peak flow 50 to < 70% OR Symptoms with intervention indicated OR Symptoms causing greater than minimal interference with usual social & functional activities	Forced expiratory volume in 1 second or peak flow 25 to < 50% OR Symptoms causing inability to perform usual social & functional activities	Forced expiratory volume in 1 second or peak flow < 25% OR Life-threatening respiratory or Hemodynamic compromise OR Intubation
**Dyspnea or Respiratory Distress**<br>*Report only one*	Dyspnea on exertion with no or minimal interference with usual social & functional activities OR Wheezing OR Minimal increase in respiratory rate for age	Dyspnea on exertion causing greater than minimal interference with usual social & functional activities OR Nasal flaring OR Intercostal retractions OR Pulse oximetry 90 to < 95%	Dyspnea at rest causing inability to perform usual social & functional activities OR Pulse oximetry < 90%	Respiratory failure with ventilator support indicated (e.g., CPAP, BPAP, intubation)

Sensory

PARAMETER	GRADE 1 MILD	GRADE 2 MODERATE	GRADE 3 SEVERE	GRADE 4 POTENTIALLY LIFE-THREATENING
**Hearing Loss**<br>*≥ 12 years of age*	NA	Hearing aid or intervention not indicated	Hearing aid or intervention indicated	Profound bilateral hearing loss (> 80 dB at 2 kHz and above) OR Non-serviceable hearing (i.e., >50 dB audiogram and <50% speech discrimination)
< 12 years of age (based on a 1, 2, 3, 4, 6 and 8 kHz audiogram)	> 20 dB hearing loss at ≤ 4 kHz	> 20 dB hearing loss at > 4 kHz	> 20 dB hearing loss at ≥ 3 kHz in one ear with additional speech language related services indicated (where available) OR Hearing loss sufficient to indicate therapeutic intervention, including hearing aids	Audiologic indication for cochlear implant and additional speech-language related services indicated (where available)

**Tinnitus**	Symptoms causing no or minimal interference with usual social & functional activities with intervention not indicated	Symptoms causing greater than minimal interference with usual social & functional activities with intervention indicated	Symptoms causing inability to perform usual social & functional activities	NA
**Uveitis**	No symptoms AND Detectable on examination	Anterior uveitis with symptoms OR Medicamylasal intervention indicated	Posterior or pan- uveitis OR Operative intervention indicated	Disabling visual loss in affected eye(s)
**Vertigo**	Vertigo causing no or minimal interference with usual social & functional activities	Vertigo causing greater than minimal interference with usual social & functional activities	Vertigo causing inability to perform usual social & functional activities	Disabling vertigo causing inability to perform basic self-care functions
**Visual Changes** (assessed from baseline)	Visual changes causing no or minimal interference with usual social & functional activities	Visual changes causing greater than minimal interference with usual social & functional activities	Visual changes causing inability to perform usual social & functional activities	Disabling visual loss in affected eye(s)

Systemic

PARAMETER	GRADE 1 MILD	GRADE 2 MODERATE	GRADE 3 SEVERE	GRADE 4 POTENTIALLY LIFE-THREATENING
**Acute Allergic Reaction**	Localized urticaria (wheals) with no medical intervention indicated	Localized urticaria with intervention indicated OR Mild angioedema with no intervention indicated	Generalized urticaria OR Angioedema with intervention indicated OR Symptoms of mild bronchospasm	Acute anaphylaxis OR Life-threatening bronchospasm OR Laryngeal edema
**Chills**	Symptoms causing no or minimal interference with usual social & functional activities	Symptoms causing greater than minimal interference with usual social & functional activities	Symptoms causing inability to perform usual social & functional activities	NA
**Cytokine Release Syndrome**[9]	Mild signs and symptoms AND Therapy (i.e., antibody infusion) interruption not indicated	Therapy (i.e., antibody infusion) interruption indicated AND Responds promptly to symptomatic treatment OR Prophylactic medications indicated for ≤ 24 hours	Prolonged severe signs and symptoms OR Recurrence of symptoms following initial improvement	Life-threatening consequences (e.g., requiring pressor or ventilator support)
**Fatigue or Malaise** *Report only one*	Symptoms causing no or minimal interference with usual social & functional activities	Symptoms causing greater than minimal interference with usual social & functional activities	Symptoms causing inability to perform usual social & functional activities	Incapacitating symptoms of fatigue or malaise causing inability to perform basic self-care functions
**Fever** (non-axillary temperatures only)	38.0 to < 38.6°C or 100.4 to < 101.5°F	≥ 38.6 to < 39.3°C or ≥101.5 to < 102.7°F	≥ 39.3 to < 40.0°C or ≥ 102.7 to < 104.0°F	≥ 40.0°C or ≥ 104.0°F
**Pain**[10] (not associated with study agent injections and not specified elsewhere) *Specify location*	Pain causing no or minimal interference with usual social & functional activities	Pain causing greater than minimal interference with usual social & functional activities	Pain causing inability to perform usual social & functional activities	Disabling pain causing inability to perform basic self-care functions OR Hospitalization indicated
**Serum Sickness**[11]	Mild signs and symptoms	Moderate signs and symptoms AND Intervention indicated (e.g., antihistamines)	Severe signs and symptoms AND Higher level intervention indicated (e.g., steroids or IV fluids)	Life-threatening consequences (e.g., requiring pressor or ventilator support)

9 ~~Definition: A disorder characterized by nausea~~, headache, tachycardia, hypotension, rash, and/or shortness of breath.
10 For pain associated with injections or infusions, see the *Site Reactions to Injections and Infusions* section (page 23).
11 Definition: A disorder characterized by fever, arthralgia, myalgia, skin eruptions, lymphadenopathy, marked discomfort, and/or dyspnea.

PARAMETER	GRADE 1 MILD	GRADE 2 MODERATE	GRADE 3 SEVERE	GRADE 4 POTENTIALLY LIFE-THREATENING
**Underweight**[12]<br>*> 5 to 19 years of age*	NA	WHO BMI z-score < -2 to ≤ -3	WHO BMI z-score < -3	WHO BMI z-score < -3 with life-threatening consequences
2 to 5 years of age	NA	WHO Weight-for-height z-score < -2 to ≤-3	WHO Weight-for- height z-score < -3	WHO Weight-for-height z-score < -3 with life-threatening consequences
< 2 years of age	NA	WHO Weight-for-length z-score < -2 to ≤-3	WHO Weight-for- length z-score < -3	WHO Weight-for-length z-score < -3 with life-threatening consequences
**Weight Loss** (excludes postpartum weight loss)	NA	5 to < 9% loss in body weight from baseline	≥ 9 to < 20% loss in body weight from baseline	≥ 20% loss in body weight from baseline OR Aggressive intervention indicated (e.g., tube feeding, total parenteral nutrition)

[12] WHO reference tables may be accesed by clicking the desired age range or by accessing the following URLs: http://www.who.int/growthref/who2007 bmi for age/en/ for participants > 5 to 19 years of age and http://www.who.int/childgrowth/standards/chart catalogue/en/ for those < 5 years of age.

Urinary

PARAMETER	GRADE 1 MILD	GRADE 2 MODERATE	GRADE 3 SEVERE	GRADE 4 POTENTIALLY LIFE-THREATENING
**Urinary Tract Obstruction**	NA	Signs or symptoms of urinary tract obstruction without hydronephrosis or renal dysfunction	Signs or symptoms of urinary tract obstruction with hydronephrosis or renal dysfunction	Obstruction causing life-threatening consequences

Site Reactions to Injections and Infusions

PARAMETER	GRADE 1 MILD	GRADE 2 MODERATE	GRADE 3 SEVERE	GRADE 4 POTENTIALLY LIFE-THREATENING
**Injection Site Pain or Tenderness**<br>*Report only one*	Pain or tenderness causing no or minimal limitation of use of limb	Pain or tenderness causing greater than minimal limitation of use of limb	Pain or tenderness causing inability to perform usual social & functional activities	Pain or tenderness causing inability to perform basic self-care function OR Hospitalization indicated

**Injection Site Erythema or Redness**[13]<br>*Report only one*<br>*> 15 years of age*	2.5 to < 5 cm in diameter OR 6.25 to < 25 cm^2 surface area AND Symptoms causing no or minimal interference with usual social & functional activities	≥ 5 to < 10 cm in diameter OR ≥ 25 to < 100 cm^2 surface area OR Symptoms causing greater than minimal interference with usual social & functional activities	≥ 10 cm in diameter OR ≥ 100 cm^2 surface area OR Ulceration OR Secondary infection OR Phlebitis OR Sterile abscess OR Drainage OR Symptoms causing inability to perform usual social & functional activities	Potentially lifethreatening consequences (e.g., abscess, exfoliative dermatitis, necrosis involving dermis or deeper tissue)
≤15 years of age	≤ 2.5 cm in diameter	> 2.5 cm in diameter with < 50% surface area of the extremity segment involved (e.g., upper arm or thigh)	≥ 50% surface area of the extremity segment involved (e.g., upper arm or thigh) OR Ulceration OR Secondary infection OR Phlebitis OR Sterile abscess OR Drainage	Potentially life-threatening consequences (e.g., abscess, exfoliative dermatitis, necrosis involving dermis or deeper tissue)
**Injection Site Induration or Swelling**<br>*Report only one*<br>*> 15 years of age*	Same as for **Injection Site Erythema or Redness,** *> 15 years of age*	Same as for **Injection Site Erythema or Redness,** *> 15 years of age*	Same as for **Injection Site Erythema or Redness,** *> 15 years of age*	Same as for **Injection Site Erythema or Redness,** *> 15 years of age*
≤ 15 years of age	Same as for **Injection Site Erythema or Redness,** *≤ 15 years of age*	Same as for **Injection Site Erythema or Redness,** *≤ 15 years of age*	Same as for **Injection Site Erythema or Redness,** *≤ 15 years of age*	Same as for **Injection Site Erythema or Redness,** *≤ 15 years of age*
**Injection Site Pruritus**	Itching localized to the injection site that is relieved spontaneously or in < 48 hours of treatment	Itching beyond the injection site that is not generalized OR Itching localized to the injection site requiring ≥ 48 hours treatment	Generalized itching causing inability to perform usual social & functional activities	NA

13 Injection Site Erythema or Redness should be evaluated and graded using the greatest single diameter or measured surface area.

LABORATORY VALUES

Chemistries

PARAMETER	GRADE 1<br>MILD	GRADE 2<br>MODERATE	GRADE 3<br>SEVERE	GRADE 4<br>POTENTIALLY LIFE-THREATENING
Acidosis	NA	pH ≥ 7.3 to < LLN	pH < 7.3 without life-threatening consequences	pH < 7.3 with life-threatening consequences
Albumin, Low (g/dL; *g/L*)	3.0 to < LLN *30 to < LLN*	≥ 2.0 to < 3.0 ≥ *20 to < 30*	< 2.0<br>*< 20*	NA
Alkaline Phosphatase, High	1.25 to < 2.5 x ULN	2.5 to < 5.0 x ULN	5.0 to < 10.0 x ULN	≥ 10.0 x ULN
Alkalosis	NA	pH > ULN to ≤ 7.5	pH > 7.5 without life-threatening consequences	pH > 7.5 with life-threatening consequences
ALT or SGPT, High *Report only one*	1.25 to < 2.5 x ULN	2.5 to < 5.0 x ULN	5.0 to < 10.0 x ULN	≥ 10.0 x ULN
Amylase (Pancreatic) or Amylase (Total), High *Report only one*	1.1 to < 1.5 x ULN	1.5 to < 3.0 x ULN	3.0 to < 5.0 x ULN	≥ 5.0 x ULN
AST or SGOT, High *Report only one*	1.25 to < 2.5 x ULN	2.5 to < 5.0 x ULN	5.0 to < 10.0 x ULN	≥ 10.0 x ULN
Bicarbonate, Low (mEq/L; *mmol/L*)	16.0 to < LLN *16.0 to < LLN*	11.0 to < 16.0 *11.0 to < 16.0*	8.0 to < 11.0 *8.0 to < 11.0*	< 8.0<br>*< 8.0*
Bilirubin<br>*Direct Bilirubin[14], High*<br>*> 28 days of age*	NA	NA	> ULN	> ULN with lifethreatening consequences (e.g., signs and symptoms of liver failure)
≤ 28 days of age	ULN to ≤ 1 mg/dL	> 1 to ≤ 1.5 mg/dL	> 1.5 to ≤ 2 mg/dL	> 2 mg/dL
Total Bilirubin, High<br>*> 28 days of age*	1.1 to < 1.6 x ULN	1.6 to < 2.6 x ULN	2.6 to < 5.0 x ULN	≥ 5.0 x ULN
≤ 28 days of age	See Appendix A. Total Bilirubin for Term and Preterm Neonates	See Appendix A. Total Bilirubin for Term and Preterm Neonates	See Appendix A. Total Bilirubin for Term and Preterm Neonates	See Appendix A. Total Bilirubin for Term and Preterm Neonates

Calcium, High (mg/dL; *mmol/L*)<br>≥ *7 days of age*	10.6 to < 11.5<br>*2.65* to < *2.88*	11.5 to < 12.5 *2.88* to < *3.13*	12.5 to < 13.5<br>*3.13* to < *3.38*	≥ 13.5<br>≥ *3.38*
< *7 days of age*	11.5 to < 12.4 *2.88* to < *3.10*	12.4 to < 12.9 *3.10* to < *3.23*	12.9 to < 13.5 *3.23* to < *3.38*	≥ 13.5<br>≥ *3.38*

PARAMETER	GRADE 1 MILD	GRADE 2 MODERATE	GRADE 3 SEVERE	GRADE 4 POTENTIALLY LIFE-THREATENING
Calcium (Ionized), High (mg/dL; *mmol/L*)	> ULN to < 6.0 *> ULN to < 1.5*	6.0 to < 6.4 *1.5 to < 1.6*	6.4 to < 7.2 *1.6 to < 1.8*	≥ 7.2<br>*≥ 1.8*
Calcium, Low (mg/dL; *mmol/L*)<br>*≥ 7 days of age*	7.8 to < 8.4 *1.95* to < *2.10*	7.0 to < 7.8<br>*1.75* to < *1.95*	6.1 to < 7.0 *1.53* to < *1.75*	< 6.1<br>*< 1.53*
< 7 days of age	6.5 to < 7.5 *1.63* to < *1.88*	6.0 to < 6.5 *1.50* to < *1.63*	5.50 to < 6.0 *1.38* to < *1.50*	< 5.50<br>*< 1.38*
Calcium (Ionized), Low (mg/dL; *mmol/L*)	< LLN to 4.0 *< LLN to 1.0*	3.6 to < 4.0 *0.9 to < 1.0*	3.2 to < 3.6 *0.8 to < 0.9*	< 3.2<br>*< 0.8*
Cardiac Troponin I, High	NA	NA	NA	Levels consistent with myocardial infarction or unstable angina as defined by the local laboratory
Creatine Kinase, High	3 to < 6 x ULN	6 to < 10 x ULN	10 to < 20 x ULN	≥ 20 x ULN
Creatinine, High	1.1 to 1.3 x ULN	> 1.3 to 1.8 x ULN OR Increase of<br>> 0.3 mg/dL above baseline	> 1.8 to < 3.5 x ULN OR Increase<br>of 1.5 to < 2.0 x above baseline	≥ 3.5 x ULN OR<br>Increase of ≥ 2.0 x above baseline
Creatinine Clearance[15] or eGFR, Low<br>*Report only one*	NA	< 90 to 60 ml/min or ml/min/1.73 m^2 OR<br>10 to < 30% decrease from baseline	< 60 to 30 ml/min or ml/min/1.73 m^2 OR<br>≥ 30 to < 50% decrease from baseline	< 30 ml/min or ml/min/1.73 m2 OR<br>≥ 50% decrease from baseline or dialysis needed
Glucose (mg/dL; *mmol/L*)<br>*Fasting, High*	110 to 125 *6.11 to < 6.95*	> 125 to 250 *6.95 to < 13.89*	> 250 to 500 *13.89 to < 27.75*	> 500<br>*≥ 27.75*
Nonfasting, High	116 to 160 *6.44 to < 8.89*	> 160 to 250 *8.89 to < 13.89*	> 250 to 500 *13.89 to < 27.75*	> 500<br>*≥ 27.75*
Glucose, Low (mg/dL; *mmol/L*)<br>*≥ 1 month of age*	55 to 64 *3.05 to 3.55*	40 to < 55 *2.22 to < 3.05*	30 to < 40 *1.67 to < 2.22*	< 30<br>*< 1.67*
< 1 month of age	50 to 54 *2.78 to 3.00*	40 to < 50 *2.22 to < 2.78*	30 to < 40 *1.67 to < 2.22*	< 30<br>*< 1.67*
Lactate, High	ULN to < 2.0 x ULN without acidosis	≥ 2.0 x ULN without acidosis	Increased lactate with pH < 7.3 without life-threatening consequences	Increased lactate with pH < 7.3 with life-threatening consequences

[15] Use the applicable formula (i.e., Cockroft-Gault in mL/min or Schwatrz in mL/min/1.73m^2).

PARAMETER	GRADE 1 MILD	GRADE 2 MODERATE	GRADE 3 SEVERE	GRADE 4 POTENTIALLY LIFE-THREATENING
Lipase, High	1.1 to < 1.5 x ULN	1.5 to < 3.0 x ULN	3.0 to < 5.0 x ULN	≥ 5.0 x ULN
Lipid Disorders (mg/dL; *mmol/L*)<br>*Cholesterol, Fasting, High*				
≥18 years of age	200 to < 240<br>*5.18 to < 6.19*	240 to < 300<br>*6.19 to < 7.77*	≥ 300<br>*≥ 7.77*	NA
< 18 years of age	170 to < 200<br>*4.40 to < 5.15*	200 to < 300<br>*5.15 to < 7.77*	≥ 300<br>*≥ 7.77*	NA
LDL, Fasting, High<br>*≥18 years of age*	130 to < 160<br>*3.37 to < 4.12*	160 to < 190<br>*4.12 to < 4.90*	≥ 190<br>*≥ 4.90*	NA
> 2 to < 18 years of age	110 to < 130<br>*2.85 to < 3.34*	130 to < 190<br>*3.34 to < 4.90*	≥ 190<br>*≥ 4.90*	NA
Triglycerides, Fasting, High	150 to 300<br>*1.71 to 3.42*	>300 to 500<br>*>3.42 to 5.7*	>500 to < 1,000<br>*>5.7 to 11.4*	> 1,000<br>*> 11.4*
Magnesium[16], Low<br>(mEq/L; *mmol/L*)	1.2 to < 1.4<br>*0.60 to < 0.70*	0.9 to < 1.2<br>*0.45 to < 0.60*	0.6 to < 0.9<br>*0.30 to < 0.45*	< 0.6<br>*< 0.30*
Phosphate, Low (mg/dL; *mmol/L*)<br>*> 14 years of age*	2.0 to < LLN<br>*0.81 to < LLN*	1.4 to < 2.0<br>*0.65 to < 0.81*	1.0 to < 1.4<br>*0.32 to < 0.65*	< 1.0<br>*< 0.32*
1 to 14 years of age	3.0 to < 3.5<br>*0.97 to < 1.13*	2.5 to < 3.0<br>*0.81 to < 0.97*	1.5 to < 2.5<br>*0.48 to < 0.81*	< 1.5<br>*< 0.48*
< 1 year of age	3.5 to < 4.5<br>*1.13 to < 1.45*	2.5 to < 3.5<br>*0.81 to < 1.13*	1.5 to < 2.5<br>*0.48 to < 0.81*	< 1.5<br>*< 0.48*
Potassium, High<br>(mEq/L; *mmol/L*)	5.6 to < 6.0<br>*5.6 to < 6.0*	6.0 to < 6.5<br>*6.0 to < 6.5*	6.5 to < 7.0<br>*6.5 to < 7.0*	≥ 7.0<br>*≥ 7.0*
Potassium, Low<br>(mEq/L; *mmol/L*)	3.0 to < 3.4<br>*3.0 to < 3.4*	2.5 to < 3.0<br>*2.5 to < 3.0*	2.0 to < 2.5<br>*2.0 to < 2.5*	< 2.0<br>*< 2.0*
Sodium, High<br>(mEq/L; *mmol/L*)	146 to < 150<br>*146 to < 150*	150 to < 154<br>*150 to < 154*	154 to < 160<br>*154 to < 160*	≥ 160<br>*≥ 160*
Sodium, Low<br>(mEq/L; *mmol/L*)	130 to < 135<br>*130 to < 135*	125 to < 130<br>*125 to < 135*	121 to < 125<br>*121 to < 125*	≤ 120<br>*≤ 120*
Uric Acid, High<br>(mg/dL; *mmol/L*)	7.5 to < 10.0<br>*0.45 to < 0.59*	10.0 to < 12.0<br>*0.59 to < 0.71*	12.0 to < 15.0<br>*0.71 to < 0.89*	≥ 15.0<br>*≥ 0.89*

16 To convert a magnesium value from mg/dL to mmol/L, laboratories should multiply by 0.4114.

Hematology

PARAMETER	GRADE 1<br>MILD	GRADE 2<br>MODERATE	GRADE 3<br>SEVERE	GRADE 4<br>POTENTIALLY LIFE-THREATENING
Absolute CD4+ Count, Low<br>(cell/mm^3; *cells/L*)<br>*> 5 years of age*<br>*(not HIV infected)*	300 to < 400<br>*300 to < 400*	200 to < 300<br>*200 to < 300*	100 to < 200<br>*100 to < 200*	< 100<br>*< 100*
Absolute Lymphocyte Count, Low<br>(cell/mm^3; *cells/L*)<br>*> 5 years of age*<br>*(not HIV infected)*	600 to < 650<br>*0.600 x 10^9 to*<br>*< 0.650 x 10^9*	500 to < 600<br>*0.500 x 10^9 to*<br>*< 0.600 x 10^9*	350 to < 500<br>*0.350 x 10^9 to*<br>*< 0.500 x 10^9*	< 350<br>*< 0.350 x 10^9*
Absolute Neutrophil Count (ANC), Low<br>(cells/mm^3; *cells/L*)<br>*> 7 days of age*	800 to 1,000<br>*0.800 x 10^9 to 1.000 x 10^9*	600 to 799<br>*0.600 x 10^9 to 0.799 x 10^9*	400 to 599<br>*0.400 x 10^9 to 0.599 x 10^9*	< 400<br>*< 0.400 x 10^9*
2 to 7 days of age	1,250 to 1,500<br>*1.250 x 10^9 to 1.500 x 10^9*	1,000 to 1,249<br>*1.000 x 10^9 to 1.249 x 10^9*	750 to 999<br>*0.750 x 10^9 to 0.999 x 10^9*	< 750<br>*< 0.750 x 10^9*
≤ 1 day of age	4,000 to 5,000<br>*4.000 x 10^9 to*<br>*5.000 x 109*	3,000 to 3,999<br>*3.000 x 10^9 to 3.999 x 10^9*	1,500 to 2,999<br>*1.500 x 10^9 to 2.999 x 10^9*	< 1,500<br>*< 1.500 x 10^9*
Fibrinogen, Decreased<br>(mg/dL; *g/L*)	100 to < 200<br>*1.00 to < 2.00*<br>OR<br>0.75 to < 1.00<br>x LLN	75 to < 100<br>*0.75 to < 1.00*<br>OR<br>≥ 0.50 to < 0.75<br>x LLN	50 to < 75<br>*0.50 to < 0.75*<br>OR<br>0.25 to < 0.50<br>x LLN	< 50<br>*< 0.50*<br>OR<br>< 0.25 x LLN<br>OR Associated with gross bleeding
Hemoglobin[17], Low (g/dL; *mmol/L*)[18]<br>*≥ 13 years of age*<br>*(male only)*	10.0 to 10.9<br>*6.19 to 6.76*	9.0 to < 10.0<br>*5.57 to < 6.19*	7.0 to < 9.0<br>*4.34 to < 5.57*	< 7.0<br>*< 4.34*
≥ 13 years of age<br>*(female only)*	9.5 to 10.4<br>*5.88 to 6.48*	8.5 to < 9.5<br>*5.25 to < 5.88*	6.5 to < 8.5<br>*4.03 to < 5.25*	< 6.5<br>*< 4.03*

[17] Male and female sex are defined as sex at birth.
[18] The conversion factor used to convert g/dL to mmol/L is 0.6206 and is the most commonly used conversion factor. For grading hemoglobin results obtained by an analytic method with a conversion factor other than 0.6206, the result must be converted to g/dL using the appropriate conversion factor for the particular laboratory.

PARAMETER	GRADE 1<br>MILD	GRADE 2<br>MODERATE	GRADE 3<br>SEVERE	GRADE 4<br>POTENTIALLY LIFE-THREATENING
57 days of age to < 13 years of age (male and female)	9.5 to 10.4<br>*5.88 to 6.48*	8.5 to < 9.5<br>*5.25 to < 5.88*	6.5 to < 8.5<br>*4.03 to < 5.25*	< 6.5<br>*< 4.03*
36 to 56 days of age (male and female)	8.5 to 9.6<br>*5.26 to 5.99*	7.0 to < 8.5<br>*4.32 to < 5.26*	6.0 to < 7.0<br>*3.72 to < 4.32*	< 6.0<br>*< 3.72*
22 to 35 days of age (male and female)	9.5 to 11.0<br>*5.88 to 6.86*	8.0 to < 9.5<br>*4.94 to < 5.88*	6.7 to < 8.0<br>*4.15 to < 4.94*	< 6.7<br>*< 4.15*
8 to < 21 days of age (male and female)	11.0 to 13.0<br>*6.81 to 8.10*	9.0 to < 11.0<br>*5.57 to < 6.81*	8.0 to < 9.0<br>*4.96 to < 5.57*	< 8.0<br>*< 4.96*
< 7 days of age (male and female)	13.0 to 14.0<br>*8.05 to 8.72*	10.0 to < 13.0<br>*6.19 to < 8.05*	9.0 to < 10.0<br>*5.59 to < 6.19*	< 9.0<br>*< 5.59*
INR, High (not on anticoagulation therapy)	1.1 to < 1.5 x ULN	1.5 to < 2.0 x ULN	2.0 to < 3.0 x ULN	≥ 3.0 x ULN
Methemoglobin (% hemoglobin)	5.0 to < 10.0%	10.0 to < 15.0%	15.0 to < 20.0%	≥ 20.0%
PTT, High (not on anticoagulation therapy)	1.1 to < 1.66 x ULN	1.66 to < 2.33 x ULN	2.33 to < 3.00 x ULN	≥ 3.00 x ULN
Platelets, Decreased (cells/mm^3; *cells/L*)	100,000 to < 124,999<br>*100.000 x 10^9 to < 124.999 x 10^9*	50,000 to < 100,000<br>*50.000 x 10^9 to < 100.000 x 10^9*	25,000 to < 50,000<br>*25.000 x 10^9 to < 50.000 x 10^9*	< 25,000<br>*< 25.000 x 10^9*
PT, High (not on anticoagulation therapy	1.1 to < 1.25 x ULN	1.25 to < 1.50 x ULN	1.50 to < 3.00 x ULN	≥ 3.00 x ULN
WBC, Decreased (cells/mm^3; *cells/L*)<br>*> 7 days of age*	2,000 to 2,499<br>*2.000 x 10^9 to 2.499 x 10^9*	1,500 to 1,999<br>*1.500 x 10^9 to 1.999 x 10^9*	1,000 to 1,499<br>*1.000 x 10^9 to 1.499 x 10^9*	< 1,000<br>*< 1.000 x 10^9*
< 7 days of age	5,500 to 6,999<br>*5.500 x 10^9 to 6.999 x 10^9*	4,000 to 5,499<br>*4.000 x 10^9 to 5.499 x 10^9*	2,500 to 3,999<br>*2.500 x 10^9 to 3.999 x 10^9*	< 2,500<br>*< 2.500 x 10^9*

Urinalysis

PARAMETER	GRADE 1	GRADE 2	GRADE 3	GRADE 4
Glycosuria (random collection tested by dipstick)	Trace to 1+ or ≤ 250 mg	2+ or > 250 to < 500 mg	> 2+ or > 500 mg	NA
Hematuria (not to be reported based on dipstick findings or on blood believed to be of menstrual origin)	6 to < 10 RBCs per high power field	≥ 10 RBCs per high power field	Gross, with or without clots OR<br>With RBC casts OR<br>Intervention indicated	Life-threatening consequences
Proteinuria (random collection tested by dipstick)	1+	2+	3+ or higher	NA

APPENDIX F CTCAE TABLE

For the first 24 hours after IP infusion, any infusion related reactions, including cytokine release syndrome, will be graded using the Common Terminology Criteria for Adverse Events (CTCAE) v4.03: June 14, 2010 (see SOM for details).

CTCAE4.03
Adapted from Common Terminology Criteria for Adverse Events (CTCAE)
Version 4.0 Published: May 28, 2009 (v4.03: June 14, 2010)

U.S. DEPARTMENT OF HEALTH AND HUMAN SERVICES National Institutes of Health National Cancer Institute

Quick Reference

The NCI Common Terminology Criteria for Adverse Events is a descriptive terminology which can be utilized for Adverse Event (AE) reporting. A grading (severity) scale is provided for each AE term.

Components and Organization

SOC

System Organ Class, the highest level of the MedDRA hierarchy, is identified by anatomical or physiological system, etiology, or purpose (e.g., SOC Investigations for laboratory test results). CTCAE terms are grouped by MedDRA Primary SOCs. Within each SOC, AEs are listed and accompanied by descriptions of severity (Grade).

CTCAE Terms

An Adverse Event (AE) is any unfavorable and unintended sign (including an abnormal laboratory finding), symptom, or disease temporally associated with the use of a medical treatment or procedure that may or may not be considered related to the medical treatment or procedure. An AE is a term that is a unique representation of a specific event used for medical documentation and scientific analyses. Each CTCAE v4.0 term is a MedDRA LLT (Lowest Level Term).

Definitions

A brief definition is provided to clarify the meaning of each AE term.

Grades

Grade refers to the severity of the AE. The CTCAE displays Grades 1 through 5 with unique clinical descriptions of severity for each AE based on this general guideline:

Grade 1	Mild; asymptomatic or mild symptoms; clinical or diagnostic observations only; intervention not indicated.
Grade 2	Moderate; minimal, local or noninvasive intervention indicated; limiting age-appropriate instrumental ADL*.

Grade 3	Severe or medically significant but not immediately life-threatening; hospitalization or prolongation of hospitalization indicated; disabling; limiting self care ADL**.
Grade 4	Life-threatening consequences; urgent intervention indicated.
Grade 5	Death related to AE.

A Semi-colon indicates ‘or’ within the description of the grade.

A single dash (-) indicates a grade is not available.

Not all Grades are appropriate for all AEs. Therefore, some AEs are listed with fewer than five options for Grade selection.

Grade 5

Grade 5 (Death) is not appropriate for some AEs and therefore is not an option.

Activities of Daily Living (ADL)

*Instrumental ADL refer to preparing meals, shopping for groceries or clothes, using the telephone, managing money, etc.

**Self care ADL refer to bathing, dressing and undressing, feeding self, using the toilet, taking medications, and not bedridden.

† CTCAE v4.0 incorporates certain elements of the MedDRA terminology. For further details on MedDRA refer to the MedDRA MSSO Web site (http://www.meddramsso.com).

Blood and lymphatic system disorders	Grade				
Adverse Event	1	2	3	4	5
Anemia	Hemoglobin (Hgb) <LLN - 10.0 g/dL; <LLN - 6.2 mmol/L; <LLN - 100 g/L	Hgb <10.0 - 8.0 g/dL; <6.2 - 4.9 mmol/L; <100 - 80g/L	Hgb <8.0 g/dL; <4.9 mmol/L; <80 g/L; transfusion indicated	Life-threatening consequences; urgent intervention indicated	Death
Bone marrow hypocellular	Mildly hypocellular or <=25% reduction from normal cellularity for age	Moderately hypocellular or >25 - <50% reduction from normal cellularity for age	Severely hypocellular or >50 - <=75% reduction cellularity from normal for age	Aplastic persistent for longer than 2 weeks	Death
Disseminated intravascular coagulation	-	Laboratory findings with no bleeding	Laboratory findings and bleeding	Life-threatening consequences; urgent intervention indicated	Death
Febrile neutropenia	-	-	ANC <1000/mm3 with a single temperature of >38.3 degrees C (101 degrees F) or a sustained temperature of >=38 degrees C (100.4 degrees F) for more than one hour	Life-threatening consequences; urgent intervention indicated	Death
Hemolysis	Laboratory evidence of hemolysis only (e.g., direct antiglobulin test; DAT; Coombs'; schistocytes; decreased haptoglobin)	Evidence of hemolysis and >=2 gm decrease in hemoglobin	Transfusion or medical intervention indicated (e.g., steroids)	Life-threatening consequences; urgent intervention indicated	Death
Hemolytic uremic syndrome	Evidence of RBC destruction (schistocytosis) without clinical consequences	-	Laboratory findings with clinical consequences (e.g., renal insufficiency, petechiae)	Life-threatening consequences, (e.g., CNS hemorrhage or thrombosis/embolism or renal failure)	Death
Leukocytosis	-	-	>100,000/mm3	Clinical manifestations of leucostasis; urgent intervention indicated	Death
Lymph node pain	Mild pain	Moderate pain; limiting instrumental ADL	Severe pain; limiting self care ADL	-	
Spleen disorder	Incidental findings (e.g., Howell-Jolly bodies); mild degree of thrombocytosis and leukocytosis	Prophylactic antibiotics indicated	-	Life-threatening consequences; urgent intervention indicated	Death
Thrombotic thrombocytopenic purpura	Evidence of RBC destruction (schistocytosis) without clinical consequences	-	Laboratory findings with clinical consequences (e.g., renal insufficiency, petechiae)	Life-threatening consequences, (e.g., CNS hemorrhage or thrombosis/embolism or renal failure)	Death

Blood and lymphatic system disorders - Other, specify	Asymptomatic or mild symptoms; clinical or diagnostic observations only; intervention not indicated	Moderate; minimal, local or noninvasive intervention indicated; limiting age-appropriate instrumental ADL	Severe or medically significant but not immediately life-threatening; hospitalization or prolongation of existing hospitalization indicated; disabling; limiting self care ADL	Life-threatening consequences; urgent intervention indicated	Death
Cardiac Disorders	Grade				
Adverse Event	1	2	3	4	5
Acute coronary syndrome	-	Symptomatic, progressive angina; cardiac enzymes normal; hemodynamically stable	Symptomatic, unstable angina and/or acute myocardial infarction, cardiac enzymes abnormal, hemodynamically stable	Symptomatic, unstable angina and/or acute myocardial infarction, cardiac enzymes abnormal, hemodynamically unstable	Death
Aortic valve disease	Asymptomatic valvular thickening with or without mild valvular regurgitation or stenosis by imaging	Asymptomatic; moderate regurgitation or stenosis by imaging	Symptomatic; severe regurgitation or stenosis by imaging; symptoms controlled with medical intervention	Life-threatening consequences; urgent intervention indicated (e.g., valve replacement, valvuloplasty)	Death
Asystole	Periods of asystole; non-urgent medical management indicated	-	-	Life-threatening consequences; urgent intervention indicated	Death
Atrial fibrillation	Asymptomatic, intervention not indicated	Non-urgent medical intervention indicated	Symptomatic and incompletely controlled medically, or controlled with device (e.g., pacemaker), or ablation	Life-threatening consequences; urgent intervention indicated	Death
Atrial flutter	Asymptomatic, intervention not indicated	Non-urgent medical intervention indicated	Symptomatic and incompletely controlled medically, or controlled with device (e.g., pacemaker), or ablation	Life-threatening consequences; urgent intervention indicated	Death
Atrioventricular block complete	-	Non-urgent intervention indicated	Symptomatic and incompletely controlled medically, or controlled with device (e.g., pacemaker)	Life-threatening consequences; urgent intervention indicated	Death
Atrioventricular block first degree	Asymptomatic, intervention not indicated	Non-urgent intervention indicated	-	-	-
Cardiac arrest	-	-	-	Life-threatening consequences; urgent intervention indicated	Death
Chest pain - cardiac	Mild pain	Moderate pain; limiting instrumental ADL	Pain at rest; limiting self care ADL	-	-
Conduction disorder	Mild symptoms; intervention not indicated	Moderate symptoms	Severe symptoms; intervention indicated	Life-threatening consequences; urgent intervention indicated	Death
Constrictive pericarditis	-	-	Symptomatic heart failure or other cardiac symptoms, responsive to intervention	Refractory heart failure or other poorly controlled cardiac symptoms	Death

Heart failure	Asymptomatic with laboratory (e.g., BNP [B-Natriuretic Peptide]) or cardiac imaging abnormalities	Symptoms with mild to moderate activity or exertion	Severe with symptoms at rest or with minimal activity or exertion; intervention indicated	Life-threatening consequences; urgent intervention indicated (e.g., continuous IV therapy or mechanical hemodynamic support)	Death
Left ventricular systolic dysfunction	-	-	Symptomatic due to drop in ejection fraction responsive to intervention	Refractory or poorly controlled heart failure due to drop in ejection fraction; intervention such as ventricular assist device, intravenous vasopressor support, or heart transplant indicated	Death
Mitral valve disease	Asymptomatic valvular thickening with or without mild valvular regurgitation or stenosis by imaging	Asymptomatic; moderate regurgitation or stenosis by imaging	Symptomatic; severe regurgitation or stenosis by imaging; symptoms controlled with medical intervention	Life-threatening consequences; urgent intervention indicated (e.g., valve replacement, valvuloplasty)	Death
Mobitz (type) II atrioventricular block	Asymptomatic, intervention not indicated	Symptomatic; medical intervention indicated	Symptomatic and incompletely controlled medically, or controlled with device (e.g., pacemaker)	Life-threatening consequences; urgent intervention indicated	Death
Mobitz type I	Asymptomatic, intervention not indicated	Symptomatic; medical intervention indicated	Symptomatic and incompletely controlled medically, or controlled with device (e.g., pacemaker)	Life-threatening consequences; urgent intervention indicated	Death
Myocardial infarction	-	Asymptomatic and cardiac enzymes minimally abnormal and no evidence of ischemic ECG changes	Severe symptoms; cardiac enzymes abnormal; hemodynamically stable; ECG changes consistent with infarction	Life-threatening consequences; hemodynamically unstable	Death
Myocarditis	Asymptomatic with laboratory (e.g., BNP [B-Natriuretic Peptide]) or cardiac imaging abnormalities	Symptoms with mild to moderate activity or exertion	Severe with symptoms at rest or with minimal activity or exertion; intervention indicated	Life-threatening consequences; urgent intervention indicated (e.g., continuous IV therapy or mechanical hemodynamic support)	Death
Palpitations	Mild symptoms; intervention not indicated	Intervention indicated	-	-	-
Paroxysmal atrial tachycardia	Asymptomatic, intervention not indicated	Symptomatic; medical management indicated	IV medication indicated	Life-threatening consequences; incompletely controlled medically; cardioversion indicated	Death
Pericardial effusion	-	Asymptomatic effusion size small to moderate	Effusion with physiologic consequences	Life-threatening consequences; urgent intervention indicated	Death
Pericardial tamponade	-	-	-	Life-threatening consequences; urgent intervention indicated	Death
Pericarditis	Asymptomatic, ECG or physical findings (e.g., rub) consistent with pericarditis	Symptomatic pericarditis (e.g., chest pain)	Pericarditis with physiologic consequences (e.g., pericardial constriction)	Life-threatening consequences; urgent intervention indicated	Death

Pulmonary valve disease	Asymptomatic valvular thickening with or without mild valvular regurgitation or stenosis by imaging	Asymptomatic; moderate regurgitation or stenosis by imaging	Symptomatic; severe regurgitation or stenosis by imaging; symptoms controlled with medical intervention	Life-threatening consequences; urgent intervention indicated (e.g., valve replacement, valvuloplasty)	Death
Restrictive cardiomyopathy	-	-	Symptomatic heart failure or other cardiac symptoms, responsive to intervention	Refractory heart failure or other poorly controlled cardiac symptoms	Death
Right ventricular dysfunction	Asymptomatic with laboratory (e.g., BNP [B-Natriuretic Peptide]) or cardiac imaging abnormalities	Symptoms with mild to moderate activity or exertion	Severe symptoms, associated with hypoxemia, right heart failure; oxygen indicated	Life-threatening consequences; urgent intervention indicated (e.g., ventricular assist device); heart transplant indicated	Death
Sick sinus syndrome	Asymptomatic, intervention not indicated	Non-urgent intervention indicated	Severe, medically significant; medical intervention indicated	Life-threatening consequences; urgent intervention indicated	Death
Sinus bradycardia	Asymptomatic, intervention not indicated	Symptomatic, medical intervention indicated	Severe, medically significant, medical intervention indicated	Life-threatening consequences; urgent intervention indicated	Death
Sinus tachycardia	Asymptomatic, intervention not indicated	Symptomatic; non-urgent medical intervention indicated	Urgent medical intervention indicated	-	-
Supraventricular tachycardia	Asymptomatic, intervention not indicated	Non-urgent medical intervention indicated	Medical intervention indicated	Life-threatening consequences; urgent intervention indicated	Death
Tricuspid valve disease	Asymptomatic valvular thickening with or without mild valvular regurgitation or stenosis	Asymptomatic; moderate regurgitation or stenosis by imaging	Symptomatic; severe regurgitation or stenosis; symptoms controlled with medical intervention	Life-threatening consequences; urgent intervention indicated (e.g., valve replacement, valvuloplasty)	Death
Ventricular arrhythmia	Asymptomatic, intervention not indicated	Non-urgent medical intervention indicated	Medical intervention indicated	Life-threatening consequences; hemodynamic compromise; urgent intervention indicated	Death
Ventricular fibrillation	-	-	-	Life-threatening consequences; hemodynamic compromise; urgent intervention indicated	Death
Ventricular tachycardia	-	Non-urgent medical intervention indicated	Medical intervention indicated	Life-threatening consequences; hemodynamic compromise; urgent intervention indicated	Death
Wolff-Parkinson-White syndrome	Asymptomatic, intervention not indicated	Non-urgent medical intervention indicated	Symptomatic and incompletely controlled medically or controlled with procedure	Life-threatening consequences; urgent intervention indicated	Death
Cardiac disorders - Other, specify	Asymptomatic or mild symptoms; clinical or diagnostic observations only; intervention not indicated	Moderate; minimal, local or noninvasive intervention indicated; limiting age-appropriate instrumental ADL	Severe or medically significant but not immediately life-threatening; hospitalization or prolongation of existing hospitalization indicated; disabling; limiting self care ADL	Life-threatening consequences; urgent intervention indicated	Death
Congenital, familial and	Grade				

genetic disorders					
Adverse Event	1	2	3	4	5
Congenital, familial and genetic disorders - Other, specify	Asymptomatic or mild symptoms; clinical or diagnostic observations only; intervention not indicated	Moderate; minimal, local or noninvasive intervention indicated; limiting age-appropriate instrumental ADL	Severe or medically significant but not immediately life-threatening; hospitalization or prolongation of existing hospitalization indicated; disabling; limiting self care ADL	Life-threatening consequences; urgent intervention indicated	Death
Ear and labyrinth disorders	Grade				
Adverse Event	1	2	3	4	5
Ear pain	Mild pain	Moderate pain; limiting instrumental ADL	Severe pain; limiting self care ADL	-	-
External ear inflammation	External otitis with erythema or dry desquamation	External otitis with moist desquamation, edema, enhanced cerumen or discharge; tympanic membrane perforation; tympanostomy	External otitis with mastoiditis; stenosis or osteomyelitis; necrosis of soft tissue or bone	Urgent operative intervention indicated	Death
External ear pain	Mild pain	Moderate pain; limiting instrumental ADL	Severe pain; limiting self care ADL	-	-
Hearing impaired	Adults Enrolled on a Monitoring Program (on a 1, 2, 4, 3, 6 and 8 kHz audiogram): Threshold shift of 15 - 25 dB averaged at 2 contiguous test frequencies in at least one ear or subjective change in the absence of a Grade 1 Threshold shift. Pediatric (on a 1, 2, 4, 3, 6 and 8 kHz audiogram): Threshold shift >20 dB at 8 kHz in at least one ear.	Adult enrolled in monitoring program (on a 1, 2, 3, 4, 6 and 8 kHz audiogram): Threshold shift of >25 dB averaged at 2 contiguous test frequencies in at least one ear. Adult not enrolled in monitoring program: hearing loss but hearing aid or intervention not indicated; limiting instrumental ADL. Pediatric (on a 1, 2, 3, 4, 6 and 8 kHz audiogram): Threshold shift >20 dB at 4 kHz and above in at least one ear.	Adult enrolled in monitoring program (on a 1, 2, 3, 4, 6 and 8 kHz audiogram): Threshold shift of >25 dB averaged at 3 contiguous test frequencies in at least one ear; therapeutic intervention indicated. Adult: Not enrolled in monitoring program: Hearing loss with hearing aid or intervention indicated; limiting self care ADL. Pediatric (on a 1, 2, 3, 4, 6 and 8kHz audiogram): hearing loss sufficient to indicate therapeutic intervention, including hearing aids): Threshold shift >20 dB at 3 kHz and above in at least one ear ; additional speech-language related services indicated.	Adults: Profound bilateral hearing loss (Threshold >80 dB HL at 2 kHz and above); nonservicable hearing Pediatric: Audiologic indication for cochlear implant and additional speech-language related services indicated.	-
Middle ear inflammation	Serous otitis	Serous otitis, medical intervention indicated	Mastoiditis; necrosis of canal soft tissue or bone	Life-threatening consequences; urgent intervention indicated	Death
Tinnitus	Mild symptoms; intervention not indicated	Moderate symptoms; limiting instrumental ADL	Severe symptoms; limiting self care ADL	-	-

Vertigo	Mild symptoms	Moderate symptoms; limiting instrumental ADL	Severe symptoms; limiting self care ADL	-	-
Vestibular disorder	-	Symptomatic; limiting instrumental ADL	Severe symptoms; limiting self care ADL	-	-
Ear and labyrinth disorders - Other, specify	Asymptomatic or mild symptoms; clinical or diagnostic observations only; intervention not indicated	Moderate; minimal, local or noninvasive intervention indicated; limiting age-appropriate instrumental ADL	Severe or medically significant but not immediately life-threatening; hospitalization or prolongation of existing hospitalization indicated; disabling; limiting self care ADL	Life-threatening consequences; urgent intervention indicated	Death
Endocrine disorders			Grade		
Adverse Event	1	2	3	4	5
Adrenal insufficiency	Asymptomatic; clinical or diagnostic observations only; intervention not indicated	Moderate symptoms; medical intervention indicated	Severe symptoms; hospitalization indicated	Life-threatening consequences; urgent intervention indicated	Death
Cushingoid	Mild symptoms; intervention not indicated	Moderate symptoms; medical intervention indicated	Severe symptoms, medical intervention or hospitalization indicated	-	-
Delayed puberty	-	No breast development by age 13 yrs for females; testes volume of <3 cc or no Tanner Stage 2 development by age 14.5 yrs for males	No breast development by age 14 yrs for females; no increase in testes volume or no Tanner Stage 2 by age 16 yrs for males; hormone replacement indicated	-	-
Growth accelerated	-	>= +2 SD (standard deviation) above mid parental height or target height	-	-	-
Hyperparathyroidism	Mild symptoms; intervention not indicated	Moderate symptoms; medical intervention indicated	-	-	-
Hyperthyroidism	Asymptomatic; clinical or diagnostic observations only; intervention not indicated	Symptomatic; thyroid suppression therapy indicated; limiting instrumental ADL	Severe symptoms; limiting self care ADL; hospitalization indicated	Life-threatening consequences; urgent intervention indicated	Death
Hypoparathyroidism	Asymptomatic; clinical or diagnostic observations only; intervention not indicated	Moderate symptoms; medical intervention indicated	Severe symptoms; medical intervention or hospitalization indicated	Life-threatening consequences; urgent intervention indicated	Death
Hypothyroidism	Asymptomatic; clinical or diagnostic observations only; intervention not indicated	Symptomatic; thyroid replacement indicated; limiting instrumental ADL	Severe symptoms; limiting self care ADL; hospitalization indicated	Life-threatening consequences; urgent intervention indicated	Death
Precocious puberty	Physical signs of puberty with no biochemical markers for females <8 years and males <9 years	Physical signs and biochemical markers of puberty for females <8 years and males <9 years	-	-	-

Virilization	Mild symptoms; intervention not indicated	Moderate symptoms; medical intervention indicated	-	-	-
Endocrine disorders - Other, specify	Asymptomatic or mild symptoms; clinical or diagnostic observations only; intervention not indicated	Moderate; minimal, local or noninvasive intervention indicated; limiting age-appropriate instrumental ADL	Severe or medically significant but not immediately life-threatening; hospitalization or prolongation of existing hospitalization indicated; disabling; limiting self care ADL	Life-threatening consequences; urgent intervention indicated	Death
Eye Disorders	Grade				
Adverse Event	1	2	3	4	5
Blurred vision	Intervention not indicated	Symptomatic; limiting instrumental ADL	Limiting self care ADL	-	-
Cataract	Asymptomatic; clinical or diagnostic observations only; intervention not indicated	Symptomatic; moderate decrease in visual acuity (20/40 or better)	Symptomatic with marked decrease in visual acuity (worse than 20/40 but better than 20/200); operative intervention indicated (e.g., cataract surgery)	Blindness (20/200 or worse) in the affected eye	-
Conjunctivitis	Asymptomatic or mild symptoms; intervention not indicated	Symptomatic; topical intervention indicated (e.g., antibiotics); limiting instrumental ADL	Limiting self care ADL	-	-
Corneal ulcer	-	Symptomatic; medical intervention indicated (e.g., topical agents); limiting instrumental ADL	Limiting self care ADL; declining vision (worse than 20/40 but better than 20/200)	Perforation or blindness (20/200 or worse) in the affected eye	-
Dry eye	Asymptomatic; clinical or diagnostic observations only; mild symptoms relieved by lubricants	Symptomatic; multiple agents indicated; limiting instrumental ADL	Decrease in visual acuity (<20/40); limiting self care ADL	-	-
Extraocular muscle paresis	Asymptomatic; clinical or diagnostic observations only	Symptomatic; limiting instrumental ADL	Limiting self care ADL; disabling	-	-
Eye pain	Mild pain	Moderate pain; limiting instrumental ADL	Severe pain; limiting self care ADL	-	-
Eyelid function disorder	Asymptomatic; clinical or diagnostic observations only; intervention not indicated	Symptomatic; nonoperative intervention indicated; limiting instrumental ADL	Limiting self care ADL; operative intervention indicated	-	-
Flashing lights	Symptomatic but not limiting ADL	Limiting instrumental ADL	Limiting self care ADL	-	-
Floaters	Symptomatic but not limiting ADL	Limiting instrumental ADL	Limiting self care ADL	-	-

Glaucoma	Elevated intraocular pressure (EIOP) with single topical agent for intervention; no visual field deficit	EIOP causing early visual field deficits; multiple topical or oral agents indicated; limiting instrumental ADL	EIOP causing marked visual field deficits (e.g., involving both superior and inferior visual fields); operative intervention indicated; limiting self care ADL	Blindness (20/200 or worse) in the affected eye	-
Keratitis	-	Symptomatic; medical intervention indicated (e.g., topical agents); limiting instrumental ADL	Decline in vision (worse than 20/40 but better than 20/200); limiting self care ADL	Perforation or blindness (20/200 or worse) in the affected eye	-
Night blindness	Symptomatic but not limiting ADL	Limiting instrumental ADL	Limiting self care ADL	Blindness (20/200 or worse) in the affected eye	-
Optic nerve disorder	Asymptomatic; clinical or diagnostic observations only	Limiting vision of the affected eye (20/40 or better)	Limiting vision in the affected eye (worse than 20/40 but better than 20/200)	Blindness (20/200 or worse) in the affected eye	-
Papilledema	Asymptomatic; no visual field defects	Symptomatic decline in vision; visual field defect present sparing the central 20 degrees	Marked visual field defect (worse than 20/40 but better than 20/200)	Blindness (20/200 or worse) in the affected eye	-
Photophobia	Symptomatic but not limiting ADL	Limiting instrumental ADL	Limiting self care ADL	-	-
Retinal detachment	Asymptomatic	Exudative and visual acuity 20/40 or better	Rhegmatogenous or exudative detachment; operative intervention indicated; decline in vision (worse than 20/40 but better than 20/200)	Blindness (20/200 or worse) in the affected eye	-
Retinal tear	-	Laser therapy or pneumopexy indicated	Vitroretinal surgical repair indicated	Blindness (20/200 or worse) in the affected eye	-
Retinal vascular disorder	-	Topical medication indicated	Intravitreal medication; operative intervention indicated	-	-
Retinopathy	Asymptomatic; clinical or diagnostic observations only	Symptomatic with moderate decrease in visual acuity (20/40 or better); limiting instrumental ADL	Symptomatic with marked decrease in visual acuity (worse than 20/40); disabling; limiting self care ADL	Blindness (20/200 or worse) in the affected eye	-
Scleral disorder	Asymptomatic; clinical or diagnostic observations only	Symptomatic, limiting instrumental ADL; moderate decrease in visual acuity (20/40 or better)	Symptomatic, limiting self care ADL; marked decrease in visual acuity (worse than 20/40)	Blindness (20/200 or worse) in the affected eye	-
Uveitis	Asymptomatic; clinical or diagnostic observations only	Anterior uveitis; medical intervention indicated	Posterior or pan-uveitis	Blindness (20/200 or worse) in the affected eye	-
Vitreous hemorrhage	Asymptomatic or mild symptoms; clinical or diagnostic observations only	Symptomatic; limiting instrumental ADL	Limiting self care ADL; vitrectomy indicated	Blindness (20/200 or worse) in the affected eye	-
Watering eyes	Intervention not indicated	Intervention indicated	Operative intervention indicated	-	-

Eye disorders - Other, specify	Asymptomatic or mild symptoms; clinical or diagnostic observations only; intervention not indicated	Moderate; minimal, local or noninvasive intervention indicated; limiting age-appropriate instrumental ADL	Severe or medically significant but not immediately sight-threatening; hospitalization or prolongation of existing hospitalization indicated; disabling; limiting self care ADL	Sight-threatening consequences; urgent intervention indicated; blindness (20/200 or worse) in the affected eye	-
Gastrointestinal disorders	Grade				
Adverse Event	1	2	3	4	5
Abdominal distension	Asymptomatic; clinical or diagnostic observations only; intervention not indicated	Symptomatic; limiting instrumental ADL	Severe discomfort; limiting self care ADL	-	-
Abdominal pain	Mild pain	Moderate pain; limiting instrumental ADL	Severe pain; limiting self care ADL	-	-
Anal fistula	Asymptomatic; clinical or diagnostic observations only; intervention not indicated	Symptomatic; altered GI function	Severely altered GI function; tube feeding, TPN or hospitalization indicated; elective operative intervention indicated	Life-threatening consequences; urgent intervention indicated	Death
Anal hemorrhage	Mild; intervention not indicated	Moderate symptoms; medical intervention or minor cauterization indicated	Transfusion, radiologic, endoscopic, or elective operative intervention indicated	Life-threatening consequences; urgent intervention indicated	Death
Anal mucositis	Asymptomatic or mild symptoms; intervention not indicated	Symptomatic; medical intervention indicated; limiting instrumental ADL	Severe symptoms; limiting self care ADL	Life-threatening consequences; urgent intervention indicated	Death
Anal necrosis	-	-	TPN or hospitalization indicated; radiologic, endoscopic, or operative intervention indicated	Life-threatening consequences; urgent operative intervention indicated	Death
Anal pain	Mild pain	Moderate pain; limiting instrumental ADL	Severe pain; limiting self care ADL	-	-
Anal stenosis	Asymptomatic; clinical or diagnostic observations only; intervention not indicated	Symptomatic; altered GI function	Symptomatic and severely altered GI function; non-emergent operative intervention indicated; TPN or hospitalization indicated	Life-threatening consequences; urgent operative intervention indicated	Death
Anal ulcer	Asymptomatic; clinical or diagnostic observations only; intervention not indicated	Symptomatic; altered GI function	Severely altered GI function; TPN indicated; elective operative or endoscopic intervention indicated; disabling	Life-threatening consequences; urgent operative intervention indicated	Death
Ascites	Asymptomatic; clinical or diagnostic observations only; intervention not indicated	Symptomatic; medical intervention indicated	Severe symptoms; invasive intervention indicated	Life-threatening consequences; urgent operative intervention indicated	Death
Bloating	No change in bowel function or oral intake	Symptomatic, decreased oral intake; change in bowel function	-	-	-

Cecal hemorrhage	Mild; intervention not indicated	Moderate symptoms; medical intervention or minor cauterization indicated	Transfusion, radiologic, endoscopic, or elective operative intervention indicated	Life-threatening consequences; urgent intervention indicated	Death
Cheilitis	Asymptomatic; clinical or diagnostic observations only; intervention not indicated	Moderate symptoms; limiting instrumental ADL	Severe symptoms; limiting self care ADL; intervention indicated	-	-
Colitis	Asymptomatic; clinical or diagnostic observations only; intervention not indicated	Abdominal pain; mucus or blood in stool	Severe abdominal pain; change in bowel habits; medical intervention indicated; peritoneal signs	Life-threatening consequences; urgent intervention indicated	Death
Colonic fistula	Asymptomatic; clinical or diagnostic observations only; intervention not indicated	Symptomatic; altered GI function	Severely altered GI function; bowel rest, TPN or hospitalization indicated; elective operative intervention indicated	Life-threatening consequences; urgent intervention indicated	Death
Colonic hemorrhage	Mild; intervention not indicated	Moderate symptoms; medical intervention or minor cauterization indicated	Transfusion, radiologic, endoscopic, or elective operative intervention indicated	Life-threatening consequences; urgent intervention indicated	Death
Colonic obstruction	Asymptomatic; clinical or diagnostic observations only; intervention not indicated	Symptomatic; altered GI function	Hospitalization indicated; elective operative intervention indicated; disabling	Life-threatening consequences; urgent operative intervention indicated	Death
Colonic perforation	-	Symptomatic; medical intervention indicated	Severe symptoms; elective operative intervention indicated	Life-threatening consequences; urgent intervention indicated	Death
Colonic stenosis	Asymptomatic; clinical or diagnostic observations only; intervention not indicated	Symptomatic; altered GI function	Severely altered GI function; tube feeding or hospitalization indicated; elective operative intervention indicated	Life-threatening consequences; urgent operative intervention indicated	Death
Colonic ulcer	Asymptomatic; clinical or diagnostic observations only; intervention not indicated	Symptomatic; altered GI function	Severely altered GI function; TPN indicated; elective operative or endoscopic intervention indicated; disabling	Life-threatening consequences; urgent operative intervention indicated	Death
Constipation	Occasional or intermittent symptoms; occasional use of stool softeners, laxatives, dietary modification, or enema	Persistent symptoms with regular use of laxatives or enemas; limiting instrumental ADL	Obstipation with manual evacuation indicated; limiting self care ADL	Life-threatening consequences; urgent intervention indicated	Death
Dental caries	One or more dental caries, not involving the root	Dental caries involving the root	Dental caries resulting in pulpitis or periapical abscess or resulting in tooth loss	-	-
Diarrhea	Increase of <4 stools per day over baseline; mild increase in ostomy output compared to baseline	Increase of 4 - 6 stools per day over baseline; moderate increase in ostomy output compared to baseline	Increase of >=7 stools per day over baseline; incontinence; hospitalization indicated; severe increase in ostomy output compared to baseline; limiting self care ADL	Life-threatening consequences; urgent intervention indicated	Death

Dry mouth	Symptomatic (e.g., dry or thick saliva) without significant dietary alteration; unstimulated saliva flow >0.2 ml/min	Moderate symptoms; oral intake alterations (e.g., copious water, other lubricants, diet limited to purees and/or soft, moist foods); unstimulated saliva 0.1 to 0.2 ml/min	Inability to adequately aliment orally; tube feeding or TPN indicated; unstimulated saliva <0.1 ml/min	-	-
Duodenal fistula	Asymptomatic; clinical or diagnostic observations only; intervention not indicated	Symptomatic; altered GI function	Severely altered GI function; tube feeding, TPN or hospitalization indicated; elective operative intervention indicated	Life-threatening consequences; urgent intervention indicated	Death
Duodenal hemorrhage	Mild; intervention not indicated	Moderate symptoms; medical intervention or minor cauterization indicated	Transfusion, radiologic, endoscopic, or elective operative intervention indicated	Life-threatening consequences; urgent intervention indicated	Death
Duodenal obstruction	Asymptomatic; clinical or diagnostic observations only; intervention not indicated	Symptomatic; altered GI function	Hospitalization or elective operative intervention indicated; disabling	Life-threatening consequences; urgent operative intervention indicated	Death
Duodenal perforation	-	Symptomatic; medical intervention indicated	Severe symptoms; elective operative intervention indicated	Life-threatening consequences; urgent operative intervention indicated	Death
Duodenal stenosis	Asymptomatic; clinical or diagnostic observations only; intervention not indicated	Symptomatic; altered GI function	Severely altered GI function; tube feeding; hospitalization indicated; elective operative intervention indicated	Life-threatening consequences; urgent operative intervention indicated	Death
Duodenal ulcer	Asymptomatic; clinical or diagnostic observations only; intervention not indicated	Moderate symptoms; medical intervention indicated; limiting instrumental ADL	Severely altered GI function; TPN indicated; elective operative or endoscopic intervention indicated; limiting self care ADL; disabling	Life-threatening consequences; urgent operative intervention indicated	Death
Dyspepsia	Mild symptoms; intervention not indicated	Moderate symptoms; medical intervention indicated	Severe symptoms; surgical intervention indicated	-	-
Dysphagia	Symptomatic, able to eat regular diet	Symptomatic and altered eating/swallowing	Severely altered eating/swallowing; tube feeding or TPN or hospitalization indicated	Life-threatening consequences; urgent intervention indicated	Death
Enterocolitis	Asymptomatic; clinical or diagnostic observations only; intervention not indicated	Abdominal pain; mucus or blood in stool	Severe or persistent abdominal pain; fever; ileus; peritoneal signs	Life-threatening consequences; urgent intervention indicated	Death
Enterovesical fistula	Asymptomatic; clinical or diagnostic observations only; intervention not indicated	Symptomatic; noninvasive intervention indicated	Severe, medically significant; medical intervention indicated	Life-threatening consequences; urgent intervention indicated	Death

Esophageal fistula	Asymptomatic; clinical or diagnostic observations only; intervention not indicated	Symptomatic; altered GI function	Severely altered GI function; tube feeding, TPN or hospitalization indicated; elective operative intervention indicated	Life-threatening consequences; urgent intervention indicated	Death
Esophageal hemorrhage	Mild; intervention not indicated	Moderate symptoms; medical intervention or minor cauterization indicated	Transfusion, radiologic, endoscopic, or elective operative intervention indicated	Life-threatening consequences; urgent intervention indicated	Death
Esophageal necrosis	-	-	Inability to aliment adequately by GI tract; radiologic, endoscopic, or operative intervention indicated	Life-threatening consequences; urgent operative intervention indicated	Death
Esophageal obstruction	Asymptomatic; clinical or diagnostic observations only; intervention not indicated	Symptomatic; altered GI function; limiting instrumental ADL	Hospitalization indicated; elective operative intervention indicated; limiting self care ADL; disabling	Life-threatening consequences; urgent intervention indicated	Death
Esophageal pain	Mild pain	Moderate pain; limiting instrumental ADL	Severe pain; limiting self care ADL	-	-
Esophageal perforation	-	Symptomatic; medical intervention indicated	Severe symptoms; elective operative intervention indicated	Life-threatening consequences; urgent operative intervention indicated	Death
Esophageal stenosis	Asymptomatic; clinical or diagnostic observations only; intervention not indicated	Symptomatic; altered GI function	Severely altered GI function; tube feeding; hospitalization indicated; elective operative intervention indicated	Life-threatening consequences; urgent operative intervention indicated	Death
Esophageal ulcer	Asymptomatic; clinical or diagnostic observations only; intervention not indicated	Symptomatic; altered GI function; limiting instrumental ADL	Severely altered GI function; TPN indicated; elective operative or endoscopic intervention indicated; limiting self care ADL; disabling	Life-threatening consequences; urgent operative intervention indicated	Death
Esophageal varices hemorrhage	-	Self-limited; intervention not indicated	Transfusion, radiologic, endoscopic, or elective operative intervention indicated	Life-threatening consequences; urgent intervention indicated	Death
Esophagitis	Asymptomatic; clinical or diagnostic observations only; intervention not indicated	Symptomatic; altered eating/swallowing; oral supplements indicated	Severely altered eating/swallowing; tube feeding, TPN or hospitalization indicated	Life-threatening consequences; urgent operative intervention indicated	Death
Fecal incontinence	Occasional use of pads required	Daily use of pads required	Severe symptoms; elective operative intervention indicated	-	-
Flatulence	Mild symptoms; intervention not indicated	Moderate; persistent; psychosocial sequelae	-	-	-
Gastric fistula	Asymptomatic; clinical or diagnostic observations only; intervention not indicated	Symptomatic; altered GI function	Severely altered GI function; bowel rest; tube feeding, TPN or hospitalization indicated; elective operative intervention indicated	Life-threatening consequences; urgent operative intervention indicated	Death

Gastric hemorrhage	Mild; intervention not indicated	Moderate symptoms; medical intervention or minor cauterization indicated	Transfusion, radiologic, endoscopic, or elective operative intervention indicated	Life-threatening consequences; urgent intervention indicated	Death
Gastric necrosis	-	-	Inability to aliment adequately by GI tract; radiologic, endoscopic, or operative intervention indicated	Life-threatening consequences; urgent operative intervention indicated	Death
Gastric perforation	-	Symptomatic; medical intervention indicated	Severe symptoms; elective operative intervention indicated	Life-threatening consequences; urgent operative intervention indicated	Death
Gastric stenosis	Asymptomatic; clinical or diagnostic observations only; intervention not indicated	Symptomatic; altered GI function	Severely altered GI function; tube feeding; hospitalization indicated; elective operative intervention indicated	Life-threatening consequences; urgent operative intervention indicated	Death
Gastric ulcer	Asymptomatic; clinical or diagnostic observations only; intervention not indicated	Symptomatic; altered GI function; medical intervention indicated; limiting instrumental ADL	Severely altered GI function; TPN indicated; elective operative or endoscopic intervention indicated; limiting self care ADL; disabling	Life-threatening consequences; urgent operative intervention indicated	Death
Gastritis	Asymptomatic; clinical or diagnostic observations only; intervention not indicated	Symptomatic; altered GI function; medical intervention indicated	Severely altered eating or gastric function; TPN or hospitalization indicated	Life-threatening consequences; urgent operative intervention indicated	Death
Gastroesophageal reflux disease	Mild symptoms; intervention not indicated	Moderate symptoms; medical intervention indicated	Severe symptoms; surgical intervention indicated	-	-
Gastrointestinal fistula	Asymptomatic; clinical or diagnostic observations only; intervention not indicated	Symptomatic; altered GI function	Severely altered GI function; tube feeding, TPN or hospitalization indicated	Life-threatening consequences; urgent operative intervention indicated	Death
Gastrointestinal pain	Mild pain	Moderate pain; limiting instrumental ADL	Severe pain; limiting self care ADL	-	-
Gastroparesis	Mild nausea, early satiety and bloating, able to maintain caloric intake on regular diet	Moderate symptoms; able to maintain nutrition with dietary and lifestyle modifications; may need pharmacologic intervention	Weight loss; refractory to medical intervention; unable to maintain nutrition orally	-	-
Gingival pain	Mild pain	Moderate pain interfering with oral intake	Severe pain; inability to aliment orally	-	-
Hemorrhoidal hemorrhage	Mild; intervention not indicated	Moderate symptoms; medical intervention or minor cauterization indicated	Transfusion, radiologic, endoscopic, or elective operative intervention indicated	Life-threatening consequences; urgent intervention indicated	Death

Hemorrhoids	Asymptomatic; clinical or diagnostic observations only; intervention not indicated	Symptomatic; banding or medical intervention indicated	Severe symptoms; radiologic, endoscopic or elective operative intervention indicated	-	-
Ileal fistula	Asymptomatic; clinical or diagnostic observations only; intervention not indicated	Symptomatic; altered GI function	Severely altered GI function; TPN or hospitalization indicated; elective operative intervention indicated	Life-threatening consequences; urgent intervention indicated	Death
Ileal hemorrhage	Mild; intervention not indicated	Moderate symptoms; medical intervention or minor cauterization indicated	Transfusion, radiologic, endoscopic, or elective operative intervention indicated	Life-threatening consequences; urgent intervention indicated	Death
Ileal obstruction	Asymptomatic; clinical or diagnostic observations only; intervention not indicated	Symptomatic; altered GI function; limiting instrumental ADL	Hospitalization indicated; elective operative intervention indicated; limiting self care ADL; disabling	Life-threatening consequences; urgent operative intervention indicated	Death
Ileal perforation	-	Symptomatic; medical intervention indicated	Severe symptoms; elective operative intervention indicated	Life-threatening consequences; urgent operative intervention indicated	Death
Ileal stenosis	Asymptomatic; clinical or diagnostic observations only; intervention not indicated	Symptomatic; altered GI function	Severely altered GI function; tube feeding or hospitalization indicated; elective operative intervention indicated	Life-threatening consequences; urgent operative intervention indicated	Death
Ileal ulcer	Asymptomatic; clinical or diagnostic observations only; intervention not indicated	Symptomatic; altered GI function	Severely altered GI function; TPN indicated; elective operative or endoscopic intervention indicated; disabling	Life-threatening consequences; urgent operative intervention indicated	Death
Ileus	-	Symptomatic; altered GI function; bowel rest indicated	Severely altered GI function; TPN indicated	Life-threatening consequences; urgent intervention indicated	Death
Intra-abdominal hemorrhage	-	Medical intervention or minor cauterization indicated	Transfusion, radiologic, endoscopic, or elective operative intervention indicated	Life-threatening consequences; urgent intervention indicated	Death
Jejunal fistula	Asymptomatic; clinical or diagnostic observations only; intervention not indicated	Symptomatic; altered GI function	Severely altered GI function; TPN or hospitalization indicated; elective operative intervention indicated	Life-threatening consequences; urgent intervention indicated	Death
Jejunal hemorrhage	Mild; intervention not indicated	Moderate symptoms; medical intervention or minor cauterization indicated	Transfusion, radiologic, endoscopic, or elective operative intervention indicated	Life-threatening consequences; urgent intervention indicated	Death
Jejunal obstruction	Asymptomatic; clinical or diagnostic observations only; intervention not indicated	Symptomatic; altered GI function; limiting instrumental ADL	Hospitalization indicated; elective operative intervention indicated; limiting self care ADL; disabling	Life-threatening consequences; urgent operative intervention indicated	Death
Jejunal perforation	-	Symptomatic; medical intervention indicated	Severe symptoms; elective operative intervention indicated	Life-threatening consequences; urgent operative intervention indicated	Death

Jejunal stenosis	Asymptomatic; clinical or diagnostic observations only; intervention not indicated	Symptomatic; altered GI function	Severely altered GI function; tube feeding or hospitalization indicated; elective operative intervention indicated	Life-threatening consequences; urgent operative intervention indicated	Death
Jejunal ulcer	Asymptomatic; clinical or diagnostic observations only; intervention not indicated	Symptomatic; altered GI function	Severely altered GI function; TPN indicated; elective operative or endoscopic intervention indicated; disabling	Life-threatening consequences; urgent operative intervention indicated	Death
Lip pain	Mild pain	Moderate pain; limiting instrumental ADL	Severe pain; limiting self care ADL	-	-
Lower gastrointestinal hemorrhage	Mild; intervention not indicated	Moderate symptoms; medical intervention or minor cauterization indicated	Transfusion, radiologic, endoscopic, or elective operative intervention indicated	Life-threatening consequences; urgent intervention indicated	Death
Malabsorption	-	Altered diet; oral intervention indicated	Inability to aliment adequately; TPN indicated	Life-threatening consequences; urgent intervention indicated	Death
Mucositis oral	Asymptomatic or mild symptoms; intervention not indicated	Moderate pain; not interfering with oral intake; modified diet indicated	Severe pain; interfering with oral intake	Life-threatening consequences; urgent intervention indicated	Death
Nausea	Loss of appetite without alteration in eating habits	Oral intake decreased without significant weight loss, dehydration or malnutrition	Inadequate oral caloric or fluid intake; tube feeding, TPN, or hospitalization indicated	-	-
Obstruction gastric	Asymptomatic; clinical or diagnostic observations only; intervention not indicated	Symptomatic; altered GI function; limiting instrumental ADL	Hospitalization indicated; elective operative intervention indicated; limiting self care ADL; disabling	Life-threatening consequences; urgent operative intervention indicated	Death
Oral cavity fistula	Asymptomatic; clinical or diagnostic observations only; intervention not indicated	Symptomatic; altered GI function	Severely altered GI function; TPN or hospitalization indicated; elective operative intervention indicated	Life-threatening consequences; urgent intervention indicated	Death
Oral dysesthesia	Mild discomfort; not interfering with oral intake	Moderate pain; interfering with oral intake	Disabling pain; tube feeding or TPN indicated	-	-
Oral hemorrhage	Mild; intervention not indicated	Moderate symptoms; medical intervention or minor cauterization indicated	Transfusion, radiologic, endoscopic, or elective operative intervention indicated	Life-threatening consequences; urgent intervention indicated	Death
Oral pain	Mild pain	Moderate pain; limiting instrumental ADL	Severe pain; limiting self care ADL	-	-
Pancreatic duct stenosis	Asymptomatic; clinical or diagnostic observations only; intervention not indicated	Symptomatic; altered GI function	Severely altered GI function; tube feeding or hospitalization indicated; elective operative intervention indicated	Life-threatening consequences; urgent operative intervention indicated	Death

Pancreatic fistula	Asymptomatic; clinical or diagnostic observations only; intervention not indicated	Symptomatic; altered GI function	Severely altered GI function; tube feeding or TPN or hospitalization indicated; elective operative intervention indicated	Life-threatening consequences; urgent operative intervention indicated	Death
Pancreatic hemorrhage	Mild; intervention not indicated	Moderate symptoms; medical intervention or minor cauterization indicated	Transfusion, radiologic, endoscopic, or elective operative intervention indicated	Life-threatening consequences; urgent intervention indicated	Death
Pancreatic necrosis	-	-	Tube feeding or TPN indicated; radiologic, endoscopic, or operative intervention indicated	Life-threatening consequences; urgent operative intervention indicated	Death
Pancreatitis	-	Enzyme elevation or radiologic findings only	Severe pain; vomiting; medical intervention indicated (e.g., analgesia, nutritional support)	Life-threatening consequences; urgent intervention indicated	Death
Periodontal disease	Gingival recession or gingivitis; limited bleeding on probing; mild local bone loss	Moderate gingival recession or gingivitis; multiple sites of bleeding on probing; moderate bone loss	Spontaneous bleeding; severe bone loss with or without tooth loss; osteonecrosis of maxilla or mandible	-	-
Peritoneal necrosis	-	-	Tube feeding or TPN indicated; radiologic, endoscopic, or operative intervention indicated	Life-threatening consequences; urgent operative intervention indicated	Death
Proctitis	Rectal discomfort, intervention not indicated	Symptoms (e.g., rectal discomfort, passing blood or mucus); medical intervention indicated; limiting instrumental ADL	Severe symptoms; fecal urgency or stool incontinence; limiting self care ADL	Life-threatening consequences; urgent intervention indicated	Death
Rectal fistula	Asymptomatic; clinical or diagnostic observations only; intervention not indicated	Symptomatic; altered GI function	Severely altered GI function; TPN or hospitalization indicated; elective operative intervention indicated	Life-threatening consequences; urgent intervention indicated	Death
Rectal hemorrhage	Mild; intervention not indicated	Moderate symptoms; medical intervention or minor cauterization indicated	Transfusion, radiologic, endoscopic, or elective operative intervention indicated	Life-threatening consequences; urgent intervention indicated	Death
Rectal mucositis	Asymptomatic or mild symptoms; intervention not indicated	Symptomatic; medical intervention indicated; limiting instrumental ADL	Severe symptoms; limiting self care ADL	Life-threatening consequences; urgent operative intervention indicated	Death
Rectal necrosis	-	-	Tube feeding or TPN indicated; radiologic, endoscopic, or operative intervention indicated	Life-threatening consequences; urgent operative intervention indicated	Death
Rectal obstruction	Asymptomatic; clinical or diagnostic observations only; intervention not indicated	Symptomatic; altered GI function; limiting instrumental ADL	Hospitalization indicated; elective operative intervention indicated; limiting self care ADL; disabling	Life-threatening consequences; urgent operative intervention indicated	Death
Rectal pain	Mild pain	Moderate pain; limiting instrumental ADL	Severe pain; limiting self care ADL	-	-

Rectal perforation	-	Symptomatic; medical intervention indicated	Severe symptoms; elective operative intervention indicated	Life-threatening consequences; urgent operative intervention indicated	Death
Rectal stenosis	Asymptomatic; clinical or diagnostic observations only; intervention not indicated	Symptomatic; altered GI function	Severely altered GI function; tube feeding or hospitalization indicated; elective operative intervention indicated	Life-threatening consequences; urgent operative intervention indicated	Death
Rectal ulcer	Asymptomatic; clinical or diagnostic observations only; intervention not indicated	Symptomatic; altered GI function (e.g. altered dietary habits, vomiting, diarrhea)	Severely altered GI function; TPN indicated; elective operative or endoscopic intervention indicated; disabling	Life-threatening consequences; urgent operative intervention indicated	Death
Retroperitoneal hemorrhage	-	Self-limited; intervention indicated	Transfusion, medical, radiologic, endoscopic, or elective operative intervention indicated	Life-threatening consequences; urgent intervention indicated	Death
Salivary duct inflammation	Slightly thickened saliva; slightly altered taste (e.g., metallic)	Thick, ropy, sticky saliva; markedly altered taste; alteration in diet indicated; secretion-induced symptoms; limiting instrumental ADL	Acute salivary gland necrosis; severe secretion-induced symptoms (e.g., thick saliva/oral secretions or gagging); tube feeding or TPN indicated; limiting self care ADL; disabling	Life-threatening consequences; urgent intervention indicated	Death
Salivary gland fistula	Asymptomatic; clinical or diagnostic observations only; intervention not indicated	Symptomatic; altered GI function; tube feeding indicated	Severely altered GI function; hospitalization indicated; elective operative intervention indicated	Life-threatening consequences; urgent operative intervention indicated	Death
Small intestinal mucositis	Asymptomatic or mild symptoms; intervention not indicated	Symptomatic; medical intervention indicated; limiting instrumental ADL	Severe pain; interfering with oral intake; tube feeding, TPN or hospitalization indicated; limiting self care ADL	Life-threatening consequences; urgent intervention indicated	Death
Small intestinal obstruction	Asymptomatic; clinical or diagnostic observations only; intervention not indicated	Symptomatic; altered GI function; limiting instrumental ADL	Hospitalization indicated; elective operative intervention indicated; limiting self care ADL; disabling	Life-threatening consequences; urgent operative intervention indicated	Death
Small intestinal perforation	-	Symptomatic; medical intervention indicated	Severe symptoms; elective operative intervention indicated	Life-threatening consequences; urgent operative intervention indicated	Death
Small intestinal stenosis	Asymptomatic; clinical or diagnostic observations only; intervention not indicated	Symptomatic; altered GI function	Symptomatic and severely altered GI function; tube feeding, TPN or hospitalization indicated; non-emergent operative intervention indicated	Life-threatening consequences; urgent operative intervention indicated	Death

Small intestine ulcer	Asymptomatic; clinical or diagnostic observations only; intervention not indicated	Symptomatic; altered GI function; limiting instrumental ADL	Severely altered GI function; TPN indicated; elective operative or endoscopic intervention indicated; limiting self care ADL; disabling	Life-threatening consequences; urgent operative intervention indicated	Death
Stomach pain	Mild pain	Moderate pain; limiting instrumental ADL	Severe pain; limiting self care ADL	-	-
Tooth development disorder	Asymptomatic; hypoplasia of tooth or enamel	Impairment correctable with oral surgery	Maldevelopment with impairment not surgically correctable; disabling	-	-
Tooth discoloration	Surface stains	-	-	-	-
Toothache	Mild pain	Moderate pain; limiting instrumental ADL	Severe pain; limiting self care ADL	-	-
Typhlitis	-	-	Symptomatic (e.g., abdominal pain, fever, change in bowel habits with ileus); peritoneal signs	Life-threatening consequences; urgent operative intervention indicated	Death
Upper gastrointestinal hemorrhage	Mild; intervention not indicated	Moderate symptoms; medical intervention or minor cauterization indicated	Transfusion, radiologic, endoscopic, or elective operative intervention indicated	Life-threatening consequences; urgent intervention indicated	Death
Vomiting	1 - 2 episodes (separated by 5 minutes) in 24 hrs	3 - 5 episodes (separated by 5 minutes) in 24 hrs	>=6 episodes (separated by 5 minutes) in 24 hrs; tube feeding, TPN or hospitalization indicated	Life-threatening consequences; urgent intervention indicated	Death
Gastrointestinal disorders - Other, specify	Asymptomatic or mild symptoms; clinical or diagnostic observations only; intervention not indicated	Moderate; minimal, local or noninvasive intervention indicated; limiting age-appropriate instrumental ADL	Severe or medically significant but not immediately life-threatening; hospitalization or prolongation of existing hospitalization indicated; disabling; limiting self care ADL	Life-threatening consequences; urgent intervention indicated	Death
General disorders and administration site conditions	Grade				
Adverse Event	1	2	3	4	5
Chills	Mild sensation of cold; shivering; chattering of teeth	Moderate tremor of the entire body; narcotics indicated	Severe or prolonged, not responsive to narcotics	-	-
Death neonatal	-	-	-	-	Death

Death NOS	-	-	-	-	Death
Edema face	Localized facial edema	Moderate localized facial edema; limiting instrumental ADL	Severe swelling; limiting self care ADL	-	-
Edema limbs	5 - 10% inter-limb discrepancy in volume or circumference at point of greatest visible difference; swelling or obscuration of anatomic architecture on close inspection	>10 - 30% inter-limb discrepancy in volume or circumference at point of greatest visible difference; readily apparent obscuration of anatomic architecture; obliteration of skin folds; readily apparent deviation from normal anatomic contour; limiting instrumental ADL	>30% inter-limb discrepancy in volume; gross deviation from normal anatomic contour; limiting self care ADL	-	-
Edema trunk	Swelling or obscuration of anatomic architecture on close inspection	Readily apparent obscuration of anatomic architecture; obliteration of skin folds; readily apparent deviation from normal anatomic contour; limiting instrumental ADL	Gross deviation from normal anatomic contour; limiting self care ADL	-	-
Facial pain	Mild pain	Moderate pain; limiting instrumental ADL	Severe pain; limiting self care ADL	-	-
Fatigue	Fatigue relieved by rest	Fatigue not relieved by rest; limiting instrumental ADL	Fatigue not relieved by rest, limiting self care ADL	-	-
Fever	38.0 - 39.0 degrees C (100.4 - 102.2 degrees F)	>39.0 - 40.0 degrees C (102.3 - 104.0 degrees F)	>40.0 degrees C (>104.0 degrees F) for <=24 hrs	>40.0 degrees C (>104.0 degrees F) for >24 hrs	Death
Flu like symptoms	Mild flu-like symptoms present	Moderate symptoms; limiting instrumental ADL	Severe symptoms; limiting self care ADL	-	-
Gait disturbance	Mild change in gait (e.g., wide-based, limping or hobbling)	Moderate change in gait (e.g., wide-based, limping or hobbling); assistive device indicated; limiting instrumental ADL	Disabling; limiting self care ADL	-	-

Hypothermia	-	35 - >32 degrees C; 95 - >89.6 degrees F	32 - >28 degrees C; 89.6 - >82.4 degrees F	<=28 degrees C; 82.4 degrees F; life-threatening consequences (e.g., coma, hypotension, pulmonary edema, acidemia, ventricular fibrillation)	Death
Infusion related reaction	Mild transient reaction; infusion interruption not indicated; intervention not indicated	Therapy or infusion interruption indicated but responds promptly to symptomatic treatment (e.g., antihistamines, NSAIDS, narcotics, IV fluids); prophylactic medications indicated for <=24 hrs	Prolonged (e.g., not rapidly responsive to symptomatic medication and/or brief interruption of infusion); recurrence of symptoms following initial improvement; hospitalization indicated for clinical sequelae	Life-threatening consequences; urgent intervention indicated	Death
Infusion site extravasation	-	Erythema with associated symptoms (e.g., edema, pain, induration, phlebitis)	Ulceration or necrosis; severe tissue damage; operative intervention indicated	Life-threatening consequences; urgent intervention indicated	Death
Injection site reaction	Tenderness with or without associated symptoms (e.g., warmth, erythema, itching)	Pain; lipodystrophy; edema; phlebitis	Ulceration or necrosis; severe tissue damage; operative intervention indicated	Life-threatening consequences; urgent intervention indicated	Death
Irritability	Mild; easily consolable	Moderate; limiting instrumental ADL; increased attention indicated	Severe abnormal or excessive response; limiting self care ADL; inconsolable	-	-
Localized edema	Localized to dependent areas, no disability or functional impairment	Moderate localized edema and intervention indicated; limiting instrumental ADL	Severe localized edema and intervention indicated; limiting self care ADL	-	-
Malaise	Uneasiness or lack of well being	Uneasiness or lack of well being; limiting instrumental ADL	-	-	-
Multi-organ failure	-	-	Shock with azotemia and acid-base disturbances; significant coagulation abnormalities	Life-threatening consequences (e.g., vasopressor dependent and oliguric or anuric or ischemic colitis or lactic acidosis)	Death
Neck edema	Asymptomatic localized neck edema	Moderate neck edema; slight obliteration of anatomic landmarks; limiting instrumental ADL	Generalized neck edema (e.g., difficulty in turning neck); limiting self care ADL	-	-

Non-cardiac chest pain	Mild pain	Moderate pain; limiting instrumental ADL	Severe pain; limiting self care ADL	-	-
Pain	Mild pain	Moderate pain; limiting instrumental ADL	Severe pain; limiting self care ADL	-	-
Sudden death NOS	-	-	-	-	Death
General disorders and administration site conditions - Other, specify	Asymptomatic or mild symptoms; clinical or diagnostic observations only; intervention not indicated	Moderate; minimal, local or noninvasive intervention indicated; limiting age-appropriate instrumental ADL	Severe or medically significant but not immediately life-threatening; hospitalization or prolongation of existing hospitalization indicated; disabling; limiting self care ADL	Life-threatening consequences; urgent intervention indicated	Death
Hepatobiliary disorders	Grade				
Adverse Event	1	2	3	4	5
Bile duct stenosis	Asymptomatic; clinical or diagnostic observations only; intervention not indicated	Symptomatic; altered GI function; IV fluids indicated <24 hrs	Severely altered GI function; radiologic, endoscopic or elective operative intervention indicated	Life-threatening consequences; urgent operative intervention indicated	Death
Biliary fistula	-	Symptomatic and intervention not indicated	Severely altered GI function; TPN indicated; endoscopic intervention indicated; elective operative intervention indicated	Life-threatening consequences; urgent operative intervention indicated	Death
Cholecystitis	-	Symptomatic; medical intervention indicated	Severe symptoms; radiologic, endoscopic or elective operative intervention indicated	Life-threatening consequences; urgent operative intervention indicated	Death
Gallbladder fistula	Asymptomatic clinical or diagnostic observations only; intervention not indicated	Symptomatic and intervention not indicated	Symptomatic or severely altered GI function; TPN indicated; radiologic, endoscopic or elective operative intervention indicated	Life-threatening consequences; urgent operative intervention indicated	Death
Gallbladder necrosis	-	-	-	Life-threatening consequences; urgent radiologic or operative intervention indicated	Death

Gallbladder obstruction	Asymptomatic; clinical or diagnostic observations only; intervention not indicated	Symptomatic; altered GI function; IV fluids indicated <24 hrs	Symptomatic and severely altered GI function; tube feeding, TPN or hospitalization indicated; non-emergent operative intervention indicated	Life-threatening consequences; urgent operative intervention indicated	Death
Gallbladder pain	Mild pain	Moderate pain; limiting instrumental ADL	Severe pain; limiting self care ADL	-	-
Gallbladder perforation	-	-	-	Life-threatening consequences; urgent intervention indicated	Death
Hepatic failure	-	-	Asterixis; mild encephalopathy; limiting self care ADL	Moderate to severe encephalopathy; coma; life-threatening consequences	Death
Hepatic hemorrhage	Mild; intervention not indicated	Symptomatic; medical intervention indicated	Transfusion indicated	Life-threatening consequences; urgent intervention indicated	Death
Hepatic necrosis	-	-	-	Life-threatening consequences; urgent radiologic or operative intervention indicated	Death
Hepatic pain	Mild pain	Moderate pain; limiting instrumental ADL	Severe pain; limiting self care ADL	-	-
Perforation bile duct	-	-	Radiologic, endoscopic or elective operative intervention indicated	Life-threatening consequences; urgent operative intervention indicated	Death
Portal hypertension	-	Decreased portal vein flow	Reversal/retrograde portal vein flow; associated with varices and/or ascites	Life-threatening consequences; urgent intervention indicated	Death
Portal vein thrombosis	-	Intervention not indicated	Medical intervention indicated	Life-threatening consequences; urgent intervention indicated	Death
Hepatobiliary disorders - Other, specify	Asymptomatic or mild symptoms; clinical or diagnostic observations only; intervention not indicated	Moderate; minimal, local or noninvasive intervention indicated; limiting age-appropriate instrumental ADL	Severe or medically significant but not immediately life-threatening; hospitalization or prolongation of existing hospitalization indicated; disabling; limiting self care ADL	Life-threatening consequences; urgent intervention indicated	Death

Immune system disorders	Grade				
Adverse Event	1	2	3	4	5
Allergic reaction	Transient flushing or rash, drug fever <38 degrees C (<100.4 degrees F); intervention not indicated	Intervention or infusion interruption indicated; responds promptly to symptomatic treatment (e.g., antihistamines, NSAIDS, narcotics); prophylactic medications indicated for <=24 hrs	Prolonged (e.g., not rapidly responsive to symptomatic medication and/or brief interruption of infusion); recurrence of symptoms following initial improvement; hospitalization indicated for clinical sequelae (e.g., renal impairment, pulmonary infiltrates)	Life-threatening consequences; urgent intervention indicated	Death
Anaphylaxis	-	-	Symptomatic bronchospasm, with or without urticaria; parenteral intervention indicated; allergy-related edema/angioedema; hypotension	Life-threatening consequences; urgent intervention indicated	Death
Autoimmune disorder	Asymptomatic; serologic or other evidence of autoimmune reaction, with normal organ function; intervention not indicated	Evidence of autoimmune reaction involving a non-essential organ or function (e.g., hypothyroidism)	Autoimmune reactions involving major organ (e.g., colitis, anemia, myocarditis, kidney)	Life-threatening consequences; urgent intervention indicated	Death
Cytokine release syndrome	Mild reaction; infusion interruption not indicated; intervention not indicated	Therapy or infusion interruption indicated but responds promptly to symptomatic treatment (e.g., antihistamines, NSAIDS, narcotics, IV fluids); prophylactic medications indicated for <=24 hrs	Prolonged (e.g., not rapidly responsive to symptomatic medication and/or brief interruption of infusion); recurrence of symptoms following initial improvement; hospitalization indicated for clinical sequelae (e.g., renal impairment, pulmonary infiltrates)	Life-threatening consequences; pressor or ventilatory support indicated	Death
Serum sickness	Asymptomatic; clinical or diagnostic observations only; intervention not indicated	Moderate arthralgia; fever, rash, urticaria, antihistamines indicated	Severe arthralgia or arthritis; extensive rash; steroids or IV fluids indicated	Life-threatening consequences; pressor or ventilatory support indicated	Death
Immune system disorders - Other, specify	Asymptomatic or mild symptoms; clinical or diagnostic observations only; intervention not indicated	Moderate; minimal, local or noninvasive intervention indicated; limiting age-appropriate instrumental ADL	Severe or medically significant but not immediately life-threatening; hospitalization or prolongation of existing hospitalization indicated; disabling; limiting self care ADL	Life-threatening consequences; urgent intervention indicated	Death

Infections and infestations	Grade				
Adverse Event	1	2	3	4	5
Abdominal infection	-	-	IV antibiotic, antifungal, or antiviral intervention indicated; radiologic or operative intervention indicated	Life-threatening consequences; urgent intervention indicated	Death
Anorectal infection	Localized; local intervention indicated	Oral intervention indicated (e.g., antibiotic, antifungal, antiviral)	IV antibiotic, antifungal, or antiviral intervention indicated; radiologic, endoscopic, or operative intervention indicated	Life-threatening consequences; urgent intervention indicated	Death
Appendicitis	-	-	IV antibiotic, antifungal, or antiviral intervention indicated; radiologic or operative intervention indicated	Life-threatening consequences; urgent intervention indicated	Death
Appendicitis perforated	-	Symptomatic; medical intervention indicated	Severe symptoms; elective operative intervention indicated	Life-threatening consequences; urgent intervention indicated	Death
Arteritis infective	-	-	IV antibiotic, antifungal, or antiviral intervention indicated; radiologic or operative intervention indicated	Life-threatening consequences; urgent intervention indicated	Death
Biliary tract infection	-	-	IV antibiotic, antifungal, or antiviral intervention indicated; radiologic or operative intervention indicated	Life-threatening consequences; urgent intervention indicated	Death
Bladder infection	-	Oral intervention indicated (e.g., antibiotic, antifungal, antiviral)	IV antibiotic, antifungal, or antiviral intervention indicated; radiologic, endoscopic, or operative intervention indicated	Life-threatening consequences; urgent intervention indicated	Death
Bone infection	-	-	IV antibiotic, antifungal, or antiviral intervention indicated; radiologic or operative intervention indicated	Life-threatening consequences; urgent intervention indicated	Death
Breast infection	-	Local infection with moderate symptoms; oral intervention indicated (e.g., antibiotic, antifungal, antiviral)	Severe infection; axillary adenitis; IV antibacterial, antifungal, or antiviral intervention indicated	Life-threatening consequences; urgent intervention indicated	Death
Bronchial infection	-	Moderate symptoms; oral intervention indicated (e.g., antibiotic, antifungal, antiviral)	IV antibiotic, antifungal, or antiviral intervention indicated; radiologic, endoscopic, or operative intervention indicated	Life-threatening consequences; urgent intervention indicated	Death

Catheter related infection	-	Localized; local intervention indicated; oral intervention indicated (e.g., antibiotic, antifungal, antiviral)	IV antibiotic, antifungal, or antiviral intervention indicated; radiologic or operative intervention indicated	Life-threatening consequences; urgent intervention indicated	Death
Cecal infection	-	-	IV antibiotic, antifungal, or antiviral intervention indicated; radiologic, endoscopic, or operative intervention indicated	Life-threatening consequences; urgent intervention indicated	Death
Cervicitis infection	-	Localized; local intervention indicated (e.g., topical antibiotic, antifungal, or antiviral)	IV antibiotic, antifungal, or antiviral intervention indicated; radiologic or operative intervention indicated	Life-threatening consequences; urgent intervention indicated	Death
Conjunctivitis infective	-	Localized; local intervention indicated (e.g., topical antibiotic, antifungal, or antiviral)	IV antibiotic, antifungal, or antiviral intervention indicated; radiologic or operative intervention indicated	Life-threatening consequences; urgent intervention indicated	Death
Corneal infection	-	Localized; local intervention indicated (e.g., topical antibiotic, antifungal, or antiviral)	IV antibiotic, antifungal, or antiviral intervention indicated; radiologic or operative intervention indicated	Life-threatening consequences; urgent intervention indicated	Death
Cranial nerve infection	-	-	IV antibiotic, antifungal, or antiviral intervention indicated; radiologic or operative intervention indicated	Life-threatening consequences; urgent intervention indicated	Death
Device related infection	-	-	IV antibiotic, antifungal, or antiviral intervention indicated; radiologic or operative intervention indicated	Life-threatening consequences; urgent intervention indicated	Death
Duodenal infection	-	Moderate symptoms; medical intervention indicated (e.g., oral antibiotics)	IV antibiotic, antifungal, or antiviral intervention indicated; radiologic or operative intervention indicated	Life-threatening consequences; urgent intervention indicated	Death
Encephalitis infection	-	-	IV antibiotic, antifungal, or antiviral intervention indicated; severe changes in mental status; self-limited seizure activity; focal neurologic abnormalities	Life-threatening consequences; urgent intervention indicated	Death
Encephalomyelitis infection	-	-	IV antibiotic, antifungal, or antiviral intervention indicated; radiologic or operative intervention indicated	Life-threatening consequences; urgent intervention indicated	Death
Endocarditis infective	-	-	IV antibiotic, antifungal, or antiviral intervention indicated; radiologic or operative intervention indicated	Life-threatening consequences; urgent intervention indicated	Death
Endophthalmitis	-	Local intervention indicated	Systemic intervention or hospitalization indicated	Blindness (20/200 or worse)	-

Enterocolitis infectious	-	Passage of >3 unformed stools per 24 hrs or duration of illness >48 hrs; moderate abdominal pain	IV antibiotic, antifungal, or antiviral intervention indicated; radiologic, endoscopic, or operative intervention indicated; profuse watery diarrhea with signs of hypovolemia; bloody diarrhea; fever; severe abdominal pain; hospitalization indicated	Life-threatening consequences; urgent intervention indicated	Death
Esophageal infection	-	Local intervention indicated (e.g., oral antibiotic, antifungal, antiviral)	IV antibiotic, antifungal, or antiviral intervention indicated; radiologic or operative intervention indicated	Life-threatening consequences; urgent intervention indicated	Death
Eye infection	-	Localized; local intervention indicated (e.g., topical antibiotic, antifungal, or antiviral)	IV antibiotic, antifungal, or antiviral intervention indicated; radiologic or operative intervention indicated	Life-threatening consequences; urgent intervention indicated; enucleation	Death
Gallbladder infection	-	-	IV antibiotic, antifungal, or antiviral intervention indicated; radiologic, endoscopic, or operative intervention indicated	Life-threatening consequences; urgent intervention indicated	Death
Gum infection	Local therapy indicated (swish and swallow)	Moderate symptoms; oral intervention indicated (e.g., antibiotic, antifungal, antiviral)	IV antibiotic, antifungal, or antiviral intervention indicated; radiologic or operative intervention indicated	Life-threatening consequences; urgent intervention indicated	Death
Hepatic infection	-	-	IV antibiotic, antifungal, or antiviral intervention indicated; radiologic or operative intervention indicated	Life-threatening consequences; urgent intervention indicated	Death
Hepatitis viral	Asymptomatic, treatment not indicated	-	Symptomatic liver dysfunction; fibrosis by biopsy; compensated cirrhosis; reactivation of chronic hepatitis	Decompensated liver function (e.g., ascites, coagulopathy, encephalopathy, coma)	Death
Infective myositis	-	Localized; local intervention indicated (e.g., topical antibiotic, antifungal, or antiviral)	IV antibiotic, antifungal, or antiviral intervention indicated; radiologic or operative intervention indicated	Life-threatening consequences; urgent intervention indicated	Death
Joint infection	-	Localized; local intervention indicated; oral intervention indicated (e.g., antibiotic, antifungal, antiviral); needle aspiration indicated (single or multiple)	Arthroscopic intervention indicated (e.g., drainage) or arthrotomy (e.g., open surgical drainage)	Life-threatening consequences; urgent intervention indicated	Death

Kidney infection	-	-	IV antibiotic, antifungal, or antiviral intervention indicated; radiologic, endoscopic, or operative intervention indicated	Life-threatening consequences; urgent intervention indicated	Death
Laryngitis	-	Moderate symptoms; oral intervention indicated (e.g., antibiotic, antifungal, antiviral)	IV antibiotic, antifungal, or antiviral intervention indicated; radiologic or operative intervention indicated	Life-threatening consequences; urgent intervention indicated	Death
Lip infection	Localized, local intervention indicated	Oral intervention indicated (e.g., antibiotic, antifungal, antiviral)	IV antibiotic, antifungal, or antiviral intervention indicated; radiologic or operative intervention indicated	-	-
Lung infection	-	Moderate symptoms; oral intervention indicated (e.g., antibiotic, antifungal, antiviral)	IV antibiotic, antifungal, or antiviral intervention indicated; radiologic, endoscopic, or operative intervention indicated	Life-threatening consequences; urgent intervention indicated	Death
Lymph gland infection	-	Localized; local intervention indicated (e.g., topical antibiotic, antifungal, or antiviral)	IV antibiotic, antifungal, or antiviral intervention indicated; radiologic or operative intervention indicated	Life-threatening consequences; urgent intervention indicated	Death
Mediastinal infection	-	-	IV antibiotic, antifungal, or antiviral intervention indicated; radiologic or operative intervention indicated	Life-threatening consequences; urgent intervention indicated	Death
Meningitis	-	-	IV antibiotic, antifungal, or antiviral intervention indicated; radiologic or operative intervention indicated; focal neurologic deficit	Life-threatening consequences; urgent intervention indicated	Death
Mucosal infection	Localized, local intervention indicated	Oral intervention indicated (e.g., antibiotic, antifungal, antiviral)	IV antibiotic, antifungal, or antiviral intervention indicated; radiologic or operative intervention indicated	Life-threatening consequences; urgent intervention indicated	Death
Nail infection	Localized, local intervention indicated	Oral intervention indicated (e.g., antibiotic, antifungal, antiviral)	IV antibiotic, antifungal, or antiviral intervention indicated; radiologic or operative intervention indicated	-	-
Otitis externa	-	Localized; local intervention indicated (e.g., topical antibiotic, antifungal, or antiviral)	IV antibiotic, antifungal, or antiviral intervention indicated; radiologic or operative intervention indicated	Life-threatening consequences; urgent intervention indicated	Death
Otitis media	-	Localized; local intervention indicated (e.g., topical antibiotic, antifungal, or antiviral)	IV antibiotic, antifungal, or antiviral intervention indicated; radiologic or operative intervention indicated	Life-threatening consequences; urgent intervention indicated	Death

Ovarian infection	-	Localized; local intervention indicated (e.g., topical antibiotic, antifungal, or antiviral)	IV antibiotic, antifungal, or antiviral intervention indicated; radiologic or operative intervention indicated	Life-threatening consequences; urgent intervention indicated	Death
Pancreas infection	-	-	IV antibiotic, antifungal, or antiviral intervention indicated; radiologic or operative intervention indicated	Life-threatening consequences; urgent intervention indicated	Death
Papulopustular rash	Papules and/or pustules covering <10% BSA, which may or may not be associated with symptoms of pruritus or tenderness	Papules and/or pustules covering 10-30% BSA, which may or may not be associated with symptoms of pruritus or tenderness; associated with psychosocial impact; limiting instrumental ADL	Papules and/or pustules covering >30% BSA, which may or may not be associated with symptoms of pruritus or tenderness; limiting self-care ADL; associated with local superinfection with oral antibiotics indicated	Papules and/or pustules covering any % BSA, which may or may not be associated with symptoms of pruritus or tenderness and are associated with extensive superinfection with IV antibiotics indicated; life-threatening consequences	Death
Paronychia	Nail fold edema or erythema; disruption of the cuticle	Localized intervention indicated; oral intervention indicated (e.g., antibiotic, antifungal, antiviral); nail fold edema or erythema with pain; associated with discharge or nail plate separation; limiting instrumental ADL	Surgical intervention or IV antibiotics indicated; limiting self care ADL	-	-
Pelvic infection	-	Moderate symptoms; oral intervention indicated (e.g., antibiotic, antifungal, antiviral)	IV antibiotic, antifungal, or antiviral intervention indicated; radiologic or operative intervention indicated	Life-threatening consequences; urgent intervention indicated	Death
Penile infection	-	Localized; local intervention indicated (e.g., topical antibiotic, antifungal, or antiviral)	IV antibiotic, antifungal, or antiviral intervention indicated; radiologic or operative intervention indicated	Life-threatening consequences; urgent intervention indicated	Death
Periorbital infection	-	Localized; local intervention indicated (e.g., topical antibiotic, antifungal, or antiviral)	IV antibiotic, antifungal, or antiviral intervention indicated; radiologic or operative intervention indicated	Life-threatening consequences; urgent intervention indicated	Death
Peripheral nerve infection	-	Localized; local intervention indicated (e.g., topical antibiotic, antifungal, or antiviral)	IV antibiotic, antifungal, or antiviral intervention indicated; radiologic or operative intervention indicated	Life-threatening consequences; urgent intervention indicated	Death
Peritoneal infection	-	-	IV antibiotic, antifungal, or antiviral intervention indicated; radiologic, endoscopic, or operative intervention indicated	Life-threatening consequences; urgent intervention indicated	Death

Pharyngitis	-	Localized; local intervention indicated (e.g., topical antibiotic, antifungal, or antiviral)	IV antibiotic, antifungal, or antiviral intervention indicated; radiologic or operative intervention indicated	Life-threatening consequences; urgent intervention indicated	Death
Phlebitis infective	-	Localized; local intervention indicated (e.g., topical antibiotic, antifungal, or antiviral)	IV antibiotic, antifungal, or antiviral intervention indicated; radiologic or operative intervention indicated	Life-threatening consequences; urgent intervention indicated	Death
Pleural infection	-	Localized; local intervention indicated (e.g., topical antibiotic, antifungal, or antiviral)	IV antibiotic, antifungal, or antiviral intervention indicated; radiologic, endoscopic, or operative intervention indicated	Life-threatening consequences; urgent intervention indicated	Death
Prostate infection	-	Moderate symptoms; oral intervention indicated (e.g., antibiotic, antifungal, antiviral)	IV antibiotic, antifungal, or antiviral intervention indicated; radiologic, endoscopic, or operative intervention indicated	Life-threatening consequences; urgent intervention indicated	Death
Rash pustular	-	Localized; local intervention indicated (e.g., topical antibiotic, antifungal, or antiviral)	IV antibiotic, antifungal, or antiviral intervention indicated; radiologic or operative intervention indicated	-	-
Rhinitis infective	-	Localized; local intervention indicated (e.g., topical antibiotic, antifungal, or antiviral)	-	-	-
Salivary gland infection	-	Moderate symptoms; oral intervention indicated (e.g., antibiotic, antifungal, antiviral)	IV antibiotic, antifungal, or antiviral intervention indicated; radiologic or operative intervention indicated	Life-threatening consequences; urgent intervention indicated	Death
Scrotal infection	-	Localized; local intervention indicated (e.g., topical antibiotic, antifungal, or antiviral)	IV antibiotic, antifungal, or antiviral intervention indicated; radiologic or operative intervention indicated	Life-threatening consequences; urgent intervention indicated	Death
Sepsis	-	-	-	Life-threatening consequences; urgent intervention indicated	Death
Sinusitis	-	Localized; local intervention indicated (e.g., topical antibiotic, antifungal, or antiviral)	IV antibiotic, antifungal, or antiviral intervention indicated; radiologic, endoscopic, or operative intervention indicated	Life-threatening consequences; urgent intervention indicated	Death
Skin infection	Localized, local intervention indicated	Oral intervention indicated (e.g., antibiotic, antifungal, antiviral)	IV antibiotic, antifungal, or antiviral intervention indicated; radiologic or operative intervention indicated	Life-threatening consequences; urgent intervention indicated	Death
Small intestine infection	-	Moderate symptoms; oral intervention indicated (e.g., antibiotic, antifungal, antiviral)	IV antibiotic, antifungal, or antiviral intervention indicated; radiologic or operative intervention indicated	Life-threatening consequences; urgent intervention indicated	Death

Soft tissue infection	-	Localized; local intervention indicated (e.g., topical antibiotic, antifungal, or antiviral)	IV antibiotic, antifungal, or antiviral intervention indicated; radiologic or operative intervention indicated	Life-threatening consequences; urgent intervention indicated	Death
Splenic infection	-	-	IV antibiotic, antifungal, or antiviral intervention indicated; radiologic or operative intervention indicated	Life-threatening consequences; urgent intervention indicated	Death
Stoma site infection	Localized, local intervention indicated	Oral intervention indicated (e.g., antibiotic, antifungal, antiviral)	IV antibiotic, antifungal, or antiviral intervention indicated; radiologic, endoscopic, or operative intervention indicated	Life-threatening consequences; urgent intervention indicated	Death
Tooth infection	-	Localized; local intervention indicated (e.g., topical antibiotic, antifungal, or antiviral)	IV antibiotic, antifungal, or antiviral intervention indicated; radiologic or operative intervention indicated	Life-threatening consequences; urgent intervention indicated	Death
Tracheitis	-	Moderate symptoms; oral intervention indicated (e.g., antibiotic, antifungal, antiviral)	IV antibiotic, antifungal, or antiviral intervention indicated; radiologic, endoscopic, or operative intervention indicated	Life-threatening consequences; urgent intervention indicated	Death
Upper respiratory infection	-	Moderate symptoms; oral intervention indicated (e.g., antibiotic, antifungal, antiviral)	IV antibiotic, antifungal, or antiviral intervention indicated; radiologic, endoscopic, or operative intervention indicated	Life-threatening consequences; urgent intervention indicated	Death
Urethral infection	-	Localized; local intervention indicated (e.g., topical antibiotic, antifungal, or antiviral)	IV antibiotic, antifungal, or antiviral intervention indicated; radiologic, endoscopic, or operative intervention indicated	Life-threatening consequences; urgent intervention indicated	Death
Urinary tract infection	-	Localized; local intervention indicated (e.g., topical antibiotic, antifungal, or antiviral)	IV antibiotic, antifungal, or antiviral intervention indicated; radiologic or operative intervention indicated	Life-threatening consequences; urgent intervention indicated	Death
Uterine infection	-	Moderate symptoms; oral intervention indicated (e.g., antibiotic, antifungal, antiviral)	IV antibiotic, antifungal, or antiviral intervention indicated; radiologic or operative intervention indicated	Life-threatening consequences; urgent intervention indicated	Death
Vaginal infection	-	Localized; local intervention indicated (e.g., topical antibiotic, antifungal, or antiviral)	IV antibiotic, antifungal, or antiviral intervention indicated; radiologic or operative intervention indicated	Life-threatening consequences; urgent intervention indicated	Death
Vulval infection	Localized, local intervention indicated	Oral intervention indicated (e.g., antibiotic, antifungal, antiviral)	IV antibiotic, antifungal, or antiviral intervention indicated; radiologic or operative intervention indicated	Life-threatening consequences; urgent intervention indicated	Death

Wound infection	-	Localized; local intervention indicated (e.g., topical antibiotic, antifungal, or antiviral)	IV antibiotic, antifungal, or antiviral intervention indicated; radiologic or operative intervention indicated	Life-threatening consequences; urgent intervention indicated	Death
Infections and infestations - Other, specify	Asymptomatic or mild symptoms; clinical or diagnostic observations only; intervention not indicated	Moderate; minimal, local or noninvasive intervention indicated; limiting age-appropriate instrumental ADL	Severe or medically significant but not immediately life-threatening; hospitalization or prolongation of existing hospitalization indicated; disabling; limiting self care ADL	Life-threatening consequences; urgent intervention indicated	Death
Injury, poisoning and procedural complications			Grade		
Adverse Event	1	2	3	4	5
Ankle fracture	Mild; non-surgical intervention indicated	Limiting instrumental ADL; operative intervention indicated	Limiting self care ADL; elective surgery indicated	-	-
Aortic injury	-	-	Severe symptoms; limiting self care ADL; disabling; repair or revision indicated	Life-threatening consequences; evidence of end organ damage; urgent operative intervention indicated	Death
Arterial injury	Asymptomatic diagnostic finding; intervention not indicated	Symptomatic (e.g., claudication); repair or revision not indicated	Severe symptoms; limiting self care ADL; disabling; repair or revision indicated	Life-threatening consequences; evidence of end organ damage; urgent operative intervention indicated	Death
Biliary anastomotic leak	Asymptomatic diagnostic observations only; intervention not indicated	Symptomatic; medical intervention indicated	Severe symptoms; radiologic, endoscopic or elective operative intervention indicated	Life-threatening consequences; urgent operative intervention indicated	Death
Bladder anastomotic leak	Asymptomatic diagnostic observations only; intervention not indicated	Symptomatic; medical intervention indicated	Severe symptoms; radiologic, endoscopic or elective operative intervention indicated	Life-threatening consequences; urgent operative intervention indicated	Death
Bruising	Localized or in a dependent area	Generalized	-	-	-
Burn	Minimal symptoms; intervention not indicated	Medical intervention; minimal debridement indicated	Moderate to major debridement or reconstruction indicated	Life-threatening consequences	Death
Dermatitis radiation	Faint erythema or dry desquamation	Moderate to brisk erythema; patchy moist desquamation, mostly confined to skin folds and creases; moderate edema	Moist desquamation in areas other than skin folds and creases; bleeding induced by minor trauma or abrasion	Life-threatening consequences; skin necrosis or ulceration of full thickness dermis; spontaneous bleeding from involved site; skin graft indicated	Death

Esophageal anastomotic leak	Asymptomatic diagnostic observations only; intervention not indicated	Symptomatic; medical intervention indicated	Severe symptoms; radiologic, endoscopic or elective operative intervention indicated	Life-threatening consequences; urgent operative intervention indicated	Death
Fall	Minor with no resultant injuries; intervention not indicated	Symptomatic; noninvasive intervention indicated	Hospitalization indicated	-	-
Fallopian tube anastomotic leak	Asymptomatic; clinical or diagnostic observations only; intervention not indicated	Symptomatic; medical intervention indicated	Severe symptoms; radiologic, endoscopic or elective operative intervention indicated	Life-threatening consequences; urgent operative intervention indicated	Death
Fallopian tube perforation	Asymptomatic diagnostic observations only; intervention not indicated	Symptomatic and intervention not indicated	Severe symptoms; elective operative intervention indicated	Life-threatening consequences; urgent operative intervention indicated (e.g., organ resection)	Death
Fracture	Asymptomatic; clinical or diagnostic observations only; intervention not indicated	Symptomatic but non-displaced; immobilization indicated	Severe symptoms; displaced or open wound with bone exposure; disabling; operative intervention indicated	Life-threatening consequences; urgent intervention indicated	Death
Gastric anastomotic leak	Asymptomatic diagnostic observations only; intervention not indicated	Symptomatic; medical intervention indicated	Severe symptoms; radiologic, endoscopic or elective operative intervention indicated	Life-threatening consequences; urgent operative intervention indicated	Death
Gastrointestinal anastomotic leak	Asymptomatic diagnostic observations only; intervention not indicated	Symptomatic; medical intervention indicated	Severe symptoms; radiologic, endoscopic or elective operative intervention indicated	Life-threatening consequences; urgent operative intervention indicated	Death
Gastrointestinal stoma necrosis	-	Superficial necrosis; intervention not indicated	Severe symptoms; hospitalization or elective operative intervention indicated	Life-threatening consequences; urgent intervention indicated	Death
Hip fracture	-	Hairline fracture; mild pain; limiting instrumental ADL; non-surgical intervention indicated	Severe pain; hospitalization or intervention indicated for pain control (e.g., traction); operative intervention indicated	Life-threatening consequences; symptoms associated with neurovascular compromise	-
Injury to carotid artery	-	-	Severe symptoms; limiting self care ADL (e.g., transient cerebral ischemia); repair or revision indicated	Life-threatening consequences; urgent intervention indicated	Death
Injury to inferior vena cava	-	-	-	Life-threatening consequences; urgent intervention indicated	Death
Injury to jugular vein	-	-	Symptomatic limiting self care ADL; disabling; repair or revision indicated	Life-threatening consequences; urgent intervention indicated	Death

Injury to superior vena cava	Asymptomatic diagnostic finding; intervention not indicated	Symptomatic; repair or revision not indicated	Severe symptoms; limiting self care ADL; disabling; repair or revision indicated	Life-threatening consequences; evidence of end organ damage; urgent operative intervention indicated	Death
Intestinal stoma leak	Asymptomatic diagnostic observations only; intervention not indicated	Symptomatic; medical intervention indicated	Severe symptoms; radiologic, endoscopic or elective operative intervention indicated	Life-threatening consequences; urgent operative intervention indicated	Death
Intestinal stoma obstruction	-	Self-limited; intervention not indicated	Severe symptoms; IV fluids, tube feeding, or TPN indicated >=24 hrs; elective operative intervention indicated	Life-threatening consequences; urgent operative intervention indicated	Death
Intestinal stoma site bleeding	Minimal bleeding identified on clinical exam; intervention not indicated	Moderate bleeding; medical intervention indicated	Severe bleeding; transfusion indicated; radiologic or endoscopic intervention indicated	Life-threatening consequences; urgent intervention indicated	Death
Intraoperative arterial injury	Primary repair of injured organ/structure indicated	Partial resection of injured organ/structure indicated	Complete resection or reconstruction of injured organ/structure indicated; disabling	Life-threatening consequences; urgent intervention indicated	Death
Intraoperative breast injury	Primary repair of injured organ/structure indicated	Partial resection of injured organ/structure indicated	Complete resection or reconstruction of injured organ/structure indicated; disabling	Life-threatening consequences; urgent intervention indicated	Death
Intraoperative cardiac injury	-	-	Primary repair of injured organ/structure indicated	Life-threatening consequences; urgent intervention indicated	Death
Intraoperative ear injury	Primary repair of injured organ/structure indicated	Partial resection of injured organ/structure indicated	Complete resection of injured organ/structure indicated; disabling (e.g., impaired hearing; impaired balance)	Life-threatening consequences; urgent intervention indicated	Death
Intraoperative endocrine injury	Primary repair of injured organ/structure indicated	Partial resection of injured organ/structure indicated	Complete resection or reconstruction of injured organ/structure indicated; disabling	Life-threatening consequences; urgent intervention indicated	Death
Intraoperative gastrointestinal injury	Primary repair of injured organ/structure indicated	Partial resection of injured organ/structure indicated	Complete resection or reconstruction of injured organ/structure indicated; disabling	Life-threatening consequences; urgent intervention indicated	Death
Intraoperative head and neck injury	Primary repair of injured organ/structure indicated	Partial resection of injured organ/structure indicated	Complete resection or reconstruction of injured organ/structure indicated; disabling	Life-threatening consequences; urgent intervention indicated	Death
Intraoperative hemorrhage	-	-	Postoperative radiologic, endoscopic, or operative intervention indicated	Life-threatening consequences; urgent intervention indicated	Death

Intraoperative hepatobiliary injury	Primary repair of injured organ/structure indicated	Partial resection of injured organ/structure indicated	Complete resection or reconstruction of injured organ/structure indicated; disabling	Life-threatening consequences; urgent intervention indicated	Death
Intraoperative musculoskeletal injury	Primary repair of injured organ/structure indicated	Partial resection of injured organ/structure indicated	Complete resection or reconstruction of injured organ/structure indicated; disabling	Life-threatening consequences; urgent intervention indicated	Death
Intraoperative neurological injury	Primary repair of injured organ/structure indicated	Partial resection of injured organ/structure indicated	Complete resection or reconstruction of injured organ/structure indicated; disabling	Life-threatening consequences; urgent intervention indicated	Death
Intraoperative ocular injury	Primary repair of injured organ/structure indicated	Partial resection of injured organ/structure indicated	Complete resection or reconstruction of injured organ/structure indicated; disabling	Life-threatening consequences; urgent intervention indicated	Death
Intraoperative renal injury	Primary repair of injured organ/structure indicated	Partial resection of injured organ/structure indicated	Complete resection or reconstruction of injured organ/structure indicated; disabling	Life-threatening consequences; urgent intervention indicated	Death
Intraoperative reproductive tract injury	Primary repair of injured organ/structure indicated	Partial resection of injured organ/structure indicated	Complete resection or reconstruction of injured organ/structure indicated; disabling	Life-threatening consequences; urgent intervention indicated	Death
Intraoperative respiratory injury	Primary repair of injured organ/structure indicated	Partial resection of injured organ/structure indicated	Complete resection or reconstruction of injured organ/structure indicated; disabling	Life-threatening consequences; urgent intervention indicated	Death
Intraoperative skin injury	Primary repair of injured organ/structure indicated	Partial resection of injured organ/structure indicated	Complete resection or reconstruction of injured organ/structure indicated; disabling	Life-threatening consequences; urgent intervention indicated	Death
Intraoperative splenic injury	-	Primary repair of injured organ/structure indicated	Resection or reconstruction of injured organ/structure indicated; disabling	Life-threatening consequences; urgent intervention indicated	Death
Intraoperative urinary injury	Primary repair of injured organ/structure indicated	Partial resection of injured organ/structure indicated	Complete resection or reconstruction of injured organ/structure indicated; disabling	Life-threatening consequences; urgent intervention indicated	Death
Intraoperative venous injury	Primary repair of injured organ/structure indicated	Partial resection of injured organ/structure indicated	Complete resection or reconstruction of injured organ/structure indicated; disabling	Life-threatening consequences; urgent intervention indicated	Death
Kidney anastomotic leak	Asymptomatic diagnostic observations only; intervention not indicated	Symptomatic; medical intervention indicated	Severe symptoms; radiologic, endoscopic or elective operative intervention indicated	Life-threatening consequences; urgent operative intervention indicated	Death

Large intestinal anastomotic leak	Asymptomatic diagnostic observations only; intervention not indicated	Symptomatic; medical intervention indicated	Severe symptoms; radiologic, endoscopic or elective operative intervention indicated	Life-threatening consequences; urgent operative intervention indicated	Death
Pancreatic anastomotic leak	Asymptomatic diagnostic observations only; intervention not indicated	Symptomatic; medical intervention indicated	Severe symptoms; radiologic, endoscopic or elective operative intervention indicated	Life-threatening consequences; urgent operative intervention indicated	Death
Pharyngeal anastomotic leak	Asymptomatic diagnostic observations only; intervention not indicated	Symptomatic; medical intervention indicated	Severe symptoms; radiologic, endoscopic or elective operative intervention indicated	Life-threatening consequences; urgent operative intervention indicated	Death
Postoperative hemorrhage	Minimal bleeding identified on clinical exam; intervention not indicated	Moderate bleeding; radiologic, endoscopic, or operative intervention indicated	Transfusion indicated of >=2 units (10 cc/kg for pediatrics) pRBCs beyond protocol specification; urgent radiologic, endoscopic, or operative intervention indicated	Life-threatening consequences; urgent intervention indicated	Death
Postoperative thoracic procedure complication	-	Extubated within 24 - 72 hrs postoperatively	Extubated >72 hrs postoperatively, but before tracheostomy indicated	Life-threatening airway compromise; urgent intervention indicated (e.g., tracheotomy or intubation)	Death
Prolapse of intestinal stoma	Asymptomatic; reducible	Recurrent after manual reduction; local irritation or stool leakage; difficulty to fit appliance; limiting instrumental ADL	Severe symptoms; elective operative intervention indicated; limiting self care ADL	Life-threatening consequences; urgent operative intervention indicated	Death
Prolapse of urostomy	Asymptomatic; clinical or diagnostic observations only; intervention not indicated	Local care or maintenance; minor revision indicated	Dysfunctional stoma; elective operative intervention or major stomal revision indicated	Life-threatening consequences; urgent intervention indicated	Death
Radiation recall reaction (dermatologic)	Faint erythema or dry desquamation	Moderate to brisk erythema; patchy moist desquamation, mostly confined to skin folds and creases; moderate edema	Moist desquamation in areas other than skin folds and creases; bleeding induced by minor trauma or abrasion	Life-threatening consequences; skin necrosis or ulceration of full thickness dermis; spontaneous bleeding from involved site; skin graft indicated	Death
Rectal anastomotic leak	Asymptomatic diagnostic observations only; intervention not indicated	Symptomatic; medical intervention indicated	Severe symptoms; radiologic, endoscopic or elective operative intervention indicated	Life-threatening consequences; urgent operative intervention indicated	Death
Seroma	Asymptomatic; clinical or diagnostic observations only; intervention not indicated	Symptomatic; simple aspiration indicated	Symptomatic, elective radiologic or operative intervention indicated	-	-
Small intestinal anastomotic leak	Asymptomatic diagnostic observations only; intervention not indicated	Symptomatic; medical intervention indicated	Severe symptoms; radiologic, endoscopic or elective operative intervention indicated	Life-threatening consequences; urgent operative intervention indicated	Death

Spermatic cord anastomotic leak	Asymptomatic diagnostic observations only; intervention not indicated	Symptomatic; medical intervention indicated	Severe symptoms; radiologic, endoscopic or elective operative intervention indicated	Life-threatening consequences; urgent operative intervention indicated	Death
Spinal fracture	Mild back pain; nonprescription analgesics indicated	Moderate back pain; prescription analgesics indicated; limiting instrumental ADL	Severe back pain; hospitalization or intervention indicated for pain control (e.g., vertebroplasty); limiting self care ADL; disability	Life-threatening consequences; symptoms associated with neurovascular compromise	Death
Stenosis of gastrointestinal stoma	-	Symptomatic; IV fluids indicated <24 hrs; manual dilation at bedside	Severely altered GI function; tube feeding, TPN or hospitalization indicated; elective operative intervention indicated	Life-threatening consequences; urgent operative intervention indicated	Death
Stomal ulcer	Asymptomatic; clinical or diagnostic observations only; intervention not indicated	Symptomatic; medical intervention indicated	Severe symptoms; elective operative intervention indicated	-	-
Tracheal hemorrhage	Minimal bleeding identified on clinical or diagnostic exam; intervention not indicated	Moderate bleeding; medical intervention indicated	Severe bleeding; transfusion indicated; radiologic or endoscopic intervention indicated	Life-threatening consequences; urgent intervention indicated	Death
Tracheal obstruction	Partial asymptomatic obstruction on examination (e.g., visual, radiologic or endoscopic)	Symptomatic (e.g., noisy airway breathing), no respiratory distress; medical intervention indicated (e.g., steroids); limiting instrumental ADL	Stridor; radiologic or endoscopic intervention indicated (e.g., stent, laser); limiting self care ADL	Life-threatening airway compromise; urgent intervention indicated (e.g., tracheotomy or intubation)	Death
Tracheostomy site bleeding	Minimal bleeding identified on clinical exam; intervention not indicated	Moderate bleeding; medical intervention indicated	Severe bleeding; transfusion indicated; radiologic or endoscopic intervention indicated	Life-threatening consequences; urgent intervention indicated	Death
Ureteric anastomotic leak	Asymptomatic diagnostic observations only; intervention not indicated	Symptomatic; medical intervention indicated	Severe symptoms; radiologic, endoscopic or elective operative intervention indicated	Life-threatening consequences; urgent operative intervention indicated	Death
Urethral anastomotic leak	Asymptomatic diagnostic observations only; intervention not indicated	Symptomatic; medical intervention indicated	Severe symptoms; radiologic, endoscopic or elective operative intervention indicated	Life-threatening consequences; urgent operative intervention indicated	Death
Urostomy leak	Asymptomatic diagnostic observations only; intervention not indicated	Symptomatic; medical intervention indicated	Severe symptoms; radiologic, endoscopic or elective operative intervention indicated	Life-threatening consequences; urgent operative intervention indicated	Death
Urostomy obstruction	Asymptomatic diagnostic observations only; intervention not indicated	Symptomatic; dilation or endoscopic repair or stent placement indicated	Altered organ function (e.g., sepsis or hydronephrosis, or renal dysfunction); elective operative intervention indicated	Life-threatening consequences; organ failure; urgent operative intervention indicated	Death

Urostomy site bleeding	Minimal bleeding identified on clinical exam; intervention not indicated	Moderate bleeding; medical intervention indicated	Severe bleeding; transfusion indicated; radiologic or endoscopic intervention indicated	Life-threatening consequences; urgent intervention indicated	Death
Urostomy stenosis	-	Symptomatic but no hydronephrosis, no sepsis or no renal dysfunction; dilation or endoscopic repair or stent placement indicated	Symptomatic (e.g., hydronephrosis, or renal dysfunction); elective operative intervention indicated	Life-threatening consequences; urgent operative intervention indicated	Death
Uterine anastomotic leak	Asymptomatic diagnostic observations only; intervention not indicated	Symptomatic; medical intervention indicated	Severe symptoms; radiologic, endoscopic or elective operative intervention indicated	Life-threatening consequences; urgent operative intervention indicated	Death
Uterine perforation	Asymptomatic diagnostic observations only; intervention not indicated	Symptomatic and intervention not indicated	Severe symptoms; elective operative intervention indicated	Life-threatening consequences; urgent intervention indicated	Death
Vaginal anastomotic leak	Asymptomatic diagnostic observations only; intervention not indicated	Symptomatic; medical intervention indicated	Severe symptoms; radiologic, endoscopic or elective operative intervention indicated	Life-threatening consequences; urgent operative intervention indicated	Death
Vas deferens anastomotic leak	Asymptomatic diagnostic observations only; intervention not indicated	Symptomatic; medical intervention indicated	Severe symptoms; radiologic, endoscopic or elective operative intervention indicated	Life-threatening consequences; urgent operative intervention indicated	Death
Vascular access complication	-	Device dislodgement, blockage, leak, or malposition; device replacement indicated	Deep vein or cardiac thrombosis; intervention indicated (e.g., anticoagulation, lysis, filter, invasive procedure)	Embolic event including pulmonary embolism or life-threatening thrombus	Death
Venous injury	Asymptomatic diagnostic finding; intervention not indicated	Symptomatic (e.g., claudication); repair or revision not indicated	Severe symptoms; limiting self care ADL; repair or revision indicated; disabling	Life-threatening consequences; evidence of end organ damage; urgent operative intervention indicated	Death
Wound complication	Incisional separation of <=25% of wound, no deeper than superficial fascia	Incisional separation >25% of wound; local care indicated	Hernia without evidence of strangulation; fascial disruption/dehiscence; primary wound closure or revision by operative intervention indicated	Hernia with evidence of strangulation; major reconstruction flap, grafting, resection, or amputation indicated	Death
Wound dehiscence	Incisional separation of <=25% of wound, no deeper than superficial fascia	Incisional separation >25% of wound with local care; asymptomatic hernia or symptomatic hernia without evidence of strangulation	Fascial disruption or dehiscence without evisceration; primary wound closure or revision by operative intervention indicated	Life-threatening consequences; symptomatic hernia with evidence of strangulation; fascial disruption with evisceration; major reconstruction flap, grafting, resection, or amputation indicated	Death

Wrist fracture	Mild; non-surgical intervention indicated	Limiting instrumental ADL; operative intervention indicated	Limiting self care ADL; elective surgery indicated	-	-
Injury, poisoning and procedural complications - Other, specify	Asymptomatic or mild symptoms; clinical or diagnostic observations only; intervention not indicated	Moderate; minimal, local or noninvasive intervention indicated; limiting age-appropriate instrumental ADL	Severe or medically significant but not immediately life-threatening; hospitalization or prolongation of existing hospitalization indicated; disabling; limiting self care ADL	Life-threatening consequences; urgent intervention indicated	Death
Investigations	Grade				
Adverse Event	1	2	3	4	5
Activated partial thromboplastin time prolonged	>ULN - 1.5 x ULN	>1.5 - 2.5 x ULN	>2.5 x ULN; hemorrhage	-	-
Alanine aminotransferase increased	>ULN - 3.0 x ULN	>3.0 - 5.0 x ULN	>5.0 - 20.0 x ULN	>20.0 x ULN	-
Alkaline phosphatase increased	>ULN - 2.5 x ULN	>2.5 - 5.0 x ULN	>5.0 - 20.0 x ULN	>20.0 x ULN	-
Aspartate aminotransferase increased	>ULN - 3.0 x ULN	>3.0 - 5.0 x ULN	>5.0 - 20.0 x ULN	>20.0 x ULN	-
Blood antidiuretic hormone abnormal	Asymptomatic; clinical or diagnostic observations only; intervention not indicated	Symptomatic; medical intervention indicated	Hospitalization indicated	-	-
Blood bilirubin increased	>ULN - 1.5 x ULN	>1.5 - 3.0 x ULN	>3.0 - 10.0 x ULN	>10.0 x ULN	-
Blood corticotrophin decreased	Asymptomatic; clinical or diagnostic observations only; intervention not indicated	Symptomatic; medical intervention indicated	Hospitalization indicated	-	-
Blood gonadotrophin abnormal	Asymptomatic; clinical or diagnostic observations only; intervention not indicated	Symptomatic; medical intervention indicated; limiting instrumental ADL	Severe symptoms; limiting self care ADL	-	-
Blood prolactin abnormal	Asymptomatic; clinical or diagnostic observations only; intervention not indicated	Moderate symptoms; limiting instrumental ADL	-	-	-
Carbon monoxide diffusing capacity decreased	3 - 5 units below LLN; for follow-up, a decrease of 3 - 5 units (ml/min/mm Hg) below the baseline value	6 - 8 units below LLN; for follow-up, an asymptomatic decrease of >5 - 8 units (ml/min/mm Hg) below the baseline value	Asymptomatic decrease of >8 units drop; >5 units drop along with the presence of pulmonary symptoms (e.g. , >Grade 2 hypoxia or >Grade 2 or higher dyspnea)	-	-

Cardiac troponin I increased	Levels above the upper limit of normal and below the level of myocardial infarction as defined by the manufacturer	-	Levels consistent with myocardial infarction as defined by the manufacturer	-	-
Cardiac troponin T increased	Levels above the upper limit of normal and below the level of myocardial infarction as defined by the manufacturer	-	Levels consistent with myocardial infarction as defined by the manufacturer	-	-
CD4 lymphocytes decreased	<LLN - 500/mm3; <LLN - 0.5 x 10e9 /L	<500 - 200/mm3; <0.5 - 0.2 x 10e9 /L	<200 - 50/mm3; <0.2 x 0.05 - 10e9 /L	<50/mm3; <0.05 x 10e9 /L	-
Cholesterol high	>ULN - 300 mg/dL; >ULN - 7.75 mmol/L	>300 - 400 mg/dL; >7.75 - 10.34 mmol/L	>400 - 500 mg/dL; >10.34 - 12.92 mmol/L	>500 mg/dL; >12.92 mmol/L	-
CPK increased	>ULN - 2.5 x ULN	>2.5 x ULN - 5 x ULN	>5 x ULN - 10 x ULN	>10 x ULN	-
Creatinine increased	>1 - 1.5 x baseline; >ULN - 1.5 x ULN	>1.5 - 3.0 x baseline; >1.5 - 3.0 x ULN	>3.0 baseline; >3.0 - 6.0 x ULN	>6.0 x ULN	-
Ejection fraction decreased	-	Resting ejection fraction (EF) 50 - 40%; 10 - 19% drop from baseline	Resting ejection fraction (EF) 39 - 20%; >20% drop from baseline	Resting ejection fraction (EF) <20%	-
Electrocardiogram QT corrected interval prolonged	QTc 450 - 480 ms	QTc 481 - 500 ms	QTc >= 501 ms on at least two separate ECGs	QTc >= 501 or >60 ms change from baseline and Torsade de pointes or polymorphic ventricular tachycardia or signs/symptoms of serious arrhythmia	-
Fibrinogen decreased	<1.0 - 0.75 x LLN or <25% decrease from baseline	<0.75 - 0.5 x LLN or 25 - <50% decrease from baseline	<0.5 - 0.25 x LLN or 50 - <75% decrease from baseline	<0.25 x LLN or 75% decrease from baseline or absolute value <50 mg/dL	-
Forced expiratory volume decreased	FEV1% (percentages of observed FEV1 and FVC related to their respective predicted values) 99 - 70% predicted	FEV1 60 - 69%	50 - 59%	<= 49%	-
GGT increased	>ULN - 2.5 x ULN	>2.5 - 5.0 x ULN	>5.0 - 20.0 x ULN	>20.0 x ULN	-
Growth hormone abnormal	Asymptomatic; clinical or diagnostic observations only; intervention not indicated	Symptomatic; medical intervention indicated; limiting instrumental ADL	-	-	-
Haptoglobin decreased	<LLN	-	-	-	-

Hemoglobin increased	Increase in >0 - 2 gm/dL above ULN or above baseline if baseline is above ULN	Increase in >2 - 4 gm/dL above ULN or above baseline if baseline is above ULN	Increase in >4 gm/dL above ULN or above baseline if baseline is above ULN	-	-
INR increased	>1 - 1.5 x ULN; >1 - 1.5 times above baseline if on anticoagulation	>1.5 - 2.5 x ULN; >1.5 - 2.5 times above baseline if on anticoagulation	>2.5 x ULN; >2.5 times above baseline if on anticoagulation	-	-
Lipase increased	>ULN - 1.5 x ULN	>1.5 - 2.0 x ULN	>2.0 - 5.0 x ULN	>5.0 x ULN	-
Lymphocyte count decreased	<LLN - 800/mm3; <LLN - 0.8 x 10e9/L	<800 - 500/mm3; <0.8 - 0.5 x 10e9 /L	<500 - 200/mm3; <0.5 - 0.2 x 10e9 /L	<200/mm3; <0.2 x 10e9 /L	-
Lymphocyte count increased	-	>4000/mm3 - 20,000/mm3	>20,000/mm3	-	-
Neutrophil count decreased	<LLN - 1500/mm3; <LLN - 1.5 x 10e9 /L	<1500 - 1000/mm3; <1.5 - 1.0 x 10e9 /L	<1000 - 500/mm3; <1.0 - 0.5 x 10e9 /L	<500/mm3; <0.5 x 10e9 /L	-
Pancreatic enzymes decreased	<LLN and asymptomatic	Increase in stool frequency, bulk, or odor; steatorrhea	Sequelae of absorption deficiency	-	-
Platelet count decreased	<LLN - 75,000/mm3; <LLN - 75.0 x 10e9 /L	<75,000 - 50,000/mm3; <75.0 - 50.0 x 10e9 /L	<50,000 - 25,000/mm3; <50.0 - 25.0 x 10e9 /L	<25,000/mm3; <25.0 x 10e9 /L	-
Serum amylase increased	>ULN - 1.5 x ULN	>1.5 - 2.0 x ULN	>2.0 - 5.0 x ULN	>5.0 x ULN	-
Urine output decreased	-	-	Oliguria (<80 ml in 8 hr)	Anuria (<240 ml in 24 hr)	-
Vital capacity abnormal	90 - 75% of predicted value	<75 - 50% of predicted value; limiting instrumental ADL	<50% of predicted value; limiting self care ADL	-	-
Weight gain	5 - <10% from baseline	10 - <20% from baseline	>=20% from baseline	-	-
Weight loss	5 to <10% from baseline; intervention not indicated	10 - <20% from baseline; nutritional support indicated	>=20% from baseline; tube feeding or TPN indicated	-	-
White blood cell decreased	<LLN - 3000/mm3; <LLN - 3.0 x 10e9 /L	<3000 - 2000/mm3; <3.0 - 2.0 x 10e9 /L	<2000 - 1000/mm3; <2.0 - 1.0 x 10e9 /L	<1000/mm3; <1.0 x 10e9 /L	-
Investigations - Other, specify	Asymptomatic or mild symptoms; clinical or diagnostic observations only; intervention not indicated	Moderate; minimal, local or noninvasive intervention indicated; limiting age-appropriate instrumental ADL	Severe or medically significant but not immediately life-threatening; hospitalization or prolongation of existing hospitalization indicated; disabling; limiting self care ADL	Life-threatening consequences; urgent intervention indicated	Death
Metabolism and nutrition disorders	Grade				
Adverse Event	1	2	3	4	5

Acidosis	pH <normal, but **>=**7.3	-	pH <7.3	Life-threatening consequences	Death
Alcohol intolerance	-	Present	Severe symptoms; limiting self care ADL	Life-threatening consequences; urgent intervention indicated	Death
Alkalosis	pH >normal, but <=7.5	-	pH >7.5	Life-threatening consequences	Death
Anorexia	Loss of appetite without alteration in eating habits	Oral intake altered without significant weight loss or malnutrition; oral nutritional supplements indicated	Associated with significant weight loss or malnutrition (e.g., inadequate oral caloric and/or fluid intake); tube feeding or TPN indicated	Life-threatening consequences; urgent intervention indicated	Death
Dehydration	Increased oral fluids indicated; dry mucous membranes; diminished skin turgor	IV fluids indicated <24 hrs	IV fluids or hospitalization indicated	Life-threatening consequences; urgent intervention indicated	Death
Glucose intolerance	Asymptomatic; clinical or diagnostic observations only; intervention not indicated	Symptomatic; dietary modification or oral agent indicated	Severe symptoms; insulin indicated	Life-threatening consequences; urgent intervention indicated	Death
Hypercalcemia	Corrected serum calcium of >ULN - 11.5 mg/dL; >ULN - 2.9 mmol/L; Ionized calcium >ULN - 1.5 mmol/L	Corrected serum calcium of >11.5 - 12.5 mg/dL; >2.9 - 3.1 mmol/L; Ionized calcium >1.5 - 1.6 mmol/L; symptomatic	Corrected serum calcium of >12.5 - 13.5 mg/dL;>3.1 - 3.4 mmol/L; Ionized calcium >1.6 - 1.8 mmol/L; hospitalization indicated	Corrected serum calcium of >13.5 mg/dL; >3.4 mmol/L; Ionized calcium >1.8 mmol/L; life-threatening consequences	Death
Hyperglycemia	Fasting glucose value >ULN - 160 mg/dL; Fasting glucose value >ULN - 8.9 mmol/L	Fasting glucose value >160 - 250 mg/dL; Fasting glucose value >8.9 - 13.9 mmol/L	>250 - 500 mg/dL; >13.9 - 27.8 mmol/L; hospitalization indicated	>500 mg/dL; >27.8 mmol/L; life-threatening consequences	Death
Hyperkalemia	>ULN - 5.5 mmol/L	>5.5 - 6.0 mmol/L	>6.0 - 7.0 mmol/L; hospitalization indicated	>7.0 mmol/L; life-threatening consequences	Death
Hypermagnesemia	>ULN - 3.0 mg/dL; >ULN - 1.23 mmol/L	-	>3.0 - 8.0 mg/dL; >1.23 - 3.30 mmol/L	>8.0 mg/dL; >3.30 mmol/L; life-threatening consequences	Death
Hypernatremia	>ULN - 150 mmol/L	>150 - 155 mmol/L	>155 - 160 mmol/L; hospitalization indicated	>160 mmol/L; life-threatening consequences	Death
Hypertriglyceridemia	150 mg/dL - 300 mg/dL; 1.71 mmol/L - 3.42 mmol/L	>300 mg/dL - 500 mg/dL; >3.42 mmol/L - 5.7 mmol/L	>500 mg/dL - 1000 mg/dL; >5.7 mmol/L - 11.4 mmol/L	>1000 mg/dL; >11.4 mmol/L; life-threatening consequences	Death

Hyperuricemia	>ULN - 10 mg/dL (0.59 mmol/L) without physiologic consequences	-	>ULN - 10 mg/dL (0.59 mmol/L) with physiologic consequences	>10 mg/dL; >0.59 mmol/L; life-threatening consequences	Death
Hypoalbuminemia	<LLN - 3 g/dL; <LLN - 30 g/L	<3 - 2 g/dL; <30 - 20 g/L	<2 g/dL; <20 g/L	Life-threatening consequences; urgent intervention indicated	Death
Hypocalcemia	Corrected serum calcium of <LLN - 8.0 mg/dL; <LLN - 2.0 mmol/L; Ionized calcium <LLN - 1.0 mmol/L	Corrected serum calcium of <8.0 - 7.0 mg/dL; <2.0 - 1.75 mmol/L; Ionized calcium <1.0 - 0.9 mmol/L; symptomatic	Corrected serum calcium of <7.0 - 6.0 mg/dL; <1.75 - 1.5 mmol/L; Ionized calcium <0.9 - 0.8 mmol/L; hospitalization indicated	Corrected serum calcium of <6.0 mg/dL; <1.5 mmol/L; Ionized calcium <0.8 mmol/L; life-threatening consequences	Death
Hypoglycemia	<LLN - 55 mg/dL; <LLN - 3.0 mmol/L	<55 - 40 mg/dL; <3.0 - 2.2 mmol/L	<40 - 30 mg/dL; <2.2 - 1.7 mmol/L	<30 mg/dL; <1.7 mmol/L; life-threatening consequences; seizures	Death
Hypokalemia	<LLN - 3.0 mmol/L	<LLN - 3.0 mmol/L; symptomatic; intervention indicated	<3.0 - 2.5 mmol/L; hospitalization indicated	<2.5 mmol/L; life-threatening consequences	Death
Hypomagnesemia	<LLN - 1.2 mg/dL; <LLN - 0.5 mmol/L	<1.2 - 0.9 mg/dL; <0.5 - 0.4 mmol/L	<0.9 - 0.7 mg/dL; <0.4 - 0.3 mmol/L	<0.7 mg/dL; <0.3 mmol/L; life-threatening consequences	Death
Hyponatremia	<LLN - 130 mmol/L	-	<130 - 120 mmol/L	<120 mmol/L; life-threatening consequences	Death
Hypophosphatemia	<LLN - 2.5 mg/dL; <LLN - 0.8 mmol/L	<2.5 - 2.0 mg/dL; <0.8 - 0.6 mmol/L	<2.0 - 1.0 mg/dL; <0.6 - 0.3 mmol/L	<1.0 mg/dL; <0.3 mmol/L; life-threatening consequences	Death
Iron overload	-	Moderate symptoms; intervention not indicated	Severe symptoms; intervention indicated	Life-threatening consequences; urgent intervention indicated	Death
Obesity	-	BMI 25 - 29.9 kg/m2	BMI 30 - 39.9 kg/m2	BMI >=40 kg/m2	
Tumor lysis syndrome	-	-	Present	Life-threatening consequences; urgent intervention indicated	Death
Metabolism and nutrition disorders - Other, specify	Asymptomatic or mild symptoms; clinical or diagnostic observations only; intervention not indicated	Moderate; minimal, local or noninvasive intervention indicated; limiting age-appropriate instrumental ADL	Severe or medically significant but not immediately life-threatening; hospitalization or prolongation of existing hospitalization indicated; disabling; limiting self care ADL	Life-threatening consequences; urgent intervention indicated	Death
Musculoskeletal and connective tissue	Grade				

disorders					
Adverse Event	1	2	3	4	5
Abdominal soft tissue necrosis	-	Local wound care; medical intervention indicated (e.g., dressings or topical medications)	Operative debridement or other invasive intervention indicated (e.g. tissue reconstruction, flap or grafting)	Life-threatening consequences; urgent intervention indicated	Death
Arthralgia	Mild pain	Moderate pain; limiting instrumental ADL	Severe pain; limiting self care ADL	-	-
Arthritis	Mild pain with inflammation, erythema, or joint swelling	Moderate pain associated with signs of inflammation, erythema, or joint swelling; limiting instrumental ADL	Severe pain associated with signs of inflammation, erythema, or joint swelling; irreversible joint damage; disabling; limiting self care ADL	-	-
Avascular necrosis	Asymptomatic; clinical or diagnostic observations only; intervention not indicated	Symptomatic; limiting instrumental ADL	Severe symptoms; limiting self care ADL; elective operative intervention indicated	Life-threatening consequences; urgent intervention indicated	Death
Back pain	Mild pain	Moderate pain; limiting instrumental ADL	Severe pain; limiting self care ADL	-	-
Bone pain	Mild pain	Moderate pain; limiting instrumental ADL	Severe pain; limiting self care ADL	-	-
Buttock pain	Mild pain	Moderate pain; limiting instrumental ADL	Severe pain; limiting self care ADL	-	-
Chest wall pain	Mild pain	Moderate pain; limiting instrumental ADL	Severe pain; limiting self care ADL	-	-
Exostosis	Asymptomatic; clinical or diagnostic observations only; intervention not indicated	Symptomatic; limiting instrumental ADL	Severe symptoms; limiting self care ADL; elective operative intervention indicated	-	-
Fibrosis deep connective tissue	Mild induration, able to move skin parallel to plane (sliding) and perpendicular to skin (pinching up)	Moderate induration, able to slide skin, unable to pinch skin; limiting instrumental ADL	Severe induration; unable to slide or pinch skin; limiting joint or orifice movement (e.g. mouth, anus); limiting self care ADL	Generalized; associated with signs or symptoms of impaired breathing or feeding	Death
Flank pain	Mild pain	Moderate pain; limiting instrumental ADL	Severe pain; limiting self care ADL	-	-

Generalized muscle weakness	Symptomatic; weakness perceived by patient but not evident on physical exam	Symptomatic; weakness evident on physical exam; weakness limiting instrumental ADL	Weakness limiting self care ADL; disabling	-	-
Growth suppression	Reduction in growth velocity by 10 - 29% ideally measured over the period of a year	Reduction in growth velocity by 30 - 49% ideally measured over the period of a year or 0 - 49% reduction in growth from the baseline growth curve	Reduction in growth velocity of >=50% ideally measured over the period of a year	-	-
Head soft tissue necrosis	-	Local wound care; medical intervention indicated (e.g., dressings or topical medications)	Operative debridement or other invasive intervention indicated (e.g., tissue reconstruction, flap or grafting)	Life-threatening consequences; urgent intervention indicated	Death
Joint effusion	Asymptomatic; clinical or diagnostic observations only; intervention not indicated	Symptomatic; limiting instrumental ADL	Severe symptoms; limiting self care ADL; elective operative intervention indicated; disabling	-	-
Joint range of motion decreased	<=25% loss of ROM (range of motion); decreased ROM limiting athletic activity	>25 - 50% decrease in ROM; limiting instrumental ADL	>50% decrease in ROM; limiting self care ADL; disabling	-	-
Joint range of motion decreased cervical spine	Mild restriction of rotation or flexion between 60 - 70 degrees	Rotation <60 degrees to right or left; <60 degrees of flexion	Ankylosed/fused over multiple segments with no C-spine rotation	-	-
Joint range of motion decreased lumbar spine	Stiffness; difficulty bending to the floor to pick up a very light object but able to do athletic activity	Pain with range of motion (ROM) in lumbar spine; requires a reaching aid to pick up a very light object from the floor	<50% lumbar spine flexion; associated with symptoms of ankylosis or fused over multiple segments with no L-spine flexion (e.g., unable to reach to floor to pick up a very light object)	-	-
Kyphosis	Asymptomatic; clinical or diagnostic observations only; intervention not indicated	Moderate accentuation; limiting instrumental ADL	Severe accentuation; operative intervention indicated; limiting self care ADL	-	-
Lordosis	Asymptomatic; clinical or diagnostic observations only; intervention not indicated	Moderate accentuation; limiting instrumental ADL	Severe accentuation; operative intervention indicated; limiting self care ADL	-	-
Muscle weakness left-sided	Symptomatic; perceived by patient but not evident on physical exam	Symptomatic; evident on physical exam; limiting instrumental ADL	Limiting self care ADL; disabling	-	-
Muscle weakness lower limb	Symptomatic; perceived by patient but not evident on physical exam	Symptomatic; evident on physical exam; limiting instrumental ADL	Limiting self care ADL; disabling	-	-

Muscle weakness right-sided	Symptomatic; perceived by patient but not evident on physical exam	Symptomatic; evident on physical exam; limiting instrumental ADL	Limiting self care ADL; disabling	-	-
Muscle weakness trunk	Symptomatic; perceived by patient but not evident on physical exam	Symptomatic; evident on physical exam; limiting instrumental ADL	Limiting self care ADL; disabling	-	-
Muscle weakness upper limb	Symptomatic; perceived by patient but not evident on physical exam	Symptomatic; evident on physical exam; limiting instrumental ADL	Limiting self care ADL; disabling	-	-
Musculoskeletal deformity	Cosmetically and functionally insignificant hypoplasia	Deformity, hypoplasia, or asymmetry able to be remediated by prosthesis (e.g., shoe insert) or covered by clothing	Significant deformity, hypoplasia, or asymmetry, unable to be remediated by prosthesis or covered by clothing; disabling	-	-
Myalgia	Mild pain	Moderate pain; limiting instrumental ADL	Severe pain; limiting self care ADL	-	-
Myositis	Mild pain	Moderate pain associated with weakness; pain limiting instrumental ADL	Pain associated with severe weakness; limiting self care ADL	-	-
Neck pain	Mild pain	Moderate pain; limiting instrumental ADL	Severe pain; limiting self care ADL	-	-
Neck soft tissue necrosis	-	Local wound care; medical intervention indicated (e.g., dressings or topical medications)	Operative debridement or other invasive intervention indicated (e.g., tissue reconstruction, flap or grafting)	Life-threatening consequences; urgent intervention indicated	Death
Osteonecrosis of jaw	Asymptomatic; clinical or diagnostic observations only; intervention not indicated	Symptomatic; medical intervention indicated (e.g., topical agents); limiting instrumental ADL	Severe symptoms; limiting self care ADL; elective operative intervention indicated	Life-threatening consequences; urgent intervention indicated	Death
Osteoporosis	Radiologic evidence of osteoporosis or Bone Mineral Density (BMD) t-score -1 to -2.5 (osteopenia); no loss of height or intervention indicated	BMD t-score <-2.5; loss of height <2 cm; anti-osteoporotic therapy indicated; limiting instrumental ADL	Loss of height >=2 cm; hospitalization indicated; limiting self care ADL	-	-
Pain in extremity	Mild pain	Moderate pain; limiting instrumental ADL	Severe pain; limiting self care ADL	-	-

Pelvic soft tissue necrosis	-	Local wound care; medical intervention indicated (e.g., dressings or topical medications)	Operative debridement or other invasive intervention indicated (e.g., tissue reconstruction, flap or grafting)	Life-threatening consequences; urgent intervention indicated	Death
Scoliosis	<20 degrees; clinically undetectable	>20 - 45 degrees; visible by forward flexion; limiting instrumental ADL	>45 degrees; scapular prominence in forward flexion; operative intervention indicated; limiting self care ADL; disabling	-	-
Soft tissue necrosis lower limb	-	Local wound care; medical intervention indicated (e.g., dressings or topical medications)	Operative debridement or other invasive intervention indicated (e.g., tissue reconstruction, flap or grafting)	Life-threatening consequences; urgent intervention indicated	Death
Soft tissue necrosis upper limb	-	Local wound care; medical intervention indicated (e.g., dressings or topical medications)	Operative debridement or other invasive intervention indicated (e.g., tissue reconstruction, flap or grafting)	Life-threatening consequences; urgent intervention indicated	Death
Superficial soft tissue fibrosis	Mild induration, able to move skin parallel to plane (sliding) and perpendicular to skin (pinching up)	Moderate induration, able to slide skin, unable to pinch skin; limiting instrumental ADL	Severe induration; unable to slide or pinch skin; limiting joint or orifice movement (e.g., mouth, anus); limiting self care ADL	Generalized; associated with signs or symptoms of impaired breathing or feeding	Death
Trismus	Decreased ROM (range of motion) without impaired eating	Decreased ROM requiring small bites, soft foods or purees	Decreased ROM with inability to adequately aliment or hydrate orally	-	-
Unequal limb length	Mild length discrepancy <2 cm	Moderate length discrepancy 2 - 5 cm; shoe lift indicated; limiting instrumental ADL	Severe length discrepancy >5 cm; limiting self care ADL; disabling; operative intervention indicated	-	-
Musculoskeletal and connective tissue disorder - Other, specify	Asymptomatic or mild symptoms; clinical or diagnostic observations only; intervention not indicated	Moderate; minimal, local or noninvasive intervention indicated; limiting age-appropriate instrumental ADL	Severe or medically significant but not immediately life-threatening; hospitalization or prolongation of existing hospitalization indicated; disabling; limiting self care ADL	Life-threatening consequences; urgent intervention indicated	Death
Neoplasms benign, malignant and unspecified (incl cysts and polyps)	Grade				
Adverse Event	1	2	3	4	5
Leukemia secondary to oncology chemotherapy	-	-	-	Present	Death
Myelodysplastic syndrome	-	-	-	Life-threatening consequences; urgent intervention indicated	Death

Treatment related secondary malignancy	-	-	Non life-threatening secondary malignancy	Acute life-threatening secondary malignancy; blast crisis in leukemia	Death
Tumor pain	Mild pain	Moderate pain; limiting instrumental ADL	Severe pain; limiting self care ADL	-	-
Neoplasms benign, malignant and unspecified (incl cysts and polyps) - Other, specify	Asymptomatic or mild symptoms; clinical or diagnostic observations only; intervention not indicated	Moderate; minimal, local or noninvasive intervention indicated; limiting age-appropriate instrumental ADL	Severe or medically significant but not immediately life-threatening; hospitalization or prolongation of existing hospitalization indicated; disabling; limiting self care ADL	Life-threatening consequences; urgent intervention indicated	Death
Nervous system disorders	Grade				
Adverse Event	1	2	3	4	5
Abducens nerve disorder	Asymptomatic; clinical or diagnostic observations only; intervention not indicated	Moderate symptoms; limiting instrumental ADL	Severe symptoms; limiting self care ADL	-	-
Accessory nerve disorder	Asymptomatic; clinical or diagnostic observations only; intervention not indicated	Moderate symptoms; limiting instrumental ADL	Severe symptoms; limiting self care ADL	-	-
Acoustic nerve disorder NOS	Asymptomatic; clinical or diagnostic observations only; intervention not indicated	Moderate symptoms; limiting instrumental ADL	Severe symptoms; limiting self care ADL	-	-
Akathisia	Mild restlessness or increased motor activity	Moderate restlessness or increased motor activity; limiting instrumental ADL	Severe restlessness or increased motor activity; limiting self care ADL	-	-
Amnesia	Mild; transient memory loss	Moderate; short term memory loss; limiting instrumental ADL	Severe; long term memory loss; limiting self care ADL	-	-
Aphonia	-	-	Voicelessness; unable to speak	-	-
Arachnoiditis	Mild symptoms	Moderate symptoms; limiting instrumental ADL	Severe symptoms; limiting self care ADL	Life-threatening consequences; urgent intervention indicated	Death
Ataxia	Asymptomatic; clinical or diagnostic observations only; intervention not indicated	Moderate symptoms; limiting instrumental ADL	Severe symptoms; limiting self care ADL; mechanical assistance indicated	-	-
Brachial plexopathy	Asymptomatic; clinical or diagnostic observations only; intervention not indicated	Moderate symptoms; limiting instrumental ADL	Severe symptoms; limiting self care ADL	-	-
Central nervous system necrosis	Asymptomatic; clinical or diagnostic observations only; intervention not indicated	Moderate symptoms; corticosteroids indicated	Severe symptoms; medical intervention indicated	Life-threatening consequences; urgent intervention indicated	Death

Cerebrospinal fluid leakage	Post-craniotomy: asymptomatic; Post-lumbar puncture: transient headache; postural care indicated	Post-craniotomy: moderate symptoms; medical intervention indicated; Post-lumbar puncture: persistent moderate symptoms; blood patch indicated	Severe symptoms; medical intervention indicated	Life-threatening consequences; urgent intervention indicated	Death
Cognitive disturbance	Mild cognitive disability; not interfering with work/school/life performance; specialized educational services/devices not indicated	Moderate cognitive disability; interfering with work/school/life performance but capable of independent living; specialized resources on part time basis indicated	Severe cognitive disability; significant impairment of work/school/life performance	-	-
Concentration impairment	Mild inattention or decreased level of concentration	Moderate impairment in attention or decreased level of concentration; limiting instrumental ADL	Severe impairment in attention or decreased level of concentration; limiting self care ADL	-	-
Depressed level of consciousness	Decreased level of alertness	Sedation; slow response to stimuli; limiting instrumental ADL	Difficult to arouse	Life-threatening consequences	Death
Dizziness	Mild unsteadiness or sensation of movement	Moderate unsteadiness or sensation of movement; limiting instrumental ADL	Severe unsteadiness or sensation of movement; limiting self care ADL	-	-
Dysarthria	Mild slurred speech	Moderate impairment of articulation or slurred speech	Severe impairment of articulation or slurred speech	-	-
Dysesthesia	Mild sensory alteration	Moderate sensory alteration; limiting instrumental ADL	Severe sensory alteration; limiting self care ADL	-	-
Dysgeusia	Altered taste but no change in diet	Altered taste with change in diet (e.g., oral supplements); noxious or unpleasant taste; loss of taste	-	-	-
Dysphasia	Awareness of receptive or expressive characteristics; not impairing ability to communicate	Moderate receptive or expressive characteristics; impairing ability to communicate spontaneously	Severe receptive or expressive characteristics; impairing ability to read, write or communicate intelligibly	-	-
Edema cerebral	-	-	-	Life-threatening consequences; urgent intervention indicated	
Encephalopathy	Mild symptoms	Moderate symptoms; limiting instrumental ADL	Severe symptoms; limiting self care ADL	Life-threatening consequences; urgent intervention indicated	Death
Extrapyramidal disorder	Mild involuntary movements	Moderate involuntary movements; limiting instrumental ADL	Severe involuntary movements or torticollis; limiting self care ADL	Life-threatening consequences; urgent intervention indicated	Death
Facial muscle weakness	Asymptomatic; clinical or diagnostic observations only; intervention not indicated	Moderate symptoms; limiting instrumental ADL	Severe symptoms; limiting self care ADL	-	-

Facial nerve disorder	Asymptomatic; clinical or diagnostic observations only; intervention not indicated	Moderate symptoms; limiting instrumental ADL	Severe symptoms; limiting self care ADL	-	-
Glossopharyngeal nerve disorder	Asymptomatic; clinical or diagnostic observations only; intervention not indicated	Moderate symptoms; limiting instrumental ADL	Severe symptoms; limiting self care ADL	Life-threatening consequences; urgent intervention indicated	Death
Headache	Mild pain	Moderate pain; limiting instrumental ADL	Severe pain; limiting self care ADL	-	-
Hydrocephalus	Asymptomatic; clinical or diagnostic observations only; intervention not indicated	Moderate symptoms; intervention not indicated	Severe symptoms or neurological deficit; intervention indicated	Life-threatening consequences; urgent intervention indicated	Death
Hypersomnia	Mild increased need for sleep	Moderate increased need for sleep	Severe increased need for sleep	-	-
Hypoglossal nerve disorder	Asymptomatic; clinical or diagnostic observations only; intervention not indicated	Moderate symptoms; limiting instrumental ADL	Severe symptoms; limiting self care ADL	-	-
Intracranial hemorrhage	Asymptomatic; clinical or diagnostic observations only; intervention not indicated	Moderate symptoms; medical intervention indicated	Ventriculostomy, ICP monitoring, intraventricular thrombolysis, or operative intervention indicated	Life-threatening consequences; urgent intervention indicated	Death
Ischemia cerebrovascular	Asymptomatic; clinical or diagnostic observations only; intervention not indicated	Moderate symptoms	-	-	-
IVth nerve disorder	Asymptomatic; clinical or diagnostic observations only; intervention not indicated	Moderate symptoms; limiting instrumental ADL	Severe symptoms; limiting self care ADL	-	-
Lethargy	Mild symptoms; reduced alertness and awareness	Moderate symptoms; limiting instrumental ADL	-	-	-
Leukoencephalopathy	Asymptomatic; small focal T2/FLAIR hyperintensities; involving periventricular white matter or <1/3 of susceptible areas of cerebrum +/- mild increase in subarachnoid space (SAS) and/or mild ventriculomegaly	Moderate symptoms; focal T2/FLAIR hyperintensities, involving periventricular white matter extending into centrum semiovale or involving 1/3 to 2/3 of susceptible areas of cerebrum +/- moderate increase in SAS and/or moderate ventriculomegaly	Severe symptoms; extensive T2/FLAIR hyperintensities, involving periventricular white matter involving 2/3 or more of susceptible areas of cerebrum +/- moderate to severe increase in SAS and/or moderate to severe ventriculomegaly	Life-threatening consequences; extensive T2/FLAIR hyperintensities, involving periventricular white matter involving most of susceptible areas of cerebrum +/- moderate to severe increase in SAS and/or moderate to severe ventriculomegaly	Death
Memory impairment	Mild memory impairment	Moderate memory impairment; limiting instrumental ADL	Severe memory impairment; limiting self care ADL	-	-
Meningismus	Mild symptoms	Moderate symptoms; limiting instrumental ADL	Severe symptoms; limiting self care ADL	Life-threatening consequences; urgent intervention indicated	Death

Movements involuntary	Mild symptoms	Moderate symptoms; limiting instrumental ADL	Severe symptoms; limiting self care ADL	-	-
Myelitis	Asymptomatic; mild signs (e.g., Babinski's reflex or Lhermitte's sign)	Moderate weakness or sensory loss; limiting instrumental ADL	Severe weakness or sensory loss; limiting self care ADL	Life-threatening consequences; urgent intervention indicated	Death
Neuralgia	Mild pain	Moderate pain; limiting instrumental ADL	Severe pain; limiting self care ADL	-	-
Nystagmus	-	Moderate symptoms; limiting instrumental ADL	Severe symptoms; limiting self care ADL	-	-
Oculomotor nerve disorder	Asymptomatic; clinical or diagnostic observations only; intervention not indicated	Moderate symptoms; limiting instrumental ADL	Severe symptoms; limiting self care ADL	-	-
Olfactory nerve disorder	-	Moderate symptoms; limiting instrumental ADL	Severe symptoms; limiting self care ADL	-	-
Paresthesia	Mild symptoms	Moderate symptoms; limiting instrumental ADL	Severe symptoms; limiting self care ADL	-	-
Peripheral motor neuropathy	Asymptomatic; clinical or diagnostic observations only; intervention not indicated	Moderate symptoms; limiting instrumental ADL	Severe symptoms; limiting self care ADL; assistive device indicated	Life-threatening consequences; urgent intervention indicated	Death
Peripheral sensory neuropathy	Asymptomatic; loss of deep tendon reflexes or paresthesia	Moderate symptoms; limiting instrumental ADL	Severe symptoms; limiting self care ADL	Life-threatening consequences; urgent intervention indicated	Death
Phantom pain	Mild pain	Moderate pain; limiting instrumental ADL	Severe pain; limiting self care ADL	-	-
Presyncope	-	Present (e.g., near fainting)	-	-	-
Pyramidal tract syndrome	Asymptomatic; clinical or diagnostic observations only; intervention not indicated	Moderate symptoms; limiting instrumental ADL	Severe symptoms; limiting self care ADL	Life-threatening consequences; urgent intervention indicated	Death
Radiculitis	Mild symptoms	Moderate symptoms; limiting instrumental ADL; medical intervention indicated	Severe symptoms; limiting self care ADL	Life-threatening consequences; urgent intervention indicated	Death
Recurrent laryngeal nerve palsy	Asymptomatic; clinical or diagnostic observations only; intervention not indicated	Moderate symptoms	Severe symptoms; medical intervention indicated (e.g., thyroplasty, vocal cord injection)	Life-threatening consequences; urgent intervention indicated	Death
Reversible posterior leukoencephalopathy syndrome	Asymptomatic; clinical or diagnostic observations only; intervention not indicated	Moderate symptoms; abnormal imaging studies; limiting instrumental ADL	Severe symptoms; very abnormal imaging studies; limiting self care ADL	Life-threatening consequences; urgent intervention indicated	Death

Seizure	Brief partial seizure; no loss of consciousness	Brief generalized seizure	Multiple seizures despite medical intervention	Life-threatening; prolonged repetitive seizures	Death
Sinus pain	Mild pain	Moderate pain; limiting instrumental ADL	Severe pain; limiting self care ADL	-	-
Somnolence	Mild but more than usual drowsiness or sleepiness	Moderate sedation; limiting instrumental ADL	Obtundation or stupor	Life-threatening consequences; urgent intervention indicated	Death
Spasticity	Mild or slight increase in muscle tone	Moderate increase in muscle tone and increase in resistance through range of motion	Severe increase in muscle tone and increase in resistance through range of motion	Life-threatening; unable to move active or passive range of motion	Death
Stroke	Asymptomatic or mild neurologic deficit; radiographic findings only	Moderate neurologic deficit	Severe neurologic deficit	Life-threatening consequences; urgent intervention indicated	Death
Syncope	-	-	Fainting; orthostatic collapse	-	-
Transient ischemic attacks	Mild neurologic deficit with or without imaging confirmation	Moderate neurologic deficit with or without imaging confirmation	-	-	-
Tremor	Mild symptoms	Moderate symptoms; limiting instrumental ADL	Severe symptoms; limiting self care ADL	-	-
Trigeminal nerve disorder	Asymptomatic; clinical or diagnostic observations only; intervention not indicated	Moderate symptoms; limiting instrumental ADL	Severe symptoms; limiting self care ADL	-	-
Vagus nerve disorder	Asymptomatic; clinical or diagnostic observations only; intervention not indicated	Moderate symptoms; limiting instrumental ADL	Severe symptoms; limiting self care ADL	Life-threatening consequences; urgent intervention indicated	Death
Vasovagal reaction	-	-	Present	Life-threatening consequences; urgent intervention indicated	Death
Nervous system disorders - Other, specify	Asymptomatic or mild symptoms; clinical or diagnostic observations only; intervention not indicated	Moderate; minimal, local or noninvasive intervention indicated; limiting age-appropriate instrumental ADL	Severe or medically significant but not immediately life-threatening; hospitalization or prolongation of existing hospitalization indicated; disabling; limiting self care ADL	Life-threatening consequences; urgent intervention indicated	Death
Pregnancy, puerperium and perinatal conditions	Grade				
Adverse Event	1	2	3	4	5
Fetal death	-	-	-	-	Fetal loss at any gestational age

Fetal growth retardation	-	<10% percentile of weight for gestational age	<5% percentile of weight for gestational age	<1% percentile of weight for gestational age	-
Premature delivery	Delivery of a liveborn infant at >34 to 37 weeks gestation	Delivery of a liveborn infant at >28 to 34 weeks gestation	Delivery of a liveborn infant at 24 to 28 weeks gestation	Delivery of a liveborn infant at 24 weeks of gestation or less	-
Unintended pregnancy	-	-	Unintended pregnancy	-	-
Pregnancy, puerperium and perinatal conditions - Other, specify	Asymptomatic or mild symptoms; clinical or diagnostic observations only; intervention not indicated	Moderate, local or noninvasive intervention indicated; limiting instrumental ADL	Severe or medically significant but not immediately life-threatening; hospitalization or prolongation of existing hospitalization indicated; disabling; limiting self care ADL	Life-threatening consequences; urgent intervention indicated	Death
Psychiatric disorders	Grade				
Adverse Event	1	2	3	4	5
Agitation	Mild mood alteration	Moderate mood alteration	Severe agitation; hospitalization not indicated	Life-threatening consequences; urgent intervention indicated	Death
Anorgasmia	Inability to achieve orgasm not adversely affecting relationship	Inability to achieve orgasm adversely affecting relationship	-	-	-
Anxiety	Mild symptoms; intervention not indicated	Moderate symptoms; limiting instrumental ADL	Severe symptoms; limiting self care ADL; hospitalization not indicated	Life-threatening; hospitalization indicated	Death
Confusion	Mild disorientation	Moderate disorientation; limiting instrumental ADL	Severe disorientation; limiting self care ADL	Life-threatening consequences; urgent intervention indicated	Death
Delayed orgasm	Delay in achieving orgasm not adversely affecting relationship	Delay in achieving orgasm adversely affecting relationship	-	-	-
Delirium	Mild acute confusional state	Moderate and acute confusional state; limiting instrumental ADL	Severe and acute confusional state; limiting self care ADL; hospitalization indicated	Life-threatening consequences, threats of harm to self or others; hospitalization indicated	Death
Delusions	-	Moderate delusional symptoms	Severe delusional symptoms; hospitalization not indicated	Life-threatening consequences, threats of harm to self or others; hospitalization indicated	Death
Depression	Mild depressive symptoms	Moderate depressive symptoms; limiting instrumental ADL	Severe depressive symptoms; limiting self care ADL; hospitalization not indicated	Life-threatening consequences, threats of harm to self or others; hospitalization indicated	Death
Euphoria	Mild mood elevation	Moderate mood elevation	Severe mood elevation (e.g., hypomania)	-	-
Hallucinations	Mild hallucinations (e.g., perceptual distortions)	Moderate hallucinations	Severe hallucinations; hospitalization not indicated	Life-threatening consequences, threats of harm to self or others; hospitalization indicated	Death

Insomnia	Mild difficulty falling asleep, staying asleep or waking up early	Moderate difficulty falling asleep, staying asleep or waking up early	Severe difficulty in falling asleep, staying asleep or waking up early	-	-
Libido decreased	Decrease in sexual interest not adversely affecting relationship	Decrease in sexual interest adversely affecting relationship	-	-	-
Libido increased	Mild increase in sexual interest not adversely affecting relationship	Moderate increase in sexual interest adversely affecting relationship	Severe increase in sexual interest leading to dangerous behavior	-	-
Mania	Mild manic symptoms (e.g., elevated mood, rapid thoughts, rapid speech, decreased need for sleep)	Moderate manic symptoms (e.g., relationship and work difficulties; poor hygiene)	Severe manic symptoms (e.g., hypomania; major sexual or financial indiscretions); hospitalization not indicated	Life-threatening consequences, threats of harm to self or others; hospitalization indicated	Death
Personality change	Mild personality change	Moderate personality change	Severe personality change; hospitalization not indicated	Life-threatening consequences, threats of harm to self or others; hospitalization indicated	Death
Psychosis	Mild psychotic symptoms	Moderate psychotic symptoms (e.g., disorganized speech; impaired reality testing)	Severe psychotic symptoms (e.g., paranoid; extreme disorganization); hospitalization not indicated	Life-threatening consequences, threats of harm to self or others; hospitalization indicated	Death
Restlessness	Mild symptoms; intervention not indicated	Moderate symptoms; limiting instrumental ADL	Severe symptoms; limiting self care ADL	-	-
Suicidal ideation	Increased thoughts of death but no wish to kill oneself	Suicidal ideation with no specific plan or intent	Specific plan to commit suicide without serious intent to die which may not require hospitalization	Specific plan to commit suicide with serious intent to die which requires hospitalization	-
Suicide attempt	-	-	Suicide attempt or gesture without intent to die which may not require hospitalization	Suicide attempt with intent to die which requires hospitalization	Death
Psychiatric disorders - Other, specify	Asymptomatic or mild symptoms; clinical or diagnostic observations only; intervention not indicated	Moderate; minimal, local or noninvasive intervention indicated; limiting age-appropriate instrumental ADL	Severe or medically significant but not immediately life-threatening; disabling; limiting self care ADL	Life-threatening consequences; hospitalization or urgent intervention indicated	Death

Renal and urinary disorders	Grade				
Adverse Event	1	2	3	4	5
Acute kidney injury	Creatinine level increase of >0.3 mg/dL; creatinine 1.5 - 2.0 x above baseline	Creatinine 2 - 3 x above baseline	Creatinine >3 x baseline or >4.0 mg/dL; hospitalization indicated	Life-threatening consequences; dialysis indicated	Death
Bladder perforation	-	Extraperitoneal perforation, indwelling catheter indicated	Intraperitoneal perforation; elective radiologic, endoscopic or operative intervention indicated	Life-threatening consequences; organ failure; urgent operative intervention indicated	Death
Bladder spasm	Intervention not indicated	Antispasmodics indicated	Hospitalization indicated	-	-

Chronic kidney disease	eGFR (estimated Glomerular Filtration Rate) or CrCl (creatinine clearance) <LLN - 60 ml/min/1.73 m2 or proteinuria 2+ present; urine protein/creatinine >0.5	eGFR or CrCl 59 - 30 ml/min/1.73 m2	eGFR or CrCl 29 - 15 ml/min/1.73 m2	eGFR or CrCl <15 ml/min/1.73 m2; dialysis or renal transplant indicated	Death
Cystitis noninfective	Microscopic hematuria; minimal increase in frequency, urgency, dysuria, or nocturia; new onset of incontinence	Moderate hematuria; moderate increase in frequency, urgency, dysuria, nocturia or incontinence; urinary catheter placement or bladder irrigation indicated; limiting instrumental ADL	Gross hematuria; transfusion, IV medications or hospitalization indicated; elective endoscopic, radiologic or operative intervention indicated	Life-threatening consequences; urgent radiologic or operative intervention indicated	Death
Hematuria	Asymptomatic; clinical or diagnostic observations only; intervention not indicated	Symptomatic; urinary catheter or bladder irrigation indicated; limiting instrumental ADL	Gross hematuria; transfusion, IV medications or hospitalization indicated; elective endoscopic, radiologic or operative intervention indicated; limiting self care ADL	Life-threatening consequences; urgent radiologic or operative intervention indicated	Death
Hemoglobinuria	Asymptomatic; clinical or diagnostic observations only; intervention not indicated	-	-	-	-
Proteinuria	1+ proteinuria; urinary protein <1.0 g/24 hrs	Adults: 2+ proteinuria; urinary protein 1.0 - 3.4 g/24 hrs; Pediatric: urine P/C (Protein/Creatinine) ratio 0.5 - 1.9	Adults: urinary protein >=3.5 g/24 hrs;<br><br>Pediatric: urine P/C >1.9	-	-
Renal calculi	Asymptomatic or mild symptoms; occasional use of nonprescription analgesics indicated	Symptomatic; oral antiemetics indicated; around the clock nonprescription analgesics or any oral narcotic analgesics indicated	Hospitalization indicated; IV intervention (e.g., analgesics, antiemetics); elective endoscopic or radiologic intervention indicated	Life-threatening consequences; urgent radiologic, endoscopic or operative intervention indicated	Death
Renal colic	Mild pain not interfering with activity; nonprescription medication indicated	Moderate pain; limiting instrumental ADL; prescription medication indicated	Hospitalization indicated; limiting self care ADL	-	-
Renal hemorrhage	Mild symptoms; intervention not indicated	Analgesics and hematocrit monitoring indicated	Transfusion, radiation, or hospitalization indicated; elective radiologic, endoscopic or operative intervention indicated	Life-threatening consequences; urgent radiologic or operative intervention indicated	Death
Urinary fistula	-	Noninvasive intervention indicated; urinary or suprapubic catheter placement indicated	Limiting self care ADL; elective radiologic, endoscopic or operative intervention indicated; permanent urinary diversion indicated	Life-threatening consequences; urgent radiologic or operative intervention indicated	Death
Urinary frequency	Present	Limiting instrumental ADL; medical management indicated	-	-	-

Urinary incontinence	Occasional (e.g., with coughing, sneezing, etc.), pads not indicated	Spontaneous; pads indicated; limiting instrumental ADL	Intervention indicated (e.g., clamp, collagen injections); operative intervention indicated; limiting self care ADL	-	-
Urinary retention	Urinary, suprapubic or intermittent catheter placement not indicated; able to void with some residual	Placement of urinary, suprapubic or intermittent catheter placement indicated; medication indicated	Elective operative or radiologic intervention indicated; substantial loss of affected kidney function or mass	Life-threatening consequences; organ failure; urgent operative intervention indicated	Death
Urinary tract obstruction	Asymptomatic; clinical or diagnostic observations only	Symptomatic but no hydronephrosis, sepsis or renal dysfunction; urethral dilation, urinary or suprapubic catheter indicated	Symptomatic and altered organ function (e.g., hydronephrosis, or renal dysfunction); elective radiologic, endoscopic or operative intervention indicated	Life-threatening consequences; urgent intervention indicated	Death
Urinary tract pain	Mild pain	Moderate pain; limiting instrumental ADL	Severe pain; limiting self care ADL	-	-
Urinary urgency	Present	Limiting instrumental ADL; medical management indicated	-	-	-
Urine discoloration	Present	-	-	-	-
Renal and urinary disorders - Other, specify	Asymptomatic or mild symptoms; clinical or diagnostic observations only; intervention not indicated	Moderate, local or noninvasive intervention indicated; limiting instrumental ADL	Severe or medically significant but not immediately life-threatening; hospitalization or prolongation of existing hospitalization indicated; disabling; limiting self care ADL	Life-threatening consequences; urgent intervention indicated	Death
Reproductive system and breast disorders			Grade		
Adverse Event	1	2	3	4	5
Azoospermia	-	-	Absence of sperm in ejaculate	-	-
Breast atrophy	Minimal asymmetry; minimal atrophy	Moderate asymmetry; moderate atrophy	Asymmetry >1/3 of breast volume; severe atrophy	-	-
Breast pain	Mild pain	Moderate pain; limiting instrumental ADL	Severe pain; limiting self care ADL	-	-
Dysmenorrhea	Mild symptoms; intervention not indicated	Moderate symptoms; limiting instrumental ADL	Severe symptoms; limiting self care ADL	-	-

Dyspareunia	Mild discomfort or pain associated with vaginal penetration; discomfort relieved with use of vaginal lubricants or estrogen	Moderate discomfort or pain associated with vaginal penetration; discomfort or pain partially relieved with use of vaginal lubricants or estrogen	Severe discomfort or pain associated with vaginal penetration; discomfort or pain unrelieved by vaginal lubricants or estrogen	-	-
Ejaculation disorder	Diminished ejaculation	Anejaculation or retrograde ejaculation	-	-	-
Erectile dysfunction	Decrease in erectile function (frequency or rigidity of erections) but intervention not indicated (e.g., medication or use of mechanical device, penile pump)	Decrease in erectile function (frequency/rigidity of erections), erectile intervention indicated, (e.g., medication or mechanical devices such as penile pump)	Decrease in erectile function (frequency/rigidity of erections) but erectile intervention not helpful (e.g., medication or mechanical devices such as penile pump); placement of a permanent penile prosthesis indicated (not previously present)	-	-
Fallopian tube obstruction	Diagnostic observations only; intervention not indicated	Mild symptoms; elective intervention indicated	Severe symptoms; elective operative intervention indicated	-	-
Fallopian tube stenosis	Asymptomatic clinical or diagnostic observations only; intervention not indicated	Symptomatic and intervention not indicated	Severe symptoms; elective operative intervention indicated	Life-threatening consequences; urgent operative intervention indicated (e.g., organ resection)	Death
Female genital tract fistula	Asymptomatic clinical or diagnostic observations only; intervention not indicated	Symptomatic and intervention not indicated	Severe symptoms; elective operative intervention indicated	Life-threatening consequences; urgent intervention indicated	Death
Feminization acquired	Mild symptoms; intervention not indicated	Moderate symptoms; medical intervention indicated	-	-	-
Genital edema	Mild swelling or obscuration of anatomic architecture on close inspection	Readily apparent obscuration of anatomic architecture; obliteration of skin folds; readily apparent deviation from normal anatomic contour	Lymphorrhea; gross deviation from normal anatomic contour; limiting self care ADL	-	-
Gynecomastia	Asymptomatic breast enlargement	Symptomatic (e.g., pain or psychosocial impact)	Severe symptoms; elective operative intervention indicated	-	-
Hematosalpinx	Minimal bleeding identified on imaging study or laparoscopy; intervention not indicated	Moderate bleeding; medical intervention indicated	Severe bleeding; transfusion indicated; radiologic or endoscopic intervention indicated	Life-threatening consequences; urgent operative intervention indicated	Death
Irregular menstruation	Intermittent menses with skipped menses for no more than 1 to 3 months	Intermittent menses with skipped menses for more than 4 to 6 months	Persistent amenorrhea for more than 6 months	-	-

Lactation disorder	Mild changes in lactation, not significantly affecting production or expression of breast milk	Changes in lactation, significantly affecting breast production or expression of breast milk	-	-	-
Menorrhagia	Mild; iron supplements indicated	Moderate symptoms; medical intervention indicated (e.g., hormones)	Severe; transfusion indicated; surgical intervention indicated (e.g., hysterectomy)	Life-threatening consequences; urgent intervention indicated	Death
Nipple deformity	Asymptomatic; asymmetry with slight retraction and/or thickening of the nipple areolar complex	Symptomatic; asymmetry of nipple areolar complex with moderate retraction and/or thickening of the nipple areolar complex	-	-	-
Oligospermia	Sperm concentration >48 million/mL or motility >68%	Sperm concentration 13 - 48 million/mL or motility 32 - 68%	Sperm concentration <13 million/mL or motility <32%	-	-
Ovarian hemorrhage	Minimal bleeding identified on imaging study or laproscopy; intervention not indicated	Moderate bleeding; medical intervention indicated	Severe bleeding; transfusion indicated; radiologic or endoscopic intervention indicated	Life-threatening consequences; urgent operative intervention indicated	Death
Ovarian rupture	Asymptomatic clinical or diagnostic observations only; intervention not indicated	Symptomatic and intervention not indicated	Transfusion, radiologic, endoscopic, or elective operative intervention indicated	Life-threatening consequences; urgent intervention indicated	Death
Ovulation pain	Mild pain	Moderate pain; limiting instrumental ADL	Severe pain; limiting self care ADL	-	-
Pelvic floor muscle weakness	Asymptomatic; clinical or diagnostic observations only; intervention not indicated	Symptomatic, not interfering with bladder, bowel, or vaginal function; limiting instrumental ADL	Severe symptoms; limiting self care ADL	Life-threatening consequences; urgent intervention indicated	Death
Pelvic pain	Mild pain	Moderate pain; limiting instrumental ADL	Severe pain; limiting self care ADL	-	-
Penile pain	Mild pain	Moderate pain; limiting instrumental ADL	Severe pain; limiting self care ADL	-	-
Perineal pain	Mild pain	Moderate pain; limiting instrumental ADL	Severe pain; limiting self care ADL	-	-
Premature menopause	-	-	Present	-	-
Prostatic hemorrhage	Minimal bleeding identified on imaging study; intervention not indicated	Moderate bleeding; medical intervention indicated	Severe bleeding; transfusion indicated; radiologic or endoscopic intervention indicated	Life-threatening consequences; urgent operative intervention indicated	Death

Prostatic obstruction	Diagnostic observations only; intervention not indicated	Mild symptoms; elective intervention indicated	Severe symptoms; elective operative intervention indicated	-	-
Prostatic pain	Mild pain	Moderate pain; limiting instrumental ADL	Severe pain; limiting self care ADL	-	-
Scrotal pain	Mild pain	Moderate pain; limiting instrumental ADL	Severe pain; limiting self care ADL	-	-
Spermatic cord hemorrhage	Minimal bleeding identified on imaging study; intervention not indicated	Moderate bleeding; medical intervention indicated	Severe bleeding; transfusion indicated; radiologic or endoscopic intervention indicated	Life-threatening consequences; urgent operative intervention indicated	Death
Spermatic cord obstruction	Diagnostic observations only; intervention not indicated	Mild symptoms; elective intervention indicated	Severe symptoms; elective operative intervention indicated	-	-
Testicular disorder	Asymptomatic; clinical or diagnostic observations only; intervention not indicated	Symptomatic but not interfering with urination or sexual activities; intervention not indicated; limiting instrumental ADL	Severe symptoms; interfering with urination or sexual function; limiting self care ADL; intervention indicated	Life-threatening consequences; urgent intervention indicated	Death
Testicular hemorrhage	Minimal bleeding identified on imaging study; intervention not indicated	Moderate bleeding; medical intervention indicated	Severe bleeding; transfusion indicated; radiologic or endoscopic intervention indicated	Life-threatening consequences; urgent operative intervention indicated	Death
Testicular pain	Mild pain	Moderate pain; limiting instrumental ADL	Severe pain; limiting self care ADL	-	-
Uterine fistula	Asymptomatic clinical or diagnostic observations only; intervention not indicated	Symptomatic and intervention not indicated	Severe symptoms; elective operative intervention indicated	Life-threatening consequences; urgent intervention indicated	Death
Uterine hemorrhage	Minimal bleeding identified on imaging study; intervention not indicated	Moderate bleeding; medical intervention indicated	Severe bleeding; transfusion indicated; radiologic or endoscopic intervention indicated	Life-threatening consequences; urgent operative intervention indicated	Death
Uterine obstruction	Diagnostic observations only; intervention not indicated	Mild symptoms; elective intervention indicated	Severe symptoms; elective operative intervention indicated	-	-
Uterine pain	Mild pain	Moderate pain; limiting instrumental ADL	Severe pain; limiting self care ADL	-	-
Vaginal discharge	Mild vaginal discharge (greater than baseline for patient)	Moderate to heavy vaginal discharge; use of perineal pad or tampon indicated	-	-	-

Vaginal dryness	Mild vaginal dryness not interfering with sexual function	Moderate vaginal dryness interfering with sexual function or causing frequent discomfort	Severe vaginal dryness resulting in dyspareunia or severe discomfort	-	-
Vaginal fistula	Asymptomatic clinical or diagnostic observations only; intervention not indicated	Symptomatic and intervention not indicated	Severe symptoms; elective operative intervention indicated	Life-threatening consequences; urgent intervention indicated	Death
Vaginal hemorrhage	Minimal bleeding identified on clinical exam or imaging study; intervention not indicated	Moderate bleeding; medical intervention indicated	Severe bleeding; transfusion indicated; radiologic or endoscopic intervention indicated	Life-threatening consequences; urgent operative intervention indicated	Death
Vaginal inflammation	Mild discomfort or pain, edema, or redness	Moderate discomfort or pain, edema, or redness; limiting instrumental ADL	Severe discomfort or pain, edema, or redness; limiting self care ADL; small areas of mucosal ulceration	Widespread areas of mucosal ulceration; life-threatening consequences; urgent intervention indicated	Death
Vaginal obstruction	Diagnostic observations only; intervention not indicated	Mild symptoms; elective intervention indicated	Severe symptoms; elective operative intervention indicated	-	-
Vaginal pain	Mild pain	Moderate pain; limiting instrumental ADL	Severe pain; limiting self care ADL	-	-
Vaginal perforation	Asymptomatic clinical or diagnostic observations only; intervention not indicated	Symptomatic and intervention not indicated	Severe symptoms; elective operative intervention indicated	Life-threatening consequences; urgent intervention indicated	Death
Vaginal stricture	Asymptomatic; mild vaginal shortening or narrowing	Vaginal narrowing and/or shortening not interfering with physical examination	Vaginal narrowing and/or shortening interfering with the use of tampons, sexual activity or physical examination	-	Death
Vaginismus	Mild discomfort or pain associated with vaginal spasm/tightening; no impact upon sexual function or physical examination	Moderate discomfort or pain associated with vaginal spasm/tightening; disruption in sexual function and physical examination	Severe discomfort or pain associated with vaginal spasm/tightening; unable to tolerate vaginal penetration or physical examination	-	-
Reproductive system and breast disorders - Other, specify	Asymptomatic or mild symptoms; clinical or diagnostic observations only; intervention not indicated	Moderate; minimal, local or noninvasive intervention indicated; limiting age-appropriate instrumental ADL	Severe or medically significant but not immediately life-threatening; hospitalization or prolongation of existing hospitalization indicated; disabling; limiting self care ADL	Life-threatening consequences; urgent intervention indicated	Death
Respiratory, thoracic and mediastinal disorders			Grade		
Adverse Event	1	2	3	4	5

Adult respiratory distress syndrome	-	-	Present with radiologic findings; intubation not indicated	Life-threatening respiratory or hemodynamic compromise; intubation or urgent intervention indicated	Death
Allergic rhinitis	Mild symptoms; intervention not indicated	Moderate symptoms; medical intervention indicated	-	-	-
Apnea	-	-	Present; medical intervention indicated	Life-threatening respiratory or hemodynamic compromise; intubation or urgent intervention indicated	Death
Aspiration	Asymptomatic; clinical or diagnostic observations only; intervention not indicated	Altered eating habits; coughing or choking episodes after eating or swallowing; medical intervention indicated (e.g., suction or oxygen)	Dyspnea and pneumonia symptoms (e.g., aspiration pneumonia); hospitalization indicated; unable to aliment orally	Life-threatening respiratory or hemodynamic compromise; intubation or urgent intervention indicated	Death
Atelectasis	Asymptomatic; clinical or diagnostic observations only; intervention not indicated	Symptomatic (e.g., dyspnea, cough); medical intervention indicated (e.g., chest physiotherapy, suctioning); bronchoscopic suctioning	Oxygen indicated; hospitalization or elective operative intervention indicated (e.g., stent, laser)	Life-threatening respiratory or hemodynamic compromise; intubation or urgent intervention indicated	Death
Bronchial fistula	Asymptomatic; clinical or diagnostic observations only; intervention not indicated	Symptomatic; tube thoracostomy or medical management indicated; limiting instrumental ADL	Severe symptoms; limiting self care ADL; endoscopic or operative intervention indicated (e.g., stent or primary closure)	Life-threatening consequences; urgent operative intervention with thoracoplasty, chronic open drainage or multiple thoracotomies indicated	Death
Bronchial obstruction	Asymptomatic; clinical or diagnostic observations only; intervention not indicated	Symptomatic (e.g., mild wheezing); endoscopic evaluation indicated; radiographic evidence of atelectasis/lobar collapse; medical management indicated (e.g., steroids, bronchodilators)	Shortness of breath with stridor; endoscopic intervention indicated (e.g., laser, stent placement)	Life-threatening respiratory or hemodynamic compromise; intubation or urgent intervention indicated	Death
Bronchial stricture	Asymptomatic; clinical or diagnostic observations only; intervention not indicated	Symptomatic (e.g., rhonchi or wheezing) but without respiratory distress; medical intervention indicated (e.g., steroids, bronchodilators)	Shortness of breath with stridor; endoscopic intervention indicated (e.g., laser, stent placement)	Life-threatening respiratory or hemodynamic compromise; intubation or urgent intervention indicated	Death

Bronchopleural fistula	Asymptomatic; clinical or diagnostic observations only; intervention not indicated	Symptomatic; tube thoracostomy or medical intervention indicated; limiting instrumental ADL	Severe symptoms; limiting self care ADL; endoscopic or operative intervention indicated (e.g., stent or primary closure)	Life-threatening consequences; urgent operative intervention with thoracoplasty, chronic open drainage or multiple thoracotomies indicated	Death
Bronchopulmonary hemorrhage	Mild symptoms; intervention not indicated	Moderate symptoms; medical intervention indicated	Transfusion, radiologic, endoscopic, or operative intervention indicated (e.g., hemostasis of bleeding site)	Life-threatening respiratory or hemodynamic compromise; intubation or urgent intervention indicated	Death
Bronchospasm	Mild symptoms; intervention not indicated	Symptomatic; medical intervention indicated; limiting instrumental ADL	Limiting self care ADL; oxygen saturation decreased	Life-threatening respiratory or hemodynamic compromise; intubation or urgent intervention indicated	Death
Chylothorax	Asymptomatic; clinical or diagnostic observations only; intervention not indicated	Symptomatic; thoracentesis or tube drainage indicated	Severe symptoms; elective operative intervention indicated	Life-threatening respiratory or hemodynamic compromise; intubation or urgent intervention indicated	Death
Cough	Mild symptoms; nonprescription intervention indicated	Moderate symptoms, medical intervention indicated; limiting instrumental ADL	Severe symptoms; limiting self care ADL	-	-
Dyspnea	Shortness of breath with moderate exertion	Shortness of breath with minimal exertion; limiting instrumental ADL	Shortness of breath at rest; limiting self care ADL	Life-threatening consequences; urgent intervention indicated	Death
Epistaxis	Mild symptoms; intervention not indicated	Moderate symptoms; medical intervention indicated (e.g., nasal packing, cauterization; topical vasoconstrictors)	Transfusion, radiologic, endoscopic, or operative intervention indicated (e.g., hemostasis of bleeding site)	Life-threatening consequences; urgent intervention indicated	Death
Hiccups	Mild symptoms; intervention not indicated	Moderate symptoms; medical intervention indicated; limiting instrumental ADL	Severe symptoms; interfering with sleep; limiting self care ADL	-	-
Hoarseness	Mild or intermittent voice change; fully understandable; self-resolves	Moderate or persistent voice changes; may require occasional repetition but understandable on telephone; medical evaluation indicated	Severe voice changes including predominantly whispered speech	-	-

Hypoxia	-	Decreased oxygen saturation with exercise (e.g., pulse oximeter <88%); intermittent supplemental oxygen	Decreased oxygen saturation at rest (e.g., pulse oximeter <88% or PaO2 <=55 mm Hg)	Life-threatening airway compromise; urgent intervention indicated (e.g., tracheotomy or intubation)	Death
Laryngeal edema	Asymptomatic; clinical or diagnostic observations only; intervention not indicated	Symptomatic; medical intervention indicated (e.g., dexamethasone, epinephrine, antihistamines)	Stridor; respiratory distress; hospitalization indicated	Life-threatening airway compromise; urgent intervention indicated (e.g., tracheotomy or intubation)	Death
Laryngeal fistula	Asymptomatic; clinical or diagnostic observations only; intervention not indicated	Symptomatic; tube thoracostomy or medical management indicated; limiting instrumental ADL	Severe symptoms; limiting self care ADL; endoscopic or operative intervention indicated (e.g., stent or primary closure)	Life-threatening consequences; urgent operative intervention indicated (e.g., thoracoplasty, chronic open drainage or multiple thoracotomies)	Death
Laryngeal hemorrhage	Mild cough or trace hemoptysis; laryngoscopic findings	Moderate symptoms; medical intervention indicated	Transfusion, radiologic, endoscopic, or operative intervention indicated (e.g., hemostasis of bleeding site)	Life-threatening airway compromise; urgent intervention indicated (e.g., tracheotomy or intubation)	Death
Laryngeal inflammation	Mild sore throat; raspy voice	Moderate sore throat; analgesics indicated	Severe throat pain; endoscopic intervention indicated	-	-
Laryngeal mucositis	Endoscopic findings only; mild discomfort with normal intake	Moderate discomfort; altered oral intake	Severe pain; severely altered eating/swallowing; medical intervention indicated	Life-threatening airway compromise; urgent intervention indicated (e.g., tracheotomy or intubation)	Death
Laryngeal obstruction	Asymptomatic; clinical or diagnostic observations only; intervention not indicated	Symptomatic (e.g., noisy airway breathing), but causing no respiratory distress; medical management indicated (e.g., steroids); limiting instrumental ADL	Limiting self care ADL; stridor; endoscopic intervention indicated (e.g., stent, laser)	Life-threatening consequences; urgent intervention indicated	Death
Laryngeal stenosis	Asymptomatic; clinical or diagnostic observations only; intervention not indicated	Symptomatic (e.g., noisy airway breathing), but causing no respiratory distress; medical management indicated (e.g., steroids)	Limiting self care ADL; stridor; endoscopic intervention indicated (e.g., stent, laser)	Life-threatening consequences; urgent intervention indicated	Death
Laryngopharyngeal dysesthesia	Mild symptoms; no anxiety; intervention not indicated	Moderate symptoms; mild anxiety, but no dyspnea; short duration of observation and or anxiolytic indicated; limiting instrumental ADL	Severe symptoms; dyspnea and swallowing difficulty; limiting self care ADL	Life-threatening consequences	Death

Laryngospasm	-	Transient episode; intervention not indicated	Recurrent episodes; noninvasive intervention indicated (e.g., breathing technique, pressure point massage)	Persistent or severe episodes associated with syncope; urgent intervention indicated (e.g., fiberoptic laryngoscopy, intubation, botox injection)	Death
Mediastinal hemorrhage	Radiologic evidence only; minimal symptoms; intervention not indicated	Moderate symptoms; medical intervention indicated	Transfusion, radiologic, endoscopic, or elective operative intervention indicated (e.g., hemostasis of bleeding site)	Life-threatening consequences; urgent intervention indicated	Death
Nasal congestion	Mild symptoms; intervention not indicated	Moderate symptoms; medical intervention indicated	Associated with bloody nasal discharge or epistaxis	-	-
Pharyngeal fistula	Asymptomatic; clinical or diagnostic observations only; intervention not indicated	Symptomatic; tube thoracostomy or medical intervention indicated; limiting instrumental ADL	Severe symptoms; limiting self care ADL; endoscopic or operative intervention indicated (e.g., stent or primary closure)	Life-threatening consequences; urgent intervention indicated	Death
Pharyngeal hemorrhage	Mild symptoms; intervention not indicated	Moderate symptoms; medical intervention indicated	Transfusion, radiologic, endoscopic, or operative intervention indicated (e.g., hemostasis of bleeding site)	Life-threatening respiratory or hemodynamic compromise; intubation or urgent intervention indicated	Death
Pharyngeal mucositis	Endoscopic findings only; minimal symptoms with normal oral intake; mild pain but analgesics not indicated	Moderate pain and analgesics indicated; altered oral intake; limiting instrumental ADL	Severe pain; unable to adequately aliment or hydrate orally; limiting self care ADL	Life-threatening consequences; urgent intervention indicated	Death
Pharyngeal necrosis	-	-	Inability to aliment adequately by GI tract; tube feeding or TPN indicated; radiologic, endoscopic, or operative intervention indicated	Life-threatening consequences; urgent operative intervention indicated	Death
Pharyngeal stenosis	Asymptomatic; clinical or diagnostic observations only; intervention not indicated	Symptomatic (e.g., noisy airway breathing), but causing no respiratory distress; medical management indicated (e.g., steroids); limiting instrumental ADL	Limiting self care ADL; stridor; endoscopic intervention indicated (e.g., stent, laser)	Life-threatening airway compromise; urgent intervention indicated (e.g., tracheotomy or intubation)	Death
Pharyngolaryngeal pain	Mild pain	Moderate pain; limiting instrumental ADL	Severe pain; limiting self care ADL	-	-

Pleural effusion	Asymptomatic; clinical or diagnostic observations only; intervention not indicated	Symptomatic; intervention indicated (e.g., diuretics or limited therapeutic thoracentesis)	Symptomatic with respiratory distress and hypoxia; surgical intervention including chest tube or pleurodesis indicated	Life-threatening respiratory or hemodynamic compromise; intubation or urgent intervention indicated	Death
Pleural hemorrhage	Asymptomatic; mild hemorrhage confirmed by thoracentesis	Symptomatic or associated with pneumothorax; chest tube drainage indicated	>1000 ml of blood evacuated; persistent bleeding (150-200 ml/hr for 2 - 4 hr); persistent transfusion indicated; elective operative intervention indicated	Life-threatening respiratory or hemodynamic compromise; intubation or urgent intervention indicated	Death
Pleuritic pain	Mild pain	Moderate pain; limiting instrumental ADL	Severe pain; limiting self care ADL	-	-
Pneumonitis	Asymptomatic; clinical or diagnostic observations only; intervention not indicated	Symptomatic; medical intervention indicated; limiting instrumental ADL	Severe symptoms; limiting self care ADL; oxygen indicated	Life-threatening respiratory compromise; urgent intervention indicated (e.g., tracheotomy or intubation)	Death
Pneumothorax	Asymptomatic; clinical or diagnostic observations only; intervention not indicated	Symptomatic; intervention indicated (e.g., tube placement without sclerosis)	Sclerosis and/or operative intervention indicated; hospitalization indicated	Life-threatening consequences; urgent intervention indicated	Death
Postnasal drip	Mild symptoms; intervention not indicated	Moderate symptoms; medical intervention indicated	-	-	-
Productive cough	Occasional/minimal production of sputum with cough	Moderate sputum production; limiting instrumental ADL	Persistent or copious production of sputum; limiting self care ADL	-	-
Pulmonary edema	Radiologic findings only; minimal dyspnea on exertion	Moderate dyspnea on exertion; medical intervention indicated; limiting instrumental ADL	Severe dyspnea or dyspnea at rest; oxygen indicated; limiting self care ADL	Life-threatening respiratory compromise; urgent intervention or intubation with ventilatory support indicated	Death
Pulmonary fibrosis	Mild hypoxemia; radiologic pulmonary fibrosis <25% of lung volume	Moderate hypoxemia; evidence of pulmonary hypertension; radiographic pulmonary fibrosis 25 - 50%	Severe hypoxemia; evidence of right-sided heart failure; radiographic pulmonary fibrosis >50 - 75%	Life-threatening consequences (e.g., hemodynamic/pulmonary complications); intubation with ventilatory support indicated; radiographic pulmonary fibrosis >75% with severe honeycombing	Death

Pulmonary fistula	Asymptomatic; clinical or diagnostic observations only; intervention not indicated	Symptomatic; tube thoracostomy or medical management indicated; limiting instrumental ADL	Limiting self care ADL; endoscopic stenting or operative intervention indicated	Life-threatening consequences; urgent operative intervention indicated	Death
Pulmonary hypertension	Minimal dyspnea; findings on physical exam or other evaluation	Moderate dyspnea, cough; requiring evaluation by cardiac catheterization and medical intervention	Severe symptoms, associated with hypoxemia, right heart failure; oxygen indicated	Life-threatening airway consequences; urgent intervention indicated (e.g., tracheotomy or intubation)	Death
Respiratory failure	-	-	-	Life-threatening consequences; urgent intervention, intubation, or ventilatory support indicated	Death
Retinoic acid syndrome	Fluid retention; <3 kg of weight gain; intervention with fluid restriction and/or diuretics indicated	Moderate signs or symptoms; steroids indicated	Severe symptoms; hospitalization indicated	Life-threatening consequences; ventilatory support indicated	Death
Sinus disorder	Asymptomatic mucosal crusting; blood-tinged secretions	Symptomatic stenosis or edema/narrowing interfering with airflow; limiting instrumental ADL	Stenosis with significant nasal obstruction; limiting self care ADL	Necrosis of soft tissue or bone; urgent operative intervention indicated	Death
Sleep apnea	Snoring and nocturnal sleep arousal without apneic periods	Moderate apnea and oxygen desaturation; excessive daytime sleepiness; medical evaluation indicated; limiting instrumental ADL	Oxygen desaturation; associated with hypertension; medical intervention indicated; limiting self care ADL	Cardiovascular or neuropsychiatric symptoms; urgent operative intervention indicated	Death
Sneezing	Mild symptoms; intervention not indicated	Moderate symptoms; medical intervention indicated	-	-	-
Sore throat	Mild pain	Moderate pain; limiting instrumental ADL	Severe pain; limiting self care ADL; limiting ability to swallow	-	-
Stridor	-	-	Respiratory distress limiting self care ADL; medical intervention indicated	Life-threatening airway compromise; urgent intervention indicated (e.g., tracheotomy or intubation)	Death
Tracheal fistula	Asymptomatic; clinical or diagnostic observations only; intervention not indicated	Symptomatic; tube thoracostomy or medical intervention indicated; limiting instrumental ADL	Severe symptoms; limiting self care ADL; endoscopic or operative intervention indicated (e.g., stent or primary closure)	Life-threatening consequences; urgent operative intervention indicated (e.g., thoracoplasty, chronic open drainage or multiple thoracotomies)	Death

Tracheal mucositis	Endoscopic findings only; minimal hemoptysis, pain, or respiratory symptoms	Moderate symptoms; medical intervention indicated; limiting instrumental ADL	Severe pain; hemorrhage or respiratory symptoms; limiting self care ADL	Life-threatening consequences; urgent intervention indicated	Death
Tracheal stenosis	Asymptomatic; clinical or diagnostic observations only; intervention not indicated	Symptomatic (e.g., noisy airway breathing), but causing no respiratory distress; medical management indicated (e.g., steroids)	Stridor or respiratory distress limiting self care ADL; endoscopic intervention indicated (e.g., stent, laser)	Life-threatening airway compromise; urgent intervention indicated (e.g., tracheotomy or intubation)	Death
Voice alteration	Mild or intermittent change from normal voice	Moderate or persistent change from normal voice; still understandable	Severe voice changes including predominantly whispered speech; may require frequent repetition or face-to-face contact for understandability; may require assistive technology	-	-
Wheezing	Detectable airway noise with minimal symptoms	Moderate symptoms; medical intervention indicated; limiting instrumental ADL	Severe respiratory symptoms limiting self care ADL; oxygen therapy or hospitalization indicated	Life-threatening consequences; urgent intervention indicated	Death
Respiratory, thoracic and mediastinal disorders - Other, specify	Asymptomatic or mild symptoms; clinical or diagnostic observations only; intervention not indicated	Moderate; minimal, local or noninvasive intervention indicated; limiting age-appropriate instrumental ADL	Severe or medically significant but not immediately life-threatening; hospitalization or prolongation of existing hospitalization indicated; disabling; limiting self care ADL	Life-threatening consequences; urgent intervention indicated	Death

Skin and subcutaneous tissue disorders	Grade				
Adverse Event	1	2	3	4	5
Alopecia	Hair loss of <50% of normal for that individual that is not obvious from a distance but only on close inspection; a different hair style may be required to cover the hair loss but it does not require a wig or hair piece to camouflage	Hair loss of >=50% normal for that individual that is readily apparent to others; a wig or hair piece is necessary if the patient desires to completely camouflage the hair loss; associated with psychosocial impact	-	-	-
Body odor	Mild odor; physician intervention not indicated; self care interventions	Pronounced odor; psychosocial impact; patient seeks medical intervention	-	-	-
Bullous dermatitis	Asymptomatic; blisters covering <10% BSA	Blisters covering 10 - 30% BSA; painful blisters; limiting instrumental ADL	Blisters covering >30% BSA; limiting self care ADL	Blisters covering >30% BSA; associated with fluid or electrolyte abnormalities; ICU care or burn unit indicated	Death
Dry skin	Covering <10% BSA and no associated erythema or pruritus	Covering 10 - 30% BSA and associated with erythema or pruritus; limiting instrumental ADL	Covering >30% BSA and associated with pruritus; limiting self care ADL	-	-
Erythema multiforme	Target lesions covering <10% BSA and not associated with skin tenderness	Target lesions covering 10 - 30% BSA and associated with skin tenderness	Target lesions covering >30% BSA and associated with oral or genital erosions	Target lesions covering >30% BSA; associated with fluid or electrolyte abnormalities; ICU care or burn unit indicated	Death
Erythroderma	-	Erythema covering >90% BSA without associated symptoms; limiting instrumental ADL	Erythema covering >90% BSA with associated symptoms (e.g., pruritus or tenderness); limiting self care ADL	Erythema covering >90% BSA with associated fluid or electrolyte abnormalities; ICU care or burn unit indicated	Death
Fat atrophy	Covering <10% BSA and asymptomatic	Covering 10 - 30% BSA and associated with erythema or tenderness; limiting instrumental ADL	Covering >30% BSA; associated with erythema or tenderness; limiting self-care ADL	-	-
Hirsutism	In women, increase in length, thickness or density of hair in a male distribution that the patient is able to camouflage by periodic shaving, bleaching, or removal of hair	In women, increase in length, thickness or density of hair in a male distribution that requires daily shaving or consistent destructive means of hair removal to camouflage; associated with psychosocial impact	-	-	-

Hyperhidrosis	Limited to one site (palms, soles, or axillae); self care interventions	Involving >1 site; patient seeks medical intervention; associated with psychosocial impact	Generalized involving sites other than palms, soles, or axillae; associated with electrolyte/hemodynamic imbalance	-	-
Hypertrichosis	Increase in length, thickness or density of hair that the patient is either able to camouflage by periodic shaving or removal of hairs or is not concerned enough about the overgrowth to use any form of hair removal	Increase in length, thickness or density of hair at least on the usual exposed areas of the body [face (not limited to beard/moustache area) plus/minus arms] that requires frequent shaving or use of destructive means of hair removal to camouflage; associated with psychosocial impact	-	-	-
Hypohidrosis	-	Symptomatic; limiting instrumental ADL	Increase in body temperature; limiting self care ADL	Heat stroke	Death
Lipohypertrophy	Asymptomatic and covering <10% BSA	Covering 10 - 30% BSA and associated tenderness; limiting instrumental ADL	Covering >30% BSA and associated tenderness and narcotics or NSAIDs indicated; lipohypertrophy; limiting self care ADL	-	-
Nail discoloration	Asymptomatic; clinical or diagnostic observations only; intervention not indicated	-	-	-	-
Nail loss	Asymptomatic separation of the nail bed from the nail plate or nail loss	Symptomatic separation of the nail bed from the nail plate or nail loss; limiting instrumental ADL	-	-	-
Nail ridging	Asymptomatic; clinical or diagnostic observations only; intervention not indicated	-	-	-	-
Pain of skin	Mild pain	Moderate pain; limiting instrumental ADL	Severe pain; limiting self care ADL	-	-
Palmar-plantar erythrodysesthesia syndrome	Minimal skin changes or dermatitis (e.g., erythema, edema, or hyperkeratosis) without pain	Skin changes (e.g., peeling, blisters, bleeding, edema, or hyperkeratosis) with pain; limiting instrumental ADL	Severe skin changes (e.g., peeling, blisters, bleeding, edema, or hyperkeratosis) with pain; limiting self care ADL	-	-

Periorbital edema	Soft or non-pitting	Indurated or pitting edema; topical intervention indicated	Edema associated with visual disturbance; increased intraocular pressure, glaucoma or retinal hemorrhage; optic neuritis; diuretics indicated; operative intervention indicated	-	-
Photosensitivity	Painless erythema and erythema covering <10% BSA	Tender erythema covering 10 - 30% BSA	Erythema covering >30% BSA and erythema with blistering; photosensitivity; oral corticosteroid therapy indicated; pain control indicated (e.g., narcotics or NSAIDs)	Life-threatening consequences; urgent intervention indicated	Death
Pruritus	Mild or localized; topical intervention indicated	Intense or widespread; intermittent; skin changes from scratching (e.g., edema, papulation, excoriations, lichenification, oozing/crusts); oral intervention indicated; limiting instrumental ADL	Intense or widespread; constant; limiting self care ADL or sleep; oral corticosteroid or immunosuppressive therapy indicated	-	-
Purpura	Combined area of lesions covering <10% BSA	Combined area of lesions covering 10 - 30% BSA; bleeding with trauma	Combined area of lesions covering >30% BSA; spontaneous bleeding	-	-
Rash acneiform	Papules and/or pustules covering <10% BSA, which may or may not be associated with symptoms of pruritus or tenderness	Papules and/or pustules covering 10 - 30% BSA, which may or may not be associated with symptoms of pruritus or tenderness; associated with psychosocial impact; limiting instrumental ADL	Papules and/or pustules covering >30% BSA, which may or may not be associated with symptoms of pruritus or tenderness; limiting self care ADL; associated with local superinfection with oral antibiotics indicated	Papules and/or pustules covering any % BSA, which may or may not be associated with symptoms of pruritus or tenderness and are associated with extensive superinfection with IV antibiotics indicated; life-threatening consequences	Death
Rash maculo-papular	Macules/papules covering <10% BSA with or without symptoms (e.g., pruritus, burning, tightness)	Macules/papules covering 10 - 30% BSA with or without symptoms (e.g., pruritus, burning, tightness); limiting instrumental ADL	Macules/papules covering >30% BSA with or without associated symptoms; limiting self care ADL	-	-
Scalp pain	Mild pain	Moderate pain; limiting instrumental ADL	Severe pain; limiting self care ADL	-	-
Skin atrophy	Covering <10% BSA; associated with telangiectasias or changes in skin color	Covering 10 - 30% BSA; associated with striae or adnexal structure loss	Covering >30% BSA; associated with ulceration	-	-
Skin hyperpigmentation	Hyperpigmentation covering <10% BSA; no psychosocial impact	Hyperpigmentation covering >10% BSA; associated psychosocial impact	-	-	-

Skin hypopigmentation	Hypopigmentation or depigmentation covering <10% BSA; no psychosocial impact	Hypopigmentation or depigmentation covering >10% BSA; associated psychosocial impact	-	-	-
Skin induration	Mild induration, able to move skin parallel to plane (sliding) and perpendicular to skin (pinching up)	Moderate induration, able to slide skin, unable to pinch skin; limiting instrumental ADL	Severe induration, unable to slide or pinch skin; limiting joint movement or orifice (e.g., mouth, anus); limiting self care ADL	Generalized; associated with signs or symptoms of impaired breathing or feeding	Death
Skin ulceration	Combined area of ulcers <1 cm; nonblanchable erythema of intact skin with associated warmth or edema	Combined area of ulcers 1 - 2 cm; partial thickness skin loss involving skin or subcutaneous fat	Combined area of ulcers >2 cm; full-thickness skin loss involving damage to or necrosis of subcutaneous tissue that may extend down to fascia	Any size ulcer with extensive destruction, tissue necrosis, or damage to muscle, bone, or supporting structures with or without full thickness skin loss	Death
Stevens-Johnson syndrome	-	-	Skin sloughing covering <10% BSA with associated signs (e.g., erythema, purpura, epidermal detachment and mucous membrane detachment)	Skin sloughing covering 10 - 30% BSA with associated signs (e.g., erythema, purpura, epidermal detachment and mucous membrane detachment)	Death
Telangiectasia	Telangiectasias covering <10% BSA	Telangiectasias covering >10% BSA; associated with psychosocial impact	-	-	-
Toxic epidermal necrolysis	-	-	-	Skin sloughing covering >=30% BSA with associated symptoms (e.g., erythema, purpura, or epidermal detachment)	Death
Urticaria	Urticarial lesions covering <10% BSA; topical intervention indicated	Urticarial lesions covering 10 - 30% BSA; oral intervention indicated	Urticarial lesions covering >30% BSA; IV intervention indicated	-	-
Skin and subcutaneous tissue disorders - Other, specify	Asymptomatic or mild symptoms; clinical or diagnostic observations only; intervention not indicated	Moderate; minimal, local or noninvasive intervention indicated; limiting age-appropriate instrumental ADL	Severe or medically significant but not immediately life-threatening; hospitalization or prolongation of existing hospitalization indicated; disabling; limiting self care ADL	Life-threatening consequences; urgent intervention indicated	Death

Social circumstances	Grade				
Adverse Event	1	2	3	4	5
Menopause	Menopause occurring at age 46 - 53 years of age	Menopause occurring at age 40 - 45 years of age	Menopause occurring before age 40 years of age	-	-
Social circumstances - Other, specify	Asymptomatic or mild symptoms; clinical or diagnostic observations only; intervention not indicated	Moderate; minimal, local or noninvasive intervention indicated; limiting age-appropriate instrumental ADL	Severe or medically significant but not immediately life-threatening; hospitalization or prolongation of existing hospitalization indicated; disabling; limiting self care ADL	Life-threatening consequences; urgent intervention indicated	Death
Surgical and medical procedures	Grade				
Adverse Event	1	2	3	4	5
Surgical and medical procedures - Other, specify	Asymptomatic or mild symptoms; clinical or diagnostic observations only; intervention not indicated	Moderate; minimal, local or noninvasive intervention indicated; limiting age-appropriate instrumental ADL	Severe or medically significant but not immediately life-threatening; hospitalization or prolongation of existing hospitalization indicated; disabling; limiting self care ADL	Life-threatening consequences; urgent intervention indicated	Death
Vascular disorders	Grade				
Adverse Event	1	2	3	4	5
Capillary leak syndrome	-	Symptomatic; medical intervention indicated	Severe symptoms; intervention indicated	Life-threatening consequences; urgent intervention indicated	Death
Flushing	Asymptomatic; clinical or diagnostic observations only; intervention not indicated	Moderate symptoms; medical intervention indicated; limiting instrumental ADL	Symptomatic, associated with hypotension and/or tachycardia; limiting self care ADL	-	-
Hematoma	Mild symptoms; intervention not indicated	Minimally invasive evacuation or aspiration indicated	Transfusion, radiologic, endoscopic, or elective operative intervention indicated	Life-threatening consequences; urgent intervention indicated	Death
Hot flashes	Mild symptoms; intervention not indicated	Moderate symptoms; limiting instrumental ADL	Severe symptoms; limiting self care ADL	-	-
Hypertension	Prehypertension (systolic BP 120 - 139 mm Hg or diastolic BP 80 - 89 mm Hg)	Stage 1 hypertension (systolic BP 140 - 159 mm Hg or diastolic BP 90 - 99 mm Hg); medical intervention indicated; recurrent or persistent (>=24 hrs); symptomatic increase by >20 mm Hg (diastolic) or to >140/90 mm Hg if previously WNL; monotherapy indicated<br><br>Pediatric: recurrent or persistent (>=24 hrs) BP >ULN; monotherapy indicated	Stage 2 hypertension (systolic BP >=160 mm Hg or diastolic BP >=100 mm Hg); medical intervention indicated; more than one drug or more intensive therapy than previously used indicated<br><br>Pediatric: Same as adult	Life-threatening consequences (e.g., malignant hypertension, transient or permanent neurologic deficit, hypertensive crisis); urgent intervention indicated<br><br>Pediatric: Same as adult	Death

Hypotension	Asymptomatic, intervention not indicated	Non-urgent medical intervention indicated	Medical intervention or hospitalization indicated	Life-threatening and urgent intervention indicated	Death
Lymph leakage	-	Symptomatic; medical intervention indicated	Severe symptoms; radiologic, endoscopic or elective operative intervention indicated	Life-threatening consequences; urgent intervention indicated	Death
Lymphedema	Trace thickening or faint discoloration	Marked discoloration; leathery skin texture; papillary formation; limiting instrumental ADL	Severe symptoms; limiting self care ADL	-	-
Lymphocele	Asymptomatic; clinical or diagnostic observations only; intervention not indicated	Symptomatic; medical intervention indicated	Severe symptoms; radiologic, endoscopic or elective operative intervention indicated	-	-
Peripheral ischemia	-	Brief (<24 hrs) episode of ischemia managed non-surgically and without permanent deficit	Recurring or prolonged (>=24 hrs) and/or invasive intervention indicated	Life-threatening consequences; evidence of end organ damage; urgent operative intervention indicated	Death
Phlebitis	-	Present	-	-	-
Superficial thrombophlebitis	-	Present	-	-	-
Superior vena cava syndrome	Asymptomatic; incidental finding of SVC thrombosis	Symptomatic; medical intervention indicated (e.g., anticoagulation, radiation or chemotherapy)	Severe symptoms; multi-modality intervention indicated (e.g., anticoagulation, chemotherapy, radiation, stenting)	Life-threatening consequences; urgent multi-modality intervention indicated (e.g., lysis, thrombectomy, surgery)	Death
Thromboembolic event	Venous thrombosis (e.g., superficial thrombosis)	Venous thrombosis (e.g., uncomplicated deep vein thrombosis), medical intervention indicated	Thrombosis (e.g., uncomplicated pulmonary embolism [venous], non-embolic cardiac mural [arterial] thrombus), medical intervention indicated	Life-threatening (e.g., pulmonary embolism, cerebrovascular event, arterial insufficiency); hemodynamic or neurologic instability; urgent intervention indicated	Death
Vasculitis	Asymptomatic, intervention not indicated	Moderate symptoms, medical intervention indicated	Severe symptoms, medical intervention indicated (e.g., steroids)	Life-threatening; evidence of peripheral or visceral ischemia; urgent intervention indicated	Death
Visceral arterial ischemia	-	Brief (<24 hrs) episode of ischemia managed medically and without permanent deficit	Prolonged (>=24 hrs) or recurring symptoms and/or invasive intervention indicated	Life-threatening consequences; evidence of end organ damage; urgent operative intervention indicated	Death
Vascular disorders - Other, specify	Asymptomatic or mild symptoms; clinical or diagnostic observations only; intervention not indicated	Moderate; minimal, local or noninvasive intervention indicated; limiting age-appropriate instrumental ADL	Severe or medically significant but not immediately life-threatening; hospitalization or prolongation of existing hospitalization indicated; disabling; limiting self care ADL	Life-threatening consequences; urgent intervention indicated	Death

APPENDIX G REFERENCES

1 (UNAIDS)., J. U. N. P. o. H. A. The Gap Report., (UNAIDS, 2014).

2 UNAIDS. AIDS by the numbers 2015. (2015).

3 CDC. CDC. Vital Signs: HIV Diagnosis, Care, and Treatment Among Persons Living with HIV- United States 2011. *MMWR* **4**, 1-6 (2014).

4 Jardine, J. *et al.* Rational HIV immunogen design to target specific germline B cell receptors. *Science* **340**, 711-716, doi:10.1126/science.1234150 (2013).

5 Sok, D. *et al.* Promiscuous glycan site recognition by antibodies to the high-mannose patch of gp120 broadens neutralization of HIV. *Sci Transl Med* **6**, 236ra263, doi:10.1126/scitranslmed.3008104 (2014).

6 Caskey, M. *et al.* Viraemia suppressed in HIV-1-infected humans by broadly neutralizing antibody 3BNC117. *Nature* **522**, 487-491, doi:10.1038/nature14411 (2015).

7 Barouch, D. H. *et al.* Therapeutic efficacy of potent neutralizing HIV-1-specific monoclonal antibodies in SHIV-infected rhesus monkeys. *Nature* **503**, 224-228, doi:10.1038/nature12744 (2013).

8 Hessell, A. J. *et al.* Broadly neutralizing human anti-HIV antibody 2G12 is effective in protection against mucosal SHIV challenge even at low serum neutralizing titers. *PLoS Pathog* **5**, e1000433, doi:10.1371/journal.ppat.1000433 (2009).

9 Hessell, A. J. *et al.* Effective, low-titer antibody protection against low-dose repeated mucosal SHIV challenge in macaques. *Nat Med* **15**, 951-954, doi:10.1038/nm.1974 (2009).

10 Moldt, B. *et al.* Highly potent HIV-specific antibody neutralization in vitro translates into effective protection against mucosal SHIV challenge in vivo. *Proceedings of the National Academy of Sciences of the United States of America* **109**, 18921-18925, doi:10.1073/pnas.1214785109 (2012).

11 Walker, L. M. *et al.* Broad neutralization coverage of HIV by multiple highly potent antibodies. *Nature* **477**, 466-470, doi:10.1038/nature10373 (2011).

12 Haynes, B. F. & McElrath, M. J. Progress in HIV-1 vaccine development. *Curr Opin HIV AIDS* **8**, 326-332, doi:10.1097/COH.0b013e328361d178 (2013).

13 Burton, D. R. & Mascola, J. R. Antibody responses to envelope glycoproteins in HIV-1 infection. *Nat Immunol* **16**, 571-576, doi:10.1038/ni.3158 (2015).

14 Sok, D. *et al.* Recombinant HIV envelope trimer selects for quaternary-dependent antibodies targeting the trimer apex. *Proc Natl Acad Sci U S A* **111**, 17624-17629, doi:10.1073/pnas.1415789111 (2014).

15 Scheid, J. F. *et al.* Sequence and structural convergence of broad and potent HIV antibodies that mimic CD4 binding. *Science* **333**, 1633-1637, doi:10.1126/science.1207227 (2011).

16 Shingai, M. *et al.* Passive transfer of modest titers of potent and broadly neutralizing anti-HIV monoclonal antibodies block SHIV infection in macaques. *J Exp Med* **211**, 2061-2074, doi:10.1084/jem.20132494 (2014).

17 Lynch, R. M. *et al.* Virologic effects of broadly neutralizing antibody VRC01 administration during chronic HIV-1 infection. *Sci Transl Med* **7**, 319ra206, doi:10.1126/scitranslmed.aad5752 (2015).

18 Andrade, A. *et al.* Three distinct phases of HIV-1 RNA decay in treatment-naive patients receiving raltegravir-based antiretroviral therapy: ACTG A5248. *J Infect Dis* **208**, 884-891, doi:10.1093/infdis/jit272 (2013).

International AIDS Vaccine Initiative

DOCUMENT NUMBER:
DOCUMENT TITLE:
DOCUMENT NOTES:

Document Information

Revision:	Vault:
Status:	Document Type:

Date Information

Effective Date:	Expiration Date:
Release Date:	Next Review Date:

Control Information

Author:	Owner:
Previous Number:	Change Number:

Signature Manifest

Document Number: TMF-02-0166 **Revision:** 4

Title: Protocol PGT121

All dates and times are in Eastern Time Zone.

T001 Protocol Amendment

Change Request Approval

Name/Signature	Title	Date	Meaning/Reason

CMO Approval

Name/Signature	Title	Date	Meaning/Reason
Frances Priddy (FPRIDDY)	Chief Medical Officer	05 Apr 2017, 04:31:12 PM	Approved

QA Final Release

Name/Signature	Title	Date	Meaning/Reason
Lisa Sunner (LSUNNER)			
Carl Verlinde (CVERLINDE)			
Dani Vooijs (DVOOIJS)			
Harriet Park (HPARK)	Director Clincal Operations	05 Apr 2017, 06:51:45 PM	Approved

Notify

Name/Signature	Title	Date	Meaning/Reason
Lisa Sunner (LSUNNER)		05 Apr 2017, 06:51:45 PM	Email Sent

**Protocol Title:**	A Phase 1 Randomized Placebo-controlled Clinical Trial of the Safety, Pharmacokinetics and Antiviral Activity of PGT121 Monoclonal Antibody (mAb) in HIV-uninfected and HIV-infected Adults
**Protocol Number:**	IAVI T001
**Regulatory Investigational Product Number**	IND 126807
**ClinicalTrials.gov Registry Number**	NCT02960581
**Phase:**	Phase 1
**Sponsor:**	International AIDS Vaccine Initiative (IAVI)<br>125 Broad Street, 9th Floor<br>New York, New York 10004<br>USA
**Sponsor Status**	Not for-Profit Organization
**Date of Protocol Version:**	14 July 2017<br>06.0<br><br>04 April 2017<br>05.0<br><br>23 November 2016<br>04.0<br><br>17 October 2016<br>03.0<br><br>09 September 2016<br>02.0<br><br>05 August 2016<br>01.0

THE CONFIDENTIAL INFORMATION IN THIS DOCUMENT IS PROVIDED TO YOU AS AN INVESTIGATOR, POTENTIAL INVESTIGATOR, OR CONSULTANT, FOR REVIEW BY YOU, YOUR STAFF, AND APPLICABLE INSTITUTIONAL REVIEW BOARDS (IRBS) AND/OR INDEPENDENT ETHICS COMMITTEES (IECS). IT IS UNDERSTOOD THAT THE INFORMATION WILL NOT BE DISCLOSED TO OTHERS, EXCEPT TO THE EXTENT NECESSARY TO OBTAIN ETHICAL AND REGULATORY APPROVAL FROM THE RESPECTIVE COMMITTEE'S AGENCIES AND INFORMED CONSENT FROM THOSE PERSONS TO WHOM THE INVESTIGATIONAL PRODUCT MAY BE ADMINISTERED.

SYNOPSIS

**TITLE**	A Phase 1 Randomized Placebo-controlled Clinical Trial of the Safety, Pharmacokinetics and Antiviral Activity of PGT121 Monoclonal Antibody (mAb) in HIV-uninfected and HIV-infected Adults
**PROTOCOL NUMBER**	IAVI T001
**PHASE**	Phase 1
**SPONSOR**	International AIDS Vaccine Initiative (IAVI)<br>125 Broad Street, 9th Floor<br>New York, New York 10004,<br>USA
**SPONSOR STATUS**	Not for Profit Organization
**STUDY PRODUCTS**	PGT121 monoclonal antibody (mAb)
**OBJECTIVES**	**Primary Objectives**<br>• To evaluate the safety and tolerability of a single intravenous (IV) infusion of PGT121 mAb at 3, 10, and 30 mg/kg in HIV-uninfected adults and HIV-infected adults<br>• To evaluate the safety and tolerability of a single subcutaneous (SC) injection of PGT121 mAb at 3 mg/kg in HIV-uninfected adults<br>• To evaluate the pharmacokinetic (PK) profile of IV infusion and SC injection of PGT121 mAb in HIV-uninfected and HIV-infected adults<br>• To evaluate the antiviral activity of IV infusion of PGT121 mAb in HIV-infected adults not on ART<br><br>**Secondary Objective**<br>• To determine if PGT121 induces anti-PGT121 antibodies<br>• To determine the effect of PGT121 mAb on CD4+ T cell counts in HIV-infected adults<br>• To determine if PGT121 mAb induces viral escape mutants in viremic HIV-infected adults not on ART<br><br>**Exploratory Objectives**<br>• To determine if PGT121 mAb has any impact on the host immune responses (i.e., HIV-specific cellular and humoral immune response)<br>• To determine the effect of PGT121 mAb on the size of the latent HIV reservoir in HIV-infected adults<br>• To determine PGT121 mAb levels in mucosal secretions in HIV-uninfected and HIV-infected adults<br>• To measure in vitro neutralization of HIV isolates with participant's serum post PGT121 mAb IV infusion<br>• To determine if PGT121 mAb has any impact on resistance mutations to ARVs

**ENDPOINTS**	***Primary***:<br><br>*Safety and Tolerability:*<br>We will measure the following endpoints to evaluate the safety and tolerability of PGT121 mAb in HIV-uninfected and HIV-infected adults:<br><br>1. Proportion of participants with moderate or greater reactogenicity (e.g., solicited adverse events) for 3 days following IV infusion or SC injection of PGT121 mAb.<br>2. Proportion of participants with moderate or greater and/or PGT121 mAb-related unsolicited adverse events (AEs), including safety laboratory (biochemical, hematological) parameters, following IV infusion or SC injection of PGT121 mAb for the first 56 days post administration of Investigational Product.<br>3. Proportion of participants with PGT121 mAb-related serious adverse events (SAEs) throughout the study period.<br><br>*Pharmacokinetics:*<br>We will measure the following endpoints to determine the pharmacokinetics following IV infusion or SC injection of PGT121 mAb in HIV-uninfected and HIV-infected adults:<br><br>1. Elimination half-life (t1/2)<br>2. Clearance (CL/F)<br>3. Volume of distribution (Vz/F)<br>4. Area under the concentration decay curve (AUC)<br>5. Impact of viral load and/or ART on PGT121 disposition (elimination half-life (t1/2), clearance (CL/F), volume of distribution (Vz/F), total exposure<br><br>*Antiviral activity:*<br>We will calculate the following endpoints to determine the antiviral activity following IV infusion of PGT121 mAb in viremic HIV-infected adults not on ART:<br><br>1. Change in plasma HIV-1 RNA levels from baseline (mean of pre-entry and entry values)<br><br>***Secondary***:<br><br>*Anti-PGT121 antibodies:*<br>We will calculate the following endpoint to determine if anti-PGT121 antibodies are elicited following IV infusion or SC injection of PGT121 mAb in HIV-uninfected and HIV-infected adults:<br><br>1. Change in serum anti-PGT121 antibody titers from baseline<br><br>*CD4+ T cell count:*<br>We will calculate the following endpoint to determine if IV infusion of

PGT121 mAb has any impact on CD4+ T cell counts in HIV-infected adults:

1. Change in CD4+ T cell count and frequency compared to baseline as measured by single platform flow cytometry

HIV genotyping/phenotyping of circulating virus for evaluation of PGT121 mAb -induced escape mutations:
We will compare plasma virus sequences before and after IV infusion of PGT121 mAb to determine if PGT121 mAb induces viral escape mutations in viremic HIV-infected adults not on ART.

1. Development of sequence variations in epitopes known to result in reduced PGT121 mAb neutralization susceptibility.

Exploratory:

Additional assessments may include, but are not limited to, the following: HIV-specific IgG/IgA binding responses by ELISA, HIV-specific cellular immune responses by ELISPOT, HIV-specific antibody function by ADCC, ADCP, and ADCVI assays, resistance mutations to current ARVs, PGT121 mAb levels in mucosal secretions, changes in total HIV-1 DNA and 2-long terminal repeat (LTR) circular HIV-1 DNA in resting or total CD4 T cells and in vitro neutralization of HIV isolates with participant's serum post administration of PGT121 mAb.

STUDY DESIGN TABLE

Part	Group	Participants	Sub-Group	Regimen	N	Dose (mg/kg) - administration
Part 1[1]	1[3]	HIV-uninfected participants	1A	PGT121/Placebo	4/1 (6/2 if DLT[2])	3 IV
			1B	PGT121/Placebo	4/1 (6/2 if DLT)	10 IV
			1C	PGT121/Placebo	4/1 (6/2 if DLT)	30 IV
			1D	PGT121/Placebo	4/1 (6/2 if DLT)	3 SC
	2[3]	HIV-infected on ART, (<50 cp/ml)	2A	PGT121/Placebo	4/1 (6/2 if DLT[2])	3 IV
			2B	PGT121/Placebo	4/1 (6/2 if DLT)	10 IV
			2C	PGT121/Placebo	4/1 (6/2 if DLT)	30 IV
Safety Monitoring Committee Review[4]						
Part 2	3[5]	HIV-infected off ART (VL $2x10^3$ – $1x10^5$cp/ml)	3A[6]	PGT121	6 (max 9)	30 IV
			3B	PGT121	8 (max 12)	10 IV
			3C	PGT121	10 (max 15)	3 IV
		HIV-Infected off ART (VL $1x10^2$ – $2x10^3$ cp/ml)	3D[7]	PGT121	3	30 IV
			3E	PGT121	3	10 IV
			3F	PGT121	3	3 IV

DLT, dose limiting toxicity; ART, antiretroviral therapy; cp, copies; ml, milliliter
Administration of PGT 121 will be by intravenous infusion (IV) or subcutaneous injection (SC)

1. Eligible participants for Groups 1 and 2 will be enrolled according to their HIV-serostatus and will occur in parallel. At each dose level in Part 1, investigational product (IP) administration will be separated by at least 24 hours for each of the first 3 participants. Randomization will ensure at least 2 participants receive active product and are observed for at least 24 hours before administration to additional participants.
2. A dose limiting toxicity (DLT) will be defined as 1) any Grade 3 or greater reactogenicity or adverse event judged by the study investigators as at least possibly related to investigational product or 2) any SAE considered at least possibly related to investigational product.
3. The PSRT will review safety data to determine dose escalation. If no DLT occurs within 2 weeks from infusion of the first 5 participants in a dose subgroup (e.g. 1A), dose escalation to the next dose subgroup will proceed (e.g. 1B). If 1 DLT occurs in a dose subgroup (e.g. 1A), 3 additional participants will be enrolled into that dose subgroup; these 3 participants will be randomized between PGT121 mAb and placebo at a 2:1 ratio. If no additional DLTs occur in that subgroup (e.g. 1A) within 2 weeks of infusion in the 8 total participants (3:1 ratio of PGT121 mAb to placebo), the study can proceed with enrolment of the next dose subgroup (e.g. 1B). If 2 or more DLTs accumulate in a dose subgroup (e.g. 1A) that are the same, similar, or in the same System Organ Class, dosing will be halted in that subgroup (e.g. 1A) and the next lower dose level will be declared the maximum tolerated dose (MTD) for that subgroup (e.g. 1A). When groups are enrolled in parallel, if the MTD is determined in one group (e.g. Group 1) due to the occurrence of 2 or more DLTs in this group, dosing of participants in the parallel group (e.g. Group 2) will be held until the PSRT has reviewed the safety data and determined

	whether the MTD should be applied to both groups. If no DLT occurs in one of the final dose subgroups after 14 days of follow up, MTD will be the highest dose given (subgroups 1C or 2C 30mg/kg). Dose escalation in Groups 1 and 2 may occur in parallel, and can occur independent of each other.<br>4. Following IP administration of IV PGT121 in the last participant in either Group 1 or 2, an independent Safety Monitoring Committee (SMC) will review at least the first 14 days of safety data to confirm MTD, and determine whether, and at what dose, Group 3 can initiate enrollment. The SMC can meet to confirm MTD when either Group 1 or Group 2 has finished enrollment, and does not need to wait for both Groups to be completed to approve initiation of Group 3 enrollment. Data from Group 1D (SC administration) is not considered by SMC for initiation of Group 3 enrollment.<br>5. Group 3 will start with the MTD as determined in Part 1. Group 3 will start with subgroups 3A and 3D if the MTD is 30mg/kg, subgroups 3B and 3E if the MTD is 10mg/kg and subgroups 3C and 3F if the MTD is 3mg/kg.<br>6. If subgroup 3A achieves a decline in HIV RNA significantly greater than 0.9 logs compared to baseline, enrolment into subgroup 3A will be stopped and the next lower dose subgroup 3B will be initiated. The same rule will apply for progression from subgroup 3B to 3C. At least 6, 8 and 10 but up to 9, 12 and 15 participants, will be enrolled in subgroups 3A, 3B, and 3C respectively, until the minimum effective dose is determined. If a decline significantly greater than 0.9 logs in HIV RNA is not achieved, enrollment will be stopped at the completion of enrolment at that dose level.<br>7. As soon as subgroup 3D has enrolled 3 participants, enrolment into subgroup 3E will be initiated. The same rule will apply for progression from subgroup 3E to 3F. In the event that dose de-escalation in subgroups 3A-C is stopped due to failure to meet virologic endpoints, dose de-escalation in subgroups 3D-F will continue.
**METHODS**	See Schedule of Procedures, Appendices A, B and C
**STUDY POPULATION**	The study population will include three different groups:<br>Group 1 will include HIV-uninfected males or females aged 18-50 years old who are willing to maintain low risk behavior for HIV infection; principal exclusion criteria include confirmed HIV-infection, pregnancy or lactation, significant acute or chronic disease and clinically significant laboratory abnormalities. Group 2 will include HIV-infected males or females aged 18-65 years old on a stable antiretroviral regimen with HIV-1 RNA plasma level <50 copies/ml, CD4 cell count ≥ 300 cells/uL; principal exclusion criteria include history of AIDS-defining illness within the previous 5 years, significant acute or chronic medical condition other than HIV infection, and clinically significant laboratory abnormalities. Group 3 will include HIV-infected males or females aged 18-65 years old, not on antiretroviral therapy for > 6 month with detectable HIV-1 viral load between 100 and 100,000 copies/ml, CD4 cell count ≥ 300 cells/uL; principal exclusion criteria include history of AIDS-defining illness within the previous 5 years, significant acute or chronic medical condition other than HIV infection, and clinically significant laboratory abnormalities.
**NUMBER OF PARTICIPANTS**	68-101 participants will be included.
**DOSE ESCALATION and PAUSE RULES**	The first part of this study is a dose-escalation trial in HIV-uninfected adults and HIV-infected adults on ART with suppressed viral load, as indicated in the study design table.<br><br>If 2 or more DLTs accumulate in a dose subgroup that are the same, similar, or in the same System Organ Class, dosing will be halted and the next lower dose level will be declared the maximum tolerated dose (MTD) within this group. If no DLT occurs in one of the final dose groups after 14

	days of follow-up, MTD will be the highest dose given (subgroups 1C or 2C 30mg/kg).<br><br>The Protocol Safety Review Team (PSRT) will review safety data through at least day 14 post-IP administration for the first 5 participants in each dose subgroup (e.g. 1A) prior to allowing enrolment of participants into the next dose subgroup (e.g. 1B). Dose escalation in Groups 1 and 2 may occur in parallel, and can occur independent of each other.<br><br>Following IP administration of IV PGT121 in the last participant in either Group 1 or 2, an independent Safety Monitoring Committee (SMC) will review safety data through at least day 14 post-IP administration for all participants to confirm MTD, and determine whether, and at what dose, Group 3 can initiate enrollment. The SMC can meet to confirm MTD when either Group 1 or Group 2 has finished enrollment, and does not need to wait for both Groups to be completed to approve initiation of Group 3 enrollment. Data from Group 1D (SC administration) is not considered by SMC for initiation of Group 3 enrollment.<br><br>The second part of this study is a dose-de-escalation trial in HIV-infected adults not on ART, as indicated in the study design table.<br><br>The study will be paused for a safety review by the investigators and the independent SMC if 1) 1 or more participants experiences a Serious Adverse Event that is judged possibly, probably or definitely related to the IP, 2) There is a participant death, regardless of relationship to the IP, 3) if 2 or more participants experience grade 3 adverse events in the same System Organ Class that are considered to be at least possibly related to IP or 4) any grade 4 adverse event that is considered to be possibly, probably, or definitely related to IP. See protocol section 17.3.
**FORMULATIONS, VOLUMES AND ROUTES OF ADMINISTRATION**	**PGT121 mAb**: PGT121 mAb is a recombinant, fully human monoclonal antibody (mAb) of the IgG1 isotype that binds to the HIV envelope. The concentration and volume of product in each vial is 50 mg/mL, 6mL in each vial. PGT121 mAb will be given intravenously or subcutaneously in this study.
**DURATION OF STUDY PARTICIPATION**	Participants will be screened up to 56 days (Groups 1 and 2) or 42 days (Group 3) before IP administration and will be followed for 24 weeks. The anticipated study duration for each participant is approximately 6 months from screening through last study visit. It is anticipated that it will take approximately 6 months to enroll Groups 1 and 2. It is anticipated that it will take approximately 16 months to enroll the entire study. The exact duration depends on the recruitment rate and how many participants will be required per dose group.
**RANDOMIZATION and BLINDING**	This is a randomized, placebo-controlled study for groups 1 and 2, and open label for group 3 who will not receive placebo.
**EVALUATION FOR INTERCURRENT HIV INFECTION:**	Participants in Group 1 (HIV-uninfected) will be tested for HIV according to the Schedule of Procedures. Test results will be interpreted according to a pre-determined diagnostic algorithm. HIV testing at additional time points may be performed upon the request of the participant and Principal Investigator or designee as medical or social circumstances warrant.

**SAFETY MONITORING AND STATISTICAL CONSIDE-RATIONS:**	All clinical trial data collected, identified only by a study identification number, will be entered into the clinical trial database.<br><br>Safety will continually be monitored by the Investigators, the Sponsor's Medical Monitor and a Protocol Safety Review Team (PSRT); detailed pause criteria are pre-defined.<br><br>Safety data will be reviewed by an independent Safety Monitoring Committee (SMC). *Ad hoc* safety review may be specifically requested by the Sponsor, the Principal Investigators, Ethics Committees, Regulatory Authorities, or by the SMC. All clinical and routine laboratory data will be included in the safety analysis. At the end of the study, a full analysis will be prepared.

TABLE OF CONTENTS

ABBREVIATIONS **12**
CONTACT INFORMATION **13**
1.0 SIGNATURE PAGE **14**
2.0 INTRODUCTION AND BACKGROUND INFORMATION **15**
2.1 Study Rationale 15
2.2 Experience with PGT121 16
3.0 STUDY OBJECTIVES **16**
3.1 Primary Objectives 16
3.2 Secondary Objectives 16
3.3 Exploratory Objectives: 16
4.0 STUDY ENDPOINTS **17**
4.1 Study Endpoints 17
4.1.1 Primary Endpoints 17
4.1.2 Secondary Endpoints 17
4.1.3 Exploratory Endpoints 18
5.0 STUDY DESIGN **18**
5.1 Definition of Dose Limiting Toxicity and Maximum Tolerated Dose 18
5.2 Dose Escalation – Groups 1 and 2: Determination of Maximum Tolerated Dose 18
5.3 Dose De-Escalation- Group 3: Determination of Minimum Effective Dose 20
5.4 Duration of the Study 22
5.5 Study Population 22
5.6 Inclusion Criteria 23
5.7 Exclusion Criteria 24
5.8 Recruitment of Participants 28
6.0 STUDY VISITS **28**
6.1 Screening Period 28
6.2 IV infusion of PGT121 mAb Visit 29
6.3 Post-IV infusion of PGT121 mAb Visits 30
6.4 Additional Follow-up Visits 30
6.5 Unscheduled Visits 30
6.6 Final Study Visit or Early Termination Visit 31
7.0 STUDY PROCEDURES **31**
7.1 Informed Consent Process 31
7.2 Medical History and Physical Examination 31
7.3 HIV Testing and HIV-test Counselling (Group 1) 32
7.4 HIV Risk Reduction Counselling 32
7.5 Family Planning Counselling 32
7.6 ART Counselling (Group 3) 32
7.7 Specimens 33
7.8 Reimbursement 33
7.9 Randomization and Blinding 33
7.10 Un-blinding Procedure for Individual Participants 34
7.11 Assessment of IP related HIV sero-positivity 34

8.0 INVESTIGATIONAL PRODUCT 34
8.1 Description 34
8.2 Shipment and Storage 35
8.3 Preparation of Investigational Product (IP) 35
8.4 Administration of Investigational Product 35
8.5 Accountability and Disposal of Investigational Product 35
9.0 ASSESSMENTS 36
9.1 Safety Assessments 36
9.1.1 Local reactogenicity 36
9.1.2 Systemic reactogenicity 36
9.1.3 Vital Signs 36
9.1.4 Other Adverse Events 37
9.1.5 Concomitant Medications 37
9.1.6 Routine laboratory parameters 37
9.1.7 Specific screening tests: 38
9.2 Virologic Assessments 38
9.3 Exploratory Immunogenicity Assessments 38
9.3.1 Antibody Responses 38
9.3.2 Cellular Responses 39
9.3.3 PBMC, Serum and Plasma Storage 39
9.4 Other Assessments 39
9.4.1 HIV Antibody Testing (Group 1) 39
9.4.2 Pharmacokinetics 39
9.4.3 HLA Typing 40
9.4.5 Pregnancy Test 40
9.4.6 HIV Risk Assessment (Group 1) 40
9.4.7 Social Impact Assessment 40
10.0 ADVERSE EVENTS 40
10.1 Definition 40
10.2 Assessment of Severity of Adverse Events 40
10.3 Relationship to Investigational Product 41
10.4 Serious Adverse Events 42
10.5 Reporting Potential Immune-Mediated Diseases 43
10.6 Clinical Management of Adverse Events 44
10.7 Pregnancy 44
10.8 Intercurrent HIV Infection (Group 1) 44
11.0 MANAGEMENT OF HIV ISSUES DURING AND FOLLOWING STUDY 45
11.1 HIV Testing – Group 1 45
11.2 Social Discrimination as a Result of IP-related antibodies 45
11.3 HIV infection – Group 1 45
11.3.1 Counselling 45
11.3.2 Referral for Support/Care 46
12.0 WITHDRAWAL FROM STUDY 46
12.1 Deferral of IV infusion of IP 46
12.2 Withdrawal from the Study (Early Termination) 46
13.0 DATA HANDLING 47
13.1 Data Collection and Record Keeping at the Study Site 47

13.3 Data Entry at the Study Site 47
13.4 Data Analysis 47
14.0 STATISTICAL CONSIDERATIONS 48
14.1 Safety and Tolerability Analysis...48
14.2 Pharmacokinetic Analysis 49
14.3 Virologic Analysis for Dose De-escalation in Groups 3A-C 49
14.4 Analysis of Antiviral Activity and Dose De-escalation in Subgroups 3D-F....................52
14.5 Secondary and Exploratory Immunologic and Virologic Analyses 52
15.0 QUALITY CONTROL AND QUALITY ASSURANCE...53
16.0 DATA AND BIOLOGICAL MATERIAL 53
17.0 ADMINISTRATIVE STRUCTURE 53
17.1 Protocol Safety Review Team 53
17.2 Safety Monitoring Committee (SMC) 54
17.2.1 Content of Interim Safety Review 54
17.2.2 SMC Review of Group 1 and 2 data prior to starting Group 3......................................54
17.3 Criteria for Pausing the Study..55
17.4 Study Supervision 56
17.5 Study Monitoring 56
17.6 Investigator's Records...56
18.0 INDEMNITY 56
19.0 PUBLICATION...57
20.0 ETHICAL CONSIDERATIONS..57
APPENDIX A: SCHEDULE OF PROCEDURES – GROUP 1 (A, B, C) 58
APPENDIX B: SCHEDULE OF PROCEDURES – GROUP 2 (A, B, C) 60
APPENDIX C: SCHEDULE OF PROCEDURES – GROUP 3 (A, B, C, D, E, F) 63
APPENDIX D: LOW RISK CRITERIA..66
APPENDIX E: ADVERSE EVENT SEVERITY ASSESSMENT TABLE 68
APPENDIX F CTCAE TABLE 92
APPENDIX G REFERENCES...166

ABBREVIATIONS

Abbreviation	Term
**AE**	Adverse Event
**AIDS**	Acquired Immunodeficiency Syndrome
**ALT**	Alanine-Aminotransferase
**ART**	Antiretroviral Therapy
**AST**	Aspartate-Aminotransferase
**CFC**	Cytokine Flow Cytometry
**CMI**	Cell Mediated Immunity
**CRF**	Case Report Form
**CTL**	Cytotoxic T Lymphocyte
**DCC**	Data Coordinating Center
**DLT**	Dose Limiting Toxicity
**DNA**	Deoxyribonucleic Acid
**ELISA**	Enzyme Linked Immunosorbent Assay
**GCP**	Good Clinical Practice
**GFR**	Glomerular Filtration Rate
**HIV**	Human Immunodeficiency Virus
**HLA**	Human Leukocyte Antigen
**HSV**	Herpes Simplex Virus
**IAVI**	International AIDS Vaccine Initiative
**ICH**	International Council for Harmonization of Technical Requirements for Pharmaceuticals for Human Use
**IP**	Investigational Product
**IND**	Investigational New Drug Application
**IV**	Intravenous
**Kg**	Kilogram
**mAb**	Monoclonal Antibody
**mg**	Milligram
**MED**	Minimum Effective Dose
**MTD**	Maximum Tolerated Dose
**NHP**	Non Human Primate
**PCR**	Polymerase Chain Reaction
**PBMC**	Peripheral Blood Mononuclear Cells
**PK**	Pharmacokinetic
**RPR**	Rapid Plasma Reagin
**SAE**	Serious Adverse Event
**SIV**	Simian Immunodeficiency Virus
**SOP**	Standard Operating Procedure
**SOM**	Study Operations Manual
**SMC**	Safety Monitoring Committee
**STD**	Sexually Transmitted Disease
**TPHA**	Treponema Pallidum Hemagglutination

CONTACT INFORMATION

Detailed contact information provided in the Study Operation Manual (SOM)

**Sponsor Contact:**	
Frances Priddy MD MPH<br>Executive Director and Chief Medical Officer<br>International AIDS Vaccine Initiative<br>125 Broad Street, 9th Floor<br>New York, New York 10004	Phone: +1-212-328-7461<br>Mobile: +1-646-287-8943<br>Fax: +1-608-203-5501<br>E-mail: fpriddy@iavi.org
**Clinical Research Center Contacts:**	
Kathryn Stephenson MD MPH<br>Center for Virology and Vaccine Research<br>Clinical Trials Unit<br>Beth Israel Deaconess Medical Center<br>E / CLS – 1036<br>330 Brookline Avenue<br>Boston, Massachusetts 02215	Phone: +1-617-735-4556<br>Mobile: +1-917-836-9150<br>Fax: +1-617-735-4566<br>E-mail: kstephen@bidmc.harvard.edu

.

1.0 SIGNATURE PAGE

The signatures below constitute the approval of this protocol and the appendices and provide the necessary assurances that this study will be conducted in compliance with the protocol, Good Clinical Practices (GCP) and the applicable regulatory requirement(s).

Sponsor:

Signed: See electronic signature manifest Date:

Frances Priddy MD MPH
Executive Director and Chief Medical Officer, Medical Affairs, IAVI

Principal Investigator:

Signed: Date:

Name (please print):

Name of institution (please print):

2.0 INTRODUCTION AND BACKGROUND INFORMATION

More than 78 million people have been infected with HIV and 39 million people have died since the beginning of the AIDS epidemic[1]. In 2014, there were 1.2 million deaths attributable to HIV infection and 2 million newly infected with HIV[2]. One reason that such high rates of AIDS-related deaths continue to occur globally – despite the advent of drugs that are highly effective at suppressing HIV replication – is that only two in five people living with HIV have access to antiretroviral therapy[1]. The other reason for continued AIDS-related mortality is that ART does not cure HIV infection and must be maintained for a lifetime. Even in the United States (US), only 30% of the 1.2 million people living with HIV have suppressed HIV to undetectable levels, despite the fact that most HIV-infected people in the US have access to ART[3] It is clear that antiretroviral therapy is necessary but not sufficient to end the AIDS epidemic, both in the US and globally, and that novel efforts to prevent and treat HIV are critically needed.

2.1 Study Rationale

This is a Phase 1 study to evaluate the safety, tolerability, pharmacokinetics and anti-viral efficacy of the PGT121 monoclonal antibody for HIV prevention and therapy. PGT121 mAb is a recombinant human IgG1 monoclonal antibody that targets a V3 glycan-dependent epitope region of the HIV envelope protein[4,5]. PGT121 mAb was chosen for this study because it is potent, neutralizes a wide array of HIV viruses, and can prevent and treat simian-human immunodeficiency virus (SHIV) in rhesus monkeys.

The recent discovery of multiple potent and broadly neutralizing antibodies (bNAbs) against HIV has led to the re-emergence of the concept that antibodies may be useful for both prevention and therapy. HIV-specific antibodies that target the HIV envelope (Env) can prevent SHIV infection in rhesus monkeys and have shown to reduce HIV RNA levels in humans temporarily[6-10]. Until recently, these antibodies were few in number, targeted a narrow spectrum of HIV strains and Env epitopes, and were not potent enough for practical use. In the last five years, the field has changed dramatically: new developments in high throughput single-cell PCR-amplification and novel soluble Env baits have led to the isolation of new monoclonal antibodies with extraordinary potency and breadth[11]. These bNAbs may be effective for prevention of HIV infection when administered passively[12,13].

PGT121 mAb was selected for development because of the following critical attributes:

- PGT121 mAb is 10 to 100-fold more potent than the previous best-in-class CD4bs antibodies VRC01, VRC07, and 3BNC117 [11,14,15].
- PGT121 mAb affords superior protective efficacy against SHIV acquisition in monkeys compared to VRC01, 3BNC117, and 10-1074 [16] (and unpublished data).
- PGT121 mAb has superior therapeutic efficacy in SHIV-infected monkeys compared to VRC01, 3BNC117, and 10-1074 [7] (and unpublished data).
- PGT121 mAb may have a higher bar to escape in vivo as compared with other V3 glycan and CD4bs antibodies as a result of making multiple glycan contacts [14].
- PGT121 mAb combined with PGDM1400 (a novel bNab targeting the envelope trimer apex) neutralizes 98-99% of global HIV-1 viruses tested and has unparalleled potency with a median IC50 of 0.007 μg/ml[14].

The potency and breadth of PGT121 mAb, both alone and in combination with other bNAbs, raise the possibility that combinations may be effective for HIV prophylaxis at

low doses and against global viruses. An antibody that is effective at low doses may eventually be given subcutaneously, which would reduce the cost. It is these features that make PGT121 mAb particularly well-suited for preventing and/or treating HIV in the developing world, where it is critical that a public health intervention be low cost, easy to deliver, and effective in diverse settings.

2.2 Experience with PGT121

There is no previous clinical experience with PGT121 mAb. Several other HIV monoclonal antibodies are currently in clinical development as passive HIV immunoprophylaxis, or as potential therapeutics. Data from phase 1 studies shows acceptable preliminary safety and tolerability profiles for these products, but varying levels of anti-viral effects [6,17]. A comprehensive summary of phase 1 studies of HIV monoclonal antibodies can be found in the Investigator's Brochure.

3.0 STUDY OBJECTIVES

3.1 Primary Objectives

- To evaluate the safety and tolerability of a single intravenous (IV) infusion of PGT121 mAb at 3, 10, and 30 mg/kg in HIV-uninfected adults and HIV-infected adults.
- To evaluate the safety and tolerability of a single subcutaneous (SC) injection of PGT121 mAb at 3 mg/kg in HIV-uninfected adults
- To evaluate the pharmacokinetic (PK) profile of IV infusion and SC injection of PGT121 mAb in HIV-uninfected and HIV-infected adults.
- To evaluate the antiviral activity of IV infusion of PGT121 mAb in HIV-infected adults not on ART.

3.2 Secondary Objectives

- To determine if PGT121 mAb induces anti-PGT121 antibodies.
- To determine the effect of PGT121 mAb on CD4 T-cell counts in HIV-infected adults.
- To determine if PGT121 mAb induces viral escape mutants in viremic HIV-infected adults not on ART.

3.3 Exploratory Objectives:

- To determine if PGT121 mAb has any impact on the host immune responses (i.e., HIV-specific cellular and humoral immune response).
- To determine the effect of PGT121 mAb on the size of the latent HIV reservoir in HIV-infected adults.
- To determine PGT121 mAb levels in mucosal secretions in HIV-uninfected and HIV-infected adults.
- To measure in vitro neutralization of HIV isolates with participant's serum post PGT121 mAb IV infusion.
- To determine if PGT121 mAb has any impact on resistance mutations to ARVs.

4.0 STUDY ENDPOINTS

4.1 Study Endpoints

4.1.1 Primary Endpoints

Safety and Tolerability:

We will measure the following endpoints to evaluate the safety and tolerability of PGT121 mAb in HIV-uninfected and HIV-infected adults:

1. Proportion of participants with moderate or greater reactogenicity (e.g., solicited adverse events) for 3 days following IV infusion or SC injection of PGT121 mAb.
2. Proportion of participants with moderate or greater and/or PGT121 mAb-related unsolicited adverse events (AEs), including safety laboratory (biochemical, hematological) parameters, following IV infusion or SC injection of PGT121 mAb for the first 56 days post administration of Investigational Product.
3. Proportion of participants with PGT121 mAb-related serious adverse events (SAEs) throughout the study period.

Pharmacokinetics:

We will measure the following endpoints to determine the pharmacokinetics following IV infusion or SC injection of PGT121 mAb in HIV-uninfected and HIV-infected adults:

1. Elimination half-life (t1/2)
2. Clearance (CL/F)
3. Volume of distribution (Vz/F)
4. Area under the concentration decay curve (AUC)
5. Impact of viral load and/or ART on PGT121 disposition (elimination half-life (t1/2), clearance (CL/F), volume of distribution (Vz/F), total exposure

Antiviral activity:

We will calculate the following endpoints to determine the antiviral activity following IV infusion of PGT121 mAb in viremic HIV-infected adults not on ART.

1. Change in plasma HIV-1 RNA levels from baseline (mean of pre-entry and entry values)

4.1.2 Secondary Endpoints

Anti-PGT121 antibodies:

We will calculate the following endpoint to determine if anti-PGT121 antibodies are elicited following IV infusion or SC injection of PGT121 mAb in HIV-uninfected and HIV-infected adults:

1. Change in serum anti-PGT121 antibody titers from baseline

CD4+ T cell count:

We will calculate the following endpoint to determine if IV infusion of PGT121 mAb has any impact on CD4+ T cell counts in HIV-infected adults:

1. Change in CD4+ T cell count and frequency compared to baseline as measured by single platform flow cytometry

HIV genotyping/phenotyping of circulating virus for evaluation of PGT121-induced escape mutations:

We will compare plasma virus sequences before and after IV infusion of PGT121 mAb to determine if PGT121 mAb induces viral escape mutations in viremic HIV-infected adults not on ART.

1. Development of sequence variations in epitopes known to result in reduced PGT121 neutralization susceptibility

4.1.3 Exploratory Endpoints

Additional assessments may include but are not limited to the following: HIV-specific IgG/IgA binding responses by ELISA, HIV-specific cellular immune responses by ELISPOT, HIV-specific antibody function by ADCC, ADCP, and ADCVI assays, resistance mutations to current ARVs, PGT121 mAb levels in mucosal secretions, changes in total HIV-1 DNA and 2-long terminal repeat (LTR) circular HIV-1 DNA in resting or total CD4 T cells and in vitro neutralization of HIV isolates with participant's serum post administration of PGT121 mAb.

5.0 STUDY DESIGN

The study is a randomized, placebo-controlled study for groups 1 and 2, and open label for group 3 who will not receive placebo.

5.1 Definition of Dose Limiting Toxicity and Maximum Tolerated Dose

A dose limiting toxicity (DLT) will be defined as 1) any Grade 3 or greater reactogenicity or adverse event judged by the study investigators as at least possibly related to investigational product or 2) any SAE considered at least possibly related to investigational product.
Maximum Tolerated Dose (MTD) will be declared when 2 or more DLTs occur that are the same, similar, or in the same System Organ Class or if no DLT occurs in the final dose subgroup after 14 days of follow-up, MTD will be the highest dose given (groups 1C or 2C 30mg/kg).

5.2 Dose Escalation – Groups 1 and 2: Determination of Maximum Tolerated Dose

In Groups 1 and 2, the administrations of PGT121 mAb escalate by dose as shown below in Table 5.3.1, Study Design (5 participants per dose subgroup, 4:1 ratio of IP to placebo for each dose subgroup).

Sentinel Recipients

Within each dose group (subgroups 1A and 2A, subgroups 1B and 2B, subgroups 1C and 2C, subgroup 1D), the first 3 participants will be separated by at least 24 hours, to allow for observation of Investigational product (IP)-related adverse events. Dose subgroups will be enrolled in parallel, meaning that the 1st participant may be from subgroup 1A, the 2nd from subgroup 2A, the 3rd from subgroup 2A, all with 24 hours in between dosing. Because there is 1 placebo in each dose subgroup and the subgroups are dosed in parallel, the first 3 recipients will be treated as sentinel recipients (randomization will ensure that at least 2 will receive the IP).

- If no reactogenicity and adverse events that are considered to be related to IP (possibly, probably or definitely related) and are graded as severe or worse (Grade 3 or 4 on the DAIDS Toxicity Table or CTCAE table, see section 9.1.2) occur within 24 hours after infusion of the first participant, the second participant may be injected.
- If no events meeting the criteria described above occur within 24 hours after the 3rd participant is infused, then the remainder of participants in that dose group will be infused.
- If events meeting the criteria described above do occur for the first, second, or third participant in a dose group, they will be reviewed by the Safety Monitoring Committee (SMC) to determine whether further infusions may proceed.

Safety information will be reviewed by the Principal Investigator. The outcome of the safety review, and decision whether or not to dose the next participant(s) or contact the SMC will be communicated with the Sponsor.

Dose Escalation and Determination of Maximum Tolerated Dose

Safety data through at least day 14 post-IP administration visit for the first 5 participants in a dose subgroup (e.g. 1A) will be reviewed by the Protocol Safety Review Team (PSRT) prior to allowing enrollment of participants into the next dose subgroup (e.g. 1B). Dose escalation in Groups 1 and 2 may occur in parallel, and can occur independent of each other. Following administration of IP for the last participant in either Group 1C or 2C, an independent Safety Monitoring Committee (SMC) will review safety data to confirm Maximum Tolerated Dose (MTD) and determine whether, and at what dose, Group 3 can initiate enrollment. The SMC can meet to confirm MTD when either Group 1C or Group 2C has finished enrollment, and does not need to wait for both Groups to be completed to approve initiation of Group 3 enrollment. Data from Group 1D (SC administration) is not considered by SMC for initiation of Group 3 enrollment.

- If no DLT occurs within 2 weeks from infusion of the first 5 participants in a dose subgroup (e.g. 1A), dose escalation to the next dose subgroup (e.g. 1B) will proceed.
- If 1 DLT occurs in a dose subgroup (e.g. 1A), 3 additional participants will be enrolled in that dose subgroup; these 3 participants will be randomized between PGT121 mAb and placebo at a 2:1 ratio.
    - If no additional DLTs occur in that dose subgroup (e.g. 1A) within 2 weeks of infusion in the 8 total participants (3:1 ratio of PGT121 mAb to placebo), the study can proceed with enrollment of the next dose subgroup (e.g. 1B).

  - If 2 or more DLTs accumulate in a dose subgroup (e.g. 1A) that are the same, similar, or in the same organ class, dosing will be halted in that subgroup (e.g. 1A) and the next lower dose level will be declared the maximum tolerated dose (MTD) for that subgroup (e.g. 1A).
- When groups are enrolled in parallel, if the MTD is determined in one group (e.g. Group 1) due to the occurrence of 2 or more DLTs in this group, dosing of participants in the parallel group (e.g. Group 2) will be held until the PSRT has reviewed the safety data and determined whether the MTD should be applied to both groups.
- If no DLT occurs in the one of the final dose subgroups, MTD will be the highest dose given (subgroups 1C or 2C 30mg/kg) after 14 days of follow-up.

5.3 Dose De-Escalation- Group 3: Determination of Minimum Effective Dose

Upon approval by the SMC (see section 17.2.2), group 3 (Part 2), PGT121 mAb administrations will de-escalate by dose as shown below in Table 5.3.1.

Group 3 will start with the MTD (i.e. subgroups 3A and 3D if the MTD is 30 mg/kg, subgroups 3B and 3C if the MTD is 10 mg/kg, or subgroups 3C and 3F if the MTD is 3 mg/kg) as determined by the SMC from data in Part 1.

If subgroup 3A (n = 6) achieves a decline in HIV RNA significantly greater than ≥0.9 log compared to baseline, enrollment into subgroup 3A will be stopped, and the next lower dose subgroup 3B will be initiated. The same rule will apply for progression from subgroup 3B to 3C. At least 6, 8 and 10 but up to 9, 12 and 15 participants will be enrolled in subgroups 3A, 3B and 3C respectively, until the minimum effective dose is determined. In each subgroup, if a decline significantly greater than 0.9 log in HIV RNA is not achieved, enrollment will be stopped at the completion of enrollment at that dose level.

Three participants will be enrolled in each group 3D, 3E and 3F. As soon as subgroup 3D has enrolled 3 participants, enrollment into subgroup 3E will be initiated. The same rule will apply for progression from subgroup 3E to 3F. In the event that dose de-escalation in subgroups 3A-C is stopped due to failure to meet virologic endpoints, dose de-escalation in subgroups 3D-F will continue.

Table 5.3.1 Study Design Table

	Group	Participants	Sub-Group	Regimen	N	Dose (mg/kg) administration
Part 1	1	HIV-uninfected participants	1A	PGT121/Placebo	4/1 (6/2 if DLT)	3 IV
			1B	PGT121/Placebo	4/1 (6/2 if DLT)	10 IV
			1C	PGT121/Placebo	4/1 (6/2 if DLT)	30 IV
			1D	PGT121/Placebo	4/1 (6/2 if DLT)	3 SC
	2	HIV-infected on ART, (<50 cp/ml)	2A	PGT121/Placebo	4/1 (6/2 if DLT)	3 IV
			2B	PGT121/Placebo	4/1	10 IV

					(6/2 if DLT)	
			2C	PGT121/Placebo	4/1<br>(6/2 if DLT)	30 IV
Safety Monitoring Committee Review						
Part 2	3	HIV-infected off ART (VL $2x10^3$ – $1x10^5$cp/ml)	3A	PGT121	6<br>(max 9)	30 IV
			3B	PGT121	8<br>(max 12)	10 IV
			3C	PGT121	10<br>(max 15)	3 IV
		HIV-infected off ART (VL $1x10^2$ – $2x10^3$ cp/ml)	3D	PGT121	3	30 IV
			3E	PGT121	3	10 IV
			3F	PGT121	3	3 IV

Table 5.3.2 Dose De-Escalation- Group 3

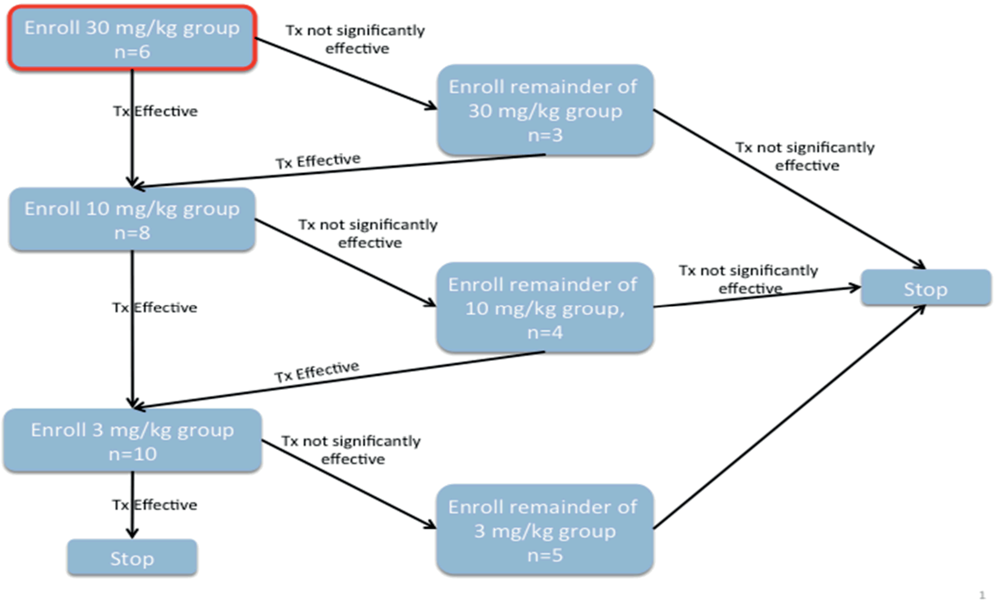

"not significantly effective" = does not achieve a decline in HIV RNA significantly greater than 0.9 logs compared to baseline

5.4 Duration of the Study

Participants will be screened up to 56 days (Groups 1 and 2) or 42 days (Group 3) before IP administration of PGT121 mAb and will be followed for 24 weeks.

It will take approximately 16 months to enroll the entire study. The exact duration depends on the recruitment rate and how many participants will be required per dose group as specified in sections 5.2 and 5.3.

5.5 Study Population

The study population consists of HIV-uninfected male or female adults (Group 1), HIV-infected male or female adults on ART (Group 2), and HIV-infected males and female adults not on ART (group 3) who meet the detailed inclusion and exclusion criteria listed below, and who in the opinion of the investigator or designee, understand the study and provide written informed consent.

Approximately 68-101 participants (87 investigational product recipients, 14 placebo recipients) who meet all eligibility criteria will be included in the study. An over-enrollment of up to 5% (up to 5 participants total) will be permitted in the study to facilitate rapid enrollment.

5.6 Inclusion Criteria

Inclusion criteria for all participants:

1. Willing to comply with the requirements of the protocol and available for follow-up for the planned duration of the study;

2. In the opinion of the Principal Investigator or designee and based on Assessment of Informed Consent Understanding results, has understood the information provided and potential impact and/or risks linked to IV infusion or SC injection and participation in the trial; written informed consent will be obtained from the participant before any study-related procedures are performed;

3. All heterosexually active female participants must commit to use an effective method of contraception for 3 months following IP administration, including:
    a. Condoms (male or female) with or without spermicide
    b. Diaphragm or cervical cap with spermicide
    c. Intrauterine device, or contraceptive implant
    d. Hormonal contraception
    e. Successful vasectomy in the male partner (considered successful if a woman reports that a male partner has [1] documentation of azoospermia by microscopy (< 1 year ago), or [2] a vasectomy more than 2 years ago with no resultant pregnancy despite sexual activity post-vasectomy)
    f. Not be of reproductive potential, such as having undergone hysterectomy, bilateral oophorectomy, or tubal ligation, postmenopausal (>45 years of age with amenorrhea for at least 2 years, or any age with amenorrhea for at least 6 months and a serum follicle stimulating hormone [FSH] level >40 IU/L); surgically sterile: no additional contraception required.

    Women, who are not heterosexually active at screening, must agree to utilize an effective method of contraception if they become hetero-sexually active, as outlined above.

4. All sexually active males, regardless of reproductive potential, must be willing to consistently use an effective method of contraception (such as consistent male condoms with male and/or female partners from the day of IP administration until at least 3 months following IP administration to avoid exposure of partners to IP in ejaculate, and to prevent conception with female partners.

5. All female participants must be willing to undergo urine pregnancy tests at time points indicated in the Schedule of Procedures and must test negative prior to IP administration;

6. A female participant must agree not to donate eggs (ova, oocytes) for the purpose of assisted reproduction until 3 months after receiving IP administration. A man must agree not to donate sperm until 3 months after IP administration;

7. Willing to forgo donations of blood and/or any other tissues, including bone marrow, during the study and, for those HIV-uninfected participants who test HIV-positive due to IP administration, until the anti-HIV antibody titers become undetectable.

Specific inclusion criteria for HIV-uninfected participants (Group 1):

8. At least 18 years of age on the day of screening and has not reached his or her 51st birthday on the day of signing the Informed Consent Document.

9. Willing to undergo HIV testing, risk reduction counselling and receive HIV test results;

10. Low risk for HIV infection (see section (9.4.6) and willing to maintain low-risk behaviour for the duration of the trial (Appendix D);

11. Healthy male or female, as assessed by a medical history, physical exam, and laboratory tests;

Specific inclusion criteria for HIV-infected participants (Groups 2 and 3):

12. At least 18 years of age on the day of screening and has not reached his or her 66th birthday on the day of signing the Informed Consent Document.

13. Confirmed HIV-1 infection (HIV Ab+ or HIV RNA+) by documentation in the medical records or in-clinic HIV testing;

14. CD4 ≥ 300 cells/µl;

15. No history of AIDS-defining illness within the previous 5 years

Group 2:

16. Currently on ART with no change in ART regimen in the 12 weeks before screening or between screening and enrolment, with suppression of plasma HIV-1 viral load < 50 copies / ml for greater than 6 months, and with a viral load < 50 copies / ml at time of screening (within 42 days prior to IP administration). cART is defined as a regimen including > 2 compounds, e.g. 2x nucleoside reverse transcriptase inhibitors plus either non-nucleoside reverse transcriptase inhibitor or protease inhibitor or integrase inhibitor. A change from tenofovir disoproxil fumarate to tenofovir alafenamide in the 12 weeks before screening is not an exclusion.

Group 3:

17. Not receiving cART, and (after appropriate counselling) willing to defer cART treatment for at least 56 days after administration of IP;

18. HIV-1 viral load either between 2000-100,000 copies / ml (Group 3A, 3B, 3C) or between 100-2000 copies / ml (Group 3D, 3E and 3F) confirmed at screening.

5.7 Exclusion Criteria

Exclusion criteria for all participants:

1. Any clinically significant acute or chronic medical condition, other than HIV infection, that is considered progressive or in the opinion of the investigator makes the participant unsuitable for participation in the study;

2. If female, pregnant, lactating or planning a pregnancy during the period of screening through completion of the study;

3. In the past 6 months a history of alcohol or substance use, including marijuana, judged by the Investigator to potentially interfere with participant study compliance;

4. Bleeding disorder that was diagnosed by a physician (e.g., factor deficiency, coagulopathy or platelet disorder that requires special precautions). Note: A participant who states that he or she has easy bruising or bleeding, but does not have a formal diagnosis and has intramuscular injections and blood draws without any adverse experience, is eligible;

5. History of a splenectomy;

6. Receipt of live attenuated vaccine within the previous 60 days or planned receipt within 60 days after administration of IP; or receipt of other vaccine within the previous 14 days or planned receipt within 14 days after infusion or injection with IP (exception is live attenuated influenza vaccine within 14 days);

7. Receipt of blood transfusion or blood-derived products within the previous 3 months;

8. Participation in another clinical trial of an investigational product currently, within the previous 3 months or expected participation during this study;

9. Prior receipt of an investigational HIV vaccine candidate, monoclonal antibody or polyclonal immunoglobulin (note: receipt of placebo in a previous HIV vaccine or monoclonal antibody trial will not exclude a participant from participation if documentation is available and the Medical Monitor gives approval);

10. History of severe local or systemic reactogenicity to injections or IV infusion (e.g., anaphylaxis, respiratory difficulties, angioedema);

11. Psychiatric condition that compromises safety of the participant and precludes compliance with the protocol. Specifically excluded are persons with psychoses within the past 3 years, ongoing risk for suicide, or history of suicide attempt or gesture within the past 3 years;

12. If, in the opinion of the Principal Investigator, it is not in the best interest of the participant to participate in the trial;

13. Seizure disorder: a participant who has had a seizure in the last 3 years is excluded. (Not excluded: a participant with a history of seizures who has neither required medications nor had a seizure for 3 years.)

14. Body mass index ≥ 35 or ≤ 18.0.

15. Infectious disease: chronic hepatitis B infection (HbsAg), current hepatitis C infection (HCV Ab positive and HCV RNA positive) or interferon-alfa treatment for chronic hepatitis C infection in the past year, or active syphilis. Hepatitis B infection that is suppressed on antiretroviral therapy with undetectable HBV DNA is allowable.

16. A history of malignancy within the past 5 years (prior to screening) or ongoing malignancy;

17. Active, serious infections (other than HIV-1 infection) requiring parenteral antibiotic, antiviral or antifungal therapy within 30 days prior to enrollment;

Specific exclusion criteria for HIV-uninfected participants (Group 1):

18. Confirmed HIV-1 or HIV-2 infection;

19. Any clinically relevant abnormality on history or examination including history of immunodeficiency or autoimmune disease; use of systemic corticosteroids, immunosuppressive, anticancer, or other medications considered significant by the investigator within the previous 6 months.

    The following exceptions are permitted and will not exclude study participation: use of corticosteroid nasal spray for rhinitis, topical corticosteroids for an acute uncomplicated dermatitis; or a short course (duration of 10 days or less, or a single injection) of corticosteroid for a non-chronic condition (based on investigator clinical judgment) at least 6 weeks prior to enrollment in this study.

Specific exclusion criteria for HIV-uninfected participants (Group 1) and HIV-infected participants who are on ART (Group 2):

20. Any of the following abnormal laboratory parameters listed below:

    Hematology

    – Hemoglobin <10.5 g/dL in females; hemoglobin <11.0 g/dL in males
    – Absolute Neutrophil Count (ANC): ≤1000/mm^3
    – Absolute Lymphocyte Count (ALC): < 650/mm^3
    – Platelets: < 125,000 mm^3 or ≥ 550,000/mm^3

    Coagulation

    - aPTT: >1.25x ULN
    - INR: ≥1.1 x ULN

    Chemistry

    - Sodium ≤135 mEq/L or ≥ 146 mEq/L
    - Potassium ≤3.4 mEq/L or ≥ 5.6 mEq/L
    - Creatinine ≥1.1 x ULN
    - AST ≥1.25 x ULN

- ALT ≥1.25 x ULN
- Total bilirubin ≥1.25 x ULN
- Alkaline phosphatase ≥1.25 x ULN
- Albumin ≤ 3.0 g/dL or ≤ 30 g/L
- Creatine kinase ≥3.0 x ULN
- C-reactive protein >10 mg/L
- C3 complement < 0.82 g/L
- C4 complement < 0.14 g/L

Urinalysis

Any of the following abnormal findings if consistent with clinically significant disease:
– Protein = greater than trace on dipstick confirmed by microscopic urinalysis outside institutional range
– Blood = greater than trace on dipstick confirmed by ≥ 3 RBCs/hpf on microscopic urinalysis (not due to menses)

Specific exclusion criteria for HIV-infected participants who are on ART (Group 2) and for HIV-infected participants who are not on ART (Group 3):

21. Any clinically relevant abnormality on history or examination including history of immunodeficiency or autoimmune disease, other than HIV; use of systemic corticosteroids, immunosuppressive, anticancer, or other medications considered significant by the investigator within the previous 6 months.

    The following exceptions are permitted and will not exclude study participation: use of corticosteroid nasal spray for rhinitis, topical corticosteroids for an acute uncomplicated dermatitis; or a short course (duration of 10 days or less, or a single injection) of corticosteroid for a non-chronic condition (based on investigator clinical judgment) at least 6 weeks prior to enrollment in this study.

Specific exclusion criteria for HIV-infected participants who are not on ART (Group 3)

22. Any of the following abnormal laboratory parameters listed below:

Hematology

– Hemoglobin < 10.0 g/dL
– Absolute Neutrophil Count (ANC): <800 cells/mm3
– Platelets: < 100,000 cells/mm3

Coagulation

- aPTT: >1.25x ULN
- INR: ≥1.1 x ULN

Chemistry

- Estimated Glomerular filtration rate (GFR) $\leq$ 80 mL/min according to the Cockcroft-Gault formula for creatinine clearance:
    - Male: $\frac{(140 - \text{age in years}) \times (\text{wt in kg}) = \text{CLcr (mL/min)}}{72 \times (\text{serum creatinine in mg/dL})}$
    - Female: $\frac{(140 - \text{age in years}) \times (\text{wt in kg}) \times 0.85 = \text{CLcr (mL/min)}}{72 \times (\text{serum creatinine in mg/dL})}$
- AST ≥ 2.5 x ULN
- ALT ≥ 2.5 x ULN
- Total bilirubin ≥ 1.6 x ULN
- Alkaline phosphatase ≥ 5 x ULN

Urinalysis

Any of the following abnormal findings if consistent with clinically significant disease:
– Protein = greater than 1+ on dipstick confirmed by microscopic urinalysis outside institutional range
– Blood = greater than 1+ on dipstick confirmed by $\geq$ 10 RBCs/hpf on microscopic urinalysis (not due to menses)
- Leukocytes = greater than 1+ on dipstick confirmed by > 10 WBCs/hpf on microscopic urinalysis

5.8 Recruitment of Participants

Adult male and female participants may be recruited through in-clinic referrals, information presented to community organizations, hospitals, colleges, other institutions and/or advertisements to the general public or from existing cohorts. The information distributed will contain contact details of the trial site.

6.0 STUDY VISITS

6.1 Screening Period

During Screening, study staff will perform the following procedures:

- Provide and/or review the Informed Consent Document and answer any questions about the study prior to obtaining written informed consent.
- Complete Assessment of Informed Consent Understanding (AOU). Please refer to the Study Operations Manual (SOM)

If the participant agrees to participate, passes the AOU and provides written informed consent, study staff will:

- Conduct HIV test counselling, HIV testing, and HIV risk reduction counselling, as applicable
- Conduct family planning counselling, refer for pregnancy prevention counselling if necessary
- Administer HIV risk assessment (Group 1)

- Conduct ART counselling (Group 3)
- Perform a comprehensive medical history
- Collect concomitant medication information
- Perform a general physical examination (Refer to Section 7.2)
- Collect specimens for all tests as indicated in the Schedule of Procedures in Appendices A, B and C (for details see Analytical Plan (AP)).

When available, the screening laboratory tests will be reviewed by the trial physician. Screening laboratory test(s) may be repeated once at the discretion of the principal investigator or designee to investigate any isolated abnormalities.

If the screening visit occurs outside the allowable screening window, all screening procedures must be repeated except the comprehensive medical history may be replaced by an interim medical history and the Participant Information Sheet of the Informed Consent Document should be reviewed.

If a participant has signed the Consent Form but does not meet the eligibility criteria, the records must be kept at the site.

6.2 IV infusion or SC injection of PGT121 mAb Visit

Prior to the administration of IP, study staff will:

- Answer any questions the participant may have about the study
- Review the Informed Consent Document with the participant
- Review screening safety laboratory data
- Conduct HIV test counselling, and HIV risk reduction counselling, as applicable
- Conduct ART counselling (Group 3)
- Conduct family planning counselling as per site specific procedures and ensure compliance with respective pregnancy prevention method, and discuss male condom use with all male participants
- Review interim medical history
- Collect concomitant medication information
- Weigh participant and record vital signs
- Perform a symptom-directed physical examination (Refer to Section 7.2)
- Assess at baseline local and systemic signs and symptoms (this includes an examination of IV infusion or SC injection site)
- Collect specimens for all tests as indicated in the Schedule of Procedures see Appendices A, B and C (for details see AP).
- Obtain pregnancy test results prior to administration of IP.

Assign an allocation number to the participant according to the instructions specified in the Study Operations Manual.

At the time of administration of IP and after IV infusion or SC injection of IP, study staff will:

- Administer the IP as specified in Section 8.4, Administration of Investigational Product and according to the instructions specified in the SOM.

- Observe participant closely during the infusion or injection of IP and for at least 30 minutes after IV infusion or SC injection of IP has ended for any acute reactogenicity. At the end of the observation period study staff will:
  - Record vital signs (pulse, respiratory rate, blood pressure and temperature)
  - Assess any local and systemic reactogenicity
  - Assess any other adverse events
- Every hour after IV infusion or SC injection of IP, for at least 6 hours, the study staff will:
  - Record vital signs (pulse, respiratory rate, blood pressure and temperature)
  - Assess any local and systemic reactogenicity
  - Assess any other adverse events
  - Collect PK samples according to the Schedule of Procedures

If a participant has an abnormal laboratory value that is known, at the time of infusion or injection, follow the specified guidelines (Section 12.0).

6.3 Post-IV infusion or SC injection of PGT121 mAb Visits

The participant will be asked to return to the clinic for post-IP administration visits as indicated in the Schedule of Procedures (see Appendices A, B and C) for an assessment by clinic staff. The participant will be asked to maintain a Memory Aid to track any local and systemic reactogenicity the participant experiences, including temperature, from the day of IP administration for the next 3 days (for a total of 4 days including day of IP administration). Study staff will review the Memory Aid with the participant and determine the severity of the reactions through discussion with the participant.

The following procedures will be conducted at these visits:

- Review interim medical history
- Collect concomitant medication information
- Perform a symptom-directed physical examination if any signs or symptoms are present
- Assess vital signs (pulse, respiratory rate, blood pressure and temperature)
- Assess any adverse events and local and systemic reactogenicity (Days 1, 2, 3) including reviewing the Memory Aid.
- Collect specimens for all tests as indicated in the Schedule of Procedures (Appendices A, B and C and AP).

6.4 Additional Follow-up Visits

Assessments and procedures will be performed according to the Schedule of Procedures (Appendices A, B and C).

6.5 Unscheduled Visits

Unscheduled Visits/Contacts are visits/contacts that are not described in the Schedule of Procedures (Appendices A, B and C). Unscheduled visits may occur any time during the study:

- For administrative reasons, e.g., the participant may have questions for study staff or may need to re-schedule a follow-up visit.

- To obtain laboratory test results from a previous visit.
- For other reasons as requested by the participant or site investigator.

All unscheduled visits will be documented in the participants' study records on applicable source documents and entered into the Case Report Form (CRF).

6.6 Final Study Visit or Early Termination Visit

Assessments and procedures will be performed according to the Schedule of Procedures (Appendices A, B and C).

7.0 STUDY PROCEDURES

7.1 Informed Consent Process

A Master Informed Consent Document consisting of a Participant Information Sheet and a Consent Form is provided by the Sponsor to the trial site. This document is made site-specific and translated (if necessary), submitted and approved by the Institutional Review Board (IRB). The Master and site specific Informed Consent Documents are separate documents and should not be part of the protocol.

<u>Participant Information Sheet</u>

A qualified member of the study staff will conduct the informed consent process by reviewing the Participant Information Sheet and document it in the clinic notes.

<u>Consent Form</u>

The participant's consent to participate must be obtained by him/her signing and dating the Consent Form. The person obtaining consent will also sign.

The signed and dated Informed Consent Document must remain at the study site. A copy of the signed/marked and dated Informed Consent Document will be offered to the participant to take home. Those participants who do not wish to take a copy will be required to document that they declined to do so.

7.2 Medical History and Physical Examination

Medical History

At screening, a comprehensive medical history will be collected including previous IV infusions and SC injections, and reaction to IV infusion or SC injection, history of sexually transmitted infection (STI) and pregnancy prevention practices. At subsequent visits, an interim medical history will be performed.

Physical Examination

<u>General Physical Examination</u>

A general physical examination includes examination of head/ears/eyes/nose and throat, skin, respiratory, cardiovascular, abdominal, limited neurological and musculoskeletal and external ano-genital systems (for HIV-infected participants only) at the time points indicated in the Schedule of Procedures (see Appendices A, B and C).

<u>Symptom-Directed Physical Examination</u>
A symptom-directed physical examination is a targeted examination based on the participant's history or observation. If deemed necessary, this examination should be done at the time points indicated in the schedule of procedures (see Appendices A, B and C).

<u>Measuring Height and Weight</u>
Includes measuring the height and weight at the time points indicated in the Schedule of Procedures (see Appendices A, B and C).

<u>Vital Signs</u>
Vital signs including pulse, respiratory rate, blood pressure and temperature are measured and recorded at the time points indicated in the Schedule of Procedures (see Appendices A, B and C)

7.3 HIV Testing and HIV-test Counselling (Group 1)

Study staff will perform pre-HIV test counselling prior to collecting blood for an HIV test, and post-HIV test counselling when HIV test results are available. This is referred to as HIV-test counselling, and done according to the CDC guidelines. For more information on HIV testing and HIV-test counselling, see Section 11.0. A screening questionnaire and other tools may be used.

7.4 HIV Risk Reduction Counselling

HIV risk reduction counselling will be provided to all participants as outlined by site-specific SOPs.

Study staff will provide HIV risk reduction counselling based on reported individual risk and provide free condoms, as appropriate, at every visit. Group 1 will receive HIV risk reduction counselling and for Groups 2 and 3, HIV risk reduction counselling will be conducted as secondary prevention to reduce onward transmission.

7.5 Family Planning Counselling

Study staff will counsel participants about the importance of preventing pregnancies and of using condoms, as well as other effective family planning methods until at least 3 months following investigational product administrations, as appropriate. Participants may be referred for family planning services as necessary according to site-specific SOPs as detailed in the SOM. Pregnancy prevention methods chosen and compliance will be documented.

7.6 ART Counselling (Group 3)

HIV-infected participants who are not on ART will receive ART counselling upon entering the study and 8 weeks after administration of IP. Participants who have not initiated or made plans to initiate ART by the final study visit will receive ART counselling again at their final study visit. HIV-infected participants who are on ART (Group 2) will be counselled on the importance of continuing ART throughout the study, and will not be required to interrupt ART after administration of IP.

7.7 Specimens

Approximately 50 ml of blood will be collected from participants in Group 1, approximately 78 ml from participants in Group 2, and approximately 150 ml of blood will be collected from participants in Group 3 at the screening visit. At later visits, approximately 8.5 ml to 175 ml of blood will be collected, depending on study procedures and group assignment (see Appendices A, B and C), usually from the antecubital fossa.

Optional collection of rectal and/or cervical mucosal secretions will be obtained using a rectal sponge (or comparable swab) or cervical Softcup (or comparable cervical fluid collection cup) for those participants that consent.

All specimens will be handled according to the procedures specified in the AP or SOPs, if applicable.

In the event of an abnormal laboratory value, participants may be asked to have an additional sample collected at the discretion of the Principal Investigator or designee.

7.8 Reimbursement

Participants will be reimbursed for their time, effort and for costs to cover their travel expenses to the study site and any inconvenience caused due to study participation. Site specific-reimbursement amounts will be documented in the site-specific Participant Information Sheet, and approved by the Institutional Review Board.

7.9 Randomization and Blinding

Participants will be identified by a unique study identification number.

Participants will be randomized according to the randomization schedule prepared by the statisticians at the Data Coordinating Center (DCC) prior to the start of the study. Participants will be automatically assigned a specific allocation number as they are enrolled into the data entry system. An unblinding list (Pharmacy List) will be provided to the unblinded site pharmacist by the DCC.

This is a randomized, double-blind placebo-controlled study for groups 1 and 2, and an open label study for group 3. For Groups 1 and 2, study staff (investigator and clinical personnel monitoring the safety and laboratory assay results) and participants will be blinded with respect to the allocation of Investigational Product (PGT121 mAb or placebo). A site pharmacist will be unblinded for the purposes of preparing study product.

A participant will be considered enrolled once he/she has been assigned an allocation number.

Blinded participants will be informed about their assignment (product/placebo) at study completion, once the database is locked. Should a study participant be unblinded during the study, the study participant will be followed up until the end of the study according to the Schedule of Procedures (see Appendices A and B).

7.10 Un-blinding Procedure for Individual Participants

Un-blinding of an individual participant may be indicated in the event of a medical emergency if the clinical management of the participant would be altered by knowledge of the treatment assignment.

The un-blinded information should be restricted to a small group of individuals involved in clinical management/medical treatment of the participant (e.g. treating physician) and the blind must be maintained for those responsible for the study assessments.

The reasons for un-blinding should be documented and the IAVI Chief Medical Officer, the Medical Monitor and the DCC should be notified as soon as possible. The procedures and contact numbers for un-blinding are outlined in the SOM.

7.11 Assessment of IP related HIV sero-positivity

It is possible that PGT121 mAb or an immune response to PGT121 mAb could cause a positive result on a diagnostic HIV antibody test. A Group 1 participant who tests HIV antibody positive at the end of the study will have additional testing to distinguish actual HIV infection from IP-related responses. The participant will be informed of his/her positive HIV antibody test result and offered continuing follow-up until the HIV antibody test becomes negative.

8.0 INVESTIGATIONAL PRODUCT

8.1 Description

A summary of the Investigational Products is shown in Table 8.1-1.

Table 8.1-1 Investigational Products

IP (Active Product / Placebo)	Dosage level	Total volume in IP container	Total IP (Active Product or placebo) volume to be injected into a 100 mL saline IV bag, or injected SC‡ (for an 88 kg body weight**)	Total volume to be Infused IV (for an 88 kg body weight**)
PGT121 (50 mg/mL)	3 mg/kg	6 mL per vial	5.3 mL	105.3 mL
	10 mg/kg		17.6 mL	117.6 mL
	30 mg/kg		52.8 mL	152.8 mL
Placebo: 0.9% Sodium Chloride Injection USP (Saline)*	3 mg/kg matching***	NA	5.3 mL***	105.3 mL***
	10 mg/kg matching***		17.6 mL***	117.6 mL***
	30 mg/kg matching***		52.8 mL***	152.8 mL***

* The Placebo provided will be a commercially-available saline partial addition IV bag.

** The actual volume to be injected will be based on the dose group and the weight of the participant at the time of IP administration. The example included here is the average weight of an adult male in the US (88kg) (http://www.cdc.gov/nchs/data/series/sr_11/sr11_252.pdf)

*** For placebo IV infusions: saline from an additional IV bag will be injected into the saline IV bag intended for administration, to match the volume used for a PGT121 mAb injection in the same dose group, to prevent unblinding.

‡ Only 3mg/kg dose will be injected SC, because of volume limitations for SC injections

8.2 Shipment and Storage

Authorization to ship the Investigational Product to the site will be provided in writing by the Sponsor, upon confirmation that all required critical documents for shipment authorization are completed. The Investigational Product will be shipped maintaining the required storage conditions and stored in a secure location in the clinical site's pharmacy.

The Investigational Product is formulated in a 20 mM Acetate, 9% Sucrose, 0.008% polysorbate 80, pH 5.2 formulation buffer at a concentration of 50 mg/mL. Each 10 ml vial will contain 6 ml of IP stored at <- 20°C. Each vial will be labelled with the name of the product, Lot number, concentration, storage temperature, date of manufacturing, contact information of the Sponsor and a US cautionary statement. Several such vials will be packaged in a box. Each box will also be labelled with similar information as the vial label.

8.3 Preparation of Investigational Product (IP)

Detailed instruction will be provided to the site pharmacist in the SOM for preparing each of the investigational products. The site pharmacist will not be blinded, but the study physician/designee administering the IP will be blinded. Product should be administered within 4 hours of preparation. Example calculations for final volume for IV infusion or SC injection are illustrated in Table 8.1-1. Procedures for handling used and partially used vials will be provided in the SOM.
Syringes or other components in direct contact with investigational products will be disposed of in a biohazard container and incinerated or autoclaved.

8.4 Administration of Investigational Product

Investigational Product will be administered at the enrollment visit.

For IV infusion, the IP will be injected into a 0.9% Saline bag. The participant will receive the IP via IV or SC infusion. Participants will receive IV infusion over approximately 60 minutes or SC injection, allowing for clinician discretion. Further information on the IV infusion or SC injection of the IP is supplied in the SOM and study documents.

8.5 Accountability and Disposal of Investigational Product

All used IP vials will be handled according to instructions in the SOM. The date, allocation number and location of storage of the returned vials will be recorded.

During the study, the IP accountability forms including receipt and dispensing of vials will be kept and monitored.

At the end of the study, the used and unused IP vials will be handled according to instructions of Sponsor.

Further information on accountability and disposal of IP is supplied in the SOM.

9.0 ASSESSMENTS

9.1 Safety Assessments

Data on local and systemic reactogenicity (i.e., solicited AEs) will be collected by structured interview and medical examination. Data on other adverse events will be collected with open-ended questions. All data will be recorded on the appropriate source documents and entered into the study database. Participants will be given a Memory Aid, which is a tool to assist with collecting reactogenicity data.

Local and systemic reactogenicity events will be assessed by study staff prior to IV infusion or SC injection of IP, at approximately 30 minutes after IP administration start, at 1 hour after IP administration, and subsequently every hour for at least the first 6 hours post-IP administration. Study staff will review the Memory Aid with the participant, and determine the severity of the reactions on days 1-3 through discussion with the participant.

9.1.1 Local reactogenicity

The presence of local reactogenicity will be assessed at the time points specified in the Schedule of Procedures (Appendices A, B and C).
Pain, tenderness, erythema/skin discoloration, swelling/hardening or pruritus will be assessed and graded using Appendix E, Adverse Event Severity Assessment Table, as a guideline.

9.1.2 Systemic reactogenicity

The presence of systemic reactogenicity will be assessed at the time points specified in the Schedule of Procedures (Appendices A, B and C).
Fever, chills, headache, nausea, vomiting, malaise, myalgia and arthralgia will be assessed and graded using the Appendix E, Adverse Event Severity Assessment Table as a guideline. For the first 24 hours after IP infusion or injection, any infusion related reactions, including cytokine release syndrome, should be graded using the Common Terminology Criteria for Adverse Events (CTCAE) v4.03: June 14, 2010 (Appendix F).

9.1.3 Vital Signs

At the administration of IP visit, vital signs (pulse, respiratory rate, blood pressure and temperature) will be measured by study staff prior to IP administration, at approximately 30 minutes post IP administration start and hourly for at least 6 hours after IV infusion or SC injection. For the other study visits vital signs will be assessed at the time points specified in the Schedule of Procedures (Appendices A, B and C).

9.1.4 Other Adverse Events

Other adverse events (AEs) will be collected through 56 days after IP administration in all participants. Serious Adverse Events (SAEs) will be collected throughout the entire study period. Potential Immune Mediated Diseases (pIMDs), as defined in Section 10.5, will be collected throughout the study period, using the SAE reporting process. Open-ended questions will be asked at time points according to the Schedule of Procedures (Appendices A, B and C). All adverse events will be graded using Appendix E, Adverse Event Severity Assessment Table, as a guideline and will be assessed for causality to the IP. For more information regarding adverse events refer to Section 10.0, Adverse Events.

9.1.5 Concomitant Medications

Concomitant receipt of Investigational Products is prohibited during the study.

Contraceptive use and use of medication at study entry will be documented. (See DCF instructions)

During the study, information regarding concomitant medications and reasons for their use will be solicited from the study participants for 56 days. Ongoing concomitant medications will be recorded until end of study.

9.1.6 Routine laboratory parameters

Table 9.1.6-1 shows the laboratory parameters that will be measured routinely. The samples for these tests will be collected at the time points indicated in the Schedule of Procedures (Appendices A, B and C).

Table 9.1.6-1: Laboratory Parameters

Laboratory Parameter	Test
Hematology and Coagulation	Hemoglobin, hematocrit, leukocytes, platelets, absolute neutrophil count (ANC), absolute lymphocyte count (ALC), activate partial thromboplastin time (aPTT), international normalized ratio (INR)
Clinical Chemistry	Sodium, potassium, creatinine, aspartate aminotransferase (AST), alanine aminotransferase (ALT), total bilirubin, alkaline phosphatase<br>Groups 1 and 2 only: albumin, creatine kinase, C-reactive protein, C3 complement, C4 complement
Urinalysis	Dipstick test for protein, blood, glucose, ketones, esterase (leukocytes) and nitrite. If clinically significant abnormalities (e.g., blood, protein, leukocytes) are found on dipstick test, then further test(s) will be performed (e.g., microscopy, culture)
T cell panel (Groups 2 and 3)	CD4 T cell count and frequency by single platform flow cytometry

9.1.7 Specific screening tests:

Participants will be screened to exclude the following diseases:

- Hepatitis B: positive for hepatitis B surface antigen (HBsAg) or detectable hepatitis B DNA if on antiretroviral therapy
- Hepatitis C: positive for hepatitis C RNA (HCV antibody test, followed by HCV RNA test if HCV antibody positive)
  Active syphilis: confirmed diagnosis.

A negative Hepatitis B and Hepatitis C result can be documented from the medical record only if the result is from a test administered less than 6 months ago.

9.1.8 Monitoring for anti-PGT121 antibodies:

Participants will be evaluated for the development of antibodies to PGT121 mAb (anti-drug antibodies, ADA) by ELISA according to the Schedule of Procedures (Appendices A, B and C).

9.2 Virologic Assessments

Table 9.2-1 shows the virologic parameters that will be measured routinely. The samples for these tests will be collected at the time points indicated in the Schedule of Procedures (Appendices B and C).

Table 9.2-1: Virologic Assessment Table

Virologic Parameter	Test
Antiviral Activity	Plasma HIV RNA levels
Anti-reservoir activity	Cell-associated HIV-1 RNA levels in resting CD4 T cells; total HIV-1 DNA and 2-long terminal repeat (LTR) HIV-1 DNA circles in resting or total CD4 T cells; quantitative viral outgrowth assay (qVOA)
Other	Genotyping of plasma HIV RNA for evaluation of PGT121-induced escape mutations; phenotyping of plasma HIV RNA for neutralization susceptibility to PGT121 in-vitro

9.3 Exploratory Immunogenicity Assessments

Humoral immune response assays will include, but are not limited to Env-specific Ab-binding assays, virus neutralization assay, and assays for Ab functionality. Cellular immune response assays will include, but are not limited to IFNγ ELISPOT assay, ICS, and multiparameter flow cytometry. Exploratory assessments on mucosal samples will include, but are not limited to characterization of Env-specific binding Abs. Priority assays are listed below.

9.3.1 Antibody Responses

- Env-specific binding Abs (titers and breadth).
- Env-specific nAbs (titers and breadth).

- Env-specific functional Abs (phagocytosis score and breadth).
- Env-specific binding Ab isotypes (IgA, IgG1-4) (titers and breadth).

9.3.2 Cellular Responses

- IFNγ peripheral blood mononuclear cell (PBMC) responders to peptide pools and subpools of Potential T-cell epitopes, PTE Env/Gag/Pol peptides.
- $CD4^+$ and $CD8^+$ T-cell functionality (% cells producing e.g. IFNγ, IL-2, IL-4, TNFα).
- T-cell development with emphasis on follicular helper T-cells and memory differentiation.

9.3.3 PBMC, Serum and Plasma Storage

Samples of cryopreserved PBMC, plasma and serum will be stored as indicated in the Schedule of Procedures (Appendix A, B and C) and the Analytical Plan (AP) and, if the participant consents, may be used for the purposes of standardization, quality control and for future assays related to HIV prevention or treatment research and development. These samples will be archived and the testing laboratories will be blinded to the participant's identity.

9.4 Other Assessments

9.4.1 HIV Antibody Testing (Group 1)

All HIV-uninfected participants (Group 1) will be tested for HIV antibodies as indicated in the Schedule of Procedures (Appendix A) or as needed, if medical or social circumstances arise. All participants will receive HIV risk reduction counselling and pre- and post-HIV-test counselling, as specified in Section 7.3 Counselling.

9.4.2 Pharmacokinetics

Blood draws for pharmacokinetics will be done on the day of IP administration immediately before IV infusion or SC injection of IP, at the end of the IP administration, and 30 minutes after the end of IP administration, and 3 hours and 6 hours after the IP administration. An additional draw will be done at 24 hours after IP administration. Thereafter, pharmacokinetic draws will be done as indicated in the Schedule of Procedures (Appendices A, B and C). PGT121 mAb serum or plasma levels will be determined using two methods: a sandwich ELISA using a murine anti-idiotype antibody to PGT121 mAb, and a neutralization assay.

PGT121 mAb pharmacokinetic analysis will be performed using standard non-compartmental analysis methods to estimate elimination half-life (t1/2), clearance (CL/F), volume of distribution (Vz/F), Area under the concentration decay curve (AUC), impact of viral load and/or ART on PGT121 mAb disposition (elimination half-life (t1/2), clearance (CL/F), volume of distribution (Vz/F) and total exposure. PGT 121 accumulation will also be examined in rectal and cervical mucosal secretions collected with rectal sponges or cervical softcups in study participants who specifically consented for these procedures. Descriptive results will be reported for the pharmacokinetic parameters by dose subgroup.

Exploratory analysis using population analysis methods simultaneously combining all pharmacokinetic data across all doses and treatment groups will be performed for quantitative characterization of differences in PGT121 mAb disposition by dose, participant group or disease state.

9.4.3 HLA Typing

Samples for HLA typing will be collected as specified in the Schedule of Procedures (Appendix A, B and C) and AP and may be analyzed as warranted.

9.4.5 Pregnancy Test

A urine pregnancy test for all female participants will be performed by measurement of human chorionic gonadotrophin (βhCG) at time points indicated in the Schedule of Procedures (Appendices A, B and C). The results of the pregnancy test must be negative prior to IV infusion of PGT121 mAb. See section 10.7 for description of pregnancy after administration of IP.

9.4.6 HIV Risk Assessment (Group 1)

Study staff will assess participants for their past and current risk of acquiring HIV at time points indicated in Schedule of Procedures (Appendix A).

9.4.7 Social Impact Assessment

A brief assessment of the impact of participation in the study will be administered to participants at their final study visit.

10.0 ADVERSE EVENTS

10.1 Definition

An adverse event (AE) is any untoward medical occurrence in a participant administered an Investigational Product and which does not necessarily have a causal relationship with the Investigational Product. An AE can therefore be any unfavourable or unintended sign (including an abnormal laboratory finding), symptom, or disease, temporally associated with the use of Investigational Product whether or not related to the Investigational Product.

Assessment of severity of all AEs, including and seriousness of AEs, is ultimately the responsibility of the Principal Investigator of each site. Refer to the DIVISION OF AIDS (DAIDS) TABLE FOR GRADING THE SEVERITY OF ADULT AND PEDIATRIC ADVERSE EVENTS Version 2.0, November 2014 and the National Cancer Institute Common Terminology Criteria for Adverse Events (CTCAE) Version 4.03: June 14, 2010 for additional guidance.

10.2 Assessment of Severity of Adverse Events

The following general criteria should be used in assessing adverse events as mild, moderate, severe or very severe at the time of evaluation:

<u>Grade 1 (Mild):</u> Symptoms causing no or minimal interference with usual social & functional activities

Grade 2 (Moderate): Symptoms causing greater than minimal interference with usual social & functional activities

Grade 3 (Severe): Symptoms causing inability to perform usual social & functional activities

Grade 4 (Very Severe): Symptoms causing inability to perform basic self-care functions OR Medical or operative intervention indicated to prevent permanent impairment, persistent disability, or death

Guidelines for assessing the severity of specific adverse events and laboratory abnormalities are listed in Appendix E, Adverse Event Severity Assessment Table.

10.3 Relationship to Investigational Product

Assessment of relationship of an AE or SAE to Investigational Product (IP) is the responsibility of the Principal Investigator or designee. All medically indicated and available diagnostic methods (e.g., laboratory, blood smear, culture, X-ray, etc.) should be used to assess the nature and cause of the AE/SAE. Best clinical and scientific judgment should be used to assess relationship of AE/SAEs to the IP and/or other cause.

The following should be considered:

- Presence/absence of a clear temporal (time) sequence between administration of the IP and the onset of AE/SAE
- Presence/absence of another cause that could more likely explain the AE/SAE (concurrent disease, concomitant medication, environmental or toxic factors)
- Whether or not the AE/SAE follows a known response pattern associated with the IP

The relationship assessment should be reported as one of the following:

Not Related: clearly explained by another cause (concurrent disease, concomitant medication, environmental or toxic factors, etc.).

Unlikely: more likely explained by another cause (concurrent disease, concomitant medication, environmental or toxic factors, etc.).

Possibly: equally likely explained by another cause but the possibility of the IP relationship cannot be ruled out (e.g., reasonably well temporally related and/or follows a known IP response pattern but equally well explained by another cause).

Probably: more likely explained by the IP (e.g., reasonably well temporally related and/or follows a known IP response pattern and less likely explained by another cause).

Definitely: clearly related and most likely explained by the IP.

For the purpose of expedited safety reporting, all possibly, probably or definitely related SAEs are considered IP-related SAEs.

10.4 Serious Adverse Events

An adverse event is reported as a "Serious Adverse Event" if it meets any of the following criteria (as per International Conference on Harmonisation [ICH] Good Clinical Practice [GCP] Guidelines):

- Results in death
- Is life threatening
- Results in persistent or significant disability/incapacity
- Requires in-participant hospitalization or prolongs existing hospitalization
- Is a congenital anomaly/birth defect or spontaneous abortion
- Any other important medical condition that requires medical or surgical intervention to prevent permanent impairment of a body function or structure

Elective surgery for pre-existing condition that did not increase in severity or frequency is not considered an SAE.

Serious Adverse Events (SAEs) should be reported within 24 hours of the site becoming aware of the event, and sent to the Sponsor as described in the SOM.

To discuss IP-related SAEs or any urgent medical questions related to the SAE, the site investigator should contact one of the IAVI Medical Monitors directly (see Contact List in the SOM).

The IAVI SAE Report Form should be completed with all the available information at the time of reporting and sent to the Sponsor as described in the SOM. The minimum data required in reporting an SAE are the study identification number, date of birth, gender, event description (in as much detail as is known at the time), onset date of event (if available), reason event is classified as serious, reporting source (name of Principal Investigator or designee), and relationship to the IP as assessed by the investigator.

The Principal Investigator or designee is required to prepare a detailed written report with follow up until resolution or until it is judged by the Principal Investigator or designee to have stabilized.

The Principal Investigator or designee must notify the local IRB/IEC of all SAEs as appropriate. In case of IP-related SAEs, the Sponsor will notify responsible regulatory authorities, Safety Monitoring Committee (SMC), and other study sites where the same IP is being tested.

More details on SAE definitions and reporting requirements are provided in the SOM.

<u>Serious Event Prior to Investigational Product Administration</u>

If a serious event occurs in the period between the participant signing the Informed Consent Form and receiving the IV infusion or SC injection of IP, the event will be reported using the SAE form and following the same procedures for SAE reporting, as indicated in Section 10.4. The timing of the event will be indicated by using the relevant checkbox on the SAE form.

10.5 Reporting Potential Immune-Mediated Diseases

Potential immune-mediated diseases (pIMDs) are a subset of AEs that include both clearly autoimmune diseases and also other inflammatory and/or neurologic disorders that may or may not have an autoimmune etiology. These events are of special interest since they could potentially be caused by immune responses to the IP. The investigator/designee should report such adverse events within the same time limits (following confirmation of an AE as a pIMD; see last paragraph of this section below), and using the same CRF pages, as utilized for SAEs. The investigator or his/her designee will evaluate the occurrence of pIMDs at every visit/contact during the study. IAVI will also expect investigators/designee to provide additional information about pIMD events. AEs to be reported and documented as pIMDs include:

Neuroinflammatory disorders: optic neuritis, cranial nerve disorders (including Bell's palsy), multiple sclerosis, demyelinating disease, transverse myelitis, Guillain-Barré syndrome, myasthenia gravis, encephalitis, neuritis.

Musculoskeletal disorders: systemic lupus erythematosus, cutaneous lupus, Sjögren's syndrome, scleroderma, dermatomyositis, polymyositis, myopathy, rheumatoid arthritis and juvenile rheumatoid arthritis, polymyalgia rheumatica or temporal arteritis, reactive arthritis, psoriatic arthropathy, ankylosing spondylitis, undifferentiated spondyloarthropathy.

Gastrointestinal disorders: Crohn's disease, ulcerative colitis or proctitis, celiac disease.

Metabolic diseases: autoimmune thyroiditis, Grave's or Basedow's disease, Hashimoto thyroiditis, insulin-dependent diabetes mellitus (IDDM), Addison's disease.

Skin disorders: psoriasis, vitiligo, Raynaud's phenomenon, erythema nodosum, autoimmune bullous skin diseases.

Others: autoimmune hemolytic anemia, thrombocytopenia, antiphospholipid syndrome, *vasculitis, pernicious anemia, autoimmune hepatitis, primary biliary cirrhosis, primary sclerosing cholangitis, autoimmune glomerulonephritis, autoimmune uveitis, autoimmune myocarditis/cardiomyopathy, sarcoidosis, Stevens-Johnson syndrome, Behçet's syndrome.

Infusion site reactions: Grade 3 or 4 infusion site reactions lasting more than 2 days.

*Vasculitis: Vasculitis, Diffuse vasculitis, leucocytoclastic vasculitis, polyarteritis nodosa, microscopic polyangiitis, Wegener's granulomatosis, anti-neutrophil cytoplasmic antibody positive vasculitis, Henoch-schönlein purpura, allergic granulomatous angiitis (Churg-Strauss disease), Kawasaki disease, Takayasu's arteritis, temporal arteritis (giant cell arteritis), renal vasculitis.

Medical judgement should be exercised in deciding whether other disorders/diseases have an autoimmune origin and should also be reported as described above, and this judgement is the investigator's prerogative. Whenever sufficient data exist to substantiate any of the diagnoses in the above list, the event must be reported as a pIMD. While the intent of pIMD reporting is to be inclusive, isolated nonspecific

symptoms, which might (or might not) represent the above diagnoses, should be captured as AEs but not reported as pIMDs until the diagnosis can be defended.

10.6 Clinical Management of Adverse Events

Adverse events (AEs) will be managed by the clinical study team who will assess, provide first line of care as appropriate and refer to health care and treatment facilities as warranted. If any treatment/medical care is required as a result of the harm caused by the IP or study procedures, this will be provided free of charge.

If a participant has an AE and/or abnormal laboratory value that is known at the time of IV infusion or SC injection of IP, the specifications of Section 12.0 will be followed.

Participants will be followed until the AE resolves or stabilizes or up to the end of the study, whichever comes last. If at the end of the study, an AE (including clinically significant laboratory abnormality) that is considered possibly, probably or definitely related to the IP is unresolved, follow-up will continue until resolution if possible and/or the participant will be referred.

If a participant from group 3 experiences a significant decrease in CD4 cell count (e.g. – 20% of baseline, or decline to <200 cells/µL) during the course of the trial, CD4+ will be monitored closely until their CD4 count returns to baseline or until the participant initiates ART. Participants whose CD4 cell counts decrease to <200 cells/µL will be promptly informed and will be referred to their primary HIV care provider. Appropriate prophylaxis against opportunistic infections will be instituted according to accepted U.S. HIV treatment guidelines.

10.7 Pregnancy

Although not considered an AE, if a female participant becomes pregnant during the study, it is the responsibility of the Principal Investigator or designee to report the pregnancy promptly to IAVI using the designated forms. The participant will be followed for safety until the end of pregnancy or study completion, whichever occurs last. If possible, approximately 2–4 weeks after delivery, the baby will be examined by a physician to assess its health status and the results will be reported to the Sponsor. The baby will be examined again by a Physician around age 1, and the results will be reported to the Sponsor.

Complications of pregnancy that meet criteria for SAEs, specified in Section 10.4 of this Protocol (e.g., hospitalization for eclampsia, spontaneous abortion, etc.) should be reported as SAEs.

10.8 Intercurrent HIV Infection (Group 1)

HIV infection cannot be directly caused by the IP. If a participant acquires HIV through exposure in the community, at any time after the IV infusion or SC injection of IP, the participant should be offered referral to appropriate care and treatment facilities. The participant will continue to be followed in the study for safety assessments.

Intercurrent HIV infection in study participants, although not considered an SAE, must be reported promptly to IAVI using the designated forms. However, medical conditions associated with the HIV infection that meet criteria for being serious specified in the

Section 10.4 of this Protocol (e.g., sepsis, *Pneumocystis jiroveci [carinii] pneumonia*, etc.) should be reported as SAEs using the SAE Report Form.

11.0 MANAGEMENT OF HIV ISSUES DURING AND FOLLOWING STUDY

11.1 HIV Testing – Group 1

Group 1 participants will be tested for HIV antibodies as indicated in the Schedule of Procedures (Appendix A) or as needed, if medical or social circumstances arise. All participants will receive HIV risk reduction counselling and pre- and post-HIV-test counselling, as specified in Section 11.3.1, Counselling (Group 1).

It is possible that PGT121 or an immune response to PGT121 mAb could cause a positive result on a diagnostic HIV antibody test. An IP recipient who falsely tests HIV positive with a diagnostic HIV antibody test at the end of the study will be informed of his/her positive test result and offered continuing follow-up until the test becomes negative.

If a participant acquires HIV through exposure in the community, at any time after the administration of IP, the participant will be offered referral to appropriate care and treatment facilities. The participant will continue to be followed in the study for safety assessments.

Should a participant require HIV testing outside of the study for personal reasons, it is recommended that the participant contact the study staff first. HIV testing can be done at the study site and then processed at an independent laboratory as above. Written evidence of HIV status (HIV-infected or HIV-uninfected) will be provided upon request.

11.2 Social Discrimination as a Result of IP-related antibodies

In order to minimize the possibility of social discrimination in participants (if any) who test positive on a diagnostic HIV antibody test due to IP-related antibodies, appropriate diagnostic HIV testing and certification will be provided both during and after the study as needed.

11.3 HIV infection – Group 1

Group 1 participants who are diagnosed with HIV infection at screening or during the study (intercurrent HIV-infection) will be provided the following:

11.3.1 Counselling

The participant will be counselled by the study investigators or designated counsellors. The counselling process will assist the participant with the following issues:

- Psychological and social implications of HIV infection
- Who to inform and what to say
- Implications for sexual partners
- Implications for child-bearing
- Avoidance of transmission to others in future

- Mandatory reporting to the state, in some instances

11.3.2 Referral for Support/Care

Participants will be referred to a participant support center or institution of his/her choice for a full discussion of the clinical aspects of HIV infection. Referral will be made to a designated physician or center

12.0 WITHDRAWAL FROM STUDY

12.1 Deferral of IV infusion or SC injection of IP

An IV infusion or SC injection of IP may be temporarily deferred if the participant is clinically ill at the time of the administration of IP visit and/or presents with fever (> 100.4 F) at the time of the administration of IP. A participant must be clinically well and afebrile for a minimum of a 24-hour consecutive period prior to administration of IP.

Any planned or unplanned deferral of infusion or injection of IP will be discussed with the Sponsor. Participants will be deferred from infusion or injection of IP for any of the following reasons:

1. Pregnancy
2. A disease or condition or adverse event that may develop, regardless of relationship to Investigational Product, if the Principal Investigator or designee is of the opinion that administration of IP will jeopardize the safety of the participant
3. Participant's request to defer infusion or injection

The following events require resolution and/or review of clinical history by the Principal Investigator or designee and consultation with the Medical Monitor, prior to administration of IP:

- Any abnormal laboratory value, as outlined in section 5.7, Exclusion Criteria, Hematology, Chemistry, Urinalysis that is known at the time of infusion or injection and have not resolved. Abnormal results should be confirmed on the original sample and/or repeated at least once to confirm abnormal values.
- Receipt of inactivated/killed/subunit vaccines (non-HIV) or immunoglobulin within the previous 14 days. Receipt of live attenuated vaccines within the previous 60 days.
- Participating in another clinical study of an Investigational Product

12.2 Withdrawal from the Study (Early Termination)

Participants may be withdrawn from the study permanently for the following reasons:

1. Participants may withdraw from the study at any time if they wish, for any reason
2. The Principal Investigator or designee has reason to believe that the participant is not complying with the protocol
3. If the Sponsor decides to terminate or suspend the study

If a participant withdraws or is withdrawn from the study, all termination visit procedures will be performed according to the Schedule of Procedures (Appendices A, B and C)

where possible. Every effort will be made to determine and document the reason for withdrawal.

13.0 DATA HANDLING

13.1 Data Collection and Record Keeping at the Study Site

Data Collection: All study data will be collected by the clinical study staff using designated source documents and entered onto the appropriate electronic CRFs (eCRFs). Access to eCRFs will be provided via an electronic data entry system hosted by the Data Coordination Center. All study data must be verifiable to the source documentation. A file will be held for each participant at the clinic(s) containing all the source documents. Source documentation will be available for review to ensure that the collected data are consistent with the CRFs.

All CRFs and laboratory reports will be reviewed by the clinical team, who will ensure that they are accurate and complete.

Source documents and other supporting documents will be kept in a secure location. Standard GCP practices will be followed to ensure accurate, reliable and consistent data collection.

Source documents include but are not limited to:

- Signed Informed Consent Documents
- Progress notes
- Data collection forms
- Documentation of any existing conditions or past conditions relevant to eligibility
- Printed laboratory results
- Print out of the eClinical generated enrollment confirmation
- All Adverse Events
- Concomitant medications
- Local and systemic reactogenicity events

13.3 Data Entry at the Study Site

The data collected at the site will be recorded onto the eCRFs by the study staff and entered into a database. To provide for real time assessment of safety, data should be entered as soon as reasonably feasible after a visit occurs.

13.4 Data Analysis

The Sponsor, PIs and Product Developers will agree on how data will be analyzed and presented prior to unblinding of the study.

The DCC will conduct the data analysis and will provide interim safety and final study reports for the Sponsor, Principal Investigators, the PSRT and SMC and the regulatory authorities, as appropriate.

14.0 STATISTICAL CONSIDERATIONS

14.1 Safety and Tolerability Analysis

14.1.1 Sample Size

The sample size for safety and tolerability analysis will be 35-56 participants according to the dose escalation design used to characterize the safety profile of one IV infusion of PGT121 mAb, at one of three dose levels, to HIV-uninfected and HIV-infected individuals (groups 1 and 2).

14.1.2 Null Hypothesis

As this is an exploratory proof of concept trial and analysis will be descriptive, no formal null hypothesis will be tested.

14.1.3 Statistical Power and Analysis and Dose Escalation Rules

The frequency of moderate or greater local and systemic reactogenicity events will be determined and compared between groups.

The frequency of SAEs judged possibly, probably or related to the IP will be determined.

All AEs will be analyzed and, grouped by seriousness, severity and relationship to the Investigational Product (as judged by the investigator).

For life-threatening adverse events related to Investigational Product: if none of the 12 (max 18) participants receiving Investigational Products experience such reactions, then the 95 % upper confidence bound for the rate of these adverse events in the population is 26.5% (or 18.5% if n=18).

All AEs will be analysed and grouped by seriousness, severity and relationship to the IP (as judged by the investigator).

For life-threatening adverse events related to IP: if none of the 12 (max 18) participants in either Group 1 or Group 2 who receive the IP experience such reactions then the 95% upper confidence bound for the rate of these adverse events in the population is 26.5% (or 18.5% if n=18).

An interim analysis of group data will be carried out according to the study schema (Table 5.3.1) without unblinding the study to investigators or participants. At the end of the study, a full analysis will be prepared.

Based on previous experience with IAVI Phase 1 IP studies, it is expected that the amount of missing, unused or spurious data will be insignificant. Unused and spurious data will be listed separately and excluded from the statistical analysis. Missing data will be excluded from the statistical analysis.

14.2 Pharmacokinetic Analysis

14.2.1 Sample Size

The sample size for pharmacokinetic analysis will be 4 per dose subgroup, sufficient to provide sufficient information for the planned analyses.

14.2.2 Null Hypothesis

As this is an exploratory proof of concept trial and analysis will be descriptive, no formal null hypothesis will be tested.

14.2.3 Statistical Power and Analysis

Disposition of PGT121 mAb will be evaluated in this study. Based on the PK profile of other human monoclonal antibodies, it is expected that the half-life of PGT121 mAb will be 14 to 21 days. Previously published data indicates that the pharmacokinetics of PGT121 and 3BNC117 are fairly similar across a non-human primate cohort and within the same non-human primate (clearance of 3BNC117 appears to be marginally faster than that for PGT121).

Commonly reported PK parameters will be calculated using standard non-compartmental slope/height/area/moment (SHAM) analysis methods. Summary descriptive results of PK parameters, including AUC, Cmax, T1/2, and clearance results will be reported by dose cohort. Dose normalized plots of PK parameters will be presented. Correlation between PK and reported safety and pharmacodynamic outcomes will also be explored parameters in order to examine exposure-effect relationships.

A more powerful exploratory analysis to quantitatively determine the dose, participant and disease impact on PGT121 mAb pharmacokinetics, and correlate exposure with response, while correctly accounting for variance based on population intrinsic factors such as weight and gender will be performed. Using the proposed population analysis approach we will be able to simultaneously examine the magnitude and the rate of change to PGT121 disposition driven by HIV-1 RNA levels and/or ART, and also examine the magnitude and the rate of decline in log copies/ml of HIV-1 RNA plasma levels from baseline.

The frequency and levels of anti-PGT121 antibodies will be calculated and tabulated.

14.3 Virologic Analysis for Dose De-escalation in Groups 3A-C

14.3.1 Sample Size

The sample size for virologic analysis in Groups 3A-C will be 24-36 participants according to the dose de-escalation design described below.

14.3.2 Null Hypothesis

The null hypothesis is that the HIV RNA viral load difference-from-baseline is greater than -0.9 logs.

14.3.3 Statistical Power and Analysis

The virologic analysis described in this section relates to Groups 3A-C of the study design, in which dose de-escalation is performed in an adaptive study design in HIV-infected participants off ART with plasma HIV RNA levels of 2 x 10^3 – 10^5 copies/ml. This section assumes that Part 1 of the study has successfully demonstrated that there is a safe dose level of the IP such that the study is carried forward into Part 2.

The primary efficacy outcome for this analysis is defined as change in log10 viral load between Day 0 (day of infusion) and Day 7. The minimum clinically significant value for this outcome is defined as a difference of -0.9 log10.

The study plan for Groups 3A-C is designed so that the IP dose level may be de-escalated in a stepwise manner from the highest dose to the lowest dose, until a given dose level cannot be concluded to be efficacious. If any given dose level is proven to be efficacious at an interim analysis, enrolment for that dose level may cease, and the next lowest dose group may be enrolled. In the unlikely event that IP administration leads to increased viral load, this may be detected by this design. No placebo participants are enrolled as part of this design.

This design represents a dose de-escalation beginning at 30 mg/kg. The actual starting dose will be the MTD as determined by the SMC based on data from Part 1, therefore the starting dose may be 30mg/kg, 10 mg/kg or 3 mg/kg. If the starting dose is 30 mg/kg, then de-escalation will begin with Group 3A. If the starting dose is 10 mg/kg, then de-escalation will begin with Group 3B. If the starting dose is 3 mg/kg, then only Group 3C will be enrolled.

Assuming the starting dose is 30 mg/kg, an interim analysis of Group 3A will be performed after all 6 participants have reached 7 days following IP administration. The following scenarios may then occur, depending on the data:

- If the response in the first 6 participants results in an HIV RNA viral load difference-from-baseline significantly greater than -0.9 log10, the IP will be determined to be effective at 30 mg/kg, enrollment into Group 3A will cease, and enrollment into Group 3B will begin.
- If the mean response in the first 6 participants is a decrease smaller than -0.9 log10 HIV RNA, then an additional 3 participants will be enrolled into Group 3A. After the additional 3 participants have reached 7 days following IP administration, an analysis of Group 3A (N=9) will be performed:
    - If the response in all Group 3A results in an HIV RNA viral load difference-from-baseline significantly greater than -0.9 log10, the IP will be determined to be effective at 30 mg/kg, and enrollment into Group 3B will begin.
    - If the response in all 9 participants results in an HIV RNA viral load difference-from-baseline not significantly greater than -0.9 log10, then the IP will be determined to be ineffective at 30 mg/kg and Groups 3B and 3C will not be enrolled. In this scenario, no dose of IP will be determined to be effective.

If 30 mg/kg is determined to be an effective dose, then Group 3B will be enrolled at 10 mg/kg. An interim analysis of Group 3B will be performed after 8 participants have reached 7 days following IP administration. The following scenarios may then occur, depending on the data:

- If the response in the first 8 participants results in an HIV RNA viral load difference-from-baseline significantly greater than -0.9 log10, the IP will be determined to be effective at 10 mg/kg, enrollment into Group 3B will cease, and enrollment into Group 3C will begin.
- If the mean response in the first 8 participants is a decrease smaller than -0.9 log10 HIV RNA, then an additional 4 participants will be enrolled into Group 3B. After the additional 4 participants have reached 7 days following IP administration, an analysis of Group 3B (N=12) will be performed:
    - If the response in all Group 3B participants results in an HIV RNA viral load difference-from-baseline significantly greater than -0.9 log10, the IP will be determined to be effective at 10 mg/kg, and enrollment into Group 3C will begin.
    - If the response in all 12 participants results in an HIV RNA viral load difference-from-baseline not significantly greater than -0.9 log10, then the IP will be determined to be ineffective at 10 mg/kg, and Group 3C will not be enrolled. In this scenario, the minimum effective dose will be determined to be 30 mg/kg.

If 10 mg/kg is determined to be an effective dose, then Group 3C will be enrolled at 3 mg/kg. An interim analysis of Group 3C will be performed after 10 participants have reached 7 days following IP administration. The following scenarios may then occur, depending on the data:

- If the response in the first 10 participants results in an HIV RNA viral load difference-from-baseline significantly greater than -0.9 log10, the IP will be determined to be effective at 3 mg/kg and enrollment into Group 3C will cease. In this scenario, the minimum effective dose of the IP will be determined to be 3 mg/kg.
- If the mean response in the first 10 participants is a decrease smaller than -0.9 log10 HIV RNA, then an additional 5 participants will be enrolled into Group 3C. After the additional 5 participants have reached 7 days following IP administration, an analysis of Group 3C (N=15) will be performed:
    - If the response in all Group 3C participants results in an HIV RNA viral load difference-from-baseline not significantly greater than -0.9 log10, the minimum effective dose will be determined to be 3 mg/kg.
    - If the response in all 15 participants results in an HIV RNA viral load difference-from-baseline not significantly greater than -0.9 log10, then the IP will be determined to be ineffective at 3 mg/kg. In this scenario, the minimum effective dose will be determined to be 10 mg/kg.

For the analysis of sample size and power, log10 viral load differences from baseline for each participant were simulated from a normal distribution, with a standard deviation of 0.5. This value was chosen by examining a study of the antiretroviral drug raltegravir, which demonstrated a mean estimated standard deviation of the change of baseline of 0.47[18]. This is a conservative estimate, as the variability of viral loads near the lower range might be expected to also be lower.

The statistical test performed will be the Signed-ranktest, which will incorporate the "shift" parameter of -0.9 log10 (the minimum clinically significant difference selected for this study). An evaluation of potential harm (increased viral load) will also be performed with the Signed ranktest; this test will examine the null hypothesis of no change in viral load (a shift of 0.0 log10 following IP administration) against the one-sided alternative hypothesis that the viral load is increased following IP administration. Each efficacy test will be performed at the level $\alpha = 0.05$. Each test for harm will be performed at level $2\alpha = 0.10$, in order to provide additional sensitivity to detect potential harm.

14.4 Analysis of Antiviral Activity and Dose De-escalation in Subgroups 3D-F

14.4.1 Sample Size

The sample size for antiviral activity will be 3-9 participants, depending on the MTD.

14.4.2 Null Hypothesis

As this is an exploratory proof of concept trial and analysis will be descriptive in this population, no formal null hypothesis will be tested.

14.4.3 Statistical Power and Analysis

No efficacy endpoints will be tested in Groups 3D-F as participants are HIV-infected with low viral loads at baseline (10^2 – 2×10^3 copies/ml). Immunologic and virologic endpoints will be determined as described in Section 4.1. As soon as subgroup 3D has enrolled 3 participants, enrollment into subgroup 3E will be initiated. The same rule will apply for progression from subgroup 3E to 3F. In the event that dose de-escalation in subgroups 3A-C is stopped due to failure to meet virologic endpoints, dose de-escalation in subgroups 3D-F will continue.

14.5 Secondary and Exploratory Immunologic and Virologic Analyses

14.5.1 Sample Size

The sample size for secondary and exploratory immunologic and virologic analysis will be 63-93 participants.

14.5.2 Null Hypothesis

No formal hypothesis on immunologic or virologic responses will be tested, with the exception of the change in viral load described in Section 14.3.

14.5.3 Statistical Power and Analysis

Descriptive statistics (actual values and changes from reference) will be calculated for continuous immunologic and virologic parameters at all time points. Graphical representations of changes in parameters will be made as applicable. Differences between groups at a specific time point will be tested for exploratory purposes by a 2-sample t-test if the data appear to be normally distributed. If not, the non-parametric Wilcoxon rank sum test will be used. If portions of the measurements are censored

below the assay quantification limit, the Gehan-Wilcoxon test will be employed. All statistical tests will be two-sided and will be considered statistically significant if $p < 0.05$.

Frequency tabulations will be calculated for discrete (qualitative) immunologic and virologic parameters at all time points. Significant differences between groups will be determined by a 2-sided Fisher's exact test.

Interim immunologic and virologic analyses of grouped data may be performed without unblinding the study to investigators or participants.

15.0 QUALITY CONTROL AND QUALITY ASSURANCE

To ensure the quality and reliability of the data collected and generated and the ethical conduct of this study, a Study Operations Manual (SOM) will be developed. All deviations will be reported and investigated. The SOM describes reporting and deviation documentation requirements and procedures.

Regular monitoring will be performed according to ICH-GCP as indicated in Section 17.5.

An independent audit of the study and study sites may be performed by the Sponsor or designee to establish the status of applicable quality systems. Inspection by regulatory authorities may also occur.

By signing the protocol, the Principal Investigators agree to facilitate study related monitoring, audits, IRB/IEC review and regulatory inspection(s) and direct access to source documents. Such information will be treated as strictly confidential and under no circumstances be made publicly available.

16.0 DATA AND BIOLOGICAL MATERIAL

All data and biological material collected through the study shall be managed in accordance with the Clinical Trial Agreement (CTA). Distribution and use of these data will be conducted by agreement of all parties.

The computerized raw data generated will be held by the DCC on behalf of the Sponsor. The study sites will also hold the final data files and tables generated for the purpose of analysis.

17.0 ADMINISTRATIVE STRUCTURE

The Principal Investigator will be responsible for all aspects of the study at the study site.

17.1 Protocol Safety Review Team

A PSRT will be formed to monitor the clinical safety data. During the administration of IP phase of the trial, the PSRT will review the clinical safety data on a weekly basis via electronic distribution of reports. An ad hoc PSRT review meeting will occur if any of the members of the PSRT requests a special review to discuss a specific safety issue or as specified in the Study Operations Manual. After the administration of IP phase the PSRT will review the clinical safety data at least monthly.

The PSRT will consist of the IAVI Medical Monitor(s), and the PI or designee from each clinical team. The study chair or an IAVI Medical Monitor may be the PSRT chair. *Ex officio* members will include the IAVI Chief Medical Officer and an unblinded IAVI Medical Monitor.

Additional PSRT participants may include the following, as needed:
- Co-investigators and trial site senior clinical research nursing staff
- Laboratory directors
- Data management, study statistician and regulatory staff

The PSRT membership and procedures are detailed in the PSRT charter.

17.2 Safety Monitoring Committee (SMC)

The SMC will consist of independent clinicians/scientists/statisticians/ethicists who are not involved in the study. Investigators responsible for the clinical care of participants or representative of the Sponsor may not be a member of the SMC. Details of membership, chair and co-chair and responsibilities are outlined in the SMC charter.

Principal Investigator(s) or designee and/or a Sponsor representative may be asked to join an open session of the SMC meeting to provide information on study conduct, present data or to respond to questions.

Safety data will be reviewed by the SMC at pre-specified time points and at an ad-hoc basis.

17.2.1 Content of Interim Safety Review

The SMC will be asked to review the following blinded data:
- Summary of reactogenicity (i.e., solicited adverse events)
- All adverse events judged by the Principal Investigator or designee to be possibly, probably or definitely related to IP
- All laboratory results confirmed on retest and judged by the Principal Investigator or designee to be clinically significant
- All SAEs

An unblinded presentation of all above noted events may also be made available for the SMC for their review if required by any member of the SMC.

17.2.2 SMC Review of Group 1 and 2 data prior to starting Group 3

Following IV infusion of IP of the last participant in either Group 1C or 2C, the Safety Monitoring Committee (SMC) will review safety data through the day 14 post-IV infusion visit for all participants to confirm MTD in each group, and determine whether, and at what dose level, Group 3 can initiate enrollment. The SMC can meet to confirm MTD when either Group 1C or Group 2C has finished enrollment, and does not need to wait for both Groups to be completed to approve initiation of Group 3 enrollment. Data from Group 1D (SC administration) is not considered by SMC for initiation of Group 3 enrollment.

17.3 Criteria for Pausing the Study

Enrollment and administration of IP will be stopped and a safety review conducted by the SMC for any of the following criteria:

1. One or more participants experience an SAE that is judged possibly, probably or definitely related to IP.
2. There is a participant death, regardless of relationship to the IP.
3. Two or more participants experience Grade 3 adverse events in the same category System Organ Class that are considered to be possibly, probably or definitely related to IP or
4. Any grade 4 adverse event that is considered to be possibly, probably or definitely related to IP.

Table 17.3-1: AE notification and safety pause/AE review rules

Event and relationship to study product	Severity	Occurrence	Site PI action	PSRT or SMC action
SAE, possibly, probably or definitely related	Any	Any	Phone, email or fax forms to sponsor within 24 hours	Study pause within 24 hours, refer to SMC for review
SAE, probably not or not related	Death	Any	Phone, email or fax forms to sponsor within 24 hours	Study pause within 24 hours, refer to SMC for review
AE, possibly, probably or definitely related	Grade 4	Any	Phone, email or fax notification to sponsor within 24 hours	Study pause within 24 hours, refer to SMC for review
AE¶, possibly, probably or definitely related	Grade 3*	First	Phone, email or fax notification to sponsor within 24 hours	PSRT review within 2 business days to consider pause
AE¶, possibly, probably or definitely related	Grade 3*	Second‡	Phone, email or fax notification to sponsor within 24 hours	Study pause within 24 hours, refer to SMC for review

¶Does not include the following reactogenicity symptoms (fever, fatigue/malaise, myalgia, arthralgia, chills, headache, nausea, vomiting).

*If no evidence of disease is present other than an abnormal laboratory value, the test must be repeated with a new blood sample at least one time within 72 hours after the investigator becoming aware of the abnormal laboratory value. When signs and symptoms are present, repeat test will not be needed.

‡PSRT will determine whether the reported related AE (Grade 3) is a second occurrence of a previously reported AE (Grade 3).

The Sponsor will request a review by the SMC, (or the SMC chair if other SMC members cannot be convened), to be held within 2 business days of the Sponsor learning of the event. The individual participant(s)/or study may be unblinded at the discretion of the SMC.

Following this review, the SMC will make a recommendation regarding the continuation or suspension of the administration of the IP or the trial and communicate this decision immediately to the Sponsor. The Sponsor then will inform the Principal Investigators without delay.

Additional *ad hoc* review may be specifically requested by the Sponsor, the Principal Investigator(s) or by the SMC.

17.4 Study Supervision

The SMC, the IAVI Chief Medical Officer (CMO) and the IAVI Medical Monitor(s) have access to progress report(s) of this study. Close cooperation will be necessary to track study progress, respond to queries about proper study implementation and management, address issues in a timely manner, and assure consistent documentation, and share information effectively. Rates of accrual, retention, and other parameters relevant to the site's performance will be regularly and closely monitored by the study team.

17.5 Study Monitoring

On-and/or off-site monitoring will ensure that the study is conducted in compliance with human subjects' protection and other research regulations and guidelines, recorded and reported in accordance with the protocol, is consistent with SOPs, GCP, applicable regulatory requirements and locally accepted practices. The monitor will confirm the quality and accuracy of data at the site by validation of CRFs against the source documents, such as clinical records. The investigators, as well as participants through consenting to the study, agree that the monitor may inspect study facilities and source records (e.g., informed consent forms, clinic and laboratory records, other source documents), as well as observe the performance of study procedures (in accordance with site IRB requirements). Such information will be treated as strictly confidential and will under no circumstances be made publicly available.

The monitoring will adhere to GCP guidelines. The Principal Investigator will permit inspection of the facilities and all study-related documentation by authorized representatives of IAVI, and Government and Regulatory Authorities responsible for this study.

17.6 Investigator's Records

Study records include administrative documentation—e.g., reports and correspondence relating to the study—as well as documentation related to each participant screened and/or enrolled in the study—including informed consent forms, case report forms, and all other source documents. The investigator will maintain and store, in a secure manner, complete, accurate, and current study records for a minimum of 2 years after marketing application approval or the study is discontinued and applicable national and local health authorities are notified. IAVI will notify the Principal Investigator of these events.

18.0 INDEMNITY

The Sponsor and Institution are responsible to have appropriate liability insurance. For research-related injuries and/or medical problems determined to result from receiving the IP,

treatment including necessary emergency treatment and proper follow-up care will be made available to the participant free of charge at the expense of the Sponsor.

19.0 PUBLICATION

A primary manuscript describing safety, anti-viral effect and immune responses in this trial will be prepared promptly after the data analysis is available.

Authors will be representatives of each trial site, the data management and statistical analysis center, the laboratories, the product developer and the sponsor, participant to the generally accepted criteria of contributions to the design and conduct of the study, the analysis of data and writing of the manuscript. Precedence will be given to authors from the site enrolling the greatest number of participants. Manuscripts will be reviewed by representatives of each participating group as specified in the CTA.

20.0 ETHICAL CONSIDERATIONS

The Principal Investigator will ensure that the study is conducted in compliance with the protocol, SOPs in accordance with guidelines formulated by the ICH for GCP in clinical studies, the ethical principles that have their origins in the Declaration of Helsinki and applicable local standards and regulatory requirements.

APPENDIX A: SCHEDULE OF PROCEDURES – GROUP 1 (A, B, C, D)

Study Month		0							1		2		3	4	5	6
**Study Week**		**0**				**1**	**2**	**3**	**4**	**6**	**8**	**10**	**12**	**16**	**20**	**24**
**Study Day**	**Scr**	**0**	**1**	**2**	**3**	**7**	**14**	**21**	**28**	**42**	**56**	**70**	**84**	**112**	**140**	**168/ET[4]**
**Visit Windows (Days)**	**-56**	**0**	**0**	**0**	**0**	**± 2**	**± 2**	**± 2**	**± 2**	**± 3**	**± 3**	**±3**	**±7**	**±7**	**±7**	**±7**
**INVESTIGATIONAL PRODUCT**																
Investigational Product /Placebo		X														
**CONSENT/ASSESSMENTS/COUNSELLING**																
Informed Consent	X															
Assessment of Understanding	X															
HIV Risk Assessment	X															X
HIV Risk Reduction Counselling	X	X							X		X		X	X	X	X
HIV-test Counselling	X	X							X							X
Family Planning Counselling	X	X														
Social Impact Assessment																X
**CLINICAL SAFETY ASSESSMENTS**																
Comprehensive Medical History	X															
Interim Medical History		X	X	X	X	X	X	X	X	X	X					
Concomitant Medications	X	X	X	X	X	X	X	X	X	X	X					
General Physical Exam	X															X
Directed Physical Exam		X	X	X	X	X	X	X	X	X	X	X	X	X	X	
Weight	X	X														X
Height	X															
Vital Signs	X	X[1]	X	X	X	X	X	X	X	X	X	X	X	X	X	X
Local & Systemic Reactogenicity		X[1]	X	X	X											
Adverse Events		X	X	X	X	X	X	X	X	X	X					
Serious Adverse Events and pIMD	X	X	X	X	X	X	X	X	X	X	X	X	X	X	X	X

Study Month		0							1		2		3	4	5	6
**Study Week**		**0**				**1**	**2**	**3**	**4**	**6**	**8**	**10**	**12**	**16**	**20**	**24**
**Study Day**	**Scr**	**0**	**1**	**2**	**3**	**7**	**14**	**21**	**28**	**42**	**56**	**70**	**84**	**112**	**140**	**168/ET[4]**
**Visit Windows (Days)**	**-56**	**0**	**0**	**0**	**0**	**± 2**	**± 2**	**± 2**	**± 2**	**± 3**	**± 3**	**±3**	**±7**	**±7**	**±7**	**±7**
**CLINICAL LABORATORY TESTS**																
Hematology and Coagulation	X	X[6]	X		X	X	X		X		X		X	X	X	X
Clinical Chemistry	X	X[6]	X		X	X	X		X		X		X	X	X	X
Urine Dipstick	X	X[6]	X		X	X	X		X		X		X	X	X	X
Urine Pregnancy test	X	X[6]							X		X		X			X
Active Syphilis	X															
Hepatitis B	X															
Hepatitis C	X															
HIV screen (4th generation Ag/Ab test)	X															
Blinded HIV diagnostic testing[2]		X[6]							X							X
**RESEARCH LABORATORY TESTS**	-	-	-	-	-	-	-	-	-	-	-	-	-	-	-	-
Anti PGT121 Antibodies (ADA)		X[6]							X		X		X			X
Humoral Assays[2]		X[6]			X	X	X		X		X		X			X
Cellular Assays[2]		X[6]					X		X		X		X			X
HLA typing		X[6]														
**PHARMACOKINETICS PGT121 ELISA**		X[3]	X	X	X	X	X	X	X	X	X	X	X	X	X	X
**MUCOSAL SAMPLING**		X[5]	X			X	X									
**PLASMA/SERUM STORAGE**		X	X	X	X	X	X	X	X		X		X			X
**PBMCs STORAGE**		X	X	X	X	X		X			X		X			X

1. At baseline, approximately 30 minutes after IP administration, and at approximately hours 1 through 6 after IV infusion or SC injection. Local and systemic reactogenicity will be assessed by clinic staff at visits on study days 1, 2 and 3. Local and Systemic reactogenicity will also be assessed by the participant using the Memory Aid on study days 1, 2, and 3
2. See Laboratory Analytical Plan for details
3. Day 0 PK draws done immediately before IP administration, at the end of IP administration, 30 minutes after end of IP administration, and 3 hours and 6 hours post IP administration. See SOM for details
4. Early Termination (ET): Procedures to be performed at ET are the same as last visit procedures
5. Cervico-vaginal and/or rectal mucosal sampling (optional) on Day 0 must be done prior to IV infusion or SC injection of IP. See SOM for details

6. Day 0 sample collections for laboratory tests must be done pre-infusion or pre-injection.

APPENDIX B: SCHEDULE OF PROCEDURES – GROUP 2 (A, B, C)

Study Month		0							1		2		3	4	5	6
Study Week		0				1	2	3	4	6	8	10	12	16	20	24
Study Day	Scr	0	1	2	3	7	14	21	28	42	56	70	84	112	140	168/ET[5]
Visit Windows (Days)	-56	0	0	0	0	± 2	± 2	± 2	± 2	± 3	± 3	±3	±7	±7	±7	±7
**INVESTIGATIONAL PRODUCT**																
Investigational Product /Placebo		X														
**CONSENT/ASSESSMENTS/COUNSELLING**																
Informed Consent	X															
Assessment of Understanding	X															
HIV Risk Reduction Counselling[1]	X	X							X		X		X	X	X	X
Family Planning Counselling	X	X														
Social Impact Assessment																X
**CLINICAL SAFETY ASSESSMENTS**																
Comprehensive Medical History	X															
Interim Medical History		X	X	X	X	X	X	X	X	X	X					
Concomitant Medications	X	X	X	X	X	X	X	X	X	X	X					
General Physical Exam	X															X
Directed Physical Exam		X	X	X	X	X	X	X	X	X	X	X	X	X	X	
Weight	X	X														X
Height	X															
Vital Signs	X	X[2]	X	X	X	X	X	X	X	X	X	X	X	X	X	X
Local & Systemic Reactogenicity		X[2]	X	X	X											
Adverse Events		X	X	X	X	X	X	X	X	X	X					
Serious Adverse Events and pIMD	X	X	X	X	X	X	X	X	X	X	X	X	X	X	X	X
**CLINICAL LABORATORY TESTS**																
Hematology and Coagulation	X	X[8]	X		X	X	X		X		X		X	X	X	X

Study Month		0							1		2		3	4	5	6
**Study Week**		**0**				**1**	**2**	**3**	**4**	**6**	**8**	**10**	**12**	**16**	**20**	**24**
**Study Day**	**Scr**	**0**	**1**	**2**	**3**	**7**	**14**	**21**	**28**	**42**	**56**	**70**	**84**	**112**	**140**	**168/ET[5]**
**Visit Windows (Days)**	**-56**	**0**	**0**	**0**	**0**	**± 2**	**± 2**	**± 2**	**± 2**	**± 3**	**± 3**	**±3**	**±7**	**±7**	**±7**	**±7**
CD4	X	X[8]				X	X		X		X					X
Clinical Chemistry	X	X[8]	X		X	X	X		X		X		X	X	X	X
Urine Dipstick	X	X[8]	X		X	X	X		X		X		X	X	X	X
Urine Pregnancy test	X	X[8]							X		X		X			X
Active Syphilis	X															
Hepatitis B	X															
Hepatitis C	X															
HIV 4th generation Ag/Ab test	X															
HIV Viral Load	X	X[8]	X	X	X	X	X	X	X	X	X	X	X	X	X	X
**RESEARCH LABORATORY TESTS**	-	-	-	-	-	-	-	-	-	-	-	-	-	-	-	-
Anti PGT121 Antibodies (ADA)		X[8]							X		X		X			X
HIV SGA sequencing[7]	X								X							X
HIV genotypic testing for ART resistance[7]	X								X				X			X
HIV reservoir size assessment	X						X						X			
Humoral Assays[3]		X[8]			X	X	X		X		X		X			X
Cellular Assays[3]		X[8]					X		X		X		X			X
HLA typing		X[8]														
**PHARMACOKINETICS PGT121 ELISA**		X[4]	X	X	X	X	X	X	X	X	X	X	X	X	X	X
**MUCOSAL SAMPLING**		X[6]	X			X	X									
**PLASMA/SERUM STORAGE**	X	X	X	X	X	X	X	X	X		X		X			X
**PBMCs STORAGE**		X	X	X	X	X		X			X		X			X

1. HIV risk reduction counselling as secondary prevention to reduce onward transmission
2. At baseline, approximately 30 minutes after IP administration, and at approximately hours 1 through 6 after IV infusion. Local and systemic reactogenicity will be assessed by clinic staff at visits on study days 1, 2 and 3. Local and Systemic reactogenicity will also be assessed by the participant using the Memory Aid on study days 1, 2, and 3
3. See Laboratory Analytical Plan for details

4. Day 0 PK draws done immediately before IP administration, at the end of IP administration, 30 minutes after end of IP administration, and 3 hours and 6 hours post IP administration. See SOM for details
5. Early Termination (ET): Procedures to be performed at ET are the same as last visit procedures
6. Cervico-vaginal and/or rectal mucosal sampling (optional) on Day 0 must be done prior to IV infusion of IP. See SOM for details
7. HIV envelope single genome amplification (SGA) sequencing and ART resistance genotyping will be only if they develop detectable plasma viremia >1000 HIV cp/ml during the study.
8. Day 0 sample collections for laboratory tests must be done pre-infusion

APPENDIX C: SCHEDULE OF PROCEDURES – GROUP 3 (A, B, C, D, E, F)

**Study Month**		**0**								**1**		**2**		**3**	**4**	**5**	**6**
**Study Week**		**0**				**1**		**2**	**3**	**4**	**6**	**8**	**10**	**12**	**16**	**20**	**24**
**Study Day**	**Scr**	**0**	**1**	**2**	**3**	**7**	**10**	**14**	**21**	**28**	**42**	**56**	**70**	**84**	**112**	**140**	**168/ET[5]**
**Visit Windows (Days)**	**-42**	**0**	**0**	**0**	**0**	**± 2**	**0**	**± 2**	**± 2**	**± 2**	**± 3**	**± 3**	**±3**	**±7**	**±7**	**±7**	**±7**
**INVESTIGATIONAL PRODUCT**																	
Investigational Product /Placebo		X															
**CONSENT/ASSESSMENTS/COUNSELLING**																	
Informed Consent	X																
Assessment of Understanding	X																
HIV Risk Reduction Counselling[1]	X	X								X		X		X	X	X	X
ART counselling	X	X										X					X
Family Planning Counselling	X	X															
Social Impact Assessment																	X
**CLINICAL SAFETY ASSESSMENTS**																	
Comprehensive Medical History	X																
Interim Medical History		X	X	X	X	X	X	X	X	X	X	X					
Concomitant Medications	X	X	X	X	X	X	X	X	X	X	X	X					
General Physical Exam	X																X
Directed Physical Exam		X	X	X	X	X	X	X	X	X	X	X	X	X	X	X	
Weight	X	X															X
Height	X																
Vital Signs	X	X[2]	X	X	X	X	X	X	X	X	X	X	X	X	X	X	X
Local & Systemic Reactogenicity		X[2]	X	X	X												
Adverse Events		X	X	X	X	X	X	X	X	X	X	X					
Serious Adverse Events and pIMD	X	X	X	X	X	X	X	X	X	X	X	X	X	X	X	X	X

Study Month		0								1		2		3	4	5	6
**Study Week**		**0**				**1**		**2**	**3**	**4**	**6**	**8**	**10**	**12**	**16**	**20**	**24**
**Study Day**	**Scr**	**0**	**1**	**2**	**3**	**7**	**10**	**14**	**21**	**28**	**42**	**56**	**70**	**84**	**112**	**140**	**168/ET[5]**
**Visit Windows (Days)**	**-42**	**0**	**0**	**0**	**0**	**± 2**	**0**	**± 2**	**± 2**	**± 2**	**± 3**	**± 3**	**±3**	**±7**	**±7**	**±7**	**±7**
**CLINICAL LABORATORY TESTS**																	
Hematology and Coagulation	X	X[9]	X		X	X		X		X		X		X	X	X	X
CD4	X	X[9]				X		X		X		X					X
Clinical Chemistry	X	X[9]	X		X	X		X		X		X		X	X	X	X
Urine Dipstick[7]	X	X[9]	X		X	X		X		X		X		X	X	X	X
Urine Pregnancy test	X	X[9]								X		X		X			X
Active Syphilis	X																
Hepatitis B	X																
Hepatitis C	X																
HIV 4th generation Ag/Ab test***	X																
HIV Viral Load	X	X[9]	X	X	X	X	X	X	X	X	X	X	X	X	X	X	X
**RESEARCH LABORATORY TESTS**	-	-	-	-	-	-	-	-	-	-	-	-	-	-	-	-	-
Anti PGT121 Antibodies (ADA)		X[9]								X		X		X			X
PGT121 susceptibility testing	X									X							X
HIV SGA sequencing[8]	X									X							X
HIV genotypic testing for ART resistance[8]	X									X				X			X
HIV reservoir size assessment[1]	X							X						X			
Humoral Assays[3]		X[9]			X	X		X		X		X		X			X
Cellular Assays[3]		X[9]						X		X		X		X			X
HLA typing		X[9]															
**PHARMACOKINETICS PGT121 ELISA**		X[4]	X	X	X	X		X	X	X	X	X	X	X	X	X	X
**MUCOSAL SAMPLING**		X[6]	X			X		X									
**PLASMA/SERUM STORAGE**		X	X	X	X	X	X	X	X	X		X		X			X
**PBMCs STORAGE**		X	X	X	X	X	X		X			X		X			X

1. HIV risk reduction counselling as secondary prevention to reduce onward transmission
2. At baseline, approximately 30 minutes after IP administration, and at approximately hours 1 through 6 after IV infusion. Local and systemic reactogenicity will be assessed by clinic staff at visits on study days 1, 2 and 3. Local and Systemic reactogenicity will also be assessed by the participant using the Memory Aid on study days 1, 2, and 3
3. See Laboratory Analytical Plan for details
4. Day 0 PK draws done immediately before IP administration, at the end of the IP administration, 30 minutes after end of IP administration and 3 hours and 6 hours post IP administration. See SOM for details
5. Early Termination (ET): Procedures to be performed at ET are the same as last visit procedures
6. Cervico-vaginal and/or rectal mucosal sampling (optional) on Day 0 must be done prior to IV infusion of IP. See SOM for details
7. Urinalysis will only be conducted at visits after screening if clinically indicated.
8. HIV envelope single genome amplification (SGA) sequencing and ART resistance genotyping will be only if they develop detectable plasma viremia >1000 HIV cp/ml during the study.
9. Day 0 sample collections for laboratory tests must be done pre-infusion.

*** Confirmed HIV-1 infection (HIV Ab+ or HIV RNA+) by documentation in the medical records or in-clinic HIV testing.

APPENDIX D: LOW RISK CRITERIA

Low risk will be defined as:

1. **SEXUAL BEHAVIORS**

   In the **last 12 months** did not:

   - Have oral, vaginal or anal intercourse with an HIV-infected partner, or partner who uses injection drugs.
   - Gave or receive money, drugs, gifts, or services in exchange for oral, vaginal or anal sex

   AND

   In the **last 6 months** has abstained from penile/anal or penile/vaginal intercourse

   OR

   In the **last 6 months**:

   - Had 4 or fewer partners of the opposite birth sex for vaginal and/or anal intercourse, OR

   Is MSM (person born male with partner(s) born male) who, in the **last 12 months**:

   - Had 2 or fewer MSM partners for anal intercourse and had no unprotected anal sex with MSM, OR
   - Had unprotected anal intercourse with only 1 MSM partner, within a monogamous relationship lasting at least 12 months (during which neither partner had any other partners). If the monogamous relationship ended, the participant may then have had protected anal intercourse with 1 other MSM partner (total 2 or fewer partners in the last 12 months).

   Is a transgender person, regardless of the point on the transition spectrum, having sex with men (born male) and/or other transgender persons, who in the last 12 months:

   - Had 2 or fewer partners for anal or vaginal intercourse, and had no unprotected anal or vaginal sex, OR
   - Had unprotected anal or vaginal intercourse sex with 1 partner only within a monogamous relationship lasting at least 12 months (during which neither partner had any other partners). If the monogamous relationship ended, may then have had protected anal or vaginal sex with one other partner (total 2 or fewer partners in the last 12 months).

   AND

   Uses or intends to use condoms in situations which may include penile/anal or penile/vaginal intercourse with new partners of unknown HIV status, occasional partners, partners outside a primary relationship, and/or partners known to have other partners.

2. **NON-SEXUAL BEHAVIORS**

   In the **last 12 months** did not:

   - Inject drugs or other substances without a prescription
   - Use cocaine, methamphetamine, or excessive alcohol, which in the investigator's judgement, rendered the participant at greater than low risk for acquiring HIV infection

The investigator's judgement should consider local epidemiologic information about HIV prevalence in the area and community networks.

A participant is NOT appropriate for inclusion if he/she:

Acquired an STI (ie, new infection) in the last 12 months:

- Syphilis
- Gonorrhea
- Non-gonococcal urethritis
- HSV-2
- Chlamydia
- Pelvic inflammatory disease (PID)
- Trichomonas
- Mucopurulent cervicitis
- Epididymitis
- Proctitis
- Lymphogranuloma venereum
- Chancroid
- Hepatitis B

APPENDIX E: ADVERSE EVENT SEVERITY ASSESSMENT TABLE

Adapted from: DIVISION OF AIDS (DAIDS) TABLE FOR GRADING THE SEVERITY OF ADULT AND PEDIATRIC ADVERSE EVENTS Version 2.0, November 2014

The *Division of AIDS* (*DAIDS) Table for Grading the Severity of Adult and Pediatric Adverse Events, Version 2.0* consists of parameters, or AEs, with severity grading guidance that are to be used in DAIDS clinical trials for safety data reporting to maintain accuracy and consistency in the evaluation of AEs.

I. Instructions and Clarifications

Grading Adult and Pediatric AEs

When a single parameter is not appropriate for grading an AE in both adult and pediatric populations, separate parameters with specified age ranges are provided. If no distinction between adult and pediatric populations has been made, the listed parameter should be used for grading an AE in both populations.

Note: The term "severe" is not the same as "serious" in classifying AEs. The severity of a specific event describes its intensity, and it is the intensity which is graded. Seriousness, which is not graded, relates to an outcome of an AE and is a regulatory definition.

Estimating Severity Grade for Parameters Not Identified in the Table

The functional table below should be used to grade the severity of an AE that is not specifically identified in the grading table. In addition, all deaths related to an AE are to be classified as grade 5.

PARAMETER	GRADE 1 MILD	GRADE 2 MODERATE	GRADE 3 SEVERE	GRADE 4 POTENTIALLY LIFE-THREATENING
**Clinical** adverse event **NOT** identified elsewhere in the grading table	Mild symptoms causing no or minimal interference with usual social & functional activities with intervention not indicated	Moderate symptoms causing greater than minimal interference with usual social & functional activities with intervention indicated	Severe symptoms causing inability to perform usual social & functional activities with intervention or hospitalization indicated	Potentially life-threatening symptoms causing inability to perform basic self-care functions with intervention indicated to prevent permanent impairment, persistent disability, or death

Determining Severity Grade for Parameters "Between Grades"

If the severity of an AE could fall in either one of two grades (e.g., the severity of an AE could be either Grade 2 or Grade 3), sites should select the higher of the two grades.

Laboratory Values

General. An asymptomatic, abnormal laboratory finding without an accompanying AE should not be reported to DAIDS in an expedited timeframe unless it meets protocol-specific reporting requirements. Sites should refer to the applicable network standards for reporting abnormal laboratory findings on the CRFs.

Values Below Grade 1

Any laboratory value that is between the ULN and grade 1 (for high values) or the LLN and grade 1 (for low values) should not be graded or reported as an AE. Sites should consult the Manual for Expedited Reporting of Adverse Events to DAIDS, Version 2.0 and their protocol when making an assessment of the need to report an AE.

Overlap of Local Laboratory Normal Values with Grading Table Ranges.

When local laboratory normal values fall within grading table laboratory ranges, the severity grading is based on the ranges in the grading table unless there is a protocol-specific grading criterion for the

laboratory value. For example, “Magnesium, Low" has a grade 1 range of 1.2 to < 1.4 mEq/L, while a particular laboratory’s normal range for magnesium may be 1.3 to 2.8 mEq/L. If a study participant’s magnesium laboratory value is 1.3 mEq/L, the laboratory value should be graded as grade 1.

II. **<u>Definitions of terms used in the Table:</u>**

Basic Self-care Functions	<u>Adult</u><br>Activities such as bathing, dressing, toileting, transfer or movement, continence, and feeding.<br><br><u>Young Children</u><br>Activities that are age and culturally appropriate, such as feeding one’s self with culturally appropriate eating implements.
LLN	Lower limit of normal
Intervention	Medical, surgical, or other procedures recommended or provided by a<br>healthcare professional for the treatment of an adverse event.
NA	Not Applicable
ULN	Upper limit of normal
Usual Social & Functional Activities	Activities which adults and children perform on a routine basis and<br>those which are part of regular activities of daily living, for example:<br><br><u>Adults</u><br>Adaptive tasks and desirable activities, such as going to work, shopping, cooking, use of transportation, or pursuing a hobby.<br><br><u>Young Children</u><br>Activities that are age and culturally appropriate, such as social interactions, play activities, or learning tasks.

Major Clinical Conditions

Cardiovascular

PARAMETER	GRADE 1 MILD	GRADE 2 MODERATE	GRADE 3 SEVERE	GRADE 4 POTENTIALLY LIFE-THREATENING
**Arrhythmia** (by ECG or physical examination) *Specify type, if applicable*	No symptoms AND No intervention indicated	No symptoms AND Non-urgent intervention indicated	Non-life-threatening symptoms AND Non-urgent intervention indicated	Life-threatening arrhythmia OR Urgent intervention indicated
**Blood Pressure Abnormalities**[1] ***Hypertension*** *(with the lowest reading taken after repeat testing during a visit)* *≥ 18 years of age*	140 to < 160 mmHg systolic OR 90 to < 100 mmHg diastolic	≥ 160 to < 180 mmHg systolic OR ≥ 100 to < 110 mmHg diastolic	≥ 180 mmHg systolic OR ≥ 110 mmHg diastolic	Life-threatening consequences in a participant not previously diagnosed with hypertension (e.g., malignant hypertension) OR Hospitalization indicated
< 18 years of age	> 120/80 mmHg	≥ 95th to < 99th percentile + 5 mmHg adjusted for age, height, and gender (systolic and/or diastolic)	≥ 99th percentile + 5 mmHg adjusted for age, height, and gender (systolic and/or diastolic)	Life-threatening consequences in a participant not previously diagnosed with hypertension (e.g., malignant hypertension) OR Hospitalization indicated
Hypotension	No symptoms	Symptoms corrected with oral fluid replacement	Symptoms AND IV fluids indicated	Shock requiring use of vasopressors or mechanical assistance to maintain blood pressure
**Cardiac Ischemia or Infarction** *Report only one*	NA	NA	New symptoms with ischemia (stable angina) OR New testing consistent with ischemia	Unstable angina OR Acute myocardial infarction
**Heart Failure**	No symptoms AND Laboratory or cardiac imaging abnormalities	Symptoms with mild to moderate activity or exertion	Symptoms at rest or with minimal activity or exertion (e.g., hypoxemia) OR Intervention indicated (e.g., oxygen)	Life-threatening consequences OR Urgent intervention indicated (e.g., vasoactive medications, ventricular assist device, heart transplant)
**Hemorrhage** (with significant acute blood loss)	NA	Symptoms AND No transfusion indicated	Symptoms AND Transfusion of ≤ 2 units packed RBCs indicated	Life-threatening hypotension OR Transfusion of > 2 units packed RBCs (for children, packed RBCs > 10 cc/kg) indicated

[1] Blood pressure norms for children < 18 years of age can be found in: Expert Panel on Integrated Guidelines for Cardiovascular Health and Risk Reduction in Children and Adolescents. *Pediatrics* 2011;128;S213; originally published online November 14, 2011; DOI: 10.1542/peds.2009- 2107C.

PARAMETER	GRADE 1 MILD	GRADE 2 MODERATE	GRADE 3 SEVERE	GRADE 4 POTENTIALLY LIFE-THREATENING
**Prolonged PR Interval or AV Block** *Report only one* *> 16 years of age*	PR interval 0.21 to < 0.25 seconds	PR interval ≥ 0.25 seconds OR Type I 2nd degree AV block	Type II 2nd degree AV block OR Ventricular pause ≥ 3.0 seconds	Complete AV block
≤ 16 years of age	1st degree AV block (PR interval > normal for age and rate)	Type I 2nd degree AV block	Type II 2nd degree AV block OR Ventricular pause ≥ 3.0 seconds	Complete AV block
**Prolonged QTc Interval**[2]	0.45 to 0.47 seconds	> 0.47 to 0.50 seconds	> 0.50 seconds OR ≥ 0.06 seconds above baseline	Life-threatening consequences (e.g., Torsade de pointes, other associated serious ventricular dysrhythmia)
**Thrombosis or Embolism** *Report only one*	NA	Symptoms AND No intervention indicated	Symptoms AND Intervention indicated	Life-threatening embolic event (e.g., pulmonary embolism, thrombus)

[2] As per Bazett's formula.

Dermatologic

PARAMETER	GRADE 1 MILD	GRADE 2 MODERATE	GRADE 3 SEVERE	GRADE 4 POTENTIALLY LIFE-THREATENING
**Alopecia** (scalp only)	Detectable by study participant, caregiver, or physician AND Causing no or minimal interference with usual social & functional activities	Obvious on visual inspection AND Causing greater than minimal interference with usual social & functional activities	NA	NA
**Bruising**	Localized to one area	Localized to more than one area	Generalized	NA
**Cellulitis**	NA	Non-parenteral treatment indicated (e.g., oral antibiotics, antifungals, antivirals)	IV treatment indicated (e.g., IV antibiotics, antifungals, antivirals)	Life-threatening consequences (e.g., sepsis, tissue necrosis)
**Hyperpigmentation**	Slight or localized causing no or minimal interference with usual social & functional activities	Marked or generalized causing greater than minimal interference with usual social & functional activities	NA	NA

**Hypopigmentation**	Slight or localized causing no or minimal interference with usual social & functional activities	Marked or generalized causing greater than minimal interference with usual social & functional activities	NA	NA
**Petechiae**	Localized to one area	Localized to more than one area	Generalized	NA
**Pruritus**[3]<br>(without skin lesions)	Itching causing no or minimal interference with usual social & functional activities	Itching causing greater than minimal interference with usual social & functional activities	Itching causing inability to perform usual social & functional activities	NA
**Rash**<br>*Specify type, if applicable*	Localized rash	Diffuse rash OR Target lesions	Diffuse rash AND Vesicles or limited number of bullae or superficial ulcerations of mucous membrane limited to one site	Extensive or generalized bullous lesions OR Ulceration of mucous membrane involving two or more distinct mucosal sites OR Stevens-Johnson syndrome OR Toxic epidermal necrolysis

[3] For pruritus associated with injections or infusions, see the *Site Reactions to Injections and Infusions* section (page 23).

Endocrine and Metabolic

PARAMETER	GRADE 1<br>MILD	GRADE 2<br>MODERATE	GRADE 3<br>SEVERE	GRADE 4<br>POTENTIALLY LIFE-THREATENING
**Diabetes Mellitus**	Controlled without medication	Controlled with medication OR Modification of current medication regimen	Uncontrolled despite treatment modification OR Hospitalization for immediate glucose control indicated	Life-threatening consequences (e.g., ketoacidosis, hyperosmolar non-ketotic coma, end organ failure)
**Gynecomastia**	Detectable by study participant, caregiver, or physician AND Causing no or minimal interference with usual social & functional activities	Obvious on visual inspection AND Causing pain with greater than minimal interference with usual social & functional activities	Disfiguring changes AND Symptoms requiring intervention or causing inability to perform usual social & functional activities	NA

**Hyperthyroidism**	No symptoms AND Abnormal laboratory value	Symptoms causing greater than minimal interference with usual social & functional activities OR Thyroid suppression therapy indicated	Symptoms causing inability to perform usual social & functional activities OR Uncontrolled despite treatment modification	Life-threatening consequences (e.g., thyroid storm)
**Hypothyroidism**	No symptoms AND Abnormal laboratory value	Symptoms causing greater than minimal interference with usual social & functional activities OR Thyroid replacement therapy indicated	Symptoms causing inability to perform usual social & functional activities OR Uncontrolled despite treatment modification	Life-threatening consequences (e.g., myxedema coma)
**Lipoatrophy**[4]	Detectable by study participant, caregiver, or physician AND Causing no or minimal interference with usual social & functional activities	Obvious on visual inspection AND Causing greater than minimal interference with usual social & functional activities	Disfiguring changes	NA
**Lipohypertrophy**[5]	Detectable by study participant, caregiver, or physician AND Causing no or minimal interference with usual social & functional activities	Obvious on visual inspection AND Causing greater than minimal interference with usual social & functional activities	Disfiguring changes	NA

[4] Definition: A disorder characterized by fat loss in the face, extremities, and buttocks.
[5] Definition: A disorder characterized by abnormal fat accumulation on the back of the neck, breasts, and abdomen.

Gastro-intestinal

PARAMETER	GRADE 1 MILD	GRADE 2 MODERATE	GRADE 3 SEVERE	GRADE 4 POTENTIALLY LIFE-THREATENING
**Anorexia**	Loss of appetite without decreased oral intake	Loss of appetite associated with decreased oral intake without significant weight loss	Loss of appetite associated with significant weight loss	Life-threatening consequences OR Aggressive intervention indicated (e.g., tube feeding, total parenteral nutrition)
**Ascites**	No symptoms	Symptoms AND Intervention indicated (e.g., diuretics, therapeutic paracentesis)	Symptoms recur or persist despite intervention	Life-threatening consequences

**Bloating or Distension**<br>*Report only one*	Symptoms causing no or minimal interference with usual social & functional activities	Symptoms causing greater than minimal interference with usual social & functional activities	Symptoms causing inability to perform usual social & functional activities	NA
**Cholecystitis**	NA	Symptoms AND Medical intervention indicated	Radiologic, endoscopic, or operative intervention indicated	Life-threatening consequences (e.g., sepsis, perforation)
**Constipation**	NA	Persistent constipation requiring regular use of dietary modifications, laxatives, or enemas	Obstipation with manual evacuation indicated	Life-threatening consequences (e.g., obstruction)
**Diarrhea**<br>*≥ 1 year of age*	Transient or intermittent episodes of unformed stools OR Increase of ≤ 3 stools over baseline per 24-hour period	Persistent episodes of unformed to watery stools OR Increase of 4 to 6 stools over baseline per 24-hour period	Increase of ≥ 7 stools per 24-hour period OR IV fluid replacement indicated	Life-threatening consequences (e.g., hypotensive shock)
< 1 year of age	Liquid stools (more unformed than usual) but usual number of stools	Liquid stools with increased number of stools OR Mild dehydration	Liquid stools with moderate dehydration	Life-threatening consequences (e.g., liquid stools resulting in severe dehydration, hypotensive shock)
**Dysphagia or Odynophagia**<br>*Report only one and specify location*	Symptoms but able to eat usual diet	Symptoms causing altered dietary intake with no intervention indicated	Symptoms causing severely altered dietary intake with intervention indicated	Life-threatening reduction in oral intake
**Gastrointestinal Bleeding**	Not requiring intervention other than iron supplement	Endoscopic intervention indicated	Transfusion indicated	Life-threatening consequences (e.g., hypotensive shock)

**PARAMETER**	**GRADE 1<br>MILD**	**GRADE 2<br>MODERATE**	**GRADE 3<br>SEVERE**	**GRADE 4<br>POTENTIALLY LIFE-THREATENING**
**Mucositis or Stomatitis**<br>*Report only one and specify location*	Mucosal erythema	Patchy pseudomembranes or ulcerations	Confluent pseudomembranes or ulcerations OR Mucosal bleeding with minor trauma	Life-threatening consequences (e.g., aspiration, choking) OR Tissue necrosis OR Diffuse spontaneous mucosal bleeding
**Nausea**	Transient (< 24 hours) or intermittent AND No or minimal interference with oral intake	Persistent nausea resulting in decreased oral intake for 24 to 48 hours	Persistent nausea resulting in minimal oral intake for > 48 hours OR Rehydration indicated (e.g., IV fluids)	Life-threatening consequences (e.g., hypotensive shock)

**Pancreatitis**	NA	Symptoms with hospitalization not indicated	Symptoms with hospitalization indicated	Life-threatening consequences (e.g., circulatory failure, hemorrhage, sepsis)
**Perforation** (colon or rectum)	NA	NA	Intervention indicated	Life-threatening consequences
**Proctitis**	Rectal discomfort with no intervention indicated	Symptoms causing greater than minimal interference with usual social & functional activities OR Medical intervention indicated	Symptoms causing inability to perform usual social & functional activities OR Operative intervention indicated	Life-threatening consequences (e.g., perforation)
**Rectal Discharge**	Visible discharge	Discharge requiring the use of pads	NA	NA
**Vomiting**	Transient or intermittent AND No or minimal interference with oral intake	Frequent episodes with no or mild dehydration	Persistent vomiting resulting in orthostatic hypotension OR Aggressive rehydration indicated (e.g., IV fluids)	Life-threatening consequences (e.g., hypotensive shock)

Musculoskeletal

PARAMETER	GRADE 1 MILD	GRADE 2 MODERATE	GRADE 3 SEVERE	GRADE 4 POTENTIALLY LIFE-THREATENING
**Arthralgia**	Joint pain causing no or minimal interference with usual social & functional activities	Joint pain causing greater than minimal interference with usual social & functional activities	Joint pain causing inability to perform usual social & functional activities	Disabling joint pain causing inability to perform basic self-care functions
**Arthritis**	Stiffness or joint swelling causing no or minimal interference with usual social & functional activities	Stiffness or joint swelling causing greater than minimal interference with usual social & functional activities	Stiffness or joint swelling causing inability to perform usual social & functional activities	Disabling joint stiffness or swelling causing inability to perform basic self-care functions
**Myalgia** (generalized)	Muscle pain causing no or minimal interference with usual social & functional activities	Muscle pain causing greater than minimal interference with usual social & functional activities	Muscle pain causing inability to perform usual social & functional activities	Disabling muscle pain causing inability to perform basic self-care functions
**Osteonecrosis**	NA	No symptoms but with radiographic findings AND No operative intervention indicated	Bone pain with radiographic findings OR Operative intervention indicated	Disabling bone pain with radiographic findings causing inability to perform basic self-care functions
**Osteopenia**[6] *≥ 30 years of age*	BMD t-score -2.5 to -1	NA	NA	NA
< 30 years of age	BMD z-score -2 to -1	NA	NA	NA
**Osteoporosis**[6] *≥ 30 years of age*	NA	BMD t-score < -2.5	Pathologic fracture (e.g., compression fracture causing loss of vertebral height)	Pathologic fracture causing life-threatening consequences
< 30 years of age	NA	BMD z-score < -2	Pathologic fracture (e.g., compression fracture causing loss of vertebral height)	Pathologic fracture causing life-threatening consequences

[6] BMD t and z scores can be found in: Kanis JA on behalf of the World Health Organization Scientific Group (2007). Assessment of osteoporosis at the primary health-care level. Technical Report. World Health Organization Collaborating Centre for Metabolic Bone Diseases, University of Sheffield, UK. 2007: Printed by the University of Sheffield.

Neurologic

PARAMETER	GRADE 1<br>MILD	GRADE 2<br>MODERATE	GRADE 3<br>SEVERE	GRADE 4<br>POTENTIALLY LIFE-THREATENING
**Acute CNS Ischemia**	NA	NA	Transient ischemic attack	Cerebral vascular accident (e.g., stroke with neurological deficit)
**Altered Mental Status** (for Dementia, see *Cognitive, Behavioral, or Attentional Disturbance* below)	Changes causing no or minimal interference with usual social & functional activities	Mild lethargy or somnolence causing greater than minimal interference with usual social & functional activities	Confusion, memory impairment, lethargy, or somnolence causing inability to perform usual social & functional activities	Delirium OR Obtundation OR Coma
**Ataxia**	Symptoms causing no or minimal interference with usual social & functional activities OR No symptoms with ataxia detected on examination	Symptoms causing greater than minimal interference with usual social & functional activities	Symptoms causing inability to perform usual social & functional activities	Disabling symptoms causing inability to perform basic self-care functions
**Cognitive, Behavioral, or Attentional Disturbance** (includes dementia and attention deficit disorder) *Specify type, if applicable*	Disability causing no or minimal interference with usual social & functional activities OR Specialized resources not indicated	Disability causing greater than minimal interference with usual social & functional activities OR Specialized resources on part- time basis indicated	Disability causing inability to perform usual social & functional activities OR Specialized resources on a full-time basis indicated	Disability causing inability to perform basic self-care functions OR Institutionalization indicated
**Developmental Delay** *< 18 years of age* *Specify type, if applicable*	Mild developmental delay, either motor or cognitive, as determined by comparison with a developmental screening tool appropriate for the setting	Moderate developmental delay, either motor or cognitive, as determined by comparison with a developmental screening tool appropriate for the setting	Severe developmental delay, either motor or cognitive, as determined by comparison with a developmental screening tool appropriate for the setting	Developmental regression, either motor or cognitive, as determined by comparison with a developmental screening tool appropriate for the setting
**Headache**	Symptoms causing no or minimal interference with usual social & functional activities	Symptoms causing greater than minimal interference with usual social & functional activities	Symptoms causing inability to perform usual social & functional activities	Symptoms causing inability to perform basic self-care functions OR Hospitalization indicated OR Headache with significant impairment of alertness or other neurologic function

PARAMETER	GRADE 1<br>MILD	GRADE 2<br>MODERATE	GRADE 3<br>SEVERE	GRADE 4<br>POTENTIALLY LIFE-THREATENING
**Neuromuscular Weakness** (includes myopathy and neuropathy)<br>*Specify type, if applicable*	Minimal muscle weakness causing no or minimal interference with usual social & functional activities OR No symptoms with decreased strength on examination	Muscle weakness causing greater than minimal interference with usual social & functional activities	Muscle weakness causing inability to perform usual social & functional activities	Disabling muscle weakness causing inability to perform basic self-care functions OR Respiratory muscle weakness impairing ventilation
**Neurosensory Alteration** (includes paresthesia and painful neuropathy)<br>*Specify type, if applicable*	Minimal paresthesia causing no or minimal interference with usual social & functional activities OR No symptoms with sensory alteration on examination	Sensory alteration or paresthesia causing greater than minimal interference with usual social & functional activities	Sensory alteration or paresthesia causing inability to perform usual social & functional activities	Disabling sensory alteration or paresthesia causing inability to perform basic self-care functions
**Seizures**<br>***New Onset Seizure*** *≥ 18 years of age*	NA	NA	1 to 3 seizures	Prolonged and repetitive seizures (e.g., status epilepticus) OR Difficult to control (e.g., refractory epilepsy)
< 18 years of age (includes new or pre- existing febrile seizures)	Seizure lasting < 5 minutes with < 24 hours postictal state	Seizure lasting 5 to < 20 minutes with < 24 hours postictal state	Seizure lasting ≥ 20 minutes OR > 24 hours postictal state	Prolonged and repetitive seizures (e.g., status epilepticus) OR Difficult to control (e.g., refractory epilepsy)
Pre-existing Seizure	NA	Increased frequency from previous level of control without change in seizure character	Change in seizure character either in duration or quality (e.g., severity or focality)	Prolonged and repetitive seizures (e.g., status epilepticus) OR Difficult to control (e.g., refractory epilepsy)
**Syncope**	Near syncope without loss of consciousness (e.g., pre-syncope)	Loss of consciousness with no intervention indicated	Loss of consciousness AND Hospitalization or intervention required	NA

Pregnancy, Puerperium, and Perinatal

PARAMETER	GRADE 1<br>MILD	GRADE 2<br>MODERATE	GRADE 3<br>SEVERE	GRADE 4<br>POTENTIALLY LIFE-THREATENING
**Fetal Death or Stillbirth** (report using mother's participant ID)<br>*Report only one*	NA	NA	Fetal loss occurring at ≥ 20 weeks gestation	NA
**Preterm Delivery**[7] (report using mother's participant ID)	Delivery at 34 to < 37 weeks gestational age	Delivery at 28 to < 34 weeks gestational age	Delivery at 24 to < 28 weeks gestational age	Delivery at < 24 weeks gestational age
**Spontaneous Abortion or Miscarriage**[8] (report using mother's participant ID)<br>*Report only one*	Chemical pregnancy	Uncomplicated spontaneous abortion or miscarriage	Complicated spontaneous abortion or miscarriage	NA

[7] Definition: A delivery of a live-born neonate occurring at ≥ 20 to < 37 weeks gestational age.
[8] Definition: A clinically recognized pregnancy occurring at < 20 weeks gestational age.

Psychiatric

PARAMETER	GRADE 1<br>MILD	GRADE 2<br>MODERATE	GRADE 3<br>SEVERE	GRADE 4<br>POTENTIALLY LIFE-THREATENING
**Insomnia**	Mild difficulty falling asleep, staying asleep, or waking up early	Moderate difficulty falling asleep, staying asleep, or waking up early	Severe difficulty falling asleep, staying asleep, or waking up early	NA
**Psychiatric Disorders** (includes anxiety, depression, mania, and psychosis)<br>*Specify disorder*	Symptoms with intervention not indicated OR Behavior causing no or minimal interference with usual social & functional activities	Symptoms with intervention indicated OR Behavior causing greater than minimal interference with usual social & functional activities	Symptoms with hospitalization indicated OR Behavior causing inability to perform usual social & functional activities	Threatens harm to self or others OR Acute psychosis OR Behavior causing inability to perform basic self-care functions
**Suicidal Ideation or Attempt**<br>*Report only one*	Preoccupied with thoughts of death AND No wish to kill oneself	Preoccupied with thoughts of death AND Wish to kill oneself with no specific plan or intent	Thoughts of killing oneself with partial or complete plans but no attempt to do so OR Hospitalization indicated	Suicide attempted

Respiratory

PARAMETER	GRADE 1 MILD	GRADE 2 MODERATE	GRADE 3 SEVERE	GRADE 4 POTENTIALLY LIFE-THREATENING
**Acute Bronchospasm**	Forced expiratory volume in 1 second or peak flow reduced to ≥ 70 to < 80% OR Mild symptoms with intervention not indicated	Forced expiratory volume in 1 second or peak flow 50 to < 70% OR Symptoms with intervention indicated OR Symptoms causing greater than minimal interference with usual social & functional activities	Forced expiratory volume in 1 second or peak flow 25 to < 50% OR Symptoms causing inability to perform usual social & functional activities	Forced expiratory volume in 1 second or peak flow < 25% OR Life-threatening respiratory or Hemodynamic compromise OR Intubation
**Dyspnea or Respiratory Distress** *Report only one*	Dyspnea on exertion with no or minimal interference with usual social & functional activities OR Wheezing OR Minimal increase in respiratory rate for age	Dyspnea on exertion causing greater than minimal interference with usual social & functional activities OR Nasal flaring OR Intercostal retractions OR Pulse oximetry 90 to < 95%	Dyspnea at rest causing inability to perform usual social & functional activities OR Pulse oximetry < 90%	Respiratory failure with ventilator support indicated (e.g., CPAP, BPAP, intubation)

Sensory

PARAMETER	GRADE 1 MILD	GRADE 2 MODERATE	GRADE 3 SEVERE	GRADE 4 POTENTIALLY LIFE-THREATENING
**Hearing Loss** *≥ 12 years of age*	NA	Hearing aid or intervention not indicated	Hearing aid or intervention indicated	Profound bilateral hearing loss (> 80 dB at 2 kHz and above) OR Non-serviceable hearing (i.e., >50 dB audiogram and <50% speech discrimination)
< 12 years of age (based on a 1, 2, 3, 4, 6 and 8 kHz audiogram)	> 20 dB hearing loss at ≤ 4 kHz	> 20 dB hearing loss at > 4 kHz	> 20 dB hearing loss at ≥ 3 kHz in one ear with additional speech language related services indicated (where available) OR Hearing loss sufficient to indicate therapeutic intervention, including hearing aids	Audiologic indication for cochlear implant and additional speech-language related services indicated (where available)

**Tinnitus**	Symptoms causing no or minimal interference with usual social & functional activities with intervention not indicated	Symptoms causing greater than minimal interference with usual social & functional activities with intervention indicated	Symptoms causing inability to perform usual social & functional activities	NA
**Uveitis**	No symptoms AND Detectable on examination	Anterior uveitis with symptoms OR Medicamylasal intervention indicated	Posterior or pan- uveitis OR Operative intervention indicated	Disabling visual loss in affected eye(s)
**Vertigo**	Vertigo causing no or minimal interference with usual social & functional activities	Vertigo causing greater than minimal interference with usual social & functional activities	Vertigo causing inability to perform usual social & functional activities	Disabling vertigo causing inability to perform basic self-care functions
**Visual Changes** (assessed from baseline)	Visual changes causing no or minimal interference with usual social & functional activities	Visual changes causing greater than minimal interference with usual social & functional activities	Visual changes causing inability to perform usual social & functional activities	Disabling visual loss in affected eye(s)

Systemic

PARAMETER	GRADE 1 MILD	GRADE 2 MODERATE	GRADE 3 SEVERE	GRADE 4 POTENTIALLY LIFE-THREATENING
**Acute Allergic Reaction**	Localized urticaria (wheals) with no medical intervention indicated	Localized urticaria with intervention indicated OR Mild angioedema with no intervention indicated	Generalized urticaria OR Angioedema with intervention indicated OR Symptoms of mild bronchospasm	Acute anaphylaxis OR Life-threatening bronchospasm OR Laryngeal edema
**Chills**	Symptoms causing no or minimal interference with usual social & functional activities	Symptoms causing greater than minimal interference with usual social & functional activities	Symptoms causing inability to perform usual social & functional activities	NA
**Cytokine Release Syndrome**[9]	Mild signs and symptoms AND Therapy (i.e., antibody infusion) interruption not indicated	Therapy (i.e., antibody infusion) interruption indicated AND Responds promptly to symptomatic treatment OR Prophylactic medications indicated for ≤ 24 hours	Prolonged severe signs and symptoms OR Recurrence of symptoms following initial improvement	Life-threatening consequences (e.g., requiring pressor or ventilator support)
**Fatigue or Malaise** *Report only one*	Symptoms causing no or minimal interference with usual social & functional activities	Symptoms causing greater than minimal interference with usual social & functional activities	Symptoms causing inability to perform usual social & functional activities	Incapacitating symptoms of fatigue or malaise causing inability to perform basic self-care functions
**Fever** (non-axillary temperatures only)	38.0 to < 38.6°C or 100.4 to < 101.5°F	≥ 38.6 to < 39.3°C or ≥101.5 to < 102.7°F	≥ 39.3 to < 40.0°C or ≥ 102.7 to < 104.0°F	≥ 40.0°C or ≥ 104.0°F
**Pain**[10] (not associated with study agent injections and not specified elsewhere) *Specify location*	Pain causing no or minimal interference with usual social & functional activities	Pain causing greater than minimal interference with usual social & functional activities	Pain causing inability to perform usual social & functional activities	Disabling pain causing inability to perform basic self-care functions OR Hospitalization indicated
**Serum Sickness**[11]	Mild signs and symptoms	Moderate signs and symptoms AND Intervention indicated (e.g., antihistamines)	Severe signs and symptoms AND Higher level intervention indicated (e.g., steroids or IV fluids)	Life-threatening consequences (e.g., requiring pressor or ventilator support)

[9] ~~Definition: A disorder characterized by nausea~~, headache, tachycardia, hypotension, rash, and/or shortness of breath.
[10] For pain associated with injections or infusions, see the *Site Reactions to Injections and Infusions* section (page 23).
[11] Definition: A disorder characterized by fever, arthralgia, myalgia, skin eruptions, lymphadenopathy, marked discomfort, and/or dyspnea.

PARAMETER	GRADE 1 MILD	GRADE 2 MODERATE	GRADE 3 SEVERE	GRADE 4 POTENTIALLY LIFE-THREATENING
**Underweight**[12] *> 5 to 19 years of age*	NA	WHO BMI z-score < -2 to ≤ -3	WHO BMI z-score < -3	WHO BMI z-score < -3 with life-threatening consequences
2 to 5 years of age	NA	WHO Weight-for-height z-score < -2 to ≤-3	WHO Weight-for- height z-score < -3	WHO Weight-for-height z-score < -3 with life-threatening consequences
< 2 years of age	NA	WHO Weight-for-length z-score < -2 to ≤-3	WHO Weight-for- length z-score < -3	WHO Weight-for-length z-score < -3 with life-threatening consequences
**Weight Loss** (excludes postpartum weight loss)	NA	5 to < 9% loss in body weight from baseline	≥ 9 to < 20% loss in body weight from baseline	≥ 20% loss in body weight from baseline OR Aggressive intervention indicated (e.g., tube feeding, total parenteral nutrition)

[12] WHO reference tables may be accesed by clicking the desired age range or by accessing the following URLs: http://www.who.int/growthref/who2007 bmi for age/en/ for participants > 5 to 19 years of age and http://www.who.int/childgrowth/standards/chart catalogue/en/ for those < 5 years of age.

Urinary

PARAMETER	GRADE 1 MILD	GRADE 2 MODERATE	GRADE 3 SEVERE	GRADE 4 POTENTIALLY LIFE-THREATENING
**Urinary Tract Obstruction**	NA	Signs or symptoms of urinary tract obstruction without hydronephrosis or renal dysfunction	Signs or symptoms of urinary tract obstruction with hydronephrosis or renal dysfunction	Obstruction causing life-threatening consequences

Site Reactions to Injections and Infusions

PARAMETER	GRADE 1 MILD	GRADE 2 MODERATE	GRADE 3 SEVERE	GRADE 4 POTENTIALLY LIFE-THREATENING
**Injection Site Pain or Tenderness** *Report only one*	Pain or tenderness causing no or minimal limitation of use of limb	Pain or tenderness causing greater than minimal limitation of use of limb	Pain or tenderness causing inability to perform usual social & functional activities	Pain or tenderness causing inability to perform basic self-care function OR Hospitalization indicated

**Injection Site Erythema or Redness**[13]<br>*Report only one*<br>*> 15 years of age*	2.5 to < 5 cm in diameter OR 6.25 to < 25 cm^2 surface area AND Symptoms causing no or minimal interference with usual social & functional activities	≥ 5 to < 10 cm in diameter OR ≥ 25 to < 100 cm^2 surface area OR Symptoms causing greater than minimal interference with usual social & functional activities	≥ 10 cm in diameter OR ≥ 100 cm^2 surface area OR Ulceration OR Secondary infection OR Phlebitis OR Sterile abscess OR Drainage OR Symptoms causing inability to perform usual social & functional activities	Potentially lifethreatening consequences (e.g., abscess, exfoliative dermatitis, necrosis involving dermis or deeper tissue)
≤15 years of age	≤ 2.5 cm in diameter	> 2.5 cm in diameter with < 50% surface area of the extremity segment involved (e.g., upper arm or thigh)	≥ 50% surface area of the extremity segment involved (e.g., upper arm or thigh) OR Ulceration OR Secondary infection OR Phlebitis OR Sterile abscess OR Drainage	Potentially life-threatening consequences (e.g., abscess, exfoliative dermatitis, necrosis involving dermis or deeper tissue)
**Injection Site Induration or Swelling**<br>*Report only one*<br>*> 15 years of age*	Same as for **Injection Site Erythema or Redness,** *> 15 years of age*	Same as for **Injection Site Erythema or Redness,** *> 15 years of age*	Same as for **Injection Site Erythema or Redness,** *> 15 years of age*	Same as for **Injection Site Erythema or Redness,** *> 15 years of age*
≤ 15 years of age	Same as for **Injection Site Erythema or Redness,** *≤ 15 years of age*	Same as for **Injection Site Erythema or Redness,** *≤ 15 years of age*	Same as for **Injection Site Erythema or Redness,** *≤ 15 years of age*	Same as for **Injection Site Erythema or Redness,** *≤ 15 years of age*
**Injection Site Pruritus**	Itching localized to the injection site that is relieved spontaneously or in < 48 hours of treatment	Itching beyond the injection site that is not generalized OR Itching localized to the injection site requiring ≥ 48 hours treatment	Generalized itching causing inability to perform usual social & functional activities	NA

[13] Injection Site Erythema or Redness should be evaluated and graded using the greatest single diameter or measured surface area.

LABORATORY VALUES

Chemistries

PARAMETER	GRADE 1 MILD	GRADE 2 MODERATE	GRADE 3 SEVERE	GRADE 4 POTENTIALLY LIFE-THREATENING
Acidosis	NA	pH ≥ 7.3 to < LLN	pH < 7.3 without life-threatening consequences	pH < 7.3 with life-threatening consequences
Albumin, Low (g/dL; *g/L*)	3.0 to < LLN *30 to < LLN*	≥ 2.0 to < 3.0 ≥ *20 to < 30*	< 2.0<br>*< 20*	NA
Alkaline Phosphatase, High	1.25 to < 2.5 x ULN	2.5 to < 5.0 x ULN	5.0 to < 10.0 x ULN	≥ 10.0 x ULN
Alkalosis	NA	pH > ULN to ≤ 7.5	pH > 7.5 without life-threatening consequences	pH > 7.5 with life-threatening consequences
ALT or SGPT, High *Report only one*	1.25 to < 2.5 x ULN	2.5 to < 5.0 x ULN	5.0 to < 10.0 x ULN	≥ 10.0 x ULN
Amylase (Pancreatic) or Amylase (Total), High *Report only one*	1.1 to < 1.5 x ULN	1.5 to < 3.0 x ULN	3.0 to < 5.0 x ULN	≥ 5.0 x ULN
AST or SGOT, High *Report only one*	1.25 to < 2.5 x ULN	2.5 to < 5.0 x ULN	5.0 to < 10.0 x ULN	≥ 10.0 x ULN
Bicarbonate, Low (mEq/L; *mmol/L*)	16.0 to < LLN *16.0 to < LLN*	11.0 to < 16.0 *11.0 to < 16.0*	8.0 to < 11.0 *8.0 to < 11.0*	< 8.0<br>*< 8.0*
Bilirubin<br>*Direct Bilirubin[14], High*<br>*> 28 days of age*	NA	NA	> ULN	> ULN with lifethreatening consequences (e.g., signs and symptoms of liver failure)
≤ 28 days of age	ULN to ≤ 1 mg/dL	> 1 to ≤ 1.5 mg/dL	> 1.5 to ≤ 2 mg/dL	> 2 mg/dL
Total Bilirubin, High<br>*> 28 days of age*	1.1 to < 1.6 x ULN	1.6 to < 2.6 x ULN	2.6 to < 5.0 x ULN	≥ 5.0 x ULN
≤ 28 days of age	See Appendix A. Total Bilirubin for Term and Preterm Neonates	See Appendix A. Total Bilirubin for Term and Preterm Neonates	See Appendix A. Total Bilirubin for Term and Preterm Neonates	See Appendix A. Total Bilirubin for Term and Preterm Neonates

Calcium, High (mg/dL; *mmol/L*)<br>≥ *7 days of age*	10.6 to < 11.5<br>*2.65* to < *2.88*	11.5 to < 12.5 *2.88* to < *3.13*	12.5 to < 13.5<br>*3.13* to < *3.38*	≥ 13.5<br>≥ *3.38*
< *7 days of age*	11.5 to < 12.4 *2.88* to < *3.10*	12.4 to < 12.9 *3.10* to < *3.23*	12.9 to < 13.5 *3.23* to < *3.38*	≥ 13.5<br>≥ *3.38*

PARAMETER	GRADE 1 MILD	GRADE 2 MODERATE	GRADE 3 SEVERE	GRADE 4 POTENTIALLY LIFE-THREATENING
Calcium (Ionized), High (mg/dL; *mmol/L*)	> ULN to < 6.0 *> ULN to < 1.5*	6.0 to < 6.4 *1.5 to < 1.6*	6.4 to < 7.2 *1.6 to < 1.8*	≥ 7.2 *≥ 1.8*
Calcium, Low (mg/dL; *mmol/L*) *≥ 7 days of age*	7.8 to < 8.4 *1.95* to < *2.10*	7.0 to < 7.8 *1.75* to < *1.95*	6.1 to < 7.0 *1.53* to < *1.75*	< 6.1 *< 1.53*
< 7 days of age	6.5 to < 7.5 *1.63* to < *1.88*	6.0 to < 6.5 *1.50* to < *1.63*	5.50 to < 6.0 *1.38* to < *1.50*	< 5.50 *< 1.38*
Calcium (Ionized), Low (mg/dL; *mmol/L*)	< LLN to 4.0 *< LLN to 1.0*	3.6 to < 4.0 *0.9 to < 1.0*	3.2 to < 3.6 *0.8 to < 0.9*	< 3.2 *< 0.8*
Cardiac Troponin I, High	NA	NA	NA	Levels consistent with myocardial infarction or unstable angina as defined by the local laboratory
Creatine Kinase, High	3 to < 6 x ULN	6 to < 10 x ULN	10 to < 20 x ULN	≥ 20 x ULN
Creatinine, High	1.1 to 1.3 x ULN	> 1.3 to 1.8 x ULN OR Increase of > 0.3 mg/dL above baseline	> 1.8 to < 3.5 x ULN OR Increase of 1.5 to < 2.0 x above baseline	≥ 3.5 x ULN OR Increase of ≥ 2.0 x above baseline
Creatinine Clearance[15] or eGFR, Low *Report only one*	NA	< 90 to 60 ml/min or ml/min/1.73 m^2 OR 10 to < 30% decrease from baseline	< 60 to 30 ml/min or ml/min/1.73 m^2 OR ≥ 30 to < 50% decrease from baseline	< 30 ml/min or ml/min/1.73 m2 OR ≥ 50% decrease from baseline or dialysis needed
Glucose (mg/dL; *mmol/L*) *Fasting, High*	110 to 125 *6.11 to < 6.95*	> 125 to 250 *6.95 to < 13.89*	> 250 to 500 *13.89 to < 27.75*	> 500 *≥ 27.75*
Nonfasting, High	116 to 160 *6.44 to < 8.89*	> 160 to 250 *8.89 to < 13.89*	> 250 to 500 *13.89 to < 27.75*	> 500 *≥ 27.75*
Glucose, Low (mg/dL; *mmol/L*) *≥ 1 month of age*	55 to 64 *3.05 to 3.55*	40 to < 55 *2.22 to < 3.05*	30 to < 40 *1.67 to < 2.22*	< 30 *< 1.67*
< 1 month of age	50 to 54 *2.78 to 3.00*	40 to < 50 *2.22 to < 2.78*	30 to < 40 *1.67 to < 2.22*	< 30 *< 1.67*
Lactate, High	ULN to < 2.0 x ULN without acidosis	≥ 2.0 x ULN without acidosis	Increased lactate with pH < 7.3 without life-threatening consequences	Increased lactate with pH < 7.3 with life-threatening consequences

[15] Use the applicable formula (i.e., Cockroft-Gault in mL/min or Schwatrz in mL/min/1.73m^2).

PARAMETER	GRADE 1<br>MILD	GRADE 2<br>MODERATE	GRADE 3<br>SEVERE	GRADE 4<br>POTENTIALLY LIFE-THREATENING
Lipase, High	1.1 to < 1.5 x ULN	1.5 to < 3.0 x ULN	3.0 to < 5.0 x ULN	≥ 5.0 x ULN
Lipid Disorders (mg/dL; *mmol/L*)<br>*Cholesterol, Fasting, High*<br>*≥18 years of age*	200 to < 240<br>*5.18 to < 6.19*	240 to < 300<br>*6.19 to < 7.77*	≥ 300<br>*≥ 7.77*	NA
< 18 years of age	170 to < 200<br>*4.40 to < 5.15*	200 to < 300<br>*5.15 to < 7.77*	≥ 300<br>*≥ 7.77*	NA
LDL, Fasting, High<br>*≥18 years of age*	130 to < 160<br>*3.37 to < 4.12*	160 to < 190<br>*4.12 to < 4.90*	≥ 190<br>*≥ 4.90*	NA
> 2 to < 18 years of age	110 to < 130<br>*2.85 to < 3.34*	130 to < 190<br>*3.34 to < 4.90*	≥ 190<br>*≥ 4.90*	NA
Triglycerides, Fasting, High	150 to 300<br>*1.71 to 3.42*	>300 to 500<br>*>3.42 to 5.7*	>500 to < 1,000<br>*>5.7 to 11.4*	> 1,000<br>*> 11.4*
Magnesium[16], Low<br>(mEq/L; *mmol/L*)	1.2 to < 1.4<br>*0.60 to < 0.70*	0.9 to < 1.2<br>*0.45 to < 0.60*	0.6 to < 0.9<br>*0.30 to < 0.45*	< 0.6<br>*< 0.30*
Phosphate, Low (mg/dL; *mmol/L*)<br>*> 14 years of age*	2.0 to < LLN<br>*0.81 to < LLN*	1.4 to < 2.0<br>*0.65 to < 0.81*	1.0 to < 1.4<br>*0.32 to < 0.65*	< 1.0<br>*< 0.32*
1 to 14 years of age	3.0 to < 3.5<br>*0.97 to < 1.13*	2.5 to < 3.0<br>*0.81 to < 0.97*	1.5 to < 2.5<br>*0.48 to < 0.81*	< 1.5<br>*< 0.48*
< 1 year of age	3.5 to < 4.5<br>*1.13 to < 1.45*	2.5 to < 3.5<br>*0.81 to < 1.13*	1.5 to < 2.5<br>*0.48 to < 0.81*	< 1.5<br>*< 0.48*
Potassium, High<br>(mEq/L; *mmol/L*)	5.6 to < 6.0<br>*5.6 to < 6.0*	6.0 to < 6.5<br>*6.0 to < 6.5*	6.5 to < 7.0<br>*6.5 to < 7.0*	≥ 7.0<br>*≥ 7.0*
Potassium, Low<br>(mEq/L; *mmol/L*)	3.0 to < 3.4<br>*3.0 to < 3.4*	2.5 to < 3.0<br>*2.5 to < 3.0*	2.0 to < 2.5<br>*2.0 to < 2.5*	< 2.0<br>*< 2.0*
Sodium, High<br>(mEq/L; *mmol/L*)	146 to < 150<br>*146 to < 150*	150 to < 154<br>*150 to < 154*	154 to < 160<br>*154 to < 160*	≥ 160<br>*≥ 160*
Sodium, Low<br>(mEq/L; *mmol/L*)	130 to < 135<br>*130 to < 135*	125 to < 130<br>*125 to < 135*	121 to < 125<br>*121 to < 125*	≤ 120<br>*≤ 120*
Uric Acid, High<br>(mg/dL; *mmol/L*)	7.5 to < 10.0<br>*0.45 to < 0.59*	10.0 to < 12.0<br>*0.59 to < 0.71*	12.0 to < 15.0<br>*0.71 to < 0.89*	≥ 15.0<br>*≥ 0.89*

[16] To convert a magnesium value from mg/dL to mmol/L, laboratories should multiply by 0.4114.

Hematology

PARAMETER	GRADE 1 MILD	GRADE 2 MODERATE	GRADE 3 SEVERE	GRADE 4 POTENTIALLY LIFE-THREATENING
Absolute CD4+ Count, Low (cell/mm^3; *cells/L*) *> 5 years of age (not HIV infected)*	300 to < 400<br>*300 to < 400*	200 to < 300<br>*200 to < 300*	100 to < 200<br>*100 to < 200*	< 100<br>*< 100*
Absolute Lymphocyte Count, Low (cell/mm^3; *cells/L*) *> 5 years of age (not HIV infected)*	600 to < 650<br>*0.600×10^9 to < 0.650×10^9*	500 to < 600<br>*0.500×10^9 to < 0.600×10^9*	350 to < 500<br>*0.350×10^9 to < 0.500×10^9*	< 350<br>*< 0.350×10^9*
Absolute Neutrophil Count (ANC), Low (cells/mm^3; *cells/L*) *> 7 days of age*	800 to 1,000<br>*0.800×10^9 to 1.000×10^9*	600 to 799<br>*0.600×10^9 to 0.799×10^9*	400 to 599<br>*0.400×10^9 to 0.599×10^9*	< 400<br>*< 0.400×10^9*
2 to 7 days of age	1,250 to 1,500<br>*1.250×10^9 to 1.500×10^9*	1,000 to 1,249<br>*1.000×10^9 to 1.249×10^9*	750 to 999<br>*0.750×10^9 to 0.999×10^9*	< 750<br>*< 0.750×10^9*
≤ 1 day of age	4,000 to 5,000<br>*4.000×10^9 to $5.000 \times 10_9$*	3,000 to 3,999<br>*3.000×10^9 to 3.999×10^9*	1,500 to 2,999<br>*1.500×10^9 to 2.999×10^9*	< 1,500<br>*< 1.500×10^9*
Fibrinogen, Decreased (mg/dL; *g/L*)	100 to < 200<br>*1.00 to < 2.00*<br>OR<br>0.75 to < 1.00 x LLN	75 to < 100<br>*0.75 to < 1.00*<br>OR<br>≥ 0.50 to < 0.75 x LLN	50 to < 75<br>*0.50 to < 0.75*<br>OR<br>0.25 to < 0.50 x LLN	< 50<br>*< 0.50*<br>OR<br>< 0.25 x LLN<br>OR Associated with gross bleeding
Hemoglobin[17], Low (g/dL; *mmol/L*)[18] *≥ 13 years of age (male only)*	10.0 to 10.9<br>*6.19 to 6.76*	9.0 to < 10.0<br>*5.57 to < 6.19*	7.0 to < 9.0<br>*4.34 to < 5.57*	< 7.0<br>*< 4.34*
≥ 13 years of age (female only)	9.5 to 10.4<br>*5.88 to 6.48*	8.5 to < 9.5<br>*5.25 to < 5.88*	6.5 to < 8.5<br>*4.03 to < 5.25*	< 6.5<br>*< 4.03*

[17] Male and female sex are defined as sex at birth.
[18] The conversion factor used to convert g/dL to mmol/L is 0.6206 and is the most commonly used conversion factor. For grading hemoglobin results obtained by an analytic method with a conversion factor other than 0.6206, the result must be converted to g/dL using the appropriate conversion factor for the particular laboratory.

PARAMETER	GRADE 1 MILD	GRADE 2 MODERATE	GRADE 3 SEVERE	GRADE 4 POTENTIALLY LIFE-THREATENING
57 days of age to < 13 years of age (male and female)	9.5 to 10.4 *5.88 to 6.48*	8.5 to < 9.5 *5.25 to < 5.88*	6.5 to < 8.5 *4.03 to < 5.25*	< 6.5 *< 4.03*
36 to 56 days of age (male and female)	8.5 to 9.6 *5.26 to 5.99*	7.0 to < 8.5 *4.32 to < 5.26*	6.0 to < 7.0 *3.72 to < 4.32*	< 6.0 *< 3.72*
22 to 35 days of age (male and female)	9.5 to 11.0 *5.88 to 6.86*	8.0 to < 9.5 *4.94 to < 5.88*	6.7 to < 8.0 *4.15 to < 4.94*	< 6.7 *< 4.15*
8 to < 21 days of age (male and female)	11.0 to 13.0 *6.81 to 8.10*	9.0 to < 11.0 *5.57 to < 6.81*	8.0 to < 9.0 *4.96 to < 5.57*	< 8.0 *< 4.96*
< 7 days of age (male and female)	13.0 to 14.0 *8.05 to 8.72*	10.0 to < 13.0 *6.19 to < 8.05*	9.0 to < 10.0 *5.59 to < 6.19*	< 9.0 *< 5.59*
INR, High (not on anticoagulation therapy)	1.1 to < 1.5 x ULN	1.5 to < 2.0 x ULN	2.0 to < 3.0 x ULN	≥ 3.0 x ULN
Methemoglobin (% hemoglobin)	5.0 to < 10.0%	10.0 to < 15.0%	15.0 to < 20.0%	≥ 20.0%
PTT, High (not on anticoagulation therapy)	1.1 to < 1.66 x ULN	1.66 to < 2.33 x ULN	2.33 to < 3.00 x ULN	≥ 3.00 x ULN
Platelets, Decreased (cells/mm^3; *cells/L*)	100,000 to < 124,999 *100.000 x 10^9 to < 124.999 x 10^9*	50,000 to < 100,000 *50.000 x 10^9 to < 100.000 x 10^9*	25,000 to < 50,000 *25.000 x 10^9 to < 50.000 x 10^9*	< 25,000 *< 25.000 x 10^9*
PT, High (not on anticoagulation therapy	1.1 to < 1.25 x ULN	1.25 to < 1.50 x ULN	1.50 to < 3.00 x ULN	≥ 3.00 x ULN
WBC, Decreased (cells/mm^3; *cells/L*) *> 7 days of age*	2,000 to 2,499 *2.000 x 10^9 to 2.499 x 10^9*	1,500 to 1,999 *1.500 x 10^9 to 1.999 x 10^9*	1,000 to 1,499 *1.000 x 10^9 to 1.499 x 10^9*	< 1,000 *< 1.000 x 10^9*
< 7 days of age	5,500 to 6,999 *5.500 x 10^9 to 6.999 x 10^9*	4,000 to 5,499 *4.000 x 10^9 to 5.499 x 10^9*	2,500 to 3,999 *2.500 x 10^9 to 3.999 x 10^9*	< 2,500 *< 2.500 x 10^9*

Urinalysis

PARAMETER	GRADE 1	GRADE 2	GRADE 3	GRADE 4
Glycosuria (random collection tested by dipstick)	Trace to 1+ or ≤ 250 mg	2+ or > 250 to < 500 mg	> 2+ or > 500 mg	NA
Hematuria (not to be reported based on dipstick findings or on blood believed to be of menstrual origin)	6 to < 10 RBCs per high power field	≥ 10 RBCs per high power field	Gross, with or without clots OR<br>With RBC casts OR<br>Intervention indicated	Life-threatening consequences
Proteinuria (random collection tested by dipstick)	1+	2+	3+ or higher	NA

APPENDIX F CTCAE TABLE

For the first 24 hours after IP infusion, any infusion related reactions, including cytokine release syndrome, will be graded using the Common Terminology Criteria for Adverse Events (CTCAE) v4.03: June 14, 2010 (see SOM for details).

CTCAE4.03
Adapted from Common Terminology Criteria for Adverse Events (CTCAE)
Version 4.0 Published: May 28, 2009 (v4.03: June 14, 2010)

U.S. DEPARTMENT OF HEALTH AND HUMAN SERVICES National Institutes of Health National Cancer Institute

Quick Reference

The NCI Common Terminology Criteria for Adverse Events is a descriptive terminology which can be utilized for Adverse Event (AE) reporting. A grading (severity) scale is provided for each AE term.

Components and Organization

SOC

System Organ Class, the highest level of the MedDRA hierarchy, is identified by anatomical or physiological system, etiology, or purpose (e.g., SOC Investigations for laboratory test results). CTCAE terms are grouped by MedDRA Primary SOCs. Within each SOC, AEs are listed and accompanied by descriptions of severity (Grade).

CTCAE Terms

An Adverse Event (AE) is any unfavorable and unintended sign (including an abnormal laboratory finding), symptom, or disease temporally associated with the use of a medical treatment or procedure that may or may not be considered related to the medical treatment or procedure. An AE is a term that is a unique representation of a specific event used for medical documentation and scientific analyses. Each CTCAE v4.0 term is a MedDRA LLT (Lowest Level Term).

Definitions

A brief definition is provided to clarify the meaning of each AE term.

Grades

Grade refers to the severity of the AE. The CTCAE displays Grades 1 through 5 with unique clinical descriptions of severity for each AE based on this general guideline:

Grade 1	Mild; asymptomatic or mild symptoms; clinical or diagnostic observations only; intervention not indicated.
Grade 2	Moderate; minimal, local or noninvasive intervention indicated; limiting age-appropriate instrumental ADL*.

Grade 3	Severe or medically significant but not immediately life-threatening; hospitalization or prolongation of hospitalization indicated; disabling; limiting self care ADL**.
Grade 4	Life-threatening consequences; urgent intervention indicated.
Grade 5	Death related to AE.

A Semi-colon indicates 'or' within the description of the grade.

A single dash (-) indicates a grade is not available.

Not all Grades are appropriate for all AEs. Therefore, some AEs are listed with fewer than five options for Grade selection.

Grade 5

Grade 5 (Death) is not appropriate for some AEs and therefore is not an option.

Activities of Daily Living (ADL)

*Instrumental ADL refer to preparing meals, shopping for groceries or clothes, using the telephone, managing money, etc.

**Self care ADL refer to bathing, dressing and undressing, feeding self, using the toilet, taking medications, and not bedridden.

† CTCAE v4.0 incorporates certain elements of the MedDRA terminology. For further details on MedDRA refer to the MedDRA MSSO Web site (http://www.meddramsso.com).

Blood and lymphatic system disorders	Grade				
Adverse Event	1	2	3	4	5
Anemia	Hemoglobin (Hgb) <LLN - 10.0 g/dL; <LLN - 6.2 mmol/L; <LLN - 100 g/L	Hgb <10.0 - 8.0 g/dL; <6.2 - 4.9 mmol/L; <100 - 80g/L	Hgb <8.0 g/dL; <4.9 mmol/L; <80 g/L; transfusion indicated	Life-threatening consequences; urgent intervention indicated	Death
Bone marrow hypocellular	Mildly hypocellular or <=25% reduction from normal cellularity for age	Moderately hypocellular or >25 - <50% reduction from normal cellularity for age	Severely hypocellular or >50 - <=75% reduction cellularity from normal for age	Aplastic persistent for longer than 2 weeks	Death
Disseminated intravascular coagulation	-	Laboratory findings with no bleeding	Laboratory findings and bleeding	Life-threatening consequences; urgent intervention indicated	Death
Febrile neutropenia	-	-	ANC <1000/mm3 with a single temperature of >38.3 degrees C (101 degrees F) or a sustained temperature of >=38 degrees C (100.4 degrees F) for more than one hour	Life-threatening consequences; urgent intervention indicated	Death
Hemolysis	Laboratory evidence of hemolysis only (e.g., direct antiglobulin test; DAT; Coombs'; schistocytes; decreased haptoglobin)	Evidence of hemolysis and >=2 gm decrease in hemoglobin	Transfusion or medical intervention indicated (e.g., steroids)	Life-threatening consequences; urgent intervention indicated	Death
Hemolytic uremic syndrome	Evidence of RBC destruction (schistocytosis) without clinical consequences	-	Laboratory findings with clinical consequences (e.g., renal insufficiency, petechiae)	Life-threatening consequences, (e.g., CNS hemorrhage or thrombosis/embolism or renal failure)	Death
Leukocytosis	-	-	>100,000/mm3	Clinical manifestations of leucostasis; urgent intervention indicated	Death
Lymph node pain	Mild pain	Moderate pain; limiting instrumental ADL	Severe pain; limiting self care ADL	-	
Spleen disorder	Incidental findings (e.g., Howell-Jolly bodies); mild degree of thrombocytosis and leukocytosis	Prophylactic antibiotics indicated	-	Life-threatening consequences; urgent intervention indicated	Death
Thrombotic thrombocytopenic purpura	Evidence of RBC destruction (schistocytosis) without clinical consequences	-	Laboratory findings with clinical consequences (e.g., renal insufficiency, petechiae)	Life-threatening consequences, (e.g., CNS hemorrhage or thrombosis/embolism or renal failure)	Death

Blood and lymphatic system disorders - Other, specify	Asymptomatic or mild symptoms; clinical or diagnostic observations only; intervention not indicated	Moderate; minimal, local or noninvasive intervention indicated; limiting age-appropriate instrumental ADL	Severe or medically significant but not immediately life-threatening; hospitalization or prolongation of existing hospitalization indicated; disabling; limiting self care ADL	Life-threatening consequences; urgent intervention indicated	Death
Cardiac Disorders	Grade				
Adverse Event	1	2	3	4	5
Acute coronary syndrome	-	Symptomatic, progressive angina; cardiac enzymes normal; hemodynamically stable	Symptomatic, unstable angina and/or acute myocardial infarction, cardiac enzymes abnormal, hemodynamically stable	Symptomatic, unstable angina and/or acute myocardial infarction, cardiac enzymes abnormal, hemodynamically unstable	Death
Aortic valve disease	Asymptomatic valvular thickening with or without mild valvular regurgitation or stenosis by imaging	Asymptomatic; moderate regurgitation or stenosis by imaging	Symptomatic; severe regurgitation or stenosis by imaging; symptoms controlled with medical intervention	Life-threatening consequences; urgent intervention indicated (e.g., valve replacement, valvuloplasty)	Death
Asystole	Periods of asystole; non-urgent medical management indicated	-	-	Life-threatening consequences; urgent intervention indicated	Death
Atrial fibrillation	Asymptomatic, intervention not indicated	Non-urgent medical intervention indicated	Symptomatic and incompletely controlled medically, or controlled with device (e.g., pacemaker), or ablation	Life-threatening consequences; urgent intervention indicated	Death
Atrial flutter	Asymptomatic, intervention not indicated	Non-urgent medical intervention indicated	Symptomatic and incompletely controlled medically, or controlled with device (e.g., pacemaker), or ablation	Life-threatening consequences; urgent intervention indicated	Death
Atrioventricular block complete	-	Non-urgent intervention indicated	Symptomatic and incompletely controlled medically, or controlled with device (e.g., pacemaker)	Life-threatening consequences; urgent intervention indicated	Death
Atrioventricular block first degree	Asymptomatic, intervention not indicated	Non-urgent intervention indicated	-	-	-
Cardiac arrest	-	-	-	Life-threatening consequences; urgent intervention indicated	Death
Chest pain - cardiac	Mild pain	Moderate pain; limiting instrumental ADL	Pain at rest; limiting self care ADL	-	-
Conduction disorder	Mild symptoms; intervention not indicated	Moderate symptoms	Severe symptoms; intervention indicated	Life-threatening consequences; urgent intervention indicated	Death
Constrictive pericarditis	-	-	Symptomatic heart failure or other cardiac symptoms, responsive to intervention	Refractory heart failure or other poorly controlled cardiac symptoms	Death

Heart failure	Asymptomatic with laboratory (e.g., BNP [B-Natriuretic Peptide]) or cardiac imaging abnormalities	Symptoms with mild to moderate activity or exertion	Severe with symptoms at rest or with minimal activity or exertion; intervention indicated	Life-threatening consequences; urgent intervention indicated (e.g., continuous IV therapy or mechanical hemodynamic support)	Death
Left ventricular systolic dysfunction	-	-	Symptomatic due to drop in ejection fraction responsive to intervention	Refractory or poorly controlled heart failure due to drop in ejection fraction; intervention such as ventricular assist device, intravenous vasopressor support, or heart transplant indicated	Death
Mitral valve disease	Asymptomatic valvular thickening with or without mild valvular regurgitation or stenosis by imaging	Asymptomatic; moderate regurgitation or stenosis by imaging	Symptomatic; severe regurgitation or stenosis by imaging; symptoms controlled with medical intervention	Life-threatening consequences; urgent intervention indicated (e.g., valve replacement, valvuloplasty)	Death
Mobitz (type) II atrioventricular block	Asymptomatic, intervention not indicated	Symptomatic; medical intervention indicated	Symptomatic and incompletely controlled medically, or controlled with device (e.g., pacemaker)	Life-threatening consequences; urgent intervention indicated	Death
Mobitz type I	Asymptomatic, intervention not indicated	Symptomatic; medical intervention indicated	Symptomatic and incompletely controlled medically, or controlled with device (e.g., pacemaker)	Life-threatening consequences; urgent intervention indicated	Death
Myocardial infarction	-	Asymptomatic and cardiac enzymes minimally abnormal and no evidence of ischemic ECG changes	Severe symptoms; cardiac enzymes abnormal; hemodynamically stable; ECG changes consistent with infarction	Life-threatening consequences; hemodynamically unstable	Death
Myocarditis	Asymptomatic with laboratory (e.g., BNP [B-Natriuretic Peptide]) or cardiac imaging abnormalities	Symptoms with mild to moderate activity or exertion	Severe with symptoms at rest or with minimal activity or exertion; intervention indicated	Life-threatening consequences; urgent intervention indicated (e.g., continuous IV therapy or mechanical hemodynamic support)	Death
Palpitations	Mild symptoms; intervention not indicated	Intervention indicated	-	-	-
Paroxysmal atrial tachycardia	Asymptomatic, intervention not indicated	Symptomatic; medical management indicated	IV medication indicated	Life-threatening consequences; incompletely controlled medically; cardioversion indicated	Death
Pericardial effusion	-	Asymptomatic effusion size small to moderate	Effusion with physiologic consequences	Life-threatening consequences; urgent intervention indicated	Death
Pericardial tamponade	-	-	-	Life-threatening consequences; urgent intervention indicated	Death
Pericarditis	Asymptomatic, ECG or physical findings (e.g., rub) consistent with pericarditis	Symptomatic pericarditis (e.g., chest pain)	Pericarditis with physiologic consequences (e.g., pericardial constriction)	Life-threatening consequences; urgent intervention indicated	Death

Pulmonary valve disease	Asymptomatic valvular thickening with or without mild valvular regurgitation or stenosis by imaging	Asymptomatic; moderate regurgitation or stenosis by imaging	Symptomatic; severe regurgitation or stenosis by imaging; symptoms controlled with medical intervention	Life-threatening consequences; urgent intervention indicated (e.g., valve replacement, valvuloplasty)	Death
Restrictive cardiomyopathy	-	-	Symptomatic heart failure or other cardiac symptoms, responsive to intervention	Refractory heart failure or other poorly controlled cardiac symptoms	Death
Right ventricular dysfunction	Asymptomatic with laboratory (e.g., BNP [B-Natriuretic Peptide]) or cardiac imaging abnormalities	Symptoms with mild to moderate activity or exertion	Severe symptoms, associated with hypoxemia, right heart failure; oxygen indicated	Life-threatening consequences; urgent intervention indicated (e.g., ventricular assist device); heart transplant indicated	Death
Sick sinus syndrome	Asymptomatic, intervention not indicated	Non-urgent intervention indicated	Severe, medically significant; medical intervention indicated	Life-threatening consequences; urgent intervention indicated	Death
Sinus bradycardia	Asymptomatic, intervention not indicated	Symptomatic, medical intervention indicated	Severe, medically significant, medical intervention indicated	Life-threatening consequences; urgent intervention indicated	Death
Sinus tachycardia	Asymptomatic, intervention not indicated	Symptomatic; non-urgent medical intervention indicated	Urgent medical intervention indicated	-	-
Supraventricular tachycardia	Asymptomatic, intervention not indicated	Non-urgent medical intervention indicated	Medical intervention indicated	Life-threatening consequences; urgent intervention indicated	Death
Tricuspid valve disease	Asymptomatic valvular thickening with or without mild valvular regurgitation or stenosis	Asymptomatic; moderate regurgitation or stenosis by imaging	Symptomatic; severe regurgitation or stenosis; symptoms controlled with medical intervention	Life-threatening consequences; urgent intervention indicated (e.g., valve replacement, valvuloplasty)	Death
Ventricular arrhythmia	Asymptomatic, intervention not indicated	Non-urgent medical intervention indicated	Medical intervention indicated	Life-threatening consequences; hemodynamic compromise; urgent intervention indicated	Death
Ventricular fibrillation	-	-	-	Life-threatening consequences; hemodynamic compromise; urgent intervention indicated	Death
Ventricular tachycardia	-	Non-urgent medical intervention indicated	Medical intervention indicated	Life-threatening consequences; hemodynamic compromise; urgent intervention indicated	Death
Wolff-Parkinson-White syndrome	Asymptomatic, intervention not indicated	Non-urgent medical intervention indicated	Symptomatic and incompletely controlled medically or controlled with procedure	Life-threatening consequences; urgent intervention indicated	Death
Cardiac disorders - Other, specify	Asymptomatic or mild symptoms; clinical or diagnostic observations only; intervention not indicated	Moderate; minimal, local or noninvasive intervention indicated; limiting age-appropriate instrumental ADL	Severe or medically significant but not immediately life-threatening; hospitalization or prolongation of existing hospitalization indicated; disabling; limiting self care ADL	Life-threatening consequences; urgent intervention indicated	Death
Congenital, familial and	Grade				

genetic disorders					
Adverse Event	1	2	3	4	5
Congenital, familial and genetic disorders - Other, specify	Asymptomatic or mild symptoms; clinical or diagnostic observations only; intervention not indicated	Moderate; minimal, local or noninvasive intervention indicated; limiting age-appropriate instrumental ADL	Severe or medically significant but not immediately life-threatening; hospitalization or prolongation of existing hospitalization indicated; disabling; limiting self care ADL	Life-threatening consequences; urgent intervention indicated	Death
Ear and labyrinth disorders	Grade				
Adverse Event	1	2	3	4	5
Ear pain	Mild pain	Moderate pain; limiting instrumental ADL	Severe pain; limiting self care ADL	-	-
External ear inflammation	External otitis with erythema or dry desquamation	External otitis with moist desquamation, edema, enhanced cerumen or discharge; tympanic membrane perforation; tympanostomy	External otitis with mastoiditis; stenosis or osteomyelitis; necrosis of soft tissue or bone	Urgent operative intervention indicated	Death
External ear pain	Mild pain	Moderate pain; limiting instrumental ADL	Severe pain; limiting self care ADL	-	-
Hearing impaired	Adults Enrolled on a Monitoring Program (on a 1, 2, 4, 3, 6 and 8 kHz audiogram): Threshold shift of 15 - 25 dB averaged at 2 contiguous test frequencies in at least one ear or subjective change in the absence of a Grade 1 Threshold shift. Pediatric (on a 1, 2, 4, 3, 6 and 8 kHz audiogram): Threshold shift >20 dB at 8 kHz in at least one ear.	Adult enrolled in monitoring program (on a 1, 2, 3, 4, 6 and 8 kHz audiogram): Threshold shift of >25 dB averaged at 2 contiguous test frequencies in at least one ear. Adult not enrolled in monitoring program: hearing loss but hearing aid or intervention not indicated; limiting instrumental ADL. Pediatric (on a 1, 2, 3, 4, 6 and 8 kHz audiogram): Threshold shift >20 dB at 4 kHz and above in at least one ear.	Adult enrolled in monitoring program (on a 1, 2, 3, 4, 6 and 8 kHz audiogram): Threshold shift of >25 dB averaged at 3 contiguous test frequencies in at least one ear; therapeutic intervention indicated. Adult: Not enrolled in monitoring program: Hearing loss with hearing aid or intervention indicated; limiting self care ADL. Pediatric (on a 1, 2, 3, 4, 6 and 8kHz audiogram): hearing loss sufficient to indicate therapeutic intervention, including hearing aids): Threshold shift >20 dB at 3 kHz and above in at least one ear ; additional speech-language related services indicated.	Adults: Profound bilateral hearing loss (Threshold >80 dB HL at 2 kHz and above); nonservicable hearing Pediatric: Audiologic indication for cochlear implant and additional speech-language related services indicated.	-
Middle ear inflammation	Serous otitis	Serous otitis, medical intervention indicated	Mastoiditis; necrosis of canal soft tissue or bone	Life-threatening consequences; urgent intervention indicated	Death
Tinnitus	Mild symptoms; intervention not indicated	Moderate symptoms; limiting instrumental ADL	Severe symptoms; limiting self care ADL	-	-

Vertigo	Mild symptoms	Moderate symptoms; limiting instrumental ADL	Severe symptoms; limiting self care ADL	-	-
Vestibular disorder	-	Symptomatic; limiting instrumental ADL	Severe symptoms; limiting self care ADL	-	-
Ear and labyrinth disorders - Other, specify	Asymptomatic or mild symptoms; clinical or diagnostic observations only; intervention not indicated	Moderate; minimal, local or noninvasive intervention indicated; limiting age-appropriate instrumental ADL	Severe or medically significant but not immediately life-threatening; hospitalization or prolongation of existing hospitalization indicated; disabling; limiting self care ADL	Life-threatening consequences; urgent intervention indicated	Death
Endocrine disorders	Grade				
Adverse Event	1	2	3	4	5
Adrenal insufficiency	Asymptomatic; clinical or diagnostic observations only; intervention not indicated	Moderate symptoms; medical intervention indicated	Severe symptoms; hospitalization indicated	Life-threatening consequences; urgent intervention indicated	Death
Cushingoid	Mild symptoms; intervention not indicated	Moderate symptoms; medical intervention indicated	Severe symptoms, medical intervention or hospitalization indicated	-	-
Delayed puberty	-	No breast development by age 13 yrs for females; testes volume of <3 cc or no Tanner Stage 2 development by age 14.5 yrs for males	No breast development by age 14 yrs for females; no increase in testes volume or no Tanner Stage 2 by age 16 yrs for males; hormone replacement indicated	-	-
Growth accelerated	-	>= +2 SD (standard deviation) above mid parental height or target height	-	-	-
Hyperparathyroidism	Mild symptoms; intervention not indicated	Moderate symptoms; medical intervention indicated	-	-	-
Hyperthyroidism	Asymptomatic; clinical or diagnostic observations only; intervention not indicated	Symptomatic; thyroid suppression therapy indicated; limiting instrumental ADL	Severe symptoms; limiting self care ADL; hospitalization indicated	Life-threatening consequences; urgent intervention indicated	Death
Hypoparathyroidism	Asymptomatic; clinical or diagnostic observations only; intervention not indicated	Moderate symptoms; medical intervention indicated	Severe symptoms; medical intervention or hospitalization indicated	Life-threatening consequences; urgent intervention indicated	Death
Hypothyroidism	Asymptomatic; clinical or diagnostic observations only; intervention not indicated	Symptomatic; thyroid replacement indicated; limiting instrumental ADL	Severe symptoms; limiting self care ADL; hospitalization indicated	Life-threatening consequences; urgent intervention indicated	Death
Precocious puberty	Physical signs of puberty with no biochemical markers for females <8 years and males <9 years	Physical signs and biochemical markers of puberty for females <8 years and males <9 years	-	-	-

Virilization	Mild symptoms; intervention not indicated	Moderate symptoms; medical intervention indicated	-	-	-
Endocrine disorders - Other, specify	Asymptomatic or mild symptoms; clinical or diagnostic observations only; intervention not indicated	Moderate; minimal, local or noninvasive intervention indicated; limiting age-appropriate instrumental ADL	Severe or medically significant but not immediately life-threatening; hospitalization or prolongation of existing hospitalization indicated; disabling; limiting self care ADL	Life-threatening consequences; urgent intervention indicated	Death
Eye Disorders	Grade				
Adverse Event	1	2	3	4	5
Blurred vision	Intervention not indicated	Symptomatic; limiting instrumental ADL	Limiting self care ADL	-	-
Cataract	Asymptomatic; clinical or diagnostic observations only; intervention not indicated	Symptomatic; moderate decrease in visual acuity (20/40 or better)	Symptomatic with marked decrease in visual acuity (worse than 20/40 but better than 20/200); operative intervention indicated (e.g., cataract surgery)	Blindness (20/200 or worse) in the affected eye	-
Conjunctivitis	Asymptomatic or mild symptoms; intervention not indicated	Symptomatic; topical intervention indicated (e.g., antibiotics); limiting instrumental ADL	Limiting self care ADL	-	-
Corneal ulcer	-	Symptomatic; medical intervention indicated (e.g., topical agents); limiting instrumental ADL	Limiting self care ADL; declining vision (worse than 20/40 but better than 20/200)	Perforation or blindness (20/200 or worse) in the affected eye	-
Dry eye	Asymptomatic; clinical or diagnostic observations only; mild symptoms relieved by lubricants	Symptomatic; multiple agents indicated; limiting instrumental ADL	Decrease in visual acuity (<20/40); limiting self care ADL	-	-
Extraocular muscle paresis	Asymptomatic; clinical or diagnostic observations only	Symptomatic; limiting instrumental ADL	Limiting self care ADL; disabling	-	-
Eye pain	Mild pain	Moderate pain; limiting instrumental ADL	Severe pain; limiting self care ADL	-	-
Eyelid function disorder	Asymptomatic; clinical or diagnostic observations only; intervention not indicated	Symptomatic; nonoperative intervention indicated; limiting instrumental ADL	Limiting self care ADL; operative intervention indicated	-	-
Flashing lights	Symptomatic but not limiting ADL	Limiting instrumental ADL	Limiting self care ADL	-	-
Floaters	Symptomatic but not limiting ADL	Limiting instrumental ADL	Limiting self care ADL	-	-

Glaucoma	Elevated intraocular pressure (EIOP) with single topical agent for intervention; no visual field deficit	EIOP causing early visual field deficits; multiple topical or oral agents indicated; limiting instrumental ADL	EIOP causing marked visual field deficits (e.g., involving both superior and inferior visual fields); operative intervention indicated; limiting self care ADL	Blindness (20/200 or worse) in the affected eye	-
Keratitis	-	Symptomatic; medical intervention indicated (e.g., topical agents); limiting instrumental ADL	Decline in vision (worse than 20/40 but better than 20/200); limiting self care ADL	Perforation or blindness (20/200 or worse) in the affected eye	-
Night blindness	Symptomatic but not limiting ADL	Limiting instrumental ADL	Limiting self care ADL	Blindness (20/200 or worse) in the affected eye	-
Optic nerve disorder	Asymptomatic; clinical or diagnostic observations only	Limiting vision of the affected eye (20/40 or better)	Limiting vision in the affected eye (worse than 20/40 but better than 20/200)	Blindness (20/200 or worse) in the affected eye	-
Papilledema	Asymptomatic; no visual field defects	Symptomatic decline in vision; visual field defect present sparing the central 20 degrees	Marked visual field defect (worse than 20/40 but better than 20/200)	Blindness (20/200 or worse) in the affected eye	-
Photophobia	Symptomatic but not limiting ADL	Limiting instrumental ADL	Limiting self care ADL	-	-
Retinal detachment	Asymptomatic	Exudative and visual acuity 20/40 or better	Rhegmatogenous or exudative detachment; operative intervention indicated; decline in vision (worse than 20/40 but better than 20/200)	Blindness (20/200 or worse) in the affected eye	-
Retinal tear	-	Laser therapy or pneumopexy indicated	Vitroretinal surgical repair indicated	Blindness (20/200 or worse) in the affected eye	-
Retinal vascular disorder	-	Topical medication indicated	Intravitreal medication; operative intervention indicated	-	-
Retinopathy	Asymptomatic; clinical or diagnostic observations only	Symptomatic with moderate decrease in visual acuity (20/40 or better); limiting instrumental ADL	Symptomatic with marked decrease in visual acuity (worse than 20/40); disabling; limiting self care ADL	Blindness (20/200 or worse) in the affected eye	-
Scleral disorder	Asymptomatic; clinical or diagnostic observations only	Symptomatic, limiting instrumental ADL; moderate decrease in visual acuity (20/40 or better)	Symptomatic, limiting self care ADL; marked decrease in visual acuity (worse than 20/40)	Blindness (20/200 or worse) in the affected eye	-
Uveitis	Asymptomatic; clinical or diagnostic observations only	Anterior uveitis; medical intervention indicated	Posterior or pan-uveitis	Blindness (20/200 or worse) in the affected eye	-
Vitreous hemorrhage	Asymptomatic or mild symptoms; clinical or diagnostic observations only	Symptomatic; limiting instrumental ADL	Limiting self care ADL; vitrectomy indicated	Blindness (20/200 or worse) in the affected eye	-
Watering eyes	Intervention not indicated	Intervention indicated	Operative intervention indicated	-	-

Eye disorders - Other, specify	Asymptomatic or mild symptoms; clinical or diagnostic observations only; intervention not indicated	Moderate; minimal, local or noninvasive intervention indicated; limiting age-appropriate instrumental ADL	Severe or medically significant but not immediately sight-threatening; hospitalization or prolongation of existing hospitalization indicated; disabling; limiting self care ADL	Sight-threatening consequences; urgent intervention indicated; blindness (20/200 or worse) in the affected eye	-
Gastrointestinal disorders	Grade				
Adverse Event	1	2	3	4	5
Abdominal distension	Asymptomatic; clinical or diagnostic observations only; intervention not indicated	Symptomatic; limiting instrumental ADL	Severe discomfort; limiting self care ADL	-	-
Abdominal pain	Mild pain	Moderate pain; limiting instrumental ADL	Severe pain; limiting self care ADL	-	-
Anal fistula	Asymptomatic; clinical or diagnostic observations only; intervention not indicated	Symptomatic; altered GI function	Severely altered GI function; tube feeding, TPN or hospitalization indicated; elective operative intervention indicated	Life-threatening consequences; urgent intervention indicated	Death
Anal hemorrhage	Mild; intervention not indicated	Moderate symptoms; medical intervention or minor cauterization indicated	Transfusion, radiologic, endoscopic, or elective operative intervention indicated	Life-threatening consequences; urgent intervention indicated	Death
Anal mucositis	Asymptomatic or mild symptoms; intervention not indicated	Symptomatic; medical intervention indicated; limiting instrumental ADL	Severe symptoms; limiting self care ADL	Life-threatening consequences; urgent intervention indicated	Death
Anal necrosis	-	-	TPN or hospitalization indicated; radiologic, endoscopic, or operative intervention indicated	Life-threatening consequences; urgent operative intervention indicated	Death
Anal pain	Mild pain	Moderate pain; limiting instrumental ADL	Severe pain; limiting self care ADL	-	-
Anal stenosis	Asymptomatic; clinical or diagnostic observations only; intervention not indicated	Symptomatic; altered GI function	Symptomatic and severely altered GI function; non-emergent operative intervention indicated; TPN or hospitalization indicated	Life-threatening consequences; urgent operative intervention indicated	Death
Anal ulcer	Asymptomatic; clinical or diagnostic observations only; intervention not indicated	Symptomatic; altered GI function	Severely altered GI function; TPN indicated; elective operative or endoscopic intervention indicated; disabling	Life-threatening consequences; urgent operative intervention indicated	Death
Ascites	Asymptomatic; clinical or diagnostic observations only; intervention not indicated	Symptomatic; medical intervention indicated	Severe symptoms; invasive intervention indicated	Life-threatening consequences; urgent operative intervention indicated	Death
Bloating	No change in bowel function or oral intake	Symptomatic, decreased oral intake; change in bowel function	-	-	-

Cecal hemorrhage	Mild; intervention not indicated	Moderate symptoms; medical intervention or minor cauterization indicated	Transfusion, radiologic, endoscopic, or elective operative intervention indicated	Life-threatening consequences; urgent intervention indicated	Death
Cheilitis	Asymptomatic; clinical or diagnostic observations only; intervention not indicated	Moderate symptoms; limiting instrumental ADL	Severe symptoms; limiting self care ADL; intervention indicated	-	-
Colitis	Asymptomatic; clinical or diagnostic observations only; intervention not indicated	Abdominal pain; mucus or blood in stool	Severe abdominal pain; change in bowel habits; medical intervention indicated; peritoneal signs	Life-threatening consequences; urgent intervention indicated	Death
Colonic fistula	Asymptomatic; clinical or diagnostic observations only; intervention not indicated	Symptomatic; altered GI function	Severely altered GI function; bowel rest, TPN or hospitalization indicated; elective operative intervention indicated	Life-threatening consequences; urgent intervention indicated	Death
Colonic hemorrhage	Mild; intervention not indicated	Moderate symptoms; medical intervention or minor cauterization indicated	Transfusion, radiologic, endoscopic, or elective operative intervention indicated	Life-threatening consequences; urgent intervention indicated	Death
Colonic obstruction	Asymptomatic; clinical or diagnostic observations only; intervention not indicated	Symptomatic; altered GI function	Hospitalization indicated; elective operative intervention indicated; disabling	Life-threatening consequences; urgent operative intervention indicated	Death
Colonic perforation	-	Symptomatic; medical intervention indicated	Severe symptoms; elective operative intervention indicated	Life-threatening consequences; urgent intervention indicated	Death
Colonic stenosis	Asymptomatic; clinical or diagnostic observations only; intervention not indicated	Symptomatic; altered GI function	Severely altered GI function; tube feeding or hospitalization indicated; elective operative intervention indicated	Life-threatening consequences; urgent operative intervention indicated	Death
Colonic ulcer	Asymptomatic; clinical or diagnostic observations only; intervention not indicated	Symptomatic; altered GI function	Severely altered GI function; TPN indicated; elective operative or endoscopic intervention indicated; disabling	Life-threatening consequences; urgent operative intervention indicated	Death
Constipation	Occasional or intermittent symptoms; occasional use of stool softeners, laxatives, dietary modification, or enema	Persistent symptoms with regular use of laxatives or enemas; limiting instrumental ADL	Obstipation with manual evacuation indicated; limiting self care ADL	Life-threatening consequences; urgent intervention indicated	Death
Dental caries	One or more dental caries, not involving the root	Dental caries involving the root	Dental caries resulting in pulpitis or periapical abscess or resulting in tooth loss	-	-
Diarrhea	Increase of <4 stools per day over baseline; mild increase in ostomy output compared to baseline	Increase of 4 - 6 stools per day over baseline; moderate increase in ostomy output compared to baseline	Increase of >=7 stools per day over baseline; incontinence; hospitalization indicated; severe increase in ostomy output compared to baseline; limiting self care ADL	Life-threatening consequences; urgent intervention indicated	Death

Dry mouth	Symptomatic (e.g., dry or thick saliva) without significant dietary alteration; unstimulated saliva flow >0.2 ml/min	Moderate symptoms; oral intake alterations (e.g., copious water, other lubricants, diet limited to purees and/or soft, moist foods); unstimulated saliva 0.1 to 0.2 ml/min	Inability to adequately aliment orally; tube feeding or TPN indicated; unstimulated saliva <0.1 ml/min	-	-
Duodenal fistula	Asymptomatic; clinical or diagnostic observations only; intervention not indicated	Symptomatic; altered GI function	Severely altered GI function; tube feeding, TPN or hospitalization indicated; elective operative intervention indicated	Life-threatening consequences; urgent intervention indicated	Death
Duodenal hemorrhage	Mild; intervention not indicated	Moderate symptoms; medical intervention or minor cauterization indicated	Transfusion, radiologic, endoscopic, or elective operative intervention indicated	Life-threatening consequences; urgent intervention indicated	Death
Duodenal obstruction	Asymptomatic; clinical or diagnostic observations only; intervention not indicated	Symptomatic; altered GI function	Hospitalization or elective operative intervention indicated; disabling	Life-threatening consequences; urgent operative intervention indicated	Death
Duodenal perforation	-	Symptomatic; medical intervention indicated	Severe symptoms; elective operative intervention indicated	Life-threatening consequences; urgent operative intervention indicated	Death
Duodenal stenosis	Asymptomatic; clinical or diagnostic observations only; intervention not indicated	Symptomatic; altered GI function	Severely altered GI function; tube feeding; hospitalization indicated; elective operative intervention indicated	Life-threatening consequences; urgent operative intervention indicated	Death
Duodenal ulcer	Asymptomatic; clinical or diagnostic observations only; intervention not indicated	Moderate symptoms; medical intervention indicated; limiting instrumental ADL	Severely altered GI function; TPN indicated; elective operative or endoscopic intervention indicated; limiting self care ADL; disabling	Life-threatening consequences; urgent operative intervention indicated	Death
Dyspepsia	Mild symptoms; intervention not indicated	Moderate symptoms; medical intervention indicated	Severe symptoms; surgical intervention indicated	-	-
Dysphagia	Symptomatic, able to eat regular diet	Symptomatic and altered eating/swallowing	Severely altered eating/swallowing; tube feeding or TPN or hospitalization indicated	Life-threatening consequences; urgent intervention indicated	Death
Enterocolitis	Asymptomatic; clinical or diagnostic observations only; intervention not indicated	Abdominal pain; mucus or blood in stool	Severe or persistent abdominal pain; fever; ileus; peritoneal signs	Life-threatening consequences; urgent intervention indicated	Death
Enterovesical fistula	Asymptomatic; clinical or diagnostic observations only; intervention not indicated	Symptomatic; noninvasive intervention indicated	Severe, medically significant; medical intervention indicated	Life-threatening consequences; urgent intervention indicated	Death

Esophageal fistula	Asymptomatic; clinical or diagnostic observations only; intervention not indicated	Symptomatic; altered GI function	Severely altered GI function; tube feeding, TPN or hospitalization indicated; elective operative intervention indicated	Life-threatening consequences; urgent intervention indicated	Death
Esophageal hemorrhage	Mild; intervention not indicated	Moderate symptoms; medical intervention or minor cauterization indicated	Transfusion, radiologic, endoscopic, or elective operative intervention indicated	Life-threatening consequences; urgent intervention indicated	Death
Esophageal necrosis	-	-	Inability to aliment adequately by GI tract; radiologic, endoscopic, or operative intervention indicated	Life-threatening consequences; urgent operative intervention indicated	Death
Esophageal obstruction	Asymptomatic; clinical or diagnostic observations only; intervention not indicated	Symptomatic; altered GI function; limiting instrumental ADL	Hospitalization indicated; elective operative intervention indicated; limiting self care ADL; disabling	Life-threatening consequences; urgent intervention indicated	Death
Esophageal pain	Mild pain	Moderate pain; limiting instrumental ADL	Severe pain; limiting self care ADL	-	-
Esophageal perforation	-	Symptomatic; medical intervention indicated	Severe symptoms; elective operative intervention indicated	Life-threatening consequences; urgent operative intervention indicated	Death
Esophageal stenosis	Asymptomatic; clinical or diagnostic observations only; intervention not indicated	Symptomatic; altered GI function	Severely altered GI function; tube feeding; hospitalization indicated; elective operative intervention indicated	Life-threatening consequences; urgent operative intervention indicated	Death
Esophageal ulcer	Asymptomatic; clinical or diagnostic observations only; intervention not indicated	Symptomatic; altered GI function; limiting instrumental ADL	Severely altered GI function; TPN indicated; elective operative or endoscopic intervention indicated; limiting self care ADL; disabling	Life-threatening consequences; urgent operative intervention indicated	Death
Esophageal varices hemorrhage	-	Self-limited; intervention not indicated	Transfusion, radiologic, endoscopic, or elective operative intervention indicated	Life-threatening consequences; urgent intervention indicated	Death
Esophagitis	Asymptomatic; clinical or diagnostic observations only; intervention not indicated	Symptomatic; altered eating/swallowing; oral supplements indicated	Severely altered eating/swallowing; tube feeding, TPN or hospitalization indicated	Life-threatening consequences; urgent operative intervention indicated	Death
Fecal incontinence	Occasional use of pads required	Daily use of pads required	Severe symptoms; elective operative intervention indicated	-	-
Flatulence	Mild symptoms; intervention not indicated	Moderate; persistent; psychosocial sequelae	-	-	-
Gastric fistula	Asymptomatic; clinical or diagnostic observations only; intervention not indicated	Symptomatic; altered GI function	Severely altered GI function; bowel rest; tube feeding, TPN or hospitalization indicated; elective operative intervention indicated	Life-threatening consequences; urgent operative intervention indicated	Death

Gastric hemorrhage	Mild; intervention not indicated	Moderate symptoms; medical intervention or minor cauterization indicated	Transfusion, radiologic, endoscopic, or elective operative intervention indicated	Life-threatening consequences; urgent intervention indicated	Death
Gastric necrosis	-	-	Inability to aliment adequately by GI tract; radiologic, endoscopic, or operative intervention indicated	Life-threatening consequences; urgent operative intervention indicated	Death
Gastric perforation	-	Symptomatic; medical intervention indicated	Severe symptoms; elective operative intervention indicated	Life-threatening consequences; urgent operative intervention indicated	Death
Gastric stenosis	Asymptomatic; clinical or diagnostic observations only; intervention not indicated	Symptomatic; altered GI function	Severely altered GI function; tube feeding; hospitalization indicated; elective operative intervention indicated	Life-threatening consequences; urgent operative intervention indicated	Death
Gastric ulcer	Asymptomatic; clinical or diagnostic observations only; intervention not indicated	Symptomatic; altered GI function; medical intervention indicated; limiting instrumental ADL	Severely altered GI function; TPN indicated; elective operative or endoscopic intervention indicated; limiting self care ADL; disabling	Life-threatening consequences; urgent operative intervention indicated	Death
Gastritis	Asymptomatic; clinical or diagnostic observations only; intervention not indicated	Symptomatic; altered GI function; medical intervention indicated	Severely altered eating or gastric function; TPN or hospitalization indicated	Life-threatening consequences; urgent operative intervention indicated	Death
Gastroesophageal reflux disease	Mild symptoms; intervention not indicated	Moderate symptoms; medical intervention indicated	Severe symptoms; surgical intervention indicated	-	-
Gastrointestinal fistula	Asymptomatic; clinical or diagnostic observations only; intervention not indicated	Symptomatic; altered GI function	Severely altered GI function; tube feeding, TPN or hospitalization indicated	Life-threatening consequences; urgent operative intervention indicated	Death
Gastrointestinal pain	Mild pain	Moderate pain; limiting instrumental ADL	Severe pain; limiting self care ADL	-	-
Gastroparesis	Mild nausea, early satiety and bloating, able to maintain caloric intake on regular diet	Moderate symptoms; able to maintain nutrition with dietary and lifestyle modifications; may need pharmacologic intervention	Weight loss; refractory to medical intervention; unable to maintain nutrition orally	-	-
Gingival pain	Mild pain	Moderate pain interfering with oral intake	Severe pain; inability to aliment orally	-	-
Hemorrhoidal hemorrhage	Mild; intervention not indicated	Moderate symptoms; medical intervention or minor cauterization indicated	Transfusion, radiologic, endoscopic, or elective operative intervention indicated	Life-threatening consequences; urgent intervention indicated	Death

Hemorrhoids	Asymptomatic; clinical or diagnostic observations only; intervention not indicated	Symptomatic; banding or medical intervention indicated	Severe symptoms; radiologic, endoscopic or elective operative intervention indicated	-	-
Ileal fistula	Asymptomatic; clinical or diagnostic observations only; intervention not indicated	Symptomatic; altered GI function	Severely altered GI function; TPN or hospitalization indicated; elective operative intervention indicated	Life-threatening consequences; urgent intervention indicated	Death
Ileal hemorrhage	Mild; intervention not indicated	Moderate symptoms; medical intervention or minor cauterization indicated	Transfusion, radiologic, endoscopic, or elective operative intervention indicated	Life-threatening consequences; urgent intervention indicated	Death
Ileal obstruction	Asymptomatic; clinical or diagnostic observations only; intervention not indicated	Symptomatic; altered GI function; limiting instrumental ADL	Hospitalization indicated; elective operative intervention indicated; limiting self care ADL; disabling	Life-threatening consequences; urgent operative intervention indicated	Death
Ileal perforation	-	Symptomatic; medical intervention indicated	Severe symptoms; elective operative intervention indicated	Life-threatening consequences; urgent operative intervention indicated	Death
Ileal stenosis	Asymptomatic; clinical or diagnostic observations only; intervention not indicated	Symptomatic; altered GI function	Severely altered GI function; tube feeding or hospitalization indicated; elective operative intervention indicated	Life-threatening consequences; urgent operative intervention indicated	Death
Ileal ulcer	Asymptomatic; clinical or diagnostic observations only; intervention not indicated	Symptomatic; altered GI function	Severely altered GI function; TPN indicated; elective operative or endoscopic intervention indicated; disabling	Life-threatening consequences; urgent operative intervention indicated	Death
Ileus	-	Symptomatic; altered GI function; bowel rest indicated	Severely altered GI function; TPN indicated	Life-threatening consequences; urgent intervention indicated	Death
Intra-abdominal hemorrhage	-	Medical intervention or minor cauterization indicated	Transfusion, radiologic, endoscopic, or elective operative intervention indicated	Life-threatening consequences; urgent intervention indicated	Death
Jejunal fistula	Asymptomatic; clinical or diagnostic observations only; intervention not indicated	Symptomatic; altered GI function	Severely altered GI function; TPN or hospitalization indicated; elective operative intervention indicated	Life-threatening consequences; urgent intervention indicated	Death
Jejunal hemorrhage	Mild; intervention not indicated	Moderate symptoms; medical intervention or minor cauterization indicated	Transfusion, radiologic, endoscopic, or elective operative intervention indicated	Life-threatening consequences; urgent intervention indicated	Death
Jejunal obstruction	Asymptomatic; clinical or diagnostic observations only; intervention not indicated	Symptomatic; altered GI function; limiting instrumental ADL	Hospitalization indicated; elective operative intervention indicated; limiting self care ADL; disabling	Life-threatening consequences; urgent operative intervention indicated	Death
Jejunal perforation	-	Symptomatic; medical intervention indicated	Severe symptoms; elective operative intervention indicated	Life-threatening consequences; urgent operative intervention indicated	Death

Jejunal stenosis	Asymptomatic; clinical or diagnostic observations only; intervention not indicated	Symptomatic; altered GI function	Severely altered GI function; tube feeding or hospitalization indicated; elective operative intervention indicated	Life-threatening consequences; urgent operative intervention indicated	Death
Jejunal ulcer	Asymptomatic; clinical or diagnostic observations only; intervention not indicated	Symptomatic; altered GI function	Severely altered GI function; TPN indicated; elective operative or endoscopic intervention indicated; disabling	Life-threatening consequences; urgent operative intervention indicated	Death
Lip pain	Mild pain	Moderate pain; limiting instrumental ADL	Severe pain; limiting self care ADL	-	-
Lower gastrointestinal hemorrhage	Mild; intervention not indicated	Moderate symptoms; medical intervention or minor cauterization indicated	Transfusion, radiologic, endoscopic, or elective operative intervention indicated	Life-threatening consequences; urgent intervention indicated	Death
Malabsorption	-	Altered diet; oral intervention indicated	Inability to aliment adequately; TPN indicated	Life-threatening consequences; urgent intervention indicated	Death
Mucositis oral	Asymptomatic or mild symptoms; intervention not indicated	Moderate pain; not interfering with oral intake; modified diet indicated	Severe pain; interfering with oral intake	Life-threatening consequences; urgent intervention indicated	Death
Nausea	Loss of appetite without alteration in eating habits	Oral intake decreased without significant weight loss, dehydration or malnutrition	Inadequate oral caloric or fluid intake; tube feeding, TPN, or hospitalization indicated	-	-
Obstruction gastric	Asymptomatic; clinical or diagnostic observations only; intervention not indicated	Symptomatic; altered GI function; limiting instrumental ADL	Hospitalization indicated; elective operative intervention indicated; limiting self care ADL; disabling	Life-threatening consequences; urgent operative intervention indicated	Death
Oral cavity fistula	Asymptomatic; clinical or diagnostic observations only; intervention not indicated	Symptomatic; altered GI function	Severely altered GI function; TPN or hospitalization indicated; elective operative intervention indicated	Life-threatening consequences; urgent intervention indicated	Death
Oral dysesthesia	Mild discomfort; not interfering with oral intake	Moderate pain; interfering with oral intake	Disabling pain; tube feeding or TPN indicated	-	-
Oral hemorrhage	Mild; intervention not indicated	Moderate symptoms; medical intervention or minor cauterization indicated	Transfusion, radiologic, endoscopic, or elective operative intervention indicated	Life-threatening consequences; urgent intervention indicated	Death
Oral pain	Mild pain	Moderate pain; limiting instrumental ADL	Severe pain; limiting self care ADL	-	-
Pancreatic duct stenosis	Asymptomatic; clinical or diagnostic observations only; intervention not indicated	Symptomatic; altered GI function	Severely altered GI function; tube feeding or hospitalization indicated; elective operative intervention indicated	Life-threatening consequences; urgent operative intervention indicated	Death

Pancreatic fistula	Asymptomatic; clinical or diagnostic observations only; intervention not indicated	Symptomatic; altered GI function	Severely altered GI function; tube feeding or TPN or hospitalization indicated; elective operative intervention indicated	Life-threatening consequences; urgent operative intervention indicated	Death
Pancreatic hemorrhage	Mild; intervention not indicated	Moderate symptoms; medical intervention or minor cauterization indicated	Transfusion, radiologic, endoscopic, or elective operative intervention indicated	Life-threatening consequences; urgent intervention indicated	Death
Pancreatic necrosis	-	-	Tube feeding or TPN indicated; radiologic, endoscopic, or operative intervention indicated	Life-threatening consequences; urgent operative intervention indicated	Death
Pancreatitis	-	Enzyme elevation or radiologic findings only	Severe pain; vomiting; medical intervention indicated (e.g., analgesia, nutritional support)	Life-threatening consequences; urgent intervention indicated	Death
Periodontal disease	Gingival recession or gingivitis; limited bleeding on probing; mild local bone loss	Moderate gingival recession or gingivitis; multiple sites of bleeding on probing; moderate bone loss	Spontaneous bleeding; severe bone loss with or without tooth loss; osteonecrosis of maxilla or mandible	-	-
Peritoneal necrosis	-	-	Tube feeding or TPN indicated; radiologic, endoscopic, or operative intervention indicated	Life-threatening consequences; urgent operative intervention indicated	Death
Proctitis	Rectal discomfort, intervention not indicated	Symptoms (e.g., rectal discomfort, passing blood or mucus); medical intervention indicated; limiting instrumental ADL	Severe symptoms; fecal urgency or stool incontinence; limiting self care ADL	Life-threatening consequences; urgent intervention indicated	Death
Rectal fistula	Asymptomatic; clinical or diagnostic observations only; intervention not indicated	Symptomatic; altered GI function	Severely altered GI function; TPN or hospitalization indicated; elective operative intervention indicated	Life-threatening consequences; urgent intervention indicated	Death
Rectal hemorrhage	Mild; intervention not indicated	Moderate symptoms; medical intervention or minor cauterization indicated	Transfusion, radiologic, endoscopic, or elective operative intervention indicated	Life-threatening consequences; urgent intervention indicated	Death
Rectal mucositis	Asymptomatic or mild symptoms; intervention not indicated	Symptomatic; medical intervention indicated; limiting instrumental ADL	Severe symptoms; limiting self care ADL	Life-threatening consequences; urgent operative intervention indicated	Death
Rectal necrosis	-	-	Tube feeding or TPN indicated; radiologic, endoscopic, or operative intervention indicated	Life-threatening consequences; urgent operative intervention indicated	Death
Rectal obstruction	Asymptomatic; clinical or diagnostic observations only; intervention not indicated	Symptomatic; altered GI function; limiting instrumental ADL	Hospitalization indicated; elective operative intervention indicated; limiting self care ADL; disabling	Life-threatening consequences; urgent operative intervention indicated	Death
Rectal pain	Mild pain	Moderate pain; limiting instrumental ADL	Severe pain; limiting self care ADL	-	-

Rectal perforation	-	Symptomatic; medical intervention indicated	Severe symptoms; elective operative intervention indicated	Life-threatening consequences; urgent operative intervention indicated	Death
Rectal stenosis	Asymptomatic; clinical or diagnostic observations only; intervention not indicated	Symptomatic; altered GI function	Severely altered GI function; tube feeding or hospitalization indicated; elective operative intervention indicated	Life-threatening consequences; urgent operative intervention indicated	Death
Rectal ulcer	Asymptomatic; clinical or diagnostic observations only; intervention not indicated	Symptomatic; altered GI function (e.g. altered dietary habits, vomiting, diarrhea)	Severely altered GI function; TPN indicated; elective operative or endoscopic intervention indicated; disabling	Life-threatening consequences; urgent operative intervention indicated	Death
Retroperitoneal hemorrhage	-	Self-limited; intervention indicated	Transfusion, medical, radiologic, endoscopic, or elective operative intervention indicated	Life-threatening consequences; urgent intervention indicated	Death
Salivary duct inflammation	Slightly thickened saliva; slightly altered taste (e.g., metallic)	Thick, ropy, sticky saliva; markedly altered taste; alteration in diet indicated; secretion-induced symptoms; limiting instrumental ADL	Acute salivary gland necrosis; severe secretion-induced symptoms (e.g., thick saliva/oral secretions or gagging); tube feeding or TPN indicated; limiting self care ADL; disabling	Life-threatening consequences; urgent intervention indicated	Death
Salivary gland fistula	Asymptomatic; clinical or diagnostic observations only; intervention not indicated	Symptomatic; altered GI function; tube feeding indicated	Severely altered GI function; hospitalization indicated; elective operative intervention indicated	Life-threatening consequences; urgent operative intervention indicated	Death
Small intestinal mucositis	Asymptomatic or mild symptoms; intervention not indicated	Symptomatic; medical intervention indicated; limiting instrumental ADL	Severe pain; interfering with oral intake; tube feeding, TPN or hospitalization indicated; limiting self care ADL	Life-threatening consequences; urgent intervention indicated	Death
Small intestinal obstruction	Asymptomatic; clinical or diagnostic observations only; intervention not indicated	Symptomatic; altered GI function; limiting instrumental ADL	Hospitalization indicated; elective operative intervention indicated; limiting self care ADL; disabling	Life-threatening consequences; urgent operative intervention indicated	Death
Small intestinal perforation	-	Symptomatic; medical intervention indicated	Severe symptoms; elective operative intervention indicated	Life-threatening consequences; urgent operative intervention indicated	Death
Small intestinal stenosis	Asymptomatic; clinical or diagnostic observations only; intervention not indicated	Symptomatic; altered GI function	Symptomatic and severely altered GI function; tube feeding, TPN or hospitalization indicated; non-emergent operative intervention indicated	Life-threatening consequences; urgent operative intervention indicated	Death

Small intestine ulcer	Asymptomatic; clinical or diagnostic observations only; intervention not indicated	Symptomatic; altered GI function; limiting instrumental ADL	Severely altered GI function; TPN indicated; elective operative or endoscopic intervention indicated; limiting self care ADL; disabling	Life-threatening consequences; urgent operative intervention indicated	Death
Stomach pain	Mild pain	Moderate pain; limiting instrumental ADL	Severe pain; limiting self care ADL	-	-
Tooth development disorder	Asymptomatic; hypoplasia of tooth or enamel	Impairment correctable with oral surgery	Maldevelopment with impairment not surgically correctable; disabling	-	-
Tooth discoloration	Surface stains	-	-	-	-
Toothache	Mild pain	Moderate pain; limiting instrumental ADL	Severe pain; limiting self care ADL	-	-
Typhlitis	-	-	Symptomatic (e.g., abdominal pain, fever, change in bowel habits with ileus); peritoneal signs	Life-threatening consequences; urgent operative intervention indicated	Death
Upper gastrointestinal hemorrhage	Mild; intervention not indicated	Moderate symptoms; medical intervention or minor cauterization indicated	Transfusion, radiologic, endoscopic, or elective operative intervention indicated	Life-threatening consequences; urgent intervention indicated	Death
Vomiting	1 - 2 episodes (separated by 5 minutes) in 24 hrs	3 - 5 episodes (separated by 5 minutes) in 24 hrs	>=6 episodes (separated by 5 minutes) in 24 hrs; tube feeding, TPN or hospitalization indicated	Life-threatening consequences; urgent intervention indicated	Death
Gastrointestinal disorders - Other, specify	Asymptomatic or mild symptoms; clinical or diagnostic observations only; intervention not indicated	Moderate; minimal, local or noninvasive intervention indicated; limiting age-appropriate instrumental ADL	Severe or medically significant but not immediately life-threatening; hospitalization or prolongation of existing hospitalization indicated; disabling; limiting self care ADL	Life-threatening consequences; urgent intervention indicated	Death
General disorders and administration site conditions	Grade				
Adverse Event	1	2	3	4	5
Chills	Mild sensation of cold; shivering; chattering of teeth	Moderate tremor of the entire body; narcotics indicated	Severe or prolonged, not responsive to narcotics	-	-
Death neonatal	-	-	-	-	Death

Death NOS	-	-	-	-	Death
Edema face	Localized facial edema	Moderate localized facial edema; limiting instrumental ADL	Severe swelling; limiting self care ADL	-	-
Edema limbs	5 - 10% inter-limb discrepancy in volume or circumference at point of greatest visible difference; swelling or obscuration of anatomic architecture on close inspection	>10 - 30% inter-limb discrepancy in volume or circumference at point of greatest visible difference; readily apparent obscuration of anatomic architecture; obliteration of skin folds; readily apparent deviation from normal anatomic contour; limiting instrumental ADL	>30% inter-limb discrepancy in volume; gross deviation from normal anatomic contour; limiting self care ADL	-	-
Edema trunk	Swelling or obscuration of anatomic architecture on close inspection	Readily apparent obscuration of anatomic architecture; obliteration of skin folds; readily apparent deviation from normal anatomic contour; limiting instrumental ADL	Gross deviation from normal anatomic contour; limiting self care ADL	-	-
Facial pain	Mild pain	Moderate pain; limiting instrumental ADL	Severe pain; limiting self care ADL	-	-
Fatigue	Fatigue relieved by rest	Fatigue not relieved by rest; limiting instrumental ADL	Fatigue not relieved by rest, limiting self care ADL	-	-
Fever	38.0 - 39.0 degrees C (100.4 - 102.2 degrees F)	>39.0 - 40.0 degrees C (102.3 - 104.0 degrees F)	>40.0 degrees C (>104.0 degrees F) for <=24 hrs	>40.0 degrees C (>104.0 degrees F) for >24 hrs	Death
Flu like symptoms	Mild flu-like symptoms present	Moderate symptoms; limiting instrumental ADL	Severe symptoms; limiting self care ADL	-	-
Gait disturbance	Mild change in gait (e.g., wide-based, limping or hobbling)	Moderate change in gait (e.g., wide-based, limping or hobbling); assistive device indicated; limiting instrumental ADL	Disabling; limiting self care ADL	-	-

Hypothermia	-	35 - >32 degrees C; 95 - >89.6 degrees F	32 - >28 degrees C; 89.6 - >82.4 degrees F	<=28 degrees C; 82.4 degrees F; life-threatening consequences (e.g., coma, hypotension, pulmonary edema, acidemia, ventricular fibrillation)	Death
Infusion related reaction	Mild transient reaction; infusion interruption not indicated; intervention not indicated	Therapy or infusion interruption indicated but responds promptly to symptomatic treatment (e.g., antihistamines, NSAIDS, narcotics, IV fluids); prophylactic medications indicated for <=24 hrs	Prolonged (e.g., not rapidly responsive to symptomatic medication and/or brief interruption of infusion); recurrence of symptoms following initial improvement; hospitalization indicated for clinical sequelae	Life-threatening consequences; urgent intervention indicated	Death
Infusion site extravasation	-	Erythema with associated symptoms (e.g., edema, pain, induration, phlebitis)	Ulceration or necrosis; severe tissue damage; operative intervention indicated	Life-threatening consequences; urgent intervention indicated	Death
Injection site reaction	Tenderness with or without associated symptoms (e.g., warmth, erythema, itching)	Pain; lipodystrophy; edema; phlebitis	Ulceration or necrosis; severe tissue damage; operative intervention indicated	Life-threatening consequences; urgent intervention indicated	Death
Irritability	Mild; easily consolable	Moderate; limiting instrumental ADL; increased attention indicated	Severe abnormal or excessive response; limiting self care ADL; inconsolable	-	-
Localized edema	Localized to dependent areas, no disability or functional impairment	Moderate localized edema and intervention indicated; limiting instrumental ADL	Severe localized edema and intervention indicated; limiting self care ADL	-	-
Malaise	Uneasiness or lack of well being	Uneasiness or lack of well being; limiting instrumental ADL	-	-	-
Multi-organ failure	-	-	Shock with azotemia and acid-base disturbances; significant coagulation abnormalities	Life-threatening consequences (e.g., vasopressor dependent and oliguric or anuric or ischemic colitis or lactic acidosis)	Death
Neck edema	Asymptomatic localized neck edema	Moderate neck edema; slight obliteration of anatomic landmarks; limiting instrumental ADL	Generalized neck edema (e.g., difficulty in turning neck); limiting self care ADL	-	-

Non-cardiac chest pain	Mild pain	Moderate pain; limiting instrumental ADL	Severe pain; limiting self care ADL	-	-
Pain	Mild pain	Moderate pain; limiting instrumental ADL	Severe pain; limiting self care ADL	-	-
Sudden death NOS	-	-	-	-	Death
General disorders and administration site conditions - Other, specify	Asymptomatic or mild symptoms; clinical or diagnostic observations only; intervention not indicated	Moderate; minimal, local or noninvasive intervention indicated; limiting age-appropriate instrumental ADL	Severe or medically significant but not immediately life-threatening; hospitalization or prolongation of existing hospitalization indicated; disabling; limiting self care ADL	Life-threatening consequences; urgent intervention indicated	Death
Hepatobiliary disorders	Grade				
Adverse Event	1	2	3	4	5
Bile duct stenosis	Asymptomatic; clinical or diagnostic observations only; intervention not indicated	Symptomatic; altered GI function; IV fluids indicated <24 hrs	Severely altered GI function; radiologic, endoscopic or elective operative intervention indicated	Life-threatening consequences; urgent operative intervention indicated	Death
Biliary fistula	-	Symptomatic and intervention not indicated	Severely altered GI function; TPN indicated; endoscopic intervention indicated; elective operative intervention indicated	Life-threatening consequences; urgent operative intervention indicated	Death
Cholecystitis	-	Symptomatic; medical intervention indicated	Severe symptoms; radiologic, endoscopic or elective operative intervention indicated	Life-threatening consequences; urgent operative intervention indicated	Death
Gallbladder fistula	Asymptomatic clinical or diagnostic observations only; intervention not indicated	Symptomatic and intervention not indicated	Symptomatic or severely altered GI function; TPN indicated; radiologic, endoscopic or elective operative intervention indicated	Life-threatening consequences; urgent operative intervention indicated	Death
Gallbladder necrosis	-	-	-	Life-threatening consequences; urgent radiologic or operative intervention indicated	Death

Gallbladder obstruction	Asymptomatic; clinical or diagnostic observations only; intervention not indicated	Symptomatic; altered GI function; IV fluids indicated <24 hrs	Symptomatic and severely altered GI function; tube feeding, TPN or hospitalization indicated; non-emergent operative intervention indicated	Life-threatening consequences; urgent operative intervention indicated	Death
Gallbladder pain	Mild pain	Moderate pain; limiting instrumental ADL	Severe pain; limiting self care ADL	-	-
Gallbladder perforation	-	-	-	Life-threatening consequences; urgent intervention indicated	Death
Hepatic failure	-	-	Asterixis; mild encephalopathy; limiting self care ADL	Moderate to severe encephalopathy; coma; life-threatening consequences	Death
Hepatic hemorrhage	Mild; intervention not indicated	Symptomatic; medical intervention indicated	Transfusion indicated	Life-threatening consequences; urgent intervention indicated	Death
Hepatic necrosis	-	-	-	Life-threatening consequences; urgent radiologic or operative intervention indicated	Death
Hepatic pain	Mild pain	Moderate pain; limiting instrumental ADL	Severe pain; limiting self care ADL	-	-
Perforation bile duct	-	-	Radiologic, endoscopic or elective operative intervention indicated	Life-threatening consequences; urgent operative intervention indicated	Death
Portal hypertension	-	Decreased portal vein flow	Reversal/retrograde portal vein flow; associated with varices and/or ascites	Life-threatening consequences; urgent intervention indicated	Death
Portal vein thrombosis	-	Intervention not indicated	Medical intervention indicated	Life-threatening consequences; urgent intervention indicated	Death
Hepatobiliary disorders - Other, specify	Asymptomatic or mild symptoms; clinical or diagnostic observations only; intervention not indicated	Moderate; minimal, local or noninvasive intervention indicated; limiting age-appropriate instrumental ADL	Severe or medically significant but not immediately life-threatening; hospitalization or prolongation of existing hospitalization indicated; disabling; limiting self care ADL	Life-threatening consequences; urgent intervention indicated	Death

Immune system disorders	Grade				
Adverse Event	1	2	3	4	5
Allergic reaction	Transient flushing or rash, drug fever <38 degrees C (<100.4 degrees F); intervention not indicated	Intervention or infusion interruption indicated; responds promptly to symptomatic treatment (e.g., antihistamines, NSAIDS, narcotics); prophylactic medications indicated for <=24 hrs	Prolonged (e.g., not rapidly responsive to symptomatic medication and/or brief interruption of infusion); recurrence of symptoms following initial improvement; hospitalization indicated for clinical sequelae (e.g., renal impairment, pulmonary infiltrates)	Life-threatening consequences; urgent intervention indicated	Death
Anaphylaxis	-	-	Symptomatic bronchospasm, with or without urticaria; parenteral intervention indicated; allergy-related edema/angioedema; hypotension	Life-threatening consequences; urgent intervention indicated	Death
Autoimmune disorder	Asymptomatic; serologic or other evidence of autoimmune reaction, with normal organ function; intervention not indicated	Evidence of autoimmune reaction involving a non-essential organ or function (e.g., hypothyroidism)	Autoimmune reactions involving major organ (e.g., colitis, anemia, myocarditis, kidney)	Life-threatening consequences; urgent intervention indicated	Death
Cytokine release syndrome	Mild reaction; infusion interruption not indicated; intervention not indicated	Therapy or infusion interruption indicated but responds promptly to symptomatic treatment (e.g., antihistamines, NSAIDS, narcotics, IV fluids); prophylactic medications indicated for <=24 hrs	Prolonged (e.g., not rapidly responsive to symptomatic medication and/or brief interruption of infusion); recurrence of symptoms following initial improvement; hospitalization indicated for clinical sequelae (e.g., renal impairment, pulmonary infiltrates)	Life-threatening consequences; pressor or ventilatory support indicated	Death
Serum sickness	Asymptomatic; clinical or diagnostic observations only; intervention not indicated	Moderate arthralgia; fever, rash, urticaria, antihistamines indicated	Severe arthralgia or arthritis; extensive rash; steroids or IV fluids indicated	Life-threatening consequences; pressor or ventilatory support indicated	Death
Immune system disorders - Other, specify	Asymptomatic or mild symptoms; clinical or diagnostic observations only; intervention not indicated	Moderate; minimal, local or noninvasive intervention indicated; limiting age-appropriate instrumental ADL	Severe or medically significant but not immediately life-threatening; hospitalization or prolongation of existing hospitalization indicated; disabling; limiting self care ADL	Life-threatening consequences; urgent intervention indicated	Death

Infections and infestations	Grade				
Adverse Event	1	2	3	4	5
Abdominal infection	-	-	IV antibiotic, antifungal, or antiviral intervention indicated; radiologic or operative intervention indicated	Life-threatening consequences; urgent intervention indicated	Death
Anorectal infection	Localized; local intervention indicated	Oral intervention indicated (e.g., antibiotic, antifungal, antiviral)	IV antibiotic, antifungal, or antiviral intervention indicated; radiologic, endoscopic, or operative intervention indicated	Life-threatening consequences; urgent intervention indicated	Death
Appendicitis	-	-	IV antibiotic, antifungal, or antiviral intervention indicated; radiologic or operative intervention indicated	Life-threatening consequences; urgent intervention indicated	Death
Appendicitis perforated	-	Symptomatic; medical intervention indicated	Severe symptoms; elective operative intervention indicated	Life-threatening consequences; urgent intervention indicated	Death
Arteritis infective	-	-	IV antibiotic, antifungal, or antiviral intervention indicated; radiologic or operative intervention indicated	Life-threatening consequences; urgent intervention indicated	Death
Biliary tract infection	-	-	IV antibiotic, antifungal, or antiviral intervention indicated; radiologic or operative intervention indicated	Life-threatening consequences; urgent intervention indicated	Death
Bladder infection	-	Oral intervention indicated (e.g., antibiotic, antifungal, antiviral)	IV antibiotic, antifungal, or antiviral intervention indicated; radiologic, endoscopic, or operative intervention indicated	Life-threatening consequences; urgent intervention indicated	Death
Bone infection	-	-	IV antibiotic, antifungal, or antiviral intervention indicated; radiologic or operative intervention indicated	Life-threatening consequences; urgent intervention indicated	Death
Breast infection	-	Local infection with moderate symptoms; oral intervention indicated (e.g., antibiotic, antifungal, antiviral)	Severe infection; axillary adenitis; IV antibacterial, antifungal, or antiviral intervention indicated	Life-threatening consequences; urgent intervention indicated	Death
Bronchial infection	-	Moderate symptoms; oral intervention indicated (e.g., antibiotic, antifungal, antiviral)	IV antibiotic, antifungal, or antiviral intervention indicated; radiologic, endoscopic, or operative intervention indicated	Life-threatening consequences; urgent intervention indicated	Death

Catheter related infection	-	Localized; local intervention indicated; oral intervention indicated (e.g., antibiotic, antifungal, antiviral)	IV antibiotic, antifungal, or antiviral intervention indicated; radiologic or operative intervention indicated	Life-threatening consequences; urgent intervention indicated	Death
Cecal infection	-	-	IV antibiotic, antifungal, or antiviral intervention indicated; radiologic, endoscopic, or operative intervention indicated	Life-threatening consequences; urgent intervention indicated	Death
Cervicitis infection	-	Localized; local intervention indicated (e.g., topical antibiotic, antifungal, or antiviral)	IV antibiotic, antifungal, or antiviral intervention indicated; radiologic or operative intervention indicated	Life-threatening consequences; urgent intervention indicated	Death
Conjunctivitis infective	-	Localized; local intervention indicated (e.g., topical antibiotic, antifungal, or antiviral)	IV antibiotic, antifungal, or antiviral intervention indicated; radiologic or operative intervention indicated	Life-threatening consequences; urgent intervention indicated	Death
Corneal infection	-	Localized; local intervention indicated (e.g., topical antibiotic, antifungal, or antiviral)	IV antibiotic, antifungal, or antiviral intervention indicated; radiologic or operative intervention indicated	Life-threatening consequences; urgent intervention indicated	Death
Cranial nerve infection	-	-	IV antibiotic, antifungal, or antiviral intervention indicated; radiologic or operative intervention indicated	Life-threatening consequences; urgent intervention indicated	Death
Device related infection	-	-	IV antibiotic, antifungal, or antiviral intervention indicated; radiologic or operative intervention indicated	Life-threatening consequences; urgent intervention indicated	Death
Duodenal infection	-	Moderate symptoms; medical intervention indicated (e.g., oral antibiotics)	IV antibiotic, antifungal, or antiviral intervention indicated; radiologic or operative intervention indicated	Life-threatening consequences; urgent intervention indicated	Death
Encephalitis infection	-	-	IV antibiotic, antifungal, or antiviral intervention indicated; severe changes in mental status; self-limited seizure activity; focal neurologic abnormalities	Life-threatening consequences; urgent intervention indicated	Death
Encephalomyelitis infection	-	-	IV antibiotic, antifungal, or antiviral intervention indicated; radiologic or operative intervention indicated	Life-threatening consequences; urgent intervention indicated	Death
Endocarditis infective	-	-	IV antibiotic, antifungal, or antiviral intervention indicated; radiologic or operative intervention indicated	Life-threatening consequences; urgent intervention indicated	Death
Endophthalmitis	-	Local intervention indicated	Systemic intervention or hospitalization indicated	Blindness (20/200 or worse)	-

Enterocolitis infectious	-	Passage of >3 unformed stools per 24 hrs or duration of illness >48 hrs; moderate abdominal pain	IV antibiotic, antifungal, or antiviral intervention indicated; radiologic, endoscopic, or operative intervention indicated; profuse watery diarrhea with signs of hypovolemia; bloody diarrhea; fever; severe abdominal pain; hospitalization indicated	Life-threatening consequences; urgent intervention indicated	Death
Esophageal infection	-	Local intervention indicated (e.g., oral antibiotic, antifungal, antiviral)	IV antibiotic, antifungal, or antiviral intervention indicated; radiologic or operative intervention indicated	Life-threatening consequences; urgent intervention indicated	Death
Eye infection	-	Localized; local intervention indicated (e.g., topical antibiotic, antifungal, or antiviral)	IV antibiotic, antifungal, or antiviral intervention indicated; radiologic or operative intervention indicated	Life-threatening consequences; urgent intervention indicated; enucleation	Death
Gallbladder infection	-	-	IV antibiotic, antifungal, or antiviral intervention indicated; radiologic, endoscopic, or operative intervention indicated	Life-threatening consequences; urgent intervention indicated	Death
Gum infection	Local therapy indicated (swish and swallow)	Moderate symptoms; oral intervention indicated (e.g., antibiotic, antifungal, antiviral)	IV antibiotic, antifungal, or antiviral intervention indicated; radiologic or operative intervention indicated	Life-threatening consequences; urgent intervention indicated	Death
Hepatic infection	-	-	IV antibiotic, antifungal, or antiviral intervention indicated; radiologic or operative intervention indicated	Life-threatening consequences; urgent intervention indicated	Death
Hepatitis viral	Asymptomatic, treatment not indicated	-	Symptomatic liver dysfunction; fibrosis by biopsy; compensated cirrhosis; reactivation of chronic hepatitis	Decompensated liver function (e.g., ascites, coagulopathy, encephalopathy, coma)	Death
Infective myositis	-	Localized; local intervention indicated (e.g., topical antibiotic, antifungal, or antiviral)	IV antibiotic, antifungal, or antiviral intervention indicated; radiologic or operative intervention indicated	Life-threatening consequences; urgent intervention indicated	Death
Joint infection	-	Localized; local intervention indicated; oral intervention indicated (e.g., antibiotic, antifungal, antiviral); needle aspiration indicated (single or multiple)	Arthroscopic intervention indicated (e.g., drainage) or arthrotomy (e.g., open surgical drainage)	Life-threatening consequences; urgent intervention indicated	Death

Kidney infection	-	-	IV antibiotic, antifungal, or antiviral intervention indicated; radiologic, endoscopic, or operative intervention indicated	Life-threatening consequences; urgent intervention indicated	Death
Laryngitis	-	Moderate symptoms; oral intervention indicated (e.g., antibiotic, antifungal, antiviral)	IV antibiotic, antifungal, or antiviral intervention indicated; radiologic or operative intervention indicated	Life-threatening consequences; urgent intervention indicated	Death
Lip infection	Localized, local intervention indicated	Oral intervention indicated (e.g., antibiotic, antifungal, antiviral)	IV antibiotic, antifungal, or antiviral intervention indicated; radiologic or operative intervention indicated	-	-
Lung infection	-	Moderate symptoms; oral intervention indicated (e.g., antibiotic, antifungal, antiviral)	IV antibiotic, antifungal, or antiviral intervention indicated; radiologic, endoscopic, or operative intervention indicated	Life-threatening consequences; urgent intervention indicated	Death
Lymph gland infection	-	Localized; local intervention indicated (e.g., topical antibiotic, antifungal, or antiviral)	IV antibiotic, antifungal, or antiviral intervention indicated; radiologic or operative intervention indicated	Life-threatening consequences; urgent intervention indicated	Death
Mediastinal infection	-	-	IV antibiotic, antifungal, or antiviral intervention indicated; radiologic or operative intervention indicated	Life-threatening consequences; urgent intervention indicated	Death
Meningitis	-	-	IV antibiotic, antifungal, or antiviral intervention indicated; radiologic or operative intervention indicated; focal neurologic deficit	Life-threatening consequences; urgent intervention indicated	Death
Mucosal infection	Localized, local intervention indicated	Oral intervention indicated (e.g., antibiotic, antifungal, antiviral)	IV antibiotic, antifungal, or antiviral intervention indicated; radiologic or operative intervention indicated	Life-threatening consequences; urgent intervention indicated	Death
Nail infection	Localized, local intervention indicated	Oral intervention indicated (e.g., antibiotic, antifungal, antiviral)	IV antibiotic, antifungal, or antiviral intervention indicated; radiologic or operative intervention indicated	-	-
Otitis externa	-	Localized; local intervention indicated (e.g., topical antibiotic, antifungal, or antiviral)	IV antibiotic, antifungal, or antiviral intervention indicated; radiologic or operative intervention indicated	Life-threatening consequences; urgent intervention indicated	Death
Otitis media	-	Localized; local intervention indicated (e.g., topical antibiotic, antifungal, or antiviral)	IV antibiotic, antifungal, or antiviral intervention indicated; radiologic or operative intervention indicated	Life-threatening consequences; urgent intervention indicated	Death

Ovarian infection	-	Localized; local intervention indicated (e.g., topical antibiotic, antifungal, or antiviral)	IV antibiotic, antifungal, or antiviral intervention indicated; radiologic or operative intervention indicated	Life-threatening consequences; urgent intervention indicated	Death
Pancreas infection	-	-	IV antibiotic, antifungal, or antiviral intervention indicated; radiologic or operative intervention indicated	Life-threatening consequences; urgent intervention indicated	Death
Papulopustular rash	Papules and/or pustules covering <10% BSA, which may or may not be associated with symptoms of pruritus or tenderness	Papules and/or pustules covering 10-30% BSA, which may or may not be associated with symptoms of pruritus or tenderness; associated with psychosocial impact; limiting instrumental ADL	Papules and/or pustules covering >30% BSA, which may or may not be associated with symptoms of pruritus or tenderness; limiting self-care ADL; associated with local superinfection with oral antibiotics indicated	Papules and/or pustules covering any % BSA, which may or may not be associated with symptoms of pruritus or tenderness and are associated with extensive superinfection with IV antibiotics indicated; life-threatening consequences	Death
Paronychia	Nail fold edema or erythema; disruption of the cuticle	Localized intervention indicated; oral intervention indicated (e.g., antibiotic, antifungal, antiviral); nail fold edema or erythema with pain; associated with discharge or nail plate separation; limiting instrumental ADL	Surgical intervention or IV antibiotics indicated; limiting self care ADL	-	-
Pelvic infection	-	Moderate symptoms; oral intervention indicated (e.g., antibiotic, antifungal, antiviral)	IV antibiotic, antifungal, or antiviral intervention indicated; radiologic or operative intervention indicated	Life-threatening consequences; urgent intervention indicated	Death
Penile infection	-	Localized; local intervention indicated (e.g., topical antibiotic, antifungal, or antiviral)	IV antibiotic, antifungal, or antiviral intervention indicated; radiologic or operative intervention indicated	Life-threatening consequences; urgent intervention indicated	Death
Periorbital infection	-	Localized; local intervention indicated (e.g., topical antibiotic, antifungal, or antiviral)	IV antibiotic, antifungal, or antiviral intervention indicated; radiologic or operative intervention indicated	Life-threatening consequences; urgent intervention indicated	Death
Peripheral nerve infection	-	Localized; local intervention indicated (e.g., topical antibiotic, antifungal, or antiviral)	IV antibiotic, antifungal, or antiviral intervention indicated; radiologic or operative intervention indicated	Life-threatening consequences; urgent intervention indicated	Death
Peritoneal infection	-	-	IV antibiotic, antifungal, or antiviral intervention indicated; radiologic, endoscopic, or operative intervention indicated	Life-threatening consequences; urgent intervention indicated	Death

Pharyngitis	-	Localized; local intervention indicated (e.g., topical antibiotic, antifungal, or antiviral)	IV antibiotic, antifungal, or antiviral intervention indicated; radiologic or operative intervention indicated	Life-threatening consequences; urgent intervention indicated	Death
Phlebitis infective	-	Localized; local intervention indicated (e.g., topical antibiotic, antifungal, or antiviral)	IV antibiotic, antifungal, or antiviral intervention indicated; radiologic or operative intervention indicated	Life-threatening consequences; urgent intervention indicated	Death
Pleural infection	-	Localized; local intervention indicated (e.g., topical antibiotic, antifungal, or antiviral)	IV antibiotic, antifungal, or antiviral intervention indicated; radiologic, endoscopic, or operative intervention indicated	Life-threatening consequences; urgent intervention indicated	Death
Prostate infection	-	Moderate symptoms; oral intervention indicated (e.g., antibiotic, antifungal, antiviral)	IV antibiotic, antifungal, or antiviral intervention indicated; radiologic, endoscopic, or operative intervention indicated	Life-threatening consequences; urgent intervention indicated	Death
Rash pustular	-	Localized; local intervention indicated (e.g., topical antibiotic, antifungal, or antiviral)	IV antibiotic, antifungal, or antiviral intervention indicated; radiologic or operative intervention indicated	-	-
Rhinitis infective	-	Localized; local intervention indicated (e.g., topical antibiotic, antifungal, or antiviral)	-	-	-
Salivary gland infection	-	Moderate symptoms; oral intervention indicated (e.g., antibiotic, antifungal, antiviral)	IV antibiotic, antifungal, or antiviral intervention indicated; radiologic or operative intervention indicated	Life-threatening consequences; urgent intervention indicated	Death
Scrotal infection	-	Localized; local intervention indicated (e.g., topical antibiotic, antifungal, or antiviral)	IV antibiotic, antifungal, or antiviral intervention indicated; radiologic or operative intervention indicated	Life-threatening consequences; urgent intervention indicated	Death
Sepsis	-	-	-	Life-threatening consequences; urgent intervention indicated	Death
Sinusitis	-	Localized; local intervention indicated (e.g., topical antibiotic, antifungal, or antiviral)	IV antibiotic, antifungal, or antiviral intervention indicated; radiologic, endoscopic, or operative intervention indicated	Life-threatening consequences; urgent intervention indicated	Death
Skin infection	Localized, local intervention indicated	Oral intervention indicated (e.g., antibiotic, antifungal, antiviral)	IV antibiotic, antifungal, or antiviral intervention indicated; radiologic or operative intervention indicated	Life-threatening consequences; urgent intervention indicated	Death
Small intestine infection	-	Moderate symptoms; oral intervention indicated (e.g., antibiotic, antifungal, antiviral)	IV antibiotic, antifungal, or antiviral intervention indicated; radiologic or operative intervention indicated	Life-threatening consequences; urgent intervention indicated	Death

Soft tissue infection	-	Localized; local intervention indicated (e.g., topical antibiotic, antifungal, or antiviral)	IV antibiotic, antifungal, or antiviral intervention indicated; radiologic or operative intervention indicated	Life-threatening consequences; urgent intervention indicated	Death
Splenic infection	-	-	IV antibiotic, antifungal, or antiviral intervention indicated; radiologic or operative intervention indicated	Life-threatening consequences; urgent intervention indicated	Death
Stoma site infection	Localized, local intervention indicated	Oral intervention indicated (e.g., antibiotic, antifungal, antiviral)	IV antibiotic, antifungal, or antiviral intervention indicated; radiologic, endoscopic, or operative intervention indicated	Life-threatening consequences; urgent intervention indicated	Death
Tooth infection	-	Localized; local intervention indicated (e.g., topical antibiotic, antifungal, or antiviral)	IV antibiotic, antifungal, or antiviral intervention indicated; radiologic or operative intervention indicated	Life-threatening consequences; urgent intervention indicated	Death
Tracheitis	-	Moderate symptoms; oral intervention indicated (e.g., antibiotic, antifungal, antiviral)	IV antibiotic, antifungal, or antiviral intervention indicated; radiologic, endoscopic, or operative intervention indicated	Life-threatening consequences; urgent intervention indicated	Death
Upper respiratory infection	-	Moderate symptoms; oral intervention indicated (e.g., antibiotic, antifungal, antiviral)	IV antibiotic, antifungal, or antiviral intervention indicated; radiologic, endoscopic, or operative intervention indicated	Life-threatening consequences; urgent intervention indicated	Death
Urethral infection	-	Localized; local intervention indicated (e.g., topical antibiotic, antifungal, or antiviral)	IV antibiotic, antifungal, or antiviral intervention indicated; radiologic, endoscopic, or operative intervention indicated	Life-threatening consequences; urgent intervention indicated	Death
Urinary tract infection	-	Localized; local intervention indicated (e.g., topical antibiotic, antifungal, or antiviral)	IV antibiotic, antifungal, or antiviral intervention indicated; radiologic or operative intervention indicated	Life-threatening consequences; urgent intervention indicated	Death
Uterine infection	-	Moderate symptoms; oral intervention indicated (e.g., antibiotic, antifungal, antiviral)	IV antibiotic, antifungal, or antiviral intervention indicated; radiologic or operative intervention indicated	Life-threatening consequences; urgent intervention indicated	Death
Vaginal infection	-	Localized; local intervention indicated (e.g., topical antibiotic, antifungal, or antiviral)	IV antibiotic, antifungal, or antiviral intervention indicated; radiologic or operative intervention indicated	Life-threatening consequences; urgent intervention indicated	Death
Vulval infection	Localized, local intervention indicated	Oral intervention indicated (e.g., antibiotic, antifungal, antiviral)	IV antibiotic, antifungal, or antiviral intervention indicated; radiologic or operative intervention indicated	Life-threatening consequences; urgent intervention indicated	Death

Wound infection	-	Localized; local intervention indicated (e.g., topical antibiotic, antifungal, or antiviral)	IV antibiotic, antifungal, or antiviral intervention indicated; radiologic or operative intervention indicated	Life-threatening consequences; urgent intervention indicated	Death
Infections and infestations - Other, specify	Asymptomatic or mild symptoms; clinical or diagnostic observations only; intervention not indicated	Moderate; minimal, local or noninvasive intervention indicated; limiting age-appropriate instrumental ADL	Severe or medically significant but not immediately life-threatening; hospitalization or prolongation of existing hospitalization indicated; disabling; limiting self care ADL	Life-threatening consequences; urgent intervention indicated	Death
Injury, poisoning and procedural complications	Grade				
Adverse Event	1	2	3	4	5
Ankle fracture	Mild; non-surgical intervention indicated	Limiting instrumental ADL; operative intervention indicated	Limiting self care ADL; elective surgery indicated	-	-
Aortic injury	-	-	Severe symptoms; limiting self care ADL; disabling; repair or revision indicated	Life-threatening consequences; evidence of end organ damage; urgent operative intervention indicated	Death
Arterial injury	Asymptomatic diagnostic finding; intervention not indicated	Symptomatic (e.g., claudication); repair or revision not indicated	Severe symptoms; limiting self care ADL; disabling; repair or revision indicated	Life-threatening consequences; evidence of end organ damage; urgent operative intervention indicated	Death
Biliary anastomotic leak	Asymptomatic diagnostic observations only; intervention not indicated	Symptomatic; medical intervention indicated	Severe symptoms; radiologic, endoscopic or elective operative intervention indicated	Life-threatening consequences; urgent operative intervention indicated	Death
Bladder anastomotic leak	Asymptomatic diagnostic observations only; intervention not indicated	Symptomatic; medical intervention indicated	Severe symptoms; radiologic, endoscopic or elective operative intervention indicated	Life-threatening consequences; urgent operative intervention indicated	Death
Bruising	Localized or in a dependent area	Generalized	-	-	-
Burn	Minimal symptoms; intervention not indicated	Medical intervention; minimal debridement indicated	Moderate to major debridement or reconstruction indicated	Life-threatening consequences	Death
Dermatitis radiation	Faint erythema or dry desquamation	Moderate to brisk erythema; patchy moist desquamation, mostly confined to skin folds and creases; moderate edema	Moist desquamation in areas other than skin folds and creases; bleeding induced by minor trauma or abrasion	Life-threatening consequences; skin necrosis or ulceration of full thickness dermis; spontaneous bleeding from involved site; skin graft indicated	Death

Esophageal anastomotic leak	Asymptomatic diagnostic observations only; intervention not indicated	Symptomatic; medical intervention indicated	Severe symptoms; radiologic, endoscopic or elective operative intervention indicated	Life-threatening consequences; urgent operative intervention indicated	Death
Fall	Minor with no resultant injuries; intervention not indicated	Symptomatic; noninvasive intervention indicated	Hospitalization indicated	-	-
Fallopian tube anastomotic leak	Asymptomatic; clinical or diagnostic observations only; intervention not indicated	Symptomatic; medical intervention indicated	Severe symptoms; radiologic, endoscopic or elective operative intervention indicated	Life-threatening consequences; urgent operative intervention indicated	Death
Fallopian tube perforation	Asymptomatic diagnostic observations only; intervention not indicated	Symptomatic and intervention not indicated	Severe symptoms; elective operative intervention indicated	Life-threatening consequences; urgent operative intervention indicated (e.g., organ resection)	Death
Fracture	Asymptomatic; clinical or diagnostic observations only; intervention not indicated	Symptomatic but non-displaced; immobilization indicated	Severe symptoms; displaced or open wound with bone exposure; disabling; operative intervention indicated	Life-threatening consequences; urgent intervention indicated	Death
Gastric anastomotic leak	Asymptomatic diagnostic observations only; intervention not indicated	Symptomatic; medical intervention indicated	Severe symptoms; radiologic, endoscopic or elective operative intervention indicated	Life-threatening consequences; urgent operative intervention indicated	Death
Gastrointestinal anastomotic leak	Asymptomatic diagnostic observations only; intervention not indicated	Symptomatic; medical intervention indicated	Severe symptoms; radiologic, endoscopic or elective operative intervention indicated	Life-threatening consequences; urgent operative intervention indicated	Death
Gastrointestinal stoma necrosis	-	Superficial necrosis; intervention not indicated	Severe symptoms; hospitalization or elective operative intervention indicated	Life-threatening consequences; urgent intervention indicated	Death
Hip fracture	-	Hairline fracture; mild pain; limiting instrumental ADL; non-surgical intervention indicated	Severe pain; hospitalization or intervention indicated for pain control (e.g., traction); operative intervention indicated	Life-threatening consequences; symptoms associated with neurovascular compromise	-
Injury to carotid artery	-	-	Severe symptoms; limiting self care ADL (e.g., transient cerebral ischemia); repair or revision indicated	Life-threatening consequences; urgent intervention indicated	Death
Injury to inferior vena cava	-	-	-	Life-threatening consequences; urgent intervention indicated	Death
Injury to jugular vein	-	-	Symptomatic limiting self care ADL; disabling; repair or revision indicated	Life-threatening consequences; urgent intervention indicated	Death

Injury to superior vena cava	Asymptomatic diagnostic finding; intervention not indicated	Symptomatic; repair or revision not indicated	Severe symptoms; limiting self care ADL; disabling; repair or revision indicated	Life-threatening consequences; evidence of end organ damage; urgent operative intervention indicated	Death
Intestinal stoma leak	Asymptomatic diagnostic observations only; intervention not indicated	Symptomatic; medical intervention indicated	Severe symptoms; radiologic, endoscopic or elective operative intervention indicated	Life-threatening consequences; urgent operative intervention indicated	Death
Intestinal stoma obstruction	-	Self-limited; intervention not indicated	Severe symptoms; IV fluids, tube feeding, or TPN indicated >=24 hrs; elective operative intervention indicated	Life-threatening consequences; urgent operative intervention indicated	Death
Intestinal stoma site bleeding	Minimal bleeding identified on clinical exam; intervention not indicated	Moderate bleeding; medical intervention indicated	Severe bleeding; transfusion indicated; radiologic or endoscopic intervention indicated	Life-threatening consequences; urgent intervention indicated	Death
Intraoperative arterial injury	Primary repair of injured organ/structure indicated	Partial resection of injured organ/structure indicated	Complete resection or reconstruction of injured organ/structure indicated; disabling	Life-threatening consequences; urgent intervention indicated	Death
Intraoperative breast injury	Primary repair of injured organ/structure indicated	Partial resection of injured organ/structure indicated	Complete resection or reconstruction of injured organ/structure indicated; disabling	Life-threatening consequences; urgent intervention indicated	Death
Intraoperative cardiac injury	-	-	Primary repair of injured organ/structure indicated	Life-threatening consequences; urgent intervention indicated	Death
Intraoperative ear injury	Primary repair of injured organ/structure indicated	Partial resection of injured organ/structure indicated	Complete resection of injured organ/structure indicated; disabling (e.g., impaired hearing; impaired balance)	Life-threatening consequences; urgent intervention indicated	Death
Intraoperative endocrine injury	Primary repair of injured organ/structure indicated	Partial resection of injured organ/structure indicated	Complete resection or reconstruction of injured organ/structure indicated; disabling	Life-threatening consequences; urgent intervention indicated	Death
Intraoperative gastrointestinal injury	Primary repair of injured organ/structure indicated	Partial resection of injured organ/structure indicated	Complete resection or reconstruction of injured organ/structure indicated; disabling	Life-threatening consequences; urgent intervention indicated	Death
Intraoperative head and neck injury	Primary repair of injured organ/structure indicated	Partial resection of injured organ/structure indicated	Complete resection or reconstruction of injured organ/structure indicated; disabling	Life-threatening consequences; urgent intervention indicated	Death
Intraoperative hemorrhage	-	-	Postoperative radiologic, endoscopic, or operative intervention indicated	Life-threatening consequences; urgent intervention indicated	Death

Intraoperative hepatobiliary injury	Primary repair of injured organ/structure indicated	Partial resection of injured organ/structure indicated	Complete resection or reconstruction of injured organ/structure indicated; disabling	Life-threatening consequences; urgent intervention indicated	Death
Intraoperative musculoskeletal injury	Primary repair of injured organ/structure indicated	Partial resection of injured organ/structure indicated	Complete resection or reconstruction of injured organ/structure indicated; disabling	Life-threatening consequences; urgent intervention indicated	Death
Intraoperative neurological injury	Primary repair of injured organ/structure indicated	Partial resection of injured organ/structure indicated	Complete resection or reconstruction of injured organ/structure indicated; disabling	Life-threatening consequences; urgent intervention indicated	Death
Intraoperative ocular injury	Primary repair of injured organ/structure indicated	Partial resection of injured organ/structure indicated	Complete resection or reconstruction of injured organ/structure indicated; disabling	Life-threatening consequences; urgent intervention indicated	Death
Intraoperative renal injury	Primary repair of injured organ/structure indicated	Partial resection of injured organ/structure indicated	Complete resection or reconstruction of injured organ/structure indicated; disabling	Life-threatening consequences; urgent intervention indicated	Death
Intraoperative reproductive tract injury	Primary repair of injured organ/structure indicated	Partial resection of injured organ/structure indicated	Complete resection or reconstruction of injured organ/structure indicated; disabling	Life-threatening consequences; urgent intervention indicated	Death
Intraoperative respiratory injury	Primary repair of injured organ/structure indicated	Partial resection of injured organ/structure indicated	Complete resection or reconstruction of injured organ/structure indicated; disabling	Life-threatening consequences; urgent intervention indicated	Death
Intraoperative skin injury	Primary repair of injured organ/structure indicated	Partial resection of injured organ/structure indicated	Complete resection or reconstruction of injured organ/structure indicated; disabling	Life-threatening consequences; urgent intervention indicated	Death
Intraoperative splenic injury	-	Primary repair of injured organ/structure indicated	Resection or reconstruction of injured organ/structure indicated; disabling	Life-threatening consequences; urgent intervention indicated	Death
Intraoperative urinary injury	Primary repair of injured organ/structure indicated	Partial resection of injured organ/structure indicated	Complete resection or reconstruction of injured organ/structure indicated; disabling	Life-threatening consequences; urgent intervention indicated	Death
Intraoperative venous injury	Primary repair of injured organ/structure indicated	Partial resection of injured organ/structure indicated	Complete resection or reconstruction of injured organ/structure indicated; disabling	Life-threatening consequences; urgent intervention indicated	Death
Kidney anastomotic leak	Asymptomatic diagnostic observations only; intervention not indicated	Symptomatic; medical intervention indicated	Severe symptoms; radiologic, endoscopic or elective operative intervention indicated	Life-threatening consequences; urgent operative intervention indicated	Death

Large intestinal anastomotic leak	Asymptomatic diagnostic observations only; intervention not indicated	Symptomatic; medical intervention indicated	Severe symptoms; radiologic, endoscopic or elective operative intervention indicated	Life-threatening consequences; urgent operative intervention indicated	Death
Pancreatic anastomotic leak	Asymptomatic diagnostic observations only; intervention not indicated	Symptomatic; medical intervention indicated	Severe symptoms; radiologic, endoscopic or elective operative intervention indicated	Life-threatening consequences; urgent operative intervention indicated	Death
Pharyngeal anastomotic leak	Asymptomatic diagnostic observations only; intervention not indicated	Symptomatic; medical intervention indicated	Severe symptoms; radiologic, endoscopic or elective operative intervention indicated	Life-threatening consequences; urgent operative intervention indicated	Death
Postoperative hemorrhage	Minimal bleeding identified on clinical exam; intervention not indicated	Moderate bleeding; radiologic, endoscopic, or operative intervention indicated	Transfusion indicated of >=2 units (10 cc/kg for pediatrics) pRBCs beyond protocol specification; urgent radiologic, endoscopic, or operative intervention indicated	Life-threatening consequences; urgent intervention indicated	Death
Postoperative thoracic procedure complication	-	Extubated within 24 - 72 hrs postoperatively	Extubated >72 hrs postoperatively, but before tracheostomy indicated	Life-threatening airway compromise; urgent intervention indicated (e.g., tracheotomy or intubation)	Death
Prolapse of intestinal stoma	Asymptomatic; reducible	Recurrent after manual reduction; local irritation or stool leakage; difficulty to fit appliance; limiting instrumental ADL	Severe symptoms; elective operative intervention indicated; limiting self care ADL	Life-threatening consequences; urgent operative intervention indicated	Death
Prolapse of urostomy	Asymptomatic; clinical or diagnostic observations only; intervention not indicated	Local care or maintenance; minor revision indicated	Dysfunctional stoma; elective operative intervention or major stomal revision indicated	Life-threatening consequences; urgent intervention indicated	Death
Radiation recall reaction (dermatologic)	Faint erythema or dry desquamation	Moderate to brisk erythema; patchy moist desquamation, mostly confined to skin folds and creases; moderate edema	Moist desquamation in areas other than skin folds and creases; bleeding induced by minor trauma or abrasion	Life-threatening consequences; skin necrosis or ulceration of full thickness dermis; spontaneous bleeding from involved site; skin graft indicated	Death
Rectal anastomotic leak	Asymptomatic diagnostic observations only; intervention not indicated	Symptomatic; medical intervention indicated	Severe symptoms; radiologic, endoscopic or elective operative intervention indicated	Life-threatening consequences; urgent operative intervention indicated	Death
Seroma	Asymptomatic; clinical or diagnostic observations only; intervention not indicated	Symptomatic; simple aspiration indicated	Symptomatic, elective radiologic or operative intervention indicated	-	-
Small intestinal anastomotic leak	Asymptomatic diagnostic observations only; intervention not indicated	Symptomatic; medical intervention indicated	Severe symptoms; radiologic, endoscopic or elective operative intervention indicated	Life-threatening consequences; urgent operative intervention indicated	Death

Spermatic cord anastomotic leak	Asymptomatic diagnostic observations only; intervention not indicated	Symptomatic; medical intervention indicated	Severe symptoms; radiologic, endoscopic or elective operative intervention indicated	Life-threatening consequences; urgent operative intervention indicated	Death
Spinal fracture	Mild back pain; nonprescription analgesics indicated	Moderate back pain; prescription analgesics indicated; limiting instrumental ADL	Severe back pain; hospitalization or intervention indicated for pain control (e.g., vertebroplasty); limiting self care ADL; disability	Life-threatening consequences; symptoms associated with neurovascular compromise	Death
Stenosis of gastrointestinal stoma	-	Symptomatic; IV fluids indicated <24 hrs; manual dilation at bedside	Severely altered GI function; tube feeding, TPN or hospitalization indicated; elective operative intervention indicated	Life-threatening consequences; urgent operative intervention indicated	Death
Stomal ulcer	Asymptomatic; clinical or diagnostic observations only; intervention not indicated	Symptomatic; medical intervention indicated	Severe symptoms; elective operative intervention indicated	-	-
Tracheal hemorrhage	Minimal bleeding identified on clinical or diagnostic exam; intervention not indicated	Moderate bleeding; medical intervention indicated	Severe bleeding; transfusion indicated; radiologic or endoscopic intervention indicated	Life-threatening consequences; urgent intervention indicated	Death
Tracheal obstruction	Partial asymptomatic obstruction on examination (e.g., visual, radiologic or endoscopic)	Symptomatic (e.g., noisy airway breathing), no respiratory distress; medical intervention indicated (e.g., steroids); limiting instrumental ADL	Stridor; radiologic or endoscopic intervention indicated (e.g., stent, laser); limiting self care ADL	Life-threatening airway compromise; urgent intervention indicated (e.g., tracheotomy or intubation)	Death
Tracheostomy site bleeding	Minimal bleeding identified on clinical exam; intervention not indicated	Moderate bleeding; medical intervention indicated	Severe bleeding; transfusion indicated; radiologic or endoscopic intervention indicated	Life-threatening consequences; urgent intervention indicated	Death
Ureteric anastomotic leak	Asymptomatic diagnostic observations only; intervention not indicated	Symptomatic; medical intervention indicated	Severe symptoms; radiologic, endoscopic or elective operative intervention indicated	Life-threatening consequences; urgent operative intervention indicated	Death
Urethral anastomotic leak	Asymptomatic diagnostic observations only; intervention not indicated	Symptomatic; medical intervention indicated	Severe symptoms; radiologic, endoscopic or elective operative intervention indicated	Life-threatening consequences; urgent operative intervention indicated	Death
Urostomy leak	Asymptomatic diagnostic observations only; intervention not indicated	Symptomatic; medical intervention indicated	Severe symptoms; radiologic, endoscopic or elective operative intervention indicated	Life-threatening consequences; urgent operative intervention indicated	Death
Urostomy obstruction	Asymptomatic diagnostic observations only; intervention not indicated	Symptomatic; dilation or endoscopic repair or stent placement indicated	Altered organ function (e.g., sepsis or hydronephrosis, or renal dysfunction); elective operative intervention indicated	Life-threatening consequences; organ failure; urgent operative intervention indicated	Death

Urostomy site bleeding	Minimal bleeding identified on clinical exam; intervention not indicated	Moderate bleeding; medical intervention indicated	Severe bleeding; transfusion indicated; radiologic or endoscopic intervention indicated	Life-threatening consequences; urgent intervention indicated	Death
Urostomy stenosis	-	Symptomatic but no hydronephrosis, no sepsis or no renal dysfunction; dilation or endoscopic repair or stent placement indicated	Symptomatic (e.g., hydronephrosis, or renal dysfunction); elective operative intervention indicated	Life-threatening consequences; urgent operative intervention indicated	Death
Uterine anastomotic leak	Asymptomatic diagnostic observations only; intervention not indicated	Symptomatic; medical intervention indicated	Severe symptoms; radiologic, endoscopic or elective operative intervention indicated	Life-threatening consequences; urgent operative intervention indicated	Death
Uterine perforation	Asymptomatic diagnostic observations only; intervention not indicated	Symptomatic and intervention not indicated	Severe symptoms; elective operative intervention indicated	Life-threatening consequences; urgent intervention indicated	Death
Vaginal anastomotic leak	Asymptomatic diagnostic observations only; intervention not indicated	Symptomatic; medical intervention indicated	Severe symptoms; radiologic, endoscopic or elective operative intervention indicated	Life-threatening consequences; urgent operative intervention indicated	Death
Vas deferens anastomotic leak	Asymptomatic diagnostic observations only; intervention not indicated	Symptomatic; medical intervention indicated	Severe symptoms; radiologic, endoscopic or elective operative intervention indicated	Life-threatening consequences; urgent operative intervention indicated	Death
Vascular access complication	-	Device dislodgement, blockage, leak, or malposition; device replacement indicated	Deep vein or cardiac thrombosis; intervention indicated (e.g., anticoagulation, lysis, filter, invasive procedure)	Embolic event including pulmonary embolism or life-threatening thrombus	Death
Venous injury	Asymptomatic diagnostic finding; intervention not indicated	Symptomatic (e.g., claudication); repair or revision not indicated	Severe symptoms; limiting self care ADL; repair or revision indicated; disabling	Life-threatening consequences; evidence of end organ damage; urgent operative intervention indicated	Death
Wound complication	Incisional separation of <=25% of wound, no deeper than superficial fascia	Incisional separation >25% of wound; local care indicated	Hernia without evidence of strangulation; fascial disruption/dehiscence; primary wound closure or revision by operative intervention indicated	Hernia with evidence of strangulation; major reconstruction flap, grafting, resection, or amputation indicated	Death
Wound dehiscence	Incisional separation of <=25% of wound, no deeper than superficial fascia	Incisional separation >25% of wound with local care; asymptomatic hernia or symptomatic hernia without evidence of strangulation	Fascial disruption or dehiscence without evisceration; primary wound closure or revision by operative intervention indicated	Life-threatening consequences; symptomatic hernia with evidence of strangulation; fascial disruption with evisceration; major reconstruction flap, grafting, resection, or amputation indicated	Death

Wrist fracture	Mild; non-surgical intervention indicated	Limiting instrumental ADL; operative intervention indicated	Limiting self care ADL; elective surgery indicated	-	-
Injury, poisoning and procedural complications - Other, specify	Asymptomatic or mild symptoms; clinical or diagnostic observations only; intervention not indicated	Moderate; minimal, local or noninvasive intervention indicated; limiting age-appropriate instrumental ADL	Severe or medically significant but not immediately life-threatening; hospitalization or prolongation of existing hospitalization indicated; disabling; limiting self care ADL	Life-threatening consequences; urgent intervention indicated	Death
Investigations	Grade				
Adverse Event	1	2	3	4	5
Activated partial thromboplastin time prolonged	>ULN - 1.5 x ULN	>1.5 - 2.5 x ULN	>2.5 x ULN; hemorrhage	-	-
Alanine aminotransferase increased	>ULN - 3.0 x ULN	>3.0 - 5.0 x ULN	>5.0 - 20.0 x ULN	>20.0 x ULN	-
Alkaline phosphatase increased	>ULN - 2.5 x ULN	>2.5 - 5.0 x ULN	>5.0 - 20.0 x ULN	>20.0 x ULN	-
Aspartate aminotransferase increased	>ULN - 3.0 x ULN	>3.0 - 5.0 x ULN	>5.0 - 20.0 x ULN	>20.0 x ULN	-
Blood antidiuretic hormone abnormal	Asymptomatic; clinical or diagnostic observations only; intervention not indicated	Symptomatic; medical intervention indicated	Hospitalization indicated	-	-
Blood bilirubin increased	>ULN - 1.5 x ULN	>1.5 - 3.0 x ULN	>3.0 - 10.0 x ULN	>10.0 x ULN	-
Blood corticotrophin decreased	Asymptomatic; clinical or diagnostic observations only; intervention not indicated	Symptomatic; medical intervention indicated	Hospitalization indicated	-	-
Blood gonadotrophin abnormal	Asymptomatic; clinical or diagnostic observations only; intervention not indicated	Symptomatic; medical intervention indicated; limiting instrumental ADL	Severe symptoms; limiting self care ADL	-	-
Blood prolactin abnormal	Asymptomatic; clinical or diagnostic observations only; intervention not indicated	Moderate symptoms; limiting instrumental ADL	-	-	-
Carbon monoxide diffusing capacity decreased	3 - 5 units below LLN; for follow-up, a decrease of 3 - 5 units (ml/min/mm Hg) below the baseline value	6 - 8 units below LLN; for follow-up, an asymptomatic decrease of >5 - 8 units (ml/min/mm Hg) below the baseline value	Asymptomatic decrease of >8 units drop; >5 units drop along with the presence of pulmonary symptoms (e.g. , >Grade 2 hypoxia or >Grade 2 or higher dyspnea)	-	-

Cardiac troponin I increased	Levels above the upper limit of normal and below the level of myocardial infarction as defined by the manufacturer	-	Levels consistent with myocardial infarction as defined by the manufacturer	-	-
Cardiac troponin T increased	Levels above the upper limit of normal and below the level of myocardial infarction as defined by the manufacturer	-	Levels consistent with myocardial infarction as defined by the manufacturer	-	-
CD4 lymphocytes decreased	<LLN - 500/mm3; <LLN - 0.5 x 10e9 /L	<500 - 200/mm3; <0.5 - 0.2 x 10e9 /L	<200 - 50/mm3; <0.2 x 0.05 - 10e9 /L	<50/mm3; <0.05 x 10e9 /L	-
Cholesterol high	>ULN - 300 mg/dL; >ULN - 7.75 mmol/L	>300 - 400 mg/dL; >7.75 - 10.34 mmol/L	>400 - 500 mg/dL; >10.34 - 12.92 mmol/L	>500 mg/dL; >12.92 mmol/L	-
CPK increased	>ULN - 2.5 x ULN	>2.5 x ULN - 5 x ULN	>5 x ULN - 10 x ULN	>10 x ULN	-
Creatinine increased	>1 - 1.5 x baseline; >ULN - 1.5 x ULN	>1.5 - 3.0 x baseline; >1.5 - 3.0 x ULN	>3.0 baseline; >3.0 - 6.0 x ULN	>6.0 x ULN	-
Ejection fraction decreased	-	Resting ejection fraction (EF) 50 - 40%; 10 - 19% drop from baseline	Resting ejection fraction (EF) 39 - 20%; >20% drop from baseline	Resting ejection fraction (EF) <20%	-
Electrocardiogram QT corrected interval prolonged	QTc 450 - 480 ms	QTc 481 - 500 ms	QTc >= 501 ms on at least two separate ECGs	QTc >= 501 or >60 ms change from baseline and Torsade de pointes or polymorphic ventricular tachycardia or signs/symptoms of serious arrhythmia	-
Fibrinogen decreased	<1.0 - 0.75 x LLN or <25% decrease from baseline	<0.75 - 0.5 x LLN or 25 - <50% decrease from baseline	<0.5 - 0.25 x LLN or 50 - <75% decrease from baseline	<0.25 x LLN or 75% decrease from baseline or absolute value <50 mg/dL	-
Forced expiratory volume decreased	FEV1% (percentages of observed FEV1 and FVC related to their respective predicted values) 99 - 70% predicted	FEV1 60 - 69%	50 - 59%	<= 49%	-
GGT increased	>ULN - 2.5 x ULN	>2.5 - 5.0 x ULN	>5.0 - 20.0 x ULN	>20.0 x ULN	-
Growth hormone abnormal	Asymptomatic; clinical or diagnostic observations only; intervention not indicated	Symptomatic; medical intervention indicated; limiting instrumental ADL	-	-	-
Haptoglobin decreased	<LLN	-	-	-	-

Hemoglobin increased	Increase in >0 - 2 gm/dL above ULN or above baseline if baseline is above ULN	Increase in >2 - 4 gm/dL above ULN or above baseline if baseline is above ULN	Increase in >4 gm/dL above ULN or above baseline if baseline is above ULN	-	-
INR increased	>1 - 1.5 x ULN; >1 - 1.5 times above baseline if on anticoagulation	>1.5 - 2.5 x ULN; >1.5 - 2.5 times above baseline if on anticoagulation	>2.5 x ULN; >2.5 times above baseline if on anticoagulation	-	-
Lipase increased	>ULN - 1.5 x ULN	>1.5 - 2.0 x ULN	>2.0 - 5.0 x ULN	>5.0 x ULN	-
Lymphocyte count decreased	<LLN - 800/mm3; <LLN - 0.8 x 10e9/L	<800 - 500/mm3; <0.8 - 0.5 x 10e9 /L	<500 - 200/mm3; <0.5 - 0.2 x 10e9 /L	<200/mm3; <0.2 x 10e9 /L	-
Lymphocyte count increased	-	>4000/mm3 - 20,000/mm3	>20,000/mm3	-	-
Neutrophil count decreased	<LLN - 1500/mm3; <LLN - 1.5 x 10e9 /L	<1500 - 1000/mm3; <1.5 - 1.0 x 10e9 /L	<1000 - 500/mm3; <1.0 - 0.5 x 10e9 /L	<500/mm3; <0.5 x 10e9 /L	-
Pancreatic enzymes decreased	<LLN and asymptomatic	Increase in stool frequency, bulk, or odor; steatorrhea	Sequelae of absorption deficiency	-	-
Platelet count decreased	<LLN - 75,000/mm3; <LLN - 75.0 x 10e9 /L	<75,000 - 50,000/mm3; <75.0 - 50.0 x 10e9 /L	<50,000 - 25,000/mm3; <50.0 - 25.0 x 10e9 /L	<25,000/mm3; <25.0 x 10e9 /L	-
Serum amylase increased	>ULN - 1.5 x ULN	>1.5 - 2.0 x ULN	>2.0 - 5.0 x ULN	>5.0 x ULN	-
Urine output decreased	-	-	Oliguria (<80 ml in 8 hr)	Anuria (<240 ml in 24 hr)	-
Vital capacity abnormal	90 - 75% of predicted value	<75 - 50% of predicted value; limiting instrumental ADL	<50% of predicted value; limiting self care ADL	-	-
Weight gain	5 - <10% from baseline	10 - <20% from baseline	>=20% from baseline	-	-
Weight loss	5 to <10% from baseline; intervention not indicated	10 - <20% from baseline; nutritional support indicated	>=20% from baseline; tube feeding or TPN indicated	-	-
White blood cell decreased	<LLN - 3000/mm3; <LLN - 3.0 x 10e9 /L	<3000 - 2000/mm3; <3.0 - 2.0 x 10e9 /L	<2000 - 1000/mm3; <2.0 - 1.0 x 10e9 /L	<1000/mm3; <1.0 x 10e9 /L	-
Investigations - Other, specify	Asymptomatic or mild symptoms; clinical or diagnostic observations only; intervention not indicated	Moderate; minimal, local or noninvasive intervention indicated; limiting age-appropriate instrumental ADL	Severe or medically significant but not immediately life-threatening; hospitalization or prolongation of existing hospitalization indicated; disabling; limiting self care ADL	Life-threatening consequences; urgent intervention indicated	Death
Metabolism and nutrition disorders	Grade				
Adverse Event	1	2	3	4	5

Acidosis	pH <normal, but **>=**7.3	-	pH <7.3	Life-threatening consequences	Death
Alcohol intolerance	-	Present	Severe symptoms; limiting self care ADL	Life-threatening consequences; urgent intervention indicated	Death
Alkalosis	pH >normal, but <=7.5	-	pH >7.5	Life-threatening consequences	Death
Anorexia	Loss of appetite without alteration in eating habits	Oral intake altered without significant weight loss or malnutrition; oral nutritional supplements indicated	Associated with significant weight loss or malnutrition (e.g., inadequate oral caloric and/or fluid intake); tube feeding or TPN indicated	Life-threatening consequences; urgent intervention indicated	Death
Dehydration	Increased oral fluids indicated; dry mucous membranes; diminished skin turgor	IV fluids indicated <24 hrs	IV fluids or hospitalization indicated	Life-threatening consequences; urgent intervention indicated	Death
Glucose intolerance	Asymptomatic; clinical or diagnostic observations only; intervention not indicated	Symptomatic; dietary modification or oral agent indicated	Severe symptoms; insulin indicated	Life-threatening consequences; urgent intervention indicated	Death
Hypercalcemia	Corrected serum calcium of >ULN - 11.5 mg/dL; >ULN - 2.9 mmol/L; Ionized calcium >ULN - 1.5 mmol/L	Corrected serum calcium of >11.5 - 12.5 mg/dL; >2.9 - 3.1 mmol/L; Ionized calcium >1.5 - 1.6 mmol/L; symptomatic	Corrected serum calcium of >12.5 - 13.5 mg/dL;>3.1 - 3.4 mmol/L; Ionized calcium >1.6 - 1.8 mmol/L; hospitalization indicated	Corrected serum calcium of >13.5 mg/dL; >3.4 mmol/L; Ionized calcium >1.8 mmol/L; life-threatening consequences	Death
Hyperglycemia	Fasting glucose value >ULN - 160 mg/dL; Fasting glucose value >ULN - 8.9 mmol/L	Fasting glucose value >160 - 250 mg/dL; Fasting glucose value >8.9 - 13.9 mmol/L	>250 - 500 mg/dL; >13.9 - 27.8 mmol/L; hospitalization indicated	>500 mg/dL; >27.8 mmol/L; life-threatening consequences	Death
Hyperkalemia	>ULN - 5.5 mmol/L	>5.5 - 6.0 mmol/L	>6.0 - 7.0 mmol/L; hospitalization indicated	>7.0 mmol/L; life-threatening consequences	Death
Hypermagnesemia	>ULN - 3.0 mg/dL; >ULN - 1.23 mmol/L	-	>3.0 - 8.0 mg/dL; >1.23 - 3.30 mmol/L	>8.0 mg/dL; >3.30 mmol/L; life-threatening consequences	Death
Hypernatremia	>ULN - 150 mmol/L	>150 - 155 mmol/L	>155 - 160 mmol/L; hospitalization indicated	>160 mmol/L; life-threatening consequences	Death
Hypertriglyceridemia	150 mg/dL - 300 mg/dL; 1.71 mmol/L - 3.42 mmol/L	>300 mg/dL - 500 mg/dL; >3.42 mmol/L - 5.7 mmol/L	>500 mg/dL - 1000 mg/dL; >5.7 mmol/L - 11.4 mmol/L	>1000 mg/dL; >11.4 mmol/L; life-threatening consequences	Death

Hyperuricemia	>ULN - 10 mg/dL (0.59 mmol/L) without physiologic consequences	-	>ULN - 10 mg/dL (0.59 mmol/L) with physiologic consequences	>10 mg/dL; >0.59 mmol/L; life-threatening consequences	Death
Hypoalbuminemia	<LLN - 3 g/dL; <LLN - 30 g/L	<3 - 2 g/dL; <30 - 20 g/L	<2 g/dL; <20 g/L	Life-threatening consequences; urgent intervention indicated	Death
Hypocalcemia	Corrected serum calcium of <LLN - 8.0 mg/dL; <LLN - 2.0 mmol/L; Ionized calcium <LLN - 1.0 mmol/L	Corrected serum calcium of <8.0 - 7.0 mg/dL; <2.0 - 1.75 mmol/L; Ionized calcium <1.0 - 0.9 mmol/L; symptomatic	Corrected serum calcium of <7.0 - 6.0 mg/dL; <1.75 - 1.5 mmol/L; Ionized calcium <0.9 - 0.8 mmol/L; hospitalization indicated	Corrected serum calcium of <6.0 mg/dL; <1.5 mmol/L; Ionized calcium <0.8 mmol/L; life-threatening consequences	Death
Hypoglycemia	<LLN - 55 mg/dL; <LLN - 3.0 mmol/L	<55 - 40 mg/dL; <3.0 - 2.2 mmol/L	<40 - 30 mg/dL; <2.2 - 1.7 mmol/L	<30 mg/dL; <1.7 mmol/L; life-threatening consequences; seizures	Death
Hypokalemia	<LLN - 3.0 mmol/L	<LLN - 3.0 mmol/L; symptomatic; intervention indicated	<3.0 - 2.5 mmol/L; hospitalization indicated	<2.5 mmol/L; life-threatening consequences	Death
Hypomagnesemia	<LLN - 1.2 mg/dL; <LLN - 0.5 mmol/L	<1.2 - 0.9 mg/dL; <0.5 - 0.4 mmol/L	<0.9 - 0.7 mg/dL; <0.4 - 0.3 mmol/L	<0.7 mg/dL; <0.3 mmol/L; life-threatening consequences	Death
Hyponatremia	<LLN - 130 mmol/L	-	<130 - 120 mmol/L	<120 mmol/L; life-threatening consequences	Death
Hypophosphatemia	<LLN - 2.5 mg/dL; <LLN - 0.8 mmol/L	<2.5 - 2.0 mg/dL; <0.8 - 0.6 mmol/L	<2.0 - 1.0 mg/dL; <0.6 - 0.3 mmol/L	<1.0 mg/dL; <0.3 mmol/L; life-threatening consequences	Death
Iron overload	-	Moderate symptoms; intervention not indicated	Severe symptoms; intervention indicated	Life-threatening consequences; urgent intervention indicated	Death
Obesity	-	BMI 25 - 29.9 kg/m2	BMI 30 - 39.9 kg/m2	BMI >=40 kg/m2	
Tumor lysis syndrome	-	-	Present	Life-threatening consequences; urgent intervention indicated	Death
Metabolism and nutrition disorders - Other, specify	Asymptomatic or mild symptoms; clinical or diagnostic observations only; intervention not indicated	Moderate; minimal, local or noninvasive intervention indicated; limiting age-appropriate instrumental ADL	Severe or medically significant but not immediately life-threatening; hospitalization or prolongation of existing hospitalization indicated; disabling; limiting self care ADL	Life-threatening consequences; urgent intervention indicated	Death
Musculoskeletal and connective tissue	Grade				

disorders					
Adverse Event	1	2	3	4	5
Abdominal soft tissue necrosis	-	Local wound care; medical intervention indicated (e.g., dressings or topical medications)	Operative debridement or other invasive intervention indicated (e.g. tissue reconstruction, flap or grafting)	Life-threatening consequences; urgent intervention indicated	Death
Arthralgia	Mild pain	Moderate pain; limiting instrumental ADL	Severe pain; limiting self care ADL	-	-
Arthritis	Mild pain with inflammation, erythema, or joint swelling	Moderate pain associated with signs of inflammation, erythema, or joint swelling; limiting instrumental ADL	Severe pain associated with signs of inflammation, erythema, or joint swelling; irreversible joint damage; disabling; limiting self care ADL	-	-
Avascular necrosis	Asymptomatic; clinical or diagnostic observations only; intervention not indicated	Symptomatic; limiting instrumental ADL	Severe symptoms; limiting self care ADL; elective operative intervention indicated	Life-threatening consequences; urgent intervention indicated	Death
Back pain	Mild pain	Moderate pain; limiting instrumental ADL	Severe pain; limiting self care ADL	-	-
Bone pain	Mild pain	Moderate pain; limiting instrumental ADL	Severe pain; limiting self care ADL	-	-
Buttock pain	Mild pain	Moderate pain; limiting instrumental ADL	Severe pain; limiting self care ADL	-	-
Chest wall pain	Mild pain	Moderate pain; limiting instrumental ADL	Severe pain; limiting self care ADL	-	-
Exostosis	Asymptomatic; clinical or diagnostic observations only; intervention not indicated	Symptomatic; limiting instrumental ADL	Severe symptoms; limiting self care ADL; elective operative intervention indicated	-	-
Fibrosis deep connective tissue	Mild induration, able to move skin parallel to plane (sliding) and perpendicular to skin (pinching up)	Moderate induration, able to slide skin, unable to pinch skin; limiting instrumental ADL	Severe induration; unable to slide or pinch skin; limiting joint or orifice movement (e.g. mouth, anus); limiting self care ADL	Generalized; associated with signs or symptoms of impaired breathing or feeding	Death
Flank pain	Mild pain	Moderate pain; limiting instrumental ADL	Severe pain; limiting self care ADL	-	-

Generalized muscle weakness	Symptomatic; weakness perceived by patient but not evident on physical exam	Symptomatic; weakness evident on physical exam; weakness limiting instrumental ADL	Weakness limiting self care ADL; disabling	-	-
Growth suppression	Reduction in growth velocity by 10 - 29% ideally measured over the period of a year	Reduction in growth velocity by 30 - 49% ideally measured over the period of a year or 0 - 49% reduction in growth from the baseline growth curve	Reduction in growth velocity of >=50% ideally measured over the period of a year	-	-
Head soft tissue necrosis	-	Local wound care; medical intervention indicated (e.g., dressings or topical medications)	Operative debridement or other invasive intervention indicated (e.g., tissue reconstruction, flap or grafting)	Life-threatening consequences; urgent intervention indicated	Death
Joint effusion	Asymptomatic; clinical or diagnostic observations only; intervention not indicated	Symptomatic; limiting instrumental ADL	Severe symptoms; limiting self care ADL; elective operative intervention indicated; disabling	-	-
Joint range of motion decreased	<=25% loss of ROM (range of motion); decreased ROM limiting athletic activity	>25 - 50% decrease in ROM; limiting instrumental ADL	>50% decrease in ROM; limiting self care ADL; disabling	-	-
Joint range of motion decreased cervical spine	Mild restriction of rotation or flexion between 60 - 70 degrees	Rotation <60 degrees to right or left; <60 degrees of flexion	Ankylosed/fused over multiple segments with no C-spine rotation	-	-
Joint range of motion decreased lumbar spine	Stiffness; difficulty bending to the floor to pick up a very light object but able to do athletic activity	Pain with range of motion (ROM) in lumbar spine; requires a reaching aid to pick up a very light object from the floor	<50% lumbar spine flexion; associated with symptoms of ankylosis or fused over multiple segments with no L-spine flexion (e.g., unable to reach to floor to pick up a very light object)	-	-
Kyphosis	Asymptomatic; clinical or diagnostic observations only; intervention not indicated	Moderate accentuation; limiting instrumental ADL	Severe accentuation; operative intervention indicated; limiting self care ADL	-	-
Lordosis	Asymptomatic; clinical or diagnostic observations only; intervention not indicated	Moderate accentuation; limiting instrumental ADL	Severe accentuation; operative intervention indicated; limiting self care ADL	-	-
Muscle weakness left-sided	Symptomatic; perceived by patient but not evident on physical exam	Symptomatic; evident on physical exam; limiting instrumental ADL	Limiting self care ADL; disabling	-	-
Muscle weakness lower limb	Symptomatic; perceived by patient but not evident on physical exam	Symptomatic; evident on physical exam; limiting instrumental ADL	Limiting self care ADL; disabling	-	-

Muscle weakness right-sided	Symptomatic; perceived by patient but not evident on physical exam	Symptomatic; evident on physical exam; limiting instrumental ADL	Limiting self care ADL; disabling	-	-
Muscle weakness trunk	Symptomatic; perceived by patient but not evident on physical exam	Symptomatic; evident on physical exam; limiting instrumental ADL	Limiting self care ADL; disabling	-	-
Muscle weakness upper limb	Symptomatic; perceived by patient but not evident on physical exam	Symptomatic; evident on physical exam; limiting instrumental ADL	Limiting self care ADL; disabling	-	-
Musculoskeletal deformity	Cosmetically and functionally insignificant hypoplasia	Deformity, hypoplasia, or asymmetry able to be remediated by prosthesis (e.g., shoe insert) or covered by clothing	Significant deformity, hypoplasia, or asymmetry, unable to be remediated by prosthesis or covered by clothing; disabling	-	-
Myalgia	Mild pain	Moderate pain; limiting instrumental ADL	Severe pain; limiting self care ADL	-	-
Myositis	Mild pain	Moderate pain associated with weakness; pain limiting instrumental ADL	Pain associated with severe weakness; limiting self care ADL	-	-
Neck pain	Mild pain	Moderate pain; limiting instrumental ADL	Severe pain; limiting self care ADL	-	-
Neck soft tissue necrosis	-	Local wound care; medical intervention indicated (e.g., dressings or topical medications)	Operative debridement or other invasive intervention indicated (e.g., tissue reconstruction, flap or grafting)	Life-threatening consequences; urgent intervention indicated	Death
Osteonecrosis of jaw	Asymptomatic; clinical or diagnostic observations only; intervention not indicated	Symptomatic; medical intervention indicated (e.g., topical agents); limiting instrumental ADL	Severe symptoms; limiting self care ADL; elective operative intervention indicated	Life-threatening consequences; urgent intervention indicated	Death
Osteoporosis	Radiologic evidence of osteoporosis or Bone Mineral Density (BMD) t-score -1 to -2.5 (osteopenia); no loss of height or intervention indicated	BMD t-score <-2.5; loss of height <2 cm; anti-osteoporotic therapy indicated; limiting instrumental ADL	Loss of height >=2 cm; hospitalization indicated; limiting self care ADL	-	-
Pain in extremity	Mild pain	Moderate pain; limiting instrumental ADL	Severe pain; limiting self care ADL	-	-

Pelvic soft tissue necrosis	-	Local wound care; medical intervention indicated (e.g., dressings or topical medications)	Operative debridement or other invasive intervention indicated (e.g., tissue reconstruction, flap or grafting)	Life-threatening consequences; urgent intervention indicated	Death
Scoliosis	<20 degrees; clinically undetectable	>20 - 45 degrees; visible by forward flexion; limiting instrumental ADL	>45 degrees; scapular prominence in forward flexion; operative intervention indicated; limiting self care ADL; disabling	-	-
Soft tissue necrosis lower limb	-	Local wound care; medical intervention indicated (e.g., dressings or topical medications)	Operative debridement or other invasive intervention indicated (e.g., tissue reconstruction, flap or grafting)	Life-threatening consequences; urgent intervention indicated	Death
Soft tissue necrosis upper limb	-	Local wound care; medical intervention indicated (e.g., dressings or topical medications)	Operative debridement or other invasive intervention indicated (e.g., tissue reconstruction, flap or grafting)	Life-threatening consequences; urgent intervention indicated	Death
Superficial soft tissue fibrosis	Mild induration, able to move skin parallel to plane (sliding) and perpendicular to skin (pinching up)	Moderate induration, able to slide skin, unable to pinch skin; limiting instrumental ADL	Severe induration; unable to slide or pinch skin; limiting joint or orifice movement (e.g., mouth, anus); limiting self care ADL	Generalized; associated with signs or symptoms of impaired breathing or feeding	Death
Trismus	Decreased ROM (range of motion) without impaired eating	Decreased ROM requiring small bites, soft foods or purees	Decreased ROM with inability to adequately aliment or hydrate orally	-	-
Unequal limb length	Mild length discrepancy <2 cm	Moderate length discrepancy 2 - 5 cm; shoe lift indicated; limiting instrumental ADL	Severe length discrepancy >5 cm; limiting self care ADL; disabling; operative intervention indicated	-	-
Musculoskeletal and connective tissue disorder - Other, specify	Asymptomatic or mild symptoms; clinical or diagnostic observations only; intervention not indicated	Moderate; minimal, local or noninvasive intervention indicated; limiting age-appropriate instrumental ADL	Severe or medically significant but not immediately life-threatening; hospitalization or prolongation of existing hospitalization indicated; disabling; limiting self care ADL	Life-threatening consequences; urgent intervention indicated	Death
Neoplasms benign, malignant and unspecified (incl cysts and polyps)	Grade				
Adverse Event	1	2	3	4	5
Leukemia secondary to oncology chemotherapy	-	-	-	Present	Death
Myelodysplastic syndrome	-	-	-	Life-threatening consequences; urgent intervention indicated	Death

Treatment related secondary malignancy	-	-	Non life-threatening secondary malignancy	Acute life-threatening secondary malignancy; blast crisis in leukemia	Death
Tumor pain	Mild pain	Moderate pain; limiting instrumental ADL	Severe pain; limiting self care ADL	-	-
Neoplasms benign, malignant and unspecified (incl cysts and polyps) - Other, specify	Asymptomatic or mild symptoms; clinical or diagnostic observations only; intervention not indicated	Moderate; minimal, local or noninvasive intervention indicated; limiting age-appropriate instrumental ADL	Severe or medically significant but not immediately life-threatening; hospitalization or prolongation of existing hospitalization indicated; disabling; limiting self care ADL	Life-threatening consequences; urgent intervention indicated	Death
Nervous system disorders	Grade				
Adverse Event	1	2	3	4	5
Abducens nerve disorder	Asymptomatic; clinical or diagnostic observations only; intervention not indicated	Moderate symptoms; limiting instrumental ADL	Severe symptoms; limiting self care ADL	-	-
Accessory nerve disorder	Asymptomatic; clinical or diagnostic observations only; intervention not indicated	Moderate symptoms; limiting instrumental ADL	Severe symptoms; limiting self care ADL	-	-
Acoustic nerve disorder NOS	Asymptomatic; clinical or diagnostic observations only; intervention not indicated	Moderate symptoms; limiting instrumental ADL	Severe symptoms; limiting self care ADL	-	-
Akathisia	Mild restlessness or increased motor activity	Moderate restlessness or increased motor activity; limiting instrumental ADL	Severe restlessness or increased motor activity; limiting self care ADL	-	-
Amnesia	Mild; transient memory loss	Moderate; short term memory loss; limiting instrumental ADL	Severe; long term memory loss; limiting self care ADL	-	-
Aphonia	-	-	Voicelessness; unable to speak	-	-
Arachnoiditis	Mild symptoms	Moderate symptoms; limiting instrumental ADL	Severe symptoms; limiting self care ADL	Life-threatening consequences; urgent intervention indicated	Death
Ataxia	Asymptomatic; clinical or diagnostic observations only; intervention not indicated	Moderate symptoms; limiting instrumental ADL	Severe symptoms; limiting self care ADL; mechanical assistance indicated	-	-
Brachial plexopathy	Asymptomatic; clinical or diagnostic observations only; intervention not indicated	Moderate symptoms; limiting instrumental ADL	Severe symptoms; limiting self care ADL	-	-
Central nervous system necrosis	Asymptomatic; clinical or diagnostic observations only; intervention not indicated	Moderate symptoms; corticosteroids indicated	Severe symptoms; medical intervention indicated	Life-threatening consequences; urgent intervention indicated	Death

Cerebrospinal fluid leakage	Post-craniotomy: asymptomatic; Post-lumbar puncture: transient headache; postural care indicated	Post-craniotomy: moderate symptoms; medical intervention indicated; Post-lumbar puncture: persistent moderate symptoms; blood patch indicated	Severe symptoms; medical intervention indicated	Life-threatening consequences; urgent intervention indicated	Death
Cognitive disturbance	Mild cognitive disability; not interfering with work/school/life performance; specialized educational services/devices not indicated	Moderate cognitive disability; interfering with work/school/life performance but capable of independent living; specialized resources on part time basis indicated	Severe cognitive disability; significant impairment of work/school/life performance	-	-
Concentration impairment	Mild inattention or decreased level of concentration	Moderate impairment in attention or decreased level of concentration; limiting instrumental ADL	Severe impairment in attention or decreased level of concentration; limiting self care ADL	-	-
Depressed level of consciousness	Decreased level of alertness	Sedation; slow response to stimuli; limiting instrumental ADL	Difficult to arouse	Life-threatening consequences	Death
Dizziness	Mild unsteadiness or sensation of movement	Moderate unsteadiness or sensation of movement; limiting instrumental ADL	Severe unsteadiness or sensation of movement; limiting self care ADL	-	-
Dysarthria	Mild slurred speech	Moderate impairment of articulation or slurred speech	Severe impairment of articulation or slurred speech	-	-
Dysesthesia	Mild sensory alteration	Moderate sensory alteration; limiting instrumental ADL	Severe sensory alteration; limiting self care ADL	-	-
Dysgeusia	Altered taste but no change in diet	Altered taste with change in diet (e.g., oral supplements); noxious or unpleasant taste; loss of taste	-	-	-
Dysphasia	Awareness of receptive or expressive characteristics; not impairing ability to communicate	Moderate receptive or expressive characteristics; impairing ability to communicate spontaneously	Severe receptive or expressive characteristics; impairing ability to read, write or communicate intelligibly	-	-
Edema cerebral	-	-	-	Life-threatening consequences; urgent intervention indicated	
Encephalopathy	Mild symptoms	Moderate symptoms; limiting instrumental ADL	Severe symptoms; limiting self care ADL	Life-threatening consequences; urgent intervention indicated	Death
Extrapyramidal disorder	Mild involuntary movements	Moderate involuntary movements; limiting instrumental ADL	Severe involuntary movements or torticollis; limiting self care ADL	Life-threatening consequences; urgent intervention indicated	Death
Facial muscle weakness	Asymptomatic; clinical or diagnostic observations only; intervention not indicated	Moderate symptoms; limiting instrumental ADL	Severe symptoms; limiting self care ADL	-	-

Facial nerve disorder	Asymptomatic; clinical or diagnostic observations only; intervention not indicated	Moderate symptoms; limiting instrumental ADL	Severe symptoms; limiting self care ADL	-	-
Glossopharyngeal nerve disorder	Asymptomatic; clinical or diagnostic observations only; intervention not indicated	Moderate symptoms; limiting instrumental ADL	Severe symptoms; limiting self care ADL	Life-threatening consequences; urgent intervention indicated	Death
Headache	Mild pain	Moderate pain; limiting instrumental ADL	Severe pain; limiting self care ADL	-	-
Hydrocephalus	Asymptomatic; clinical or diagnostic observations only; intervention not indicated	Moderate symptoms; intervention not indicated	Severe symptoms or neurological deficit; intervention indicated	Life-threatening consequences; urgent intervention indicated	Death
Hypersomnia	Mild increased need for sleep	Moderate increased need for sleep	Severe increased need for sleep	-	-
Hypoglossal nerve disorder	Asymptomatic; clinical or diagnostic observations only; intervention not indicated	Moderate symptoms; limiting instrumental ADL	Severe symptoms; limiting self care ADL	-	-
Intracranial hemorrhage	Asymptomatic; clinical or diagnostic observations only; intervention not indicated	Moderate symptoms; medical intervention indicated	Ventriculostomy, ICP monitoring, intraventricular thrombolysis, or operative intervention indicated	Life-threatening consequences; urgent intervention indicated	Death
Ischemia cerebrovascular	Asymptomatic; clinical or diagnostic observations only; intervention not indicated	Moderate symptoms	-	-	-
IVth nerve disorder	Asymptomatic; clinical or diagnostic observations only; intervention not indicated	Moderate symptoms; limiting instrumental ADL	Severe symptoms; limiting self care ADL	-	-
Lethargy	Mild symptoms; reduced alertness and awareness	Moderate symptoms; limiting instrumental ADL	-	-	-
Leukoencephalopathy	Asymptomatic; small focal T2/FLAIR hyperintensities; involving periventricular white matter or <1/3 of susceptible areas of cerebrum +/- mild increase in subarachnoid space (SAS) and/or mild ventriculomegaly	Moderate symptoms; focal T2/FLAIR hyperintensities, involving periventricular white matter extending into centrum semiovale or involving 1/3 to 2/3 of susceptible areas of cerebrum +/- moderate increase in SAS and/or moderate ventriculomegaly	Severe symptoms; extensive T2/FLAIR hyperintensities, involving periventricular white matter involving 2/3 or more of susceptible areas of cerebrum +/- moderate to severe increase in SAS and/or moderate to severe ventriculomegaly	Life-threatening consequences; extensive T2/FLAIR hyperintensities, involving periventricular white matter involving most of susceptible areas of cerebrum +/- moderate to severe increase in SAS and/or moderate to severe ventriculomegaly	Death
Memory impairment	Mild memory impairment	Moderate memory impairment; limiting instrumental ADL	Severe memory impairment; limiting self care ADL	-	-
Meningismus	Mild symptoms	Moderate symptoms; limiting instrumental ADL	Severe symptoms; limiting self care ADL	Life-threatening consequences; urgent intervention indicated	Death

Movements involuntary	Mild symptoms	Moderate symptoms; limiting instrumental ADL	Severe symptoms; limiting self care ADL	-	-
Myelitis	Asymptomatic; mild signs (e.g., Babinski's reflex or Lhermitte's sign)	Moderate weakness or sensory loss; limiting instrumental ADL	Severe weakness or sensory loss; limiting self care ADL	Life-threatening consequences; urgent intervention indicated	Death
Neuralgia	Mild pain	Moderate pain; limiting instrumental ADL	Severe pain; limiting self care ADL	-	-
Nystagmus	-	Moderate symptoms; limiting instrumental ADL	Severe symptoms; limiting self care ADL	-	-
Oculomotor nerve disorder	Asymptomatic; clinical or diagnostic observations only; intervention not indicated	Moderate symptoms; limiting instrumental ADL	Severe symptoms; limiting self care ADL	-	-
Olfactory nerve disorder	-	Moderate symptoms; limiting instrumental ADL	Severe symptoms; limiting self care ADL	-	-
Paresthesia	Mild symptoms	Moderate symptoms; limiting instrumental ADL	Severe symptoms; limiting self care ADL	-	-
Peripheral motor neuropathy	Asymptomatic; clinical or diagnostic observations only; intervention not indicated	Moderate symptoms; limiting instrumental ADL	Severe symptoms; limiting self care ADL; assistive device indicated	Life-threatening consequences; urgent intervention indicated	Death
Peripheral sensory neuropathy	Asymptomatic; loss of deep tendon reflexes or paresthesia	Moderate symptoms; limiting instrumental ADL	Severe symptoms; limiting self care ADL	Life-threatening consequences; urgent intervention indicated	Death
Phantom pain	Mild pain	Moderate pain; limiting instrumental ADL	Severe pain; limiting self care ADL	-	-
Presyncope	-	Present (e.g., near fainting)	-	-	-
Pyramidal tract syndrome	Asymptomatic; clinical or diagnostic observations only; intervention not indicated	Moderate symptoms; limiting instrumental ADL	Severe symptoms; limiting self care ADL	Life-threatening consequences; urgent intervention indicated	Death
Radiculitis	Mild symptoms	Moderate symptoms; limiting instrumental ADL; medical intervention indicated	Severe symptoms; limiting self care ADL	Life-threatening consequences; urgent intervention indicated	Death
Recurrent laryngeal nerve palsy	Asymptomatic; clinical or diagnostic observations only; intervention not indicated	Moderate symptoms	Severe symptoms; medical intervention indicated (e.g., thyroplasty, vocal cord injection)	Life-threatening consequences; urgent intervention indicated	Death
Reversible posterior leukoencephalopathy syndrome	Asymptomatic; clinical or diagnostic observations only; intervention not indicated	Moderate symptoms; abnormal imaging studies; limiting instrumental ADL	Severe symptoms; very abnormal imaging studies; limiting self care ADL	Life-threatening consequences; urgent intervention indicated	Death

Seizure	Brief partial seizure; no loss of consciousness	Brief generalized seizure	Multiple seizures despite medical intervention	Life-threatening; prolonged repetitive seizures	Death
Sinus pain	Mild pain	Moderate pain; limiting instrumental ADL	Severe pain; limiting self care ADL	-	-
Somnolence	Mild but more than usual drowsiness or sleepiness	Moderate sedation; limiting instrumental ADL	Obtundation or stupor	Life-threatening consequences; urgent intervention indicated	Death
Spasticity	Mild or slight increase in muscle tone	Moderate increase in muscle tone and increase in resistance through range of motion	Severe increase in muscle tone and increase in resistance through range of motion	Life-threatening; unable to move active or passive range of motion	Death
Stroke	Asymptomatic or mild neurologic deficit; radiographic findings only	Moderate neurologic deficit	Severe neurologic deficit	Life-threatening consequences; urgent intervention indicated	Death
Syncope	-	-	Fainting; orthostatic collapse	-	-
Transient ischemic attacks	Mild neurologic deficit with or without imaging confirmation	Moderate neurologic deficit with or without imaging confirmation	-	-	-
Tremor	Mild symptoms	Moderate symptoms; limiting instrumental ADL	Severe symptoms; limiting self care ADL	-	-
Trigeminal nerve disorder	Asymptomatic; clinical or diagnostic observations only; intervention not indicated	Moderate symptoms; limiting instrumental ADL	Severe symptoms; limiting self care ADL	-	-
Vagus nerve disorder	Asymptomatic; clinical or diagnostic observations only; intervention not indicated	Moderate symptoms; limiting instrumental ADL	Severe symptoms; limiting self care ADL	Life-threatening consequences; urgent intervention indicated	Death
Vasovagal reaction	-	-	Present	Life-threatening consequences; urgent intervention indicated	Death
Nervous system disorders - Other, specify	Asymptomatic or mild symptoms; clinical or diagnostic observations only; intervention not indicated	Moderate; minimal, local or noninvasive intervention indicated; limiting age-appropriate instrumental ADL	Severe or medically significant but not immediately life-threatening; hospitalization or prolongation of existing hospitalization indicated; disabling; limiting self care ADL	Life-threatening consequences; urgent intervention indicated	Death
Pregnancy, puerperium and perinatal conditions	Grade				
Adverse Event	1	2	3	4	5
Fetal death	-	-	-	-	Fetal loss at any gestational age

Fetal growth retardation	-	<10% percentile of weight for gestational age	<5% percentile of weight for gestational age	<1% percentile of weight for gestational age	-
Premature delivery	Delivery of a liveborn infant at >34 to 37 weeks gestation	Delivery of a liveborn infant at >28 to 34 weeks gestation	Delivery of a liveborn infant at 24 to 28 weeks gestation	Delivery of a liveborn infant at 24 weeks of gestation or less	-
Unintended pregnancy	-	-	Unintended pregnancy	-	-
Pregnancy, puerperium and perinatal conditions - Other, specify	Asymptomatic or mild symptoms; clinical or diagnostic observations only; intervention not indicated	Moderate, local or noninvasive intervention indicated; limiting instrumental ADL	Severe or medically significant but not immediately life-threatening; hospitalization or prolongation of existing hospitalization indicated; disabling; limiting self care ADL	Life-threatening consequences; urgent intervention indicated	Death
Psychiatric disorders	Grade				
Adverse Event	1	2	3	4	5
Agitation	Mild mood alteration	Moderate mood alteration	Severe agitation; hospitalization not indicated	Life-threatening consequences; urgent intervention indicated	Death
Anorgasmia	Inability to achieve orgasm not adversely affecting relationship	Inability to achieve orgasm adversely affecting relationship	-	-	-
Anxiety	Mild symptoms; intervention not indicated	Moderate symptoms; limiting instrumental ADL	Severe symptoms; limiting self care ADL; hospitalization not indicated	Life-threatening; hospitalization indicated	Death
Confusion	Mild disorientation	Moderate disorientation; limiting instrumental ADL	Severe disorientation; limiting self care ADL	Life-threatening consequences; urgent intervention indicated	Death
Delayed orgasm	Delay in achieving orgasm not adversely affecting relationship	Delay in achieving orgasm adversely affecting relationship	-	-	-
Delirium	Mild acute confusional state	Moderate and acute confusional state; limiting instrumental ADL	Severe and acute confusional state; limiting self care ADL; hospitalization indicated	Life-threatening consequences, threats of harm to self or others; hospitalization indicated	Death
Delusions	-	Moderate delusional symptoms	Severe delusional symptoms; hospitalization not indicated	Life-threatening consequences, threats of harm to self or others; hospitalization indicated	Death
Depression	Mild depressive symptoms	Moderate depressive symptoms; limiting instrumental ADL	Severe depressive symptoms; limiting self care ADL; hospitalization not indicated	Life-threatening consequences, threats of harm to self or others; hospitalization indicated	Death
Euphoria	Mild mood elevation	Moderate mood elevation	Severe mood elevation (e.g., hypomania)	-	-
Hallucinations	Mild hallucinations (e.g., perceptual distortions)	Moderate hallucinations	Severe hallucinations; hospitalization not indicated	Life-threatening consequences, threats of harm to self or others; hospitalization indicated	Death

Insomnia	Mild difficulty falling asleep, staying asleep or waking up early	Moderate difficulty falling asleep, staying asleep or waking up early	Severe difficulty in falling asleep, staying asleep or waking up early	-	-
Libido decreased	Decrease in sexual interest not adversely affecting relationship	Decrease in sexual interest adversely affecting relationship	-	-	-
Libido increased	Mild increase in sexual interest not adversely affecting relationship	Moderate increase in sexual interest adversely affecting relationship	Severe increase in sexual interest leading to dangerous behavior	-	-
Mania	Mild manic symptoms (e.g., elevated mood, rapid thoughts, rapid speech, decreased need for sleep)	Moderate manic symptoms (e.g., relationship and work difficulties; poor hygiene)	Severe manic symptoms (e.g., hypomania; major sexual or financial indiscretions); hospitalization not indicated	Life-threatening consequences, threats of harm to self or others; hospitalization indicated	Death
Personality change	Mild personality change	Moderate personality change	Severe personality change; hospitalization not indicated	Life-threatening consequences, threats of harm to self or others; hospitalization indicated	Death
Psychosis	Mild psychotic symptoms	Moderate psychotic symptoms (e.g., disorganized speech; impaired reality testing)	Severe psychotic symptoms (e.g., paranoid; extreme disorganization); hospitalization not indicated	Life-threatening consequences, threats of harm to self or others; hospitalization indicated	Death
Restlessness	Mild symptoms; intervention not indicated	Moderate symptoms; limiting instrumental ADL	Severe symptoms; limiting self care ADL	-	-
Suicidal ideation	Increased thoughts of death but no wish to kill oneself	Suicidal ideation with no specific plan or intent	Specific plan to commit suicide without serious intent to die which may not require hospitalization	Specific plan to commit suicide with serious intent to die which requires hospitalization	-
Suicide attempt	-	-	Suicide attempt or gesture without intent to die which may not require hospitalization	Suicide attempt with intent to die which requires hospitalization	Death
Psychiatric disorders - Other, specify	Asymptomatic or mild symptoms; clinical or diagnostic observations only; intervention not indicated	Moderate; minimal, local or noninvasive intervention indicated; limiting age-appropriate instrumental ADL	Severe or medically significant but not immediately life-threatening; disabling; limiting self care ADL	Life-threatening consequences; hospitalization or urgent intervention indicated	Death
Renal and urinary disorders	Grade				
Adverse Event	1	2	3	4	5
Acute kidney injury	Creatinine level increase of >0.3 mg/dL; creatinine 1.5 - 2.0 x above baseline	Creatinine 2 - 3 x above baseline	Creatinine >3 x baseline or >4.0 mg/dL; hospitalization indicated	Life-threatening consequences; dialysis indicated	Death
Bladder perforation	-	Extraperitoneal perforation, indwelling catheter indicated	Intraperitoneal perforation; elective radiologic, endoscopic or operative intervention indicated	Life-threatening consequences; organ failure; urgent operative intervention indicated	Death
Bladder spasm	Intervention not indicated	Antispasmodics indicated	Hospitalization indicated	-	-

Chronic kidney disease	eGFR (estimated Glomerular Filtration Rate) or CrCl (creatinine clearance) <LLN - 60 ml/min/1.73 m2 or proteinuria 2+ present; urine protein/creatinine >0.5	eGFR or CrCl 59 - 30 ml/min/1.73 m2	eGFR or CrCl 29 - 15 ml/min/1.73 m2	eGFR or CrCl <15 ml/min/1.73 m2; dialysis or renal transplant indicated	Death
Cystitis noninfective	Microscopic hematuria; minimal increase in frequency, urgency, dysuria, or nocturia; new onset of incontinence	Moderate hematuria; moderate increase in frequency, urgency, dysuria, nocturia or incontinence; urinary catheter placement or bladder irrigation indicated; limiting instrumental ADL	Gross hematuria; transfusion, IV medications or hospitalization indicated; elective endoscopic, radiologic or operative intervention indicated	Life-threatening consequences; urgent radiologic or operative intervention indicated	Death
Hematuria	Asymptomatic; clinical or diagnostic observations only; intervention not indicated	Symptomatic; urinary catheter or bladder irrigation indicated; limiting instrumental ADL	Gross hematuria; transfusion, IV medications or hospitalization indicated; elective endoscopic, radiologic or operative intervention indicated; limiting self care ADL	Life-threatening consequences; urgent radiologic or operative intervention indicated	Death
Hemoglobinuria	Asymptomatic; clinical or diagnostic observations only; intervention not indicated	-	-	-	-
Proteinuria	1+ proteinuria; urinary protein <1.0 g/24 hrs	Adults: 2+ proteinuria; urinary protein 1.0 - 3.4 g/24 hrs; Pediatric: urine P/C (Protein/Creatinine) ratio 0.5 - 1.9	Adults: urinary protein >=3.5 g/24 hrs;<br><br>Pediatric: urine P/C >1.9	-	-
Renal calculi	Asymptomatic or mild symptoms; occasional use of nonprescription analgesics indicated	Symptomatic; oral antiemetics indicated; around the clock nonprescription analgesics or any oral narcotic analgesics indicated	Hospitalization indicated; IV intervention (e.g., analgesics, antiemetics); elective endoscopic or radiologic intervention indicated	Life-threatening consequences; urgent radiologic, endoscopic or operative intervention indicated	Death
Renal colic	Mild pain not interfering with activity; nonprescription medication indicated	Moderate pain; limiting instrumental ADL; prescription medication indicated	Hospitalization indicated; limiting self care ADL	-	-
Renal hemorrhage	Mild symptoms; intervention not indicated	Analgesics and hematocrit monitoring indicated	Transfusion, radiation, or hospitalization indicated; elective radiologic, endoscopic or operative intervention indicated	Life-threatening consequences; urgent radiologic or operative intervention indicated	Death
Urinary fistula	-	Noninvasive intervention indicated; urinary or suprapubic catheter placement indicated	Limiting self care ADL; elective radiologic, endoscopic or operative intervention indicated; permanent urinary diversion indicated	Life-threatening consequences; urgent radiologic or operative intervention indicated	Death
Urinary frequency	Present	Limiting instrumental ADL; medical management indicated	-	-	-

Urinary incontinence	Occasional (e.g., with coughing, sneezing, etc.), pads not indicated	Spontaneous; pads indicated; limiting instrumental ADL	Intervention indicated (e.g., clamp, collagen injections); operative intervention indicated; limiting self care ADL	-	-
Urinary retention	Urinary, suprapubic or intermittent catheter placement not indicated; able to void with some residual	Placement of urinary, suprapubic or intermittent catheter placement indicated; medication indicated	Elective operative or radiologic intervention indicated; substantial loss of affected kidney function or mass	Life-threatening consequences; organ failure; urgent operative intervention indicated	Death
Urinary tract obstruction	Asymptomatic; clinical or diagnostic observations only	Symptomatic but no hydronephrosis, sepsis or renal dysfunction; urethral dilation, urinary or suprapubic catheter indicated	Symptomatic and altered organ function (e.g., hydronephrosis, or renal dysfunction); elective radiologic, endoscopic or operative intervention indicated	Life-threatening consequences; urgent intervention indicated	Death
Urinary tract pain	Mild pain	Moderate pain; limiting instrumental ADL	Severe pain; limiting self care ADL	-	-
Urinary urgency	Present	Limiting instrumental ADL; medical management indicated	-	-	-
Urine discoloration	Present	-	-	-	-
Renal and urinary disorders - Other, specify	Asymptomatic or mild symptoms; clinical or diagnostic observations only; intervention not indicated	Moderate, local or noninvasive intervention indicated; limiting instrumental ADL	Severe or medically significant but not immediately life-threatening; hospitalization or prolongation of existing hospitalization indicated; disabling; limiting self care ADL	Life-threatening consequences; urgent intervention indicated	Death
Reproductive system and breast disorders			Grade		
Adverse Event	1	2	3	4	5
Azoospermia	-	-	Absence of sperm in ejaculate	-	-
Breast atrophy	Minimal asymmetry; minimal atrophy	Moderate asymmetry; moderate atrophy	Asymmetry >1/3 of breast volume; severe atrophy	-	-
Breast pain	Mild pain	Moderate pain; limiting instrumental ADL	Severe pain; limiting self care ADL	-	-
Dysmenorrhea	Mild symptoms; intervention not indicated	Moderate symptoms; limiting instrumental ADL	Severe symptoms; limiting self care ADL	-	-

Dyspareunia	Mild discomfort or pain associated with vaginal penetration; discomfort relieved with use of vaginal lubricants or estrogen	Moderate discomfort or pain associated with vaginal penetration; discomfort or pain partially relieved with use of vaginal lubricants or estrogen	Severe discomfort or pain associated with vaginal penetration; discomfort or pain unrelieved by vaginal lubricants or estrogen	-	-
Ejaculation disorder	Diminished ejaculation	Anejaculation or retrograde ejaculation	-	-	-
Erectile dysfunction	Decrease in erectile function (frequency or rigidity of erections) but intervention not indicated (e.g., medication or use of mechanical device, penile pump)	Decrease in erectile function (frequency/rigidity of erections), erectile intervention indicated, (e.g., medication or mechanical devices such as penile pump)	Decrease in erectile function (frequency/rigidity of erections) but erectile intervention not helpful (e.g., medication or mechanical devices such as penile pump); placement of a permanent penile prosthesis indicated (not previously present)	-	-
Fallopian tube obstruction	Diagnostic observations only; intervention not indicated	Mild symptoms; elective intervention indicated	Severe symptoms; elective operative intervention indicated	-	-
Fallopian tube stenosis	Asymptomatic clinical or diagnostic observations only; intervention not indicated	Symptomatic and intervention not indicated	Severe symptoms; elective operative intervention indicated	Life-threatening consequences; urgent operative intervention indicated (e.g., organ resection)	Death
Female genital tract fistula	Asymptomatic clinical or diagnostic observations only; intervention not indicated	Symptomatic and intervention not indicated	Severe symptoms; elective operative intervention indicated	Life-threatening consequences; urgent intervention indicated	Death
Feminization acquired	Mild symptoms; intervention not indicated	Moderate symptoms; medical intervention indicated	-	-	-
Genital edema	Mild swelling or obscuration of anatomic architecture on close inspection	Readily apparent obscuration of anatomic architecture; obliteration of skin folds; readily apparent deviation from normal anatomic contour	Lymphorrhea; gross deviation from normal anatomic contour; limiting self care ADL	-	-
Gynecomastia	Asymptomatic breast enlargement	Symptomatic (e.g., pain or psychosocial impact)	Severe symptoms; elective operative intervention indicated	-	-
Hematosalpinx	Minimal bleeding identified on imaging study or laparoscopy; intervention not indicated	Moderate bleeding; medical intervention indicated	Severe bleeding; transfusion indicated; radiologic or endoscopic intervention indicated	Life-threatening consequences; urgent operative intervention indicated	Death
Irregular menstruation	Intermittent menses with skipped menses for no more than 1 to 3 months	Intermittent menses with skipped menses for more than 4 to 6 months	Persistent amenorrhea for more than 6 months	-	-

Lactation disorder	Mild changes in lactation, not significantly affecting production or expression of breast milk	Changes in lactation, significantly affecting breast production or expression of breast milk	-	-	-
Menorrhagia	Mild; iron supplements indicated	Moderate symptoms; medical intervention indicated (e.g., hormones)	Severe; transfusion indicated; surgical intervention indicated (e.g., hysterectomy)	Life-threatening consequences; urgent intervention indicated	Death
Nipple deformity	Asymptomatic; asymmetry with slight retraction and/or thickening of the nipple areolar complex	Symptomatic; asymmetry of nipple areolar complex with moderate retraction and/or thickening of the nipple areolar complex	-	-	-
Oligospermia	Sperm concentration >48 million/mL or motility >68%	Sperm concentration 13 - 48 million/mL or motility 32 - 68%	Sperm concentration <13 million/mL or motility <32%	-	-
Ovarian hemorrhage	Minimal bleeding identified on imaging study or laproscopy; intervention not indicated	Moderate bleeding; medical intervention indicated	Severe bleeding; transfusion indicated; radiologic or endoscopic intervention indicated	Life-threatening consequences; urgent operative intervention indicated	Death
Ovarian rupture	Asymptomatic clinical or diagnostic observations only; intervention not indicated	Symptomatic and intervention not indicated	Transfusion, radiologic, endoscopic, or elective operative intervention indicated	Life-threatening consequences; urgent intervention indicated	Death
Ovulation pain	Mild pain	Moderate pain; limiting instrumental ADL	Severe pain; limiting self care ADL	-	-
Pelvic floor muscle weakness	Asymptomatic; clinical or diagnostic observations only; intervention not indicated	Symptomatic, not interfering with bladder, bowel, or vaginal function; limiting instrumental ADL	Severe symptoms; limiting self care ADL	Life-threatening consequences; urgent intervention indicated	Death
Pelvic pain	Mild pain	Moderate pain; limiting instrumental ADL	Severe pain; limiting self care ADL	-	-
Penile pain	Mild pain	Moderate pain; limiting instrumental ADL	Severe pain; limiting self care ADL	-	-
Perineal pain	Mild pain	Moderate pain; limiting instrumental ADL	Severe pain; limiting self care ADL	-	-
Premature menopause	-	-	Present	-	-
Prostatic hemorrhage	Minimal bleeding identified on imaging study; intervention not indicated	Moderate bleeding; medical intervention indicated	Severe bleeding; transfusion indicated; radiologic or endoscopic intervention indicated	Life-threatening consequences; urgent operative intervention indicated	Death

Prostatic obstruction	Diagnostic observations only; intervention not indicated	Mild symptoms; elective intervention indicated	Severe symptoms; elective operative intervention indicated	-	-
Prostatic pain	Mild pain	Moderate pain; limiting instrumental ADL	Severe pain; limiting self care ADL	-	-
Scrotal pain	Mild pain	Moderate pain; limiting instrumental ADL	Severe pain; limiting self care ADL	-	-
Spermatic cord hemorrhage	Minimal bleeding identified on imaging study; intervention not indicated	Moderate bleeding; medical intervention indicated	Severe bleeding; transfusion indicated; radiologic or endoscopic intervention indicated	Life-threatening consequences; urgent operative intervention indicated	Death
Spermatic cord obstruction	Diagnostic observations only; intervention not indicated	Mild symptoms; elective intervention indicated	Severe symptoms; elective operative intervention indicated	-	-
Testicular disorder	Asymptomatic; clinical or diagnostic observations only; intervention not indicated	Symptomatic but not interfering with urination or sexual activities; intervention not indicated; limiting instrumental ADL	Severe symptoms; interfering with urination or sexual function; limiting self care ADL; intervention indicated	Life-threatening consequences; urgent intervention indicated	Death
Testicular hemorrhage	Minimal bleeding identified on imaging study; intervention not indicated	Moderate bleeding; medical intervention indicated	Severe bleeding; transfusion indicated; radiologic or endoscopic intervention indicated	Life-threatening consequences; urgent operative intervention indicated	Death
Testicular pain	Mild pain	Moderate pain; limiting instrumental ADL	Severe pain; limiting self care ADL	-	-
Uterine fistula	Asymptomatic clinical or diagnostic observations only; intervention not indicated	Symptomatic and intervention not indicated	Severe symptoms; elective operative intervention indicated	Life-threatening consequences; urgent intervention indicated	Death
Uterine hemorrhage	Minimal bleeding identified on imaging study; intervention not indicated	Moderate bleeding; medical intervention indicated	Severe bleeding; transfusion indicated; radiologic or endoscopic intervention indicated	Life-threatening consequences; urgent operative intervention indicated	Death
Uterine obstruction	Diagnostic observations only; intervention not indicated	Mild symptoms; elective intervention indicated	Severe symptoms; elective operative intervention indicated	-	-
Uterine pain	Mild pain	Moderate pain; limiting instrumental ADL	Severe pain; limiting self care ADL	-	-
Vaginal discharge	Mild vaginal discharge (greater than baseline for patient)	Moderate to heavy vaginal discharge; use of perineal pad or tampon indicated	-	-	-

Vaginal dryness	Mild vaginal dryness not interfering with sexual function	Moderate vaginal dryness interfering with sexual function or causing frequent discomfort	Severe vaginal dryness resulting in dyspareunia or severe discomfort	-	-
Vaginal fistula	Asymptomatic clinical or diagnostic observations only; intervention not indicated	Symptomatic and intervention not indicated	Severe symptoms; elective operative intervention indicated	Life-threatening consequences; urgent intervention indicated	Death
Vaginal hemorrhage	Minimal bleeding identified on clinical exam or imaging study; intervention not indicated	Moderate bleeding; medical intervention indicated	Severe bleeding; transfusion indicated; radiologic or endoscopic intervention indicated	Life-threatening consequences; urgent operative intervention indicated	Death
Vaginal inflammation	Mild discomfort or pain, edema, or redness	Moderate discomfort or pain, edema, or redness; limiting instrumental ADL	Severe discomfort or pain, edema, or redness; limiting self care ADL; small areas of mucosal ulceration	Widespread areas of mucosal ulceration; life-threatening consequences; urgent intervention indicated	Death
Vaginal obstruction	Diagnostic observations only; intervention not indicated	Mild symptoms; elective intervention indicated	Severe symptoms; elective operative intervention indicated	-	-
Vaginal pain	Mild pain	Moderate pain; limiting instrumental ADL	Severe pain; limiting self care ADL	-	-
Vaginal perforation	Asymptomatic clinical or diagnostic observations only; intervention not indicated	Symptomatic and intervention not indicated	Severe symptoms; elective operative intervention indicated	Life-threatening consequences; urgent intervention indicated	Death
Vaginal stricture	Asymptomatic; mild vaginal shortening or narrowing	Vaginal narrowing and/or shortening not interfering with physical examination	Vaginal narrowing and/or shortening interfering with the use of tampons, sexual activity or physical examination	-	Death
Vaginismus	Mild discomfort or pain associated with vaginal spasm/tightening; no impact upon sexual function or physical examination	Moderate discomfort or pain associated with vaginal spasm/tightening; disruption in sexual function and physical examination	Severe discomfort or pain associated with vaginal spasm/tightening; unable to tolerate vaginal penetration or physical examination	-	-
Reproductive system and breast disorders - Other, specify	Asymptomatic or mild symptoms; clinical or diagnostic observations only; intervention not indicated	Moderate; minimal, local or noninvasive intervention indicated; limiting age-appropriate instrumental ADL	Severe or medically significant but not immediately life-threatening; hospitalization or prolongation of existing hospitalization indicated; disabling; limiting self care ADL	Life-threatening consequences; urgent intervention indicated	Death
Respiratory, thoracic and mediastinal disorders			Grade		
Adverse Event	1	2	3	4	5

Adult respiratory distress syndrome	-	-	Present with radiologic findings; intubation not indicated	Life-threatening respiratory or hemodynamic compromise; intubation or urgent intervention indicated	Death
Allergic rhinitis	Mild symptoms; intervention not indicated	Moderate symptoms; medical intervention indicated	-	-	-
Apnea	-	-	Present; medical intervention indicated	Life-threatening respiratory or hemodynamic compromise; intubation or urgent intervention indicated	Death
Aspiration	Asymptomatic; clinical or diagnostic observations only; intervention not indicated	Altered eating habits; coughing or choking episodes after eating or swallowing; medical intervention indicated (e.g., suction or oxygen)	Dyspnea and pneumonia symptoms (e.g., aspiration pneumonia); hospitalization indicated; unable to aliment orally	Life-threatening respiratory or hemodynamic compromise; intubation or urgent intervention indicated	Death
Atelectasis	Asymptomatic; clinical or diagnostic observations only; intervention not indicated	Symptomatic (e.g., dyspnea, cough); medical intervention indicated (e.g., chest physiotherapy, suctioning); bronchoscopic suctioning	Oxygen indicated; hospitalization or elective operative intervention indicated (e.g., stent, laser)	Life-threatening respiratory or hemodynamic compromise; intubation or urgent intervention indicated	Death
Bronchial fistula	Asymptomatic; clinical or diagnostic observations only; intervention not indicated	Symptomatic; tube thoracostomy or medical management indicated; limiting instrumental ADL	Severe symptoms; limiting self care ADL; endoscopic or operative intervention indicated (e.g., stent or primary closure)	Life-threatening consequences; urgent operative intervention with thoracoplasty, chronic open drainage or multiple thoracotomies indicated	Death
Bronchial obstruction	Asymptomatic; clinical or diagnostic observations only; intervention not indicated	Symptomatic (e.g., mild wheezing); endoscopic evaluation indicated; radiographic evidence of atelectasis/lobar collapse; medical management indicated (e.g., steroids, bronchodilators)	Shortness of breath with stridor; endoscopic intervention indicated (e.g., laser, stent placement)	Life-threatening respiratory or hemodynamic compromise; intubation or urgent intervention indicated	Death
Bronchial stricture	Asymptomatic; clinical or diagnostic observations only; intervention not indicated	Symptomatic (e.g., rhonchi or wheezing) but without respiratory distress; medical intervention indicated (e.g., steroids, bronchodilators)	Shortness of breath with stridor; endoscopic intervention indicated (e.g., laser, stent placement)	Life-threatening respiratory or hemodynamic compromise; intubation or urgent intervention indicated	Death

Bronchopleural fistula	Asymptomatic; clinical or diagnostic observations only; intervention not indicated	Symptomatic; tube thoracostomy or medical intervention indicated; limiting instrumental ADL	Severe symptoms; limiting self care ADL; endoscopic or operative intervention indicated (e.g., stent or primary closure)	Life-threatening consequences; urgent operative intervention with thoracoplasty, chronic open drainage or multiple thoracotomies indicated	Death
Bronchopulmonary hemorrhage	Mild symptoms; intervention not indicated	Moderate symptoms; medical intervention indicated	Transfusion, radiologic, endoscopic, or operative intervention indicated (e.g., hemostasis of bleeding site)	Life-threatening respiratory or hemodynamic compromise; intubation or urgent intervention indicated	Death
Bronchospasm	Mild symptoms; intervention not indicated	Symptomatic; medical intervention indicated; limiting instrumental ADL	Limiting self care ADL; oxygen saturation decreased	Life-threatening respiratory or hemodynamic compromise; intubation or urgent intervention indicated	Death
Chylothorax	Asymptomatic; clinical or diagnostic observations only; intervention not indicated	Symptomatic; thoracentesis or tube drainage indicated	Severe symptoms; elective operative intervention indicated	Life-threatening respiratory or hemodynamic compromise; intubation or urgent intervention indicated	Death
Cough	Mild symptoms; nonprescription intervention indicated	Moderate symptoms, medical intervention indicated; limiting instrumental ADL	Severe symptoms; limiting self care ADL	-	-
Dyspnea	Shortness of breath with moderate exertion	Shortness of breath with minimal exertion; limiting instrumental ADL	Shortness of breath at rest; limiting self care ADL	Life-threatening consequences; urgent intervention indicated	Death
Epistaxis	Mild symptoms; intervention not indicated	Moderate symptoms; medical intervention indicated (e.g., nasal packing, cauterization; topical vasoconstrictors)	Transfusion, radiologic, endoscopic, or operative intervention indicated (e.g., hemostasis of bleeding site)	Life-threatening consequences; urgent intervention indicated	Death
Hiccups	Mild symptoms; intervention not indicated	Moderate symptoms; medical intervention indicated; limiting instrumental ADL	Severe symptoms; interfering with sleep; limiting self care ADL	-	-
Hoarseness	Mild or intermittent voice change; fully understandable; self-resolves	Moderate or persistent voice changes; may require occasional repetition but understandable on telephone; medical evaluation indicated	Severe voice changes including predominantly whispered speech	-	-

Hypoxia	-	Decreased oxygen saturation with exercise (e.g., pulse oximeter <88%); intermittent supplemental oxygen	Decreased oxygen saturation at rest (e.g., pulse oximeter <88% or PaO2 <=55 mm Hg)	Life-threatening airway compromise; urgent intervention indicated (e.g., tracheotomy or intubation)	Death
Laryngeal edema	Asymptomatic; clinical or diagnostic observations only; intervention not indicated	Symptomatic; medical intervention indicated (e.g., dexamethasone, epinephrine, antihistamines)	Stridor; respiratory distress; hospitalization indicated	Life-threatening airway compromise; urgent intervention indicated (e.g., tracheotomy or intubation)	Death
Laryngeal fistula	Asymptomatic; clinical or diagnostic observations only; intervention not indicated	Symptomatic; tube thoracostomy or medical management indicated; limiting instrumental ADL	Severe symptoms; limiting self care ADL; endoscopic or operative intervention indicated (e.g., stent or primary closure)	Life-threatening consequences; urgent operative intervention indicated (e.g., thoracoplasty, chronic open drainage or multiple thoracotomies)	Death
Laryngeal hemorrhage	Mild cough or trace hemoptysis; laryngoscopic findings	Moderate symptoms; medical intervention indicated	Transfusion, radiologic, endoscopic, or operative intervention indicated (e.g., hemostasis of bleeding site)	Life-threatening airway compromise; urgent intervention indicated (e.g., tracheotomy or intubation)	Death
Laryngeal inflammation	Mild sore throat; raspy voice	Moderate sore throat; analgesics indicated	Severe throat pain; endoscopic intervention indicated	-	-
Laryngeal mucositis	Endoscopic findings only; mild discomfort with normal intake	Moderate discomfort; altered oral intake	Severe pain; severely altered eating/swallowing; medical intervention indicated	Life-threatening airway compromise; urgent intervention indicated (e.g., tracheotomy or intubation)	Death
Laryngeal obstruction	Asymptomatic; clinical or diagnostic observations only; intervention not indicated	Symptomatic (e.g., noisy airway breathing), but causing no respiratory distress; medical management indicated (e.g., steroids); limiting instrumental ADL	Limiting self care ADL; stridor; endoscopic intervention indicated (e.g., stent, laser)	Life-threatening consequences; urgent intervention indicated	Death
Laryngeal stenosis	Asymptomatic; clinical or diagnostic observations only; intervention not indicated	Symptomatic (e.g., noisy airway breathing), but causing no respiratory distress; medical management indicated (e.g., steroids)	Limiting self care ADL; stridor; endoscopic intervention indicated (e.g., stent, laser)	Life-threatening consequences; urgent intervention indicated	Death
Laryngopharyngeal dysesthesia	Mild symptoms; no anxiety; intervention not indicated	Moderate symptoms; mild anxiety, but no dyspnea; short duration of observation and or anxiolytic indicated; limiting instrumental ADL	Severe symptoms; dyspnea and swallowing difficulty; limiting self care ADL	Life-threatening consequences	Death

Laryngospasm	-	Transient episode; intervention not indicated	Recurrent episodes; noninvasive intervention indicated (e.g., breathing technique, pressure point massage)	Persistent or severe episodes associated with syncope; urgent intervention indicated (e.g., fiberoptic laryngoscopy, intubation, botox injection)	Death
Mediastinal hemorrhage	Radiologic evidence only; minimal symptoms; intervention not indicated	Moderate symptoms; medical intervention indicated	Transfusion, radiologic, endoscopic, or elective operative intervention indicated (e.g., hemostasis of bleeding site)	Life-threatening consequences; urgent intervention indicated	Death
Nasal congestion	Mild symptoms; intervention not indicated	Moderate symptoms; medical intervention indicated	Associated with bloody nasal discharge or epistaxis	-	-
Pharyngeal fistula	Asymptomatic; clinical or diagnostic observations only; intervention not indicated	Symptomatic; tube thoracostomy or medical intervention indicated; limiting instrumental ADL	Severe symptoms; limiting self care ADL; endoscopic or operative intervention indicated (e.g., stent or primary closure)	Life-threatening consequences; urgent intervention indicated	Death
Pharyngeal hemorrhage	Mild symptoms; intervention not indicated	Moderate symptoms; medical intervention indicated	Transfusion, radiologic, endoscopic, or operative intervention indicated (e.g., hemostasis of bleeding site)	Life-threatening respiratory or hemodynamic compromise; intubation or urgent intervention indicated	Death
Pharyngeal mucositis	Endoscopic findings only; minimal symptoms with normal oral intake; mild pain but analgesics not indicated	Moderate pain and analgesics indicated; altered oral intake; limiting instrumental ADL	Severe pain; unable to adequately aliment or hydrate orally; limiting self care ADL	Life-threatening consequences; urgent intervention indicated	Death
Pharyngeal necrosis	-	-	Inability to aliment adequately by GI tract; tube feeding or TPN indicated; radiologic, endoscopic, or operative intervention indicated	Life-threatening consequences; urgent operative intervention indicated	Death
Pharyngeal stenosis	Asymptomatic; clinical or diagnostic observations only; intervention not indicated	Symptomatic (e.g., noisy airway breathing), but causing no respiratory distress; medical management indicated (e.g., steroids); limiting instrumental ADL	Limiting self care ADL; stridor; endoscopic intervention indicated (e.g., stent, laser)	Life-threatening airway compromise; urgent intervention indicated (e.g., tracheotomy or intubation)	Death
Pharyngolaryngeal pain	Mild pain	Moderate pain; limiting instrumental ADL	Severe pain; limiting self care ADL	-	-

Pleural effusion	Asymptomatic; clinical or diagnostic observations only; intervention not indicated	Symptomatic; intervention indicated (e.g., diuretics or limited therapeutic thoracentesis)	Symptomatic with respiratory distress and hypoxia; surgical intervention including chest tube or pleurodesis indicated	Life-threatening respiratory or hemodynamic compromise; intubation or urgent intervention indicated	Death
Pleural hemorrhage	Asymptomatic; mild hemorrhage confirmed by thoracentesis	Symptomatic or associated with pneumothorax; chest tube drainage indicated	>1000 ml of blood evacuated; persistent bleeding (150-200 ml/hr for 2 - 4 hr); persistent transfusion indicated; elective operative intervention indicated	Life-threatening respiratory or hemodynamic compromise; intubation or urgent intervention indicated	Death
Pleuritic pain	Mild pain	Moderate pain; limiting instrumental ADL	Severe pain; limiting self care ADL	-	-
Pneumonitis	Asymptomatic; clinical or diagnostic observations only; intervention not indicated	Symptomatic; medical intervention indicated; limiting instrumental ADL	Severe symptoms; limiting self care ADL; oxygen indicated	Life-threatening respiratory compromise; urgent intervention indicated (e.g., tracheotomy or intubation)	Death
Pneumothorax	Asymptomatic; clinical or diagnostic observations only; intervention not indicated	Symptomatic; intervention indicated (e.g., tube placement without sclerosis)	Sclerosis and/or operative intervention indicated; hospitalization indicated	Life-threatening consequences; urgent intervention indicated	Death
Postnasal drip	Mild symptoms; intervention not indicated	Moderate symptoms; medical intervention indicated	-	-	-
Productive cough	Occasional/minimal production of sputum with cough	Moderate sputum production; limiting instrumental ADL	Persistent or copious production of sputum; limiting self care ADL	-	-
Pulmonary edema	Radiologic findings only; minimal dyspnea on exertion	Moderate dyspnea on exertion; medical intervention indicated; limiting instrumental ADL	Severe dyspnea or dyspnea at rest; oxygen indicated; limiting self care ADL	Life-threatening respiratory compromise; urgent intervention or intubation with ventilatory support indicated	Death
Pulmonary fibrosis	Mild hypoxemia; radiologic pulmonary fibrosis <25% of lung volume	Moderate hypoxemia; evidence of pulmonary hypertension; radiographic pulmonary fibrosis 25 - 50%	Severe hypoxemia; evidence of right-sided heart failure; radiographic pulmonary fibrosis >50 - 75%	Life-threatening consequences (e.g., hemodynamic/pulmonary complications); intubation with ventilatory support indicated; radiographic pulmonary fibrosis >75% with severe honeycombing	Death

Pulmonary fistula	Asymptomatic; clinical or diagnostic observations only; intervention not indicated	Symptomatic; tube thoracostomy or medical management indicated; limiting instrumental ADL	Limiting self care ADL; endoscopic stenting or operative intervention indicated	Life-threatening consequences; urgent operative intervention indicated	Death
Pulmonary hypertension	Minimal dyspnea; findings on physical exam or other evaluation	Moderate dyspnea, cough; requiring evaluation by cardiac catheterization and medical intervention	Severe symptoms, associated with hypoxemia, right heart failure; oxygen indicated	Life-threatening airway consequences; urgent intervention indicated (e.g., tracheotomy or intubation)	Death
Respiratory failure	-	-	-	Life-threatening consequences; urgent intervention, intubation, or ventilatory support indicated	Death
Retinoic acid syndrome	Fluid retention; <3 kg of weight gain; intervention with fluid restriction and/or diuretics indicated	Moderate signs or symptoms; steroids indicated	Severe symptoms; hospitalization indicated	Life-threatening consequences; ventilatory support indicated	Death
Sinus disorder	Asymptomatic mucosal crusting; blood-tinged secretions	Symptomatic stenosis or edema/narrowing interfering with airflow; limiting instrumental ADL	Stenosis with significant nasal obstruction; limiting self care ADL	Necrosis of soft tissue or bone; urgent operative intervention indicated	Death
Sleep apnea	Snoring and nocturnal sleep arousal without apneic periods	Moderate apnea and oxygen desaturation; excessive daytime sleepiness; medical evaluation indicated; limiting instrumental ADL	Oxygen desaturation; associated with hypertension; medical intervention indicated; limiting self care ADL	Cardiovascular or neuropsychiatric symptoms; urgent operative intervention indicated	Death
Sneezing	Mild symptoms; intervention not indicated	Moderate symptoms; medical intervention indicated	-	-	-
Sore throat	Mild pain	Moderate pain; limiting instrumental ADL	Severe pain; limiting self care ADL; limiting ability to swallow	-	-
Stridor	-	-	Respiratory distress limiting self care ADL; medical intervention indicated	Life-threatening airway compromise; urgent intervention indicated (e.g., tracheotomy or intubation)	Death
Tracheal fistula	Asymptomatic; clinical or diagnostic observations only; intervention not indicated	Symptomatic; tube thoracostomy or medical intervention indicated; limiting instrumental ADL	Severe symptoms; limiting self care ADL; endoscopic or operative intervention indicated (e.g., stent or primary closure)	Life-threatening consequences; urgent operative intervention indicated (e.g., thoracoplasty, chronic open drainage or multiple thoracotomies)	Death

Tracheal mucositis	Endoscopic findings only; minimal hemoptysis, pain, or respiratory symptoms	Moderate symptoms; medical intervention indicated; limiting instrumental ADL	Severe pain; hemorrhage or respiratory symptoms; limiting self care ADL	Life-threatening consequences; urgent intervention indicated	Death
Tracheal stenosis	Asymptomatic; clinical or diagnostic observations only; intervention not indicated	Symptomatic (e.g., noisy airway breathing), but causing no respiratory distress; medical management indicated (e.g., steroids)	Stridor or respiratory distress limiting self care ADL; endoscopic intervention indicated (e.g., stent, laser)	Life-threatening airway compromise; urgent intervention indicated (e.g., tracheotomy or intubation)	Death
Voice alteration	Mild or intermittent change from normal voice	Moderate or persistent change from normal voice; still understandable	Severe voice changes including predominantly whispered speech; may require frequent repetition or face-to-face contact for understandability; may require assistive technology	-	-
Wheezing	Detectable airway noise with minimal symptoms	Moderate symptoms; medical intervention indicated; limiting instrumental ADL	Severe respiratory symptoms limiting self care ADL; oxygen therapy or hospitalization indicated	Life-threatening consequences; urgent intervention indicated	Death
Respiratory, thoracic and mediastinal disorders - Other, specify	Asymptomatic or mild symptoms; clinical or diagnostic observations only; intervention not indicated	Moderate; minimal, local or noninvasive intervention indicated; limiting age-appropriate instrumental ADL	Severe or medically significant but not immediately life-threatening; hospitalization or prolongation of existing hospitalization indicated; disabling; limiting self care ADL	Life-threatening consequences; urgent intervention indicated	Death

Skin and subcutaneous tissue disorders	Grade				
Adverse Event	1	2	3	4	5
Alopecia	Hair loss of <50% of normal for that individual that is not obvious from a distance but only on close inspection; a different hair style may be required to cover the hair loss but it does not require a wig or hair piece to camouflage	Hair loss of >=50% normal for that individual that is readily apparent to others; a wig or hair piece is necessary if the patient desires to completely camouflage the hair loss; associated with psychosocial impact	-	-	-
Body odor	Mild odor; physician intervention not indicated; self care interventions	Pronounced odor; psychosocial impact; patient seeks medical intervention	-	-	-
Bullous dermatitis	Asymptomatic; blisters covering <10% BSA	Blisters covering 10 - 30% BSA; painful blisters; limiting instrumental ADL	Blisters covering >30% BSA; limiting self care ADL	Blisters covering >30% BSA; associated with fluid or electrolyte abnormalities; ICU care or burn unit indicated	Death
Dry skin	Covering <10% BSA and no associated erythema or pruritus	Covering 10 - 30% BSA and associated with erythema or pruritus; limiting instrumental ADL	Covering >30% BSA and associated with pruritus; limiting self care ADL	-	-
Erythema multiforme	Target lesions covering <10% BSA and not associated with skin tenderness	Target lesions covering 10 - 30% BSA and associated with skin tenderness	Target lesions covering >30% BSA and associated with oral or genital erosions	Target lesions covering >30% BSA; associated with fluid or electrolyte abnormalities; ICU care or burn unit indicated	Death
Erythroderma	-	Erythema covering >90% BSA without associated symptoms; limiting instrumental ADL	Erythema covering >90% BSA with associated symptoms (e.g., pruritus or tenderness); limiting self care ADL	Erythema covering >90% BSA with associated fluid or electrolyte abnormalities; ICU care or burn unit indicated	Death
Fat atrophy	Covering <10% BSA and asymptomatic	Covering 10 - 30% BSA and associated with erythema or tenderness; limiting instrumental ADL	Covering >30% BSA; associated with erythema or tenderness; limiting self-care ADL	-	-
Hirsutism	In women, increase in length, thickness or density of hair in a male distribution that the patient is able to camouflage by periodic shaving, bleaching, or removal of hair	In women, increase in length, thickness or density of hair in a male distribution that requires daily shaving or consistent destructive means of hair removal to camouflage; associated with psychosocial impact	-	-	-

Hyperhidrosis	Limited to one site (palms, soles, or axillae); self care interventions	Involving >1 site; patient seeks medical intervention; associated with psychosocial impact	Generalized involving sites other than palms, soles, or axillae; associated with electrolyte/hemodynamic imbalance	-	-
Hypertrichosis	Increase in length, thickness or density of hair that the patient is either able to camouflage by periodic shaving or removal of hairs or is not concerned enough about the overgrowth to use any form of hair removal	Increase in length, thickness or density of hair at least on the usual exposed areas of the body [face (not limited to beard/moustache area) plus/minus arms] that requires frequent shaving or use of destructive means of hair removal to camouflage; associated with psychosocial impact	-	-	-
Hypohidrosis	-	Symptomatic; limiting instrumental ADL	Increase in body temperature; limiting self care ADL	Heat stroke	Death
Lipohypertrophy	Asymptomatic and covering <10% BSA	Covering 10 - 30% BSA and associated tenderness; limiting instrumental ADL	Covering >30% BSA and associated tenderness and narcotics or NSAIDs indicated; lipohypertrophy; limiting self care ADL	-	-
Nail discoloration	Asymptomatic; clinical or diagnostic observations only; intervention not indicated	-	-	-	-
Nail loss	Asymptomatic separation of the nail bed from the nail plate or nail loss	Symptomatic separation of the nail bed from the nail plate or nail loss; limiting instrumental ADL	-	-	-
Nail ridging	Asymptomatic; clinical or diagnostic observations only; intervention not indicated	-	-	-	-
Pain of skin	Mild pain	Moderate pain; limiting instrumental ADL	Severe pain; limiting self care ADL	-	-
Palmar-plantar erythrodysesthesia syndrome	Minimal skin changes or dermatitis (e.g., erythema, edema, or hyperkeratosis) without pain	Skin changes (e.g., peeling, blisters, bleeding, edema, or hyperkeratosis) with pain; limiting instrumental ADL	Severe skin changes (e.g., peeling, blisters, bleeding, edema, or hyperkeratosis) with pain; limiting self care ADL	-	-

Periorbital edema	Soft or non-pitting	Indurated or pitting edema; topical intervention indicated	Edema associated with visual disturbance; increased intraocular pressure, glaucoma or retinal hemorrhage; optic neuritis; diuretics indicated; operative intervention indicated	-	-
Photosensitivity	Painless erythema and erythema covering <10% BSA	Tender erythema covering 10 - 30% BSA	Erythema covering >30% BSA and erythema with blistering; photosensitivity; oral corticosteroid therapy indicated; pain control indicated (e.g., narcotics or NSAIDs)	Life-threatening consequences; urgent intervention indicated	Death
Pruritus	Mild or localized; topical intervention indicated	Intense or widespread; intermittent; skin changes from scratching (e.g., edema, papulation, excoriations, lichenification, oozing/crusts); oral intervention indicated; limiting instrumental ADL	Intense or widespread; constant; limiting self care ADL or sleep; oral corticosteroid or immunosuppressive therapy indicated	-	-
Purpura	Combined area of lesions covering <10% BSA	Combined area of lesions covering 10 - 30% BSA; bleeding with trauma	Combined area of lesions covering >30% BSA; spontaneous bleeding	-	-
Rash acneiform	Papules and/or pustules covering <10% BSA, which may or may not be associated with symptoms of pruritus or tenderness	Papules and/or pustules covering 10 - 30% BSA, which may or may not be associated with symptoms of pruritus or tenderness; associated with psychosocial impact; limiting instrumental ADL	Papules and/or pustules covering >30% BSA, which may or may not be associated with symptoms of pruritus or tenderness; limiting self care ADL; associated with local superinfection with oral antibiotics indicated	Papules and/or pustules covering any % BSA, which may or may not be associated with symptoms of pruritus or tenderness and are associated with extensive superinfection with IV antibiotics indicated; life-threatening consequences	Death
Rash maculo-papular	Macules/papules covering <10% BSA with or without symptoms (e.g., pruritus, burning, tightness)	Macules/papules covering 10 - 30% BSA with or without symptoms (e.g., pruritus, burning, tightness); limiting instrumental ADL	Macules/papules covering >30% BSA with or without associated symptoms; limiting self care ADL	-	-
Scalp pain	Mild pain	Moderate pain; limiting instrumental ADL	Severe pain; limiting self care ADL	-	-
Skin atrophy	Covering <10% BSA; associated with telangiectasias or changes in skin color	Covering 10 - 30% BSA; associated with striae or adnexal structure loss	Covering >30% BSA; associated with ulceration	-	-
Skin hyperpigmentation	Hyperpigmentation covering <10% BSA; no psychosocial impact	Hyperpigmentation covering >10% BSA; associated psychosocial impact	-	-	-

Skin hypopigmentation	Hypopigmentation or depigmentation covering <10% BSA; no psychosocial impact	Hypopigmentation or depigmentation covering >10% BSA; associated psychosocial impact	-	-	-
Skin induration	Mild induration, able to move skin parallel to plane (sliding) and perpendicular to skin (pinching up)	Moderate induration, able to slide skin, unable to pinch skin; limiting instrumental ADL	Severe induration, unable to slide or pinch skin; limiting joint movement or orifice (e.g., mouth, anus); limiting self care ADL	Generalized; associated with signs or symptoms of impaired breathing or feeding	Death
Skin ulceration	Combined area of ulcers <1 cm; nonblanchable erythema of intact skin with associated warmth or edema	Combined area of ulcers 1 - 2 cm; partial thickness skin loss involving skin or subcutaneous fat	Combined area of ulcers >2 cm; full-thickness skin loss involving damage to or necrosis of subcutaneous tissue that may extend down to fascia	Any size ulcer with extensive destruction, tissue necrosis, or damage to muscle, bone, or supporting structures with or without full thickness skin loss	Death
Stevens-Johnson syndrome	-	-	Skin sloughing covering <10% BSA with associated signs (e.g., erythema, purpura, epidermal detachment and mucous membrane detachment)	Skin sloughing covering 10 - 30% BSA with associated signs (e.g., erythema, purpura, epidermal detachment and mucous membrane detachment)	Death
Telangiectasia	Telangiectasias covering <10% BSA	Telangiectasias covering >10% BSA; associated with psychosocial impact	-	-	-
Toxic epidermal necrolysis	-	-	-	Skin sloughing covering >=30% BSA with associated symptoms (e.g., erythema, purpura, or epidermal detachment)	Death
Urticaria	Urticarial lesions covering <10% BSA; topical intervention indicated	Urticarial lesions covering 10 - 30% BSA; oral intervention indicated	Urticarial lesions covering >30% BSA; IV intervention indicated	-	-
Skin and subcutaneous tissue disorders - Other, specify	Asymptomatic or mild symptoms; clinical or diagnostic observations only; intervention not indicated	Moderate; minimal, local or noninvasive intervention indicated; limiting age-appropriate instrumental ADL	Severe or medically significant but not immediately life-threatening; hospitalization or prolongation of existing hospitalization indicated; disabling; limiting self care ADL	Life-threatening consequences; urgent intervention indicated	Death

Social circumstances	Grade				
Adverse Event	1	2	3	4	5
Menopause	Menopause occurring at age 46 - 53 years of age	Menopause occurring at age 40 - 45 years of age	Menopause occurring before age 40 years of age	-	-
Social circumstances - Other, specify	Asymptomatic or mild symptoms; clinical or diagnostic observations only; intervention not indicated	Moderate; minimal, local or noninvasive intervention indicated; limiting age-appropriate instrumental ADL	Severe or medically significant but not immediately life-threatening; hospitalization or prolongation of existing hospitalization indicated; disabling; limiting self care ADL	Life-threatening consequences; urgent intervention indicated	Death
Surgical and medical procedures	Grade				
Adverse Event	1	2	3	4	5
Surgical and medical procedures - Other, specify	Asymptomatic or mild symptoms; clinical or diagnostic observations only; intervention not indicated	Moderate; minimal, local or noninvasive intervention indicated; limiting age-appropriate instrumental ADL	Severe or medically significant but not immediately life-threatening; hospitalization or prolongation of existing hospitalization indicated; disabling; limiting self care ADL	Life-threatening consequences; urgent intervention indicated	Death
Vascular disorders	Grade				
Adverse Event	1	2	3	4	5
Capillary leak syndrome	-	Symptomatic; medical intervention indicated	Severe symptoms; intervention indicated	Life-threatening consequences; urgent intervention indicated	Death
Flushing	Asymptomatic; clinical or diagnostic observations only; intervention not indicated	Moderate symptoms; medical intervention indicated; limiting instrumental ADL	Symptomatic, associated with hypotension and/or tachycardia; limiting self care ADL	-	-
Hematoma	Mild symptoms; intervention not indicated	Minimally invasive evacuation or aspiration indicated	Transfusion, radiologic, endoscopic, or elective operative intervention indicated	Life-threatening consequences; urgent intervention indicated	Death
Hot flashes	Mild symptoms; intervention not indicated	Moderate symptoms; limiting instrumental ADL	Severe symptoms; limiting self care ADL	-	-
Hypertension	Prehypertension (systolic BP 120 - 139 mm Hg or diastolic BP 80 - 89 mm Hg)	Stage 1 hypertension (systolic BP 140 - 159 mm Hg or diastolic BP 90 - 99 mm Hg); medical intervention indicated; recurrent or persistent (>=24 hrs); symptomatic increase by >20 mm Hg (diastolic) or to >140/90 mm Hg if previously WNL; monotherapy indicated<br><br>Pediatric: recurrent or persistent (>=24 hrs) BP >ULN; monotherapy indicated	Stage 2 hypertension (systolic BP >=160 mm Hg or diastolic BP >=100 mm Hg); medical intervention indicated; more than one drug or more intensive therapy than previously used indicated<br><br>Pediatric: Same as adult	Life-threatening consequences (e.g., malignant hypertension, transient or permanent neurologic deficit, hypertensive crisis); urgent intervention indicated<br><br>Pediatric: Same as adult	Death

Hypotension	Asymptomatic, intervention not indicated	Non-urgent medical intervention indicated	Medical intervention or hospitalization indicated	Life-threatening and urgent intervention indicated	Death
Lymph leakage	-	Symptomatic; medical intervention indicated	Severe symptoms; radiologic, endoscopic or elective operative intervention indicated	Life-threatening consequences; urgent intervention indicated	Death
Lymphedema	Trace thickening or faint discoloration	Marked discoloration; leathery skin texture; papillary formation; limiting instrumental ADL	Severe symptoms; limiting self care ADL	-	-
Lymphocele	Asymptomatic; clinical or diagnostic observations only; intervention not indicated	Symptomatic; medical intervention indicated	Severe symptoms; radiologic, endoscopic or elective operative intervention indicated	-	-
Peripheral ischemia	-	Brief (<24 hrs) episode of ischemia managed non-surgically and without permanent deficit	Recurring or prolonged (>=24 hrs) and/or invasive intervention indicated	Life-threatening consequences; evidence of end organ damage; urgent operative intervention indicated	Death
Phlebitis	-	Present	-	-	-
Superficial thrombophlebitis	-	Present	-	-	-
Superior vena cava syndrome	Asymptomatic; incidental finding of SVC thrombosis	Symptomatic; medical intervention indicated (e.g., anticoagulation, radiation or chemotherapy)	Severe symptoms; multi-modality intervention indicated (e.g., anticoagulation, chemotherapy, radiation, stenting)	Life-threatening consequences; urgent multi-modality intervention indicated (e.g., lysis, thrombectomy, surgery)	Death
Thromboembolic event	Venous thrombosis (e.g., superficial thrombosis)	Venous thrombosis (e.g., uncomplicated deep vein thrombosis), medical intervention indicated	Thrombosis (e.g., uncomplicated pulmonary embolism [venous], non-embolic cardiac mural [arterial] thrombus), medical intervention indicated	Life-threatening (e.g., pulmonary embolism, cerebrovascular event, arterial insufficiency); hemodynamic or neurologic instability; urgent intervention indicated	Death
Vasculitis	Asymptomatic, intervention not indicated	Moderate symptoms, medical intervention indicated	Severe symptoms, medical intervention indicated (e.g., steroids)	Life-threatening; evidence of peripheral or visceral ischemia; urgent intervention indicated	Death
Visceral arterial ischemia	-	Brief (<24 hrs) episode of ischemia managed medically and without permanent deficit	Prolonged (>=24 hrs) or recurring symptoms and/or invasive intervention indicated	Life-threatening consequences; evidence of end organ damage; urgent operative intervention indicated	Death
Vascular disorders - Other, specify	Asymptomatic or mild symptoms; clinical or diagnostic observations only; intervention not indicated	Moderate; minimal, local or noninvasive intervention indicated; limiting age-appropriate instrumental ADL	Severe or medically significant but not immediately life-threatening; hospitalization or prolongation of existing hospitalization indicated; disabling; limiting self care ADL	Life-threatening consequences; urgent intervention indicated	Death

APPENDIX G REFERENCES

1 (UNAIDS)., J. U. N. P. o. H. A. The Gap Report., (UNAIDS, 2014).
2 UNAIDS. AIDS by the numbers 2015. (2015).
3 CDC. CDC. Vital Signs: HIV Diagnosis, Care, and Treatment Among Persons Living with HIV- United States 2011. *MMWR* **4**, 1-6 (2014).
4 Jardine, J. *et al.* Rational HIV immunogen design to target specific germline B cell receptors. *Science* **340**, 711-716, doi:10.1126/science.1234150 (2013).
5 Sok, D. *et al.* Promiscuous glycan site recognition by antibodies to the high-mannose patch of gp120 broadens neutralization of HIV. *Sci Transl Med* **6**, 236ra263, doi:10.1126/scitranslmed.3008104 (2014).
6 Caskey, M. *et al.* Viraemia suppressed in HIV-1-infected humans by broadly neutralizing antibody 3BNC117. *Nature* **522**, 487-491, doi:10.1038/nature14411 (2015).
7 Barouch, D. H. *et al.* Therapeutic efficacy of potent neutralizing HIV-1-specific monoclonal antibodies in SHIV-infected rhesus monkeys. *Nature* **503**, 224-228, doi:10.1038/nature12744 (2013).
8 Hessell, A. J. *et al.* Broadly neutralizing human anti-HIV antibody 2G12 is effective in protection against mucosal SHIV challenge even at low serum neutralizing titers. *PLoS Pathog* **5**, e1000433, doi:10.1371/journal.ppat.1000433 (2009).
9 Hessell, A. J. *et al.* Effective, low-titer antibody protection against low-dose repeated mucosal SHIV challenge in macaques. *Nat Med* **15**, 951-954, doi:10.1038/nm.1974 (2009).
10 Moldt, B. *et al.* Highly potent HIV-specific antibody neutralization in vitro translates into effective protection against mucosal SHIV challenge in vivo. *Proceedings of the National Academy of Sciences of the United States of America* **109**, 18921-18925, doi:10.1073/pnas.1214785109 (2012).
11 Walker, L. M. *et al.* Broad neutralization coverage of HIV by multiple highly potent antibodies. *Nature* **477**, 466-470, doi:10.1038/nature10373 (2011).
12 Haynes, B. F. & McElrath, M. J. Progress in HIV-1 vaccine development. *Curr Opin HIV AIDS* **8**, 326-332, doi:10.1097/COH.0b013e328361d178 (2013).
13 Burton, D. R. & Mascola, J. R. Antibody responses to envelope glycoproteins in HIV-1 infection. *Nat Immunol* **16**, 571-576, doi:10.1038/ni.3158 (2015).
14 Sok, D. *et al.* Recombinant HIV envelope trimer selects for quaternary-dependent antibodies targeting the trimer apex. *Proc Natl Acad Sci U S A* **111**, 17624-17629, doi:10.1073/pnas.1415789111 (2014).
15 Scheid, J. F. *et al.* Sequence and structural convergence of broad and potent HIV antibodies that mimic CD4 binding. *Science* **333**, 1633-1637, doi:10.1126/science.1207227 (2011).
16 Shingai, M. *et al.* Passive transfer of modest titers of potent and broadly neutralizing anti-HIV monoclonal antibodies block SHIV infection in macaques. *J Exp Med* **211**, 2061-2074, doi:10.1084/jem.20132494 (2014).
17 Lynch, R. M. *et al.* Virologic effects of broadly neutralizing antibody VRC01 administration during chronic HIV-1 infection. *Sci Transl Med* **7**, 319ra206, doi:10.1126/scitranslmed.aad5752 (2015).
18 Andrade, A. *et al.* Three distinct phases of HIV-1 RNA decay in treatment-naive patients receiving raltegravir-based antiretroviral therapy: ACTG A5248. *J Infect Dis* **208**, 884-891, doi:10.1093/infdis/jit272 (2013).

International AIDS Vaccine Initiative

DOCUMENT NUMBER:
DOCUMENT TITLE:
DOCUMENT NOTES:

Document Information

Revision:	Vault:
Status:	Document Type:

Date Information

Effective Date:	Expiration Date:
Release Date:	Next Review Date:

Control Information

Author:	Owner:
Previous Number:	Change Number:

Signature Manifest

Document Number: TMF-02-0166 **Revision:** 5

Title: Protocol PGT121

All dates and times are in Eastern Time Zone.

T001 Protocol

Change Request Approval

Name/Signature	Title	Date	Meaning/Reason
Harriet Park (HPARK)			
Jeniffer Kigera (JKIGERA)			
Lisa Sunner (LSUNNER)			
Carl Verlinde (CVERLINDE)			
Dani Vooijs (DVOOIJS)	Sr. Manager, Clinical Programs	29 Jun 2017, 03:30:19 AM	Approved

CMO Approval

Name/Signature	Title	Date	Meaning/Reason
Frances Priddy (FPRIDDY)	Chief Medical Officer	16 Jul 2017, 01:22:16 PM	Approved

QA Final Release

Name/Signature	Title	Date	Meaning/Reason
Dani Vooijs (DVOOIJS)			
Harriet Park (HPARK)			
Jeniffer Kigera (JKIGERA)			
Lisa Sunner (LSUNNER)			
Carl Verlinde (CVERLINDE)	Director, Data Management	17 Jul 2017, 09:21:02 AM	Approved

Notify

Name/Signature	Title	Date	Meaning/Reason
Lisa Sunner (LSUNNER)		17 Jul 2017, 09:21:02 AM	Email Sent

CONFIDENTIAL

**Protocol Title:**	A Phase 1 Randomized Placebo-controlled Clinical Trial of the Safety, Pharmacokinetics and Antiviral Activity of PGT121 Monoclonal Antibody (mAb) in HIV-uninfected and HIV-infected Adults
**Protocol Number:**	IAVI T001
**Regulatory Investigational Product Number**	IND 126807
**ClinicalTrials.gov Registry Number**	NCT02960581
**Phase:**	Phase 1
**Sponsor:**	International AIDS Vaccine Initiative (IAVI)<br>125 Broad Street, 9th Floor<br>New York, New York 10004<br>USA
**Sponsor Status**	Not for-Profit Organization
**Date of Protocol Version:**	16 October 2018<br>07.0<br><br>14 July 2017<br>06.0<br><br>04 April 2017<br>05.0<br><br>23 November 2016<br>04.0<br><br>17 October 2016<br>03.0<br><br>09 September 2016<br>02.0<br><br>05 August 2016<br>01.0

THE CONFIDENTIAL INFORMATION IN THIS DOCUMENT IS PROVIDED TO YOU AS AN INVESTIGATOR, POTENTIAL INVESTIGATOR, OR CONSULTANT, FOR REVIEW BY YOU, YOUR STAFF, AND APPLICABLE INSTITUTIONAL REVIEW BOARDS (IRBS) AND/OR INDEPENDENT ETHICS COMMITTEES (IECS). IT IS UNDERSTOOD THAT THE INFORMATION WILL NOT BE DISCLOSED TO OTHERS, EXCEPT TO THE EXTENT NECESSARY TO OBTAIN ETHICAL AND REGULATORY APPROVAL FROM THE RESPECTIVE COMMITTEE'S AGENCIES AND INFORMED CONSENT FROM THOSE PERSONS TO WHOM THE INVESTIGATIONAL PRODUCT MAY BE ADMINISTERED.

TMF-03562 | 7.0 | Status:Approved | 18 Oct 2018
This copy of the document was retrieved from the system by Lisa Sunner on 23 Oct 2018

SYNOPSIS

**TITLE**	A Phase 1 Randomized Placebo-controlled Clinical Trial of the Safety, Pharmacokinetics and Antiviral Activity of PGT121 Monoclonal Antibody (mAb) in HIV-uninfected and HIV-infected Adults
**PROTOCOL NUMBER**	IAVI T001
**PHASE**	Phase 1
**SPONSOR**	International AIDS Vaccine Initiative (IAVI)<br>125 Broad Street, 9th Floor<br>New York, New York 10004,<br>USA
**SPONSOR STATUS**	Not for Profit Organization
**STUDY PRODUCTS**	PGT121 monoclonal antibody (mAb)
**OBJECTIVES**	**Primary Objectives**<br>• To evaluate the safety and tolerability of a single intravenous (IV) infusion of PGT121 mAb at 3, 10, and 30 mg/kg in HIV-uninfected adults and HIV-infected adults<br>• To evaluate the safety and tolerability of a single subcutaneous (SC) injection of PGT121 mAb at 3 mg/kg in HIV-uninfected adults<br>• To evaluate the pharmacokinetic (PK) profile of IV infusion and SC injection of PGT121 mAb in HIV-uninfected and HIV-infected adults<br>• To evaluate the antiviral activity of IV infusion of PGT121 mAb in HIV-infected adults not on ART<br><br>**Secondary Objective**<br>• To determine if PGT121 induces anti-PGT121 antibodies<br>• To determine the effect of PGT121 mAb on CD4+ T cell counts in HIV-infected adults<br>• To determine if PGT121 mAb induces viral escape mutants in viremic HIV-infected adults not on ART<br><br>**Exploratory Objectives**<br>• To determine if PGT121 mAb has any impact on the host immune responses (i.e., HIV-specific cellular and humoral immune response)<br>• To determine the effect of PGT121 mAb on the size of the latent HIV reservoir in HIV-infected adults<br>• To determine PGT121 mAb levels in mucosal secretions in HIV-uninfected and HIV-infected adults<br>• To measure in vitro neutralization of HIV isolates with participant's serum post PGT121 mAb IV infusion<br>• To determine if PGT121 mAb has any impact on resistance mutations to ARVs

This copy of the document was retrieved from the system by Lisa Sunner on 23 Oct 2018

**ENDPOINTS**	***Primary*:**<br><br>*Safety and Tolerability:*<br>We will measure the following endpoints to evaluate the safety and tolerability of PGT121 mAb in HIV-uninfected and HIV-infected adults:<br><br>1. Proportion of participants with moderate or greater reactogenicity (e.g., solicited adverse events) for 3 days following IV infusion or SC injection of PGT121 mAb.<br>2. Proportion of participants with moderate or greater and/or PGT121 mAb-related unsolicited adverse events (AEs), including safety laboratory (biochemical, hematological) parameters, following IV infusion or SC injection of PGT121 mAb for the first 56 days post administration of Investigational Product.<br>3. Proportion of participants with PGT121 mAb-related serious adverse events (SAEs) throughout the study period.<br><br>*Pharmacokinetics:*<br>We will measure the following endpoints to determine the pharmacokinetics following IV infusion or SC injection of PGT121 mAb in HIV-uninfected and HIV-infected adults:<br><br>1. Elimination half-life (t1/2)<br>2. Clearance (CL/F)<br>3. Volume of distribution (Vz/F)<br>4. Area under the concentration decay curve (AUC)<br>5. Impact of viral load and/or ART on PGT121 disposition (elimination half-life (t1/2), clearance (CL/F), volume of distribution (Vz/F), total exposure<br><br>*Antiviral activity:*<br>We will calculate the following endpoints to determine the antiviral activity following IV infusion of PGT121 mAb in viremic HIV-infected adults not on ART:<br><br>1. Change in plasma HIV-1 RNA levels from baseline (mean of pre-entry and entry values)<br><br>***Secondary*:**<br><br>*Anti-PGT121 antibodies:*<br>We will calculate the following endpoint to determine if anti-PGT121 antibodies are elicited following IV infusion or SC injection of PGT121 mAb in HIV-uninfected and HIV-infected adults:<br><br>1. Change in serum anti-PGT121 antibody titers from baseline<br><br>*CD4+ T cell count:*<br>We will calculate the following endpoint to determine if IV infusion of

This copy of the document was retrieved from the system by Lisa Sunner on 23 Oct 2018

PGT121 mAb has any impact on CD4+ T cell counts in HIV-infected adults:

1. Change in CD4+ T cell count and frequency compared to baseline as measured by single platform flow cytometry

HIV genotyping/phenotyping of circulating virus for evaluation of PGT121 mAb -induced escape mutations:
We will compare plasma virus sequences before and after IV infusion of PGT121 mAb to determine if PGT121 mAb induces viral escape mutations in viremic HIV-infected adults not on ART.

1. Development of sequence variations in epitopes known to result in reduced PGT121 mAb neutralization susceptibility.

Exploratory:

Additional assessments may include, but are not limited to, the following: HIV-specific IgG/IgA binding responses by ELISA, HIV-specific cellular immune responses by ELISPOT, HIV-specific antibody function by ADCC, ADCP, and ADCVI assays, resistance mutations to current ARVs, PGT121 mAb levels in mucosal secretions, changes in total HIV-1 DNA and 2-long terminal repeat (LTR) circular HIV-1 DNA in resting or total CD4 T cells and in vitro neutralization of HIV isolates with participant's serum post administration of PGT121 mAb.

This copy of the document was retrieved from the system by Lisa Sunner on 23 Oct 2018

CONFIDENTIAL

STUDY DESIGN TABLE

	Group	Participants	Sub-Group	Regimen	N	Dose (mg/kg) - administration
Part 1[1]	1[3]	HIV-uninfected participants	1A	PGT121/Placebo	4/1 (6/2 if DLT[2])	3 IV
			1B	PGT121/Placebo	4/1 (6/2 if DLT)	10 IV
			1C	PGT121/Placebo	4/1 (6/2 if DLT)	30 IV
			1D	PGT121/Placebo	4/1 (6/2 if DLT)	3 SC
	2[3]	HIV-infected on ART, (<50 cp/ml)	2A	PGT121/Placebo	4/1 (6/2 if DLT[2])	3 IV
			2B	PGT121/Placebo	4/1 (6/2 if DLT)	10 IV
			2C	PGT121/Placebo	4/1 (6/2 if DLT)	30 IV
Safety Monitoring Committee Review[4]						
Part 2	3[5]	HIV-infected off ART (VL $2x10^3$ – $1x10^5$cp/ml)	3A	PGT121	6 (max 9)	30 IV
		HIV-Infected off ART (VL $1x10^2$ – $2x10^3$ cp/ml)	3D	PGT121	6	30 IV

DLT, dose limiting toxicity; ART, antiretroviral therapy; cp, copies; ml, milliliter
Administration of PGT 121 will be by intravenous infusion (IV) or subcutaneous injection (SC)

1. Eligible participants for Groups 1 and 2 will be enrolled according to their HIV-serostatus and will occur in parallel. At each dose level in Part 1, investigational product (IP) administration will be separated by at least 24 hours for each of the first 3 participants. Randomization will ensure at least 2 participants receive active product and are observed for at least 24 hours before administration to additional participants.
2. A dose limiting toxicity (DLT) will be defined as 1) any Grade 3 or greater reactogenicity or adverse event judged by the study investigators as at least possibly related to investigational product or 2) any SAE considered at least possibly related to investigational product.
3. The PSRT will review safety data to determine dose escalation. If no DLT occurs within 2 weeks from infusion of the first 5 participants in a dose subgroup (e.g. 1A), dose escalation to the next dose subgroup will proceed (e.g. 1B). If 1 DLT occurs in a dose subgroup (e.g. 1A), 3 additional participants will be enrolled into that dose subgroup; these 3 participants will be randomized between PGT121 mAb and placebo at a 2:1 ratio. If no additional DLTs occur in that subgroup (e.g. 1A) within 2 weeks of infusion in the 8 total participants (3:1 ratio of PGT121 mAb to placebo), the study can proceed with enrolment of the next dose subgroup (e.g. 1B). If 2 or more DLTs accumulate in a dose subgroup (e.g. 1A) that are the same, similar, or in the same System Organ Class, dosing will be halted in that subgroup (e.g. 1A) and the next lower dose level will be declared the maximum tolerated dose (MTD) for that subgroup (e.g. 1A). When groups are enrolled in parallel, if the MTD is determined in one group (e.g. Group 1) due to the occurrence of 2 or more DLTs in this group, dosing of participants in the parallel group (e.g. Group 2) will be held until the PSRT has reviewed the safety data and determined whether the MTD should be applied to both groups. If no DLT occurs in one of the final dose subgroups after 14 days of follow up, MTD will be the highest dose given (subgroups 1C or 2C 30mg/kg). Dose escalation in Groups 1 and 2 may occur in parallel, and can occur independent of each other.

This copy of the document was retrieved from the system by Lisa Sunner on 23 Oct 2018

	4. Following IP administration of IV PGT121 in the last participant in either Group 1 or 2, an independent Safety Monitoring Committee (SMC) will review at least the first 14 days of safety data to confirm MTD, and determine whether, and at what dose, Group 3 can initiate enrolment. The SMC can meet to confirm MTD when either Group 1 or Group 2 has finished enrolment, and does not need to wait for both groups to be completed to approve initiation of Group 3 enrolment. Data from Group 1D (SC administration) is not considered by SMC for initiation of Group 3 enrolment.<br>*5.* Group 3 will start with the MTD as determined in Part 1.
**METHODS**	See Schedule of Procedures, Appendices A, B and C
**STUDY POPULATION**	The study population will include three different groups:<br>Group 1 will include HIV-uninfected males or females aged 18-50 years old who are willing to maintain low risk behavior for HIV infection; principal exclusion criteria include confirmed HIV-infection, pregnancy or lactation, significant acute or chronic disease and clinically significant laboratory abnormalities. Group 2 will include HIV-infected males or females aged 18-65 years old on a stable antiretroviral regimen with HIV-1 RNA plasma level <50 copies/ml, CD4 cell count ≥ 300 cells/uL; principal exclusion criteria include history of AIDS-defining illness within the previous 5 years, significant acute or chronic medical condition other than HIV infection, and clinically significant laboratory abnormalities. Group 3 will include HIV-infected males or females aged 18-65 years old, not on antiretroviral therapy for > 6 month with detectable HIV-1 viral load between 100 and 100,000 copies/ml, CD4 cell count ≥ 300 cells/uL; principal exclusion criteria include history of AIDS-defining illness within the previous 5 years, significant acute or chronic medical condition other than HIV infection, and clinically significant laboratory abnormalities.
**NUMBER OF PARTICIPANTS**	47-71 participants will be included.
**DOSE ESCALATION and PAUSE RULES**	The first part of this study is a dose-escalation trial in HIV-uninfected adults and HIV-infected adults on ART with suppressed viral load, as indicated in the study design table.<br><br>If 2 or more DLTs accumulate in a dose subgroup that are the same, similar, or in the same System Organ Class, dosing will be halted and the next lower dose level will be declared the maximum tolerated dose (MTD) within this group. If no DLT occurs in one of the final dose groups after 14 days of follow-up, MTD will be the highest dose given (subgroups 1C or 2C 30mg/kg).<br><br>The Protocol Safety Review Team (PSRT) will review safety data through at least day 14 post-IP administration for the first 5 participants in each dose subgroup (e.g. 1A) prior to allowing enrolment of participants into the next dose subgroup (e.g. 1B). Dose escalation in Groups 1 and 2 may occur in parallel, and can occur independent of each other.<br><br>Following IP administration of IV PGT121 in the last participant in either Group 1 or 2, an independent Safety Monitoring Committee (SMC) will review safety data through at least day 14 post-IP administration for all participants to confirm MTD, and determine whether, and at what dose, Group 3 can initiate enrolment. The SMC can meet to confirm MTD when either Group 1 or Group 2 has finished enrolment, and does not need to

This copy of the document was retrieved from the system by Lisa Sunner on 23 Oct 2018

	wait for both groups to be completed to approve initiation of Group 3 enrolment. Data from Group 1D (SC administration) is not considered by SMC for initiation of Group 3 enrolment.<br><br>The second part of this study is conducted in HIV-infected adults not on ART, as indicated in the study design table.<br><br>The study will be paused for a safety review by the investigators and the independent SMC if 1) 1 or more participants experiences a Serious Adverse Event that is judged possibly, probably or definitely related to the IP, 2) There is a participant death, regardless of relationship to the IP, 3) if 2 or more participants experience grade 3 adverse events in the same System Organ Class that are considered to be at least possibly related to IP or 4) any grade 4 adverse event that is considered to be possibly, probably, or definitely related to IP. See protocol section 17.3.
**FORMULATIONS, VOLUMES AND ROUTES OF ADMINISTRATION**	**PGT121 mAb**: PGT121 mAb is a recombinant, fully human monoclonal antibody (mAb) of the IgG1 isotype that binds to the HIV envelope. The concentration and volume of product in each vial is 50 mg/mL, 6mL in each vial. PGT121 mAb will be given intravenously or subcutaneously in this study.
**DURATION OF STUDY PARTICIPATION**	Participants will be screened up to 56 days (Groups 1 and 2) or 42 days (Group 3) before IP administration and will be followed for 24 weeks. The anticipated study duration for each participant is approximately 6 months from screening through last study visit. It is anticipated that it will take approximately 6 months to enroll Groups 1 and 2. It is anticipated that it will take approximately 16 months to enroll the entire study. The exact duration depends on the recruitment rate and how many participants will be required per dose group.
**RANDOMIZATION and BLINDING**	This is a randomized, placebo-controlled study for groups 1 and 2, and open label for group 3 who will not receive placebo.
**EVALUATION FOR INTERCURRENT HIV INFECTION:**	Participants in Group 1 (HIV-uninfected) will be tested for HIV according to the Schedule of Procedures. Test results will be interpreted according to a pre-determined diagnostic algorithm. HIV testing at additional time points may be performed upon the request of the participant and Principal Investigator or designee as medical or social circumstances warrant.
**SAFETY MONITORING AND STATISTICAL CONSIDE-RATIONS:**	All clinical trial data collected, identified only by a study identification number, will be entered into the clinical trial database.<br><br>Safety will continually be monitored by the Investigators, the Sponsor's Medical Monitor and a Protocol Safety Review Team (PSRT); detailed pause criteria are pre-defined.<br><br>Safety data will be reviewed by an independent Safety Monitoring Committee (SMC). *Ad hoc* safety review may be specifically requested by the Sponsor, the Principal Investigators, Ethics Committees, Regulatory Authorities, or by the SMC. All clinical and routine laboratory data will be included in the safety analysis. At the end of the study, a full analysis will be prepared.

This copy of the document was retrieved from the system by Lisa Sunner on 23 Oct 2018

TABLE OF CONTENTS

ABBREVIATIONS **11**

CONTACT INFORMATION **12**

1.0 SIGNATURE PAGE **13**

2.0 INTRODUCTION AND BACKGROUND INFORMATION **14**

2.1 Study Rationale 14
2.2 Experience with PGT121 15

3.0 STUDY OBJECTIVES **15**

3.1 Primary Objectives 15
3.2 Secondary Objectives 15
3.3 Exploratory Objectives: 15

4.0 STUDY ENDPOINTS **16**

4.1 Study Endpoints 16
4.1.1 Primary Endpoints 16
4.1.2 Secondary Endpoints 16
4.1.3 Exploratory Endpoints 17

5.0 STUDY DESIGN **17**

5.1 Definition of Dose Limiting Toxicity and Maximum Tolerated Dose 17
5.2 Dose Escalation – Groups 1 and 2: Determination of Maximum Tolerated Dose 17
5.3 Group 3: Determination of Antiviral Activity 19
5.4 Duration of the Study 20
5.5 Study Population 20
5.6 Inclusion Criteria 21
5.7 Exclusion Criteria 23
5.8 Recruitment of Participants 26

6.0 STUDY VISITS **26**

6.1 Screening Period 26
6.2 IV infusion or SC injection of PGT121 mAb Visit 27
6.3 Post-IV infusion or SC injection of PGT121 mAb Visits 28
6.4 Additional Follow-up Visits 28
6.5 Unscheduled Visits 29
6.6 Final Study Visit or Early Termination Visit 29

7.0 STUDY PROCEDURES **29**

7.1 Informed Consent Process 29
7.2 Medical History and Physical Examination 29
7.3 HIV Testing and HIV-test Counselling (Group 1) 30
7.4 HIV Risk Reduction Counselling 30
7.5 Family Planning Counselling 30
7.6 ART Counselling (Group 3) 31
7.7 Specimens 31
7.8 Reimbursement 31
7.9 Randomization and Blinding 31
7.10 Un-blinding Procedure for Individual Participants 32
7.11 Assessment of IP related HIV sero-positivity 32

This copy of the document was retrieved from the system by Lisa Sunner on 23 Oct 2018

8.0 INVESTIGATIONAL PRODUCT 32

8.1 Description 32
8.2 Shipment and Storage 33
8.3 Preparation of Investigational Product (IP) 33
8.4 Administration of Investigational Product 34
8.5 Accountability and Disposal of Investigational Product 34

9.0 ASSESSMENTS 34

9.1 Safety Assessments 34
9.1.1 Local reactogenicity 34
9.1.2 Systemic reactogenicity 34
9.1.3 Vital Signs 35
9.1.4 Other Adverse Events 35
9.1.5 Concomitant Medications 35
9.1.6 Routine laboratory parameters 35
9.1.7 Specific screening tests: 36
9.2 Virologic Assessments 36
9.3 Exploratory Immunogenicity Assessments 37
9.3.1 Antibody Responses 37
9.3.2 Cellular Responses 37
9.3.3 PBMC, Serum and Plasma Storage 37
9.4 Other Assessments 38
9.4.1 HIV Antibody Testing (Group 1) 38
9.4.2 Pharmacokinetics 38
9.4.3 HLA Typing 38
9.4.5 Pregnancy Test 38
9.4.6 HIV Risk Assessment (Group 1) 38
9.4.7 Social Impact Assessment 39

10.0 ADVERSE EVENTS 39

10.1 Definition 39
10.2 Assessment of Severity of Adverse Events 39
10.3 Relationship to Investigational Product 39
10.4 Serious Adverse Events 40
10.5 Reporting Potential Immune-Mediated Diseases 41
10.6 Clinical Management of Adverse Events 42
10.7 Pregnancy 43
10.8 Intercurrent HIV Infection (Group 1) 43

11.0 MANAGEMENT OF HIV ISSUES DURING AND FOLLOWING STUDY 43

11.1 HIV Testing – Group 1 43
11.2 Social Discrimination as a Result of IP-related antibodies 44
11.3 HIV infection – Group 1 44
11.3.1 Counselling 44
11.3.2 Referral for Support/Care 44

12.0 WITHDRAWAL FROM STUDY 44

12.1 Deferral of IV infusion or SC injection of IP 44
12.2 Withdrawal from the Study (Early Termination) 45

13.0 DATA HANDLING 45

13.1 Data Collection and Record Keeping at the Study Site 45

This copy of the document was retrieved from the system by Lisa Sunner on 23 Oct 2018

13.3 Data Entry at the Study Site 46
13.4 Data Analysis 46

14.0 STATISTICAL CONSIDERATIONS 47

14.1 Safety and Tolerability Analysis 47
14.2 Pharmacokinetic Analysis 48
14.3 Virologic Analysis for Group 3A 48
14.4 Analysis of Antiviral Activity in Subgroup 3D 50
14.5 Secondary and Exploratory Immunologic and Virologic Analyses 50

15.0 QUALITY CONTROL AND QUALITY ASSURANCE 51

16.0 DATA AND BIOLOGICAL MATERIAL 51

17.0 ADMINISTRATIVE STRUCTURE 51

17.1 Protocol Safety Review Team 51
17.2 Safety Monitoring Committee (SMC) 52
17.2.1 Content of Interim Safety Review 52
17.2.2 SMC Review of Group 1 and 2 data prior to starting Group 3 52
17.3 Criteria for Pausing the Study 52
17.4 Study Supervision 53
17.5 Study Monitoring 54
17.6 Investigator's Records 54

18.0 INDEMNITY 54

19.0 PUBLICATION 54

20.0 ETHICAL CONSIDERATIONS 55

APPENDIX A: SCHEDULE OF PROCEDURES – GROUP 1 (A, B, C, D) 56

APPENDIX B: SCHEDULE OF PROCEDURES – GROUP 2 (A, B, C) 58

APPENDIX C: SCHEDULE OF PROCEDURES – GROUP 3 (A, D) 61

APPENDIX D: LOW RISK CRITERIA 64

APPENDIX E: ADVERSE EVENT SEVERITY ASSESSMENT TABLE 66

APPENDIX F CTCAE TABLE 66

APPENDIX G REFERENCES 77

ABBREVIATIONS

Abbreviation	Term
**AE**	Adverse Event
**AIDS**	Acquired Immunodeficiency Syndrome
**ALT**	Alanine-Aminotransferase
**ART**	Antiretroviral Therapy
**AST**	Aspartate-Aminotransferase
**CFC**	Cytokine Flow Cytometry
**CMI**	Cell Mediated Immunity
**CRF**	Case Report Form
**CTL**	Cytotoxic T Lymphocyte
**DCC**	Data Coordinating Center
**DLT**	Dose Limiting Toxicity
**DNA**	Deoxyribonucleic Acid
**ELISA**	Enzyme Linked Immunosorbent Assay
**GCP**	Good Clinical Practice
**GFR**	Glomerular Filtration Rate
**HIV**	Human Immunodeficiency Virus
**HLA**	Human Leukocyte Antigen
**HSV**	Herpes Simplex Virus
**IAVI**	International AIDS Vaccine Initiative
**ICH**	International Council for Harmonization of Technical Requirements for Pharmaceuticals for Human Use
**IP**	Investigational Product
**IND**	Investigational New Drug Application
**IV**	Intravenous
**Kg**	Kilogram
**mAb**	Monoclonal Antibody
**mg**	Milligram
**MED**	Minimum Effective Dose
**MTD**	Maximum Tolerated Dose
**NHP**	Non Human Primate
**PCR**	Polymerase Chain Reaction
**PBMC**	Peripheral Blood Mononuclear Cells
**PK**	Pharmacokinetic
**RPR**	Rapid Plasma Reagin
**SAE**	Serious Adverse Event
**SIV**	Simian Immunodeficiency Virus
**SOP**	Standard Operating Procedure
**SOM**	Study Operations Manual
**SMC**	Safety Monitoring Committee
**STD**	Sexually Transmitted Disease
**TPHA**	Treponema Pallidum Hemagglutination

This copy of the document was retrieved from the system by Lisa Sunner on 23 Oct 2018

CONTACT INFORMATION

Detailed contact information provided in the Study Operation Manual (SOM)

**Sponsor Contact:**	
Frances Priddy MD MPH<br>Executive Director and Chief Medical Officer<br>International AIDS Vaccine Initiative<br>125 Broad Street, 9th Floor<br>New York, New York 10004	Phone: +1-212-328-7461<br>Mobile: +1-646-287-8943<br>Fax: +1-608-203-5501<br>E-mail: fpriddy@iavi.org
**Clinical Research Center Contacts:**	
Kathryn Stephenson MD MPH<br>Center for Virology and Vaccine Research<br>Clinical Trials Unit<br>Beth Israel Deaconess Medical Center<br>E / CLS – 1036<br>330 Brookline Avenue<br>Boston, Massachusetts 02215	Phone: +1-617-735-4556<br>Mobile: +1-917-836-9150<br>Fax: +1-617-735-4566<br>E-mail: kstephen@bidmc.harvard.edu

.

This copy of the document was retrieved from the system by Lisa Sunner on 23 Oct 2018

1.0 SIGNATURE PAGE

The signatures below constitute the approval of this protocol and the appendices and provide the necessary assurances that this study will be conducted in compliance with the protocol, Good Clinical Practices (GCP) and the applicable regulatory requirement(s).

Principal Investigator:

Signed: ______________________________ Date: ______________

Name (please print): ______________________________

Name of institution (please print): ______________________________

This copy of the document was retrieved from the system by Lisa Sunner on 23 Oct 2018

2.0 INTRODUCTION AND BACKGROUND INFORMATION

More than 78 million people have been infected with HIV and 39 million people have died since the beginning of the AIDS epidemic[1]. In 2014, there were 1.2 million deaths attributable to HIV infection and 2 million newly infected with HIV[2]. One reason that such high rates of AIDS-related deaths continue to occur globally – despite the advent of drugs that are highly effective at suppressing HIV replication – is that only two in five people living with HIV have access to antiretroviral therapy[1]. The other reason for continued AIDS-related mortality is that ART does not cure HIV infection and must be maintained for a lifetime. Even in the United States (US), only 30% of the 1.2 million people living with HIV have suppressed HIV to undetectable levels, despite the fact that most HIV-infected people in the US have access to ART[3] It is clear that antiretroviral therapy is necessary but not sufficient to end the AIDS epidemic, both in the US and globally, and that novel efforts to prevent and treat HIV are critically needed.

2.1 Study Rationale

This is a Phase 1 study to evaluate the safety, tolerability, pharmacokinetics and anti-viral efficacy of the PGT121 monoclonal antibody for HIV prevention and therapy. PGT121 mAb is a recombinant human IgG1 monoclonal antibody that targets a V3 glycan-dependent epitope region of the HIV envelope protein[4,5]. PGT121 mAb was chosen for this study because it is potent, neutralizes a wide array of HIV viruses, and can prevent and treat simian-human immunodeficiency virus (SHIV) in rhesus monkeys.

The recent discovery of multiple potent and broadly neutralizing antibodies (bNAbs) against HIV has led to the re-emergence of the concept that antibodies may be useful for both prevention and therapy. HIV-specific antibodies that target the HIV envelope (Env) can prevent SHIV infection in rhesus monkeys and have shown to reduce HIV RNA levels in humans temporarily[6-10]. Until recently, these antibodies were few in number, targeted a narrow spectrum of HIV strains and Env epitopes, and were not potent enough for practical use. In the last five years, the field has changed dramatically: new developments in high throughput single-cell PCR-amplification and novel soluble Env baits have led to the isolation of new monoclonal antibodies with extraordinary potency and breadth[11]. These bNAbs may be effective for prevention of HIV infection when administered passively[12,13].

PGT121 mAb was selected for development because of the following critical attributes:

- PGT121 mAb is 10 to 100-fold more potent than the previous best-in-class CD4bs antibodies VRC01, VRC07, and 3BNC117 [11,14,15].
- PGT121 mAb affords superior protective efficacy against SHIV acquisition in monkeys compared to VRC01, 3BNC117, and 10-1074 [16] (and unpublished data).
- PGT121 mAb has superior therapeutic efficacy in SHIV-infected monkeys compared to VRC01, 3BNC117, and 10-1074 [7] (and unpublished data).
- PGT121 mAb may have a higher bar to escape in vivo as compared with other V3 glycan and CD4bs antibodies as a result of making multiple glycan contacts [14].
- PGT121 mAb combined with PGDM1400 (a novel bNab targeting the envelope trimer apex) neutralizes 98-99% of global HIV-1 viruses tested and has unparalleled potency with a median IC50 of 0.007 µg/ml[14].

The potency and breadth of PGT121 mAb, both alone and in combination with other bNAbs, raise the possibility that combinations may be effective for HIV prophylaxis at low doses and against global viruses. An antibody that is effective at low doses may eventually be given subcutaneously, which would reduce the cost. It is these features that make PGT121 mAb particularly well-suited for preventing and/or treating HIV in the developing world, where it is critical that a public health intervention be low cost, easy to deliver, and effective in diverse settings.

2.2 Experience with PGT121

There is no previous clinical experience with PGT121 mAb. Several other HIV monoclonal antibodies are currently in clinical development as passive HIV immunoprophylaxis, or as potential therapeutics. Data from phase 1 studies shows acceptable preliminary safety and tolerability profiles for these products, but varying levels of anti-viral effects [6,17]. A comprehensive summary of phase 1 studies of HIV monoclonal antibodies can be found in the Investigator's Brochure.

3.0 STUDY OBJECTIVES

3.1 Primary Objectives

- To evaluate the safety and tolerability of a single intravenous (IV) infusion of PGT121 mAb at 3, 10, and 30 mg/kg in HIV-uninfected adults and HIV-infected adults.
- To evaluate the safety and tolerability of a single subcutaneous (SC) injection of PGT121 mAb at 3 mg/kg in HIV-uninfected adults
- To evaluate the pharmacokinetic (PK) profile of IV infusion and SC injection of PGT121 mAb in HIV-uninfected and HIV-infected adults.
- To evaluate the antiviral activity of IV infusion of PGT121 mAb in HIV-infected adults not on ART.

3.2 Secondary Objectives

- To determine if PGT121 mAb induces anti-PGT121 antibodies.
- To determine the effect of PGT121 mAb on CD4 T-cell counts in HIV-infected adults.
- To determine if PGT121 mAb induces viral escape mutants in viremic HIV-infected adults not on ART.

3.3 Exploratory Objectives:

- To determine if PGT121 mAb has any impact on the host immune responses (i.e., HIV-specific cellular and humoral immune response).
- To determine the effect of PGT121 mAb on the size of the latent HIV reservoir in HIV-infected adults.
- To determine PGT121 mAb levels in mucosal secretions in HIV-uninfected and HIV-infected adults.
- To measure in vitro neutralization of HIV isolates with participant's serum post PGT121 mAb IV infusion.
- To determine if PGT121 mAb has any impact on resistance mutations to ARVs.

This copy of the document was retrieved from the system by Lisa Sunner on 23 Oct 2018

4.0 STUDY ENDPOINTS

4.1 Study Endpoints

4.1.1 Primary Endpoints

Safety and Tolerability:

We will measure the following endpoints to evaluate the safety and tolerability of PGT121 mAb in HIV-uninfected and HIV-infected adults:

1. Proportion of participants with moderate or greater reactogenicity (e.g., solicited adverse events) for 3 days following IV infusion or SC injection of PGT121 mAb.
2. Proportion of participants with moderate or greater and/or PGT121 mAb-related unsolicited adverse events (AEs), including safety laboratory (biochemical, hematological) parameters, following IV infusion or SC injection of PGT121 mAb for the first 56 days post administration of Investigational Product.
3. Proportion of participants with PGT121 mAb-related serious adverse events (SAEs) throughout the study period.

Pharmacokinetics:

We will measure the following endpoints to determine the pharmacokinetics following IV infusion or SC injection of PGT121 mAb in HIV-uninfected and HIV-infected adults:

1. Elimination half-life (t1/2)
2. Clearance (CL/F)
3. Volume of distribution (Vz/F)
4. Area under the concentration decay curve (AUC)
5. Impact of viral load and/or ART on PGT121 disposition (elimination half-life (t1/2), clearance (CL/F), volume of distribution (Vz/F), total exposure

Antiviral activity:

We will calculate the following endpoints to determine the antiviral activity following IV infusion of PGT121 mAb in viremic HIV-infected adults not on ART.

1. Change in plasma HIV-1 RNA levels from baseline (mean of pre-entry and entry values)

4.1.2 Secondary Endpoints

Anti-PGT121 antibodies:

We will calculate the following endpoint to determine if anti-PGT121 antibodies are elicited following IV infusion or SC injection of PGT121 mAb in HIV-uninfected and HIV-infected adults:

This copy of the document was retrieved from the system by Lisa Sunner on 23 Oct 2018

1. Change in serum anti-PGT121 antibody titers from baseline

CD4+ T cell count:

We will calculate the following endpoint to determine if IV infusion of PGT121 mAb has any impact on CD4+ T cell counts in HIV-infected adults:

1. Change in CD4+ T cell count and frequency compared to baseline as measured by single platform flow cytometry

HIV genotyping/phenotyping of circulating virus for evaluation of PGT121-induced escape mutations:

We will compare plasma virus sequences before and after IV infusion of PGT121 mAb to determine if PGT121 mAb induces viral escape mutations in viremic HIV-infected adults not on ART.

1. Development of sequence variations in epitopes known to result in reduced PGT121 neutralization susceptibility

4.1.3 Exploratory Endpoints

Additional assessments may include but are not limited to the following: HIV-specific IgG/IgA binding responses by ELISA, HIV-specific cellular immune responses by ELISPOT, HIV-specific antibody function by ADCC, ADCP, and ADCVI assays, resistance mutations to current ARVs, PGT121 mAb levels in mucosal secretions, changes in total HIV-1 DNA and 2-long terminal repeat (LTR) circular HIV-1 DNA in resting or total CD4 T cells and in vitro neutralization of HIV isolates with participant's serum post administration of PGT121 mAb.

5.0 STUDY DESIGN

The study is a randomized, placebo-controlled study for Groups 1 and 2, and open label for Group 3 who will not receive placebo.

5.1 Definition of Dose Limiting Toxicity and Maximum Tolerated Dose

A dose limiting toxicity (DLT) will be defined as 1) any Grade 3 or greater reactogenicity or adverse event judged by the study investigators as at least possibly related to investigational product or 2) any SAE considered at least possibly related to investigational product.
Maximum Tolerated Dose (MTD) will be declared when 2 or more DLTs occur that are the same, similar, or in the same System Organ Class or if no DLT occurs in the final dose subgroup after 14 days of follow-up, MTD will be the highest dose given (groups 1C or 2C 30mg/kg).

5.2 Dose Escalation – Groups 1 and 2: Determination of Maximum Tolerated Dose

In Groups 1 and 2, the administrations of PGT121 mAb escalate by dose as shown below in Table 5.3.1, Study Design (5 participants per dose subgroup, 4:1 ratio of IP to placebo for each dose subgroup).

This copy of the document was retrieved from the system by Lisa Sunner on 23 Oct 2018

Sentinel Recipients

Within each dose group (subgroups 1A and 2A, subgroups 1B and 2B, subgroups 1C and 2C, subgroup 1D), the first 3 participants will be separated by at least 24 hours, to allow for observation of Investigational product (IP)-related adverse events. Dose subgroups will be enrolled in parallel, meaning that the 1st participant may be from subgroup 1A, the 2nd from subgroup 2A, the 3rd from subgroup 2A, all with 24 hours in between dosing. Because there is 1 placebo in each dose subgroup and the subgroups are dosed in parallel, the first 3 recipients will be treated as sentinel recipients (randomization will ensure that at least 2 will receive the IP).

- If no reactogenicity and adverse events that are considered to be related to IP (possibly, probably or definitely related) and are graded as severe or worse (Grade 3 or 4 on the DAIDS Toxicity Table or CTCAE table, see section 9.1.2) occur within 24 hours after infusion of the first participant, the second participant may be injected.
- If no events meeting the criteria described above occur within 24 hours after the 3rd participant is infused, then the remainder of participants in that dose group will be infused.
- If events meeting the criteria described above do occur for the first, second, or third participant in a dose group, they will be reviewed by the Safety Monitoring Committee (SMC) to determine whether further infusions may proceed.

Safety information will be reviewed by the Principal Investigator. The outcome of the safety review, and decision whether or not to dose the next participant(s) or contact the SMC will be communicated with the Sponsor.

Dose Escalation and Determination of Maximum Tolerated Dose

Safety data through at least day 14 post-IP administration visit for the first 5 participants in a dose subgroup (e.g. 1A) will be reviewed by the Protocol Safety Review Team (PSRT) prior to allowing enrolment of participants into the next dose subgroup (e.g. 1B). Dose escalation in Groups 1 and 2 may occur in parallel, and can occur independent of each other. Following administration of IP for the last participant in either Group 1C or 2C, an independent Safety Monitoring Committee (SMC) will review safety data to confirm Maximum Tolerated Dose (MTD) and determine whether, and at what dose, Group 3 can initiate enrolment. The SMC can meet to confirm MTD when either Group 1C or Group 2C has finished enrolment, and does not need to wait for both Groups to be completed to approve initiation of Group 3 enrolment. Data from Group 1D (SC administration) is not considered by SMC for initiation of Group 3 enrolment.

- If no DLT occurs within 2 weeks from infusion of the first 5 participants in a dose subgroup (e.g. 1A), dose escalation to the next dose subgroup (e.g. 1B) will proceed.
- If 1 DLT occurs in a dose subgroup (e.g. 1A), 3 additional participants will be enrolled in that dose subgroup; these 3 participants will be randomized between PGT121 mAb and placebo at a 2:1 ratio.
    - If no additional DLTs occur in that dose subgroup (e.g. 1A) within 2 weeks of infusion in the 8 total participants (3:1 ratio of PGT121 mAb to placebo), the study can proceed with enrolment of the next dose subgroup (e.g. 1B).

This copy of the document was retrieved from the system by Lisa Sunner on 23 Oct 2018

- If 2 or more DLTs accumulate in a dose subgroup (e.g. 1A) that are the same, similar, or in the same organ class, dosing will be halted in that subgroup (e.g. 1A) and the next lower dose level will be declared the maximum tolerated dose (MTD) for that subgroup (e.g. 1A).

- When groups are enrolled in parallel, if the MTD is determined in one group (e.g. Group 1) due to the occurrence of 2 or more DLTs in this group, dosing of participants in the parallel group (e.g. Group 2) will be held until the PSRT has reviewed the safety data and determined whether the MTD should be applied to both groups.
- If no DLT occurs in the one of the final dose subgroups, MTD will be the highest dose given (subgroups 1C or 2C 30mg/kg) after 14 days of follow-up.

5.3 Group 3: Determination of Antiviral Activity

Group 3 will initiate enrolment upon approval by the SMC (see section 17.2.2), Group 3 (Part 2) will start with the MTD (i.e. subgroups 3A and 3D if the MTD is 30 mg/kg).

At least 6 but up to 9 participants will be enrolled in subgroup 3A. Six participants will be enrolled in subgroup 3D.

Table 5.3.1 Study Design Table

	Group	Participants	Sub-Group	Regimen	N	Dose (mg/kg) administration
Part 1	1	HIV-uninfected participants	1A	PGT121/Placebo	4/1 (6/2 if DLT)	3 IV
			1B	PGT121/Placebo	4/1 (6/2 if DLT)	10 IV
			1C	PGT121/Placebo	4/1 (6/2 if DLT)	30 IV
			1D	PGT121/Placebo	4/1 (6/2 if DLT)	3 SC
	2	HIV-infected on ART, (<50 cp/ml)	2A	PGT121/Placebo	4/1 (6/2 if DLT)	3 IV
			2B	PGT121/Placebo	4/1 (6/2 if DLT)	10 IV
			2C	PGT121/Placebo	4/1 (6/2 if DLT)	30 IV
Safety Monitoring Committee Review						
Part 2	3	HIV-infected off ART (VL $2x10^3$ – $1x10^5$cp/ml)	3A	PGT121	6 (max 9)	30 IV
		HIV-infected off ART (VL $1x10^2$ – $2x10^3$ cp/ml)	3D	PGT121	6	30 IV

This copy of the document was retrieved from the system by Lisa Sunner on 23 Oct 2018

5.4 Duration of the Study

Participants will be screened up to 56 days (Groups 1 and 2) or 42 days (Group 3) before IP administration of PGT121 mAb and will be followed for 24 weeks.

It will take approximately 16 months to enroll the entire study. The exact duration depends on the recruitment rate and how many participants will be required per dose group as specified in sections 5.2 and 5.3.

5.5 Study Population

The study population consists of HIV-uninfected male or female adults (Group 1), HIV-infected male or female adults on ART (Group 2), and HIV-infected males and female adults not on ART (Group 3) who meet the detailed inclusion and exclusion criteria listed below, and who in the opinion of the investigator or designee, understand the study and provide written informed consent.

Approximately 47-71 participants (max 57 PGT121 recipients, 14 placebo recipients) who meet all eligibility criteria will be included in the study. An over-enrolment of up to 5% (up to 5 participants total) will be permitted in the study to facilitate rapid enrolment.

This copy of the document was retrieved from the system by Lisa Sunner on 23 Oct 2018

5.6 Inclusion Criteria

Inclusion criteria for all participants:

1. Willing to comply with the requirements of the protocol and available for follow-up for the planned duration of the study;

2. In the opinion of the Principal Investigator or designee and based on Assessment of Informed Consent Understanding results, has understood the information provided and potential impact and/or risks linked to IV infusion or SC injection and participation in the trial; written informed consent will be obtained from the participant before any study-related procedures are performed;

3. All heterosexually active female participants must commit to use an effective method of contraception for 3 months following IP administration, including:
    a. Condoms (male or female) with or without spermicide
    b. Diaphragm or cervical cap with spermicide
    c. Intrauterine device, or contraceptive implant
    d. Hormonal contraception
    e. Successful vasectomy in the male partner (considered successful if a woman reports that a male partner has [1] documentation of azoospermia by microscopy (< 1 year ago), or [2] a vasectomy more than 2 years ago with no resultant pregnancy despite sexual activity post-vasectomy)
    f. Not be of reproductive potential, such as having undergone hysterectomy, bilateral oophorectomy, or tubal ligation, postmenopausal (>45 years of age with amenorrhea for at least 2 years, or any age with amenorrhea for at least 6 months and a serum follicle stimulating hormone [FSH] level >40 IU/L); surgically sterile: no additional contraception required.

    Women, who are not heterosexually active at screening, must agree to utilize an effective method of contraception if they become hetero-sexually active, as outlined above.

4. All sexually active males, regardless of reproductive potential, must be willing to consistently use an effective method of contraception (such as consistent male condoms with male and/or female partners from the day of IP administration until at least 3 months following IP administration to avoid exposure of partners to IP in ejaculate, and to prevent conception with female partners.

5. All female participants must be willing to undergo urine pregnancy tests at time points indicated in the Schedule of Procedures and must test negative prior to IP administration;

6. A female participant must agree not to donate eggs (ova, oocytes) for the purpose of assisted reproduction until 3 months after receiving IP administration. A man must agree not to donate sperm until 3 months after IP administration;

This copy of the document was retrieved from the system by Lisa Sunner on 23 Oct 2018

7. Willing to forgo donations of blood and/or any other tissues, including bone marrow, during the study and, for those HIV-uninfected participants who test HIV-positive due to IP administration, until the anti-HIV antibody titers become undetectable.

Specific inclusion criteria for HIV-uninfected participants (Group 1):

8. At least 18 years of age on the day of screening and has not reached his or her 51st birthday on the day of signing the Informed Consent Document.

9. Willing to undergo HIV testing, risk reduction counselling and receive HIV test results;

10. Low risk for HIV infection (see section (9.4.6) and willing to maintain low-risk behaviour for the duration of the trial (Appendix D);

11. Healthy male or female, as assessed by a medical history, physical exam, and laboratory tests;

Specific inclusion criteria for HIV-infected participants (Groups 2 and 3):

12. At least 18 years of age on the day of screening and has not reached his or her 66th birthday on the day of signing the Informed Consent Document.

13. Confirmed HIV-1 infection (HIV Ab+ or HIV RNA+) by documentation in the medical records or in-clinic HIV testing;

14. CD4 ≥ 300 cells/μl;

15. No history of AIDS-defining illness within the previous 5 years

Group 2:

16. Currently on ART with no change in ART regimen in the 12 weeks before screening or between screening and enrolment, with suppression of plasma HIV-1 viral load < 50 copies / ml for greater than 6 months, and with a viral load < 50 copies / ml at time of screening (within 42 days prior to IP administration). cART is defined as a regimen including > 2 compounds, e.g. 2x nucleoside reverse transcriptase inhibitors plus either non-nucleoside reverse transcriptase inhibitor or protease inhibitor or integrase inhibitor. A change from tenofovir disoproxil fumarate to tenofovir alafenamide in the 12 weeks before screening is not an exclusion.

Group 3:

17. Not receiving cART, and (after appropriate counselling) willing to defer cART treatment for at least 56 days after administration of IP;

18. HIV-1 viral load either between 2000-100,000 copies / ml (Group 3A) or between 100-2000 copies / ml (Group 3D) confirmed at screening.

This copy of the document was retrieved from the system by Lisa Sunner on 23 Oct 2018

5.7 Exclusion Criteria

Exclusion criteria for all participants:

1. Any clinically significant acute or chronic medical condition, other than HIV infection, that is considered progressive or in the opinion of the investigator makes the participant unsuitable for participation in the study;

2. If female, pregnant, lactating or planning a pregnancy during the period of screening through completion of the study;

3. In the past 6 months a history of alcohol or substance use, including marijuana, judged by the Investigator to potentially interfere with participant study compliance;

4. Bleeding disorder that was diagnosed by a physician (e.g., factor deficiency, coagulopathy or platelet disorder that requires special precautions). Note: A participant who states that he or she has easy bruising or bleeding, but does not have a formal diagnosis and has intramuscular injections and blood draws without any adverse experience, is eligible;

5. History of a splenectomy;

6. Receipt of live attenuated vaccine within the previous 60 days or planned receipt within 60 days after administration of IP; or receipt of other vaccine within the previous 14 days or planned receipt within 14 days after infusion or injection with IP (exception is live attenuated influenza vaccine within 14 days);

7. Receipt of blood transfusion or blood-derived products within the previous 3 months;

8. Participation in another clinical trial of an investigational product currently, within the previous 3 months or expected participation during this study;

9. Prior receipt of an investigational HIV vaccine candidate, monoclonal antibody or polyclonal immunoglobulin (note: receipt of placebo in a previous HIV vaccine or monoclonal antibody trial will not exclude a participant from participation if documentation is available and the Medical Monitor gives approval);

10. History of severe local or systemic reactogenicity to injections or IV infusion (e.g., anaphylaxis, respiratory difficulties, angioedema);

11. Psychiatric condition that compromises safety of the participant and precludes compliance with the protocol. Specifically excluded are persons with psychoses within the past 3 years, ongoing risk for suicide, or history of suicide attempt or gesture within the past 3 years;

12. If, in the opinion of the Principal Investigator, it is not in the best interest of the participant to participate in the trial;

This copy of the document was retrieved from the system by Lisa Sunner on 23 Oct 2018

13. Seizure disorder: a participant who has had a seizure in the last 3 years is excluded. (Not excluded: a participant with a history of seizures who has neither required medications nor had a seizure for 3 years.)

14. Body mass index ≥ 35 or ≤ 18.0.

15. Infectious disease: chronic hepatitis B infection (HbsAg), current hepatitis C infection (HCV Ab positive and HCV RNA positive) or interferon-alfa treatment for chronic hepatitis C infection in the past year, or active syphilis. Hepatitis B infection that is suppressed on antiretroviral therapy with undetectable HBV DNA is allowable.

16. A history of malignancy within the past 5 years (prior to screening) or ongoing malignancy;

17. Active, serious infections (other than HIV-1 infection) requiring parenteral antibiotic, antiviral or antifungal therapy within 30 days prior to enrolment;

Specific exclusion criteria for HIV-uninfected participants (Group 1):

18. Confirmed HIV-1 or HIV-2 infection;

19. Any clinically relevant abnormality on history or examination including history of immunodeficiency or autoimmune disease; use of systemic corticosteroids, immunosuppressive, anticancer, or other medications considered significant by the investigator within the previous 6 months.

    The following exceptions are permitted and will not exclude study participation: use of corticosteroid nasal spray for rhinitis, topical corticosteroids for an acute uncomplicated dermatitis; or a short course (duration of 10 days or less, or a single injection) of corticosteroid for a non-chronic condition (based on investigator clinical judgment) at least 6 weeks prior to enrolment in this study.

Specific exclusion criteria for HIV-uninfected participants (Group 1) and HIV-infected participants who are on ART (Group 2):

20. Any of the following abnormal laboratory parameters listed below:

    <u>Hematology</u>

    – Hemoglobin <10.5 g/dL in females; hemoglobin <11.0 g/dL in males
    – Absolute Neutrophil Count (ANC): ≤1000/mm^3
    – Absolute Lymphocyte Count (ALC): < 650/mm^3
    – Platelets: < 125,000 mm^3 or ≥ 550,000/mm^3

    <u>Coagulation</u>

    - aPTT: >1.25x ULN
    - INR: ≥1.1 x ULN

Chemistry

- Sodium ≤135 mEq/L or ≥ 146 mEq/L
- Potassium ≤3.4 mEq/L or ≥ 5.6 mEq/L
- Creatinine ≥1.1 x ULN
- AST ≥1.25 x ULN
- ALT ≥1.25 x ULN
- Total bilirubin ≥1.25 x ULN
- Alkaline phosphatase ≥1.25 x ULN
- Albumin ≤ 3.0 g/dL or ≤ 30 g/L
- Creatine kinase ≥3.0 x ULN
- C-reactive protein >10 mg/L
- C3 complement < 0.82 g/L
- C4 complement < 0.14 g/L

Urinalysis

Any of the following abnormal findings if consistent with clinically significant disease:
– Protein = greater than trace on dipstick confirmed by microscopic urinalysis outside institutional range
– Blood = greater than trace on dipstick confirmed by ≥ 3 RBCs/hpf on microscopic urinalysis (not due to menses)

Specific exclusion criteria for HIV-infected participants who are on ART (Group 2) and for HIV-infected participants who are not on ART (Group 3):

21. Any clinically relevant abnormality on history or examination including history of immunodeficiency or autoimmune disease, other than HIV; use of systemic corticosteroids, immunosuppressive, anticancer, or other medications considered significant by the investigator within the previous 6 months.

    The following exceptions are permitted and will not exclude study participation: use of corticosteroid nasal spray for rhinitis, topical corticosteroids for an acute uncomplicated dermatitis; or a short course (duration of 10 days or less, or a single injection) of corticosteroid for a non-chronic condition (based on investigator clinical judgment) at least 6 weeks prior to enrolment in this study.

Specific exclusion criteria for HIV-infected participants who are not on ART (Group 3)

22. Any of the following abnormal laboratory parameters listed below:

Hematology

– Hemoglobin < 10.0 g/dL
– Absolute Neutrophil Count (ANC): <800 cells/mm3
– Platelets: < 100,000 cells/mm3

Coagulation

This copy of the document was retrieved from the system by Lisa Sunner on 23 Oct 2018

- aPTT: >1.25x ULN
- INR: ≥1.1 x ULN

Chemistry

- Estimated Glomerular filtration rate (GFR) ≤ 80 mL/min according to the Cockcroft-Gault formula for creatinine clearance:
  - Male: $\frac{(140 - \text{age in years}) \times (\text{wt in kg})}{72 \times (\text{serum creatinine in mg/dL})} = \text{CLcr (mL/min)}$
  - Female: $\frac{(140 - \text{age in years}) \times (\text{wt in kg}) \times 0.85}{72 \times (\text{serum creatinine in mg/dL})} = \text{CLcr (mL/min)}$
- AST ≥ 2.5 x ULN
- ALT ≥ 2.5 x ULN
- Total bilirubin ≥ 1.6 x ULN
- Alkaline phosphatase ≥ 5 x ULN

Urinalysis

Any of the following abnormal findings if consistent with clinically significant disease:

– Protein = greater than 1+ on dipstick confirmed by microscopic urinalysis outside institutional range

– Blood = greater than 1+ on dipstick confirmed by ≥ 10 RBCs/hpf on microscopic urinalysis (not due to menses)

- Leukocytes = greater than 1+ on dipstick confirmed by > 10 WBCs/hpf on microscopic urinalysis

5.8 Recruitment of Participants

Adult male and female participants may be recruited through in-clinic referrals, information presented to community organizations, hospitals, colleges, other institutions and/or advertisements to the general public or from existing cohorts. The information distributed will contain contact details of the trial site.

6.0 STUDY VISITS

6.1 Screening Period

During Screening, study staff will perform the following procedures:

- Provide and/or review the Informed Consent Document and answer any questions about the study prior to obtaining written informed consent.
- Complete Assessment of Informed Consent Understanding (AOU). Please refer to the Study Operations Manual (SOM)

If the participant agrees to participate, passes the AOU and provides written informed consent, study staff will:

- Conduct HIV test counselling, HIV testing, and HIV risk reduction counselling, as applicable

TMF-03562 | 7.0 | Status:Approved | 18 Oct 2018
This copy of the document was retrieved from the system by Lisa Sunner on 23 Oct 2018

- Conduct family planning counselling, refer for pregnancy prevention counselling if necessary
- Administer HIV risk assessment (Group 1)
- Conduct ART counselling (Group 3)
- Perform a comprehensive medical history
- Collect concomitant medication information
- Perform a general physical examination (Refer to Section 7.2)
- Collect specimens for all tests as indicated in the Schedule of Procedures in Appendices A, B and C (for details see Analytical Plan (AP)).

When available, the screening laboratory tests will be reviewed by the trial physician. Screening laboratory test(s) may be repeated once at the discretion of the principal investigator or designee to investigate any isolated abnormalities.

If the screening visit occurs outside the allowable screening window, all screening procedures must be repeated except the comprehensive medical history may be replaced by an interim medical history and the Participant Information Sheet of the Informed Consent Document should be reviewed.

If a participant has signed the Consent Form but does not meet the eligibility criteria, the records must be kept at the site.

6.2 IV infusion or SC injection of PGT121 mAb Visit

Prior to the administration of IP, study staff will:

- Answer any questions the participant may have about the study
- Review the Informed Consent Document with the participant
- Review screening safety laboratory data
- Conduct HIV test counselling, and HIV risk reduction counselling, as applicable
- Conduct ART counselling (Group 3)
- Conduct family planning counselling as per site specific procedures and ensure compliance with respective pregnancy prevention method, and discuss male condom use with all male participants
- Review interim medical history
- Collect concomitant medication information
- Weigh participant and record vital signs
- Perform a symptom-directed physical examination (Refer to Section 7.2)
- Assess at baseline local and systemic signs and symptoms (this includes an examination of IV infusion or SC injection site)
- Collect specimens for all tests as indicated in the Schedule of Procedures see Appendices A, B and C (for details see AP).
- Obtain pregnancy test results prior to administration of IP.

Assign an allocation number to the participant according to the instructions specified in the Study Operations Manual.

This copy of the document was retrieved from the system by Lisa Sunner on 23 Oct 2018

At the time of administration of IP and after IV infusion or SC injection of IP, study staff will:

- Administer the IP as specified in Section 8.4, Administration of Investigational Product and according to the instructions specified in the SOM.
- Observe participant closely during the infusion or injection of IP and for at least 30 minutes after IV infusion or SC injection of IP has ended for any acute reactogenicity. At the end of the observation period study staff will:
  - Record vital signs (pulse, respiratory rate, blood pressure and temperature)
  - Assess any local and systemic reactogenicity
  - Assess any other adverse events
- Every hour after IV infusion or SC injection of IP, for at least 6 hours, the study staff will:
  - Record vital signs (pulse, respiratory rate, blood pressure and temperature)
  - Assess any local and systemic reactogenicity
  - Assess any other adverse events
  - Collect PK samples according to the Schedule of Procedures

If a participant has an abnormal laboratory value that is known, at the time of infusion or injection, follow the specified guidelines (Section 12.0).

6.3 Post-IV infusion or SC injection of PGT121 mAb Visits

The participant will be asked to return to the clinic for post-IP administration visits as indicated in the Schedule of Procedures (see Appendices A, B and C) for an assessment by clinic staff. The participant will be asked to maintain a Memory Aid to track any local and systemic reactogenicity the participant experiences, including temperature, from the day of IP administration for the next 3 days (for a total of 4 days including day of IP administration). Study staff will review the Memory Aid with the participant and determine the severity of the reactions through discussion with the participant.

The following procedures will be conducted at these visits:

- Review interim medical history
- Collect concomitant medication information
- Perform a symptom-directed physical examination if any signs or symptoms are present
- Assess vital signs (pulse, respiratory rate, blood pressure and temperature)
- Assess any adverse events and local and systemic reactogenicity (Days 1, 2, 3) including reviewing the Memory Aid.
- Collect specimens for all tests as indicated in the Schedule of Procedures (Appendices A, B and C and AP).

6.4 Additional Follow-up Visits

Assessments and procedures will be performed according to the Schedule of Procedures (Appendices A, B and C).

This copy of the document was retrieved from the system by Lisa Sunner on 23 Oct 2018

6.5 Unscheduled Visits

Unscheduled Visits/Contacts are visits/contacts that are not described in the Schedule of Procedures (Appendices A, B and C). Unscheduled visits may occur any time during the study:

- For administrative reasons, e.g., the participant may have questions for study staff or may need to re-schedule a follow-up visit.
- To obtain laboratory test results from a previous visit.
- For other reasons as requested by the participant or site investigator.

All unscheduled visits will be documented in the participants' study records on applicable source documents and entered into the Case Report Form (CRF).

6.6 Final Study Visit or Early Termination Visit

Assessments and procedures will be performed according to the Schedule of Procedures (Appendices A, B and C).

7.0 STUDY PROCEDURES

7.1 Informed Consent Process

A Master Informed Consent Document consisting of a Participant Information Sheet and a Consent Form is provided by the Sponsor to the trial site. This document is made site-specific and translated (if necessary), submitted and approved by the Institutional Review Board (IRB). The Master and site specific Informed Consent Documents are separate documents and should not be part of the protocol.

Participant Information Sheet

A qualified member of the study staff will conduct the informed consent process by reviewing the Participant Information Sheet and document it in the clinic notes.

Consent Form

The participant's consent to participate must be obtained by him/her signing and dating the Consent Form. The person obtaining consent will also sign.

The signed and dated Informed Consent Document must remain at the study site. A copy of the signed/marked and dated Informed Consent Document will be offered to the participant to take home. Those participants who do not wish to take a copy will be required to document that they declined to do so.

7.2 Medical History and Physical Examination

Medical History

At screening, a comprehensive medical history will be collected including previous IV infusions and SC injections, and reaction to IV infusion or SC injection, history of sexually transmitted infection (STI) and pregnancy prevention practices. At subsequent visits, an interim medical history will be performed.

This copy of the document was retrieved from the system by Lisa Sunner on 23 Oct 2018

Physical Examination

General Physical Examination
A general physical examination includes examination of head/ears/eyes/nose and throat, skin, respiratory, cardiovascular, abdominal, limited neurological and musculoskeletal and external ano-genital systems (for HIV-infected participants only) at the time points indicated in the Schedule of Procedures (see Appendices A, B and C).

Symptom-Directed Physical Examination
A symptom-directed physical examination is a targeted examination based on the participant's history or observation. If deemed necessary, this examination should be done at the time points indicated in the schedule of procedures (see Appendices A, B and C).

Measuring Height and Weight
Includes measuring the height and weight at the time points indicated in the Schedule of Procedures (see Appendices A, B and C).

Vital Signs
Vital signs including pulse, respiratory rate, blood pressure and temperature are measured and recorded at the time points indicated in the Schedule of Procedures (see Appendices A, B and C)

7.3 HIV Testing and HIV-test Counselling (Group 1)

Study staff will perform pre-HIV test counselling prior to collecting blood for an HIV test, and post-HIV test counselling when HIV test results are available. This is referred to as HIV-test counselling, and done according to the CDC guidelines. For more information on HIV testing and HIV-test counselling, see Section 11.0. A screening questionnaire and other tools may be used.

7.4 HIV Risk Reduction Counselling

HIV risk reduction counselling will be provided to all participants as outlined by site-specific SOPs.

Study staff will provide HIV risk reduction counselling based on reported individual risk and provide free condoms, as appropriate, at every visit. Group 1 will receive HIV risk reduction counselling and for Groups 2 and 3, HIV risk reduction counselling will be conducted as secondary prevention to reduce onward transmission.

7.5 Family Planning Counselling

Study staff will counsel participants about the importance of preventing pregnancies and of using condoms, as well as other effective family planning methods until at least 3 months following investigational product administrations, as appropriate. Participants may be referred for family planning services as necessary according to site-specific SOPs as detailed in the SOM. Pregnancy prevention methods chosen and compliance will be documented.

This copy of the document was retrieved from the system by Lisa Sunner on 23 Oct 2018

7.6 ART Counselling (Group 3)

HIV-infected participants who are not on ART will receive ART counselling upon entering the study and 8 weeks after administration of IP. Participants who have not initiated or made plans to initiate ART by the final study visit will receive ART counselling again at their final study visit. HIV-infected participants who are on ART (Group 2) will be counselled on the importance of continuing ART throughout the study, and will not be required to interrupt ART after administration of IP.

7.7 Specimens

Approximately 50 ml of blood will be collected from participants in Group 1, approximately 78 ml from participants in Group 2, and approximately 150 ml of blood will be collected from participants in Group 3 at the screening visit. At later visits, approximately 8.5 ml to 175 ml of blood will be collected, depending on study procedures and group assignment (see Appendices A, B and C), usually from the antecubital fossa.

Optional collection of rectal and/or cervical mucosal secretions will be obtained using a rectal sponge (or comparable swab) or cervical Softcup (or comparable cervical fluid collection cup) for those participants that consent.

All specimens will be handled according to the procedures specified in the AP or SOPs, if applicable.

In the event of an abnormal laboratory value, participants may be asked to have an additional sample collected at the discretion of the Principal Investigator or designee.

7.8 Reimbursement

Participants will be reimbursed for their time, effort and for costs to cover their travel expenses to the study site and any inconvenience caused due to study participation. Site specific-reimbursement amounts will be documented in the site-specific Participant Information Sheet, and approved by the Institutional Review Board.

7.9 Randomization and Blinding

Participants will be identified by a unique study identification number.

Participants will be randomized according to the randomization schedule prepared by the statisticians at the Data Coordinating Center (DCC) prior to the start of the study. Participants will be automatically assigned a specific allocation number as they are enrolled into the data entry system. An unblinding list (Pharmacy List) will be provided to the unblinded site pharmacist by the DCC.

This is a randomized, double-blind placebo-controlled study for Groups 1 and 2, and an open label study for Group 3. For Groups 1 and 2, study staff (investigator and clinical personnel monitoring the safety and laboratory assay results) and participants will be blinded with respect to the allocation of Investigational Product (PGT121 mAb or placebo). A site pharmacist will be unblinded for the purposes of preparing study product.

A participant will be considered enrolled once he/she has been assigned an allocation number.

This copy of the document was retrieved from the system by Lisa Sunner on 23 Oct 2018

Blinded participants will be informed about their assignment (product/placebo) at study completion, once the data is locked. Group 1 and Group 2 can be unblinded separately after the last volunteer in the respective groups completes study participation.
Should a study participant be unblinded during the study, the study participant will be followed up until the end of the study according to the Schedule of Procedures (see Appendices A and B).

7.10 Un-blinding Procedure for Individual Participants

Un-blinding of an individual participant may be indicated in the event of a medical emergency if the clinical management of the participant would be altered by knowledge of the treatment assignment.

The un-blinded information should be restricted to a small group of individuals involved in clinical management/medical treatment of the participant (e.g. treating physician) and the blind must be maintained for those responsible for the study assessments.

The reasons for un-blinding should be documented and the IAVI Chief Medical Officer, the Medical Monitor and the DCC should be notified as soon as possible. The procedures and contact numbers for un-blinding are outlined in the SOM.

7.11 Assessment of IP related HIV sero-positivity

It is possible that PGT121 mAb or an immune response to PGT121 mAb could cause a positive result on a diagnostic HIV antibody test. A Group 1 participant who tests HIV antibody positive at the end of the study will have additional testing to distinguish actual HIV infection from IP-related responses. The participant will be informed of his/her positive HIV antibody test result and offered continuing follow-up until the HIV antibody test becomes negative.

8.0 INVESTIGATIONAL PRODUCT

8.1 Description

A summary of the Investigational Products is shown in Table 8.1-1.

Table 8.1-1 Investigational Products

**IP (Active Product / Placebo)**	**Dosage level**	**Total volume in IP container**	**Total IP (Active Product or placebo) volume to be injected into a 100 mL saline IV bag^, or injected SC‡** (for an 88 kg body weight**)	**Total volume to be Infused IV** (for an 88 kg body weight**)
PGT121 (50 mg/mL)	3 mg/kg	6 mL per vial	5.3 mL	105.3 mL
	10 mg/kg		17.6 mL	117.6 mL
	30 mg/kg		52.8 mL	152.8 mL

This copy of the document was retrieved from the system by Lisa Sunner on 23 Oct 2018

Placebo: 0.9% Sodium Chloride Injection USP (Saline)*	3 mg/kg matching***	NA	5.3 mL***	105.3 mL***
	10 mg/kg matching***		17.6 mL***	117.6 mL***
	30 mg/kg matching***		52.8 mL***	152.8 mL***

* The Placebo provided will be a commercially-available saline partial addition IV bag.

** The actual volume to be injected will be based on the dose group and the weight of the participant at the time of IP administration. The example included here is the average weight of an adult male in the US (88kg) (http://www.cdc.gov/nchs/data/series/sr_11/sr11_252.pdf)

*** For placebo IV infusions: saline from an additional IV bag will be injected into the saline IV bag intended for administration, to match the volume used for a PGT121 mAb injection in the same dose group, to prevent unblinding.

‡ Only 3mg/kg dose will be injected SC, because of volume limitations for SC injections

^ In the case of a saline IV bag shortage, a larger size bag may be substituted as long as additional saline is aspirated out so that 100mL remains in the bag.

8.2 Shipment and Storage

Authorization to ship the Investigational Product to the site will be provided in writing by the Sponsor, upon confirmation that all required critical documents for shipment authorization are completed. The Investigational Product will be shipped maintaining the required storage conditions and stored in a secure location in the clinical site's pharmacy.

PGT121 Investigational Product is formulated in a 20 mM Acetate, 9% Sucrose, 0.008% polysorbate 80, pH 5.2 formulation buffer at a concentration of 50 mg/mL. Each 10 ml vial will contain 6 ml of IP and the PGT121 is stored at <- 20°C. Each vial will be labelled with the name of the product, lot number, concentration, storage temperature, date of manufacturing, manufacturer and a US cautionary statement. Multiple vials will be packaged in a box. Each box will also be labelled with similar information as the vial label, including contact information for the manufacturer.

8.3 Preparation of Investigational Product (IP)

The pharmacist will thaw and inject the appropriate volume of PGT121 into a 0.9% Sodium Chloride for Injection (USP) bag. Detailed instructions for preparing the investigational product are provided in the SOM. The site pharmacist will not be blinded, but the study physician/designee administering the IP will be blinded. Product should be administered within 4 hours of preparation. Example calculations for final volume for IV infusion or SC injection are illustrated in Table 8.1-1. Procedures for handling used and partially used vials will be provided in the SOM. Syringes or other components in direct contact with investigational products will be disposed of in a biohazard container and incinerated or autoclaved.

This copy of the document was retrieved from the system by Lisa Sunner on 23 Oct 2018

8.4 Administration of Investigational Product

Investigational Product will be administered at the enrolment visit. The participant will receive the IP via IV or SC infusion. Participants will receive IV infusion over approximately 60 minutes or SC injection, allowing for clinician discretion. Further information on the IV infusion or SC injection of the IP is supplied in the SOM and study documents.

8.5 Accountability and Disposal of Investigational Product

All used IP vials will be handled according to instructions in the SOM.

During the study, IP accountability forms including receipt and dispensing of vials will be kept and monitored.

At the end of the study, the used and unused IP vials will be handled according to instructions of Sponsor.

Further information on accountability and disposal of IP is supplied in the SOM.

9.0 ASSESSMENTS

9.1 Safety Assessments

Data on local and systemic reactogenicity (i.e., solicited AEs) will be collected by structured interview and medical examination. Data on other adverse events will be collected with open-ended questions. All data will be recorded on the appropriate source documents and entered into the study database. Participants will be given a Memory Aid, which is a tool to assist with collecting reactogenicity data.

Local and systemic reactogenicity events will be assessed by study staff prior to IV infusion or SC injection of IP, at approximately 30 minutes after IP administration start, at 1 hour after IP administration, and subsequently every hour for at least the first 6 hours post-IP administration. Study staff will review the Memory Aid with the participant, and determine the severity of the reactions on days 1-3 through discussion with the participant.

9.1.1 Local reactogenicity

The presence of local reactogenicity will be assessed at the time points specified in the Schedule of Procedures (Appendices A, B and C).
Pain, tenderness, erythema/skin discoloration, swelling/hardening or pruritus will be assessed and graded using Appendix E, Adverse Event Severity Assessment Tables, as a guideline.

9.1.2 Systemic reactogenicity

The presence of systemic reactogenicity will be assessed at the time points specified in the Schedule of Procedures (Appendices A, B and C).
Fever, chills, headache, nausea, vomiting, malaise, myalgia and arthralgia will be assessed and graded using the Appendix E, Adverse Event Severity Assessment Tables as a guideline. For the first 24 hours after IP infusion or injection, any

This copy of the document was retrieved from the system by Lisa Sunner on 23 Oct 2018

infusion related reactions, including cytokine release syndrome, should be graded using the Common Terminology Criteria for Adverse Events (CTCAE) v5.0: November 27, 2017 (Appendix F).

9.1.3 Vital Signs

At the administration of IP visit, vital signs (pulse, respiratory rate, blood pressure and temperature) will be measured by study staff prior to IP administration, at approximately 30 minutes post IP administration start and hourly for at least 6 hours after IV infusion or SC injection. For the other study visits vital signs will be assessed at the time points specified in the Schedule of Procedures (Appendices A, B and C).

9.1.4 Other Adverse Events

Other adverse events (AEs) will be collected through 56 days after IP administration in all participants. Serious Adverse Events (SAEs) will be collected throughout the entire study period. Potential Immune Mediated Diseases (pIMDs), as defined in Section 10.5, will be collected throughout the study period, using the SAE reporting process. Open-ended questions will be asked at time points according to the Schedule of Procedures (Appendices A, B and C). All adverse events will be graded using Appendix E, Adverse Event Severity Assessment Table, as a guideline and will be assessed for causality to the IP. For more information regarding adverse events refer to Section 10.0, Adverse Events.

9.1.5 Concomitant Medications

Concomitant receipt of Investigational Products is prohibited during the study.

Contraceptive use and use of medication at study entry will be documented. (See DCF instructions)

During the study, information regarding concomitant medications and reasons for their use will be solicited from the study participants for 56 days. Ongoing concomitant medications will be recorded until end of study.

9.1.6 Routine laboratory parameters

Table 9.1.6-1 shows the laboratory parameters that will be measured routinely. The samples for these tests will be collected at the time points indicated in the Schedule of Procedures (Appendices A, B and C).

This copy of the document was retrieved from the system by Lisa Sunner on 23 Oct 2018

Table 9.1.6-1: Laboratory Parameters

Laboratory Parameter	Test
Hematology and Coagulation	Hemoglobin, hematocrit, leukocytes, platelets, absolute neutrophil count (ANC), absolute lymphocyte count (ALC), activate partial thromboplastin time (aPTT), international normalized ratio (INR)
Clinical Chemistry	Sodium, potassium, creatinine, aspartate aminotransferase (AST), alanine aminotransferase (ALT), total bilirubin, alkaline phosphatase<br>Groups 1 and 2 only: albumin, creatine kinase, C-reactive protein, C3 complement, C4 complement
Urinalysis	Dipstick test for protein, blood, glucose, ketones, esterase (leukocytes) and nitrite. If clinically significant abnormalities (e.g., blood, protein, leukocytes) are found on dipstick test, then further test(s) will be performed (e.g., microscopy, culture)
T cell panel (Groups 2 and 3)	CD4 T cell count and frequency by single platform flow cytometry

9.1.7 Specific screening tests:

Participants will be screened to exclude the following diseases:

- Hepatitis B: positive for hepatitis B surface antigen (HBsAg) or detectable hepatitis B DNA if on antiretroviral therapy
- Hepatitis C: positive for hepatitis C RNA (HCV antibody test, followed by HCV RNA test if HCV antibody positive)
  Active syphilis: confirmed diagnosis.

A negative Hepatitis B and Hepatitis C result can be documented from the medical record only if the result is from a test administered less than 6 months ago.

9.1.8 Monitoring for anti-PGT121 antibodies:

Participants will be evaluated for the development of antibodies to PGT121 mAb (anti-drug antibodies, ADA) by ELISA according to the Schedule of Procedures (Appendices A, B and C).

9.2 Virologic Assessments

Table 9.2-1 shows the virologic parameters that will be measured routinely. The samples for these tests will be collected at the time points indicated in the Schedule of Procedures (Appendices B and C).

This copy of the document was retrieved from the system by Lisa Sunner on 23 Oct 2018

Table 9.2-1: Virologic Assessment Table

Virologic Parameter	Test
Antiviral Activity	Plasma HIV RNA levels
Anti-reservoir activity	Cell-associated HIV-1 RNA levels in resting CD4 T cells; total HIV-1 DNA and 2-long terminal repeat (LTR) HIV-1 DNA circles in resting or total CD4 T cells; quantitative viral outgrowth assay (qVOA)
Other	Genotyping of plasma HIV RNA for evaluation of PGT121-induced escape mutations; phenotyping of plasma HIV RNA for neutralization susceptibility to PGT121 in-vitro

9.3 Exploratory Immunogenicity Assessments

Humoral immune response assays will include, but are not limited to Env-specific Ab-binding assays, virus neutralization assay, and assays for Ab functionality. Cellular immune response assays will include, but are not limited to IFNγ ELISPOT assay, ICS, and multiparameter flow cytometry. Exploratory assessments on mucosal samples will include, but are not limited to characterization of Env-specific binding Abs. Priority assays are listed below.

9.3.1 Antibody Responses

- Env-specific binding Abs (titers and breadth).
- Env-specific nAbs (titers and breadth).
- Env-specific functional Abs (phagocytosis score and breadth).
- Env-specific binding Ab isotypes (IgA, IgG1-4) (titers and breadth).

9.3.2 Cellular Responses

- IFNγ peripheral blood mononuclear cell (PBMC) responders to peptide pools and subpools of Potential T-cell epitopes, PTE Env/Gag/Pol peptides.
- $CD4^+$ and $CD8^+$ T-cell functionality (% cells producing e.g. IFNγ, IL-2, IL-4, TNFα).
- T-cell development with emphasis on follicular helper T-cells and memory differentiation.

9.3.3 PBMC, Serum and Plasma Storage

Samples of cryopreserved PBMC, plasma and serum will be stored as indicated in the Schedule of Procedures (Appendix A, B and C) and the Analytical Plan (AP) and, if the participant consents, may be used for the purposes of standardization, quality control and for future assays related to HIV prevention or treatment research and development. These samples will be archived and the testing laboratories will be blinded to the participant's identity.

This copy of the document was retrieved from the system by Lisa Sunner on 23 Oct 2018

9.4 Other Assessments

9.4.1 HIV Antibody Testing (Group 1)

All HIV-uninfected participants (Group 1) will be tested for HIV antibodies as indicated in the Schedule of Procedures (Appendix A) or as needed, if medical or social circumstances arise. All participants will receive HIV risk reduction counselling and pre- and post-HIV-test counselling, as specified in Section 7.3 Counselling.

9.4.2 Pharmacokinetics

Blood draws for pharmacokinetics will be done on the day of IP administration immediately before IV infusion or SC injection of IP, at the end of the IP administration, and 30 minutes after the end of IP administration, and 3 hours and 6 hours after the IP administration. An additional draw will be done at 24 hours after IP administration. Thereafter, pharmacokinetic draws will be done as indicated in the Schedule of Procedures (Appendices A, B and C). PGT121 mAb serum or plasma levels will be determined using two methods: a sandwich ELISA using a murine anti-idiotype antibody to PGT121 mAb, and a neutralization assay.

PGT121 mAb pharmacokinetic analysis will be performed using standard non-compartmental analysis methods to estimate elimination half-life (t1/2), clearance (CL/F), volume of distribution (Vz/F), Area under the concentration decay curve (AUC), impact of viral load and/or ART on PGT121 mAb disposition (elimination half-life (t1/2), clearance (CL/F), volume of distribution (Vz/F) and total exposure. PGT 121 accumulation will also be examined in rectal and cervical mucosal secretions collected with rectal sponges or cervical softcups in study participants who specifically consented for these procedures. Descriptive results will be reported for the pharmacokinetic parameters by dose subgroup.

Exploratory analysis using population analysis methods simultaneously combining all pharmacokinetic data across all doses and treatment groups will be performed for quantitative characterization of differences in PGT121 mAb disposition by dose, participant group or disease state.

9.4.3 HLA Typing

Samples for HLA typing will be collected as specified in the Schedule of Procedures (Appendix A, B and C) and AP and may be analyzed as warranted.

9.4.5 Pregnancy Test

A urine pregnancy test for all female participants will be performed by measurement of human chorionic gonadotrophin (βhCG) at time points indicated in the Schedule of Procedures (Appendices A, B and C). The results of the pregnancy test must be negative prior to IV infusion of PGT121 mAb. See section 10.7 for description of pregnancy after administration of IP.

9.4.6 HIV Risk Assessment (Group 1)

Study staff will assess participants for their past and current risk of acquiring HIV at time points indicated in Schedule of Procedures (Appendix A).

This copy of the document was retrieved from the system by Lisa Sunner on 23 Oct 2018

9.4.7 Social Impact Assessment

A brief assessment of the impact of participation in the study will be administered to participants at their final study visit.

10.0 ADVERSE EVENTS

10.1 Definition

An adverse event (AE) is any untoward medical occurrence in a participant administered an Investigational Product and which does not necessarily have a causal relationship with the Investigational Product. An AE can therefore be any unfavourable or unintended sign (including an abnormal laboratory finding), symptom, or disease, temporally associated with the use of Investigational Product whether or not related to the Investigational Product.

Assessment of severity of all AEs, including and seriousness of AEs, is ultimately the responsibility of the Principal Investigator of each site. Refer to the Division of AIDS (DAIDS) Table for Grading the Severity of Adult and Pediatric Adverse Events Version 2.1, July 2017 and the National Cancer Institute Common Terminology Criteria for Adverse Events (CTCAE) v5.0: November 27, 2017 for additional guidance.

10.2 Assessment of Severity of Adverse Events

The following general criteria should be used in assessing adverse events as mild, moderate, severe or very severe at the time of evaluation:

Grade 1 (Mild): Symptoms causing no or minimal interference with usual social & functional activities

Grade 2 (Moderate): Symptoms causing greater than minimal interference with usual social & functional activities

Grade 3 (Severe): Symptoms causing inability to perform usual social & functional activities

Grade 4 (Very Severe): Symptoms causing inability to perform basic self-care functions OR Medical or operative intervention indicated to prevent permanent impairment, persistent disability, or death

Guidelines for assessing the severity of specific adverse events and laboratory abnormalities are listed in Appendix E, Adverse Event Severity Assessment Table.

10.3 Relationship to Investigational Product

Assessment of relationship of an AE or SAE to Investigational Product (IP) is the responsibility of the Principal Investigator or designee. All medically indicated and available diagnostic methods (e.g., laboratory, blood smear, culture, X-ray, etc.) should be used to assess the nature and cause of the AE/SAE. Best clinical and scientific judgment should be used to assess relationship of AE/SAEs to the IP and/or other cause.

This copy of the document was retrieved from the system by Lisa Sunner on 23 Oct 2018

The following should be considered:

- Presence/absence of a clear temporal (time) sequence between administration of the IP and the onset of AE/SAE
- Presence/absence of another cause that could more likely explain the AE/SAE (concurrent disease, concomitant medication, environmental or toxic factors)
- Whether or not the AE/SAE follows a known response pattern associated with the IP

The relationship assessment should be reported as one of the following:

Not Related: clearly explained by another cause (concurrent disease, concomitant medication, environmental or toxic factors, etc.).

Unlikely: more likely explained by another cause (concurrent disease, concomitant medication, environmental or toxic factors, etc.).

Possibly: equally likely explained by another cause but the possibility of the IP relationship cannot be ruled out (e.g., reasonably well temporally related and/or follows a known IP response pattern but equally well explained by another cause).

Probably: more likely explained by the IP (e.g., reasonably well temporally related and/or follows a known IP response pattern and less likely explained by another cause).

Definitely: clearly related and most likely explained by the IP.

For the purpose of expedited safety reporting, all possibly, probably or definitely related SAEs are considered IP-related SAEs.

10.4 Serious Adverse Events

An adverse event is reported as a "Serious Adverse Event" if it meets any of the following criteria (as per International Conference on Harmonisation [ICH] Good Clinical Practice [GCP] Guidelines):

- Results in death
- Is life threatening
- Results in persistent or significant disability/incapacity
- Requires in-participant hospitalization or prolongs existing hospitalization
- Is a congenital anomaly/birth defect or spontaneous abortion
- Any other important medical condition that requires medical or surgical intervention to prevent permanent impairment of a body function or structure

Elective surgery for pre-existing condition that did not increase in severity or frequency is not considered an SAE.

Serious Adverse Events (SAEs) should be reported within 24 hours of the site becoming aware of the event, and sent to the Sponsor as described in the SOM.

To discuss IP-related SAEs or any urgent medical questions related to the SAE, the site investigator should contact one of the IAVI Medical Monitors directly (see Contact List in the SOM).

This copy of the document was retrieved from the system by Lisa Sunner on 23 Oct 2018

The IAVI SAE Report Form should be completed with all the available information at the time of reporting and sent to the Sponsor as described in the SOM. The minimum data required in reporting an SAE are the study identification number, date of birth, gender, event description (in as much detail as is known at the time), onset date of event (if available), reason event is classified as serious, reporting source (name of Principal Investigator or designee), and relationship to the IP as assessed by the investigator.

The Principal Investigator or designee is required to prepare a detailed written report with follow up until resolution or until it is judged by the Principal Investigator or designee to have stabilized.

The Principal Investigator or designee must notify the local IRB/IEC of all SAEs as appropriate. In case of IP-related SAEs, the Sponsor will notify responsible regulatory authorities, Safety Monitoring Committee (SMC), and other study sites where the same IP is being tested.

More details on SAE definitions and reporting requirements are provided in the SOM.

Serious Event Prior to Investigational Product Administration

If a serious event occurs in the period between the participant signing the Informed Consent Form and receiving the IV infusion or SC injection of IP, the event will be reported using the SAE form and following the same procedures for SAE reporting, as indicated in Section 10.4. The timing of the event will be indicated by using the relevant checkbox on the SAE form.

10.5 Reporting Potential Immune-Mediated Diseases

Potential immune-mediated diseases (pIMDs) are a subset of AEs that include both clearly autoimmune diseases and also other inflammatory and/or neurologic disorders that may or may not have an autoimmune etiology. These events are of special interest since they could potentially be caused by immune responses to the IP. The investigator/designee should report such adverse events within the same time limits (following confirmation of an AE as a pIMD; see last paragraph of this section below), and using the same CRF pages, as utilized for SAEs. The investigator or his/her designee will evaluate the occurrence of pIMDs at every visit/contact during the study. IAVI will also expect investigators/designee to provide additional information about pIMD events. AEs to be reported and documented as pIMDs include:

Neuroinflammatory disorders: optic neuritis, cranial nerve disorders (including Bell's palsy), multiple sclerosis, demyelinating disease, transverse myelitis, Guillain-Barré syndrome, myasthenia gravis, encephalitis, neuritis.

Musculoskeletal disorders: systemic lupus erythematosus, cutaneous lupus, Sjögren's syndrome, scleroderma, dermatomyositis, polymyositis, myopathy, rheumatoid arthritis and juvenile rheumatoid arthritis, polymyalgia rheumatica or temporal arteritis, reactive arthritis, psoriatic arthropathy, ankylosing spondylitis, undifferentiated spondyloarthropathy.

This copy of the document was retrieved from the system by Lisa Sunner on 23 Oct 2018

Gastrointestinal disorders: Crohn's disease, ulcerative colitis or proctitis, celiac disease.

Metabolic diseases: autoimmune thyroiditis, Grave's or Basedow's disease, Hashimoto thyroiditis, insulin-dependent diabetes mellitus (IDDM), Addison's disease.

Skin disorders: psoriasis, vitiligo, Raynaud's phenomenon, erythema nodosum, autoimmune bullous skin diseases.

Others: autoimmune hemolytic anemia, thrombocytopenia, antiphospholipid syndrome, *vasculitis, pernicious anemia, autoimmune hepatitis, primary biliary cirrhosis, primary sclerosing cholangitis, autoimmune glomerulonephritis, autoimmune uveitis, autoimmune myocarditis/cardiomyopathy, sarcoidosis, Stevens-Johnson syndrome, Behçet's syndrome.

Infusion site reactions: Grade 3 or 4 infusion site reactions lasting more than 2 days.

*Vasculitis: Vasculitis, Diffuse vasculitis, leucocytoclastic vasculitis, polyarteritis nodosa, microscopic polyangiitis, Wegener's granulomatosis, anti-neutrophil cytoplasmic antibody positive vasculitis, Henoch-schönlein purpura, allergic granulomatous angiitis (Churg-Strauss disease), Kawasaki disease, Takayasu's arteritis, temporal arteritis (giant cell arteritis), renal vasculitis.

Medical judgement should be exercised in deciding whether other disorders/diseases have an autoimmune origin and should also be reported as described above, and this judgement is the investigator's prerogative. Whenever sufficient data exist to substantiate any of the diagnoses in the above list, the event must be reported as a pIMD. While the intent of pIMD reporting is to be inclusive, isolated nonspecific symptoms, which might (or might not) represent the above diagnoses, should be captured as AEs but not reported as pIMDs until the diagnosis can be defended.

10.6 Clinical Management of Adverse Events

Adverse events (AEs) will be managed by the clinical study team who will assess, provide first line of care as appropriate and refer to health care and treatment facilities as warranted. If any treatment/medical care is required as a result of the harm caused by the IP or study procedures, this will be provided free of charge.

If a participant has an AE and/or abnormal laboratory value that is known at the time of IV infusion or SC injection of IP, the specifications of Section 12.0 will be followed.

Participants will be followed until the AE resolves or stabilizes or up to the end of the study, whichever comes last. If at the end of the study, an AE (including clinically significant laboratory abnormality) that is considered possibly, probably or definitely related to the IP is unresolved, follow-up will continue until resolution if possible and/or the participant will be referred.

If a participant from Group 3 experiences a significant decrease in CD4 cell count (e.g. – 20% of baseline, or decline to <200 cells/μL) during the course of the trial, CD4+ will be monitored closely until their CD4 count returns to baseline or until the participant initiates ART. Participants whose CD4 cell counts decrease to <200 cells/μL will be promptly informed and will be referred to their primary HIV care provider. Appropriate prophylaxis

This copy of the document was retrieved from the system by Lisa Sunner on 23 Oct 2018

against opportunistic infections will be instituted according to accepted U.S. HIV treatment guidelines.

10.7 Pregnancy

Although not considered an AE, if a female participant becomes pregnant during the study, it is the responsibility of the Principal Investigator or designee to report the pregnancy promptly to IAVI using the designated forms. The participant will be followed for safety until the end of pregnancy or study completion, whichever occurs last. If possible, approximately 2–4 weeks after delivery, the baby will be examined by a physician to assess its health status and the results will be reported to the Sponsor. The baby will be examined again by a Physician around age 1, and the results will be reported to the Sponsor.

Complications of pregnancy that meet criteria for SAEs, specified in Section 10.4 of this Protocol (e.g., hospitalization for eclampsia, spontaneous abortion, etc.) should be reported as SAEs.

10.8 Intercurrent HIV Infection (Group 1)

HIV infection cannot be directly caused by the IP. If a participant acquires HIV through exposure in the community, at any time after the IV infusion or SC injection of IP, the participant should be offered referral to appropriate care and treatment facilities. The participant will continue to be followed in the study for safety assessments.

Intercurrent HIV infection in study participants, although not considered an SAE, must be reported promptly to IAVI using the designated forms. However, medical conditions associated with the HIV infection that meet criteria for being serious specified in the Section 10.4 of this Protocol (e.g., sepsis, *Pneumocystis jiroveci [carinii] pneumonia*, etc.) should be reported as SAEs using the SAE Report Form.

11.0 MANAGEMENT OF HIV ISSUES DURING AND FOLLOWING STUDY

11.1 HIV Testing – Group 1

Group 1 participants will be tested for HIV antibodies as indicated in the Schedule of Procedures (Appendix A) or as needed, if medical or social circumstances arise. All participants will receive HIV risk reduction counselling and pre- and post-HIV-test counselling, as specified in Section 11.3.1, Counselling (Group 1).

It is possible that PGT121 or an immune response to PGT121 mAb could cause a positive result on a diagnostic HIV antibody test. An IP recipient who falsely tests HIV positive with a diagnostic HIV antibody test at the end of the study will be informed of his/her positive test result and offered continuing follow-up until the test becomes negative.

If a participant acquires HIV through exposure in the community, at any time after the administration of IP, the participant will be offered referral to appropriate care and treatment facilities. The participant will continue to be followed in the study for safety assessments.

Should a participant require HIV testing outside of the study for personal reasons, it is recommended that the participant contact the study staff first. HIV testing can be done at the study site and then processed at an independent laboratory as above. Written evidence of HIV status (HIV-infected or HIV-uninfected) will be provided upon request.

11.2 Social Discrimination as a Result of IP-related antibodies

In order to minimize the possibility of social discrimination in participants (if any) who test positive on a diagnostic HIV antibody test due to IP-related antibodies, appropriate diagnostic HIV testing and certification will be provided both during and after the study as needed.

11.3 HIV infection – Group 1

Group 1 participants who are diagnosed with HIV infection at screening or during the study (intercurrent HIV-infection) will be provided the following:

11.3.1 Counselling

The participant will be counselled by the study investigators or designated counsellors. The counselling process will assist the participant with the following issues:

- Psychological and social implications of HIV infection
- Who to inform and what to say
- Implications for sexual partners
- Implications for child-bearing
- Avoidance of transmission to others in future
- Mandatory reporting to the state, in some instances

11.3.2 Referral for Support/Care

Participants will be referred to a participant support center or institution of his/her choice for a full discussion of the clinical aspects of HIV infection. Referral will be made to a designated physician or center

12.0 WITHDRAWAL FROM STUDY

12.1 Deferral of IV infusion or SC injection of IP

An IV infusion or SC injection of IP may be temporarily deferred if the participant is clinically ill at the time of the administration of IP visit and/or presents with fever (≥ 100.4 F) at the time of the administration of IP. A participant must be clinically well and afebrile for a minimum of a 24-hour consecutive period prior to administration of IP.

Any planned or unplanned deferral of infusion or injection of IP will be discussed with the Sponsor. Participants will be deferred from infusion or injection of IP for any of the following reasons:

1. Pregnancy
2. A disease or condition or adverse event that may develop, regardless of relationship to Investigational Product, if the Principal Investigator or designee is of the opinion that administration of IP will jeopardize the safety of the participant

This copy of the document was retrieved from the system by Lisa Sunner on 23 Oct 2018

3. Participant's request to defer infusion or injection

The following events require resolution and/or review of clinical history by the Principal Investigator or designee and consultation with the Medical Monitor, prior to administration of IP:

- Any abnormal laboratory value, as outlined in section 5.7, Exclusion Criteria, Hematology, Chemistry, Urinalysis that is known at the time of infusion or injection and have not resolved. Abnormal results should be confirmed on the original sample and/or repeated at least once to confirm abnormal values.
- Receipt of inactivated/killed/subunit vaccines (non-HIV) or immunoglobulin within the previous 14 days. Receipt of live attenuated vaccines within the previous 60 days.
- Participating in another clinical study of an Investigational Product

12.2 Withdrawal from the Study (Early Termination)

Participants may be withdrawn from the study permanently for the following reasons:

1. Participants may withdraw from the study at any time if they wish, for any reason
2. The Principal Investigator or designee has reason to believe that the participant is not complying with the protocol
3. If the Sponsor decides to terminate or suspend the study

If a participant withdraws or is withdrawn from the study, all termination visit procedures will be performed according to the Schedule of Procedures (Appendices A, B and C) where possible. Every effort will be made to determine and document the reason for withdrawal.

13.0 DATA HANDLING

13.1 Data Collection and Record Keeping at the Study Site

Data Collection: All study data will be collected by the clinical study staff using designated source documents and entered onto the appropriate electronic CRFs (eCRFs). Access to eCRFs will be provided via an electronic data entry system hosted by the Data Coordination Center. All study data must be verifiable to the source documentation. A file will be held for each participant at the clinic(s) containing all the source documents. Source documentation will be available for review to ensure that the collected data are consistent with the CRFs.

All CRFs and laboratory reports will be reviewed by the clinical team, who will ensure that they are accurate and complete.

Source documents and other supporting documents will be kept in a secure location. Standard GCP practices will be followed to ensure accurate, reliable and consistent data collection.

Source documents include but are not limited to:
- Signed Informed Consent Documents
- Progress notes

This copy of the document was retrieved from the system by Lisa Sunner on 23 Oct 2018

- Data collection forms
- Documentation of any existing conditions or past conditions relevant to eligibility
- Printed laboratory results
- Print out of the eClinical generated enrolment confirmation
- All Adverse Events
- Concomitant medications
- Local and systemic reactogenicity events

13.3 Data Entry at the Study Site

The data collected at the site will be recorded onto the eCRFs by the study staff and entered into a database. To provide for real time assessment of safety, data should be entered as soon as reasonably feasible after a visit occurs.

13.4 Data Analysis

The Sponsor, PIs and Product Developers will agree on how data will be analyzed and presented prior to unblinding.

The DCC will conduct the data analysis and will provide interim safety and final study reports for the Sponsor, Principal Investigators, the PSRT and SMC and the regulatory authorities, as appropriate.

This copy of the document was retrieved from the system by Lisa Sunner on 23 Oct 2018

14.0 STATISTICAL CONSIDERATIONS

14.1 Safety and Tolerability Analysis

14.1.1 Sample Size

The sample size for safety and tolerability analysis will be 35-56 participants according to the dose escalation design used to characterize the safety profile of one IV infusion of PGT121 mAb, at one of three dose levels, to HIV-uninfected and HIV-infected individuals (Groups 1 and 2).

14.1.2 Null Hypothesis

As this is an exploratory proof of concept trial and analysis will be descriptive, no formal null hypothesis will be tested.

14.1.3 Statistical Power and Analysis and Dose Escalation Rules

The frequency of moderate or greater local and systemic reactogenicity events will be determined and compared between groups.

The frequency of SAEs judged possibly, probably or related to the IP will be determined.

All AEs will be analyzed and, grouped by seriousness, severity and relationship to the Investigational Product (as judged by the investigator).

For life-threatening adverse events related to Investigational Product: if none of the 12 (max 18) participants receiving Investigational Products experience such reactions, then the 95 % upper confidence bound for the rate of these adverse events in the population is 26.5% (or 18.5% if n=18).

All AEs will be analysed and grouped by seriousness, severity and relationship to the IP (as judged by the investigator).

For life-threatening adverse events related to IP: if none of the 12 (max 18) participants in either Group 1 or Group 2 who receive the IP experience such reactions then the 95% upper confidence bound for the rate of these adverse events in the population is 26.5% (or 18.5% if n=18).

An interim analysis of group data will be carried out according to the study schema (Table 5.3.1) without unblinding the study to investigators or participants. At the end of the study, a full analysis will be prepared.

Based on previous experience with IAVI Phase 1 IP studies, it is expected that the amount of missing, unused or spurious data will be insignificant. Unused and spurious data will be listed separately and excluded from the statistical analysis. Missing data will be excluded from the statistical analysis.

This copy of the document was retrieved from the system by Lisa Sunner on 23 Oct 2018

14.2 Pharmacokinetic Analysis

14.2.1 Sample Size

The sample size for pharmacokinetic analysis will be 4 per dose subgroup, sufficient to provide sufficient information for the planned analyses.

14.2.2 Null Hypothesis

As this is an exploratory proof of concept trial and analysis will be descriptive, no formal null hypothesis will be tested.

14.2.3 Statistical Power and Analysis

Disposition of PGT121 mAb will be evaluated in this study. Based on the PK profile of other human monoclonal antibodies, it is expected that the half-life of PGT121 mAb will be 14 to 21 days. Previously published data indicates that the pharmacokinetics of PGT121 and 3BNC117 are fairly similar across a non-human primate cohort and within the same non-human primate (clearance of 3BNC117 appears to be marginally faster than that for PGT121).

Commonly reported PK parameters will be calculated using standard non-compartmental slope/height/area/moment (SHAM) analysis methods. Summary descriptive results of PK parameters, including AUC, Cmax, T1/2, and clearance results will be reported by dose cohort. Dose normalized plots of PK parameters will be presented. Correlation between PK and reported safety and pharmacodynamic outcomes will also be explored parameters in order to examine exposure-effect relationships.

A more powerful exploratory analysis to quantitatively determine the dose, participant and disease impact on PGT121 mAb pharmacokinetics, and correlate exposure with response, while correctly accounting for variance based on population intrinsic factors such as weight and gender will be performed. Using the proposed population analysis approach we will be able to simultaneously examine the magnitude and the rate of change to PGT121 disposition driven by HIV-1 RNA levels and/or ART, and also examine the magnitude and the rate of decline in log copies/ml of HIV-1 RNA plasma levels from baseline.

The frequency and levels of anti-PGT121 antibodies will be calculated and tabulated.

14.3 Virologic Analysis for Group 3A

14.3.1 Sample Size

The sample size for virologic analysis in Groups 3A will be maximum of 9 participants.

14.3.2 Null Hypothesis

TMF-03562 | 7.0 | Status:Approved | 18 Oct 2018
This copy of the document was retrieved from the system by Lisa Sunner on 23 Oct 2018

As this is an exploratory proof of concept trial and the analysis will be descriptive, no formal null hypothesis will be tested.

14.3.3 Statistical Power and Analysis

The virologic analysis described in this section relates to the sample size of Group 3A of the study design, in which IP is given to HIV-infected participants off ART with plasma HIV RNA levels of 2 x 10^3 – 10^5 copies/ml. This section assumes that Part 1 of the study has successfully demonstrated that there is a safe dose level of the IP such that the study is carried forward into Part 2.

The primary outcome for this analysis is defined as change in log10 viral load between Day 0 (day of infusion) and Day 7.

No placebo participants are enrolled as part of this design.

The actual starting dose will be the MTD as determined by the SMC based on data from Part 1, therefore the starting dose may be 30mg/kg, 10 mg/kg or 3 mg/kg.

Assuming the starting dose is 30 mg/kg, an interim analysis of Group 3A will be performed after all 6 participants have reached 7 days following IP administration. The following scenarios may then occur, depending on the data:

- If the response in the first 6 participants results in an HIV RNA viral load difference-from-baseline significantly greater than -0.9 log10, the IP will be determined to be effective at 30 mg/kg, and enrolment into Group 3A will cease.
- If the mean response in the first 6 participants is a decrease smaller than -0.9 log10 HIV RNA, then an additional 3 participants will be enrolled into Group 3A.

For the analysis of sample size and power, log10 viral load differences from baseline for each participant were simulated from a normal distribution, with a standard deviation of 0.5. This value was chosen by examining a study of the antiretroviral drug raltegravir, which demonstrated a mean estimated standard deviation of the change of baseline of 0.47[18]. This is a conservative estimate, as the variability of viral loads near the lower range might be expected to also be lower.

The statistical test performed will be the Signed-ranktest, which will incorporate the “shift” parameter of -0.9 log10. An evaluation of potential harm (increased viral load) will also be performed with the Signed ranktest; this test will examine the null hypothesis of no change in viral load (a shift of 0.0 log10 following IP administration) against the one-sided alternative hypothesis that the viral load is increased following IP administration. Each efficacy test will be performed at the level $\alpha = 0.05$. Each test for harm will be performed at level $2\alpha = 0.10$, in order to provide additional sensitivity to detect potential harm.

This copy of the document was retrieved from the system by Lisa Sunner on 23 Oct 2018

14.4 Analysis of Antiviral Activity in Subgroup 3D

14.4.1 Sample Size

The sample size for antiviral activity will be 6 participants.

14.4.2 Null Hypothesis

As this is an exploratory proof of concept trial and the analysis will be descriptive in this population, no formal null hypothesis will be tested.

14.4.3 Statistical Power and Analysis

No efficacy endpoints will be tested in Groups 3D as participants are HIV-infected with low viral loads at baseline (10^2 – $2x10^3$ copies/ml). Immunologic and virologic endpoints will be determined as described in Section 4.1.

14.5 Secondary and Exploratory Immunologic and Virologic Analyses

14.5.1 Sample Size

The sample size for secondary and exploratory immunologic and virologic analysis will be 47-71 participants.

14.5.2 Null Hypothesis

No formal hypothesis on immunologic or virologic responses will be tested, with the exception of the change in viral load described in Section 14.3.

14.5.3 Statistical Power and Analysis

Descriptive statistics (actual values and changes from reference) will be calculated for continuous immunologic and virologic parameters at all time points. Graphical representations of changes in parameters will be made as applicable. Differences between groups at a specific time point will be tested for exploratory purposes by a 2-sample t-test if the data appear to be normally distributed. If not, the non-parametric Wilcoxon rank sum test will be used. If portions of the measurements are censored below the assay quantification limit, the Gehan-Wilcoxon test will be employed. All statistical tests will be two-sided and will be considered statistically significant if $p < 0.05$.

Frequency tabulations will be calculated for discrete (qualitative) immunologic and virologic parameters at all time points. Significant differences between groups will be determined by a 2-sided Fisher's exact test.

Interim immunologic and virologic analyses of grouped data may be performed without unblinding the study to investigators or participants.

This copy of the document was retrieved from the system by Lisa Sunner on 23 Oct 2018

15.0 QUALITY CONTROL AND QUALITY ASSURANCE

To ensure the quality and reliability of the data collected and generated and the ethical conduct of this study, a Study Operations Manual (SOM) will be developed. All deviations will be reported and investigated. The SOM describes reporting and deviation documentation requirements and procedures.

Regular monitoring will be performed according to ICH-GCP as indicated in Section 17.5.

An independent audit of the study and study sites may be performed by the Sponsor or designee to establish the status of applicable quality systems. Inspection by regulatory authorities may also occur.

By signing the protocol, the Principal Investigators agree to facilitate study related monitoring, audits, IRB/IEC review and regulatory inspection(s) and direct access to source documents. Such information will be treated as strictly confidential and under no circumstances be made publicly available.

16.0 DATA AND BIOLOGICAL MATERIAL

All data and biological material collected through the study shall be managed in accordance with the Clinical Trial Agreement (CTA). Distribution and use of these data will be conducted by agreement of all parties.

The computerized raw data generated will be held by the DCC on behalf of the Sponsor. The study sites will also hold the final data files and tables generated for the purpose of analysis.

17.0 ADMINISTRATIVE STRUCTURE

The Principal Investigator will be responsible for all aspects of the study at the study site.

17.1 Protocol Safety Review Team

A PSRT will be formed to monitor the clinical safety data. During the administration of IP phase of the trial, the PSRT will review the clinical safety data on a weekly basis via electronic distribution of reports. An ad hoc PSRT review meeting will occur if any of the members of the PSRT requests a special review to discuss a specific safety issue or as specified in the Study Operations Manual. After the administration of IP phase the PSRT will review the clinical safety data at least monthly.

The PSRT will consist of the IAVI Medical Monitor(s), and the PI or designee from each clinical team. The study chair or an IAVI Medical Monitor may be the PSRT chair. *Ex officio* members will include the IAVI Chief Medical Officer and an unblinded IAVI Medical Monitor.

Additional PSRT participants may include the following, as needed:

- Co-investigators and trial site senior clinical research nursing staff
- Laboratory directors
- Data management, study statistician and regulatory staff

The PSRT membership and procedures are detailed in the PSRT charter.

This copy of the document was retrieved from the system by Lisa Sunner on 23 Oct 2018

17.2 Safety Monitoring Committee (SMC)

The SMC will consist of independent clinicians/scientists/statisticians/ethicists who are not involved in the study. Investigators responsible for the clinical care of participants or representative of the Sponsor may not be a member of the SMC. Details of membership, chair and co-chair and responsibilities are outlined in the SMC charter.

Principal Investigator(s) or designee and/or a Sponsor representative may be asked to join an open session of the SMC meeting to provide information on study conduct, present data or to respond to questions.

Safety data will be reviewed by the SMC at pre-specified time points and at an ad-hoc basis.

17.2.1 Content of Interim Safety Review

The SMC will be asked to review the following blinded data:

- Summary of reactogenicity (i.e., solicited adverse events)
- All adverse events judged by the Principal Investigator or designee to be possibly, probably or definitely related to IP
- All laboratory results confirmed on retest and judged by the Principal Investigator or designee to be clinically significant
- All SAEs

An unblinded presentation of all above noted events may also be made available for the SMC for their review if required by any member of the SMC.

17.2.2 SMC Review of Group 1 and 2 data prior to starting Group 3

Following IV infusion of IP of the last participant in either Group 1C or 2C, the Safety Monitoring Committee (SMC) will review safety data through the day 14 post-IV infusion visit for all participants to confirm MTD in each group, and determine whether, and at what dose level, Group 3 can initiate enrolment. The SMC can meet to confirm MTD when either Group 1C or Group 2C has finished enrolment, and does not need to wait for both groups to be completed to approve initiation of Group 3 enrolment. Data from Group 1D (SC administration) is not considered by SMC for initiation of Group 3 enrolment.

17.3 Criteria for Pausing the Study

Enrolment and administration of IP will be stopped and a safety review conducted by the SMC for any of the following criteria:

1. One or more participants experience an SAE that is judged possibly, probably or definitely related to IP.
2. There is a participant death, regardless of relationship to the IP.
3. Two or more participants experience Grade 3 adverse events in the same category System Organ Class that are considered to be possibly, probably or definitely related to IP or
4. Any grade 4 adverse event that is considered to be possibly, probably or definitely related to IP.

Table 17.3-1: AE notification and safety pause/AE review rules

This copy of the document was retrieved from the system by Lisa Sunner on 23 Oct 2018

Event and relationship to study product	Severity	Occurrence	Site PI action	PSRT or SMC action
SAE, possibly, probably or definitely related	Any	Any	Phone, email or fax forms to sponsor within 24 hours	Study pause within 24 hours, refer to SMC for review
SAE, probably not or not related	Death	Any	Phone, email or fax forms to sponsor within 24 hours	Study pause within 24 hours, refer to SMC for review
AE, possibly, probably or definitely related	Grade 4	Any	Phone, email or fax notification to sponsor within 24 hours	Study pause within 24 hours, refer to SMC for review
AE¶, possibly, probably or definitely related	Grade 3*	First	Phone, email or fax notification to sponsor within 24 hours	PSRT review within 2 business days to consider pause
AE¶, possibly, probably or definitely related	Grade 3*	Second‡	Phone, email or fax notification to sponsor within 24 hours	Study pause within 24 hours, refer to SMC for review

¶Does not include the following reactogenicity symptoms (fever, malaise, myalgia, arthralgia, chills, headache, nausea, vomiting).

*If no evidence of disease is present other than an abnormal laboratory value, the test must be repeated with a new blood sample at least one time within 72 hours after the investigator becoming aware of the abnormal laboratory value. When signs and symptoms are present, repeat test will not be needed.

‡PSRT will determine whether the reported related AE (Grade 3) is a second occurrence of a previously reported AE (Grade 3).

The Sponsor will request a review by the SMC, (or the SMC chair if other SMC members cannot be convened), to be held within 2 business days of the Sponsor learning of the event. The individual participant(s)/or study may be unblinded at the discretion of the SMC.

Following this review, the SMC will make a recommendation regarding the continuation or suspension of the administration of the IP or the trial and communicate this decision immediately to the Sponsor. The Sponsor then will inform the Principal Investigators without delay.

Additional *ad hoc* review may be specifically requested by the Sponsor, the Principal Investigator(s) or by the SMC.

17.4 Study Supervision

The SMC, the IAVI Chief Medical Officer (CMO) and the IAVI Medical Monitor(s) have access to progress report(s) of this study. Close cooperation will be necessary to track study progress, respond to queries about proper study implementation and management, address issues in a timely manner, and assure consistent documentation, and share information effectively. Rates of accrual, retention, and other parameters

relevant to the site's performance will be regularly and closely monitored by the study team.

17.5 Study Monitoring

On-and/or off-site monitoring will ensure that the study is conducted in compliance with human subjects' protection and other research regulations and guidelines, recorded and reported in accordance with the protocol, is consistent with SOPs, GCP, applicable regulatory requirements and locally accepted practices. The monitor will confirm the quality and accuracy of data at the site by validation of CRFs against the source documents, such as clinical records. The investigators, as well as participants through consenting to the study, agree that the monitor may inspect study facilities and source records (e.g., informed consent forms, clinic and laboratory records, other source documents), as well as observe the performance of study procedures (in accordance with site IRB requirements). Such information will be treated as strictly confidential and will under no circumstances be made publicly available.

The monitoring will adhere to GCP guidelines. The Principal Investigator will permit inspection of the facilities and all study-related documentation by authorized representatives of IAVI, and Government and Regulatory Authorities responsible for this study.

17.6 Investigator's Records

Study records include administrative documentation—e.g., reports and correspondence relating to the study—as well as documentation related to each participant screened and/or enrolled in the study—including informed consent forms, case report forms, and all other source documents. The investigator will maintain and store, in a secure manner, complete, accurate, and current study records for a minimum of 2 years after marketing application approval or the study is discontinued and applicable national and local health authorities are notified. IAVI will notify the Principal Investigator of these events.

18.0 INDEMNITY

The Sponsor and Institution are responsible to have appropriate liability insurance. For research-related injuries and/or medical problems determined to result from receiving the IP, treatment including necessary emergency treatment and proper follow-up care will be made available to the participant free of charge at the expense of the Sponsor.

19.0 PUBLICATION

A primary manuscript describing safety, anti-viral effect and immune responses in this trial will be prepared promptly after the data analysis is available.

Authors will be representatives of each trial site, the data management and statistical analysis center, the laboratories, the product developer and the sponsor, participant to the generally accepted criteria of contributions to the design and conduct of the study, the analysis of data and writing of the manuscript. Precedence will be given to authors from the site enrolling the greatest number of participants. Manuscripts will be reviewed by representatives of each participating group as specified in the CTA.

TMF-03562 | 7.0 | Status:Approved | 18 Oct 2018
This copy of the document was retrieved from the system by Lisa Sunner on 23 Oct 2018

20.0 ETHICAL CONSIDERATIONS

The Principal Investigator will ensure that the study is conducted in compliance with the protocol, SOPs in accordance with guidelines formulated by the ICH for GCP in clinical studies, the ethical principles that have their origins in the Declaration of Helsinki and applicable local standards and regulatory requirements.

This copy of the document was retrieved from the system by Lisa Sunner on 23 Oct 2018

APPENDIX A: SCHEDULE OF PROCEDURES – GROUP 1 (A, B, C, D)

Study Month		0							1		2		3	4	5	6
**Study Week**		**0**				**1**	**2**	**3**	**4**	**6**	**8**	**10**	**12**	**16**	**20**	**24**
**Study Day**	**Scr**	**0**	**1**	**2**	**3**	**7**	**14**	**21**	**28**	**42**	**56**	**70**	**84**	**112**	**140**	**168/ET[4]**
**Visit Windows (Days)**	**-56**	**0**	**0**	**0**	**0**	**± 2**	**± 2**	**± 2**	**± 2**	**± 3**	**± 3**	**±3**	**±7**	**±7**	**±7**	**±7**
**INVESTIGATIONAL PRODUCT**																
Investigational Product /Placebo		X														
**CONSENT/ASSESSMENTS/COUNSELLING**																
Informed Consent	X															
Assessment of Understanding	X															
HIV Risk Assessment	X															X
HIV Risk Reduction Counselling	X	X							X		X		X	X	X	X
HIV-test Counselling	X	X							X							X
Family Planning Counselling	X	X														
Social Impact Assessment																X
**CLINICAL SAFETY ASSESSMENTS**																
Comprehensive Medical History	X															
Interim Medical History		X	X	X	X	X	X	X	X	X	X					
Concomitant Medications	X	X	X	X	X	X	X	X	X	X	X					
General Physical Exam	X															X
Directed Physical Exam		X	X	X	X	X	X	X	X	X	X	X	X	X	X	
Weight	X	X														X
Height	X															
Vital Signs	X	X[1]	X	X	X	X	X	X	X	X	X	X	X	X	X	X
Local & Systemic Reactogenicity		X[1]	X	X	X											
Adverse Events		X	X	X	X	X	X	X	X	X	X					
Serious Adverse Events and pIMD	X	X	X	X	X	X	X	X	X	X	X	X	X	X	X	X

CONFIDENTIAL

Study Month		0							1		2		3	4	5	6
**Study Week**		**0**				**1**	**2**	**3**	**4**	**6**	**8**	**10**	**12**	**16**	**20**	**24**
**Study Day**	**Scr**	**0**	**1**	**2**	**3**	**7**	**14**	**21**	**28**	**42**	**56**	**70**	**84**	**112**	**140**	**168/ET[4]**
**Visit Windows (Days)**	**-56**	**0**	**0**	**0**	**0**	**± 2**	**± 2**	**± 2**	**± 2**	**± 3**	**± 3**	**±3**	**±7**	**±7**	**±7**	**±7**
**CLINICAL LABORATORY TESTS**																
Hematology and Coagulation	X	X[6]	X		X	X	X		X		X		X	X	X	X
Clinical Chemistry	X	X[6]	X		X	X	X		X		X		X	X	X	X
Urine Dipstick	X	X[6]	X		X	X	X		X		X		X	X	X	X
Urine Pregnancy test	X	X[6]							X		X		X			X
Active Syphilis	X															
Hepatitis B	X															
Hepatitis C	X															
HIV screen (4th generation Ag/Ab test)	X															
Blinded HIV diagnostic testing[2]		X[6]							X							X
**RESEARCH LABORATORY TESTS**	-	-	-	-	-	-	-	-	-	-	-	-	-	-	-	-
Anti PGT121 Antibodies (ADA)		X[6]							X		X		X			X
Humoral Assays[2]		X[6]			X	X	X		X		X		X			X
Cellular Assays[2]		X[6]					X		X		X		X			X
HLA typing		X[6]														
**PHARMACOKINETICS PGT121 ELISA**		X[3]	X	X	X	X	X	X	X	X	X	X	X	X	X	X
**MUCOSAL SAMPLING**		X[5]	X			X	X									
**PLASMA/SERUM STORAGE**		X	X	X	X	X	X	X	X		X		X			X
**PBMCs STORAGE**		X	X	X	X	X		X			X		X			X

1. At baseline, approximately 30 minutes after IP administration, and at approximately hours 1 through 6 after IV infusion or SC injection. Local and systemic reactogenicity will be assessed by clinic staff at visits on study days 1, 2 and 3. Local and Systemic reactogenicity will also be assessed by the participant using the Memory Aid on study days 1, 2, and 3
2. See Laboratory Analytical Plan for details
3. Day 0 PK draws done immediately before IP administration, at the end of IP administration, 30 minutes after end of IP administration, and 3 hours and 6 hours post IP administration. See SOM for details
4. Early Termination (ET): Procedures to be performed at ET are the same as last visit procedures
5. Cervico-vaginal and/or rectal mucosal sampling (optional) on Day 0 must be done prior to IV infusion or SC injection of IP. See SOM for details
6. Day 0 sample collections for laboratory tests must be done pre-infusion or pre-injection.

This copy of the document was retrieved from the system by Lisa Sunner on 23 Oct 2018

APPENDIX B: SCHEDULE OF PROCEDURES – GROUP 2 (A, B, C)

Study Month		0							1		2		3	4	5	6
Study Week		0				1	2	3	4	6	8	10	12	16	20	24
Study Day	Scr	0	1	2	3	7	14	21	28	42	56	70	84	112	140	168/ET[5]
Visit Windows (Days)	-56	0	0	0	0	± 2	± 2	± 2	± 2	± 3	± 3	±3	±7	±7	±7	±7
**INVESTIGATIONAL PRODUCT**																
Investigational Product /Placebo		X														
**CONSENT/ASSESSMENTS/COUNSELLING**																
Informed Consent	X															
Assessment of Understanding	X															
HIV Risk Reduction Counselling[1]	X	X							X		X		X	X	X	X
Family Planning Counselling	X	X														
Social Impact Assessment																X
**CLINICAL SAFETY ASSESSMENTS**																
Comprehensive Medical History	X															
Interim Medical History		X	X	X	X	X	X	X	X	X	X					
Concomitant Medications	X	X	X	X	X	X	X	X	X	X	X					
General Physical Exam	X															X
Directed Physical Exam		X	X	X	X	X	X	X	X	X	X	X	X	X	X	
Weight	X	X														X
Height	X															
Vital Signs	X	X[2]	X	X	X	X	X	X	X	X	X	X	X	X	X	X
Local & Systemic Reactogenicity		X[2]	X	X	X											
Adverse Events		X	X	X	X	X	X	X	X	X	X					
Serious Adverse Events and pIMD	X	X	X	X	X	X	X	X	X	X	X	X	X	X	X	X
**CLINICAL LABORATORY TESTS**																
Hematology and Coagulation	X	X[8]	X		X	X	X		X		X		X	X	X	X
CD4	X	X[8]				X	X		X		X					X

CONFIDENTIAL

Study Month		0							1		2		3	4	5	6
Study Week		0				1	2	3	4	6	8	10	12	16	20	24
Study Day	Scr	0	1	2	3	7	14	21	28	42	56	70	84	112	140	168/ET[5]
Visit Windows (Days)	-56	0	0	0	0	± 2	± 2	± 2	± 2	± 3	± 3	±3	±7	±7	±7	±7
Clinical Chemistry	X	X[8]	X		X	X	X		X		X		X	X	X	X
Urine Dipstick	X	X[8]	X		X	X	X		X		X		X	X	X	X
Urine Pregnancy test	X	X[8]							X		X		X			X
Active Syphilis	X															
Hepatitis B	X															
Hepatitis C	X															
HIV 4th generation Ag/Ab test	X															
HIV Viral Load	X	X[8]	X	X	X	X	X	X	X	X	X	X	X	X	X	X
**RESEARCH LABORATORY TESTS**	-	-	-	-	-	-	-	-	-	-	-	-	-	-	-	-
Anti PGT121 Antibodies (ADA)		X[8]							X		X		X			X
HIV SGA sequencing[7]	X								X							X
HIV genotypic testing for ART resistance[7]	X								X				X			X
HIV reservoir size assessment	X						X						X			
Humoral Assays[3]		X[8]			X	X	X		X		X		X			X
Cellular Assays[3]		X[8]					X		X		X		X			X
HLA typing		X[8]														
**PHARMACOKINETICS PGT121 ELISA**		X[4]	X	X	X	X	X	X	X	X	X	X	X	X	X	X
**MUCOSAL SAMPLING**		X[6]	X			X	X									
**PLASMA/SERUM STORAGE**	X	X	X	X	X	X	X	X	X		X		X			X
**PBMCs STORAGE**		X	X	X	X	X		X			X		X			X

1. HIV risk reduction counselling as secondary prevention to reduce onward transmission
2. At baseline, approximately 30 minutes after IP administration, and at approximately hours 1 through 6 after IV infusion. Local and systemic reactogenicity will be assessed by clinic staff at visits on study days 1, 2 and 3. Local and Systemic reactogenicity will also be assessed by the participant using the Memory Aid on study days 1, 2, and 3
3. See Laboratory Analytical Plan for details
4. Day 0 PK draws done immediately before IP administration, at the end of IP administration, 30 minutes after end of IP administration, and 3 hours and 6 hours post IP administration. See SOM for details
5. Early Termination (ET): Procedures to be performed at ET are the same as last visit procedures

This copy of the document was retrieved from the system by Lisa Sunner on 23 Oct 2018

6. Cervico-vaginal and/or rectal mucosal sampling (optional) on Day 0 must be done prior to IV infusion of IP. See SOM for details
7. HIV envelope single genome amplification (SGA) sequencing and ART resistance genotyping will be only if they develop detectable plasma viremia >1000 HIV cp/ml during the study.
8. Day 0 sample collections for laboratory tests must be done pre-infusion

TMF-03562 | 7.0 | Status:Approved | 18 Oct 2018
This copy of the document was retrieved from the system by Lisa Sunner on 23 Oct 2018

APPENDIX C: SCHEDULE OF PROCEDURES – GROUP 3 (A, D)

Study Month		0								1		2		3	4	5	6
**Study Week**		**0**				**1**		**2**	**3**	**4**	**6**	**8**	**10**	**12**	**16**	**20**	**24**
**Study Day**	**Scr**	**0**	**1**	**2**	**3**	**7**	**10**	**14**	**21**	**28**	**42**	**56**	**70**	**84**	**112**	**140**	**168/ET[5]**
**Visit Windows (Days)**	**-42**	**0**	**0**	**0**	**0**	**± 2**	**0**	**± 2**	**± 2**	**± 2**	**± 3**	**± 3**	**±3**	**±7**	**±7**	**±7**	**±7**
**INVESTIGATIONAL PRODUCT**																	
Investigational Product /Placebo		X															
**CONSENT/ASSESSMENTS/COUNSELLING**																	
Informed Consent	X																
Assessment of Understanding	X																
HIV Risk Reduction Counselling[1]	X	X								X		X		X	X	X	X
ART counselling	X	X										X					X
Family Planning Counselling	X	X															
Social Impact Assessment																	X
**CLINICAL SAFETY ASSESSMENTS**																	
Comprehensive Medical History	X																
Interim Medical History		X	X	X	X	X	X	X	X	X	X	X					
Concomitant Medications	X	X	X	X	X	X	X	X	X	X	X	X					
General Physical Exam	X																X
Directed Physical Exam		X	X	X	X	X	X	X	X	X	X	X	X	X	X	X	
Weight	X	X															X
Height	X																
Vital Signs	X	X[2]	X	X	X	X	X	X	X	X	X	X	X	X	X	X	X
Local & Systemic Reactogenicity		X[2]	X	X	X												
Adverse Events		X	X	X	X	X	X	X	X	X	X	X					
Serious Adverse Events and pIMD	X	X	X	X	X	X	X	X	X	X	X	X	X	X	X	X	X

Study Month		0								1		2		3	4	5	6
**Study Week**		**0**				**1**		**2**	**3**	**4**	**6**	**8**	**10**	**12**	**16**	**20**	**24**
**Study Day**	**Scr**	**0**	**1**	**2**	**3**	**7**	**10**	**14**	**21**	**28**	**42**	**56**	**70**	**84**	**112**	**140**	**168/ET[5]**
**Visit Windows (Days)**	**-42**	**0**	**0**	**0**	**0**	**± 2**	**0**	**± 2**	**± 2**	**± 2**	**± 3**	**± 3**	**±3**	**±7**	**±7**	**±7**	**±7**
**CLINICAL LABORATORY TESTS**																	
Hematology and Coagulation	X	X[9]	X		X	X		X		X		X		X	X	X	X
CD4	X	X[9]				X		X		X		X					X
Clinical Chemistry	X	X[9]	X		X	X		X		X		X		X	X	X	X
Urine Dipstick[7]	X	X[9]	X		X	X		X		X		X		X	X	X	X
Urine Pregnancy test	X	X[9]								X		X		X			X
Active Syphilis	X																
Hepatitis B	X																
Hepatitis C	X																
HIV 4th generation Ag/Ab test***	X																
HIV Viral Load	X	X[9]	X	X	X	X	X	X	X	X	X	X	X	X	X	X	X
**RESEARCH LABORATORY TESTS**	-	-	-	-	-	-	-	-	-	-	-	-	-	-	-	-	-
Anti PGT121 Antibodies (ADA)		X[9]								X		X		X			X
PGT121 susceptibility testing	X									X							X
HIV SGA sequencing[8]	X									X							X
HIV genotypic testing for ART resistance[8]	X									X				X			X
HIV reservoir size assessment[1]	X							X						X			
Humoral Assays[3]		X[9]			X	X		X		X		X		X			X
Cellular Assays[3]		X[9]						X		X		X		X			X
HLA typing		X[9]															
**PHARMACOKINETICS PGT121 ELISA**		X[4]	X	X	X	X		X	X	X	X	X	X	X	X	X	X
**MUCOSAL SAMPLING**		X[6]	X			X		X									
**PLASMA/SERUM STORAGE**		X	X	X	X	X	X	X	X	X		X		X			X
**PBMCs STORAGE**		X	X	X	X	X	X		X			X		X			X

1. HIV risk reduction counselling as secondary prevention to reduce onward transmission
2. At baseline, approximately 30 minutes after IP administration, and at approximately hours 1 through 6 after IV infusion. Local and systemic reactogenicity will be assessed by clinic staff at visits on study days 1, 2 and 3. Local and Systemic reactogenicity will also be assessed by the participant using the Memory Aid on study days 1, 2, and 3
3. See Laboratory Analytical Plan for details
4. Day 0 PK draws done immediately before IP administration, at the end of the IP administration, 30 minutes after end of IP administration and 3 hours and 6 hours post IP administration. See SOM for details
5. Early Termination (ET): Procedures to be performed at ET are the same as last visit procedures
6. Cervico-vaginal and/or rectal mucosal sampling (optional) on Day 0 must be done prior to IV infusion of IP. See SOM for details
7. Urinalysis will only be conducted at visits after screening if clinically indicated.
8. HIV envelope single genome amplification (SGA) sequencing and ART resistance genotyping will be only if they develop detectable plasma viremia >1000 HIV cp/ml during the study.
9. Day 0 sample collections for laboratory tests must be done pre-infusion.

*** Confirmed HIV-1 infection (HIV Ab+ or HIV RNA+) by documentation in the medical records or in-clinic HIV testing.

This copy of the document was retrieved from the system by Lisa Sunner on 23 Oct 2018

APPENDIX D: LOW RISK CRITERIA

Low risk will be defined as:

1. **SEXUAL BEHAVIORS**

   In the **last 12 months** did not:

   - Have oral, vaginal or anal intercourse with an HIV-infected partner, or partner who uses injection drugs.
   - Gave or receive money, drugs, gifts, or services in exchange for oral, vaginal or anal sex

   AND

   In the **last 6 months** has abstained from penile/anal or penile/vaginal intercourse

   OR

   In the **last 6 months**:

   - Had 4 or fewer partners of the opposite birth sex for vaginal and/or anal intercourse, OR

Is MSM (person born male with partner(s) born male) who, in the **last 12 months**:

- Had 2 or fewer MSM partners for anal intercourse and had no unprotected anal sex with MSM, OR
- Had unprotected anal intercourse with only 1 MSM partner, within a monogamous relationship lasting at least 12 months (during which neither partner had any other partners). If the monogamous relationship ended, the participant may then have had protected anal intercourse with 1 other MSM partner (total 2 or fewer partners in the last 12 months).

Is a transgender person, regardless of the point on the transition spectrum, having sex with men (born male) and/or other transgender persons, who in the last 12 months:

- Had 2 or fewer partners for anal or vaginal intercourse, and had no unprotected anal or vaginal sex, OR
- Had unprotected anal or vaginal intercourse sex with 1 partner only within a monogamous relationship lasting at least 12 months (during which neither partner had any other partners). If the monogamous relationship ended, may then have had protected anal or vaginal sex with one other partner (total 2 or fewer partners in the last 12 months).

AND

Uses or intends to use condoms in situations which may include penile/anal or penile/vaginal intercourse with new partners of unknown HIV status, occasional partners, partners outside a primary relationship, and/or partners known to have other partners.

This copy of the document was retrieved from the system by Lisa Sunner on 23 Oct 2018

2. **NON-SEXUAL BEHAVIORS**

In the **last 12 months** did not:

- Inject drugs or other substances without a prescription
- Use cocaine, methamphetamine, or excessive alcohol, which in the investigator's judgement, rendered the participant at greater than low risk for acquiring HIV infection

The investigator's judgement should consider local epidemiologic information about HIV prevalence in the area and community networks.

A participant is NOT appropriate for inclusion if he/she:

Acquired an STI (ie, new infection) in the last 12 months:

- Syphilis
- Gonorrhea
- Non-gonococcal urethritis
- HSV-2
- Chlamydia
- Pelvic inflammatory disease (PID)
- Trichomonas
- Mucopurulent cervicitis
- Epididymitis
- Proctitis
- Lymphogranuloma venereum
- Chancroid
- Hepatitis B

TMF-03562 | 7.0 | Status:Approved | 18 Oct 2018
This copy of the document was retrieved from the system by Lisa Sunner on 23 Oct 2018

APPENDIX E: ADVERSE EVENT SEVERITY ASSESSMENT TABLE

Please reference the appropriate Division of AIDS (DAIDS) Table for Grading and Severity of Adult and Pediatric Events Version 2.1, July 2017

https://rsc.niaid.nih.gov/sites/default/files/daidsgradingcorrectedv21.pdf

APPENDIX F CTCAE TABLE

Please reference the appropriate Common Terminology Criteria for Adverse Events (CTCAE) v5.0: November 27, 2017

CTCAE 5.0 Relevant For T001
Common Terminology Criteria for Adverse Events (CTCAE)
Version 5.0 Published: November 27, 2017

U.S. DEPARTMENT OF HEALTH AND HUMAN SERVICES National Institutes of Health National Cancer Institute

Quick Reference

The NCI Common Terminology Criteria for Adverse Events is a descriptive terminology which can be utilized for Adverse Event (AE) reporting. A grading (severity) scale is provided for each AE term.

Components and Organization

SOC

System Organ Class (SOC), the highest level of the MedDRA1 hierarchy, is identified by anatomical or physiological system, etiology, or purpose (e.g., SOC Investigations for laboratory test results). CTCAE terms are grouped by MedDRA Primary SOCs. Within each SOC, AEs are listed and accompanied by descriptions of severity (Grade).

CTCAE Terms

An Adverse Event (AE) is any unfavorable and unintended sign (including an abnormal laboratory finding), symptom, or disease temporally associated with the use of a medical treatment or procedure that may or may not be considered related to the medical treatment or procedure. An AE is a term that is a unique representation of a specific event used for medical documentation and scientific analyses. Each CTCAE v4.0 term is a MedDRA LLT (Lowest Level Term).

Definitions

A brief definition is provided to clarify the meaning of each AE term.

Grades

Grade refers to the severity of the AE. The CTCAE displays Grades 1 through 5 with unique clinical descriptions of severity for each AE based on this general guideline:

Grade 1	Mild; asymptomatic or mild symptoms; clinical or diagnostic observations only; intervention not indicated.
Grade 2	Moderate; minimal, local or noninvasive intervention indicated; limiting age-appropriate instrumental ADL*.
Grade 3	Severe or medically significant but not immediately life-threatening; hospitalization or prolongation of hospitalization indicated; disabling; limiting self care ADL**.
Grade 4	Life-threatening consequences; urgent intervention indicated.
Grade 5	Death related to AE.

A Semi-colon indicates 'or' within the description of the grade.

A single dash (-) indicates a grade is not available.

Not all Grades are appropriate for all AEs. Therefore, some AEs are listed with fewer than five options for Grade selection.

Grade 5

Grade 5 (Death) is not appropriate for some AEs and therefore is not an option.

Activities of Daily Living (ADL)

*Instrumental ADL refer to preparing meals, shopping for groceries or clothes, using the telephone, managing money, etc.
**Self care ADL refer to bathing, dressing and undressing, feeding self, using the toilet, taking medications, and not bedridden.

† CTCAE v5.0 incorporates certain elements of the MedDRA terminology. For further details on MedDRA refer to the MedDRA MSSO Web site (https://www.meddra.org/).

MedDRA Code	MedDRA SOC	CTCAE Term	Grade 1	Grade 2	Grade 3	Grade 4	Grade 5	Definition	Navigational Note
10001718	Immune system disorders	Allergic reaction	Systemic intervention not indicated	Oral intervention indicated	Bronchospasm; hospitalization indicated for clinical sequelae; intravenous intervention indicated	Life-threatening consequences; urgent intervention indicated	Death	A disorder characterized by an adverse local or general response from exposure to an allergen.	If related to infusion, use Injury, poisoning and procedural complications: Infusion related reaction. Do not report both.
10002218	Immune system disorders	Anaphylaxis	-	-	Symptomatic bronchospasm, with or without urticaria; parenteral intervention indicated; allergy-related edema/angioedema; hypotension	Life-threatening consequences; urgent intervention indicated	Death	A disorder characterized by an acute inflammatory reaction resulting from the release of histamine and histamine-like substances from mast cells, causing a hypersensitivity immune response. Clinically, it presents with breathing difficulty, dizziness, hypotension, cyanosis and loss of consciousness and may lead to death.	

10003239	Musculoskeletal and connective tissue disorders	Arthralgia	Mild pain	Moderate pain; limiting instrumental ADL	Severe pain; limiting self care ADL	-	-	A disorder characterized by a sensation of marked discomfort in a joint.	
10008531	General disorders and administration site conditions	Chills	Mild sensation of cold; shivering; chattering of teeth	Moderate tremor of the entire body; narcotics indicated	Severe or prolonged, not responsive to narcotics	-	-	A disorder characterized by a sensation of cold that often marks a physiologic response to sweating after a fever.	
10052015	Immune system disorders	Cytokine release syndrome	Fever with or without constitutional symptoms	Hypotension responding to fluids; hypoxia responding to <40% O2	Hypotension managed with one pressor; hypoxia requiring ≥ 40% O2	Life-threatening consequences; urgent intervention indicated	Death	A disorder characterized by fever, tachypnea, headache, tachycardia, hypotension, rash, and/or hypoxia caused by the release of cytokines.	Also consider reporting other organ dysfunctions including neurological toxicities such as: Psychiatric disorders: Hallucinations or Confusion; Nervous system disorders: Seizure, Dysphasia, Tremor, or Headache
10013573	Nervous system disorders	Dizziness	Mild unsteadiness or sensation of movement	Moderate unsteadiness or sensation of movement; limiting instrumental ADL	Severe unsteadiness or sensation of movement; limiting self care ADL	-	-	A disorder characterized by a disturbing sensation of lightheadedness, unsteadiness, giddiness, spinning or rocking.	

10013963	Respiratory, thoracic and mediastinal disorders	Dyspnea	Shortness of breath with moderate exertion	Shortness of breath with minimal exertion; limiting instrumental ADL	Shortness of breath at rest; limiting self care ADL	Life-threatening consequences; urgent intervention indicated	Death	A disorder characterized by an uncomfortable sensation of difficulty breathing.	
10015218	Skin and subcutaneous tissue disorders	Erythema multiforme	Target lesions covering <10% BSA and not associated with skin tenderness	Target lesions covering 10 - 30% BSA and associated with skin tenderness	Target lesions covering >30% BSA and associated with oral or genital erosions	Target lesions covering >30% BSA; associated with fluid or electrolyte abnormalities; ICU care or burn unit indicated	Death	A disorder characterized by target lesions (a pink-red ring around a pale center).	
10016558	General disorders and administration site conditions	Fever	38.0 - 39.0 degrees C (100.4 - 102.2 degrees F)	>39.0 - 40.0 degrees C (102.3 - 104.0 degrees F)	>40.0 degrees C (>104.0 degrees F) for <=24 hrs	>40.0 degrees C (>104.0 degrees F) for >24 hrs	Death	A disorder characterized by elevation of the body's temperature above the upper limit of normal.	
10016825	Vascular disorders	Flushing	Asymptomatic; clinical or diagnostic observations only	Moderate symptoms; limiting instrumental ADL	Symptomatic, associated with hypotension and/or tachycardia; limiting self care ADL	-	-	A disorder characterized by episodic reddening of the skin, especially face, neck, or chest.	
10019211	Nervous system disorders	Headache	Mild pain	Moderate pain; limiting instrumental ADL	Severe pain; limiting self care ADL	-	-	A disorder characterized by a sensation of marked discomfort in various parts of the head, not confined to the area of distribution of any nerve.	

10020772	Vascular disorders	Hypertension	**Adult:** Systolic BP 120 - 139 mm Hg or diastolic BP 80 - 89 mm Hg;	**Adult:** Systolic BP 140 - 159 mm Hg or diastolic BP 90 - 99 mm Hg if previously WNL; change in baseline medical intervention indicated; recurrent or persistent (>=24 hrs); symptomatic increase by >20 mm Hg (diastolic) or to >140/90 mm Hg; monotherapy indicated initiated;	**Adult:** Systolic BP >=160 mm Hg or diastolic BP >=100 mm Hg; medical intervention indicated; more than one drug or more intensive therapy than previously used indicated;	**Adult and Pediatric:** Life-threatening consequences (e.g., malignant hypertension, transient or permanent neurologic deficit, hypertensive crisis); urgent intervention indicated	Death	A disorder characterized by a pathological increase in blood pressure.	
10021097	Vascular disorders	Hypotension	Asymptomatic, intervention not indicated	Non-urgent medical intervention indicated	Medical intervention indicated; hospitalization indicated	Life-threatening consequences and urgent intervention indicated	Death	A disorder characterized by a blood pressure that is below the normal expected for an individual in a given environment.	

10051792	Injury, poisoning and procedural complications	Infusion related reaction	Mild transient reaction; infusion interruption not indicated; intervention not indicated	Therapy or infusion interruption indicated but responds promptly to symptomatic treatment (e.g., antihistamines, NSAIDS, narcotics, IV fluids); prophylactic medications indicated for <=24 hrs	Prolonged (e.g., not rapidly responsive to symptomatic medication and/or brief interruption of infusion); recurrence of symptoms following initial improvement; hospitalization indicated for clinical sequelae	Life-threatening consequences; urgent intervention indicated	Death	A disorder characterized by adverse reaction to the infusion of pharmacological or biological substances.	
10064774	General disorders and administration site conditions	Infusion site extravasation	Painless edema	Erythema with associated symptoms (e.g., edema, pain, induration, phlebitis)	Ulceration or necrosis; severe tissue damage; operative intervention indicated	Life-threatening consequences; urgent intervention indicated	Death	A disorder characterized by leakage of the infusion into the surrounding tissue. Signs and symptoms may include induration, erythema, swelling, burning sensation and marked discomfort at the infusion site.	
10022095	General disorders and administration site conditions	Injection site reaction	Tenderness with or without associated symptoms (e.g., warmth, erythema, itching)	Pain; lipodystrophy; edema; phlebitis	Ulceration or necrosis; severe tissue damage; operative intervention indicated	Life-threatening consequences; urgent intervention indicated	Death	A disorder characterized by an intense adverse reaction (usually immunologic) developing at the site of an injection.	

10025482	General disorders and administration site conditions	Malaise	Uneasiness or lack of well being	Uneasiness or lack of well being limiting instrumental ADL	Uneasiness or lack of well being limiting self-care ADL	-	-	A disorder characterized by a feeling of general discomfort or uneasiness, an out-of-sorts feeling.	
10028411	Musculoskeletal and connective tissue disorders	Myalgia	Mild pain	Moderate pain; limiting instrumental ADL	Severe pain; limiting self care ADL	-	-	A disorder characterized by marked discomfort sensation originating from a muscle or group of muscles.	
10028813	Gastrointestinal disorders	Nausea	Loss of appetite without alteration in eating habits	Oral intake decreased without significant weight loss, dehydration or malnutrition	Inadequate oral caloric or fluid intake; tube feeding, TPN, or hospitalization indicated	-	-	A disorder characterized by a queasy sensation and/or the urge to vomit.	
10033371	General disorders and administration site conditions	Pain	Mild pain	Moderate pain; limiting instrumental ADL	Severe pain; limiting self care ADL	-	-	A disorder characterized by the sensation of marked discomfort, distress or agony.	Prior to using this term consider using a specific body part pain term found throughout the CTCAE (over 40 different pain terms).
10033557	Cardiac disorders	Palpitations	Mild symptoms; intervention not indicated	Intervention indicated	-	-	-	A disorder characterized by an unpleasant sensation of irregular and/or forceful beating of the heart.	

10037087	Skin and subcutaneous tissue disorders	Pruritus	Mild or localized; topical intervention indicated	Widespread and intermittent; skin changes from scratching (e.g., edema, papulation, excoriations, lichenification, oozing/crusts); oral intervention indicated; limiting instrumental ADL	Widespread and constant; limiting self care ADL or sleep; systemic corticosteroid or immunosuppressive therapy indicated	-	-	A disorder characterized by an intense itching sensation.	
10037868	Skin and subcutaneous tissue disorders	Rash maculo-papular	Macules/papules covering <10% BSA with or without symptoms (e.g., pruritus, burning, tightness)	Macules/papules covering 10 - 30% BSA with or without symptoms (e.g., pruritus, burning, tightness); limiting instrumental ADL; rash covering > 30% BSA with or without mild symptoms	Macules/papules covering >30% BSA with moderate or severe symptoms; limiting self care ADL	-	-	A disorder characterized by the presence of macules (flat) and papules (elevated). Also known as morbillform rash, it is one of the most common cutaneous adverse events, frequently affecting the upper trunk, spreading centripetally and associated with pruritis.	

This copy of the document was retrieved from the system by Lisa Sunner on 23 Oct 2018

10040400	Immune system disorders	Serum sickness	Asymptomatic; clinical or diagnostic observations only; intervention not indicated	Moderate arthralgia; fever, rash, urticaria, antihistamines indicated	Severe arthralgia or arthritis; extensive rash; steroids or IV fluids indicated	Life-threatening consequences; pressor or ventilatory support indicated	Death	A disorder characterized by a delayed-type hypersensitivity reaction to foreign proteins derived from an animal serum. It occurs approximately six to twenty-one days following the administration of the foreign antigen. Symptoms include fever, arthralgias, myalgias, skin eruptions, lymphadenopathy, chest marked discomfort and dyspnea.	
10051837	Skin and subcutaneous tissue disorders	Skin induration	Mild induration, able to move skin parallel to plane (sliding) and perpendicular to skin (pinching up)	Moderate induration, able to slide skin, unable to pinch skin; limiting instrumental ADL	Severe induration; unable to slide or pinch skin; limiting joint or orifice movement (e.g., mouth, anus); limiting self care ADL	Generalized; associated with signs or symptoms of impaired breathing or feeding	Death	A disorder characterized by an area of hardness in the skin.	
10042241	Respiratory, thoracic and mediastinal disorders	Stridor	-	-	Respiratory distress limiting self care ADL; medical intervention indicated	Life-threatening airway compromise; urgent intervention indicated (e.g., tracheotomy or intubation)	Death	A disorder characterized by a high pitched breathing sound due to laryngeal or upper airway obstruction.	

This copy of the document was retrieved from the system by Lisa Sunner on 23 Oct 2018

10046735	Skin and subcutaneous tissue disorders	Urticaria	Urticarial lesions covering <10% BSA; topical intervention indicated	Urticarial lesions covering 10 - 30% BSA; oral intervention indicated	Urticarial lesions covering >30% BSA; IV intervention indicated	-	-	A disorder characterized by an itchy skin eruption characterized by wheals with pale interiors and well-defined red margins.	
10047700	Gastrointestinal disorders	Vomiting	Intervention not indicated	Outpatient IV hydration; medical intervention indicated	Tube feeding, TPN, or hospitalization indicated	Life-threatening consequences	Death	A disorder characterized by the reflexive act of ejecting the contents of the stomach through the mouth.	

APPENDIX G REFERENCES

1 (UNAIDS)., J. U. N. P. o. H. A. The Gap Report., (UNAIDS, 2014).
2 UNAIDS. AIDS by the numbers 2015. (2015).
3 CDC. CDC. Vital Signs: HIV Diagnosis, Care, and Treatment Among Persons Living with HIV- United States 2011. *MMWR* **4**, 1-6 (2014).
4 Jardine, J. *et al.* Rational HIV immunogen design to target specific germline B cell receptors. *Science* **340**, 711-716, doi:10.1126/science.1234150 (2013).
5 Sok, D. *et al.* Promiscuous glycan site recognition by antibodies to the high-mannose patch of gp120 broadens neutralization of HIV. *Sci Transl Med* **6**, 236ra263, doi:10.1126/scitranslmed.3008104 (2014).
6 Caskey, M. *et al.* Viraemia suppressed in HIV-1-infected humans by broadly neutralizing antibody 3BNC117. *Nature* **522**, 487-491, doi:10.1038/nature14411 (2015).
7 Barouch, D. H. *et al.* Therapeutic efficacy of potent neutralizing HIV-1-specific monoclonal antibodies in SHIV-infected rhesus monkeys. *Nature* **503**, 224-228, doi:10.1038/nature12744 (2013).
8 Hessell, A. J. *et al.* Broadly neutralizing human anti-HIV antibody 2G12 is effective in protection against mucosal SHIV challenge even at low serum neutralizing titers. *PLoS Pathog* **5**, e1000433, doi:10.1371/journal.ppat.1000433 (2009).
9 Hessell, A. J. *et al.* Effective, low-titer antibody protection against low-dose repeated mucosal SHIV challenge in macaques. *Nat Med* **15**, 951-954, doi:10.1038/nm.1974 (2009).
10 Moldt, B. *et al.* Highly potent HIV-specific antibody neutralization in vitro translates into effective protection against mucosal SHIV challenge in vivo. *Proceedings of the National Academy of Sciences of the United States of America* **109**, 18921-18925, doi:10.1073/pnas.1214785109 (2012).
11 Walker, L. M. *et al.* Broad neutralization coverage of HIV by multiple highly potent antibodies. *Nature* **477**, 466-470, doi:10.1038/nature10373 (2011).
12 Haynes, B. F. & McElrath, M. J. Progress in HIV-1 vaccine development. *Curr Opin HIV AIDS* **8**, 326-332, doi:10.1097/COH.0b013e328361d178 (2013).
13 Burton, D. R. & Mascola, J. R. Antibody responses to envelope glycoproteins in HIV-1 infection. *Nat Immunol* **16**, 571-576, doi:10.1038/ni.3158 (2015).
14 Sok, D. *et al.* Recombinant HIV envelope trimer selects for quaternary-dependent antibodies targeting the trimer apex. *Proc Natl Acad Sci U S A* **111**, 17624-17629, doi:10.1073/pnas.1415789111 (2014).
15 Scheid, J. F. *et al.* Sequence and structural convergence of broad and potent HIV antibodies that mimic CD4 binding. *Science* **333**, 1633-1637, doi:10.1126/science.1207227 (2011).
16 Shingai, M. *et al.* Passive transfer of modest titers of potent and broadly neutralizing anti-HIV monoclonal antibodies block SHIV infection in macaques. *J Exp Med* **211**, 2061-2074, doi:10.1084/jem.20132494 (2014).
17 Lynch, R. M. *et al.* Virologic effects of broadly neutralizing antibody VRC01 administration during chronic HIV-1 infection. *Sci Transl Med* **7**, 319ra206, doi:10.1126/scitranslmed.aad5752 (2015).
18 Andrade, A. *et al.* Three distinct phases of HIV-1 RNA decay in treatment-naive patients receiving raltegravir-based antiretroviral therapy: ACTG A5248. *J Infect Dis* **208**, 884-891, doi:10.1093/infdis/jit272 (2013).

Signature Page for TMF-03562 v7.0

Reason for signing: Approved	Name: Carl Verlinde<br>Role: Medical Affairs Document Review Team<br>Date of signature: 18-Oct-2018 17:05:44 GMT+0000

Reason for signing: Approved	Name: Fran Priddy<br>Role: Chief Medical Officer<br>Date of signature: 18-Oct-2018 17:43:20 GMT+0000

Signature Page for TMF-03562 v7.0

CONFIDENTIAL

**Protocol Title:**	A Phase 1 Randomized Placebo-controlled Clinical Trial of the Safety, Pharmacokinetics and Antiviral Activity of PGT121 Monoclonal Antibody (mAb) in HIV-uninfected and HIV-infected Adults
**Protocol Number:**	IAVI T001
**Regulatory Investigational Product Number**	IND 126807
**ClinicalTrials.gov Registry Number**	NCT02960581
**Phase:**	Phase 1
**Sponsor:**	International AIDS Vaccine Initiative (IAVI)<br>125 Broad Street, 9th Floor<br>New York, New York 10004<br>USA
**Sponsor Status**	Not for-Profit Organization
**Date of Protocol Version:**	23January 2019<br>08.0<br><br>16 October 2018<br>07.0<br><br>14 July 2017<br>06.0<br><br>04 April 2017<br>05.0<br><br>23 November 2016<br>04.0<br><br>17 October 2016<br>03.0<br><br>09 September 2016<br>02.0<br><br>05 August 2016<br>01.0

THE CONFIDENTIAL INFORMATION IN THIS DOCUMENT IS PROVIDED TO YOU AS AN INVESTIGATOR, POTENTIAL INVESTIGATOR, OR CONSULTANT, FOR REVIEW BY YOU, YOUR STAFF, AND APPLICABLE INSTITUTIONAL REVIEW BOARDS (IRBS) AND/OR INDEPENDENT ETHICS COMMITTEES (IECS). IT IS UNDERSTOOD THAT THE INFORMATION WILL NOT BE DISCLOSED TO OTHERS, EXCEPT TO THE EXTENT NECESSARY TO OBTAIN ETHICAL AND REGULATORY APPROVAL FROM THE RESPECTIVE COMMITTEE'S AGENCIES AND INFORMED CONSENT FROM THOSE PERSONS TO WHOM THE INVESTIGATIONAL PRODUCT MAY BE ADMINISTERED.

TMF-03562 | 9.0 | Status:Approved | 31 Jan 2019
This copy of the document was retrieved from the system by Krysten Brown on 05 Feb 2019

SYNOPSIS

**TITLE**	A Phase 1 Randomized Placebo-controlled Clinical Trial of the Safety, Pharmacokinetics and Antiviral Activity of PGT121 Monoclonal Antibody (mAb) in HIV-uninfected and HIV-infected Adults
**PROTOCOL NUMBER**	IAVI T001
**PHASE**	Phase 1
**SPONSOR**	International AIDS Vaccine Initiative (IAVI)<br>125 Broad Street, 9th Floor<br>New York, New York 10004, USA
**SPONSOR STATUS**	Not for Profit Organization
**STUDY PRODUCTS**	PGT121 monoclonal antibody (mAb)
**OBJECTIVES**	**Primary Objectives**<br>• To evaluate the safety and tolerability of a single intravenous (IV) infusion of PGT121 mAb at 3, 10, and 30 mg/kg in HIV-uninfected adults and HIV-infected adults<br>• To evaluate the safety and tolerability of a single subcutaneous (SC) injection of PGT121 mAb at 3 mg/kg in HIV-uninfected adults<br>• To evaluate the pharmacokinetic (PK) profile of IV infusion and SC injection of PGT121 mAb in HIV-uninfected and HIV-infected adults<br>• To evaluate the antiviral activity of IV infusion of PGT121 mAb in HIV-infected adults not on ART<br><br>**Secondary Objective**<br>• To determine if PGT121 induces anti-PGT121 antibodies<br>• To determine the effect of PGT121 mAb on CD4+ T cell counts in HIV-infected adults<br>• To determine if PGT121 mAb induces viral escape mutants in viremic HIV-infected adults not on ART<br><br>**Exploratory Objectives**<br>• To determine if PGT121 mAb has any impact on the host immune responses (i.e., HIV-specific cellular and humoral immune response)<br>• To determine the effect of PGT121 mAb on the size of the latent HIV reservoir in HIV-infected adults<br>• To determine PGT121 mAb levels in mucosal secretions in HIV-uninfected and HIV-infected adults<br>• To measure in vitro neutralization of HIV isolates with participant's serum post PGT121 mAb IV infusion<br>• To determine if PGT121 mAb has any impact on resistance mutations to ARVs<br>• To explore durability of virological responses following PGT121 mAb infusion in HIV-infected adults (group 3D) in the optional Long-Term Extension (LTE) phase.

This copy of the document was retrieved from the system by Krysten Brown on 05 Feb 2019

**ENDPOINTS**	***Primary***:<br><br>*Safety and Tolerability:*<br>We will measure the following endpoints to evaluate the safety and tolerability of PGT121 mAb in HIV-uninfected and HIV-infected adults:<br><br>1. Proportion of participants with moderate or greater reactogenicity (e.g., solicited adverse events) for 3 days following IV infusion or SC injection of PGT121 mAb.<br>2. Proportion of participants with moderate or greater and/or PGT121 mAb-related unsolicited adverse events (AEs), including safety laboratory (biochemical, hematological) parameters, following IV infusion or SC injection of PGT121 mAb for the first 56 days post administration of Investigational Product.<br>3. Proportion of participants with PGT121 mAb-related serious adverse events (SAEs) throughout the study period.<br><br>*Pharmacokinetics:*<br>We will measure the following endpoints to determine the pharmacokinetics following IV infusion or SC injection of PGT121 mAb in HIV-uninfected and HIV-infected adults:<br><br>1. Elimination half-life (t1/2)<br>2. Clearance (CL/F)<br>3. Volume of distribution (Vz/F)<br>4. Area under the concentration decay curve (AUC)<br>5. Impact of viral load and/or ART on PGT121 disposition (elimination half-life (t1/2), clearance (CL/F), volume of distribution (Vz/F), total exposure<br><br>*Antiviral activity:*<br>We will calculate the following endpoints to determine the antiviral activity following IV infusion of PGT121 mAb in viremic HIV-infected adults not on ART:<br><br>1. Change in plasma HIV-1 RNA levels from baseline (mean of pre-entry and entry values)<br><br>***Secondary***:<br><br>*Anti-PGT121 antibodies:*<br>We will calculate the following endpoint to determine if anti-PGT121 antibodies are elicited following IV infusion or SC injection of PGT121 mAb in HIV-uninfected and HIV-infected adults:<br><br>1. Change in serum anti-PGT121 antibody titers from baseline<br><br>*CD4+ T cell count:*<br>We will calculate the following endpoint to determine if IV infusion of

This copy of the document was retrieved from the system by Krysten Brown on 05 Feb 2019

PGT121 mAb has any impact on CD4+ T cell counts in HIV-infected adults:

1. Change in CD4+ T cell count and frequency compared to baseline as measured by single platform flow cytometry

HIV genotyping/phenotyping of circulating virus for evaluation of PGT121 mAb -induced escape mutations:
We will compare plasma virus sequences before and after IV infusion of PGT121 mAb to determine if PGT121 mAb induces viral escape mutations in viremic HIV-infected adults not on ART.

1. Development of sequence variations in epitopes known to result in reduced PGT121 mAb neutralization susceptibility.

Exploratory:

Additional assessments may include, but are not limited to, the following: HIV-specific IgG/IgA binding responses by ELISA, HIV-specific cellular immune responses by ELISPOT, HIV-specific antibody function by ADCC, ADCP, and ADCVI assays, resistance mutations to current ARVs, PGT121 mAb levels in mucosal secretions, changes in total HIV-1 DNA and 2-long terminal repeat (LTR) circular HIV-1 DNA in resting or total CD4 T cells and in vitro neutralization of HIV isolates with participant's serum post administration of PGT121 mAb. Available samples from time points during the optional LTE phase for group 3D will also be used for determination of long-term durability of the immune responses.

This copy of the document was retrieved from the system by Krysten Brown on 05 Feb 2019

CONFIDENTIAL

STUDY DESIGN TABLE

	Group	Participants	Sub-Group	Regimen	N	Dose (mg/kg) - administration
Part 1(1)	1(3)	HIV-uninfected participants	1A	PGT121/Placebo	4/1 (6/2 if DLT(2))	3 IV
			1B	PGT121/Placebo	4/1 (6/2 if DLT)	10 IV
			1C	PGT121/Placebo	4/1 (6/2 if DLT)	30 IV
			1D	PGT121/Placebo	4/1 (6/2 if DLT)	3 SC
	2(3)	HIV-infected on ART, (<50 cp/ml)	2A	PGT121/Placebo	4/1 (6/2 if DLT(2))	3 IV
			2B	PGT121/Placebo	4/1 (6/2 if DLT)	10 IV
			2C	PGT121/Placebo	4/1 (6/2 if DLT)	30 IV
Safety Monitoring Committee Review(4)						
Part 2	3(5)	HIV-infected off ART (VL $2x10^3$ – $1x10^5$cp/ml)	3A	PGT121	6 (max 9)	30 IV
		HIV-Infected off ART (VL $1x10^2$ – $2x10^3$ cp/ml)	3D6	PGT121	6	30 IV

DLT, dose limiting toxicity; ART, antiretroviral therapy; cp, copies; ml, milliliter
Administration of PGT 121 will be by intravenous infusion (IV) or subcutaneous injection (SC)

1. Eligible participants for Groups 1 and 2 will be enrolled according to their HIV-serostatus and will occur in parallel. At each dose level in Part 1, investigational product (IP) administration will be separated by at least 24 hours for each of the first 3 participants. Randomization will ensure at least 2 participants receive active product and are observed for at least 24 hours before administration to additional participants.
2. A dose limiting toxicity (DLT) will be defined as 1) any Grade 3 or greater reactogenicity or adverse event judged by the study investigators as at least possibly related to investigational product or 2) any SAE considered at least possibly related to investigational product.
3. The PSRT will review safety data to determine dose escalation. If no DLT occurs within 2 weeks from infusion of the first 5 participants in a dose subgroup (e.g. 1A), dose escalation to the next dose subgroup will proceed (e.g. 1B). If 1 DLT occurs in a dose subgroup (e.g. 1A), 3 additional participants will be enrolled into that dose subgroup; these 3 participants will be randomized between PGT121 mAb and placebo at a 2:1 ratio. If no additional DLTs occur in that subgroup (e.g. 1A) within 2 weeks of infusion in the 8 total participants (3:1 ratio of PGT121 mAb to placebo), the study can proceed with enrolment of the next dose subgroup (e.g. 1B). If 2 or more DLTs accumulate in a dose subgroup (e.g. 1A) that are the same, similar, or in the same System Organ Class, dosing will be halted in that subgroup (e.g. 1A) and the next lower dose level will be declared the maximum tolerated dose (MTD) for that subgroup (e.g. 1A). When groups are enrolled in parallel, if the MTD is determined in one group (e.g. Group 1) due to the occurrence of 2 or more DLTs in this group, dosing of participants in the parallel group (e.g. Group 2) will be held until the PSRT has reviewed the safety data and determined whether the MTD should be applied to both groups. If no DLT occurs in one of the final dose subgroups after 14 days of follow up, MTD will be the highest dose given (subgroups 1C or 2C 30mg/kg). Dose

This copy of the document was retrieved from the system by Krysten Brown on 05 Feb 2019

	escalation in Groups 1 and 2 may occur in parallel, and can occur independent of each other.<br>4. Following IP administration of IV PGT121 in the last participant in either Group 1 or 2, an independent Safety Monitoring Committee (SMC) will review at least the first 14 days of safety data to confirm MTD, and determine whether, and at what dose, Group 3 can initiate enrolment. The SMC can meet to confirm MTD when either Group 1 or Group 2 has finished enrolment, and does not need to wait for both groups to be completed to approve initiation of Group 3 enrolment. Data from Group 1D (SC administration) is not considered by SMC for initiation of Group 3 enrolment.<br>5. Group 3 will start with the MTD as determined in Part 1.<br>6. An optional LTE phase (approximately 1 year after week 24) will be performed for participants enrolled in Group 3D who have HIV-1 RNA plasma level <50 copies/ml at week 24 and are off cART.
**METHODS**	See Schedule of Procedures, Appendices A, B, C
**STUDY POPULATION**	The study population will include three different groups:<br>Group 1 will include HIV-uninfected males or females aged 18-50 years old who are willing to maintain low risk behavior for HIV infection; principal exclusion criteria include confirmed HIV-infection, pregnancy or lactation, significant acute or chronic disease and clinically significant laboratory abnormalities. Group 2 will include HIV-infected males or females aged 18-65 years old on a stable antiretroviral regimen with HIV-1 RNA plasma level <50 copies/ml, CD4 cell count ≥ 300 cells/uL; principal exclusion criteria include history of AIDS-defining illness within the previous 5 years, significant acute or chronic medical condition other than HIV infection, and clinically significant laboratory abnormalities. Group 3 will include HIV-infected males or females aged 18-65 years old, not on antiretroviral therapy for > 6 month with detectable HIV-1 viral load between 100 and 100,000 copies/ml, CD4 cell count ≥ 300 cells/uL; principal exclusion criteria include history of AIDS-defining illness within the previous 5 years, significant acute or chronic medical condition other than HIV infection, and clinically significant laboratory abnormalities.
**NUMBER OF PARTICIPANTS**	47-71 participants will be included.
**DOSE ESCALATION and PAUSE RULES**	The first part of this study is a dose-escalation trial in HIV-uninfected adults and HIV-infected adults on ART with suppressed viral load, as indicated in the study design table.<br><br>If 2 or more DLTs accumulate in a dose subgroup that are the same, similar, or in the same System Organ Class, dosing will be halted and the next lower dose level will be declared the maximum tolerated dose (MTD) within this group. If no DLT occurs in one of the final dose groups after 14 days of follow-up, MTD will be the highest dose given (subgroups 1C or 2C 30mg/kg).<br><br>The Protocol Safety Review Team (PSRT) will review safety data through at least day 14 post-IP administration for the first 5 participants in each dose subgroup (e.g. 1A) prior to allowing enrolment of participants into the next dose subgroup (e.g. 1B). Dose escalation in Groups 1 and 2 may occur in parallel, and can occur independent of each other.<br><br>Following IP administration of IV PGT121 in the last participant in either Group 1 or 2, an independent Safety Monitoring Committee (SMC) will review safety data through at least day 14 post-IP administration for all

This copy of the document was retrieved from the system by Krysten Brown on 05 Feb 2019

	participants to confirm MTD, and determine whether, and at what dose, Group 3 can initiate enrolment. The SMC can meet to confirm MTD when either Group 1 or Group 2 has finished enrolment, and does not need to wait for both groups to be completed to approve initiation of Group 3 enrolment. Data from Group 1D (SC administration) is not considered by SMC for initiation of Group 3 enrolment.<br><br>The second part of this study is conducted in HIV-infected adults not on ART, as indicated in the study design table.<br><br>The study will be paused for a safety review by the investigators and the independent SMC if 1) 1 or more participants experiences a Serious Adverse Event that is judged possibly, probably or definitely related to the IP, 2) There is a participant death, regardless of relationship to the IP, 3) if 2 or more participants experience grade 3 adverse events in the same System Organ Class that are considered to be at least possibly related to IP or 4) any grade 4 adverse event that is considered to be possibly, probably, or definitely related to IP. See protocol section 17.3.
**FORMULATIONS, VOLUMES AND ROUTES OF ADMINISTRATION**	**PGT121 mAb**: PGT121 mAb is a recombinant, fully human monoclonal antibody (mAb) of the IgG1 isotype that binds to the HIV envelope. The concentration and volume of product in each vial is 50 mg/mL, 6mL in each vial. PGT121 mAb will be given intravenously or subcutaneously in this study.
**DURATION OF STUDY PARTICIPATION**	Participants will be screened up to 56 days (Groups 1 and 2) or 42 days (Group 3) before IP administration and will be followed for 24 weeks. The anticipated study duration for each participant is approximately 6 months from screening through last visit of the main study. For group 3D participants enrolled in the LTE phase of the study will be followed for an additional 12 months. It is anticipated that it will take approximately 6 months to enroll Groups 1 and 2. It is anticipated that it will take approximately 16 months to enroll the entire study. The exact duration depends on the recruitment rate and how many participants will be required per dose group.
**RANDOMIZATION and BLINDING**	This is a randomized, placebo-controlled study for groups 1 and 2, and open label for group 3 who will not receive placebo.
**EVALUATION FOR INTERCURRENT HIV INFECTION:**	Participants in Group 1 (HIV-uninfected) will be tested for HIV according to the Schedule of Procedures. Test results will be interpreted according to a pre-determined diagnostic algorithm. HIV testing at additional time points may be performed upon the request of the participant and Principal Investigator or designee as medical or social circumstances warrant.
**SAFETY MONITORING AND STATISTICAL CONSIDE-RATIONS:**	All clinical trial data collected, identified only by a study identification number, will be entered into the clinical trial database.<br><br>Safety will continually be monitored by the Investigators, the Sponsor's Medical Monitor and a Protocol Safety Review Team (PSRT); detailed pause criteria are pre-defined.<br><br>Safety data will be reviewed by an independent Safety Monitoring Committee (SMC). *Ad hoc* safety review may be specifically requested by the Sponsor, the Principal Investigators, Ethics Committees, Regulatory Authorities, or by the SMC. All clinical and routine laboratory data will be

This copy of the document was retrieved from the system by Krysten Brown on 05 Feb 2019

	included in the safety analysis. At the end of the study, a full analysis will be prepared.

This copy of the document was retrieved from the system by Krysten Brown on 05 Feb 2019

TABLE OF CONTENTS

ABBREVIATIONS **12**

CONTACT INFORMATION **13**

1.0 SIGNATURE PAGE **14**

2.0 INTRODUCTION AND BACKGROUND INFORMATION **15**

2.1 Study Rationale 15
2.2 Experience with PGT121 16

3.0 STUDY OBJECTIVES **16**

3.1 Primary Objectives 16
3.2 Secondary Objectives 16
3.3 Exploratory Objectives: 16

4.0 STUDY ENDPOINTS **17**

4.1 Study Endpoints 17
4.1.1 Primary Endpoints 17
4.1.2 Secondary Endpoints 17
4.1.3 Exploratory Endpoints 18

5.0 STUDY DESIGN **18**

5.1 Definition of Dose Limiting Toxicity and Maximum Tolerated Dose 18
5.2 Dose Escalation – Groups 1 and 2: Determination of Maximum Tolerated Dose 19
5.3 Group 3: Determination of Antiviral Activity 20
5.4 Duration of the Study 22
5.5 Study Population 22
5.6 Inclusion Criteria for the Main Study 23
5.7 Exclusion Criteria for the Main Study 25
5.7 Inclusion and Exclusion Criteria for the Optional Long-Term Extension Phase 28
5.8 Recruitment of Participants 29

6.0 STUDY VISITS **29**

6.1 Screening Period 29
6.2 IV infusion or SC injection of PGT121 mAb Visit 30
6.3 Post-IV infusion or SC injection of PGT121 mAb Visits 31
6.4 Additional Follow-up Visits in the Main Study 31
6.5 Unscheduled Visits 32
6.6 Final Study Visit or Early Termination Visit in the Main Study 32
6.7 Optional Long-Term Extension Phase 32

7.0 STUDY PROCEDURES **32**

7.1 Informed Consent Process 32
7.2 Medical History and Physical Examination 33
7.3 HIV Testing and HIV-test Counselling (Group 1) 33
7.4 HIV Risk Reduction Counselling 33
7.5 Family Planning Counselling 34
7.6 ART Counselling (Group 3) 34
7.7 Specimens 34
7.8 Reimbursement 34
7.9 Randomization and Blinding 35

This copy of the document was retrieved from the system by Krysten Brown on 05 Feb 2019

7.10 Un-blinding Procedure for Individual Participants 35
7.11 Assessment of IP related HIV sero-positivity 35

8.0 INVESTIGATIONAL PRODUCT 36

8.1 Description 36
8.2 Shipment and Storage 36
8.3 Preparation of Investigational Product (IP) 37
8.4 Administration of Investigational Product 37
8.5 Accountability and Disposal of Investigational Product 37

9.0 ASSESSMENTS 37

9.1 Safety Assessments 37
9.1.1 Local reactogenicity 38
9.1.2 Systemic reactogenicity 38
9.1.3 Vital Signs 38
9.1.4 Other Adverse Events 38
9.1.5 Concomitant Medications 38
9.1.6 Routine laboratory parameters 39
9.1.7 Specific screening tests: 39
9.2 Virologic Assessments 40
9.3 Exploratory Immunogenicity Assessments 40
9.3.1 Antibody Responses 40
9.3.2 Cellular Responses 40
9.3.3 PBMC, Serum and Plasma Storage 40
9.4 Other Assessments 41
9.4.1 HIV Antibody Testing (Group 1) 41
9.4.2 Pharmacokinetics 41
9.4.3 HLA Typing 41
9.4.5 Pregnancy Test 41
9.4.6 HIV Risk Assessment (Group 1) 42
9.4.7 Social Impact Assessment 42

10.0 ADVERSE EVENTS 42

10.1 Definition 42
10.2 Assessment of Severity of Adverse Events 42
10.3 Relationship to Investigational Product 42
10.4 Serious Adverse Events 43
10.5 Reporting Potential Immune-Mediated Diseases 44
10.6 Clinical Management of Adverse Events 45
10.7 Pregnancy 46
10.8 Intercurrent HIV Infection (Group 1) 46

11.0 MANAGEMENT OF HIV ISSUES DURING AND FOLLOWING STUDY 46

11.1 HIV Testing – Group 1 46
11.2 Social Discrimination as a Result of IP-related antibodies 47
11.3 HIV infection – Group 1 47
11.3.1 Counselling 47
11.3.2 Referral for Support/Care 47

12.0 WITHDRAWAL FROM STUDY 47

12.1 Deferral of IV infusion or SC injection of IP 47
12.2 Withdrawal from the Study (Early Termination) 48

This copy of the document was retrieved from the system by Krysten Brown on 05 Feb 2019

13.0 DATA HANDLING 48

13.1 Data Collection and Record Keeping at the Study Site 48
13.3 Data Entry at the Study Site 49
13.4 Data Analysis 49

14.0 STATISTICAL CONSIDERATIONS 50

14.1 Safety and Tolerability Analysis 50
14.2 Pharmacokinetic Analysis 51
14.3 Virologic Analysis for Group 3A 51
14.4 Analysis of Antiviral Activity in Subgroup 3D 53
14.5 Secondary and Exploratory Immunologic and Virologic Analyses 53

15.0 QUALITY CONTROL AND QUALITY ASSURANCE 54

16.0 DATA AND BIOLOGICAL MATERIAL 54

17.0 ADMINISTRATIVE STRUCTURE 54

17.1 Protocol Safety Review Team 54
17.2 Safety Monitoring Committee (SMC) 55
17.2.1 Content of Interim Safety Review 55
17.2.2 SMC Review of Group 1 and 2 data prior to starting Group 3 55
17.3 Criteria for Pausing the Study 55
17.4 Study Supervision 56
17.5 Study Monitoring 57
17.6 Investigator's Records 57

18.0 INDEMNITY 57

19.0 PUBLICATION 57

20.0 ETHICAL CONSIDERATIONS 58

APPENDIX A: SCHEDULE OF PROCEDURES – GROUP 1 (A, B, C, D) 59

APPENDIX B: SCHEDULE OF PROCEDURES – GROUP 2 (A, B, C) 61

APPENDIX C.1: SCHEDULE OF PROCEDURES – GROUP 3 (A, D) 64

APPENDIX C.2: SCHEDULE OF PROCEDURES – LONG-TERM EXTENSION PHASE (WEEK 24 ONWARDS) 67

APPENDIX D: LOW RISK CRITERIA 68

APPENDIX E: ADVERSE EVENT SEVERITY ASSESSMENT TABLE 70

APPENDIX F CTCAE TABLE 70

APPENDIX G REFERENCES 81

This copy of the document was retrieved from the system by Krysten Brown on 05 Feb 2019

ABBREVIATIONS

Abbreviation	Term
**AE**	Adverse Event
**AIDS**	Acquired Immunodeficiency Syndrome
**ALT**	Alanine-Aminotransferase
**ART**	Antiretroviral Therapy
**AST**	Aspartate-Aminotransferase
**CFC**	Cytokine Flow Cytometry
**CMI**	Cell Mediated Immunity
**CRF**	Case Report Form
**CTL**	Cytotoxic T Lymphocyte
**DCC**	Data Coordinating Center
**DLT**	Dose Limiting Toxicity
**DNA**	Deoxyribonucleic Acid
**ELISA**	Enzyme Linked Immunosorbent Assay
**GCP**	Good Clinical Practice
**GFR**	Glomerular Filtration Rate
**HIV**	Human Immunodeficiency Virus
**HLA**	Human Leukocyte Antigen
**HSV**	Herpes Simplex Virus
**IAVI**	International AIDS Vaccine Initiative
**ICH**	International Council for Harmonization of Technical Requirements for Pharmaceuticals for Human Use
**IP**	Investigational Product
**IND**	Investigational New Drug Application
**IV**	Intravenous
**Kg**	Kilogram
**LTE**	Long Term Extension phase
**mAb**	Monoclonal Antibody
**mg**	Milligram
**MED**	Minimum Effective Dose
**MTD**	Maximum Tolerated Dose
**NHP**	Non Human Primate
**PCR**	Polymerase Chain Reaction
**PBMC**	Peripheral Blood Mononuclear Cells
**PK**	Pharmacokinetic
**RPR**	Rapid Plasma Reagin
**SAE**	Serious Adverse Event
**SIV**	Simian Immunodeficiency Virus
**SOP**	Standard Operating Procedure
**SOM**	Study Operations Manual
**SMC**	Safety Monitoring Committee
**STD**	Sexually Transmitted Disease
**TPHA**	Treponema Pallidum Hemagglutination

This copy of the document was retrieved from the system by Krysten Brown on 05 Feb 2019

CONTACT INFORMATION

Detailed contact information provided in the Study Operation Manual (SOM)

Sponsor Contact:	
Frances Priddy MD MPH<br>Executive Director and Chief Medical Officer<br>International AIDS Vaccine Initiative<br>125 Broad Street, 9th Floor<br>New York, New York 10004	Phone: +1-212-328-7461<br>Mobile: +1-646-287-8943<br>Fax: +1-608-203-5501<br>E-mail: fpriddy@iavi.org
**Clinical Research Center Contacts:**	
Kathryn Stephenson MD MPH<br>Center for Virology and Vaccine Research<br>Clinical Trials Unit<br>Beth Israel Deaconess Medical Center<br>E / CLS – 1036<br>330 Brookline Avenue<br>Boston, Massachusetts 02215	Phone: +1-617-735-4556<br>Mobile: +1-917-836-9150<br>Fax: +1-617-735-4566<br>E-mail: kstephen@bidmc.harvard.edu

.

This copy of the document was retrieved from the system by Krysten Brown on 05 Feb 2019

1.0 SIGNATURE PAGE

The signatures below constitute the approval of this protocol and the appendices and provide the necessary assurances that this study will be conducted in compliance with the protocol, Good Clinical Practices (GCP) and the applicable regulatory requirement(s).

Principal Investigator:

Signed: ______________________________ Date: ______________

Name (please print): ______________________________

Name of institution (please print): ______________________________

TMF-03562 | 9.0 | Status:Approved | 31 Jan 2019
This copy of the document was retrieved from the system by Krysten Brown on 05 Feb 2019

2.0 INTRODUCTION AND BACKGROUND INFORMATION

More than 78 million people have been infected with HIV and 39 million people have died since the beginning of the AIDS epidemic[1]. In 2014, there were 1.2 million deaths attributable to HIV infection and 2 million newly infected with HIV[2]. One reason that such high rates of AIDS-related deaths continue to occur globally – despite the advent of drugs that are highly effective at suppressing HIV replication – is that only two in five people living with HIV have access to antiretroviral therapy[1]. The other reason for continued AIDS-related mortality is that ART does not cure HIV infection and must be maintained for a lifetime. Even in the United States (US), only 30% of the 1.2 million people living with HIV have suppressed HIV to undetectable levels, despite the fact that most HIV-infected people in the US have access to ART[3] It is clear that antiretroviral therapy is necessary but not sufficient to end the AIDS epidemic, both in the US and globally, and that novel efforts to prevent and treat HIV are critically needed.

2.1 Study Rationale

This is a Phase 1 study to evaluate the safety, tolerability, pharmacokinetics and anti-viral efficacy of the PGT121 monoclonal antibody for HIV prevention and therapy. PGT121 mAb is a recombinant human IgG1 monoclonal antibody that targets a V3 glycan-dependent epitope region of the HIV envelope protein[4,5]. PGT121 mAb was chosen for this study because it is potent, neutralizes a wide array of HIV viruses, and can prevent and treat simian-human immunodeficiency virus (SHIV) in rhesus monkeys.

The recent discovery of multiple potent and broadly neutralizing antibodies (bNAbs) against HIV has led to the re-emergence of the concept that antibodies may be useful for both prevention and therapy. HIV-specific antibodies that target the HIV envelope (Env) can prevent SHIV infection in rhesus monkeys and have shown to reduce HIV RNA levels in humans temporarily[6-10]. Until recently, these antibodies were few in number, targeted a narrow spectrum of HIV strains and Env epitopes, and were not potent enough for practical use. In the last five years, the field has changed dramatically: new developments in high throughput single-cell PCR-amplification and novel soluble Env baits have led to the isolation of new monoclonal antibodies with extraordinary potency and breadth[11]. These bNAbs may be effective for prevention of HIV infection when administered passively[12,13].

PGT121 mAb was selected for development because of the following critical attributes:

- PGT121 mAb is 10 to 100-fold more potent than the previous best-in-class CD4bs antibodies VRC01, VRC07, and 3BNC117 [11,14,15].
- PGT121 mAb affords superior protective efficacy against SHIV acquisition in monkeys compared to VRC01, 3BNC117, and 10-1074 [16] (and unpublished data).
- PGT121 mAb has superior therapeutic efficacy in SHIV-infected monkeys compared to VRC01, 3BNC117, and 10-1074 [7] (and unpublished data).
- PGT121 mAb may have a higher bar to escape in vivo as compared with other V3 glycan and CD4bs antibodies as a result of making multiple glycan contacts [14].
- PGT121 mAb combined with PGDM1400 (a novel bNab targeting the envelope trimer apex) neutralizes 98-99% of global HIV-1 viruses tested and has unparalleled potency with a median IC50 of 0.007 µg/ml[14].

The potency and breadth of PGT121 mAb, both alone and in combination with other bNAbs, raise the possibility that combinations may be effective for HIV prophylaxis at low doses and against global viruses. An antibody that is effective at low doses may eventually be given subcutaneously, which would reduce the cost. It is these features that make PGT121 mAb particularly well-suited for preventing and/or treating HIV in the developing world, where it is critical that a public health intervention be low cost, easy to deliver, and effective in diverse settings.

2.2 Experience with PGT121

There is no previous clinical experience with PGT121 mAb. Several other HIV monoclonal antibodies are currently in clinical development as passive HIV immunoprophylaxis, or as potential therapeutics. Data from phase 1 studies shows acceptable preliminary safety and tolerability profiles for these products, but varying levels of anti-viral effects [6,17]. A comprehensive summary of phase 1 studies of HIV monoclonal antibodies can be found in the Investigator's Brochure.

3.0 STUDY OBJECTIVES

3.1 Primary Objectives

- To evaluate the safety and tolerability of a single intravenous (IV) infusion of PGT121 mAb at 3, 10, and 30 mg/kg in HIV-uninfected adults and HIV-infected adults.
- To evaluate the safety and tolerability of a single subcutaneous (SC) injection of PGT121 mAb at 3 mg/kg in HIV-uninfected adults
- To evaluate the pharmacokinetic (PK) profile of IV infusion and SC injection of PGT121 mAb in HIV-uninfected and HIV-infected adults.
- To evaluate the antiviral activity of IV infusion of PGT121 mAb in HIV-infected adults not on ART.

3.2 Secondary Objectives

- To determine if PGT121 mAb induces anti-PGT121 antibodies.
- To determine the effect of PGT121 mAb on CD4 T-cell counts in HIV-infected adults.
- To determine if PGT121 mAb induces viral escape mutants in viremic HIV-infected adults not on ART.

3.3 Exploratory Objectives:

- To determine if PGT121 mAb has any impact on the host immune responses (i.e., HIV-specific cellular and humoral immune response).
- To determine the effect of PGT121 mAb on the size of the latent HIV reservoir in HIV-infected adults.
- To determine PGT121 mAb levels in mucosal secretions in HIV-uninfected and HIV-infected adults.
- To measure in vitro neutralization of HIV isolates with participant's serum post PGT121 mAb IV infusion.
- To determine if PGT121 mAb has any impact on resistance mutations to ARVs.
- To explore durability of virological responses following PGT121 mAb infusion in HIV-infected adults of group 3D in the optional Long-Term Extension (LTE) phase.

This copy of the document was retrieved from the system by Krysten Brown on 05 Feb 2019

4.0 STUDY ENDPOINTS

4.1 Study Endpoints

4.1.1 Primary Endpoints

Safety and Tolerability:

We will measure the following endpoints to evaluate the safety and tolerability of PGT121 mAb in HIV-uninfected and HIV-infected adults:

1. Proportion of participants with moderate or greater reactogenicity (e.g., solicited adverse events) for 3 days following IV infusion or SC injection of PGT121 mAb.
2. Proportion of participants with moderate or greater and/or PGT121 mAb-related unsolicited adverse events (AEs), including safety laboratory (biochemical, hematological) parameters, following IV infusion or SC injection of PGT121 mAb for the first 56 days post administration of Investigational Product.
3. Proportion of participants with PGT121 mAb-related serious adverse events (SAEs) throughout the study period, including LTE phase.

Pharmacokinetics:

We will measure the following endpoints to determine the pharmacokinetics following IV infusion or SC injection of PGT121 mAb in HIV-uninfected and HIV-infected adults:

1. Elimination half-life (t1/2)
2. Clearance (CL/F)
3. Volume of distribution (Vz/F)
4. Area under the concentration decay curve (AUC)
5. Impact of viral load and/or ART on PGT121 disposition (elimination half-life (t1/2), clearance (CL/F), volume of distribution (Vz/F), total exposure

Antiviral activity:

We will calculate the following endpoints to determine the antiviral activity following IV infusion of PGT121 mAb in viremic HIV-infected adults not on ART.

1. Change in plasma HIV-1 RNA levels from baseline (mean of pre-entry and entry values)

4.1.2 Secondary Endpoints

Anti-PGT121 antibodies:

We will calculate the following endpoint to determine if anti-PGT121 antibodies are elicited following IV infusion or SC injection of PGT121 mAb in HIV-uninfected and HIV-infected adults:

This copy of the document was retrieved from the system by Krysten Brown on 05 Feb 2019

1. Change in serum anti-PGT121 antibody titers from baseline

CD4+ T cell count:

We will calculate the following endpoint to determine if IV infusion of PGT121 mAb has any impact on CD4+ T cell counts in HIV-infected adults:

1. Change in CD4+ T cell count and frequency compared to baseline as measured by single platform flow cytometry

HIV genotyping/phenotyping of circulating virus for evaluation of PGT121-induced escape mutations:

We will compare plasma virus sequences before and after IV infusion of PGT121 mAb to determine if PGT121 mAb induces viral escape mutations in viremic HIV-infected adults not on ART.

1. Development of sequence variations in epitopes known to result in reduced PGT121 neutralization susceptibility

4.1.3 Exploratory Endpoints

Additional assessments may include but are not limited to the following: HIV-specific IgG/IgA binding responses by ELISA, HIV-specific cellular immune responses by ELISPOT, HIV-specific antibody function by ADCC, ADCP, and ADCVI assays, resistance mutations to current ARVs, PGT121 mAb levels in mucosal secretions, changes in total HIV-1 DNA and 2-long terminal repeat (LTR) circular HIV-1 DNA in resting or total CD4 T cells and in vitro neutralization of HIV isolates with participant's serum post administration of PGT121 mAb. Available samples from time points of group 3D during the optional LTE phase will also be used for determination of long-term durability of the immune responses.

5.0 STUDY DESIGN

The study is a randomized, placebo-controlled study for Groups 1 and 2, and open label for Group 3 who will not receive placebo.

5.1 Definition of Dose Limiting Toxicity and Maximum Tolerated Dose

A dose limiting toxicity (DLT) will be defined as 1) any Grade 3 or greater reactogenicity or adverse event judged by the study investigators as at least possibly related to investigational product or 2) any SAE considered at least possibly related to investigational product.
Maximum Tolerated Dose (MTD) will be declared when 2 or more DLTs occur that are the same, similar, or in the same System Organ Class or if no DLT occurs in the final dose subgroup after 14 days of follow-up, MTD will be the highest dose given (groups 1C or 2C 30mg/kg).

This copy of the document was retrieved from the system by Krysten Brown on 05 Feb 2019

5.2 Dose Escalation – Groups 1 and 2: Determination of Maximum Tolerated Dose

In Groups 1 and 2, the administrations of PGT121 mAb escalate by dose as shown below in Table 5.3.1, Study Design (5 participants per dose subgroup, 4:1 ratio of IP to placebo for each dose subgroup).

Sentinel Recipients

Within each dose group (subgroups 1A and 2A, subgroups 1B and 2B, subgroups 1C and 2C, subgroup 1D), the first 3 participants will be separated by at least 24 hours, to allow for observation of Investigational product (IP)-related adverse events. Dose subgroups will be enrolled in parallel, meaning that the 1st participant may be from subgroup 1A, the 2nd from subgroup 2A, the 3rd from subgroup 2A, all with 24 hours in between dosing. Because there is 1 placebo in each dose subgroup and the subgroups are dosed in parallel, the first 3 recipients will be treated as sentinel recipients (randomization will ensure that at least 2 will receive the IP).

- If no reactogenicity and adverse events that are considered to be related to IP (possibly, probably or definitely related) and are graded as severe or worse (Grade 3 or 4 on the DAIDS Toxicity Table or CTCAE table, see section 9.1.2) occur within 24 hours after infusion of the first participant, the second participant may be injected.
- If no events meeting the criteria described above occur within 24 hours after the 3rd participant is infused, then the remainder of participants in that dose group will be infused.
- If events meeting the criteria described above do occur for the first, second, or third participant in a dose group, they will be reviewed by the Safety Monitoring Committee (SMC) to determine whether further infusions may proceed.

Safety information will be reviewed by the Principal Investigator. The outcome of the safety review, and decision whether or not to dose the next participant(s) or contact the SMC will be communicated with the Sponsor.

Dose Escalation and Determination of Maximum Tolerated Dose

Safety data through at least day 14 post-IP administration visit for the first 5 participants in a dose subgroup (e.g. 1A) will be reviewed by the Protocol Safety Review Team (PSRT) prior to allowing enrolment of participants into the next dose subgroup (e.g. 1B). Dose escalation in Groups 1 and 2 may occur in parallel, and can occur independent of each other. Following administration of IP for the last participant in either Group 1C or 2C, an independent Safety Monitoring Committee (SMC) will review safety data to confirm Maximum Tolerated Dose (MTD) and determine whether, and at what dose, Group 3 can initiate enrolment. The SMC can meet to confirm MTD when either Group 1C or Group 2C has finished enrolment, and does not need to wait for both Groups to be completed to approve initiation of Group 3 enrolment. Data from Group 1D (SC administration) is not considered by SMC for initiation of Group 3 enrolment.

- If no DLT occurs within 2 weeks from infusion of the first 5 participants in a dose subgroup (e.g. 1A), dose escalation to the next dose subgroup (e.g. 1B) will proceed.

This copy of the document was retrieved from the system by Krysten Brown on 05 Feb 2019

- If 1 DLT occurs in a dose subgroup (e.g. 1A), 3 additional participants will be enrolled in that dose subgroup; these 3 participants will be randomized between PGT121 mAb and placebo at a 2:1 ratio.
    - If no additional DLTs occur in that dose subgroup (e.g. 1A) within 2 weeks of infusion in the 8 total participants (3:1 ratio of PGT121 mAb to placebo), the study can proceed with enrolment of the next dose subgroup (e.g. 1B).
    - If 2 or more DLTs accumulate in a dose subgroup (e.g. 1A) that are the same, similar, or in the same organ class, dosing will be halted in that subgroup (e.g. 1A) and the next lower dose level will be declared the maximum tolerated dose (MTD) for that subgroup (e.g. 1A).
- When groups are enrolled in parallel, if the MTD is determined in one group (e.g. Group 1) due to the occurrence of 2 or more DLTs in this group, dosing of participants in the parallel group (e.g. Group 2) will be held until the PSRT has reviewed the safety data and determined whether the MTD should be applied to both groups.
- If no DLT occurs in the one of the final dose subgroups, MTD will be the highest dose given (subgroups 1C or 2C 30mg/kg) after 14 days of follow-up.

5.3 Group 3: Determination of Antiviral Activity

Group 3 will initiate enrolment upon approval by the SMC (see section 17.2.2), Group 3 (Part 2) will start with the MTD (i.e. subgroups 3A and 3D if the MTD is 30 mg/kg).

At least 6 but up to 9 participants will be enrolled in subgroup 3A. Six participants will be enrolled in subgroup 3D.

Table 5.3.1 Study Design Table

	Group	Participants	Sub-Group	Regimen	N	Dose (mg/kg) administration
Part 1	1	HIV-uninfected participants	1A	PGT121/Placebo	4/1 (6/2 if DLT)	3 IV
			1B	PGT121/Placebo	4/1 (6/2 if DLT)	10 IV
			1C	PGT121/Placebo	4/1 (6/2 if DLT)	30 IV
			1D	PGT121/Placebo	4/1 (6/2 if DLT)	3 SC
	2	HIV-infected on ART, (<50 cp/ml)	2A	PGT121/Placebo	4/1 (6/2 if DLT)	3 IV
			2B	PGT121/Placebo	4/1 (6/2 if DLT)	10 IV
			2C	PGT121/Placebo	4/1 (6/2 if DLT)	30 IV
Safety Monitoring Committee Review						
Part 2	3	HIV-infected off ART (VL $2x10^3$ – $1x10^5$cp/ml)	3A	PGT121	6 (max 9)	30 IV

TMF-03562 | 9.0 | Status:Approved | 31 Jan 2019
This copy of the document was retrieved from the system by Krysten Brown on 05 Feb 2019

	HIV-infected off ART (VL $1x10^2$ – $2x10^3$ cp/ml)	3D	PGT121	6	30 IV

This copy of the document was retrieved from the system by Krysten Brown on 05 Feb 2019

5.4 Duration of the Study

Participants will be screened up to 56 days (Groups 1 and 2) or 42 days (Group 3) before IP administration of PGT121 mAb and will be followed for 24 weeks. An optional LTE phase (approximately 1 year after week 24) will be performed for participants enrolled in Group 3D who have HIV-1 RNA plasma level <50 copies/ml at week 24 and are off cART.

It will take approximately 16 months to enroll the entire study. The exact duration depends on the recruitment rate and how many participants will be required per dose group (as specified in sections 5.2 and 5.3), as well as enrollment in the Long Term Extension phase of the study.

5.5 Study Population

The study population consists of HIV-uninfected male or female adults (Group 1), HIV-infected male or female adults on ART (Group 2), and HIV-infected males and female adults not on ART (Group 3) who meet the detailed inclusion and exclusion criteria listed below, and who in the opinion of the investigator or designee, understand the study and provide written informed consent.

Approximately 47-71 participants (max 57 PGT121 recipients, 14 placebo recipients) who meet all eligibility criteria will be included in the study. An over-enrolment of up to 5% (up to 5 participants total) will be permitted in the study to facilitate rapid enrolment.

This copy of the document was retrieved from the system by Krysten Brown on 05 Feb 2019

5.6 Inclusion Criteria for the Main Study

Inclusion criteria for all participants:

1. Willing to comply with the requirements of the protocol and available for follow-up for the planned duration of the study;

2. In the opinion of the Principal Investigator or designee and based on Assessment of Informed Consent Understanding results, has understood the information provided and potential impact and/or risks linked to IV infusion or SC injection and participation in the trial; written informed consent will be obtained from the participant before any study-related procedures are performed;

3. All heterosexually active female participants must commit to use an effective method of contraception for 3 months following IP administration, including:
    a. Condoms (male or female) with or without spermicide
    b. Diaphragm or cervical cap with spermicide
    c. Intrauterine device, or contraceptive implant
    d. Hormonal contraception
    e. Successful vasectomy in the male partner (considered successful if a woman reports that a male partner has [1] documentation of azoospermia by microscopy (< 1 year ago), or [2] a vasectomy more than 2 years ago with no resultant pregnancy despite sexual activity post-vasectomy)
    f. Not be of reproductive potential, such as having undergone hysterectomy, bilateral oophorectomy, or tubal ligation, postmenopausal (>45 years of age with amenorrhea for at least 2 years, or any age with amenorrhea for at least 6 months and a serum follicle stimulating hormone [FSH] level >40 IU/L); surgically sterile: no additional contraception required.

   Women, who are not heterosexually active at screening, must agree to utilize an effective method of contraception if they become hetero-sexually active, as outlined above.

4. All sexually active males, regardless of reproductive potential, must be willing to consistently use an effective method of contraception (such as consistent male condoms with male and/or female partners from the day of IP administration until at least 3 months following IP administration to avoid exposure of partners to IP in ejaculate, and to prevent conception with female partners.

5. All female participants must be willing to undergo urine pregnancy tests at time points indicated in the Schedule of Procedures and must test negative prior to IP administration;

6. A female participant must agree not to donate eggs (ova, oocytes) for the purpose of assisted reproduction until 3 months after receiving IP administration. A man must agree not to donate sperm until 3 months after IP administration;

This copy of the document was retrieved from the system by Krysten Brown on 05 Feb 2019

7. Willing to forgo donations of blood and/or any other tissues, including bone marrow, during the study and, for those HIV-uninfected participants who test HIV-positive due to IP administration, until the anti-HIV antibody titers become undetectable.

Specific inclusion criteria for HIV-uninfected participants (Group 1):

8. At least 18 years of age on the day of screening and has not reached his or her 51st birthday on the day of signing the Informed Consent Document.

9. Willing to undergo HIV testing, risk reduction counselling and receive HIV test results;

10. Low risk for HIV infection (see section (9.4.6) and willing to maintain low-risk behaviour for the duration of the trial (Appendix D);

11. Healthy male or female, as assessed by a medical history, physical exam, and laboratory tests;

Specific inclusion criteria for HIV-infected participants (Groups 2 and 3):

12. At least 18 years of age on the day of screening and has not reached his or her 66th birthday on the day of signing the Informed Consent Document.

13. Confirmed HIV-1 infection (HIV Ab+ or HIV RNA+) by documentation in the medical records or in-clinic HIV testing;

14. CD4 ≥ 300 cells/µl;

15. No history of AIDS-defining illness within the previous 5 years

Group 2:

16. Currently on ART with no change in ART regimen in the 12 weeks before screening or between screening and enrolment, with suppression of plasma HIV-1 viral load < 50 copies / ml for greater than 6 months, and with a viral load < 50 copies / ml at time of screening (within 42 days prior to IP administration). cART is defined as a regimen including > 2 compounds, e.g. 2x nucleoside reverse transcriptase inhibitors plus either non-nucleoside reverse transcriptase inhibitor or protease inhibitor or integrase inhibitor. A change from tenofovir disoproxil fumarate to tenofovir alafenamide in the 12 weeks before screening is not an exclusion.

Group 3:

17. Not receiving cART, and (after appropriate counselling) willing to defer cART treatment for at least 56 days after administration of IP;

18. HIV-1 viral load either between 2000-100,000 copies / ml (Group 3A) or between 100-2000 copies / ml (Group 3D) confirmed at screening.

This copy of the document was retrieved from the system by Krysten Brown on 05 Feb 2019

5.7 Exclusion Criteria for the Main Study

Exclusion criteria for all participants:

1. Any clinically significant acute or chronic medical condition, other than HIV infection, that is considered progressive or in the opinion of the investigator makes the participant unsuitable for participation in the study;

2. If female, pregnant, lactating or planning a pregnancy during the period of screening through completion of the study;

3. In the past 6 months a history of alcohol or substance use, including marijuana, judged by the Investigator to potentially interfere with participant study compliance;

4. Bleeding disorder that was diagnosed by a physician (e.g., factor deficiency, coagulopathy or platelet disorder that requires special precautions). Note: A participant who states that he or she has easy bruising or bleeding, but does not have a formal diagnosis and has intramuscular injections and blood draws without any adverse experience, is eligible;

5. History of a splenectomy;

6. Receipt of live attenuated vaccine within the previous 60 days or planned receipt within 60 days after administration of IP; or receipt of other vaccine within the previous 14 days or planned receipt within 14 days after infusion or injection with IP (exception is live attenuated influenza vaccine within 14 days);

7. Receipt of blood transfusion or blood-derived products within the previous 3 months;

8. Participation in another clinical trial of an investigational product currently, within the previous 3 months or expected participation during this study;

9. Prior receipt of an investigational HIV vaccine candidate, monoclonal antibody or polyclonal immunoglobulin (note: receipt of placebo in a previous HIV vaccine or monoclonal antibody trial will not exclude a participant from participation if documentation is available and the Medical Monitor gives approval);

10. History of severe local or systemic reactogenicity to injections or IV infusion (e.g., anaphylaxis, respiratory difficulties, angioedema);

11. Psychiatric condition that compromises safety of the participant and precludes compliance with the protocol. Specifically excluded are persons with psychoses within the past 3 years, ongoing risk for suicide, or history of suicide attempt or gesture within the past 3 years;

12. If, in the opinion of the Principal Investigator, it is not in the best interest of the participant to participate in the trial;

TMF-03562 | 9.0 | Status:Approved | 31 Jan 2019
This copy of the document was retrieved from the system by Krysten Brown on 05 Feb 2019

13. Seizure disorder: a participant who has had a seizure in the last 3 years is excluded. (Not excluded: a participant with a history of seizures who has neither required medications nor had a seizure for 3 years.)

14. Body mass index ≥ 35 or ≤ 18.0.

15. Infectious disease: chronic hepatitis B infection (HbsAg), current hepatitis C infection (HCV Ab positive and HCV RNA positive) or interferon-alfa treatment for chronic hepatitis C infection in the past year, or active syphilis. Hepatitis B infection that is suppressed on antiretroviral therapy with undetectable HBV DNA is allowable.

16. A history of malignancy within the past 5 years (prior to screening) or ongoing malignancy;

17. Active, serious infections (other than HIV-1 infection) requiring parenteral antibiotic, antiviral or antifungal therapy within 30 days prior to enrolment;

Specific exclusion criteria for HIV-uninfected participants (Group 1):

18. Confirmed HIV-1 or HIV-2 infection;

19. Any clinically relevant abnormality on history or examination including history of immunodeficiency or autoimmune disease; use of systemic corticosteroids, immunosuppressive, anticancer, or other medications considered significant by the investigator within the previous 6 months.

    The following exceptions are permitted and will not exclude study participation: use of corticosteroid nasal spray for rhinitis, topical corticosteroids for an acute uncomplicated dermatitis; or a short course (duration of 10 days or less, or a single injection) of corticosteroid for a non-chronic condition (based on investigator clinical judgment) at least 6 weeks prior to enrolment in this study.

Specific exclusion criteria for HIV-uninfected participants (Group 1) and HIV-infected participants who are on ART (Group 2):

20. Any of the following abnormal laboratory parameters listed below:

    Hematology

    – Hemoglobin <10.5 g/dL in females; hemoglobin <11.0 g/dL in males
    – Absolute Neutrophil Count (ANC): ≤1000/mm^3
    – Absolute Lymphocyte Count (ALC): < 650/mm^3
    – Platelets: < 125,000 mm^3 or ≥ 550,000/mm^3

    Coagulation

    - aPTT: >1.25x ULN
    - INR: ≥1.1 x ULN

This copy of the document was retrieved from the system by Krysten Brown on 05 Feb 2019

Chemistry

- Sodium ≤135 mEq/L or ≥ 146 mEq/L
- Potassium ≤3.4 mEq/L or ≥ 5.6 mEq/L
- Creatinine ≥1.1 x ULN
- AST ≥1.25 x ULN
- ALT ≥1.25 x ULN
- Total bilirubin ≥1.25 x ULN
- Alkaline phosphatase ≥1.25 x ULN
- Albumin ≤ 3.0 g/dL or ≤ 30 g/L
- Creatine kinase ≥3.0 x ULN
- C-reactive protein >10 mg/L
- C3 complement < 0.82 g/L
- C4 complement < 0.14 g/L

Urinalysis

Any of the following abnormal findings if consistent with clinically significant disease:
– Protein = greater than trace on dipstick confirmed by microscopic urinalysis outside institutional range
– Blood = greater than trace on dipstick confirmed by ≥ 3 RBCs/hpf on microscopic urinalysis (not due to menses)

Specific exclusion criteria for HIV-infected participants who are on ART (Group 2) and for HIV-infected participants who are not on ART (Group 3):

21. Any clinically relevant abnormality on history or examination including history of immunodeficiency or autoimmune disease, other than HIV; use of systemic corticosteroids, immunosuppressive, anticancer, or other medications considered significant by the investigator within the previous 6 months.

    The following exceptions are permitted and will not exclude study participation: use of corticosteroid nasal spray for rhinitis, topical corticosteroids for an acute uncomplicated dermatitis; or a short course (duration of 10 days or less, or a single injection) of corticosteroid for a non-chronic condition (based on investigator clinical judgment) at least 6 weeks prior to enrolment in this study.

Specific exclusion criteria for HIV-infected participants who are not on ART (Group 3)

22. Any of the following abnormal laboratory parameters listed below:

Hematology

– Hemoglobin < 10.0 g/dL
– Absolute Neutrophil Count (ANC): <800 cells/mm_3
– Platelets: < 100,000 cells/mm3

Coagulation

This copy of the document was retrieved from the system by Krysten Brown on 05 Feb 2019

- aPTT: >1.25x ULN
- INR: ≥1.1 x ULN

Chemistry

- Estimated Glomerular filtration rate (GFR) ≤ 80 mL/min according to the Cockcroft-Gault formula for creatinine clearance:
  - Male: $\frac{(140 - \text{age in years}) \times (\text{wt in kg}) = \text{CLcr (mL/min)}}{72 \times (\text{serum creatinine in mg/dL})}$
  - Female: $\frac{(140 - \text{age in years}) \times (\text{wt in kg}) \times 0.85 = \text{CLcr (mL/min)}}{72 \times (\text{serum creatinine in mg/dL})}$
- AST ≥ 2.5 x ULN
- ALT ≥ 2.5 x ULN
- Total bilirubin ≥ 1.6 x ULN
- Alkaline phosphatase ≥ 5 x ULN

Urinalysis

Any of the following abnormal findings if consistent with clinically significant disease:
– Protein = greater than 1+ on dipstick confirmed by microscopic urinalysis outside institutional range
– Blood = greater than 1+ on dipstick confirmed by ≥ 10 RBCs/hpf on microscopic urinalysis (not due to menses)
- Leukocytes = greater than 1+ on dipstick confirmed by > 10 WBCs/hpf on microscopic urinalysis

5.7 Inclusion and Exclusion Criteria for the Optional Long-Term Extension Phase

5.7.1. Each potential participant of group 3D must satisfy all of the following inclusion criteria to be enrolled in the optional LTE phase of the study upon completion of the final main study visit at Week 24.

1. Each participant must sign an ICF appendix for the optional LTE phase indicating that he or she understands the purpose of and procedures required for the optional LTE phase and is voluntarily willing to participate in the optional LTE phase of the study.

2. Participant must have received the planned PGT121 mAb infusion in the main study.

3. Participant must have an HIV-1 RNA plasma viral load <50 copies/ml at week 24. Plasma viral load at week 24 may be repeated once at the discretion of the principal investigator or designee to confirm results.

4. Participant must have willingly deferred cART at day 56 of the main study, and remained off cART through week 24.

5. After appropriate counselling, participant must be willing to continue to defer cART treatment while HIV-1 RNA plasma viral load remains <50 copies/ml.

This copy of the document was retrieved from the system by Krysten Brown on 05 Feb 2019

6. Participant must be willing/able to adhere to the prohibitions and restrictions specified in the protocol and study procedures.

5.7.2. Any potential participant of group 3D who meets any of the following exclusion criteria at the end of the main study will be excluded from participating in the optional LTE phase of the study.

1. Participant has any condition for which, in the opinion of the investigator, participation would not be in the best interest of the participant (e.g., compromise the well-being) or that could prevent, limit, or confound the protocol-specified assessments.

2. Participant is planning to participate in an interventional study during the optional LTE phase concerning a therapeutic HIV vaccine, HIV latency reversal agent, experimental antiretroviral treatment or other experimental HIV treatment, including immunotherapy.

5.8 Recruitment of Participants

Adult male and female participants may be recruited through in-clinic referrals, information presented to community organizations, hospitals, colleges, other institutions and/or advertisements to the general public or from existing cohorts. The information distributed will contain contact details of the trial site.

6.0 STUDY VISITS

6.1 Screening Period

During Screening, study staff will perform the following procedures:

- Provide and/or review the Informed Consent Document and answer any questions about the study prior to obtaining written informed consent.
- Complete Assessment of Informed Consent Understanding (AOU). Please refer to the Study Operations Manual (SOM)

If the participant agrees to participate, passes the AOU and provides written informed consent, study staff will:

- Conduct HIV test counselling, HIV testing, and HIV risk reduction counselling, as applicable
- Conduct family planning counselling, refer for pregnancy prevention counselling if necessary
- Administer HIV risk assessment (Group 1)
- Conduct ART counselling (Group 3)
- Perform a comprehensive medical history
- Collect concomitant medication information
- Perform a general physical examination (Refer to Section 7.2)
- Collect specimens for all tests as indicated in the Schedule of Procedures in Appendices A, B and C (for details see Analytical Plan (AP)).

This copy of the document was retrieved from the system by Krysten Brown on 05 Feb 2019

When available, the screening laboratory tests will be reviewed by the trial physician. Screening laboratory test(s) may be repeated once at the discretion of the principal investigator or designee to investigate any isolated abnormalities.

If the screening visit occurs outside the allowable screening window, all screening procedures must be repeated except the comprehensive medical history may be replaced by an interim medical history and the Participant Information Sheet of the Informed Consent Document should be reviewed.

If a participant has signed the Consent Form but does not meet the eligibility criteria, the records must be kept at the site.

6.2 IV infusion or SC injection of PGT121 mAb Visit

Prior to the administration of IP, study staff will:

- Answer any questions the participant may have about the study
- Review the Informed Consent Document with the participant
- Review screening safety laboratory data
- Conduct HIV test counselling, and HIV risk reduction counselling, as applicable
- Conduct ART counselling (Group 3)
- Conduct family planning counselling as per site specific procedures and ensure compliance with respective pregnancy prevention method, and discuss male condom use with all male participants
- Review interim medical history
- Collect concomitant medication information
- Weigh participant and record vital signs
- Perform a symptom-directed physical examination (Refer to Section 7.2)
- Assess at baseline local and systemic signs and symptoms (this includes an examination of IV infusion or SC injection site)
- Collect specimens for all tests as indicated in the Schedule of Procedures see Appendices A, B and C (for details see AP).
- Obtain pregnancy test results prior to administration of IP.

Assign an allocation number to the participant according to the instructions specified in the Study Operations Manual.

This copy of the document was retrieved from the system by Krysten Brown on 05 Feb 2019

At the time of administration of IP and after IV infusion or SC injection of IP, study staff will:

- Administer the IP as specified in Section 8.4, Administration of Investigational Product and according to the instructions specified in the SOM.
- Observe participant closely during the infusion or injection of IP and for at least 30 minutes after IV infusion or SC injection of IP has ended for any acute reactogenicity. At the end of the observation period study staff will:
  - Record vital signs (pulse, respiratory rate, blood pressure and temperature)
  - Assess any local and systemic reactogenicity
  - Assess any other adverse events
- Every hour after IV infusion or SC injection of IP, for at least 6 hours, the study staff will:
  - Record vital signs (pulse, respiratory rate, blood pressure and temperature)
  - Assess any local and systemic reactogenicity
  - Assess any other adverse events
  - Collect PK samples according to the Schedule of Procedures

If a participant has an abnormal laboratory value that is known, at the time of infusion or injection, follow the specified guidelines (Section 12.0).

6.3 Post-IV infusion or SC injection of PGT121 mAb Visits

The participant will be asked to return to the clinic for post-IP administration visits as indicated in the Schedule of Procedures (see Appendices A, B and C) for an assessment by clinic staff. The participant will be asked to maintain a Memory Aid to track any local and systemic reactogenicity the participant experiences, including temperature, from the day of IP administration for the next 3 days (for a total of 4 days including day of IP administration). Study staff will review the Memory Aid with the participant and determine the severity of the reactions through discussion with the participant.

The following procedures will be conducted at these visits:

- Review interim medical history
- Collect concomitant medication information
- Perform a symptom-directed physical examination if any signs or symptoms are present
- Assess vital signs (pulse, respiratory rate, blood pressure and temperature)
- Assess any adverse events and local and systemic reactogenicity (Days 1, 2, 3) including reviewing the Memory Aid.
- Collect specimens for all tests as indicated in the Schedule of Procedures (Appendices A, B and C and AP).

6.4 Additional Follow-up Visits in the Main Study

Assessments and procedures will be performed according to the Schedule of Procedures (Appendices A, B and C).

This copy of the document was retrieved from the system by Krysten Brown on 05 Feb 2019

6.5 Unscheduled Visits

Unscheduled Visits/Contacts are visits/contacts that are not described in the Schedule of Procedures (Appendices A, B and C). Unscheduled visits may occur any time during the main study or LTE phase:

- For administrative reasons, e.g., the participant may have questions for study staff or may need to re-schedule a follow-up visit.
- To obtain laboratory test results from a previous visit.
- For other reasons as requested by the participant or site investigator.

All unscheduled visits will be documented in the participants' study records on applicable source documents and entered into the Case Report Form (CRF).

6.6 Final Study Visit or Early Termination Visit in the Main Study

Assessments and procedures will be performed according to the Schedule of Procedures (Appendices A, B and C).

6.7 Optional Long-Term Extension Phase

An optional LTE phase will be performed for participants in Subgroup 3D who have deferred cART through week 24 and also have HIV-1 viral load <50 copies/ml at the end of the main study. Following week 24, these participants will be asked to participate in the optional LTE phase and sign the ICF appendix for the optional LTE phase. Signing the ICF may be performed at an extra visit as soon as possible after week 24 and at the latest before any assessment is done on the first visit of the optional LTE phase.

Participants will attend follow-up visits at the clinic every 4 weeks for 1 year, or until HIV-1 viral load is >50 copies/ml, at which point the participant's next visit will also be the exit visit. Each visit includes recording of any SAEs, pIMDs, and concomitant medications, and collection of samples for virologic, pharmacokinetic, and immunological assays. HIV risk reduction and ART counselling will also be provided.

7.0 STUDY PROCEDURES

7.1 Informed Consent Process

A Master Informed Consent Document consisting of a Participant Information Sheet and a Consent Form is provided by the Sponsor to the trial site. This document is made site-specific and translated (if necessary), submitted and approved by the Institutional Review Board (IRB). The Master and site-specific Informed Consent Documents are separate documents and should not be part of the protocol.

Participant Information Sheet

A qualified member of the study staff will conduct the informed consent process by reviewing the Participant Information Sheet and document it in the clinic notes.

Consent Form

This copy of the document was retrieved from the system by Krysten Brown on 05 Feb 2019

The participant's consent to participate must be obtained by him/her signing and dating the Consent Form. The person obtaining consent will also sign.

The signed and dated Informed Consent Document must remain at the study site. A copy of the signed/marked and dated Informed Consent Document will be offered to the participant to take home. Those participants who do not wish to take a copy will be required to document that they declined to do so.

7.2 Medical History and Physical Examination

Medical History

At screening, a comprehensive medical history will be collected including previous IV infusions and SC injections, and reaction to IV infusion or SC injection, history of sexually transmitted infection (STI) and pregnancy prevention practices. At subsequent visits, an interim medical history will be performed.

Physical Examination

General Physical Examination

A general physical examination includes examination of head/ears/eyes/nose and throat, skin, respiratory, cardiovascular, abdominal, limited neurological and musculoskeletal and external ano-genital systems (for HIV-infected participants only) at the time points indicated in the Schedule of Procedures (see Appendices A, B and C).

Symptom-Directed Physical Examination

A symptom-directed physical examination is a targeted examination based on the participant's history or observation. If deemed necessary, this examination should be done at the time points indicated in the schedule of procedures (see Appendices A, B and C).

Measuring Height and Weight

Includes measuring the height and weight at the time points indicated in the Schedule of Procedures (see Appendices A, B and C).

Vital Signs

Vital signs including pulse, respiratory rate, blood pressure and temperature are measured and recorded at the time points indicated in the Schedule of Procedures (see Appendices A, B and C)

7.3 HIV Testing and HIV-test Counselling (Group 1)

Study staff will perform pre-HIV test counselling prior to collecting blood for an HIV test, and post-HIV test counselling when HIV test results are available. This is referred to as HIV-test counselling, and done according to the CDC guidelines. For more information on HIV testing and HIV-test counselling, see Section 11.0. A screening questionnaire and other tools may be used.

7.4 HIV Risk Reduction Counselling

HIV risk reduction counselling will be provided to all participants as outlined by site-specific SOPs.

TMF-03562 | 9.0 | Status:Approved | 31 Jan 2019
This copy of the document was retrieved from the system by Krysten Brown on 05 Feb 2019

Study staff will provide HIV risk reduction counselling based on reported individual risk and provide free condoms, as appropriate, at every visit. Group 1 will receive HIV risk reduction counselling and for Groups 2 and 3, HIV risk reduction counselling will be conducted as secondary prevention to reduce onward transmission.

7.5 Family Planning Counselling

Study staff will counsel participants about the importance of preventing pregnancies and of using condoms, as well as other effective family planning methods until at least 3 months following investigational product administrations, as appropriate. Participants may be referred for family planning services as necessary according to site-specific SOPs as detailed in the SOM. Pregnancy prevention methods chosen and compliance will be documented.

7.6 ART Counselling (Group 3)

HIV-infected participants who are not on ART will receive ART counselling upon entering the study and 8 weeks after administration of IP. Participants who have not initiated or made plans to initiate ART by the final main study visit will receive ART counselling again at their final main study visit. Participants in Subgroup 3D may be eligible for participation in the LTE phase of the study if they have deferred ART through week 24 and have HIV-1 RNA viral load <50 copies/ml at the end of the main study; these participants will receive ART counselling during the LTE phase. HIV-infected participants who are on ART (Group 2) will be counselled on the importance of continuing ART throughout the study, and will not be required to interrupt ART after administration of IP.

7.7 Specimens

Approximately 50 ml of blood will be collected from participants in Group 1, approximately 78 ml from participants in Group 2, and approximately 150 ml of blood will be collected from participants in Group 3 at the screening visit. At later visits, approximately 8.5 ml to 175 ml of blood will be collected, depending on study procedures, group assignment, and enrollment in the LTE phase (see Appendices A, B and C), usually from the antecubital fossa.

Optional collection of rectal and/or cervical mucosal secretions will be obtained using a rectal sponge (or comparable swab) or cervical Softcup (or comparable cervical fluid collection cup) for those participants that consent.

All specimens will be handled according to the procedures specified in the AP or SOPs, if applicable.

In the event of an abnormal laboratory value, participants may be asked to have an additional sample collected at the discretion of the Principal Investigator or designee.

7.8 Reimbursement

Participants will be reimbursed for their time, effort and for costs to cover their travel expenses to the study site and any inconvenience caused due to study participation. Site specific-reimbursement amounts will be documented in the site-specific Participant Information Sheet, and approved by the Institutional Review Board.

This copy of the document was retrieved from the system by Krysten Brown on 05 Feb 2019

7.9 Randomization and Blinding

Participants will be identified by a unique study identification number.

Participants will be randomized according to the randomization schedule prepared by the statisticians at the Data Coordinating Center (DCC) prior to the start of the study. Participants will be automatically assigned a specific allocation number as they are enrolled into the data entry system. An unblinding list (Pharmacy List) will be provided to the unblinded site pharmacist by the DCC.

This is a randomized, double-blind placebo-controlled study for Groups 1 and 2, and an open label study for Group 3. For Groups 1 and 2, study staff (investigator and clinical personnel monitoring the safety and laboratory assay results) and participants will be blinded with respect to the allocation of Investigational Product (PGT121 mAb or placebo). A site pharmacist will be unblinded for the purposes of preparing study product.

A participant will be considered enrolled once he/she has been assigned an allocation number.

Blinded participants will be informed about their assignment (product/placebo) at study completion, once the data is locked. Group 1 and Group 2 can be unblinded separately after the last volunteer in the respective groups completes study participation.
Should a study participant be unblinded during the study, the study participant will be followed up until the end of the study according to the Schedule of Procedures (see Appendices A and B).

7.10 Un-blinding Procedure for Individual Participants

Un-blinding of an individual participant may be indicated in the event of a medical emergency if the clinical management of the participant would be altered by knowledge of the treatment assignment.

The un-blinded information should be restricted to a small group of individuals involved in clinical management/medical treatment of the participant (e.g. treating physician) and the blind must be maintained for those responsible for the study assessments.

The reasons for un-blinding should be documented and the IAVI Chief Medical Officer, the Medical Monitor and the DCC should be notified as soon as possible. The procedures and contact numbers for un-blinding are outlined in the SOM.

7.11 Assessment of IP related HIV sero-positivity

It is possible that PGT121 mAb or an immune response to PGT121 mAb could cause a positive result on a diagnostic HIV antibody test. A Group 1 participant who tests HIV antibody positive at the end of the study will have additional testing to distinguish actual HIV infection from IP-related responses. The participant will be informed of his/her positive HIV antibody test result and offered continuing follow-up until the HIV antibody test becomes negative.

TMF-03562 | 9.0 | Status:Approved | 31 Jan 2019
This copy of the document was retrieved from the system by Krysten Brown on 05 Feb 2019

8.0 INVESTIGATIONAL PRODUCT

8.1 Description

A summary of the Investigational Products is shown in Table 8.1-1.

Table 8.1-1 Investigational Products

**IP (Active Product / Placebo)**	**Dosage level**	**Total volume in IP container**	**Total IP (Active Product or placebo) volume to be injected into a 100 mL saline IV bag^, or injected SC‡** (for an 88 kg body weight**)	**Total volume to be Infused IV** (for an 88 kg body weight**)
PGT121 (50 mg/mL)	3 mg/kg	6 mL per vial	5.3 mL	105.3 mL
	10 mg/kg		17.6 mL	117.6 mL
	30 mg/kg		52.8 mL	152.8 mL
Placebo: 0.9% Sodium Chloride Injection USP (Saline)*	3 mg/kg matching***	NA	5.3 mL***	105.3 mL***
	10 mg/kg matching***		17.6 mL***	117.6 mL***
	30 mg/kg matching***		52.8 mL***	152.8 mL***

* The Placebo provided will be a commercially-available saline partial addition IV bag.

** The actual volume to be injected will be based on the dose group and the weight of the participant at the time of IP administration. The example included here is the average weight of an adult male in the US (88kg) (http://www.cdc.gov/nchs/data/series/sr_11/sr11_252.pdf)

*** For placebo IV infusions: saline from an additional IV bag will be injected into the saline IV bag intended for administration, to match the volume used for a PGT121 mAb injection in the same dose group, to prevent unblinding.

‡ Only 3mg/kg dose will be injected SC, because of volume limitations for SC injections

^ In the case of a saline IV bag shortage, a larger size bag may be substituted as long as additional saline is aspirated out so that 100mL remains in the bag.

8.2 Shipment and Storage

Authorization to ship the Investigational Product to the site will be provided in writing by the Sponsor, upon confirmation that all required critical documents for shipment authorization are completed. The Investigational Product will be shipped maintaining the required storage conditions and stored in a secure location in the clinical site's pharmacy.

PGT121 Investigational Product is formulated in a 20 mM Acetate, 9% Sucrose, 0.008% polysorbate 80, pH 5.2 formulation buffer at a concentration of 50 mg/mL. Each 10 ml

This copy of the document was retrieved from the system by Krysten Brown on 05 Feb 2019

vial will contain 6 ml of IP and the PGT121 is stored at <- 20°C. Each vial will be labelled with the name of the product, lot number, concentration, storage temperature, date of manufacturing, manufacturer and a US cautionary statement. Multiple vials will be packaged in a box. Each box will also be labelled with similar information as the vial label, including contact information for the manufacturer.

8.3 Preparation of Investigational Product (IP)

The pharmacist will thaw and inject the appropriate volume of PGT121 into a 0.9% Sodium Chloride for Injection (USP) bag. Detailed instructions for preparing the investigational product are provided in the SOM. The site pharmacist will not be blinded, but the study physician/designee administering the IP will be blinded. Product should be administered within 4 hours of preparation. Example calculations for final volume for IV infusion or SC injection are illustrated in Table 8.1-1. Procedures for handling used and partially used vials will be provided in the SOM. Syringes or other components in direct contact with investigational products will be disposed of in a biohazard container and incinerated or autoclaved.

8.4 Administration of Investigational Product

Investigational Product will be administered at the enrolment visit. The participant will receive the IP via IV or SC infusion. Participants will receive IV infusion over approximately 60 minutes or SC injection, allowing for clinician discretion. Further information on the IV infusion or SC injection of the IP is supplied in the SOM and study documents.

8.5 Accountability and Disposal of Investigational Product

All used IP vials will be handled according to instructions in the SOM.

During the study, IP accountability forms including receipt and dispensing of vials will be kept and monitored.

At the end of the study, the used and unused IP vials will be handled according to instructions of Sponsor.

Further information on accountability and disposal of IP is supplied in the SOM.

9.0 ASSESSMENTS

9.1 Safety Assessments

Data on local and systemic reactogenicity (i.e., solicited AEs) will be collected by structured interview and medical examination. Data on other adverse events will be collected with open-ended questions. All data will be recorded on the appropriate source documents and entered into the study database. Participants will be given a Memory Aid, which is a tool to assist with collecting reactogenicity data.

Local and systemic reactogenicity events will be assessed by study staff prior to IV infusion or SC injection of IP, at approximately 30 minutes after IP administration start, at 1 hour after IP administration, and subsequently every hour for at least the first 6 hours post-IP administration. Study staff will review the Memory Aid with the participant, and

This copy of the document was retrieved from the system by Krysten Brown on 05 Feb 2019

determine the severity of the reactions on days 1-3 through discussion with the participant.

9.1.1 Local reactogenicity

The presence of local reactogenicity will be assessed at the time points specified in the Schedule of Procedures (Appendices A, B and C).
Pain, tenderness, erythema/skin discoloration, swelling/hardening or pruritus will be assessed and graded using Appendix E, Adverse Event Severity Assessment Tables, as a guideline.

9.1.2 Systemic reactogenicity

The presence of systemic reactogenicity will be assessed at the time points specified in the Schedule of Procedures (Appendices A, B and C).
Fever, chills, headache, nausea, vomiting, malaise, myalgia and arthralgia will be assessed and graded using the Appendix E, Adverse Event Severity Assessment Tables as a guideline. For the first 24 hours after IP infusion or injection, any infusion related reactions, including cytokine release syndrome, should be graded using the Common Terminology Criteria for Adverse Events (CTCAE) v5.0: November 27, 2017 (Appendix F).

9.1.3 Vital Signs

At the administration of IP visit, vital signs (pulse, respiratory rate, blood pressure and temperature) will be measured by study staff prior to IP administration, at approximately 30 minutes post IP administration start and hourly for at least 6 hours after IV infusion or SC injection. For the other study visits vital signs will be assessed at the time points specified in the Schedule of Procedures (Appendices A, B and C).

9.1.4 Other Adverse Events

Other adverse events (AEs) will be collected through 56 days after IP administration in all participants in the main study. Serious Adverse Events (SAEs) will be collected throughout the entire study period. Potential Immune Mediated Diseases (pIMDs), as defined in Section 10.5, will be collected throughout the study period, using the SAE reporting process. During the optional LTE phase, only SAEs and pIMDs are to be reported. Open-ended questions will be asked at time points according to the Schedule of Procedures (Appendices A, B and C). All adverse events will be graded using Appendix E, Adverse Event Severity Assessment Table, as a guideline and will be assessed for causality to the IP. For more information regarding adverse events refer to Section 10.0, Adverse Events.

9.1.5 Concomitant Medications

Concomitant receipt of Investigational Products is prohibited during the study.

Contraceptive use and use of medication at study entry will be documented. (See DCF instructions)

During the study, information regarding concomitant medications and reasons for their use will be solicited from the study participants for 56 days. Ongoing

This copy of the document was retrieved from the system by Krysten Brown on 05 Feb 2019

concomitant medications will be recorded until end of study, or longer for participants enrolled in the LTE phase.

9.1.6 Routine laboratory parameters

Table 9.1.6-1 shows the laboratory parameters that will be measured routinely. The samples for these tests will be collected at the time points indicated in the Schedule of Procedures (Appendices A, B and C).

Table 9.1.6-1: Laboratory Parameters

**Laboratory Parameter**	**Test**
Hematology and Coagulation	Hemoglobin, hematocrit, leukocytes, platelets, absolute neutrophil count (ANC), absolute lymphocyte count (ALC), activate partial thromboplastin time (aPTT), international normalized ratio (INR)
Clinical Chemistry	Sodium, potassium, creatinine, aspartate aminotransferase (AST), alanine aminotransferase (ALT), total bilirubin, alkaline phosphatase<br>Groups 1 and 2 only: albumin, creatine kinase, C-reactive protein, C3 complement, C4 complement
Urinalysis	Dipstick test for protein, blood, glucose, ketones, esterase (leukocytes) and nitrite. If clinically significant abnormalities (e.g., blood, protein, leukocytes) are found on dipstick test, then further test(s) will be performed (e.g., microscopy, culture)
T cell panel (Groups 2 and 3)	CD4 T cell count and frequency by single platform flow cytometry

9.1.7 Specific screening tests:

Participants will be screened to exclude the following diseases:

- Hepatitis B: positive for hepatitis B surface antigen (HBsAg) or detectable hepatitis B DNA if on antiretroviral therapy
- Hepatitis C: positive for hepatitis C RNA (HCV antibody test, followed by HCV RNA test if HCV antibody positive)
  Active syphilis: confirmed diagnosis.

A negative Hepatitis B and Hepatitis C result can be documented from the medical record only if the result is from a test administered less than 6 months ago.

9.1.8 Monitoring for anti-PGT121 antibodies:

Participants will be evaluated for the development of antibodies to PGT121 mAb (anti-drug antibodies, ADA) by ELISA according to the Schedule of Procedures (Appendices A, B and C).

This copy of the document was retrieved from the system by Krysten Brown on 05 Feb 2019

9.2 Virologic Assessments

Table 9.2-1 shows the virologic parameters that will be measured routinely in the main study and the LTE phase. The samples for these tests will be collected at the time points indicated in the Schedule of Procedures (Appendices B, C and H).

Table 9.2-1: Virologic Assessment Table

**Virologic Parameter**	**Test**
Antiviral Activity	Plasma HIV RNA levels
Anti-reservoir activity	Cell-associated HIV-1 RNA levels in resting CD4 T cells; total HIV-1 DNA and 2-long terminal repeat (LTR) HIV-1 DNA circles in resting or total CD4 T cells; quantitative viral outgrowth assay (qVOA)
Other	Genotyping of plasma HIV RNA for evaluation of PGT121-induced escape mutations; phenotyping of plasma HIV RNA for neutralization susceptibility to PGT121 in-vitro

9.3 Exploratory Immunogenicity Assessments

Humoral immune response assays will include, but are not limited to Env-specific Ab-binding assays, virus neutralization assay, and assays for Ab functionality. Cellular immune response assays will include, but are not limited to IFNγ ELISPOT assay, ICS, and multiparameter flow cytometry. Exploratory assessments on mucosal samples will include, but are not limited to characterization of Env-specific binding Abs. Priority assays are listed below.

9.3.1 Antibody Responses

- Env-specific binding Abs (titers and breadth).
- Env-specific nAbs (titers and breadth).
- Env-specific functional Abs (phagocytosis score and breadth).
- Env-specific binding Ab isotypes (IgA, IgG1-4) (titers and breadth).

9.3.2 Cellular Responses

- IFNγ peripheral blood mononuclear cell (PBMC) responders to peptide pools and subpools of Potential T-cell epitopes, PTE Env/Gag/Pol peptides.
- $CD4^+$ and $CD8^+$ T-cell functionality (% cells producing e.g. IFNγ, IL-2, IL-4, TNFα).
- T-cell development with emphasis on follicular helper T-cells and memory differentiation.

9.3.3 PBMC, Serum and Plasma Storage

Samples of cryopreserved PBMC, plasma and serum will be stored as indicated in the Schedule of Procedures (Appendix A, B and C) and the Analytical Plan (AP) and, if the participant consents, may be used for the purposes of standardization, quality control and for future assays related to HIV prevention or

TMF-03562 | 9.0 | Status:Approved | 31 Jan 2019
This copy of the document was retrieved from the system by Krysten Brown on 05 Feb 2019

treatment research and development. These samples will be archived and the testing laboratories will be blinded to the participant's identity.

9.4 Other Assessments

9.4.1 HIV Antibody Testing (Group 1)

All HIV-uninfected participants (Group 1) will be tested for HIV antibodies as indicated in the Schedule of Procedures (Appendix A) or as needed, if medical or social circumstances arise. All participants will receive HIV risk reduction counselling and pre- and post-HIV-test counselling, as specified in Section 7.3 Counselling.

9.4.2 Pharmacokinetics

Blood draws for pharmacokinetics will be done on the day of IP administration immediately before IV infusion or SC injection of IP, at the end of the IP administration, and 30 minutes after the end of IP administration, and 3 hours and 6 hours after the IP administration. An additional draw will be done at 24 hours after IP administration. Thereafter, pharmacokinetic draws will be done as indicated in the Schedule of Procedures (Appendices A, B and C). PGT121 mAb serum or plasma levels will be determined using two methods: a sandwich ELISA using a murine anti-idiotype antibody to PGT121 mAb, and a neutralization assay.

PGT121 mAb pharmacokinetic analysis will be performed using standard non-compartmental analysis methods to estimate elimination half-life (t1/2), clearance (CL/F), volume of distribution (Vz/F), Area under the concentration decay curve (AUC), impact of viral load and/or ART on PGT121 mAb disposition (elimination half-life (t1/2), clearance (CL/F), volume of distribution (Vz/F) and total exposure. PGT 121 accumulation will also be examined in rectal and cervical mucosal secretions collected with rectal sponges or cervical softcups in study participants who specifically consented for these procedures. Descriptive results will be reported for the pharmacokinetic parameters by dose subgroup.

Exploratory analysis using population analysis methods simultaneously combining all pharmacokinetic data across all doses and treatment groups will be performed for quantitative characterization of differences in PGT121 mAb disposition by dose, participant group or disease state.

9.4.3 HLA Typing

Samples for HLA typing will be collected as specified in the Schedule of Procedures (Appendix A, B and C) and AP and may be analyzed as warranted.

9.4.5 Pregnancy Test

A urine pregnancy test for all female participants will be performed by measurement of human chorionic gonadotrophin (βhCG) at time points indicated in the Schedule of Procedures (Appendices A, B and C). The results of the pregnancy test must be negative prior to IV infusion of PGT121 mAb. See section 10.7 for description of pregnancy after administration of IP.

This copy of the document was retrieved from the system by Krysten Brown on 05 Feb 2019

9.4.6 HIV Risk Assessment (Group 1)

Study staff will assess participants for their past and current risk of acquiring HIV at time points indicated in Schedule of Procedures (Appendix A).

9.4.7 Social Impact Assessment

A brief assessment of the impact of participation in the study will be administered to participants at their final study visit, either in the main study or LTE phase.

10.0 ADVERSE EVENTS

10.1 Definition

An adverse event (AE) is any untoward medical occurrence in a participant administered an Investigational Product and which does not necessarily have a causal relationship with the Investigational Product. An AE can therefore be any unfavourable or unintended sign (including an abnormal laboratory finding), symptom, or disease, temporally associated with the use of Investigational Product whether or not related to the Investigational Product.

Assessment of severity of all AEs, including and seriousness of AEs, is ultimately the responsibility of the Principal Investigator of each site. Refer to the Division of AIDS (DAIDS) Table for Grading the Severity of Adult and Pediatric Adverse Events Version 2.1, July 2017 and the National Cancer Institute Common Terminology Criteria for Adverse Events (CTCAE) v5.0: November 27, 2017 for additional guidance.

10.2 Assessment of Severity of Adverse Events

The following general criteria should be used in assessing adverse events as mild, moderate, severe or very severe at the time of evaluation:

<u>Grade 1 (Mild):</u> Symptoms causing no or minimal interference with usual social & functional activities

<u>Grade 2 (Moderate):</u> Symptoms causing greater than minimal interference with usual social & functional activities

<u>Grade 3 (Severe):</u> Symptoms causing inability to perform usual social & functional activities

<u>Grade 4 (Very Severe):</u> Symptoms causing inability to perform basic self-care functions OR Medical or operative intervention indicated to prevent permanent impairment, persistent disability, or death

Guidelines for assessing the severity of specific adverse events and laboratory abnormalities are listed in Appendix E, Adverse Event Severity Assessment Table.

10.3 Relationship to Investigational Product

Assessment of relationship of an AE or SAE to Investigational Product (IP) is the responsibility of the Principal Investigator or designee. All medically indicated and available diagnostic methods (e.g., laboratory, blood smear, culture, X-ray, etc.) should

This copy of the document was retrieved from the system by Krysten Brown on 05 Feb 2019

be used to assess the nature and cause of the AE/SAE. Best clinical and scientific judgment should be used to assess relationship of AE/SAEs to the IP and/or other cause.

The following should be considered:

- Presence/absence of a clear temporal (time) sequence between administration of the IP and the onset of AE/SAE
- Presence/absence of another cause that could more likely explain the AE/SAE (concurrent disease, concomitant medication, environmental or toxic factors)
- Whether or not the AE/SAE follows a known response pattern associated with the IP

The relationship assessment should be reported as one of the following:

Not Related: clearly explained by another cause (concurrent disease, concomitant medication, environmental or toxic factors, etc.).

Unlikely: more likely explained by another cause (concurrent disease, concomitant medication, environmental or toxic factors, etc.).

Possibly: equally likely explained by another cause but the possibility of the IP relationship cannot be ruled out (e.g., reasonably well temporally related and/or follows a known IP response pattern but equally well explained by another cause).

Probably: more likely explained by the IP (e.g., reasonably well temporally related and/or follows a known IP response pattern and less likely explained by another cause).

Definitely: clearly related and most likely explained by the IP.

For the purpose of expedited safety reporting, all possibly, probably or definitely related SAEs are considered IP-related SAEs.

10.4 Serious Adverse Events

An adverse event is reported as a "Serious Adverse Event" if it meets any of the following criteria (as per International Conference on Harmonisation [ICH] Good Clinical Practice [GCP] Guidelines):

- Results in death
- Is life threatening
- Results in persistent or significant disability/incapacity
- Requires in-participant hospitalization or prolongs existing hospitalization
- Is a congenital anomaly/birth defect or spontaneous abortion
- Any other important medical condition that requires medical or surgical intervention to prevent permanent impairment of a body function or structure

Elective surgery for pre-existing condition that did not increase in severity or frequency is not considered an SAE.

Serious Adverse Events (SAEs) should be reported within 24 hours of the site becoming aware of the event, and sent to the Sponsor as described in the SOM.

This copy of the document was retrieved from the system by Krysten Brown on 05 Feb 2019

To discuss IP-related SAEs or any urgent medical questions related to the SAE, the site investigator should contact one of the IAVI Medical Monitors directly (see Contact List in the SOM).

The IAVI SAE Report Form should be completed with all the available information at the time of reporting and sent to the Sponsor as described in the SOM. The minimum data required in reporting an SAE are the study identification number, date of birth, gender, event description (in as much detail as is known at the time), onset date of event (if available), reason event is classified as serious, reporting source (name of Principal Investigator or designee), and relationship to the IP as assessed by the investigator.

The Principal Investigator or designee is required to prepare a detailed written report with follow up until resolution or until it is judged by the Principal Investigator or designee to have stabilized.

The Principal Investigator or designee must notify the local IRB/IEC of all SAEs as appropriate. In case of IP-related SAEs, the Sponsor will notify responsible regulatory authorities, Safety Monitoring Committee (SMC), and other study sites where the same IP is being tested.

More details on SAE definitions and reporting requirements are provided in the SOM.

Serious Event Prior to Investigational Product Administration

If a serious event occurs in the period between the participant signing the Informed Consent Form and receiving the IV infusion or SC injection of IP, the event will be reported using the SAE form and following the same procedures for SAE reporting, as indicated in Section 10.4. The timing of the event will be indicated by using the relevant checkbox on the SAE form.

10.5 Reporting Potential Immune-Mediated Diseases

Potential immune-mediated diseases (pIMDs) are a subset of AEs that include both clearly autoimmune diseases and also other inflammatory and/or neurologic disorders that may or may not have an autoimmune etiology. These events are of special interest since they could potentially be caused by immune responses to the IP. The investigator/designee should report such adverse events within the same time limits (following confirmation of an AE as a pIMD; see last paragraph of this section below), and using the same CRF pages, as utilized for SAEs. The investigator or his/her designee will evaluate the occurrence of pIMDs at every visit/contact during the study. IAVI will also expect investigators/designee to provide additional information about pIMD events. AEs to be reported and documented as pIMDs include:

Neuroinflammatory disorders: optic neuritis, cranial nerve disorders (including Bell's palsy), multiple sclerosis, demyelinating disease, transverse myelitis, Guillain-Barré syndrome, myasthenia gravis, encephalitis, neuritis.

Musculoskeletal disorders: systemic lupus erythematosus, cutaneous lupus, Sjögren's syndrome, scleroderma, dermatomyositis, polymyositis, myopathy, rheumatoid arthritis

This copy of the document was retrieved from the system by Krysten Brown on 05 Feb 2019

and juvenile rheumatoid arthritis, polymyalgia rheumatica or temporal arteritis, reactive arthritis, psoriatic arthropathy, ankylosing spondylitis, undifferentiated spondyloarthropathy.

Gastrointestinal disorders: Crohn's disease, ulcerative colitis or proctitis, celiac disease.

Metabolic diseases: autoimmune thyroiditis, Grave's or Basedow's disease, Hashimoto thyroiditis, insulin-dependent diabetes mellitus (IDDM), Addison's disease.

Skin disorders: psoriasis, vitiligo, Raynaud's phenomenon, erythema nodosum, autoimmune bullous skin diseases.

Others: autoimmune hemolytic anemia, thrombocytopenia, antiphospholipid syndrome, *vasculitis, pernicious anemia, autoimmune hepatitis, primary biliary cirrhosis, primary sclerosing cholangitis, autoimmune glomerulonephritis, autoimmune uveitis, autoimmune myocarditis/cardiomyopathy, sarcoidosis, Stevens-Johnson syndrome, Behçet's syndrome.

Infusion site reactions: Grade 3 or 4 infusion site reactions lasting more than 2 days.

*Vasculitis: Vasculitis, Diffuse vasculitis, leucocytoclastic vasculitis, polyarteritis nodosa, microscopic polyangiitis, Wegener's granulomatosis, anti-neutrophil cytoplasmic antibody positive vasculitis, Henoch-schönlein purpura, allergic granulomatous angiitis (Churg-Strauss disease), Kawasaki disease, Takayasu's arteritis, temporal arteritis (giant cell arteritis), renal vasculitis.

Medical judgement should be exercised in deciding whether other disorders/diseases have an autoimmune origin and should also be reported as described above, and this judgement is the investigator's prerogative. Whenever sufficient data exist to substantiate any of the diagnoses in the above list, the event must be reported as a pIMD. While the intent of pIMD reporting is to be inclusive, isolated nonspecific symptoms, which might (or might not) represent the above diagnoses, should be captured as AEs but not reported as pIMDs until the diagnosis can be defended.

10.6 Clinical Management of Adverse Events

Adverse events (AEs) will be managed by the clinical study team who will assess, provide first line of care as appropriate and refer to health care and treatment facilities as warranted. If any treatment/medical care is required as a result of the harm caused by the IP or study procedures, this will be provided free of charge.

If a participant has an AE and/or abnormal laboratory value that is known at the time of IV infusion or SC injection of IP, the specifications of Section 12.0 will be followed.

Participants will be followed until the AE resolves or stabilizes or up to the end of the study, whichever comes last. If at the end of the study, an AE (including clinically significant laboratory abnormality) that is considered possibly, probably or definitely related to the IP is unresolved, follow-up will continue until resolution if possible and/or the participant will be referred.

TMF-03562 | 9.0 | Status:Approved | 31 Jan 2019
This copy of the document was retrieved from the system by Krysten Brown on 05 Feb 2019

If a participant from Group 3 experiences a significant decrease in CD4 cell count (e.g. – 20% of baseline, or decline to <200 cells/μL) during the course of the trial, CD4+ will be monitored closely until their CD4 count returns to baseline or until the participant initiates ART. Participants whose CD4 cell counts decrease to <200 cells/μL will be promptly informed and will be referred to their primary HIV care provider. Appropriate prophylaxis against opportunistic infections will be instituted according to accepted U.S. HIV treatment guidelines.

10.7 Pregnancy

Although not considered an AE, if a female participant becomes pregnant during the study, it is the responsibility of the Principal Investigator or designee to report the pregnancy promptly to IAVI using the designated forms. The participant will be followed for safety until the end of pregnancy or study completion, whichever occurs last. If possible, approximately 2–4 weeks after delivery, the baby will be examined by a physician to assess its health status and the results will be reported to the Sponsor. The baby will be examined again by a Physician around age 1, and the results will be reported to the Sponsor.

Complications of pregnancy that meet criteria for SAEs, specified in Section 10.4 of this Protocol (e.g., hospitalization for eclampsia, spontaneous abortion, etc.) should be reported as SAEs.

10.8 Intercurrent HIV Infection (Group 1)

HIV infection cannot be directly caused by the IP. If a participant acquires HIV through exposure in the community, at any time after the IV infusion or SC injection of IP, the participant should be offered referral to appropriate care and treatment facilities. The participant will continue to be followed in the study for safety assessments.

Intercurrent HIV infection in study participants, although not considered an SAE, must be reported promptly to IAVI using the designated forms. However, medical conditions associated with the HIV infection that meet criteria for being serious specified in the Section 10.4 of this Protocol (e.g., sepsis, *Pneumocystis jiroveci [carinii] pneumonia*, etc.) should be reported as SAEs using the SAE Report Form.

11.0 MANAGEMENT OF HIV ISSUES DURING AND FOLLOWING STUDY

11.1 HIV Testing – Group 1

Group 1 participants will be tested for HIV antibodies as indicated in the Schedule of Procedures (Appendix A) or as needed, if medical or social circumstances arise. All participants will receive HIV risk reduction counselling and pre- and post-HIV-test counselling, as specified in Section 11.3.1, Counselling (Group 1).

It is possible that PGT121 or an immune response to PGT121 mAb could cause a positive result on a diagnostic HIV antibody test. An IP recipient who falsely tests HIV positive with a diagnostic HIV antibody test at the end of the study will be informed of his/her positive test result and offered continuing follow-up until the test becomes negative.

This copy of the document was retrieved from the system by Krysten Brown on 05 Feb 2019

If a participant acquires HIV through exposure in the community, at any time after the administration of IP, the participant will be offered referral to appropriate care and treatment facilities. The participant will continue to be followed in the study for safety assessments.

Should a participant require HIV testing outside of the study for personal reasons, it is recommended that the participant contact the study staff first. HIV testing can be done at the study site and then processed at an independent laboratory as above. Written evidence of HIV status (HIV-infected or HIV-uninfected) will be provided upon request.

11.2 Social Discrimination as a Result of IP-related antibodies

In order to minimize the possibility of social discrimination in participants (if any) who test positive on a diagnostic HIV antibody test due to IP-related antibodies, appropriate diagnostic HIV testing and certification will be provided both during and after the study as needed.

11.3 HIV infection – Group 1

Group 1 participants who are diagnosed with HIV infection at screening or during the study (intercurrent HIV-infection) will be provided the following:

11.3.1 Counselling

The participant will be counselled by the study investigators or designated counsellors. The counselling process will assist the participant with the following issues:

- Psychological and social implications of HIV infection
- Who to inform and what to say
- Implications for sexual partners
- Implications for child-bearing
- Avoidance of transmission to others in future
- Mandatory reporting to the state, in some instances

11.3.2 Referral for Support/Care

Participants will be referred to a participant support center or institution of his/her choice for a full discussion of the clinical aspects of HIV infection. Referral will be made to a designated physician or center.

12.0 WITHDRAWAL FROM STUDY

12.1 Deferral of IV infusion or SC injection of IP

An IV infusion or SC injection of IP may be temporarily deferred if the participant is clinically ill at the time of the administration of IP visit and/or presents with fever (> 100.4 F) at the time of the administration of IP. A participant must be clinically well and afebrile for a minimum of a 24-hour consecutive period prior to administration of IP.

Any planned or unplanned deferral of infusion or injection of IP will be discussed with the Sponsor. Participants will be deferred from infusion or injection of IP for any of the following reasons:

This copy of the document was retrieved from the system by Krysten Brown on 05 Feb 2019

1. Pregnancy
2. A disease or condition or adverse event that may develop, regardless of relationship to Investigational Product, if the Principal Investigator or designee is of the opinion that administration of IP will jeopardize the safety of the participant
3. Participant's request to defer infusion or injection

The following events require resolution and/or review of clinical history by the Principal Investigator or designee and consultation with the Medical Monitor, prior to administration of IP:

- Any abnormal laboratory value, as outlined in section 5.7, Exclusion Criteria, Hematology, Chemistry, Urinalysis that is known at the time of infusion or injection and have not resolved. Abnormal results should be confirmed on the original sample and/or repeated at least once to confirm abnormal values.
- Receipt of inactivated/killed/subunit vaccines (non-HIV) or immunoglobulin within the previous 14 days. Receipt of live attenuated vaccines within the previous 60 days.
- Participating in another clinical study of an Investigational Product

12.2 Withdrawal from the Study (Early Termination)

Participants may be withdrawn from the study permanently for the following reasons:

1. Participants may withdraw from the study at any time if they wish, for any reason
2. The Principal Investigator or designee has reason to believe that the participant is not complying with the protocol
3. If the Sponsor decides to terminate or suspend the study

If a participant withdraws or is withdrawn from the study, all termination visit procedures will be performed according to the Schedule of Procedures (Appendices A, B, C and H) where possible. Every effort will be made to determine and document the reason for withdrawal.

13.0 DATA HANDLING

13.1 Data Collection and Record Keeping at the Study Site

Data Collection: All study data will be collected by the clinical study staff using designated source documents and entered onto the appropriate electronic CRFs (eCRFs). Access to eCRFs will be provided via an electronic data entry system hosted by the Data Coordination Center. All study data must be verifiable to the source documentation. A file will be held for each participant at the clinic(s) containing all the source documents. Source documentation will be available for review to ensure that the collected data are consistent with the CRFs.

All CRFs and laboratory reports will be reviewed by the clinical team, who will ensure that they are accurate and complete.

This copy of the document was retrieved from the system by Krysten Brown on 05 Feb 2019

Source documents and other supporting documents will be kept in a secure location. Standard GCP practices will be followed to ensure accurate, reliable and consistent data collection.

Source documents include but are not limited to:

- Signed Informed Consent Documents
- Progress notes
- Data collection forms
- Documentation of any existing conditions or past conditions relevant to eligibility
- Printed laboratory results
- Print out of the eClinical generated enrolment confirmation
- All Adverse Events
- Concomitant medications
- Local and systemic reactogenicity events

13.3 Data Entry at the Study Site

The data collected at the site will be recorded onto the eCRFs by the study staff and entered into a database. To provide for real time assessment of safety, data should be entered as soon as reasonably feasible after a visit occurs.

13.4 Data Analysis

The Sponsor, PIs and Product Developers will agree on how data will be analyzed and presented prior to unblinding.

The DCC will conduct the data analysis and will provide interim safety and final study reports for the Sponsor, Principal Investigators, the PSRT and SMC and the regulatory authorities, as appropriate.

This copy of the document was retrieved from the system by Krysten Brown on 05 Feb 2019

14.0 STATISTICAL CONSIDERATIONS

14.1 Safety and Tolerability Analysis

14.1.1 Sample Size

The sample size for safety and tolerability analysis will be 35-56 participants according to the dose escalation design used to characterize the safety profile of one IV infusion of PGT121 mAb, at one of three dose levels, to HIV-uninfected and HIV-infected individuals (Groups 1 and 2).

14.1.2 Null Hypothesis

As this is an exploratory proof of concept trial and analysis will be descriptive, no formal null hypothesis will be tested.

14.1.3 Statistical Power and Analysis and Dose Escalation Rules

The frequency of moderate or greater local and systemic reactogenicity events will be determined and compared between groups.

The frequency of SAEs judged possibly, probably or related to the IP will be determined.

All AEs will be analyzed and, grouped by seriousness, severity and relationship to the Investigational Product (as judged by the investigator).

For life-threatening adverse events related to Investigational Product: if none of the 12 (max 18) participants receiving Investigational Products experience such reactions, then the 95 % upper confidence bound for the rate of these adverse events in the population is 26.5% (or 18.5% if n=18).

All AEs will be analysed and grouped by seriousness, severity and relationship to the IP (as judged by the investigator).

For life-threatening adverse events related to IP: if none of the 12 (max 18) participants in either Group 1 or Group 2 who receive the IP experience such reactions then the 95% upper confidence bound for the rate of these adverse events in the population is 26.5% (or 18.5% if n=18).

An interim analysis of group data will be carried out according to the study schema (Table 5.3.1) without unblinding the study to investigators or participants. At the end of the study, a full analysis will be prepared.

Based on previous experience with IAVI Phase 1 IP studies, it is expected that the amount of missing, unused or spurious data will be insignificant. Unused and spurious data will be listed separately and excluded from the statistical analysis. Missing data will be excluded from the statistical analysis.

14.2 Pharmacokinetic Analysis

14.2.1 Sample Size

The sample size for pharmacokinetic analysis will be 4 per dose subgroup, sufficient to provide sufficient information for the planned analyses.

14.2.2 Null Hypothesis

As this is an exploratory proof of concept trial and analysis will be descriptive, no formal null hypothesis will be tested.

14.2.3 Statistical Power and Analysis

Disposition of PGT121 mAb will be evaluated in this study. Based on the PK profile of other human monoclonal antibodies, it is expected that the half-life of PGT121 mAb will be 14 to 21 days. Previously published data indicates that the pharmacokinetics of PGT121 and 3BNC117 are fairly similar across a non-human primate cohort and within the same non-human primate (clearance of 3BNC117 appears to be marginally faster than that for PGT121).

Commonly reported PK parameters will be calculated using standard non-compartmental slope/height/area/moment (SHAM) analysis methods. Summary descriptive results of PK parameters, including AUC, Cmax, T1/2, and clearance results will be reported by dose cohort. Dose normalized plots of PK parameters will be presented. Correlation between PK and reported safety and pharmacodynamic outcomes will also be explored parameters in order to examine exposure-effect relationships.

A more powerful exploratory analysis to quantitatively determine the dose, participant and disease impact on PGT121 mAb pharmacokinetics, and correlate exposure with response, while correctly accounting for variance based on population intrinsic factors such as weight and gender will be performed. Using the proposed population analysis approach we will be able to simultaneously examine the magnitude and the rate of change to PGT121 disposition driven by HIV-1 RNA levels and/or ART, and also examine the magnitude and the rate of decline in log copies/ml of HIV-1 RNA plasma levels from baseline.

The frequency and levels of anti-PGT121 antibodies will be calculated and tabulated.

14.3 Virologic Analysis for Group 3A

14.3.1 Sample Size

The sample size for virologic analysis in Groups 3A will be maximum of 9 participants.

14.3.2 Null Hypothesis

This copy of the document was retrieved from the system by Krysten Brown on 05 Feb 2019

As this is an exploratory proof of concept trial and the analysis will be descriptive, no formal null hypothesis will be tested.

14.3.3 Statistical Power and Analysis

The virologic analysis described in this section relates to the sample size of Group 3A of the study design, in which IP is given to HIV-infected participants off ART with plasma HIV RNA levels of $2 \times 10^3 – 10^5$ copies/ml. This section assumes that Part 1 of the study has successfully demonstrated that there is a safe dose level of the IP such that the study is carried forward into Part 2.

The primary outcome for this analysis is defined as change in log10 viral load between Day 0 (day of infusion) and Day 7.

No placebo participants are enrolled as part of this design.

The actual starting dose will be the MTD as determined by the SMC based on data from Part 1, therefore the starting dose may be 30mg/kg, 10 mg/kg or 3 mg/kg.

Assuming the starting dose is 30 mg/kg, an interim analysis of Group 3A will be performed after all 6 participants have reached 7 days following IP administration. The following scenarios may then occur, depending on the data:

- If the response in the first 6 participants results in an HIV RNA viral load difference-from-baseline significantly greater than -0.9 log10, the IP will be determined to be effective at 30 mg/kg, and enrolment into Group 3A will cease.
- If the mean response in the first 6 participants is a decrease smaller than -0.9 log10 HIV RNA, then an additional 3 participants will be enrolled into Group 3A.

For the analysis of sample size and power, log10 viral load differences from baseline for each participant were simulated from a normal distribution, with a standard deviation of 0.5. This value was chosen by examining a study of the antiretroviral drug raltegravir, which demonstrated a mean estimated standard deviation of the change of baseline of 0.47[18]. This is a conservative estimate, as the variability of viral loads near the lower range might be expected to also be lower.

The statistical test performed will be the Signed-ranktest, which will incorporate the “shift” parameter of -0.9 log10. An evaluation of potential harm (increased viral load) will also be performed with the Signed ranktest; this test will examine the null hypothesis of no change in viral load (a shift of 0.0 log10 following IP administration) against the one-sided alternative hypothesis that the viral load is increased following IP administration. Each efficacy test will be performed at the level $\alpha = 0.05$. Each test for harm will be performed at level $2\alpha = 0.10$, in order to provide additional sensitivity to detect potential harm.

This copy of the document was retrieved from the system by Krysten Brown on 05 Feb 2019

14.4 Analysis of Antiviral Activity in Subgroup 3D

14.4.1 Sample Size

The sample size for antiviral activity will be 6 participants.

14.4.2 Null Hypothesis

As this is an exploratory proof of concept trial and the analysis will be descriptive in this population, no formal null hypothesis will be tested.

14.4.3 Statistical Power and Analysis

No efficacy endpoints will be tested in Groups 3D as participants are HIV-infected with low viral loads at baseline (10^2 – $2x10^3$ copies/ml). Immunologic and virologic endpoints will be determined as described in Section 4.1.

14.5 Secondary and Exploratory Immunologic and Virologic Analyses

14.5.1 Sample Size

The sample size for secondary and exploratory immunologic and virologic analysis will be 47-71 participants.

14.5.2 Null Hypothesis

No formal hypothesis on immunologic or virologic responses will be tested, with the exception of the change in viral load described in Section 14.3.

14.5.3 Statistical Power and Analysis

Descriptive statistics (actual values and changes from reference) will be calculated for continuous immunologic and virologic parameters at all time points. Graphical representations of changes in parameters will be made as applicable. Differences between groups at a specific time point will be tested for exploratory purposes by a 2-sample t-test if the data appear to be normally distributed. If not, the non-parametric Wilcoxon rank sum test will be used. If portions of the measurements are censored below the assay quantification limit, the Gehan-Wilcoxon test will be employed. All statistical tests will be two-sided and will be considered statistically significant if $p < 0.05$.

Frequency tabulations will be calculated for discrete (qualitative) immunologic and virologic parameters at all time points. Significant differences between groups will be determined by a 2-sided Fisher's exact test.

Interim immunologic and virologic analyses of grouped data may be performed without unblinding the study to investigators or participants.

This copy of the document was retrieved from the system by Krysten Brown on 05 Feb 2019

15.0 QUALITY CONTROL AND QUALITY ASSURANCE

To ensure the quality and reliability of the data collected and generated and the ethical conduct of this study, a Study Operations Manual (SOM) will be developed. All deviations will be reported and investigated. The SOM describes reporting and deviation documentation requirements and procedures.

Regular monitoring will be performed according to ICH-GCP as indicated in Section 17.5.

An independent audit of the study and study sites may be performed by the Sponsor or designee to establish the status of applicable quality systems. Inspection by regulatory authorities may also occur.

By signing the protocol, the Principal Investigators agree to facilitate study related monitoring, audits, IRB/IEC review and regulatory inspection(s) and direct access to source documents. Such information will be treated as strictly confidential and under no circumstances be made publicly available.

16.0 DATA AND BIOLOGICAL MATERIAL

All data and biological material collected through the study shall be managed in accordance with the Clinical Trial Agreement (CTA). Distribution and use of these data will be conducted by agreement of all parties.

The computerized raw data generated will be held by the DCC on behalf of the Sponsor. The study sites will also hold the final data files and tables generated for the purpose of analysis.

17.0 ADMINISTRATIVE STRUCTURE

The Principal Investigator will be responsible for all aspects of the study at the study site.

17.1 Protocol Safety Review Team

A PSRT will be formed to monitor the clinical safety data. During the administration of IP phase of the trial, the PSRT will review the clinical safety data on a weekly basis via electronic distribution of reports. An ad hoc PSRT review meeting will occur if any of the members of the PSRT requests a special review to discuss a specific safety issue or as specified in the Study Operations Manual. After the administration of IP phase the PSRT will review the clinical safety data at least monthly.

The PSRT will consist of the IAVI Medical Monitor(s), and the PI or designee from each clinical team. The study chair or an IAVI Medical Monitor may be the PSRT chair. *Ex officio* members will include the IAVI Chief Medical Officer and an unblinded IAVI Medical Monitor.

Additional PSRT participants may include the following, as needed:

- Co-investigators and trial site senior clinical research nursing staff
- Laboratory directors
- Data management, study statistician and regulatory staff

The PSRT membership and procedures are detailed in the PSRT charter.

This copy of the document was retrieved from the system by Krysten Brown on 05 Feb 2019

17.2 Safety Monitoring Committee (SMC)

The SMC will consist of independent clinicians/scientists/statisticians/ethicists who are not involved in the study. Investigators responsible for the clinical care of participants or representative of the Sponsor may not be a member of the SMC. Details of membership, chair and co-chair and responsibilities are outlined in the SMC charter.

Principal Investigator(s) or designee and/or a Sponsor representative may be asked to join an open session of the SMC meeting to provide information on study conduct, present data or to respond to questions.

Safety data will be reviewed by the SMC at pre-specified time points and at an ad-hoc basis.

17.2.1 Content of Interim Safety Review

The SMC will be asked to review the following blinded data:

- Summary of reactogenicity (i.e., solicited adverse events)
- All adverse events judged by the Principal Investigator or designee to be possibly, probably or definitely related to IP
- All laboratory results confirmed on retest and judged by the Principal Investigator or designee to be clinically significant
- All SAEs

An unblinded presentation of all above noted events may also be made available for the SMC for their review if required by any member of the SMC.

17.2.2 SMC Review of Group 1 and 2 data prior to starting Group 3

Following IV infusion of IP of the last participant in either Group 1C or 2C, the Safety Monitoring Committee (SMC) will review safety data through the day 14 post-IV infusion visit for all participants to confirm MTD in each group, and determine whether, and at what dose level, Group 3 can initiate enrolment. The SMC can meet to confirm MTD when either Group 1C or Group 2C has finished enrolment, and does not need to wait for both groups to be completed to approve initiation of Group 3 enrolment. Data from Group 1D (SC administration) is not considered by SMC for initiation of Group 3 enrolment.

17.3 Criteria for Pausing the Study

Enrolment and administration of IP will be stopped and a safety review conducted by the SMC for any of the following criteria:

1. One or more participants experience an SAE that is judged possibly, probably or definitely related to IP.
2. There is a participant death, regardless of relationship to the IP.
3. Two or more participants experience Grade 3 adverse events in the same category System Organ Class that are considered to be possibly, probably or definitely related to IP or
4. Any grade 4 adverse event that is considered to be possibly, probably or definitely related to IP.

This copy of the document was retrieved from the system by Krysten Brown on 05 Feb 2019

Table 17.3-1: AE notification and safety pause/AE review rules

Event and relationship to study product	Severity	Occurrence	Site PI action	PSRT or SMC action
SAE, possibly, probably or definitely related	Any	Any	Phone, email or fax forms to sponsor within 24 hours	Study pause within 24 hours, refer to SMC for review
SAE, probably not or not related	Death	Any	Phone, email or fax forms to sponsor within 24 hours	Study pause within 24 hours, refer to SMC for review
AE, possibly, probably or definitely related	Grade 4	Any	Phone, email or fax notification to sponsor within 24 hours	Study pause within 24 hours, refer to SMC for review
AE¶, possibly, probably or definitely related	Grade 3*	First	Phone, email or fax notification to sponsor within 24 hours	PSRT review within 2 business days to consider pause
AE¶, possibly, probably or definitely related	Grade 3*	Second‡	Phone, email or fax notification to sponsor within 24 hours	Study pause within 24 hours, refer to SMC for review

¶Does not include the following reactogenicity symptoms (fever, malaise, myalgia, arthralgia, chills, headache, nausea, vomiting).

*If no evidence of disease is present other than an abnormal laboratory value, the test must be repeated with a new blood sample at least one time within 72 hours after the investigator becoming aware of the abnormal laboratory value. When signs and symptoms are present, repeat test will not be needed.

‡PSRT will determine whether the reported related AE (Grade 3) is a second occurrence of a previously reported AE (Grade 3).

The Sponsor will request a review by the SMC, (or the SMC chair if other SMC members cannot be convened), to be held within 2 business days of the Sponsor learning of the event. The individual participant(s)/or study may be unblinded at the discretion of the SMC.

Following this review, the SMC will make a recommendation regarding the continuation or suspension of the administration of the IP or the trial and communicate this decision immediately to the Sponsor. The Sponsor then will inform the Principal Investigators without delay.

Additional *ad hoc* review may be specifically requested by the Sponsor, the Principal Investigator(s) or by the SMC.

17.4 Study Supervision

The SMC, the IAVI Chief Medical Officer (CMO) and the IAVI Medical Monitor(s) have access to progress report(s) of this study. Close cooperation will be necessary to track study progress, respond to queries about proper study implementation and management, address issues in a timely manner, and assure consistent documentation,

This copy of the document was retrieved from the system by Krysten Brown on 05 Feb 2019

and share information effectively. Rates of accrual, retention, and other parameters relevant to the site's performance will be regularly and closely monitored by the study team.

17.5 Study Monitoring

On-and/or off-site monitoring will ensure that the study is conducted in compliance with human subjects' protection and other research regulations and guidelines, recorded and reported in accordance with the protocol, is consistent with SOPs, GCP, applicable regulatory requirements and locally accepted practices. The monitor will confirm the quality and accuracy of data at the site by validation of CRFs against the source documents, such as clinical records. The investigators, as well as participants through consenting to the study, agree that the monitor may inspect study facilities and source records (e.g., informed consent forms, clinic and laboratory records, other source documents), as well as observe the performance of study procedures (in accordance with site IRB requirements). Such information will be treated as strictly confidential and will under no circumstances be made publicly available.

The monitoring will adhere to GCP guidelines. The Principal Investigator will permit inspection of the facilities and all study-related documentation by authorized representatives of IAVI, and Government and Regulatory Authorities responsible for this study.

17.6 Investigator's Records

Study records include administrative documentation—e.g., reports and correspondence relating to the study—as well as documentation related to each participant screened and/or enrolled in the study—including informed consent forms, case report forms, and all other source documents. The investigator will maintain and store, in a secure manner, complete, accurate, and current study records for a minimum of 2 years after marketing application approval or the study is discontinued and applicable national and local health authorities are notified. IAVI will notify the Principal Investigator of these events.

18.0 INDEMNITY

The Sponsor and Institution are responsible to have appropriate liability insurance. For research-related injuries and/or medical problems determined to result from receiving the IP, treatment including necessary emergency treatment and proper follow-up care will be made available to the participant free of charge at the expense of the Sponsor.

19.0 PUBLICATION

A primary manuscript describing safety, anti-viral effect and immune responses in this trial will be prepared promptly after the data analysis is available.

Authors will be representatives of each trial site, the data management and statistical analysis center, the laboratories, the product developer and the sponsor, participant to the generally accepted criteria of contributions to the design and conduct of the study, the analysis of data and writing of the manuscript. Precedence will be given to authors from the site enrolling the

TMF-03562 | 9.0 | Status:Approved | 31 Jan 2019
This copy of the document was retrieved from the system by Krysten Brown on 05 Feb 2019

greatest number of participants. Manuscripts will be reviewed by representatives of each participating group as specified in the CTA**.**

20.0 ETHICAL CONSIDERATIONS

The Principal Investigator will ensure that the study is conducted in compliance with the protocol, SOPs in accordance with guidelines formulated by the ICH for GCP in clinical studies, the ethical principles that have their origins in the Declaration of Helsinki and applicable local standards and regulatory requirements.

TMF-03562 | 9.0 | Status:Approved | 31 Jan 2019
This copy of the document was retrieved from the system by Krysten Brown on 05 Feb 2019

APPENDIX A: SCHEDULE OF PROCEDURES – GROUP 1 (A, B, C, D)

**Study Month**		**0**							**1**		**2**		**3**	**4**	**5**	**6**
**Study Week**		**0**				**1**	**2**	**3**	**4**	**6**	**8**	**10**	**12**	**16**	**20**	**24**
**Study Day**	**Scr**	**0**	**1**	**2**	**3**	**7**	**14**	**21**	**28**	**42**	**56**	**70**	**84**	**112**	**140**	**168/ET[4]**
**Visit Windows (Days)**	**-56**	**0**	**0**	**0**	**0**	**± 2**	**± 2**	**± 2**	**± 2**	**± 3**	**± 3**	**±3**	**±7**	**±7**	**±7**	**±7**
**INVESTIGATIONAL PRODUCT**																
Investigational Product /Placebo		X														
**CONSENT/ASSESSMENTS/COUNSELLING**																
Informed Consent	X															
Assessment of Understanding	X															
HIV Risk Assessment	X															X
HIV Risk Reduction Counselling	X	X							X		X		X	X	X	X
HIV-test Counselling	X	X							X							X
Family Planning Counselling	X	X														
Social Impact Assessment																X
**CLINICAL SAFETY ASSESSMENTS**																
Comprehensive Medical History	X															
Interim Medical History		X	X	X	X	X	X	X	X	X	X					
Concomitant Medications	X	X	X	X	X	X	X	X	X	X	X					
General Physical Exam	X															X
Directed Physical Exam		X	X	X	X	X	X	X	X	X	X	X	X	X	X	
Weight	X	X														X
Height	X															
Vital Signs	X	X[1]	X	X	X	X	X	X	X	X	X	X	X	X	X	X
Local & Systemic Reactogenicity		X[1]	X	X	X											
Adverse Events		X	X	X	X	X	X	X	X	X	X					
Serious Adverse Events and pIMD	X	X	X	X	X	X	X	X	X	X	X	X	X	X	X	X

CONFIDENTIAL

**Study Month**		**0**							**1**		**2**		**3**	**4**	**5**	**6**
**Study Week**		**0**				**1**	**2**	**3**	**4**	**6**	**8**	**10**	**12**	**16**	**20**	**24**
**Study Day**	**Scr**	**0**	**1**	**2**	**3**	**7**	**14**	**21**	**28**	**42**	**56**	**70**	**84**	**112**	**140**	**168/ET[4]**
**Visit Windows (Days)**	**-56**	**0**	**0**	**0**	**0**	**± 2**	**± 2**	**± 2**	**± 2**	**± 3**	**± 3**	**±3**	**±7**	**±7**	**±7**	**±7**
**CLINICAL LABORATORY TESTS**																
Hematology and Coagulation	X	X[6]	X		X	X	X		X		X		X	X	X	X
Clinical Chemistry	X	X[6]	X		X	X	X		X		X		X	X	X	X
Urine Dipstick	X	X[6]	X		X	X	X		X		X		X	X	X	X
Urine Pregnancy test	X	X[6]							X		X		X			X
Active Syphilis	X															
Hepatitis B	X															
Hepatitis C	X															
HIV screen (4th generation Ag/Ab test)	X															
Blinded HIV diagnostic testing[2]		X[6]							X							X
**RESEARCH LABORATORY TESTS**	-	-	-	-	-	-	-	-	-	-	-	-	-	-	-	-
Anti PGT121 Antibodies (ADA)		X[6]							X		X		X			X
Humoral Assays[2]		X[6]			X	X	X		X		X		X			X
Cellular Assays[2]		X[6]					X		X		X		X			X
HLA typing		X[6]														
**PHARMACOKINETICS PGT121 ELISA**		X[3]	X	X	X	X	X	X	X	X	X	X	X	X	X	X
**MUCOSAL SAMPLING**		X[5]	X			X	X									
**PLASMA/SERUM STORAGE**		X	X	X	X	X	X	X	X		X		X			X
**PBMCs STORAGE**		X	X	X	X	X		X			X		X			X

1. At baseline, approximately 30 minutes after IP administration, and at approximately hours 1 through 6 after IV infusion or SC injection. Local and systemic reactogenicity will be assessed by clinic staff at visits on study days 1, 2 and 3. Local and Systemic reactogenicity will also be assessed by the participant using the Memory Aid on study days 1, 2, and 3
2. See Laboratory Analytical Plan for details
3. Day 0 PK draws done immediately before IP administration, at the end of IP administration, 30 minutes after end of IP administration, and 3 hours and 6 hours post IP administration. See SOM for details
4. Early Termination (ET): Procedures to be performed at ET are the same as last visit procedures
5. Cervico-vaginal and/or rectal mucosal sampling (optional) on Day 0 must be done prior to IV infusion or SC injection of IP. See SOM for details
6. Day 0 sample collections for laboratory tests must be done pre-infusion or pre-injection.

This copy of the document was retrieved from the system by Krysten Brown on 05 Feb 2019

APPENDIX B: SCHEDULE OF PROCEDURES – GROUP 2 (A, B, C)

**Study Month**		**0**							**1**		**2**		**3**	**4**	**5**	**6**
**Study Week**		**0**				**1**	**2**	**3**	**4**	**6**	**8**	**10**	**12**	**16**	**20**	**24**
**Study Day**	**Scr**	**0**	**1**	**2**	**3**	**7**	**14**	**21**	**28**	**42**	**56**	**70**	**84**	**112**	**140**	**168/ET[5]**
**Visit Windows (Days)**	**-56**	**0**	**0**	**0**	**0**	**± 2**	**± 2**	**± 2**	**± 2**	**± 3**	**± 3**	**±3**	**±7**	**±7**	**±7**	**±7**
**INVESTIGATIONAL PRODUCT**																
Investigational Product /Placebo		X														
**CONSENT/ASSESSMENTS/COUNSELLING**																
Informed Consent	X															
Assessment of Understanding	X															
HIV Risk Reduction Counselling[1]	X	X							X		X		X	X	X	X
Family Planning Counselling	X	X														
Social Impact Assessment																X
**CLINICAL SAFETY ASSESSMENTS**																
Comprehensive Medical History	X															
Interim Medical History		X	X	X	X	X	X	X	X	X	X					
Concomitant Medications	X	X	X	X	X	X	X	X	X	X	X					
General Physical Exam	X															X
Directed Physical Exam		X	X	X	X	X	X	X	X	X	X	X	X	X	X	
Weight	X	X														X
Height	X															
Vital Signs	X	X[2]	X	X	X	X	X	X	X	X	X	X	X	X	X	X
Local & Systemic Reactogenicity		X[2]	X	X	X											
Adverse Events		X	X	X	X	X	X	X	X	X	X					
Serious Adverse Events and pIMD	X	X	X	X	X	X	X	X	X	X	X	X	X	X	X	X
**CLINICAL LABORATORY TESTS**																
Hematology and Coagulation	X	X[8]	X		X	X	X		X		X		X	X	X	X
CD4	X	X[8]				X	X		X		X					X

CONFIDENTIAL

Study Month		0							1		2		3	4	5	6
**Study Week**		**0**				**1**	**2**	**3**	**4**	**6**	**8**	**10**	**12**	**16**	**20**	**24**
**Study Day**	**Scr**	**0**	**1**	**2**	**3**	**7**	**14**	**21**	**28**	**42**	**56**	**70**	**84**	**112**	**140**	**168/ET[5]**
**Visit Windows (Days)**	**-56**	**0**	**0**	**0**	**0**	**± 2**	**± 2**	**± 2**	**± 2**	**± 3**	**± 3**	**±3**	**±7**	**±7**	**±7**	**±7**
Clinical Chemistry	X	X[8]	X		X	X	X		X		X		X	X	X	X
Urine Dipstick	X	X[8]	X		X	X	X		X		X		X	X	X	X
Urine Pregnancy test	X	X[8]							X		X		X			X
Active Syphilis	X															
Hepatitis B	X															
Hepatitis C	X															
HIV 4th generation Ag/Ab test	X															
HIV Viral Load	X	X[8]	X	X	X	X	X	X	X	X	X	X	X	X	X	X
**RESEARCH LABORATORY TESTS**	-	-	-	-	-	-	-	-	-	-	-	-	-	-	-	-
Anti PGT121 Antibodies (ADA)		X[8]							X		X		X			X
HIV SGA sequencing[7]	X								X							X
HIV genotypic testing for ART resistance[7]	X								X				X			X
HIV reservoir size assessment	X						X						X			
Humoral Assays[3]		X[8]			X	X	X		X		X		X			X
Cellular Assays[3]		X[8]					X		X		X		X			X
HLA typing		X[8]														
**PHARMACOKINETICS PGT121 ELISA**		X[4]	X	X	X	X	X	X	X	X	X	X	X	X	X	X
**MUCOSAL SAMPLING**		X[6]	X			X	X									
**PLASMA/SERUM STORAGE**	X	X	X	X	X	X	X	X	X		X		X			X
**PBMCs STORAGE**		X	X	X	X	X		X			X		X			X

1. HIV risk reduction counselling as secondary prevention to reduce onward transmission
2. At baseline, approximately 30 minutes after IP administration, and at approximately hours 1 through 6 after IV infusion. Local and systemic reactogenicity will be assessed by clinic staff at visits on study days 1, 2 and 3. Local and Systemic reactogenicity will also be assessed by the participant using the Memory Aid on study days 1, 2, and 3
3. See Laboratory Analytical Plan for details
4. Day 0 PK draws done immediately before IP administration, at the end of IP administration, 30 minutes after end of IP administration, and 3 hours and 6 hours post IP administration. See SOM for details
5. Early Termination (ET): Procedures to be performed at ET are the same as last visit procedures

6. Cervico-vaginal and/or rectal mucosal sampling (optional) on Day 0 must be done prior to IV infusion of IP. See SOM for details
7. HIV envelope single genome amplification (SGA) sequencing and ART resistance genotyping will be only if they develop detectable plasma viremia >1000 HIV cp/ml during the study.
8. Day 0 sample collections for laboratory tests must be done pre-infusion

TMF-03562 | 9.0 | Status:Approved | 31 Jan 2019
This copy of the document was retrieved from the system by Krysten Brown on 05 Feb 2019

APPENDIX C.1: SCHEDULE OF PROCEDURES – GROUP 3 (A, D)

Study Month		0								1		2		3	4	5	6
**Study Week**		**0**				**1**		**2**	**3**	**4**	**6**	**8**	**10**	**12**	**16**	**20**	**24**
**Study Day**	**Scr**	**0**	**1**	**2**	**3**	**7**	**10**	**14**	**21**	**28**	**42**	**56**	**70**	**84**	**112**	**140**	**168/ET[5]**
**Visit Windows (Days)**	**-42**	**0**	**0**	**0**	**0**	**± 2**	**0**	**± 2**	**± 2**	**± 2**	**± 3**	**± 3**	**±3**	**±7**	**±7**	**±7**	**±7**
**INVESTIGATIONAL PRODUCT**																	
Investigational Product /Placebo		X															
**CONSENT/ASSESSMENTS/COUNSELLING**																	
Informed Consent	X																
Assessment of Understanding	X																
HIV Risk Reduction Counselling[1]	X	X								X		X		X	X	X	X
ART counselling	X	X										X					X
Family Planning Counselling	X	X															
Social Impact Assessment																	X
**CLINICAL SAFETY ASSESSMENTS**																	
Comprehensive Medical History	X																
Interim Medical History		X	X	X	X	X	X	X	X	X	X	X					
Concomitant Medications	X	X	X	X	X	X	X	X	X	X	X	X					
General Physical Exam	X																X
Directed Physical Exam		X	X	X	X	X	X	X	X	X	X	X	X	X	X	X	
Weight	X	X															X
Height	X																
Vital Signs	X	X[2]	X	X	X	X	X	X	X	X	X	X	X	X	X	X	X
Local & Systemic Reactogenicity		X[2]	X	X	X												
Adverse Events		X	X	X	X	X	X	X	X	X	X	X					
Serious Adverse Events and pIMD	X	X	X	X	X	X	X	X	X	X	X	X	X	X	X	X	X

TMF-03562 | 9.0 | Status:Approved | 31 Jan 2019
This copy of the document was retrieved from the system by Krysten Brown on 05 Feb 2019

Study Month		0								1		2		3	4	5	6
**Study Week**		**0**				**1**		**2**	**3**	**4**	**6**	**8**	**10**	**12**	**16**	**20**	**24**
**Study Day**	**Scr**	**0**	**1**	**2**	**3**	**7**	**10**	**14**	**21**	**28**	**42**	**56**	**70**	**84**	**112**	**140**	**168/ET[5]**
**Visit Windows (Days)**	**-42**	**0**	**0**	**0**	**0**	**± 2**	**0**	**± 2**	**± 2**	**± 2**	**± 3**	**± 3**	**±3**	**±7**	**±7**	**±7**	**±7**
**CLINICAL LABORATORY TESTS**																	
Hematology and Coagulation	X	X[9]	X		X	X		X		X		X		X	X	X	X
CD4	X	X[9]				X		X		X		X					X
Clinical Chemistry	X	X[9]	X		X	X		X		X		X		X	X	X	X
Urine Dipstick[7]	X	X[9]	X		X	X		X		X		X		X	X	X	X
Urine Pregnancy test	X	X[9]								X		X		X			X
Active Syphilis	X																
Hepatitis B	X																
Hepatitis C	X																
HIV 4th generation Ag/Ab test***	X																
HIV Viral Load	X	X[9]	X	X	X	X	X	X	X	X	X	X	X	X	X	X	X
**RESEARCH LABORATORY TESTS**	-	-	-	-	-	-	-	-	-	-	-	-	-	-	-	-	-
Anti PGT121 Antibodies (ADA)		X[9]								X		X		X			X
PGT121 susceptibility testing	X									X							X
HIV SGA sequencing[8]	X									X							X
HIV genotypic testing for ART resistance[8]	X									X				X			X
HIV reservoir size assessment[1]	X							X						X			
Humoral Assays[3]		X[9]			X	X		X		X		X		X			X
Cellular Assays[3]		X[9]						X		X		X		X			X
HLA typing		X[9]															
**PHARMACOKINETICS PGT121 ELISA**		X[4]	X	X	X	X		X	X	X	X	X	X	X	X	X	X
**MUCOSAL SAMPLING**		X[6]	X			X		X									
**PLASMA/SERUM STORAGE**		X	X	X	X	X	X	X	X	X		X		X			X

This copy of the document was retrieved from the system by Krysten Brown on 05 Feb 2019

Study Month		0								1		2		3	4	5	6
Study Week		0				1		2	3	4	6	8	10	12	16	20	24
Study Day	Scr	0	1	2	3	7	10	14	21	28	42	56	70	84	112	140	168/ET[5]
Visit Windows (Days)	-42	0	0	0	0	± 2	0	± 2	± 2	± 2	± 3	± 3	±3	±7	±7	±7	±7
PBMCs STORAGE		X	X	X	X	X	X		X			X		X			X

1. HIV risk reduction counselling as secondary prevention to reduce onward transmission
2. At baseline, approximately 30 minutes after IP administration, and at approximately hours 1 through 6 after IV infusion. Local and systemic reactogenicity will be assessed by clinic staff at visits on study days 1, 2 and 3. Local and Systemic reactogenicity will also be assessed by the participant using the Memory Aid on study days 1, 2, and 3
3. See Laboratory Analytical Plan for details
4. Day 0 PK draws done immediately before IP administration, at the end of the IP administration, 30 minutes after end of IP administration and 3 hours and 6 hours post IP administration. See SOM for details
5. Early Termination (ET): Procedures to be performed at ET are the same as last visit procedures
6. Cervico-vaginal and/or rectal mucosal sampling (optional) on Day 0 must be done prior to IV infusion of IP. See SOM for details
7. Urinalysis will only be conducted at visits after screening if clinically indicated.
8. HIV envelope single genome amplification (SGA) sequencing and ART resistance genotyping will be only if they develop detectable plasma viremia >1000 HIV cp/ml during the study.
9. Day 0 sample collections for laboratory tests must be done pre-infusion.

*** Confirmed HIV-1 infection (HIV Ab+ or HIV RNA+) by documentation in the medical records or in-clinic HIV testing.

This copy of the document was retrieved from the system by Krysten Brown on 05 Feb 2019

APPENDIX C.2: SCHEDULE OF PROCEDURES – LONG-TERM EXTENSION PHASE (WEEK 24 ONWARDS)

**Study Month**		**7**	**8**	**9**	**10**	**11**	**12**	**13**	**14**	**15**	**16**	**17**	**18**
**Study Week**		**28**	**32**	**36**	**40**	**44**	**48**	**52**	**56**	**60**	**64**	**68**	**72**
**Study Day**	**LTE Consent**	**196**	**224**	**252**	**280**	**308**	**336**	**364**	**392**	**420**	**448**	**476**	**504/ET[4]**
**Visit Windows (Days)**	**-28**	**±7**	**±7**	**±7**	**±7**	**±7**	**±7**	**±7**	**±7**	**±7**	**±7**	**±7**	**±7**
Informed Consent	X												
Assessment of Understanding	X												
Review of Inclusion/Exclusion Criteria	X												
HIV Risk Reduction Counselling[1]	X	X	X	X	X	X	X	X	X	X	X	X	X
ART Counselling	X	X	X	X	X	X	X	X	X	X	X	X	X
Social Impact Assessment													X
Concomitant Medications	X	X	X	X	X	X	X	X	X	X	X	X	X
Serious Adverse Events and pIMD[2]	X	X	X	X	X	X	X	X	X	X	X	X	X
Clinical HIV Viral Load[2,3]	X	X	X	X	X	X	X	X	X	X	X	X	X
Research Plasma Assays / Storage[2,3]	X	X	X	X	X	X	X	X	X	X	X	X	X
Research Serum Assays / Storage[2,3]	X	X	X	X	X	X	X	X	X	X	X	X	X
Research PBMC Assays / Storage[2,3]	X	X	X	X	X	X	X	X	X	X	X	X	X

1. HIV risk reduction counselling as secondary prevention to reduce onward transmission
2. Directed physical exam, vital signs, and/or other safety assessments may be performed to evaluate for potential SAE or pIMD, and/or prior to drawing blood, at the discretion of the Principal Investigator or designee.
3. See Laboratory Analytical Plan for details
4. Early Termination (ET): Procedures to be performed at ET are the same as last visit procedures

This copy of the document was retrieved from the system by Krysten Brown on 05 Feb 2019

APPENDIX D: LOW RISK CRITERIA

Low risk will be defined as:

1. **SEXUAL BEHAVIORS**

In the **last 12 months** did not:

- Have oral, vaginal or anal intercourse with an HIV-infected partner, or partner who uses injection drugs.
- Gave or receive money, drugs, gifts, or services in exchange for oral, vaginal or anal sex

AND

In the **last 6 months** has abstained from penile/anal or penile/vaginal intercourse

OR

In the **last 6 months**:

- Had 4 or fewer partners of the opposite birth sex for vaginal and/or anal intercourse, OR

Is MSM (person born male with partner(s) born male) who, in the **last 12 months**:

- Had 2 or fewer MSM partners for anal intercourse and had no unprotected anal sex with MSM, OR
- Had unprotected anal intercourse with only 1 MSM partner, within a monogamous relationship lasting at least 12 months (during which neither partner had any other partners). If the monogamous relationship ended, the participant may then have had protected anal intercourse with 1 other MSM partner (total 2 or fewer partners in the last 12 months).

Is a transgender person, regardless of the point on the transition spectrum, having sex with men (born male) and/or other transgender persons, who in the last 12 months:

- Had 2 or fewer partners for anal or vaginal intercourse, and had no unprotected anal or vaginal sex, OR
- Had unprotected anal or vaginal intercourse sex with 1 partner only within a monogamous relationship lasting at least 12 months (during which neither partner had any other partners). If the monogamous relationship ended, may then have had protected anal or vaginal sex with one other partner (total 2 or fewer partners in the last 12 months).

AND

This copy of the document was retrieved from the system by Krysten Brown on 05 Feb 2019

Uses or intends to use condoms in situations which may include penile/anal or penile/vaginal intercourse with new partners of unknown HIV status, occasional partners, partners outside a primary relationship, and/or partners known to have other partners.

2. **NON-SEXUAL BEHAVIORS**

In the **last 12 months** did not:

- Inject drugs or other substances without a prescription
- Use cocaine, methamphetamine, or excessive alcohol, which in the investigator's judgement, rendered the participant at greater than low risk for acquiring HIV infection

The investigator's judgement should consider local epidemiologic information about HIV prevalence in the area and community networks.

A participant is NOT appropriate for inclusion if he/she:

Acquired an STI (ie, new infection) in the last 12 months:

- Syphilis
- Gonorrhea
- Non-gonococcal urethritis
- HSV-2
- Chlamydia
- Pelvic inflammatory disease (PID)
- Trichomonas
- Mucopurulent cervicitis
- Epididymitis
- Proctitis
- Lymphogranuloma venereum
- Chancroid
- Hepatitis B

This copy of the document was retrieved from the system by Krysten Brown on 05 Feb 2019

APPENDIX E: ADVERSE EVENT SEVERITY ASSESSMENT TABLE

Please reference the appropriate Division of AIDS (DAIDS) Table for Grading and Severity of Adult and Pediatric Events Version 2.1, July 2017

https://rsc.niaid.nih.gov/sites/default/files/daidsgradingcorrectedv21.pdf

APPENDIX F CTCAE TABLE

Please reference the appropriate Common Terminology Criteria for Adverse Events (CTCAE) v5.0: November 27, 2017

CTCAE 5.0 Relevant For T001
Common Terminology Criteria for Adverse Events (CTCAE)
Version 5.0 Published: November 27, 2017

U.S. DEPARTMENT OF HEALTH AND HUMAN SERVICES National Institutes of Health National Cancer Institute

Quick Reference

The NCI Common Terminology Criteria for Adverse Events is a descriptive terminology which can be utilized for Adverse Event (AE) reporting. A grading (severity) scale is provided for each AE term.

Components and Organization

SOC

System Organ Class (SOC), the highest level of the MedDRA1 hierarchy, is identified by anatomical or physiological system, etiology, or purpose (e.g., SOC Investigations for laboratory test results). CTCAE terms are grouped by MedDRA Primary SOCs. Within each SOC, AEs are listed and accompanied by descriptions of severity (Grade).

CTCAE Terms

An Adverse Event (AE) is any unfavorable and unintended sign (including an abnormal laboratory finding), symptom, or disease temporally associated with the use of a medical treatment or procedure that may or may not be considered related to the medical treatment or procedure. An AE is a term that is a unique representation of a specific event used for medical documentation and scientific analyses. Each CTCAE v4.0 term is a MedDRA LLT (Lowest Level Term).

Definitions

This copy of the document was retrieved from the system by Krysten Brown on 05 Feb 2019

A brief definition is provided to clarify the meaning of each AE term.

Grades

Grade refers to the severity of the AE. The CTCAE displays Grades 1 through 5 with unique clinical descriptions of severity for each AE based on this general guideline:

Grade 1	Mild; asymptomatic or mild symptoms; clinical or diagnostic observations only; intervention not indicated.
Grade 2	Moderate; minimal, local or noninvasive intervention indicated; limiting age-appropriate instrumental ADL*.
Grade 3	Severe or medically significant but not immediately life-threatening; hospitalization or prolongation of hospitalization indicated; disabling; limiting self care ADL**.
Grade 4	Life-threatening consequences; urgent intervention indicated.
Grade 5	Death related to AE.

A Semi-colon indicates ‘or’ within the description of the grade.

A single dash (-) indicates a grade is not available.

Not all Grades are appropriate for all AEs. Therefore, some AEs are listed with fewer than five options for Grade selection.

Grade 5

Grade 5 (Death) is not appropriate for some AEs and therefore is not an option.

Activities of Daily Living (ADL)

*Instrumental ADL refer to preparing meals, shopping for groceries or clothes, using the telephone, managing money, etc.
**Self care ADL refer to bathing, dressing and undressing, feeding self, using the toilet, taking medications, and not bedridden.

† CTCAE v5.0 incorporates certain elements of the MedDRA terminology. For further details on MedDRA refer to the MedDRA MSSO Web site (https://www.meddra.org/).

MedDRA Code	MedDRA SOC	CTCAE Term	Grade 1	Grade 2	Grade 3	Grade 4	Grade 5	Definition	Navigational Note
10001718	Immune system disorders	Allergic reaction	Systemic intervention not indicated	Oral intervention indicated	Bronchospasm; hospitalization indicated for clinical sequelae; intravenous intervention indicated	Life-threatening consequences; urgent intervention indicated	Death	A disorder characterized by an adverse local or general response from exposure to an allergen.	If related to infusion, use Injury, poisoning and procedural complications: Infusion related reaction. Do not report both.
10002218	Immune system disorders	Anaphylaxis	-	-	Symptomatic bronchospasm, with or without urticaria; parenteral intervention indicated; allergy-related edema/angioedema; hypotension	Life-threatening consequences; urgent intervention indicated	Death	A disorder characterized by an acute inflammatory reaction resulting from the release of histamine and histamine-like substances from mast cells, causing a hypersensitivity immune response. Clinically, it presents with breathing difficulty, dizziness, hypotension, cyanosis and loss of consciousness and may lead to death.	

This copy of the document was retrieved from the system by Krysten Brown on 05 Feb 2019

10003239	Musculoskeletal and connective tissue disorders	Arthralgia	Mild pain	Moderate pain; limiting instrumental ADL	Severe pain; limiting self care ADL	-	-	A disorder characterized by a sensation of marked discomfort in a joint.	
10008531	General disorders and administration site conditions	Chills	Mild sensation of cold; shivering; chattering of teeth	Moderate tremor of the entire body; narcotics indicated	Severe or prolonged, not responsive to narcotics	-	-	A disorder characterized by a sensation of cold that often marks a physiologic response to sweating after a fever.	
10052015	Immune system disorders	Cytokine release syndrome	Fever with or without constitutional symptoms	Hypotension responding to fluids; hypoxia responding to <40% O2	Hypotension managed with one pressor; hypoxia requiring ≥ 40% O2	Life-threatening consequences; urgent intervention indicated	Death	A disorder characterized by fever, tachypnea, headache, tachycardia, hypotension, rash, and/or hypoxia caused by the release of cytokines.	Also consider reporting other organ dysfunctions including neurological toxicities such as: Psychiatric disorders: Hallucinations or Confusion; Nervous system disorders: Seizure, Dysphasia, Tremor, or Headache
10013573	Nervous system disorders	Dizziness	Mild unsteadiness or sensation of movement	Moderate unsteadiness or sensation of movement; limiting instrumental ADL	Severe unsteadiness or sensation of movement; limiting self care ADL	-	-	A disorder characterized by a disturbing sensation of lightheadedness, unsteadiness, giddiness, spinning or rocking.	

10013963	Respiratory, thoracic and mediastinal disorders	Dyspnea	Shortness of breath with moderate exertion	Shortness of breath with minimal exertion; limiting instrumental ADL	Shortness of breath at rest; limiting self care ADL	Life-threatening consequences; urgent intervention indicated	Death	A disorder characterized by an uncomfortable sensation of difficulty breathing.
10015218	Skin and subcutaneous tissue disorders	Erythema multiforme	Target lesions covering <10% BSA and not associated with skin tenderness	Target lesions covering 10 - 30% BSA and associated with skin tenderness	Target lesions covering >30% BSA and associated with oral or genital erosions	Target lesions covering >30% BSA; associated with fluid or electrolyte abnormalities; ICU care or burn unit indicated	Death	A disorder characterized by target lesions (a pink-red ring around a pale center).
10016558	General disorders and administration site conditions	Fever	38.0 - 39.0 degrees C (100.4 - 102.2 degrees F)	>39.0 - 40.0 degrees C (102.3 - 104.0 degrees F)	>40.0 degrees C (>104.0 degrees F) for <=24 hrs	>40.0 degrees C (>104.0 degrees F) for >24 hrs	Death	A disorder characterized by elevation of the body's temperature above the upper limit of normal.
10016825	Vascular disorders	Flushing	Asymptomatic; clinical or diagnostic observations only	Moderate symptoms; limiting instrumental ADL	Symptomatic, associated with hypotension and/or tachycardia; limiting self care ADL	-	-	A disorder characterized by episodic reddening of the skin, especially face, neck, or chest.
10019211	Nervous system disorders	Headache	Mild pain	Moderate pain; limiting instrumental ADL	Severe pain; limiting self care ADL	-	-	A disorder characterized by a sensation of marked discomfort in various parts of the head, not confined to the area of distribution of any nerve.

10020772	Vascular disorders	Hypertension	**Adult:** Systolic BP 120 - 139 mm Hg or diastolic BP 80 - 89 mm Hg;	**Adult:** Systolic BP 140 - 159 mm Hg or diastolic BP 90 - 99 mm Hg if previously WNL; change in baseline medical intervention indicated; recurrent or persistent (>=24 hrs); symptomatic increase by >20 mm Hg (diastolic) or to >140/90 mm Hg; monotherapy indicated initiated;	**Adult:** Systolic BP >=160 mm Hg or diastolic BP >=100 mm Hg; medical intervention indicated; more than one drug or more intensive therapy than previously used indicated;	**Adult and Pediatric:** Life-threatening consequences (e.g., malignant hypertension, transient or permanent neurologic deficit, hypertensive crisis); urgent intervention indicated	Death	A disorder characterized by a pathological increase in blood pressure.
10021097	Vascular disorders	Hypotension	Asymptomatic, intervention not indicated	Non-urgent medical intervention indicated	Medical intervention indicated; hospitalization indicated	Life-threatening consequences and urgent intervention indicated	Death	A disorder characterized by a blood pressure that is below the normal expected for an individual in a given environment.

10051792	Injury, poisoning and procedural complications	Infusion related reaction	Mild transient reaction; infusion interruption not indicated; intervention not indicated	Therapy or infusion interruption indicated but responds promptly to symptomatic treatment (e.g., antihistamines, NSAIDS, narcotics, IV fluids); prophylactic medications indicated for <=24 hrs	Prolonged (e.g., not rapidly responsive to symptomatic medication and/or brief interruption of infusion); recurrence of symptoms following initial improvement; hospitalization indicated for clinical sequelae	Life-threatening consequences; urgent intervention indicated	Death	A disorder characterized by adverse reaction to the infusion of pharmacological or biological substances.
10064774	General disorders and administration site conditions	Infusion site extravasation	Painless edema	Erythema with associated symptoms (e.g., edema, pain, induration, phlebitis)	Ulceration or necrosis; severe tissue damage; operative intervention indicated	Life-threatening consequences; urgent intervention indicated	Death	A disorder characterized by leakage of the infusion into the surrounding tissue. Signs and symptoms may include induration, erythema, swelling, burning sensation and marked discomfort at the infusion site.
10022095	General disorders and administration site conditions	Injection site reaction	Tenderness with or without associated symptoms (e.g., warmth, erythema, itching)	Pain; lipodystrophy; edema; phlebitis	Ulceration or necrosis; severe tissue damage; operative intervention indicated	Life-threatening consequences; urgent intervention indicated	Death	A disorder characterized by an intense adverse reaction (usually immunologic) developing at the site of an injection.

This copy of the document was retrieved from the system by Krysten Brown on 05 Feb 2019

10025482	General disorders and administration site conditions	Malaise	Uneasiness or lack of well being	Uneasiness or lack of well being limiting instrumental ADL	Uneasiness or lack of well being limiting self-care ADL	-	-	A disorder characterized by a feeling of general discomfort or uneasiness, an out-of-sorts feeling.	
10028411	Musculoskeletal and connective tissue disorders	Myalgia	Mild pain	Moderate pain; limiting instrumental ADL	Severe pain; limiting self care ADL	-	-	A disorder characterized by marked discomfort sensation originating from a muscle or group of muscles.	
10028813	Gastrointestinal disorders	Nausea	Loss of appetite without alteration in eating habits	Oral intake decreased without significant weight loss, dehydration or malnutrition	Inadequate oral caloric or fluid intake; tube feeding, TPN, or hospitalization indicated	-	-	A disorder characterized by a queasy sensation and/or the urge to vomit.	
10033371	General disorders and administration site conditions	Pain	Mild pain	Moderate pain; limiting instrumental ADL	Severe pain; limiting self care ADL	-	-	A disorder characterized by the sensation of marked discomfort, distress or agony.	Prior to using this term consider using a specific body part pain term found throughout the CTCAE (over 40 different pain terms).
10033557	Cardiac disorders	Palpitations	Mild symptoms; intervention not indicated	Intervention indicated	-	-	-	A disorder characterized by an unpleasant sensation of irregular and/or forceful beating of the heart.	

This copy of the document was retrieved from the system by Krysten Brown on 05 Feb 2019

10037087	Skin and subcutaneous tissue disorders	Pruritus	Mild or localized; topical intervention indicated	Widespread and intermittent; skin changes from scratching (e.g., edema, papulation, excoriations, lichenification, oozing/crusts); oral intervention indicated; limiting instrumental ADL	Widespread and constant; limiting self care ADL or sleep; systemic corticosteroid or immunosuppressive therapy indicated	-	-	A disorder characterized by an intense itching sensation.
10037868	Skin and subcutaneous tissue disorders	Rash maculo-papular	Macules/papules covering <10% BSA with or without symptoms (e.g., pruritus, burning, tightness)	Macules/papules covering 10 - 30% BSA with or without symptoms (e.g., pruritus, burning, tightness); limiting instrumental ADL; rash covering > 30% BSA with or without mild symptoms	Macules/papules covering >30% BSA with moderate or severe symptoms; limiting self care ADL	-	-	A disorder characterized by the presence of macules (flat) and papules (elevated). Also known as morbillform rash, it is one of the most common cutaneous adverse events, frequently affecting the upper trunk, spreading centripetally and associated with pruritis.

10040400	Immune system disorders	Serum sickness	Asymptomatic; clinical or diagnostic observations only; intervention not indicated	Moderate arthralgia; fever, rash, urticaria, antihistamines indicated	Severe arthralgia or arthritis; extensive rash; steroids or IV fluids indicated	Life-threatening consequences; pressor or ventilatory support indicated	Death	A disorder characterized by a delayed-type hypersensitivity reaction to foreign proteins derived from an animal serum. It occurs approximately six to twenty-one days following the administration of the foreign antigen. Symptoms include fever, arthralgias, myalgias, skin eruptions, lymphadenopathy, chest marked discomfort and dyspnea.
10051837	Skin and subcutaneous tissue disorders	Skin induration	Mild induration, able to move skin parallel to plane (sliding) and perpendicular to skin (pinching up)	Moderate induration, able to slide skin, unable to pinch skin; limiting instrumental ADL	Severe induration; unable to slide or pinch skin; limiting joint or orifice movement (e.g., mouth, anus); limiting self care ADL	Generalized; associated with signs or symptoms of impaired breathing or feeding	Death	A disorder characterized by an area of hardness in the skin.
10042241	Respiratory, thoracic and mediastinal disorders	Stridor	-	-	Respiratory distress limiting self care ADL; medical intervention indicated	Life-threatening airway compromise; urgent intervention indicated (e.g., tracheotomy or intubation)	Death	A disorder characterized by a high pitched breathing sound due to laryngeal or upper airway obstruction.

10046735	Skin and subcutaneous tissue disorders	Urticaria	Urticarial lesions covering <10% BSA; topical intervention indicated	Urticarial lesions covering 10 - 30% BSA; oral intervention indicated	Urticarial lesions covering >30% BSA; IV intervention indicated	-	-	A disorder characterized by an itchy skin eruption characterized by wheals with pale interiors and well-defined red margins.
10047700	Gastrointestinal disorders	Vomiting	Intervention not indicated	Outpatient IV hydration; medical intervention indicated	Tube feeding, TPN, or hospitalization indicated	Life-threatening consequences	Death	A disorder characterized by the reflexive act of ejecting the contents of the stomach through the mouth.

APPENDIX G REFERENCES

1 (UNAIDS)., J. U. N. P. o. H. A. The Gap Report., (UNAIDS, 2014).
2 UNAIDS. AIDS by the numbers 2015. (2015).
3 CDC. CDC. Vital Signs: HIV Diagnosis, Care, and Treatment Among Persons Living with HIV- United States 2011. *MMWR* **4**, 1-6 (2014).
4 Jardine, J. *et al.* Rational HIV immunogen design to target specific germline B cell receptors. *Science* **340**, 711-716, doi:10.1126/science.1234150 (2013).
5 Sok, D. *et al.* Promiscuous glycan site recognition by antibodies to the high-mannose patch of gp120 broadens neutralization of HIV. *Sci Transl Med* **6**, 236ra263, doi:10.1126/scitranslmed.3008104 (2014).
6 Caskey, M. *et al.* Viraemia suppressed in HIV-1-infected humans by broadly neutralizing antibody 3BNC117. *Nature* **522**, 487-491, doi:10.1038/nature14411 (2015).
7 Barouch, D. H. *et al.* Therapeutic efficacy of potent neutralizing HIV-1-specific monoclonal antibodies in SHIV-infected rhesus monkeys. *Nature* **503**, 224-228, doi:10.1038/nature12744 (2013).
8 Hessell, A. J. *et al.* Broadly neutralizing human anti-HIV antibody 2G12 is effective in protection against mucosal SHIV challenge even at low serum neutralizing titers. *PLoS Pathog* **5**, e1000433, doi:10.1371/journal.ppat.1000433 (2009).
9 Hessell, A. J. *et al.* Effective, low-titer antibody protection against low-dose repeated mucosal SHIV challenge in macaques. *Nat Med* **15**, 951-954, doi:10.1038/nm.1974 (2009).
10 Moldt, B. *et al.* Highly potent HIV-specific antibody neutralization in vitro translates into effective protection against mucosal SHIV challenge in vivo. *Proceedings of the National Academy of Sciences of the United States of America* **109**, 18921-18925, doi:10.1073/pnas.1214785109 (2012).
11 Walker, L. M. *et al.* Broad neutralization coverage of HIV by multiple highly potent antibodies. *Nature* **477**, 466-470, doi:10.1038/nature10373 (2011).
12 Haynes, B. F. & McElrath, M. J. Progress in HIV-1 vaccine development. *Curr Opin HIV AIDS* **8**, 326-332, doi:10.1097/COH.0b013e328361d178 (2013).
13 Burton, D. R. & Mascola, J. R. Antibody responses to envelope glycoproteins in HIV-1 infection. *Nat Immunol* **16**, 571-576, doi:10.1038/ni.3158 (2015).
14 Sok, D. *et al.* Recombinant HIV envelope trimer selects for quaternary-dependent antibodies targeting the trimer apex. *Proc Natl Acad Sci U S A* **111**, 17624-17629, doi:10.1073/pnas.1415789111 (2014).
15 Scheid, J. F. *et al.* Sequence and structural convergence of broad and potent HIV antibodies that mimic CD4 binding. *Science* **333**, 1633-1637, doi:10.1126/science.1207227 (2011).
16 Shingai, M. *et al.* Passive transfer of modest titers of potent and broadly neutralizing anti-HIV monoclonal antibodies block SHIV infection in macaques. *J Exp Med* **211**, 2061-2074, doi:10.1084/jem.20132494 (2014).
17 Lynch, R. M. *et al.* Virologic effects of broadly neutralizing antibody VRC01 administration during chronic HIV-1 infection. *Sci Transl Med* **7**, 319ra206, doi:10.1126/scitranslmed.aad5752 (2015).
18 Andrade, A. *et al.* Three distinct phases of HIV-1 RNA decay in treatment-naive patients receiving raltegravir-based antiretroviral therapy: ACTG A5248. *J Infect Dis* **208**, 884-891, doi:10.1093/infdis/jit272 (2013).

Signature Page for TMF-03562 v9.0

Reason for signing: Approved	Name: Fran Priddy<br>Role: Chief Medical Officer<br>Date of signature: 31-Jan-2019 20:50:47 GMT+0000

Signature Page for TMF-03562 v9.0
