# Supplementary material for: Safety, pharmacokinetics and antiviral activity of PGT121, a broadly neutralizing monoclonal antibody against HIV-1: a randomized, placebo-controlled, phase 1 clinical trial
Source: Nat Med. 2021 Oct 7;27(10):1718–24. doi: 10.1038/s41591-021-01509-0 (PMC8516645; doi:10.1038/s41591-021-01509-0)
Supplement: Supplementary file 2 — Reporting Summary [file 41591_2021_1509_MOESM2_ESM.pdf]

nature portfolio

Corresponding author(s): Dan H. Barouch, MD, PhD
Last updated by author(s): August 5, 2021

Reporting Summary

Nature Portfolio wishes to improve the reproducibility of the work that we publish. This form provides structure for consistency and transparency in reporting. For further information on Nature Portfolio policies, see our Editorial Policies and the Editorial Policy Checklist.

Statistics

For all statistical analyses, confirm that the following items are present in the figure legend, table legend, main text, or Methods section.

n/a	Confirmed	
☐	☒	The exact sample size (*n*) for each experimental group/condition, given as a discrete number and unit of measurement
☐	☒	A statement on whether measurements were taken from distinct samples or whether the same sample was measured repeatedly
☐	☒	The statistical test(s) used AND whether they are one- or two-sided<br>*Only common tests should be described solely by name; describe more complex techniques in the Methods section.*
☐	☒	A description of all covariates tested
☐	☒	A description of any assumptions or corrections, such as tests of normality and adjustment for multiple comparisons
☐	☒	A full description of the statistical parameters including central tendency (e.g. means) or other basic estimates (e.g. regression coefficient) AND variation (e.g. standard deviation) or associated estimates of uncertainty (e.g. confidence intervals)
☐	☒	For null hypothesis testing, the test statistic (e.g. F, t, r) with confidence intervals, effect sizes, degrees of freedom and P value noted<br>*Give P values as exact values whenever suitable.*
☐	☒	For Bayesian analysis, information on the choice of priors and Markov chain Monte Carlo settings
☐	☒	For hierarchical and complex designs, identification of the appropriate level for tests and full reporting of outcomes
☐	☒	Estimates of effect sizes (e.g. Cohen's d, Pearson's r), indicating how they were calculated

Our web collection on statistics for biologists contains articles on many of the points above.

Software and code

Policy information about availability of computer code

Data collection	eClinical V3.7
Data analysis	PK data was analyzed in Monolix (version 2019R1, Antony, France: Lixoft SAS, 2019). Virus neutralization modeling was done using Python Scipy Optimize package (https://docs.scipy.org/doc/scipy/reference/optimize.minimize-lbfgsb.html). Additional analysis was done with Scipy (https://docs.scipy.org/doc/scipy/reference/generated/scipy.integrate.simps.html). Viral load kinetics were modeled with Scipy as well (https://docs.scipy.org/doc/scipy-0.15.1/reference/generated/scipy.stats.mstats.theilslopes.html). Phylogenetic trees were constructed using IQ-tree and recombination analysis was performed using RAPR.

For manuscripts utilizing custom algorithms or software that are central to the research but not yet described in published literature, software must be made available to editors and reviewers. We strongly encourage code deposition in a community repository (e.g. GitHub). See the Nature Portfolio guidelines for submitting code & software for further information.

Data

Policy information about availability of data

All manuscripts must include a data availability statement. This statement should provide the following information, where applicable:

- Accession codes, unique identifiers, or web links for publicly available datasets
- A description of any restrictions on data availability
- For clinical datasets or third party data, please ensure that the statement adheres to our policy

All viral sequences identified in this study are publicly available via GenBank (see Suppl. Table 12 in manuscript for GenBank Accession Numbers). Comprehensive data on HIV genetic sequences and immunological epitopes used for analysis in this study are publicly available via Los Alamos National Laboratory (hiv.lanl.gov/

content/index). Additional requests for access to the study data can be submitted to D.H.B. (dbarouch@bidmc.harvard.edu). Data that contains protected health information or may identify a participant is restricted and therefore additional data requests must be reviewed prior to release.

Field-specific reporting

Please select the one below that is the best fit for your research. If you are not sure, read the appropriate sections before making your selection.

☒ Life sciences ☐ Behavioural & social sciences ☐ Ecological, evolutionary & environmental sciences

For a reference copy of the document with all sections, see nature.com/documents/nr-reporting-summary-flat.pdf

Life sciences study design

All studies must disclose on these points even when the disclosure is negative.

Sample size

The sample size for safety and tolerability analysis was 35-56 participants according to the dose escalation design. As this was an exploratory proof of concept trial and analysis was descriptive, no formal null hypothesis was tested. The frequency of moderate or greater reactogenicity events was determined and compared between groups. The frequency of SAEs judged possibly, probably or related to the IP was determined. All AEs were analyzed and, grouped by seriousness, severity and relationship to the IP (as judged by the investigator). An interim analysis of group data was carried out according to the study schema without unblinding the study to investigators or participants. At the end of the study, a full analysis was prepared. Unused and spurious data was listed separately and excluded from the statistical analysis. Missing data was excluded from the statistical analysis. (2) The sample size for pharmacokinetic (PK) analysis was 4 per dose sub-group, sufficient to provide information for the planned analyses. As this was an exploratory proof of concept trial and analysis was descriptive, no formal null hypothesis was tested. The data were fit to standard two-compartment population models using the Stochastic Approximation Expectation-Maximization (SAEM) estimation method in Monolix (version 2019Rl, Antony, France: Lixoft SAS, 2019). Residual variability was estimated using an additive plus proportional error model, and models were performed separately by HIV infection status. Correlations between Cl and Ve were significant (Pearson's correlation coefficient p-value s 0.05) and were included in the models. Median-adjusted logl0-transformed weight was significantly correlated (Pearson's correlation coefficient p-value s 0.05) with Vp in the HIV-uninfected model and with Ve and Cl in the HIV-infected model and those relationships were also included in the models. Distribution and elimination half-lives were computed using the resulting model. AUC was estimated by calculating the integral of the predicted concentration-time curve from the infusion time to infinity. Peak concentration (Cmax) was computed as the maximum observed concentration. Summary descriptive results of individual estimates of PK parameters, including AUC, Cmax, Tl/2, and clearance results were reported by dose cohort. Correlation between PK and reported safety and pharmacodynamic outcomes were also explored parameters in order to examine exposure-effect relationships. (3) The frequency and levels of anti-PGT121 antibodies were calculated and tabulated. (4) The sample size for virologic analysis in Group 3A was 9 participants. As this was an exploratory proof of concept trial and the analysis was descriptive, no formal null hypothesis was tested. The primary outcome for this analysis was defined as change in logl0 viral load between Day 0 (day of infusion) and Day 7. No placebo participants were enrolled as part of this design. The statistical test performed was the Signed-rank test, which incorporated the "shift" parameter of -0.9 logl0. An evaluation of potential harm (increased viral load) was also performed with the Signed rank test; this test examined the null hypothesis of no change in viral load (a shift of 0.0 logl0 following IP administration) against the one-sided alternative hypothesis that the viral load is increased following IP administration. Each efficacy test was performed at the level a= 0.05. Each test for harm was performed at level 2a = 0.10, in order to provide additional sensitivity to detect potential harm. (5) The sample size for antiviral activity in group 3D was up to 6 participants. As this was an exploratory proof of concept trial and the analysis was descriptive in this population, no formal null hypothesis was tested. No efficacy endpoints were tested in Groups 3D as participants were HIV-infected with low viral loads at baseline (102 - 2x103 copies/ml). Immunologic and virologic endpoints were determined as described in Protocol Version 8.0 Section 4.1

Data exclusions

None

Replication

This section does not apply to our study which was a clinical trial with unique participants who could not be replicated. There were, however, 48 participants enrolled who received some of the same interventions as outlined below.

Randomization

In Part 1, eligible participants were enrolled into the lowest dose sub-group (Group lA and 2A) first according to their HIV-serostatus and in parallel. Participants in each sub-group were identified by a unique study identification number. Participants were randomized according to the randomization schedule prepared by the statisticians at the Data Coordinating Center (DCC) prior to the start of the study. Participants were automatically assigned a specific allocation number as they were enrolled into the data entry system. The 5 participants in each sub-group were randomized in a ratio of four PGT121 recipients to one placebo recipient. At each dose level in Part 1, IP administration was separated by at least 24 hours for each of the first 3 participants. Randomization ensured at least 2 participants received active product and were observed for at least 24 hours before administration to additional participants.

Blinding

An unblinding list (Pharmacy List) was provided to the unblinded site pharmacist by the DCC. Study staff (investigator and clinical personnel monitoring the safety and laboratory assay results) and participants were blinded with respect to the allocation of investigational product (IP). A site pharmacist was unblinded for the purposes of preparing study product. Blinded participants were informed about their assignment (product/placebo) at study completion, once the data was locked. Group 1 and Group 2 were unblinded separately after the last participant in the respective groups completed study participation. As PGT121 and placebo (saline) looked identical in the infusion bag, no masking was required.

Reporting for specific materials, systems and methods

We require information from authors about some types of materials, experimental systems and methods used in many studies. Here, indicate whether each material, system or method listed is relevant to your study. If you are not sure if a list item applies to your research, read the appropriate section before selecting a response.

Materials & experimental systems

n/a	Involved in the study
☐	☒ Antibodies
☒	☐ Eukaryotic cell lines
☒	☐ Palaeontology and archaeology
☒	☐ Animals and other organisms
☐	☒ Human research participants
☐	☒ Clinical data
☒	☐ Dual use research of concern

Methods

n/a	Involved in the study
☒	☐ ChIP-seq
☒	☐ Flow cytometry
☒	☐ MRI-based neuroimaging

Antibodies

Antibodies used	Drug Product: PGT121. Althea/Catalent Lot Number 1-FIN-2442. Date of Fill: 18AUG2016. The product was produced by Catalent Pharma Solutions, Madison WI, USA and Ajinomoto Althea, San Diego, CA in accordance with GMP.
Validation	All detailed product information on PGT121 is available in the Investigator's Brochure which was submitted for this trial under IND 126807, including specifically Section 3, "Physical, Chemical and Pharmaceutical Properties." The IB is available upon request from IAVI, the sponsor.

Human research participants

Policy information about studies involving human research participants

Population characteristics	ales and females aged 18-50 years old who were willing to maintain low risk behavior for HIV infection (Group 1); HIV-infected males and females aged 18-65 years old on a stable antiretroviral regimen with HIV-1 RNA plasma level <50 copies/ml, CD4 cell count? 300 cells/µL, and no history of AIDS-defining illness within the previous 5 years (Group 2); and HIV-infected males and females aged 18-65 years old, not on antiretroviral therapy for> 6 months with detectable HIV-1 viral load between 100 to 2,000 copies/ml and 2,000 to 100,000 copies/ml (as per protocol), CD4 cell count> 300 cells/µL, and no history of AIDS-defining illness within the previous 5 years (Group 3).
Recruitment	Adult male and female participants were recruited through in-clinic referrals, information presented to community organizations, hospitals, colleges, other institutions and/or advertisements to the general public or from existing cohorts. The information distributed contained information about the trial and contact information for the site. Study staff members also attended events related to public health, HIV/AIDS, sexual health, and other topics as appropriate. Because participants were recruited from North America, HIV sequence diversity was biased towards clade B viruses that may be less susceptible to PGT121 compared to other clades. For our HIV-negative population, recruits were from the Boston area and were relatively white compared to the general population of HIV-infected patients; this may limit generalisability to other populations.
Ethics oversight	BIDMC Institutional Review Board, the OIC Institutional Review Board, and the HART Committee for the Protection of Human Subjects

Note that full information on the approval of the study protocol must also be provided in the manuscript.

Clinical data

Policy information about clinical studies

All manuscripts should comply with the ICMJE guidelines for publication of clinical research and a completed CONSORT checklist must be included with all submissions.

Clinical trial registration	NCT02960581
Study protocol	The protocol and amendments were provided as an appendix to the manuscript.
Data collection	Ninety-eight participants were screened, and 50 were found to be ineligible or excluded for other reasons (Fig. 1). The first participant was enrolled on Jan 17, 2017, the last participant was enrolled on Jan 22, 2019, and the last study visit was completed on Jul 8, 2019. Part 1 of the study was a single-center, randomized, double-blind, dose-escalation, placebo-controlled trial of PGT121 in HIV-uninfected adults and HIV-infected adults on antiretroviral therapy (ART) at Beth Israel Deaconess Medical Center (BIDMC), Boston MA. Part 2 of the study was a multi-center, open-label trial of PGT121 in viremic HIV-infected adults not on ART at three sites: BIDMC, Boston MA, Orlando Immunology Center (OIC), Orlando FL, and Houston AIDS Research Team (HART), McGovern Medical School at The University of Texas Health Science Center at Houston TX.
Outcomes	The primary endpoints were, for safety and tolerability: (1) proportion of participants with moderate or greater reactogenicity (e.g., solicited AEs) for 3 days following IV infusion or SC injection of PGT121 mAb, (2) proportion of participants with moderate or greater and/or PGT121 mAb-related unsolicited AEs, including safety laboratory (biochemical, hematological) parameters, following IV infusion or SC injection of PGT121 mAb for the first 56 days post administration of IP, and (3) proportion of participants with PGT121 mAb-related SAEs throughout the study period. The primary endpoints, for pharmacokinetics, were elimination half-life (tl/2), clearance (CL/F), volume of distribution (Vz/F), area under the concentration decay curve (AUC), and impact of viral load and/or ART on PGT121 disposition (elimination half-life (tl/2), clearance (CL/F), volume of distribution (Vz/F), and total exposure. The primary endpoint for antiviral activity among viremic HIV-infected adults not on ART was the change in plasma HIV-1 RNA levels from baseline

(mean of pre-entry and entry values). The secondary endpoints were change in serum anti-PGT121 antibody titers from baseline, change in CD4+ T cell count and frequency compared to baseline as measured by single platform flow cytometry, and development of HIV-1 sequence variations in epitopes known to result in reduced PGT121 mAb neutralization susceptibility. Additional exploratory assessments could include, but were not limited to, the following: HIV-specific
lgG/lgA binding responses by ELISA, HIV-specific cellular immune responses by ELISPOT, HIV-specific antibody function by ADCC, ADCP, and ADCVI assays, resistance mutations to current ARVs, PGT121 mAb levels in mucosal secretions, changes in total HIV-1 DNA and 2-long terminal repeat (LTR) circular HIV-1 DNA in resting or total CD4 T cells and in vitro neutralization of HIV isolates with participant's serum post administration of PGT121 mAb. Available samples from time points during the optional LTE phase were also used for determination of
long-term durability of the immune responses. The primary endpoints for safety, tolerability and pharmacokinetics were changed in Protocol Version 6.0 to nclude evaluate of both IV and SC routes, after the addition of sub-group 1D to the trial design. All primary endpoints were also changed in Protocol Version 8.0 to include evaluation of data from participants enrolled in the optional LTE phase.
